# Small RNAs are differentially expressed in autoimmune and non-autoimmune diabetes and controls

**DOI:** 10.1530/EJE-22-0083

**Published:** 2022-05-26

**Authors:** Elin Pettersen Sørgjerd, Robin Mjelle, Vidar Beisvåg, Arnar Flatberg, Valdemar Grill, Bjørn O Åsvold

**Affiliations:** 1 HUNT Research Centre, Department of Public Health and Nursing, Faculty of Medicine and Health Sciences, NTNU, Norwegian University of Science and Technology, Levanger, Norway; 2 Department of Endocrinology, Clinic of Medicine, St. Olavs Hospital, Trondheim University Hospital, Trondheim, Norway; 3 Bioinformatics Core Facility – BioCore, Norwegian University of Science and Technology NTNU, Trondheim, Norway; 4 Department of Clinical and Molecular Medicine, Faculty of Medicine and Health Sciences, NTNU, Norwegian University of Science and Technology, Trondheim, Norway; 5 Central Administration, St. Olav’s Hospital, Trondheim University Hospital, Trondheim, Norway; 6 K.G. Jebsen Center for Genetic Epidemiology, Department of Public Health and Nursing, Faculty of Medicine and Health Sciences, NTNU, Norwegian University of Science and Technology, Trondheim, Norway

## Abstract

**Objective:**

Diabetes is a heterogeneous disease and a precise diagnosis of diabetes subgroups is necessary to initiate proper early treatment and clinical management of the disease. Circulating small RNAs (sRNAs) are potentially diagnostic biomarkers in diseases, including diabetes. Here we aimed to examine whether profiles of circulating sRNAs differed between patients with autoimmune and non-autoimmune diabetes and non-diabetic controls.

**Design:**

This cross-sectional case–control study included participants from the third survey of the HUNT study.

**Methods:**

We performed sRNA sequencing in serum from adult-onset type 1 diabetes (*n* = 51), type 2 diabetes (*n* = 50) and latent autoimmune diabetes in adult (LADA, *n*  = 51), as well as non-diabetic HUNT3 participants as control group (*n* = 51). Differential expression analysis of the sRNAs was performed in R using limma-voom.

**Results:**

We identified differences in sRNA expression between autoimmune (type 1 diabetes and LADA) and non-autoimmune diabetes (type 2 diabetes) and between patients with diabetes and non-diabetic controls. Focusing on miRNA, we identified 10 differentially expressed mature miRNAs and 30 differentially expressed miRNA variants (isomiRs). We also identified significant changes within other sRNA classes, including a pronounced downregulation of a tRNA fragment in patients with diabetes compared to non-diabetic controls. We created cross-validated sRNA signatures based on the significant sRNAs that distinguished patients with diabetes from non-diabetic controls, and autoimmune from non-autoimmune diabetes, with high specificity and sensitivity. sRNA profiles did not distinguish between type 1 diabetes and LADA.

**Conclusions:**

Circulating sRNAs are differentially expressed between patients with diabetes and non-diabetic controls and between autoimmune and non-autoimmune diabetes.

## Introduction

Most individuals with diabetes are classified as having type 1 or type 2 diabetes. Type 1 diabetes is basically an autoimmune disease. Autoimmunity is also present in patients with a phenotype of type 2 diabetes but with detectable autoantibodies against pancreatic beta-cells (1, 2, 3, 4, 5). This form of diabetes, latent autoimmune diabetes in adults (LADA), constitutes 4–10% of what was formerly regarded as type 2 diabetes (6, 7). The diagnosis of LADA is generally based on the presence of autoantibodies (mainly GAD autoantibody) at diabetes diagnosis, age >30 years old at diabetes onset and at least 6 months between the time of diagnosis and initiation time of insulin treatment.

A high GADA level in LADA has been associated with earlier insulin initiation, lower BMI and type 1 diabetes-associated genes, for example, being more type 1 diabetes-like both phenotypically and genotypically (7, 8, 9). Conversely, low GADA levels have been associated with a more type 2 diabetes-like phenotype and genotype.

Overall, both the pathophysiology and the genetic characterization of LADA are far less understood than its counterparts, type 1 and type 2 diabetes. While LADA and type 2 diabetes today are easily separated by measurements of pancreatic autoantibodies like GADA, LADA and adult-onset type 1 diabetes are primarily separated by time of insulin initiation and are therefore subjected to the clinicians’ decisions. Individuals with adult-onset type 1 diabetes more often have elevated levels of two or more autoantibodies than those having LADA, although LADA patients may also be positive for other autoantibodies like Islet antigen 2 (IA-2) and Zink transporter 8 (ZnT8) (10). The C-peptide concentration would also be low or not detectible in type 1 diabetes, but usually normal or low in LADA. More precise differentiation between LADA and adult-onset type 1 diabetes is therefore a relevant clinical issue and biomarkers that can differentiate between the subgroups of diabetes are needed for more precise diagnosis. Ketosis-prone diabetes (KPD) is an intermediate form of diabetes that shares features of both type 1 and 2 diabetes, highlighting the complexity of diabetes diagnostics (11). KPD differs from LADA in that LADA is generally not prone to diabetic ketoacidosis (12). To improve the characterization of LADA and better distinguish LADA from type 1 (especially adult-onset type 1 diabetes) and type 2 diabetes, new roads need to be explored.

miRNA are found to be useful as biomarkers for both the development and progression of many diseases including type 1 and type 2 diabetes. Studies have shown that miRNAs are stable and readily detectable in serum from biobanks and therefore can be used for biomarker discovery several years after collection (13). A study by Zampetaki *et al.* found that using an expression profile of the five most significant miRNAs (miR-15a, miR-126, miR-320, miR-223 and miR-28-3p) could identify 70% of the patients with type 2 diabetes (14). These miRNAs were altered before diabetes diagnosis and could also predict about 50% of those who later developed type 2 diabetes. Knowledge on miRNAs in LADA is sparse. Two recent studies, Seyhan *et al.* and Yu *et al.*, suggested that miRNAs could be useful biomarkers to distinguish between subtypes of diabetes; however, the number of LADA patients in these two studies was limited (15, 16). Thus, it is still not clear whether miRNAs profiles can identify individuals at risk of developing LADA and if characterization of miRNAs could lead to a better differentiation between LADA, type 1 and type 2 diabetes. This study aimed to explore the notion that circulating small RNAs (sRNAs) in serum could be differentially expressed between three main subgroups of diabetes, including adult-onset type 1 diabetes and LADA as autoimmune diabetes and non-autoimmune type 2 diabetes and between the subgroups of patients with diabetes and non-diabetic controls. For this purpose, we used the data from the Trøndelag Health Study (HUNT).

## Methods

### Study population

In this cross-sectional case–control study, we included participants from the third HUNT survey (HUNT3, 2006–2008, *n*  = 50 800, response rate 54.1%). Details about HUNT3 are available elsewhere (17). Study participants were categorized into four groups, including three groups with self-reported diabetes: LADA (*n* = 51), adult-onset type 1 (*n* = 51) and type 2 diabetes (*n* = 50), and one group with self-reported non-diabetic participants serving as controls (*n* = 51). The clinical characteristics of the four groups are shown in Table 1. More information regarding the selection of study population and diabetes classification is described in Supplementary methods (see section on supplementary materials given at the end of this article). Power calculations using *RnaSeqSampleSize* in R showed that with group sizes of 50, our sequencing data has a power of 0.91 when using a minimum fold change of 1.5 and a false discovery rate threshold of 0.05.

**Table 1:** Descriptive statistics of study participants. The *P*-values represent the statistical differences between the groups for the specific variables after multivariable adjustment. Data are presented as mean ± s.d. or as median (IQR).

Variable	Non-diabetic controls	Type 2 diabetes	LADA	Type 1 diabetes	*P*-value
*n*	51	50	51	51	
Males, *n* (%)	24 (47)	28 (56)	27 (53)	28 (55)	1
Age at participation (years)	52.8 ±15.7	63.7 ± 11.2	67.9 ± 9.3	59.8 ± 11.8	0.8
Age at diabetes diagnosis (years)	NA	55.9 ± 10.9	55.1 ± 10.1	44.9 ± 11.7	0.04
Diabetes duration (years)	NA	5.8 (3.2–10.7)	12.6 (4.8–20.3)	13.1 (6.9–22.6)	0.008
BMI (kg/m^2^)	27.9 ± 4.2	30.6 ± 4.8	29.0 ±4.5	27.4 ± 3.6	0.3
Waist circumference (cm)	96.8 ± 13.5	103.6 ± 12.4	100.4 ± 11.6	94.4 ± 9.2	0.1
Systolic blood pressure (mm Hg)	129 ± 19	140 ± 19	137 ± 22	133 ± 16	1
Cholesterol (mmol/L)	5.4 ± 1.1	5.1 ± 0.9	4.9 ± 0.8	4.7 ± 1.0	0.4
HDL cholesterol (mmol/L)	1.3 ± 0.4	1.2 ± 0.3	1.3 ± 0.4	1.4 ± 0.3	0.3
Triglycerides (mmol/L)	1.6 ± 0.8	2.4 ± 1.2	2.1 ± 1.6	1.2 ± 0.7	0.003
Non-fasting glucose (mmol/L)	5.4 ± 0.9	8.5 ± 2.8	9.9 ± 4.1	9.8 ± 4.8	1
HbA1c (% HbA1c)	NA	7.2 ± 1.4	7.3 ± 1.2	8.2 ± 1.2	0.07
HbA1c (mmol/mol)	NA	55 ± 15	56 ± 13	66± 13	0.07
Fasting C-peptide (nmol/L)	NA	0.90 ± 0.42	0.60 ± 0.43	0.08 ± 0.14	9e-15
GADA titer (ai)	0.00 (0.00–0.01)	0.00 (0.00–0.01)	0.34 (0.11–1.11)	0.23 (0.02–0.98)	0.0001

### RNA isolation and sequencing

Total RNA was isolated from 100 µL of serum using the miRNeasy Serum/Plasma Kit (Qiagen, Cat. No./ID: 217184). The serum samples were stored at −80°C at the HUNT biobank prior to RNA isolation and the RNA was isolated immediately prior to sequencing.

Small RNA library preparation was performed using the NEXTflex sRNA-seq kit v3 (Bio Scientific, Austin, TX, USA) using 10.5 µL total RNA. In the first ligation step, ten calibrator RNAs were mixed with the RNA to control for technical variation during the data analysis.

Single read sequencing (sRNA-seq) was performed for 51 cycles on one HiSeq4000 flow cell, according to the manufacturer's instructions. (Illumina, Inc., San Diego, CA, USA). More detailed information regarding RNA isolation, library preparation and sequencing are described in Supplementary methods.

### Data processing

The raw sequencing data were processed as previously described (18), in addition to removing the random nucleotides associated with the NEXTFLEX sRNA library preparation kit. For more information on how this was performed see Supplementary methods. The databases miRBase (19) and RNACentral (20) were used for miRNA and other small ncRNAs, respectively.

### Statistical analysis

The correlation of clinical variables with the diabetes groups was examined using the *manova* function in R combined with *summary.aov* to extract the *P*-values. The *P*-values were adjusted for multiple testing using the Bonferroni method.

Differentially expressed sRNAs between the groups were detected using the limma-voom procedure in R (21). Calibrator RNAs were used for normalization by using the CalcNormFactors from on the calibrator matrix. For the differential expression analysis, a design matrix was created with the groups being compared, as well as the variables age, sex and BMI as additional covariates. An example of the limma-voom procedure for isomiR is show in Supplementary methods.

Small RNAs with a benjamini-hochberg adjusted *P*-value < 0.05 were considered as differentially expressed. For mature miRNAs, we required an expression of at least 1 count-per million (cpm) in all the samples. For isomiRs and other small ncRNAs, we required an expression of at least 1 cpm in 50% of the samples. The reason for the less conservative filter for the isomiRs and sRNAs is that isomiRs by definition are less expressed than their corresponding mature miRNAs, since the mature miRNAs are the sum of all corresponding isomiRs. Furthermore, sRNAs are also less expressed than the miRNAs since our protocols aim for miRNAs. Applying too strict filters will risk removing true positive sRNAs that are for instance absent in one particular sample. The heatmaps were created using the *pheatmap* function in R for which the expression values were normalized first by cpm-log_2_ normalization followed by z-score normalization. The leave-one-out cross-validation (LOOCV) on the signatures was conducted in R by performing feature selection in *limma* for each iteration followed by selecting the significant sRNAs after benjamini-hochberg adjustment, which was then used to predict the left-out sample. The predicted values were then used as input to the *roc* function in R from the *pROC* package. The specificities- and sensitivities-vectors from the *roc* function were used to plot the ROC-curves using *ggplot2*. The confidence intervals for the AUC were extracted from the *ci.auc* function and the *P*-values were calculated from the *roc.area* function. The LOOCV model is available at https://github.com/MjelleLab/Leave-one-out-cross-validation. The expression of miRNAs and other sRNAs can be browsed in the following shiny app: https://mjellelab.shinyapps.io/shiny_diabetes_sRNA/.

Gene enrichment analysis were performed using *clusterProfiler* in R by using mRNA targets predicted by TargetScan. Different context score thresholds from TargetScan were tested.

## Results

### Sequencing statistics and clinical variables

On average, 12 million reads were sequenced across all 203 samples (Table 1) of which about 1 million aligned to miRNAs and 1 million aligned to other sRNAs (Supplementary Figs 2 and 3). miRNAs were the most abundant class of sRNAs, followed by tRNAs and ribosomal RNAs (Supplementary Figs 2 and 4). 70 miRNAs and 128 isomiRs were consistently expressed in all samples. A multivariable analysis was performed to examine differences in the variables between groups (Table 1). Mainly GADA, C-peptide and diabetes duration differed between the four groups.

### Circulating miRNAs and isomiRs expression differ between autoimmune and non-autoimmune diabetes and between types of diabetes and non-diabetic controls

**Figure 1:**
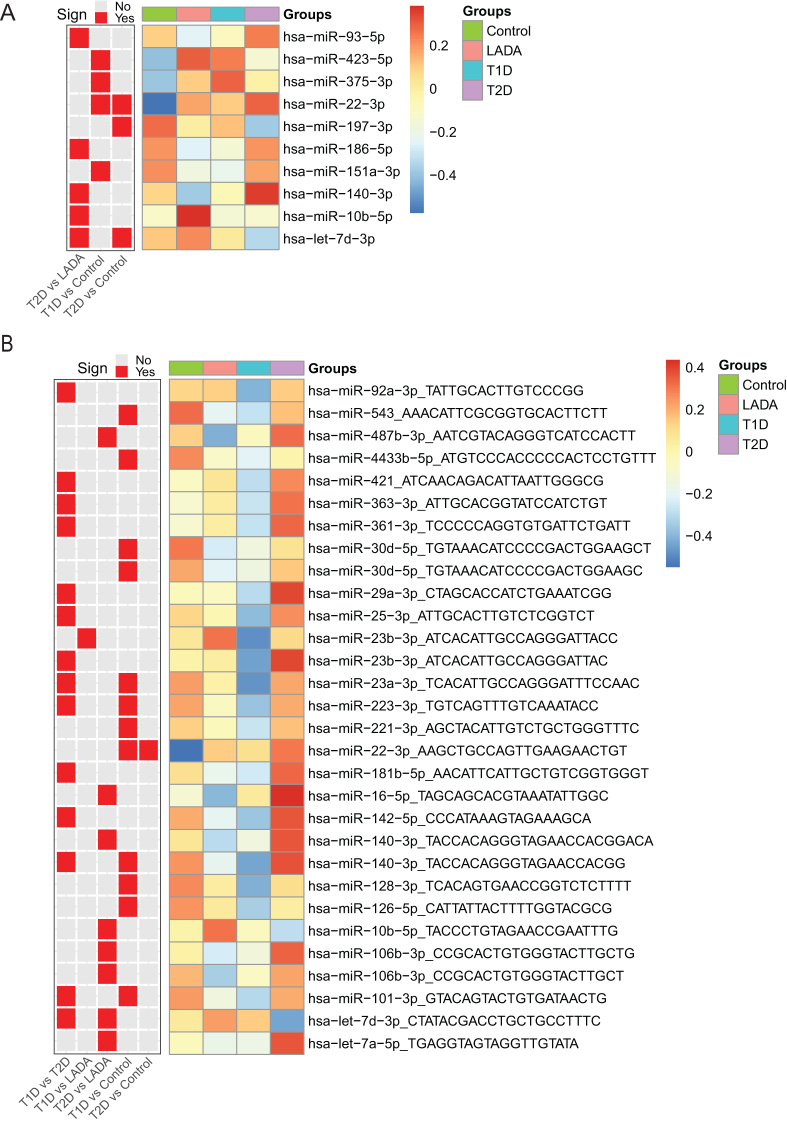
(A) Differentially expressed mature miRNAs. Shown are miRNAs that were significantly detected as differentially expressed between the groups using limma-voom. The heatmap shows the average z-score normalized expression of the miRNAs within the groups. The comparisons for which the different miRNAs were detected as significant are shown as a separate heatmap in which red and gray color indicate that the miRNA difference is significant and non-significant, respectively. (B) Similar as in (A) for isomiRs. The isomiR-sequence for the different miRNAs is indicated as part of the miRNA ID. See (A) for explanation of the heatmaps.

We performed differential expression analysis on both miRNAs and isomiRs and observed significant differences between the groups. First, we observed differences in expression of mature miRNAs between type 2 diabetes and controls, between type 2 diabetes and LADA and between type 1 diabetes and controls (Fig. 1A and Supplementary Table 1). The miRNA miR-22-3p was downregulated in controls compared to type 1 diabetes and type 2 diabetes and lower in LADA compared to controls. Most differences were found between type 2 diabetes and LADA and between type 1 diabetes and controls with five and four differentially expressed miRNAs, respectively. To investigate if time from diagnosis to samples collection could affect the results for the three diabetes groups, we included this variable as a covariate in the analysis. We did not observe any major changes in the differentially expressed miRNAs after including this covariate, and the miRNAs that were significant before were still significant, albeit with slightly higher *P*-values (Supplementary Tables 1 and 2). Finally, we tested if the mRNA targets of the significant miRNAs were enriched for specific gene ontology terms; however, no significant enrichments were observed.

Next, focusing on isomiRs, we detected 30 differentially expressed isomiRs, originating from 26 different mature miRNAs (Fig. 1B and Supplementary Table 2). We observed significant differences of five of the comparisons, of which type 1 diabetes vs type 2 diabetes showed the largest differences with 14 differentially expressed isomiRs. For the type 1 diabetes vs type 2 diabetes comparison, 13 of the 14 significant isomiRs were higher expressed in type 2 diabetes compared to type 1 diabetes.

### Decreased expression of tRNA-gly in diabetes

**Figure 2:**
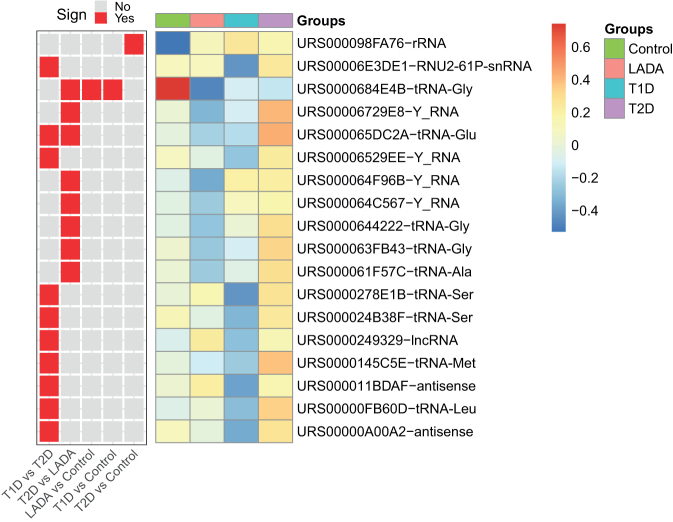
Differentially expressed other sRNAs. Shown are sRNAs that were significantly detected as differentially expressed between the groups using limma-voom. The IDs correspond to the RNACentral IDs. The comparisons for which the different sRNAs were detected as significant are shown as a separate heatmap in which red and gray color indicate that the sRNA difference is significant and non-significant, respectively.

Having established that several miRNAs and isomiRs were differentially expressed between the study-groups, we went on analyzing other sRNAs that were detected in the serum samples. We detected 18 differentially expressed sRNAs across all comparisons (Fig. 2 and Supplementary Table 3). The highest number of differentially expressed sRNAs were found between type 1 diabetes and type 2 diabetes and between type 2 diabetes and LADA with ten and eight significantly differentially expressed sRNAs for the two comparisons, respectively. In total, we detected nine differentially expressed tRNAs, four Y-RNAs, two antisense RNAs, one snRNA and one lncRNA. The most significant sRNA difference across all comparisons was for the tRNA URS0000684E4B, which encodes the amino acid glycine. The expression of this tRNA was strongly reduced in the diabetes groups compared to controls, in particular LADA, which showed the lowest expression. We investigated if the observed difference could be due to length differences in the sequencing libraries between the groups and observed only minor fragment-length differences between the groups, indicating that the expression differences are indeed related to the biology of the groups (Supplementary Figs 5 and 6). To further test the possibility that length differences could affect the differential expression of the tRNA, we updated the limma-voom model by including the total read abundance for the fragment lengths 30–33 nucleotides as a covariate, corresponding to the lengths of tRNAs in our data. The updated model showed that URS0000684E4B was still significantly downregulated in LADA vs controls when adjusting for the abundance of tRNAs in the dataset (*P* = 1.2e-05, benjamini-hochberg adjusted).

### MicroRNA and sRNA signatures discriminate diabetes subgroups and non-diabetic controls

**Figure 3:**
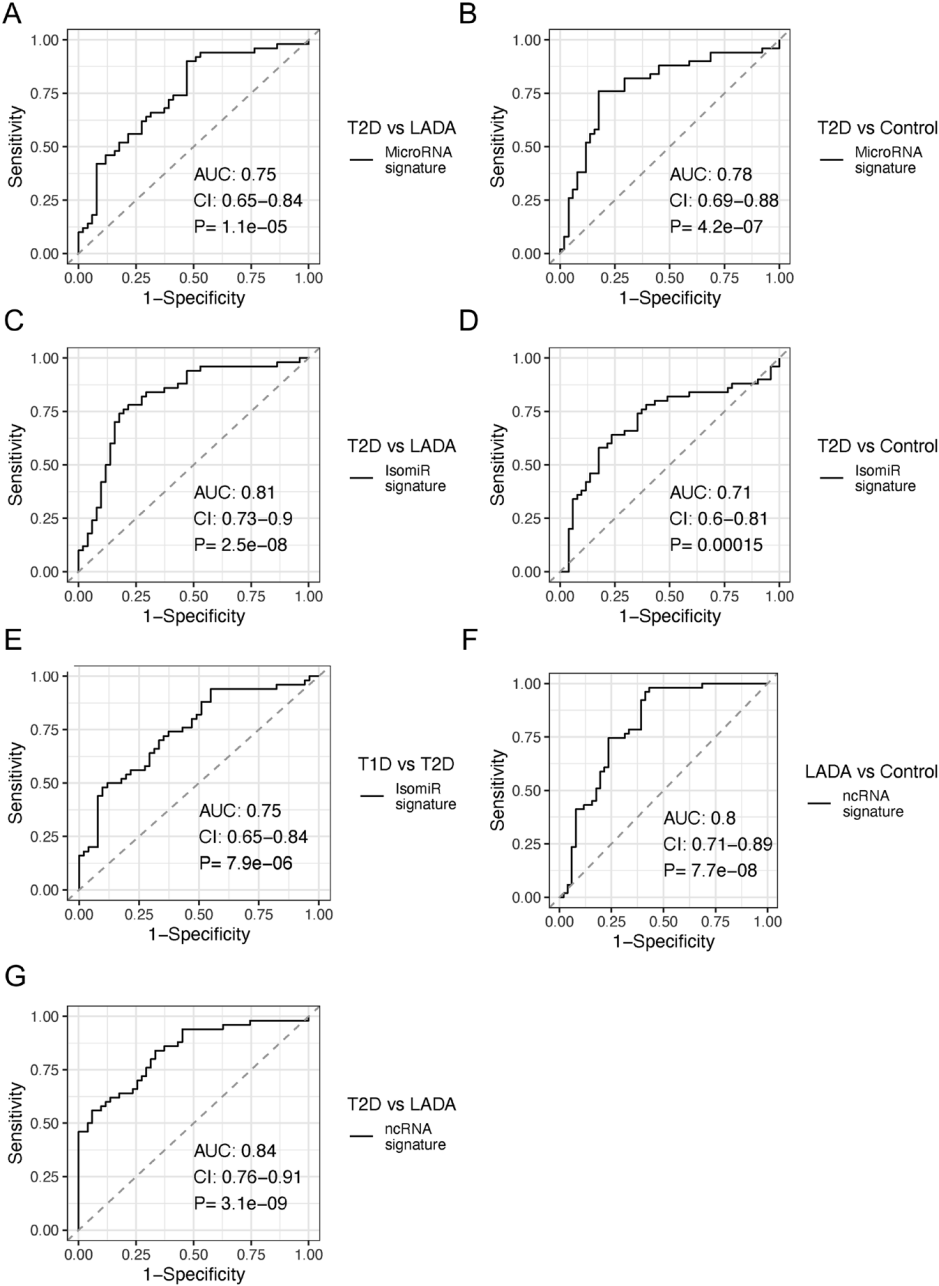
ROC-curves for the leave-one-out cross-validated models for the miRNA- (A and B), isomiR- (C, D and E) and other sRNA- (F and G) signatures. Shown are only signatures that passed the leave-one-out cross-validation. The signatures include those RNAs that were significant for the different group-comparisons after adjusting for multiple testing (see Methods for details on the leave-one-out cross-validation).

Having shown that multiple miRNAs are differentially expressed between different subgroups of diabetes and between diabetes and controls, we assessed the diagnostic potential of our newly identified miRNA, isomiRs and sRNAs. We combined the significant sRNAs for each specific group comparison into signatures and evaluated their ability to separate the different groups in our data set. That is, each signature consists of all the sRNAs that were significant for the different comparisons using limma-voom. To test the ability of the models to correctly classify new samples, we performed LOOCV and considered validated signatures with an area under the curve (AUC) value above 0.7 to have a predictive value (see Methods). In total, we tested three signatures for the mature miRNAs, five for isomiRs and five signatures for other sRNAs, corresponding to the comparisons for which we had significant results. Prior to the LOOCV, all the tested signatures showed AUC-values above 0.7. After the LOOCV, we identified two miRNA-signatures and three isomiR signatures with an AUC above 0.7. The two miRNA signatures separated type 2 diabetes from LADA (AUC = 0.75 (0.65–0.84), *P*-value = 1.1 × 10^−5^) and type 2 diabetes from controls (AUC = 0.78 (0.69–0.90), *P*-value = 4.2 × 10^−7^) (Fig. 3A and B). The three isomiR signatures separated type 2 diabetes from LADA (AUC = 0.81 (0.73–0.9), *P*-value = 2.5 × 10^−8^), type 2 diabetes from controls (AUC = 0.71 (0.60–81), *P*-value = 1.5 × 10^−4^) and type 1 diabetes from type 2 diabetes (AUC = 0.75 [0.65–0.84], *P*-value = 7.9 × 10^−6^) (Fig. 3C, D and E).

Next, given the pronounced difference in expression of other sRNAs between many of the groups, we investigated the diagnostic ability of the other sRNAs in classifying diabetes. Similar as for miRNAs, sRNAs that were significant for the specific group-comparisons were included in the signature. We identified two significant signatures after LOOCV, one that separated LADA from controls (AUC = 0.80 (0.71–89), *P*-value = 7.7 × 10^−8^) and one that separated type 2 diabetes and LADA (AUC = 0.84 (0.76–91), *P*-value = 3.1 × 10^−9^) (Fig. 3F and G). The glycine tRNA was included in all LOOCV-signatures for the LADA vs controls comparison, indicating a diagnostic potential. Taken together, these results show that not only miRNAs but also other sRNAs, such as tRNAs, can differentiate between diabetes subgroups.

## Discussion

Our study is the first to perform comprehensive sRNA-seq on all three major diabetes subgroups simultaneously in addition to non-diabetic controls. In this study we demonstrate that several sRNAs are differentially expressed in serum of diabetes patients and therefore might be useful to distinguish between diabetes and non-diabetic controls and partly between subgroups of diabetes.

The miRNA miR-22-3p was more highly expressed in all diabetes groups compared to the control group. A study in rats showed that upon 3,5-diiodo-l-thyronine (T2) treatment, a drug shown to have beneficial effect on glucose tolerance and insulin-resistance, serum levels of miR-22-3p decreased compared to rats not receiving T2 treatment. This finding is in line with our results in the way that T2 treated rats and non-diabetic controls in our study represent a healthier state, with lower miR-22-3p levels (22). In addition to miR-22-3p, the miRNAs miR-423-5p and miR-375-3p were also more highly expressed in all diabetes groups compared to control, although differences in these miRNAs were only significant in type 1 diabetes vs controls.

Seyhan *et al.* identified up-regulation of miR-375 in type 1 diabetes compared to LADA and non-diabetic controls (15). This finding is in concordance with our study; however, other studies have shown downregulation or unchanged levels of miR-375 (23), indicating the variability of miRNA expression in serum and that different methods and study population could affect which sRNAs are detected as differentially expressed. Anyway, miR-375 is shown to be enriched in human islet cells (24), a regulator for insulin secretion (25) and increased miR-375 in the blood is shown to be associated with beta-cell death in mice (26).

In general, we observed larger differences between type 2 diabetes and LADA than between type 1 diabetes and LADA. This could be explained by the fact that both type 1 diabetes and LADA are autoimmune diseases and that the same sRNAs are affected in both diseases, but also lack of power, although our power calculation indicates good power with current sample sizes. The only sRNA that significantly separated LADA from type 1 diabetes was an isomiR of miR-23b-3p which was more highly expressed in LADA compared to type 1 diabetes. The miR-23b-3p is enriched in human beta-cells and shown to be downregulated in cytokine (IL-1beta and TNF-gamma) treated beta-cells and potentially involved in beta-cells death (27). This makes the miR-23b-3p a potential biomarker for differentiating LADA from type 1 diabetes; however, this needs to be tested in future studies with larger groups. We were not able to create a predictive signature that separated LADA from type 1 diabetes, indicating that these groups are too similar with respect to serum sRNA expression to be distinguished with high confidence.

By performing sRNA-seq, we were able to measure other sRNAs in addition to miRNAs. Among the many groups of sRNAs, we detected significant sRNA differences in several of the comparisons. The most striking difference was the pronounced depletion of a glycine tRNA within the three diabetes groups. The highest depletion was detected in the LADA group; however, all three diabetes groups showed significant down-regulation of this tRNA compared to the control group. This tRNA is located within an intron of the ULK2 gene. No previous literature supports a role for this particular tRNA in diabetes; however, tRNAs are emerging as a novel group of molecules playing a role in diabetes-related processes such as glucose metabolism, pancreatic β-cell function, obesity and insulin resistance in type 2 diabetes (28). Dysregulation of amino acids in diabetes is a known phenomenon where for instance glycine has been shown to be reduced in patients with diabetic ketoacidosis (29). Indeed, several studies have observed reduced levels of plasma glycine in patients with obesity and diabetes (30, 31, 32, 33, 34). Other studies have reported difference in dysregulation of amino acids such as elevated levels of Val, ILe and Leu in impaired fasting glucose (IFG) or T2D (35, 36). Patients with impaired glucose tolerance and type 2 diabetes are shown to have lower levels of certain amino acids such as arginine and glutamine (37). Together, dysregulation of amino acids appears to be a common phenomenon in diabetes and our finding of a dysregulated tRNA coding for glycine supports current literature and could explain some of the previously observed changes at the amino acid level.

One possible limitation of this study is that the diabetes diagnosis is based on self-report. However, the self-reported diagnosis of diabetes in HUNT has previous been shown to have good validity (38). Another limitation is the lack of complete clinical measurements for all relevant variables. For C-peptide, we only have measurements for 94 patients and none of the controls. miRNA that are correlated with the missing variables could potentially be false positives. However, we do have complete measurement for BMI which is considered an important clinical variable in diabetes studies. Several studies do not adjust for age, sex and BMI in the analysis. BMI may be particularly important as this phenotype shares many of the same inflammatory responses seen in diabetes and many miRNAs are shown to be differentially expressed in obese individuals (39). One could argue that we should had adjusted for other risk factors for diabetes such as physical activity and diet which might also influence the expression of small RNAs. However, these risk factors are hard to measure in a valid way, and we lack accurate measures of these risk factors in HUNT. Nonetheless, much of the excess risk of diabetes associated with low physical activity and poor diet is likely mediated through BMI.

No consensus on the circulating miRNA profile for diabetes has been established and findings between most previous studies show little or no overlap. The lack of consistency between studies could have several reasons. First, the date of sample collection relative to the diagnosis date is not the same between studies, meaning that the phenotype of the patients, such as the degree of beta-cell death, may not be comparable between studies, leading to different sets of miRNAs being detected as differentially expressed. It should be noted that our serum samples were collected several years after diabetes diagnosis (at a median time of 7.4 years) and therefore not necessarily reflect the differences at the time of diagnosis. Moreover, high-throughput sequencing like many other technologies, such as RT-qPCR and microarray, has different biases that could affect the results.

In conclusion, by performing sRNA-seq on serum from diabetes patients we identified several miRNAs, isomiRs and other sRNAs that are differentially expressed between patients with diabetes and non-diabetic controls and between LADA and type 2 diabetes. Moreover, we describe a novel tRNA fragment encoding for glycine that could potentially be used as a diagnostic tool in combination with other biomarkers.

## Supplementary Material

Supplementary Material

## Declaration of interest

Bjørn O Åsvold is on the editorial board of EJE. Bjørn O Åsvold1was not involved in the review or editorial process for this paper, on which he/she is listed as an author. The other authors have nothing to disclose.

## Funding

This project was funded by Liaison Committee between the Central Norway Regional Health Authority (RHA) and the Norwegian University of Science and Technology (NTNU) (grant 90154900) and The Joint Research Committee between St. Olavs Hospital and the Faculty of Medicine and Health Sciences, NTNU (FFU, grant 90502900).

## Data availability

Due to Norwegian privacy laws, sequencing data will not be uploaded to a publicly available repository. The data sets analyzed in the current study can be made available upon reasonable request to the corresponding author or HUNT Research Center (kontakt@hunt.ntnu.no, https://www.ntnu.edu/hunt/data).

## Author contribution statement

E P S conceptualized the project and R M performed the data analysis. E P S and R M drafted the manuscript and take full responsibility for the accuracy of the analyses. V B and A F contributed with the RNA sequencing. B O Å and V G were involved in project design. All authors contributed to the interpretation of the results and critically revised and approved the final version of the manuscript.
