# Supplementary material for: Small RNAs are differentially expressed in autoimmune and non-autoimmune diabetes and controls
Source: Eur J Endocrinol. 2022 May 26;187(2):231–40. doi: 10.1530/EJE-22-0083 (PMC9254298; doi:10.1530/EJE-22-0083)
Supplement: Supplementary Material [file supplementary_material.pdf]

Electronic supplementary material (ESM):

Small RNAs are differentially expressed in autoimmune and non-autoimmune diabetes and controls

Elin P. Sørgjerd[*,1,2], Robin Mjelle[*,3,4], Vidar Beisvåg[4,6], Arnar Flatberg[4,6], Valdemar Grill[4], Bjørn O. Åsvold[1,2,5]

*Equal contribution

1. HUNT Research Centre, Department of Public Health and Nursing, Faculty of Medicine and Health Sciences, NTNU, Norwegian University of Science and Technology, Levanger, Norway.

2. Department of Endocrinology, Clinic of Medicine, St. Olavs Hospital, Trondheim University Hospital, Trondheim, Norway.

3. Bioinformatics Core Facility—BioCore, Norwegian University of Science and Technology NTNU, 7491 Trondheim, Norway

4. Department of Clinical and Molecular Medicine, Faculty of Medicine and Health Sciences, NTNU, Norwegian University of Science and Technology, Trondheim, Norway.

5. K.G. Jebsen Center for Genetic Epidemiology, Department of Public Health and Nursing, Faculty of Medicine and Health Sciences, NTNU, Norwegian University of Science and Technology, Trondheim, Norway.

6. Central Administration, St. Olav's Hospital, Trondheim University Hospital, Norway

Table of Contents

Supplementary methods 3

Study population 3

RNA isolation and sequencing 3

Data processing 4

Example of the limma-voom procedure for isomiR 4

References 5

ESM Figure 1 6

ESM Figure 2 7

ESM Figure 3 8

ESM Figure 4 9

ESM Figure 5 10

ESM Figure 6 11

ESM Table 1 12

ESM table 2 22

ESM Table 3 140

Supplementary methods

Study population

The HUNT study is a longitudinal population-based study comprising all residents aged 20 years or older from the Nord-Trøndelag region in Norway. Four consecutive surveys have been conducted, HUNT1 in 1984-1986, HUNT2 in 1995-1997, HUNT3 in 2006-2008 and HUNT4 in 2017-2019. In this cross-sectional case-control study we included participants from the third HUNT survey (HUNT3, 2006-2008, n=50,800, response rate 54.1%). Details about HUNT3 are available elsewhere (1). In brief, the study included questionnaires, clinical examinations, and blood sampling. The biological samples were stored at -80℃

Participants with diabetes were identified by their self-reporting answer yes to the question "Do you have, or have you had diabetes?" (n=2264). In HUNT3 all participants with diabetes and a serum sample available were measured for GADA positivity (96%). They were also given a diabetes-related questionnaire which included questions about treatment (replied by 81%) and invited to a clinical investigation to provide fasting samples for measurements of HbA1c, glucose and C-peptide (attended by 55%). The data in HUNT3 was collected at a median of 7.4 (IQR 3.2-14.3) years after diabetes diagnosis.

Participants with self-reported diabetes were classified as having adult-onset type 1 diabetes (n=63) if they had age of diabetes diagnosis ≥30 years of age, started with insulin treatment within one year after diagnosis and were i) positive for GADA (≥0.08 ai) or ii) GADA negative and C-peptide values below 0.15 nmol/l. They were classified as having LADA (n=91) if age at diagnosis was ≥30 years of age and they were GADA positive and did not start on insulin treatment before one year after diagnosis. Criteria for type 2 diabetes (n=1545) were age at diagnosis ≥30 years of age and being GADA negative and having no insulin treatment before one year after diagnosis.

Possible non-diabetic controls were identified by answering no to the question of having diabetes (n=48,516). About 1481 non-diabetic controls had GADA measured in HUNT3 and 1422 were GADA negative. Participants with a non-fasting glucose value ≥11 mmol/l were excluded to reduce the possibility of including people with undiagnosed diabetes in the control group.

Fifty-five participants from each group where randomly selected by means of the Select Cases - Random sample of cases function in IBM SPSS Statistics (version 25). Serum was available from 51 patients with type 1 diabetes, 51 patients with LADA, 50 patients with type 2 diabetes and 51 GADA-negative non-diabetic controls.

Power was calculated using *RnaSeqSampleSize* in R using the functions *est_count_dispersion* with expression matrix and groups as input and *est_power_distribution* with the parameters n=51, m = 70, f=0.05, m1 = 10, rho=1.5 and repNumber=50. This showed that with group sizes of 50, our sequencing data has a power of 0.91 when using a minimum fold change of 1.5 and FDR threshold of 0.05.

RNA isolation and sequencing

RNA was isolated from 100 µl of serum using the miRNeasy Serum/Plasma Kit (Qiagen, Cat. No. / ID: 217184). For assessment of RNA quality and relative size, the samples were measured using Eukaryote

total RNA pico assay on the 2100 Bioanalyzer (Agilent Technologies, Santa Clara, CA, USA). RNA isolation from serum generally only consists of RNAs of lengths less than 200 nucleotides. For quality control we check for the presence of this peak on the bioanalyzer trace. RIN values are not considered since ribosomal RNA are degraded in serum and plasma. See ESM Figure 1 for a representative bioanalyzer trace for our samples.

Small RNA sequencing (sRNA-seq) of 203 samples/libraries were performed using the NEXTflex sRNA-seq kit v3 (Bio Scientific, Austin, TX, USA). The adapter-dimer reduction technology incorporated into this kit allows low input library preparation. Reducing ligation-associated bias involves the use of adapters with randomized bases at the ligation junctions, resulting in greatly decreased bias in comparison to standard protocols. In brief, 10.5 µl total RNA, extracted from 100 µl serum, was used as a template for 3' 4N and 5' 4N adenylated adapter ligation, followed by reverse transcription-first strand synthesis. In the first ligation step, 10 calibrator RNAs were mixed with the RNA to control for technical variation during the data analysis. The sequences of the calibrators are previously descried by our group (2). By applying these products as a template for second-strand synthesis, double-stranded cDNA was prepared by PCR amplification (22 cycles). Fragments/libraries were run on a Labchip GX (Caliper Life Sciences, Hopkinton, MA, USA), for quality control and quantitation. Individual libraries were normalized to 25 nM and pooled. The library pool was purified with the QIAquick PCR Purification Kit (Qiagen AB, Sweden) according to instructions. Automated size selection was performed using the Blue Pippin (Sage Science, Beverly, MA, USA), with a range of 135-165 bp to select the ~ 152 bp fragment. Following size selection, the pool was evaluated on Bioanalyzer (Agilent Technologies, Santa Clara, CA, USA) using the High Sensitivity DNA kit. The pool of libraries was quantified with the KAPA Library Quantification Kit (Roche, Pleasanton, CA, USA).

Libraries were normalized to 2.6 pM subjected to clustering. Single read sequencing was performed for 51 cycles on one HiSeq4000 flow cell, according to the manufacturer's instructions. (Illumina, Inc., San Diego, CA, USA). Sequence reads were demultiplexed and converted from BCL to fastq file format using bcl2fastq2 conversion software V2.20.0422 (Illumina, Inc. San Diego, CA, USA).

Data processing

The raw sequencing data was processed as previously described (3), in addition to removing the random nucleotides associated with the NEXTFLEX sRNA library preparation kit. Specifically, the adapters were removed using cutadapt (v.3.7) followed by alignment to the human genome (hg38) using bowtie2 (v2.4.5). The aligned reads were counted using htseq-count (v2.0) with the corresponding GFF files from miRBase and RNACentral. Expression matrices for mature miRNAs, their isomers (isomiRs) and other small non-coding RNAs (ncRNAs) were constructed from the htseq-count output by combining the individual expression data for each sample and used for statistical analyses in R.

Example of the limma-voom procedure for isomiR

Differentially expressed sRNAs between the groups were detected using the limma-voom procedure in R. The limma-voom procedure for the comparison LADA vs T1 for isomiR is exemplified below:

```
data.dge <- DGEList(data)

keep <- rowSums(data.dge$counts>1) >= dim(data.dge)[2]/2

data.dge <- data.dge[keep,]

data.dge <- calcNormFactors(data.dge, method="TMM")

data.dge$samples$norm.factors <- Calibrator.dge$samples$norm.factors #add spike-ins

des <- model.matrix(~0+factor(clinical$Group)+ clinical$Sex+ clinical$Age+ clinical$BMI)

v <- voom(Sorgjerd.exp.dge,plot = T,design = des)

fit <- lmFit(v, design=des)

contrasts <- makeContrasts(cond=LADA-T1, levels=des)

fit2 <- contrasts.fit(fit, contrasts=contrasts)

fit2 <- eBayes(fit2)

topTable(fit2, coef="cond",sort.by="P", adjust.method="BH",n=Inf)

#data=expression matrix

#clinical=samplesheet with clinical data

#Calibrator.dge=calibrator DGE-object
```

References

1. Krokstad S, Langhammer A, Hveem K, Holmen TL, Midthjell K, Stene TR, et al. Cohort Profile: the HUNT Study, Norway. Int J Epidemiol. 2013;42(4):968-77.
2. Mjelle R, Dima SO, Bacalbasa N, Chawla K, Sorop A, Cucu D, et al. Comprehensive transcriptomic analyses of tissue, serum, and serum exosomes from hepatocellular carcinoma patients. BMC Cancer. 2019;19(1):1007.
3. Mjelle R, Sellaeg K, Saetrom P, Thommesen L, Sjursen W, Hofsli E. Identification of metastasis-associated microRNAs in serum from rectal cancer patients. Oncotarget. 2017;8(52):90077-89

ESM Figure 1

ESM Figure 1: A representative bioanalyzer trace for one of the samples. The y-axis shows the intensity and the x-axis the RNA fragment length. The 25nt peak is the bioanalyzer marker.

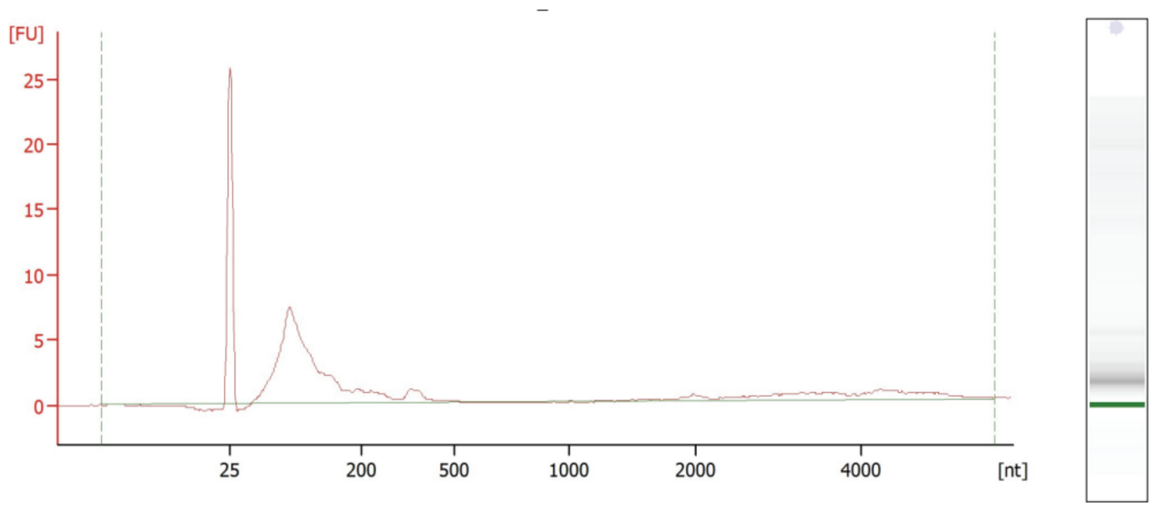

RNA Area:	73,0	RNA Integrity Number (RIN):	2.4 (B.02.08)
RNA Concentration:	120 pg/µl	Result Flagging Color:	
rRNA Ratio [28s / 18s]:	0,0	Result Flagging Label:	RIN: 2.40

ESM Figure 2

ESM Figure 2: Processing statistics per group. The plot shows the average number of reads for all samples within the four groups. Shown is the number of raw reads “Library size”; Alignments results “Alignment”; number of reads overlapping either miRBase or RNACentral database “Features”, and number of reads overlapping the main sRNA classes “RNAs”.

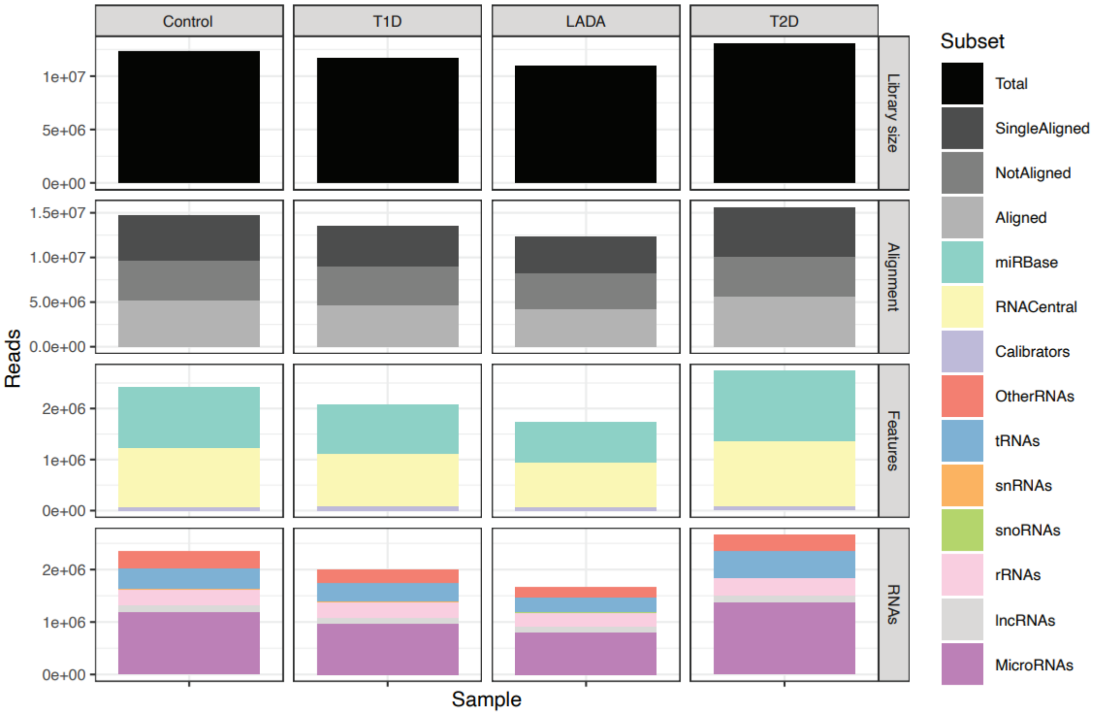

ESM Figure 3

ESM Figure 3: Boxplot showing the expression (log2) of calibrator RNAs per group. Anova p-values for the difference in calibrator RNA across the groups.

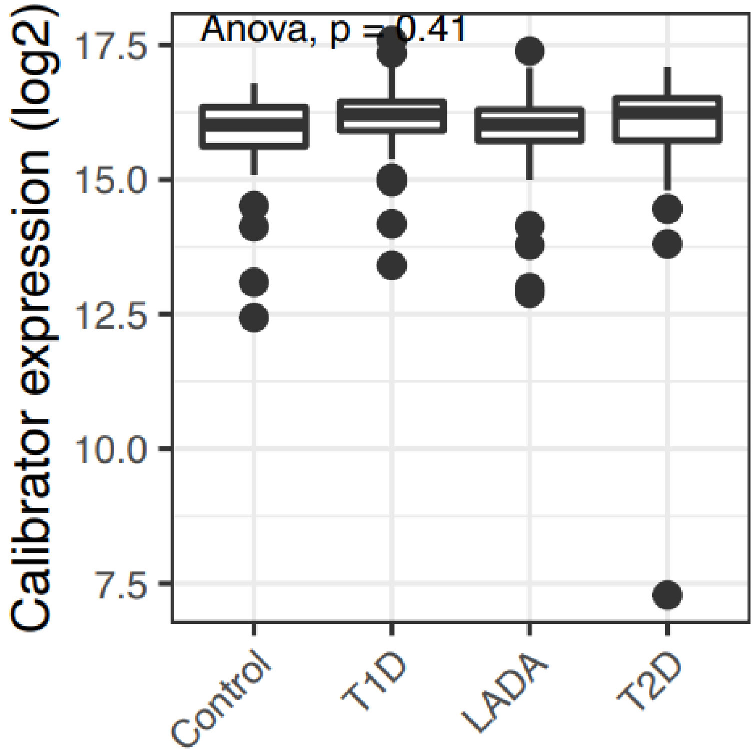

ESM Figure 4

ESM Figure 4: Abundance of the different RNA-classes shows as ratios.

B

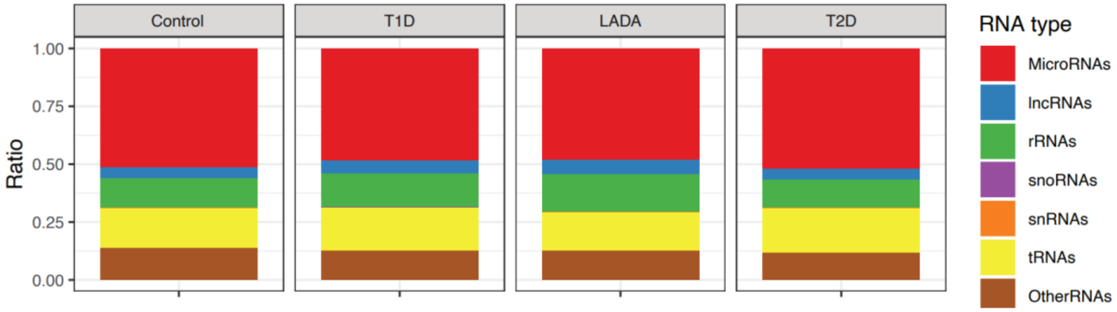

ESM Figure 5

ESM Figure 5: Fragment lengths for the sequencing libraries per group. The y-axis shows the fragment length of the raw reads after adapter trimming.

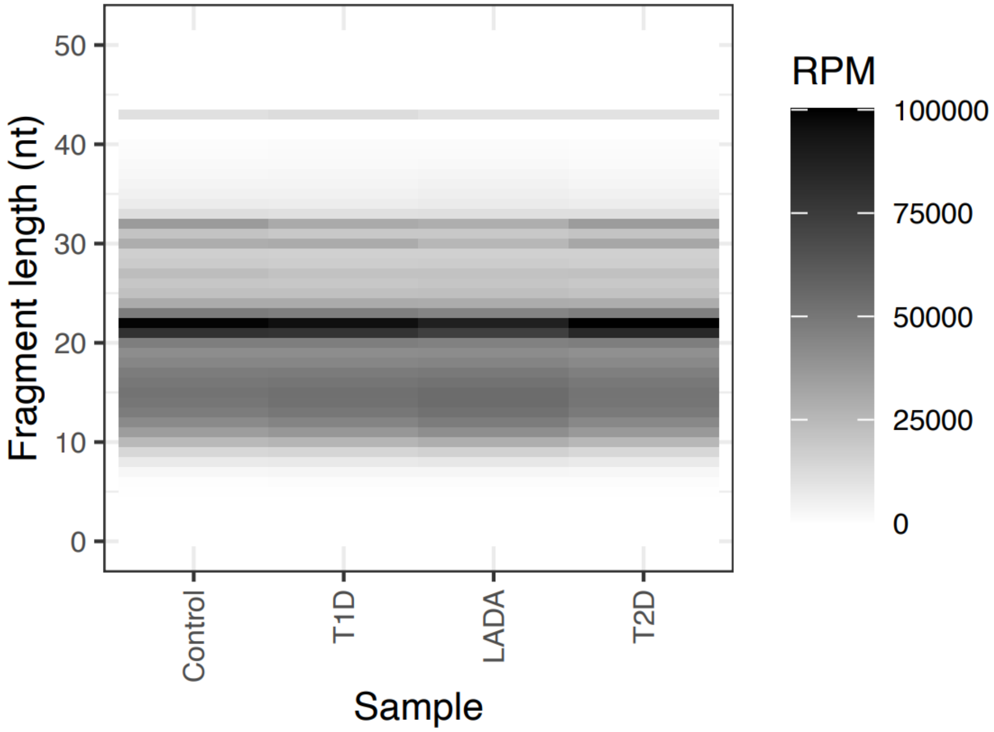

ESM Figure 6

ESM Figure 6: Fragment length of reads per million (rpm)-normalized reads per group.

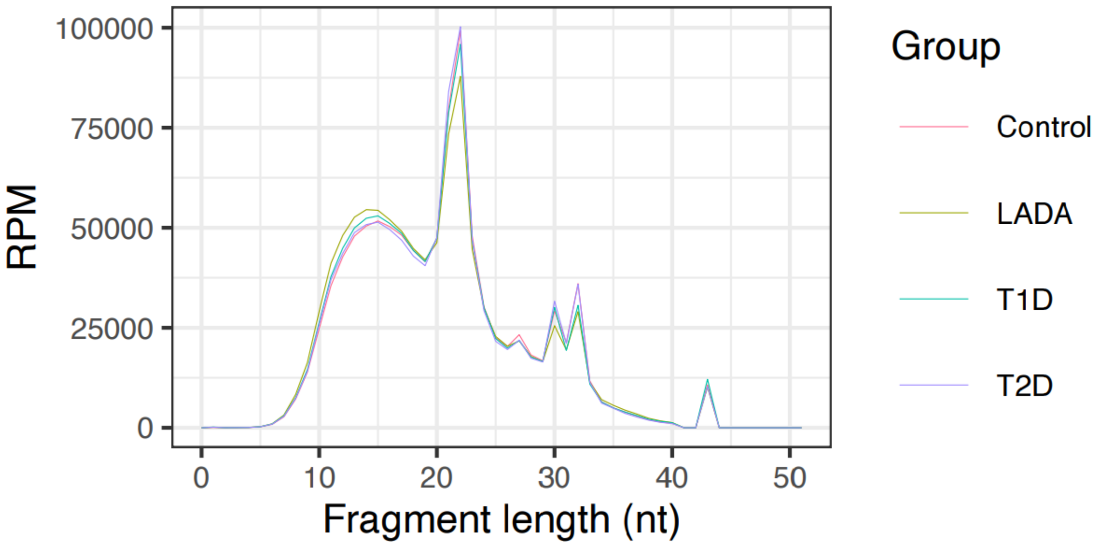

ESM Table 1

ESM Table 1: Results from differentially expression analysis of mature miRNAs. The comparions are indicated in the column "Comparison" such that a miRNA with positive "logFC" value in T1D vs T2D means that the miRNA is up-regulated in T1D vs T2D. "miRNA" indicate the miRNA name; "logFC" in the log2 fold change from limma; "Average Expression" is the average expression of the miRNA across all samples; "P-Value" is the P-value for the specific miRNA for the specific compariosn before multiple testing; "Adjusted P-Value" is the benjamini-hochberg adjusted P-value. Time-adjusted comparisons is the results from limma-voom with time from diagnosis to serum collections as a covariate. Time-adjusted comparisons is the results from limma-voom with time from diagnosis to serum collections as a covariate.

miRNA	logFC	Average Expression	P-Value	Adjusted P-Value	Comparison
hsa-miR-197-3p	0,435762218	10,71414886	0,001175057	0,073369896	T1D vs T2D
hsa-let-7d-3p	0,234140574	12,74297653	0,002096283	0,073369896	T1D vs T2D
hsa-miR-186-5p	-0,27360864	9,753658884	0,006856007	0,128649148	T1D vs T2D
hsa-miR-151a-3p	-0,422643317	9,101827398	0,009104535	0,128649148	T1D vs T2D
hsa-miR-423-5p	0,187958355	15,38689191	0,009189225	0,128649148	T1D vs T2D
hsa-let-7e-5p	-0,361254204	8,239214195	0,037592856	0,353638925	T1D vs T2D
hsa-miR-93-5p	-0,222852274	10,25947632	0,039134201	0,353638925	T1D vs T2D
hsa-miR-146b-5p	-0,234479974	9,113840715	0,041755416	0,353638925	T1D vs T2D
hsa-miR-140-3p	-0,262771072	9,550538435	0,049215672	0,353638925	T1D vs T2D
hsa-miR-425-5p	-0,157628504	11,65414966	0,050519846	0,353638925	T1D vs T2D
hsa-miR-361-5p	-0,236283238	7,743245637	0,065627724	0,417630969	T1D vs T2D
hsa-miR-363-3p	-0,252101039	9,933281027	0,077536343	0,452295333	T1D vs T2D
hsa-miR-125b-5p	0,208132092	10,46377314	0,095798262	0,494188817	T1D vs T2D
hsa-miR-375-3p	0,348752047	10,48979782	0,10291788	0,494188817	T1D vs T2D
hsa-miR-30c-5p	-0,171591128	10,77418095	0,113234747	0,494188817	T1D vs T2D
hsa-miR-30d-5p	-0,105517184	14,45578756	0,116796159	0,494188817	T1D vs T2D
hsa-miR-320a-3p	0,158064193	12,07431198	0,123204575	0,494188817	T1D vs T2D
hsa-miR-451a	-0,221047203	16,82769003	0,127077124	0,494188817	T1D vs T2D
hsa-miR-146a-5p	-0,143432354	12,47099261	0,14851127	0,547146786	T1D vs T2D
hsa-miR-92a-3p	0,145090581	17,46006468	0,159860913	0,55905155	T1D vs T2D
hsa-miR-484	-0,202573405	11,45694783	0,167715465	0,55905155	T1D vs T2D
hsa-miR-27b-3p	-0,151925751	10,06790199	0,183257904	0,566824722	T1D vs T2D
hsa-miR-16-5p	-0,14304869	14,33881148	0,186242409	0,566824722	T1D vs T2D
hsa-miR-30a-5p	-0,126535827	10,38201877	0,2044692	0,589215248	T1D vs T2D
hsa-miR-142-5p	-0,128069915	11,41969176	0,210434017	0,589215248	T1D vs T2D
hsa-miR-223-3p	-0,19484522	11,99339426	0,219265531	0,590330275	T1D vs T2D
hsa-miR-126-3p	-0,126146012	11,77700488	0,274336402	0,711242523	T1D vs T2D
hsa-miR-148a-3p	-0,108629294	9,865728499	0,316348691	0,718469097	T1D vs T2D
hsa-miR-181a-5p	-0,080343243	10,83967257	0,3443412	0,718469097	T1D vs T2D
hsa-miR-486-5p	0,135709324	18,27283556	0,344465062	0,718469097	T1D vs T2D
hsa-miR-182-5p	-0,14579728	9,621258824	0,344769065	0,718469097	T1D vs T2D
hsa-miR-4433b-5p	0,266127044	10,44673565	0,348993948	0,718469097	T1D vs T2D
hsa-miR-25-3p	-0,133972664	12,72047871	0,352850636	0,718469097	T1D vs T2D
hsa-miR-125a-5p	-0,092579768	12,04595729	0,367044008	0,718469097	T1D vs T2D
hsa-miR-23a-3p	0,072840418	12,54260289	0,367521839	0,718469097	T1D vs T2D
hsa-miR-15b-5p	0,091202876	9,98101658	0,369498393	0,718469097	T1D vs T2D
hsa-let-7b-5p	0,060678328	14,2837785	0,382698196	0,724023614	T1D vs T2D
hsa-let-7g-5p	-0,060843352	12,37806536	0,428315816	0,777459859	T1D vs T2D
hsa-miR-10b-5p	0,074379644	11,92933233	0,472315501	0,777459859	T1D vs T2D
hsa-miR-222-3p	-0,092151381	9,659340777	0,477489118	0,777459859	T1D vs T2D
hsa-miR-122-5p	-0,165038736	13,99900332	0,479380346	0,777459859	T1D vs T2D
hsa-miR-21-5p	0,055345941	13,68926132	0,493274422	0,777459859	T1D vs T2D
hsa-miR-101-3p	-0,079613152	9,504552453	0,493851947	0,777459859	T1D vs T2D
hsa-miR-27a-3p	0,07400398	10,10512206	0,49548366	0,777459859	T1D vs T2D
hsa-miR-24-3p	0,052472449	13,21412419	0,504348299	0,777459859	T1D vs T2D
hsa-miR-98-5p	0,113129231	8,792738384	0,510902193	0,777459859	T1D vs T2D
hsa-let-7c-5p	-0,097122358	8,230590245	0,52503514	0,78196723	T1D vs T2D
hsa-miR-26b-5p	-0,085767471	10,23866557	0,562748945	0,80612987	T1D vs T2D
hsa-miR-22-3p	-0,045959978	12,67951126	0,564290909	0,80612987	T1D vs T2D
hsa-miR-143-3p	-0,062085096	10,70582385	0,63417052	0,85748567	T1D vs T2D

hsa-miR-652-3p	-0,051595417	8,861681645	0,636105705	0,85748567	T1D vs T2D
hsa-miR-30e-5p	-0,029273083	12,20081412	0,636989355	0,85748567	T1D vs T2D
hsa-miR-144-5p	-0,092346167	7,794091027	0,649849894	0,858292313	T1D vs T2D
hsa-miR-99b-5p	-0,038933628	10,12326758	0,728803601	0,918938438	T1D vs T2D
hsa-miR-486-3p	-0,063586507	8,565942792	0,728863706	0,918938438	T1D vs T2D
hsa-miR-150-5p	0,055183299	11,98788735	0,742261038	0,918938438	T1D vs T2D
hsa-let-7i-5p	-0,030426123	12,52330537	0,75716846	0,918938438	T1D vs T2D
hsa-miR-342-3p	0,046683761	10,72352034	0,761406134	0,918938438	T1D vs T2D
hsa-miR-99a-5p	-0,037349637	9,621789786	0,774554904	0,918963446	T1D vs T2D
hsa-miR-103a-3p	0,030344721	9,566845601	0,819947177	0,935927092	T1D vs T2D
hsa-miR-10a-5p	0,021935111	11,37122715	0,826287005	0,935927092	T1D vs T2D
hsa-miR-423-3p	0,020968017	12,14274205	0,828963996	0,935927092	T1D vs T2D
hsa-miR-191-5p	-0,01431596	12,36621321	0,842963421	0,936626024	T1D vs T2D
hsa-let-7a-5p	-0,01248406	14,36018109	0,874599548	0,946701705	T1D vs T2D
hsa-miR-128-3p	-0,012188545	9,606700756	0,885281079	0,946701705	T1D vs T2D
hsa-miR-26a-5p	-0,019048217	12,12416215	0,892604465	0,946701705	T1D vs T2D
hsa-miR-335-5p	0,013020812	9,256688168	0,92006848	0,961265576	T1D vs T2D
hsa-let-7f-5p	-0,008068811	13,28674134	0,939789009	0,967429862	T1D vs T2D
hsa-miR-29a-3p	0,00101566	10,23347773	0,993596896	0,999254312	T1D vs T2D
hsa-miR-193a-5p	0,000138415	9,154888768	0,999254312	0,999254312	T1D vs T2D
hsa-miR-10b-5p	0,263178876	11,92933233	0,011489528	0,276986435	LADA vs T1D
hsa-miR-125a-5p	0,259060232	12,04595729	0,012116202	0,276986435	LADA vs T1D
hsa-let-7e-5p	0,420793004	8,239214195	0,015595578	0,276986435	LADA vs T1D
hsa-miR-30a-5p	0,241787887	10,38201877	0,015827796	0,276986435	LADA vs T1D
hsa-miR-99b-5p	0,239870702	10,12326758	0,033479519	0,468713271	LADA vs T1D
hsa-miR-486-3p	-0,355259955	8,565942792	0,054003253	0,551452665	LADA vs T1D
hsa-miR-15b-5p	-0,193331197	9,98101658	0,057881941	0,551452665	LADA vs T1D
hsa-miR-10a-5p	0,186387616	11,37122715	0,063023162	0,551452665	LADA vs T1D
hsa-miR-126-3p	0,185755618	11,77700488	0,1076769	0,837487001	LADA vs T1D
hsa-miR-375-3p	-0,321254499	10,48979782	0,132406337	0,855127283	LADA vs T1D
hsa-miR-140-3p	-0,199428622	9,550538435	0,134377144	0,855127283	LADA vs T1D
hsa-miR-27a-3p	0,157134676	10,10512206	0,148511904	0,866319442	LADA vs T1D
hsa-miR-652-3p	-0,15154994	8,861681645	0,165333889	0,890259403	LADA vs T1D
hsa-miR-423-3p	-0,126130866	12,14274205	0,194389482	0,918961223	LADA vs T1D
hsa-miR-30e-5p	-0,075578983	12,20081412	0,223551652	0,918961223	LADA vs T1D
hsa-miR-146a-5p	0,11394395	12,47099261	0,250127173	0,918961223	LADA vs T1D
hsa-miR-93-5p	-0,117036125	10,25947632	0,276724689	0,918961223	LADA vs T1D
hsa-miR-320a-3p	-0,11097954	12,07431198	0,277943797	0,918961223	LADA vs T1D
hsa-miR-24-3p	-0,084966597	13,21412419	0,278970799	0,918961223	LADA vs T1D
hsa-let-7b-5p	-0,073795142	14,2837785	0,287960995	0,918961223	LADA vs T1D
hsa-miR-25-3p	-0,144611698	12,72047871	0,315273216	0,918961223	LADA vs T1D
hsa-miR-30d-5p	-0,067054884	14,45578756	0,317262454	0,918961223	LADA vs T1D
hsa-miR-122-5p	-0,218633959	13,99900332	0,348791886	0,918961223	LADA vs T1D
hsa-miR-143-3p	-0,121731803	10,70582385	0,351115056	0,918961223	LADA vs T1D
hsa-miR-423-5p	-0,063849826	15,38689191	0,372020248	0,918961223	LADA vs T1D
hsa-miR-23a-3p	-0,068784853	12,54260289	0,394301436	0,918961223	LADA vs T1D
hsa-miR-16-5p	-0,082871209	14,33881148	0,442160265	0,918961223	LADA vs T1D
hsa-miR-92a-3p	-0,075797712	17,46006468	0,46096382	0,918961223	LADA vs T1D
hsa-miR-26b-5p	0,108429746	10,23866557	0,464184012	0,918961223	LADA vs T1D
hsa-miR-223-3p	-0,112734266	11,99339426	0,476634305	0,918961223	LADA vs T1D
hsa-miR-27b-3p	0,079514158	10,06790199	0,485316409	0,918961223	LADA vs T1D
hsa-miR-425-5p	-0,055638111	11,65414966	0,488289498	0,918961223	LADA vs T1D
hsa-miR-142-5p	0,070697864	11,41969176	0,488836133	0,918961223	LADA vs T1D
hsa-let-7i-5p	-0,06689282	12,52330537	0,496345064	0,918961223	LADA vs T1D
hsa-miR-451a1	0,097694756	16,82769003	0,497968242	0,918961223	LADA vs T1D
hsa-miR-335-5p	0,076558589	9,256688168	0,55488991	0,918961223	LADA vs T1D
hsa-miR-98-5p	-0,100197298	8,792738384	0,560174093	0,918961223	LADA vs T1D
hsa-miR-197-3p	-0,075585449	10,71414886	0,568794819	0,918961223	LADA vs T1D
hsa-miR-150-5p	0,09208053	11,98788735	0,583244798	0,918961223	LADA vs T1D
hsa-miR-26a-5p	0,07653979	12,12416215	0,587344724	0,918961223	LADA vs T1D
hsa-let-7a-5p	0,042803249	14,36018109	0,588049375	0,918961223	LADA vs T1D
hsa-miR-125b-5p	-0,064571789	10,46377314	0,604069677	0,918961223	LADA vs T1D
hsa-miR-181a-5p	-0,042936988	10,83967257	0,61289643	0,918961223	LADA vs T1D
hsa-miR-22-3p	-0,039453625	12,67951126	0,620153989	0,918961223	LADA vs T1D
hsa-miR-21-5p	0,038822047	13,68926132	0,630278392	0,918961223	LADA vs T1D
hsa-miR-191-5p	0,03467221	12,36621321	0,631241465	0,918961223	LADA vs T1D

hsa-miR-4841	-0,069069149	11,45694783	0,637375206	0,918961223	LADA vs T1D
hsa-miR-361-5p	0,059839617	7,743245637	0,639525471	0,918961223	LADA vs T1D
hsa-let-7d-3p	0,033217388	12,74297653	0,658637407	0,918961223	LADA vs T1D
hsa-miR-486-5p	-0,060249421	18,27283556	0,673891334	0,918961223	LADA vs T1D
hsa-miR-222-3p	-0,050873864	9,659340777	0,694618383	0,918961223	LADA vs T1D
hsa-miR-101-3p	-0,043878528	9,504552453	0,705643506	0,918961223	LADA vs T1D
hsa-miR-128-3p	-0,030745476	9,606700756	0,715707198	0,918961223	LADA vs T1D
hsa-miR-146b-5p	-0,038290441	9,113840715	0,737948892	0,918961223	LADA vs T1D
hsa-miR-144-5p	-0,063792563	7,794091027	0,753694658	0,918961223	LADA vs T1D
hsa-let-7c-5p	0,045492094	8,230590245	0,765170831	0,918961223	LADA vs T1D
hsa-let-7f-5p	-0,030476695	13,28674134	0,774946001	0,918961223	LADA vs T1D
hsa-miR-186-5p	-0,028621819	9,753658884	0,775150495	0,918961223	LADA vs T1D
hsa-miR-151a-3p	0,044687208	9,101827398	0,780754551	0,918961223	LADA vs T1D
hsa-miR-99a-5p	-0,029917011	9,621789786	0,818407289	0,918961223	LADA vs T1D
hsa-miR-342-3p	-0,034444657	10,72352034	0,822741025	0,918961223	LADA vs T1D
hsa-miR-30c-5p	0,023118109	10,77418095	0,830485333	0,918961223	LADA vs T1D
hsa-miR-148a-3p	-0,023113297	9,865728499	0,830917116	0,918961223	LADA vs T1D
hsa-miR-29a-3p	-0,025521736	10,23347773	0,840193119	0,918961223	LADA vs T1D
hsa-let-7g-5p	0,012778689	12,37806536	0,867657947	0,934400866	LADA vs T1D
hsa-miR-103a-3p	0,017813605	9,566845601	0,893622539	0,947781481	LADA vs T1D
hsa-miR-363-3p	0,016163236	9,933281027	0,909499581	0,950223443	LADA vs T1D
hsa-miR-193a-5p	-0,006734198	9,154888768	0,963683635	0,981012849	LADA vs T1D
hsa-miR-182-5p	-0,006369653	9,621258824	0,96699838	0,981012849	LADA vs T1D
hsa-miR-4433b-5p	0,005914592	10,44673565	0,983370642	0,983370642	LADA vs T1D
hsa-let-7d-3p	0,267357963	12,74297653	0,000426702	0,019599751	LADA vs T2D
hsa-miR-140-3p	-0,462199694	9,550538435	0,000559993	0,019599751	LADA vs T2D
hsa-miR-10b-5p	0,33755852	11,92933233	0,001191229	0,027795335	LADA vs T2D
hsa-miR-93-5p	-0,339888399	10,25947632	0,001664117	0,02912204	LADA vs T2D
hsa-miR-186-5p	-0,302230459	9,753658884	0,002686906	0,037616689	LADA vs T2D
hsa-miR-197-3p	0,360176769	10,71414886	0,006778226	0,079079305	LADA vs T2D
hsa-miR-425-5p	-0,213266615	11,65414966	0,007995617	0,079956171	LADA vs T2D
hsa-miR-30d-5p	-0,172572068	14,45578756	0,010173841	0,089021104	LADA vs T2D
hsa-miR-146b-5p	-0,272770415	9,113840715	0,017291521	0,130679807	LADA vs T2D
hsa-miR-151a-3p	-0,377956109	9,101827398	0,018668544	0,130679807	LADA vs T2D
hsa-miR-486-3p	-0,418846461	8,565942792	0,02246401	0,142952788	LADA vs T2D
hsa-miR-27a-3p	0,231138655	10,10512206	0,033000629	0,184722534	LADA vs T2D
hsa-miR-16-5p	-0,225919899	14,33881148	0,036107459	0,184722534	LADA vs T2D
hsa-miR-10a-5p	0,208322727	11,37122715	0,036944507	0,184722534	LADA vs T2D
hsa-miR-223-3p	-0,307579486	11,99339426	0,051607558	0,228404223	LADA vs T2D
hsa-miR-25-3p	-0,278584361	12,72047871	0,05220668	0,228404223	LADA vs T2D
hsa-miR-652-3p	-0,203145357	8,861681645	0,061759594	0,245399079	LADA vs T2D
hsa-miR-4842	-0,271642553	11,45694783	0,06310262	0,245399079	LADA vs T2D
hsa-miR-99b-5p	0,200937074	10,12326758	0,072712757	0,267889105	LADA vs T2D
hsa-miR-423-5p	0,124108529	15,38689191	0,082632027	0,289212096	LADA vs T2D
hsa-miR-30e-5p	-0,104852066	12,20081412	0,089882425	0,299608084	LADA vs T2D
hsa-miR-363-3p	-0,235937803	9,933281027	0,096104403	0,300514066	LADA vs T2D
hsa-miR-122-5p	-0,383672695	13,99900332	0,098740336	0,300514066	LADA vs T2D
hsa-miR-125a-5p	0,166480464	12,04595729	0,103398119	0,301577848	LADA vs T2D
hsa-miR-181a-5p	-0,123280231	10,83967257	0,144939491	0,405830576	LADA vs T2D
hsa-miR-143-3p	-0,183816899	10,70582385	0,15724579	0,419063698	LADA vs T2D
hsa-miR-361-5p	-0,176443621	7,743245637	0,166933421	0,419063698	LADA vs T2D
hsa-miR-30c-5p	-0,14847302	10,77418095	0,167625479	0,419063698	LADA vs T2D
hsa-miR-148a-3p	-0,131742592	9,865728499	0,221499415	0,534653761	LADA vs T2D
hsa-miR-21-5p	0,094167988	13,68926132	0,240830046	0,540398873	LADA vs T2D
hsa-miR-30a-5p	0,11525206	10,38201877	0,244701269	0,540398873	LADA vs T2D
hsa-miR-125b-5p	0,143560303	10,46377314	0,247039485	0,540398873	LADA vs T2D
hsa-miR-222-3p	-0,143025246	9,659340777	0,267488628	0,555207114	LADA vs T2D
hsa-miR-423-3p	-0,10516285	12,14274205	0,276058694	0,555207114	LADA vs T2D
hsa-miR-22-3p	-0,085413604	12,67951126	0,280822479	0,555207114	LADA vs T2D
hsa-miR-101-3p	-0,12349168	9,504552453	0,285535087	0,555207114	LADA vs T2D
hsa-miR-15b-5p	-0,102128321	9,98101658	0,311815155	0,575617431	LADA vs T2D
hsa-let-7i-5p	-0,097318943	12,52330537	0,31960772	0,575617431	LADA vs T2D
hsa-miR-182-5p	-0,152166933	9,621258824	0,32070114	0,575617431	LADA vs T2D
hsa-miR-4433b-5p	0,272041636	10,44673565	0,335418327	0,586982072	LADA vs T2D
hsa-miR-150-5p	0,14726383	11,98788735	0,377840038	0,645092748	LADA vs T2D
hsa-miR-451a2	-0,123352447	16,82769003	0,3922239	0,6537065	LADA vs T2D

hsa-miR-144-5p	-0,15613873	7,794091027	0,441157594	0,718163525	LADA vs T2D
hsa-miR-335-5p	0,089579401	9,256688168	0,487388987	0,77539157	LADA vs T2D
hsa-miR-92a-3p	0,069292869	17,46006468	0,4999613	0,777717578	LADA vs T2D
hsa-miR-27b-3p	-0,072411593	10,06790199	0,522518627	0,786974031	LADA vs T2D
hsa-let-7g-5p	-0,048064663	12,37806536	0,52839685	0,786974031	LADA vs T2D
hsa-miR-142-5p	-0,057372052	11,41969176	0,571796002	0,819749186	LADA vs T2D
hsa-miR-486-5p	0,075459903	18,27283556	0,597839432	0,819749186	LADA vs T2D
hsa-miR-126-3p	0,059609606	11,77700488	0,602502195	0,819749186	LADA vs T2D
hsa-miR-99a-5p	-0,067266648	9,621789786	0,603817412	0,819749186	LADA vs T2D
hsa-miR-128-3p	-0,042934022	9,606700756	0,608956538	0,819749186	LADA vs T2D
hsa-miR-320a-3p	0,047084653	12,07431198	0,643046299	0,849306433	LADA vs T2D
hsa-miR-24-3p	-0,032494148	13,21412419	0,676565574	0,85586102	LADA vs T2D
hsa-miR-26a-5p	0,057491573	12,12416215	0,681718936	0,85586102	LADA vs T2D
hsa-let-7a-5p	0,030319189	14,36018109	0,699675113	0,85586102	LADA vs T2D
hsa-let-7f-5p	-0,038545506	13,28674134	0,715962497	0,85586102	LADA vs T2D
hsa-miR-103a-3p	0,048158326	9,566845601	0,716111016	0,85586102	LADA vs T2D
hsa-let-7e-5p	0,0595388	8,239214195	0,729175286	0,85586102	LADA vs T2D
hsa-let-7c-5p	-0,051630264	8,230590245	0,73359516	0,85586102	LADA vs T2D
hsa-miR-146a-5p	-0,029488404	12,47099261	0,76430027	0,876960919	LADA vs T2D
hsa-miR-191-5p	0,02035625	12,36621321	0,776736814	0,876960919	LADA vs T2D
hsa-miR-29a-3p	-0,024506076	10,23347773	0,845556621	0,928855881	LADA vs T2D
hsa-let-7b-5p	-0,013116814	14,2837785	0,849239663	0,928855881	LADA vs T2D
hsa-miR-26b-5p	0,022662275	10,23866557	0,877604191	0,945112206	LADA vs T2D
hsa-miR-375-3p	0,027497548	10,48979782	0,896688638	0,951033404	LADA vs T2D
hsa-miR-342-3p	0,012239104	10,72352034	0,936189902	0,964262431	LADA vs T2D
hsa-miR-98-5p	0,012931933	8,792738384	0,939675775	0,964262431	LADA vs T2D
hsa-miR-23a-3p	0,004055564	12,54260289	0,959659086	0,964262431	LADA vs T2D
hsa-miR-193a-5p	-0,006595783	9,154888768	0,964262431	0,964262431	LADA vs T2D
hsa-miR-30d-5p	-0,24031914	14,45578756	0,000913504	0,063945247	LADA vs Control
hsa-miR-22-3p	0,246780476	12,67951126	0,003801498	0,077506745	LADA vs Control
hsa-miR-425-5p	-0,246829582	11,65414966	0,004193027	0,077506745	LADA vs Control
hsa-miR-30a-5p	0,303094227	10,38201877	0,004428957	0,077506745	LADA vs Control
hsa-miR-151a-3p	-0,459795736	9,101827398	0,007342243	0,102791403	LADA vs Control
hsa-miR-30e-5p	-0,165608423	12,20081412	0,012594818	0,144709539	LADA vs Control
hsa-miR-186-5p	-0,261930665	9,753658884	0,014470954	0,144709539	LADA vs Control
hsa-miR-223-3p	-0,394882948	11,99339426	0,019803576	0,173281291	LADA vs Control
hsa-miR-93-5p	-0,244569987	10,25947632	0,033175305	0,250652441	LADA vs Control
hsa-miR-101-3p	-0,259779166	9,504552453	0,035807492	0,250652441	LADA vs Control
hsa-miR-423-5p	0,156317897	15,38689191	0,04258351	0,270985973	LADA vs Control
hsa-miR-126-3p	0,231290516	11,77700488	0,059409533	0,336927628	LADA vs Control
hsa-miR-140-3p	-0,263446323	9,550538435	0,062572274	0,336927628	LADA vs Control
hsa-miR-375-3p	0,383566376	10,48979782	0,091104318	0,396331081	LADA vs Control
hsa-miR-4433b-5p	-0,502097447	10,44673565	0,098306313	0,396331081	LADA vs Control
hsa-miR-150-5p	0,29005433	11,98788735	0,103613345	0,396331081	LADA vs Control
hsa-miR-27b-3p	0,196780905	10,06790199	0,104309226	0,396331081	LADA vs Control
hsa-miR-320a-3p	0,175500823	12,07431198	0,106336249	0,396331081	LADA vs Control
hsa-miR-10a-5p	0,170961654	11,37122715	0,107575579	0,396331081	LADA vs Control
hsa-miR-29a-3p	0,21222231	10,23347773	0,114909286	0,402182502	LADA vs Control
hsa-miR-10b-5p	0,156340169	11,92933233	0,153664333	0,481084872	LADA vs Control
hsa-miR-197-3p	-0,201552613	10,71414886	0,155272283	0,481084872	LADA vs Control
hsa-miR-148a-3p	-0,162963999	9,865728499	0,158070744	0,481084872	LADA vs Control
hsa-let-7f-5p	-0,149618407	13,28674134	0,186156174	0,524727042	LADA vs Control
hsa-miR-191-5p	-0,101241378	12,36621321	0,187402515	0,524727042	LADA vs Control
hsa-miR-128-3p	-0,11199718	9,606700756	0,211426727	0,527705361	LADA vs Control
hsa-miR-361-5p	0,167617505	7,743245637	0,220877063	0,527705361	LADA vs Control
hsa-miR-146b-5p	-0,147796559	9,113840715	0,222672791	0,527705361	LADA vs Control
hsa-miR-125a-5p	0,132423728	12,04595729	0,223387177	0,527705361	LADA vs Control
hsa-miR-26b-5p	-0,19059962	10,23866557	0,22615944	0,527705361	LADA vs Control
hsa-miR-652-3p	0,135088983	8,861681645	0,245289739	0,55069731	LADA vs Control
hsa-miR-143-3p	-0,159664641	10,70582385	0,251747342	0,55069731	LADA vs Control
hsa-miR-222-3p	-0,14974427	9,659340777	0,275765607	0,584957348	LADA vs Control
hsa-miR-486-3p	-0,196356408	8,565942792	0,316884353	0,637625579	LADA vs Control
hsa-let-7e-5p	0,18095602	8,239214195	0,326501834	0,637625579	LADA vs Control
hsa-miR-99a-5p	0,130532656	9,621789786	0,342604586	0,637625579	LADA vs Control
hsa-let-7c-5p	0,150838089	8,230590245	0,350656414	0,637625579	LADA vs Control
hsa-miR-24-3p	0,077216678	13,21412419	0,353793246	0,637625579	LADA vs Control

hsa-miR-27a-3p	0,105067555	10,10512206	0,362017062	0,637625579	LADA vs Control
hsa-miR-423-3p	-0,093543345	12,14274205	0,364357474	0,637625579	LADA vs Control
hsa-miR-363-3p	0,133651894	9,933281027	0,376113306	0,642144669	LADA vs Control
hsa-miR-335-5p	-0,116643341	9,256688168	0,396554162	0,65207269	LADA vs Control
hsa-miR-25-3p	-0,128452993	12,72047871	0,400558938	0,65207269	LADA vs Control
hsa-miR-92a-3p	-0,086803773	17,46006468	0,430637429	0,685105001	LADA vs Control
hsa-miR-342-3p	0,121302767	10,72352034	0,45770453	0,711984824	LADA vs Control
hsa-miR-122-5p	0,171949006	13,99900332	0,486887758	0,740916154	LADA vs Control
hsa-miR-486-5p	-0,102158522	18,27283556	0,504949536	0,752052501	LADA vs Control
hsa-miR-23a-3p	-0,0523932	12,54260289	0,540754626	0,788600496	LADA vs Control
hsa-miR-181a-5p	0,053339231	10,83967257	0,554606688	0,792295269	LADA vs Control
hsa-miR-26a-5p	-0,08583084	12,12416215	0,566586858	0,793221601	LADA vs Control
hsa-miR-15b-5p	-0,059308722	9,98101658	0,582487216	0,799492258	LADA vs Control
hsa-miR-182-5p	-0,079788271	9,621258824	0,624755178	0,822322133	LADA vs Control
hsa-miR-21-5p	-0,041177095	13,68926132	0,629799569	0,822322133	LADA vs Control
hsa-miR-16-5p	-0,05292549	14,33881148	0,644106962	0,822322133	LADA vs Control
hsa-miR-98-5p	-0,081754256	8,792738384	0,6547243	0,822322133	LADA vs Control
hsa-miR-30c-5p	-0,050099771	10,77418095	0,663045513	0,822322133	LADA vs Control
hsa-miR-4843	-0,065047893	11,45694783	0,676161307	0,822322133	LADA vs Control
hsa-let-7a-5p	-0,034597688	14,36018109	0,681352625	0,822322133	LADA vs Control
hsa-miR-146a-5p	0,039862723	12,47099261	0,70380936	0,835028054	LADA vs Control
hsa-miR-103a-3p	-0,050630945	9,566845601	0,720098572	0,840091791	LADA vs Control
hsa-let-7d-3p	-0,026474831	12,74297653	0,739830554	0,840091791	LADA vs Control
hsa-miR-142-5p	-0,0336089	11,41969176	0,757192088	0,840091791	LADA vs Control
hsa-miR-99b-5p	0,035326816	10,12326758	0,766181885	0,840091791	LADA vs Control
hsa-let-7g-5p	-0,024012164	12,37806536	0,768083924	0,840091791	LADA vs Control
hsa-miR-144-5p	-0,05105583	7,794091027	0,814148322	0,876775116	LADA vs Control
hsa-miR-125b-5p	-0,017937168	10,46377314	0,891971584	0,943397193	LADA vs Control
hsa-miR-451a3	0,018836988	16,82769003	0,902965885	0,943397193	LADA vs Control
hsa-miR-193a-5p	-0,012281564	9,154888768	0,937144844	0,964707928	LADA vs Control
hsa-let-7b-5p	-0,003454396	14,2837785	0,962732402	0,976685046	LADA vs Control
hsa-let-7i-5p	0,000186137	12,52330537	0,998577447	0,998577447	LADA vs Control
hsa-miR-22-3p	0,286234102	12,67951126	0,000348038	0,024362655	T1D vs Control
hsa-miR-375-3p	0,704820875	10,48979782	0,000984173	0,034446064	T1D vs Control
hsa-miR-151a-3p	-0,504482944	9,101827398	0,001697087	0,039598693	T1D vs Control
hsa-miR-423-5p	0,220167722	15,38689191	0,002296171	0,040182992	T1D vs Control
hsa-miR-320a-3p	0,286480363	12,07431198	0,005024358	0,070341013	T1D vs Control
hsa-miR-652-3p	0,286638923	8,861681645	0,008504249	0,098529362	T1D vs Control
hsa-miR-30d-5p	-0,173264256	14,45578756	0,009852936	0,098529362	T1D vs Control
hsa-miR-425-5p	-0,191191471	11,65414966	0,016834028	0,147297747	T1D vs Control
hsa-miR-186-5p	-0,233308846	9,753658884	0,019467535	0,151414159	T1D vs Control
hsa-miR-24-3p	0,162183275	13,21412419	0,037776033	0,264432234	T1D vs Control
hsa-miR-26b-5p	-0,299029366	10,23866557	0,042365685	0,269599813	T1D vs Control
hsa-miR-191-5p	-0,135913588	12,36621321	0,058314576	0,300387984	T1D vs Control
hsa-miR-29a-3p	0,237744045	10,23347773	0,058578948	0,300387984	T1D vs Control
hsa-miR-101-3p	-0,215900638	9,504552453	0,061467799	0,300387984	T1D vs Control
hsa-miR-99b-5p	-0,204543886	10,12326758	0,066329146	0,300387984	T1D vs Control
hsa-miR-4433b-5p	-0,508012039	10,44673565	0,072324749	0,300387984	T1D vs Control
hsa-miR-223-3p	-0,282148682	11,99339426	0,072951367	0,300387984	T1D vs Control
hsa-miR-122-5p	0,390582965	13,99900332	0,09130263	0,355065785	T1D vs Control
hsa-miR-335-5p	-0,193201931	9,256688168	0,133384611	0,491416988	T1D vs Control
hsa-miR-30e-5p	-0,09002944	12,20081412	0,143488353	0,502209234	T1D vs Control
hsa-let-7e-5p	-0,239836984	8,239214195	0,163102798	0,543675994	T1D vs Control
hsa-miR-15b-5p	0,134022474	9,98101658	0,183183407	0,582856295	T1D vs Control
hsa-miR-148a-3p	-0,139850701	9,865728499	0,19307367	0,587615516	T1D vs Control
hsa-miR-125a-5p	-0,126636505	12,04595729	0,212442276	0,592807322	T1D vs Control
hsa-miR-99a-5p	0,160449667	9,621789786	0,213103746	0,592807322	T1D vs Control
hsa-miR-93-5p	-0,127533862	10,25947632	0,231379331	0,592807322	T1D vs Control
hsa-miR-150-5p	0,197973799	11,98788735	0,233374684	0,592807322	T1D vs Control
hsa-miR-26a-5p	-0,16237063	12,12416215	0,245579762	0,592807322	T1D vs Control
hsa-miR-181a-5p	0,096276219	10,83967257	0,252880884	0,592807322	T1D vs Control
hsa-let-7f-5p	-0,119141712	13,28674134	0,25959833	0,592807322	T1D vs Control
hsa-miR-10b-5p	-0,106838707	11,92933233	0,296661988	0,592807322	T1D vs Control
hsa-miR-27b-3p	0,117266747	10,06790199	0,29838631	0,592807322	T1D vs Control
hsa-miR-142-5p	-0,104306763	11,41969176	0,302885811	0,592807322	T1D vs Control
hsa-miR-342-3p	0,155747424	10,72352034	0,306638047	0,592807322	T1D vs Control

hsa-let-7b-5p	0,070340746	14,2837785	0,307914031	0,592807322	T1D vs Control
hsa-miR-21-5p	-0,079999142	13,68926132	0,316544604	0,592807322	T1D vs Control
hsa-let-7a-5p	-0,077400937	14,36018109	0,324615639	0,592807322	T1D vs Control
hsa-miR-128-3p	-0,081251703	9,606700756	0,331080788	0,592807322	T1D vs Control
hsa-miR-146b-5p	-0,109506119	9,113840715	0,333695657	0,592807322	T1D vs Control
hsa-miR-197-3p	-0,125967164	10,71414886	0,338747041	0,592807322	T1D vs Control
hsa-miR-486-3p	0,158903547	8,565942792	0,382880536	0,653698476	T1D vs Control
hsa-miR-361-5p	0,107777887	7,743245637	0,397616773	0,657746056	T1D vs Control
hsa-miR-363-3p	0,117488658	9,933281027	0,404044006	0,657746056	T1D vs Control
hsa-let-7d-3p	-0,05969222	12,74297653	0,422713964	0,672499489	T1D vs Control
hsa-miR-222-3p	-0,098870406	9,659340777	0,440570573	0,683963278	T1D vs Control
hsa-miR-146a-5p	-0,074081227	12,47099261	0,449461583	0,683963278	T1D vs Control
hsa-let-7c-5p	0,105345995	8,230590245	0,486199075	0,705919085	T1D vs Control
hsa-let-7i-5p	0,067078958	12,52330537	0,491000149	0,705919085	T1D vs Control
hsa-miR-30c-5p	-0,07321788	10,77418095	0,49414336	0,705919085	T1D vs Control
hsa-miR-30a-5p	0,06130634	10,38201877	0,533424815	0,746794741	T1D vs Control
hsa-miR-451a4	-0,078857768	16,82769003	0,585062687	0,787405594	T1D vs Control
hsa-miR-103a-3p	-0,06844455	9,566845601	0,603694058	0,787405594	T1D vs Control
hsa-miR-140-3p	-0,064017701	9,550538435	0,626299245	0,787405594	T1D vs Control
hsa-miR-27a-3p	-0,05206712	10,10512206	0,627698784	0,787405594	T1D vs Control
hsa-let-7g-5p	-0,036790854	12,37806536	0,628048327	0,787405594	T1D vs Control
hsa-miR-182-5p	-0,073418618	9,621258824	0,629924475	0,787405594	T1D vs Control
hsa-miR-126-3p	0,045534899	11,77700488	0,689579032	0,846851443	T1D vs Control
hsa-miR-125b-5p	0,046634621	10,46377314	0,705176405	0,851074971	T1D vs Control
hsa-miR-423-3p	0,032587522	12,14274205	0,734407404	0,871330818	T1D vs Control
hsa-miR-143-3p	-0,037932837	10,70582385	0,769371651	0,880146727	T1D vs Control
hsa-miR-486-5p	-0,041909101	18,27283556	0,769523552	0,880146727	T1D vs Control
hsa-miR-16-5p	0,029945719	14,33881148	0,77955853	0,880146727	T1D vs Control
hsa-miR-23a-3p	0,016391653	12,54260289	0,837380113	0,930422348	T1D vs Control
hsa-miR-10a-5p	-0,015425962	11,37122715	0,876048184	0,95570513	T1D vs Control
hsa-miR-25-3p	0,016158705	12,72047871	0,909632958	0,95570513	T1D vs Control
hsa-miR-98-5p	0,018443042	8,792738384	0,913748205	0,95570513	T1D vs Control
hsa-miR-92a-3p	-0,011006061	17,46006468	0,914746338	0,95570513	T1D vs Control
hsa-miR-144-5p	0,012736733	7,794091027	0,94979339	0,977728489	T1D vs Control
hsa-miR-193a-5p	-0,005547365	9,154888768	0,969706683	0,977871487	T1D vs Control
hsa-miR-4844	0,004021256	11,45694783	0,977871487	0,977871487	T1D vs Control
hsa-miR-197-3p	-0,561729381	10,71414886	6,16327E-05	0,002670561	T2D vs Control
hsa-miR-22-3p	0,33219408	12,67951126	7,63017E-05	0,002670561	T2D vs Control
hsa-let-7d-3p	-0,293832794	12,74297653	0,000201035	0,004690828	T2D vs Control
hsa-miR-652-3p	0,33823434	8,861681645	0,003061818	0,053581813	T2D vs Control
hsa-miR-4433b-5p	-0,774139083	10,44673565	0,009126872	0,117894902	T2D vs Control
hsa-miR-361-5p	0,344061126	7,743245637	0,010105277	0,117894902	T2D vs Control
hsa-miR-363-3p	0,369589697	9,933281027	0,012600926	0,126009261	T2D vs Control
hsa-miR-122-5p	0,5556217	13,99900332	0,021894867	0,178535798	T2D vs Control
hsa-miR-27b-3p	0,269192498	10,06790199	0,022954603	0,178535798	T2D vs Control
hsa-miR-181a-5p	0,176619461	10,83967257	0,045375089	0,317625626	T2D vs Control
hsa-miR-30a-5p	0,187842167	10,38201877	0,06853631	0,414791942	T2D vs Control
hsa-miR-29a-3p	0,236728385	10,23347773	0,07110719	0,414791942	T2D vs Control
hsa-miR-10b-5p	-0,181218351	11,92933233	0,090245195	0,468087102	T2D vs Control
hsa-miR-191-5p	-0,121597628	12,36621321	0,10456161	0,468087102	T2D vs Control
hsa-miR-21-5p	-0,135345082	13,68926132	0,10553167	0,468087102	T2D vs Control
hsa-miR-375-3p	0,356068828	10,48979782	0,107427156	0,468087102	T2D vs Control
hsa-miR-16-5p	0,172994409	14,33881148	0,12293626	0,468087102	T2D vs Control
hsa-miR-335-5p	-0,206222742	9,256688168	0,124992602	0,468087102	T2D vs Control
hsa-miR-99a-5p	0,197799304	9,621789786	0,141088608	0,468087102	T2D vs Control
hsa-miR-92a-3p	-0,156096642	17,46006468	0,145588329	0,468087102	T2D vs Control
hsa-miR-140-3p	0,198753371	9,550538435	0,149032144	0,468087102	T2D vs Control
hsa-miR-126-3p	0,171680911	11,77700488	0,150561625	0,468087102	T2D vs Control
hsa-miR-99b-5p	-0,165610257	10,12326758	0,153800048	0,468087102	T2D vs Control
hsa-miR-26b-5p	-0,213261895	10,23866557	0,164775909	0,479704991	T2D vs Control
hsa-miR-4845	0,20659466	11,45694783	0,173749189	0,479704991	T2D vs Control
hsa-miR-24-3p	0,109710826	13,21412419	0,178176139	0,479704991	T2D vs Control
hsa-let-7c-5p	0,202468353	8,230590245	0,200382512	0,519510215	T2D vs Control
hsa-miR-125b-5p	-0,161497471	10,46377314	0,210078564	0,52519641	T2D vs Control
hsa-miR-320a-3p	0,12841617	12,07431198	0,224843688	0,542726143	T2D vs Control
hsa-miR-486-5p	-0,177618425	18,27283556	0,233340593	0,544461384	T2D vs Control

hsa-miR-486-3p	0,222490053	8,565942792	0,243113798	0,548966641	T2D vs Control
hsa-miR-101-3p	-0,136287486	9,504552453	0,257179073	0,554999411	T2D vs Control
hsa-miR-27a-3p	-0,1260711	10,10512206	0,261642579	0,554999411	T2D vs Control
hsa-miR-146b-5p	0,124973856	9,113840715	0,290606196	0,587970855	T2D vs Control
hsa-miR-25-3p	0,150131368	12,72047871	0,314479877	0,587970855	T2D vs Control
hsa-let-7f-5p	-0,1110729	13,28674134	0,314972131	0,587970855	T2D vs Control
hsa-miR-26a-5p	-0,143322414	12,12416215	0,326390378	0,587970855	T2D vs Control
hsa-miR-30d-5p	-0,067747072	14,45578756	0,331471115	0,587970855	T2D vs Control
hsa-let-7i-5p	0,097505081	12,52330537	0,338757568	0,587970855	T2D vs Control
hsa-miR-30e-5p	-0,060756357	12,20081412	0,344130619	0,587970855	T2D vs Control
hsa-miR-451a5	0,142189435	16,82769003	0,344382929	0,587970855	T2D vs Control
hsa-miR-30c-5p	0,098373249	10,77418095	0,379562129	0,632603548	T2D vs Control
hsa-miR-93-5p	0,095318412	10,25947632	0,391612655	0,637508973	T2D vs Control
hsa-miR-150-5p	0,1427905	11,98788735	0,409436918	0,651376915	T2D vs Control
hsa-miR-128-3p	-0,069063158	9,606700756	0,428813499	0,653278916	T2D vs Control
hsa-let-7a-5p	-0,064916877	14,36018109	0,429297573	0,653278916	T2D vs Control
hsa-miR-103a-3p	-0,098789271	9,566845601	0,473409152	0,685094506	T2D vs Control
hsa-miR-342-3p	0,109063663	10,72352034	0,4924645	0,685094506	T2D vs Control
hsa-miR-146a-5p	0,069351127	12,47099261	0,49782351	0,685094506	T2D vs Control
hsa-let-7e-5p	0,121417221	8,239214195	0,498006735	0,685094506	T2D vs Control
hsa-miR-23a-3p	-0,056448765	12,54260289	0,499140283	0,685094506	T2D vs Control
hsa-miR-223-3p	-0,087303462	11,99339426	0,594252285	0,785559787	T2D vs Control
hsa-miR-98-5p	-0,094686189	8,792738384	0,594780982	0,785559787	T2D vs Control
hsa-miR-144-5p	0,1050829	7,794091027	0,618397737	0,7912732	T2D vs Control
hsa-miR-151a-3p	-0,081839627	9,101827398	0,621714657	0,7912732	T2D vs Control
hsa-miR-182-5p	0,072378662	9,621258824	0,649303087	0,811628859	T2D vs Control
hsa-miR-423-5p	0,032209368	15,38689191	0,665630308	0,813781525	T2D vs Control
hsa-miR-15b-5p	0,042819599	9,98101658	0,683553824	0,813781525	T2D vs Control
hsa-miR-425-5p	-0,033562967	11,65414966	0,686102565	0,813781525	T2D vs Control
hsa-miR-186-5p	0,040299794	9,753658884	0,697527021	0,813781525	T2D vs Control
hsa-miR-10a-5p	-0,037361074	11,37122715	0,717462142	0,823317212	T2D vs Control
hsa-miR-125a-5p	-0,034056736	12,04595729	0,74756518	0,844025203	T2D vs Control
hsa-let-7g-5p	0,024052499	12,37806536	0,76191966	0,8465774	T2D vs Control
hsa-miR-148a-3p	-0,031221407	9,865728499	0,780670545	0,853858408	T2D vs Control
hsa-miR-142-5p	0,023763152	11,41969176	0,822146801	0,885388862	T2D vs Control
hsa-miR-143-3p	0,024152259	10,70582385	0,858279636	0,910296584	T2D vs Control
hsa-let-7b-5p	0,009662418	14,2837785	0,89328021	0,933277831	T2D vs Control
hsa-miR-423-3p	0,011619505	12,14274205	0,90783356	0,934534547	T2D vs Control
hsa-miR-222-3p	-0,006719024	9,659340777	0,959934797	0,97018057	T2D vs Control
hsa-miR-193a-5p	-0,005685781	9,154888768	0,97018057	0,97018057	T2D vs Control
hsa-miR-423-5p	0,251116142	15,43363167	0,003003161	0,150148404	T1D vs T2D Time-adjusted
hsa-miR-197-3p	0,460953435	10,63836899	0,004289954	0,150148404	T1D vs T2D Time-adjusted
hsa-let-7d-3p	0,217915299	12,72440102	0,017448508	0,328702337	T1D vs T2D Time-adjusted
hsa-miR-186-5p	-0,26945066	9,709133341	0,020516706	0,328702337	T1D vs T2D Time-adjusted
hsa-miR-140-3p	-0,316044112	9,535668537	0,027706998	0,328702337	T1D vs T2D Time-adjusted
hsa-miR-93-5p	-0,246996318	10,24046846	0,033390762	0,328702337	T1D vs T2D Time-adjusted
hsa-miR-151a-3p	-0,372842774	9,028748392	0,039562484	0,328702337	T1D vs T2D Time-adjusted
hsa-miR-320a-3p	0,238305328	12,14832719	0,040109081	0,328702337	T1D vs T2D Time-adjusted
hsa-miR-425-5p	-0,177232517	11,613117	0,042261729	0,328702337	T1D vs T2D Time-adjusted
hsa-miR-363-3p	-0,282394591	9,989550224	0,072618557	0,508329897	T1D vs T2D Time-adjusted
hsa-miR-15b-5p	0,199071618	9,988569024	0,084501819	0,528214758	T1D vs T2D Time-adjusted
hsa-miR-361-5p	-0,235918391	7,799497125	0,090551101	0,528214758	T1D vs T2D Time-adjusted
hsa-miR-146b-5p	-0,181642291	9,126180253	0,125402135	0,581222023	T1D vs T2D Time-adjusted
hsa-miR-451a	-0,248092574	16,83312299	0,126149079	0,581222023	T1D vs T2D Time-adjusted
hsa-miR-4433b-5p	0,524658242	10,31487352	0,128903982	0,581222023	T1D vs T2D Time-adjusted
hsa-miR-30a-5p	-0,166955063	10,44721948	0,132850748	0,581222023	T1D vs T2D Time-adjusted
hsa-miR-122-5p	-0,381609927	14,13006949	0,148567167	0,588305322	T1D vs T2D Time-adjusted
hsa-miR-24-3p	0,123860867	13,24628442	0,170407827	0,588305322	T1D vs T2D Time-adjusted
hsa-miR-223-3p	-0,248674729	11,91868399	0,173158644	0,588305322	T1D vs T2D Time-adjusted
hsa-let-7e-5p	-0,265300582	8,252607353	0,174904881	0,588305322	T1D vs T2D Time-adjusted
hsa-miR-30c-5p	-0,148183094	10,78220275	0,183147208	0,588305322	T1D vs T2D Time-adjusted
hsa-miR-103a-3p	0,199176096	9,552423125	0,184959194	0,588305322	T1D vs T2D Time-adjusted
hsa-miR-92a-3p	0,153555992	17,44154735	0,213476531	0,588305322	T1D vs T2D Time-adjusted
hsa-miR-27b-3p	-0,156766519	10,12130923	0,215916756	0,588305322	T1D vs T2D Time-adjusted
hsa-miR-181a-5p	-0,114264465	10,87889244	0,222738646	0,588305322	T1D vs T2D Time-adjusted
hsa-miR-16-5p	-0,148799218	14,33676009	0,2240095	0,588305322	T1D vs T2D Time-adjusted

hsa-miR-142-5p	-0,138345008	11,39821686	0,232828688	0,588305322	T1D vs T2D Time-adjusted
hsa-miR-21-5p	0,107133715	13,68104609	0,235322129	0,588305322	T1D vs T2D Time-adjusted
hsa-miR-25-3p	-0,194462529	12,71958248	0,250964941	0,592303268	T1D vs T2D Time-adjusted
hsa-miR-484	-0,191262938	11,47479359	0,253844258	0,592303268	T1D vs T2D Time-adjusted
hsa-miR-148a-3p	-0,135321271	9,813572581	0,278746589	0,626444098	T1D vs T2D Time-adjusted
hsa-let-7b-5p	0,082233809	14,30597172	0,286374445	0,626444098	T1D vs T2D Time-adjusted
hsa-miR-486-5p	0,161512361	18,24569138	0,330699247	0,678801045	T1D vs T2D Time-adjusted
hsa-miR-23a-3p	0,086707822	12,54700785	0,331494683	0,678801045	T1D vs T2D Time-adjusted
hsa-miR-143-3p	-0,136948364	10,68681172	0,339869704	0,678801045	T1D vs T2D Time-adjusted
hsa-miR-30d-5p	-0,070948828	14,41746295	0,354822058	0,678801045	T1D vs T2D Time-adjusted
hsa-miR-423-3p	0,103432742	12,15169654	0,358794838	0,678801045	T1D vs T2D Time-adjusted
hsa-miR-125b-5p	0,134441428	10,48393745	0,374092256	0,689117315	T1D vs T2D Time-adjusted
hsa-miR-144-5p	-0,180244358	7,813745406	0,388298313	0,69694569	T1D vs T2D Time-adjusted
hsa-miR-146a-5p	-0,089408833	12,49620569	0,407345542	0,698876242	T1D vs T2D Time-adjusted
hsa-miR-98-5p	0,153123051	8,777064989	0,413469774	0,698876242	T1D vs T2D Time-adjusted
hsa-miR-182-5p	-0,135296138	9,626298557	0,419325745	0,698876242	T1D vs T2D Time-adjusted
hsa-miR-27a-3p	0,096501043	10,10682419	0,440824774	0,717621725	T1D vs T2D Time-adjusted
hsa-miR-99a-5p	-0,109597336	9,686792179	0,47688661	0,758683244	T1D vs T2D Time-adjusted
hsa-miR-125a-5p	-0,075159478	12,06715528	0,545036849	0,847835099	T1D vs T2D Time-adjusted
hsa-miR-375-3p	0,145408928	10,64120473	0,557685017	0,848651112	T1D vs T2D Time-adjusted
hsa-let-7f-5p	0,06667581	13,26251625	0,574606526	0,849848142	T1D vs T2D Time-adjusted
hsa-miR-335-5p	0,082056438	9,217895277	0,587879099	0,849848142	T1D vs T2D Time-adjusted
hsa-let-7g-5p	-0,041791643	12,38158425	0,605521316	0,849848142	T1D vs T2D Time-adjusted
hsa-miR-26a-5p	0,079724408	12,10206399	0,607034387	0,849848142	T1D vs T2D Time-adjusted
hsa-miR-652-3p	0,056110372	8,921155655	0,641057069	0,865211516	T1D vs T2D Time-adjusted
hsa-miR-126-3p	-0,058194079	11,83461714	0,642728555	0,865211516	T1D vs T2D Time-adjusted
hsa-let-7c-5p	-0,070927627	8,318110139	0,666093503	0,87352652	T1D vs T2D Time-adjusted
hsa-miR-22-3p	-0,040209373	12,7696365	0,673863315	0,87352652	T1D vs T2D Time-adjusted
hsa-miR-29a-3p	-0,05858164	10,29575836	0,68662499	0,873886351	T1D vs T2D Time-adjusted
hsa-let-7a-5p	0,032267374	14,35554361	0,710221899	0,887777374	T1D vs T2D Time-adjusted
hsa-miR-10a-5p	-0,036384649	11,41317012	0,757146285	0,929828771	T1D vs T2D Time-adjusted
hsa-miR-30e-5p	0,017605057	12,17327791	0,786364397	0,94906048	T1D vs T2D Time-adjusted
hsa-miR-486-3p	-0,045614657	8,576611099	0,829126424	0,95135937	T1D vs T2D Time-adjusted
hsa-miR-222-3p	-0,03114122	9,646980979	0,831159218	0,95135937	T1D vs T2D Time-adjusted
hsa-miR-10b-5p	0,02540842	11,95396841	0,832353172	0,95135937	T1D vs T2D Time-adjusted
hsa-let-7i-5p	0,020938534	12,5425511	0,842632585	0,95135937	T1D vs T2D Time-adjusted
hsa-miR-99b-5p	0,021226689	10,13632976	0,869355806	0,958147187	T1D vs T2D Time-adjusted
hsa-miR-342-3p	0,027711813	10,76380446	0,876020285	0,958147187	T1D vs T2D Time-adjusted
hsa-miR-128-3p	0,009027545	9,594398688	0,924273846	0,98290668	T1D vs T2D Time-adjusted
hsa-miR-26b-5p	0,013191321	10,19799103	0,936918886	0,98290668	T1D vs T2D Time-adjusted
hsa-miR-150-5p	0,011968493	12,04909227	0,950487028	0,98290668	T1D vs T2D Time-adjusted
hsa-miR-101-3p	-0,005804227	9,461671448	0,964460711	0,98290668	T1D vs T2D Time-adjusted
hsa-miR-193a-5p	0,004329638	9,188089353	0,980244496	0,98290668	T1D vs T2D Time-adjusted
hsa-miR-191-5p	0,001666258	12,34906605	0,98290668	0,98290668	T1D vs T2D Time-adjusted
hsa-miR-10b-5p	0,321120984	11,95396841	0,005501393	0,29715342	LADA vs T1D Time-adjusted
hsa-miR-30a-5p	0,280498101	10,44721948	0,008490098	0,29715342	LADA vs T1D Time-adjusted
hsa-miR-125a-5p	0,282210743	12,06715528	0,017915456	0,338254579	LADA vs T1D Time-adjusted
hsa-miR-15b-5p	-0,257755126	9,988569024	0,019328833	0,338254579	LADA vs T1D Time-adjusted
hsa-miR-10a-5p	0,252731577	11,41317012	0,025251299	0,353518186	LADA vs T1D Time-adjusted
hsa-let-7e-5p	0,380240879	8,252607353	0,041961325	0,489548787	LADA vs T1D Time-adjusted
hsa-miR-652-3p	-0,219159503	8,921155655	0,057056925	0,533370996	LADA vs T1D Time-adjusted
hsa-miR-99b-5p	0,227904392	10,13632976	0,064920801	0,533370996	LADA vs T1D Time-adjusted
hsa-miR-486-3p	-0,369356984	8,576611099	0,068576271	0,533370996	LADA vs T1D Time-adjusted
hsa-miR-30e-5p	-0,10642878	12,17327791	0,086359608	0,576226319	LADA vs T1D Time-adjusted
hsa-miR-320a-3p	-0,186307416	12,14832719	0,09054985	0,576226319	LADA vs T1D Time-adjusted
hsa-miR-423-3p	-0,164376454	12,15169654	0,126103779	0,686339791	LADA vs T1D Time-adjusted
hsa-miR-423-5p	-0,121593703	15,43363167	0,127463104	0,686339791	LADA vs T1D Time-adjusted
hsa-miR-24-3p	-0,120792153	13,24628442	0,158093897	0,743836133	LADA vs T1D Time-adjusted
hsa-miR-126-3p	0,168341225	11,83461714	0,159393457	0,743836133	LADA vs T1D Time-adjusted
hsa-miR-27a-3p	0,150392251	10,10682419	0,207713773	0,908747755	LADA vs T1D Time-adjusted
hsa-let-7i-5p	-0,114832634	12,5425511	0,252366363	0,995576483	LADA vs T1D Time-adjusted
hsa-let-7b-5p	-0,080879931	14,30597172	0,269685677	0,995576483	LADA vs T1D Time-adjusted
hsa-miR-140-3p	-0,142992561	9,535668537	0,29122425	0,995576483	LADA vs T1D Time-adjusted
hsa-miR-30d-5p	-0,071315449	14,41746295	0,326983856	0,995576483	LADA vs T1D Time-adjusted
hsa-miR-150-5p	0,172806504	12,04909227	0,347510466	0,995576483	LADA vs T1D Time-adjusted
hsa-miR-375-3p	-0,207727959	10,64120473	0,378869703	0,995576483	LADA vs T1D Time-adjusted

hsa-miR-146a-5p	0,086705158	12,49620569	0,397670146	0,995576483	LADA vs T1D Time-adjusted
hsa-miR-101-3p	-0,096585315	9,461671448	0,43496175	0,995576483	LADA vs T1D Time-adjusted
hsa-miR-142-5p	0,084241542	11,39821686	0,44527455	0,995576483	LADA vs T1D Time-adjusted
hsa-miR-451a1	0,112274629	16,83312299	0,465773935	0,995576483	LADA vs T1D Time-adjusted
hsa-miR-191-5p	0,052951323	12,34906605	0,474295293	0,995576483	LADA vs T1D Time-adjusted
hsa-miR-93-5p	-0,076177296	10,24046846	0,487515815	0,995576483	LADA vs T1D Time-adjusted
hsa-miR-23a-3p	-0,055641251	12,54700785	0,511318186	0,995576483	LADA vs T1D Time-adjusted
hsa-miR-16-5p	-0,075106976	14,33676009	0,516219796	0,995576483	LADA vs T1D Time-adjusted
hsa-miR-92a-3p	-0,07555822	17,44154735	0,51938381	0,995576483	LADA vs T1D Time-adjusted
hsa-miR-27b-3p	0,069926585	10,12130923	0,561242939	0,995576483	LADA vs T1D Time-adjusted
hsa-let-7d-3p	0,048266198	12,72440102	0,57604051	0,995576483	LADA vs T1D Time-adjusted
hsa-let-7f-5p	-0,062060427	13,26251625	0,58120818	0,995576483	LADA vs T1D Time-adjusted
hsa-miR-103a-3p	-0,075915749	9,552423125	0,594047572	0,995576483	LADA vs T1D Time-adjusted
hsa-miR-98-5p	-0,092516381	8,777064989	0,603513421	0,995576483	LADA vs T1D Time-adjusted
hsa-miR-143-3p	-0,070639551	10,68681172	0,604640638	0,995576483	LADA vs T1D Time-adjusted
hsa-miR-222-3p	-0,07085268	9,646980979	0,610059237	0,995576483	LADA vs T1D Time-adjusted
hsa-miR-363-3p	0,072846638	9,989550224	0,624384617	0,995576483	LADA vs T1D Time-adjusted
hsa-miR-22-3p	-0,043365408	12,7696365	0,632467384	0,995576483	LADA vs T1D Time-adjusted
hsa-miR-30c-5p	0,049253251	10,78220275	0,641560592	0,995576483	LADA vs T1D Time-adjusted
hsa-miR-486-5p	-0,073156707	18,24569138	0,643478883	0,995576483	LADA vs T1D Time-adjusted
hsa-let-7a-5p	0,037063087	14,35554361	0,653088825	0,995576483	LADA vs T1D Time-adjusted
hsa-miR-361-5p	0,055929354	7,799497125	0,672820614	0,995576483	LADA vs T1D Time-adjusted
hsa-miR-25-3p	-0,066907607	12,71958248	0,675986339	0,995576483	LADA vs T1D Time-adjusted
hsa-miR-4433b-5p	-0,135399959	10,31487352	0,679615856	0,995576483	LADA vs T1D Time-adjusted
hsa-miR-26b-5p	0,062680125	10,19799103	0,69280309	0,995576483	LADA vs T1D Time-adjusted
hsa-miR-197-3p	-0,059670895	10,63836899	0,694310042	0,995576483	LADA vs T1D Time-adjusted
hsa-miR-4841	-0,062096023	11,47479359	0,696903538	0,995576483	LADA vs T1D Time-adjusted
hsa-miR-122-5p	-0,083196119	14,13006949	0,738911682	0,996727444	LADA vs T1D Time-adjusted
hsa-miR-128-3p	-0,029214858	9,594398688	0,746108645	0,996727444	LADA vs T1D Time-adjusted
hsa-miR-146b-5p	-0,03021649	9,126180253	0,787747108	0,996727444	LADA vs T1D Time-adjusted
hsa-miR-26a-5p	0,038456041	12,10206399	0,794174448	0,996727444	LADA vs T1D Time-adjusted
hsa-miR-29a-3p	0,034801497	10,29575836	0,801244438	0,996727444	LADA vs T1D Time-adjusted
hsa-let-7c-5p	0,037387806	8,318110139	0,810448764	0,996727444	LADA vs T1D Time-adjusted
hsa-miR-99a-5p	0,034409561	9,686792179	0,814268264	0,996727444	LADA vs T1D Time-adjusted
hsa-miR-151a-3p	0,038810173	9,028748392	0,820357791	0,996727444	LADA vs T1D Time-adjusted
hsa-miR-186-5p	-0,022685526	9,709133341	0,835839746	0,996727444	LADA vs T1D Time-adjusted
hsa-miR-342-3p	0,024226094	10,76380446	0,886167041	0,996727444	LADA vs T1D Time-adjusted
hsa-miR-425-5p	-0,010730675	11,613117	0,896686581	0,996727444	LADA vs T1D Time-adjusted
hsa-miR-21-5p	-0,010475673	13,68104609	0,902555917	0,996727444	LADA vs T1D Time-adjusted
hsa-miR-182-5p	-0,017343324	9,626298557	0,913060018	0,996727444	LADA vs T1D Time-adjusted
hsa-miR-148a-3p	-0,012792667	9,813572581	0,914044015	0,996727444	LADA vs T1D Time-adjusted
hsa-miR-335-5p	0,013587923	9,217895277	0,924651781	0,996727444	LADA vs T1D Time-adjusted
hsa-let-7g-5p	0,00718178	12,38158425	0,925532626	0,996727444	LADA vs T1D Time-adjusted
hsa-miR-223-3p	-0,0097745	11,91868399	0,955040356	0,999512455	LADA vs T1D Time-adjusted
hsa-miR-193a-5p	-0,003619496	9,188089353	0,982623095	0,999512455	LADA vs T1D Time-adjusted
hsa-miR-181a-5p	0,001059303	10,87889244	0,990507151	0,999512455	LADA vs T1D Time-adjusted
hsa-miR-144-5p	-0,002165013	7,813745406	0,991344397	0,999512455	LADA vs T1D Time-adjusted
hsa-miR-125b-5p	8,78883E-05	10,48393745	0,999512455	0,999512455	LADA vs T1D Time-adjusted
hsa-miR-140-3p	-0,459036674	9,535668537	0,000574584	0,040220862	LADA vs T2D Time-adjusted
hsa-let-7d-3p	0,266181497	12,72440102	0,001706786	0,046371547	LADA vs T2D Time-adjusted
hsa-miR-10b-5p	0,346529404	11,95396841	0,002056551	0,046371547	LADA vs T2D Time-adjusted
hsa-miR-93-5p	-0,323173614	10,24046846	0,002649803	0,046371547	LADA vs T2D Time-adjusted
hsa-miR-186-5p	-0,292136186	9,709133341	0,006447616	0,080146942	LADA vs T2D Time-adjusted
hsa-miR-197-3p	0,40128254	10,63836899	0,006869738	0,080146942	LADA vs T2D Time-adjusted
hsa-miR-425-5p	-0,187963192	11,613117	0,019436332	0,194363322	LADA vs T2D Time-adjusted
hsa-miR-27a-3p	0,246893294	10,10682419	0,033246102	0,236714788	LADA vs T2D Time-adjusted
hsa-miR-486-3p	-0,414971641	8,576611099	0,03421688	0,236714788	LADA vs T2D Time-adjusted
hsa-miR-99b-5p	0,249131081	10,13632976	0,037297077	0,236714788	LADA vs T2D Time-adjusted
hsa-miR-30d-5p	-0,142264277	14,41746295	0,044562937	0,236714788	LADA vs T2D Time-adjusted
hsa-miR-151a-3p	-0,334032601	9,028748392	0,044709441	0,236714788	LADA vs T2D Time-adjusted
hsa-miR-16-5p	-0,223906194	14,33676009	0,047177637	0,236714788	LADA vs T2D Time-adjusted
hsa-miR-10a-5p	0,216346928	11,41317012	0,047342958	0,236714788	LADA vs T2D Time-adjusted
hsa-miR-146b-5p	-0,211858781	9,126180253	0,052480075	0,242232821	LADA vs T2D Time-adjusted
hsa-miR-122-5p	-0,464806046	14,13006949	0,055367502	0,242232821	LADA vs T2D Time-adjusted
hsa-miR-125a-5p	0,207051266	12,06715528	0,07151769	0,294484606	LADA vs T2D Time-adjusted
hsa-miR-25-3p	-0,261370136	12,71958248	0,092435276	0,346908168	LADA vs T2D Time-adjusted

hsa-miR-423-5p	0,12952244	15,43363167	0,094160789	0,346908168	LADA vs T2D Time-adjusted
hsa-miR-4842	-0,253358961	11,47479359	0,101085166	0,353798081	LADA vs T2D Time-adjusted
hsa-miR-143-3p	-0,207587915	10,68681172	0,116947816	0,389826054	LADA vs T2D Time-adjusted
hsa-miR-223-3p	-0,258449229	11,91868399	0,12327417	0,392235994	LADA vs T2D Time-adjusted
hsa-miR-30e-5p	-0,088823723	12,17327791	0,138385215	0,409777081	LADA vs T2D Time-adjusted
hsa-miR-652-3p	-0,163049131	8,921155655	0,142050522	0,409777081	LADA vs T2D Time-adjusted
hsa-miR-363-3p	-0,209547952	9,989550224	0,146348957	0,409777081	LADA vs T2D Time-adjusted
hsa-miR-361-5p	-0,179989037	7,799497125	0,162714609	0,438077794	LADA vs T2D Time-adjusted
hsa-miR-181a-5p	-0,113205162	10,87889244	0,189806245	0,492090264	LADA vs T2D Time-adjusted
hsa-miR-148a-3p	-0,148113938	9,813572581	0,197390968	0,493477419	LADA vs T2D Time-adjusted
hsa-miR-4433b-5p	0,389258283	10,31487352	0,22086008	0,533110538	LADA vs T2D Time-adjusted
hsa-miR-21-5p	0,096658043	13,68104609	0,243985266	0,569298954	LADA vs T2D Time-adjusted
hsa-miR-30a-5p	0,113543038	10,44721948	0,266037599	0,600730062	LADA vs T2D Time-adjusted
hsa-miR-150-5p	0,184774997	12,04909227	0,29840585	0,621371128	LADA vs T2D Time-adjusted
hsa-miR-182-5p	-0,152639462	9,626298557	0,321928121	0,621371128	LADA vs T2D Time-adjusted
hsa-let-7i-5p	-0,0938941	12,5425511	0,333300935	0,621371128	LADA vs T2D Time-adjusted
hsa-miR-30c-5p	-0,098929843	10,78220275	0,333754046	0,621371128	LADA vs T2D Time-adjusted
hsa-miR-125b-5p	0,134529316	10,48393745	0,334626902	0,621371128	LADA vs T2D Time-adjusted
hsa-miR-126-3p	0,110147146	11,83461714	0,340758526	0,621371128	LADA vs T2D Time-adjusted
hsa-miR-22-3p	-0,08357478	12,7696365	0,34163089	0,621371128	LADA vs T2D Time-adjusted
hsa-miR-144-5p	-0,18240937	7,813745406	0,346192486	0,621371128	LADA vs T2D Time-adjusted
hsa-miR-451a2	-0,135817944	16,83312299	0,365291252	0,634849152	LADA vs T2D Time-adjusted
hsa-miR-103a-3p	0,123260347	9,552423125	0,371840218	0,634849152	LADA vs T2D Time-adjusted
hsa-let-7a-5p	0,069330461	14,35554361	0,385582544	0,639869014	LADA vs T2D Time-adjusted
hsa-miR-101-3p	-0,102389543	9,461671448	0,393062394	0,639869014	LADA vs T2D Time-adjusted
hsa-miR-26a-5p	0,118180449	12,10206399	0,407665226	0,648558314	LADA vs T2D Time-adjusted
hsa-miR-191-5p	0,054617581	12,34906605	0,445207465	0,678742821	LADA vs T2D Time-adjusted
hsa-miR-222-3p	-0,101993899	9,646980979	0,448203808	0,678742821	LADA vs T2D Time-adjusted
hsa-miR-27b-3p	-0,086839933	10,12130923	0,455727323	0,678742821	LADA vs T2D Time-adjusted
hsa-miR-335-5p	0,09564436	9,217895277	0,49265062	0,705947004	LADA vs T2D Time-adjusted
hsa-miR-92a-3p	0,077997771	17,44154735	0,494162903	0,705947004	LADA vs T2D Time-adjusted
hsa-let-7e-5p	0,114940297	8,252607353	0,522910378	0,732074529	LADA vs T2D Time-adjusted
hsa-miR-423-3p	-0,060943711	12,15169654	0,556317052	0,76074375	LADA vs T2D Time-adjusted
hsa-miR-486-5p	0,088355654	18,24569138	0,565123929	0,76074375	LADA vs T2D Time-adjusted
hsa-miR-15b-5p	-0,058683507	9,988569024	0,578429574	0,763963588	LADA vs T2D Time-adjusted
hsa-miR-99a-5p	-0,075187775	9,686792179	0,596075499	0,76697685	LADA vs T2D Time-adjusted
hsa-miR-142-5p	-0,054103466	11,39821686	0,611464659	0,76697685	LADA vs T2D Time-adjusted
hsa-miR-26b-5p	0,075871446	10,19799103	0,621046385	0,76697685	LADA vs T2D Time-adjusted
hsa-miR-320a-3p	0,051997912	12,14832719	0,624538292	0,76697685	LADA vs T2D Time-adjusted
hsa-let-7g-5p	-0,034609863	12,38158425	0,641493801	0,774216657	LADA vs T2D Time-adjusted
hsa-miR-23a-3p	0,031066572	12,54700785	0,704432392	0,835767245	LADA vs T2D Time-adjusted
hsa-miR-98-5p	0,06060667	8,777064989	0,724700883	0,845484364	LADA vs T2D Time-adjusted
hsa-miR-342-3p	0,051937908	10,76380446	0,750818255	0,861594719	LADA vs T2D Time-adjusted
hsa-miR-375-3p	-0,062319031	10,64120473	0,784940104	0,886222698	LADA vs T2D Time-adjusted
hsa-miR-128-3p	-0,020187313	9,594398688	0,81713816	0,902142758	LADA vs T2D Time-adjusted
hsa-let-7c-5p	-0,033539821	8,318110139	0,824816236	0,902142758	LADA vs T2D Time-adjusted
hsa-miR-29a-3p	-0,023780143	10,29575836	0,858702034	0,924756037	LADA vs T2D Time-adjusted
hsa-let-7f-5p	0,004615383	13,26251625	0,966190778	0,996483038	LADA vs T2D Time-adjusted
hsa-miR-24-3p	0,003068714	13,24628442	0,970364376	0,996483038	LADA vs T2D Time-adjusted
hsa-miR-146a-5p	-0,002703675	12,49620569	0,978244063	0,996483038	LADA vs T2D Time-adjusted
hsa-let-7b-5p	0,001353877	14,30597172	0,984746411	0,996483038	LADA vs T2D Time-adjusted
hsa-miR-193a-5p	0,000710142	9,188089353	0,996483038	0,996483038	LADA vs T2D Time-adjusted

ESM table 2

ESM Table 2: Results from differentially expression analysis of IsomiRs. The comparions are indicated in the column "Comparison" such that an isomiR with positive "logFC" value in T1D vs T2D means that the isomiR is up-regulated in T1D vs T2D. "IsomiR" indicate the isomiR name and sequence; "logFC" in the log2 fold change from limma; "Average Expression" is the average expression of the isomiR across all samples; "P-Value" is the P-value for the specific isomiR for the specific compariosn before multiple testing; "Adjusted P-Value" is the benjamini-hochberg adjusted P-value. Time-adjusted comparisons is the results from limma-voom with time from diagnosis to serum collections as a covariate.

IsomiR	logFC	Average Expression	P-Value	Adjusted P-Value	Comparison
hsa-miR-142-5p_CCCATAAAGTAGAAAGCA	-2,349690077	2,550307907	9,89919E-07	0,000788965	T1D vs T2D
hsa-miR-140-3p_TACCACAGGGTAGAACCACGG	-1,458928826	5,563733367	4,63703E-06	0,001847856	T1D vs T2D
hsa-miR-23b-3p_ATCACATTGCCAGGGATTAC	-2,170993918	2,694770844	4,51554E-05	0,011996274	T1D vs T2D
hsa-miR-421_ATCAACAGACATTAATTGGGCG	-2,034012713	2,09434672	7,03505E-05	0,012148004	T1D vs T2D
hsa-miR-361-3p_TCCCCCAGGTGTGATTCTGATT	-1,894938966	3,650889743	7,62108E-05	0,012148004	T1D vs T2D
hsa-miR-101-3p_GTACAGTACTGTGATAACTG	-1,449994288	5,734652626	9,93845E-05	0,01320157	T1D vs T2D
hsa-miR-25-3p_ATTGCACTTGTCTCGGTCT	-1,850630464	3,620590845	0,000143228	0,016307571	T1D vs T2D
hsa-miR-223-3p_TGTCAGTTTGTCAAATACC	-1,487934395	5,409428462	0,000178124	0,017745615	T1D vs T2D
hsa-miR-363-3p_ATTGCACGGTATCCATCTGT	-1,686887765	4,343538892	0,000474167	0,039256218	T1D vs T2D
hsa-miR-29a-3p_CTAGCACCATCTGAAATCGG	-1,830029147	3,050349736	0,00049255	0,039256218	T1D vs T2D
hsa-miR-181b-5p_AACATTCATTGCTGTCGGTGGGT	-1,674501451	3,594503523	0,000618709	0,039988409	T1D vs T2D
hsa-miR-92a-3p_TATTGCACTTGTCCCGG	-1,493952059	4,594134091	0,000643097	0,039988409	T1D vs T2D
hsa-let-7d-3p_CTATACGACCTGCTGCCTTTC	0,347401156	10,32598693	0,000652258	0,039988409	T1D vs T2D
hsa-miR-23a-3p_TCACATTGCCAGGGATTTCCAAC	-1,763158446	2,126412114	0,000749898	0,042690645	T1D vs T2D
hsa-miR-16-2-3p_ACCAATATTACTGTGCTGCT	-1,49297703	4,606374691	0,001005428	0,052525474	T1D vs T2D
hsa-miR-186-5p_CAAAGAATTCTCCTTTTGGGC	-0,925454338	6,535812742	0,001054464	0,052525474	T1D vs T2D
hsa-miR-130b-3p_CAGTGCAATGATGAAAGGGCA	-1,389997871	4,137452675	0,001261108	0,059123723	T1D vs T2D
hsa-miR-584-5p_TTATGGTTTGCCTGGGACT	-1,413137175	4,595669388	0,001343357	0,059480883	T1D vs T2D
hsa-miR-140-3p_TACCACAGGGTAGAACCACGGACA	-1,552926286	3,637619462	0,001667404	0,067448696	T1D vs T2D
hsa-miR-23b-3p_ATCACATTGCCAGGGATTACC	-1,056814361	5,483511586	0,00181535	0,067448696	T1D vs T2D
hsa-miR-128-3p_TCACAGTGAACCGGTCTCTTTT	-1,189136406	5,404238817	0,001882318	0,067448696	T1D vs T2D
hsa-miR-139-5p_TCTACAGTGCACGTGTCTCCA	-1,482420592	4,69390328	0,001992854	0,067448696	T1D vs T2D
hsa-miR-340-3p_TCCGTCTCAGTTACTTTATAGCC	-1,762828395	3,094594983	0,002036595	0,067448696	T1D vs T2D
hsa-miR-106b-3p_CCGCACTGTGGGTACTTGCTG	-1,396167296	4,162035136	0,00215432	0,067448696	T1D vs T2D
hsa-miR-451a_GAAACCGTTACCATTACTGAG	-1,566641356	3,176702933	0,002295516	0,067448696	T1D vs T2D
hsa-let-7e-5p_TGAGGTAGGAGGTTGTATAGT	-1,41083011	4,645402812	0,002295906	0,067448696	T1D vs T2D
hsa-miR-150-3p_CTGGTACAGGCCTGGGGGACA	-1,625517817	4,060108896	0,002306189	0,067448696	T1D vs T2D
hsa-miR-25-3p_ATTGCACTTGTCTCGGTCTG	-1,516953748	3,200649251	0,002462459	0,067448696	T1D vs T2D
hsa-miR-142-5p_CATAAAGTAGAAAGCACT	-0,81863993	5,539841362	0,002526967	0,067448696	T1D vs T2D
hsa-miR-30a-5p_TGTAAACATCCTCGACTGGAA	-0,613388341	7,169227794	0,002538847	0,067448696	T1D vs T2D
hsa-miR-186-5p_CAAAGAATTCTCCTTTTGGGCT	-0,37369356	9,032677455	0,003050128	0,077328071	T1D vs T2D
hsa-miR-151a-3p_TACTAGACTGAAGCTCCTTGAGG	-1,512263244	4,127573648	0,003167426	0,077328071	T1D vs T2D
hsa-miR-215-5p_ATGACCTATGAATTGACAGA	1,51288982	4,790158483	0,00320179	0,077328071	T1D vs T2D
hsa-miR-423-5p_CTGAGGGGCAGAGAGCGAGACT	-1,57837071	2,475285897	0,003362011	0,078809481	T1D vs T2D
hsa-miR-451a_AAACCGTTACCATTACTG	-1,496950751	2,491678877	0,003569612	0,080764712	T1D vs T2D
hsa-let-7a-5p_TGAGGTAGTAGGTTGTATA	-1,003667207	5,663865648	0,003648092	0,080764712	T1D vs T2D
hsa-miR-335-5p_TCAAGAGCAATAACGAAAAATGT	-0,989808644	6,149787911	0,003861165	0,083171582	T1D vs T2D
hsa-miR-7-5p_TGGAAGACTAGTGATTTTGTTG	-1,353309464	3,890826404	0,004398783	0,08914882	T1D vs T2D
hsa-miR-409-3p_GAATGTTGCTCGGTGAACCCCTTT	-1,677855791	4,139609499	0,004448353	0,08914882	T1D vs T2D
hsa-miR-181a-2-3p_ACCACTGACCGTTGACTGT	-1,55505958	2,531276889	0,00459	0,08914882	T1D vs T2D
hsa-miR-25-3p_ATTGCACTTGTCTCGGTCTGA	-0,933585724	5,837958082	0,004717141	0,08914882	T1D vs T2D
hsa-miR-93-5p_CAAAGTGCTGTTCGTGCAGGT	-1,051425093	4,918359962	0,004762929	0,08914882	T1D vs T2D
hsa-miR-10a-5p_TACCCTGTAGATCCGAATTTGTGT	-1,54609117	2,404757373	0,004994424	0,08914882	T1D vs T2D
hsa-miR-30d-5p_GTAAACATCCCCGACTGGAA	-1,133186123	4,975135303	0,005041921	0,08914882	T1D vs T2D
hsa-miR-542-3p_TGTGACAGATTGATAACTGA	-1,551421637	1,954666629	0,005113629	0,08914882	T1D vs T2D
hsa-miR-181a-2-3p_ACCACTGACCGTTGACTGTAC	-1,481453195	3,544826687	0,005218464	0,08914882	T1D vs T2D
hsa-let-7f-5p_TGAGGTAGTAGATTGTATA	-1,474908219	3,61271535	0,00546427	0,08914882	T1D vs T2D
hsa-miR-423-3p_AAGCTCGGTCTGAGGCCCCTCA	-1,488857035	2,923364981	0,005504782	0,08914882	T1D vs T2D

hsa-let-7b-5p_TGAGGTAGTAGGTTGTGT	-1,041313837	5,292834315	0,005574155	0,08914882	T1D vs T2D
hsa-miR-423-3p_AAGCTCGGTCTGAGGCCCCTCAG	-1,424161303	3,402313099	0,005677152	0,08914882	T1D vs T2D
hsa-miR-543_AAACATTCGCGGTGCACTTCTT	-1,453251464	3,845833985	0,005769521	0,08914882	T1D vs T2D
hsa-miR-423-3p_AGCTCGGTCTGAGGCCCCTCA	-0,890679071	6,171912051	0,005838484	0,08914882	T1D vs T2D
hsa-miR-197-3p_TTCACCACCTTCTCCACCCAG	0,483215396	8,762482756	0,005928341	0,08914882	T1D vs T2D
hsa-miR-451a_AACCGTTACCATTACTGAGTT	-0,828411592	6,297926904	0,006335463	0,090668353	T1D vs T2D
hsa-miR-126-5p_CATTATTACTTTTGGTACGCG	-0,645666525	6,678225687	0,006359191	0,090668353	T1D vs T2D
hsa-miR-27b-3p_TTCACAGTGGCTAAGTTCTGCA	-1,440550345	3,596858077	0,006451307	0,090668353	T1D vs T2D
hsa-miR-25-3p_CATTGCACTTGTCTCGGT	-1,341203259	3,623128964	0,006484437	0,090668353	T1D vs T2D
hsa-miR-150-5p_GTCTCCCAACCCTTGTACCAGT	-1,467428984	2,340151294	0,006819772	0,093380879	T1D vs T2D
hsa-miR-1294_TGTGAGGTTGGCATTGTTGT	-1,492833009	2,481086687	0,006985297	0,093380879	T1D vs T2D
hsa-miR-221-3p_AGCTACATTGTCTGCTGGGTTTC	-0,830772545	6,752581432	0,007029928	0,093380879	T1D vs T2D
hsa-miR-222-3p_AGCTACATCTGGCTACTGGGTCT	-0,74041211	6,5045561	0,007979972	0,104262908	T1D vs T2D
hsa-miR-1306-5p_CCACCTCCCCTGCAAACGT	1,598697315	2,734536196	0,008522131	0,109550621	T1D vs T2D
hsa-miR-30d-5p_TGTAAACATCCCCGACTGGA	-0,378679548	9,18587305	0,009835895	0,121360812	T1D vs T2D
hsa-miR-505-3p_CGTCAACACTTGCTGGTTTCCT	-1,356560646	3,899410508	0,009849695	0,121360812	T1D vs T2D
hsa-miR-93-5p_CAAAGTGCTGTTCGTGCAGGTA	-0,995126908	5,442816943	0,009897682	0,121360812	T1D vs T2D
hsa-miR-10b-5p_TACCCTGTAGAACCGAATTTGTGT	-1,422446231	2,268128931	0,010249328	0,123768403	T1D vs T2D
hsa-miR-192-5p_TGACCTATGAATTGACAGCCAGT	-1,335523259	3,322199511	0,010518279	0,125120429	T1D vs T2D
hsa-miR-183-5p_TATGGCACTGGTAGAATTC	-1,336151132	3,376816673	0,01205568	0,141299656	T1D vs T2D
hsa-miR-2110_TTGGGGAAACGGCCGCTGAGT	-0,817413993	5,974874685	0,012518501	0,144400988	T1D vs T2D
hsa-miR-140-3p_ACCACAGGGTAGAACCACGGACA	-1,186401475	4,192134912	0,012818014	0,144400988	T1D vs T2D
hsa-miR-502-3p_AATGCACCTGGGCAAGGATTCA	-1,023729619	4,709212787	0,012981832	0,144400988	T1D vs T2D
hsa-miR-92a-3p_GTATTGCACTTGTCCCGGCCTG	-1,361346473	3,065032086	0,013045008	0,144400988	T1D vs T2D
hsa-miR-451a_ACCGTTACCATTACTGAGT	-0,595750208	7,452815255	0,013859276	0,149375208	T1D vs T2D
hsa-miR-142-5p_CCCATAAAGTAGAAAGCAC	-0,846931802	5,201334155	0,013869216	0,149375208	T1D vs T2D
hsa-miR-1180-3p_TTTCCGGCTCGCGTGGGTGT	-1,140393927	4,070138113	0,014431971	0,152612693	T1D vs T2D
hsa-miR-183-5p_ATGGCACTGGTAGAATTCACTGT	-1,360970083	2,224937851	0,014649771	0,152612693	T1D vs T2D
hsa-miR-140-3p_ACCACAGGGTAGAACCACGG	-1,246946438	3,491351538	0,014819083	0,152612693	T1D vs T2D
hsa-miR-151a-3p_TACTAGACTGAAGCTCCTTGAG	-1,313512038	3,055291554	0,014935747	0,152612693	T1D vs T2D
hsa-let-7e-5p_TGAGGTAGGAGGTTGTATAGTT	-0,507177727	7,813417368	0,015145894	0,15280098	T1D vs T2D
hsa-miR-484_TCAGGCTCAGTCCCCTCCCGAT	-0,321020337	9,18414004	0,015811181	0,157518887	T1D vs T2D
hsa-miR-139-5p_TCTACAGTGCACGTGTCTCCAGT	-0,631314859	6,587018882	0,016035257	0,157779011	T1D vs T2D
hsa-miR-126-3p_TCGTACCGTGAGTAATAATGC	-0,56624782	6,426310401	0,016830645	0,16358566	T1D vs T2D
hsa-miR-145-5p_GTCCAGTTTTCCCAGGAATCCC	-1,364872002	3,12605684	0,017886038	0,171749063	T1D vs T2D
hsa-miR-10a-5p_TACCCTGTAGATCCGAATTTGTG	-0,53084561	7,257876357	0,01827512	0,173396082	T1D vs T2D
hsa-miR-30c-5p_GTAAACATCCTACACTCTCAGCT	-1,328805356	3,052199181	0,018654189	0,173529135	T1D vs T2D
hsa-let-7i-5p_GAGGTAGTAGTTTGTGCTGTT	-1,311649145	3,257044134	0,018888298	0,173529135	T1D vs T2D
hsa-miR-21-5p_AGCTTATCAGACTGATGTTGA	-0,507097424	6,721668578	0,018942327	0,173529135	T1D vs T2D
hsa-miR-126-5p_ATTATTACTTTTGGTACGCGCT	-1,27226683	2,122638684	0,019576805	0,17537124	T1D vs T2D
hsa-miR-451a_GAAACCGTTACCATTACTGAGT	-0,540750562	6,98332366	0,019583488	0,17537124	T1D vs T2D
hsa-miR-125a-5p_TCCCTGAGACCCTTTAACCT	-0,562241924	6,34415738	0,021138126	0,18359107	T1D vs T2D
hsa-miR-186-5p_CAAAGAATTCTCCTTTTGGGCTT	-0,731060329	6,345307365	0,021310649	0,18359107	T1D vs T2D
hsa-miR-361-5p_TTATCAGAATCTCCAGGGGTA	-1,231697393	3,298750595	0,02147932	0,18359107	T1D vs T2D
hsa-miR-451a_AAACCGTTACCATTACTGA	-0,405224689	8,929457213	0,021809929	0,18359107	T1D vs T2D
hsa-miR-374b-5p_ATATAATACAACCTGCTAAGTG	-1,237133086	2,723967557	0,02186313	0,18359107	T1D vs T2D
hsa-miR-484_CAGGCTCAGTCCCCTCCCGA	-1,28221904	3,768443499	0,022332689	0,18359107	T1D vs T2D
hsa-miR-197-3p_TTCACCACCTTCTCCACCCAGC	0,354047906	10,1606215	0,022492639	0,18359107	T1D vs T2D
hsa-miR-3615_TCTCTCGGCTCCTCGCGGCTCG	0,307874663	8,091694617	0,022495918	0,18359107	T1D vs T2D
hsa-miR-140-3p_TACCACAGGGTAGAACCACGGA	-0,468477502	7,894124989	0,022574561	0,18359107	T1D vs T2D
hsa-miR-142-5p_CCCATAAAGTAGAAAGCACTAC	-0,540380596	7,021131164	0,022813559	0,183660673	T1D vs T2D
hsa-miR-30b-5p_TGTAAACATCCTACACTCAGCT	-0,582714659	6,722043115	0,023070654	0,18387311	T1D vs T2D
hsa-miR-451a_AAACCGTTACCATTACTGAGTT	-0,332221264	12,14012514	0,024675703	0,194718169	T1D vs T2D
hsa-miR-942-5p_TCTTCTCTGTTTTGGCCATGT	-1,250400558	2,592733465	0,025374184	0,196370177	T1D vs T2D
hsa-miR-125b-5p_TCCCTGAGACCCTAACTTGTGA	-0,826827408	5,6455681	0,025377827	0,196370177	T1D vs T2D
hsa-miR-142-5p_CATAAAGTAGAAAGCACTA	-1,149189534	2,153538022	0,02602824	0,197322552	T1D vs T2D
hsa-miR-28-3p_CACTAGATTGTGAGCTCCTGGA	-0,593759785	6,806864009	0,026144923	0,197322552	T1D vs T2D
hsa-let-7a-5p_GAGGTAGTAGGTTGTATAG	-0,871652461	5,403501302	0,026243652	0,197322552	T1D vs T2D
hsa-miR-145-5p_GTCCAGTTTTCCCAGGAATCC	-1,208534858	2,283533911	0,027076967	0,201281301	T1D vs T2D
hsa-miR-181a-5p_AACATTCAACGCTGTCGGTGAGT	-0,426443228	8,031521361	0,027558069	0,201281301	T1D vs T2D
hsa-miR-1180-3p_TTTCCGGCTCGCGTGGGTGTGT	-1,155742481	2,874816557	0,027589164	0,201281301	T1D vs T2D

hsa-miR-30e-3p_CTTTCAGTCGGATGTTTACAGC	-1,222904591	3,314941715	0,027780355	0,201281301	T1D vs T2D
hsa-miR-93-5p_AAAGTGCTGTTCGTGCAGGTAG	-1,112730473	3,611226251	0,028505041	0,204367625	T1D vs T2D
hsa-miR-30d-5p_GTAAACATCCCCGACTGGAAGCT	-0,549480046	6,998439264	0,028719164	0,204367625	T1D vs T2D
hsa-miR-2110_TTGGGGAAACGGCCGCTGAGTG	-1,280334486	2,932814217	0,029425363	0,206792345	T1D vs T2D
hsa-miR-92b-3p_TATTGCACTCGTCCCGGCC	-1,142149326	3,837730088	0,029754242	0,206792345	T1D vs T2D
hsa-miR-139-5p_TCTACAGTGCACGTGTCTCCAG	-0,962391614	4,676823231	0,029871165	0,206792345	T1D vs T2D
hsa-miR-122-5p_GGAGTGTGACAATGGTGTTTG	-0,934824562	5,615777051	0,030287743	0,206792345	T1D vs T2D
hsa-miR-30d-5p_TGTAAACATCCCCGACTGGAAGC	-0,215196195	11,59455452	0,03035722	0,206792345	T1D vs T2D
hsa-miR-146b-5p_TGAGAACTGAATTCCATAGGCTG	-0,781018758	5,765289472	0,031028367	0,20957295	T1D vs T2D
hsa-miR-361-5p_TTATCAGAATCTCCAGGGGT	-0,875256383	4,747764862	0,031996079	0,213659549	T1D vs T2D
hsa-miR-30c-5p_TGTAAACATCCTACACTCTCAGC	-0,583807046	6,545485648	0,032169568	0,213659549	T1D vs T2D
hsa-miR-574-3p_CACGCTCATGCACACACCCAC	-1,006967773	4,117697394	0,033734927	0,221955062	T1D vs T2D
hsa-miR-182-5p_TTTGGCAATGGTAGAACTCACACTG	-1,207673738	3,598930548	0,034169318	0,221955062	T1D vs T2D
hsa-miR-185-5p_TGGAGAGAAAGGCAGTTCCTG	-1,031906578	4,508281621	0,034254043	0,221955062	T1D vs T2D
hsa-miR-151a-3p_CTAGACTGAAGCTCCTTGAGGA	-0,368756193	8,444952042	0,035867567	0,22884998	T1D vs T2D
hsa-miR-183-5p_ATGGCACTGGTAGAATTCACTG	-1,178104495	2,888906189	0,035892406	0,22884998	T1D vs T2D
hsa-miR-363-3p_AATTGCACGGTATCCATCTG	-0,354816683	8,264741807	0,036863662	0,231547163	T1D vs T2D
hsa-miR-93-5p_CAAAGTGCTGTTCGTGCAGGTAG	-0,243219628	9,991178453	0,037017515	0,231547163	T1D vs T2D
hsa-miR-487b-3p_AATCGTACAGGGTCATCCACTT	-1,222812356	4,048228768	0,037205308	0,231547163	T1D vs T2D
hsa-miR-181b-5p_AACATTCATTGCTGTCGGTGG	-0,763689286	5,135854331	0,037734899	0,231547163	T1D vs T2D
hsa-miR-29c-3p_TAGCACCATTTGAAATCGGTT	-0,99137064	3,850371057	0,037932915	0,231547163	T1D vs T2D
hsa-miR-194-5p_TGTAACAGCAACTCCATGTGGAA	-1,103791178	2,659899918	0,038058568	0,231547163	T1D vs T2D
hsa-miR-92a-3p_ATTGCACTTGTCCCGGCCTGTT	-0,708542881	5,604292671	0,038730776	0,23385173	T1D vs T2D
hsa-miR-193a-5p_TGGGTCTTTGCGGGCGAGAT	-0,91776943	4,669337288	0,039196834	0,234886291	T1D vs T2D
hsa-miR-425-5p_AATGACACGATCACTCCCGTTG	-0,255274766	9,36928751	0,039572681	0,235368857	T1D vs T2D
hsa-miR-27b-3p_TTCACAGTGGCTAAGTTCTG	-0,361563499	8,279294906	0,040134868	0,236944368	T1D vs T2D
hsa-miR-363-3p_AATTGCACGGTATCCATCTGT	-0,302537458	8,977144446	0,040466695	0,236965401	T1D vs T2D
hsa-miR-182-5p_TTTGGCAATGGTAGAACTCAC	-1,109267226	3,148572393	0,040733074	0,236965401	T1D vs T2D
hsa-miR-132-3p_TAACAGTCTACAGCCATGGTCG	-1,115259927	2,233528136	0,041285017	0,238435932	T1D vs T2D
hsa-miR-484_CAGGCTCAGTCCCCTCCCGAT	-1,05203933	2,404164158	0,041867962	0,239510984	T1D vs T2D
hsa-miR-21-5p_AGCTTATCAGACTGATGTTGAC	-1,202241447	3,189772648	0,042072193	0,239510984	T1D vs T2D
hsa-miR-6803-3p_TCCCTCGCCTTCTCACCCTCA	0,640946689	6,298415012	0,043610149	0,246505593	T1D vs T2D
hsa-miR-183-5p_ATGGCACTGGTAGAATTCACT	-0,946652112	4,652978697	0,044096846	0,247501314	T1D vs T2D
hsa-miR-425-5p_ATGACACGATCACTCCCGTTG	-1,11412647	2,398751928	0,044600039	0,248575045	T1D vs T2D
hsa-miR-146a-5p_TGAGAACTGAATTCCATGGGTTGT	-0,572677228	7,265118969	0,045339119	0,249576323	T1D vs T2D
hsa-miR-92b-3p_TATTGCACTCGTCCCGGCCTCC	-0,927698799	4,414239688	0,045405981	0,249576323	T1D vs T2D
hsa-miR-10a-5p_ACCCTGTAGATCCGAATTTGTG	-0,606424902	6,001615458	0,045750643	0,249748372	T1D vs T2D
hsa-miR-146b-5p_TGAGAACTGAATTCCATAGGCTGT	-0,337058637	8,368012324	0,04642793	0,251721496	T1D vs T2D
hsa-miR-192-5p_TGACCTATGAATTGACAGCCAG	-1,074105922	3,652730706	0,046909938	0,252616355	T1D vs T2D
hsa-miR-451a_AAACCGTTACCATTACTGAGTTTAG	-1,080365308	2,164703683	0,048332609	0,257106865	T1D vs T2D
hsa-let-7f-5p_GAGGTAGTAGATTGTATAG	-1,014508911	3,245137192	0,048388996	0,257106865	T1D vs T2D
hsa-miR-191-5p_AACGGAATCCCAAAAGCAGCTG	-0,632172939	5,747755673	0,048983559	0,257562735	T1D vs T2D
hsa-miR-451a_ACCGTTACCATTACTGAGTT	-1,066388535	2,004581894	0,049121124	0,257562735	T1D vs T2D
hsa-miR-16-5p_TTAGCAGCACGTAAATATTGGCG	-0,956141086	4,199452303	0,049697804	0,258883331	T1D vs T2D
hsa-miR-181a-5p_AACATTCAACGCTGTCGGTGAGTT	-1,094397502	2,334562434	0,051065787	0,262208549	T1D vs T2D
hsa-let-7f-5p_TGAGGTAGTAGATTGTAT	-1,153577526	3,174737293	0,05118073	0,262208549	T1D vs T2D
hsa-miR-16-5p_TAGCAGCACGTAAATATTGGC	-0,237042685	9,94541903	0,051323129	0,262208549	T1D vs T2D
hsa-miR-584-5p_TTATGGTTTGCCTGGGACTGA	-0,384806996	8,070467342	0,053482273	0,271487131	T1D vs T2D
hsa-let-7b-5p_GAGGTAGTAGGTTGTGTGGT	-0,831885797	4,944887656	0,054103807	0,271487131	T1D vs T2D
hsa-miR-361-5p_TTATCAGAATCTCCAGGGGTAC	-0,476635866	6,634693775	0,054161172	0,271487131	T1D vs T2D
hsa-miR-16-5p_CTAGCAGCACGTAAATATTGGCG	-0,805195709	4,959212286	0,055204677	0,272345322	T1D vs T2D
hsa-miR-584-5p_TTATGGTTTGCCTGGGACTG	-1,008739226	2,558465624	0,055510153	0,272345322	T1D vs T2D
hsa-miR-425-5p_AATGACACGATCACTCCCGTT	-0,388087905	8,024407696	0,055986805	0,272345322	T1D vs T2D
hsa-miR-30a-5p_TGTAAACATCCTCGACTGGAAGC	-0,328024785	7,798673355	0,056762765	0,272345322	T1D vs T2D
hsa-miR-21-3p_CAACACCAGTCGATGGGCTGT	-0,981697565	2,296740898	0,056802091	0,272345322	T1D vs T2D
hsa-miR-501-3p_AATGCACCCGGGCAAGGATTC	-1,025425155	3,102124992	0,056843231	0,272345322	T1D vs T2D
hsa-miR-140-3p_TACCACAGGGTAGAACCACG	-0,71468088	5,026346909	0,057194925	0,272345322	T1D vs T2D
hsa-miR-495-3p_AAACAAACATGGTGCACTTCTT	-1,150159751	2,485368847	0,057307493	0,272345322	T1D vs T2D
hsa-miR-191-5p_CAACGGAATCCCAAAAGCA	-0,543616624	6,203677568	0,057407797	0,272345322	T1D vs T2D
hsa-let-7b-5p_GAGGTAGTAGGTTGTGTGGTTT	-0,995647096	3,859782449	0,058967209	0,278087961	T1D vs T2D
hsa-miR-485-5p_AGAGGCTGGCCGTGATGAATTCG	-1,011508803	4,543642396	0,059888627	0,27965449	T1D vs T2D

hsa-miR-320a-3p_AAAAGCTGGGTTGAGAGGGCGAA	0,217928712	9,770949231	0,060556753	0,27965449	T1D vs T2D
hsa-miR-148b-3p_TCAGTGCATCACAGAACTTTGT	-0,371745411	6,965383646	0,060794744	0,27965449	T1D vs T2D
hsa-miR-1228-3p_TCACACCTGCCTCGCCCCCCA	1,141877073	1,951009842	0,061049196	0,27965449	T1D vs T2D
hsa-miR-16-5p_AGCAGCACGTAAATATTGG	-0,979461546	3,167012316	0,061148102	0,27965449	T1D vs T2D
hsa-miR-100-5p_AACCCGTAGATCCGAACTTGTG	-1,093757233	3,590895666	0,061404687	0,27965449	T1D vs T2D
hsa-miR-29a-3p_TAGCACCATCTGAAATCGGTTAT	-1,034923923	2,486065735	0,062072239	0,281088493	T1D vs T2D
hsa-miR-183-5p_TATGGCACTGGTAGAATTCACT	-0,72032368	5,796604044	0,062767418	0,28263069	T1D vs T2D
hsa-miR-10b-5p_ACCCTGTAGAACCGAATTTGTGT	-0,963066833	3,356457419	0,063230868	0,28282913	T1D vs T2D
hsa-miR-29c-3p_TAGCACCATTTGAAATCGG	-1,004128456	3,272314395	0,063896859	0,28282913	T1D vs T2D
hsa-miR-7-5p_TGGAAGACTAGTGATTTTGT	-0,992762763	3,91952121	0,063929442	0,28282913	T1D vs T2D
hsa-miR-532-5p_CATGCCTTGAGTGTAGGACCG	-0,97093052	2,388684445	0,064230957	0,28282913	T1D vs T2D
hsa-miR-22-3p_AAGCTGCCAGTTGAAGAACTG	-0,318726252	9,134304454	0,065341847	0,285156356	T1D vs T2D
hsa-miR-423-3p_AGCTCGGTCTGAGGCCCCT	-0,562972327	6,038348398	0,066595607	0,285156356	T1D vs T2D
hsa-miR-30a-5p_GTAAACATCCTCGACTGGAAGCT	-0,919977183	4,1060576	0,066681698	0,285156356	T1D vs T2D
hsa-miR-142-5p_CCCATAAAGTAGAAAGCACTA	-0,660907681	5,405299379	0,066760636	0,285156356	T1D vs T2D
hsa-miR-320b_AAAAGCTGGGTTGAGAGGGCA	-0,901211054	2,618155177	0,066785916	0,285156356	T1D vs T2D
hsa-miR-1306-5p_CCACCTCCCCTGCAAACGTC	0,821042268	4,129732962	0,066906196	0,285156356	T1D vs T2D
hsa-miR-25-3p_CATTGCACTTGTCTCGGTCTGA	-0,262031628	11,99390817	0,068773861	0,291557274	T1D vs T2D
hsa-miR-10b-5p_TACCCTGTAGAACCGAATTTG	0,225395021	9,833049317	0,069795063	0,294320981	T1D vs T2D
hsa-miR-24-3p_GCTCAGTTCAGCAGGAACAG	-0,943319442	2,25101941	0,072617958	0,304564057	T1D vs T2D
hsa-miR-140-3p_ACCACAGGGTAGAACCACGGAC	-0,597639492	5,852260421	0,073177167	0,304564057	T1D vs T2D
hsa-miR-181a-5p_ACATTCAACGCTGTCGGTGA	-0,925913175	3,569893472	0,073370513	0,304564057	T1D vs T2D
hsa-let-7c-5p_TGAGGTAGTAGGTTGTATGG	-0,635843898	3,04066672	0,07392655	0,305055008	T1D vs T2D
hsa-miR-10b-5p_TACCCTGTAGAACCGAATTT	-0,691777067	5,2763128	0,074254293	0,305055008	T1D vs T2D
hsa-miR-16-2-3p_CCAATATTACTGTGCTGCTT	-0,702109102	4,446564737	0,076757034	0,313632856	T1D vs T2D
hsa-miR-505-3p_CGTCAACACTTGCTGGTTT	-0,946543361	3,249239604	0,077129284	0,313632856	T1D vs T2D
hsa-let-7g-5p_TGAGGTAGTAGTTTGTACA	-0,757119855	5,425002874	0,077866521	0,315023438	T1D vs T2D
hsa-miR-93-3p_ACTGCTGAGCTAGCACTTCCCGA	-0,952503546	3,371774968	0,078818262	0,31726341	T1D vs T2D
hsa-miR-30c-5p_TGTAAACATCCTACACTCTCA	-0,364325076	7,955436132	0,080656089	0,320417029	T1D vs T2D
hsa-miR-101-3p_GTACAGTACTGTGATAACTGAA	-0,964992586	3,655178095	0,080927955	0,320417029	T1D vs T2D
hsa-miR-30d-5p_TAAACATCCCCGACTGGAAGCT	-0,941311074	2,174183718	0,081033943	0,320417029	T1D vs T2D
hsa-miR-423-5p_CTGAGGGGCAGAGAGCGAGACTTT	-0,851003803	4,419437576	0,081209837	0,320417029	T1D vs T2D
hsa-miR-223-3p_TGTCAGTTTGTCAAATACCCCAA	-0,324766563	10,91791178	0,082512802	0,323954201	T1D vs T2D
hsa-miR-21-5p_TAGCTTATCAGACTGATG	-0,93439549	1,940313304	0,084061159	0,32733503	T1D vs T2D
hsa-miR-106b-3p_CCGCACTGTGGGTACTTGCT	-0,548577097	6,396206525	0,084195334	0,32733503	T1D vs T2D
hsa-miR-484_TCAGGCTCAGTCCCCTCCCGATA	-0,748030088	4,637634197	0,085668556	0,331445822	T1D vs T2D
hsa-miR-451a_ACCGTTACCATTACTGAG	-0,852911297	3,857793403	0,087435531	0,336647913	T1D vs T2D
hsa-let-7g-5p_GAGGTAGTAGTTTGTACAGT	-0,953372816	3,423010066	0,089692285	0,341350262	T1D vs T2D
hsa-miR-155-5p_TTAATGCTAATCGTGATAGGGGTT	-0,973725041	2,993899159	0,089827037	0,341350262	T1D vs T2D
hsa-miR-3613-5p_TGTTGTACTTTTTTTTTTGTTC	-0,742494036	4,388236924	0,089989439	0,341350262	T1D vs T2D
hsa-miR-22-3p_AAGCTGCCAGTTGAAGAA	-0,798854441	4,931866064	0,090370019	0,341350262	T1D vs T2D
hsa-miR-101-3p_TACAGTACTGTGATAACTGAA	-0,938824184	3,070531279	0,091457675	0,343608947	T1D vs T2D
hsa-miR-652-3p_AATGGCGCCACTAGGGTTGTG	-0,45799977	6,468098031	0,092844305	0,343608947	T1D vs T2D
hsa-miR-500a-3p_AATGCACCTGGGCAAGGATTCT	-0,994321118	2,780066987	0,092850973	0,343608947	T1D vs T2D
hsa-miR-181a-5p_AACATTCAACGCTGTCGG	-0,847454056	2,192700831	0,093374802	0,343608947	T1D vs T2D
hsa-miR-423-5p_TGAGGGGCAGAGAGCGAGACTTT	0,11678672	14,42674435	0,093740225	0,343608947	T1D vs T2D
hsa-miR-339-5p_TCCCTGTCCTCCAGGAGCTCACG	-0,937694226	3,416847552	0,094056423	0,343608947	T1D vs T2D
hsa-miR-4433b-5p_TGTCCCACCCCCACTCCTG	1,012622003	2,497163245	0,094623023	0,343608947	T1D vs T2D
hsa-miR-425-5p_ATGACACGATCACTCCCGTTGA	-0,749228212	4,729605379	0,094830504	0,343608947	T1D vs T2D
hsa-miR-2110_TTGGGGAAACGGCCGCTGAG	-0,921969576	2,165591375	0,094848141	0,343608947	T1D vs T2D
hsa-miR-191-5p_CAACGGAATCCCAAAAGCAGC	-0,315481215	8,150484012	0,095664742	0,344999091	T1D vs T2D
hsa-miR-143-3p_TGAGATGAAGCACTGTAGCTCA	-0,48043599	6,738882834	0,096283609	0,345666832	T1D vs T2D
hsa-miR-92a-3p_TTGCACTTGTCCCGGCCTG	-0,785296973	4,313701526	0,097762683	0,349402951	T1D vs T2D
hsa-let-7a-5p_GAGGTAGTAGGTTGTATAGT	-0,763310103	4,701370889	0,099768163	0,353615954	T1D vs T2D
hsa-miR-320a-3p_AAAAGCTGGGTTGAGAGGGCGAAAA	-0,908137679	3,186668725	0,099828845	0,353615954	T1D vs T2D
hsa-miR-361-3p_TCCCCCAGGTGTGATTCTGATTT	-0,795863989	3,977031543	0,100874555	0,35573903	T1D vs T2D
hsa-miR-151a-3p_TACTAGACTGAAGCTCCTTGA	-0,864500427	1,940948476	0,101776653	0,357339172	T1D vs T2D
hsa-miR-425-5p_AATGACACGATCACTCCCGTTGA	-0,168394959	10,37803313	0,102425362	0,358039532	T1D vs T2D
hsa-miR-146b-5p_TGAGAACTGAATTCCATAGGCT	-0,458435453	6,34592746	0,105445926	0,366430998	T1D vs T2D
hsa-miR-191-5p_AACGGAATCCCAAAAGCAGC	-0,858817556	2,920397045	0,105745457	0,366430998	T1D vs T2D
hsa-miR-122-5p_GGAGTGTGACAATGGTGTTT	-0,560290278	7,666405464	0,10646325	0,366487124	T1D vs T2D

hsa-miR-99a-5p_AACCCGTAGATCCGATCTTGTG	-0,588356288	5,156061862	0,106681321	0,366487124	T1D vs T2D
hsa-miR-423-5p_GAGGGGCAGAGAGCGAGACTT	-0,63690438	5,367382166	0,108830505	0,371397767	T1D vs T2D
hsa-miR-106b-5p_TAAAGTGCTGACAGTGCAGAT	-0,82588916	2,655083548	0,109042757	0,371397767	T1D vs T2D
hsa-let-7i-5p_GAGGTAGTAGTTTGTGCTGT	-0,734019905	4,789553904	0,109627864	0,371801733	T1D vs T2D
hsa-miR-30d-5p_TGTAAACATCCCCGACTGGAAGCT	-0,195780316	13,20515822	0,110466572	0,37305872	T1D vs T2D
hsa-miR-223-3p_TGTCAGTTTGTCAAATACCC	-0,719422231	4,516322797	0,112380561	0,376374686	T1D vs T2D
hsa-miR-125a-5p_TCCCTGAGACCCTTTAACCTGTG	-0,244971818	8,600810288	0,112392943	0,376374686	T1D vs T2D
hsa-miR-92a-3p_ACTTGTCCCGGCCTGT	-0,888785373	2,134254049	0,112949318	0,376655257	T1D vs T2D
hsa-miR-664a-5p_ACTGGCTAGGGAAAATGATTGG	-0,817923671	3,587998944	0,115241288	0,381180293	T1D vs T2D
hsa-miR-140-5p_CAGTGGTTTTACCCTATGGTAG	-0,779218507	3,871751643	0,115262799	0,381180293	T1D vs T2D
hsa-miR-451a_AAACCGTTACCATTACTGAGTTTAGT	-0,67752426	4,187049919	0,116469567	0,383579524	T1D vs T2D
hsa-miR-194-5p_TGTAACAGCAACTCCATGTGG	-0,381911547	6,660853419	0,119722832	0,391519567	T1D vs T2D
hsa-miR-423-5p_GAGGGGCAGAGAGCGAGACTTT	0,195344706	8,388365906	0,1203503	0,391519567	T1D vs T2D
hsa-miR-375-3p_TTTGTTCGTTCGGCTCGCGTGA	0,362247664	10,08392589	0,120354196	0,391519567	T1D vs T2D
hsa-let-7d-3p_TATACGACCTGCTGCCTTTCT	0,197215416	8,488338468	0,121265961	0,392044414	T1D vs T2D
hsa-miR-425-5p_AATGACACGATCACTCCCG	-0,723171473	4,547943713	0,121640581	0,392044414	T1D vs T2D
hsa-let-7g-5p_TGAGGTAGTAGTTTGTACAGT	-0,130441793	10,7052364	0,122118798	0,392044414	T1D vs T2D
hsa-miR-22-3p_AGCTGCCAGTTGAAGAACT	-0,878435791	2,67043052	0,122483136	0,392044414	T1D vs T2D
hsa-miR-194-5p_TGTAACAGCAACTCCATGTGGA	-0,838824923	2,048630169	0,124176683	0,395270344	T1D vs T2D
hsa-miR-28-3p_CACTAGATTGTGAGCTCCTGG	-0,484235645	5,876182682	0,124717392	0,395270344	T1D vs T2D
hsa-miR-30d-5p_TGTAAACATCCCCGACTGGAA	-0,148604008	11,66083446	0,124978829	0,395270344	T1D vs T2D
hsa-miR-4433b-5p_ATGTCCCACCCCCACTCCTGTTT	-0,699384036	5,620502964	0,125651955	0,395828491	T1D vs T2D
hsa-miR-18a-3p_ACTGCCCTAAGTGCTCCTTCTG	-0,809858503	2,22678804	0,127041938	0,397968829	T1D vs T2D
hsa-miR-142-5p_CCATAAAGTAGAAAGCACT	-0,556023576	4,774985235	0,127330052	0,397968829	T1D vs T2D
hsa-miR-505-3p_CGTCAACACTTGCTGGTTTCCTCT	-0,884245335	2,947139365	0,130196349	0,403875331	T1D vs T2D
hsa-miR-142-5p_CATAAAGTAGAAAGCACTAC	-0,835556936	2,376484394	0,130547603	0,403875331	T1D vs T2D
hsa-miR-7-5p_TGGAAGACTAGTGATTTTGTTGT	-0,577331346	6,176558156	0,130831549	0,403875331	T1D vs T2D
hsa-miR-30a-3p_CTTTCAGTCGGATGTTTGCAG	-0,818834519	1,943754996	0,131246814	0,403875331	T1D vs T2D
hsa-miR-629-5p_TGGGTTTACGTTGGGAGAA	-0,880236095	2,515869067	0,131913411	0,404365339	T1D vs T2D
hsa-miR-486-5p_TCCTGTACTGAGCTGCCCCGAGG	-0,265882015	7,467002764	0,134746011	0,411465788	T1D vs T2D
hsa-miR-146a-5p_TGAGAACTGAATTCCATGGGT	-0,19850167	8,67354952	0,137179923	0,414879464	T1D vs T2D
hsa-miR-148a-3p_TCAGTGCACTACAGAACTTTGT	-0,203380939	9,289411705	0,137680484	0,414879464	T1D vs T2D
hsa-miR-484_TCAGGCTCAGTCCCCTCCCG	-0,353710256	7,780454359	0,137683173	0,414879464	T1D vs T2D
hsa-miR-222-3p_AGCTACATCTGGCTACTGGGTCTC	-0,583495495	6,101293098	0,13794612	0,414879464	T1D vs T2D
hsa-miR-146a-5p_TGAGAACTGAATTCCATGGGTT	-0,171158672	12,1607009	0,140070478	0,418045443	T1D vs T2D
hsa-miR-182-5p_TTTGGCAATGGTAGAACTC	-0,839900929	2,382812326	0,140374765	0,418045443	T1D vs T2D
hsa-let-7d-3p_CTATACGACCTGCTGCCTTT	0,188605645	10,24742095	0,140693897	0,418045443	T1D vs T2D
hsa-miR-25-3p_CATTGCACTTGTCTCGGTCTG	-0,251603718	9,990945876	0,141096894	0,418045443	T1D vs T2D
hsa-miR-22-3p_AGCTGCCAGTTGAAGAACTGT	-0,550179776	5,523006419	0,142595003	0,419922761	T1D vs T2D
hsa-miR-125b-5p_TCCCTGAGACCCTAACTTGT	0,208023816	9,802438562	0,142784276	0,419922761	T1D vs T2D
hsa-miR-21-5p_TAGCTTATCAGACTGATGT	-0,6747799	4,699515355	0,145479119	0,424365452	T1D vs T2D
hsa-miR-29a-3p_TAGCACCATCTGAAATCGGTTA	-0,661000465	4,716466347	0,145934804	0,424365452	T1D vs T2D
hsa-miR-451a_AAACCGTTACCATTACTGAGTTT	-0,231380747	8,99265157	0,146193478	0,424365452	T1D vs T2D
hsa-miR-23a-3p_TCACATTGCCAGGGATTTCCA	-0,572959945	5,236791923	0,146424717	0,424365452	T1D vs T2D
hsa-miR-494-3p_TGAAACATACACGGGAAACCTCT	-0,77415788	4,269868739	0,147590489	0,426194273	T1D vs T2D
hsa-miR-16-5p_GCAGCACGTAAATATTGGCG	-0,695903773	3,625120505	0,149210006	0,428248344	T1D vs T2D
hsa-miR-26a-5p_TCAAGTAATCCAGGATAGGCT	-0,419626541	6,522605962	0,149893213	0,428248344	T1D vs T2D
hsa-miR-99b-5p_CACCCGTAGAACCGACCTTGCG	-0,22572311	9,378733966	0,149913787	0,428248344	T1D vs T2D
hsa-miR-16-5p_TAGCAGCACGTAAATATTG	-0,483524558	5,566162995	0,15052984	0,428472438	T1D vs T2D
hsa-miR-30c-5p_TGTAAACATCCTACACTCTC	-0,796094952	2,069916362	0,152051399	0,431007858	T1D vs T2D
hsa-miR-486-5p_CTGTACTGAGCTGCCCCG	-0,785307424	3,40982355	0,152502153	0,431007858	T1D vs T2D
hsa-miR-342-3p_TCACACAGAAATCGCACCCGTCA	-0,755342109	3,930198822	0,154130162	0,431909839	T1D vs T2D
hsa-miR-451a_AAACCGTTACCATTACTGAG	-0,228059184	13,72832534	0,154371648	0,431909839	T1D vs T2D
hsa-miR-744-5p_TGCGGGGCTAGGGCTAACAGC	-0,704373619	3,991128142	0,154447057	0,431909839	T1D vs T2D
hsa-miR-339-3p_TGAGCGCCTCGACGACAGAGCCG	-0,769785453	4,16247252	0,157340535	0,438100137	T1D vs T2D
hsa-miR-181a-5p_AACATTCAACGCTGTCGGTGAG	-0,25567296	7,366086853	0,157997816	0,438100137	T1D vs T2D
hsa-miR-30a-5p_TGTAAACATCCTCGACTGGAAGCT	-0,171344842	8,778765877	0,158309711	0,438100137	T1D vs T2D
hsa-miR-340-3p_TCCGTCTCAGTTACTTTATAGC	-0,733228881	1,820184636	0,159108671	0,438787579	T1D vs T2D
hsa-miR-342-3p_TCTCACACAGAAATCGCACCCGTC	-0,274549619	7,583667414	0,160377127	0,440760587	T1D vs T2D
hsa-miR-320a-3p_GAAAAGCTGGGTTGAGAGGGCGA	-0,733630834	3,456353792	0,161116905	0,441272073	T1D vs T2D
hsa-miR-320b_AAAAGCTGGGTTGAGAGGGCAA	-0,705966846	1,963551094	0,161806545	0,441643207	T1D vs T2D

hsa-miR-146a-5p_TGAGAACTGAATTCCATGGGTTG	-0,282812208	7,878355546	0,163972405	0,446027327	T1D vs T2D
hsa-miR-423-5p_TGAGGGGCAGAGAGCGAGACTT	0,128394724	12,19565674	0,164945278	0,44609999	T1D vs T2D
hsa-miR-885-5p_TCCATTACACTACCCTGCCTCT	-1,508858269	4,714772841	0,165118566	0,44609999	T1D vs T2D
hsa-miR-26b-5p_TCAAGTAATTCAGGATAGGTT	-0,738423763	3,747955651	0,166898834	0,449386386	T1D vs T2D
hsa-miR-425-5p_AATGACACGATCACTCCCGTTGAGT	-0,318142031	8,386717544	0,168303347	0,451642316	T1D vs T2D
hsa-miR-182-5p_TTTGGCAATGGTAGAACT	-0,770859121	3,421424723	0,170023518	0,452536473	T1D vs T2D
hsa-miR-30e-5p_GTAAACATCCTTGACTGGAAGC	-0,769849353	2,49990334	0,170250079	0,452536473	T1D vs T2D
hsa-miR-329-3p_AACACACCTGGTTAACCTCTT	-0,783548948	3,637419766	0,170339952	0,452536473	T1D vs T2D
hsa-miR-423-5p_GAGGGGCAGAGAGCGAGACTTTT	-0,668560723	4,029696332	0,171043434	0,452895737	T1D vs T2D
hsa-miR-338-5p_AACAATATCCTGGTGCTGAGT	-0,422658515	5,993094666	0,172522875	0,455163528	T1D vs T2D
hsa-miR-500a-3p_ATGCACCTGGGCAAGGATTCT	-0,667701828	4,513942296	0,173209349	0,455163528	T1D vs T2D
hsa-miR-484_TCAGGCTCAGTCCCCTCCCGA	-0,218295167	10,80053241	0,173885803	0,455163528	T1D vs T2D
hsa-miR-152-3p_TCAGTGCATGACAGAACTTGG	-0,693514543	2,073986305	0,174494356	0,455163528	T1D vs T2D
hsa-miR-27a-3p_TCACAGTGGCTAAGTTCCG	-0,750819988	2,668037972	0,174888856	0,455163528	T1D vs T2D
hsa-miR-19a-3p_TGTGCAAATCTATGCAAAACTGA	-0,729990825	2,326203362	0,175326478	0,455163528	T1D vs T2D
hsa-let-7a-5p_TTGAGGTAGTAGGTTGTATAGTT	-0,742770184	1,956823269	0,176346019	0,456323953	T1D vs T2D
hsa-miR-183-5p_TATGGCACTGGTAGAATT	-0,768883549	2,805102899	0,177365983	0,456751526	T1D vs T2D
hsa-miR-28-3p_ACTAGATTGTGAGCTCCTGGAG	-0,616629101	4,615150746	0,177657432	0,456751526	T1D vs T2D
hsa-miR-100-5p_AACCCGTAGATCCGAACTTG	-0,41001923	5,941970306	0,179524854	0,460068517	T1D vs T2D
hsa-miR-370-3p_GCCTGCTGGGGTGGAACCTGGT	-0,80312152	2,498088509	0,180143668	0,460084165	T1D vs T2D
hsa-miR-126-3p_TCGTACCGTGAGTAATAATGCG	-0,181054275	11,19647801	0,1806855	0,460084165	T1D vs T2D
hsa-miR-423-5p_TGAGGGGCAGAGAGCGAGA	0,150194848	9,723922848	0,182700606	0,463733705	T1D vs T2D
hsa-miR-532-5p_CATGCCTTGAGTGTAGGACCGT	-0,595999194	4,730944055	0,183821952	0,463976493	T1D vs T2D
hsa-miR-532-3p_CCTCCCACACCCAAGGCTTG	-0,750462668	2,745637111	0,183960567	0,463976493	T1D vs T2D
hsa-miR-22-3p_AAGCTGCCAGTTGAAGAACTGT	-0,111041021	12,01293	0,185221265	0,465682486	T1D vs T2D
hsa-let-7f-5p_TGAGGTAGTAGATTG	-0,688971298	4,164688749	0,186881992	0,468380338	T1D vs T2D
hsa-miR-23b-3p_ATCACATTGCCAGGGATTACCA	-0,380459119	6,460852955	0,188282297	0,468406761	T1D vs T2D
hsa-miR-23a-3p_ATCACATTGCCAGGGATTTC	0,186764255	9,459376505	0,188482881	0,468406761	T1D vs T2D
hsa-miR-224-5p_CAAGTCACTAGTGGTTCCGTTTAG	-0,711871267	4,65780231	0,188655672	0,468406761	T1D vs T2D
hsa-miR-3613-5p_TGTTGTACTTTTTTTTTTGTT	-0,501976801	5,269511804	0,194008178	0,479054575	T1D vs T2D
hsa-miR-19b-3p_TGTGCAAATCCATGCAAAACTGA	-0,602171126	4,916784309	0,194146333	0,479054575	T1D vs T2D
hsa-miR-222-3p_AGCTACATCTGGCTACTGGGTC	-0,653038052	3,362212617	0,196321774	0,479318618	T1D vs T2D
hsa-miR-99b-5p_CACCCGTAGAACCGACCTTG	-0,311188609	6,765454723	0,196381026	0,479318618	T1D vs T2D
hsa-miR-30e-5p_TGTAAACATCCTTGACTGGAAGCT	-0,093723647	11,72480372	0,196516048	0,479318618	T1D vs T2D
hsa-miR-221-3p_AGCTACATTGTCTGCTGGGTT	-0,402192363	6,683307876	0,19711335	0,479318618	T1D vs T2D
hsa-miR-143-3p_TGAGATGAAGCACTGTAGCTC	-0,195753584	9,842973082	0,19726036	0,479318618	T1D vs T2D
hsa-miR-25-3p_CATTGCACTTGTCTCGGTC	-0,443162346	5,77988375	0,199383572	0,482297396	T1D vs T2D
hsa-let-7d-3p_CTATACGACCTGCTGCCTT	-0,466417648	4,847124715	0,199696538	0,482297396	T1D vs T2D
hsa-miR-23a-5p_GGGGTTCCTGGGGATGGGATT	-0,679194733	3,585240207	0,201357316	0,484839218	T1D vs T2D
hsa-miR-27a-3p_TTCACAGTGGCTAAGTTCCGC	-0,350673054	6,28650114	0,202760468	0,486747269	T1D vs T2D
hsa-miR-125a-5p_CCCTGAGACCCTTTAACCTGT	-0,506769389	5,351640533	0,204393296	0,489193564	T1D vs T2D
hsa-miR-142-5p_CCCATAAAGTAGAAAGCACT	-0,121796005	11,13638127	0,205159158	0,489556434	T1D vs T2D
hsa-miR-574-3p_CACGCTCATGCACACACCCACA	-0,360481354	6,300877473	0,206320396	0,490203514	T1D vs T2D
hsa-miR-432-5p_TCTTGGAGTAGGTCATTGGGTGG	-0,769563708	3,502755168	0,206660453	0,490203514	T1D vs T2D
hsa-let-7b-3p_CTATACAACCTACTGCCTTCC	-0,346155954	6,023895535	0,207468208	0,490659234	T1D vs T2D
hsa-miR-7-5p_TGGAAGACTAGTGATTTTGTTGTT	-0,678676051	4,415695214	0,208532727	0,491685665	T1D vs T2D
hsa-miR-150-3p_CTGGTACAGGCCTGGGGGAC	-0,712018987	2,308717969	0,209995915	0,491685665	T1D vs T2D
hsa-miR-409-3p_GAATGTTGCTCGGTGAACCCCTT	-0,691508237	5,101558557	0,210157489	0,491685665	T1D vs T2D
hsa-miR-16-2-3p_CCAATATTACTGTGCTGCTTT	-0,558115363	4,35154905	0,210917048	0,491685665	T1D vs T2D
hsa-let-7i-5p_TGAGGTAGTAGTTTGTGCTGTTG	-0,643066461	3,894577335	0,211442904	0,491685665	T1D vs T2D
hsa-miR-409-3p_CGAATGTTGCTCGGTGAACCCCTTT	-0,769384647	2,598256943	0,211603743	0,491685665	T1D vs T2D
hsa-miR-3615_TCTCTCGGCTCCTCGCGGCT	0,33297263	6,584121427	0,215342161	0,49891774	T1D vs T2D
hsa-miR-589-5p_TGAGAACCACGTCTGCTCTGA	-0,673951846	2,896824842	0,218980138	0,50556696	T1D vs T2D
hsa-miR-221-3p_AGCTACATTGTCTGCTGGGTTTCA	-0,689580888	2,390031097	0,219480763	0,50556696	T1D vs T2D
hsa-let-7f-5p_GAGGTAGTAGATTGTATAGTT	-0,425303288	6,051011881	0,221272166	0,508224543	T1D vs T2D
hsa-miR-335-5p_TCAAGAGCAATAACGAAAAAT	0,368716678	6,715048961	0,222132112	0,508733601	T1D vs T2D
hsa-let-7b-5p_TGAGGTAGTAGGTTGTGTGG	0,128652695	12,43476497	0,223068455	0,509414209	T1D vs T2D
hsa-miR-103a-3p_AGCAGCATTGTACAGGGCTATG	-0,642175058	4,276844631	0,224195249	0,51052461	T1D vs T2D
hsa-miR-148b-3p_TCAGTGCATCACAGAACTTTG	-0,517049364	4,882892663	0,225955146	0,512038874	T1D vs T2D
hsa-miR-335-3p_TTTTTCATTATTGCTCCTGACC	-0,652729909	4,076732498	0,226145149	0,512038874	T1D vs T2D
hsa-let-7a-5p_TGAGGTAGTAGGTTGTATAGTTTT	-0,696096792	3,044731157	0,227138282	0,512830625	T1D vs T2D

hsa-let-7i-5p_TGAGGTAGTAGTTTGTGCT	-0,253458888	7,615350025	0,229275861	0,515356841	T1D vs T2D
hsa-miR-148a-3p_TCAGTGCACTACAGAACTTT	-0,639159442	4,280926656	0,229550412	0,515356841	T1D vs T2D
hsa-miR-185-5p_TGGAGAGAAAGGCAGTTCCTGA	-0,162889539	8,851967404	0,230477235	0,515984147	T1D vs T2D
hsa-miR-27b-3p_TTCACAGTGGCTAAGTTCTGC	-0,217345539	7,903620851	0,233177471	0,520567071	T1D vs T2D
hsa-miR-148a-3p_CAGTGCACTACAGAACTTTGT	-0,6738974	2,126116505	0,235266146	0,523762901	T1D vs T2D
hsa-miR-486-5p_GTACTGAGCTGCCCCGAG	-0,536044274	4,883397866	0,236342974	0,52469457	T1D vs T2D
hsa-miR-487b-3p_TCGTACAGGGTCATCCACTTT	-0,701801927	2,776617041	0,240273109	0,53059405	T1D vs T2D
hsa-miR-363-3p_ATTGCACGGTATCCATCTG	-0,643183347	2,060471037	0,240343636	0,53059405	T1D vs T2D
hsa-miR-361-3p_TCCCCCAGGTGTGATTCTGATTTG	-0,413100436	5,241633262	0,240997548	0,53059405	T1D vs T2D
hsa-miR-363-3p_AATTGCACGGTATCCATCTGTA	-0,5826148	3,912624597	0,242270821	0,531927947	T1D vs T2D
hsa-miR-15b-5p_TAGCAGCACATCATGGTTTACA	-0,349873073	6,32576652	0,243349263	0,531990706	T1D vs T2D
hsa-miR-15b-5p_TAGCAGCACATCATGGTTTAC	-0,233823205	7,368236859	0,244505225	0,531990706	T1D vs T2D
hsa-miR-4732-3p_GCCCTGACCTGTCCTGTTCTG	0,99710376	4,156168491	0,244803723	0,531990706	T1D vs T2D
hsa-miR-30e-5p_TGTAAACATCCTTGACTGG	-0,513898393	5,076073317	0,244969372	0,531990706	T1D vs T2D
hsa-let-7g-5p_TGAGGTAGTAGTTTGTACAGTTT	-0,299990242	6,811439564	0,246726225	0,534350003	T1D vs T2D
hsa-miR-486-5p_TGTACTGAGCTGCCCCGA	-0,560067506	4,617811312	0,248466144	0,535684095	T1D vs T2D
hsa-miR-26b-5p_TTCAAGTAATTCAGGATAGG	-0,530374089	3,436379997	0,248919271	0,535684095	T1D vs T2D
hsa-miR-181b-5p_AACATTCATTGCTGTCGGTG	-0,63305803	2,058689202	0,250065779	0,535684095	T1D vs T2D
hsa-miR-92a-3p_ATTGCACTTGTCCCGGCCTG	0,18353223	9,121941427	0,250110274	0,535684095	T1D vs T2D
hsa-miR-486-5p_ATCCTGTACTGAGCTGCCCCGA	0,195361099	8,988899635	0,250702845	0,535684095	T1D vs T2D
hsa-miR-133a-3p_TTGGTCCCCTTCAACCAGCTGT	0,685107607	2,527387136	0,253368883	0,539933155	T1D vs T2D
hsa-miR-423-3p_GCTCGGTCTGAGGCCCCTCAG	-0,633316473	1,954419998	0,255455102	0,542927243	T1D vs T2D
hsa-miR-223-3p_GTCAGTTTGTCAAATACCCCAA	-0,298131247	7,740128632	0,260386054	0,55006204	T1D vs T2D
hsa-miR-122-5p_TGGAGTGTGACAATGGTGTTTG	-0,303167037	11,63439763	0,260493318	0,55006204	T1D vs T2D
hsa-miR-30e-5p_TAAACATCCTTGACTGGAAGCT	-0,625623715	2,076536633	0,260882624	0,55006204	T1D vs T2D
hsa-miR-4732-5p_TGTAGAGCAGGGAGCAGGAAGCT	-0,347884274	6,276959061	0,263435757	0,553529069	T1D vs T2D
hsa-miR-3605-3p_CCTCCGTGTTACCTGTCCTCT	-0,480414052	5,168364179	0,263915993	0,553529069	T1D vs T2D
hsa-miR-10b-5p_ACCCTGTAGAACCGAATTTGTG	-0,348187605	5,829978228	0,264668849	0,553651109	T1D vs T2D
hsa-miR-146a-5p_TGAGAACTGAATTCCATGGG	-0,581752969	2,424854764	0,267670507	0,558464382	T1D vs T2D
hsa-miR-101-3p_GTACAGTACTGTGATAACT	-0,469954543	4,990449736	0,268533979	0,558803085	T1D vs T2D
hsa-miR-148a-3p_TCAGTGCACTACAGAACTTTG	-0,221187168	7,287277231	0,270389876	0,561199821	T1D vs T2D
hsa-miR-629-5p_TGGGTTTACGTTGGGAGAACT	0,254793632	7,034835335	0,272117547	0,561598096	T1D vs T2D
hsa-miR-3173-5p_TGCCCTGCCTGTTTTCTCCTT	0,609670103	2,97364428	0,272256233	0,561598096	T1D vs T2D
hsa-miR-3615_TCTCTCGGCTCCTCGCGGC	-0,591360635	2,019798482	0,272695688	0,561598096	T1D vs T2D
hsa-miR-424-3p_CAAAACGTGAGGCGCTGCT	-0,579418312	2,803316018	0,275918875	0,566771504	T1D vs T2D
hsa-miR-23a-3p_ATCACATTGCCAGGGATTTCCA	-0,149456446	11,15500363	0,279377931	0,570436168	T1D vs T2D
hsa-miR-342-3p_TCTCACACAGAAATCGCACCC	-0,595758857	2,049205595	0,279453903	0,570436168	T1D vs T2D
hsa-miR-126-3p_CGTACCGTGAGTAATAATGC	-0,458189712	4,46578231	0,280587812	0,570436168	T1D vs T2D
hsa-miR-155-5p_TTAATGCTAATCGTGATAGGGGT	-0,533421522	3,972069578	0,280954063	0,570436168	T1D vs T2D
hsa-miR-125b-5p_TCCCTGAGACCCTAACTT	-0,546701481	3,016083692	0,281281573	0,570436168	T1D vs T2D
hsa-miR-125b-5p_TCCCTGAGACCCTAACT	-0,64988425	2,233733889	0,282956802	0,572377085	T1D vs T2D
hsa-miR-486-3p_CGGGGCAGCTCAGTACAGGAT	-0,252454074	7,803889597	0,284267632	0,572688622	T1D vs T2D
hsa-miR-501-3p_ATGCACCCGGGCAAGGATTCT	-0,603442845	2,081940648	0,284726194	0,572688622	T1D vs T2D
hsa-miR-409-3p_AATGTTGCTCGGTGAACCCCT	-0,603939255	4,366315065	0,285900135	0,572688622	T1D vs T2D
hsa-let-7g-5p_GAGGTAGTAGTTTGTACAG	-0,601290256	2,076526463	0,286255192	0,572688622	T1D vs T2D
hsa-miR-122-5p_GAGTGTGACAATGGTGTTT	-0,645006298	2,929528654	0,286703589	0,572688622	T1D vs T2D
hsa-miR-99a-5p_AACCCGTAGATCCGATCTTGT	-0,175553969	8,529135227	0,288487376	0,574112094	T1D vs T2D
hsa-miR-181a-2-3p_ACCACTGACCGTTGACTGTACC	-0,60545593	2,397891497	0,28931816	0,574112094	T1D vs T2D
hsa-miR-1249-3p_ACGCCCTTCCCCCCCTTCTTCA	-0,64874814	2,195504284	0,289577242	0,574112094	T1D vs T2D
hsa-miR-192-5p_TGACCTATGAATTGACAGCCA	-0,496266445	4,726203979	0,291374891	0,57532782	T1D vs T2D
hsa-miR-361-5p_TTATCAGAATCTCCAGGGG	-0,567901523	2,056970291	0,291788723	0,57532782	T1D vs T2D
hsa-miR-379-5p_TGGTAGACTATGGAACGTAGG	-0,617019846	3,839701767	0,293443113	0,57532782	T1D vs T2D
hsa-miR-223-5p_CGTGTATTTGACAAGCTGAGTT	-0,492763699	3,93706767	0,293999418	0,57532782	T1D vs T2D
hsa-miR-150-5p_CTCCCAACCCTTGTACCAGT	-0,580975889	3,181356832	0,294260138	0,57532782	T1D vs T2D
hsa-let-7g-5p_TGAGGTAGTAGTTTGTACAG	-0,134629925	9,407543438	0,294521645	0,57532782	T1D vs T2D
hsa-miR-99b-5p_CACCCGTAGAACCGACCTT	-0,57570542	2,002435028	0,298242929	0,578255294	T1D vs T2D
hsa-miR-15b-3p_CGAATCATTATTTGCTGCTCT	0,456967936	4,350054835	0,299611139	0,578255294	T1D vs T2D
hsa-miR-193a-5p_TGGGTCTTTGCGGGCGAGA	0,600535889	3,488597754	0,300682376	0,578255294	T1D vs T2D
hsa-miR-224-5p_CAAGTCACTAGTGGTTCCGTTT	-0,573603644	2,885758309	0,301914606	0,578255294	T1D vs T2D
hsa-miR-150-5p_CTCCCAACCCTTGTACCAGTG	-0,492806677	4,313974888	0,302057286	0,578255294	T1D vs T2D
hsa-miR-10b-5p_TACCCTGTAGAACCGAAT	-0,580088103	2,220184724	0,302289287	0,578255294	T1D vs T2D

hsa-miR-652-3p_AATGGCGCCACTAGGGTTG	-0,541645075	4,499546053	0,302355826	0,578255294	T1D vs T2D
hsa-let-7d-3p_CTATACGACCTGCTGCCTTTCT	0,113208579	11,76128234	0,303171742	0,578255294	T1D vs T2D
hsa-miR-451a_CCGTTACCATTACTGAGT	-0,496119524	4,189894806	0,303492643	0,578255294	T1D vs T2D
hsa-miR-144-3p_CTACAGTATAGATGATGTAC	-0,552998581	3,24422031	0,304005588	0,578255294	T1D vs T2D
hsa-miR-17-5p_CAAAGTGCTTACAGTGCAGGTAG	-0,56103407	3,276961308	0,30515256	0,578255294	T1D vs T2D
hsa-let-7d-5p_AGAGGTAGTAGGTTGCATAGTTT	-0,479739332	5,420694347	0,305610825	0,578255294	T1D vs T2D
hsa-miR-342-3p_TCTCACACAGAAATCGCACCCGT	-0,193534037	8,706959622	0,306152075	0,578255294	T1D vs T2D
hsa-miR-342-3p_CTCACACAGAAATCGCACCCG	-0,585817882	2,495329338	0,306324555	0,578255294	T1D vs T2D
hsa-miR-182-5p_TTTGGCAATGGTAGAACTCA	-0,233337717	7,910992501	0,307207421	0,578255294	T1D vs T2D
hsa-miR-30a-5p_TGTAAACATCCTCGACTGGAAG	-0,179686448	8,337829601	0,307628914	0,578255294	T1D vs T2D
hsa-miR-186-5p_CAAAGAATTCTCCTTTTGGGCTTT	-0,527957869	4,003798957	0,308487719	0,578505205	T1D vs T2D
hsa-let-7a-5p_TGAGGTAGTAGGTTGTATAG	-0,107251852	12,05467063	0,310184381	0,578611718	T1D vs T2D
hsa-miR-375-3p_TTGTTCGTTCGGCTCGCGTGA	0,414958098	5,662970821	0,310812006	0,578611718	T1D vs T2D
hsa-miR-451a_AACCGTTACCATTACTGAGT	-0,155984686	10,87302044	0,311093758	0,578611718	T1D vs T2D
hsa-let-7d-3p_TATACGACCTGCTGCCTTTC	-0,250620432	6,429871038	0,312686951	0,578611718	T1D vs T2D
hsa-miR-126-3p_CTCGTACCGTGAGTAATAATGCG	-0,475015727	4,682348296	0,313202093	0,578611718	T1D vs T2D
hsa-miR-140-3p_ACCACAGGGTAGAACCACGGA	-0,280836155	6,646596672	0,313268805	0,578611718	T1D vs T2D
hsa-miR-126-5p_CATTATTACTTTTGGTACGC	0,515753292	3,620244945	0,313845878	0,578611718	T1D vs T2D
hsa-miR-221-3p_AGCTACATTGTCTGCTGGGT	-0,310140784	6,226471989	0,314352414	0,578611718	T1D vs T2D
hsa-miR-21-5p_TAGCTTATCAGACTGATGTTG	0,112600323	11,59436943	0,315129873	0,578706242	T1D vs T2D
hsa-miR-191-5p_CAACGGAATCCCAAAAGCAGCTGT	-0,437600125	5,688164696	0,316541785	0,579962765	T1D vs T2D
hsa-miR-223-3p_GTCAGTTTGTCAAATACCCCA	-0,298845419	6,454169749	0,317800056	0,58093267	T1D vs T2D
hsa-let-7c-5p_TGAGGTAGTAGGTTGTATGGTT	-0,194192634	7,467576739	0,318558849	0,580987192	T1D vs T2D
hsa-miR-2110_TTGGGGAAACGGCCGCTGAGTGA	-0,306775827	6,066188802	0,321472415	0,584429428	T1D vs T2D
hsa-miR-451a_AACCGTTACCATTACTGAGTTT	-0,540642442	2,400911204	0,321912822	0,584429428	T1D vs T2D
hsa-miR-29a-3p_CTAGCACCATCTGAAATCGGTT	-0,478721307	3,457099062	0,322775928	0,584664579	T1D vs T2D
hsa-let-7b-3p_CTATACAACCTACTGCCTTC	-0,521886106	3,159200497	0,323558292	0,584752741	T1D vs T2D
hsa-miR-23a-3p_ATCACATTGCCAGGGATTTCCAA	-0,318283043	6,530990344	0,324910699	0,585868388	T1D vs T2D
hsa-miR-27b-3p_TTCACAGTGGCTAAGTTCT	-0,176702981	8,299608545	0,326914367	0,588150678	T1D vs T2D
hsa-miR-26a-5p_TTCAAGTAATCCAGGATAGGC	-0,15743354	7,510198846	0,328605265	0,589861252	T1D vs T2D
hsa-miR-320a-3p_AAAGCTGGGTTGAGAGGGCGAA	-0,449131122	4,271030608	0,330381566	0,591717096	T1D vs T2D
hsa-miR-3173-5p_CCCTGCCTGTTTTCTCCTTTGT	-0,495007213	1,879782778	0,331928179	0,592775829	T1D vs T2D
hsa-miR-23a-3p_ATCACATTGCCAGGGATTT	0,166611683	7,810237088	0,33246022	0,592775829	T1D vs T2D
hsa-miR-425-5p_ATGACACGATCACTCCCGTTGAGT	0,438762563	4,72212581	0,334061115	0,593149811	T1D vs T2D
hsa-miR-92a-3p_ATTGCACTTGTCCCGGCCTGT	0,102422867	12,31240602	0,334158426	0,593149811	T1D vs T2D
hsa-let-7b-5p_TGAGGTAGTAGGTTGTGTGGT	-0,100579868	11,84645942	0,335102027	0,593168414	T1D vs T2D
hsa-miR-30c-5p_TGTAAACATCCTACACTCTCAGCT	-0,141905835	10,20131272	0,336422083	0,593168414	T1D vs T2D
hsa-miR-101-3p_TACAGTACTGTGATAACTGA	-0,481206292	4,333316093	0,336807778	0,593168414	T1D vs T2D
hsa-miR-29a-3p_TAGCACCATCTGAAATCGGTT	-0,166201984	8,641484144	0,338065579	0,593168414	T1D vs T2D
hsa-miR-192-5p_TGACCTATGAATTGACAGC	0,545571712	3,665629227	0,338229257	0,593168414	T1D vs T2D
hsa-miR-425-5p_AATGACACGATCACTCCCGTTGAG	-0,365186718	6,063293525	0,338634414	0,593168414	T1D vs T2D
hsa-miR-24-3p_GGCTCAGTTCAGCAGGAACAG	-0,217127481	6,812383366	0,341279604	0,595569376	T1D vs T2D
hsa-miR-23b-3p_ATCACATTGCCAGGGATTACCAC	-0,434912475	5,113281158	0,34149963	0,595569376	T1D vs T2D
hsa-miR-423-5p_AGGGGCAGAGAGCGAGACTTTT	-0,458168724	4,864713364	0,342311573	0,595681929	T1D vs T2D
hsa-miR-423-5p_AGGGGCAGAGAGCGAGACTTT	-0,329918966	5,332499304	0,34335453	0,596195121	T1D vs T2D
hsa-miR-451a_CGTTACCATTACTGAGT	-0,450291593	4,012413349	0,346160707	0,599423754	T1D vs T2D
hsa-miR-4446-3p_CAGGGCTGGCAGTGACATGGGT	-0,571155671	3,598813459	0,346913041	0,599423754	T1D vs T2D
hsa-miR-100-5p_AACCCGTAGATCCGAACTTGT	-0,228957947	7,211971456	0,347470231	0,599423754	T1D vs T2D
hsa-miR-144-5p_GATATCATCATATACTGTAAGTT	-0,530555857	2,459029553	0,349303092	0,601284156	T1D vs T2D
hsa-miR-223-3p_TGTCAGTTTGTCAAATACCCCA	-0,16389383	10,41019198	0,350819718	0,601533623	T1D vs T2D
hsa-miR-485-3p_GTCATACACGGCTCTCCTCTCT	-0,471940486	5,913980943	0,351187609	0,601533623	T1D vs T2D
hsa-miR-3615_TCTCTCGGCTCCTCGCGGCTCGC	-0,460645274	3,835837244	0,351712256	0,601533623	T1D vs T2D
hsa-miR-99a-5p_AACCCGTAGATCCGATCTT	0,519810481	3,048788798	0,353021274	0,601589493	T1D vs T2D
hsa-miR-92a-3p_TATTGCACTTGTCCCGGCCTG	0,127996464	13,95782504	0,353254558	0,601589493	T1D vs T2D
hsa-miR-574-3p_CACGCTCATGCACACACCCA	-0,494109219	3,365756558	0,355522328	0,604160544	T1D vs T2D
hsa-miR-186-5p_AAAGAATTCTCCTTTTGGGCT	-0,48337043	3,890936215	0,358169855	0,607364626	T1D vs T2D
hsa-miR-30d-5p_TGTAAACATCCCCGACTGGAAG	-0,072073573	12,16128446	0,35977703	0,608197579	T1D vs T2D
hsa-miR-99b-5p_CACCCGTAGAACCGACCTTGC	0,14070744	7,880557566	0,360187274	0,608197579	T1D vs T2D
hsa-miR-652-3p_AATGGCGCCACTAGGGTTGT	-0,142147781	7,904624885	0,362981209	0,610368696	T1D vs T2D
hsa-miR-92a-3p_GCACTTGTCCCGGCCTGT	-0,38238958	4,476222162	0,36300472	0,610368696	T1D vs T2D
hsa-miR-125a-5p_TCCCTGAGACCCTTTAACCTGT	-0,107351174	11,48945871	0,364616661	0,611597088	T1D vs T2D

hsa-miR-150-5p_GTCTCCCAACCCTTGTACCAGTG	-0,51098703	2,975164068	0,365354202	0,611597088	T1D vs T2D
hsa-miR-10b-5p_ACCCTGTAGAACCGAATTTGT	-0,195329479	6,785991828	0,366037404	0,611597088	T1D vs T2D
hsa-miR-342-5p_AGGGGTGCTATCTGTGATTGA	-0,515171567	3,958584465	0,368230163	0,612792981	T1D vs T2D
hsa-miR-16-2-3p_ACCAATATTACTGTGCTGCTTT	-0,139028135	7,879139867	0,368290888	0,612792981	T1D vs T2D
hsa-miR-10a-5p_TACCCTGTAGATCCGAATTT	-0,437550173	4,308439737	0,37106961	0,616130165	T1D vs T2D
hsa-miR-486-5p_CCTGTACTGAGCTGCCCCGA	0,133611685	11,08007536	0,372965124	0,616151263	T1D vs T2D
hsa-miR-128-3p_TCACAGTGAACCGGTCTCTT	-0,191419922	6,808413156	0,373716606	0,616151263	T1D vs T2D
hsa-let-7f-5p_TGAGGTAGTAGATTGTATAGTTG	-0,513089339	2,64125494	0,374019826	0,616151263	T1D vs T2D
hsa-miR-423-5p_TGAGGGGCAGAGAGCGAGACT	0,103066127	13,29479943	0,374174669	0,616151263	T1D vs T2D
hsa-miR-1301-3p_TTGCAGCTGCCTGGGAGTGACTTC	-0,51650775	2,697320641	0,375480654	0,616501852	T1D vs T2D
hsa-miR-191-5p_ACGGAATCCCAAAAGCAGCT	-0,490005702	1,904502114	0,37593463	0,616501852	T1D vs T2D
hsa-miR-30e-5p_TGTAAACATCCTTGACTGGA	-0,484662491	2,511168014	0,377583335	0,617620196	T1D vs T2D
hsa-miR-150-5p_TCTCCCAACCCTTGTACCAG	0,189914411	9,063580505	0,378166444	0,617620196	T1D vs T2D
hsa-miR-199a-5p_CCCAGTGTTCAGACTACCTGTTC	-0,418134031	4,567460238	0,380260834	0,619770725	T1D vs T2D
hsa-miR-16-5p_TAGCAGCACGTAAATATTGGCGT	-0,161661023	7,90073048	0,381087342	0,619850228	T1D vs T2D
hsa-miR-1306-5p_CCACCTCCCCTGCAAACGTCC	0,400531068	4,76371819	0,38271268	0,621226082	T1D vs T2D
hsa-miR-103a-3p_AGCAGCATTGTACAGGGCTAT	-0,584090326	3,428123511	0,387058183	0,625456456	T1D vs T2D
hsa-miR-361-3p_TCCCCCAGGTGTGATTCTGA	-0,447028435	3,113214743	0,387570935	0,625456456	T1D vs T2D
hsa-miR-125a-5p_TCCCTGAGACCCTTTAACCTGTGA	-0,174323014	8,237628665	0,38791414	0,625456456	T1D vs T2D
hsa-miR-181a-5p_AACATTCAACGCTGTCGGT	-0,311525572	5,505188114	0,388457899	0,625456456	T1D vs T2D
hsa-miR-145-3p_ATTCCTGGAAATACTGTTCTT	0,46947308	3,50539909	0,389770496	0,626304608	T1D vs T2D
hsa-miR-26b-5p_TTCAAGTAATTCAGGATAGGT	-0,167038085	7,343481575	0,391335817	0,62660775	T1D vs T2D
hsa-miR-345-5p_GCTGACTCCTAGTCCAGGGCTC	-0,480551206	3,656556535	0,391531568	0,62660775	T1D vs T2D
hsa-let-7a-5p_GTGAGGTAGTAGGTTGTATAGTT	-0,505691712	2,866169762	0,396764237	0,631636665	T1D vs T2D
hsa-miR-32-5p_TATTGCACATTACTAAGTTGC	-0,466936916	2,00688677	0,397049424	0,631636665	T1D vs T2D
hsa-miR-20a-5p_TAAAGTGCTTATAGTGCAGGTAG	-0,382948389	4,004629847	0,397051404	0,631636665	T1D vs T2D
hsa-miR-486-5p_TCCTGTACTGAGCTGCCC	-0,313778582	3,88539215	0,40015744	0,63530972	T1D vs T2D
hsa-miR-10b-5p_TACCCTGTAGAACCGAATTTGTG	-0,169006823	7,78613959	0,403615869	0,639526535	T1D vs T2D
hsa-miR-15b-5p_TAGCAGCACATCATGGTTTA	-0,275105577	5,794504936	0,406054632	0,641234207	T1D vs T2D
hsa-miR-92a-3p_TATTGCACTTGTCCCGGCCTGT	0,078545278	17,23184948	0,406302728	0,641234207	T1D vs T2D
hsa-miR-150-5p_TCTCCCAACCCTTGTACCA	-0,275212377	6,588601483	0,408825145	0,643940001	T1D vs T2D
hsa-miR-375-3p_TTTGTTCGTTCGGCTCGCGT	-0,451586833	3,894636907	0,412262496	0,648073391	T1D vs T2D
hsa-miR-92a-3p_ATTGCACTTGTCCCGGCCT	-0,395768225	4,477303001	0,413954423	0,649452117	T1D vs T2D
hsa-miR-486-5p_TCCTGTACTGAGCTGCCCCGA	0,112552349	17,03890679	0,415517619	0,650623856	T1D vs T2D
hsa-miR-122-5p_TGGAGTGTGACAATGGTGTTT	-0,21054483	13,55430102	0,420041561	0,655398551	T1D vs T2D
hsa-miR-374b-5p_ATATAATACAACCTGCTAAGT	-0,398759434	3,95574137	0,420636611	0,655398551	T1D vs T2D
hsa-miR-191-5p_CAACGGAATCCCAAAAGC	-0,434601018	3,190312182	0,422520599	0,655398551	T1D vs T2D
hsa-miR-1908-5p_CGGCGGGGACGGCGATTGGT	-0,477401593	2,686402078	0,422548789	0,655398551	T1D vs T2D
hsa-let-7c-5p_TGAGGTAGTAGGTTGTATGGTTT	-0,397139282	3,566946574	0,422678614	0,655398551	T1D vs T2D
hsa-miR-339-3p_TGAGCGCCTCGACGACAGAG	-0,323240785	5,154516456	0,426136448	0,657278107	T1D vs T2D
hsa-miR-182-5p_TTTGGCAATGGTAGAACTCACACTGG	-0,461227163	2,315372824	0,426158056	0,657278107	T1D vs T2D
hsa-miR-363-3p_AATTGCACGGTATCCATCT	-0,381780866	4,478964619	0,427178249	0,657278107	T1D vs T2D
hsa-let-7i-5p_TGAGGTAGTAGTTTGTGCTG	-0,116653123	10,07708045	0,427670517	0,657278107	T1D vs T2D
hsa-miR-409-3p_GAATGTTGCTCGGTGAACCCCT	-0,277363309	7,973406619	0,428014225	0,657278107	T1D vs T2D
hsa-miR-223-3p_TCAGTTTGTCAAATACCCCAA	-0,418585531	2,060154898	0,434263332	0,664656858	T1D vs T2D
hsa-miR-24-3p_TGGCTCAGTTCAGCAGGAA	-0,302767622	5,648746767	0,434813027	0,664656858	T1D vs T2D
hsa-miR-451a_AAACCGTTACCATTACTGAGT	-0,113080495	16,51409079	0,436969191	0,664656858	T1D vs T2D
hsa-miR-629-5p_TGGGTTTACGTTGGGAGAAC	0,421047528	3,68380122	0,436987778	0,664656858	T1D vs T2D
hsa-miR-142-3p_TGTAGTGTTTCCTACTTTATGGA	0,427072687	3,252758122	0,437260597	0,664656858	T1D vs T2D
hsa-miR-30e-5p_TGTAAACATCCTTGACTGGAAGC	-0,091677656	8,385302075	0,437822899	0,664656858	T1D vs T2D
hsa-miR-181a-5p_AACATTCAACGCTGTCGGTGA	-0,08012419	9,980124889	0,439481071	0,66590573	T1D vs T2D
hsa-miR-26a-5p_TTCAAGTAATCCAGGATAGG	-0,162629661	6,813190078	0,441650936	0,667923712	T1D vs T2D
hsa-miR-486-3p_CGGGGCAGCTCAGTACAGGA	-0,228667213	6,539637794	0,444092309	0,670343884	T1D vs T2D
hsa-miR-4732-5p_TGTAGAGCAGGGAGCAGGAAG	-0,428691119	3,155376397	0,447250924	0,67128228	T1D vs T2D
hsa-let-7d-5p_AGAGGTAGTAGGTTGCATAGT	-0,179032617	7,455921597	0,447587308	0,67128228	T1D vs T2D
hsa-miR-339-3p_TGAGCGCCTCGACGACAGAGC	-0,376076903	3,99309945	0,447890864	0,67128228	T1D vs T2D
hsa-miR-483-3p_TCACTCCTCTCCTCCCGTCT	0,448760498	2,217026305	0,448876259	0,67128228	T1D vs T2D
hsa-miR-24-3p_TGGCTCAGTTCAGCAGGAACA	-0,083060692	9,988806411	0,448925289	0,67128228	T1D vs T2D
hsa-miR-409-3p_CGAATGTTGCTCGGTGAACCCCTT	-0,453333344	3,646109852	0,452547699	0,672642061	T1D vs T2D
hsa-miR-92a-3p_TATTGCACTTGTCCCGGCCT	0,109984965	10,27945719	0,453619749	0,672642061	T1D vs T2D
hsa-miR-1307-3p_CTCGGCGTGGCGTCGGTCGTGG	-0,430759714	2,190246459	0,453806367	0,672642061	T1D vs T2D

hsa-miR-486-5p_TCCTGTACTGAGCTGCCCCG	0,118136002	14,12822837	0,45388476	0,672642061	T1D vs T2D
hsa-miR-28-5p_AAGGAGCTCACAGTCTATTGA	-0,446771382	3,11393162	0,45405449	0,672642061	T1D vs T2D
hsa-miR-21-5p_GTAGCTTATCAGACTGATGTTGA	-0,420879137	2,217922154	0,455362744	0,673328584	T1D vs T2D
hsa-miR-144-3p_TACAGTATAGATGATGTACT	-0,344287592	4,501383498	0,457267374	0,674132586	T1D vs T2D
hsa-miR-126-3p_GTACCGTGAGTAATAATGCG	-0,402636556	2,067690175	0,457598155	0,674132586	T1D vs T2D
hsa-miR-183-5p_TATGGCACTGGTAGAATTCA	-0,206142494	7,32183424	0,458588781	0,674345495	T1D vs T2D
hsa-miR-144-3p_TACAGTATAGATGATGTAC	-0,198017745	6,460339014	0,460150865	0,674636636	T1D vs T2D
hsa-miR-92a-3p_TATTGCACTTGTCCCGGC	-0,191093647	6,608463448	0,460976367	0,674636636	T1D vs T2D
hsa-miR-32-5p_TATTGCACATTACTAAGTTG	-0,38037851	3,984130302	0,461326182	0,674636636	T1D vs T2D
hsa-miR-30e-3p_CTTTCAGTCGGATGTTTACAG	-0,400177489	2,31966353	0,4637273	0,676905967	T1D vs T2D
hsa-miR-4433b-5p_TGTCCCACCCCCACTCCTGTT	0,318429955	6,729375179	0,467309896	0,680162123	T1D vs T2D
hsa-miR-181a-5p_AACATTCAACGCTGTCGGTG	-0,177617377	6,983532365	0,467664798	0,680162123	T1D vs T2D
hsa-miR-126-3p_CGTACCGTGAGTAATAATGCG	-0,102809045	9,683358758	0,472548039	0,686012363	T1D vs T2D
hsa-miR-192-5p_TGACCTATGAATTGACAGCC	-0,202143222	6,449264096	0,474959213	0,688259077	T1D vs T2D
hsa-miR-192-5p_CTGACCTATGAATTGACAGC	-0,399872767	1,977431527	0,47667561	0,689330394	T1D vs T2D
hsa-miR-223-5p_CGTGTATTTGACAAGCTGAGTTGG	-0,402120402	2,566442122	0,478496824	0,689330394	T1D vs T2D
hsa-miR-223-3p_TGTCAGTTTGTCAAATACCCC	-0,199817298	7,314719746	0,479021244	0,689330394	T1D vs T2D
hsa-miR-101-3p_TACAGTACTGTGATAACTGAAG	-0,159515197	7,012156412	0,479158141	0,689330394	T1D vs T2D
hsa-let-7f-5p_GAGGTAGTAGATTGTATAGT	-0,408426692	3,023408308	0,480463159	0,68996241	T1D vs T2D
hsa-miR-30d-5p_GTAAACATCCCCGACTGGAAGC	-0,301948335	4,874885211	0,482536323	0,69127085	T1D vs T2D
hsa-miR-92b-3p_TATTGCACTCGTCCCGGCCTC	0,338154035	3,886574178	0,483108988	0,69127085	T1D vs T2D
hsa-miR-4433b-5p_TATGTCCCACCCCCACTCCTGT	-0,332650055	5,397905397	0,484014418	0,691325253	T1D vs T2D
hsa-miR-16-5p_TAGCAGCACGTAAATATTGG	-0,091051084	10,97240393	0,489338707	0,696408982	T1D vs T2D
hsa-miR-182-5p_TTTGGCAATGGTAGAACTCACACT	0,155505024	7,651119737	0,490004762	0,696408982	T1D vs T2D
hsa-miR-4433b-5p_TATGTCCCACCCCCACTCCTG	0,432470953	3,068037559	0,49019503	0,696408982	T1D vs T2D
hsa-miR-125b-5p_CCCTGAGACCCTAACTTGT	-0,37833023	2,290772166	0,49308843	0,699273093	T1D vs T2D
hsa-miR-382-5p_AAGTTGTTCGTGGTGGATTCG	-0,408340518	2,227150711	0,494735561	0,700362775	T1D vs T2D
hsa-miR-23a-5p_GGGGTTCCTGGGGATGGGATTT	-0,352342598	3,81817711	0,496150075	0,701119875	T1D vs T2D
hsa-let-7f-5p_TGAGGTAGTAGATTGTATAG	-0,079052976	10,59314063	0,498159597	0,702713626	T1D vs T2D
hsa-miR-486-5p_ATCCTGTACTGAGCTGCCCCGAG	-0,131417663	7,990918242	0,499739151	0,703696296	T1D vs T2D
hsa-miR-26b-5p_TTCAAGTAATTCAGGATAGGTT	-0,113415924	9,842825606	0,514604556	0,722114555	T1D vs T2D
hsa-miR-4433b-5p_ATGTCCCACCCCCACTCCTGTT	0,241596252	8,226543503	0,514631201	0,722114555	T1D vs T2D
hsa-miR-328-3p_CTGGCCCTCTCTGCCCTTCCG	-0,309570739	3,770890003	0,516961996	0,72340122	T1D vs T2D
hsa-miR-22-3p_AAGCTGCCAGTTGAAGAACTGTT	0,349850152	2,793276847	0,517363483	0,72340122	T1D vs T2D
hsa-miR-23a-3p_TCACATTGCCAGGGATTTCC	0,307459313	4,540513631	0,518381441	0,723555181	T1D vs T2D
hsa-miR-486-5p_ATCCTGTACTGAGCTGCCCCG	0,175965631	6,350028468	0,523927529	0,730017903	T1D vs T2D
hsa-miR-99b-5p_ACCCGTAGAACCGACCTTGCG	-0,343935649	2,082938158	0,52871196	0,734234137	T1D vs T2D
hsa-miR-125a-5p_TCCCTGAGACCCTTTAACC	-0,333416683	2,158659604	0,528901774	0,734234137	T1D vs T2D
hsa-miR-98-5p_TGAGGTAGTAAGTTGTATTGT	0,199052647	6,55627531	0,529717226	0,734234137	T1D vs T2D
hsa-miR-22-3p_AAGCTGCCAGTTGAAGAACT	-0,074621073	10,21399499	0,531339661	0,735204357	T1D vs T2D
hsa-miR-30c-5p_TGTAAACATCCTACACTCTCAG	-0,267938191	4,767658059	0,535732479	0,739443808	T1D vs T2D
hsa-miR-93-5p_CAAAGTGCTGTTCGTGCAGGTAGT	-0,321654752	4,129822813	0,536838957	0,739443808	T1D vs T2D
hsa-miR-24-3p_GGCTCAGTTCAGCAGGAAC	0,372696697	2,859699768	0,537563414	0,739443808	T1D vs T2D
hsa-miR-28-5p_AAGGAGCTCACAGTCTATTGAG	-0,348243288	3,384277878	0,538114691	0,739443808	T1D vs T2D
hsa-miR-323a-3p_CACATTACACGGTCGACCTCT	-0,344752283	3,585538292	0,542108475	0,743649664	T1D vs T2D
hsa-miR-191-5p_CAACGGAATCCCAAAAGCAG	-0,065800932	10,17632421	0,545149048	0,745069959	T1D vs T2D
hsa-miR-409-3p_CGAATGTTGCTCGGTGAACCCCT	-0,302863868	5,738161577	0,545924584	0,745069959	T1D vs T2D
hsa-miR-222-3p_AGCTACATCTGGCTACTGGGTCTCT	-0,150892121	7,967322162	0,546215888	0,745069959	T1D vs T2D
hsa-miR-6803-3p_TCCCTCGCCTTCTCACCCTCAG	-0,31920854	2,710817974	0,54688322	0,745069959	T1D vs T2D
hsa-miR-144-5p_GGATATCATCATATACTGTAAGT	0,167083234	6,405140764	0,548934184	0,746003117	T1D vs T2D
hsa-miR-423-5p_GAGGGGCAGAGAGCGAGACT	-0,17860473	6,874414686	0,549440187	0,746003117	T1D vs T2D
hsa-miR-342-3p_TCACACAGAAATCGCACCCGTC	-0,324150754	2,309823181	0,553544407	0,750297435	T1D vs T2D
hsa-miR-375-3p_TTTGTTCGTTCGGCTCGCGTG	0,196701215	6,919662337	0,556270036	0,751936003	T1D vs T2D
hsa-miR-345-5p_GCTGACTCCTAGTCCAGGGCT	-0,24309061	5,057881275	0,556640203	0,751936003	T1D vs T2D
hsa-miR-24-3p_TGGCTCAGTTCAGCAGGAAC	0,065934822	10,07194833	0,56320243	0,758963514	T1D vs T2D
hsa-miR-10a-5p_TACCCTGTAGATCCGAATTTG	0,084839278	8,855203262	0,563747052	0,758963514	T1D vs T2D
hsa-let-7d-5p_AGAGGTAGTAGGTTGCATAG	-0,105534129	8,573362788	0,564855945	0,75917401	T1D vs T2D
hsa-miR-4433b-5p_ATGTCCCACCCCCACTCCTGT	0,205572126	7,752662063	0,56682186	0,760533708	T1D vs T2D
hsa-miR-125b-5p_TCCCTGAGACCCTAACTTG	0,093503643	8,171400371	0,569846194	0,763306582	T1D vs T2D
hsa-miR-199a-3p_ACAGTAGTCTGCACATTGGT	0,321046511	1,952185074	0,571582257	0,763749027	T1D vs T2D
hsa-miR-660-5p_TACCCATTGCATATCGGAGTTGT	-0,287376752	3,90808508	0,572093061	0,763749027	T1D vs T2D

hsa-miR-342-3p_TCTCACACAGAAATCGCACCCG	0,114498137	9,658047597	0,574852617	0,766149725	T1D vs T2D
hsa-let-7f-5p_TGAGGTAGTAGATTGTATAGTTT	-0,139950741	8,114676232	0,579744497	0,768268289	T1D vs T2D
hsa-miR-128-3p_TCACAGTGAACCGGTCTCT	-0,099382381	7,683472613	0,579812406	0,768268289	T1D vs T2D
hsa-miR-191-5p_AACGGAATCCCAAAAGCAGCT	-0,140023354	6,816281646	0,579993101	0,768268289	T1D vs T2D
hsa-miR-193a-5p_TGGGTCTTTGCGGGCGAGATG	-0,209822433	5,884704134	0,580791106	0,768268289	T1D vs T2D
hsa-miR-654-5p_TGGTGGGCCGCAGAACATGTGC	0,347940279	3,027916369	0,581261955	0,768268289	T1D vs T2D
hsa-miR-433-3p_ATCATGATGGGCTCCTCGGTGT	0,314717952	4,303379714	0,582372169	0,768461289	T1D vs T2D
hsa-miR-130b-5p_ACTCTTTCCCTGTTGCACTACT	-0,190902224	5,742085569	0,586926373	0,773190611	T1D vs T2D
hsa-miR-144-5p_GGATATCATCATATACTGTAAG	-0,303387742	4,224705339	0,58959722	0,775427366	T1D vs T2D
hsa-miR-182-5p_TTTGGCAATGGTAGAACTCACA	-0,143831884	6,943076472	0,595669402	0,782122757	T1D vs T2D
hsa-miR-199a-3p_ACAGTAGTCTGCACATTGGTTA	-0,443844985	4,827856003	0,596840301	0,78237125	T1D vs T2D
hsa-miR-486-5p_TCCTGTACTGAGCTGCCCC	0,112174505	6,793601467	0,599648585	0,784761777	T1D vs T2D
hsa-miR-320a-3p_AAAGCTGGGTTGAGAGGGCGA	-0,248191117	4,98279454	0,601825745	0,786151001	T1D vs T2D
hsa-miR-103a-3p_AGCAGCATTGTACAGGGC	0,345432252	3,081760646	0,602682888	0,786151001	T1D vs T2D
hsa-miR-654-3p_TATGTCTGCTGACCATCACC	-0,307210405	2,711988254	0,604452239	0,787170644	T1D vs T2D
hsa-miR-369-3p_AATAATACATGGTTGATCTTT	-0,285756635	4,326255371	0,606842411	0,787725845	T1D vs T2D
hsa-let-7i-5p_TGAGGTAGTAGTTTGTGCTGTT	-0,079474822	10,48466024	0,606855293	0,787725845	T1D vs T2D
hsa-miR-423-5p_AGGGGCAGAGAGCGAGACT	-0,284204032	3,04923014	0,612516757	0,792715191	T1D vs T2D
hsa-miR-10a-5p_ACCCTGTAGATCCGAATTTG	-0,157784233	5,718851669	0,613618046	0,792715191	T1D vs T2D
hsa-miR-486-5p_CTGTACTGAGCTGCCCCGAG	0,102202521	7,787642307	0,614193329	0,792715191	T1D vs T2D
hsa-miR-98-5p_TGAGGTAGTAAGTTGTATTG	-0,236743875	4,611342852	0,614677526	0,792715191	T1D vs T2D
hsa-miR-486-5p_CTGTACTGAGCTGCCCCGA	-0,096868888	7,583597035	0,617728561	0,795362946	T1D vs T2D
hsa-miR-18a-3p_ACTGCCCTAAGTGCTCCTTCT	-0,27444521	3,273299722	0,619705109	0,796620923	T1D vs T2D
hsa-miR-340-5p_TTATAAAGCAATGAGACTGAT	0,272190666	1,968507467	0,622212873	0,798556618	T1D vs T2D
hsa-miR-3158-3p_AAGGGCTTCCTCTCTGCAGGA	-0,284948125	2,734692583	0,623273132	0,798631328	T1D vs T2D
hsa-miR-320a-3p_AAAAGCTGGGTTGAGAGGGCGA	0,050919263	10,74836293	0,62855635	0,804108204	T1D vs T2D
hsa-miR-329-3p_ACACACCTGGTTAACCTCTTTT	-0,280563034	2,079797897	0,630625802	0,805462764	T1D vs T2D
hsa-miR-10b-5p_TACCCTGTAGAACCGAATT	0,226421162	4,675674869	0,633551304	0,807068689	T1D vs T2D
hsa-let-7d-3p_TATACGACCTGCTGCCTTT	-0,122628311	6,275229414	0,633908406	0,807068689	T1D vs T2D
hsa-miR-92a-3p_TGCACTTGTCCCGGCCTGT	-0,173857858	5,28105363	0,63706937	0,80907404	T1D vs T2D
hsa-miR-16-5p_TAGCAGCACGTAAATATTGGCG	-0,051463672	14,03055319	0,638420708	0,80907404	T1D vs T2D
hsa-miR-126-3p_TCGTACCGTGAGTAATAATG	-0,142826931	5,867396204	0,638632334	0,80907404	T1D vs T2D
hsa-miR-501-3p_AATGCACCCGGGCAAGGAT	-0,255167927	2,760223516	0,641973242	0,80907404	T1D vs T2D
hsa-miR-222-3p_AGCTACATCTGGCTACTGGG	-0,260537487	2,549442498	0,642162845	0,80907404	T1D vs T2D
hsa-miR-29a-3p_TAGCACCATCTGAAATCGG	-0,111462669	7,213630267	0,642264544	0,80907404	T1D vs T2D
hsa-miR-486-5p_CCTGTACTGAGCTGCCCCGAG	0,068944462	11,29662351	0,643188251	0,80907404	T1D vs T2D
hsa-miR-145-5p_GTCCAGTTTTCCCAGGAATCCCT	-0,265085212	2,146327793	0,644333418	0,80907404	T1D vs T2D
hsa-miR-320a-3p_GAAAAGCTGGGTTGAGAGGGCG	0,144359988	6,114738168	0,644619844	0,80907404	T1D vs T2D
hsa-miR-150-5p_TCTCCCAACCCTTGTACCAGTG	0,068777266	10,93881631	0,647515078	0,809616593	T1D vs T2D
hsa-miR-4433b-5p_TGTCCCACCCCCACTCCTGTTT	0,146904013	8,596146134	0,648350491	0,809616593	T1D vs T2D
hsa-miR-26a-5p_TTCAAGTAATCCAGGATAGGCT	-0,07509324	11,92893731	0,648563568	0,809616593	T1D vs T2D
hsa-miR-342-3p_TCTCACACAGAAATCGCACCCGTCA	-0,248608021	3,422364811	0,649115437	0,809616593	T1D vs T2D
hsa-let-7c-5p_TGAGGTAGTAGGTTGTATGGT	0,16307665	4,585920454	0,652584017	0,812671034	T1D vs T2D
hsa-miR-664a-3p_TATTCATTTATCCCCAGCCTACA	0,238763024	3,022366025	0,653621368	0,812693027	T1D vs T2D
hsa-miR-140-3p_TACCACAGGGTAGAACCACGGAC	-0,236857234	3,947173881	0,656803576	0,81446103	T1D vs T2D
hsa-miR-3173-5p_TGCCCTGCCTGTTTTCTCCTTT	-0,186909647	4,766192857	0,65708713	0,81446103	T1D vs T2D
hsa-miR-193a-5p_TGGGTCTTTGCGGGCGAGATGA	-0,073863763	8,630407946	0,659281745	0,815912346	T1D vs T2D
hsa-miR-92a-3p_TATTGCACTTGTCCCGGCCTGTT	0,052989193	10,41769689	0,661672322	0,816909821	T1D vs T2D
hsa-miR-543_AAACATTCGCGGTGCACTTCTTT	-0,268080635	3,173881718	0,662137697	0,816909821	T1D vs T2D
hsa-miR-6803-3p_TCCCTCGCCTTCTCACCCTC	0,232001459	2,004774634	0,668858289	0,822488612	T1D vs T2D
hsa-miR-451a_AACCGTTACCATTACTGAG	-0,07866439	8,074719646	0,669263456	0,822488612	T1D vs T2D
hsa-miR-421_ATCAACAGACATTAATTGGGCGC	-0,222797484	2,04052248	0,670207535	0,822488612	T1D vs T2D
hsa-let-7a-5p_TGAGGTAGTAGGTTGTATAGTTT	-0,089604797	9,423297293	0,67078745	0,822488612	T1D vs T2D
hsa-miR-197-3p_TTCACCACCTTCTCCACCCA	0,207542421	3,958344822	0,674228531	0,824241566	T1D vs T2D
hsa-miR-128-3p_TCACAGTGAACCGGTCTCTTT	0,055251237	8,228392837	0,675445037	0,824241566	T1D vs T2D
hsa-miR-106b-5p_TAAAGTGCTGACAGTGCAGA	-0,162492851	5,144709005	0,676976802	0,824241566	T1D vs T2D
hsa-let-7g-5p_TGAGGTAGTAGTTTGTACAGTT	-0,053933255	11,23391478	0,677203125	0,824241566	T1D vs T2D
hsa-miR-92a-3p_TATTGCACTTGTCCCGGCC	-0,083762559	8,088512139	0,677387987	0,824241566	T1D vs T2D
hsa-miR-361-5p_TTATCAGAATCTCCAGGGGTACT	0,222516182	2,611707431	0,681272067	0,827704021	T1D vs T2D
hsa-miR-140-3p_ACCACAGGGTAGAACCACG	-0,22087513	2,665108795	0,683555072	0,829213687	T1D vs T2D
hsa-miR-450b-5p_TTTTGCAATATGTTCCTGAAT	-0,174587087	5,016886951	0,686389241	0,830573664	T1D vs T2D

hsa-miR-425-5p_AATGACACGATCACTCCCGT	-0,098919275	6,738341736	0,687555943	0,830573664	T1D vs T2D
hsa-miR-1908-5p_CGGCGGGGACGGCGATTGGTC	-0,228804439	2,612171571	0,687802532	0,830573664	T1D vs T2D
hsa-miR-221-3p_AGCTACATTGTCTGCTGGGTTT	-0,070740586	7,916859704	0,691349456	0,833593822	T1D vs T2D
hsa-miR-21-5p_AGCTTATCAGACTGATGTTG	-0,146805552	5,031654467	0,694505501	0,835382508	T1D vs T2D
hsa-miR-4433b-5p_ATGTCCCACCCCCACTCCTG	-0,212354074	2,006165102	0,695258169	0,835382508	T1D vs T2D
hsa-miR-30e-5p_TGTAAACATCCTTGACTGGAAG	-0,067560713	7,619075745	0,695977397	0,835382508	T1D vs T2D
hsa-miR-423-5p_TGAGGGGCAGAGAGCGAGAC	0,038195429	9,751921415	0,702235355	0,838020525	T1D vs T2D
hsa-miR-340-5p_TTATAAAGCAATGAGACTGATT	-0,135789947	5,815012818	0,702427608	0,838020525	T1D vs T2D
hsa-miR-144-5p_GGATATCATCATATACTGTAA	-0,207907868	3,901874288	0,702624964	0,838020525	T1D vs T2D
hsa-miR-320a-3p_AAAGCTGGGTTGAGAGGGCG	-0,162001596	4,890613401	0,703407709	0,838020525	T1D vs T2D
hsa-miR-142-5p_CATAAAGTAGAAAGCACTACT	-0,190263131	3,650597591	0,703492764	0,838020525	T1D vs T2D
hsa-miR-423-3p_AAGCTCGGTCTGAGGCCCCT	-0,214968181	2,46496229	0,704484004	0,838020525	T1D vs T2D
hsa-miR-320a-3p_AAAAGCTGGGTTGAGAGGGCGAAA	-0,103973601	6,744969015	0,707082871	0,839858492	T1D vs T2D
hsa-miR-1301-3p_TTGCAGCTGCCTGGGAGTGA	-0,201470108	3,347749467	0,711778351	0,844177598	T1D vs T2D
hsa-miR-23a-3p_TCACATTGCCAGGGATTTC	-0,209545842	2,403793771	0,712939346	0,844298156	T1D vs T2D
hsa-let-7d-3p_ATACGACCTGCTGCCTTTCT	-0,197357193	2,048536833	0,71841826	0,848689216	T1D vs T2D
hsa-miR-29c-3p_TAGCACCATTTGAAATCGGT	-0,184364579	4,339459305	0,719441174	0,848689216	T1D vs T2D
hsa-miR-652-3p_AATGGCGCCACTAGGGTTGTGC	-0,179338673	3,594273542	0,721177764	0,848689216	T1D vs T2D
hsa-miR-30e-5p_GTAAACATCCTTGACTGGAAGCT	-0,038467804	8,781643893	0,721331568	0,848689216	T1D vs T2D
hsa-let-7i-5p_TGAGGTAGTAGTTTGTGCTGT	0,039496648	11,39282357	0,721971504	0,848689216	T1D vs T2D
hsa-miR-193b-5p_CGGGGTTTTGAGGGCGAGATGA	-0,205477607	2,282241357	0,726198405	0,852400778	T1D vs T2D
hsa-miR-424-3p_CAAAACGTGAGGCGCTGCTAT	-0,114973626	5,519904886	0,730614657	0,856323355	T1D vs T2D
hsa-miR-342-3p_TCACACAGAAATCGCACCCGT	-0,18212949	2,35866661	0,735270174	0,860514433	T1D vs T2D
hsa-let-7a-5p_AGGTAGTAGGTTGTATAGTT	-0,187353016	1,879378714	0,737203813	0,861512373	T1D vs T2D
hsa-miR-382-5p_GAAGTTGTTCGTGGTGGATTCG	-0,111409629	7,607601722	0,743967367	0,868143472	T1D vs T2D
hsa-let-7a-5p_TGAGGTAGTAGGTTGTAT	-0,215845676	3,216663859	0,747052175	0,8698122	T1D vs T2D
hsa-miR-485-5p_AGAGGCTGGCCGTGATGAATTC	-0,19374419	2,300221452	0,747580122	0,8698122	T1D vs T2D
hsa-miR-21-5p_TAGCTTATCAGACTGATGTT	-0,062181517	7,170728426	0,752375658	0,873719071	T1D vs T2D
hsa-miR-3615_TCTCTCGGCTCCTCGCGGCTC	-0,158782625	2,193732344	0,753130492	0,873719071	T1D vs T2D
hsa-miR-4433b-5p_TGTCCCACCCCCACTCCTGT	-0,146976477	4,771975689	0,761878296	0,880907694	T1D vs T2D
hsa-miR-150-5p_TCTCCCAACCCTTGTACCAGT	0,053227668	10,25222562	0,763446596	0,880907694	T1D vs T2D
hsa-let-7b-5p_GAGGTAGTAGGTTGTGTGG	0,095924293	6,125260792	0,76348976	0,880907694	T1D vs T2D
hsa-miR-423-3p_AGCTCGGTCTGAGGCCCC	-0,156678514	3,540860079	0,763748076	0,880907694	T1D vs T2D
hsa-miR-483-5p_AAGACGGGAGGAAAGAAGGGAGT	0,17133944	3,821071217	0,768290128	0,882429594	T1D vs T2D
hsa-miR-16-5p_AGCAGCACGTAAATATTGGC	-0,155234281	2,995434135	0,768682703	0,882429594	T1D vs T2D
hsa-miR-323b-3p_CCCAATACACGGTCGACCTCT	-0,140663057	5,853793118	0,76930195	0,882429594	T1D vs T2D
hsa-miR-26a-5p_TTCAAGTAATCCAGGATAG	-0,150790268	3,646262645	0,770278793	0,882429594	T1D vs T2D
hsa-miR-10a-5p_TACCCTGTAGATCCGAATTTGT	-0,032672646	10,44682905	0,77060351	0,882429594	T1D vs T2D
hsa-miR-374a-5p_TTATAATACAACCTGATAAGT	-0,159458321	2,487762486	0,772761025	0,883630612	T1D vs T2D
hsa-miR-486-5p_GTACTGAGCTGCCCCGA	-0,111956259	4,609129379	0,774990599	0,883675659	T1D vs T2D
hsa-miR-423-3p_AAGCTCGGTCTGAGGCCCCTCAGT	-0,068539855	7,286433576	0,775017924	0,883675659	T1D vs T2D
hsa-miR-122-5p_TGGAGTGTGACAATGGTGTTTGT	0,083468598	8,198260953	0,787335395	0,893079753	T1D vs T2D
hsa-miR-501-3p_AATGCACCCGGGCAAGGATT	-0,073298458	6,44960483	0,788192672	0,893079753	T1D vs T2D
hsa-miR-30e-5p_TGTAAACATCCTTGACTGGAA	-0,05741764	6,536675988	0,788700977	0,893079753	T1D vs T2D
hsa-miR-139-3p_TGGAGACGCGGCCCTGTTGGAGT	-0,150786607	3,435697412	0,790686624	0,893079753	T1D vs T2D
hsa-miR-23a-3p_ATCACATTGCCAGGGATTTCC	0,027214512	11,08225248	0,791752146	0,893079753	T1D vs T2D
hsa-miR-486-5p_TGTACTGAGCTGCCCCGAG	-0,108172778	5,036658236	0,791832657	0,893079753	T1D vs T2D
hsa-miR-27a-3p_TTCACAGTGGCTAAGTTCCG	0,032032611	9,740987062	0,792093355	0,893079753	T1D vs T2D
hsa-miR-423-5p_GAGGGGCAGAGAGCGAGA	0,145809868	1,916584014	0,792230095	0,893079753	T1D vs T2D
hsa-let-7b-5p_TGAGGTAGTAGGTTGTGTGGTTT	0,031078973	11,28589984	0,797048048	0,894982416	T1D vs T2D
hsa-miR-486-5p_TCCTGTACTGAGCTGCCCCGAG	0,033894288	17,24661108	0,797800809	0,894982416	T1D vs T2D
hsa-miR-223-5p_CGTGTATTTGACAAGCTGAGTTG	-0,084918607	6,07891061	0,799419627	0,894982416	T1D vs T2D
hsa-miR-27a-3p_TTCACAGTGGCTAAGTTCC	-0,086739881	5,369078847	0,799843014	0,894982416	T1D vs T2D
hsa-let-7b-5p_GAGGTAGTAGGTTGTGTGGTT	-0,072134218	6,209954592	0,801750323	0,894982416	T1D vs T2D
hsa-miR-103a-3p_AGCAGCATTGTACAGGGCTATGA	-0,053137113	8,631709381	0,801963815	0,894982416	T1D vs T2D
hsa-miR-493-5p_TTGTACATGGTAGGCTTTCATT	0,150699698	3,64649227	0,802408581	0,894982416	T1D vs T2D
hsa-let-7f-5p_TGAGGTAGTAGATTGTATAGT	-0,025987605	10,56301224	0,802901414	0,894982416	T1D vs T2D
hsa-miR-21-5p_TAGCTTATCAGACTGATGTTGACT	0,108694071	5,943834972	0,805773509	0,894997398	T1D vs T2D
hsa-miR-505-3p_GTCAACACTTGCTGGTTTCCTCT	0,141935326	2,066469778	0,806024907	0,894997398	T1D vs T2D
hsa-miR-130a-3p_CAGTGCAATGTTAAAAGGGCA	-0,110332458	4,214432691	0,806638891	0,894997398	T1D vs T2D
hsa-miR-100-5p_AACCCGTAGATCCGAACT	-0,128944385	1,926738636	0,807406686	0,894997398	T1D vs T2D

hsa-miR-486-5p_TCCTGTACTGAGCTGCCCCGAGC	-0,052012398	6,188232343	0,809641943	0,895210392	T1D vs T2D
hsa-miR-191-5p_CAACGGAATCCCAAAAGCAGCTG	-0,042491218	9,658129048	0,810119795	0,895210392	T1D vs T2D
hsa-miR-30a-5p_TGTAAACATCCTCGACTGG	0,112372121	4,494972654	0,811620396	0,895210392	T1D vs T2D
hsa-miR-125a-5p_TCCCTGAGACCCTTTAACCTG	0,042265033	8,290201937	0,812091736	0,895210392	T1D vs T2D
hsa-miR-323a-3p_GCACATTACACGGTCGACCTCT	0,127197115	3,90036256	0,814648853	0,895596547	T1D vs T2D
hsa-miR-92a-3p_TATTGCACTTGTCCCGGCCTGTG	0,037864051	7,105169059	0,814689456	0,895596547	T1D vs T2D
hsa-miR-92a-3p_CACTTGTCCCGGCCTGT	-0,115527034	4,072780053	0,819172142	0,899284018	T1D vs T2D
hsa-miR-30a-5p_TGTAAACATCCTCGACTGGA	-0,120173346	3,19956619	0,820669152	0,89968819	T1D vs T2D
hsa-miR-92a-3p_GTATTGCACTTGTCCCGGCCTGT	-0,037896376	7,715362913	0,823395695	0,899926417	T1D vs T2D
hsa-miR-328-3p_CTGGCCCTCTCTGCCCTTCCGT	0,047651907	7,228551867	0,825447115	0,899926417	T1D vs T2D
hsa-miR-483-5p_AAGACGGGAGGAAAGAAGGGAG	0,07671163	6,618430366	0,825794947	0,899926417	T1D vs T2D
hsa-let-7d-5p_AGAGGTAGTAGGTTGCATAGTT	-0,043496185	9,075101282	0,825919532	0,899926417	T1D vs T2D
hsa-miR-320b_AAAAGCTGGGTTGAGAGGGC	-0,181159789	5,061955788	0,826532167	0,899926417	T1D vs T2D
hsa-let-7f-5p_TGAGGTAGTAGATTGTATAGTT	-0,034431913	12,51171334	0,829745033	0,902192076	T1D vs T2D
hsa-miR-25-3p_CATTGCACTTGTCTCGGTCT	-0,030898232	10,26006574	0,831403123	0,902763337	T1D vs T2D
hsa-miR-24-3p_TGGCTCAGTTCAGCAGGAACAG	0,021870305	12,76835022	0,837114147	0,907727858	T1D vs T2D
hsa-miR-145-3p_ATTCCTGGAAATACTGTTCT	-0,105980576	3,209907444	0,845225782	0,915278462	T1D vs T2D
hsa-miR-30d-5p_GTAAACATCCCCGACTGGAAG	0,062031894	5,727486807	0,847636755	0,915678603	T1D vs T2D
hsa-miR-21-5p_TAGCTTATCAGACTGATGTTGAC	-0,04305779	10,44971368	0,84789311	0,915678603	T1D vs T2D
hsa-miR-483-5p_AAGACGGGAGGAAAGAAGGGA	0,097856616	4,456894738	0,855640029	0,921884966	T1D vs T2D
hsa-let-7i-5p_GAGGTAGTAGTTTGTGCTG	-0,101262159	2,272109077	0,856293385	0,921884966	T1D vs T2D
hsa-miR-6803-3p_TCCCTCGCCTTCTCACCCTCAGT	0,105797009	2,14031641	0,857752004	0,921884966	T1D vs T2D
hsa-miR-148a-3p_TCAGTGCACTACAGAACTT	0,105366764	2,124123364	0,858266807	0,921884966	T1D vs T2D
hsa-miR-10a-5p_TACCCTGTAGATCCGAAT	0,099881013	2,33323359	0,861322845	0,922409774	T1D vs T2D
hsa-miR-375-3p_TTTGTTCGTTCGGCTCGCG	-0,066828961	5,830563474	0,86132788	0,922409774	T1D vs T2D
hsa-miR-125b-5p_TCCCTGAGACCCTAACTTGTG	-0,073195511	5,039062696	0,862364927	0,922409774	T1D vs T2D
hsa-miR-486-5p_CCTGTACTGAGCTGCCCCG	0,035570409	8,015321722	0,863601245	0,922409774	T1D vs T2D
hsa-miR-10a-5p_ACCCTGTAGATCCGAATTTGT	-0,036452716	7,643285241	0,864542159	0,922409774	T1D vs T2D
hsa-let-7a-5p_ATGAGGTAGTAGGTTGTATAGTT	0,094417258	2,103462699	0,869732611	0,925120818	T1D vs T2D
hsa-miR-335-5p_TCAAGAGCAATAACGAAAAATG	-0,029256576	8,412522999	0,87019835	0,925120818	T1D vs T2D
hsa-miR-335-3p_GTTTTTCATTATTGCTCCTGACC	-0,093154954	2,935136252	0,871965519	0,925120818	T1D vs T2D
hsa-miR-1306-5p_CCACCTCCCCTGCAAACGTCCA	0,092624146	3,283279778	0,872111791	0,925120818	T1D vs T2D
hsa-miR-15a-5p_TAGCAGCACATAATGGTTTGT	0,048127207	6,478410353	0,872886895	0,925120818	T1D vs T2D
hsa-let-7a-5p_TGAGGTAGTAGGTTGTATAGTT	-0,019860345	13,39549393	0,884650138	0,936342842	T1D vs T2D
hsa-miR-423-5p_TGAGGGGCAGAGAGCGAGACTTTT	0,015199509	11,39034963	0,886915461	0,93749552	T1D vs T2D
hsa-miR-143-3p_TGAGATGAAGCACTGTAGCT	0,023522708	9,047255511	0,888279515	0,93769374	T1D vs T2D
hsa-miR-16-2-3p_ACCAATATTACTGTGCTGCTT	0,022009356	8,312552057	0,889977434	0,938243406	T1D vs T2D
hsa-miR-92a-3p_TTGCACTTGTCCCGGCCTGT	0,017934545	8,384006303	0,896276088	0,943635458	T1D vs T2D
hsa-miR-222-3p_AGCTACATCTGGCTACTGGGT	-0,024400493	7,595221992	0,900080516	0,945644368	T1D vs T2D
hsa-miR-629-5p_TGGGTTTACGTTGGGAGAACTT	-0,061617145	4,034612462	0,900557183	0,945644368	T1D vs T2D
hsa-miR-320a-3p_AAAAGCTGGGTTGAGAGGGCG	0,014859553	10,00052492	0,902119148	0,946038107	T1D vs T2D
hsa-miR-146a-5p_GAGAACTGAATTCCATGGGTT	-0,038256469	6,481720435	0,903545887	0,946289188	T1D vs T2D
hsa-miR-501-3p_AATGCACCCGGGCAAGGATTCT	0,017116053	7,911300847	0,905509193	0,947100823	T1D vs T2D
hsa-miR-329-3p_AACACACCTGGTTAACCTCTTT	-0,063966131	3,759288959	0,907957429	0,948275825	T1D vs T2D
hsa-miR-98-5p_TGAGGTAGTAAGTTGTATTGTT	0,029031948	7,877215034	0,909012208	0,948275825	T1D vs T2D
hsa-miR-92b-3p_TATTGCACTCGTCCCGGCCT	0,040946937	5,68469958	0,913375702	0,951320594	T1D vs T2D
hsa-miR-423-3p_AGCTCGGTCTGAGGCCCCTCAG	-0,013088034	8,372128456	0,914318162	0,951320594	T1D vs T2D
hsa-miR-99a-5p_AACCCGTAGATCCGATCTTG	-0,018821287	8,041845005	0,916365122	0,951433022	T1D vs T2D
hsa-miR-101-3p_GTACAGTACTGTGATAACTGA	0,015757279	8,248925167	0,916813753	0,951433022	T1D vs T2D
hsa-miR-423-3p_AGCTCGGTCTGAGGCCCCTCAGT	0,011823422	11,76682308	0,923401304	0,956338777	T1D vs T2D
hsa-miR-10b-5p_TACCCTGTAGAACCGAATTTGT	-0,011190027	11,12588043	0,923940851	0,956338777	T1D vs T2D
hsa-miR-183-5p_TATGGCACTGGTAGAATTCACTG	-0,047174316	3,516695948	0,933650854	0,965135838	T1D vs T2D
hsa-let-7b-5p_TGAGGTAGTAGGTTGTGTGGTT	-0,007034361	12,77995871	0,938798265	0,967511683	T1D vs T2D
hsa-let-7a-5p_GAGGTAGTAGGTTGTATAGTT	-0,019160469	7,081547935	0,939398	0,967511683	T1D vs T2D
hsa-miR-1-3p_TGGAATGTAAAGAAGTATGTAT	0,038413096	5,995923161	0,939591019	0,967511683	T1D vs T2D
hsa-miR-423-3p_GCTCGGTCTGAGGCCCCTCAGT	-0,020233748	6,432183419	0,947165132	0,971704323	T1D vs T2D
hsa-miR-21-5p_TAGCTTATCAGACTGATGTTGA	0,00534815	12,88530966	0,947393437	0,971704323	T1D vs T2D
hsa-miR-10a-5p_TACCCTGTAGATCCGAATT	-0,033876623	3,933865051	0,947951008	0,971704323	T1D vs T2D
hsa-miR-3613-5p_TGTTGTACTTTTTTTTTTGT	-0,010128551	7,586394808	0,94887465	0,971704323	T1D vs T2D
hsa-let-7g-5p_GAGGTAGTAGTTTGTACAGTT	-0,034902049	4,344091711	0,949892574	0,971704323	T1D vs T2D
hsa-let-7a-5p_TGAGGTAGTAGGTTGTATAGT	0,006039065	11,86713156	0,950977882	0,971704323	T1D vs T2D

hsa-let-7b-5p_TGAGGTAGTAGGTTGTGTG	-0,010577967	6,962968331	0,959851362	0,97951541	T1D vs T2D
hsa-miR-15b-5p_TAGCAGCACATCATGGTT	0,011749639	7,417344831	0,962199317	0,979989412	T1D vs T2D
hsa-miR-4685-3p_TCTCCCTTCCTGCCCTGGCT	0,020667513	4,862788515	0,963125407	0,979989412	T1D vs T2D
hsa-miR-424-5p_CAGCAGCAATTCATGTTTTGA	0,025026117	2,646439112	0,964004641	0,979989412	T1D vs T2D
hsa-miR-374a-5p_TTATAATACAACCTGATAAGTG	0,01919731	4,272038394	0,965281252	0,980037144	T1D vs T2D
hsa-miR-199a-3p_ACAGTAGTCTGCACATTGGTT	-0,035745374	4,64378834	0,966743833	0,980273327	T1D vs T2D
hsa-miR-191-5p_CAACGGAATCCCAAAAGCAGCT	0,003594826	11,11779618	0,976769095	0,989180392	T1D vs T2D
hsa-miR-16-5p_AGCAGCACGTAAATATTGGCG	0,004340485	8,17366975	0,978071592	0,989242461	T1D vs T2D
hsa-miR-29b-3p_TAGCACCATTTGAAATCAGT	0,007964327	4,363760053	0,986634517	0,995971474	T1D vs T2D
hsa-miR-29a-3p_TAGCACCATCTGAAATCGGT	-0,002821395	8,663780214	0,987755792	0,995971474	T1D vs T2D
hsa-miR-22-3p_AAGCTGCCAGTTGAAGAAC	-0,002682454	8,203664635	0,988473571	0,995971474	T1D vs T2D
hsa-miR-103a-3p_AGCAGCATTGTACAGGGCT	0,010207677	5,118424827	0,990166593	0,996417645	T1D vs T2D
hsa-miR-15b-5p_TAGCAGCACATCATGGTTT	-0,001220182	8,514548835	0,99416729	0,999182005	T1D vs T2D
hsa-miR-24-3p_GGCTCAGTTCAGCAGGAACA	-0,001481793	2,640585933	0,997853135	0,99952527	T1D vs T2D
hsa-miR-30d-5p_TGTAAACATCCCCGACTGG	-0,000318036	8,045778015	0,998110712	0,99952527	T1D vs T2D
hsa-miR-28-3p_CACTAGATTGTGAGCTCCTGGAG	-0,00115254	2,845963244	0,998271161	0,99952527	T1D vs T2D
hsa-miR-10b-5p_ACCCTGTAGAACCGAATTTG	2,39322E-05	4,835525593	0,999956037	0,999956037	T1D vs T2D
hsa-miR-23b-3p_ATCACATTGCCAGGGATTACC1	1,374839546	5,483511586	5,38023E-05	0,042880428	LADA vs T1D
hsa-miR-125a-5p_TCCCTGAGACCCTTTAACCT1	0,903936193	6,34415738	0,00024171	0,072030076	LADA vs T1D
hsa-miR-130b-3p_CAGTGCAATGATGAAAGGGCA1	1,572809958	4,137452675	0,00027113	0,072030076	LADA vs T1D
hsa-miR-423-5p_CTGAGGGGCAGAGAGCGAGACT1	1,714284443	2,475285897	0,001458462	0,290598568	LADA vs T1D
hsa-miR-126-5p_CATTATTACTTTTGGTACGCG1	0,724070741	6,678225687	0,002253414	0,335118574	LADA vs T1D
hsa-miR-542-3p_TGTGACAGATTGATAACTGA1	1,665168094	1,954666629	0,002678166	0,335118574	LADA vs T1D
hsa-miR-30a-5p_TGTAAACATCCTCGACTGGAAGCT1	0,359791383	8,778765877	0,00324526	0,335118574	LADA vs T1D
hsa-miR-27b-3p_TTCACAGTGGCTAAGTTCTGCA1	1,512444246	3,596858077	0,004197076	0,335118574	LADA vs T1D
hsa-miR-99b-5p_CACCCGTAGAACCGACCTTGCG1	0,445981995	9,378733966	0,00468224	0,335118574	LADA vs T1D
hsa-miR-139-5p_TCTACAGTGCACGTGTCTCCA1	1,344750781	4,69390328	0,004767092	0,335118574	LADA vs T1D
hsa-miR-10b-5p_TACCCTGTAGAACCGAATTTGT1	0,332734271	11,12588043	0,004827464	0,335118574	LADA vs T1D
hsa-miR-125a-5p_TCCCTGAGACCCTTTAACCTGTG1	0,425516473	8,600810288	0,006031706	0,335118574	LADA vs T1D
hsa-miR-10a-5p_TACCCTGTAGATCCGAATTTGTG1	0,617427561	7,257876357	0,006111837	0,335118574	LADA vs T1D
hsa-miR-23a-3p_TCACATTGCCAGGGATTTCCAAC1	1,421793569	2,126412114	0,006280556	0,335118574	LADA vs T1D
hsa-let-7f-5p_TGAGGTAGTAGATTGTATA1	1,426618376	3,61271535	0,007019262	0,335118574	LADA vs T1D
hsa-miR-23b-3p_ATCACATTGCCAGGGATTAC1	1,413132104	2,694770844	0,007293807	0,335118574	LADA vs T1D
hsa-miR-92a-3p_TATTGCACTTGTCCCGG1	1,165365326	4,594134091	0,007311428	0,335118574	LADA vs T1D
hsa-miR-30a-5p_TGTAAACATCCTCGACTGGAA1	0,54042366	7,169227794	0,00756855	0,335118574	LADA vs T1D
hsa-miR-128-3p_TCACAGTGAACCGGTCTCTTTT1	0,997238092	5,404238817	0,008900649	0,365240312	LADA vs T1D
hsa-let-7e-5p_TGAGGTAGGAGGTTGTATAGTT1	0,543363496	7,813417368	0,00926821	0,365240312	LADA vs T1D
hsa-miR-125a-5p_TCCCTGAGACCCTTTAACCTGT1	0,307342343	11,48945871	0,009855327	0,365240312	LADA vs T1D
hsa-miR-421_ATCAACAGACATTAATTGGGCG1	1,301066793	2,09434672	0,010081916	0,365240312	LADA vs T1D
hsa-miR-30a-5p_TGTAAACATCCTCGACTGGAAGC1	0,433626757	7,798673355	0,01208341	0,398937509	LADA vs T1D
hsa-miR-451a_AAACCGTTACCATTACTG1	1,279814785	2,491678877	0,012430212	0,398937509	LADA vs T1D
hsa-let-7e-5p_TGAGGTAGGAGGTTGTATAGT1	1,146492316	4,645402812	0,012603569	0,398937509	LADA vs T1D
hsa-miR-451a_GAAACCGTTACCATTACTGAG1	1,264373694	3,176702933	0,01343805	0,398937509	LADA vs T1D
hsa-miR-342-3p_TCTCACACAGAAATCGCACCCGTC1	0,485341182	7,583667414	0,013514821	0,398937509	LADA vs T1D
hsa-miR-223-3p_TGTCAGTTTGTCAAATACC1	0,961520186	5,409428462	0,014154847	0,402907611	LADA vs T1D
hsa-miR-92b-3p_TATTGCACTCGTCCCGGCCTC1	-1,165564288	3,886574178	0,015509621	0,424385814	LADA vs T1D
hsa-miR-142-5p_CCCATAAAGTAGAAAGCAC1	0,828114872	5,201334155	0,016442576	0,424385814	LADA vs T1D
hsa-miR-361-3p_TCCCCCAGGTGTGATTCTGATT1	1,132030599	3,650889743	0,016948054	0,424385814	LADA vs T1D
hsa-miR-423-5p_GAGGGGCAGAGAGCGAGACTT1	0,943382722	5,367382166	0,017998468	0,424385814	LADA vs T1D
hsa-miR-150-3p_CTGGTACAGGCCTGGGGGACA1	1,250586021	4,060108896	0,01811182	0,424385814	LADA vs T1D
hsa-miR-142-5p_CATAAAGTAGAAAGCACT1	0,636936381	5,539841362	0,018371492	0,424385814	LADA vs T1D
hsa-miR-10b-5p_TACCCTGTAGAACCGAATTTGTG1	0,47643105	7,78613959	0,01877982	0,424385814	LADA vs T1D
hsa-miR-140-3p_ACCACAGGGTAGAACCACGGA1	-0,655760344	6,646596672	0,019169246	0,424385814	LADA vs T1D
hsa-miR-6803-3p_TCCCTCGCCTTCTCACCCTCAGT1	1,372435743	2,14031641	0,02071951	0,439992166	LADA vs T1D
hsa-miR-20a-5p_TAAAGTGCTTATAGTGCAGGTAG1	-1,05080146	4,004629847	0,020978296	0,439992166	LADA vs T1D
hsa-miR-10b-5p_ACCCTGTAGAACCGAATTTGT1	0,494396051	6,785991828	0,022429915	0,458375452	LADA vs T1D
hsa-miR-320a-3p_AAAGCTGGGTTGAGAGGGCGAA1	-1,036090095	4,271030608	0,025134375	0,50080243	LADA vs T1D
hsa-let-7b-5p_TGAGGTAGTAGGTTGTGTG1	-0,470724082	6,962968331	0,025842891	0,502360592	LADA vs T1D
hsa-miR-15b-3p_CGAATCATTATTTGCTGCTCT1	-0,987672547	4,350054835	0,026596659	0,504703268	LADA vs T1D
hsa-miR-375-3p_TTGTTCGTTCGGCTCGCGTGA1	-0,900475129	5,662970821	0,027766799	0,511303595	LADA vs T1D
hsa-miR-92a-3p_ACTTGTCCCGGCCTGT1	1,230171872	2,134254049	0,028462267	0,511303595	LADA vs T1D

hsa-miR-16-5p_TAGCAGCACGTAAATATTGGC1	-0,265978379	9,94541903	0,028869086	0,511303595	LADA vs T1D
hsa-miR-500a-3p_AATGCACCTGGGCAAGGATTCT1	1,283028715	2,780066987	0,030348437	0,51317398	LADA vs T1D
hsa-miR-342-3p_TCTCACACAGAAATCGCACCCGTCA1	1,182755157	3,422364811	0,030996628	0,51317398	LADA vs T1D
hsa-miR-145-5p_GTCCAGTTTTCCCAGGAATCC1	1,173661753	2,283533911	0,031503061	0,51317398	LADA vs T1D
hsa-miR-23a-3p_TCACATTGCCAGGGATTTCCA1	0,851595557	5,236791923	0,03155022	0,51317398	LADA vs T1D
hsa-miR-27a-3p_TCACAGTGGCTAAGTTCCG1	1,18664952	2,668037972	0,032442646	0,517135776	LADA vs T1D
hsa-miR-10b-5p_TACCCTGTAGAACCGAATTTGTGT1	1,166004126	2,268128931	0,034641658	0,541360805	LADA vs T1D
hsa-miR-191-5p_CAACGGAATCCCAAAAGCAG1	0,228930308	10,17632421	0,036168884	0,551947996	LADA vs T1D
hsa-miR-24-3p_GGCTCAGTTCAGCAGGAACA1	-1,155843862	2,640585933	0,036852096	0,551947996	LADA vs T1D
hsa-miR-10b-5p_TACCCTGTAGAACCGAATTT1	0,801428473	5,2763128	0,038024581	0,551947996	LADA vs T1D
hsa-miR-139-5p_TCTACAGTGCACGTGTCTCCAG1	0,908241042	4,676823231	0,040682147	0,551947996	LADA vs T1D
hsa-miR-101-3p_GTACAGTACTGTGATAACTG1	0,749762526	5,734652626	0,041624192	0,551947996	LADA vs T1D
hsa-miR-145-5p_GTCCAGTTTTCCCAGGAATCCCT1	1,172978257	2,146327793	0,041862214	0,551947996	LADA vs T1D
hsa-miR-423-5p_CTGAGGGGCAGAGAGCGAGACTTT1	0,990433634	4,419437576	0,042929909	0,551947996	LADA vs T1D
hsa-miR-30d-5p_TGTAAACATCCCCGACTGGA1	0,295606783	9,18587305	0,043108558	0,551947996	LADA vs T1D
hsa-miR-335-5p_TCAAGAGCAATAACGAAAAATGT1	0,686378884	6,149787911	0,043601465	0,551947996	LADA vs T1D
hsa-miR-21-5p_AGCTTATCAGACTGATGTTGA1	0,434999674	6,721668578	0,043704205	0,551947996	LADA vs T1D
hsa-let-7b-5p_TGAGGTAGTAGGTTGTGT1	0,74904024	5,292834315	0,044442531	0,551947996	LADA vs T1D
hsa-miR-335-3p_GTTTTTCATTATTGCTCCTGACC1	-1,164759279	2,935136252	0,044604165	0,551947996	LADA vs T1D
hsa-miR-151a-3p_TACTAGACTGAAGCTCCTTGAGG1	1,023819151	4,127573648	0,044757926	0,551947996	LADA vs T1D
hsa-miR-3615_TCTCTCGGCTCCTCGCGGCTCGC1	0,993816493	3,835837244	0,045014579	0,551947996	LADA vs T1D
hsa-miR-10a-5p_ACCCTGTAGATCCGAATTTGTG1	0,598206333	6,001615458	0,047611759	0,57494806	LADA vs T1D
hsa-miR-423-3p_AGCTCGGTCTGAGGCCCCTCAG1	-0,240633241	8,372128456	0,048752193	0,579932802	LADA vs T1D
hsa-let-7b-5p_GAGGTAGTAGGTTGTGTGGTT1	-0,559285433	6,209954592	0,052569899	0,607578925	LADA vs T1D
hsa-miR-139-5p_TCTACAGTGCACGTGTCTCCAGT1	0,505096673	6,587018882	0,052600936	0,607578925	LADA vs T1D
hsa-miR-1249-3p_ACGCCCTTCCCCCCCTTCTTCA1	1,172699596	2,195504284	0,05607051	0,637290424	LADA vs T1D
hsa-miR-142-5p_CCCATAAAGTAGAAAGCA1	0,893071788	2,550307907	0,056772422	0,637290424	LADA vs T1D
hsa-miR-320a-3p_GAAAAGCTGGGTTGAGAGGGCG1	-0,585398159	6,114738168	0,061277304	0,672314251	LADA vs T1D
hsa-miR-150-3p_CTGGTACAGGCCTGGGGGAC1	1,060686912	2,308717969	0,061990985	0,672314251	LADA vs T1D
hsa-miR-93-5p_AAAGTGCTGTTCGTGCAGGTAG1	0,944940186	3,611226251	0,062423155	0,672314251	LADA vs T1D
hsa-miR-145-5p_GTCCAGTTTTCCCAGGAATCCC1	1,065423016	3,12605684	0,063502851	0,674823635	LADA vs T1D
hsa-miR-374b-5p_ATATAATACAACCTGCTAAGTG1	0,994975424	2,723967557	0,064381679	0,675160505	LADA vs T1D
hsa-miR-106b-3p_CCGCACTGTGGGTACTTGCT1	-0,584763213	6,396206525	0,065756452	0,678873856	LADA vs T1D
hsa-miR-329-3p_ACACACCTGGTTAACCTCTTTT1	1,072980834	2,079797897	0,066719739	0,678873856	LADA vs T1D
hsa-miR-99b-5p_CACCCGTAGAACCGACCTTG1	0,43987942	6,765454723	0,067291135	0,678873856	LADA vs T1D
hsa-miR-125a-5p_CCCTGAGACCCTTTAACCTGT1	0,725299362	5,351640533	0,069661186	0,680496804	LADA vs T1D
hsa-miR-24-3p_TGGCTCAGTTCAGCAGGAAC1	0,207322007	10,07194833	0,069832073	0,680496804	LADA vs T1D
hsa-miR-126-5p_ATTATTACTTTTGGTACGCGCT1	0,981247561	2,122638684	0,070724348	0,680496804	LADA vs T1D
hsa-miR-10b-5p_TACCCTGTAGAACCGAATT1	-0,858119781	4,675674869	0,070867296	0,680496804	LADA vs T1D
hsa-miR-106b-5p_TAAAGTGCTGACAGTGCAGAT1	0,921504523	2,655083548	0,074018543	0,700994008	LADA vs T1D
hsa-miR-25-3p_CATTGCACTTGTCTCGGT1	0,873392595	3,623128964	0,074760967	0,700994008	LADA vs T1D
hsa-miR-150-5p_GTCTCCCAACCCTTGTACCAGT1	0,957732465	2,340151294	0,075955268	0,701675469	LADA vs T1D
hsa-miR-140-3p_TACCACAGGGTAGAACCACGG1	0,552380618	5,563733367	0,076800464	0,701675469	LADA vs T1D
hsa-miR-181b-5p_AACATTCATTGCTGTCGGTG1	0,968336308	2,058689202	0,078812022	0,701675469	LADA vs T1D
hsa-miR-2110_TTGGGGAAACGGCCGCTGAGT1	0,571013701	5,974874685	0,079224883	0,701675469	LADA vs T1D
hsa-miR-30e-5p_TAAACATCCTTGACTGGAAGCT1	0,974523906	2,076536633	0,080451349	0,701675469	LADA vs T1D
hsa-miR-30d-5p_GTAAACATCCCCGACTGGAA1	0,700396688	4,975135303	0,080682226	0,701675469	LADA vs T1D
hsa-miR-215-5p_ATGACCTATGAATTGACAGA1	-0,886716686	4,790158483	0,081237504	0,701675469	LADA vs T1D
hsa-miR-340-3p_TCCGTCTCAGTTACTTTATAGCC1	0,98379767	3,094594983	0,081876811	0,701675469	LADA vs T1D
hsa-miR-126-3p_TCGTACCGTGAGTAATAATGCG1	0,234046106	11,19647801	0,083412386	0,703109504	LADA vs T1D
hsa-miR-10b-5p_TACCCTGTAGAACCGAATTTG1	0,214405555	9,833049317	0,083808536	0,703109504	LADA vs T1D
hsa-miR-10a-5p_TACCCTGTAGATCCGAATTTGT1	0,193198509	10,44682905	0,085208891	0,707411311	LADA vs T1D
hsa-miR-1306-5p_CCACCTCCCCTGCAAACGTC1	-0,76579048	4,129732962	0,087092761	0,707745123	LADA vs T1D
hsa-miR-342-3p_CTCACACAGAAATCGCACCCG1	0,979228064	2,495329338	0,087737731	0,707745123	LADA vs T1D
hsa-miR-125a-5p_TCCCTGAGACCCTTTAACCTGTGA1	0,345255708	8,237628665	0,087913133	0,707745123	LADA vs T1D
hsa-miR-30e-3p_CTTTCAGTCGGATGTTTACAGC1	0,932378493	3,314941715	0,091821935	0,719837933	LADA vs T1D
hsa-miR-25-3p_ATTGCACTTGTCTCGGTCT1	0,806820226	3,620590845	0,091829802	0,719837933	LADA vs T1D
hsa-miR-26b-5p_TTCAAGTAATTCAGGATAGG1	-0,77443367	3,436379997	0,092124804	0,719837933	LADA vs T1D
hsa-miR-30d-5p_TGTAAACATCCCCGACTGG1	0,226237073	8,045778015	0,09325737	0,721612852	LADA vs T1D
hsa-miR-409-3p_GAATGTTGCTCGGTGAACCCCT1	-0,579711259	7,973406619	0,097885135	0,743412753	LADA vs T1D
hsa-miR-487b-3p_AATCGTACAGGGTCATCCACTT1	-0,966965508	4,048228768	0,098588076	0,743412753	LADA vs T1D

hsa-miR-361-3p_TCCCCCAGGTGTGATTCTGATTT1	-0,799859739	3,977031543	0,100501485	0,743412753	LADA vs T1D
hsa-miR-16-5p_CTAGCAGCACGTAAATATTGGCG1	0,687112546	4,959212286	0,101090659	0,743412753	LADA vs T1D
hsa-miR-584-5p_TTATGGTTTGCCTGGGACT1	0,708895804	4,595669388	0,104814221	0,743412753	LADA vs T1D
hsa-miR-423-5p_GAGGGGCAGAGAGCGAGACTTTT1	0,791195692	4,029696332	0,106439999	0,743412753	LADA vs T1D
hsa-miR-29a-3p_TAGCACCATCTGAAATCGGTTAT1	0,892780827	2,486065735	0,106681216	0,743412753	LADA vs T1D
hsa-miR-10b-5p_ACCCTGTAGAACCGAATTTGTG1	0,502917815	5,829978228	0,106760703	0,743412753	LADA vs T1D
hsa-miR-10a-5p_ACCCTGTAGATCCGAATTTG1	0,503629903	5,718851669	0,106881948	0,743412753	LADA vs T1D
hsa-miR-345-5p_GCTGACTCCTAGTCCAGGGCT1	-0,668304604	5,057881275	0,106993888	0,743412753	LADA vs T1D
hsa-miR-29c-3p_TAGCACCATTTGAAATCGGTT1	0,765525922	3,850371057	0,107735013	0,743412753	LADA vs T1D
hsa-miR-29a-3p_TAGCACCATCTGAAATCGGTT1	0,27917793	8,641484144	0,108102916	0,743412753	LADA vs T1D
hsa-miR-27a-3p_TTCACAGTGGCTAAGTTCCG1	0,195573725	9,740987062	0,108200601	0,743412753	LADA vs T1D
hsa-miR-181a-5p_AACATTCAACGCTGTCGGTGAGT1	0,308618625	8,031521361	0,109336908	0,744799278	LADA vs T1D
hsa-miR-320b_AAAAGCTGGGTTGAGAGGGC1	-1,305072171	5,061955788	0,113001578	0,758205009	LADA vs T1D
hsa-miR-338-5p_AACAATATCCTGGTGCTGAGT1	0,490956254	5,993094666	0,113207523	0,758205009	LADA vs T1D
hsa-miR-505-3p_CGTCAACACTTGCTGGTTTCCTCT1	0,919316485	2,947139365	0,115793416	0,76181147	LADA vs T1D
hsa-miR-192-5p_TGACCTATGAATTGACAGC1	-0,897054981	3,665629227	0,115984851	0,76181147	LADA vs T1D
hsa-let-7f-5p_GAGGTAGTAGATTGTATAG1	0,802899074	3,245137192	0,117279311	0,76181147	LADA vs T1D
hsa-miR-92a-3p_GTATTGCACTTGTCCCGGCCTG1	0,852615025	3,065032086	0,117869068	0,76181147	LADA vs T1D
hsa-miR-495-3p_AAACAAACATGGTGCACTTCTT1	0,94085249	2,485368847	0,119004798	0,76181147	LADA vs T1D
hsa-miR-30a-5p_TGTAAACATCCTCGACTGGAAG1	0,27360956	8,337829601	0,120385401	0,76181147	LADA vs T1D
hsa-miR-15b-5p_TAGCAGCACATCATGGTT1	-0,385351247	7,417344831	0,120867425	0,76181147	LADA vs T1D
hsa-let-7i-5p_GAGGTAGTAGTTTGTGCTG1	0,866648149	2,272109077	0,122133849	0,76181147	LADA vs T1D
hsa-miR-375-3p_TTTGTTCGTTCGGCTCGCGTGA1	-0,359258756	10,08392589	0,122348643	0,76181147	LADA vs T1D
hsa-miR-23a-5p_GGGGTTCCTGGGGATGGGATTT1	0,797217643	3,81817711	0,123493456	0,762978951	LADA vs T1D
hsa-miR-328-3p_CTGGCCCTCTCTGCCCTTCCGT1	-0,331464028	7,228551867	0,126081771	0,772978241	LADA vs T1D
hsa-miR-16-5p_TAGCAGCACGTAAATATTG1	-0,51055042	5,566162995	0,129270373	0,780307128	LADA vs T1D
hsa-miR-486-5p_TCCTGTACTGAGCTGCCCC1	-0,323493832	6,793601467	0,130880774	0,780307128	LADA vs T1D
hsa-miR-146a-5p_TGAGAACTGAATTCCATGGGTT1	0,174543451	12,1607009	0,13177396	0,780307128	LADA vs T1D
hsa-miR-182-5p_TTTGGCAATGGTAGAACTCAC1	0,814214666	3,148572393	0,131873112	0,780307128	LADA vs T1D
hsa-miR-221-3p_AGCTACATTGTCTGCTGGGTTTCA1	0,843367759	2,390031097	0,132987336	0,780307128	LADA vs T1D
hsa-miR-191-5p_CAACGGAATCCCAAAAGCA1	0,42850858	6,203677568	0,13315153	0,780307128	LADA vs T1D
hsa-miR-423-5p_GAGGGGCAGAGAGCGAGA1	0,823696849	1,916584014	0,137342734	0,791609343	LADA vs T1D
hsa-miR-425-5p_AATGACACGATCACTCCCGTTGAGT1	0,341967037	8,386717544	0,138477895	0,791609343	LADA vs T1D
hsa-miR-423-3p_GCTCGGTCTGAGGCCCCTCAG1	0,818208901	1,954419998	0,142062753	0,791609343	LADA vs T1D
hsa-miR-329-3p_AACACACCTGGTTAACCTCTT1	0,831513074	3,637419766	0,144893397	0,791609343	LADA vs T1D
hsa-miR-126-3p_TCGTACCGTGAGTAATAATG1	0,442313687	5,867396204	0,146226257	0,791609343	LADA vs T1D
hsa-miR-29c-3p_TAGCACCATTTGAAATCGG1	0,78203532	3,272314395	0,147350642	0,791609343	LADA vs T1D
hsa-miR-22-3p_AGCTGCCAGTTGAAGAACTGT1	-0,542485832	5,523006419	0,147757705	0,791609343	LADA vs T1D
hsa-miR-144-3p_CTACAGTATAGATGATGTAC1	0,777472995	3,24422031	0,148363016	0,791609343	LADA vs T1D
hsa-miR-652-3p_AATGGCGCCACTAGGGTTGTG1	-0,393342756	6,468098031	0,148657512	0,791609343	LADA vs T1D
hsa-miR-629-5p_TGGGTTTACGTTGGGAGAA1	0,842000791	2,515869067	0,14887058	0,791609343	LADA vs T1D
hsa-miR-1908-5p_CGGCGGGGACGGCGATTGGTC1	0,819762284	2,612171571	0,150743327	0,791609343	LADA vs T1D
hsa-miR-181a-5p_AACATTCAACGCTGTCGGTGAG1	0,259247638	7,366086853	0,151827949	0,791609343	LADA vs T1D
hsa-miR-451a_GAAACCGTTACCATTACTGAGT1	0,330356028	6,98332366	0,152550778	0,791609343	LADA vs T1D
hsa-miR-30a-3p_CTTTCAGTCGGATGTTTGCAG1	0,773047974	1,943754996	0,153651004	0,791609343	LADA vs T1D
hsa-miR-30b-5p_TGTAAACATCCTACACTCAGCT1	0,3639473	6,722043115	0,154521839	0,791609343	LADA vs T1D
hsa-miR-363-3p_AATTGCACGGTATCCATCT1	-0,684175338	4,478964619	0,15470237	0,791609343	LADA vs T1D
hsa-miR-100-5p_AACCCGTAGATCCGAACTTG1	0,429691994	5,941970306	0,157962951	0,791609343	LADA vs T1D
hsa-let-7a-5p_GTGAGGTAGTAGGTTGTATAGTT1	0,842428031	2,866169762	0,158277576	0,791609343	LADA vs T1D
hsa-miR-185-5p_TGGAGAGAAAGGCAGTTCCTGA1	-0,191614517	8,851967404	0,158529763	0,791609343	LADA vs T1D
hsa-miR-1306-5p_CCACCTCCCCTGCAAACGT1	-0,84774942	2,734536196	0,160246889	0,791609343	LADA vs T1D
hsa-miR-486-5p_TGTACTGAGCTGCCCCGAG1	-0,578372472	5,036658236	0,160340551	0,791609343	LADA vs T1D
hsa-miR-194-5p_TGTAACAGCAACTCCATGTGGA1	0,763977404	2,048630169	0,16090756	0,791609343	LADA vs T1D
hsa-miR-486-3p_CGGGGCAGCTCAGTACAGGA1	-0,419973692	6,539637794	0,161137381	0,791609343	LADA vs T1D
hsa-miR-26a-5p_TTCAAGTAATCCAGGATAGGC1	0,225427944	7,510198846	0,161792229	0,791609343	LADA vs T1D
hsa-miR-191-5p_AACGGAATCCCAAAAGCAGC1	0,741880942	2,920397045	0,161876982	0,791609343	LADA vs T1D
hsa-miR-652-3p_AATGGCGCCACTAGGGTTGTGC1	-0,705516995	3,594273542	0,162007431	0,791609343	LADA vs T1D
hsa-miR-103a-3p_AGCAGCATTGTACAGGGCTATG1	0,739232474	4,276844631	0,162534648	0,791609343	LADA vs T1D
hsa-miR-10a-5p_TACCCTGTAGATCCGAAT1	0,798781788	2,33323359	0,162890756	0,791609343	LADA vs T1D
hsa-miR-122-5p_TGGAGTGTGACAATGGTGTTTG1	-0,371758695	11,63439763	0,16728689	0,797129375	LADA vs T1D
hsa-miR-191-5p_CAACGGAATCCCAAAAGCAGCTGT1	0,601035214	5,688164696	0,16768396	0,797129375	LADA vs T1D

hsa-miR-144-5p_GATATCATCATATACTGTAAGTT1	0,781539382	2,459029553	0,168019691	0,797129375	LADA vs T1D
hsa-miR-423-5p_AGGGGCAGAGAGCGAGACTTT1	0,48092773	5,332499304	0,168027271	0,797129375	LADA vs T1D
hsa-let-7a-5p_GAGGTAGTAGGTTGTATAGTT1	0,34500303	7,081547935	0,171057061	0,801783964	LADA vs T1D
hsa-miR-126-3p_CTCGTACCGTGAGTAATAATGCG1	0,643664694	4,682348296	0,172008163	0,801783964	LADA vs T1D
hsa-miR-486-5p_TGTACTGAGCTGCCCCGA1	0,663506001	4,617811312	0,172026421	0,801783964	LADA vs T1D
hsa-miR-7-5p_TGGAAGACTAGTGATTTTGT1	0,728781955	3,91952121	0,174109858	0,804230575	LADA vs T1D
hsa-miR-142-5p_CATAAAGTAGAAAGCACTA1	0,697480725	2,153538022	0,174569498	0,804230575	LADA vs T1D
hsa-miR-363-3p_ATTGCACGGTATCCATCTGT1	0,640885856	4,343538892	0,177666762	0,809287389	LADA vs T1D
hsa-miR-30c-5p_GTAAACATCCTACACTCTCAGCT1	0,75512216	3,052199181	0,178093525	0,809287389	LADA vs T1D
hsa-miR-16-5p_AGCAGCACGTAAATATTGGC1	-0,709898647	2,995434135	0,179245411	0,809287389	LADA vs T1D
hsa-miR-486-5p_GTACTGAGCTGCCCCGA1	-0,526908299	4,609129379	0,1801652	0,809287389	LADA vs T1D
hsa-miR-24-3p_TGGCTCAGTTCAGCAGGAACA1	-0,146786653	9,988806411	0,181410617	0,809287389	LADA vs T1D
hsa-miR-342-3p_TCACACAGAAATCGCACCCGT1	0,71985536	2,35866661	0,181759652	0,809287389	LADA vs T1D
hsa-miR-101-3p_GTACAGTACTGTGATAACTGAA1	0,731296009	3,655178095	0,185650182	0,812366102	LADA vs T1D
hsa-miR-500a-3p_ATGCACCTGGGCAAGGATTCT1	0,645059028	4,513942296	0,187150479	0,812366102	LADA vs T1D
hsa-miR-421_ATCAACAGACATTAATTGGGCGC1	-0,688062214	2,04052248	0,188481128	0,812366102	LADA vs T1D
hsa-miR-150-5p_CTCCCAACCCTTGTACCAGTG1	0,627813671	4,313974888	0,189329482	0,812366102	LADA vs T1D
hsa-miR-15b-5p_TAGCAGCACATCATGGTTTACA1	0,393123956	6,32576652	0,18973833	0,812366102	LADA vs T1D
hsa-miR-451a_AAACCGTTACCATTACTGA1	0,228910454	8,929457213	0,192987695	0,812366102	LADA vs T1D
hsa-miR-3605-3p_CCTCCGTGTTACCTGTCCTCT1	0,556996987	5,168364179	0,193953835	0,812366102	LADA vs T1D
hsa-miR-126-3p_TCGTACCGTGAGTAATAATGC1	0,304891373	6,426310401	0,194571175	0,812366102	LADA vs T1D
hsa-miR-122-5p_GGAGTGTGACAATGGTGTTT1	0,449234234	7,666405464	0,194594199	0,812366102	LADA vs T1D
hsa-let-7i-5p_GAGGTAGTAGTTTGTGCTGT1	0,595017676	4,789553904	0,194728584	0,812366102	LADA vs T1D
hsa-miR-27b-3p_TTCACAGTGGCTAAGTTCTG1	0,22753082	8,279294906	0,194754811	0,812366102	LADA vs T1D
hsa-let-7g-5p_TGAGGTAGTAGTTTGTACAG1	-0,166478815	9,407543438	0,195124179	0,812366102	LADA vs T1D
hsa-miR-664a-5p_ACTGGCTAGGGAAAATGATTGG1	0,66861298	3,587998944	0,195943601	0,812366102	LADA vs T1D
hsa-miR-4433b-5p_TGTCCCACCCCCACTCCTGTT1	-0,565729812	6,729375179	0,197041976	0,812366102	LADA vs T1D
hsa-miR-18a-3p_ACTGCCCTAAGTGCTCCTTCT1	0,712216743	3,273299722	0,198511725	0,812366102	LADA vs T1D
hsa-miR-92a-3p_ATTGCACTTGTCCCGGCCTGTT1	0,436665302	5,604292671	0,199066604	0,812366102	LADA vs T1D
hsa-miR-182-5p_TTTGGCAATGGTAGAACTCACA1	-0,347248613	6,943076472	0,199927389	0,812366102	LADA vs T1D
hsa-let-7b-3p_CTATACAACCTACTGCCTTCC1	0,351286139	6,023895535	0,200798146	0,812366102	LADA vs T1D
hsa-let-7c-5p_TGAGGTAGTAGGTTGTATGG1	-0,450806867	3,04066672	0,204464939	0,81280967	LADA vs T1D
hsa-miR-193a-5p_TGGGTCTTTGCGGGCGAGA1	0,735044462	3,488597754	0,204678611	0,81280967	LADA vs T1D
hsa-miR-16-5p_TAGCAGCACGTAAATATTGGCGT1	-0,234255719	7,90073048	0,204766632	0,81280967	LADA vs T1D
hsa-let-7a-5p_AGGTAGTAGGTTGTATAGTT1	-0,708338614	1,879378714	0,204987131	0,81280967	LADA vs T1D
hsa-miR-29a-3p_CTAGCACCATCTGAAATCGG1	0,64963563	3,050349736	0,209544474	0,826767057	LADA vs T1D
hsa-miR-28-5p_AAGGAGCTCACAGTCTATTGAG1	-0,704706603	3,384277878	0,212768655	0,832152253	LADA vs T1D
hsa-miR-223-5p_CGTGTATTTGACAAGCTGAGTTGG1	0,70627595	2,566442122	0,213620521	0,832152253	LADA vs T1D
hsa-miR-150-5p_CTCCCAACCCTTGTACCAGT1	0,683170652	3,181356832	0,216921726	0,832152253	LADA vs T1D
hsa-miR-10a-5p_TACCCTGTAGATCCGAATTTG1	0,18137429	8,855203262	0,216991315	0,832152253	LADA vs T1D
hsa-let-7i-5p_TGAGGTAGTAGTTTGTGCT1	-0,258422542	7,615350025	0,219624717	0,832152253	LADA vs T1D
hsa-let-7g-5p_TGAGGTAGTAGTTTGTACAGTT1	0,159002807	11,23391478	0,220051887	0,832152253	LADA vs T1D
hsa-miR-323a-3p_CACATTACACGGTCGACCTCT1	0,693504778	3,585538292	0,220435695	0,832152253	LADA vs T1D
hsa-let-7c-5p_TGAGGTAGTAGGTTGTATGGTT1	0,237294147	7,467576739	0,221166123	0,832152253	LADA vs T1D
hsa-miR-10a-5p_ACCCTGTAGATCCGAATTTGT1	0,261134152	7,643285241	0,221344441	0,832152253	LADA vs T1D
hsa-miR-4685-3p_TCTCCCTTCCTGCCCTGGCT1	0,546141634	4,862788515	0,221350411	0,832152253	LADA vs T1D
hsa-miR-451a_AAACCGTTACCATTACTGAGTTTAGT1	0,523724421	4,187049919	0,222939337	0,834190852	LADA vs T1D
hsa-miR-26b-5p_TTCAAGTAATTCAGGATAGGTT1	0,211163389	9,842825606	0,224931244	0,83771122	LADA vs T1D
hsa-miR-126-3p_CGTACCGTGAGTAATAATGCG1	0,172120279	9,683358758	0,228936747	0,838457847	LADA vs T1D
hsa-miR-16-5p_AGCAGCACGTAAATATTGGCG1	-0,189737677	8,17366975	0,230260645	0,838457847	LADA vs T1D
hsa-miR-21-5p_TAGCTTATCAGACTGATGT1	0,554569197	4,699515355	0,230743808	0,838457847	LADA vs T1D
hsa-miR-342-3p_TCTCACACAGAAATCGCACCC1	0,658933369	2,049205595	0,231248363	0,838457847	LADA vs T1D
hsa-miR-7-5p_TGGAAGACTAGTGATTTTGTTGTT1	0,643159415	4,415695214	0,231569873	0,838457847	LADA vs T1D
hsa-miR-182-5p_TTTGGCAATGGTAGAACT1	-0,671287838	3,421424723	0,231842785	0,838457847	LADA vs T1D
hsa-let-7g-5p_TGAGGTAGTAGTTTGTACAGTTT1	-0,308377545	6,811439564	0,23249584	0,838457847	LADA vs T1D
hsa-let-7d-3p_TATACGACCTGCTGCCTTTC1	0,293848627	6,429871038	0,235445201	0,843074891	LADA vs T1D
hsa-miR-30e-5p_GTAAACATCCTTGACTGGAAGC1	0,661947076	2,49990334	0,237317519	0,843074891	LADA vs T1D
hsa-miR-140-3p_ACCACAGGGTAGAACCACGGAC1	-0,392476083	5,852260421	0,23879204	0,843074891	LADA vs T1D
hsa-miR-30a-5p_TGTAAACATCCTCGACTGGA1	0,624480108	3,19956619	0,238877707	0,843074891	LADA vs T1D
hsa-miR-375-3p_TTTGTTCGTTCGGCTCGCGTG1	-0,391369345	6,919662337	0,240525026	0,843074891	LADA vs T1D
hsa-miR-140-3p_TACCACAGGGTAGAACCACGGA1	-0,239132037	7,894124989	0,241813163	0,843074891	LADA vs T1D

hsa-miR-126-3p_GTACCGTGAGTAATAATGCG1	0,634633095	2,067690175	0,242017178	0,843074891	LADA vs T1D
hsa-miR-140-5p_CAGTGGTTTTACCCTATGGTAG1	0,580209613	3,871751643	0,242238582	0,843074891	LADA vs T1D
hsa-let-7i-5p_GAGGTAGTAGTTTGTGCTGTT1	0,64564348	3,257044134	0,243992047	0,845485483	LADA vs T1D
hsa-miR-320a-3p_AAAAGCTGGGTTGAGAGGGCGAA1	-0,134348133	9,770949231	0,245396019	0,845661824	LADA vs T1D
hsa-miR-424-5p_CAGCAGCAATTCATGTTTTGA1	0,644030569	2,646439112	0,246165048	0,845661824	LADA vs T1D
hsa-miR-4433b-5p_ATGTCCCACCCCCACTCCTGTTT1	0,525499089	5,620502964	0,249164986	0,852293966	LADA vs T1D
hsa-miR-375-3p_TTTGTTCGTTCGGCTCGCGT1	0,624550981	3,894636907	0,255455163	0,856422295	LADA vs T1D
hsa-let-7d-3p_CTATACGACCTGCTGCCTTTCT1	0,124962273	11,76128234	0,25563807	0,856422295	LADA vs T1D
hsa-miR-23b-3p_ATCACATTGCCAGGGATTACCA1	0,327393695	6,460852955	0,257169354	0,856422295	LADA vs T1D
hsa-let-7a-5p_TGAGGTAGTAGGTTGTATAG1	-0,119777199	12,05467063	0,257543824	0,856422295	LADA vs T1D
hsa-miR-543_AAACATTCGCGGTGCACTTCTTT1	-0,69124169	3,173881718	0,258588427	0,856422295	LADA vs T1D
hsa-miR-150-5p_TCTCCCAACCCTTGTACCA1	0,374348639	6,588601483	0,260907298	0,856422295	LADA vs T1D
hsa-miR-93-5p_CAAAGTGCTGTTCGTGCAGGTAG1	-0,130503982	9,991178453	0,261279662	0,856422295	LADA vs T1D
hsa-miR-99a-5p_AACCCGTAGATCCGATCTT1	-0,62628267	3,048788798	0,261694683	0,856422295	LADA vs T1D
hsa-let-7a-5p_TGAGGTAGTAGGTTGTATAGTT1	0,153557504	13,39549393	0,262191886	0,856422295	LADA vs T1D
hsa-miR-221-3p_AGCTACATTGTCTGCTGGGTTTC1	0,341338493	6,752581432	0,262527614	0,856422295	LADA vs T1D
hsa-miR-409-3p_GAATGTTGCTCGGTGAACCCCTTT1	0,65389401	4,139609499	0,263127402	0,856422295	LADA vs T1D
hsa-miR-23a-3p_ATCACATTGCCAGGGATTTCCAA1	0,361779964	6,530990344	0,263266578	0,856422295	LADA vs T1D
hsa-miR-155-5p_TTAATGCTAATCGTGATAGGGGT1	0,544334594	3,972069578	0,269832885	0,873427269	LADA vs T1D
hsa-miR-18a-3p_ACTGCCCTAAGTGCTCCTTCTG1	-0,583211373	2,22678804	0,270685741	0,873427269	LADA vs T1D
hsa-miR-19b-3p_TGTGCAAATCCATGCAAAACTGA1	0,5077287	4,916784309	0,272843102	0,875948969	LADA vs T1D
hsa-miR-100-5p_AACCCGTAGATCCGAACTTGTG1	0,634912359	3,590895666	0,273665362	0,875948969	LADA vs T1D
hsa-let-7b-3p_CTATACAACCTACTGCCTTC1	0,573089418	3,159200497	0,277286511	0,883989397	LADA vs T1D
hsa-miR-103a-3p_AGCAGCATTGTACAGGGCTATGA1	0,22957751	8,631709381	0,279031835	0,884697343	LADA vs T1D
hsa-miR-320b_AAAAGCTGGGTTGAGAGGGCA1	-0,529209599	2,618155177	0,279728646	0,884697343	LADA vs T1D
hsa-miR-4433b-5p_TGTCCCACCCCCACTCCTG1	-0,651849667	2,497163245	0,281059522	0,88539304	LADA vs T1D
hsa-miR-532-5p_CATGCCTTGAGTGTAGGACCG1	0,559873285	2,388684445	0,284355821	0,890942561	LADA vs T1D
hsa-miR-501-3p_ATGCACCCGGGCAAGGATTCT1	0,601643071	2,081940648	0,285917301	0,890942561	LADA vs T1D
hsa-miR-361-5p_TTATCAGAATCTCCAGGGGTAC1	0,262162702	6,634693775	0,287330593	0,890942561	LADA vs T1D
hsa-miR-361-5p_TTATCAGAATCTCCAGGGG1	0,570950383	2,056970291	0,288553906	0,890942561	LADA vs T1D
hsa-miR-101-3p_TACAGTACTGTGATAACTGAAG1	-0,239179762	7,012156412	0,288602134	0,890942561	LADA vs T1D
hsa-miR-142-3p_TGTAGTGTTTCCTACTTTATGGA1	-0,581951043	3,252758122	0,289949768	0,890942561	LADA vs T1D
hsa-let-7b-5p_TGAGGTAGTAGGTTGTGTGGT1	-0,109721951	11,84645942	0,292467346	0,890942561	LADA vs T1D
hsa-miR-22-3p_AAGCTGCCAGTTGAAGAACTGTT1	-0,567188761	2,793276847	0,295049126	0,890942561	LADA vs T1D
hsa-miR-99b-5p_CACCCGTAGAACCGACCTTGC1	0,16043915	7,880557566	0,295861686	0,890942561	LADA vs T1D
hsa-miR-30e-5p_GTAAACATCCTTGACTGGAAGCT1	0,112667403	8,781643893	0,296824097	0,890942561	LADA vs T1D
hsa-miR-423-5p_GAGGGGCAGAGAGCGAGACTTT1	-0,130681932	8,388365906	0,297356258	0,890942561	LADA vs T1D
hsa-miR-484_TCAGGCTCAGTCCCCTCCCGATA1	-0,44790658	4,637634197	0,300545708	0,890942561	LADA vs T1D
hsa-miR-4732-5p_TGTAGAGCAGGGAGCAGGAAG1	0,582991098	3,155376397	0,301192682	0,890942561	LADA vs T1D
hsa-miR-485-5p_AGAGGCTGGCCGTGATGAATTCG1	0,552431428	4,543642396	0,30169985	0,890942561	LADA vs T1D
hsa-miR-629-5p_TGGGTTTACGTTGGGAGAACTT1	-0,507468187	4,034612462	0,302194365	0,890942561	LADA vs T1D
hsa-let-7a-5p_TGAGGTAGTAGGTTGTATAGT1	0,101313388	11,86713156	0,302386027	0,890942561	LADA vs T1D
hsa-miR-320a-3p_AAAGCTGGGTTGAGAGGGCGA1	-0,486396331	4,98279454	0,305449964	0,890942561	LADA vs T1D
hsa-let-7a-5p_TGAGGTAGTAGGTTGTATAGTTT1	0,215370464	9,423297293	0,307388692	0,890942561	LADA vs T1D
hsa-miR-27b-3p_TTCACAGTGGCTAAGTTCT1	0,183527056	8,299608545	0,307771951	0,890942561	LADA vs T1D
hsa-miR-3615_TCTCTCGGCTCCTCGCGGCTCG1	-0,136599956	8,091694617	0,3084473	0,890942561	LADA vs T1D
hsa-miR-101-3p_GTACAGTACTGTGATAACT1	-0,432464846	4,990449736	0,308516355	0,890942561	LADA vs T1D
hsa-miR-146a-5p_TGAGAACTGAATTCCATGGG1	0,533043993	2,424854764	0,309330274	0,890942561	LADA vs T1D
hsa-miR-125b-5p_TCCCTGAGACCCTAACTTGTG1	-0,427058482	5,039062696	0,309516137	0,890942561	LADA vs T1D
hsa-miR-128-3p_TCACAGTGAACCGGTCTCTT1	0,218597557	6,808413156	0,309676277	0,890942561	LADA vs T1D
hsa-miR-99b-5p_CACCCGTAGAACCGACCTT1	0,559458519	2,002435028	0,311275339	0,890942561	LADA vs T1D
hsa-miR-25-3p_CATTGCACTTGTCTCGGTCTG1	-0,172744701	9,990945876	0,31188579	0,890942561	LADA vs T1D
hsa-miR-28-3p_CACTAGATTGTGAGCTCCTGG1	0,315453937	5,876182682	0,314524582	0,893477291	LADA vs T1D
hsa-miR-183-5p_TATGGCACTGGTAGAATT1	0,57118758	2,805102899	0,315291928	0,893477291	LADA vs T1D
hsa-miR-451a_AAACCGTTACCATTACTGAGTTTAG1	0,545935753	2,164703683	0,316136256	0,893477291	LADA vs T1D
hsa-miR-140-3p_TACCACAGGGTAGAACCACGGACA1	-0,489577681	3,637619462	0,317938462	0,894465715	LADA vs T1D
hsa-miR-16-2-3p_CCAATATTACTGTGCTGCTTT1	0,443888869	4,35154905	0,319008096	0,894465715	LADA vs T1D
hsa-miR-363-3p_AATTGCACGGTATCCATCTGT1	0,146192355	8,977144446	0,319852859	0,894465715	LADA vs T1D
hsa-miR-328-3p_CTGGCCCTCTCTGCCCTTCCG1	-0,475232567	3,770890003	0,32131579	0,895414982	LADA vs T1D
hsa-miR-122-5p_TGGAGTGTGACAATGGTGTTTGT1	-0,304466912	8,198260953	0,324370991	0,896875172	LADA vs T1D
hsa-miR-25-3p_ATTGCACTTGTCTCGGTCTGA1	0,322388796	5,837958082	0,32446147	0,896875172	LADA vs T1D

hsa-miR-128-3p_TCACAGTGAACCGGTCTCTTT1	-0,129935327	8,228392837	0,325215715	0,896875172	LADA vs T1D
hsa-miR-10a-5p_TACCCTGTAGATCCGAATTTGTGT1	0,534404471	2,404757373	0,326542626	0,897429216	LADA vs T1D
hsa-miR-144-5p_GGATATCATCATATACTGTAA1	-0,533105812	3,901874288	0,328452344	0,899575663	LADA vs T1D
hsa-miR-199a-3p_ACAGTAGTCTGCACATTGGT1	-0,551623854	1,952185074	0,330896719	0,900882896	LADA vs T1D
hsa-miR-29b-3p_TAGCACCATTTGAAATCAGT1	-0,462499832	4,363760053	0,331964377	0,900882896	LADA vs T1D
hsa-miR-30c-5p_TGTAAACATCCTACACTCTCAGC1	0,26337948	6,545485648	0,332437834	0,900882896	LADA vs T1D
hsa-let-7d-3p_CTATACGACCTGCTGCCTTT1	0,123191529	10,24742095	0,334666772	0,900882896	LADA vs T1D
hsa-let-7b-5p_TGAGGTAGTAGGTTGTGTGGTT1	-0,08810141	12,77995871	0,336109238	0,900882896	LADA vs T1D
hsa-miR-320a-3p_AAAGCTGGGTTGAGAGGGCG1	-0,407430217	4,890613401	0,33734398	0,900882896	LADA vs T1D
hsa-miR-3615_TCTCTCGGCTCCTCGCGGCTC1	0,482858822	2,193732344	0,339072016	0,900882896	LADA vs T1D
hsa-miR-222-3p_AGCTACATCTGGCTACTGGGT1	-0,18570403	7,595221992	0,339798725	0,900882896	LADA vs T1D
hsa-miR-16-5p_TAGCAGCACGTAAATATTGGCG1	-0,104680365	14,03055319	0,340143655	0,900882896	LADA vs T1D
hsa-miR-92a-3p_GTATTGCACTTGTCCCGGCCTGT1	0,161418019	7,715362913	0,341965876	0,900882896	LADA vs T1D
hsa-miR-126-3p_CGTACCGTGAGTAATAATGC1	-0,401537964	4,46578231	0,344268061	0,900882896	LADA vs T1D
hsa-miR-484_CAGGCTCAGTCCCCTCCCGAT1	-0,486203321	2,404164158	0,34508526	0,900882896	LADA vs T1D
hsa-miR-451a_CGTTACCATTACTGAGT1	-0,450425095	4,012413349	0,345128221	0,900882896	LADA vs T1D
hsa-miR-320a-3p_AAAAGCTGGGTTGAGAGGGCGAAAA1	0,518703495	3,186668725	0,345754397	0,900882896	LADA vs T1D
hsa-miR-21-5p_TAGCTTATCAGACTGATGTTG1	0,105157112	11,59436943	0,346882601	0,900882896	LADA vs T1D
hsa-miR-193a-5p_TGGGTCTTTGCGGGCGAGAT1	0,412193277	4,669337288	0,350067895	0,900882896	LADA vs T1D
hsa-miR-106b-5p_TAAAGTGCTGACAGTGCAGA1	-0,36577566	5,144709005	0,350089191	0,900882896	LADA vs T1D
hsa-miR-15b-5p_TAGCAGCACATCATGGTTTA1	-0,309398549	5,794504936	0,350411316	0,900882896	LADA vs T1D
hsa-miR-16-2-3p_ACCAATATTACTGTGCTGCTTT1	-0,144332179	7,879139867	0,350513334	0,900882896	LADA vs T1D
hsa-miR-942-5p_TCTTCTCTGTTTTGGCCATGT1	0,518097491	2,592733465	0,351536488	0,900882896	LADA vs T1D
hsa-miR-502-3p_AATGCACCTGGGCAAGGATTCA1	0,379128236	4,709212787	0,352911216	0,901507177	LADA vs T1D
hsa-miR-505-3p_CGTCAACACTTGCTGGTTTCCT1	0,483935505	3,899410508	0,354148647	0,901777865	LADA vs T1D
hsa-miR-148b-3p_TCAGTGCATCACAGAACTTTGT1	0,182097774	6,965383646	0,357030777	0,903469337	LADA vs T1D
hsa-miR-223-3p_GTCAGTTTGTCAAATACCCCAA1	-0,243823477	7,740128632	0,357080102	0,903469337	LADA vs T1D
hsa-miR-186-5p_CAAAGAATTCTCCTTTTGGGCTTT1	-0,474290923	4,003798957	0,358642758	0,904551513	LADA vs T1D
hsa-miR-148b-3p_TCAGTGCATCACAGAACTTTG1	-0,390668665	4,882892663	0,360101165	0,905364759	LADA vs T1D
hsa-miR-183-5p_ATGGCACTGGTAGAATTCACTGT1	0,503809902	2,224937851	0,362470203	0,908455195	LADA vs T1D
hsa-miR-1306-5p_CCACCTCCCCTGCAAACGTCC1	0,414640562	4,76371819	0,365450083	0,913052402	LADA vs T1D
hsa-let-7f-5p_TGAGGTAGTAGATTGTAT1	0,529228999	3,174737293	0,368778615	0,918489238	LADA vs T1D
hsa-miR-486-5p_TCCTGTACTGAGCTGCCCCGAGG1	0,158286004	7,467002764	0,372477949	0,920817975	LADA vs T1D
hsa-let-7f-5p_TGAGGTAGTAGATTGTATAG1	-0,103919635	10,59314063	0,37354825	0,920817975	LADA vs T1D
hsa-miR-629-5p_TGGGTTTACGTTGGGAGAACT1	-0,205563861	7,034835335	0,37414143	0,920817975	LADA vs T1D
hsa-miR-25-3p_CATTGCACTTGTCTCGGTCTGA1	-0,127639801	11,99390817	0,374335036	0,920817975	LADA vs T1D
hsa-miR-532-3p_CCTCCCACACCCAAGGCTTG1	0,497933464	2,745637111	0,377574897	0,925929824	LADA vs T1D
hsa-miR-15b-5p_TAGCAGCACATCATGGTTTAC1	0,175678642	7,368236859	0,381945245	0,929999962	LADA vs T1D
hsa-miR-92a-3p_TGCACTTGTCCCGGCCTGT1	0,321979892	5,28105363	0,384120052	0,929999962	LADA vs T1D
hsa-miR-486-5p_GTACTGAGCTGCCCCGAG1	-0,393611827	4,883397866	0,384751986	0,929999962	LADA vs T1D
hsa-miR-423-3p_AGCTCGGTCTGAGGCCCCT1	0,266582086	6,038348398	0,385955789	0,929999962	LADA vs T1D
hsa-miR-182-5p_TTTGGCAATGGTAGAACTCACACTG1	0,487192768	3,598930548	0,389972741	0,929999962	LADA vs T1D
hsa-miR-22-3p_AAGCTGCCAGTTGAAGAAC1	0,15923867	8,203664635	0,390596084	0,929999962	LADA vs T1D
hsa-miR-629-5p_TGGGTTTACGTTGGGAGAAC1	-0,463066314	3,68380122	0,391235046	0,929999962	LADA vs T1D
hsa-miR-3615_TCTCTCGGCTCCTCGCGGCT1	-0,229004787	6,584121427	0,392368749	0,929999962	LADA vs T1D
hsa-miR-363-3p_AATTGCACGGTATCCATCTGTA1	0,425341246	3,912624597	0,392952578	0,929999962	LADA vs T1D
hsa-miR-30d-5p_GTAAACATCCCCGACTGGAAGC1	-0,365939115	4,874885211	0,394454812	0,929999962	LADA vs T1D
hsa-miR-30d-5p_TGTAAACATCCCCGACTGGAAG1	0,066935977	12,16128446	0,394748025	0,929999962	LADA vs T1D
hsa-miR-30c-5p_TGTAAACATCCTACACTCTCAGCT1	0,125103367	10,20131272	0,395889145	0,929999962	LADA vs T1D
hsa-miR-30e-5p_TGTAAACATCCTTGACTGGAA1	-0,180926704	6,536675988	0,398699701	0,929999962	LADA vs T1D
hsa-miR-142-5p_CCATAAAGTAGAAAGCACT1	0,306736351	4,774985235	0,399198535	0,929999962	LADA vs T1D
hsa-miR-133a-3p_TTGGTCCCCTTCAACCAGCTGT1	-0,504717086	2,527387136	0,399231821	0,929999962	LADA vs T1D
hsa-miR-146a-5p_TGAGAACTGAATTCCATGGGT1	0,111983239	8,67354952	0,400279714	0,929999962	LADA vs T1D
hsa-miR-423-3p_AAGCTCGGTCTGAGGCCCCTCA1	0,445639736	2,923364981	0,401556025	0,929999962	LADA vs T1D
hsa-miR-423-3p_AGCTCGGTCTGAGGCCCCTCA1	0,267631978	6,171912051	0,402833347	0,929999962	LADA vs T1D
hsa-miR-30e-5p_TGTAAACATCCTTGACTGGAAGCT1	-0,060487679	11,72480372	0,403813329	0,929999962	LADA vs T1D
hsa-miR-425-5p_ATGACACGATCACTCCCGTTG1	-0,460542129	2,398751928	0,404388536	0,929999962	LADA vs T1D
hsa-let-7a-5p_TGAGGTAGTAGGTTGTATAGTTTT1	0,478503209	3,044731157	0,406759393	0,929999962	LADA vs T1D
hsa-miR-543_AAACATTCGCGGTGCACTTCTT1	0,432881619	3,845833985	0,407239914	0,929999962	LADA vs T1D
hsa-miR-92a-3p_CACTTGTCCCGGCCTGT1	-0,419043596	4,072780053	0,407433435	0,929999962	LADA vs T1D
hsa-let-7c-5p_TGAGGTAGTAGGTTGTATGGT1	-0,298308286	4,585920454	0,407776902	0,929999962	LADA vs T1D

hsa-let-7a-5p_ATGAGGTAGTAGGTTGTATAGTT1	-0,475781169	2,103462699	0,408406508	0,929999962	LADA vs T1D
hsa-miR-181a-5p_AACATTCAACGCTGTCGGTGA1	-0,084489298	9,980124889	0,414556574	0,930683432	LADA vs T1D
hsa-miR-100-5p_AACCCGTAGATCCGAACT1	0,428701239	1,926738636	0,417602225	0,930683432	LADA vs T1D
hsa-miR-183-5p_TATGGCACTGGTAGAATTCA1	-0,225202783	7,32183424	0,418336293	0,930683432	LADA vs T1D
hsa-miR-186-5p_CAAAGAATTCTCCTTTTGGGCTT1	0,254742505	6,345307365	0,418782836	0,930683432	LADA vs T1D
hsa-miR-3615_TCTCTCGGCTCCTCGCGGC1	0,433793182	2,019798482	0,420235411	0,930683432	LADA vs T1D
hsa-miR-26a-5p_TTCAAGTAATCCAGGATAG1	-0,415109916	3,646262645	0,421160553	0,930683432	LADA vs T1D
hsa-miR-28-3p_CACTAGATTGTGAGCTCCTGGAG1	-0,427949623	2,845963244	0,42162405	0,930683432	LADA vs T1D
hsa-miR-323a-3p_GCACATTACACGGTCGACCTCT1	-0,435500521	3,90036256	0,421660797	0,930683432	LADA vs T1D
hsa-miR-486-5p_TCCTGTACTGAGCTGCCCCGAGC1	-0,173246691	6,188232343	0,423033083	0,930683432	LADA vs T1D
hsa-miR-29a-3p_TAGCACCATCTGAAATCGG1	-0,192211514	7,213630267	0,423339534	0,930683432	LADA vs T1D
hsa-miR-151a-3p_TACTAGACTGAAGCTCCTTGA1	0,418585096	1,940948476	0,427024805	0,930683432	LADA vs T1D
hsa-miR-24-3p_TGGCTCAGTTCAGCAGGAACAG1	-0,084503046	12,76835022	0,427187237	0,930683432	LADA vs T1D
hsa-miR-4433b-5p_ATGTCCCACCCCCACTCCTGT1	0,283703902	7,752662063	0,428998344	0,930683432	LADA vs T1D
hsa-miR-125a-5p_TCCCTGAGACCCTTTAACC1	0,41815753	2,158659604	0,429513779	0,930683432	LADA vs T1D
hsa-miR-584-5p_TTATGGTTTGCCTGGGACTGA1	0,155716988	8,070467342	0,431839495	0,930683432	LADA vs T1D
hsa-miR-451a_AAACCGTTACCATTACTGAGTT1	-0,115052689	12,14012514	0,434489076	0,930683432	LADA vs T1D
hsa-miR-339-3p_TGAGCGCCTCGACGACAGAG1	-0,314324942	5,154516456	0,436171884	0,930683432	LADA vs T1D
hsa-miR-92a-3p_TTGCACTTGTCCCGGCCTGT1	-0,10700165	8,384006303	0,436409323	0,930683432	LADA vs T1D
hsa-miR-30e-3p_CTTTCAGTCGGATGTTTACAG1	-0,419476042	2,31966353	0,441952457	0,930683432	LADA vs T1D
hsa-miR-222-3p_AGCTACATCTGGCTACTGGGTCT1	0,211895261	6,5045561	0,442548573	0,930683432	LADA vs T1D
hsa-miR-424-3p_CAAAACGTGAGGCGCTGCT1	-0,408914032	2,803316018	0,442668026	0,930683432	LADA vs T1D
hsa-miR-4732-5p_TGTAGAGCAGGGAGCAGGAAGCT1	-0,238556106	6,276959061	0,443251608	0,930683432	LADA vs T1D
hsa-miR-339-3p_TGAGCGCCTCGACGACAGAGC1	0,381106171	3,99309945	0,443379059	0,930683432	LADA vs T1D
hsa-miR-22-3p_AGCTGCCAGTTGAAGAACT1	0,430548508	2,67043052	0,448734217	0,930683432	LADA vs T1D
hsa-miR-23a-3p_TCACATTGCCAGGGATTTCC1	-0,358552997	4,540513631	0,449553979	0,930683432	LADA vs T1D
hsa-miR-1301-3p_TTGCAGCTGCCTGGGAGTGA1	-0,409599397	3,347749467	0,451755496	0,930683432	LADA vs T1D
hsa-miR-106b-3p_CCGCACTGTGGGTACTTGCTG1	-0,337018883	4,162035136	0,452015181	0,930683432	LADA vs T1D
hsa-miR-423-3p_AGCTCGGTCTGAGGCCCC1	-0,392049297	3,540860079	0,453186839	0,930683432	LADA vs T1D
hsa-miR-26a-5p_TTCAAGTAATCCAGGATAGGCT1	0,123439031	11,92893731	0,453505671	0,930683432	LADA vs T1D
hsa-miR-425-5p_AATGACACGATCACTCCCGTTGA1	-0,077029665	10,37803313	0,454079147	0,930683432	LADA vs T1D
hsa-miR-19a-3p_TGTGCAAATCTATGCAAAACTGA1	0,401201281	2,326203362	0,455462757	0,930683432	LADA vs T1D
hsa-miR-451a_AAACCGTTACCATTACTGAG1	0,118717821	13,72832534	0,458093346	0,930683432	LADA vs T1D
hsa-miR-424-3p_CAAAACGTGAGGCGCTGCTAT1	0,246562571	5,519904886	0,458615794	0,930683432	LADA vs T1D
hsa-miR-191-5p_CAACGGAATCCCAAAAGCAGCT1	0,091442602	11,11779618	0,45886859	0,930683432	LADA vs T1D
hsa-miR-181a-5p_AACATTCAACGCTGTCGGTGAGTT1	0,406300991	2,334562434	0,466841469	0,930683432	LADA vs T1D
hsa-miR-423-3p_AAGCTCGGTCTGAGGCCCCT1	0,411622147	2,46496229	0,467670755	0,930683432	LADA vs T1D
hsa-miR-155-5p_TTAATGCTAATCGTGATAGGGGTT1	0,415875668	2,993899159	0,467802813	0,930683432	LADA vs T1D
hsa-miR-4433b-5p_TATGTCCCACCCCCACTCCTG1	0,452774105	3,068037559	0,469855664	0,930683432	LADA vs T1D
hsa-miR-142-5p_CCCATAAAGTAGAAAGCACTA1	0,259600418	5,405299379	0,471022503	0,930683432	LADA vs T1D
hsa-miR-93-5p_CAAAGTGCTGTTCGTGCAGGTAGT1	0,373170546	4,129822813	0,473261374	0,930683432	LADA vs T1D
hsa-miR-486-5p_CCTGTACTGAGCTGCCCCGAG1	-0,106725513	11,29662351	0,47338461	0,930683432	LADA vs T1D
hsa-miR-126-5p_CATTATTACTTTTGGTACGC1	-0,365677168	3,620244945	0,473677331	0,930683432	LADA vs T1D
hsa-miR-150-5p_TCTCCCAACCCTTGTACCAGTG1	0,107637757	10,93881631	0,474046951	0,930683432	LADA vs T1D
hsa-miR-486-5p_CTGTACTGAGCTGCCCCGA1	0,138175686	7,583597035	0,47595387	0,930683432	LADA vs T1D
hsa-miR-451a_AAACCGTTACCATTACTGAGT1	0,102631868	16,51409079	0,479609084	0,930683432	LADA vs T1D
hsa-miR-423-3p_AGCTCGGTCTGAGGCCCCTCAGT1	-0,086822538	11,76682308	0,480149975	0,930683432	LADA vs T1D
hsa-miR-323b-3p_CCCAATACACGGTCGACCTCT1	-0,338518071	5,853793118	0,480591699	0,930683432	LADA vs T1D
hsa-miR-340-3p_TCCGTCTCAGTTACTTTATAGC1	0,366305374	1,820184636	0,480607607	0,930683432	LADA vs T1D
hsa-miR-17-5p_CAAAGTGCTTACAGTGCAGGTAG1	0,385237555	3,276961308	0,481335726	0,930683432	LADA vs T1D
hsa-miR-25-3p_CATTGCACTTGTCTCGGTC1	-0,242088166	5,77988375	0,482246065	0,930683432	LADA vs T1D
hsa-miR-122-5p_GGAGTGTGACAATGGTGTTTG1	-0,300665868	5,615777051	0,483285691	0,930683432	LADA vs T1D
hsa-miR-142-5p_CCCATAAAGTAGAAAGCACT1	0,06722738	11,13638127	0,483793347	0,930683432	LADA vs T1D
hsa-miR-451a_AACCGTTACCATTACTGAGTTT1	0,381431846	2,400911204	0,483848927	0,930683432	LADA vs T1D
hsa-miR-99b-5p_ACCCGTAGAACCGACCTTGCG1	0,381525013	2,082938158	0,484396896	0,930683432	LADA vs T1D
hsa-miR-4732-3p_GCCCTGACCTGTCCTGTTCTG1	-0,599651758	4,156168491	0,486291507	0,930683432	LADA vs T1D
hsa-miR-182-5p_TTTGGCAATGGTAGAACTCA1	-0,158299885	7,910992501	0,487489127	0,930683432	LADA vs T1D
hsa-miR-181b-5p_AACATTCATTGCTGTCGGTGG1	0,253145865	5,135854331	0,48753585	0,930683432	LADA vs T1D
hsa-miR-21-5p_TAGCTTATCAGACTGATGTTGA1	0,055929854	12,88530966	0,489931417	0,930683432	LADA vs T1D
hsa-miR-92b-3p_TATTGCACTCGTCCCGGCC1	0,359475358	3,837730088	0,490211902	0,930683432	LADA vs T1D
hsa-miR-150-5p_TCTCCCAACCCTTGTACCAG1	0,148451829	9,063580505	0,490714823	0,930683432	LADA vs T1D

hsa-miR-423-5p_AGGGGCAGAGAGCGAGACTTTT1	0,331056513	4,864713364	0,491923145	0,930683432	LADA vs T1D
hsa-miR-329-3p_AACACACCTGGTTAACCTCTTT1	0,37972137	3,759288959	0,492738912	0,930683432	LADA vs T1D
hsa-miR-140-3p_ACCACAGGGTAGAACCACG1	-0,369685193	2,665108795	0,494097373	0,930683432	LADA vs T1D
hsa-miR-98-5p_TGAGGTAGTAAGTTGTATTGT1	-0,215978227	6,55627531	0,494619452	0,930683432	LADA vs T1D
hsa-miR-125b-5p_TCCCTGAGACCCTAACTTG1	0,112132749	8,171400371	0,495141707	0,930683432	LADA vs T1D
hsa-miR-342-3p_TCTCACACAGAAATCGCACCCG1	-0,138849931	9,658047597	0,496448558	0,930683432	LADA vs T1D
hsa-miR-103a-3p_AGCAGCATTGTACAGGGC1	0,449894762	3,081760646	0,49755115	0,930683432	LADA vs T1D
hsa-miR-140-3p_ACCACAGGGTAGAACCACGGACA1	-0,321226376	4,192134912	0,498096285	0,930683432	LADA vs T1D
hsa-miR-425-5p_AATGACACGATCACTCCCGTT1	0,136987901	8,024407696	0,498627258	0,930683432	LADA vs T1D
hsa-miR-339-5p_TCCCTGTCCTCCAGGAGCTCACG1	0,375630762	3,416847552	0,500665452	0,930683432	LADA vs T1D
hsa-miR-125b-5p_TCCCTGAGACCCTAACTT1	-0,339936437	3,016083692	0,500692574	0,930683432	LADA vs T1D
hsa-miR-4433b-5p_ATGTCCCACCCCCACTCCTGTT1	0,249631769	8,226543503	0,500807953	0,930683432	LADA vs T1D
hsa-miR-151a-3p_TACTAGACTGAAGCTCCTTGAG1	0,360317195	3,055291554	0,501188383	0,930683432	LADA vs T1D
hsa-miR-144-5p_GGATATCATCATATACTGTAAGT1	-0,18672112	6,405140764	0,502789842	0,930683432	LADA vs T1D
hsa-miR-23a-5p_GGGGTTCCTGGGGATGGGATT1	0,354258079	3,585240207	0,503911581	0,930683432	LADA vs T1D
hsa-miR-146b-5p_TGAGAACTGAATTCCATAGGCTGT1	0,11236766	8,368012324	0,50408993	0,930683432	LADA vs T1D
hsa-miR-450b-5p_TTTTGCAATATGTTCCTGAAT1	-0,287633717	5,016886951	0,504917097	0,930683432	LADA vs T1D
hsa-miR-532-5p_CATGCCTTGAGTGTAGGACCGT1	-0,29725965	4,730944055	0,505513898	0,930683432	LADA vs T1D
hsa-miR-181a-2-3p_ACCACTGACCGTTGACTGTACC1	-0,379533328	2,397891497	0,505592392	0,930683432	LADA vs T1D
hsa-miR-221-3p_AGCTACATTGTCTGCTGGGTT1	-0,206580565	6,683307876	0,506024902	0,930683432	LADA vs T1D
hsa-miR-409-3p_CGAATGTTGCTCGGTGAACCCCT1	-0,333429545	5,738161577	0,506512513	0,930683432	LADA vs T1D
hsa-let-7g-5p_GAGGTAGTAGTTTGTACAG1	0,373740903	2,076526463	0,50725028	0,930683432	LADA vs T1D
hsa-miR-223-3p_TCAGTTTGTCAAATACCCCAA1	-0,354980743	2,060154898	0,507316366	0,930683432	LADA vs T1D
hsa-miR-183-5p_TATGGCACTGGTAGAATTCACTG1	-0,374638921	3,516695948	0,507667702	0,930683432	LADA vs T1D
hsa-miR-1180-3p_TTTCCGGCTCGCGTGGGTGTGT1	-0,3454766	2,874816557	0,507963981	0,930683432	LADA vs T1D
hsa-miR-27b-3p_TTCACAGTGGCTAAGTTCTGC1	-0,119662771	7,903620851	0,510648128	0,933455409	LADA vs T1D
hsa-miR-342-3p_TCACACAGAAATCGCACCCGTCA1	0,34591257	3,930198822	0,512765957	0,935088278	LADA vs T1D
hsa-miR-382-5p_AAGTTGTTCGTGGTGGATTCG1	0,389770973	2,227150711	0,514100549	0,935088278	LADA vs T1D
hsa-miR-1-3p_TGGAATGTAAAGAAGTATGTAT1	0,327934091	5,995923161	0,51628742	0,935088278	LADA vs T1D
hsa-miR-10b-5p_ACCCTGTAGAACCGAATTTGTGT1	0,334016481	3,356457419	0,516816206	0,935088278	LADA vs T1D
hsa-miR-1301-3p_TTGCAGCTGCCTGGGAGTGACTTC1	-0,376245835	2,697320641	0,517407692	0,935088278	LADA vs T1D
hsa-miR-191-5p_AACGGAATCCCAAAAGCAGCTG1	0,206249826	5,747755673	0,519621858	0,936965205	LADA vs T1D
hsa-miR-486-3p_CGGGGCAGCTCAGTACAGGAT1	-0,151107654	7,803889597	0,521507867	0,938243274	LADA vs T1D
hsa-miR-16-5p_TTAGCAGCACGTAAATATTGGCG1	0,308584726	4,199452303	0,523658295	0,939079318	LADA vs T1D
hsa-miR-191-5p_CAACGGAATCCCAAAAGC1	0,344657765	3,190312182	0,524329105	0,939079318	LADA vs T1D
hsa-miR-150-5p_GTCTCCCAACCCTTGTACCAGTG1	0,355756305	2,975164068	0,527444885	0,942541644	LADA vs T1D
hsa-miR-423-5p_TGAGGGGCAGAGAGCGAGACT1	-0,072187571	13,29479943	0,532782602	0,94291225	LADA vs T1D
hsa-miR-145-3p_ATTCCTGGAAATACTGTTCT1	-0,336546111	3,209907444	0,534379465	0,94291225	LADA vs T1D
hsa-miR-30d-5p_GTAAACATCCCCGACTGGAAGCT1	-0,154614648	6,998439264	0,535974974	0,94291225	LADA vs T1D
hsa-miR-186-5p_CAAAGAATTCTCCTTTTGGGCT1	0,077199038	9,032677455	0,536223847	0,94291225	LADA vs T1D
hsa-miR-93-5p_CAAAGTGCTGTTCGTGCAGGT1	0,227453549	4,918359962	0,536411661	0,94291225	LADA vs T1D
hsa-miR-142-5p_CCCATAAAGTAGAAAGCACTAC1	0,145682049	7,021131164	0,537059467	0,94291225	LADA vs T1D
hsa-miR-29a-3p_TAGCACCATCTGAAATCGGTTA1	-0,280474671	4,716466347	0,53706389	0,94291225	LADA vs T1D
hsa-miR-92a-3p_TATTGCACTTGTCCCGGCC1	-0,12306856	8,088512139	0,540978734	0,94291225	LADA vs T1D
hsa-miR-23a-3p_ATCACATTGCCAGGGATTTC1	-0,086380127	9,459376505	0,541637285	0,94291225	LADA vs T1D
hsa-let-7d-5p_AGAGGTAGTAGGTTGCATAGTT1	0,11981895	9,075101282	0,544323754	0,94291225	LADA vs T1D
hsa-miR-501-3p_AATGCACCCGGGCAAGGATTC1	0,323992326	3,102124992	0,545177262	0,94291225	LADA vs T1D
hsa-miR-143-3p_TGAGATGAAGCACTGTAGCT1	-0,101274872	9,047255511	0,545211663	0,94291225	LADA vs T1D
hsa-miR-101-3p_TACAGTACTGTGATAACTGA1	-0,302861367	4,333316093	0,545296988	0,94291225	LADA vs T1D
hsa-miR-185-5p_TGGAGAGAAAGGCAGTTCCTG1	0,291695809	4,508281621	0,547119393	0,94291225	LADA vs T1D
hsa-miR-103a-3p_AGCAGCATTGTACAGGGCT1	0,497918921	5,118424827	0,548178825	0,94291225	LADA vs T1D
hsa-miR-148a-3p_TCAGTGCACTACAGAACTTT1	0,31861139	4,280926656	0,548986851	0,94291225	LADA vs T1D
hsa-miR-6803-3p_TCCCTCGCCTTCTCACCCTCAG1	-0,316954673	2,710817974	0,549751619	0,94291225	LADA vs T1D
hsa-miR-423-5p_TGAGGGGCAGAGAGCGAGA1	-0,067136128	9,723922848	0,549957517	0,94291225	LADA vs T1D
hsa-miR-194-5p_TGTAACAGCAACTCCATGTGGAA1	-0,315344482	2,659899918	0,550130736	0,94291225	LADA vs T1D
hsa-miR-92a-3p_GCACTTGTCCCGGCCTGT1	-0,246488496	4,476222162	0,555593373	0,946163988	LADA vs T1D
hsa-miR-199a-5p_CCCAGTGTTCAGACTACCTGTTC1	-0,281187256	4,567460238	0,555618758	0,946163988	LADA vs T1D
hsa-miR-24-3p_GGCTCAGTTCAGCAGGAACAG1	-0,133638954	6,812383366	0,557578195	0,946163988	LADA vs T1D
hsa-miR-15b-5p_TAGCAGCACATCATGGTTT1	-0,09776352	8,514548835	0,557941947	0,946163988	LADA vs T1D
hsa-miR-26b-5p_TTCAAGTAATTCAGGATAGGT1	-0,114000336	7,343481575	0,557963707	0,946163988	LADA vs T1D
hsa-let-7i-5p_TGAGGTAGTAGTTTGTGCTGT1	-0,064769906	11,39282357	0,559452154	0,946673815	LADA vs T1D

hsa-miR-30e-5p_TGTAAACATCCTTGACTGGAAGC1	0,068183506	8,385302075	0,563550957	0,951027278	LADA vs T1D
hsa-let-7a-5p_TGAGGTAGTAGGTTGTATA1	-0,194947277	5,663865648	0,566815271	0,951027278	LADA vs T1D
hsa-miR-370-3p_GCCTGCTGGGGTGGAACCTGGT1	0,340295399	2,498088509	0,568963395	0,951027278	LADA vs T1D
hsa-miR-150-5p_TCTCCCAACCCTTGTACCAGT1	0,100331265	10,25222562	0,570532904	0,951027278	LADA vs T1D
hsa-miR-320a-3p_AAAAGCTGGGTTGAGAGGGCGAAA1	-0,156816736	6,744969015	0,571146227	0,951027278	LADA vs T1D
hsa-miR-486-5p_CTGTACTGAGCTGCCCCGAG1	-0,114327725	7,787642307	0,572446714	0,951027278	LADA vs T1D
hsa-miR-486-5p_ATCCTGTACTGAGCTGCCCCG1	-0,155984171	6,350028468	0,572528067	0,951027278	LADA vs T1D
hsa-miR-29a-3p_TAGCACCATCTGAAATCGGT1	-0,103588141	8,663780214	0,5727272	0,951027278	LADA vs T1D
hsa-miR-7-5p_TGGAAGACTAGTGATTTTGTTG1	0,265542996	3,890826404	0,572764233	0,951027278	LADA vs T1D
hsa-miR-374a-5p_TTATAATACAACCTGATAAGT1	0,309616652	2,487762486	0,575037981	0,95281761	LADA vs T1D
hsa-let-7d-3p_TATACGACCTGCTGCCTTT1	0,143073181	6,275229414	0,578695822	0,953016185	LADA vs T1D
hsa-miR-21-5p_TAGCTTATCAGACTGATGTT1	0,109235923	7,170728426	0,579196972	0,953016185	LADA vs T1D
hsa-let-7g-5p_GAGGTAGTAGTTTGTACAGTT1	-0,305179512	4,344091711	0,581964789	0,953016185	LADA vs T1D
hsa-miR-16-5p_TAGCAGCACGTAAATATTGG1	-0,072154632	10,97240393	0,583637359	0,953016185	LADA vs T1D
hsa-miR-132-3p_TAACAGTCTACAGCCATGGTCG1	0,297006053	2,233528136	0,585223006	0,953016185	LADA vs T1D
hsa-miR-320a-3p_AAAAGCTGGGTTGAGAGGGCG1	-0,06575849	10,00052492	0,585699067	0,953016185	LADA vs T1D
hsa-miR-22-3p_AAGCTGCCAGTTGAAGAA1	0,253851854	4,931866064	0,587905456	0,953016185	LADA vs T1D
hsa-miR-6803-3p_TCCCTCGCCTTCTCACCCTCA1	-0,171145475	6,298415012	0,588825956	0,953016185	LADA vs T1D
hsa-miR-423-5p_AGGGGCAGAGAGCGAGACT1	0,301902362	3,04923014	0,590710205	0,953016185	LADA vs T1D
hsa-miR-16-2-3p_ACCAATATTACTGTGCTGCTT1	-0,085432731	8,312552057	0,591298625	0,953016185	LADA vs T1D
hsa-miR-192-5p_TGACCTATGAATTGACAGCCA1	-0,251707209	4,726203979	0,592578496	0,953016185	LADA vs T1D
hsa-miR-182-5p_TTTGGCAATGGTAGAACTCACACTGG1	0,308826106	2,315372824	0,593618434	0,953016185	LADA vs T1D
hsa-miR-23a-3p_ATCACATTGCCAGGGATTT1	0,09146932	7,810237088	0,593696389	0,953016185	LADA vs T1D
hsa-miR-24-3p_TGGCTCAGTTCAGCAGGAA1	0,206161661	5,648746767	0,594697186	0,953016185	LADA vs T1D
hsa-miR-151a-3p_CTAGACTGAAGCTCCTTGAGGA1	0,092845137	8,444952042	0,595182042	0,953016185	LADA vs T1D
hsa-miR-140-3p_TACCACAGGGTAGAACCACGGAC1	-0,282633087	3,947173881	0,596027956	0,953016185	LADA vs T1D
hsa-let-7a-5p_GAGGTAGTAGGTTGTATAG1	0,206553873	5,403501302	0,596891763	0,953016185	LADA vs T1D
hsa-miR-574-3p_CACGCTCATGCACACACCCA1	-0,282219261	3,365756558	0,597125215	0,953016185	LADA vs T1D
hsa-miR-192-5p_TGACCTATGAATTGACAGCCAGT1	0,273680673	3,322199511	0,597877155	0,953016185	LADA vs T1D
hsa-miR-142-5p_CATAAAGTAGAAAGCACTAC1	0,289146844	2,376484394	0,599562386	0,953053367	LADA vs T1D
hsa-let-7d-5p_AGAGGTAGTAGGTTGCATAGT1	0,123505629	7,455921597	0,600292083	0,953053367	LADA vs T1D
hsa-let-7a-5p_GAGGTAGTAGGTTGTATAGT1	0,239418627	4,701370889	0,605314405	0,954321045	LADA vs T1D
hsa-miR-484_TCAGGCTCAGTCCCCTCCCG1	-0,122629606	7,780454359	0,605330911	0,954321045	LADA vs T1D
hsa-miR-451a_CCGTTACCATTACTGAGT1	-0,247223059	4,189894806	0,606847929	0,954321045	LADA vs T1D
hsa-miR-103a-3p_AGCAGCATTGTACAGGGCTAT1	0,346030548	3,428123511	0,607098938	0,954321045	LADA vs T1D
hsa-miR-92a-3p_ATTGCACTTGTCCCGGCCTG1	-0,081886715	9,121941427	0,607140264	0,954321045	LADA vs T1D
hsa-miR-483-3p_TCACTCCTCTCCTCCCGTCT1	-0,302656425	2,217026305	0,609010229	0,954321045	LADA vs T1D
hsa-miR-146a-5p_TGAGAACTGAATTCCATGGGTTG1	0,103425457	7,878355546	0,609915013	0,954321045	LADA vs T1D
hsa-miR-423-5p_TGAGGGGCAGAGAGCGAGACTTTT1	-0,054388967	11,39034963	0,610669677	0,954321045	LADA vs T1D
hsa-miR-15a-5p_TAGCAGCACATAATGGTTTGT1	0,152280084	6,478410353	0,612338102	0,955055709	LADA vs T1D
hsa-miR-363-3p_ATTGCACGGTATCCATCTG1	0,271709343	2,060471037	0,619292043	0,964015153	LADA vs T1D
hsa-miR-486-5p_ATCCTGTACTGAGCTGCCCCGA1	-0,08378168	8,988899635	0,621552938	0,965648521	LADA vs T1D
hsa-miR-223-3p_TGTCAGTTTGTCAAATACCCCA1	-0,086176544	10,41019198	0,623585223	0,966921056	LADA vs T1D
hsa-miR-24-3p_GGCTCAGTTCAGCAGGAAC1	0,295111259	2,859699768	0,624838535	0,966983131	LADA vs T1D
hsa-miR-26a-5p_TCAAGTAATCCAGGATAGGCT1	0,140453488	6,522605962	0,628998829	0,971535012	LADA vs T1D
hsa-let-7b-5p_TGAGGTAGTAGGTTGTGTGG1	0,050341073	12,43476497	0,632805082	0,973277381	LADA vs T1D
hsa-miR-451a_ACCGTTACCATTACTGAGT1	0,114059645	7,452815255	0,635287635	0,973277381	LADA vs T1D
hsa-miR-222-3p_AGCTACATCTGGCTACTGGGTC1	0,237416125	3,362212617	0,636554535	0,973277381	LADA vs T1D
hsa-miR-181a-2-3p_ACCACTGACCGTTGACTGT1	0,255476006	2,531276889	0,637540524	0,973277381	LADA vs T1D
hsa-miR-654-5p_TGGTGGGCCGCAGAACATGTGC1	-0,296728505	3,027916369	0,637924021	0,973277381	LADA vs T1D
hsa-miR-32-5p_TATTGCACATTACTAAGTTG1	-0,241852747	3,984130302	0,638911676	0,973277381	LADA vs T1D
hsa-miR-92b-3p_TATTGCACTCGTCCCGGCCT1	-0,176116213	5,68469958	0,638940928	0,973277381	LADA vs T1D
hsa-miR-23a-3p_ATCACATTGCCAGGGATTTCC1	-0,048022235	11,08225248	0,64109275	0,973277381	LADA vs T1D
hsa-let-7c-5p_TGAGGTAGTAGGTTGTATGGTTT1	-0,230196864	3,566946574	0,641117471	0,973277381	LADA vs T1D
hsa-miR-342-3p_TCTCACACAGAAATCGCACCCGT1	0,087731471	8,706959622	0,642381749	0,973342688	LADA vs T1D
hsa-miR-146b-5p_TGAGAACTGAATTCCATAGGCTG1	-0,165676109	5,765289472	0,644736147	0,97353355	LADA vs T1D
hsa-miR-140-3p_TACCACAGGGTAGAACCACG1	0,172608505	5,026346909	0,645649373	0,97353355	LADA vs T1D
hsa-miR-423-3p_AAGCTCGGTCTGAGGCCCCTCAGT1	0,109749376	7,286433576	0,646432515	0,97353355	LADA vs T1D
hsa-miR-320b_AAAAGCTGGGTTGAGAGGGCAA1	0,229529361	1,963551094	0,648335985	0,97353355	LADA vs T1D
hsa-miR-30d-5p_GTAAACATCCCCGACTGGAAG1	-0,14672994	5,727486807	0,648615201	0,97353355	LADA vs T1D
hsa-miR-6803-3p_TCCCTCGCCTTCTCACCCTC1	-0,243628362	2,004774634	0,653172421	0,975269802	LADA vs T1D

hsa-miR-1306-5p_CCACCTCCCCTGCAAACGTCCA1	0,257432903	3,283279778	0,654489156	0,975269802	LADA vs T1D
hsa-miR-652-3p_AATGGCGCCACTAGGGTTG1	0,233819445	4,499546053	0,655179732	0,975269802	LADA vs T1D
hsa-miR-26b-5p_TCAAGTAATTCAGGATAGGTT1	0,237319132	3,747955651	0,655780928	0,975269802	LADA vs T1D
hsa-miR-99a-5p_AACCCGTAGATCCGATCTTG1	0,079386137	8,041845005	0,657194523	0,975269802	LADA vs T1D
hsa-miR-494-3p_TGAAACATACACGGGAAACCTCT1	-0,235951439	4,269868739	0,659161564	0,975269802	LADA vs T1D
hsa-miR-4433b-5p_ATGTCCCACCCCCACTCCTG1	0,23881981	2,006165102	0,659463088	0,975269802	LADA vs T1D
hsa-miR-92a-3p_TATTGCACTTGTCCCGGCCTGTT1	0,053292409	10,41769689	0,659561384	0,975269802	LADA vs T1D
hsa-miR-186-5p_CAAAGAATTCTCCTTTTGGGC1	0,12155867	6,535812742	0,663062731	0,977726158	LADA vs T1D
hsa-miR-505-3p_GTCAACACTTGCTGGTTTCCTCT1	0,248805014	2,066469778	0,666586085	0,977726158	LADA vs T1D
hsa-miR-192-5p_CTGACCTATGAATTGACAGC1	-0,241596212	1,977431527	0,666735663	0,977726158	LADA vs T1D
hsa-miR-2110_TTGGGGAAACGGCCGCTGAGTG1	0,250608914	2,932814217	0,667107007	0,977726158	LADA vs T1D
hsa-miR-92a-3p_TATTGCACTTGTCCCGGCCTGTG1	-0,069117146	7,105169059	0,668556547	0,977726158	LADA vs T1D
hsa-miR-142-5p_CATAAAGTAGAAAGCACTACT1	0,213352346	3,650597591	0,669131998	0,977726158	LADA vs T1D
hsa-miR-30e-5p_TGTAAACATCCTTGACTGG1	0,185924958	5,076073317	0,672998918	0,977726158	LADA vs T1D
hsa-let-7d-3p_CTATACGACCTGCTGCCTTTC1	0,042283849	10,32598693	0,673605609	0,977726158	LADA vs T1D
hsa-miR-222-3p_AGCTACATCTGGCTACTGGG1	0,234951767	2,549442498	0,674948411	0,977726158	LADA vs T1D
hsa-miR-122-5p_GAGTGTGACAATGGTGTTT1	0,251909539	2,929528654	0,676716	0,977726158	LADA vs T1D
hsa-miR-423-5p_TGAGGGGCAGAGAGCGAGACTT1	-0,038374941	12,19565674	0,676900494	0,977726158	LADA vs T1D
hsa-miR-3173-5p_TGCCCTGCCTGTTTTCTCCTTT1	-0,174949417	4,766192857	0,678502036	0,977726158	LADA vs T1D
hsa-miR-101-3p_TACAGTACTGTGATAACTGAA1	0,228823317	3,070531279	0,679150268	0,977726158	LADA vs T1D
hsa-miR-92a-3p_TATTGCACTTGTCCCGGCCTG1	-0,055869094	13,95782504	0,684985232	0,977726158	LADA vs T1D
hsa-miR-486-5p_CCTGTACTGAGCTGCCCCG1	0,083855494	8,015321722	0,685270484	0,977726158	LADA vs T1D
hsa-miR-181a-5p_AACATTCAACGCTGTCGGTG1	-0,098793581	6,983532365	0,685538306	0,977726158	LADA vs T1D
hsa-miR-197-3p_TTCACCACCTTCTCCACCCAG1	0,070463559	8,762482756	0,685544402	0,977726158	LADA vs T1D
hsa-miR-92a-3p_TTGCACTTGTCCCGGCCTG1	0,191313807	4,313701526	0,685651721	0,977726158	LADA vs T1D
hsa-miR-409-3p_AATGTTGCTCGGTGAACCCCT1	0,227390963	4,366315065	0,687361483	0,977726158	LADA vs T1D
hsa-miR-320a-3p_AAAAGCTGGGTTGAGAGGGCGA1	-0,04224722	10,74836293	0,68755334	0,977726158	LADA vs T1D
hsa-miR-99a-5p_AACCCGTAGATCCGATCTTGTG1	0,144425928	5,156061862	0,688778564	0,977726158	LADA vs T1D
hsa-miR-140-3p_ACCACAGGGTAGAACCACGG1	0,203177762	3,491351538	0,689112373	0,977726158	LADA vs T1D
hsa-miR-423-3p_GCTCGGTCTGAGGCCCCTCAGT1	-0,121471217	6,432183419	0,690629909	0,977726158	LADA vs T1D
hsa-miR-3613-5p_TGTTGTACTTTTTTTTTTGTTC1	0,172249533	4,388236924	0,692555517	0,977726158	LADA vs T1D
hsa-miR-409-3p_GAATGTTGCTCGGTGAACCCCTT1	-0,217766814	5,101558557	0,692743216	0,977726158	LADA vs T1D
hsa-miR-21-5p_GTAGCTTATCAGACTGATGTTGA1	-0,220984078	2,217922154	0,69473667	0,977726158	LADA vs T1D
hsa-miR-197-3p_TTCACCACCTTCTCCACCCAGC1	-0,060007205	10,1606215	0,697171863	0,977726158	LADA vs T1D
hsa-miR-122-5p_TGGAGTGTGACAATGGTGTTT1	-0,100952667	13,55430102	0,697993672	0,977726158	LADA vs T1D
hsa-miR-223-3p_TGTCAGTTTGTCAAATACCCCAA1	-0,072141324	10,91791178	0,698957641	0,977726158	LADA vs T1D
hsa-miR-4433b-5p_TATGTCCCACCCCCACTCCTGT1	-0,183477926	5,397905397	0,699540047	0,977726158	LADA vs T1D
hsa-miR-223-5p_CGTGTATTTGACAAGCTGAGTTG1	0,128706033	6,07891061	0,700627289	0,977726158	LADA vs T1D
hsa-miR-92a-3p_TATTGCACTTGTCCCGGCCT1	-0,056300529	10,27945719	0,701174065	0,977726158	LADA vs T1D
hsa-miR-21-5p_TAGCTTATCAGACTGATGTTGACT1	0,169211004	5,943834972	0,70265386	0,977726158	LADA vs T1D
hsa-miR-483-5p_AAGACGGGAGGAAAGAAGGGA1	-0,202891802	4,456894738	0,703836571	0,977726158	LADA vs T1D
hsa-miR-25-3p_CATTGCACTTGTCTCGGTCT1	-0,054296977	10,26006574	0,708038786	0,977726158	LADA vs T1D
hsa-let-7f-5p_TGAGGTAGTAGATTG1	0,194035793	4,164688749	0,708486758	0,977726158	LADA vs T1D
hsa-miR-191-5p_CAACGGAATCCCAAAAGCAGCTG1	-0,0657633	9,658129048	0,709719859	0,977726158	LADA vs T1D
hsa-miR-361-3p_TCCCCCAGGTGTGATTCTGA1	-0,192267568	3,113214743	0,709954305	0,977726158	LADA vs T1D
hsa-miR-224-5p_CAAGTCACTAGTGGTTCCGTTT1	0,2060082	2,885758309	0,709988091	0,977726158	LADA vs T1D
hsa-miR-589-5p_TGAGAACCACGTCTGCTCTGA1	0,203136409	2,896824842	0,710292905	0,977726158	LADA vs T1D
hsa-miR-451a_ACCGTTACCATTACTGAGTT1	-0,197789009	2,004581894	0,713775776	0,980826368	LADA vs T1D
hsa-miR-146a-5p_TGAGAACTGAATTCCATGGGTTGT1	0,103227259	7,265118969	0,716305549	0,982608473	LADA vs T1D
hsa-miR-101-3p_GTACAGTACTGTGATAACTGA1	-0,054251916	8,248925167	0,718766377	0,982866717	LADA vs T1D
hsa-miR-193a-5p_TGGGTCTTTGCGGGCGAGATG1	0,136661967	5,884704134	0,718960221	0,982866717	LADA vs T1D
hsa-miR-335-3p_TTTTTCATTATTGCTCCTGACC1	-0,19111511	4,076732498	0,723628731	0,983409016	LADA vs T1D
hsa-miR-181a-2-3p_ACCACTGACCGTTGACTGTAC1	0,184990317	3,544826687	0,725153279	0,983409016	LADA vs T1D
hsa-miR-374a-5p_TTATAATACAACCTGATAAGTG1	-0,154783381	4,272038394	0,725366909	0,983409016	LADA vs T1D
hsa-miR-21-5p_AGCTTATCAGACTGATGTTGAC1	0,205563495	3,189772648	0,726409616	0,983409016	LADA vs T1D
hsa-miR-3613-5p_TGTTGTACTTTTTTTTTTGT1	-0,055119965	7,586394808	0,7272204	0,983409016	LADA vs T1D
hsa-miR-1294_TGTGAGGTTGGCATTGTTGT1	0,190863329	2,481086687	0,727920609	0,983409016	LADA vs T1D
hsa-let-7i-5p_TGAGGTAGTAGTTTGTGCTGTTG1	-0,178323713	3,894577335	0,727994127	0,983409016	LADA vs T1D
hsa-miR-335-5p_TCAAGAGCAATAACGAAAAAT1	0,102171353	6,715048961	0,734731906	0,984299213	LADA vs T1D
hsa-miR-92a-3p_TATTGCACTTGTCCCGGC1	0,087487151	6,608463448	0,735366677	0,984299213	LADA vs T1D
hsa-let-7f-5p_GAGGTAGTAGATTGTATAGTT1	0,117097224	6,051011881	0,735936009	0,984299213	LADA vs T1D

hsa-miR-3173-5p_CCCTGCCTGTTTTCTCCTTTGT1	0,170272989	1,879782778	0,738344864	0,984299213	LADA vs T1D
hsa-miR-199a-3p_ACAGTAGTCTGCACATTGGTTA1	-0,277430655	4,827856003	0,739876539	0,984299213	LADA vs T1D
hsa-miR-92a-3p_ATTGCACTTGTCCCGGCCT1	-0,160737787	4,477303001	0,740730694	0,984299213	LADA vs T1D
hsa-miR-423-5p_TGAGGGGCAGAGAGCGAGACTTT1	0,022923847	14,42674435	0,741020829	0,984299213	LADA vs T1D
hsa-miR-485-5p_AGAGGCTGGCCGTGATGAATTC1	-0,198562474	2,300221452	0,741256637	0,984299213	LADA vs T1D
hsa-miR-425-5p_AATGACACGATCACTCCCGT1	-0,081474832	6,738341736	0,74126049	0,984299213	LADA vs T1D
hsa-miR-363-3p_AATTGCACGGTATCCATCTG1	-0,055604861	8,264741807	0,741957082	0,984299213	LADA vs T1D
hsa-miR-143-3p_TGAGATGAAGCACTGTAGCTCA1	0,094741132	6,738882834	0,742238177	0,984299213	LADA vs T1D
hsa-miR-144-5p_GGATATCATCATATACTGTAAG1	0,182967081	4,224705339	0,744544193	0,985717146	LADA vs T1D
hsa-miR-92a-3p_TATTGCACTTGTCCCGGCCTGT1	-0,02982226	17,23184948	0,751934287	0,987488947	LADA vs T1D
hsa-miR-486-5p_TCCTGTACTGAGCTGCCCCGAG1	-0,04149098	17,24661108	0,753284556	0,987488947	LADA vs T1D
hsa-miR-320a-3p_GAAAAGCTGGGTTGAGAGGGCGA1	-0,160320233	3,456353792	0,758065518	0,987488947	LADA vs T1D
hsa-miR-574-3p_CACGCTCATGCACACACCCAC1	-0,145353533	4,117697394	0,758575059	0,987488947	LADA vs T1D
hsa-miR-182-5p_TTTGGCAATGGTAGAACTC1	-0,174031409	2,382812326	0,759346029	0,987488947	LADA vs T1D
hsa-miR-183-5p_ATGGCACTGGTAGAATTCACT1	-0,143343424	4,652978697	0,759353298	0,987488947	LADA vs T1D
hsa-miR-125b-5p_TCCCTGAGACCCTAACT1	-0,181906245	2,233733889	0,76299246	0,987488947	LADA vs T1D
hsa-miR-16-2-3p_ACCAATATTACTGTGCTGCT1	0,134460682	4,606374691	0,763378439	0,987488947	LADA vs T1D
hsa-miR-451a_AACCGTTACCATTACTGAG1	0,055249129	8,074719646	0,764102232	0,987488947	LADA vs T1D
hsa-miR-21-3p_CAACACCAGTCGATGGGCTGT1	-0,153454752	2,296740898	0,764608912	0,987488947	LADA vs T1D
hsa-miR-361-5p_TTATCAGAATCTCCAGGGGTA1	-0,158900863	3,298750595	0,765063744	0,987488947	LADA vs T1D
hsa-miR-191-5p_ACGGAATCCCAAAAGCAGCT1	0,164285226	1,904502114	0,766087342	0,987488947	LADA vs T1D
hsa-miR-487b-3p_TCGTACAGGGTCATCCACTTT1	0,176814796	2,776617041	0,766149228	0,987488947	LADA vs T1D
hsa-miR-199a-3p_ACAGTAGTCTGCACATTGGTT1	0,254654189	4,64378834	0,766603643	0,987488947	LADA vs T1D
hsa-let-7d-5p_AGAGGTAGTAGGTTGCATAGTTT1	0,137693539	5,420694347	0,7678137	0,987488947	LADA vs T1D
hsa-miR-28-3p_ACTAGATTGTGAGCTCCTGGAG1	0,133121139	4,615150746	0,769676323	0,987488947	LADA vs T1D
hsa-let-7f-5p_TGAGGTAGTAGATTGTATAGTT1	0,045887949	12,51171334	0,774309584	0,987488947	LADA vs T1D
hsa-miR-1307-3p_CTCGGCGTGGCGTCGGTCGTGG1	-0,162203131	2,190246459	0,777322588	0,987488947	LADA vs T1D
hsa-miR-425-5p_ATGACACGATCACTCCCGTTGA1	0,126078232	4,729605379	0,778132367	0,987488947	LADA vs T1D
hsa-miR-181a-5p_AACATTCAACGCTGTCGGT1	0,101218379	5,505188114	0,778556289	0,987488947	LADA vs T1D
hsa-miR-375-3p_TTTGTTCGTTCGGCTCGCG1	-0,104443049	5,830563474	0,783690518	0,987488947	LADA vs T1D
hsa-miR-222-3p_AGCTACATCTGGCTACTGGGTCTCT1	0,068400652	7,967322162	0,784419024	0,987488947	LADA vs T1D
hsa-miR-223-5p_CGTGTATTTGACAAGCTGAGTT1	0,127626417	3,93706767	0,785850457	0,987488947	LADA vs T1D
hsa-miR-28-5p_AAGGAGCTCACAGTCTATTGA1	0,162019669	3,11393162	0,785886212	0,987488947	LADA vs T1D
hsa-miR-100-5p_AACCCGTAGATCCGAACTTGT1	0,065913703	7,211971456	0,786240036	0,987488947	LADA vs T1D
hsa-miR-197-3p_TTCACCACCTTCTCCACCCA1	-0,131262386	3,958344822	0,79091587	0,987488947	LADA vs T1D
hsa-miR-486-5p_CCTGTACTGAGCTGCCCCGA1	-0,039419793	11,08007536	0,792308985	0,987488947	LADA vs T1D
hsa-miR-148a-3p_TCAGTGCACTACAGAACTTTG1	-0,052659265	7,287277231	0,79272746	0,987488947	LADA vs T1D
hsa-miR-30e-5p_TGTAAACATCCTTGACTGGA1	0,144096907	2,511168014	0,79280788	0,987488947	LADA vs T1D
hsa-miR-193a-5p_TGGGTCTTTGCGGGCGAGATGA1	0,043828593	8,630407946	0,793176672	0,987488947	LADA vs T1D
hsa-miR-486-5p_TCCTGTACTGAGCTGCCCCGA1	0,036100085	17,03890679	0,793583167	0,987488947	LADA vs T1D
hsa-miR-21-5p_TAGCTTATCAGACTGATGTTGAC1	0,058601384	10,44971368	0,793801712	0,987488947	LADA vs T1D
hsa-miR-27a-3p_TTCACAGTGGCTAAGTTCCGC1	-0,070146895	6,28650114	0,798388866	0,987488947	LADA vs T1D
hsa-miR-340-5p_TTATAAAGCAATGAGACTGATT1	0,090407297	5,815012818	0,798867607	0,987488947	LADA vs T1D
hsa-miR-484_CAGGCTCAGTCCCCTCCCGA1	-0,140723131	3,768443499	0,799885197	0,987488947	LADA vs T1D
hsa-miR-3173-5p_TGCCCTGCCTGTTTTCTCCTT1	0,14027232	2,97364428	0,800352276	0,987488947	LADA vs T1D
hsa-miR-148a-3p_TCAGTGCACTACAGAACTT1	-0,147614448	2,124123364	0,802242523	0,987488947	LADA vs T1D
hsa-miR-654-3p_TATGTCTGCTGACCATCACC1	-0,14812306	2,711988254	0,802630816	0,987488947	LADA vs T1D
hsa-miR-451a_AACCGTTACCATTACTGAGTT1	-0,073732749	6,297926904	0,8061451	0,987488947	LADA vs T1D
hsa-miR-369-3p_AATAATACATGGTTGATCTTT1	0,135395233	4,326255371	0,807011816	0,987488947	LADA vs T1D
hsa-miR-146a-5p_GAGAACTGAATTCCATGGGTT1	0,075725045	6,481720435	0,810054148	0,987488947	LADA vs T1D
hsa-miR-125b-5p_TCCCTGAGACCCTAACTTGTGA1	0,086811553	5,6455681	0,812718882	0,987488947	LADA vs T1D
hsa-miR-143-3p_TGAGATGAAGCACTGTAGCTC1	-0,035609569	9,842973082	0,814052117	0,987488947	LADA vs T1D
hsa-miR-342-3p_TCACACAGAAATCGCACCCGTC1	0,126384943	2,309823181	0,816900408	0,987488947	LADA vs T1D
hsa-miR-493-5p_TTGTACATGGTAGGCTTTCATT1	-0,138796156	3,64649227	0,818040901	0,987488947	LADA vs T1D
hsa-miR-98-5p_TGAGGTAGTAAGTTGTATTG1	0,10816617	4,611342852	0,818700531	0,987488947	LADA vs T1D
hsa-miR-10a-5p_TACCCTGTAGATCCGAATTT1	0,109747643	4,308439737	0,821703088	0,987488947	LADA vs T1D
hsa-let-7f-5p_TGAGGTAGTAGATTGTATAGTTT1	0,056104123	8,114676232	0,824134644	0,987488947	LADA vs T1D
hsa-miR-4446-3p_CAGGGCTGGCAGTGACATGGGT1	0,134216075	3,598813459	0,824641859	0,987488947	LADA vs T1D
hsa-miR-125a-5p_TCCCTGAGACCCTTTAACCTG1	-0,03921348	8,290201937	0,825388534	0,987488947	LADA vs T1D
hsa-miR-22-3p_AAGCTGCCAGTTGAAGAACT1	0,026046257	10,21399499	0,826667456	0,987488947	LADA vs T1D
hsa-let-7d-3p_ATACGACCTGCTGCCTTTCT1	-0,119643524	2,048536833	0,826726339	0,987488947	LADA vs T1D

hsa-miR-335-5p_TCAAGAGCAATAACGAAAAATG1	-0,038911447	8,412522999	0,82771756	0,987488947	LADA vs T1D
hsa-miR-186-5p_AAAGAATTCTCCTTTTGGGCT1	-0,112493498	3,890936215	0,830167407	0,987488947	LADA vs T1D
hsa-miR-148a-3p_CAGTGCACTACAGAACTTTGT1	-0,120622335	2,126116505	0,831427593	0,987488947	LADA vs T1D
hsa-miR-152-3p_TCAGTGCATGACAGAACTTGG1	0,108211669	2,073986305	0,831816249	0,987488947	LADA vs T1D
hsa-miR-222-3p_AGCTACATCTGGCTACTGGGTCTC1	0,08195764	6,101293098	0,834073907	0,987488947	LADA vs T1D
hsa-miR-191-5p_AACGGAATCCCAAAAGCAGCT1	-0,052994715	6,816281646	0,834143216	0,987488947	LADA vs T1D
hsa-miR-2110_TTGGGGAAACGGCCGCTGAGTGA1	-0,064495242	6,066188802	0,83483092	0,987488947	LADA vs T1D
hsa-miR-148a-3p_TCAGTGCACTACAGAACTTTGT1	0,028418726	9,289411705	0,835268678	0,987488947	LADA vs T1D
hsa-miR-16-2-3p_CCAATATTACTGTGCTGCTT1	0,082122162	4,446564737	0,835358558	0,987488947	LADA vs T1D
hsa-miR-146b-5p_TGAGAACTGAATTCCATAGGCT1	-0,058336982	6,34592746	0,835974703	0,987488947	LADA vs T1D
hsa-miR-7-5p_TGGAAGACTAGTGATTTTGTTGT1	0,078668827	6,176558156	0,836405473	0,987488947	LADA vs T1D
hsa-miR-652-3p_AATGGCGCCACTAGGGTTGT1	-0,031836643	7,904624885	0,838315724	0,987488947	LADA vs T1D
hsa-miR-144-3p_TACAGTATAGATGATGTAC1	-0,054400505	6,460339014	0,838856043	0,987488947	LADA vs T1D
hsa-miR-2110_TTGGGGAAACGGCCGCTGAG1	0,111228981	2,165591375	0,839446391	0,987488947	LADA vs T1D
hsa-miR-432-5p_TCTTGGAGTAGGTCATTGGGTGG1	0,123505741	3,502755168	0,839446632	0,987488947	LADA vs T1D
hsa-miR-99a-5p_AACCCGTAGATCCGATCTTGT1	0,03314415	8,529135227	0,840682964	0,987488947	LADA vs T1D
hsa-miR-1180-3p_TTTCCGGCTCGCGTGGGTGT1	-0,091901128	4,070138113	0,841939242	0,987488947	LADA vs T1D
hsa-miR-130a-3p_CAGTGCAATGTTAAAAGGGCA1	-0,088447797	4,214432691	0,844713121	0,987488947	LADA vs T1D
hsa-let-7f-5p_GAGGTAGTAGATTGTATAGT1	0,112313879	3,023408308	0,845941518	0,987488947	LADA vs T1D
hsa-miR-194-5p_TGTAACAGCAACTCCATGTGG1	0,047473923	6,660853419	0,84631552	0,987488947	LADA vs T1D
hsa-miR-191-5p_CAACGGAATCCCAAAAGCAGC1	0,036547618	8,150484012	0,84644423	0,987488947	LADA vs T1D
hsa-let-7b-5p_TGAGGTAGTAGGTTGTGTGGTTT1	-0,022946236	11,28589984	0,849162055	0,987488947	LADA vs T1D
hsa-miR-26a-5p_TTCAAGTAATCCAGGATAGG1	0,03963391	6,813190078	0,851372969	0,987488947	LADA vs T1D
hsa-miR-744-5p_TGCGGGGCTAGGGCTAACAGC1	-0,092193698	3,991128142	0,851444261	0,987488947	LADA vs T1D
hsa-miR-139-3p_TGGAGACGCGGCCCTGTTGGAGT1	0,103941921	3,435697412	0,854744015	0,987488947	LADA vs T1D
hsa-miR-425-5p_AATGACACGATCACTCCCG1	-0,083114302	4,547943713	0,858303762	0,987488947	LADA vs T1D
hsa-miR-125b-5p_CCCTGAGACCCTAACTTGT1	0,09819795	2,290772166	0,858431966	0,987488947	LADA vs T1D
hsa-miR-486-5p_TCCTGTACTGAGCTGCCC1	0,066273565	3,88539215	0,858689474	0,987488947	LADA vs T1D
hsa-miR-32-5p_TATTGCACATTACTAAGTTGC1	-0,096898314	2,00688677	0,860164743	0,987488947	LADA vs T1D
hsa-miR-23a-3p_ATCACATTGCCAGGGATTTCCA1	0,024003944	11,15500363	0,861956963	0,987488947	LADA vs T1D
hsa-miR-340-5p_TTATAAAGCAATGAGACTGAT1	-0,095725867	1,968507467	0,862252886	0,987488947	LADA vs T1D
hsa-miR-885-5p_TCCATTACACTACCCTGCCTCT1	0,187265869	4,714772841	0,862567635	0,987488947	LADA vs T1D
hsa-miR-574-3p_CACGCTCATGCACACACCCACA1	0,049169117	6,300877473	0,863242801	0,987488947	LADA vs T1D
hsa-miR-423-5p_GAGGGGCAGAGAGCGAGACT1	-0,051249371	6,874414686	0,863504697	0,987488947	LADA vs T1D
hsa-miR-486-5p_TCCTGTACTGAGCTGCCCCG1	0,027072159	14,12822837	0,86355484	0,987488947	LADA vs T1D
hsa-let-7b-5p_GAGGTAGTAGGTTGTGTGGT1	-0,072843996	4,944887656	0,865543511	0,987488947	LADA vs T1D
hsa-let-7f-5p_TGAGGTAGTAGATTGTATAGT1	0,017070192	10,56301224	0,869702514	0,987488947	LADA vs T1D
hsa-miR-130b-5p_ACTCTTTCCCTGTTGCACTACT1	-0,056330682	5,742085569	0,872207979	0,987488947	LADA vs T1D
hsa-miR-22-3p_AAGCTGCCAGTTGAAGAACTGT1	-0,01336045	12,01293	0,87288129	0,987488947	LADA vs T1D
hsa-miR-423-5p_TGAGGGGCAGAGAGCGAGAC1	0,015602653	9,751921415	0,875644919	0,987488947	LADA vs T1D
hsa-miR-93-5p_CAAAGTGCTGTTCGTGCAGGTA1	-0,05967944	5,442816943	0,876255664	0,987488947	LADA vs T1D
hsa-miR-181b-5p_AACATTCATTGCTGTCGGTGGGT1	0,074323846	3,594503523	0,876722968	0,987488947	LADA vs T1D
hsa-let-7d-5p_AGAGGTAGTAGGTTGCATAG1	-0,028239671	8,573362788	0,877373207	0,987488947	LADA vs T1D
hsa-let-7i-5p_TGAGGTAGTAGTTTGTGCTGTT1	-0,023724714	10,48466024	0,877685895	0,987488947	LADA vs T1D
hsa-miR-145-3p_ATTCCTGGAAATACTGTTCTT1	-0,083285757	3,50539909	0,878242023	0,987488947	LADA vs T1D
hsa-miR-660-5p_TACCCATTGCATATCGGAGTTGT1	-0,07621485	3,90808508	0,880390264	0,987488947	LADA vs T1D
hsa-miR-584-5p_TTATGGTTTGCCTGGGACTG1	0,078695122	2,558465624	0,880647897	0,987488947	LADA vs T1D
hsa-miR-30e-5p_TGTAAACATCCTTGACTGGAAG1	-0,025288072	7,619075745	0,883822315	0,987488947	LADA vs T1D
hsa-miR-483-5p_AAGACGGGAGGAAAGAAGGGAG1	0,050131015	6,618430366	0,885073605	0,987488947	LADA vs T1D
hsa-miR-505-3p_CGTCAACACTTGCTGGTTT1	-0,076037754	3,249239604	0,886416874	0,987488947	LADA vs T1D
hsa-miR-93-3p_ACTGCTGAGCTAGCACTTCCCGA1	0,075408098	3,371774968	0,889181282	0,987488947	LADA vs T1D
hsa-miR-342-5p_AGGGGTGCTATCTGTGATTGA1	-0,07917583	3,958584465	0,889729803	0,987488947	LADA vs T1D
hsa-miR-425-5p_ATGACACGATCACTCCCGTTGAGT1	-0,061958171	4,72212581	0,89128973	0,987488947	LADA vs T1D
hsa-miR-1228-3p_TCACACCTGCCTCGCCCCCCA1	-0,082248336	1,951009842	0,89210836	0,987488947	LADA vs T1D
hsa-miR-25-3p_ATTGCACTTGTCTCGGTCTG1	-0,066416122	3,200649251	0,893063558	0,987488947	LADA vs T1D
hsa-miR-425-5p_AATGACACGATCACTCCCGTTGAG1	0,050807481	6,063293525	0,89387267	0,987488947	LADA vs T1D
hsa-let-7a-5p_TGAGGTAGTAGGTTGTAT1	-0,088678425	3,216663859	0,894628713	0,987488947	LADA vs T1D
hsa-miR-3613-5p_TGTTGTACTTTTTTTTTTGTT1	-0,050447665	5,269511804	0,895918716	0,987488947	LADA vs T1D
hsa-miR-483-5p_AAGACGGGAGGAAAGAAGGGAGT1	-0,075573763	3,821071217	0,896023085	0,987488947	LADA vs T1D
hsa-miR-30a-5p_TGTAAACATCCTCGACTGG1	-0,060242204	4,494972654	0,897628746	0,987488947	LADA vs T1D
hsa-miR-221-3p_AGCTACATTGTCTGCTGGGT1	0,039355574	6,226471989	0,898261659	0,987488947	LADA vs T1D

hsa-let-7g-5p_TGAGGTAGTAGTTTGTACAGT1	-0,010624354	10,7052364	0,899444381	0,987488947	LADA vs T1D
hsa-miR-182-5p_TTTGGCAATGGTAGAACTCACACT1	0,027482598	7,651119737	0,902652558	0,987488947	LADA vs T1D
hsa-miR-23b-3p_ATCACATTGCCAGGGATTACCAC1	-0,055670136	5,113281158	0,902932937	0,987488947	LADA vs T1D
hsa-miR-183-5p_ATGGCACTGGTAGAATTCACTG1	0,068315368	2,888906189	0,902967482	0,987488947	LADA vs T1D
hsa-miR-181a-5p_ACATTCAACGCTGTCGGTGA1	-0,062703339	3,569893472	0,903028625	0,987488947	LADA vs T1D
hsa-miR-22-3p_AAGCTGCCAGTTGAAGAACTG1	0,020715209	9,134304454	0,904211353	0,987488947	LADA vs T1D
hsa-miR-16-5p_AGCAGCACGTAAATATTGG1	-0,06246674	3,167012316	0,904665307	0,987488947	LADA vs T1D
hsa-miR-92b-3p_TATTGCACTCGTCCCGGCCTCC1	-0,05474771	4,414239688	0,905289452	0,987488947	LADA vs T1D
hsa-miR-501-3p_AATGCACCCGGGCAAGGAT1	-0,064953894	2,760223516	0,905589477	0,987488947	LADA vs T1D
hsa-miR-21-5p_AGCTTATCAGACTGATGTTG1	-0,042036211	5,031654467	0,910175792	0,987488947	LADA vs T1D
hsa-miR-423-3p_AAGCTCGGTCTGAGGCCCCTCAG1	-0,056492142	3,402313099	0,911756474	0,987488947	LADA vs T1D
hsa-let-7d-3p_TATACGACCTGCTGCCTTTCT1	-0,01377924	8,488338468	0,913492141	0,987488947	LADA vs T1D
hsa-miR-10a-5p_TACCCTGTAGATCCGAATT1	-0,056022346	3,933865051	0,914084733	0,987488947	LADA vs T1D
hsa-miR-30c-5p_TGTAAACATCCTACACTCTCA1	0,02207424	7,955436132	0,915395607	0,987488947	LADA vs T1D
hsa-miR-98-5p_TGAGGTAGTAAGTTGTATTGTT1	-0,026755169	7,877215034	0,916051	0,987488947	LADA vs T1D
hsa-miR-192-5p_TGACCTATGAATTGACAGCC1	-0,029469633	6,449264096	0,916870745	0,987488947	LADA vs T1D
hsa-miR-29c-3p_TAGCACCATTTGAAATCGGT1	-0,052627657	4,339459305	0,918134208	0,987488947	LADA vs T1D
hsa-miR-193b-5p_CGGGGTTTTGAGGGCGAGATGA1	0,059165761	2,282241357	0,919577318	0,987488947	LADA vs T1D
hsa-miR-92a-3p_ATTGCACTTGTCCCGGCCTGT1	-0,010679337	12,31240602	0,919633721	0,987488947	LADA vs T1D
hsa-miR-30c-5p_TGTAAACATCCTACACTCTCAG1	0,043407353	4,767658059	0,920331555	0,987488947	LADA vs T1D
hsa-miR-409-3p_CGAATGTTGCTCGGTGAACCCCTT1	-0,060242113	3,646109852	0,9204358	0,987488947	LADA vs T1D
hsa-miR-181a-5p_AACATTCAACGCTGTCGG1	-0,04967892	2,192700831	0,921333118	0,987488947	LADA vs T1D
hsa-miR-183-5p_TATGGCACTGGTAGAATTCACT1	-0,037747028	5,796604044	0,921920827	0,987488947	LADA vs T1D
hsa-miR-223-3p_TGTCAGTTTGTCAAATACCCC1	0,027404641	7,314719746	0,922514078	0,987488947	LADA vs T1D
hsa-miR-361-5p_TTATCAGAATCTCCAGGGGT1	-0,038819402	4,747764862	0,923584683	0,987488947	LADA vs T1D
hsa-miR-451a_ACCGTTACCATTACTGAG1	-0,046114256	3,857793403	0,926253728	0,987488947	LADA vs T1D
hsa-let-7b-5p_GAGGTAGTAGGTTGTGTGG1	-0,029485495	6,125260792	0,926269543	0,987488947	LADA vs T1D
hsa-let-7f-5p_TGAGGTAGTAGATTGTATAGTTG1	-0,053221718	2,64125494	0,926384929	0,987488947	LADA vs T1D
hsa-miR-223-3p_TGTCAGTTTGTCAAATACCC1	-0,041594822	4,516322797	0,926898878	0,987488947	LADA vs T1D
hsa-miR-10b-5p_TACCCTGTAGAACCGAAT1	-0,050941836	2,220184724	0,927610577	0,987488947	LADA vs T1D
hsa-miR-361-3p_TCCCCCAGGTGTGATTCTGATTTG1	-0,031419684	5,241633262	0,928790898	0,987488947	LADA vs T1D
hsa-miR-192-5p_TGACCTATGAATTGACAGCCAG1	-0,047264856	3,652730706	0,929939141	0,987488947	LADA vs T1D
hsa-miR-30d-5p_TGTAAACATCCCCGACTGGAA1	0,008288455	11,66083446	0,931610824	0,987488947	LADA vs T1D
hsa-miR-24-3p_GCTCAGTTCAGCAGGAACAG1	0,044548337	2,25101941	0,93202982	0,987488947	LADA vs T1D
hsa-miR-433-3p_ATCATGATGGGCTCCTCGGTGT1	-0,048009421	4,303379714	0,933099185	0,987488947	LADA vs T1D
hsa-miR-21-5p_TAGCTTATCAGACTGATG1	-0,044959646	1,940313304	0,933463038	0,987488947	LADA vs T1D
hsa-miR-144-3p_TACAGTATAGATGATGTACT1	0,038527882	4,501383498	0,933544959	0,987488947	LADA vs T1D
hsa-miR-501-3p_AATGCACCCGGGCAAGGATT1	-0,022584656	6,44960483	0,933834945	0,987488947	LADA vs T1D
hsa-miR-339-3p_TGAGCGCCTCGACGACAGAGCCG1	-0,042722902	4,16247252	0,937287461	0,987488947	LADA vs T1D
hsa-miR-23a-3p_TCACATTGCCAGGGATTTC1	0,043653109	2,403793771	0,938853716	0,987488947	LADA vs T1D
hsa-miR-382-5p_GAAGTTGTTCGTGGTGGATTCG1	-0,026029435	7,607601722	0,938987085	0,987488947	LADA vs T1D
hsa-miR-221-3p_AGCTACATTGTCTGCTGGGTTT1	-0,013603965	7,916859704	0,939061556	0,987488947	LADA vs T1D
hsa-miR-485-3p_GTCATACACGGCTCTCCTCTCT1	-0,03864188	5,913980943	0,939167656	0,987488947	LADA vs T1D
hsa-miR-30d-5p_TAAACATCCCCGACTGGAAGCT1	-0,039647222	2,174183718	0,941223221	0,988346387	LADA vs T1D
hsa-miR-128-3p_TCACAGTGAACCGGTCTCT1	0,01202629	7,683472613	0,946546932	0,992159522	LADA vs T1D
hsa-miR-30d-5p_TGTAAACATCCCCGACTGGAAGCT1	-0,008077372	13,20515822	0,947344286	0,992159522	LADA vs T1D
hsa-let-7g-5p_TGAGGTAGTAGTTTGTACA1	0,027077447	5,425002874	0,949342656	0,992715979	LADA vs T1D
hsa-miR-16-5p_GCAGCACGTAAATATTGGCG1	0,030055197	3,625120505	0,950366741	0,992715979	LADA vs T1D
hsa-let-7b-5p_GAGGTAGTAGGTTGTGTGGTTT1	-0,029869947	3,859782449	0,954532567	0,995391388	LADA vs T1D
hsa-miR-30d-5p_TGTAAACATCCCCGACTGGAAGC1	-0,005520795	11,59455452	0,955425862	0,995391388	LADA vs T1D
hsa-miR-361-5p_TTATCAGAATCTCCAGGGGTACT1	-0,02850988	2,611707431	0,957997393	0,996750812	LADA vs T1D
hsa-miR-664a-3p_TATTCATTTATCCCCAGCCTACA1	-0,027180009	3,022366025	0,959231961	0,996750812	LADA vs T1D
hsa-miR-425-5p_AATGACACGATCACTCCCGTTG1	0,005623542	9,36928751	0,963647365	0,998307222	LADA vs T1D
hsa-miR-27a-3p_TTCACAGTGGCTAAGTTCC1	-0,014892941	5,369078847	0,965137476	0,998307222	LADA vs T1D
hsa-miR-183-5p_TATGGCACTGGTAGAATTC1	-0,022764578	3,376816673	0,965587846	0,998307222	LADA vs T1D
hsa-miR-484_TCAGGCTCAGTCCCCTCCCGAT1	-0,005671847	9,18414004	0,965740111	0,998307222	LADA vs T1D
hsa-miR-451a_AACCGTTACCATTACTGAGT1	0,006113256	10,87302044	0,968290575	0,998929416	LADA vs T1D
hsa-miR-10b-5p_ACCCTGTAGAACCGAATTTG1	0,015977699	4,835525593	0,970518182	0,998929416	LADA vs T1D
hsa-miR-345-5p_GCTGACTCCTAGTCCAGGGCTC1	0,01964225	3,656556535	0,971894241	0,998929416	LADA vs T1D
hsa-miR-486-5p_ATCCTGTACTGAGCTGCCCCGAG1	0,0065481	7,990918242	0,973156737	0,998929416	LADA vs T1D
hsa-miR-409-3p_CGAATGTTGCTCGGTGAACCCCTTT1	-0,020032973	2,598256943	0,973984491	0,998929416	LADA vs T1D

hsa-miR-3158-3p_AAGGGCTTCCTCTCTGCAGGA1	0,016426492	2,734692583	0,977426756	0,998929416	LADA vs T1D
hsa-miR-30a-5p_GTAAACATCCTCGACTGGAAGCT1	-0,012600602	4,1060576	0,979767542	0,998929416	LADA vs T1D
hsa-let-7a-5p_TTGAGGTAGTAGGTTGTATAGTT1	-0,013852792	1,956823269	0,979815733	0,998929416	LADA vs T1D
hsa-miR-379-5p_TGGTAGACTATGGAACGTAGG1	-0,014716054	3,839701767	0,979968815	0,998929416	LADA vs T1D
hsa-miR-501-3p_AATGCACCCGGGCAAGGATTCT1	0,003399244	7,911300847	0,981177354	0,998929416	LADA vs T1D
hsa-miR-374b-5p_ATATAATACAACCTGCTAAGT1	0,011389106	3,95574137	0,981608278	0,998929416	LADA vs T1D
hsa-miR-125b-5p_TCCCTGAGACCCTAACTTGT1	-0,00278006	9,802438562	0,984294086	0,998929416	LADA vs T1D
hsa-let-7g-5p_GAGGTAGTAGTTTGTACAGT1	-0,010609616	3,423010066	0,984849955	0,998929416	LADA vs T1D
hsa-let-7d-3p_CTATACGACCTGCTGCCTT1	0,006534678	4,847124715	0,985609854	0,998929416	LADA vs T1D
hsa-miR-4433b-5p_TGTCCCACCCCCACTCCTGTTT1	-0,005473185	8,596146134	0,986421333	0,998929416	LADA vs T1D
hsa-miR-1908-5p_CGGCGGGGACGGCGATTGGT1	0,009115764	2,686402078	0,987743719	0,998929416	LADA vs T1D
hsa-let-7i-5p_TGAGGTAGTAGTTTGTGCTG1	-0,002214271	10,07708045	0,987962022	0,998929416	LADA vs T1D
hsa-miR-484_TCAGGCTCAGTCCCCTCCCGA1	-0,001755981	10,80053241	0,991253064	0,998929416	LADA vs T1D
hsa-miR-30c-5p_TGTAAACATCCTACACTCTC1	-0,005166262	2,069916362	0,992559111	0,998929416	LADA vs T1D
hsa-miR-4433b-5p_TGTCCCACCCCCACTCCTGT1	-0,003610183	4,771975689	0,994052093	0,998929416	LADA vs T1D
hsa-miR-224-5p_CAAGTCACTAGTGGTTCCGTTTAG1	-0,002576454	4,65780231	0,996166376	0,998929416	LADA vs T1D
hsa-miR-29a-3p_CTAGCACCATCTGAAATCGGTT1	0,002255483	3,457099062	0,99629399	0,998929416	LADA vs T1D
hsa-miR-223-3p_GTCAGTTTGTCAAATACCCCA1	0,001203824	6,454169749	0,996793607	0,998929416	LADA vs T1D
hsa-miR-451a_AAACCGTTACCATTACTGAGTTT1	0,000253378	8,99265157	0,998727583	0,998929416	LADA vs T1D
hsa-miR-28-3p_CACTAGATTGTGAGCTCCTGGA1	0,000400022	6,806864009	0,998796385	0,998929416	LADA vs T1D
hsa-miR-486-5p_CTGTACTGAGCTGCCCCG1	0,000731974	3,40982355	0,998929416	0,998929416	LADA vs T1D
hsa-miR-16-5p_TAGCAGCACGTAAATATTGGC2	-0,503021064	9,94541903	4,09756E-05	0,016932398	LADA vs T2D
hsa-miR-140-3p_TACCACAGGGTAGAACCACGGACA2	-2,042503966	3,637619462	4,24903E-05	0,016932398	LADA vs T2D
hsa-let-7d-3p_CTATACGACCTGCTGCCTTTC2	0,389685005	10,32598693	0,000126219	0,025611629	LADA vs T2D
hsa-miR-106b-3p_CCGCACTGTGGGTACTTGCTG2	-1,73318618	4,162035136	0,00012854	0,025611629	LADA vs T2D
hsa-miR-487b-3p_AATCGTACAGGGTCATCCACTT2	-2,189777865	4,048228768	0,000209221	0,033349762	LADA vs T2D
hsa-miR-106b-3p_CCGCACTGTGGGTACTTGCT2	-1,13334031	6,396206525	0,00037883	0,047477017	LADA vs T2D
hsa-miR-10b-5p_TACCCTGTAGAACCGAATTTG2	0,439800576	9,833049317	0,000426936	0,047477017	LADA vs T2D
hsa-let-7a-5p_TGAGGTAGTAGGTTGTATA2	-1,198614484	5,663865648	0,000476557	0,047477017	LADA vs T2D
hsa-miR-140-3p_TACCACAGGGTAGAACCACGGA2	-0,70760954	7,894124989	0,000570846	0,050551584	LADA vs T2D
hsa-miR-140-3p_ACCACAGGGTAGAACCACGGA2	-0,936596499	6,646596672	0,000810292	0,06458025	LADA vs T2D
hsa-miR-181b-5p_AACATTCATTGCTGTCGGTGGGT2	-1,600177605	3,594503523	0,000899537	0,065175526	LADA vs T2D
hsa-miR-361-3p_TCCCCCAGGTGTGATTCTGATTT2	-1,595723728	3,977031543	0,001112197	0,072801339	LADA vs T2D
hsa-miR-93-5p_CAAAGTGCTGTTCGTGCAGGTAG2	-0,37372361	9,991178453	0,001352685	0,072801339	LADA vs T2D
hsa-miR-320a-3p_AAAGCTGGGTTGAGAGGGCGAA2	-1,485221217	4,271030608	0,001366155	0,072801339	LADA vs T2D
hsa-miR-25-3p_ATTGCACTTGTCTCGGTCTG2	-1,583369871	3,200649251	0,001441506	0,072801339	LADA vs T2D
hsa-miR-197-3p_TTCACCACCTTCTCCACCCAG2	0,553678955	8,762482756	0,001556019	0,072801339	LADA vs T2D
hsa-miR-140-3p_ACCACAGGGTAGAACCACGGACA2	-1,507627852	4,192134912	0,001613396	0,072801339	LADA vs T2D
hsa-miR-20a-5p_TAAAGTGCTTATAGTGCAGGTAG2	-1,433749849	4,004629847	0,001644196	0,072801339	LADA vs T2D
hsa-miR-652-3p_AATGGCGCCACTAGGGTTGTG2	-0,851342526	6,468098031	0,00179591	0,075333689	LADA vs T2D
hsa-miR-142-5p_CCCATAAAGTAGAAAGCA2	-1,456618289	2,550307907	0,002018892	0,080452835	LADA vs T2D
hsa-let-7c-5p_TGAGGTAGTAGGTTGTATGG2	-1,086650764	3,04066672	0,002362235	0,086756663	LADA vs T2D
hsa-miR-451a_AAACCGTTACCATTACTGAGTT2	-0,447273953	12,14012514	0,002463529	0,086756663	LADA vs T2D
hsa-miR-16-2-3p_ACCAATATTACTGTGCTGCT2	-1,358516349	4,606374691	0,002503643	0,086756663	LADA vs T2D
hsa-miR-451a_AACCGTTACCATTACTGAGTT2	-0,90214434	6,297926904	0,002802016	0,092228056	LADA vs T2D
hsa-miR-140-3p_ACCACAGGGTAGAACCACGGAC2	-0,990115576	5,852260421	0,002970161	0,092228056	LADA vs T2D
hsa-miR-484_CAGGCTCAGTCCCCTCCCGAT2	-1,538242652	2,404164158	0,00301038	0,092228056	LADA vs T2D
hsa-miR-16-5p_TAGCAGCACGTAAATATTG2	-0,994074978	5,566162995	0,003124413	0,092228056	LADA vs T2D
hsa-miR-22-3p_AGCTGCCAGTTGAAGAACTGT2	-1,092665608	5,523006419	0,003555827	0,09856423	LADA vs T2D
hsa-miR-320b_AAAAGCTGGGTTGAGAGGGCA2	-1,430420653	2,618155177	0,003689042	0,09856423	LADA vs T2D
hsa-miR-140-3p_TACCACAGGGTAGAACCACGG2	-0,906548207	5,563733367	0,003710071	0,09856423	LADA vs T2D
hsa-miR-423-3p_AAGCTCGGTCTGAGGCCCCTCAG2	-1,480653446	3,402313099	0,003874978	0,099624422	LADA vs T2D
hsa-miR-186-5p_CAAAGAATTCTCCTTTTGGGC2	-0,803895668	6,535812742	0,004039309	0,099776034	LADA vs T2D
hsa-miR-122-5p_GGAGTGTGACAATGGTGTTTG2	-1,23549043	5,615777051	0,004131254	0,099776034	LADA vs T2D
hsa-miR-1180-3p_TTTCCGGCTCGCGTGGGTGTGT2	-1,501219081	2,874816557	0,004262708	0,099922897	LADA vs T2D
hsa-miR-425-5p_ATGACACGATCACTCCCGTTG2	-1,5746686	2,398751928	0,004596709	0,101785308	LADA vs T2D
hsa-miR-26b-5p_TTCAAGTAATTCAGGATAGG2	-1,304807759	3,436379997	0,00459758	0,101785308	LADA vs T2D
hsa-miR-30d-5p_GTAAACATCCCCGACTGGAAGCT2	-0,704094694	6,998439264	0,004894277	0,10542537	LADA vs T2D
hsa-miR-484_TCAGGCTCAGTCCCCTCCCGATA2	-1,195936668	4,637634197	0,005954277	0,123033857	LADA vs T2D
hsa-miR-93-5p_CAAAGTGCTGTTCGTGCAGGTA2	-1,054806348	5,442816943	0,006035713	0,123033857	LADA vs T2D
hsa-miR-10b-5p_TACCCTGTAGAACCGAATTTGT2	0,321544245	11,12588043	0,006174849	0,123033857	LADA vs T2D

hsa-miR-25-3p_CATTGCACTTGTCTCGGTCTGA2	-0,389671429	11,99390817	0,006740721	0,131033039	LADA vs T2D
hsa-miR-194-5p_TGTAACAGCAACTCCATGTGGAA2	-1,41913566	2,659899918	0,00735971	0,13965925	LADA vs T2D
hsa-miR-1180-3p_TTTCCGGCTCGCGTGGGTGT2	-1,232295055	4,070138113	0,007761489	0,143858301	LADA vs T2D
hsa-miR-146b-5p_TGAGAACTGAATTCCATAGGCTG2	-0,946694867	5,765289472	0,008700803	0,155181149	LADA vs T2D
hsa-miR-18a-3p_ACTGCCCTAAGTGCTCCTTCTG2	-1,393069875	2,22678804	0,008761796	0,155181149	LADA vs T2D
hsa-miR-185-5p_TGGAGAGAAAGGCAGTTCCTGA2	-0,354504056	8,851967404	0,008983659	0,155651655	LADA vs T2D
hsa-miR-361-5p_TTATCAGAATCTCCAGGGGTA2	-1,390598255	3,298750595	0,009198037	0,155975224	LADA vs T2D
hsa-miR-183-5p_TATGGCACTGGTAGAATTC2	-1,35891571	3,376816673	0,010288754	0,167980069	LADA vs T2D
hsa-miR-182-5p_TTTGGCAATGGTAGAACT2	-1,442146959	3,421424723	0,010379663	0,167980069	LADA vs T2D
hsa-miR-484_CAGGCTCAGTCCCCTCCCGA2	-1,422942171	3,768443499	0,010538273	0,167980069	LADA vs T2D
hsa-miR-122-5p_TGGAGTGTGACAATGGTGTTTG2	-0,674925733	11,63439763	0,012168606	0,190164295	LADA vs T2D
hsa-miR-6803-3p_TCCCTCGCCTTCTCACCCTCAGT2	1,478232752	2,14031641	0,012553764	0,192410571	LADA vs T2D
hsa-miR-25-3p_CATTGCACTTGTCTCGGTCTG2	-0,424348419	9,990945876	0,012955508	0,194821511	LADA vs T2D
hsa-miR-484_TCAGGCTCAGTCCCCTCCCGAT2	-0,326692184	9,18414004	0,013431912	0,198245073	LADA vs T2D
hsa-miR-409-3p_GAATGTTGCTCGGTGAACCCCT2	-0,857074568	7,973406619	0,014161611	0,201667415	LADA vs T2D
hsa-miR-181a-2-3p_ACCACTGACCGTTGACTGTAC2	-1,296462878	3,544826687	0,014169856	0,201667415	LADA vs T2D
hsa-let-7d-3p_CTATACGACCTGCTGCCTTT2	0,311797174	10,24742095	0,014663665	0,203108259	LADA vs T2D
hsa-let-7i-5p_TGAGGTAGTAGTTTGTGCT2	-0,511881431	7,615350025	0,014845663	0,203108259	LADA vs T2D
hsa-miR-363-3p_AATTGCACGGTATCCATCTG2	-0,410421544	8,264741807	0,015069715	0,203108259	LADA vs T2D
hsa-miR-574-3p_CACGCTCATGCACACACCCAC2	-1,152321306	4,117697394	0,015290459	0,203108259	LADA vs T2D
hsa-miR-24-3p_TGGCTCAGTTCAGCAGGAAC2	0,273256829	10,07194833	0,016504204	0,214901441	LADA vs T2D
hsa-miR-181a-2-3p_ACCACTGACCGTTGACTGT2	-1,299583574	2,531276889	0,016792974	0,214901441	LADA vs T2D
hsa-miR-425-5p_AATGACACGATCACTCCCGTTGA2	-0,245424623	10,37803313	0,01698719	0,214901441	LADA vs T2D
hsa-miR-186-5p_CAAAGAATTCTCCTTTTGGGCT2	-0,296494522	9,032677455	0,017457469	0,217400037	LADA vs T2D
hsa-miR-1294_TGTGAGGTTGGCATTGTTGT2	-1,301969681	2,481086687	0,018158335	0,222126058	LADA vs T2D
hsa-let-7g-5p_TGAGGTAGTAGTTTGTACAGTTT2	-0,608367787	6,811439564	0,018394379	0,222126058	LADA vs T2D
hsa-let-7g-5p_TGAGGTAGTAGTTTGTACAG2	-0,30110874	9,407543438	0,018778269	0,223377316	LADA vs T2D
hsa-miR-451a_ACCGTTACCATTACTGAGTT2	-1,264177543	2,004581894	0,019512996	0,22870379	LADA vs T2D
hsa-miR-183-5p_ATGGCACTGGTAGAATTCACT2	-1,089995535	4,652978697	0,019923945	0,230136	LADA vs T2D
hsa-miR-7-5p_TGGAAGACTAGTGATTTTGTTG2	-1,087766468	3,890826404	0,021310095	0,240959157	LADA vs T2D
hsa-miR-193a-5p_TGGGTCTTTGCGGGCGAGA2	1,335580351	3,488597754	0,021465621	0,240959157	LADA vs T2D
hsa-let-7b-5p_TGAGGTAGTAGGTTGTGTG2	-0,48130205	6,962968331	0,021810829	0,241433759	LADA vs T2D
hsa-miR-29a-3p_CTAGCACCATCTGAAATCGG2	-1,180393517	3,050349736	0,022682554	0,247643772	LADA vs T2D
hsa-miR-361-5p_TTATCAGAATCTCCAGGGGT2	-0,914075785	4,747764862	0,02438145	0,262503171	LADA vs T2D
hsa-miR-28-3p_CACTAGATTGTGAGCTCCTGGA2	-0,593359763	6,806864009	0,025170794	0,262503171	LADA vs T2D
hsa-miR-93-5p_CAAAGTGCTGTTCGTGCAGGT2	-0,823971544	4,918359962	0,025242138	0,262503171	LADA vs T2D
hsa-miR-30d-5p_TGTAAACATCCCCGACTGGAAGC2	-0,22071699	11,59455452	0,025361034	0,262503171	LADA vs T2D
hsa-miR-363-3p_AATTGCACGGTATCCATCT2	-1,065956204	4,478964619	0,026415022	0,267509083	LADA vs T2D
hsa-miR-21-3p_CAACACCAGTCGATGGGCTGT2	-1,135152317	2,296740898	0,027070517	0,267509083	LADA vs T2D
hsa-miR-363-3p_ATTGCACGGTATCCATCTGT2	-1,046001909	4,343538892	0,027300578	0,267509083	LADA vs T2D
hsa-miR-345-5p_GCTGACTCCTAGTCCAGGGCT2	-0,911395214	5,057881275	0,02740704	0,267509083	LADA vs T2D
hsa-let-7b-5p_GAGGTAGTAGGTTGTGTGGTT2	-0,631419651	6,209954592	0,027522892	0,267509083	LADA vs T2D
hsa-miR-25-3p_ATTGCACTTGTCTCGGTCT2	-1,043810238	3,620590845	0,028742406	0,27599636	LADA vs T2D
hsa-miR-486-3p_CGGGGCAGCTCAGTACAGGA2	-0,648640905	6,539637794	0,029613038	0,277510769	LADA vs T2D
hsa-miR-335-3p_GTTTTTCATTATTGCTCCTGACC2	-1,257914233	2,935136252	0,029713827	0,277510769	LADA vs T2D
hsa-let-7d-3p_CTATACGACCTGCTGCCTTTCT2	0,238170852	11,76128234	0,0299447	0,277510769	LADA vs T2D
hsa-let-7a-5p_TGAGGTAGTAGGTTGTATAG2	-0,227029051	12,05467063	0,031261858	0,284373083	LADA vs T2D
hsa-miR-16-5p_TAGCAGCACGTAAATATTGGCGT2	-0,395916742	7,90073048	0,031398785	0,284373083	LADA vs T2D
hsa-miR-92b-3p_TATTGCACTCGTCCCGGCCTCC2	-0,982446509	4,414239688	0,032512555	0,285565852	LADA vs T2D
hsa-miR-148b-3p_TCAGTGCATCACAGAACTTTG2	-0,907718029	4,882892663	0,032827616	0,285565852	LADA vs T2D
hsa-miR-30e-5p_TGTAAACATCCTTGACTGGAAGCT2	-0,154211326	11,72480372	0,032912686	0,285565852	LADA vs T2D
hsa-miR-223-3p_TGTCAGTTTGTCAAATACCCCAA2	-0,396907888	10,91791178	0,033064205	0,285565852	LADA vs T2D
hsa-miR-101-3p_GTACAGTACTGTGATAACT2	-0,902419389	4,990449736	0,033321988	0,285565852	LADA vs T2D
hsa-let-7b-5p_GAGGTAGTAGGTTGTGTGGT2	-0,904729793	4,944887656	0,035072701	0,297371733	LADA vs T2D
hsa-miR-24-3p_TGGCTCAGTTCAGCAGGAACA2	-0,229847346	9,988806411	0,035612592	0,29819659	LADA vs T2D
hsa-miR-24-3p_GGCTCAGTTCAGCAGGAACA2	-1,157325655	2,640585933	0,035918284	0,29819659	LADA vs T2D
hsa-miR-423-3p_AGCTCGGTCTGAGGCCCCTCAG2	-0,253721274	8,372128456	0,036620388	0,300891232	LADA vs T2D
hsa-miR-192-5p_TGACCTATGAATTGACAGCCAG2	-1,121370779	3,652730706	0,037225973	0,30274592	LADA vs T2D
hsa-miR-29a-3p_TAGCACCATCTGAAATCGGTTA2	-0,941475135	4,716466347	0,038324243	0,30852951	LADA vs T2D
hsa-miR-140-3p_ACCACAGGGTAGAACCACGG2	-1,043768676	3,491351538	0,039682767	0,312193284	LADA vs T2D
hsa-miR-486-5p_GTACTGAGCTGCCCCGAG2	-0,929656101	4,883397866	0,039946315	0,312193284	LADA vs T2D

hsa-miR-223-3p_GTCAGTTTGTCAAATACCCCAA2	-0,541954724	7,740128632	0,039954473	0,312193284	LADA vs T2D
hsa-miR-192-5p_TGACCTATGAATTGACAGCCAGT2	-1,061842586	3,322199511	0,041166301	0,318539242	LADA vs T2D
hsa-miR-126-3p_CGTACCGTGAGTAATAATGC2	-0,859727675	4,46578231	0,042246432	0,322278585	LADA vs T2D
hsa-miR-425-5p_AATGACACGATCACTCCCGTTG2	-0,249651224	9,36928751	0,042575983	0,322278585	LADA vs T2D
hsa-let-7b-5p_TGAGGTAGTAGGTTGTGTGGT2	-0,210301819	11,84645942	0,042900228	0,322278585	LADA vs T2D
hsa-miR-423-5p_TGAGGGGCAGAGAGCGAGACTTT2	0,139710568	14,42674435	0,043627735	0,322278585	LADA vs T2D
hsa-miR-125b-5p_TCCCTGAGACCCTAACTTGTGA2	-0,740015855	5,6455681	0,043679524	0,322278585	LADA vs T2D
hsa-miR-451a_ACCGTTACCATTACTGAGT2	-0,481690563	7,452815255	0,04447009	0,322278585	LADA vs T2D
hsa-miR-484_TCAGGCTCAGTCCCCTCCCG2	-0,476339862	7,780454359	0,044480106	0,322278585	LADA vs T2D
hsa-miR-532-5p_CATGCCTTGAGTGTAGGACCGT2	-0,893258844	4,730944055	0,045287596	0,325173099	LADA vs T2D
hsa-miR-16-5p_AGCAGCACGTAAATATTGG2	-1,041928287	3,167012316	0,046156308	0,327673956	LADA vs T2D
hsa-miR-25-3p_CATTGCACTTGTCTCGGTC2	-0,685250512	5,77988375	0,046458164	0,327673956	LADA vs T2D
hsa-miR-183-5p_TATGGCACTGGTAGAATTCACT2	-0,758070708	5,796604044	0,048503844	0,33520793	LADA vs T2D
hsa-miR-183-5p_ATGGCACTGGTAGAATTCACTG2	-1,109789127	2,888906189	0,048665411	0,33520793	LADA vs T2D
hsa-miR-99b-5p_CACCCGTAGAACCGACCTTGC2	0,301146589	7,880557566	0,049312238	0,33520793	LADA vs T2D
hsa-miR-423-3p_AAGCTCGGTCTGAGGCCCCTCA2	-1,043217299	2,923364981	0,049442314	0,33520793	LADA vs T2D
hsa-miR-221-3p_AGCTACATTGTCTGCTGGGTT2	-0,608772928	6,683307876	0,049957177	0,33520793	LADA vs T2D
hsa-let-7b-5p_GAGGTAGTAGGTTGTGTGGTTT2	-1,025517043	3,859782449	0,050302452	0,33520793	LADA vs T2D
hsa-miR-423-3p_AGCTCGGTCTGAGGCCCCTCA2	-0,623047094	6,171912051	0,050470454	0,33520793	LADA vs T2D
hsa-miR-21-5p_TAGCTTATCAGACTGATGTTG2	0,217757436	11,59436943	0,051084662	0,335745901	LADA vs T2D
hsa-miR-543_AAACATTCGCGGTGCACTTCTT2	-1,020369844	3,845833985	0,051393977	0,335745901	LADA vs T2D
hsa-miR-186-5p_CAAAGAATTCTCCTTTTGGGCTTT2	-1,002248792	4,003798957	0,052299636	0,338884631	LADA vs T2D
hsa-miR-181a-5p_ACATTCAACGCTGTCGGTGA2	-0,988616514	3,569893472	0,054810464	0,347267215	LADA vs T2D
hsa-miR-505-3p_CGTCAACACTTGCTGGTTT2	-1,022581115	3,249239604	0,05510241	0,347267215	LADA vs T2D
hsa-miR-222-3p_AGCTACATCTGGCTACTGGGTCT2	-0,528516849	6,5045561	0,055252539	0,347267215	LADA vs T2D
hsa-miR-101-3p_GTACAGTACTGTGATAACTG2	-0,700231762	5,734652626	0,055336181	0,347267215	LADA vs T2D
hsa-miR-197-3p_TTCACCACCTTCTCCACCCAGC2	0,294040701	10,1606215	0,055985669	0,348598267	LADA vs T2D
hsa-miR-4732-5p_TGTAGAGCAGGGAGCAGGAAGCT2	-0,58644038	6,276959061	0,058178171	0,359441879	LADA vs T2D
hsa-miR-494-3p_TGAAACATACACGGGAAACCTCT2	-1,010109319	4,269868739	0,059032445	0,359808306	LADA vs T2D
hsa-miR-451a_CGTTACCATTACTGAGT2	-0,900716688	4,012413349	0,059140386	0,359808306	LADA vs T2D
hsa-miR-27a-3p_TTCACAGTGGCTAAGTTCCG2	0,227606336	9,740987062	0,060323354	0,36224935	LADA vs T2D
hsa-miR-30a-5p_GTAAACATCCTCGACTGGAAGCT2	-0,932577784	4,1060576	0,060450644	0,36224935	LADA vs T2D
hsa-miR-25-3p_ATTGCACTTGTCTCGGTCTGA2	-0,611196928	5,837958082	0,061605774	0,366012417	LADA vs T2D
hsa-miR-28-5p_AAGGAGCTCACAGTCTATTGAG2	-1,052949891	3,384277878	0,062131321	0,366012417	LADA vs T2D
hsa-miR-10a-5p_TACCCTGTAGATCCGAATTTGTGT2	-1,011686699	2,404757373	0,06278122	0,366012417	LADA vs T2D
hsa-miR-27b-3p_TTCACAGTGGCTAAGTTCTGC2	-0,33700831	7,903620851	0,06291556	0,366012417	LADA vs T2D
hsa-miR-424-3p_CAAAACGTGAGGCGCTGCT2	-0,988332344	2,803316018	0,063457502	0,366490063	LADA vs T2D
hsa-miR-16-2-3p_ACCAATATTACTGTGCTGCTTT2	-0,283360313	7,879139867	0,065800895	0,377290022	LADA vs T2D
hsa-miR-146b-5p_TGAGAACTGAATTCCATAGGCT2	-0,516772435	6,34592746	0,066595384	0,379118008	LADA vs T2D
hsa-miR-30d-5p_TAAACATCCCCGACTGGAAGCT2	-0,980958296	2,174183718	0,068595753	0,383195297	LADA vs T2D
hsa-miR-182-5p_TTTGGCAATGGTAGAACTCACA2	-0,491080496	6,943076472	0,068819845	0,383195297	LADA vs T2D
hsa-miR-10a-5p_TACCCTGTAGATCCGAATTTG2	0,266213568	8,855203262	0,069101446	0,383195297	LADA vs T2D
hsa-miR-21-5p_TAGCTTATCAGACTGATG2	-0,979355136	1,940313304	0,069234784	0,383195297	LADA vs T2D
hsa-miR-320b_AAAAGCTGGGTTGAGAGGGC2	-1,48623196	5,061955788	0,069852802	0,383949537	LADA vs T2D
hsa-miR-451a_ACCGTTACCATTACTGAG2	-0,899025553	3,857793403	0,07136672	0,389584084	LADA vs T2D
hsa-miR-182-5p_TTTGGCAATGGTAGAACTC2	-1,013932338	2,382812326	0,074524682	0,395805513	LADA vs T2D
hsa-miR-1306-5p_CCACCTCCCCTGCAAACGTCC2	0,81517163	4,76371819	0,074717241	0,395805513	LADA vs T2D
hsa-miR-181a-5p_AACATTCAACGCTGTCGG2	-0,897132976	2,192700831	0,074779013	0,395805513	LADA vs T2D
hsa-miR-151a-3p_TACTAGACTGAAGCTCCTTGAG2	-0,953194842	3,055291554	0,075087159	0,395805513	LADA vs T2D
hsa-miR-101-3p_TACAGTACTGTGATAACTGAAG2	-0,398694959	7,012156412	0,075634627	0,395805513	LADA vs T2D
hsa-miR-584-5p_TTATGGTTTGCCTGGGACTG2	-0,930044103	2,558465624	0,076133838	0,395805513	LADA vs T2D
hsa-miR-15b-5p_TAGCAGCACATCATGGTTTA2	-0,584504126	5,794504936	0,076380439	0,395805513	LADA vs T2D
hsa-miR-2110_TTGGGGAAACGGCCGCTGAGTG2	-1,029725572	2,932814217	0,076479359	0,395805513	LADA vs T2D
hsa-miR-125b-5p_TCCCTGAGACCCTAACTT2	-0,886637918	3,016083692	0,078448554	0,40042227	LADA vs T2D
hsa-miR-409-3p_GAATGTTGCTCGGTGAACCCCTTT2	-1,023961781	4,139609499	0,078451024	0,40042227	LADA vs T2D
hsa-miR-652-3p_AATGGCGCCACTAGGGTTGTGC2	-0,884855668	3,594273542	0,078878666	0,40042227	LADA vs T2D
hsa-miR-423-5p_GAGGGGCAGAGAGCGAGA2	0,969506717	1,916584014	0,079688778	0,401974403	LADA vs T2D
hsa-miR-1228-3p_TCACACCTGCCTCGCCCCCCA2	1,059628737	1,951009842	0,08069077	0,403002861	LADA vs T2D
hsa-miR-421_ATCAACAGACATTAATTGGGCGC2	-0,910859698	2,04052248	0,081105943	0,403002861	LADA vs T2D
hsa-miR-22-3p_AAGCTGCCAGTTGAAGAACTG2	-0,298011043	9,134304454	0,082324057	0,403002861	LADA vs T2D
hsa-miR-425-5p_AATGACACGATCACTCCCG2	-0,806285775	4,547943713	0,083272477	0,403002861	LADA vs T2D

hsa-miR-181a-2-3p_ACCACTGACCGTTGACTGTACC2	-0,984989258	2,397891497	0,083714297	0,403002861	LADA vs T2D
hsa-let-7g-5p_GAGGTAGTAGTTTGTACAGT2	-0,963982432	3,423010066	0,084286506	0,403002861	LADA vs T2D
hsa-miR-182-5p_TTTGGCAATGGTAGAACTCA2	-0,391637602	7,910992501	0,084587833	0,403002861	LADA vs T2D
hsa-miR-92b-3p_TATTGCACTCGTCCCGGCCTC2	-0,827410253	3,886574178	0,084748061	0,403002861	LADA vs T2D
hsa-miR-24-3p_GCTCAGTTCAGCAGGAACAG2	-0,898771105	2,25101941	0,084986951	0,403002861	LADA vs T2D
hsa-miR-320a-3p_GAAAAGCTGGGTTGAGAGGGCGA2	-0,893951066	3,456353792	0,085232455	0,403002861	LADA vs T2D
hsa-miR-486-3p_CGGGGCAGCTCAGTACAGGAT2	-0,403561728	7,803889597	0,08545481	0,403002861	LADA vs T2D
hsa-let-7g-5p_TGAGGTAGTAGTTTGTACA2	-0,730042407	5,425002874	0,086114614	0,403725575	LADA vs T2D
hsa-miR-342-3p_TCTCACACAGAAATCGCACCCGTCA2	0,934147137	3,422364811	0,086858093	0,404829825	LADA vs T2D
hsa-let-7a-5p_GAGGTAGTAGGTTGTATAG2	-0,665098588	5,403501302	0,087924778	0,406484621	LADA vs T2D
hsa-let-7b-5p_TGAGGTAGTAGGTTGTGTGG2	0,178993768	12,43476497	0,088233174	0,406484621	LADA vs T2D
hsa-miR-21-5p_AGCTTATCAGACTGATGTTGAC2	-0,996677952	3,189772648	0,089626539	0,408455823	LADA vs T2D
hsa-miR-125a-5p_TCCCTGAGACCCTTTAACCTGT2	0,199991169	11,48945871	0,089686034	0,408455823	LADA vs T2D
hsa-miR-30d-5p_TGTAAACATCCCCGACTGG2	0,225919038	8,045778015	0,091461537	0,414175258	LADA vs T2D
hsa-let-7g-5p_TGAGGTAGTAGTTTGTACAGT2	-0,141066146	10,7052364	0,092295295	0,414487805	LADA vs T2D
hsa-miR-142-5p_CCCATAAAGTAGAAAGCACTAC2	-0,394698547	7,021131164	0,092777339	0,414487805	LADA vs T2D
hsa-miR-223-3p_TGTCAGTTTGTCAAATACCC2	-0,761017054	4,516322797	0,093172585	0,414487805	LADA vs T2D
hsa-miR-486-5p_TGTACTGAGCTGCCCCGAG2	-0,68654525	5,036658236	0,093613391	0,414487805	LADA vs T2D
hsa-miR-30d-5p_TGTAAACATCCCCGACTGGAAGCT2	-0,203857688	13,20515822	0,094339913	0,414487805	LADA vs T2D
hsa-miR-505-3p_CGTCAACACTTGCTGGTTTCCT2	-0,872625141	3,899410508	0,094650917	0,414487805	LADA vs T2D
hsa-miR-146a-5p_TGAGAACTGAATTCCATGGGTTGT2	-0,469449969	7,265118969	0,097161186	0,421157586	LADA vs T2D
hsa-miR-409-3p_GAATGTTGCTCGGTGAACCCCTT2	-0,909275051	5,101558557	0,097324066	0,421157586	LADA vs T2D
hsa-miR-30c-5p_TGTAAACATCCTACACTCTCA2	-0,342250836	7,955436132	0,097759289	0,421157586	LADA vs T2D
hsa-miR-16-5p_AGCAGCACGTAAATATTGGC2	-0,865132928	2,995434135	0,10067197	0,430875701	LADA vs T2D
hsa-miR-328-3p_CTGGCCCTCTCTGCCCTTCCG2	-0,784803306	3,770890003	0,101096306	0,430875701	LADA vs T2D
hsa-miR-486-5p_GTACTGAGCTGCCCCGA2	-0,638864558	4,609129379	0,103251397	0,437720016	LADA vs T2D
hsa-miR-93-3p_ACTGCTGAGCTAGCACTTCCCGA2	-0,877095448	3,371774968	0,105614801	0,440628181	LADA vs T2D
hsa-miR-584-5p_TTATGGTTTGCCTGGGACT2	-0,704241371	4,595669388	0,105760537	0,440628181	LADA vs T2D
hsa-miR-744-5p_TGCGGGGCTAGGGCTAACAGC2	-0,796567317	3,991128142	0,105943311	0,440628181	LADA vs T2D
hsa-miR-221-3p_AGCTACATTGTCTGCTGGGTTTC2	-0,489434052	6,752581432	0,106634151	0,440628181	LADA vs T2D
hsa-miR-361-3p_TCCCCCAGGTGTGATTCTGATT2	-0,762908367	3,650889743	0,10670168	0,440628181	LADA vs T2D
hsa-let-7a-5p_AGGTAGTAGGTTGTATAGTT2	-0,89569163	1,879378714	0,108196711	0,444196429	LADA vs T2D
hsa-let-7i-5p_TGAGGTAGTAGTTTGTGCTGTTG2	-0,821390175	3,894577335	0,108680431	0,444196429	LADA vs T2D
hsa-miR-181a-5p_AACATTCAACGCTGTCGGTGA2	-0,164613489	9,980124889	0,110462964	0,447124699	LADA vs T2D
hsa-miR-192-5p_TGACCTATGAATTGACAGCCA2	-0,747973654	4,726203979	0,110896666	0,447124699	LADA vs T2D
hsa-miR-151a-3p_CTAGACTGAAGCTCCTTGAGGA2	-0,275911056	8,444952042	0,112863848	0,447124699	LADA vs T2D
hsa-miR-145-5p_GTCCAGTTTTCCCAGGAATCCCT2	0,907893044	2,146327793	0,113235232	0,447124699	LADA vs T2D
hsa-miR-502-3p_AATGCACCTGGGCAAGGATTCA2	-0,644601383	4,709212787	0,113576239	0,447124699	LADA vs T2D
hsa-miR-339-3p_TGAGCGCCTCGACGACAGAG2	-0,637565727	5,154516456	0,113607868	0,447124699	LADA vs T2D
hsa-miR-150-5p_TCTCCCAACCCTTGTACCAG2	0,33836624	9,063580505	0,115134063	0,447124699	LADA vs T2D
hsa-let-7f-5p_TGAGGTAGTAGATTGTATAG2	-0,182972611	10,59314063	0,115236016	0,447124699	LADA vs T2D
hsa-miR-543_AAACATTCGCGGTGCACTTCTTT2	-0,959322325	3,173881718	0,115518999	0,447124699	LADA vs T2D
hsa-miR-10a-5p_TACCCTGTAGATCCGAAT2	0,898662801	2,33323359	0,115537187	0,447124699	LADA vs T2D
hsa-miR-16-2-3p_CCAATATTACTGTGCTGCTT2	-0,61998694	4,446564737	0,116624136	0,447124699	LADA vs T2D
hsa-miR-101-3p_TACAGTACTGTGATAACTGA2	-0,78406766	4,333316093	0,11703889	0,447124699	LADA vs T2D
hsa-miR-335-5p_TCAAGAGCAATAACGAAAAAT2	0,470888031	6,715048961	0,117623879	0,447124699	LADA vs T2D
hsa-miR-335-3p_TTTTTCATTATTGCTCCTGACC2	-0,843845019	4,076732498	0,118127381	0,447124699	LADA vs T2D
hsa-miR-30a-5p_TGTAAACATCCTCGACTGGAAGCT2	0,188446542	8,778765877	0,118130634	0,447124699	LADA vs T2D
hsa-miR-30d-5p_GTAAACATCCCCGACTGGAAGC2	-0,66788745	4,874885211	0,11879781	0,447124699	LADA vs T2D
hsa-miR-183-5p_TATGGCACTGGTAGAATTCA2	-0,431345277	7,32183424	0,118934048	0,447124699	LADA vs T2D
hsa-miR-320a-3p_AAAGCTGGGTTGAGAGGGCGA2	-0,734587447	4,98279454	0,120074388	0,448117095	LADA vs T2D
hsa-miR-183-5p_ATGGCACTGGTAGAATTCACTGT2	-0,857160181	2,224937851	0,12052591	0,448117095	LADA vs T2D
hsa-miR-451a_CCGTTACCATTACTGAGT2	-0,743342584	4,189894806	0,120884787	0,448117095	LADA vs T2D
hsa-miR-24-3p_GGCTCAGTTCAGCAGGAACAG2	-0,350766436	6,812383366	0,122277278	0,45118051	LADA vs T2D
hsa-miR-1301-3p_TTGCAGCTGCCTGGGAGTGACTTC2	-0,892753585	2,697320641	0,123927085	0,454269161	LADA vs T2D
hsa-miR-27a-3p_TTCACAGTGGCTAAGTTCCGC2	-0,420819949	6,28650114	0,1242543	0,454269161	LADA vs T2D
hsa-miR-143-3p_TGAGATGAAGCACTGTAGCTC2	-0,231363153	9,842973082	0,124903108	0,454509461	LADA vs T2D
hsa-miR-185-5p_TGGAGAGAAAGGCAGTTCCTG2	-0,740210769	4,508281621	0,125460579	0,454509461	LADA vs T2D
hsa-miR-10b-5p_TACCCTGTAGAACCGAATTTGTG2	0,307424227	7,78613959	0,126647409	0,456732964	LADA vs T2D
hsa-miR-186-5p_CAAAGAATTCTCCTTTTGGGCTT2	-0,476317824	6,345307365	0,128511065	0,461366299	LADA vs T2D
hsa-miR-15b-5p_TAGCAGCACATCATGGTT2	-0,373601608	7,417344831	0,129690505	0,461712126	LADA vs T2D

hsa-miR-23a-3p_ATCACATTGCCAGGGATTT2	0,258081004	7,810237088	0,131161713	0,461712126	LADA vs T2D
hsa-miR-191-5p_CAACGGAATCCCAAAAGCAG2	0,163129377	10,17632421	0,13183028	0,461712126	LADA vs T2D
hsa-miR-92b-3p_TATTGCACTCGTCCCGGCC2	-0,782673967	3,837730088	0,132150609	0,461712126	LADA vs T2D
hsa-miR-92a-3p_GCACTTGTCCCGGCCTGT2	-0,628878076	4,476222162	0,13221112	0,461712126	LADA vs T2D
hsa-miR-30e-3p_CTTTCAGTCGGATGTTTACAG2	-0,819653531	2,31966353	0,132408872	0,461712126	LADA vs T2D
hsa-miR-132-3p_TAACAGTCTACAGCCATGGTCG2	-0,818253874	2,233528136	0,132662581	0,461712126	LADA vs T2D
hsa-miR-339-3p_TGAGCGCCTCGACGACAGAGCCG2	-0,812508355	4,16247252	0,133648426	0,46312085	LADA vs T2D
hsa-miR-22-3p_AAGCTGCCAGTTGAAGAACTGT2	-0,124401471	12,01293	0,134818789	0,465154005	LADA vs T2D
hsa-miR-6803-3p_TCCCTCGCCTTCTCACCCTCA2	0,469801213	6,298415012	0,136370161	0,468478528	LADA vs T2D
hsa-miR-191-5p_CAACGGAATCCCAAAAGCAGC2	-0,278933597	8,150484012	0,137577026	0,470596092	LADA vs T2D
hsa-miR-2110_TTGGGGAAACGGCCGCTGAG2	-0,810740595	2,165591375	0,139108245	0,473800304	LADA vs T2D
hsa-miR-199a-5p_CCCAGTGTTCAGACTACCTGTTC2	-0,699321287	4,567460238	0,141893495	0,480401326	LADA vs T2D
hsa-miR-451a_AAACCGTTACCATTACTGAGTTT2	-0,231127369	8,99265157	0,143880922	0,480401326	LADA vs T2D
hsa-miR-421_ATCAACAGACATTAATTGGGCG2	-0,73294592	2,09434672	0,143887351	0,480401326	LADA vs T2D
hsa-miR-30d-5p_TGTAAACATCCCCGACTGGAA2	-0,140315553	11,66083446	0,14476094	0,480401326	LADA vs T2D
hsa-miR-125b-5p_TCCCTGAGACCCTAACTTGT2	0,205243756	9,802438562	0,145324067	0,480401326	LADA vs T2D
hsa-miR-574-3p_CACGCTCATGCACACACCCA2	-0,776328479	3,365756558	0,145715926	0,480401326	LADA vs T2D
hsa-miR-26b-5p_TTCAAGTAATTCAGGATAGGT2	-0,281038421	7,343481575	0,146429225	0,480401326	LADA vs T2D
hsa-let-7d-3p_TATACGACCTGCTGCCTTTCT2	0,183436176	8,488338468	0,146714415	0,480401326	LADA vs T2D
hsa-miR-30c-5p_TGTAAACATCCTACACTCTC2	-0,801261214	2,069916362	0,147662736	0,480401326	LADA vs T2D
hsa-miR-23b-3p_ATCACATTGCCAGGGATTAC2	-0,757861814	2,694770844	0,148009465	0,480401326	LADA vs T2D
hsa-miR-140-3p_TACCACAGGGTAGAACCACG2	-0,542072375	5,026346909	0,148084337	0,480401326	LADA vs T2D
hsa-miR-223-3p_TCAGTTTGTCAAATACCCCAA2	-0,773566273	2,060154898	0,148279456	0,480401326	LADA vs T2D
hsa-miR-486-5p_CTGTACTGAGCTGCCCCG2	-0,784575449	3,40982355	0,149039251	0,480908028	LADA vs T2D
hsa-miR-10a-5p_TACCCTGTAGATCCGAATTTGT2	0,160525863	10,44682905	0,150030242	0,481264879	LADA vs T2D
hsa-miR-3613-5p_TGTTGTACTTTTTTTTTTGTT2	-0,552424465	5,269511804	0,150357534	0,481264879	LADA vs T2D
hsa-miR-16-5p_TAGCAGCACGTAAATATTGGCG2	-0,156144037	14,03055319	0,152323118	0,484495114	LADA vs T2D
hsa-miR-223-3p_TGTCAGTTTGTCAAATACCCCA2	-0,250070375	10,41019198	0,152582527	0,484495114	LADA vs T2D
hsa-miR-320a-3p_GAAAAGCTGGGTTGAGAGGGCG2	-0,441038171	6,114738168	0,156492179	0,490482916	LADA vs T2D
hsa-miR-125a-5p_TCCCTGAGACCCTTTAACCT2	0,341694269	6,34415738	0,156577682	0,490482916	LADA vs T2D
hsa-miR-99b-5p_CACCCGTAGAACCGACCTTGCG2	0,220258886	9,378733966	0,156742442	0,490482916	LADA vs T2D
hsa-miR-4433b-5p_TATGTCCCACCCCCACTCCTG2	0,885245058	3,068037559	0,156929917	0,490482916	LADA vs T2D
hsa-miR-148a-3p_CAGTGCACTACAGAACTTTGT2	-0,794519735	2,126116505	0,160598523	0,498338109	LADA vs T2D
hsa-miR-181b-5p_AACATTCATTGCTGTCGGTGG2	-0,510543421	5,135854331	0,160693719	0,498338109	LADA vs T2D
hsa-miR-425-5p_ATGACACGATCACTCCCGTTGA2	-0,62314998	4,729605379	0,162832426	0,503013348	LADA vs T2D
hsa-miR-10b-5p_ACCCTGTAGAACCGAATTTGT2	0,299066572	6,785991828	0,164119116	0,505030639	LADA vs T2D
hsa-miR-340-3p_TCCGTCTCAGTTACTTTATAGCC2	-0,779030725	3,094594983	0,165522483	0,506983171	LADA vs T2D
hsa-let-7a-5p_TTGAGGTAGTAGGTTGTATAGTT2	-0,756622976	1,956823269	0,166565495	0,506983171	LADA vs T2D
hsa-miR-125b-5p_TCCCTGAGACCCTAACT2	-0,831790495	2,233733889	0,16722251	0,506983171	LADA vs T2D
hsa-miR-484_TCAGGCTCAGTCCCCTCCCGA2	-0,220051148	10,80053241	0,167564359	0,506983171	LADA vs T2D
hsa-miR-16-5p_GCAGCACGTAAATATTGGCG2	-0,665848576	3,625120505	0,1679342	0,506983171	LADA vs T2D
hsa-miR-148a-3p_TCAGTGCACTACAGAACTTTG2	-0,273846433	7,287277231	0,16965137	0,508153288	LADA vs T2D
hsa-miR-4433b-5p_ATGTCCCACCCCCACTCCTGT2	0,489276028	7,752662063	0,170562471	0,508153288	LADA vs T2D
hsa-miR-194-5p_TGTAACAGCAACTCCATGTGG2	-0,334437624	6,660853419	0,170572125	0,508153288	LADA vs T2D
hsa-let-7i-5p_GAGGTAGTAGTTTGTGCTG2	0,765385991	2,272109077	0,170872122	0,508153288	LADA vs T2D
hsa-miR-144-5p_GGATATCATCATATACTGTAA2	-0,74101368	3,901874288	0,172530671	0,511178232	LADA vs T2D
hsa-miR-329-3p_ACACACCTGGTTAACCTCTTTT2	0,7924178	2,079797897	0,173591725	0,512417054	LADA vs T2D
hsa-miR-106b-5p_TAAAGTGCTGACAGTGCAGA2	-0,528268511	5,144709005	0,174356118	0,512774265	LADA vs T2D
hsa-miR-223-3p_TGTCAGTTTGTCAAATACC2	-0,526414209	5,409428462	0,175045952	0,512804236	LADA vs T2D
hsa-miR-3173-5p_TGCCCTGCCTGTTTTCTCCTT2	0,749942423	2,97364428	0,175653145	0,512804236	LADA vs T2D
hsa-miR-143-3p_TGAGATGAAGCACTGTAGCTCA2	-0,385694858	6,738882834	0,177837996	0,516944678	LADA vs T2D
hsa-miR-320a-3p_AAAGCTGGGTTGAGAGGGCG2	-0,569431813	4,890613401	0,178509239	0,516944678	LADA vs T2D
hsa-miR-146b-5p_TGAGAACTGAATTCCATAGGCTGT2	-0,224690977	8,368012324	0,179557798	0,516944678	LADA vs T2D
hsa-miR-16-5p_TTAGCAGCACGTAAATATTGGCG2	-0,64755636	4,199452303	0,179879967	0,516944678	LADA vs T2D
hsa-miR-191-5p_AACGGAATCCCAAAAGCAGCTG2	-0,425923113	5,747755673	0,180314455	0,516944678	LADA vs T2D
hsa-miR-224-5p_CAAGTCACTAGTGGTTCCGTTTAG2	-0,714447721	4,65780231	0,182424447	0,519603889	LADA vs T2D
hsa-miR-10b-5p_TACCCTGTAGAACCGAATT2	-0,631698619	4,675674869	0,182674854	0,519603889	LADA vs T2D
hsa-miR-4433b-5p_ATGTCCCACCCCCACTCCTGTT2	0,49122802	8,226543503	0,183197858	0,519603889	LADA vs T2D
hsa-miR-328-3p_CTGGCCCTCTCTGCCCTTCCGT2	-0,283812121	7,228551867	0,186852105	0,526472643	LADA vs T2D
hsa-miR-942-5p_TCTTCTCTGTTTTGGCCATGT2	-0,732303067	2,592733465	0,186940725	0,526472643	LADA vs T2D
hsa-miR-7-5p_TGGAAGACTAGTGATTTTGTTGT2	-0,49866252	6,176558156	0,188134821	0,5269847	LADA vs T2D

hsa-miR-3613-5p_TGTTGTACTTTTTTTTTTGTTC2	-0,570244502	4,388236924	0,188444968	0,5269847	LADA vs T2D
hsa-miR-501-3p_AATGCACCCGGGCAAGGATTC2	-0,701432829	3,102124992	0,189345946	0,527652864	LADA vs T2D
hsa-let-7a-5p_GAGGTAGTAGGTTGTATAGTT2	0,325842561	7,081547935	0,192781628	0,535355253	LADA vs T2D
hsa-miR-101-3p_TACAGTACTGTGATAACTGAA2	-0,710000867	3,070531279	0,198063261	0,543684814	LADA vs T2D
hsa-miR-148a-3p_TCAGTGCACTACAGAACTTTGT2	-0,174962213	9,289411705	0,198192647	0,543684814	LADA vs T2D
hsa-miR-409-3p_CGAATGTTGCTCGGTGAACCCCTTT2	-0,78941762	2,598256943	0,198244831	0,543684814	LADA vs T2D
hsa-miR-222-3p_AGCTACATCTGGCTACTGGGTCTC2	-0,501537855	6,101293098	0,198665127	0,543684814	LADA vs T2D
hsa-miR-3615_TCTCTCGGCTCCTCGCGGCTCG2	0,171274707	8,091694617	0,199191927	0,543684814	LADA vs T2D
hsa-miR-409-3p_CGAATGTTGCTCGGTGAACCCCT2	-0,636293413	5,738161577	0,201579814	0,54506876	LADA vs T2D
hsa-miR-4685-3p_TCTCCCTTCCTGCCCTGGCT2	0,566809147	4,862788515	0,202499857	0,54506876	LADA vs T2D
hsa-let-7c-5p_TGAGGTAGTAGGTTGTATGGTTT2	-0,627336147	3,566946574	0,202572306	0,54506876	LADA vs T2D
hsa-miR-182-5p_TTTGGCAATGGTAGAACTCACACTG2	-0,72048097	3,598930548	0,202707536	0,54506876	LADA vs T2D
hsa-let-7d-3p_CTATACGACCTGCTGCCTT2	-0,45988297	4,847124715	0,203525188	0,54506876	LADA vs T2D
hsa-miR-29a-3p_TAGCACCATCTGAAATCGG2	-0,303674183	7,213630267	0,203802372	0,54506876	LADA vs T2D
hsa-miR-361-3p_TCCCCCAGGTGTGATTCTGATTTG2	-0,44452012	5,241633262	0,204903763	0,546181603	LADA vs T2D
hsa-miR-92a-3p_TTGCACTTGTCCCGGCCTG2	-0,593983166	4,313701526	0,208012512	0,552619907	LADA vs T2D
hsa-miR-125b-5p_TCCCTGAGACCCTAACTTG2	0,205636392	8,171400371	0,209286601	0,554157546	LADA vs T2D
hsa-miR-1306-5p_CCACCTCCCCTGCAAACGT2	0,750947894	2,734536196	0,211583944	0,55713097	LADA vs T2D
hsa-miR-425-5p_AATGACACGATCACTCCCGTT2	-0,251100004	8,024407696	0,211924047	0,55713097	LADA vs T2D
hsa-miR-16-5p_TAGCAGCACGTAAATATTGG2	-0,163205716	10,97240393	0,212506668	0,55713097	LADA vs T2D
hsa-miR-215-5p_ATGACCTATGAATTGACAGA2	0,626173133	4,790158483	0,214550231	0,560644374	LADA vs T2D
hsa-miR-361-3p_TCCCCCAGGTGTGATTCTGA2	-0,639296004	3,113214743	0,215546324	0,561406601	LADA vs T2D
hsa-miR-181a-5p_AACATTCAACGCTGTCGGTGAGTT2	-0,688096511	2,334562434	0,216970699	0,563275724	LADA vs T2D
hsa-miR-99a-5p_AACCCGTAGATCCGATCTTGTG2	-0,443930361	5,156061862	0,218350059	0,565016223	LADA vs T2D
hsa-miR-885-5p_TCCATTACACTACCCTGCCTCT2	-1,3215924	4,714772841	0,220670223	0,568575649	LADA vs T2D
hsa-miR-10b-5p_ACCCTGTAGAACCGAATTTGTGT2	-0,629050352	3,356457419	0,221152386	0,568575649	LADA vs T2D
hsa-miR-32-5p_TATTGCACATTACTAAGTTG2	-0,622231257	3,984130302	0,226664359	0,574945319	LADA vs T2D
hsa-miR-15b-3p_CGAATCATTATTTGCTGCTCT2	-0,530704611	4,350054835	0,226743456	0,574945319	LADA vs T2D
hsa-miR-424-5p_CAGCAGCAATTCATGTTTTGA2	0,669056686	2,646439112	0,226782094	0,574945319	LADA vs T2D
hsa-miR-2110_TTGGGGAAACGGCCGCTGAGTGA2	-0,371271069	6,066188802	0,227003915	0,574945319	LADA vs T2D
hsa-let-7i-5p_GAGGTAGTAGTTTGTGCTGTT2	-0,666005664	3,257044134	0,227236858	0,574945319	LADA vs T2D
hsa-miR-6803-3p_TCCCTCGCCTTCTCACCCTCAG2	-0,636163213	2,710817974	0,229271405	0,576311301	LADA vs T2D
hsa-miR-122-5p_TGGAGTGTGACAATGGTGTTT2	-0,311497497	13,55430102	0,229821991	0,576311301	LADA vs T2D
hsa-miR-103a-3p_AGCAGCATTGTACAGGGC2	0,795327014	3,081760646	0,22994604	0,576311301	LADA vs T2D
hsa-miR-375-3p_TTGTTCGTTCGGCTCGCGTGA2	-0,485517032	5,662970821	0,230989728	0,577112268	LADA vs T2D
hsa-miR-125b-5p_TCCCTGAGACCCTAACTTGTG2	-0,500253994	5,039062696	0,232683765	0,579528002	LADA vs T2D
hsa-miR-30c-5p_TGTAAACATCCTACACTCTCAGC2	-0,320427566	6,545485648	0,235039096	0,58357059	LADA vs T2D
hsa-miR-125a-5p_TCCCTGAGACCCTTTAACCTGTG2	0,180544655	8,600810288	0,237787539	0,586755057	LADA vs T2D
hsa-miR-16-5p_AGCAGCACGTAAATATTGGCG2	-0,185397191	8,17366975	0,237794082	0,586755057	LADA vs T2D
hsa-miR-150-5p_TCTCCCAACCCTTGTACCAGTG2	0,176415022	10,93881631	0,238774525	0,587355852	LADA vs T2D
hsa-miR-22-3p_AAGCTGCCAGTTGAAGAA2	-0,545002588	4,931866064	0,242430069	0,594513123	LADA vs T2D
hsa-miR-584-5p_TTATGGTTTGCCTGGGACTGA2	-0,229090007	8,070467342	0,244923972	0,598786521	LADA vs T2D
hsa-miR-629-5p_TGGGTTTACGTTGGGAGAACTT2	-0,569085332	4,034612462	0,246497503	0,600790551	LADA vs T2D
hsa-miR-92a-3p_ATTGCACTTGTCCCGGCCT2	-0,556506012	4,477303001	0,249261942	0,604346318	LADA vs T2D
hsa-miR-193a-5p_TGGGTCTTTGCGGGCGAGAT2	-0,505576154	4,669337288	0,249902395	0,604346318	LADA vs T2D
hsa-miR-152-3p_TCAGTGCATGACAGAACTTGG2	-0,585302874	2,073986305	0,250231223	0,604346318	LADA vs T2D
hsa-miR-192-5p_CTGACCTATGAATTGACAGC2	-0,641468978	1,977431527	0,252011612	0,606807417	LADA vs T2D
hsa-miR-21-5p_GTAGCTTATCAGACTGATGTTGA2	-0,641863215	2,217922154	0,253399339	0,607911603	LADA vs T2D
hsa-miR-186-5p_AAAGAATTCTCCTTTTGGGCT2	-0,595863927	3,890936215	0,253995689	0,607911603	LADA vs T2D
hsa-miR-181a-5p_AACATTCAACGCTGTCGGTG2	-0,276410958	6,983532365	0,255366314	0,609362133	LADA vs T2D
hsa-let-7a-5p_GAGGTAGTAGGTTGTATAGT2	-0,523891475	4,701370889	0,256583345	0,610438584	LADA vs T2D
hsa-miR-10b-5p_TACCCTGTAGAACCGAAT2	-0,631029939	2,220184724	0,259387188	0,615272585	LADA vs T2D
hsa-miR-1301-3p_TTGCAGCTGCCTGGGAGTGA2	-0,611069505	3,347749467	0,260312862	0,615636057	LADA vs T2D
hsa-miR-652-3p_AATGGCGCCACTAGGGTTGT2	-0,173984424	7,904624885	0,261965971	0,616081772	LADA vs T2D
hsa-miR-142-5p_CCCATAAAGTAGAAAGCACTA2	-0,401307264	5,405299379	0,263092365	0,616081772	LADA vs T2D
hsa-miR-126-3p_TCGTACCGTGAGTAATAATGC2	-0,261356447	6,426310401	0,263266621	0,616081772	LADA vs T2D
hsa-miR-30e-5p_TGTAAACATCCTTGACTGGAA2	-0,238344343	6,536675988	0,26359333	0,616081772	LADA vs T2D
hsa-miR-10a-5p_ACCCTGTAGATCCGAATTTG2	0,34584567	5,718851669	0,265776677	0,619368455	LADA vs T2D
hsa-miR-24-3p_GGCTCAGTTCAGCAGGAAC2	0,667807956	2,859699768	0,267578382	0,621749185	LADA vs T2D
hsa-miR-26a-5p_TTCAAGTAATCCAGGATAG2	-0,565900184	3,646262645	0,271272207	0,627075883	LADA vs T2D
hsa-let-7a-5p_TGAGGTAGTAGGTTGTATAGT2	0,107352453	11,86713156	0,271444391	0,627075883	LADA vs T2D

hsa-miR-574-3p_CACGCTCATGCACACACCCACA2	-0,311312237	6,300877473	0,273234472	0,627817978	LADA vs T2D
hsa-miR-140-3p_ACCACAGGGTAGAACCACG2	-0,590560323	2,665108795	0,273341077	0,627817978	LADA vs T2D
hsa-miR-4433b-5p_TATGTCCCACCCCCACTCCTGT2	-0,51612798	5,397905397	0,276085207	0,629223831	LADA vs T2D
hsa-miR-222-3p_AGCTACATCTGGCTACTGGGT2	-0,210104523	7,595221992	0,2773481	0,629223831	LADA vs T2D
hsa-miR-30d-5p_GTAAACATCCCCGACTGGAA2	-0,432789435	4,975135303	0,277450692	0,629223831	LADA vs T2D
hsa-miR-342-3p_TCTCACACAGAAATCGCACCCGTC2	0,210791563	7,583667414	0,277557635	0,629223831	LADA vs T2D
hsa-miR-486-5p_TCCTGTACTGAGCTGCCCCGA2	0,148652435	17,03890679	0,278892536	0,629223831	LADA vs T2D
hsa-miR-3615_TCTCTCGGCTCCTCGCGGCTCGC2	0,533171219	3,835837244	0,279137649	0,629223831	LADA vs T2D
hsa-miR-379-5p_TGGTAGACTATGGAACGTAGG2	-0,6317359	3,839701767	0,279479594	0,629223831	LADA vs T2D
hsa-miR-23b-3p_ATCACATTGCCAGGGATTACCAC2	-0,490582611	5,113281158	0,281141808	0,631183158	LADA vs T2D
hsa-miR-450b-5p_TTTTGCAATATGTTCCTGAAT2	-0,462220804	5,016886951	0,282381326	0,63218516	LADA vs T2D
hsa-miR-363-3p_AATTGCACGGTATCCATCTGT2	-0,156345104	8,977144446	0,283986407	0,633997666	LADA vs T2D
hsa-miR-28-3p_ACTAGATTGTGAGCTCCTGGAG2	-0,483507962	4,615150746	0,286130522	0,637000072	LADA vs T2D
hsa-let-7f-5p_TGAGGTAGTAGATTGTAT2	-0,624348527	3,174737293	0,287167976	0,637528904	LADA vs T2D
hsa-miR-432-5p_TCTTGGAGTAGGTCATTGGGTGG2	-0,646057967	3,502755168	0,288648717	0,638095107	LADA vs T2D
hsa-miR-92a-3p_CACTTGTCCCGGCCTGT2	-0,53457063	4,072780053	0,289024258	0,638095107	LADA vs T2D
hsa-miR-10a-5p_ACCCTGTAGATCCGAATTTGT2	0,224681436	7,643285241	0,28992556	0,638316772	LADA vs T2D
hsa-miR-423-3p_AGCTCGGTCTGAGGCCCC2	-0,548727811	3,540860079	0,292815211	0,642902819	LADA vs T2D
hsa-miR-486-5p_TCCTGTACTGAGCTGCCCCGAGC2	-0,22525909	6,188232343	0,294673709	0,645205895	LADA vs T2D
hsa-let-7b-5p_TGAGGTAGTAGGTTGTGTGGTT2	-0,09513577	12,77995871	0,295796028	0,645888861	LADA vs T2D
hsa-miR-342-5p_AGGGGTGCTATCTGTGATTGA2	-0,594347397	3,958584465	0,297355549	0,647520144	LADA vs T2D
hsa-miR-1908-5p_CGGCGGGGACGGCGATTGGTC2	0,590957845	2,612171571	0,29846102	0,648156492	LADA vs T2D
hsa-miR-1307-3p_CTCGGCGTGGCGTCGGTCGTGG2	-0,592962845	2,190246459	0,300107016	0,649960031	LADA vs T2D
hsa-miR-92a-3p_TATTGCACTTGTCCCGGCC2	-0,20683112	8,088512139	0,301357084	0,650898634	LADA vs T2D
hsa-miR-30c-5p_GTAAACATCCTACACTCTCAGCT2	-0,573683196	3,052199181	0,303045031	0,652775377	LADA vs T2D
hsa-miR-32-5p_TATTGCACATTACTAAGTTGC2	-0,563835229	2,00688677	0,304161803	0,653414979	LADA vs T2D
hsa-miR-485-3p_GTCATACACGGCTCTCCTCTCT2	-0,510582366	5,913980943	0,311597477	0,664851213	LADA vs T2D
hsa-miR-451a_AAACCGTTACCATTACTGA2	-0,176314235	8,929457213	0,311941968	0,664851213	LADA vs T2D
hsa-miR-339-5p_TCCCTGTCCTCCAGGAGCTCACG2	-0,562063464	3,416847552	0,311987897	0,664851213	LADA vs T2D
hsa-miR-323b-3p_CCCAATACACGGTCGACCTCT2	-0,479181128	5,853793118	0,315112102	0,669718255	LADA vs T2D
hsa-miR-342-3p_TCACACAGAAATCGCACCCGT2	0,53772587	2,35866661	0,316483869	0,669823939	LADA vs T2D
hsa-miR-223-3p_GTCAGTTTGTCAAATACCCCA2	-0,297641595	6,454169749	0,316842691	0,669823939	LADA vs T2D
hsa-miR-142-5p_CATAAAGTAGAAAGCACTAC2	-0,546410092	2,376484394	0,319984829	0,673667442	LADA vs T2D
hsa-miR-486-5p_TCCTGTACTGAGCTGCCCC2	-0,211319327	6,793601467	0,320351268	0,673667442	LADA vs T2D
hsa-miR-126-3p_TCGTACCGTGAGTAATAATG2	0,299486756	5,867396204	0,321593858	0,674500803	LADA vs T2D
hsa-let-7f-5p_TGAGGTAGTAGATTGTATAGTTG2	-0,566311057	2,64125494	0,324516323	0,674952054	LADA vs T2D
hsa-miR-451a_AAACCGTTACCATTACTGAGTTTAG2	-0,534429555	2,164703683	0,324848497	0,674952054	LADA vs T2D
hsa-let-7a-5p_TGAGGTAGTAGGTTGTATAGTT2	0,133697158	13,39549393	0,325545834	0,674952054	LADA vs T2D
hsa-miR-423-5p_TGAGGGGCAGAGAGCGAGACTT2	0,090019783	12,19565674	0,325854463	0,674952054	LADA vs T2D
hsa-miR-29a-3p_CTAGCACCATCTGAAATCGGTT2	-0,476465824	3,457099062	0,326043339	0,674952054	LADA vs T2D
hsa-miR-451a_AACCGTTACCATTACTGAGT2	-0,149871431	10,87302044	0,327190635	0,675572373	LADA vs T2D
hsa-miR-140-3p_TACCACAGGGTAGAACCACGGAC2	-0,519490321	3,947173881	0,328575856	0,676679476	LADA vs T2D
hsa-miR-155-5p_TTAATGCTAATCGTGATAGGGGTT2	-0,557849374	2,993899159	0,329533536	0,676902651	LADA vs T2D
hsa-miR-423-3p_AGCTCGGTCTGAGGCCCCT2	-0,296390241	6,038348398	0,330890383	0,677942507	LADA vs T2D
hsa-miR-26a-5p_TCAAGTAATCCAGGATAGGCT2	-0,279173053	6,522605962	0,333162456	0,680847378	LADA vs T2D
hsa-miR-148b-3p_TCAGTGCATCACAGAACTTTGT2	-0,189647637	6,965383646	0,334324841	0,681275938	LADA vs T2D
hsa-miR-151a-3p_TACTAGACTGAAGCTCCTTGAGG2	-0,488444094	4,127573648	0,335669415	0,681275938	LADA vs T2D
hsa-let-7f-5p_TGAGGTAGTAGATTG2	-0,494935505	4,164688749	0,336835881	0,681275938	LADA vs T2D
hsa-miR-25-3p_CATTGCACTTGTCTCGGT2	-0,467810664	3,623128964	0,337055854	0,681275938	LADA vs T2D
hsa-miR-30a-5p_TGTAAACATCCTCGACTGGA2	0,504306762	3,19956619	0,338763088	0,681275938	LADA vs T2D
hsa-miR-23b-3p_ATCACATTGCCAGGGATTACC2	0,318025185	5,483511586	0,338824231	0,681275938	LADA vs T2D
hsa-miR-29b-3p_TAGCACCATTTGAAATCAGT2	-0,454535505	4,363760053	0,339355768	0,681275938	LADA vs T2D
hsa-miR-320a-3p_AAAAGCTGGGTTGAGAGGGCGAAA2	-0,260790338	6,744969015	0,342550243	0,681613721	LADA vs T2D
hsa-miR-144-3p_TACAGTATAGATGATGTAC2	-0,25241825	6,460339014	0,342629498	0,681613721	LADA vs T2D
hsa-miR-150-5p_GTCTCCCAACCCTTGTACCAGT2	-0,509696519	2,340151294	0,342799693	0,681613721	LADA vs T2D
hsa-miR-320b_AAAAGCTGGGTTGAGAGGGCAA2	-0,476437485	1,963551094	0,342944921	0,681613721	LADA vs T2D
hsa-miR-26b-5p_TCAAGTAATTCAGGATAGGTT2	-0,50110463	3,747955651	0,345202407	0,684393827	LADA vs T2D
hsa-miR-92a-3p_GTATTGCACTTGTCCCGGCCTG2	-0,508731448	3,065032086	0,347711555	0,68765784	LADA vs T2D
hsa-miR-486-5p_TCCTGTACTGAGCTGCCCCG2	0,14520816	14,12822837	0,354146289	0,698649981	LADA vs T2D
hsa-miR-451a_GAAACCGTTACCATTACTGAGT2	-0,210394534	6,98332366	0,358398807	0,705293455	LADA vs T2D
hsa-miR-23a-3p_ATCACATTGCCAGGGATTTCCA2	-0,125452502	11,15500363	0,360439861	0,707562978	LADA vs T2D

hsa-miR-335-5p_TCAAGAGCAATAACGAAAAATGT2	-0,30342976	6,149787911	0,367112955	0,718891954	LADA vs T2D
hsa-let-7f-5p_GAGGTAGTAGATTGTATAGTT2	-0,308206065	6,051011881	0,371879525	0,726441132	LADA vs T2D
hsa-miR-146a-5p_TGAGAACTGAATTCCATGGGTTG2	-0,179386751	7,878355546	0,373197514	0,727233297	LADA vs T2D
hsa-miR-487b-3p_TCGTACAGGGTCATCCACTTT2	-0,52498713	2,776617041	0,374950719	0,727861429	LADA vs T2D
hsa-miR-221-3p_AGCTACATTGTCTGCTGGGT2	-0,27078521	6,226471989	0,376557207	0,727861429	LADA vs T2D
hsa-miR-142-5p_CATAAAGTAGAAAGCACTA2	-0,451708809	2,153538022	0,376925457	0,727861429	LADA vs T2D
hsa-miR-92a-3p_TATTGCACTTGTCCCGGCCTGTT2	0,106281602	10,41769689	0,377172861	0,727861429	LADA vs T2D
hsa-miR-361-5p_TTATCAGAATCTCCAGGGGTAC2	-0,214473163	6,634693775	0,380906902	0,733015326	LADA vs T2D
hsa-miR-150-5p_TCTCCCAACCCTTGTACCAGT2	0,153558933	10,25222562	0,382812504	0,733015326	LADA vs T2D
hsa-miR-92a-3p_ATTGCACTTGTCCCGGCCTGT2	0,09174353	12,31240602	0,383696205	0,733015326	LADA vs T2D
hsa-miR-99a-5p_AACCCGTAGATCCGATCTTGT2	-0,142409819	8,529135227	0,385471553	0,733015326	LADA vs T2D
hsa-miR-199a-3p_ACAGTAGTCTGCACATTGGTTA2	-0,721275641	4,827856003	0,385982561	0,733015326	LADA vs T2D
hsa-miR-23a-5p_GGGGTTCCTGGGGATGGGATTT2	0,444875045	3,81817711	0,38689875	0,733015326	LADA vs T2D
hsa-miR-485-5p_AGAGGCTGGCCGTGATGAATTCG2	-0,459077375	4,543642396	0,387357105	0,733015326	LADA vs T2D
hsa-miR-30b-5p_TGTAAACATCCTACACTCAGCT2	-0,21876736	6,722043115	0,387640214	0,733015326	LADA vs T2D
hsa-miR-589-5p_TGAGAACCACGTCTGCTCTGA2	-0,470815437	2,896824842	0,388121038	0,733015326	LADA vs T2D
hsa-miR-1249-3p_ACGCCCTTCCCCCCCTTCTTCA2	0,523951457	2,195504284	0,389783526	0,734414823	LADA vs T2D
hsa-miR-3173-5p_TGCCCTGCCTGTTTTCTCCTTT2	-0,361859063	4,766192857	0,390804217	0,734601323	LADA vs T2D
hsa-miR-409-3p_CGAATGTTGCTCGGTGAACCCCTT2	-0,513575457	3,646109852	0,392918369	0,736496992	LADA vs T2D
hsa-miR-125a-5p_TCCCTGAGACCCTTTAACCTGTGA2	0,170932694	8,237628665	0,393660877	0,736496992	LADA vs T2D
hsa-miR-22-3p_AAGCTGCCAGTTGAAGAAC2	0,156556216	8,203664635	0,395820434	0,738116976	LADA vs T2D
hsa-miR-151a-3p_TACTAGACTGAAGCTCCTTGA2	-0,44591533	1,940948476	0,396379003	0,738116976	LADA vs T2D
hsa-miR-103a-3p_AGCAGCATTGTACAGGGCTATGA2	0,176440398	8,631709381	0,401757544	0,746388724	LADA vs T2D
hsa-miR-425-5p_ATGACACGATCACTCCCGTTGAGT2	0,376804392	4,72212581	0,405050274	0,748934915	LADA vs T2D
hsa-miR-345-5p_GCTGACTCCTAGTCCAGGGCTC2	-0,460908957	3,656556535	0,406183303	0,748934915	LADA vs T2D
hsa-miR-222-3p_AGCTACATCTGGCTACTGGGTC2	-0,415621927	3,362212617	0,406695054	0,748934915	LADA vs T2D
hsa-miR-425-5p_AATGACACGATCACTCCCGTTGAG2	-0,314379237	6,063293525	0,406886848	0,748934915	LADA vs T2D
hsa-miR-192-5p_TGACCTATGAATTGACAGCC2	-0,231612855	6,449264096	0,408846862	0,750808639	LADA vs T2D
hsa-miR-145-3p_ATTCCTGGAAATACTGTTCT2	-0,442526688	3,209907444	0,412813232	0,755091681	LADA vs T2D
hsa-miR-182-5p_TTTGGCAATGGTAGAACTCACACT2	0,182987621	7,651119737	0,413075183	0,755091681	LADA vs T2D
hsa-let-7g-5p_TGAGGTAGTAGTTTGTACAGTT2	0,105069552	11,23391478	0,414021411	0,755091681	LADA vs T2D
hsa-let-7i-5p_TGAGGTAGTAGTTTGTGCTG2	-0,118867394	10,07708045	0,415133855	0,755391969	LADA vs T2D
hsa-miR-28-3p_CACTAGATTGTGAGCTCCTGGAG2	-0,429102163	2,845963244	0,418695063	0,760136596	LADA vs T2D
hsa-miR-92a-3p_ATTGCACTTGTCCCGGCCTGTT2	-0,271877579	5,604292671	0,420803612	0,762228361	LADA vs T2D
hsa-miR-100-5p_AACCCGTAGATCCGAACTTGTG2	-0,458844874	3,590895666	0,424328651	0,7668706	LADA vs T2D
hsa-miR-18a-3p_ACTGCCCTAAGTGCTCCTTCT2	0,437771533	3,273299722	0,427627276	0,766965228	LADA vs T2D
hsa-miR-27a-3p_TCACAGTGGCTAAGTTCCG2	0,435829532	2,668037972	0,428251011	0,766965228	LADA vs T2D
hsa-miR-1908-5p_CGGCGGGGACGGCGATTGGT2	-0,468285829	2,686402078	0,428448857	0,766965228	LADA vs T2D
hsa-let-7b-5p_TGAGGTAGTAGGTTGTGT2	-0,292273597	5,292834315	0,428519119	0,766965228	LADA vs T2D
hsa-miR-22-3p_AGCTGCCAGTTGAAGAACT2	-0,447887283	2,67043052	0,430081658	0,766965228	LADA vs T2D
hsa-miR-532-5p_CATGCCTTGAGTGTAGGACCG2	-0,411057235	2,388684445	0,430154902	0,766965228	LADA vs T2D
hsa-miR-374b-5p_ATATAATACAACCTGCTAAGT2	-0,387370328	3,95574137	0,431574867	0,767779396	LADA vs T2D
hsa-miR-342-3p_TCACACAGAAATCGCACCCGTCA2	-0,409429539	3,930198822	0,4362102	0,769913108	LADA vs T2D
hsa-miR-223-5p_CGTGTATTTGACAAGCTGAGTT2	-0,365137282	3,93706767	0,43635852	0,769913108	LADA vs T2D
hsa-miR-423-5p_GAGGGGCAGAGAGCGAGACTT2	0,306478341	5,367382166	0,436878105	0,769913108	LADA vs T2D
hsa-miR-370-3p_GCCTGCTGGGGTGGAACCTGGT2	-0,462826121	2,498088509	0,436938574	0,769913108	LADA vs T2D
hsa-miR-423-5p_GAGGGGCAGAGAGCGAGACT2	-0,229854101	6,874414686	0,438032347	0,769913108	LADA vs T2D
hsa-miR-191-5p_CAACGGAATCCCAAAAGCAGCT2	0,095037428	11,11779618	0,438570327	0,769913108	LADA vs T2D
hsa-miR-654-3p_TATGTCTGCTGACCATCACC2	-0,455333465	2,711988254	0,441062275	0,771484185	LADA vs T2D
hsa-miR-27b-3p_TTCACAGTGGCTAAGTTCTG2	-0,134032679	8,279294906	0,44140124	0,771484185	LADA vs T2D
hsa-miR-191-5p_AACGGAATCCCAAAAGCAGCT2	-0,193018069	6,816281646	0,443040959	0,772655677	LADA vs T2D
hsa-miR-92a-3p_TATTGCACTTGTCCCGG2	-0,328586734	4,594134091	0,444235633	0,773047598	LADA vs T2D
hsa-miR-2110_TTGGGGAAACGGCCGCTGAGT2	-0,246400292	5,974874685	0,445536198	0,773621678	LADA vs T2D
hsa-miR-21-5p_TAGCTTATCAGACTGATGTTGA2	0,061278004	12,88530966	0,446605311	0,773792245	LADA vs T2D
hsa-miR-30e-5p_TGTAAACATCCTTGACTGG2	-0,327973434	5,076073317	0,454435571	0,784396848	LADA vs T2D
hsa-miR-183-5p_TATGGCACTGGTAGAATTCACTG2	-0,421813237	3,516695948	0,454694283	0,784396848	LADA vs T2D
hsa-miR-423-5p_TGAGGGGCAGAGAGCGAGA2	0,08305872	9,723922848	0,456903761	0,786506042	LADA vs T2D
hsa-miR-425-5p_AATGACACGATCACTCCCGT2	-0,180394108	6,738341736	0,461409392	0,790665816	LADA vs T2D
hsa-let-7d-5p_AGAGGTAGTAGGTTGCATAG2	-0,1337738	8,573362788	0,461976308	0,790665816	LADA vs T2D
hsa-let-7d-5p_AGAGGTAGTAGGTTGCATAGTTT2	-0,342045793	5,420694347	0,462296449	0,790665816	LADA vs T2D
hsa-miR-92a-3p_GTATTGCACTTGTCCCGGCCTGT2	0,123521643	7,715362913	0,464031663	0,791934122	LADA vs T2D

hsa-miR-1-3p_TGGAATGTAAAGAAGTATGTAT2	0,366347186	5,995923161	0,46537739	0,792533717	LADA vs T2D
hsa-miR-320a-3p_AAAAGCTGGGTTGAGAGGGCGAA2	0,083580579	9,770949231	0,466611324	0,792940778	LADA vs T2D
hsa-miR-4446-3p_CAGGGCTGGCAGTGACATGGGT2	-0,436939596	3,598813459	0,468509039	0,794471711	LADA vs T2D
hsa-miR-122-5p_TGGAGTGTGACAATGGTGTTTGT2	-0,220998314	8,198260953	0,471859661	0,796685059	LADA vs T2D
hsa-miR-660-5p_TACCCATTGCATATCGGAGTTGT2	-0,363591602	3,90808508	0,471890148	0,796685059	LADA vs T2D
hsa-miR-150-3p_CTGGTACAGGCCTGGGGGACA2	-0,374931797	4,060108896	0,473542531	0,796685059	LADA vs T2D
hsa-miR-23a-3p_ATCACATTGCCAGGGATTTC2	0,100384129	9,459376505	0,475760469	0,796685059	LADA vs T2D
hsa-miR-145-3p_ATTCCTGGAAATACTGTTCTT2	0,386187323	3,50539909	0,477241096	0,796685059	LADA vs T2D
hsa-miR-23a-3p_TCACATTGCCAGGGATTTCCA2	0,278635612	5,236791923	0,477518819	0,796685059	LADA vs T2D
hsa-miR-320a-3p_AAAAGCTGGGTTGAGAGGGCGAAAA2	-0,389434184	3,186668725	0,477654616	0,796685059	LADA vs T2D
hsa-miR-340-3p_TCCGTCTCAGTTACTTTATAGC2	-0,366923507	1,820184636	0,478474792	0,796685059	LADA vs T2D
hsa-miR-130b-5p_ACTCTTTCCCTGTTGCACTACT2	-0,247232906	5,742085569	0,478810719	0,796685059	LADA vs T2D
hsa-miR-30e-5p_GTAAACATCCTTGACTGGAAGCT2	0,074199599	8,781643893	0,488545449	0,8105525	LADA vs T2D
hsa-miR-342-3p_CTCACACAGAAATCGCACCCG2	0,393410182	2,495329338	0,490379498	0,8105525	LADA vs T2D
hsa-miR-142-5p_CCATAAAGTAGAAAGCACT2	-0,249287225	4,774985235	0,490426996	0,8105525	LADA vs T2D
hsa-miR-451a_AAACCGTTACCATTACTGAG2	-0,109341363	13,72832534	0,491213121	0,8105525	LADA vs T2D
hsa-miR-363-3p_ATTGCACGGTATCCATCTG2	-0,371474004	2,060471037	0,495946624	0,81456231	LADA vs T2D
hsa-miR-142-5p_CATAAAGTAGAAAGCACT2	-0,181703548	5,539841362	0,496119825	0,81456231	LADA vs T2D
hsa-miR-505-3p_GTCAACACTTGCTGGTTTCCTCT2	0,39074034	2,066469778	0,497442927	0,81456231	LADA vs T2D
hsa-miR-10a-5p_TACCCTGTAGATCCGAATTT2	-0,32780253	4,308439737	0,499388445	0,81456231	LADA vs T2D
hsa-miR-100-5p_AACCCGTAGATCCGAACTTGT2	-0,163044244	7,211971456	0,500467548	0,81456231	LADA vs T2D
hsa-let-7i-5p_TGAGGTAGTAGTTTGTGCTGTT2	-0,103199536	10,48466024	0,500628675	0,81456231	LADA vs T2D
hsa-miR-15a-5p_TAGCAGCACATAATGGTTTGT2	0,200407291	6,478410353	0,502461052	0,81456231	LADA vs T2D
hsa-miR-409-3p_AATGTTGCTCGGTGAACCCCT2	-0,376548292	4,366315065	0,503665979	0,81456231	LADA vs T2D
hsa-miR-486-5p_TCCTGTACTGAGCTGCCC2	-0,247505018	3,88539215	0,504796206	0,81456231	LADA vs T2D
hsa-let-7a-5p_ATGAGGTAGTAGGTTGTATAGTT2	-0,381363911	2,103462699	0,505944759	0,81456231	LADA vs T2D
hsa-miR-224-5p_CAAGTCACTAGTGGTTCCGTTT2	-0,367595444	2,885758309	0,505958461	0,81456231	LADA vs T2D
hsa-miR-144-3p_TACAGTATAGATGATGTACT2	-0,305759709	4,501383498	0,506732605	0,81456231	LADA vs T2D
hsa-miR-23a-3p_TCACATTGCCAGGGATTTCCAAC2	-0,341364877	2,126412114	0,506929618	0,81456231	LADA vs T2D
hsa-miR-486-5p_ATCCTGTACTGAGCTGCCCCGA2	0,111579419	8,988899635	0,508355155	0,815209373	LADA vs T2D
hsa-miR-29a-3p_TAGCACCATCTGAAATCGGTT2	0,112975946	8,641484144	0,511722126	0,816973288	LADA vs T2D
hsa-miR-485-5p_AGAGGCTGGCCGTGATGAATTC2	-0,392306664	2,300221452	0,512853832	0,816973288	LADA vs T2D
hsa-miR-146a-5p_TGAGAACTGAATTCCATGGGT2	-0,086518431	8,67354952	0,512985127	0,816973288	LADA vs T2D
hsa-miR-122-5p_GAGTGTGACAATGGTGTTT2	-0,393096759	2,929528654	0,513795636	0,816973288	LADA vs T2D
hsa-miR-92a-3p_TTGCACTTGTCCCGGCCTGT2	-0,089067105	8,384006303	0,514580415	0,816973288	LADA vs T2D
hsa-miR-486-5p_ATCCTGTACTGAGCTGCCCCGAG2	-0,124869564	7,990918242	0,518609715	0,821155398	LADA vs T2D
hsa-miR-3615_TCTCTCGGCTCCTCGCGGCTC2	0,324076196	2,193732344	0,519797892	0,821155398	LADA vs T2D
hsa-miR-92a-3p_ATTGCACTTGTCCCGGCCTG2	0,101645515	9,121941427	0,520904057	0,821155398	LADA vs T2D
hsa-miR-3173-5p_CCCTGCCTGTTTTCTCCTTTGT2	-0,324734224	1,879782778	0,523226943	0,821155398	LADA vs T2D
hsa-miR-486-5p_CCTGTACTGAGCTGCCCCGA2	0,094191892	11,08007536	0,526873263	0,821155398	LADA vs T2D
hsa-miR-21-5p_TAGCTTATCAGACTGATGTTGACT2	0,277905074	5,943834972	0,528616705	0,821155398	LADA vs T2D
hsa-miR-30e-5p_TAAACATCCTTGACTGGAAGCT2	0,348900191	2,076536633	0,529161027	0,821155398	LADA vs T2D
hsa-miR-30e-5p_TGTAAACATCCTTGACTGGA2	-0,340565584	2,511168014	0,533527386	0,821155398	LADA vs T2D
hsa-miR-192-5p_TGACCTATGAATTGACAGC2	-0,351483269	3,665629227	0,535328423	0,821155398	LADA vs T2D
hsa-miR-30a-5p_TGTAAACATCCTCGACTGGAAGC2	0,105601972	7,798673355	0,535654511	0,821155398	LADA vs T2D
hsa-miR-323a-3p_CACATTACACGGTCGACCTCT2	0,348752494	3,585538292	0,535810281	0,821155398	LADA vs T2D
hsa-let-7g-5p_GAGGTAGTAGTTTGTACAGTT2	-0,340081561	4,344091711	0,53657306	0,821155398	LADA vs T2D
hsa-miR-150-3p_CTGGTACAGGCCTGGGGGAC2	0,348667925	2,308717969	0,536599986	0,821155398	LADA vs T2D
hsa-miR-181a-5p_AACATTCAACGCTGTCGGTGAGT2	-0,117824603	8,031521361	0,537041957	0,821155398	LADA vs T2D
hsa-miR-191-5p_CAACGGAATCCCAAAAGCAGCTG2	-0,108254517	9,658129048	0,537586107	0,821155398	LADA vs T2D
hsa-miR-23a-5p_GGGGTTCCTGGGGATGGGATT2	-0,324936655	3,585240207	0,538047814	0,821155398	LADA vs T2D
hsa-miR-223-3p_TGTCAGTTTGTCAAATACCCC2	-0,172412656	7,314719746	0,53862188	0,821155398	LADA vs T2D
hsa-miR-103a-3p_AGCAGCATTGTACAGGGCT2	0,508126597	5,118424827	0,539029492	0,821155398	LADA vs T2D
hsa-miR-423-3p_AGCTCGGTCTGAGGCCCCTCAGT2	-0,074999116	11,76682308	0,539142167	0,821155398	LADA vs T2D
hsa-miR-92a-3p_ACTTGTCCCGGCCTGT2	0,341386499	2,134254049	0,539330978	0,821155398	LADA vs T2D
hsa-miR-19a-3p_TGTGCAAATCTATGCAAAACTGA2	-0,328789544	2,326203362	0,539647911	0,821155398	LADA vs T2D
hsa-miR-181b-5p_AACATTCATTGCTGTCGGTG2	0,335278279	2,058689202	0,540039569	0,821155398	LADA vs T2D
hsa-miR-486-5p_TCCTGTACTGAGCTGCCCCGAGG2	-0,107596011	7,467002764	0,541364987	0,821155398	LADA vs T2D
hsa-miR-1306-5p_CCACCTCCCCTGCAAACGTCCA2	0,35005705	3,283279778	0,541941957	0,821155398	LADA vs T2D
hsa-miR-148a-3p_TCAGTGCACTACAGAACTTT2	-0,320548052	4,280926656	0,54486514	0,823994339	LADA vs T2D
hsa-let-7a-5p_TGAGGTAGTAGGTTGTATAGTTT2	0,125765667	9,423297293	0,548019544	0,823994339	LADA vs T2D

hsa-miR-4433b-5p_TGTCCCACCCCCACTCCTG2	0,360772336	2,497163245	0,548400386	0,823994339	LADA vs T2D
hsa-miR-15b-5p_TAGCAGCACATCATGGTTT2	-0,098983703	8,514548835	0,550287796	0,823994339	LADA vs T2D
hsa-miR-451a_GAAACCGTTACCATTACTGAG2	-0,302267661	3,176702933	0,550735357	0,823994339	LADA vs T2D
hsa-miR-24-3p_TGGCTCAGTTCAGCAGGAACAG2	-0,062632741	12,76835022	0,553053232	0,823994339	LADA vs T2D
hsa-miR-191-5p_ACGGAATCCCAAAAGCAGCT2	-0,325720476	1,904502114	0,554057957	0,823994339	LADA vs T2D
hsa-miR-25-3p_CATTGCACTTGTCTCGGTCT2	-0,085195209	10,26006574	0,554418033	0,823994339	LADA vs T2D
hsa-miR-652-3p_AATGGCGCCACTAGGGTTG2	-0,30782563	4,499546053	0,554630698	0,823994339	LADA vs T2D
hsa-miR-375-3p_TTTGTTCGTTCGGCTCGCGTG2	-0,19466813	6,919662337	0,556743356	0,823994339	LADA vs T2D
hsa-miR-181a-5p_AACATTCAACGCTGTCGGT2	-0,210307193	5,505188114	0,556881342	0,823994339	LADA vs T2D
hsa-miR-501-3p_AATGCACCCGGGCAAGGAT2	-0,320121822	2,760223516	0,557505946	0,823994339	LADA vs T2D
hsa-miR-26a-5p_TTCAAGTAATCCAGGATAGG2	-0,122995751	6,813190078	0,557883256	0,823994339	LADA vs T2D
hsa-miR-29a-3p_TAGCACCATCTGAAATCGGT2	-0,106409537	8,663780214	0,559998193	0,823994339	LADA vs T2D
hsa-let-7e-5p_TGAGGTAGGAGGTTGTATAGT2	-0,264337795	4,645402812	0,56010517	0,823994339	LADA vs T2D
hsa-let-7d-3p_ATACGACCTGCTGCCTTTCT2	-0,317000717	2,048536833	0,560679601	0,823994339	LADA vs T2D
hsa-miR-486-5p_CCTGTACTGAGCTGCCCCG2	0,119425902	8,015321722	0,561391376	0,823994339	LADA vs T2D
hsa-miR-30d-5p_TGTAAACATCCCCGACTGGA2	-0,083072765	9,18587305	0,56558412	0,824918582	LADA vs T2D
hsa-miR-142-5p_CCCATAAAGTAGAAAGCACT2	-0,054568625	11,13638127	0,567107783	0,824918582	LADA vs T2D
hsa-miR-329-3p_AACACACCTGGTTAACCTCTTT2	0,31575524	3,759288959	0,567426055	0,824918582	LADA vs T2D
hsa-miR-128-3p_TCACAGTGAACCGGTCTCTTT2	-0,074684091	8,228392837	0,568686544	0,824918582	LADA vs T2D
hsa-miR-323a-3p_GCACATTACACGGTCGACCTCT2	-0,308303406	3,90036256	0,568768142	0,824918582	LADA vs T2D
hsa-miR-100-5p_AACCCGTAGATCCGAACT2	0,299756854	1,926738636	0,569537777	0,824918582	LADA vs T2D
hsa-miR-4433b-5p_TGTCCCACCCCCACTCCTGTT2	-0,247299857	6,729375179	0,569700968	0,824918582	LADA vs T2D
hsa-let-7a-5p_GTGAGGTAGTAGGTTGTATAGTT2	0,336736319	2,866169762	0,570434498	0,824918582	LADA vs T2D
hsa-miR-26b-5p_TTCAAGTAATTCAGGATAGGTT2	0,097747465	9,842825606	0,571336333	0,824918582	LADA vs T2D
hsa-miR-342-3p_TCTCACACAGAAATCGCACCCGT2	-0,105802566	8,706959622	0,572878879	0,825650029	LADA vs T2D
hsa-miR-125a-5p_CCCTGAGACCCTTTAACCTGT2	0,218529973	5,351640533	0,58086846	0,835653723	LADA vs T2D
hsa-miR-182-5p_TTTGGCAATGGTAGAACTCAC2	-0,29505256	3,148572393	0,582797914	0,836918807	LADA vs T2D
hsa-miR-423-5p_TGAGGGGCAGAGAGCGAGAC2	0,053798082	9,751921415	0,587441507	0,837847726	LADA vs T2D
hsa-miR-28-3p_CACTAGATTGTGAGCTCCTGG2	-0,168781708	5,876182682	0,588221955	0,837847726	LADA vs T2D
hsa-miR-30e-5p_TGTAAACATCCTTGACTGGAAG2	-0,092848785	7,619075745	0,58919811	0,837847726	LADA vs T2D
hsa-miR-99b-5p_CACCCGTAGAACCGACCTTG2	0,128690812	6,765454723	0,589302878	0,837847726	LADA vs T2D
hsa-miR-126-5p_ATTATTACTTTTGGTACGCGCT2	-0,291019268	2,122638684	0,589417083	0,837847726	LADA vs T2D
hsa-miR-223-5p_CGTGTATTTGACAAGCTGAGTTGG2	0,304155548	2,566442122	0,590549009	0,837847726	LADA vs T2D
hsa-miR-30a-5p_TGTAAACATCCTCGACTGGAAG2	0,093923112	8,337829601	0,590803541	0,837847726	LADA vs T2D
hsa-miR-30e-3p_CTTTCAGTCGGATGTTTACAGC2	-0,290526099	3,314941715	0,596722194	0,844738168	LADA vs T2D
hsa-miR-92a-3p_TATTGCACTTGTCCCGGCCTG2	0,072127369	13,95782504	0,598173755	0,845291636	LADA vs T2D
hsa-miR-145-5p_GTCCAGTTTTCCCAGGAATCCC2	-0,299448987	3,12605684	0,59940751	0,845535903	LADA vs T2D
hsa-miR-92a-3p_TATTGCACTTGTCCCGGCCTGT2	0,048723018	17,23184948	0,602962768	0,846911466	LADA vs T2D
hsa-miR-423-5p_GAGGGGCAGAGAGCGAGACTTT2	0,064662774	8,388365906	0,603461442	0,846911466	LADA vs T2D
hsa-miR-30c-5p_TGTAAACATCCTACACTCTCAG2	-0,224530838	4,767658059	0,603570531	0,846911466	LADA vs T2D
hsa-let-7f-5p_GAGGTAGTAGATTGTATAGT2	-0,296112813	3,023408308	0,607121787	0,850397301	LADA vs T2D
hsa-miR-125b-5p_CCCTGAGACCCTAACTTGT2	-0,28013228	2,290772166	0,60966948	0,85083868	LADA vs T2D
hsa-miR-128-3p_TCACAGTGAACCGGTCTCTTTT2	-0,191898314	5,404238817	0,609713161	0,85083868	LADA vs T2D
hsa-miR-21-5p_AGCTTATCAGACTGATGTTG2	-0,188841763	5,031654467	0,610639554	0,85083868	LADA vs T2D
hsa-miR-10b-5p_ACCCTGTAGAACCGAATTTGTG2	0,15473021	5,829978228	0,617612554	0,859052715	LADA vs T2D
hsa-miR-7-5p_TGGAAGACTAGTGATTTTGT2	-0,263980808	3,91952121	0,621035244	0,862074467	LADA vs T2D
hsa-miR-500a-3p_AATGCACCTGGGCAAGGATTCT2	0,288707597	2,780066987	0,622890895	0,862074467	LADA vs T2D
hsa-miR-128-3p_TCACAGTGAACCGGTCTCT2	-0,087356091	7,683472613	0,624245368	0,862074467	LADA vs T2D
hsa-miR-139-5p_TCTACAGTGCACGTGTCTCCAGT2	-0,126218187	6,587018882	0,624409842	0,862074467	LADA vs T2D
hsa-miR-126-3p_CGTACCGTGAGTAATAATGCG2	0,069311233	9,683358758	0,625193277	0,862074467	LADA vs T2D
hsa-miR-28-5p_AAGGAGCTCACAGTCTATTGA2	-0,284751713	3,11393162	0,63196404	0,868948547	LADA vs T2D
hsa-miR-29c-3p_TAGCACCATTTGAAATCGGTT2	-0,225844718	3,850371057	0,633335942	0,868948547	LADA vs T2D
hsa-miR-221-3p_AGCTACATTGTCTGCTGGGTTT2	-0,084344551	7,916859704	0,633449317	0,868948547	LADA vs T2D
hsa-miR-10b-5p_TACCCTGTAGAACCGAATTTGTGT2	-0,256442105	2,268128931	0,639358265	0,871399691	LADA vs T2D
hsa-miR-433-3p_ATCATGATGGGCTCCTCGGTGT2	0,266708531	4,303379714	0,63964368	0,871399691	LADA vs T2D
hsa-miR-143-3p_TGAGATGAAGCACTGTAGCT2	-0,077752163	9,047255511	0,639966609	0,871399691	LADA vs T2D
hsa-miR-423-3p_GCTCGGTCTGAGGCCCCTCAGT2	-0,141704965	6,432183419	0,640272261	0,871399691	LADA vs T2D
hsa-miR-29c-3p_TAGCACCATTTGAAATCGGT2	-0,236992236	4,339459305	0,642453094	0,871399691	LADA vs T2D
hsa-miR-4732-3p_GCCCTGACCTGTCCTGTTCTG2	0,397452003	4,156168491	0,642683807	0,871399691	LADA vs T2D
hsa-miR-3158-3p_AAGGGCTTCCTCTCTGCAGGA2	-0,268521634	2,734692583	0,642889609	0,871399691	LADA vs T2D
hsa-let-7a-5p_TGAGGTAGTAGGTTGTAT2	-0,304524101	3,216663859	0,648257755	0,877184092	LADA vs T2D

hsa-miR-374b-5p_ATATAATACAACCTGCTAAGTG2	-0,242157662	2,723967557	0,650447701	0,878252609	LADA vs T2D
hsa-miR-375-3p_TTTGTTCGTTCGGCTCGCG2	-0,17127201	5,830563474	0,651251308	0,878252609	LADA vs T2D
hsa-miR-532-3p_CCTCCCACACCCAAGGCTTG2	-0,252529203	2,745637111	0,65382099	0,880228596	LADA vs T2D
hsa-miR-144-5p_GATATCATCATATACTGTAAGTT2	0,250983524	2,459029553	0,656116223	0,881829055	LADA vs T2D
hsa-miR-4433b-5p_TGTCCCACCCCCACTCCTGTTT2	0,141430828	8,596146134	0,658236433	0,882080792	LADA vs T2D
hsa-miR-130a-3p_CAGTGCAATGTTAAAAGGGCA2	-0,198780255	4,214432691	0,658517028	0,882080792	LADA vs T2D
hsa-miR-423-5p_AGGGGCAGAGAGCGAGACTTT2	0,151008764	5,332499304	0,663655695	0,887472465	LADA vs T2D
hsa-miR-130b-3p_CAGTGCAATGATGAAAGGGCA2	0,182812088	4,137452675	0,666146895	0,888885962	LADA vs T2D
hsa-miR-126-3p_GTACCGTGAGTAATAATGCG2	0,231996539	2,067690175	0,667687202	0,888885962	LADA vs T2D
hsa-miR-451a_AAACCGTTACCATTACTG2	-0,217135967	2,491678877	0,668490475	0,888885962	LADA vs T2D
hsa-miR-26a-5p_TTCAAGTAATCCAGGATAGGC2	0,067994405	7,510198846	0,670367563	0,888885962	LADA vs T2D
hsa-miR-320a-3p_AAAAGCTGGGTTGAGAGGGCG2	-0,050898937	10,00052492	0,671243376	0,888885962	LADA vs T2D
hsa-miR-101-3p_GTACAGTACTGTGATAACTGAA2	-0,233696577	3,655178095	0,671404453	0,888885962	LADA vs T2D
hsa-miR-144-3p_CTACAGTATAGATGATGTAC2	0,224474414	3,24422031	0,674381466	0,890201294	LADA vs T2D
hsa-miR-3613-5p_TGTTGTACTTTTTTTTTTGT2	-0,065248516	7,586394808	0,677358531	0,890201294	LADA vs T2D
hsa-let-7f-5p_GAGGTAGTAGATTGTATAG2	-0,211609837	3,245137192	0,677996186	0,890201294	LADA vs T2D
hsa-miR-29c-3p_TAGCACCATTTGAAATCGG2	-0,222093136	3,272314395	0,678379027	0,890201294	LADA vs T2D
hsa-miR-22-3p_AAGCTGCCAGTTGAAGAACT2	-0,048574815	10,21399499	0,6812343	0,890201294	LADA vs T2D
hsa-miR-199a-3p_ACAGTAGTCTGCACATTGGT2	-0,230577344	1,952185074	0,683185201	0,890201294	LADA vs T2D
hsa-miR-191-5p_CAACGGAATCCCAAAAGCA2	-0,115108044	6,203677568	0,684221226	0,890201294	LADA vs T2D
hsa-miR-382-5p_GAAGTTGTTCGTGGTGGATTCG2	-0,137439064	7,607601722	0,684546812	0,890201294	LADA vs T2D
hsa-let-7g-5p_GAGGTAGTAGTTTGTACAG2	-0,227549353	2,076526463	0,685598591	0,890201294	LADA vs T2D
hsa-miR-22-3p_AAGCTGCCAGTTGAAGAACTGTT2	-0,217338609	2,793276847	0,687038393	0,890201294	LADA vs T2D
hsa-miR-92a-3p_TATTGCACTTGTCCCGGC2	-0,103606496	6,608463448	0,687315806	0,890201294	LADA vs T2D
hsa-miR-92a-3p_TGCACTTGTCCCGGCCTGT2	0,148122034	5,28105363	0,687377004	0,890201294	LADA vs T2D
hsa-miR-140-5p_CAGTGGTTTTACCCTATGGTAG2	-0,199008894	3,871751643	0,6876508	0,890201294	LADA vs T2D
hsa-miR-16-2-3p_ACCAATATTACTGTGCTGCTT2	-0,063423375	8,312552057	0,688035128	0,890201294	LADA vs T2D
hsa-miR-664a-3p_TATTCATTTATCCCCAGCCTACA2	0,211583015	3,022366025	0,689827072	0,890852436	LADA vs T2D
hsa-miR-424-3p_CAAAACGTGAGGCGCTGCTAT2	0,131588944	5,519904886	0,690773909	0,890852436	LADA vs T2D
hsa-miR-126-3p_TCGTACCGTGAGTAATAATGCG2	0,052991831	11,19647801	0,692172257	0,891213714	LADA vs T2D
hsa-miR-10a-5p_TACCCTGTAGATCCGAATTTGTG2	0,086581952	7,257876357	0,696337049	0,893472189	LADA vs T2D
hsa-miR-3615_TCTCTCGGCTCCTCGCGGCT2	0,103967843	6,584121427	0,696548992	0,893472189	LADA vs T2D
hsa-let-7d-5p_AGAGGTAGTAGGTTGCATAGTT2	0,076322764	9,075101282	0,697289463	0,893472189	LADA vs T2D
hsa-miR-335-5p_TCAAGAGCAATAACGAAAAATG2	-0,068168023	8,412522999	0,701074771	0,89593068	LADA vs T2D
hsa-miR-4433b-5p_ATGTCCCACCCCCACTCCTGTTT2	-0,173884947	5,620502964	0,701456391	0,89593068	LADA vs T2D
hsa-miR-191-5p_CAACGGAATCCCAAAAGCAGCTGT2	0,163435088	5,688164696	0,70511434	0,897841621	LADA vs T2D
hsa-let-7a-5p_TGAGGTAGTAGGTTGTATAGTTTT2	-0,217593583	3,044731157	0,705205589	0,897841621	LADA vs T2D
hsa-let-7c-5p_TGAGGTAGTAGGTTGTATGGT2	-0,135231636	4,585920454	0,706729635	0,898346921	LADA vs T2D
hsa-miR-423-5p_TGAGGGGCAGAGAGCGAGACTTTT2	-0,039189458	11,39034963	0,711754877	0,902077056	LADA vs T2D
hsa-miR-92a-3p_TATTGCACTTGTCCCGGCCT2	0,053684435	10,27945719	0,712608212	0,902077056	LADA vs T2D
hsa-miR-483-5p_AAGACGGGAGGAAAGAAGGGAG2	0,126842645	6,618430366	0,71395343	0,902077056	LADA vs T2D
hsa-miR-30a-5p_TGTAAACATCCTCGACTGGAA2	-0,072964681	7,169227794	0,714235826	0,902077056	LADA vs T2D
hsa-miR-342-3p_TCACACAGAAATCGCACCCGTC2	-0,197765811	2,309823181	0,716205674	0,902077056	LADA vs T2D
hsa-miR-92b-3p_TATTGCACTCGTCCCGGCCT2	-0,135169276	5,68469958	0,717853919	0,902077056	LADA vs T2D
hsa-miR-126-3p_CTCGTACCGTGAGTAATAATGCG2	0,168648968	4,682348296	0,718907043	0,902077056	LADA vs T2D
hsa-miR-361-5p_TTATCAGAATCTCCAGGGGTACT2	0,194006302	2,611707431	0,719129353	0,902077056	LADA vs T2D
hsa-miR-451a_AAACCGTTACCATTACTGAGTTTAGT2	-0,15379984	4,187049919	0,719850699	0,902077056	LADA vs T2D
hsa-miR-103a-3p_AGCAGCATTGTACAGGGCTAT2	-0,238059778	3,428123511	0,72287405	0,903307239	LADA vs T2D
hsa-miR-501-3p_AATGCACCCGGGCAAGGATT2	-0,095883114	6,44960483	0,723099145	0,903307239	LADA vs T2D
hsa-miR-183-5p_TATGGCACTGGTAGAATT2	-0,197695969	2,805102899	0,726766305	0,904890688	LADA vs T2D
hsa-miR-495-3p_AAACAAACATGGTGCACTTCTT2	-0,20930726	2,485368847	0,727081386	0,904890688	LADA vs T2D
hsa-miR-423-3p_AAGCTCGGTCTGAGGCCCCT2	0,196653966	2,46496229	0,727772812	0,904890688	LADA vs T2D
hsa-miR-99a-5p_AACCCGTAGATCCGATCTTG2	0,060564851	8,041845005	0,733717974	0,909904274	LADA vs T2D
hsa-miR-21-5p_AGCTTATCAGACTGATGTTGA2	-0,07209775	6,721668578	0,735153168	0,909904274	LADA vs T2D
hsa-miR-126-5p_CATTATTACTTTTGGTACGCG2	0,078404216	6,678225687	0,736116237	0,909904274	LADA vs T2D
hsa-let-7f-5p_TGAGGTAGTAGATTGTATAGTTT2	-0,083846618	8,114676232	0,738035527	0,909904274	LADA vs T2D
hsa-miR-423-3p_GCTCGGTCTGAGGCCCCTCAG2	0,184892429	1,954419998	0,738807218	0,909904274	LADA vs T2D
hsa-miR-93-5p_AAAGTGCTGTTCGTGCAGGTAG2	-0,167790286	3,611226251	0,738877952	0,909904274	LADA vs T2D
hsa-miR-222-3p_AGCTACATCTGGCTACTGGGTCTCT2	-0,082491469	7,967322162	0,7397967	0,909904274	LADA vs T2D
hsa-miR-122-5p_GGAGTGTGACAATGGTGTTT2	-0,111056044	7,666405464	0,746607491	0,915774839	LADA vs T2D
hsa-miR-17-5p_CAAAGTGCTTACAGTGCAGGTAG2	-0,175796515	3,276961308	0,746867811	0,915774839	LADA vs T2D

hsa-miR-340-5p_TTATAAAGCAATGAGACTGAT2	0,176464799	1,968507467	0,748279217	0,916096062	LADA vs T2D
hsa-miR-363-3p_AATTGCACGGTATCCATCTGTA2	-0,157273554	3,912624597	0,750850642	0,916902126	LADA vs T2D
hsa-miR-375-3p_TTTGTTCGTTCGGCTCGCGT2	0,172964149	3,894636907	0,751238505	0,916902126	LADA vs T2D
hsa-miR-4433b-5p_TGTCCCACCCCCACTCCTGT2	-0,15058666	4,771975689	0,754854973	0,91990736	LADA vs T2D
hsa-miR-374a-5p_TTATAATACAACCTGATAAGTG2	-0,135586071	4,272038394	0,757375179	0,921082145	LADA vs T2D
hsa-let-7i-5p_GAGGTAGTAGTTTGTGCTGT2	-0,139002229	4,789553904	0,760267023	0,921082145	LADA vs T2D
hsa-miR-133a-3p_TTGGTCCCCTTCAACCAGCTGT2	0,180390521	2,527387136	0,762247346	0,921082145	LADA vs T2D
hsa-miR-27a-3p_TTCACAGTGGCTAAGTTCC2	-0,101632822	5,369078847	0,764011756	0,921082145	LADA vs T2D
hsa-miR-150-5p_TCTCCCAACCCTTGTACCA2	0,099136263	6,588601483	0,764581722	0,921082145	LADA vs T2D
hsa-miR-26a-5p_TTCAAGTAATCCAGGATAGGCT2	0,048345791	11,92893731	0,76742225	0,921082145	LADA vs T2D
hsa-miR-126-5p_CATTATTACTTTTGGTACGC2	0,150076124	3,620244945	0,767957689	0,921082145	LADA vs T2D
hsa-miR-139-5p_TCTACAGTGCACGTGTCTCCA2	-0,137669811	4,69390328	0,768735951	0,921082145	LADA vs T2D
hsa-miR-3615_TCTCTCGGCTCCTCGCGGC2	-0,157567453	2,019798482	0,768852804	0,921082145	LADA vs T2D
hsa-miR-451a_AACCGTTACCATTACTGAGTTT2	-0,159210596	2,400911204	0,769285231	0,921082145	LADA vs T2D
hsa-miR-23a-3p_TCACATTGCCAGGGATTTC2	-0,165892733	2,403793771	0,769999695	0,921082145	LADA vs T2D
hsa-miR-15b-5p_TAGCAGCACATCATGGTTTAC2	-0,058144563	7,368236859	0,77019252	0,921082145	LADA vs T2D
hsa-miR-664a-5p_ACTGGCTAGGGAAAATGATTGG2	-0,149310692	3,587998944	0,7708429	0,921082145	LADA vs T2D
hsa-miR-423-5p_CTGAGGGGCAGAGAGCGAGACTTT2	0,139429832	4,419437576	0,773849366	0,921399003	LADA vs T2D
hsa-miR-10b-5p_TACCCTGTAGAACCGAATTT2	0,109651405	5,2763128	0,774424056	0,921399003	LADA vs T2D
hsa-miR-16-5p_CTAGCAGCACGTAAATATTGGCG2	-0,118083164	4,959212286	0,776264282	0,921399003	LADA vs T2D
hsa-miR-142-3p_TGTAGTGTTTCCTACTTTATGGA2	-0,154878356	3,252758122	0,776833805	0,921399003	LADA vs T2D
hsa-miR-150-5p_CTCCCAACCCTTGTACCAGTG2	0,135006995	4,313974888	0,776888495	0,921399003	LADA vs T2D
hsa-miR-150-5p_GTCTCCCAACCCTTGTACCAGTG2	-0,155230725	2,975164068	0,781812631	0,922739652	LADA vs T2D
hsa-miR-221-3p_AGCTACATTGTCTGCTGGGTTTCA2	0,153786871	2,390031097	0,782757142	0,922739652	LADA vs T2D
hsa-miR-4732-5p_TGTAGAGCAGGGAGCAGGAAG2	0,154299979	3,155376397	0,783445456	0,922739652	LADA vs T2D
hsa-miR-98-5p_TGAGGTAGTAAGTTGTATTG2	-0,128577704	4,611342852	0,783978174	0,922739652	LADA vs T2D
hsa-miR-374a-5p_TTATAATACAACCTGATAAGT2	0,150158331	2,487762486	0,784955787	0,922739652	LADA vs T2D
hsa-miR-369-3p_AATAATACATGGTTGATCTTT2	-0,150361402	4,326255371	0,784965476	0,922739652	LADA vs T2D
hsa-miR-423-5p_TGAGGGGCAGAGAGCGAGACT2	0,030878556	13,29479943	0,788213392	0,923469945	LADA vs T2D
hsa-miR-423-5p_AGGGGCAGAGAGCGAGACTTTT2	-0,127112211	4,864713364	0,79090609	0,923469945	LADA vs T2D
hsa-miR-182-5p_TTTGGCAATGGTAGAACTCACACTGG2	-0,152401057	2,315372824	0,791587173	0,923469945	LADA vs T2D
hsa-miR-30d-5p_GTAAACATCCCCGACTGGAAG2	-0,084698046	5,727486807	0,791684927	0,923469945	LADA vs T2D
hsa-miR-21-5p_TAGCTTATCAGACTGATGT2	-0,120210703	4,699515355	0,793881828	0,923469945	LADA vs T2D
hsa-miR-29a-3p_TAGCACCATCTGAAATCGGTTAT2	-0,142143096	2,486065735	0,795982357	0,923469945	LADA vs T2D
hsa-miR-16-2-3p_CCAATATTACTGTGCTGCTTT2	-0,114226494	4,35154905	0,796739915	0,923469945	LADA vs T2D
hsa-miR-101-3p_GTACAGTACTGTGATAACTGA2	-0,038494637	8,248925167	0,797027336	0,923469945	LADA vs T2D
hsa-miR-199a-3p_ACAGTAGTCTGCACATTGGTT2	0,218908814	4,64378834	0,797213932	0,923469945	LADA vs T2D
hsa-miR-423-5p_CTGAGGGGCAGAGAGCGAGACT2	0,135913733	2,475285897	0,79759423	0,923469945	LADA vs T2D
hsa-miR-486-5p_CCTGTACTGAGCTGCCCCGAG2	-0,037781051	11,29662351	0,798332236	0,923469945	LADA vs T2D
hsa-miR-423-5p_GAGGGGCAGAGAGCGAGACTTTT2	0,122634969	4,029696332	0,801845948	0,923988536	LADA vs T2D
hsa-miR-24-3p_TGGCTCAGTTCAGCAGGAA2	-0,096605962	5,648746767	0,801908334	0,923988536	LADA vs T2D
hsa-miR-193b-5p_CGGGGTTTTGAGGGCGAGATGA2	-0,146311846	2,282241357	0,802258553	0,923988536	LADA vs T2D
hsa-miR-483-3p_TCACTCCTCTCCTCCCGTCT2	0,146104074	2,217026305	0,804324969	0,925031746	LADA vs T2D
hsa-miR-21-5p_TAGCTTATCAGACTGATGTT2	0,047054406	7,170728426	0,809883671	0,930082544	LADA vs T2D
hsa-let-7d-5p_AGAGGTAGTAGGTTGCATAGT2	-0,055526988	7,455921597	0,812379959	0,931606946	LADA vs T2D
hsa-let-7i-5p_TGAGGTAGTAGTTTGTGCTGT2	-0,025273259	11,39282357	0,818554124	0,937338558	LADA vs T2D
hsa-let-7c-5p_TGAGGTAGTAGGTTGTATGGTT2	0,043101513	7,467576739	0,823085704	0,940312605	LADA vs T2D
hsa-miR-338-5p_AACAATATCCTGGTGCTGAGT2	0,068297739	5,993094666	0,824003646	0,940312605	LADA vs T2D
hsa-miR-191-5p_AACGGAATCCCAAAAGCAGC2	-0,116936613	2,920397045	0,824690729	0,940312605	LADA vs T2D
hsa-miR-144-5p_GGATATCATCATATACTGTAAG2	-0,120420662	4,224705339	0,829730317	0,941716027	LADA vs T2D
hsa-miR-486-5p_CTGTACTGAGCTGCCCCGA2	0,041306798	7,583597035	0,830164129	0,941716027	LADA vs T2D
hsa-miR-486-5p_TGTACTGAGCTGCCCCGA2	0,103438495	4,617811312	0,830549348	0,941716027	LADA vs T2D
hsa-miR-629-5p_TGGGTTTACGTTGGGAGAACT2	0,049229771	7,034835335	0,830647889	0,941716027	LADA vs T2D
hsa-let-7b-5p_GAGGTAGTAGGTTGTGTGG2	0,066438798	6,125260792	0,833665243	0,943794316	LADA vs T2D
hsa-miR-542-3p_TGTGACAGATTGATAACTGA2	0,113746457	1,954666629	0,835342955	0,944352248	LADA vs T2D
hsa-miR-19b-3p_TGTGCAAATCCATGCAAAACTGA2	-0,094442427	4,916784309	0,837294884	0,945218162	LADA vs T2D
hsa-miR-23a-3p_ATCACATTGCCAGGGATTTCC2	-0,020807723	11,08225248	0,838907526	0,945699148	LADA vs T2D
hsa-miR-30e-5p_TGTAAACATCCTTGACTGGAAGC2	-0,023494149	8,385302075	0,841148722	0,946886344	LADA vs T2D
hsa-miR-483-5p_AAGACGGGAGGAAAGAAGGGA2	-0,105035186	4,456894738	0,843692108	0,947559544	LADA vs T2D
hsa-miR-92a-3p_TATTGCACTTGTCCCGGCCTGTG2	-0,031253095	7,105169059	0,845589206	0,947559544	LADA vs T2D
hsa-miR-30e-5p_GTAAACATCCTTGACTGGAAGC2	-0,107902276	2,49990334	0,846374514	0,947559544	LADA vs T2D

hsa-miR-193a-5p_TGGGTCTTTGCGGGCGAGATG2	-0,073160466	5,884704134	0,846502378	0,947559544	LADA vs T2D
hsa-miR-99a-5p_AACCCGTAGATCCGATCTT2	-0,106472188	3,048788798	0,848148456	0,948070575	LADA vs T2D
hsa-miR-106b-5p_TAAAGTGCTGACAGTGCAGAT2	0,095615363	2,655083548	0,852050518	0,949006211	LADA vs T2D
hsa-miR-150-5p_CTCCCAACCCTTGTACCAGT2	0,102194763	3,181356832	0,852492854	0,949006211	LADA vs T2D
hsa-miR-23b-3p_ATCACATTGCCAGGGATTACCA2	-0,053065425	6,460852955	0,85301958	0,949006211	LADA vs T2D
hsa-miR-103a-3p_AGCAGCATTGTACAGGGCTATG2	0,097057416	4,276844631	0,853748373	0,949006211	LADA vs T2D
hsa-miR-193a-5p_TGGGTCTTTGCGGGCGAGATGA2	-0,03003517	8,630407946	0,856651537	0,950599036	LADA vs T2D
hsa-miR-3605-3p_CCTCCGTGTTACCTGTCCTCT2	0,076582935	5,168364179	0,857578677	0,950599036	LADA vs T2D
hsa-let-7e-5p_TGAGGTAGGAGGTTGTATAGTT2	0,036185769	7,813417368	0,860253206	0,950599036	LADA vs T2D
hsa-let-7d-3p_TATACGACCTGCTGCCTTTC2	0,043228194	6,429871038	0,860430515	0,950599036	LADA vs T2D
hsa-miR-10a-5p_TACCCTGTAGATCCGAATT2	-0,089898969	3,933865051	0,861817748	0,950599036	LADA vs T2D
hsa-miR-423-3p_AAGCTCGGTCTGAGGCCCCTCAGT2	0,041209521	7,286433576	0,862337645	0,950599036	LADA vs T2D
hsa-miR-191-5p_CAACGGAATCCCAAAAGC2	-0,089943254	3,190312182	0,86763271	0,954480347	LADA vs T2D
hsa-miR-483-5p_AAGACGGGAGGAAAGAAGGGAGT2	0,095765676	3,821071217	0,868253766	0,954480347	LADA vs T2D
hsa-miR-125a-5p_TCCCTGAGACCCTTTAACC2	0,084740847	2,158659604	0,87231838	0,957627753	LADA vs T2D
hsa-miR-197-3p_TTCACCACCTTCTCCACCCA2	0,076280035	3,958344822	0,877249605	0,961716555	LADA vs T2D
hsa-miR-15b-5p_TAGCAGCACATCATGGTTTACA2	0,043250883	6,32576652	0,884440038	0,968267459	LADA vs T2D
hsa-miR-501-3p_AATGCACCCGGGCAAGGATTCT2	0,020515297	7,911300847	0,886126518	0,968783039	LADA vs T2D
hsa-miR-27b-3p_TTCACAGTGGCTAAGTTCTGCA2	0,071893901	3,596858077	0,890188149	0,97057036	LADA vs T2D
hsa-miR-194-5p_TGTAACAGCAACTCCATGTGGA2	-0,074847519	2,048630169	0,890196905	0,97057036	LADA vs T2D
hsa-miR-23a-3p_ATCACATTGCCAGGGATTTCCAA2	0,04349692	6,530990344	0,892049786	0,971261857	LADA vs T2D
hsa-miR-223-5p_CGTGTATTTGACAAGCTGAGTTG2	0,043787426	6,07891061	0,89524116	0,973275832	LADA vs T2D
hsa-miR-340-5p_TTATAAAGCAATGAGACTGATT2	-0,04538265	5,815012818	0,897746092	0,973275832	LADA vs T2D
hsa-miR-451a_AACCGTTACCATTACTGAG2	-0,023415261	8,074719646	0,898079278	0,973275832	LADA vs T2D
hsa-miR-128-3p_TCACAGTGAACCGGTCTCTT2	0,027177635	6,808413156	0,898784206	0,973275832	LADA vs T2D
hsa-miR-1306-5p_CCACCTCCCCTGCAAACGTC2	0,055251788	4,129732962	0,901039466	0,974185454	LADA vs T2D
hsa-miR-139-5p_TCTACAGTGCACGTGTCTCCAG2	-0,054150572	4,676823231	0,90206884	0,974185454	LADA vs T2D
hsa-miR-342-3p_TCTCACACAGAAATCGCACCCG2	-0,024351794	9,658047597	0,904444661	0,974524301	LADA vs T2D
hsa-miR-146a-5p_GAGAACTGAATTCCATGGGTT2	0,037468576	6,481720435	0,904828084	0,974524301	LADA vs T2D
hsa-miR-342-3p_TCTCACACAGAAATCGCACCC2	0,063174512	2,049205595	0,908168762	0,975925105	LADA vs T2D
hsa-miR-30c-5p_TGTAAACATCCTACACTCTCAGCT2	-0,016802468	10,20131272	0,908577701	0,975925105	LADA vs T2D
hsa-miR-30a-5p_TGTAAACATCCTCGACTGG2	0,052129917	4,494972654	0,910943919	0,977149803	LADA vs T2D
hsa-miR-23a-3p_TCACATTGCCAGGGATTTCC2	-0,051093684	4,540513631	0,913998068	0,979108146	LADA vs T2D
hsa-miR-425-5p_AATGACACGATCACTCCCGTTGAGT2	0,023825006	8,386717544	0,917014983	0,981021398	LADA vs T2D
hsa-miR-93-5p_CAAAGTGCTGTTCGTGCAGGTAGT2	0,051515794	4,129822813	0,92072055	0,981808416	LADA vs T2D
hsa-let-7b-3p_CTATACAACCTACTGCCTTC2	0,051203312	3,159200497	0,922257938	0,981808416	LADA vs T2D
hsa-miR-146a-5p_TGAGAACTGAATTCCATGGG2	-0,048708976	2,424854764	0,925712347	0,981808416	LADA vs T2D
hsa-let-7f-5p_TGAGGTAGTAGATTGTATA2	-0,048289843	3,61271535	0,926149153	0,981808416	LADA vs T2D
hsa-let-7f-5p_TGAGGTAGTAGATTGTATAGT2	-0,008917413	10,56301224	0,931223482	0,981808416	LADA vs T2D
hsa-miR-30a-3p_CTTTCAGTCGGATGTTTGCAG2	-0,045786544	1,943754996	0,932275974	0,981808416	LADA vs T2D
hsa-miR-329-3p_AACACACCTGGTTAACCTCTT2	0,047964126	3,637419766	0,9324087	0,981808416	LADA vs T2D
hsa-miR-320a-3p_AAAAGCTGGGTTGAGAGGGCGA2	0,008672043	10,74836293	0,933805461	0,981808416	LADA vs T2D
hsa-miR-139-3p_TGGAGACGCGGCCCTGTTGGAGT2	-0,046844686	3,435697412	0,933945545	0,981808416	LADA vs T2D
hsa-miR-654-5p_TGGTGGGCCGCAGAACATGTGC2	0,051211774	3,027916369	0,934959035	0,981808416	LADA vs T2D
hsa-let-7d-3p_TATACGACCTGCTGCCTTT2	0,02044487	6,275229414	0,93633344	0,981808416	LADA vs T2D
hsa-miR-629-5p_TGGGTTTACGTTGGGAGAAC2	-0,042018786	3,68380122	0,937756223	0,981808416	LADA vs T2D
hsa-miR-486-5p_ATCCTGTACTGAGCTGCCCCG2	0,01998146	6,350028468	0,941983862	0,981808416	LADA vs T2D
hsa-miR-451a_AAACCGTTACCATTACTGAGT2	-0,010448627	16,51409079	0,942192058	0,981808416	LADA vs T2D
hsa-let-7f-5p_TGAGGTAGTAGATTGTATAGTT2	0,011456036	12,51171334	0,942527296	0,981808416	LADA vs T2D
hsa-miR-148a-3p_TCAGTGCACTACAGAACTT2	-0,042247684	2,124123364	0,942652942	0,981808416	LADA vs T2D
hsa-miR-144-5p_GGATATCATCATATACTGTAAGT2	-0,019637886	6,405140764	0,943419017	0,981808416	LADA vs T2D
hsa-miR-21-5p_TAGCTTATCAGACTGATGTTGAC2	0,015543595	10,44971368	0,94435988	0,981808416	LADA vs T2D
hsa-miR-99b-5p_ACCCGTAGAACCGACCTTGCG2	0,037589364	2,082938158	0,944876197	0,981808416	LADA vs T2D
hsa-let-7b-5p_TGAGGTAGTAGGTTGTGTGGTTT2	0,008132737	11,28589984	0,94590704	0,981808416	LADA vs T2D
hsa-miR-7-5p_TGGAAGACTAGTGATTTTGTTGTT2	-0,035516636	4,415695214	0,946959462	0,981808416	LADA vs T2D
hsa-miR-629-5p_TGGGTTTACGTTGGGAGAA2	-0,038235304	2,515869067	0,947402172	0,981808416	LADA vs T2D
hsa-miR-30d-5p_TGTAAACATCCCCGACTGGAAG2	-0,005137597	12,16128446	0,947552352	0,981808416	LADA vs T2D
hsa-miR-100-5p_AACCCGTAGATCCGAACTTG2	0,019672764	5,941970306	0,948034994	0,981808416	LADA vs T2D
hsa-miR-145-5p_GTCCAGTTTTCCCAGGAATCC2	-0,034873106	2,283533911	0,948547654	0,981808416	LADA vs T2D
hsa-miR-505-3p_CGTCAACACTTGCTGGTTTCCTCT2	0,03507115	2,947139365	0,951855882	0,982775156	LADA vs T2D
hsa-miR-486-5p_CTGTACTGAGCTGCCCCGAG2	-0,012125203	7,787642307	0,951947829	0,982775156	LADA vs T2D

hsa-miR-486-5p_TCCTGTACTGAGCTGCCCCGAG2	-0,007596692	17,24661108	0,953752241	0,983364212	LADA vs T2D
hsa-miR-142-5p_CCCATAAAGTAGAAAGCAC2	-0,01881693	5,201334155	0,956106692	0,984163874	LADA vs T2D
hsa-miR-98-5p_TGAGGTAGTAAGTTGTATTGT2	-0,016925579	6,55627531	0,956997493	0,984163874	LADA vs T2D
hsa-miR-4433b-5p_ATGTCCCACCCCCACTCCTG2	0,026465736	2,006165102	0,9609239	0,9857673	LADA vs T2D
hsa-miR-500a-3p_ATGCACCTGGGCAAGGATTCT2	-0,0226428	4,513942296	0,962830766	0,9857673	LADA vs T2D
hsa-miR-142-5p_CATAAAGTAGAAAGCACTACT2	0,023089216	3,650597591	0,963025188	0,9857673	LADA vs T2D
hsa-miR-222-3p_AGCTACATCTGGCTACTGGG2	-0,02558572	2,549442498	0,963504049	0,9857673	LADA vs T2D
hsa-miR-27b-3p_TTCACAGTGGCTAAGTTCT2	0,006824075	8,299608545	0,969530593	0,990277523	LADA vs T2D
hsa-miR-10b-5p_ACCCTGTAGAACCGAATTTG2	0,016001632	4,835525593	0,970397422	0,990277523	LADA vs T2D
hsa-miR-423-5p_AGGGGCAGAGAGCGAGACT2	0,01769833	3,04923014	0,974780904	0,991402441	LADA vs T2D
hsa-miR-382-5p_AAGTTGTTCGTGGTGGATTCG2	-0,018569545	2,227150711	0,975103291	0,991402441	LADA vs T2D
hsa-miR-99b-5p_CACCCGTAGAACCGACCTT2	-0,016246901	2,002435028	0,976422445	0,991402441	LADA vs T2D
hsa-miR-146a-5p_TGAGAACTGAATTCCATGGGTT2	0,00338478	12,1607009	0,976475428	0,991402441	LADA vs T2D
hsa-miR-10a-5p_ACCCTGTAGATCCGAATTTGTG2	-0,008218569	6,001615458	0,978112085	0,991800677	LADA vs T2D
hsa-miR-155-5p_TTAATGCTAATCGTGATAGGGGT2	0,010913072	3,972069578	0,982271132	0,99244265	LADA vs T2D
hsa-miR-6803-3p_TCCCTCGCCTTCTCACCCTC2	-0,011626903	2,004774634	0,982832516	0,99244265	LADA vs T2D
hsa-miR-181a-5p_AACATTCAACGCTGTCGGTGAG2	0,003574678	7,366086853	0,984105566	0,99244265	LADA vs T2D
hsa-miR-493-5p_TTGTACATGGTAGGCTTTCATT2	0,011903542	3,64649227	0,984217102	0,99244265	LADA vs T2D
hsa-let-7b-3p_CTATACAACCTACTGCCTTCC2	0,005130185	6,023895535	0,984989043	0,99244265	LADA vs T2D
hsa-miR-125a-5p_TCCCTGAGACCCTTTAACCTG2	0,003051553	8,290201937	0,986216536	0,99244265	LADA vs T2D
hsa-miR-375-3p_TTTGTTCGTTCGGCTCGCGTGA2	0,002988909	10,08392589	0,989657166	0,994649131	LADA vs T2D
hsa-miR-339-3p_TGAGCGCCTCGACGACAGAGC2	0,005029267	3,99309945	0,991889331	0,995295307	LADA vs T2D
hsa-miR-98-5p_TGAGGTAGTAAGTTGTATTGTT2	0,002276779	7,877215034	0,992797703	0,995295307	LADA vs T2D
hsa-miR-361-5p_TTATCAGAATCTCCAGGGG2	0,003048859	2,056970291	0,99545755	0,996708125	LADA vs T2D
hsa-miR-501-3p_ATGCACCCGGGCAAGGATTCT2	-0,001799774	2,081940648	0,997442183	0,997442183	LADA vs T2D
hsa-miR-10a-5p_ACCCTGTAGATCCGAATTTGTG3	1,173774313	6,001615458	0,000260621	0,111878504	LADA vs Control
hsa-miR-26b-5p_TTCAAGTAATTCAGGATAGG3	-1,694953711	3,436379997	0,000558282	0,111878504	LADA vs Control
hsa-miR-30a-5p_TGTAAACATCCTCGACTGGAAGCT3	0,440600371	8,778765877	0,000664813	0,111878504	LADA vs Control
hsa-miR-30d-5p_TGTAAACATCCCCGACTGGAAGCT3	-0,44904132	13,20515822	0,000681301	0,111878504	LADA vs Control
hsa-miR-30d-5p_GTAAACATCCCCGACTGGAAGCT3	-0,910410638	6,998439264	0,000701873	0,111878504	LADA vs Control
hsa-miR-22-3p_AAGCTGCCAGTTGAAGAACTGT3	0,287657542	12,01293	0,001357149	0,137307033	LADA vs Control
hsa-miR-30d-5p_TGTAAACATCCCCGACTGGAAGC3	-0,340112558	11,59455452	0,001376841	0,137307033	LADA vs Control
hsa-miR-139-5p_TCTACAGTGCACGTGTCTCCAG3	1,516170028	4,676823231	0,00143402	0,137307033	LADA vs Control
hsa-miR-106b-3p_CCGCACTGTGGGTACTTGCT3	-1,064979271	6,396206525	0,001713705	0,137307033	LADA vs Control
hsa-miR-409-3p_GAATGTTGCTCGGTGAACCCCT3	-1,16975837	7,973406619	0,001759949	0,137307033	LADA vs Control
hsa-miR-130b-3p_CAGTGCAATGATGAAAGGGCA3	1,399581102	4,137452675	0,002250169	0,137307033	LADA vs Control
hsa-miR-150-5p_TCTCCCAACCCTTGTACCAGTG3	0,490508822	10,93881631	0,002307703	0,137307033	LADA vs Control
hsa-miR-20a-5p_TAAAGTGCTTATAGTGCAGGTAG3	-1,48220052	4,004629847	0,002333913	0,137307033	LADA vs Control
hsa-miR-10b-5p_ACCCTGTAGAACCGAATTTGTG3	1,007454477	5,829978228	0,00241774	0,137307033	LADA vs Control
hsa-miR-1301-3p_TTGCAGCTGCCTGGGAGTGACTTC3	-1,852573495	2,697320641	0,002705527	0,137307033	LADA vs Control
hsa-miR-185-5p_TGGAGAGAAAGGCAGTTCCTGA3	-0,437448974	8,851967404	0,002807967	0,137307033	LADA vs Control
hsa-miR-30d-5p_GTAAACATCCCCGACTGGAAGC3	-1,371871158	4,874885211	0,002928757	0,137307033	LADA vs Control
hsa-miR-3173-5p_TGCCCTGCCTGTTTTCTCCTTT3	-1,318831652	4,766192857	0,003984121	0,167095073	LADA vs Control
hsa-miR-30e-5p_TGTAAACATCCTTGACTGGAAGCT3	-0,224036086	11,72480372	0,004044501	0,167095073	LADA vs Control
hsa-miR-199a-3p_ACAGTAGTCTGCACATTGGT3	-1,738560312	1,952185074	0,004282313	0,167095073	LADA vs Control
hsa-miR-328-3p_CTGGCCCTCTCTGCCCTTCCGT3	-0,65963119	7,228551867	0,004402756	0,167095073	LADA vs Control
hsa-miR-30d-5p_TGTAAACATCCCCGACTGG3	0,408986946	8,045778015	0,004637705	0,168011422	LADA vs Control
hsa-miR-487b-3p_AATCGTACAGGGTCATCCACTT3	-1,757197688	4,048228768	0,004973777	0,172352172	LADA vs Control
hsa-miR-543_AAACATTCGCGGTGCACTTCTT3	-1,534360959	3,845833985	0,006220964	0,204789261	LADA vs Control
hsa-let-7d-3p_CTATACGACCTGCTGCCTT3	-1,056549693	4,847124715	0,006423753	0,204789261	LADA vs Control
hsa-miR-423-5p_CTGAGGGGCAGAGAGCGAGACT3	1,548761163	2,475285897	0,006813863	0,208871101	LADA vs Control
hsa-miR-101-3p_GTACAGTACTGTGATAACT3	-1,222145887	4,990449736	0,007223257	0,210172205	LADA vs Control
hsa-let-7g-5p_TGAGGTAGTAGTTTGTACAGTTT3	-0,739433253	6,811439564	0,007383716	0,210172205	LADA vs Control
hsa-miR-181b-5p_AACATTCATTGCTGTCGGTGGGT3	-1,351014961	3,594503523	0,007789619	0,211451093	LADA vs Control
hsa-miR-425-5p_AATGACACGATCACTCCCGTTGA3	-0,292208883	10,37803313	0,008220072	0,211451093	LADA vs Control
hsa-miR-27b-3p_TTCACAGTGGCTAAGTTCTGCA3	1,488573	3,596858077	0,008224572	0,211451093	LADA vs Control
hsa-miR-379-5p_TGGTAGACTATGGAACGTAGG3	-1,6636894	3,839701767	0,008553002	0,213023208	LADA vs Control
hsa-miR-484_TCAGGCTCAGTCCCCTCCCGATA3	-1,1810904	4,637634197	0,009972776	0,240857644	LADA vs Control
hsa-miR-1306-5p_CCACCTCCCCTGCAAACGTC3	-1,220140372	4,129732962	0,010640595	0,249428076	LADA vs Control
hsa-let-7b-5p_TGAGGTAGTAGGTTGTGTG3	-0,569678537	6,962968331	0,011160051	0,251500292	LADA vs Control
hsa-miR-222-3p_AGCTACATCTGGCTACTGGGTCTC3	-1,048765739	6,101293098	0,011621899	0,251500292	LADA vs Control

hsa-miR-10a-5p_TACCCTGTAGATCCGAATTTGTG3	0,597179824	7,257876357	0,011761508	0,251500292	LADA vs Control
hsa-miR-4433b-5p_TGTCCCACCCCCACTCCTGTT3	-1,181444986	6,729375179	0,012408914	0,251500292	LADA vs Control
hsa-miR-151a-3p_CTAGACTGAAGCTCCTTGAGGA3	-0,466136538	8,444952042	0,012529563	0,251500292	LADA vs Control
hsa-miR-21-5p_TAGCTTATCAGACTGATGTTG3	0,296615281	11,59436943	0,012622348	0,251500292	LADA vs Control
hsa-miR-27b-3p_TTCACAGTGGCTAAGTTCT3	0,474758322	8,299608545	0,012999377	0,252695212	LADA vs Control
hsa-miR-221-3p_AGCTACATTGTCTGCTGGGTTTC3	-0,77990903	6,752581432	0,014942121	0,28354454	LADA vs Control
hsa-miR-6803-3p_TCCCTCGCCTTCTCACCCTC3	-1,418097444	2,004774634	0,015505811	0,287398397	LADA vs Control
hsa-miR-10b-5p_TACCCTGTAGAACCGAATTTGTG3	0,509948556	7,78613959	0,015889714	0,287820497	LADA vs Control
hsa-miR-423-5p_GAGGGGCAGAGAGCGAGACTT3	1,020339397	5,367382166	0,01714088	0,303584029	LADA vs Control
hsa-miR-128-3p_TCACAGTGAACCGGTCTCTTT3	-0,333122447	8,228392837	0,018009778	0,305651935	LADA vs Control
hsa-miR-186-5p_CAAAGAATTCTCCTTTTGGGC3	-0,704506554	6,535812742	0,018227213	0,305651935	LADA vs Control
hsa-miR-4433b-5p_ATGTCCCACCCCCACTCCTGTTT3	-1,142814235	5,620502964	0,019246145	0,305651935	LADA vs Control
hsa-miR-7-5p_TGGAAGACTAGTGATTTTGT3	1,34183572	3,91952121	0,019467972	0,305651935	LADA vs Control
hsa-miR-140-3p_ACCACAGGGTAGAACCACGGA3	-0,697811211	6,646596672	0,019556626	0,305651935	LADA vs Control
hsa-miR-93-5p_CAAAGTGCTGTTCGTGCAGGTAG3	-0,290211618	9,991178453	0,019558656	0,305651935	LADA vs Control
hsa-miR-199a-5p_CCCAGTGTTCAGACTACCTGTTC3	-1,188486963	4,567460238	0,021583068	0,330205857	LADA vs Control
hsa-miR-320a-3p_AAAGCTGGGTTGAGAGGGCGAA3	-1,115237534	4,271030608	0,022575976	0,330205857	LADA vs Control
hsa-miR-125b-5p_TCCCTGAGACCCTAACTT3	-1,21300672	3,016083692	0,02305665	0,330205857	LADA vs Control
hsa-miR-652-3p_AATGGCGCCACTAGGGTTGTGC3	-1,224859457	3,594273542	0,023594024	0,330205857	LADA vs Control
hsa-miR-361-5p_TTATCAGAATCTCCAGGGG3	1,305474916	2,056970291	0,023598113	0,330205857	LADA vs Control
hsa-miR-652-3p_AATGGCGCCACTAGGGTTGT3	0,379381207	7,904624885	0,023615726	0,330205857	LADA vs Control
hsa-miR-101-3p_TACAGTACTGTGATAACTGAAG3	-0,540554568	7,012156412	0,024804525	0,335792749	LADA vs Control
hsa-miR-223-3p_TGTCAGTTTGTCAAATACCCCAA3	-0,448504631	10,91791178	0,025232555	0,335792749	LADA vs Control
hsa-miR-4433b-5p_TGTCCCACCCCCACTCCTGTTT3	-0,76976627	8,596146134	0,025279253	0,335792749	LADA vs Control
hsa-let-7c-5p_TGAGGTAGTAGGTTGTATGGTT3	0,450228118	7,467576739	0,027255203	0,356104866	LADA vs Control
hsa-miR-339-3p_TGAGCGCCTCGACGACAGAGC3	1,175858084	3,99309945	0,028314835	0,357607545	LADA vs Control
hsa-miR-126-3p_TCGTACCGTGAGTAATAATG3	0,710306044	5,867396204	0,028932855	0,357607545	LADA vs Control
hsa-miR-10b-5p_TACCCTGTAGAACCGAATT3	-1,090236389	4,675674869	0,029117203	0,357607545	LADA vs Control
hsa-miR-409-3p_CGAATGTTGCTCGGTGAACCCCTTT3	-1,440131795	2,598256943	0,029164982	0,357607545	LADA vs Control
hsa-miR-4433b-5p_TGTCCCACCCCCACTCCTGT3	-1,126178251	4,771975689	0,030352936	0,361354391	LADA vs Control
hsa-miR-654-3p_TATGTCTGCTGACCATCACC3	-1,372841753	2,711988254	0,030616015	0,361354391	LADA vs Control
hsa-miR-106b-3p_CCGCACTGTGGGTACTTGCTG3	-1,023725778	4,162035136	0,030830739	0,361354391	LADA vs Control
hsa-miR-24-3p_TGGCTCAGTTCAGCAGGAAC3	0,26115966	10,07194833	0,03203452	0,370021922	LADA vs Control
hsa-miR-320a-3p_AAAGCTGGGTTGAGAGGGCG3	-0,94861769	4,890613401	0,03485787	0,391523035	LADA vs Control
hsa-miR-223-3p_GTCAGTTTGTCAAATACCCCAA3	-0,598190482	7,740128632	0,034878464	0,391523035	LADA vs Control
hsa-miR-652-3p_AATGGCGCCACTAGGGTTGTG3	-0,612495439	6,468098031	0,036172183	0,400405966	LADA vs Control
hsa-let-7f-5p_TGAGGTAGTAGATTGTATAG3	-0,259889726	10,59314063	0,037297578	0,401055944	LADA vs Control
hsa-miR-409-3p_CGAATGTTGCTCGGTGAACCCCT3	-1,117717016	5,738161577	0,037595161	0,401055944	LADA vs Control
hsa-miR-130b-5p_ACTCTTTCCCTGTTGCACTACT3	-0,775844614	5,742085569	0,037740522	0,401055944	LADA vs Control
hsa-miR-335-3p_GTTTTTCATTATTGCTCCTGACC3	-1,272913945	2,935136252	0,03982371	0,417624961	LADA vs Control
hsa-miR-18a-3p_ACTGCCCTAAGTGCTCCTTCTG3	-1,155794933	2,22678804	0,041402657	0,428544387	LADA vs Control
hsa-miR-423-5p_TGAGGGGCAGAGAGCGAGACTT3	0,199645324	12,19565674	0,042348229	0,432712033	LADA vs Control
hsa-miR-92a-3p_TATTGCACTTGTCCCGGCCTGTT3	-0,259568262	10,41769689	0,04400032	0,441663373	LADA vs Control
hsa-miR-126-3p_TCGTACCGTGAGTAATAATGCG3	0,288649421	11,19647801	0,044332585	0,441663373	LADA vs Control
hsa-miR-186-5p_CAAAGAATTCTCCTTTTGGGCTTT3	-1,096829447	4,003798957	0,045408956	0,446801707	LADA vs Control
hsa-miR-1908-5p_CGGCGGGGACGGCGATTGGTC3	1,212554614	2,612171571	0,047213952	0,458896584	LADA vs Control
hsa-miR-27a-3p_TTCACAGTGGCTAAGTTCCGC3	-0,578981991	6,28650114	0,048515629	0,465866944	LADA vs Control
hsa-miR-99a-5p_AACCCGTAGATCCGATCTTGTG3	0,740479587	5,156061862	0,049250441	0,467292872	LADA vs Control
hsa-miR-320a-3p_AAAAGCTGGGTTGAGAGGGCG3	0,250818859	10,00052492	0,05040032	0,467725396	LADA vs Control
hsa-miR-423-3p_AGCTCGGTCTGAGGCCCCTCA3	-0,668766918	6,171912051	0,050469742	0,467725396	LADA vs Control
hsa-miR-150-5p_CTCCCAACCCTTGTACCAGTG3	0,994545143	4,313974888	0,052121317	0,469032943	LADA vs Control
hsa-miR-181a-5p_AACATTCAACGCTGTCGGTGAGT3	0,398369226	8,031521361	0,052128577	0,469032943	LADA vs Control
hsa-miR-3615_TCTCTCGGCTCCTCGCGGCTC3	1,049079266	2,193732344	0,052376326	0,469032943	LADA vs Control
hsa-miR-485-3p_GTCATACACGGCTCTCCTCTCT3	-1,042547357	5,913980943	0,05423356	0,475940446	LADA vs Control
hsa-miR-363-3p_AATTGCACGGTATCCATCTGT3	0,301715469	8,977144446	0,054977378	0,475940446	LADA vs Control
hsa-miR-574-3p_CACGCTCATGCACACACCCAC3	-0,973809857	4,117697394	0,054989087	0,475940446	LADA vs Control
hsa-miR-483-5p_AAGACGGGAGGAAAGAAGGGA3	1,087499207	4,456894738	0,055536338	0,475940446	LADA vs Control
hsa-miR-140-3p_TACCACAGGGTAGAACCACGGACA3	-0,998553137	3,637619462	0,058954612	0,499859848	LADA vs Control
hsa-miR-4433b-5p_TATGTCCCACCCCCACTCCTGT3	-0,968869092	5,397905397	0,059657189	0,500492419	LADA vs Control
hsa-let-7d-5p_AGAGGTAGTAGGTTGCATAGTTT3	-0,921197847	5,420694347	0,063135822	0,506762823	LADA vs Control
hsa-miR-1180-3p_TTTCCGGCTCGCGTGGGTGT3	-0,902476701	4,070138113	0,063586513	0,506762823	LADA vs Control

hsa-miR-483-5p_AAGACGGGAGGAAAGAAGGGAGT3	1,146290971	3,821071217	0,063732292	0,506762823	LADA vs Control
hsa-miR-484_CAGGCTCAGTCCCCTCCCGAT3	-1,020567809	2,404164158	0,063927333	0,506762823	LADA vs Control
hsa-miR-106b-5p_TAAAGTGCTGACAGTGCAGA3	-0,779762792	5,144709005	0,064121553	0,506762823	LADA vs Control
hsa-miR-182-5p_TTTGGCAATGGTAGAACTC3	-1,130273892	2,382812326	0,06421963	0,506762823	LADA vs Control
hsa-miR-425-5p_ATGACACGATCACTCCCGTTGAGT3	-0,895622242	4,72212581	0,065576118	0,512393783	LADA vs Control
hsa-miR-27b-3p_TTCACAGTGGCTAAGTTCTG3	0,342809763	8,279294906	0,066563433	0,513258445	LADA vs Control
hsa-miR-140-3p_TACCACAGGGTAGAACCACGG3	-0,617393574	5,563733367	0,066974753	0,513258445	LADA vs Control
hsa-miR-140-3p_ACCACAGGGTAGAACCACGGAC3	-0,650671802	5,852260421	0,068432746	0,516466878	LADA vs Control
hsa-miR-542-3p_TGTGACAGATTGATAACTGA3	1,07037431	1,954666629	0,068869194	0,516466878	LADA vs Control
hsa-miR-155-5p_TTAATGCTAATCGTGATAGGGGT3	0,953774734	3,972069578	0,06933746	0,516466878	LADA vs Control
hsa-miR-183-5p_ATGGCACTGGTAGAATTCACT3	-0,895277357	4,652978697	0,0733541	0,522729226	LADA vs Control
hsa-miR-369-3p_AATAATACATGGTTGATCTTT3	-1,049410131	4,326255371	0,073704824	0,522729226	LADA vs Control
hsa-miR-505-3p_CGTCAACACTTGCTGGTTTCCTCT3	1,115461875	2,947139365	0,074095474	0,522729226	LADA vs Control
hsa-miR-122-5p_TGGAGTGTGACAATGGTGTTT3	0,489074284	13,55430102	0,074622361	0,522729226	LADA vs Control
hsa-miR-345-5p_GCTGACTCCTAGTCCAGGGCT3	-0,783943139	5,057881275	0,074933142	0,522729226	LADA vs Control
hsa-miR-30a-5p_TGTAAACATCCTCGACTGGAAGC3	0,324263612	7,798673355	0,075493583	0,522729226	LADA vs Control
hsa-miR-486-5p_CCTGTACTGAGCTGCCCCGAG3	-0,283099812	11,29662351	0,075563996	0,522729226	LADA vs Control
hsa-miR-421_ATCAACAGACATTAATTGGGCGC3	-0,98801761	2,04052248	0,076701979	0,522729226	LADA vs Control
hsa-miR-424-3p_CAAAACGTGAGGCGCTGCT3	1,008437585	2,803316018	0,07705032	0,522729226	LADA vs Control
hsa-miR-92b-3p_TATTGCACTCGTCCCGGCCTC3	-0,891848182	3,886574178	0,078128678	0,522729226	LADA vs Control
hsa-miR-451a_AAACCGTTACCATTACTGAGTT3	-0,277534186	12,14012514	0,078329474	0,522729226	LADA vs Control
hsa-miR-382-5p_GAAGTTGTTCGTGGTGGATTCG3	-0,630566007	7,607601722	0,08046789	0,522729226	LADA vs Control
hsa-miR-21-5p_TAGCTTATCAGACTGATGTTGAC3	-0,419176027	10,44971368	0,08051338	0,522729226	LADA vs Control
hsa-miR-10b-5p_TACCCTGTAGAACCGAATTTGT3	0,215880941	11,12588043	0,081441029	0,522729226	LADA vs Control
hsa-miR-532-5p_CATGCCTTGAGTGTAGGACCGT3	-0,822644841	4,730944055	0,081920365	0,522729226	LADA vs Control
hsa-miR-145-3p_ATTCCTGGAAATACTGTTCT3	-1,000629606	3,209907444	0,082257055	0,522729226	LADA vs Control
hsa-miR-223-3p_TGTCAGTTTGTCAAATACCCCA3	-0,326689414	10,41019198	0,082283213	0,522729226	LADA vs Control
hsa-miR-25-3p_CATTGCACTTGTCTCGGT3	0,903500781	3,623128964	0,083253702	0,522729226	LADA vs Control
hsa-miR-142-3p_TGTAGTGTTTCCTACTTTATGGA3	1,013947957	3,252758122	0,084517002	0,522729226	LADA vs Control
hsa-miR-16-2-3p_ACCAATATTACTGTGCTGCTTT3	-0,284196326	7,879139867	0,084703081	0,522729226	LADA vs Control
hsa-miR-183-5p_TATGGCACTGGTAGAATTC3	-0,976879507	3,376816673	0,085411054	0,522729226	LADA vs Control
hsa-miR-92b-3p_TATTGCACTCGTCCCGGCCTCC3	-0,839591821	4,414239688	0,087181646	0,522729226	LADA vs Control
hsa-miR-103a-3p_AGCAGCATTGTACAGGGC3	1,213324751	3,081760646	0,087606091	0,522729226	LADA vs Control
hsa-miR-140-3p_TACCACAGGGTAGAACCACGGA3	-0,371899925	7,894124989	0,087891601	0,522729226	LADA vs Control
hsa-miR-486-5p_TCCTGTACTGAGCTGCCCC3	-0,388116701	6,793601467	0,088912724	0,522729226	LADA vs Control
hsa-miR-139-5p_TCTACAGTGCACGTGTCTCCA3	0,840901851	4,69390328	0,08985732	0,522729226	LADA vs Control
hsa-miR-425-5p_AATGACACGATCACTCCCGTTGAG3	-0,68247264	6,063293525	0,090232371	0,522729226	LADA vs Control
hsa-miR-30a-5p_TGTAAACATCCTCGACTGGAA3	0,360570592	7,169227794	0,091072127	0,522729226	LADA vs Control
hsa-let-7f-5p_TGAGGTAGTAGATTGTATAGTTG3	-1,038507103	2,64125494	0,091320496	0,522729226	LADA vs Control
hsa-let-7d-3p_TATACGACCTGCTGCCTTTCT3	-0,227249238	8,488338468	0,092638996	0,522729226	LADA vs Control
hsa-miR-425-5p_AATGACACGATCACTCCCGTTG3	-0,221688734	9,36928751	0,092968165	0,522729226	LADA vs Control
hsa-miR-340-3p_TCCGTCTCAGTTACTTTATAGC3	-0,933361306	1,820184636	0,092994609	0,522729226	LADA vs Control
hsa-miR-27a-3p_TCACAGTGGCTAAGTTCCG3	0,991015681	2,668037972	0,093208726	0,522729226	LADA vs Control
hsa-miR-139-5p_TCTACAGTGCACGTGTCTCCAGT3	0,457866751	6,587018882	0,093441815	0,522729226	LADA vs Control
hsa-miR-320b_AAAAGCTGGGTTGAGAGGGCA3	-0,876589765	2,618155177	0,093479025	0,522729226	LADA vs Control
hsa-miR-21-5p_AGCTTATCAGACTGATGTTGAC3	-1,051483542	3,189772648	0,094010338	0,522729226	LADA vs Control
hsa-miR-197-3p_TTCACCACCTTCTCCACCCAGC3	-0,275811558	10,1606215	0,094919922	0,522729226	LADA vs Control
hsa-miR-6803-3p_TCCCTCGCCTTCTCACCCTCAG3	-0,949646953	2,710817974	0,095101302	0,522729226	LADA vs Control
hsa-miR-423-5p_AGGGGCAGAGAGCGAGACT3	1,000550554	3,04923014	0,09632622	0,525835596	LADA vs Control
hsa-miR-375-3p_TTTGTTCGTTCGGCTCGCGTGA3	0,41126843	10,08392589	0,097053386	0,526201011	LADA vs Control
hsa-miR-186-5p_CAAAGAATTCTCCTTTTGGGCT3	-0,219000379	9,032677455	0,099956069	0,53827694	LADA vs Control
hsa-miR-4732-5p_TGTAGAGCAGGGAGCAGGAAG3	-0,98838758	3,155376397	0,100843747	0,539412528	LADA vs Control
hsa-miR-409-3p_GAATGTTGCTCGGTGAACCCCTT3	-0,958543568	5,101558557	0,104628318	0,552661801	LADA vs Control
hsa-miR-451a_AACCGTTACCATTACTGAGTT3	-0,51877008	6,297926904	0,104707568	0,552661801	LADA vs Control
hsa-miR-29a-3p_TAGCACCATCTGAAATCGGTT3	0,296626441	8,641484144	0,107374634	0,563010417	LADA vs Control
hsa-miR-122-5p_GGAGTGTGACAATGGTGTTT3	0,583852612	7,666405464	0,10858206	0,565620273	LADA vs Control
hsa-miR-125a-5p_TCCCTGAGACCCTTTAACCTGTG3	0,259995096	8,600810288	0,109385762	0,566106832	LADA vs Control
hsa-miR-92a-3p_GCACTTGTCCCGGCCTGT3	-0,704063254	4,476222162	0,111191652	0,568209071	LADA vs Control
hsa-miR-10b-5p_TACCCTGTAGAACCGAATTTGTGT3	0,936217135	2,268128931	0,111217836	0,568209071	LADA vs Control
hsa-miR-23a-3p_ATCACATTGCCAGGGATTT3	0,286211892	7,810237088	0,113338064	0,571746246	LADA vs Control
hsa-miR-101-3p_TACAGTACTGTGATAACTGA3	-0,84868331	4,333316093	0,113344927	0,571746246	LADA vs Control

hsa-miR-10b-5p_TACCCTGTAGAACCGAAT3	-0,944433568	2,220184724	0,114703739	0,574961511	LADA vs Control
hsa-miR-29c-3p_TAGCACCATTTGAAATCGGTT3	0,786273334	3,850371057	0,120596581	0,57563713	LADA vs Control
hsa-miR-320b_AAAAGCTGGGTTGAGAGGGC3	-1,343760174	5,061955788	0,120893754	0,57563713	LADA vs Control
hsa-miR-29a-3p_TAGCACCATCTGAAATCGG3	0,396778225	7,213630267	0,120908467	0,57563713	LADA vs Control
hsa-miR-142-5p_CCCATAAAGTAGAAAGCACTA3	-0,601274594	5,405299379	0,121016706	0,57563713	LADA vs Control
hsa-miR-125a-5p_TCCCTGAGACCCTTTAACCT3	0,403016179	6,34415738	0,121244885	0,57563713	LADA vs Control
hsa-miR-23a-3p_ATCACATTGCCAGGGATTTCCA3	-0,228609191	11,15500363	0,121930308	0,57563713	LADA vs Control
hsa-miR-30e-3p_CTTTCAGTCGGATGTTTACAGC3	-0,894908769	3,314941715	0,122844777	0,57563713	LADA vs Control
hsa-miR-140-3p_TACCACAGGGTAGAACCACG3	-0,619445333	5,026346909	0,12308259	0,57563713	LADA vs Control
hsa-miR-101-3p_TACAGTACTGTGATAACTGAA3	-0,903876308	3,070531279	0,124121654	0,57563713	LADA vs Control
hsa-miR-584-5p_TTATGGTTTGCCTGGGACTGA3	-0,324034233	8,070467342	0,124388905	0,57563713	LADA vs Control
hsa-let-7b-5p_GAGGTAGTAGGTTGTGTGGT3	-0,705235841	4,944887656	0,126108482	0,57563713	LADA vs Control
hsa-miR-409-3p_GAATGTTGCTCGGTGAACCCCTTT3	-0,958791925	4,139609499	0,126456868	0,57563713	LADA vs Control
hsa-miR-543_AAACATTCGCGGTGCACTTCTTT3	-0,990259077	3,173881718	0,127455207	0,57563713	LADA vs Control
hsa-let-7b-5p_GAGGTAGTAGGTTGTGTGGTTT3	-0,846025919	3,859782449	0,128313586	0,57563713	LADA vs Control
hsa-let-7a-5p_TGAGGTAGTAGGTTGTATAG3	-0,17148818	12,05467063	0,1290242	0,57563713	LADA vs Control
hsa-miR-30a-5p_TGTAAACATCCTCGACTGGAAG3	0,28217938	8,337829601	0,130019346	0,57563713	LADA vs Control
hsa-miR-101-3p_GTACAGTACTGTGATAACTGA3	-0,242657528	8,248925167	0,130049055	0,57563713	LADA vs Control
hsa-miR-191-5p_CAACGGAATCCCAAAAGCAGCTG3	-0,284348196	9,658129048	0,130124054	0,57563713	LADA vs Control
hsa-miR-500a-3p_AATGCACCTGGGCAAGGATTCT3	0,950412853	2,780066987	0,131398834	0,57563713	LADA vs Control
hsa-miR-183-5p_ATGGCACTGGTAGAATTCACTGT3	0,889899703	2,224937851	0,132234439	0,57563713	LADA vs Control
hsa-miR-222-3p_AGCTACATCTGGCTACTGGGTCT3	-0,436685939	6,5045561	0,132533743	0,57563713	LADA vs Control
hsa-let-7f-5p_TGAGGTAGTAGATTGTATAGTTT3	-0,405675454	8,114676232	0,132681634	0,57563713	LADA vs Control
hsa-miR-16-5p_TAGCAGCACGTAAATATTG3	-0,543935683	5,566162995	0,132842256	0,57563713	LADA vs Control
hsa-miR-323a-3p_GCACATTACACGGTCGACCTCT3	-0,861266705	3,90036256	0,133915328	0,57563713	LADA vs Control
hsa-let-7b-5p_TGAGGTAGTAGGTTGTGTGGTTT3	-0,191516343	11,28589984	0,135166902	0,57563713	LADA vs Control
hsa-miR-584-5p_TTATGGTTTGCCTGGGACTG3	-0,837342392	2,558465624	0,135559692	0,57563713	LADA vs Control
hsa-let-7b-5p_TGAGGTAGTAGGTTGTGTGGT3	-0,164397129	11,84645942	0,136689308	0,57563713	LADA vs Control
hsa-miR-130a-3p_CAGTGCAATGTTAAAAGGGCA3	0,71816841	4,214432691	0,137365252	0,57563713	LADA vs Control
hsa-miR-126-3p_CGTACCGTGAGTAATAATGCG3	0,225432341	9,683358758	0,137860076	0,57563713	LADA vs Control
hsa-miR-494-3p_TGAAACATACACGGGAAACCTCT3	-0,856427315	4,269868739	0,140558517	0,57563713	LADA vs Control
hsa-miR-320a-3p_AAAAGCTGGGTTGAGAGGGCGA3	0,164134689	10,74836293	0,141631625	0,57563713	LADA vs Control
hsa-miR-186-5p_CAAAGAATTCTCCTTTTGGGCTT3	-0,493910792	6,345307365	0,141869518	0,57563713	LADA vs Control
hsa-miR-28-5p_AAGGAGCTCACAGTCTATTGAG3	-0,88912696	3,384277878	0,142284895	0,57563713	LADA vs Control
hsa-miR-423-5p_TGAGGGGCAGAGAGCGAGACTTTT3	-0,167383003	11,39034963	0,142532185	0,57563713	LADA vs Control
hsa-miR-224-5p_CAAGTCACTAGTGGTTCCGTTTAG3	-0,821058861	4,65780231	0,142587154	0,57563713	LADA vs Control
hsa-miR-363-3p_AATTGCACGGTATCCATCT3	-0,747769376	4,478964619	0,143017671	0,57563713	LADA vs Control
hsa-miR-223-3p_TGTCAGTTTGTCAAATACCCC3	-0,436454969	7,314719746	0,143650987	0,57563713	LADA vs Control
hsa-miR-423-5p_TGAGGGGCAGAGAGCGAGACT3	0,179835554	13,29479943	0,144395431	0,57563713	LADA vs Control
hsa-miR-3613-5p_TGTTGTACTTTTTTTTTTGTT3	-0,601855806	5,269511804	0,144555933	0,57563713	LADA vs Control
hsa-miR-339-3p_TGAGCGCCTCGACGACAGAGCCG3	-0,848390521	4,16247252	0,144722084	0,57563713	LADA vs Control
hsa-miR-10a-5p_TACCCTGTAGATCCGAATTTGT3	0,173099009	10,44682905	0,144896038	0,57563713	LADA vs Control
hsa-miR-125b-5p_TCCCTGAGACCCTAACTTGTGA3	-0,560222266	5,6455681	0,145173229	0,57563713	LADA vs Control
hsa-miR-29a-3p_TAGCACCATCTGAAATCGGT3	0,282427108	8,663780214	0,147075139	0,580291515	LADA vs Control
hsa-miR-409-3p_AATGTTGCTCGGTGAACCCCT3	-0,870636802	4,366315065	0,147953107	0,580879933	LADA vs Control
hsa-miR-222-3p_AGCTACATCTGGCTACTGGGTCTCT3	-0,381892929	7,967322162	0,150947205	0,589663152	LADA vs Control
hsa-miR-342-3p_TCACACAGAAATCGCACCCGT3	0,824561226	2,35866661	0,151669945	0,589663152	LADA vs Control
hsa-miR-22-3p_AAGCTGCCAGTTGAAGAACT3	0,179721725	10,21399499	0,154340957	0,597134674	LADA vs Control
hsa-miR-145-5p_GTCCAGTTTTCCCAGGAATCCC3	0,871460435	3,12605684	0,155246515	0,597736582	LADA vs Control
hsa-miR-27b-3p_TTCACAGTGGCTAAGTTCTGC3	-0,274133319	7,903620851	0,157602947	0,603316917	LADA vs Control
hsa-miR-215-5p_ATGACCTATGAATTGACAGA3	0,768995287	4,790158483	0,158209832	0,603316917	LADA vs Control
hsa-miR-132-3p_TAACAGTCTACAGCCATGGTCG3	0,816553931	2,233528136	0,160546404	0,607839494	LADA vs Control
hsa-miR-451a_AAACCGTTACCATTACTGAGTTTAGT3	0,638043992	4,187049919	0,160921121	0,607839494	LADA vs Control
hsa-miR-335-5p_TCAAGAGCAATAACGAAAAATG3	-0,266814525	8,412522999	0,162345895	0,61032867	LADA vs Control
hsa-miR-25-3p_CATTGCACTTGTCTCGGTCTG3	-0,252325435	9,990945876	0,165425232	0,618985491	LADA vs Control
hsa-miR-335-3p_TTTTTCATTATTGCTCCTGACC3	-0,802684436	4,076732498	0,167808285	0,624968237	LADA vs Control
hsa-let-7g-5p_TGAGGTAGTAGTTTGTACAG3	-0,185694321	9,407543438	0,174427933	0,639748415	LADA vs Control
hsa-miR-486-5p_CTGTACTGAGCTGCCCCG3	-0,781844809	3,40982355	0,175514237	0,639748415	LADA vs Control
hsa-miR-125b-5p_CCCTGAGACCCTAACTTGT3	-0,789483942	2,290772166	0,176637995	0,639748415	LADA vs Control
hsa-let-7a-5p_AGGTAGTAGGTTGTATAGTT3	-0,804463978	1,879378714	0,177093632	0,639748415	LADA vs Control
hsa-miR-223-5p_CGTGTATTTGACAAGCTGAGTTGG3	-0,82492987	2,566442122	0,177557358	0,639748415	LADA vs Control

hsa-miR-30e-5p_TGTAAACATCCTTGACTGG3	0,63240876	5,076073317	0,177918058	0,639748415	LADA vs Control
hsa-miR-194-5p_TGTAACAGCAACTCCATGTGGAA3	-0,755590274	2,659899918	0,178417647	0,639748415	LADA vs Control
hsa-miR-342-3p_TCTCACACAGAAATCGCACCCGTCA3	0,785253453	3,422364811	0,178596084	0,639748415	LADA vs Control
hsa-miR-484_TCAGGCTCAGTCCCCTCCCGAT3	-0,188948813	9,18414004	0,179001125	0,639748415	LADA vs Control
hsa-miR-125a-5p_TCCCTGAGACCCTTTAACCTGT3	0,167868706	11,48945871	0,180276838	0,641431429	LADA vs Control
hsa-miR-128-3p_TCACAGTGAACCGGTCTCTTTT3	-0,542981046	5,404238817	0,182540768	0,646599966	LADA vs Control
hsa-miR-3615_TCTCTCGGCTCCTCGCGGCTCG3	0,189456003	8,091694617	0,183406814	0,646720717	LADA vs Control
hsa-let-7b-5p_TGAGGTAGTAGGTTGTGTGG3	0,148574259	12,43476497	0,18553941	0,646720717	LADA vs Control
hsa-miR-21-5p_AGCTTATCAGACTGATGTTG3	0,519639129	5,031654467	0,185632336	0,646720717	LADA vs Control
hsa-miR-30c-5p_TGTAAACATCCTACACTCTC3	0,782149383	2,069916362	0,186506606	0,646720717	LADA vs Control
hsa-miR-423-5p_TGAGGGGCAGAGAGCGAGACTTT3	0,097693074	14,42674435	0,186938708	0,646720717	LADA vs Control
hsa-miR-146a-5p_TGAGAACTGAATTCCATGGGTTGT3	-0,396059076	7,265118969	0,187539465	0,646720717	LADA vs Control
hsa-miR-1180-3p_TTTCCGGCTCGCGTGGGTGTGT3	-0,734945404	2,874816557	0,188550689	0,646720717	LADA vs Control
hsa-miR-1301-3p_TTGCAGCTGCCTGGGAGTGA3	-0,7553875	3,347749467	0,190744626	0,646720717	LADA vs Control
hsa-miR-223-3p_TGTCAGTTTGTCAAATACC3	-0,536107939	5,409428462	0,19111429	0,646720717	LADA vs Control
hsa-let-7a-5p_GTGAGGTAGTAGGTTGTATAGTT3	0,830452324	2,866169762	0,192072844	0,646720717	LADA vs Control
hsa-miR-101-3p_GTACAGTACTGTGATAACTG3	-0,51484367	5,734652626	0,192280119	0,646720717	LADA vs Control
hsa-miR-423-3p_AAGCTCGGTCTGAGGCCCCTCAG3	-0,710951938	3,402313099	0,192363	0,646720717	LADA vs Control
hsa-miR-28-3p_CACTAGATTGTGAGCTCCTGGAG3	0,740665398	2,845963244	0,193123627	0,646720717	LADA vs Control
hsa-let-7d-5p_AGAGGTAGTAGGTTGCATAGT3	-0,323656305	7,455921597	0,195539067	0,652069607	LADA vs Control
hsa-let-7e-5p_TGAGGTAGGAGGTTGTATAGT3	0,634097057	4,645402812	0,196738107	0,652262932	LADA vs Control
hsa-miR-485-5p_AGAGGCTGGCCGTGATGAATTCG3	-0,729605932	4,543642396	0,197307006	0,652262932	LADA vs Control
hsa-miR-92a-3p_TATTGCACTTGTCCCGGCC3	-0,274939031	8,088512139	0,198052233	0,652262932	LADA vs Control
hsa-miR-197-3p_TTCACCACCTTCTCCACCCA3	-0,684587612	3,958344822	0,199850244	0,653267633	LADA vs Control
hsa-miR-182-5p_TTTGGCAATGGTAGAACT3	-0,764885307	3,421424723	0,201458449	0,653267633	LADA vs Control
hsa-let-7a-5p_TGAGGTAGTAGGTTGTATA3	-0,459326762	5,663865648	0,201569062	0,653267633	LADA vs Control
hsa-miR-181a-5p_AACATTCAACGCTGTCGGTGAG3	0,244059353	7,366086853	0,201635932	0,653267633	LADA vs Control
hsa-miR-23a-5p_GGGGTTCCTGGGGATGGGATT3	0,720308612	3,585240207	0,203847487	0,653540751	LADA vs Control
hsa-miR-423-5p_TGAGGGGCAGAGAGCGAGAC3	0,134476168	9,751921415	0,204020795	0,653540751	LADA vs Control
hsa-miR-150-5p_TCTCCCAACCCTTGTACCAGT3	0,237238369	10,25222562	0,20591395	0,653540751	LADA vs Control
hsa-miR-363-3p_AATTGCACGGTATCCATCTGTA3	0,671436409	3,912624597	0,206853975	0,653540751	LADA vs Control
hsa-let-7f-5p_GAGGTAGTAGATTGTATAGTT3	-0,467054889	6,051011881	0,207465601	0,653540751	LADA vs Control
hsa-miR-142-5p_CCCATAAAGTAGAAAGCA3	-0,63284341	2,550307907	0,208442192	0,653540751	LADA vs Control
hsa-miR-6803-3p_TCCCTCGCCTTCTCACCCTCA3	-0,428514741	6,298415012	0,208730241	0,653540751	LADA vs Control
hsa-miR-18a-3p_ACTGCCCTAAGTGCTCCTTCT3	0,741438111	3,273299722	0,210331994	0,653540751	LADA vs Control
hsa-miR-574-3p_CACGCTCATGCACACACCCA3	-0,71056526	3,365756558	0,211110172	0,653540751	LADA vs Control
hsa-miR-30e-5p_GTAAACATCCTTGACTGGAAGCT3	0,144112909	8,781643893	0,211305134	0,653540751	LADA vs Control
hsa-miR-148a-3p_TCAGTGCACTACAGAACTTTGT3	-0,182645894	9,289411705	0,211907377	0,653540751	LADA vs Control
hsa-miR-191-5p_CAACGGAATCCCAAAAGCAGCT3	-0,163587022	11,11779618	0,212737346	0,653540751	LADA vs Control
hsa-miR-425-5p_AATGACACGATCACTCCCGTTGAGT3	-0,305658638	8,386717544	0,21319018	0,653540751	LADA vs Control
hsa-miR-484_TCAGGCTCAGTCCCCTCCCG3	-0,310366906	7,780454359	0,214136661	0,653540751	LADA vs Control
hsa-miR-144-5p_GATATCATCATATACTGTAAGTT3	-0,74713183	2,459029553	0,214415411	0,653540751	LADA vs Control
hsa-miR-150-3p_CTGGTACAGGCCTGGGGGAC3	0,748599978	2,308717969	0,215086206	0,653540751	LADA vs Control
hsa-miR-21-3p_CAACACCAGTCGATGGGCTGT3	-0,676750169	2,296740898	0,215660248	0,653540751	LADA vs Control
hsa-miR-425-5p_ATGACACGATCACTCCCGTTG3	-0,727694261	2,398751928	0,218343915	0,654960239	LADA vs Control
hsa-miR-486-5p_ATCCTGTACTGAGCTGCCCCG3	-0,361818553	6,350028468	0,218878327	0,654960239	LADA vs Control
hsa-miR-27a-3p_TTCACAGTGGCTAAGTTCCG3	0,158547694	9,740987062	0,219572916	0,654960239	LADA vs Control
hsa-miR-629-5p_TGGGTTTACGTTGGGAGAA3	0,762492441	2,515869067	0,220127493	0,654960239	LADA vs Control
hsa-miR-151a-3p_TACTAGACTGAAGCTCCTTGAG3	-0,702829223	3,055291554	0,220237571	0,654960239	LADA vs Control
hsa-miR-485-5p_AGAGGCTGGCCGTGATGAATTC3	-0,78201308	2,300221452	0,223189358	0,660567663	LADA vs Control
hsa-miR-182-5p_TTTGGCAATGGTAGAACTCACA3	-0,34839097	6,943076472	0,223780764	0,660567663	LADA vs Control
hsa-miR-361-3p_TCCCCCAGGTGTGATTCTGATT3	0,613937751	3,650889743	0,224922049	0,661486616	LADA vs Control
hsa-miR-29a-3p_TAGCACCATCTGAAATCGGTTA3	-0,585278757	4,716466347	0,227782457	0,665492896	LADA vs Control
hsa-miR-140-5p_CAGTGGTTTTACCCTATGGTAG3	0,641321224	3,871751643	0,228450458	0,665492896	LADA vs Control
hsa-miR-450b-5p_TTTTGCAATATGTTCCTGAAT3	-0,547497732	5,016886951	0,228789277	0,665492896	LADA vs Control
hsa-miR-23b-3p_ATCACATTGCCAGGGATTAC3	0,670346727	2,694770844	0,230653114	0,666252977	LADA vs Control
hsa-miR-191-5p_CAACGGAATCCCAAAAGCAG3	0,138905392	10,17632421	0,230722486	0,666252977	LADA vs Control
hsa-miR-144-3p_CTACAGTATAGATGATGTAC3	0,686357765	3,24422031	0,231559479	0,666255973	LADA vs Control
hsa-miR-30d-5p_GTAAACATCCCCGACTGGAAG3	0,405443354	5,727486807	0,233846651	0,670416479	LADA vs Control
hsa-miR-10a-5p_TACCCTGTAGATCCGAAT3	0,722862777	2,33323359	0,236243746	0,674861167	LADA vs Control
hsa-miR-15a-5p_TAGCAGCACATAATGGTTTGT3	0,379555	6,478410353	0,238052264	0,676258096	LADA vs Control

hsa-miR-146b-5p_TGAGAACTGAATTCCATAGGCTG3	-0,446794938	5,765289472	0,238495412	0,676258096	LADA vs Control
hsa-let-7b-5p_GAGGTAGTAGGTTGTGTGG3	0,399028048	6,125260792	0,240383562	0,676258096	LADA vs Control
hsa-let-7i-5p_TGAGGTAGTAGTTTGTGCTGTT3	-0,192369997	10,48466024	0,240717337	0,676258096	LADA vs Control
hsa-miR-143-3p_TGAGATGAAGCACTGTAGCTCA3	-0,363763342	6,738882834	0,240975281	0,676258096	LADA vs Control
hsa-miR-370-3p_GCCTGCTGGGGTGGAACCTGGT3	-0,745491314	2,498088509	0,242768244	0,678899264	LADA vs Control
hsa-miR-223-3p_GTCAGTTTGTCAAATACCCCA3	-0,370217863	6,454169749	0,25171404	0,689204239	LADA vs Control
hsa-miR-19b-3p_TGTGCAAATCCATGCAAAACTGA3	0,571062257	4,916784309	0,252309713	0,689204239	LADA vs Control
hsa-miR-423-5p_AGGGGCAGAGAGCGAGACTTTT3	0,586013007	4,864713364	0,252905789	0,689204239	LADA vs Control
hsa-miR-486-3p_CGGGGCAGCTCAGTACAGGAT3	-0,287116593	7,803889597	0,253124373	0,689204239	LADA vs Control
hsa-miR-15b-5p_TAGCAGCACATCATGGTTTA3	-0,40057101	5,794504936	0,254903087	0,689204239	LADA vs Control
hsa-miR-423-3p_GCTCGGTCTGAGGCCCCTCAG3	0,675882455	1,954419998	0,255736265	0,689204239	LADA vs Control
hsa-miR-424-5p_CAGCAGCAATTCATGTTTTGA3	0,674295426	2,646439112	0,256201947	0,689204239	LADA vs Control
hsa-let-7c-5p_TGAGGTAGTAGGTTGTATGGT3	0,431471861	4,585920454	0,256466464	0,689204239	LADA vs Control
hsa-miR-1306-5p_CCACCTCCCCTGCAAACGT3	-0,727520582	2,734536196	0,257381522	0,689204239	LADA vs Control
hsa-miR-486-5p_TCCTGTACTGAGCTGCCCCGAG3	-0,15776644	17,24661108	0,257421823	0,689204239	LADA vs Control
hsa-miR-16-5p_TAGCAGCACGTAAATATTGGC3	-0,145683559	9,94541903	0,259135563	0,689204239	LADA vs Control
hsa-miR-2110_TTGGGGAAACGGCCGCTGAG3	-0,65745461	2,165591375	0,261157195	0,689204239	LADA vs Control
hsa-let-7a-5p_TTGAGGTAGTAGGTTGTATAGTT3	-0,654885371	1,956823269	0,263070442	0,689204239	LADA vs Control
hsa-miR-486-5p_TCCTGTACTGAGCTGCCCCGAGG3	-0,211946999	7,467002764	0,263139591	0,689204239	LADA vs Control
hsa-let-7a-5p_GAGGTAGTAGGTTGTATAGTT3	0,299355834	7,081547935	0,263578511	0,689204239	LADA vs Control
hsa-miR-421_ATCAACAGACATTAATTGGGCG3	0,599589108	2,09434672	0,264094596	0,689204239	LADA vs Control
hsa-miR-323b-3p_CCCAATACACGGTCGACCTCT3	-0,571906082	5,853793118	0,264471579	0,689204239	LADA vs Control
hsa-miR-92a-3p_ATTGCACTTGTCCCGGCCTGTT3	-0,396508986	5,604292671	0,264747876	0,689204239	LADA vs Control
hsa-miR-25-3p_CATTGCACTTGTCTCGGTCTGA3	-0,170906592	11,99390817	0,265130714	0,689204239	LADA vs Control
hsa-miR-125a-5p_TCCCTGAGACCCTTTAACC3	0,629078173	2,158659604	0,265269069	0,689204239	LADA vs Control
hsa-miR-100-5p_AACCCGTAGATCCGAACT3	-0,630460238	1,926738636	0,265660479	0,689204239	LADA vs Control
hsa-miR-329-3p_AACACACCTGGTTAACCTCTTT3	-0,659735598	3,759288959	0,266554853	0,689204239	LADA vs Control
hsa-miR-342-3p_TCTCACACAGAAATCGCACCCGTC3	0,229643036	7,583667414	0,266627922	0,689204239	LADA vs Control
hsa-miR-484_CAGGCTCAGTCCCCTCCCGA3	-0,649374318	3,768443499	0,267528463	0,689204239	LADA vs Control
hsa-miR-23a-3p_TCACATTGCCAGGGATTTCC3	-0,548573902	4,540513631	0,268891937	0,689204239	LADA vs Control
hsa-miR-22-3p_AAGCTGCCAGTTGAAGAACTG3	0,20291102	9,134304454	0,269583378	0,689204239	LADA vs Control
hsa-miR-451a_AAACCGTTACCATTACTGAGT3	0,169314983	16,51409079	0,269801409	0,689204239	LADA vs Control
hsa-miR-423-5p_TGAGGGGCAGAGAGCGAGA3	0,130914225	9,723922848	0,272008912	0,689561276	LADA vs Control
hsa-miR-7-5p_TGGAAGACTAGTGATTTTGTTG3	-0,549567518	3,890826404	0,272372175	0,689561276	LADA vs Control
hsa-miR-486-5p_TCCTGTACTGAGCTGCCCCGAGC3	-0,254121164	6,188232343	0,272536765	0,689561276	LADA vs Control
hsa-miR-24-3p_GGCTCAGTTCAGCAGGAACA3	-0,640521238	2,640585933	0,27628202	0,69682522	LADA vs Control
hsa-miR-125a-5p_TCCCTGAGACCCTTTAACCTG3	0,204272897	8,290201937	0,279283459	0,69907864	LADA vs Control
hsa-miR-181a-5p_ACATTCAACGCTGTCGGTGA3	0,59281781	3,569893472	0,280571948	0,69907864	LADA vs Control
hsa-miR-340-5p_TTATAAAGCAATGAGACTGATT3	-0,407067531	5,815012818	0,280979831	0,69907864	LADA vs Control
hsa-let-7i-5p_GAGGTAGTAGTTTGTGCTGTT3	-0,631144833	3,257044134	0,28123187	0,69907864	LADA vs Control
hsa-miR-10b-5p_ACCCTGTAGAACCGAATTTGT3	0,24350846	6,785991828	0,282257917	0,69907864	LADA vs Control
hsa-miR-361-3p_TCCCCCAGGTGTGATTCTGATTT3	-0,559148942	3,977031543	0,282438296	0,69907864	LADA vs Control
hsa-miR-4433b-5p_ATGTCCCACCCCCACTCCTGTT3	-0,424066293	8,226543503	0,284208962	0,701283415	LADA vs Control
hsa-let-7a-5p_TGAGGTAGTAGGTTGTATAGTTT3	-0,240101497	9,423297293	0,28541035	0,702074224	LADA vs Control
hsa-miR-339-5p_TCCCTGTCCTCCAGGAGCTCACG3	-0,632060393	3,416847552	0,288601895	0,707740646	LADA vs Control
hsa-miR-148b-3p_TCAGTGCATCACAGAACTTTGT3	-0,223169402	6,965383646	0,290436702	0,710003713	LADA vs Control
hsa-miR-342-3p_CTCACACAGAAATCGCACCCG3	0,643105903	2,495329338	0,29210888	0,710003713	LADA vs Control
hsa-miR-423-3p_AAGCTCGGTCTGAGGCCCCTCA3	-0,599206866	2,923364981	0,292197262	0,710003713	LADA vs Control
hsa-miR-103a-3p_AGCAGCATTGTACAGGGCTAT3	-0,746809924	3,428123511	0,293674235	0,711423603	LADA vs Control
hsa-let-7i-5p_GAGGTAGTAGTTTGTGCTGT3	0,514757088	4,789553904	0,295867296	0,71389496	LADA vs Control
hsa-miR-29c-3p_TAGCACCATTTGAAATCGGT3	0,569273578	4,339459305	0,297416802	0,71389496	LADA vs Control
hsa-miR-26b-5p_TCAAGTAATTCAGGATAGGTT3	-0,59621033	3,747955651	0,298472	0,71389496	LADA vs Control
hsa-miR-23a-3p_ATCACATTGCCAGGGATTTC3	0,155710001	9,459376505	0,299188923	0,71389496	LADA vs Control
hsa-let-7i-5p_GAGGTAGTAGTTTGTGCTG3	0,622602221	2,272109077	0,299192007	0,71389496	LADA vs Control
hsa-let-7g-5p_GAGGTAGTAGTTTGTACAGTT3	-0,610293345	4,344091711	0,300491212	0,71389496	LADA vs Control
hsa-miR-92b-3p_TATTGCACTCGTCCCGGCCT3	-0,40810681	5,68469958	0,301722665	0,71389496	LADA vs Control
hsa-miR-99b-5p_CACCCGTAGAACCGACCTTGC3	0,166062503	7,880557566	0,303169438	0,71389496	LADA vs Control
hsa-miR-19a-3p_TGTGCAAATCTATGCAAAACTGA3	0,59060743	2,326203362	0,303338132	0,71389496	LADA vs Control
hsa-miR-21-5p_TAGCTTATCAGACTGATGTT3	0,215819391	7,170728426	0,303651683	0,71389496	LADA vs Control
hsa-miR-340-3p_TCCGTCTCAGTTACTTTATAGCC3	-0,611010224	3,094594983	0,305355665	0,714693461	LADA vs Control
hsa-miR-143-3p_TGAGATGAAGCACTGTAGCTC3	-0,165634096	9,842973082	0,305784781	0,714693461	LADA vs Control

hsa-miR-2110_TTGGGGAAACGGCCGCTGAGTGA3	-0,336504962	6,066188802	0,308578614	0,717933954	LADA vs Control
hsa-let-7d-3p_ATACGACCTGCTGCCTTTCT3	-0,592403453	2,048536833	0,30955256	0,717933954	LADA vs Control
hsa-miR-30a-5p_TGTAAACATCCTCGACTGGA3	0,570577605	3,19956619	0,310400131	0,717933954	LADA vs Control
hsa-miR-26a-5p_TTCAAGTAATCCAGGATAGGC3	0,172754833	7,510198846	0,310774422	0,717933954	LADA vs Control
hsa-let-7a-5p_GAGGTAGTAGGTTGTATAGT3	-0,503803474	4,701370889	0,3124655	0,719754345	LADA vs Control
hsa-miR-30d-5p_TGTAAACATCCCCGACTGGAA3	-0,10359177	11,66083446	0,313607111	0,720302211	LADA vs Control
hsa-miR-374a-5p_TTATAATACAACCTGATAAGT3	-0,595510495	2,487762486	0,315398339	0,722334701	LADA vs Control
hsa-miR-125b-5p_TCCCTGAGACCCTAACT3	-0,644509959	2,233733889	0,316332337	0,722397917	LADA vs Control
hsa-miR-128-3p_TCACAGTGAACCGGTCTCT3	0,191108355	7,683472613	0,317272632	0,722475107	LADA vs Control
hsa-miR-432-5p_TCTTGGAGTAGGTCATTGGGTGG3	-0,653254923	3,502755168	0,322129698	0,729129599	LADA vs Control
hsa-miR-451a_AAACCGTTACCATTACTG3	0,536228255	2,491678877	0,323181738	0,729129599	LADA vs Control
hsa-miR-185-5p_TGGAGAGAAAGGCAGTTCCTG3	-0,509417363	4,508281621	0,323799417	0,729129599	LADA vs Control
hsa-miR-30e-5p_TGTAAACATCCTTGACTGGAAGC3	-0,123634865	8,385302075	0,323854301	0,729129599	LADA vs Control
hsa-miR-486-5p_GTACTGAGCTGCCCCGA3	-0,413122402	4,609129379	0,326136849	0,731622874	LADA vs Control
hsa-miR-22-3p_AAGCTGCCAGTTGAAGAAC3	0,192438955	8,203664635	0,32679767	0,731622874	LADA vs Control
hsa-let-7f-5p_TGAGGTAGTAGATTGTATA3	0,543742045	3,61271535	0,328862712	0,733430612	LADA vs Control
hsa-miR-493-5p_TTGTACATGGTAGGCTTTCATT3	-0,632931517	3,64649227	0,32944562	0,733430612	LADA vs Control
hsa-let-7a-5p_GAGGTAGTAGGTTGTATAG3	-0,407634574	5,403501302	0,331352091	0,735620101	LADA vs Control
hsa-miR-223-5p_CGTGTATTTGACAAGCTGAGTTG3	-0,345352966	6,07891061	0,338263159	0,748877048	LADA vs Control
hsa-miR-25-3p_ATTGCACTTGTCTCGGTCT3	-0,48536938	3,620590845	0,3424982	0,753470731	LADA vs Control
hsa-let-7e-5p_TGAGGTAGGAGGTTGTATAGTT3	0,208442781	7,813417368	0,343490499	0,753470731	LADA vs Control
hsa-miR-99a-5p_AACCCGTAGATCCGATCTTGT3	0,164373614	8,529135227	0,344634684	0,753470731	LADA vs Control
hsa-let-7i-5p_TGAGGTAGTAGTTTGTGCT3	-0,210123912	7,615350025	0,345380386	0,753470731	LADA vs Control
hsa-miR-574-3p_CACGCTCATGCACACACCCACA3	-0,286326196	6,300877473	0,346906447	0,753470731	LADA vs Control
hsa-miR-21-5p_TAGCTTATCAGACTGATGTTGACT3	-0,45073497	5,943834972	0,34803025	0,753470731	LADA vs Control
hsa-let-7d-5p_AGAGGTAGTAGGTTGCATAGTT3	-0,196788954	9,075101282	0,348272617	0,753470731	LADA vs Control
hsa-miR-23b-3p_ATCACATTGCCAGGGATTACC3	0,327728065	5,483511586	0,348327577	0,753470731	LADA vs Control
hsa-miR-145-3p_ATTCCTGGAAATACTGTTCTT3	0,542789405	3,50539909	0,349392316	0,753470731	LADA vs Control
hsa-miR-126-5p_ATTATTACTTTTGGTACGCGCT3	0,539001462	2,122638684	0,350578279	0,753470731	LADA vs Control
hsa-miR-501-3p_AATGCACCCGGGCAAGGATTCT3	-0,142319835	7,911300847	0,352140675	0,753470731	LADA vs Control
hsa-miR-155-5p_TTAATGCTAATCGTGATAGGGGTT3	0,567627799	2,993899159	0,353142206	0,753470731	LADA vs Control
hsa-let-7b-5p_TGAGGTAGTAGGTTGTGTGGTT3	-0,089959675	12,77995871	0,353817432	0,753470731	LADA vs Control
hsa-miR-146a-5p_TGAGAACTGAATTCCATGGGTT3	0,113221434	12,1607009	0,355159018	0,753470731	LADA vs Control
hsa-miR-10a-5p_ACCCTGTAGATCCGAATTTGT3	0,206731883	7,643285241	0,356466919	0,753470731	LADA vs Control
hsa-miR-23a-3p_TCACATTGCCAGGGATTTCCAAC3	-0,508353586	2,126412114	0,356688503	0,753470731	LADA vs Control
hsa-miR-589-5p_TGAGAACCACGTCTGCTCTGA3	0,536648665	2,896824842	0,358449308	0,753470731	LADA vs Control
hsa-miR-98-5p_TGAGGTAGTAAGTTGTATTG3	0,461633988	4,611342852	0,359099169	0,753470731	LADA vs Control
hsa-miR-16-2-3p_ACCAATATTACTGTGCTGCT3	-0,429044092	4,606374691	0,360824165	0,753470731	LADA vs Control
hsa-miR-23b-3p_ATCACATTGCCAGGGATTACCA3	0,281972537	6,460852955	0,362329636	0,753470731	LADA vs Control
hsa-miR-122-5p_GGAGTGTGACAATGGTGTTTG3	-0,411389957	5,615777051	0,362497972	0,753470731	LADA vs Control
hsa-let-7i-5p_TGAGGTAGTAGTTTGTGCTGT3	0,107797707	11,39282357	0,362794985	0,753470731	LADA vs Control
hsa-miR-363-3p_ATTGCACGGTATCCATCTG3	0,531496718	2,060471037	0,362841781	0,753470731	LADA vs Control
hsa-miR-23a-5p_GGGGTTCCTGGGGATGGGATTT3	0,501089866	3,81817711	0,363027303	0,753470731	LADA vs Control
hsa-miR-139-3p_TGGAGACGCGGCCCTGTTGGAGT3	0,548362771	3,435697412	0,365242475	0,756099357	LADA vs Control
hsa-miR-221-3p_AGCTACATTGTCTGCTGGGTT3	-0,296913288	6,683307876	0,367534484	0,758873015	LADA vs Control
hsa-miR-744-5p_TGCGGGGCTAGGGCTAACAGC3	-0,47048701	3,991128142	0,368835102	0,759287453	LADA vs Control
hsa-miR-122-5p_TGGAGTGTGACAATGGTGTTTGT3	0,289953687	8,198260953	0,37128338	0,759287453	LADA vs Control
hsa-miR-423-5p_AGGGGCAGAGAGCGAGACTTT3	-0,330788243	5,332499304	0,371464271	0,759287453	LADA vs Control
hsa-miR-361-5p_TTATCAGAATCTCCAGGGGTA3	-0,509140462	3,298750595	0,371545931	0,759287453	LADA vs Control
hsa-miR-133a-3p_TTGGTCCCCTTCAACCAGCTGT3	-0,565985803	2,527387136	0,373331806	0,760603729	LADA vs Control
hsa-let-7b-5p_GAGGTAGTAGGTTGTGTGGTT3	-0,271429993	6,209954592	0,374098697	0,760603729	LADA vs Control
hsa-miR-1306-5p_CCACCTCCCCTGCAAACGTCCA3	-0,547141928	3,283279778	0,37606868	0,762663455	LADA vs Control
hsa-miR-6803-3p_TCCCTCGCCTTCTCACCCTCAGT3	0,556730043	2,14031641	0,37781958	0,76389235	LADA vs Control
hsa-miR-150-5p_TCTCCCAACCCTTGTACCAG3	0,200302425	9,063580505	0,380069241	0,76389235	LADA vs Control
hsa-miR-375-3p_TTTGTTCGTTCGGCTCGCGT3	0,511499316	3,894636907	0,381771147	0,76389235	LADA vs Control
hsa-miR-126-5p_CATTATTACTTTTGGTACGCG3	-0,218765945	6,678225687	0,38285946	0,76389235	LADA vs Control
hsa-miR-10b-5p_ACCCTGTAGAACCGAATTTG3	-0,397911529	4,835525593	0,382967891	0,76389235	LADA vs Control
hsa-miR-182-5p_TTTGGCAATGGTAGAACTCA3	-0,210265581	7,910992501	0,384634271	0,76389235	LADA vs Control
hsa-miR-423-3p_AAGCTCGGTCTGAGGCCCCT3	0,524132678	2,46496229	0,386814726	0,76389235	LADA vs Control
hsa-miR-323a-3p_CACATTACACGGTCGACCTCT3	-0,520982042	3,585538292	0,388258783	0,76389235	LADA vs Control
hsa-miR-501-3p_AATGCACCCGGGCAAGGATT3	0,248439072	6,44960483	0,388734299	0,76389235	LADA vs Control

hsa-miR-140-3p_ACCACAGGGTAGAACCACG3	-0,493842018	2,665108795	0,389669383	0,76389235	LADA vs Control
hsa-let-7d-3p_TATACGACCTGCTGCCTTTC3	-0,224981396	6,429871038	0,389857489	0,76389235	LADA vs Control
hsa-miR-181a-5p_AACATTCAACGCTGTCGGTG3	-0,221794166	6,983532365	0,39027733	0,76389235	LADA vs Control
hsa-miR-125a-5p_TCCCTGAGACCCTTTAACCTGTGA3	-0,183649556	8,237628665	0,390495402	0,76389235	LADA vs Control
hsa-miR-423-3p_AGCTCGGTCTGAGGCCCCTCAG3	-0,110454007	8,372128456	0,390529685	0,76389235	LADA vs Control
hsa-miR-145-5p_GTCCAGTTTTCCCAGGAATCC3	0,496095726	2,283533911	0,391905448	0,76389235	LADA vs Control
hsa-miR-30e-5p_TGTAAACATCCTTGACTGGAA3	-0,195648856	6,536675988	0,392010001	0,76389235	LADA vs Control
hsa-miR-423-3p_AGCTCGGTCTGAGGCCCCT3	0,285057135	6,038348398	0,393690695	0,765057981	LADA vs Control
hsa-miR-409-3p_CGAATGTTGCTCGGTGAACCCCTT3	-0,54590129	3,646109852	0,394528018	0,765057981	LADA vs Control
hsa-miR-1-3p_TGGAATGTAAAGAAGTATGTAT3	-0,450874011	5,995923161	0,397963781	0,766666575	LADA vs Control
hsa-miR-125a-5p_CCCTGAGACCCTTTAACCTGT3	0,35962284	5,351640533	0,397988052	0,766666575	LADA vs Control
hsa-miR-22-3p_AGCTGCCAGTTGAAGAACT3	0,514439902	2,67043052	0,398615804	0,766666575	LADA vs Control
hsa-miR-148a-3p_TCAGTGCACTACAGAACTT3	0,529931397	2,124123364	0,39954142	0,766666575	LADA vs Control
hsa-miR-501-3p_AATGCACCCGGGCAAGGATTC3	-0,482602065	3,102124992	0,400167246	0,766666575	LADA vs Control
hsa-miR-146a-5p_TGAGAACTGAATTCCATGGGTTG3	-0,178823926	7,878355546	0,404228434	0,77154835	LADA vs Control
hsa-miR-93-5p_AAAGTGCTGTTCGTGCAGGTAG3	0,448674286	3,611226251	0,405607857	0,77154835	LADA vs Control
hsa-miR-30a-3p_CTTTCAGTCGGATGTTTGCAG3	0,479168257	1,943754996	0,406316638	0,77154835	LADA vs Control
hsa-miR-191-5p_CAACGGAATCCCAAAAGCAGC3	-0,166854203	8,150484012	0,406587587	0,77154835	LADA vs Control
hsa-miR-151a-3p_TACTAGACTGAAGCTCCTTGAGG3	-0,45377468	4,127573648	0,407993754	0,772377725	LADA vs Control
hsa-miR-92a-3p_TATTGCACTTGTCCCGGCCTG3	-0,120018795	13,95782504	0,413809991	0,774836583	LADA vs Control
hsa-miR-423-3p_GCTCGGTCTGAGGCCCCTCAGT3	-0,266175689	6,432183419	0,414767867	0,774836583	LADA vs Control
hsa-let-7f-5p_GAGGTAGTAGATTGTATAG3	-0,440075017	3,245137192	0,416508261	0,774836583	LADA vs Control
hsa-miR-181a-2-3p_ACCACTGACCGTTGACTGTACC3	-0,493905454	2,397891497	0,416581698	0,774836583	LADA vs Control
hsa-miR-26b-5p_TTCAAGTAATTCAGGATAGGTT3	-0,149415099	9,842825606	0,417255146	0,774836583	LADA vs Control
hsa-miR-25-3p_CATTGCACTTGTCTCGGTCT3	0,124504785	10,26006574	0,418687475	0,774836583	LADA vs Control
hsa-miR-99b-5p_CACCCGTAGAACCGACCTTG3	0,203792536	6,765454723	0,419219761	0,774836583	LADA vs Control
hsa-miR-101-3p_GTACAGTACTGTGATAACTGAA3	0,475699636	3,655178095	0,41966044	0,774836583	LADA vs Control
hsa-miR-193a-5p_TGGGTCTTTGCGGGCGAGA3	0,490799554	3,488597754	0,420099762	0,774836583	LADA vs Control
hsa-miR-3613-5p_TGTTGTACTTTTTTTTTTGTTC3	-0,373974941	4,388236924	0,420166688	0,774836583	LADA vs Control
hsa-miR-142-5p_CCCATAAAGTAGAAAGCACTAC3	-0,203231885	7,021131164	0,421438198	0,774836583	LADA vs Control
hsa-let-7f-5p_TGAGGTAGTAGATTGTATAGTT3	-0,136747897	12,51171334	0,422797692	0,774836583	LADA vs Control
hsa-let-7a-5p_TGAGGTAGTAGGTTGTATAGT3	0,083320207	11,86713156	0,423186321	0,774836583	LADA vs Control
hsa-miR-23b-3p_ATCACATTGCCAGGGATTACCAC3	-0,386475386	5,113281158	0,423815176	0,774836583	LADA vs Control
hsa-miR-191-5p_AACGGAATCCCAAAAGCAGC3	0,452216769	2,920397045	0,424437243	0,774836583	LADA vs Control
hsa-miR-99b-5p_CACCCGTAGAACCGACCTT3	0,46989048	2,002435028	0,424847662	0,774836583	LADA vs Control
hsa-miR-181a-5p_AACATTCAACGCTGTCGGTGAGTT3	0,471119647	2,334562434	0,429199233	0,780985819	LADA vs Control
hsa-miR-192-5p_TGACCTATGAATTGACAGCCAG3	-0,448963129	3,652730706	0,435864169	0,791306931	LADA vs Control
hsa-miR-92a-3p_TGCACTTGTCCCGGCCTGT3	0,308675366	5,28105363	0,437325799	0,792156049	LADA vs Control
hsa-miR-423-3p_AGCTCGGTCTGAGGCCCCTCAGT3	-0,101127545	11,76682308	0,439617808	0,794213052	LADA vs Control
hsa-miR-126-3p_TCGTACCGTGAGTAATAATGC3	-0,190668683	6,426310401	0,44051007	0,794213052	LADA vs Control
hsa-miR-92a-3p_ACTTGTCCCGGCCTGT3	0,458086186	2,134254049	0,441450918	0,794213052	LADA vs Control
hsa-miR-423-5p_CTGAGGGGCAGAGAGCGAGACTTT3	0,406339599	4,419437576	0,443013911	0,795229927	LADA vs Control
hsa-miR-1294_TGTGAGGTTGGCATTGTTGT3	-0,448275165	2,481086687	0,444890744	0,795311237	LADA vs Control
hsa-miR-340-5p_TTATAAAGCAATGAGACTGAT3	-0,449465986	1,968507467	0,445054971	0,795311237	LADA vs Control
hsa-let-7f-5p_TGAGGTAGTAGATTG3	0,416783762	4,164688749	0,448538007	0,799742263	LADA vs Control
hsa-miR-320a-3p_GAAAAGCTGGGTTGAGAGGGCGA3	0,41818237	3,456353792	0,450534526	0,801508967	LADA vs Control
hsa-miR-16-2-3p_CCAATATTACTGTGCTGCTTT3	0,356852155	4,35154905	0,454235777	0,804139453	LADA vs Control
hsa-miR-150-5p_GTCTCCCAACCCTTGTACCAGT3	0,429817691	2,340151294	0,454726705	0,804139453	LADA vs Control
hsa-miR-501-3p_ATGCACCCGGGCAAGGATTCT3	0,449808878	2,081940648	0,455040017	0,804139453	LADA vs Control
hsa-miR-93-5p_CAAAGTGCTGTTCGTGCAGGTA3	-0,30713264	5,442816943	0,456284233	0,804554278	LADA vs Control
hsa-miR-28-3p_CACTAGATTGTGAGCTCCTGGA3	-0,210352796	6,806864009	0,457807573	0,805347647	LADA vs Control
hsa-miR-142-5p_CATAAAGTAGAAAGCACTACT3	0,394729656	3,650597591	0,458755121	0,805347647	LADA vs Control
hsa-miR-30d-5p_TAAACATCCCCGACTGGAAGCT3	-0,421976112	2,174183718	0,46303025	0,811066174	LADA vs Control
hsa-let-7d-3p_CTATACGACCTGCTGCCTTT3	0,098241731	10,24742095	0,468131197	0,818202991	LADA vs Control
hsa-miR-146b-5p_TGAGAACTGAATTCCATAGGCT3	-0,214230366	6,34592746	0,469637765	0,818937163	LADA vs Control
hsa-miR-361-5p_TTATCAGAATCTCCAGGGGTAC3	0,18985762	6,634693775	0,4706063	0,818937163	LADA vs Control
hsa-miR-25-3p_ATTGCACTTGTCTCGGTCTGA3	-0,247993553	5,837958082	0,47191745	0,819429647	LADA vs Control
hsa-miR-329-3p_AACACACCTGGTTAACCTCTT3	-0,430040236	3,637419766	0,476531965	0,824225322	LADA vs Control
hsa-miR-451a_CCGTTACCATTACTGAGT3	0,365055062	4,189894806	0,477430698	0,824225322	LADA vs Control
hsa-miR-10b-5p_ACCCTGTAGAACCGAATTTGTGT3	-0,386791589	3,356457419	0,477781805	0,824225322	LADA vs Control
hsa-miR-125b-5p_TCCCTGAGACCCTAACTTGTG3	-0,309585156	5,039062696	0,483680432	0,832598929	LADA vs Control

hsa-miR-148a-3p_TCAGTGCACTACAGAACTTT3	-0,399563253	4,280926656	0,486571328	0,83411733	LADA vs Control
hsa-miR-92a-3p_TATTGCACTTGTCCCGGCCTGT3	-0,069122164	17,23184948	0,486910065	0,83411733	LADA vs Control
hsa-miR-320b_AAAAGCTGGGTTGAGAGGGCAA3	0,372987671	1,963551094	0,487702228	0,83411733	LADA vs Control
hsa-miR-30a-5p_GTAAACATCCTCGACTGGAAGCT3	0,365069995	4,1060576	0,489068866	0,834663569	LADA vs Control
hsa-miR-1307-3p_CTCGGCGTGGCGTCGGTCGTGG3	-0,421637732	2,190246459	0,490122565	0,83467454	LADA vs Control
hsa-miR-144-3p_TACAGTATAGATGATGTAC3	-0,196901498	6,460339014	0,493074652	0,835682147	LADA vs Control
hsa-miR-16-5p_AGCAGCACGTAAATATTGGCG3	-0,115313722	8,17366975	0,493661894	0,835682147	LADA vs Control
hsa-miR-30d-5p_GTAAACATCCCCGACTGGAA3	-0,289594867	4,975135303	0,493859839	0,835682147	LADA vs Control
hsa-miR-375-3p_TTTGTTCGTTCGGCTCGCGTG3	0,240243438	6,919662337	0,499905355	0,844119847	LADA vs Control
hsa-miR-24-3p_TGGCTCAGTTCAGCAGGAACAG3	0,076335108	12,76835022	0,501635106	0,845249851	LADA vs Control
hsa-miR-30c-5p_TGTAAACATCCTACACTCTCAGC3	0,194507917	6,545485648	0,502972668	0,845715646	LADA vs Control
hsa-miR-10a-5p_TACCCTGTAGATCCGAATTT3	-0,344845192	4,308439737	0,504135834	0,845886862	LADA vs Control
hsa-miR-486-5p_ATCCTGTACTGAGCTGCCCCGA3	0,119509035	8,988899635	0,508422277	0,849532454	LADA vs Control
hsa-miR-342-3p_TCTCACACAGAAATCGCACCCG3	0,142950199	9,658047597	0,508440377	0,849532454	LADA vs Control
hsa-miR-103a-3p_AGCAGCATTGTACAGGGCT3	0,582161756	5,118424827	0,511133755	0,85224603	LADA vs Control
hsa-miR-25-3p_CATTGCACTTGTCTCGGTC3	-0,239340972	5,77988375	0,512277294	0,852369527	LADA vs Control
hsa-miR-183-5p_TATGGCACTGGTAGAATTCA3	-0,192154474	7,32183424	0,515614675	0,854358123	LADA vs Control
hsa-miR-26b-5p_TTCAAGTAATTCAGGATAGGT3	-0,134687052	7,343481575	0,516193039	0,854358123	LADA vs Control
hsa-miR-4433b-5p_ATGTCCCACCCCCACTCCTGT3	-0,247694281	7,752662063	0,517068056	0,854358123	LADA vs Control
hsa-miR-99b-5p_ACCCGTAGAACCGACCTTGCG3	0,376613005	2,082938158	0,517767729	0,854358123	LADA vs Control
hsa-miR-125b-5p_TCCCTGAGACCCTAACTTGT3	0,096363486	9,802438562	0,518832285	0,854358123	LADA vs Control
hsa-miR-142-5p_CATAAAGTAGAAAGCACTA3	-0,351245359	2,153538022	0,520263679	0,854948768	LADA vs Control
hsa-miR-92a-3p_TTGCACTTGTCCCGGCCTGT3	-0,093234936	8,384006303	0,522678277	0,856137208	LADA vs Control
hsa-miR-27a-3p_TTCACAGTGGCTAAGTTCC3	-0,229939019	5,369078847	0,526005467	0,856137208	LADA vs Control
hsa-miR-335-5p_TCAAGAGCAATAACGAAAAAT3	-0,201619579	6,715048961	0,527128561	0,856137208	LADA vs Control
hsa-miR-223-5p_CGTGTATTTGACAAGCTGAGTT3	-0,31996724	3,93706767	0,528161786	0,856137208	LADA vs Control
hsa-miR-146a-5p_TGAGAACTGAATTCCATGGGT3	0,088742967	8,67354952	0,529558017	0,856137208	LADA vs Control
hsa-let-7a-5p_TGAGGTAGTAGGTTGTAT3	0,448921886	3,216663859	0,530453859	0,856137208	LADA vs Control
hsa-miR-146b-5p_TGAGAACTGAATTCCATAGGCTGT3	-0,111613953	8,368012324	0,530959046	0,856137208	LADA vs Control
hsa-miR-16-5p_TAGCAGCACGTAAATATTGGCGT3	-0,123125141	7,90073048	0,532581586	0,856137208	LADA vs Control
hsa-miR-22-3p_AGCTGCCAGTTGAAGAACTGT3	-0,24735969	5,523006419	0,532884424	0,856137208	LADA vs Control
hsa-miR-23a-3p_ATCACATTGCCAGGGATTTCC3	0,067799527	11,08225248	0,535560504	0,856137208	LADA vs Control
hsa-miR-486-5p_CCTGTACTGAGCTGCCCCGA3	-0,098818119	11,08007536	0,535617599	0,856137208	LADA vs Control
hsa-miR-26a-5p_TTCAAGTAATCCAGGATAG3	-0,338956436	3,646262645	0,535829408	0,856137208	LADA vs Control
hsa-miR-92a-3p_TATTGCACTTGTCCCGG3	-0,282536506	4,594134091	0,536307609	0,856137208	LADA vs Control
hsa-miR-98-5p_TGAGGTAGTAAGTTGTATTGTT3	-0,167090654	7,877215034	0,536651259	0,856137208	LADA vs Control
hsa-miR-192-5p_TGACCTATGAATTGACAGCCAGT3	0,341714756	3,322199511	0,537698878	0,856137208	LADA vs Control
hsa-miR-451a_ACCGTTACCATTACTGAGTT3	-0,354189642	2,004581894	0,538174079	0,856137208	LADA vs Control
hsa-miR-28-3p_ACTAGATTGTGAGCTCCTGGAG3	-0,291675307	4,615150746	0,542818396	0,860620678	LADA vs Control
hsa-miR-15b-3p_CGAATCATTATTTGCTGCTCT3	-0,289158027	4,350054835	0,543152071	0,860620678	LADA vs Control
hsa-miR-10a-5p_TACCCTGTAGATCCGAATTTGTGT3	-0,350454215	2,404757373	0,544536281	0,861102016	LADA vs Control
hsa-miR-320a-3p_AAAAGCTGGGTTGAGAGGGCGAA3	0,073417017	9,770949231	0,549884998	0,867838303	LADA vs Control
hsa-miR-126-3p_CGTACCGTGAGTAATAATGC3	-0,271271528	4,46578231	0,551616997	0,86787876	LADA vs Control
hsa-miR-654-5p_TGGTGGGCCGCAGAACATGTGC3	0,399887145	3,027916369	0,552088496	0,86787876	LADA vs Control
hsa-miR-221-3p_AGCTACATTGTCTGCTGGGTTTCA3	-0,352003574	2,390031097	0,554702498	0,868801163	LADA vs Control
hsa-miR-451a_ACCGTTACCATTACTGAG3	-0,315640591	3,857793403	0,555349471	0,868801163	LADA vs Control
hsa-miR-329-3p_ACACACCTGGTTAACCTCTTTT3	-0,367170345	2,079797897	0,555945537	0,868801163	LADA vs Control
hsa-miR-140-3p_TACCACAGGGTAGAACCACGGAC3	0,332985249	3,947173881	0,557676009	0,869053687	LADA vs Control
hsa-miR-501-3p_AATGCACCCGGGCAAGGAT3	-0,341423458	2,760223516	0,558287939	0,869053687	LADA vs Control
hsa-miR-335-5p_TCAAGAGCAATAACGAAAAATGT3	-0,205872956	6,149787911	0,566189742	0,874371693	LADA vs Control
hsa-miR-194-5p_TGTAACAGCAACTCCATGTGGA3	-0,333837521	2,048630169	0,566466961	0,874371693	LADA vs Control
hsa-miR-99a-5p_AACCCGTAGATCCGATCTTG3	0,107835476	8,041845005	0,567141376	0,874371693	LADA vs Control
hsa-let-7d-3p_CTATACGACCTGCTGCCTTTCT3	-0,0666568	11,76128234	0,568269922	0,874371693	LADA vs Control
hsa-let-7d-3p_CTATACGACCTGCTGCCTTTC3	0,060295585	10,32598693	0,571154718	0,874371693	LADA vs Control
hsa-miR-3173-5p_TGCCCTGCCTGTTTTCTCCTT3	0,335932165	2,97364428	0,571166603	0,874371693	LADA vs Control
hsa-miR-342-3p_TCTCACACAGAAATCGCACCCGT3	0,113218253	8,706959622	0,571492398	0,874371693	LADA vs Control
hsa-miR-885-5p_TCCATTACACTACCCTGCCTCT3	-0,655908491	4,714772841	0,572065556	0,874371693	LADA vs Control
hsa-let-7f-5p_GAGGTAGTAGATTGTATAGT3	-0,34785823	3,023408308	0,572098164	0,874371693	LADA vs Control
hsa-miR-495-3p_AAACAAACATGGTGCACTTCTT3	-0,361608431	2,485368847	0,573458181	0,874371693	LADA vs Control
hsa-miR-486-5p_TGTACTGAGCTGCCCCGAG3	-0,249191542	5,036658236	0,57377214	0,874371693	LADA vs Control
hsa-miR-10b-5p_TACCCTGTAGAACCGAATTTG3	0,073325665	9,833049317	0,57493339	0,874469297	LADA vs Control

hsa-miR-4433b-5p_TATGTCCCACCCCCACTCCTG3	-0,375078259	3,068037559	0,577416386	0,876573066	LADA vs Control
hsa-miR-483-3p_TCACTCCTCTCCTCCCGTCT3	-0,349488309	2,217026305	0,579775029	0,878480415	LADA vs Control
hsa-let-7g-5p_TGAGGTAGTAGTTTGTACA3	0,247961706	5,425002874	0,581574448	0,878653397	LADA vs Control
hsa-miR-15b-5p_TAGCAGCACATCATGGTT3	-0,145384455	7,417344831	0,582094095	0,878653397	LADA vs Control
hsa-miR-30c-5p_GTAAACATCCTACACTCTCAGCT3	-0,3263456	3,052199181	0,585261898	0,880478564	LADA vs Control
hsa-miR-361-5p_TTATCAGAATCTCCAGGGGTACT3	0,315143054	2,611707431	0,585512722	0,880478564	LADA vs Control
hsa-miR-24-3p_GGCTCAGTTCAGCAGGAAC3	0,346293361	2,859699768	0,590305578	0,88601421	LADA vs Control
hsa-miR-433-3p_ATCATGATGGGCTCCTCGGTGT3	-0,32864228	4,303379714	0,592717315	0,886519378	LADA vs Control
hsa-miR-361-5p_TTATCAGAATCTCCAGGGGT3	0,229843519	4,747764862	0,59541747	0,886519378	LADA vs Control
hsa-miR-30d-5p_TGTAAACATCCCCGACTGGA3	-0,081530227	9,18587305	0,596621903	0,886519378	LADA vs Control
hsa-miR-1228-3p_TCACACCTGCCTCGCCCCCCA3	0,342676947	1,951009842	0,596695626	0,886519378	LADA vs Control
hsa-miR-30b-5p_TGTAAACATCCTACACTCAGCT3	0,143831516	6,722043115	0,597969727	0,886519378	LADA vs Control
hsa-miR-122-5p_GAGTGTGACAATGGTGTTT3	0,338143958	2,929528654	0,599548243	0,886519378	LADA vs Control
hsa-let-7g-5p_TGAGGTAGTAGTTTGTACAGTT3	0,072045226	11,23391478	0,60045544	0,886519378	LADA vs Control
hsa-miR-181b-5p_AACATTCATTGCTGTCGGTGG3	-0,200457507	5,135854331	0,600526101	0,886519378	LADA vs Control
hsa-miR-2110_TTGGGGAAACGGCCGCTGAGT3	0,178770458	5,974874685	0,600653029	0,886519378	LADA vs Control
hsa-let-7i-5p_TGAGGTAGTAGTTTGTGCTGTTG3	-0,283580176	3,894577335	0,602593564	0,887739501	LADA vs Control
hsa-miR-222-3p_AGCTACATCTGGCTACTGGGT3	0,105789063	7,595221992	0,608676657	0,892656721	LADA vs Control
hsa-let-7f-5p_TGAGGTAGTAGATTGTATAGT3	0,056225451	10,56301224	0,611420584	0,892656721	LADA vs Control
hsa-miR-500a-3p_ATGCACCTGGGCAAGGATTCT3	0,263447519	4,513942296	0,61142104	0,892656721	LADA vs Control
hsa-miR-151a-3p_TACTAGACTGAAGCTCCTTGA3	-0,286097453	1,940948476	0,611422159	0,892656721	LADA vs Control
hsa-miR-122-5p_TGGAGTGTGACAATGGTGTTTG3	0,14342753	11,63439763	0,612648318	0,892656721	LADA vs Control
hsa-miR-142-5p_CATAAAGTAGAAAGCACTAC3	-0,297447293	2,376484394	0,613281186	0,892656721	LADA vs Control
hsa-miR-199a-3p_ACAGTAGTCTGCACATTGGTT3	-0,4583013	4,64378834	0,613771497	0,892656721	LADA vs Control
hsa-miR-486-3p_CGGGGCAGCTCAGTACAGGA3	-0,161715192	6,539637794	0,615437624	0,892985739	LADA vs Control
hsa-miR-320a-3p_AAAAGCTGGGTTGAGAGGGCGAAAA3	-0,293803446	3,186668725	0,616238591	0,892985739	LADA vs Control
hsa-miR-10a-5p_TACCCTGTAGATCCGAATTTG3	0,077558743	8,855203262	0,617477838	0,893157599	LADA vs Control
hsa-miR-451a_CGTTACCATTACTGAGT3	0,251534908	4,012413349	0,620212455	0,893574954	LADA vs Control
hsa-miR-483-5p_AAGACGGGAGGAAAGAAGGGAG3	0,178115734	6,618430366	0,622240455	0,893574954	LADA vs Control
hsa-miR-140-3p_ACCACAGGGTAGAACCACGG3	-0,267829697	3,491351538	0,622726397	0,893574954	LADA vs Control
hsa-miR-425-5p_AATGACACGATCACTCCCG3	-0,243051096	4,547943713	0,622885869	0,893574954	LADA vs Control
hsa-miR-1908-5p_CGGCGGGGACGGCGATTGGT3	-0,309181943	2,686402078	0,624704325	0,893574954	LADA vs Control
hsa-miR-3605-3p_CCTCCGTGTTACCTGTCCTCT3	-0,219253756	5,168364179	0,625841867	0,893574954	LADA vs Control
hsa-miR-16-5p_GCAGCACGTAAATATTGGCG3	-0,25290005	3,625120505	0,626494554	0,893574954	LADA vs Control
hsa-miR-4433b-5p_TGTCCCACCCCCACTCCTG3	0,313140393	2,497163245	0,627271848	0,893574954	LADA vs Control
hsa-miR-25-3p_ATTGCACTTGTCTCGGTCTG3	-0,254261943	3,200649251	0,627856932	0,893574954	LADA vs Control
hsa-miR-3158-3p_AAGGGCTTCCTCTCTGCAGGA3	0,299418278	2,734692583	0,629461311	0,894261435	LADA vs Control
hsa-miR-23a-3p_TCACATTGCCAGGGATTTC3	0,290565401	2,403793771	0,63215749	0,896493807	LADA vs Control
hsa-miR-182-5p_TTTGGCAATGGTAGAACTCACACTG3	0,28620734	3,598930548	0,635126534	0,899104525	LADA vs Control
hsa-miR-152-3p_TCAGTGCATGACAGAACTTGG3	-0,25698044	2,073986305	0,636811148	0,899195886	LADA vs Control
hsa-miR-505-3p_GTCAACACTTGCTGGTTTCCTCT3	0,288528762	2,066469778	0,639623439	0,899195886	LADA vs Control
hsa-miR-361-3p_TCCCCCAGGTGTGATTCTGA3	0,256875568	3,113214743	0,641148796	0,899195886	LADA vs Control
hsa-let-7c-5p_TGAGGTAGTAGGTTGTATGG3	-0,176200996	3,04066672	0,641807063	0,899195886	LADA vs Control
hsa-miR-320a-3p_AAAGCTGGGTTGAGAGGGCGA3	0,234200879	4,98279454	0,642939246	0,899195886	LADA vs Control
hsa-miR-26a-5p_TTCAAGTAATCCAGGATAGGCT3	-0,080994252	11,92893731	0,643532566	0,899195886	LADA vs Control
hsa-miR-339-3p_TGAGCGCCTCGACGACAGAG3	-0,19431792	5,154516456	0,645779821	0,899195886	LADA vs Control
hsa-miR-99a-5p_AACCCGTAGATCCGATCTT3	-0,271139726	3,048788798	0,646252526	0,899195886	LADA vs Control
hsa-miR-92a-3p_TATTGCACTTGTCCCGGCCT3	0,071810505	10,27945719	0,64639233	0,899195886	LADA vs Control
hsa-miR-199a-3p_ACAGTAGTCTGCACATTGGTTA3	0,418478553	4,827856003	0,646473329	0,899195886	LADA vs Control
hsa-miR-181a-2-3p_ACCACTGACCGTTGACTGTAC3	-0,258741612	3,544826687	0,647751648	0,899404292	LADA vs Control
hsa-miR-1306-5p_CCACCTCCCCTGCAAACGTCC3	-0,222059339	4,76371819	0,649907335	0,90082808	LADA vs Control
hsa-miR-150-5p_CTCCCAACCCTTGTACCAGT3	-0,261826369	3,181356832	0,656368659	0,907274942	LADA vs Control
hsa-miR-193a-5p_TGGGTCTTTGCGGGCGAGAT3	-0,205897681	4,669337288	0,657319662	0,907274942	LADA vs Control
hsa-let-7d-5p_AGAGGTAGTAGGTTGCATAG3	-0,086036204	8,573362788	0,657973546	0,907274942	LADA vs Control
hsa-miR-16-2-3p_ACCAATATTACTGTGCTGCTT3	0,07360017	8,312552057	0,664612622	0,914237661	LADA vs Control
hsa-let-7b-3p_CTATACAACCTACTGCCTTCC3	-0,126696882	6,023895535	0,666378719	0,914237661	LADA vs Control
hsa-let-7g-5p_GAGGTAGTAGTTTGTACAGT3	-0,255755674	3,423010066	0,666464342	0,914237661	LADA vs Control
hsa-miR-150-5p_GTCTCCCAACCCTTGTACCAGTG3	0,256722488	2,975164068	0,668760873	0,915811711	LADA vs Control
hsa-miR-21-5p_AGCTTATCAGACTGATGTTGA3	0,097473155	6,721668578	0,670227016	0,916245166	LADA vs Control
hsa-miR-21-5p_GTAGCTTATCAGACTGATGTTGA3	-0,251277566	2,217922154	0,675966676	0,920812786	LADA vs Control
hsa-miR-140-3p_ACCACAGGGTAGAACCACGGACA3	-0,212519489	4,192134912	0,676570231	0,920812786	LADA vs Control

hsa-miR-423-5p_GAGGGGCAGAGAGCGAGACTTTT3	-0,219193624	4,029696332	0,677034244	0,920812786	LADA vs Control
hsa-miR-98-5p_TGAGGTAGTAAGTTGTATTGT3	-0,137898334	6,55627531	0,678875372	0,921587875	LADA vs Control
hsa-miR-222-3p_AGCTACATCTGGCTACTGGG3	0,247025874	2,549442498	0,680216591	0,921587875	LADA vs Control
hsa-miR-374b-5p_ATATAATACAACCTGCTAAGTG3	-0,234188098	2,723967557	0,683003518	0,921587875	LADA vs Control
hsa-miR-17-5p_CAAAGTGCTTACAGTGCAGGTAG3	-0,237232573	3,276961308	0,684328448	0,921587875	LADA vs Control
hsa-miR-128-3p_TCACAGTGAACCGGTCTCTT3	-0,092752121	6,808413156	0,685464001	0,921587875	LADA vs Control
hsa-miR-423-3p_AAGCTCGGTCTGAGGCCCCTCAGT3	0,101593999	7,286433576	0,687068105	0,921587875	LADA vs Control
hsa-miR-144-5p_GGATATCATCATATACTGTAAGT3	0,118774796	6,405140764	0,688366321	0,921587875	LADA vs Control
hsa-miR-92a-3p_TTGCACTTGTCCCGGCCTG3	-0,204331406	4,313701526	0,68867127	0,921587875	LADA vs Control
hsa-miR-181a-5p_AACATTCAACGCTGTCGGT3	-0,150177978	5,505188114	0,691294005	0,921587875	LADA vs Control
hsa-miR-30c-5p_TGTAAACATCCTACACTCTCAGCT3	-0,06106424	10,20131272	0,695430252	0,921587875	LADA vs Control
hsa-miR-425-5p_AATGACACGATCACTCCCGT3	-0,103725562	6,738341736	0,696398471	0,921587875	LADA vs Control
hsa-miR-192-5p_TGACCTATGAATTGACAGCCA3	-0,194574677	4,726203979	0,698291397	0,921587875	LADA vs Control
hsa-miR-451a_GAAACCGTTACCATTACTGAG3	0,208839019	3,176702933	0,699662045	0,921587875	LADA vs Control
hsa-miR-23a-3p_TCACATTGCCAGGGATTTCCA3	0,161570798	5,236791923	0,701744185	0,921587875	LADA vs Control
hsa-miR-92a-3p_ATTGCACTTGTCCCGGCCTGT3	-0,043136922	12,31240602	0,701785738	0,921587875	LADA vs Control
hsa-miR-182-5p_TTTGGCAATGGTAGAACTCACACT3	-0,090453087	7,651119737	0,702844339	0,921587875	LADA vs Control
hsa-miR-15b-5p_TAGCAGCACATCATGGTTT3	-0,06741252	8,514548835	0,704029154	0,921587875	LADA vs Control
hsa-miR-224-5p_CAAGTCACTAGTGGTTCCGTTT3	-0,22290157	2,885758309	0,705193821	0,921587875	LADA vs Control
hsa-miR-532-3p_CCTCCCACACCCAAGGCTTG3	0,227205132	2,745637111	0,706252712	0,921587875	LADA vs Control
hsa-miR-10b-5p_TACCCTGTAGAACCGAATTT3	0,150852307	5,2763128	0,707521737	0,921587875	LADA vs Control
hsa-miR-182-5p_TTTGGCAATGGTAGAACTCAC3	-0,213978605	3,148572393	0,709703308	0,921587875	LADA vs Control
hsa-miR-374a-5p_TTATAATACAACCTGATAAGTG3	-0,173206186	4,272038394	0,71028022	0,921587875	LADA vs Control
hsa-miR-24-3p_TGGCTCAGTTCAGCAGGAACA3	0,043225467	9,988806411	0,71160929	0,921587875	LADA vs Control
hsa-miR-320a-3p_AAAAGCTGGGTTGAGAGGGCGAAA3	-0,108353461	6,744969015	0,714736015	0,921587875	LADA vs Control
hsa-miR-191-5p_AACGGAATCCCAAAAGCAGCTG3	0,125710997	5,747755673	0,714838229	0,921587875	LADA vs Control
hsa-miR-191-5p_CAACGGAATCCCAAAAGC3	-0,210376843	3,190312182	0,715206157	0,921587875	LADA vs Control
hsa-miR-487b-3p_TCGTACAGGGTCATCCACTTT3	0,228290872	2,776617041	0,718122467	0,921587875	LADA vs Control
hsa-miR-92a-3p_GTATTGCACTTGTCCCGGCCTGT3	0,065070659	7,715362913	0,71821716	0,921587875	LADA vs Control
hsa-let-7a-5p_TGAGGTAGTAGGTTGTATAGTTTT3	-0,223372025	3,044731157	0,718660372	0,921587875	LADA vs Control
hsa-miR-505-3p_CGTCAACACTTGCTGGTTT3	-0,202836509	3,249239604	0,720177985	0,921587875	LADA vs Control
hsa-miR-30e-5p_TGTAAACATCCTTGACTGGAAG3	-0,065881306	7,619075745	0,721279761	0,921587875	LADA vs Control
hsa-miR-486-5p_TCCTGTACTGAGCTGCCC3	-0,141491541	3,88539215	0,721734815	0,921587875	LADA vs Control
hsa-miR-181b-5p_AACATTCATTGCTGTCGGTG3	0,208145163	2,058689202	0,722060935	0,921587875	LADA vs Control
hsa-miR-16-5p_CTAGCAGCACGTAAATATTGGCG3	-0,159336309	4,959212286	0,722392583	0,921587875	LADA vs Control
hsa-miR-3173-5p_CCCTGCCTGTTTTCTCCTTTGT3	0,191974944	1,879782778	0,724285601	0,921587875	LADA vs Control
hsa-miR-451a_GAAACCGTTACCATTACTGAGT3	-0,086595683	6,98332366	0,724557543	0,921587875	LADA vs Control
hsa-miR-21-5p_TAGCTTATCAGACTGATG3	0,201934213	1,940313304	0,725589681	0,921587875	LADA vs Control
hsa-miR-144-3p_TACAGTATAGATGATGTACT3	0,171483301	4,501383498	0,727165736	0,921587875	LADA vs Control
hsa-miR-24-3p_GCTCAGTTCAGCAGGAACAG3	-0,193231818	2,25101941	0,72835993	0,921587875	LADA vs Control
hsa-let-7a-5p_ATGAGGTAGTAGGTTGTATAGTT3	-0,212659925	2,103462699	0,728967526	0,921587875	LADA vs Control
hsa-miR-942-5p_TCTTCTCTGTTTTGGCCATGT3	-0,204932627	2,592733465	0,729719545	0,921587875	LADA vs Control
hsa-miR-363-3p_AATTGCACGGTATCCATCTG3	-0,061600241	8,264741807	0,730564346	0,921587875	LADA vs Control
hsa-miR-103a-3p_AGCAGCATTGTACAGGGCTATGA3	-0,077311006	8,631709381	0,731769211	0,921587875	LADA vs Control
hsa-miR-24-3p_GGCTCAGTTCAGCAGGAACAG3	-0,082829368	6,812383366	0,732307464	0,921587875	LADA vs Control
hsa-miR-222-3p_AGCTACATCTGGCTACTGGGTC3	0,180963293	3,362212617	0,732883505	0,921587875	LADA vs Control
hsa-miR-146a-5p_GAGAACTGAATTCCATGGGTT3	-0,113620535	6,481720435	0,733722431	0,921587875	LADA vs Control
hsa-miR-146a-5p_TGAGAACTGAATTCCATGGG3	-0,190664155	2,424854764	0,733730868	0,921587875	LADA vs Control
hsa-miR-145-5p_GTCCAGTTTTCCCAGGAATCCCT3	-0,20677022	2,146327793	0,735440212	0,921587875	LADA vs Control
hsa-miR-7-5p_TGGAAGACTAGTGATTTTGTTGTT3	0,191629446	4,415695214	0,736390144	0,921587875	LADA vs Control
hsa-miR-320a-3p_GAAAAGCTGGGTTGAGAGGGCG3	0,110679873	6,114738168	0,737319934	0,921587875	LADA vs Control
hsa-miR-486-5p_CTGTACTGAGCTGCCCCGAG3	-0,071903349	7,787642307	0,737670062	0,921587875	LADA vs Control
hsa-miR-92a-3p_TATTGCACTTGTCCCGGC3	0,09131538	6,608463448	0,738551065	0,921587875	LADA vs Control
hsa-miR-142-5p_CATAAAGTAGAAAGCACT3	-0,095887308	5,539841362	0,74071661	0,921587875	LADA vs Control
hsa-miR-22-3p_AAGCTGCCAGTTGAAGAA3	0,164063667	4,931866064	0,741244613	0,921587875	LADA vs Control
hsa-miR-30c-5p_TGTAAACATCCTACACTCTCAG3	-0,154125697	4,767658059	0,74215849	0,921587875	LADA vs Control
hsa-miR-26a-5p_TCAAGTAATCCAGGATAGGCT3	-0,101845908	6,522605962	0,742358113	0,921587875	LADA vs Control
hsa-miR-93-5p_CAAAGTGCTGTTCGTGCAGGTAGT3	-0,180202908	4,129822813	0,745489458	0,924035922	LADA vs Control
hsa-miR-16-2-3p_CCAATATTACTGTGCTGCTT3	-0,13644046	4,446564737	0,748010613	0,924329592	LADA vs Control
hsa-miR-30d-5p_TGTAAACATCCCCGACTGGAAG3	0,026794206	12,16128446	0,748453212	0,924329592	LADA vs Control
hsa-miR-186-5p_AAAGAATTCTCCTTTTGGGCT3	-0,178872727	3,890936215	0,749205667	0,924329592	LADA vs Control

hsa-miR-191-5p_CAACGGAATCCCAAAAGCAGCTGT3	0,142931546	5,688164696	0,755839998	0,930393301	LADA vs Control
hsa-miR-16-5p_AGCAGCACGTAAATATTGGC3	-0,173932023	2,995434135	0,757525984	0,930393301	LADA vs Control
hsa-miR-191-5p_ACGGAATCCCAAAAGCAGCT3	-0,181746699	1,904502114	0,757622651	0,930393301	LADA vs Control
hsa-miR-193a-5p_TGGGTCTTTGCGGGCGAGATG3	0,119224354	5,884704134	0,767377149	0,939657948	LADA vs Control
hsa-miR-16-5p_TTAGCAGCACGTAAATATTGGCG3	-0,150859452	4,199452303	0,769276475	0,939657948	LADA vs Control
hsa-let-7c-5p_TGAGGTAGTAGGTTGTATGGTTT3	-0,153798732	3,566946574	0,769853089	0,939657948	LADA vs Control
hsa-miR-629-5p_TGGGTTTACGTTGGGAGAACTT3	-0,149654236	4,034612462	0,77505323	0,939657948	LADA vs Control
hsa-miR-142-5p_CCATAAAGTAGAAAGCACT3	-0,109553566	4,774985235	0,778352622	0,939657948	LADA vs Control
hsa-miR-338-5p_AACAATATCCTGGTGCTGAGT3	-0,092504777	5,993094666	0,778587581	0,939657948	LADA vs Control
hsa-miR-192-5p_TGACCTATGAATTGACAGCC3	-0,084225728	6,449264096	0,77968774	0,939657948	LADA vs Control
hsa-miR-16-5p_AGCAGCACGTAAATATTGG3	-0,155805234	3,167012316	0,780311514	0,939657948	LADA vs Control
hsa-miR-15b-5p_TAGCAGCACATCATGGTTTAC3	0,059813746	7,368236859	0,780853239	0,939657948	LADA vs Control
hsa-let-7f-5p_TGAGGTAGTAGATTGTAT3	0,174979632	3,174737293	0,780972455	0,939657948	LADA vs Control
hsa-miR-532-5p_CATGCCTTGAGTGTAGGACCG3	-0,155157794	2,388684445	0,781005802	0,939657948	LADA vs Control
hsa-miR-629-5p_TGGGTTTACGTTGGGAGAACT3	0,067886008	7,034835335	0,781298958	0,939657948	LADA vs Control
hsa-miR-342-5p_AGGGGTGCTATCTGTGATTGA3	-0,167732388	3,958584465	0,781943299	0,939657948	LADA vs Control
hsa-miR-183-5p_TATGGCACTGGTAGAATT3	0,166501498	2,805102899	0,783532859	0,939657948	LADA vs Control
hsa-miR-144-5p_GGATATCATCATATACTGTAAG3	-0,163878178	4,224705339	0,783954428	0,939657948	LADA vs Control
hsa-miR-375-3p_TTGTTCGTTCGGCTCGCGTGA3	-0,117460732	5,662970821	0,784988759	0,939657948	LADA vs Control
hsa-miR-342-3p_TCACACAGAAATCGCACCCGTCA3	-0,15205759	3,930198822	0,786009589	0,939657948	LADA vs Control
hsa-miR-375-3p_TTTGTTCGTTCGGCTCGCG3	0,109957288	5,830563474	0,786388772	0,939657948	LADA vs Control
hsa-miR-183-5p_TATGGCACTGGTAGAATTCACTG3	-0,161120997	3,516695948	0,788667367	0,940969898	LADA vs Control
hsa-miR-451a_AACCGTTACCATTACTGAG3	0,051818798	8,074719646	0,790567618	0,941827192	LADA vs Control
hsa-miR-374b-5p_ATATAATACAACCTGCTAAGT3	-0,138290034	3,95574137	0,792810556	0,942084197	LADA vs Control
hsa-miR-363-3p_ATTGCACGGTATCCATCTGT3	0,132663375	4,343538892	0,793147423	0,942084197	LADA vs Control
hsa-miR-584-5p_TTATGGTTTGCCTGGGACT3	-0,119870859	4,595669388	0,799410965	0,944820942	LADA vs Control
hsa-miR-191-5p_AACGGAATCCCAAAAGCAGCT3	-0,067768987	6,816281646	0,800150866	0,944820942	LADA vs Control
hsa-miR-382-5p_AAGTTGTTCGTGGTGGATTCG3	0,161067186	2,227150711	0,800258163	0,944820942	LADA vs Control
hsa-miR-30a-5p_TGTAAACATCCTCGACTGG3	0,123594364	4,494972654	0,800345093	0,944820942	LADA vs Control
hsa-miR-21-5p_TAGCTTATCAGACTGATGTTGA3	-0,021654377	12,88530966	0,801378866	0,944820942	LADA vs Control
hsa-miR-100-5p_AACCCGTAGATCCGAACTTG3	0,078524843	5,941970306	0,80464638	0,947272031	LADA vs Control
hsa-miR-21-5p_TAGCTTATCAGACTGATGT3	-0,119351769	4,699515355	0,80947427	0,950128618	LADA vs Control
hsa-miR-2110_TTGGGGAAACGGCCGCTGAGTG3	-0,149299788	2,932814217	0,809812638	0,950128618	LADA vs Control
hsa-miR-30e-3p_CTTTCAGTCGGATGTTTACAG3	0,139315354	2,31966353	0,81064926	0,950128618	LADA vs Control
hsa-let-7d-3p_TATACGACCTGCTGCCTTT3	-0,064744778	6,275229414	0,813975515	0,951171379	LADA vs Control
hsa-miR-10a-5p_TACCCTGTAGATCCGAATT3	-0,131291785	3,933865051	0,814836253	0,951171379	LADA vs Control
hsa-miR-32-5p_TATTGCACATTACTAAGTTGC3	0,137225784	2,00688677	0,815119262	0,951171379	LADA vs Control
hsa-miR-181a-2-3p_ACCACTGACCGTTGACTGT3	-0,133305324	2,531276889	0,817654503	0,952553392	LADA vs Control
hsa-miR-423-5p_GAGGGGCAGAGAGCGAGACTTT3	-0,030434146	8,388365906	0,818693944	0,952553392	LADA vs Control
hsa-miR-15b-5p_TAGCAGCACATCATGGTTTACA3	0,068660543	6,32576652	0,829492331	0,962397059	LADA vs Control
hsa-miR-345-5p_GCTGACTCCTAGTCCAGGGCTC3	0,127569708	3,656556535	0,829569359	0,962397059	LADA vs Control
hsa-miR-183-5p_ATGGCACTGGTAGAATTCACTG3	0,127506211	2,888906189	0,831833583	0,962830145	LADA vs Control
hsa-miR-486-5p_TCCTGTACTGAGCTGCCCCG3	-0,035568768	14,12822837	0,832358808	0,962830145	LADA vs Control
hsa-miR-181a-5p_AACATTCAACGCTGTCGGTGA3	0,022519824	9,980124889	0,837359999	0,966717004	LADA vs Control
hsa-miR-16-5p_TAGCAGCACGTAAATATTGGCG3	-0,024036632	14,03055319	0,838144855	0,966717004	LADA vs Control
hsa-miR-664a-5p_ACTGGCTAGGGAAAATGATTGG3	-0,109495311	3,587998944	0,840334811	0,967842261	LADA vs Control
hsa-miR-32-5p_TATTGCACATTACTAAGTTG3	0,109273613	3,984130302	0,842704142	0,968004333	LADA vs Control
hsa-miR-629-5p_TGGGTTTACGTTGGGAGAAC3	0,112251957	3,68380122	0,845136953	0,968004333	LADA vs Control
hsa-miR-10a-5p_ACCCTGTAGATCCGAATTTG3	-0,063473382	5,718851669	0,845835298	0,968004333	LADA vs Control
hsa-miR-193b-5p_CGGGGTTTTGAGGGCGAGATGA3	0,121173028	2,282241357	0,846257558	0,968004333	LADA vs Control
hsa-let-7b-5p_TGAGGTAGTAGGTTGTGT3	0,075193101	5,292834315	0,847239224	0,968004333	LADA vs Control
hsa-miR-125b-5p_TCCCTGAGACCCTAACTTG3	0,033042909	8,171400371	0,849204742	0,968004333	LADA vs Control
hsa-miR-4732-3p_GCCCTGACCTGTCCTGTTCTG3	-0,171526981	4,156168491	0,852038642	0,968004333	LADA vs Control
hsa-miR-142-5p_CCCATAAAGTAGAAAGCAC3	-0,068749944	5,201334155	0,85256492	0,968004333	LADA vs Control
hsa-miR-183-5p_TATGGCACTGGTAGAATTCACT3	0,075191117	5,796604044	0,854009467	0,968004333	LADA vs Control
hsa-miR-486-5p_GTACTGAGCTGCCCCGAG3	0,088162871	4,883397866	0,8559701	0,968004333	LADA vs Control
hsa-miR-451a_AAACCGTTACCATTACTGAGTTT3	0,030787205	8,99265157	0,856238614	0,968004333	LADA vs Control
hsa-miR-505-3p_CGTCAACACTTGCTGGTTTCCT3	0,10067096	3,899410508	0,857190513	0,968004333	LADA vs Control
hsa-miR-3615_TCTCTCGGCTCCTCGCGGC3	0,103233553	2,019798482	0,857345661	0,968004333	LADA vs Control
hsa-miR-22-3p_AAGCTGCCAGTTGAAGAACTGTT3	-0,103702554	2,793276847	0,857479371	0,968004333	LADA vs Control
hsa-miR-92a-3p_ATTGCACTTGTCCCGGCCTG3	-0,029727721	9,121941427	0,860654679	0,970204815	LADA vs Control

hsa-miR-221-3p_AGCTACATTGTCTGCTGGGTTT3	-0,032225146	7,916859704	0,864163711	0,970204815	LADA vs Control
hsa-let-7g-5p_TGAGGTAGTAGTTTGTACAGT3	-0,014975855	10,7052364	0,866977439	0,970204815	LADA vs Control
hsa-miR-1249-3p_ACGCCCTTCCCCCCCTTCTTCA3	0,108223473	2,195504284	0,868296649	0,970204815	LADA vs Control
hsa-miR-143-3p_TGAGATGAAGCACTGTAGCT3	-0,02941451	9,047255511	0,868988342	0,970204815	LADA vs Control
hsa-miR-451a_ACCGTTACCATTACTGAGT3	-0,042062803	7,452815255	0,869944763	0,970204815	LADA vs Control
hsa-miR-16-5p_TAGCAGCACGTAAATATTGG3	0,022852261	10,97240393	0,870841135	0,970204815	LADA vs Control
hsa-miR-30c-5p_TGTAAACATCCTACACTCTCA3	0,036001488	7,955436132	0,871218753	0,970204815	LADA vs Control
hsa-miR-221-3p_AGCTACATTGTCTGCTGGGT3	0,053051018	6,226471989	0,871378865	0,970204815	LADA vs Control
hsa-miR-451a_AAACCGTTACCATTACTGAGTTTAG3	0,093724152	2,164703683	0,871601816	0,970204815	LADA vs Control
hsa-miR-92a-3p_TATTGCACTTGTCCCGGCCTGTG3	-0,027277289	7,105169059	0,873492902	0,970953756	LADA vs Control
hsa-miR-29b-3p_TAGCACCATTTGAAATCAGT3	-0,079471414	4,363760053	0,876208232	0,972615545	LADA vs Control
hsa-miR-664a-3p_TATTCATTTATCCCCAGCCTACA3	-0,084741293	3,022366025	0,881467874	0,976094478	LADA vs Control
hsa-miR-28-3p_CACTAGATTGTGAGCTCCTGG3	0,048587689	5,876182682	0,882435839	0,976094478	LADA vs Control
hsa-miR-451a_AAACCGTTACCATTACTGA3	0,0274862	8,929457213	0,88301646	0,976094478	LADA vs Control
hsa-miR-93-3p_ACTGCTGAGCTAGCACTTCCCGA3	0,081266691	3,371774968	0,888524695	0,979484547	LADA vs Control
hsa-miR-660-5p_TACCCATTGCATATCGGAGTTGT3	0,075319613	3,90808508	0,888541189	0,979484547	LADA vs Control
hsa-miR-191-5p_CAACGGAATCCCAAAAGCA3	0,041384552	6,203677568	0,889830313	0,979550773	LADA vs Control
hsa-miR-148b-3p_TCAGTGCATCACAGAACTTTG3	-0,062391885	4,882892663	0,891946352	0,980525852	LADA vs Control
hsa-miR-106b-5p_TAAAGTGCTGACAGTGCAGAT3	0,07219818	2,655083548	0,895782871	0,983386981	LADA vs Control
hsa-let-7b-3p_CTATACAACCTACTGCCTTC3	-0,071581529	3,159200497	0,8982135	0,983785896	LADA vs Control
hsa-miR-150-3p_CTGGTACAGGCCTGGGGGACA3	0,070635469	4,060108896	0,898614972	0,983785896	LADA vs Control
hsa-miR-103a-3p_AGCAGCATTGTACAGGGCTATG3	-0,069540762	4,276844631	0,90213676	0,986286692	LADA vs Control
hsa-miR-99b-5p_CACCCGTAGAACCGACCTTGCG3	0,019907071	9,378733966	0,904031595	0,986658849	LADA vs Control
hsa-miR-423-3p_AGCTCGGTCTGAGGCCCC3	0,065039265	3,540860079	0,907334861	0,986658849	LADA vs Control
hsa-miR-29a-3p_CTAGCACCATCTGAAATCGG3	-0,063954839	3,050349736	0,907425293	0,986658849	LADA vs Control
hsa-miR-197-3p_TTCACCACCTTCTCCACCCAG3	-0,021562373	8,762482756	0,907640406	0,986658849	LADA vs Control
hsa-miR-93-5p_CAAAGTGCTGTTCGTGCAGGT3	0,042674023	4,918359962	0,912026355	0,986658849	LADA vs Control
hsa-miR-3613-5p_TGTTGTACTTTTTTTTTTGT3	0,018653406	7,586394808	0,912259824	0,986658849	LADA vs Control
hsa-miR-7-5p_TGGAAGACTAGTGATTTTGTTGT3	0,044780394	6,176558156	0,912689813	0,986658849	LADA vs Control
hsa-miR-24-3p_TGGCTCAGTTCAGCAGGAA3	0,045441956	5,648746767	0,913050753	0,986658849	LADA vs Control
hsa-miR-92b-3p_TATTGCACTCGTCCCGGCC3	-0,057768372	3,837730088	0,916771222	0,986658849	LADA vs Control
hsa-let-7g-5p_GAGGTAGTAGTTTGTACAG3	-0,060959383	2,076526463	0,919327138	0,986658849	LADA vs Control
hsa-miR-30e-5p_GTAAACATCCTTGACTGGAAGC3	0,059926446	2,49990334	0,919916032	0,986658849	LADA vs Control
hsa-miR-29a-3p_CTAGCACCATCTGAAATCGGTT3	-0,051413924	3,457099062	0,921279728	0,986658849	LADA vs Control
hsa-miR-451a_AACCGTTACCATTACTGAGT3	-0,015283276	10,87302044	0,92568078	0,986658849	LADA vs Control
hsa-miR-4732-5p_TGTAGAGCAGGGAGCAGGAAGCT3	-0,0310889	6,276959061	0,9260434	0,986658849	LADA vs Control
hsa-miR-150-5p_TCTCCCAACCCTTGTACCA3	-0,031901458	6,588601483	0,927511157	0,986658849	LADA vs Control
hsa-miR-425-5p_AATGACACGATCACTCCCGTT3	-0,019602511	8,024407696	0,927712717	0,986658849	LADA vs Control
hsa-miR-423-5p_GAGGGGCAGAGAGCGAGA3	-0,05276109	1,916584014	0,928733426	0,986658849	LADA vs Control
hsa-miR-484_TCAGGCTCAGTCCCCTCCCGA3	0,014850011	10,80053241	0,930492292	0,986658849	LADA vs Control
hsa-miR-223-3p_TGTCAGTTTGTCAAATACCC3	0,041985828	4,516322797	0,93168226	0,986658849	LADA vs Control
hsa-miR-148a-3p_CAGTGCACTACAGAACTTTGT3	-0,050886109	2,126116505	0,933028735	0,986658849	LADA vs Control
hsa-miR-100-5p_AACCCGTAGATCCGAACTTGTG3	-0,051194643	3,590895666	0,933100776	0,986658849	LADA vs Control
hsa-miR-486-5p_CCTGTACTGAGCTGCCCCG3	-0,018299163	8,015321722	0,933572964	0,986658849	LADA vs Control
hsa-miR-182-5p_TTTGGCAATGGTAGAACTCACACTGG3	0,051308794	2,315372824	0,933701033	0,986658849	LADA vs Control
hsa-miR-92a-3p_CACTTGTCCCGGCCTGT3	0,044553693	4,072780053	0,934029946	0,986658849	LADA vs Control
hsa-miR-451a_AACCGTTACCATTACTGAGTTT3	-0,047781649	2,400911204	0,934452595	0,986658849	LADA vs Control
hsa-miR-423-5p_GAGGGGCAGAGAGCGAGACT3	-0,025699511	6,874414686	0,935317695	0,986658849	LADA vs Control
hsa-miR-425-5p_ATGACACGATCACTCCCGTTGA3	-0,038340982	4,729605379	0,935902246	0,986658849	LADA vs Control
hsa-miR-424-3p_CAAAACGTGAGGCGCTGCTAT3	-0,026711531	5,519904886	0,938448361	0,987664839	LADA vs Control
hsa-miR-3615_TCTCTCGGCTCCTCGCGGCTCGC3	0,038568403	3,835837244	0,941318891	0,987664839	LADA vs Control
hsa-miR-652-3p_AATGGCGCCACTAGGGTTG3	0,039723539	4,499546053	0,943188632	0,987664839	LADA vs Control
hsa-miR-4685-3p_TCTCCCTTCCTGCCCTGGCT3	0,033274916	4,862788515	0,943578573	0,987664839	LADA vs Control
hsa-miR-144-5p_GGATATCATCATATACTGTAA3	-0,039899374	3,901874288	0,945316191	0,987664839	LADA vs Control
hsa-miR-194-5p_TGTAACAGCAACTCCATGTGG3	0,017735085	6,660853419	0,945835761	0,987664839	LADA vs Control
hsa-miR-192-5p_TGACCTATGAATTGACAGC3	-0,03899918	3,665629227	0,948817614	0,987664839	LADA vs Control
hsa-miR-23a-3p_ATCACATTGCCAGGGATTTCCAA3	0,022043682	6,530990344	0,948832373	0,987664839	LADA vs Control
hsa-miR-148a-3p_TCAGTGCACTACAGAACTTTG3	-0,013191423	7,287277231	0,950916955	0,987664839	LADA vs Control
hsa-miR-486-5p_CTGTACTGAGCTGCCCCGA3	-0,012166033	7,583597035	0,952818203	0,987664839	LADA vs Control
hsa-miR-342-3p_TCTCACACAGAAATCGCACCC3	-0,032362752	2,049205595	0,955930273	0,987664839	LADA vs Control
hsa-miR-29c-3p_TAGCACCATTTGAAATCGG3	-0,031279092	3,272314395	0,956578256	0,987664839	LADA vs Control

hsa-miR-3615_TCTCTCGGCTCCTCGCGGCT3	-0,015278159	6,584121427	0,956710527	0,987664839	LADA vs Control
hsa-miR-193a-5p_TGGGTCTTTGCGGGCGAGATGA3	-0,009102639	8,630407946	0,95880315	0,987664839	LADA vs Control
hsa-miR-92a-3p_ATTGCACTTGTCCCGGCCT3	-0,026340329	4,477303001	0,959745093	0,987664839	LADA vs Control
hsa-miR-30e-5p_TGTAAACATCCTTGACTGGA3	0,029044703	2,511168014	0,960476882	0,987664839	LADA vs Control
hsa-miR-328-3p_CTGGCCCTCTCTGCCCTTCCG3	0,024732797	3,770890003	0,961311512	0,987664839	LADA vs Control
hsa-miR-29a-3p_TAGCACCATCTGAAATCGGTTAT3	-0,027234529	2,486065735	0,963074491	0,987664839	LADA vs Control
hsa-miR-126-5p_CATTATTACTTTTGGTACGC3	-0,02439883	3,620244945	0,964088735	0,987664839	LADA vs Control
hsa-miR-142-5p_CCCATAAAGTAGAAAGCACT3	0,004504077	11,13638127	0,964885125	0,987664839	LADA vs Control
hsa-miR-502-3p_AATGCACCTGGGCAAGGATTCA3	0,018393113	4,709212787	0,965930862	0,987664839	LADA vs Control
hsa-miR-28-5p_AAGGAGCTCACAGTCTATTGA3	-0,027038035	3,11393162	0,966057036	0,987664839	LADA vs Control
hsa-miR-4446-3p_CAGGGCTGGCAGTGACATGGGT3	0,026476317	3,598813459	0,967538593	0,987664839	LADA vs Control
hsa-let-7i-5p_TGAGGTAGTAGTTTGTGCTG3	0,006053681	10,07708045	0,969052158	0,987664839	LADA vs Control
hsa-miR-4433b-5p_ATGTCCCACCCCCACTCCTG3	-0,020152401	2,006165102	0,97237222	0,987664839	LADA vs Control
hsa-miR-486-5p_ATCCTGTACTGAGCTGCCCCGAG3	0,007133684	7,990918242	0,972440171	0,987664839	LADA vs Control
hsa-miR-361-3p_TCCCCCAGGTGTGATTCTGATTTG3	0,012669774	5,241633262	0,972948479	0,987664839	LADA vs Control
hsa-miR-126-3p_CTCGTACCGTGAGTAATAATGCG3	-0,016948268	4,682348296	0,972996792	0,987664839	LADA vs Control
hsa-miR-26a-5p_TTCAAGTAATCCAGGATAGG3	-0,007030775	6,813190078	0,975011692	0,987664839	LADA vs Control
hsa-miR-181a-5p_AACATTCAACGCTGTCGG3	-0,016188076	2,192700831	0,975962699	0,987664839	LADA vs Control
hsa-miR-342-3p_TCACACAGAAATCGCACCCGTC3	0,016792227	2,309823181	0,97695083	0,987664839	LADA vs Control
hsa-miR-92a-3p_GTATTGCACTTGTCCCGGCCTG3	-0,015223221	3,065032086	0,979095271	0,987664839	LADA vs Control
hsa-miR-30e-5p_TAAACATCCTTGACTGGAAGCT3	-0,015399553	2,076536633	0,979366103	0,987664839	LADA vs Control
hsa-miR-126-3p_GTACCGTGAGTAATAATGCG3	0,014359147	2,067690175	0,980187575	0,987664839	LADA vs Control
hsa-miR-223-3p_TCAGTTTGTCAAATACCCCAA3	0,013799324	2,060154898	0,980744526	0,987664839	LADA vs Control
hsa-miR-486-5p_TGTACTGAGCTGCCCCGA3	-0,012134854	4,617811312	0,981468699	0,987664839	LADA vs Control
hsa-miR-100-5p_AACCCGTAGATCCGAACTTGT3	-0,005191916	7,211971456	0,983894728	0,988857627	LADA vs Control
hsa-miR-486-5p_TCCTGTACTGAGCTGCCCCGA3	0,002456688	17,03890679	0,986498739	0,989106802	LADA vs Control
hsa-miR-192-5p_CTGACCTATGAATTGACAGC3	0,010034255	1,977431527	0,986624727	0,989106802	LADA vs Control
hsa-let-7a-5p_TGAGGTAGTAGGTTGTATAGTT3	0,001791419	13,39549393	0,990155123	0,991399037	LADA vs Control
hsa-miR-451a_AAACCGTTACCATTACTGAG3	-0,000305909	13,72832534	0,998567291	0,998567291	LADA vs Control
hsa-miR-128-3p_TCACAGTGAACCGGTCTCTTTT4	-1,540219138	5,404238817	6,07303E-05	0,026720516	T1D vs Control
hsa-miR-126-5p_CATTATTACTTTTGGTACGCG4	-0,942836686	6,678225687	6,96688E-05	0,026720516	T1D vs Control
hsa-miR-223-3p_TGTCAGTTTGTCAAATACC4	-1,497628125	5,409428462	0,000130738	0,026720516	T1D vs Control
hsa-miR-543_AAACATTCGCGGTGCACTTCTT4	-1,967242578	3,845833985	0,000193109	0,026720516	T1D vs Control
hsa-miR-140-3p_TACCACAGGGTAGAACCACGG4	-1,169774192	5,563733367	0,0002071	0,026720516	T1D vs Control
hsa-miR-23a-3p_TCACATTGCCAGGGATTTCCAAC4	-1,930147155	2,126412114	0,000218614	0,026720516	T1D vs Control
hsa-miR-221-3p_AGCTACATTGTCTGCTGGGTTTC4	-1,121247523	6,752581432	0,000234685	0,026720516	T1D vs Control
hsa-miR-4433b-5p_ATGTCCCACCCCCACTCCTGTTT4	-1,668313324	5,620502964	0,000282271	0,02709671	T1D vs Control
hsa-miR-30d-5p_TGTAAACATCCCCGACTGGAAGCT4	-0,440963948	13,20515822	0,00033838	0,02709671	T1D vs Control
hsa-miR-22-3p_AAGCTGCCAGTTGAAGAACTGT4	0,301017992	12,01293	0,000339984	0,02709671	T1D vs Control
hsa-miR-101-3p_GTACAGTACTGTGATAACTG4	-1,264606196	5,734652626	0,000622811	0,04512551	T1D vs Control
hsa-miR-30d-5p_TGTAAACATCCCCGACTGGAAGC4	-0,334591763	11,59455452	0,000738296	0,049035191	T1D vs Control
hsa-miR-92a-3p_TATTGCACTTGTCCCGG4	-1,447901832	4,594134091	0,000835221	0,051205445	T1D vs Control
hsa-miR-30e-3p_CTTTCAGTCGGATGTTTACAGC4	-1,827287261	3,314941715	0,00092225	0,051518118	T1D vs Control
hsa-miR-375-3p_TTTGTTCGTTCGGCTCGCGTGA4	0,770527185	10,08392589	0,000969601	0,051518118	T1D vs Control
hsa-miR-215-5p_ATGACCTATGAATTGACAGA4	1,655711973	4,790158483	0,001208802	0,058126	T1D vs Control
hsa-miR-142-5p_CCCATAAAGTAGAAAGCA4	-1,525915197	2,550307907	0,001239827	0,058126	T1D vs Control
hsa-miR-151a-3p_CTAGACTGAAGCTCCTTGAGGA4	-0,558981675	8,444952042	0,001393488	0,061700568	T1D vs Control
hsa-miR-23b-3p_ATCACATTGCCAGGGATTACC4	-1,047111481	5,483511586	0,00166714	0,069932146	T1D vs Control
hsa-miR-30d-5p_GTAAACATCCCCGACTGGAAGCT4	-0,75579599	6,998439264	0,00244909	0,097596218	T1D vs Control
hsa-miR-181b-5p_AACATTCATTGCTGTCGGTGGGT4	-1,425338806	3,594503523	0,00288084	0,10933472	T1D vs Control
hsa-miR-186-5p_CAAAGAATTCTCCTTTTGGGC4	-0,826065225	6,535812742	0,003029224	0,109740535	T1D vs Control
hsa-miR-423-3p_AGCTCGGTCTGAGGCCCCTCA4	-0,936398896	6,171912051	0,003415975	0,113881702	T1D vs Control
hsa-let-7d-3p_CTATACGACCTGCTGCCTT4	-1,063084371	4,847124715	0,003429311	0,113881702	T1D vs Control
hsa-miR-142-3p_TGTAGTGTTTCCTACTTTATGGA4	1,595899	3,252758122	0,00376208	0,117341074	T1D vs Control
hsa-miR-222-3p_AGCTACATCTGGCTACTGGGTCTC4	-1,130723378	6,101293098	0,00382794	0,117341074	T1D vs Control
hsa-miR-151a-3p_TACTAGACTGAAGCTCCTTGAGG4	-1,477593831	4,127573648	0,003980676	0,117503672	T1D vs Control
hsa-miR-340-3p_TCCGTCTCAGTTACTTTATAGCC4	-1,594807894	3,094594983	0,004630914	0,131815665	T1D vs Control
hsa-miR-425-5p_AATGACACGATCACTCCCGTTGAGT4	-0,647625676	8,386717544	0,004924566	0,135340652	T1D vs Control
hsa-miR-379-5p_TGGTAGACTATGGAACGTAGG4	-1,648973346	3,839701767	0,005115969	0,135914238	T1D vs Control
hsa-miR-4732-5p_TGTAGAGCAGGGAGCAGGAAG4	-1,571378678	3,155376397	0,005456507	0,14028504	T1D vs Control
hsa-miR-409-3p_GAATGTTGCTCGGTGAACCCCTTT4	-1,612685934	4,139609499	0,005923191	0,147524482	T1D vs Control

hsa-miR-99b-5p_CACCCGTAGAACCGACCTTGCG4	-0,426074925	9,378733966	0,006216838	0,150146051	T1D vs Control
hsa-miR-142-5p_CATAAAGTAGAAAGCACT4	-0,73282369	5,539841362	0,006693255	0,152181463	T1D vs Control
hsa-miR-144-5p_GATATCATCATATACTGTAAGTT4	-1,528671211	2,459029553	0,006952317	0,152181463	T1D vs Control
hsa-miR-3173-5p_TGCCCTGCCTGTTTTCTCCTTT4	-1,143882236	4,766192857	0,007021589	0,152181463	T1D vs Control
hsa-miR-25-3p_ATTGCACTTGTCTCGGTCT4	-1,292189606	3,620590845	0,007064886	0,152181463	T1D vs Control
hsa-miR-223-5p_CGTGTATTTGACAAGCTGAGTTGG4	-1,53120582	2,566442122	0,007404563	0,155300956	T1D vs Control
hsa-miR-424-3p_CAAAACGTGAGGCGCTGCT4	1,417351617	2,803316018	0,007778622	0,157837199	T1D vs Control
hsa-miR-335-5p_TCAAGAGCAATAACGAAAAATGT4	-0,89225184	6,149787911	0,008238008	0,157837199	T1D vs Control
hsa-miR-652-3p_AATGGCGCCACTAGGGTTGT4	0,41121785	7,904624885	0,008397434	0,157837199	T1D vs Control
hsa-miR-125a-5p_TCCCTGAGACCCTTTAACCTGTGA4	-0,528905264	8,237628665	0,00848553	0,157837199	T1D vs Control
hsa-miR-320a-3p_AAAAGCTGGGTTGAGAGGGCG4	0,316577349	10,00052492	0,008515683	0,157837199	T1D vs Control
hsa-miR-30d-5p_TGTAAACATCCCCGACTGGA4	-0,377137009	9,18587305	0,009266709	0,164483759	T1D vs Control
hsa-miR-142-5p_CCCATAAAGTAGAAAGCAC4	-0,896864817	5,201334155	0,009287038	0,164483759	T1D vs Control
hsa-miR-92a-3p_TATTGCACTTGTCCCGGCCTGTT4	-0,312860672	10,41769689	0,009529409	0,164505989	T1D vs Control
hsa-miR-423-5p_TGAGGGGCAGAGAGCGAGACTT4	0,238020265	12,19565674	0,009701106	0,164505989	T1D vs Control
hsa-miR-150-5p_TCTCCCAACCCTTGTACCAGTG4	0,382871066	10,93881631	0,010646497	0,174688943	T1D vs Control
hsa-miR-1301-3p_TTGCAGCTGCCTGGGAGTGACTTC4	-1,47632766	2,697320641	0,010739973	0,174688943	T1D vs Control
hsa-miR-340-3p_TCCGTCTCAGTTACTTTATAGC4	-1,29966668	1,820184636	0,0125076	0,199371144	T1D vs Control
hsa-miR-30d-5p_GTAAACATCCCCGACTGGAA4	-0,989991555	4,975135303	0,013103395	0,20456064	T1D vs Control
hsa-miR-92a-3p_ATTGCACTTGTCCCGGCCTGTT4	-0,833174288	5,604292671	0,01349213	0,20456064	T1D vs Control
hsa-miR-329-3p_ACACACCTGGTTAACCTCTTTT4	-1,440151179	2,079797897	0,013828785	0,20456064	T1D vs Control
hsa-miR-29a-3p_TAGCACCATCTGAAATCGG4	0,588989739	7,213630267	0,013859818	0,20456064	T1D vs Control
hsa-miR-3615_TCTCTCGGCTCCTCGCGGCTCG4	0,326055959	8,091694617	0,014623139	0,211488737	T1D vs Control
hsa-let-7f-5p_GAGGTAGTAGATTGTATAG4	-1,242974091	3,245137192	0,014859936	0,211488737	T1D vs Control
hsa-miR-584-5p_TTATGGTTTGCCTGGGACTGA4	-0,479751221	8,070467342	0,015193584	0,212443619	T1D vs Control
hsa-miR-485-5p_AGAGGCTGGCCGTGATGAATTCG4	-1,28203736	4,543642396	0,015903819	0,215201036	T1D vs Control
hsa-miR-483-5p_AAGACGGGAGGAAAGAAGGGA4	1,290391008	4,456894738	0,016187545	0,215201036	T1D vs Control
hsa-miR-145-5p_GTCCAGTTTTCCCAGGAATCCCT4	-1,379748476	2,146327793	0,016327482	0,215201036	T1D vs Control
hsa-miR-142-5p_CCCATAAAGTAGAAAGCACTA4	-0,860875012	5,405299379	0,016870286	0,215201036	T1D vs Control
hsa-miR-186-5p_CAAAGAATTCTCCTTTTGGGCT4	-0,296199417	9,032677455	0,017210658	0,215201036	T1D vs Control
hsa-miR-186-5p_CAAAGAATTCTCCTTTTGGGCTT4	-0,748653298	6,345307365	0,017235437	0,215201036	T1D vs Control
hsa-miR-4433b-5p_TGTCCCACCCCCACTCCTGTTT4	-0,764293085	8,596146134	0,017280886	0,215201036	T1D vs Control
hsa-miR-222-3p_AGCTACATCTGGCTACTGGGTCT4	-0,648581199	6,5045561	0,018056491	0,22140036	T1D vs Control
hsa-miR-30d-5p_GTAAACATCCCCGACTGGAAGC4	-1,005932043	4,874885211	0,018835523	0,227453207	T1D vs Control
hsa-miR-423-5p_AGGGGCAGAGAGCGAGACTTT4	-0,811715973	5,332499304	0,019495145	0,231904936	T1D vs Control
hsa-miR-4433b-5p_TGTCCCACCCCCACTCCTGT4	-1,122568068	4,771975689	0,020655252	0,238658929	T1D vs Control
hsa-let-7i-5p_GAGGTAGTAGTTTGTGCTGTT4	-1,276788313	3,257044134	0,020661814	0,238658929	T1D vs Control
hsa-miR-409-3p_CGAATGTTGCTCGGTGAACCCCTTT4	-1,420098822	2,598256943	0,021080342	0,24001475	T1D vs Control
hsa-miR-374b-5p_ATATAATACAACCTGCTAAGTG4	-1,229163523	2,723967557	0,022217631	0,249374867	T1D vs Control
hsa-miR-122-5p_TGGAGTGTGACAATGGTGTTT4	0,590026951	13,55430102	0,022528219	0,249374867	T1D vs Control
hsa-let-7d-5p_AGAGGTAGTAGGTTGCATAGTTT4	-1,058891386	5,420694347	0,023070452	0,251878768	T1D vs Control
hsa-miR-30e-5p_TGTAAACATCCTTGACTGGAAGCT4	-0,163548406	11,72480372	0,023474877	0,252830773	T1D vs Control
hsa-miR-150-3p_CTGGTACAGGCCTGGGGGACA4	-1,179950551	4,060108896	0,024141614	0,256544886	T1D vs Control
hsa-miR-320a-3p_GAAAAGCTGGGTTGAGAGGGCG4	0,696078033	6,114738168	0,025515289	0,267574805	T1D vs Control
hsa-miR-329-3p_AACACACCTGGTTAACCTCTT4	-1,261553311	3,637419766	0,026136274	0,270527405	T1D vs Control
hsa-miR-28-3p_CACTAGATTGTGAGCTCCTGGAG4	1,168615021	2,845963244	0,028020769	0,286314786	T1D vs Control
hsa-miR-423-5p_TGAGGGGCAGAGAGCGAGACT4	0,252023125	13,29479943	0,028801712	0,290569174	T1D vs Control
hsa-let-7a-5p_TGAGGTAGTAGGTTGTATAGTTT4	-0,455471961	9,423297293	0,030011833	0,298992888	T1D vs Control
hsa-miR-495-3p_AAACAAACATGGTGCACTTCTT4	-1,302460922	2,485368847	0,030550373	0,299117299	T1D vs Control
hsa-miR-6803-3p_TCCCTCGCCTTCTCACCCTC4	-1,174469082	2,004774634	0,030858961	0,299117299	T1D vs Control
hsa-miR-369-3p_AATAATACATGGTTGATCTTT4	-1,184805364	4,326255371	0,031381665	0,299117299	T1D vs Control
hsa-miR-323a-3p_CACATTACACGGTCGACCTCT4	-1,214486819	3,585538292	0,031694752	0,299117299	T1D vs Control
hsa-miR-21-5p_AGCTTATCAGACTGATGTTGAC4	-1,257047037	3,189772648	0,032368532	0,299117299	T1D vs Control
hsa-miR-221-3p_AGCTACATTGTCTGCTGGGTTTCA4	-1,195371334	2,390031097	0,032579531	0,299117299	T1D vs Control
hsa-miR-21-5p_TAGCTTATCAGACTGATGTTGAC4	-0,477777412	10,44971368	0,032651449	0,299117299	T1D vs Control
hsa-miR-126-3p_TCGTACCGTGAGTAATAATGC4	-0,495560056	6,426310401	0,033483848	0,302823948	T1D vs Control
hsa-miR-140-3p_TACCACAGGGTAGAACCACG4	-0,792053838	5,026346909	0,0341644	0,302823948	T1D vs Control
hsa-miR-29a-3p_TAGCACCATCTGAAATCGGT4	0,38601525	8,663780214	0,034489296	0,302823948	T1D vs Control
hsa-let-7d-3p_TATACGACCTGCTGCCTTTC4	-0,518830022	6,429871038	0,034992626	0,302823948	T1D vs Control
hsa-miR-483-5p_AAGACGGGAGGAAAGAAGGGAGT4	1,221864734	3,821071217	0,035486631	0,302823948	T1D vs Control
hsa-miR-425-5p_AATGACACGATCACTCCCGTTGA4	-0,215179218	10,37803313	0,035578498	0,302823948	T1D vs Control

hsa-miR-199a-3p_ACAGTAGTCTGCACATTGGT4	-1,186936457	1,952185074	0,035981143	0,302823948	T1D vs Control
hsa-miR-486-5p_TCCTGTACTGAGCTGCCCCGAGG4	-0,370233003	7,467002764	0,036095703	0,302823948	T1D vs Control
hsa-miR-26b-5p_TTCAAGTAATTCAGGATAGGTT4	-0,360578487	9,842825606	0,036879918	0,305645181	T1D vs Control
hsa-miR-451a_GAAACCGTTACCATTACTGAG4	-1,055534676	3,176702933	0,0376353	0,305645181	T1D vs Control
hsa-miR-191-5p_CAACGGAATCCCAAAAGCAGCT4	-0,255029624	11,11779618	0,037790248	0,305645181	T1D vs Control
hsa-miR-125a-5p_TCCCTGAGACCCTTTAACCT4	-0,500920014	6,34415738	0,038380598	0,305645181	T1D vs Control
hsa-miR-654-3p_TATGTCTGCTGACCATCACC4	-1,224718693	2,711988254	0,038571642	0,305645181	T1D vs Control
hsa-miR-423-5p_GAGGGGCAGAGAGCGAGACTTTT4	-1,010389317	4,029696332	0,039454668	0,305645181	T1D vs Control
hsa-miR-148b-3p_TCAGTGCATCACAGAACTTTGT4	-0,405267177	6,965383646	0,039483332	0,305645181	T1D vs Control
hsa-miR-130b-5p_ACTCTTTCCCTGTTGCACTACT4	-0,719513932	5,742085569	0,03963135	0,305645181	T1D vs Control
hsa-miR-101-3p_TACAGTACTGTGATAACTGAA4	-1,132699625	3,070531279	0,039883436	0,305645181	T1D vs Control
hsa-miR-142-5p_CATAAAGTAGAAAGCACTA4	-1,048726083	2,153538022	0,040616792	0,308300796	T1D vs Control
hsa-let-7c-5p_TGAGGTAGTAGGTTGTATGGT4	0,729780147	4,585920454	0,042502099	0,317336778	T1D vs Control
hsa-miR-223-3p_TGTCAGTTTGTCAAATACCCCAA4	-0,376363307	10,91791178	0,042754339	0,317336778	T1D vs Control
hsa-miR-16-5p_CTAGCAGCACGTAAATATTGGCG4	-0,846448855	4,959212286	0,043062165	0,317336778	T1D vs Control
hsa-miR-26b-5p_TTCAAGTAATTCAGGATAGG4	-0,920520042	3,436379997	0,043399886	0,317336778	T1D vs Control
hsa-miR-194-5p_TGTAACAGCAACTCCATGTGGA4	-1,097814925	2,048630169	0,04391392	0,318176313	T1D vs Control
hsa-miR-100-5p_AACCCGTAGATCCGAACT4	-1,059161476	1,926738636	0,045409153	0,326045897	T1D vs Control
hsa-miR-485-3p_GTCATACACGGCTCTCCTCTCT4	-1,003905477	5,913980943	0,04644216	0,330485728	T1D vs Control
hsa-miR-151a-3p_TACTAGACTGAAGCTCCTTGAG4	-1,063146418	3,055291554	0,04714128	0,332492039	T1D vs Control
hsa-miR-320a-3p_AAAAGCTGGGTTGAGAGGGCGA4	0,206381909	10,74836293	0,048339474	0,337952289	T1D vs Control
hsa-miR-423-3p_AAGCTCGGTCTGAGGCCCCTCA4	-1,044846602	2,923364981	0,049154386	0,340661269	T1D vs Control
hsa-miR-409-3p_AATGTTGCTCGGTGAACCCCT4	-1,098027765	4,366315065	0,051129957	0,34958332	T1D vs Control
hsa-miR-122-5p_TGGAGTGTGACAATGGTGTTTGT4	0,594420599	8,198260953	0,052008858	0,34958332	T1D vs Control
hsa-miR-425-5p_AATGACACGATCACTCCCGTTGAG4	-0,733280121	6,063293525	0,052190716	0,34958332	T1D vs Control
hsa-miR-3615_TCTCTCGGCTCCTCGCGGCTCGC4	-0,95524809	3,835837244	0,05236965	0,34958332	T1D vs Control
hsa-miR-30c-5p_GTAAACATCCTACACTCTCAGCT4	-1,08146776	3,052199181	0,053205903	0,34958332	T1D vs Control
hsa-miR-122-5p_TGGAGTGTGACAATGGTGTTTG4	0,515186225	11,63439763	0,053263461	0,34958332	T1D vs Control
hsa-miR-375-3p_TTGTTCGTTCGGCTCGCGTGA4	0,783014397	5,662970821	0,053512127	0,34958332	T1D vs Control
hsa-miR-10a-5p_ACCCTGTAGATCCGAATTTGTG4	0,57556798	6,001615458	0,054464865	0,352914615	T1D vs Control
hsa-let-7d-5p_AGAGGTAGTAGGTTGCATAGT4	-0,447161933	7,455921597	0,055608103	0,3574166	T1D vs Control
hsa-miR-182-5p_TTTGGCAATGGTAGAACTCAC4	-1,028193271	3,148572393	0,056123824	0,357845499	T1D vs Control
hsa-miR-199a-5p_CCCAGTGTTCAGACTACCTGTTC4	-0,907299707	4,567460238	0,057524967	0,361076915	T1D vs Control
hsa-miR-338-5p_AACAATATCCTGGTGCTGAGT4	-0,58346103	5,993094666	0,057821781	0,361076915	T1D vs Control
hsa-miR-375-3p_TTTGTTCGTTCGGCTCGCGTG4	0,631612782	6,919662337	0,058062142	0,361076915	T1D vs Control
hsa-miR-584-5p_TTATGGTTTGCCTGGGACT4	-0,828766663	4,595669388	0,058442813	0,361076915	T1D vs Control
hsa-miR-329-3p_AACACACCTGGTTAACCTCTTT4	-1,039456968	3,759288959	0,060681598	0,372024872	T1D vs Control
hsa-miR-101-3p_GTACAGTACTGTGATAACT4	-0,789681041	4,990449736	0,061204962	0,372369118	T1D vs Control
hsa-miR-27a-3p_TTCACAGTGGCTAAGTTCCGC4	-0,508835096	6,28650114	0,062613939	0,378055375	T1D vs Control
hsa-miR-425-5p_AATGACACGATCACTCCCGTTG4	-0,227312276	9,36928751	0,063988394	0,383313571	T1D vs Control
hsa-miR-23a-3p_ATCACATTGCCAGGGATTTCCA4	-0,252613134	11,15500363	0,065858986	0,383313571	T1D vs Control
hsa-miR-425-5p_ATGACACGATCACTCCCGTTGAGT4	-0,833664071	4,72212581	0,066039592	0,383313571	T1D vs Control
hsa-let-7f-5p_TGAGGTAGTAGATTGTATAGTTT4	-0,461779577	8,114676232	0,066063338	0,383313571	T1D vs Control
hsa-miR-10a-5p_ACCCTGTAGATCCGAATTTG4	-0,567103285	5,718851669	0,066417667	0,383313571	T1D vs Control
hsa-let-7b-5p_TGAGGTAGTAGGTTGTGT4	-0,673847139	5,292834315	0,067164352	0,383313571	T1D vs Control
hsa-miR-4433b-5p_ATGTCCCACCCCCACTCCTGTT4	-0,673698062	8,226543503	0,067904271	0,383313571	T1D vs Control
hsa-miR-3605-3p_CCTCCGTGTTACCTGTCCTCT4	-0,776250744	5,168364179	0,067928471	0,383313571	T1D vs Control
hsa-miR-185-5p_TGGAGAGAAAGGCAGTTCCTGA4	-0,245834457	8,851967404	0,068558416	0,383313571	T1D vs Control
hsa-miR-370-3p_GCCTGCTGGGGTGGAACCTGGT4	-1,085786713	2,498088509	0,06871283	0,383313571	T1D vs Control
hsa-miR-451a_GAAACCGTTACCATTACTGAGT4	-0,416951711	6,98332366	0,068775208	0,383313571	T1D vs Control
hsa-miR-222-3p_AGCTACATCTGGCTACTGGGTCTCT4	-0,450293581	7,967322162	0,069510725	0,384033751	T1D vs Control
hsa-miR-339-5p_TCCCTGTCCTCCAGGAGCTCACG4	-1,007691156	3,416847552	0,070280046	0,384033751	T1D vs Control
hsa-miR-320a-3p_AAAAGCTGGGTTGAGAGGGCGAA4	0,20776515	9,770949231	0,07049244	0,384033751	T1D vs Control
hsa-miR-183-5p_TATGGCACTGGTAGAATTC4	-0,954114928	3,376816673	0,070831821	0,384033751	T1D vs Control
hsa-miR-130a-3p_CAGTGCAATGTTAAAAGGGCA4	0,806616207	4,214432691	0,072903365	0,392594475	T1D vs Control
hsa-miR-382-5p_GAAGTTGTTCGTGGTGGATTCG4	-0,604536572	7,607601722	0,073686919	0,394150834	T1D vs Control
hsa-miR-125b-5p_TCCCTGAGACCCTAACTTGTGA4	-0,647033819	5,6455681	0,074776978	0,396302191	T1D vs Control
hsa-miR-30e-5p_TAAACATCCTTGACTGGAAGCT4	-0,989923458	2,076536633	0,07520237	0,396302191	T1D vs Control
hsa-miR-423-5p_TGAGGGGCAGAGAGCGAGA4	0,198050353	9,723922848	0,075922345	0,396302191	T1D vs Control
hsa-miR-146a-5p_TGAGAACTGAATTCCATGGGTTGT4	-0,499286335	7,265118969	0,076309265	0,396302191	T1D vs Control
hsa-miR-1180-3p_TTTCCGGCTCGCGTGGGTGT4	-0,810575573	4,070138113	0,076575329	0,396302191	T1D vs Control

hsa-miR-25-3p_ATTGCACTTGTCTCGGTCTGA4	-0,570382349	5,837958082	0,078680339	0,397486135	T1D vs Control
hsa-miR-574-3p_CACGCTCATGCACACACCCAC4	-0,828456324	4,117697394	0,078789354	0,397486135	T1D vs Control
hsa-let-7d-3p_CTATACGACCTGCTGCCTTTCT4	-0,191619073	11,76128234	0,079185957	0,397486135	T1D vs Control
hsa-miR-23a-3p_TCACATTGCCAGGGATTTCCA4	-0,690024759	5,236791923	0,079201277	0,397486135	T1D vs Control
hsa-miR-584-5p_TTATGGTTTGCCTGGGACTG4	-0,916037514	2,558465624	0,080136391	0,397486135	T1D vs Control
hsa-let-7b-3p_CTATACAACCTACTGCCTTCC4	-0,477983021	6,023895535	0,080873959	0,397486135	T1D vs Control
hsa-miR-1249-3p_ACGCCCTTCCCCCCCTTCTTCA4	-1,064476124	2,195504284	0,081161904	0,397486135	T1D vs Control
hsa-miR-125b-5p_TCCCTGAGACCCTAACTT4	-0,873070283	3,016083692	0,081207549	0,397486135	T1D vs Control
hsa-miR-24-3p_TGGCTCAGTTCAGCAGGAACA4	0,19001212	9,988806411	0,081292647	0,397486135	T1D vs Control
hsa-miR-7-5p_TGGAAGACTAGTGATTTTGTTG4	-0,815110514	3,890826404	0,081966456	0,398336983	T1D vs Control
hsa-miR-21-5p_TAGCTTATCAGACTGATGTTG4	0,191458168	11,59436943	0,084455459	0,405455677	T1D vs Control
hsa-miR-30d-5p_GTAAACATCCCCGACTGGAAG4	0,552173294	5,727486807	0,084569654	0,405455677	T1D vs Control
hsa-miR-23a-3p_ATCACATTGCCAGGGATTTC4	0,242090128	9,459376505	0,084985425	0,405455677	T1D vs Control
hsa-miR-150-5p_CTCCCAACCCTTGTACCAGT4	-0,944997021	3,181356832	0,086160447	0,405455677	T1D vs Control
hsa-let-7f-5p_TGAGGTAGTAGATTGTATAGTTG4	-0,985285385	2,64125494	0,086310012	0,405455677	T1D vs Control
hsa-miR-92b-3p_TATTGCACTCGTCCCGGCCTCC4	-0,784844112	4,414239688	0,086483645	0,405455677	T1D vs Control
hsa-miR-10b-5p_TACCCTGTAGAACCGAATTT4	-0,650576166	5,2763128	0,087476729	0,407093786	T1D vs Control
hsa-miR-484_TCAGGCTCAGTCCCCTCCCGATA4	-0,73318382	4,637634197	0,087854619	0,407093786	T1D vs Control
hsa-miR-409-3p_GAATGTTGCTCGGTGAACCCCT4	-0,590047111	7,973406619	0,088557014	0,407976532	T1D vs Control
hsa-let-7d-3p_TATACGACCTGCTGCCTTTCT4	-0,213469999	8,488338468	0,090229444	0,409384758	T1D vs Control
hsa-let-7f-5p_TGAGGTAGTAGATTGTATA4	-0,882876331	3,61271535	0,090310783	0,409384758	T1D vs Control
hsa-let-7f-5p_GAGGTAGTAGATTGTATAGTT4	-0,584152113	6,051011881	0,09040366	0,409384758	T1D vs Control
hsa-miR-182-5p_TTTGGCAATGGTAGAACTC4	-0,956242483	2,382812326	0,092282668	0,415532692	T1D vs Control
hsa-let-7g-5p_TGAGGTAGTAGTTTGTACAGTTT4	-0,431055708	6,811439564	0,0928879	0,415908183	T1D vs Control
hsa-miR-29a-3p_TAGCACCATCTGAAATCGGTTAT4	-0,920015357	2,486065735	0,09480033	0,422099791	T1D vs Control
hsa-miR-99a-5p_AACCCGTAGATCCGATCTTGTG4	0,59605366	5,156061862	0,095871624	0,424498244	T1D vs Control
hsa-miR-185-5p_TGGAGAGAAAGGCAGTTCCTG4	-0,801113173	4,508281621	0,096661694	0,424804971	T1D vs Control
hsa-miR-223-3p_TGTCAGTTTGTCAAATACCCC4	-0,463859611	7,314719746	0,097006907	0,424804971	T1D vs Control
hsa-miR-106b-5p_TAAAGTGCTGACAGTGCAGAT4	-0,849306343	2,655083548	0,098712077	0,428608779	T1D vs Control
hsa-miR-4433b-5p_TATGTCCCACCCCCACTCCTGT4	-0,785391166	5,397905397	0,098951086	0,428608779	T1D vs Control
hsa-miR-30e-5p_TGTAAACATCCTTGACTGGAAGC4	-0,191818371	8,385302075	0,101348733	0,429938281	T1D vs Control
hsa-miR-374a-5p_TTATAATACAACCTGATAAGT4	-0,905127147	2,487762486	0,101373942	0,429938281	T1D vs Control
hsa-miR-27b-3p_TTCACAGTGGCTAAGTTCT4	0,291231266	8,299608545	0,102124063	0,429938281	T1D vs Control
hsa-miR-10a-5p_TACCCTGTAGATCCGAATTTGTGT4	-0,884858687	2,404757373	0,10253686	0,429938281	T1D vs Control
hsa-miR-103a-3p_AGCAGCATTGTACAGGGCTAT4	-1,092840472	3,428123511	0,102670178	0,429938281	T1D vs Control
hsa-miR-10b-5p_ACCCTGTAGAACCGAATTTGTG4	0,504536662	5,829978228	0,102780557	0,429938281	T1D vs Control
hsa-let-7e-5p_TGAGGTAGGAGGTTGTATAGTT4	-0,334920715	7,813417368	0,103034143	0,429938281	T1D vs Control
hsa-miR-125b-5p_CCCTGAGACCCTAACTTGT4	-0,887681891	2,290772166	0,105139621	0,436438948	T1D vs Control
hsa-miR-183-5p_ATGGCACTGGTAGAATTCACT4	-0,751933934	4,652978697	0,105976773	0,436702883	T1D vs Control
hsa-let-7d-5p_AGAGGTAGTAGGTTGCATAGTT4	-0,316607904	9,075101282	0,106299071	0,436702883	T1D vs Control
hsa-let-7a-5p_GAGGTAGTAGGTTGTATAGT4	-0,743222101	4,701370889	0,108137224	0,439412417	T1D vs Control
hsa-miR-339-3p_TGAGCGCCTCGACGACAGAGC4	0,794751914	3,99309945	0,108383361	0,439412417	T1D vs Control
hsa-miR-4433b-5p_TGTCCCACCCCCACTCCTG4	0,964990061	2,497163245	0,108666546	0,439412417	T1D vs Control
hsa-miR-92a-3p_GTATTGCACTTGTCCCGGCCTG4	-0,867838246	3,065032086	0,109789646	0,439412417	T1D vs Control
hsa-miR-10b-5p_TACCCTGTAGAACCGAAT4	-0,893491731	2,220184724	0,110066226	0,439412417	T1D vs Control
hsa-miR-143-3p_TGAGATGAAGCACTGTAGCTCA4	-0,458504474	6,738882834	0,110266604	0,439412417	T1D vs Control
hsa-miR-15b-3p_CGAATCATTATTTGCTGCTCT4	0,698514521	4,350054835	0,11082656	0,439446609	T1D vs Control
hsa-miR-423-5p_GAGGGGCAGAGAGCGAGA4	-0,876457939	1,916584014	0,112308375	0,443117697	T1D vs Control
hsa-miR-21-5p_AGCTTATCAGACTGATGTTGA4	-0,337526519	6,721668578	0,113592014	0,445974558	T1D vs Control
hsa-let-7a-5p_GAGGTAGTAGGTTGTATAG4	-0,614188446	5,403501302	0,114803279	0,446549161	T1D vs Control
hsa-miR-409-3p_CGAATGTTGCTCGGTGAACCCCT4	-0,784287471	5,738161577	0,114858944	0,446549161	T1D vs Control
hsa-let-7b-5p_GAGGTAGTAGGTTGTGTGGTTT4	-0,816155971	3,859782449	0,117030763	0,45278407	T1D vs Control
hsa-let-7i-5p_TGAGGTAGTAGTTTGTGCTGT4	0,172567613	11,39282357	0,117752548	0,453375753	T1D vs Control
hsa-miR-26b-5p_TCAAGTAATTCAGGATAGGTT4	-0,833529462	3,747955651	0,11832298	0,453381803	T1D vs Control
hsa-miR-1-3p_TGGAATGTAAAGAAGTATGTAT4	-0,778808101	5,995923161	0,119847994	0,455241557	T1D vs Control
hsa-miR-128-3p_TCACAGTGAACCGGTCTCTTT4	-0,20318712	8,228392837	0,120078083	0,455241557	T1D vs Control
hsa-miR-148a-3p_TCAGTGCACTACAGAACTTTGT4	-0,21106462	9,289411705	0,120521918	0,455241557	T1D vs Control
hsa-miR-106b-3p_CCGCACTGTGGGTACTTGCTG4	-0,686706894	4,162035136	0,121578048	0,457064642	T1D vs Control
hsa-miR-224-5p_CAAGTCACTAGTGGTTCCGTTTAG4	-0,818482407	4,65780231	0,123725322	0,462953436	T1D vs Control
hsa-miR-103a-3p_AGCAGCATTGTACAGGGCTATG4	-0,808773236	4,276844631	0,124898372	0,463095294	T1D vs Control
hsa-miR-106b-3p_CCGCACTGTGGGTACTTGCT4	-0,480216058	6,396206525	0,12540162	0,463095294	T1D vs Control

hsa-miR-328-3p_CTGGCCCTCTCTGCCCTTCCGT4	-0,328167163	7,228551867	0,125506378	0,463095294	T1D vs Control
hsa-miR-320a-3p_AAAGCTGGGTTGAGAGGGCGA4	0,72059721	4,98279454	0,127182839	0,464259563	T1D vs Control
hsa-miR-664a-5p_ACTGGCTAGGGAAAATGATTGG4	-0,778108291	3,587998944	0,127864891	0,464259563	T1D vs Control
hsa-miR-24-3p_TGGCTCAGTTCAGCAGGAACAG4	0,160838154	12,76835022	0,128146185	0,464259563	T1D vs Control
hsa-miR-21-5p_AGCTTATCAGACTGATGTTG4	0,561675341	5,031654467	0,12815195	0,464259563	T1D vs Control
hsa-miR-29c-3p_TAGCACCATTTGAAATCGG4	-0,813314412	3,272314395	0,129867716	0,467444587	T1D vs Control
hsa-miR-192-5p_TGACCTATGAATTGACAGC4	0,858055802	3,665629227	0,130362289	0,467444587	T1D vs Control
hsa-miR-222-3p_AGCTACATCTGGCTACTGGGT4	0,291493093	7,595221992	0,130790644	0,467444587	T1D vs Control
hsa-miR-501-3p_AATGCACCCGGGCAAGGATTC4	-0,806594392	3,102124992	0,131777558	0,468869259	T1D vs Control
hsa-miR-451a_AACCGTTACCATTACTGAGTT4	-0,445037331	6,297926904	0,135523721	0,478981548	T1D vs Control
hsa-miR-4433b-5p_ATGTCCCACCCCCACTCCTGT4	-0,531398183	7,752662063	0,136189153	0,478981548	T1D vs Control
hsa-miR-339-3p_TGAGCGCCTCGACGACAGAGCCG4	-0,80566762	4,16247252	0,136425886	0,478981548	T1D vs Control
hsa-miR-142-5p_CCCATAAAGTAGAAAGCACTAC4	-0,348913934	7,021131164	0,137023579	0,478981548	T1D vs Control
hsa-miR-320a-3p_AAAAGCTGGGTTGAGAGGGCGAAAA4	-0,812506941	3,186668725	0,13843225	0,48179259	T1D vs Control
hsa-let-7b-5p_GAGGTAGTAGGTTGTGTGGT4	-0,632391845	4,944887656	0,139084558	0,481958228	T1D vs Control
hsa-miR-451a_CGTTACCATTACTGAGT4	0,701960003	4,012413349	0,139891348	0,482655431	T1D vs Control
hsa-miR-451a_AAACCGTTACCATTACTG4	-0,74358653	2,491678877	0,142622092	0,489956066	T1D vs Control
hsa-miR-21-5p_TAGCTTATCAGACTGATGT4	-0,673920966	4,699515355	0,144314443	0,491771811	T1D vs Control
hsa-miR-103a-3p_AGCAGCATTGTACAGGGCTATGA4	-0,306888516	8,631709381	0,144384697	0,491771811	T1D vs Control
hsa-miR-128-3p_TCACAGTGAACCGGTCTCTT4	-0,311349678	6,808413156	0,145087062	0,492061227	T1D vs Control
hsa-miR-486-5p_CTGTACTGAGCTGCCCCG4	-0,782576783	3,40982355	0,14820052	0,500490738	T1D vs Control
hsa-miR-3613-5p_TGTTGTACTTTTTTTTTTGTT4	-0,551408142	5,269511804	0,150376978	0,504222825	T1D vs Control
hsa-miR-30c-5p_TGTAAACATCCTACACTCTC4	0,787315645	2,069916362	0,154012345	0,504222825	T1D vs Control
hsa-miR-23b-3p_ATCACATTGCCAGGGATTAC4	-0,742785376	2,694770844	0,154620601	0,504222825	T1D vs Control
hsa-miR-223-5p_CGTGTATTTGACAAGCTGAGTTG4	-0,474059	6,07891061	0,155558377	0,504222825	T1D vs Control
hsa-miR-193a-5p_TGGGTCTTTGCGGGCGAGAT4	-0,618090958	4,669337288	0,157759418	0,504222825	T1D vs Control
hsa-miR-4433b-5p_TGTCCCACCCCCACTCCTGTT4	-0,615715174	6,729375179	0,158039835	0,504222825	T1D vs Control
hsa-miR-126-3p_CTCGTACCGTGAGTAATAATGCG4	-0,660612962	4,682348296	0,158226166	0,504222825	T1D vs Control
hsa-miR-340-5p_TTATAAAGCAATGAGACTGATT4	-0,497474828	5,815012818	0,158727532	0,504222825	T1D vs Control
hsa-let-7b-5p_TGAGGTAGTAGGTTGTGTGGTTT4	-0,168570107	11,28589984	0,158900819	0,504222825	T1D vs Control
hsa-miR-10b-5p_ACCCTGTAGAACCGAATTTGTGT4	-0,72080807	3,356457419	0,159109521	0,504222825	T1D vs Control
hsa-miR-146a-5p_TGAGAACTGAATTCCATGGGTTG4	-0,282249383	7,878355546	0,15913328	0,504222825	T1D vs Control
hsa-miR-197-3p_TTCACCACCTTCTCCACCCAGC4	-0,215804353	10,1606215	0,159180799	0,504222825	T1D vs Control
hsa-miR-2110_TTGGGGAAACGGCCGCTGAG4	-0,76868359	2,165591375	0,159878279	0,504222825	T1D vs Control
hsa-miR-421_ATCAACAGACATTAATTGGGCG4	-0,701477685	2,09434672	0,16130982	0,504222825	T1D vs Control
hsa-miR-484_TCAGGCTCAGTCCCCTCCCGAT4	-0,183276965	9,18414004	0,161700674	0,504222825	T1D vs Control
hsa-miR-21-5p_TAGCTTATCAGACTGATGTTGACT4	-0,619945973	5,943834972	0,162058227	0,504222825	T1D vs Control
hsa-miR-1306-5p_CCACCTCCCCTGCAAACGTCCA4	-0,804574831	3,283279778	0,162326803	0,504222825	T1D vs Control
hsa-miR-342-3p_TCTCACACAGAAATCGCACCCG4	0,28180013	9,658047597	0,163256567	0,504222825	T1D vs Control
hsa-miR-1306-5p_CCACCTCCCCTGCAAACGTCC4	-0,636699901	4,76371819	0,163275517	0,504222825	T1D vs Control
hsa-miR-486-5p_TGTACTGAGCTGCCCCGA4	-0,675640855	4,617811312	0,164199954	0,504222825	T1D vs Control
hsa-miR-181b-5p_AACATTCATTGCTGTCGGTG4	-0,760191146	2,058689202	0,164727549	0,504222825	T1D vs Control
hsa-miR-92a-3p_ACTTGTCCCGGCCTGT4	-0,772085687	2,134254049	0,165046938	0,504222825	T1D vs Control
hsa-miR-93-5p_CAAAGTGCTGTTCGTGCAGGTAG4	-0,159707636	9,991178453	0,165270973	0,504222825	T1D vs Control
hsa-miR-29a-3p_CTAGCACCATCTGAAATCGG4	-0,713590469	3,050349736	0,1655765	0,504222825	T1D vs Control
hsa-miR-139-5p_TCTACAGTGCACGTGTCTCCAG4	0,607928986	4,676823231	0,165606807	0,504222825	T1D vs Control
hsa-miR-6803-3p_TCCCTCGCCTTCTCACCCTCAGT4	-0,8157057	2,14031641	0,1661835	0,504222825	T1D vs Control
hsa-miR-146a-5p_TGAGAACTGAATTCCATGGG4	-0,723708148	2,424854764	0,166766744	0,504222825	T1D vs Control
hsa-miR-125a-5p_TCCCTGAGACCCTTTAACCTG4	0,243486376	8,290201937	0,167019857	0,504222825	T1D vs Control
hsa-miR-223-3p_TGTCAGTTTGTCAAATACCCCA4	-0,24051287	10,41019198	0,167891311	0,504340608	T1D vs Control
hsa-miR-191-5p_CAACGGAATCCCAAAAGCA4	-0,387124028	6,203677568	0,168324469	0,504340608	T1D vs Control
hsa-miR-532-5p_CATGCCTTGAGTGTAGGACCG4	-0,71503108	2,388684445	0,1700522	0,505427858	T1D vs Control
hsa-miR-30d-5p_TGTAAACATCCCCGACTGG4	0,182749873	8,045778015	0,170413322	0,505427858	T1D vs Control
hsa-miR-361-5p_TTATCAGAATCTCCAGGGG4	0,734524533	2,056970291	0,170589829	0,505427858	T1D vs Control
hsa-miR-487b-3p_AATCGTACAGGGTCATCCACTT4	-0,790232179	4,048228768	0,172779078	0,510018242	T1D vs Control
hsa-let-7b-5p_GAGGTAGTAGGTTGTGTGG4	0,428513543	6,125260792	0,175447235	0,512636875	T1D vs Control
hsa-miR-409-3p_GAATGTTGCTCGGTGAACCCCTT4	-0,740776754	5,101558557	0,175728523	0,512636875	T1D vs Control
hsa-miR-223-3p_GTCAGTTTGTCAAATACCCCAA4	-0,354367005	7,740128632	0,17702194	0,512636875	T1D vs Control
hsa-miR-148a-3p_TCAGTGCACTACAGAACTTT4	-0,718174642	4,280926656	0,177276081	0,512636875	T1D vs Control
hsa-miR-101-3p_TACAGTACTGTGATAACTGAAG4	-0,301374806	7,012156412	0,177338551	0,512636875	T1D vs Control
hsa-let-7f-5p_TGAGGTAGTAGATTGTATAG4	-0,155970091	10,59314063	0,177525442	0,512636875	T1D vs Control

hsa-miR-146b-5p_TGAGAACTGAATTCCATAGGCTGT4	-0,223981613	8,368012324	0,179134511	0,515415905	T1D vs Control
hsa-miR-151a-3p_TACTAGACTGAAGCTCCTTGA4	-0,704682549	1,940948476	0,18001365	0,516082299	T1D vs Control
hsa-miR-342-3p_TCTCACACAGAAATCGCACCCGTC4	-0,255698146	7,583667414	0,185499794	0,52990443	T1D vs Control
hsa-miR-4433b-5p_TATGTCCCACCCCCACTCCTG4	-0,827852364	3,068037559	0,186352988	0,530440468	T1D vs Control
hsa-miR-942-5p_TCTTCTCTGTTTTGGCCATGT4	-0,723030118	2,592733465	0,191831035	0,543954861	T1D vs Control
hsa-miR-22-3p_AAGCTGCCAGTTGAAGAACT4	0,153675468	10,21399499	0,192465835	0,543954861	T1D vs Control
hsa-miR-423-3p_AAGCTCGGTCTGAGGCCCCTCAG4	-0,654459795	3,402313099	0,197513363	0,55624788	T1D vs Control
hsa-miR-320a-3p_AAAGCTGGGTTGAGAGGGCG4	-0,541187473	4,890613401	0,198245446	0,556343733	T1D vs Control
hsa-miR-335-5p_TCAAGAGCAATAACGAAAAATG4	-0,227903078	8,412522999	0,199534828	0,557850833	T1D vs Control
hsa-miR-181a-5p_ACATTCAACGCTGTCGGTGA4	0,655521149	3,569893472	0,200862159	0,557850833	T1D vs Control
hsa-miR-451a_CCGTTACCATTACTGAGT4	0,612278121	4,189894806	0,201503975	0,557850833	T1D vs Control
hsa-miR-30c-5p_TGTAAACATCCTACACTCTCAGCT4	-0,186167607	10,20131272	0,202143892	0,557850833	T1D vs Control
hsa-miR-16-2-3p_ACCAATATTACTGTGCTGCT4	-0,563504774	4,606374691	0,202282172	0,557850833	T1D vs Control
hsa-miR-432-5p_TCTTGGAGTAGGTCATTGGGTGG4	-0,776760664	3,502755168	0,203380266	0,558945076	T1D vs Control
hsa-miR-342-3p_TCTCACACAGAAATCGCACCC4	-0,691296121	2,049205595	0,206785341	0,563987637	T1D vs Control
hsa-miR-3613-5p_TGTTGTACTTTTTTTTTTGTTC4	-0,546224474	4,388236924	0,206901081	0,563987637	T1D vs Control
hsa-miR-101-3p_GTACAGTACTGTGATAACTGA4	-0,188405612	8,248925167	0,207337989	0,563987637	T1D vs Control
hsa-miR-181b-5p_AACATTCATTGCTGTCGGTGG4	-0,453603372	5,135854331	0,208933166	0,566050782	T1D vs Control
hsa-miR-145-5p_GTCCAGTTTTCCCAGGAATCC4	-0,677566026	2,283533911	0,21031807	0,566050782	T1D vs Control
hsa-miR-26a-5p_TTCAAGTAATCCAGGATAGGCT4	-0,204433283	11,92893731	0,210579505	0,566050782	T1D vs Control
hsa-miR-423-5p_AGGGGCAGAGAGCGAGACT4	0,698648192	3,04923014	0,21187263	0,566050782	T1D vs Control
hsa-miR-191-5p_CAACGGAATCCCAAAAGCAGCTG4	-0,218584897	9,658129048	0,211966449	0,566050782	T1D vs Control
hsa-miR-223-3p_GTCAGTTTGTCAAATACCCCA4	-0,371421687	6,454169749	0,212357821	0,566050782	T1D vs Control
hsa-miR-25-3p_CATTGCACTTGTCTCGGTCT4	0,178801762	10,26006574	0,213616346	0,567507426	T1D vs Control
hsa-miR-150-5p_TCTCCCAACCCTTGTACCA4	-0,406250097	6,588601483	0,217328604	0,57482888	T1D vs Control
hsa-miR-145-3p_ATTCCTGGAAATACTGTTCT4	-0,664083494	3,209907444	0,217814707	0,57482888	T1D vs Control
hsa-let-7b-3p_CTATACAACCTACTGCCTTC4	-0,644670947	3,159200497	0,21887178	0,575712239	T1D vs Control
hsa-miR-2110_TTGGGGAAACGGCCGCTGAGT4	-0,392243243	5,974874685	0,221902158	0,581584259	T1D vs Control
hsa-let-7a-5p_TGAGGTAGTAGGTTGTATAGTTTT4	-0,701875233	3,044731157	0,223104827	0,581584259	T1D vs Control
hsa-miR-29c-3p_TAGCACCATTTGAAATCGGT4	0,621901234	4,339459305	0,223293329	0,581584259	T1D vs Control
hsa-miR-186-5p_CAAAGAATTCTCCTTTTGGGCTTT4	-0,622538524	4,003798957	0,22460183	0,583086837	T1D vs Control
hsa-miR-486-5p_ATCCTGTACTGAGCTGCCCCGA4	0,203290716	8,988899635	0,227617379	0,588996918	T1D vs Control
hsa-miR-423-5p_TGAGGGGCAGAGAGCGAGAC4	0,118873515	9,751921415	0,229416087	0,590863187	T1D vs Control
hsa-miR-100-5p_AACCCGTAGATCCGAACTTGTG4	-0,686107002	3,590895666	0,230551301	0,590863187	T1D vs Control
hsa-miR-6803-3p_TCCCTCGCCTTCTCACCCTCAG4	-0,63269228	2,710817974	0,231374855	0,590863187	T1D vs Control
hsa-miR-423-5p_CTGAGGGGCAGAGAGCGAGACTTT4	-0,584094035	4,419437576	0,232726237	0,590863187	T1D vs Control
hsa-miR-486-5p_CCTGTACTGAGCTGCCCCGAG4	-0,176374299	11,29662351	0,232832341	0,590863187	T1D vs Control
hsa-miR-125a-5p_TCCCTGAGACCCTTTAACCTGT4	-0,139473636	11,48945871	0,233418489	0,590863187	T1D vs Control
hsa-miR-629-5p_TGGGTTTACGTTGGGAGAACT4	0,273449869	7,034835335	0,23352811	0,590863187	T1D vs Control
hsa-miR-532-5p_CATGCCTTGAGTGTAGGACCGT4	-0,525385191	4,730944055	0,235077977	0,591831308	T1D vs Control
hsa-miR-574-3p_CACGCTCATGCACACACCCACA4	-0,335495314	6,300877473	0,23539589	0,591831308	T1D vs Control
hsa-miR-10b-5p_ACCCTGTAGAACCGAATTTGT4	-0,250887591	6,785991828	0,239325683	0,596391176	T1D vs Control
hsa-let-7a-5p_TTGAGGTAGTAGGTTGTATAGTT4	-0,641032579	1,956823269	0,240203355	0,596391176	T1D vs Control
hsa-miR-100-5p_AACCCGTAGATCCGAACTTG4	-0,351167151	5,941970306	0,241301265	0,596391176	T1D vs Control
hsa-miR-30d-5p_TGTAAACATCCCCGACTGGAA4	-0,111880225	11,66083446	0,242687697	0,596391176	T1D vs Control
hsa-miR-1294_TGTGAGGTTGGCATTGTTGT4	-0,639138494	2,481086687	0,242861955	0,596391176	T1D vs Control
hsa-miR-140-3p_TACCACAGGGTAGAACCACGGAC4	0,615618336	3,947173881	0,245559971	0,596391176	T1D vs Control
hsa-miR-4685-3p_TCTCCCTTCCTGCCCTGGCT4	-0,512866718	4,862788515	0,245929875	0,596391176	T1D vs Control
hsa-miR-494-3p_TGAAACATACACGGGAAACCTCT4	-0,620475876	4,269868739	0,246470279	0,596391176	T1D vs Control
hsa-miR-451a_AAACCGTTACCATTACTGA4	-0,201424254	8,929457213	0,246921771	0,596391176	T1D vs Control
hsa-miR-103a-3p_AGCAGCATTGTACAGGGC4	0,76342999	3,081760646	0,248365129	0,596391176	T1D vs Control
hsa-miR-148a-3p_TCAGTGCACTACAGAACTT4	0,677545846	2,124123364	0,248562089	0,596391176	T1D vs Control
hsa-miR-7-5p_TGGAAGACTAGTGATTTTGT4	0,613053764	3,91952121	0,248825151	0,596391176	T1D vs Control
hsa-miR-145-3p_ATTCCTGGAAATACTGTTCTT4	0,626075162	3,50539909	0,24946927	0,596391176	T1D vs Control
hsa-miR-10b-5p_TACCCTGTAGAACCGAATTTG4	-0,14107989	9,833049317	0,249683553	0,596391176	T1D vs Control
hsa-miR-142-5p_CCATAAAGTAGAAAGCACT4	-0,416289917	4,774985235	0,250008339	0,596391176	T1D vs Control
hsa-let-7f-5p_TGAGGTAGTAGATTGTATAGTT4	-0,182635845	12,51171334	0,250149751	0,596391176	T1D vs Control
hsa-miR-23a-3p_ATCACATTGCCAGGGATTT4	0,194742572	7,810237088	0,250355263	0,596391176	T1D vs Control
hsa-miR-126-3p_GTACCGTGAGTAATAATGCG4	-0,620273948	2,067690175	0,25067885	0,596391176	T1D vs Control
hsa-miR-17-5p_CAAAGTGCTTACAGTGCAGGTAG4	-0,622470128	3,276961308	0,252368952	0,598625162	T1D vs Control
hsa-miR-335-3p_TTTTTCATTATTGCTCCTGACC4	-0,611569325	4,076732498	0,255581907	0,604447417	T1D vs Control

hsa-miR-23a-3p_ATCACATTGCCAGGGATTTCC4	0,115821762	11,08225248	0,256757706	0,605431633	T1D vs Control
hsa-miR-3615_TCTCTCGGCTCCTCGCGGCTC4	0,566220445	2,193732344	0,260197118	0,611731867	T1D vs Control
hsa-let-7e-5p_TGAGGTAGGAGGTTGTATAGT4	-0,512395258	4,645402812	0,261396343	0,612611268	T1D vs Control
hsa-miR-197-3p_TTCACCACCTTCTCCACCCA4	-0,553325226	3,958344822	0,262865952	0,612611268	T1D vs Control
hsa-let-7a-5p_TGAGGTAGTAGGTTGTATAGTT4	-0,151766085	13,39549393	0,262877106	0,612611268	T1D vs Control
hsa-miR-320a-3p_GAAAAGCTGGGTTGAGAGGGCGA4	0,578502603	3,456353792	0,26412508	0,61372504	T1D vs Control
hsa-miR-451a_AAACCGTTACCATTACTGAGTT4	-0,162481497	12,14012514	0,265665423	0,614157127	T1D vs Control
hsa-let-7c-5p_TGAGGTAGTAGGTTGTATGGTT4	0,212933971	7,467576739	0,266159912	0,614157127	T1D vs Control
hsa-miR-654-5p_TGGTGGGCCGCAGAACATGTGC4	0,696615651	3,027916369	0,266622793	0,614157127	T1D vs Control
hsa-miR-144-5p_GGATATCATCATATACTGTAAGT4	0,305495916	6,405140764	0,268671198	0,61709206	T1D vs Control
hsa-let-7i-5p_TGAGGTAGTAGTTTGTGCTGTT4	-0,168645282	10,48466024	0,270046417	0,617110736	T1D vs Control
hsa-miR-92a-3p_GCACTTGTCCCGGCCTGT4	-0,457574758	4,476222162	0,270417591	0,617110736	T1D vs Control
hsa-miR-361-3p_TCCCCCAGGTGTGATTCTGATT4	-0,518092848	3,650889743	0,271002205	0,617110736	T1D vs Control
hsa-miR-101-3p_TACAGTACTGTGATAACTGA4	-0,545821943	4,333316093	0,273392948	0,618761005	T1D vs Control
hsa-miR-15b-5p_TAGCAGCACATCATGGTTTACA4	-0,324463413	6,32576652	0,275247796	0,618761005	T1D vs Control
hsa-miR-125a-5p_TCCCTGAGACCCTTTAACCTGTG4	-0,165521377	8,600810288	0,275896259	0,618761005	T1D vs Control
hsa-miR-191-5p_CAACGGAATCCCAAAAGCAGC4	-0,203401821	8,150484012	0,276810005	0,618761005	T1D vs Control
hsa-miR-542-3p_TGTGACAGATTGATAACTGA4	-0,594793784	1,954666629	0,277012929	0,618761005	T1D vs Control
hsa-miR-18a-3p_ACTGCCCTAAGTGCTCCTTCTG4	-0,572583561	2,22678804	0,277096512	0,618761005	T1D vs Control
hsa-miR-423-5p_TGAGGGGCAGAGAGCGAGACTTT4	0,074769227	14,42674435	0,27771316	0,618761005	T1D vs Control
hsa-miR-139-5p_TCTACAGTGCACGTGTCTCCA4	-0,50384893	4,69390328	0,27880632	0,618761005	T1D vs Control
hsa-miR-423-3p_AGCTCGGTCTGAGGCCCCTCAG4	0,130179234	8,372128456	0,278966798	0,618761005	T1D vs Control
hsa-miR-30e-5p_GTAAACATCCTTGACTGGAAGC4	-0,60202063	2,49990334	0,279645562	0,618761005	T1D vs Control
hsa-miR-363-3p_ATTGCACGGTATCCATCTGT4	-0,508222481	4,343538892	0,281219425	0,618761005	T1D vs Control
hsa-miR-93-5p_CAAAGTGCTGTTCGTGCAGGTAGT4	-0,553373453	4,129822813	0,285195342	0,618761005	T1D vs Control
hsa-miR-486-5p_GTACTGAGCTGCCCCGAG4	0,481774698	4,883397866	0,285248442	0,618761005	T1D vs Control
hsa-miR-629-5p_TGGGTTTACGTTGGGAGAAC4	0,57531827	3,68380122	0,285258411	0,618761005	T1D vs Control
hsa-miR-142-5p_CATAAAGTAGAAAGCACTAC4	-0,586594136	2,376484394	0,285430792	0,618761005	T1D vs Control
hsa-miR-363-3p_AATTGCACGGTATCCATCTGT4	0,155523114	8,977144446	0,285790703	0,618761005	T1D vs Control
hsa-miR-106b-5p_TAAAGTGCTGACAGTGCAGA4	-0,413987132	5,144709005	0,286067448	0,618761005	T1D vs Control
hsa-miR-22-3p_AAGCTGCCAGTTGAAGAACTG4	0,182195811	9,134304454	0,286207545	0,618761005	T1D vs Control
hsa-miR-423-5p_TGAGGGGCAGAGAGCGAGACTTTT4	-0,112994036	11,39034963	0,286477806	0,618761005	T1D vs Control
hsa-miR-191-5p_CAACGGAATCCCAAAAGCAGCTGT4	-0,458103667	5,688164696	0,287753137	0,619218744	T1D vs Control
hsa-miR-23a-3p_ATCACATTGCCAGGGATTTCCAA4	-0,339736282	6,530990344	0,288243606	0,619218744	T1D vs Control
hsa-miR-328-3p_CTGGCCCTCTCTGCCCTTCCG4	0,499965364	3,770890003	0,292559552	0,626800975	T1D vs Control
hsa-miR-181a-5p_AACATTCAACGCTGTCGGTGA4	0,107009122	9,980124889	0,296473051	0,632045366	T1D vs Control
hsa-miR-484_CAGGCTCAGTCCCCTCCCGAT4	-0,534364487	2,404164158	0,296782837	0,632045366	T1D vs Control
hsa-miR-140-3p_TACCACAGGGTAGAACCACGGACA4	-0,508975456	3,637619462	0,297386464	0,632045366	T1D vs Control
hsa-miR-652-3p_AATGGCGCCACTAGGGTTGTGC4	-0,519342461	3,594273542	0,299449747	0,634737895	T1D vs Control
hsa-miR-191-5p_CAACGGAATCCCAAAAGC4	-0,555034608	3,190312182	0,302976234	0,639901432	T1D vs Control
hsa-miR-30e-3p_CTTTCAGTCGGATGTTTACAG4	0,558791397	2,31966353	0,30349152	0,639901432	T1D vs Control
hsa-miR-21-3p_CAACACCAGTCGATGGGCTGT4	-0,523295417	2,296740898	0,30478269	0,640049573	T1D vs Control
hsa-miR-1306-5p_CCACCTCCCCTGCAAACGTC4	-0,454349892	4,129732962	0,305167927	0,640049573	T1D vs Control
hsa-miR-501-3p_AATGCACCCGGGCAAGGATTCT4	-0,145719079	7,911300847	0,307458808	0,64017737	T1D vs Control
hsa-miR-16-5p_AGCAGCACGTAAATATTGGC4	0,535966624	2,995434135	0,307611643	0,64017737	T1D vs Control
hsa-miR-30e-5p_TGTAAACATCCTTGACTGG4	0,446483802	5,076073317	0,307697369	0,64017737	T1D vs Control
hsa-miR-335-5p_TCAAGAGCAATAACGAAAAAT4	-0,303790932	6,715048961	0,308441794	0,64017737	T1D vs Control
hsa-let-7b-5p_GAGGTAGTAGGTTGTGTGGTT4	0,28785544	6,209954592	0,310925427	0,643656015	T1D vs Control
hsa-miR-10b-5p_TACCCTGTAGAACCGAATTTGT4	-0,116853331	11,12588043	0,313139871	0,645409086	T1D vs Control
hsa-miR-16-2-3p_ACCAATATTACTGTGCTGCTT4	0,159032901	8,312552057	0,31378664	0,645409086	T1D vs Control
hsa-miR-128-3p_TCACAGTGAACCGGTCTCT4	0,179082065	7,683472613	0,314201663	0,645409086	T1D vs Control
hsa-miR-501-3p_AATGCACCCGGGCAAGGATT4	0,271023728	6,44960483	0,315239372	0,64587604	T1D vs Control
hsa-miR-16-5p_TAGCAGCACGTAAATATTGGC4	0,120294819	9,94541903	0,316170619	0,646123035	T1D vs Control
hsa-miR-99b-5p_CACCCGTAGAACCGACCTTG4	-0,236086884	6,765454723	0,319621648	0,651504996	T1D vs Control
hsa-miR-93-5p_AAAGTGCTGTTCGTGCAGGTAG4	-0,4962659	3,611226251	0,32364546	0,658024059	T1D vs Control
hsa-miR-150-5p_GTCTCCCAACCCTTGTACCAGT4	-0,527914774	2,340151294	0,324846322	0,658785034	T1D vs Control
hsa-miR-15b-5p_TAGCAGCACATCATGGTT4	0,239966792	7,417344831	0,328867352	0,665246902	T1D vs Control
hsa-miR-485-5p_AGAGGCTGGCCGTGATGAATTC4	-0,583450605	2,300221452	0,329731749	0,665306845	T1D vs Control
hsa-miR-21-5p_TAGCTTATCAGACTGATGTTGA4	-0,077584231	12,88530966	0,334052834	0,672323507	T1D vs Control
hsa-miR-10b-5p_ACCCTGTAGAACCGAATTTG4	-0,413889228	4,835525593	0,335294406	0,673122523	T1D vs Control
hsa-miR-20a-5p_TAAAGTGCTTATAGTGCAGGTAG4	-0,43139906	4,004629847	0,336145123	0,673134832	T1D vs Control

hsa-miR-132-3p_TAACAGTCTACAGCCATGGTCG4	0,519547877	2,233528136	0,337412796	0,673979947	T1D vs Control
hsa-miR-16-5p_TTAGCAGCACGTAAATATTGGCG4	-0,459444178	4,199452303	0,339878336	0,677207585	T1D vs Control
hsa-miR-223-5p_CGTGTATTTGACAAGCTGAGTT4	-0,447593657	3,93706767	0,34114623	0,677254449	T1D vs Control
hsa-miR-342-3p_TCACACAGAAATCGCACCCGTCA4	-0,49797016	3,930198822	0,341601366	0,677254449	T1D vs Control
hsa-miR-28-3p_ACTAGATTGTGAGCTCCTGGAG4	-0,424796446	4,615150746	0,345973634	0,682567497	T1D vs Control
hsa-let-7b-5p_TGAGGTAGTAGGTTGTGTGG4	0,098233186	12,43476497	0,346727143	0,682567497	T1D vs Control
hsa-miR-24-3p_GGCTCAGTTCAGCAGGAACA4	0,515322624	2,640585933	0,346850485	0,682567497	T1D vs Control
hsa-miR-10a-5p_TACCCTGTAGATCCGAATTT4	-0,454592836	4,308439737	0,347711079	0,68257569	T1D vs Control
hsa-miR-140-3p_ACCACAGGGTAGAACCACGG4	-0,471007459	3,491351538	0,351698506	0,688706902	T1D vs Control
hsa-miR-484_CAGGCTCAGTCCCCTCCCGA4	-0,508651187	3,768443499	0,354895374	0,691906841	T1D vs Control
hsa-miR-125a-5p_CCCTGAGACCCTTTAACCTGT4	-0,365676522	5,351640533	0,3553795	0,691906841	T1D vs Control
hsa-miR-92a-3p_CACTTGTCCCGGCCTGT4	0,463597289	4,072780053	0,35593702	0,691906841	T1D vs Control
hsa-miR-16-2-3p_ACCAATATTACTGTGCTGCTTT4	-0,139864147	7,879139867	0,36048806	0,699048623	T1D vs Control
hsa-miR-144-5p_GGATATCATCATATACTGTAA4	0,493206437	3,901874288	0,362105923	0,700481604	T1D vs Control
hsa-miR-30a-5p_TGTAAACATCCTCGACTGGAA4	-0,179853068	7,169227794	0,365311611	0,704971802	T1D vs Control
hsa-miR-486-5p_TCCTGTACTGAGCTGCCCCGAG4	-0,11627546	17,24661108	0,372186579	0,715486958	T1D vs Control
hsa-miR-502-3p_AATGCACCTGGGCAAGGATTCA4	-0,360735123	4,709212787	0,372555944	0,715486958	T1D vs Control
hsa-miR-126-3p_TCGTACCGTGAGTAATAATG4	0,267992358	5,867396204	0,374061917	0,716021595	T1D vs Control
hsa-miR-2110_TTGGGGAAACGGCCGCTGAGTGA4	-0,27200972	6,066188802	0,374631123	0,716021595	T1D vs Control
hsa-miR-92a-3p_TATTGCACTTGTCCCGGCCT4	0,128111035	10,27945719	0,378723149	0,721102677	T1D vs Control
hsa-miR-423-3p_AGCTCGGTCTGAGGCCCC4	0,457088562	3,540860079	0,379099149	0,721102677	T1D vs Control
hsa-miR-361-3p_TCCCCCAGGTGTGATTCTGA4	0,449143136	3,113214743	0,382372732	0,725456074	T1D vs Control
hsa-miR-30b-5p_TGTAAACATCCTACACTCAGCT4	-0,220115783	6,722043115	0,384193196	0,725456074	T1D vs Control
hsa-let-7d-3p_ATACGACCTGCTGCCTTTCT4	-0,472759928	2,048536833	0,384804937	0,725456074	T1D vs Control
hsa-miR-486-3p_CGGGGCAGCTCAGTACAGGA4	0,2582585	6,539637794	0,385028757	0,725456074	T1D vs Control
hsa-miR-143-3p_TGAGATGAAGCACTGTAGCTC4	-0,130024527	9,842973082	0,386970359	0,727280504	T1D vs Control
hsa-miR-28-3p_CACTAGATTGTGAGCTCCTGG4	-0,266866248	5,876182682	0,388654155	0,727280504	T1D vs Control
hsa-miR-22-3p_AAGCTGCCAGTTGAAGAACTGTT4	0,463486207	2,793276847	0,388734623	0,727280504	T1D vs Control
hsa-miR-27b-3p_TTCACAGTGGCTAAGTTCTGC4	-0,154470548	7,903620851	0,391320004	0,730402911	T1D vs Control
hsa-miR-7-5p_TGGAAGACTAGTGATTTTGTTGTT4	-0,451529968	4,415695214	0,396999556	0,736651191	T1D vs Control
hsa-miR-181a-2-3p_ACCACTGACCGTTGACTGTAC4	-0,443731929	3,544826687	0,398420095	0,736651191	T1D vs Control
hsa-miR-26a-5p_TCAAGTAATCCAGGATAGGCT4	-0,242299396	6,522605962	0,399858173	0,736651191	T1D vs Control
hsa-miR-199a-3p_ACAGTAGTCTGCACATTGGTT4	-0,712955489	4,64378834	0,399978633	0,736651191	T1D vs Control
hsa-miR-194-5p_TGTAACAGCAACTCCATGTGGAA4	-0,440245792	2,659899918	0,401262786	0,736651191	T1D vs Control
hsa-miR-92a-3p_TTGCACTTGTCCCGGCCTG4	-0,395645213	4,313701526	0,403056719	0,736651191	T1D vs Control
hsa-miR-191-5p_CAACGGAATCCCAAAAGCAG4	-0,090024916	10,17632421	0,403314268	0,736651191	T1D vs Control
hsa-miR-155-5p_TTAATGCTAATCGTGATAGGGGT4	0,409440141	3,972069578	0,403687985	0,736651191	T1D vs Control
hsa-miR-451a_AAACCGTTACCATTACTGAGTTTAG4	-0,452211601	2,164703683	0,403743568	0,736651191	T1D vs Control
hsa-miR-424-3p_CAAAACGTGAGGCGCTGCTAT4	-0,273274102	5,519904886	0,403910377	0,736651191	T1D vs Control
hsa-miR-199a-3p_ACAGTAGTCTGCACATTGGTTA4	0,695909208	4,827856003	0,40972782	0,741505261	T1D vs Control
hsa-miR-126-5p_ATTATTACTTTTGGTACGCGCT4	-0,442246099	2,122638684	0,41157432	0,741505261	T1D vs Control
hsa-miR-493-5p_TTGTACATGGTAGGCTTTCATT4	-0,49413536	3,64649227	0,411648685	0,741505261	T1D vs Control
hsa-miR-6803-3p_TCCCTCGCCTTCTCACCCTCA4	-0,257369266	6,298415012	0,414011479	0,741505261	T1D vs Control
hsa-miR-652-3p_AATGGCGCCACTAGGGTTGTG4	-0,219152683	6,468098031	0,416339777	0,741505261	T1D vs Control
hsa-let-7d-3p_TATACGACCTGCTGCCTTT4	-0,207817959	6,275229414	0,416358117	0,741505261	T1D vs Control
hsa-miR-409-3p_CGAATGTTGCTCGGTGAACCCCTT4	-0,485659177	3,646109852	0,416891962	0,741505261	T1D vs Control
hsa-miR-423-5p_GAGGGGCAGAGAGCGAGACTTT4	0,100247787	8,388365906	0,418896082	0,741505261	T1D vs Control
hsa-miR-574-3p_CACGCTCATGCACACACCCA4	-0,428345999	3,365756558	0,41942421	0,741505261	T1D vs Control
hsa-miR-29b-3p_TAGCACCATTTGAAATCAGT4	0,383028418	4,363760053	0,419560751	0,741505261	T1D vs Control
hsa-miR-3615_TCTCTCGGCTCCTCGCGGCT4	0,213726628	6,584121427	0,419678117	0,741505261	T1D vs Control
hsa-let-7a-5p_TGAGGTAGTAGGTTGTAT4	0,537600311	3,216663859	0,419764481	0,741505261	T1D vs Control
hsa-miR-92b-3p_TATTGCACTCGTCCCGGCC4	-0,41724373	3,837730088	0,42033366	0,741505261	T1D vs Control
hsa-miR-99a-5p_AACCCGTAGATCCGATCTTGT4	0,131229463	8,529135227	0,420621956	0,741505261	T1D vs Control
hsa-miR-486-5p_TGTACTGAGCTGCCCCGAG4	0,32918093	5,036658236	0,420658602	0,741505261	T1D vs Control
hsa-miR-484_TCAGGCTCAGTCCCCTCCCG4	-0,1877373	7,780454359	0,422687418	0,741505261	T1D vs Control
hsa-let-7f-5p_GAGGTAGTAGATTGTATAGT4	-0,46017211	3,023408308	0,423224661	0,741505261	T1D vs Control
hsa-miR-28-3p_CACTAGATTGTGAGCTCCTGGA4	-0,210752818	6,806864009	0,423596176	0,741505261	T1D vs Control
hsa-miR-22-3p_AGCTGCCAGTTGAAGAACTGT4	0,295126142	5,523006419	0,42483752	0,741505261	T1D vs Control
hsa-miR-451a_AACCGTTACCATTACTGAGTTT4	-0,429213495	2,400911204	0,428769939	0,741505261	T1D vs Control
hsa-miR-323a-3p_GCACATTACACGGTCGACCTCT4	-0,425766185	3,90036256	0,428870915	0,741505261	T1D vs Control
hsa-miR-146b-5p_TGAGAACTGAATTCCATAGGCTG4	-0,28111883	5,765289472	0,429270633	0,741505261	T1D vs Control

hsa-miR-139-3p_TGGAGACGCGGCCCTGTTGGAGT4	0,44442085	3,435697412	0,430905353	0,741505261	T1D vs Control
hsa-miR-500a-3p_ATGCACCTGGGCAAGGATTCT4	-0,381611509	4,513942296	0,431372884	0,741505261	T1D vs Control
hsa-let-7a-5p_TGAGGTAGTAGGTTGTATA4	-0,264379485	5,663865648	0,432459178	0,741505261	T1D vs Control
hsa-miR-140-3p_ACCACAGGGTAGAACCACGGAC4	-0,258195719	5,852260421	0,433289964	0,741505261	T1D vs Control
hsa-miR-486-5p_CTGTACTGAGCTGCCCCGA4	-0,150341719	7,583597035	0,433759838	0,741505261	T1D vs Control
hsa-miR-150-5p_TCTCCCAACCCTTGTACCAGT4	0,136907105	10,25222562	0,433771923	0,741505261	T1D vs Control
hsa-miR-425-5p_AATGACACGATCACTCCCGTT4	-0,156590413	8,024407696	0,434840523	0,741505261	T1D vs Control
hsa-miR-885-5p_TCCATTACACTACCCTGCCTCT4	-0,84317436	4,714772841	0,435394571	0,741505261	T1D vs Control
hsa-let-7c-5p_TGAGGTAGTAGGTTGTATGG4	0,27460587	3,04066672	0,436357035	0,741505261	T1D vs Control
hsa-miR-224-5p_CAAGTCACTAGTGGTTCCGTTT4	-0,42890977	2,885758309	0,436584527	0,741505261	T1D vs Control
hsa-let-7g-5p_GAGGTAGTAGTTTGTACAG4	-0,434700286	2,076526463	0,438450093	0,741505261	T1D vs Control
hsa-miR-148b-3p_TCAGTGCATCACAGAACTTTG4	0,328276779	4,882892663	0,439358336	0,741505261	T1D vs Control
hsa-miR-744-5p_TGCGGGGCTAGGGCTAACAGC4	-0,378293312	3,991128142	0,440218926	0,741505261	T1D vs Control
hsa-miR-150-5p_CTCCCAACCCTTGTACCAGTG4	0,366731472	4,313974888	0,440244615	0,741505261	T1D vs Control
hsa-miR-125b-5p_TCCCTGAGACCCTAACT4	-0,462603714	2,233733889	0,440995601	0,741505261	T1D vs Control
hsa-miR-30a-5p_GTAAACATCCTCGACTGGAAGCT4	0,377670597	4,1060576	0,445214424	0,745566831	T1D vs Control
hsa-miR-92a-3p_TATTGCACTTGTCCCGGCC4	-0,15187047	8,088512139	0,445282072	0,745566831	T1D vs Control
hsa-miR-15a-5p_TAGCAGCACATAATGGTTTGT4	0,227274916	6,478410353	0,44703463	0,746932075	T1D vs Control
hsa-miR-98-5p_TGAGGTAGTAAGTTGTATTG4	0,353467818	4,611342852	0,448674784	0,74810419	T1D vs Control
hsa-miR-486-5p_ATCCTGTACTGAGCTGCCCCG4	-0,205834382	6,350028468	0,451560166	0,749978666	T1D vs Control
hsa-miR-451a_AAACCGTTACCATTACTGAG4	-0,119023729	13,72832534	0,452164411	0,749978666	T1D vs Control
hsa-miR-1180-3p_TTTCCGGCTCGCGTGGGTGTGT4	-0,389468804	2,874816557	0,453534867	0,749978666	T1D vs Control
hsa-miR-192-5p_TGACCTATGAATTGACAGCCAG4	-0,401698272	3,652730706	0,453563007	0,749978666	T1D vs Control
hsa-miR-16-5p_TAGCAGCACGTAAATATTGGCG4	0,080643734	14,03055319	0,459093521	0,757551835	T1D vs Control
hsa-miR-505-3p_CGTCAACACTTGCTGGTTTCCT4	-0,383264546	3,899410508	0,461323661	0,759659004	T1D vs Control
hsa-miR-23b-3p_ATCACATTGCCAGGGATTACCAC4	-0,33080525	5,113281158	0,464224938	0,762438273	T1D vs Control
hsa-miR-342-3p_TCTCACACAGAAATCGCACCCGTCA4	-0,397501704	3,422364811	0,464924719	0,762438273	T1D vs Control
hsa-miR-629-5p_TGGGTTTACGTTGGGAGAACTT4	0,357813951	4,034612462	0,465882364	0,762439926	T1D vs Control
hsa-miR-16-5p_TAGCAGCACGTAAATATTGG4	0,095006893	10,97240393	0,46689442	0,762530436	T1D vs Control
hsa-miR-181a-2-3p_ACCACTGACCGTTGACTGT4	-0,38878133	2,531276889	0,471300237	0,76722087	T1D vs Control
hsa-miR-152-3p_TCAGTGCATGACAGAACTTGG4	-0,365192109	2,073986305	0,471763221	0,76722087	T1D vs Control
hsa-miR-183-5p_TATGGCACTGGTAGAATT4	-0,404686081	2,805102899	0,474001247	0,76722087	T1D vs Control
hsa-miR-10a-5p_TACCCTGTAGATCCGAATTTG4	-0,103815546	8,855203262	0,474857721	0,76722087	T1D vs Control
hsa-miR-30d-5p_TAAACATCCCCGACTGGAAGCT4	-0,38232889	2,174183718	0,475337073	0,76722087	T1D vs Control
hsa-miR-320b_AAAAGCTGGGTTGAGAGGGCA4	-0,347380166	2,618155177	0,47554217	0,76722087	T1D vs Control
hsa-miR-125b-5p_TCCCTGAGACCCTAACTTGT4	0,099143545	9,802438562	0,478759962	0,770116784	T1D vs Control
hsa-miR-181a-5p_AACATTCAACGCTGTCGGT4	-0,251396357	5,505188114	0,479269667	0,770116784	T1D vs Control
hsa-miR-1228-3p_TCACACCTGCCTCGCCCCCCA4	0,424925283	1,951009842	0,481742199	0,772532259	T1D vs Control
hsa-miR-183-5p_ATGGCACTGGTAGAATTCACTGT4	0,386089801	2,224937851	0,482837106	0,77273328	T1D vs Control
hsa-miR-23a-5p_GGGGTTCCTGGGGATGGGATT4	0,366050534	3,585240207	0,487706922	0,777701998	T1D vs Control
hsa-miR-1908-5p_CGGCGGGGACGGCGATTGGTC4	0,39279233	2,612171571	0,488536599	0,777701998	T1D vs Control
hsa-miR-223-3p_TCAGTTTGTCAAATACCCCAA4	0,368780067	2,060154898	0,488869136	0,777701998	T1D vs Control
hsa-miR-2110_TTGGGGAAACGGCCGCTGAGTG4	-0,399908702	2,932814217	0,489948563	0,777866544	T1D vs Control
hsa-miR-32-5p_TATTGCACATTACTAAGTTG4	0,35112636	3,984130302	0,494365575	0,783318813	T1D vs Control
hsa-let-7g-5p_TGAGGTAGTAGTTTGTACAGTT4	-0,086957581	11,23391478	0,497506843	0,786149643	T1D vs Control
hsa-miR-29a-3p_TAGCACCATCTGAAATCGGTTA4	-0,304804086	4,716466347	0,498746297	0,786149643	T1D vs Control
hsa-miR-30a-5p_TGTAAACATCCTCGACTGGAAGCT4	0,080808988	8,778765877	0,499111317	0,786149643	T1D vs Control
hsa-miR-126-5p_CATTATTACTTTTGGTACGC4	0,341278338	3,620244945	0,501435571	0,787963516	T1D vs Control
hsa-miR-4732-5p_TGTAGAGCAGGGAGCAGGAAGCT4	0,207467206	6,276959061	0,502240234	0,787963516	T1D vs Control
hsa-miR-361-5p_TTATCAGAATCTCCAGGGGT4	0,268662921	4,747764862	0,506314749	0,791386671	T1D vs Control
hsa-miR-27b-3p_TTCACAGTGGCTAAGTTCTG4	0,115278942	8,279294906	0,506408033	0,791386671	T1D vs Control
hsa-miR-361-5p_TTATCAGAATCTCCAGGGGTA4	-0,3502396	3,298750595	0,508723078	0,793422539	T1D vs Control
hsa-miR-142-5p_CCCATAAAGTAGAAAGCACT4	-0,062723304	11,13638127	0,509701806	0,793422539	T1D vs Control
hsa-miR-140-3p_TACCACAGGGTAGAACCACGGA4	-0,132767888	7,894124989	0,511173801	0,794162806	T1D vs Control
hsa-miR-451a_ACCGTTACCATTACTGAGT4	-0,156122448	7,452815255	0,512410438	0,794535251	T1D vs Control
hsa-miR-93-5p_CAAAGTGCTGTTCGTGCAGGTA4	-0,2474532	5,442816943	0,516199313	0,798856025	T1D vs Control
hsa-miR-30a-5p_TGTAAACATCCTCGACTGGAAGC4	-0,109363145	7,798673355	0,518909605	0,800628382	T1D vs Control
hsa-miR-340-5p_TTATAAAGCAATGAGACTGAT4	-0,353740119	1,968507467	0,519353668	0,800628382	T1D vs Control
hsa-miR-1301-3p_TTGCAGCTGCCTGGGAGTGA4	-0,345788103	3,347749467	0,521940911	0,801549048	T1D vs Control
hsa-miR-99a-5p_AACCCGTAGATCCGATCTT4	0,355142944	3,048788798	0,521962303	0,801549048	T1D vs Control
hsa-miR-361-5p_TTATCAGAATCTCCAGGGGTACT4	0,343652934	2,611707431	0,523492128	0,802352358	T1D vs Control

hsa-miR-27a-3p_TTCACAGTGGCTAAGTTCC4	-0,215046078	5,369078847	0,525233467	0,803476148	T1D vs Control
hsa-miR-191-5p_ACGGAATCCCAAAAGCAGCT4	-0,346031926	1,904502114	0,529009314	0,80770196	T1D vs Control
hsa-miR-92b-3p_TATTGCACTCGTCCCGGCCT4	-0,231990596	5,68469958	0,532322746	0,811206938	T1D vs Control
hsa-miR-144-5p_GGATATCATCATATACTGTAAG4	-0,346845259	4,224705339	0,534766961	0,813376465	T1D vs Control
hsa-miR-3615_TCTCTCGGCTCCTCGCGGC4	-0,330559629	2,019798482	0,537243386	0,815586626	T1D vs Control
hsa-miR-589-5p_TGAGAACCACGTCTGCTCTGA4	0,333512256	2,896824842	0,540242099	0,818579758	T1D vs Control
hsa-miR-450b-5p_TTTTGCAATATGTTCCTGAAT4	-0,259864014	5,016886951	0,542551539	0,820147843	T1D vs Control
hsa-miR-16-5p_TAGCAGCACGTAAATATTGGCGT4	0,111130577	7,90073048	0,543577555	0,820147843	T1D vs Control
hsa-miR-146a-5p_GAGAACTGAATTCCATGGGTT4	-0,18934558	6,481720435	0,544364126	0,820147843	T1D vs Control
hsa-let-7f-5p_TGAGGTAGTAGATTGTAT4	-0,354249367	3,174737293	0,545507025	0,820319055	T1D vs Control
hsa-miR-342-3p_CTCACACAGAAATCGCACCCG4	-0,336122161	2,495329338	0,554805271	0,832730321	T1D vs Control
hsa-miR-16-5p_GCAGCACGTAAATATTGGCG4	-0,282955247	3,625120505	0,556530497	0,833749636	T1D vs Control
hsa-miR-486-3p_CGGGGCAGCTCAGTACAGGAT4	-0,136008939	7,803889597	0,559544137	0,83649105	T1D vs Control
hsa-miR-15b-5p_TAGCAGCACATCATGGTTTAC4	-0,115864896	7,368236859	0,560459499	0,83649105	T1D vs Control
hsa-miR-421_ATCAACAGACATTAATTGGGCGC4	-0,299955397	2,04052248	0,563688122	0,83912715	T1D vs Control
hsa-miR-23a-5p_GGGGTTCCTGGGGATGGGATTT4	-0,296127777	3,81817711	0,564331433	0,83912715	T1D vs Control
hsa-miR-92b-3p_TATTGCACTCGTCCCGGCCTC4	0,273716106	3,886574178	0,566364952	0,839153194	T1D vs Control
hsa-miR-92a-3p_GTATTGCACTTGTCCCGGCCTGT4	-0,09634736	7,715362913	0,566454728	0,839153194	T1D vs Control
hsa-miR-500a-3p_AATGCACCTGGGCAAGGATTCT4	-0,332615862	2,780066987	0,570333107	0,843331143	T1D vs Control
hsa-miR-375-3p_TTTGTTCGTTCGGCTCGCG4	0,214400337	5,830563474	0,572345692	0,844739845	T1D vs Control
hsa-miR-486-5p_TCCTGTACTGAGCTGCCC4	-0,207765106	3,88539215	0,574990553	0,84610379	T1D vs Control
hsa-miR-146b-5p_TGAGAACTGAATTCCATAGGCT4	-0,155893384	6,34592746	0,575393041	0,84610379	T1D vs Control
hsa-miR-98-5p_TGAGGTAGTAAGTTGTATTGTT4	-0,140335485	7,877215034	0,57721651	0,846301292	T1D vs Control
hsa-let-7g-5p_GAGGTAGTAGTTTGTACAGTT4	-0,305113833	4,344091711	0,578351866	0,846301292	T1D vs Control
hsa-miR-150-3p_CTGGTACAGGCCTGGGGGAC4	-0,312086934	2,308717969	0,579437837	0,846301292	T1D vs Control
hsa-miR-16-2-3p_CCAATATTACTGTGCTGCTT4	-0,218562621	4,446564737	0,579774787	0,846301292	T1D vs Control
hsa-miR-191-5p_AACGGAATCCCAAAAGCAGC4	-0,289664173	2,920397045	0,582778332	0,849130403	T1D vs Control
hsa-miR-21-5p_TAGCTTATCAGACTGATGTT4	0,106583468	7,170728426	0,584929397	0,849179916	T1D vs Control
hsa-miR-30a-3p_CTTTCAGTCGGATGTTTGCAG4	-0,293879717	1,943754996	0,584943254	0,849179916	T1D vs Control
hsa-miR-451a_ACCGTTACCATTACTGAG4	-0,269526335	3,857793403	0,587103355	0,850766134	T1D vs Control
hsa-miR-1908-5p_CGGCGGGGACGGCGATTGGT4	-0,318297706	2,686402078	0,589749457	0,851983336	T1D vs Control
hsa-miR-146a-5p_TGAGAACTGAATTCCATGGGTT4	-0,061322017	12,1607009	0,591699343	0,851983336	T1D vs Control
hsa-miR-144-3p_TACAGTATAGATGATGTAC4	-0,142500993	6,460339014	0,592814802	0,851983336	T1D vs Control
hsa-miR-423-5p_AGGGGCAGAGAGCGAGACTTTT4	0,254956494	4,864713364	0,593726625	0,851983336	T1D vs Control
hsa-miR-197-3p_TTCACCACCTTCTCCACCCAG4	-0,092025932	8,762482756	0,594120682	0,851983336	T1D vs Control
hsa-let-7b-5p_TGAGGTAGTAGGTTGTGTGGT4	-0,054675178	11,84645942	0,595139129	0,851983336	T1D vs Control
hsa-miR-182-5p_TTTGGCAATGGTAGAACTCACACT4	-0,117935685	7,651119737	0,595426246	0,851983336	T1D vs Control
hsa-let-7g-5p_TGAGGTAGTAGTTTGTACA4	0,220884258	5,425002874	0,600672639	0,857949988	T1D vs Control
hsa-miR-30d-5p_TGTAAACATCCCCGACTGGAAG4	-0,040141771	12,16128446	0,606144063	0,864216133	T1D vs Control
hsa-miR-181a-5p_AACATTCAACGCTGTCGGTG4	-0,123000585	6,983532365	0,610471591	0,867529548	T1D vs Control
hsa-miR-93-5p_CAAAGTGCTGTTCGTGCAGGT4	-0,184779526	4,918359962	0,61108448	0,867529548	T1D vs Control
hsa-miR-501-3p_AATGCACCCGGGCAAGGAT4	-0,276469564	2,760223516	0,611733508	0,867529548	T1D vs Control
hsa-miR-4732-3p_GCCCTGACCTGTCCTGTTCTG4	0,428124777	4,156168491	0,614816543	0,869593439	T1D vs Control
hsa-miR-361-3p_TCCCCCAGGTGTGATTCTGATTT4	0,240710797	3,977031543	0,616880489	0,869593439	T1D vs Control
hsa-miR-486-5p_CCTGTACTGAGCTGCCCCG4	-0,102154656	8,015321722	0,61790628	0,869593439	T1D vs Control
hsa-miR-363-3p_AATTGCACGGTATCCATCTGTA4	0,246095163	3,912624597	0,618579266	0,869593439	T1D vs Control
hsa-miR-10b-5p_TACCCTGTAGAACCGAATT4	-0,232116609	4,675674869	0,620833466	0,869593439	T1D vs Control
hsa-let-7a-5p_TGAGGTAGTAGGTTGTATAG4	-0,051710981	12,05467063	0,620991241	0,869593439	T1D vs Control
hsa-miR-543_AAACATTCGCGGTGCACTTCTTT4	-0,299017387	3,173881718	0,622068678	0,869593439	T1D vs Control
hsa-miR-433-3p_ATCATGATGGGCTCCTCGGTGT4	-0,280632859	4,303379714	0,622797062	0,869593439	T1D vs Control
hsa-miR-323b-3p_CCCAATACACGGTCGACCTCT4	-0,23338801	5,853793118	0,623605621	0,869593439	T1D vs Control
hsa-miR-3158-3p_AAGGGCTTCCTCTCTGCAGGA4	0,282991786	2,734692583	0,624099683	0,869593439	T1D vs Control
hsa-miR-125b-5p_TCCCTGAGACCCTAACTTG4	-0,07908984	8,171400371	0,626667067	0,869850239	T1D vs Control
hsa-miR-425-5p_ATGACACGATCACTCCCGTTG4	-0,267152132	2,398751928	0,626976191	0,869850239	T1D vs Control
hsa-miR-532-3p_CCTCCCACACCCAAGGCTTG4	-0,270728332	2,745637111	0,629928975	0,869850239	T1D vs Control
hsa-miR-4433b-5p_ATGTCCCACCCCCACTCCTG4	-0,258972211	2,006165102	0,632157851	0,869850239	T1D vs Control
hsa-miR-423-3p_GCTCGGTCTGAGGCCCCTCAGT4	-0,144704471	6,432183419	0,633033321	0,869850239	T1D vs Control
hsa-miR-363-3p_ATTGCACGGTATCCATCTG4	0,259787376	2,060471037	0,633109502	0,869850239	T1D vs Control
hsa-miR-24-3p_TGGCTCAGTTCAGCAGGAAC4	0,053837652	10,07194833	0,633426776	0,869850239	T1D vs Control
hsa-let-7b-5p_TGAGGTAGTAGGTTGTGTG4	-0,098954454	6,962968331	0,63377526	0,869850239	T1D vs Control
hsa-miR-16-5p_AGCAGCACGTAAATATTGGCG4	0,074423955	8,17366975	0,634432448	0,869850239	T1D vs Control

hsa-miR-181a-5p_AACATTCAACGCTGTCGGTGAGT4	0,089750601	8,031521361	0,637157774	0,869850239	T1D vs Control
hsa-miR-25-3p_CATTGCACTTGTCTCGGTCTG4	-0,079580734	9,990945876	0,637442102	0,869850239	T1D vs Control
hsa-miR-3613-5p_TGTTGTACTTTTTTTTTTGT4	0,073773371	7,586394808	0,63816204	0,869850239	T1D vs Control
hsa-miR-92a-3p_TATTGCACTTGTCCCGGCCTG4	-0,0641497	13,95782504	0,638472258	0,869850239	T1D vs Control
hsa-miR-101-3p_GTACAGTACTGTGATAACTGAA4	-0,255596373	3,655178095	0,641557206	0,871310214	T1D vs Control
hsa-miR-451a_AAACCGTTACCATTACTGAGT4	0,066683115	16,51409079	0,641730358	0,871310214	T1D vs Control
hsa-miR-21-5p_TAGCTTATCAGACTGATG4	0,246893859	1,940313304	0,645266401	0,87373452	T1D vs Control
hsa-let-7a-5p_ATGAGGTAGTAGGTTGTATAGTT4	0,263121244	2,103462699	0,645751205	0,87373452	T1D vs Control
hsa-miR-24-3p_GCTCAGTTCAGCAGGAACAG4	-0,237780155	2,25101941	0,647055259	0,87373452	T1D vs Control
hsa-miR-30c-5p_TGTAAACATCCTACACTCTCAG4	-0,19753305	4,767658059	0,647901005	0,87373452	T1D vs Control
hsa-miR-1307-3p_CTCGGCGTGGCGTCGGTCGTGG4	-0,259434601	2,190246459	0,649370564	0,87423706	T1D vs Control
hsa-miR-192-5p_CTGACCTATGAATTGACAGC4	0,251630466	1,977431527	0,652292883	0,876690435	T1D vs Control
hsa-miR-182-5p_TTTGGCAATGGTAGAACTCACACTGG4	-0,257517312	2,315372824	0,654608872	0,878322005	T1D vs Control
hsa-let-7g-5p_GAGGTAGTAGTTTGTACAGT4	-0,245146059	3,423010066	0,658560161	0,882138569	T1D vs Control
hsa-let-7i-5p_GAGGTAGTAGTTTGTGCTG4	-0,244045928	2,272109077	0,661663436	0,884656657	T1D vs Control
hsa-miR-23a-3p_TCACATTGCCAGGGATTTC4	0,246912292	2,403793771	0,662777121	0,884656657	T1D vs Control
hsa-let-7f-5p_TGAGGTAGTAGATTG4	0,222747969	4,164688749	0,664451735	0,884656657	T1D vs Control
hsa-miR-143-3p_TGAGATGAAGCACTGTAGCT4	0,071860362	9,047255511	0,664879972	0,884656657	T1D vs Control
hsa-miR-32-5p_TATTGCACATTACTAAGTTGC4	0,234124098	2,00688677	0,668949981	0,887905067	T1D vs Control
hsa-miR-193a-5p_TGGGTCTTTGCGGGCGAGA4	-0,244244908	3,488597754	0,669549492	0,887905067	T1D vs Control
hsa-miR-92a-3p_TATTGCACTTGTCCCGGCCTGT4	-0,039299903	17,23184948	0,672683883	0,890579825	T1D vs Control
hsa-miR-10b-5p_TACCCTGTAGAACCGAATTTGTGT4	-0,229786991	2,268128931	0,674202479	0,891110075	T1D vs Control
hsa-miR-24-3p_TGGCTCAGTTCAGCAGGAA4	-0,160719704	5,648746767	0,676984102	0,893305181	T1D vs Control
hsa-miR-130b-3p_CAGTGCAATGATGAAAGGGCA4	-0,173228856	4,137452675	0,682128202	0,897275515	T1D vs Control
hsa-miR-126-3p_TCGTACCGTGAGTAATAATGCG4	0,054603315	11,19647801	0,68224462	0,897275515	T1D vs Control
hsa-miR-23a-3p_TCACATTGCCAGGGATTTCC4	-0,190020905	4,540513631	0,685197385	0,899674326	T1D vs Control
hsa-miR-125a-5p_TCCCTGAGACCCTTTAACC4	0,210920643	2,158659604	0,688477978	0,900404675	T1D vs Control
hsa-miR-486-5p_TCCTGTACTGAGCTGCCCCG4	-0,062640926	14,12822837	0,688641048	0,900404675	T1D vs Control
hsa-miR-486-5p_CCTGTACTGAGCTGCCCCGA4	-0,059398326	11,08007536	0,68914285	0,900404675	T1D vs Control
hsa-miR-30a-5p_TGTAAACATCCTCGACTGG4	0,183836568	4,494972654	0,690990387	0,901340979	T1D vs Control
hsa-miR-122-5p_GGAGTGTGACAATGGTGTTT4	0,134618378	7,666405464	0,692593525	0,901955947	T1D vs Control
hsa-miR-382-5p_AAGTTGTTCGTGGTGGATTCG4	-0,228703786	2,227150711	0,700201513	0,910300438	T1D vs Control
hsa-miR-25-3p_ATTGCACTTGTCTCGGTCTG4	-0,18784582	3,200649251	0,701285406	0,910300438	T1D vs Control
hsa-let-7f-5p_TGAGGTAGTAGATTGTATAGT4	0,039155259	10,56301224	0,703842237	0,910676791	T1D vs Control
hsa-miR-183-5p_TATGGCACTGGTAGAATTCACTG4	0,213517924	3,516695948	0,704357718	0,910676791	T1D vs Control
hsa-miR-126-3p_CGTACCGTGAGTAATAATGCG4	0,053312063	9,683358758	0,706081537	0,910676791	T1D vs Control
hsa-miR-486-5p_TCCTGTACTGAGCTGCCCCGAGC4	-0,080874473	6,188232343	0,706145868	0,910676791	T1D vs Control
hsa-miR-483-5p_AAGACGGGAGGAAAGAAGGGAG4	0,127984719	6,618430366	0,708658797	0,911401788	T1D vs Control
hsa-miR-652-3p_AATGGCGCCACTAGGGTTG4	-0,194095906	4,499546053	0,708995118	0,911401788	T1D vs Control
hsa-miR-425-5p_ATGACACGATCACTCCCGTTGA4	-0,164419215	4,729605379	0,711015438	0,91252706	T1D vs Control
hsa-miR-142-5p_CATAAAGTAGAAAGCACTACT4	0,181377309	3,650597591	0,71531952	0,916575012	T1D vs Control
hsa-miR-182-5p_TTTGGCAATGGTAGAACTCACACTG4	-0,200985428	3,598930548	0,721249392	0,920651077	T1D vs Control
hsa-miR-27a-3p_TCACAGTGGCTAAGTTCCG4	-0,195633839	2,668037972	0,721457789	0,920651077	T1D vs Control
hsa-miR-3173-5p_TGCCCTGCCTGTTTTCTCCTT4	0,195659845	2,97364428	0,722868435	0,920651077	T1D vs Control
hsa-miR-19a-3p_TGTGCAAATCTATGCAAAACTGA4	0,189406149	2,326203362	0,723121172	0,920651077	T1D vs Control
hsa-miR-425-5p_AATGACACGATCACTCCCG4	-0,159936794	4,547943713	0,729069033	0,926743253	T1D vs Control
hsa-miR-145-5p_GTCCAGTTTTCCCAGGAATCCC4	-0,193962581	3,12605684	0,733656995	0,931090167	T1D vs Control
hsa-miR-505-3p_CGTCAACACTTGCTGGTTTCCTCT4	0,196145391	2,947139365	0,735014646	0,931330163	T1D vs Control
hsa-miR-26a-5p_TTCAAGTAATCCAGGATAGGC4	-0,052673111	7,510198846	0,740413772	0,935925024	T1D vs Control
hsa-miR-92a-3p_ATTGCACTTGTCCCGGCCTG4	0,052158994	9,121941427	0,740989573	0,935925024	T1D vs Control
hsa-miR-28-5p_AAGGAGCTCACAGTCTATTGAG4	-0,184420357	3,384277878	0,74309026	0,937093255	T1D vs Control
hsa-miR-193a-5p_TGGGTCTTTGCGGGCGAGATGA4	-0,052931232	8,630407946	0,748663425	0,940942861	T1D vs Control
hsa-miR-28-5p_AAGGAGCTCACAGTCTATTGA4	-0,189057703	3,11393162	0,749643644	0,940942861	T1D vs Control
hsa-let-7d-5p_AGAGGTAGTAGGTTGCATAG4	-0,057796533	8,573362788	0,749684714	0,940942861	T1D vs Control
hsa-miR-423-5p_CTGAGGGGCAGAGAGCGAGACT4	-0,165523281	2,475285897	0,75440792	0,943490612	T1D vs Control
hsa-miR-92a-3p_ATTGCACTTGTCCCGGCCTGT4	-0,032457586	12,31240602	0,757115336	0,943490612	T1D vs Control
hsa-miR-126-3p_CGTACCGTGAGTAATAATGC4	0,130266435	4,46578231	0,757470999	0,943490612	T1D vs Control
hsa-miR-27a-3p_TTCACAGTGGCTAAGTTCCG4	-0,037026031	9,740987062	0,758157434	0,943490612	T1D vs Control
hsa-miR-486-5p_TCCTGTACTGAGCTGCCCC4	-0,06462287	6,793601467	0,760274582	0,943490612	T1D vs Control
hsa-miR-374b-5p_ATATAATACAACCTGCTAAGT4	-0,14967914	3,95574137	0,760639873	0,943490612	T1D vs Control
hsa-miR-25-3p_CATTGCACTTGTCTCGGTCTGA4	-0,043266791	11,99390817	0,760875235	0,943490612	T1D vs Control

hsa-miR-339-3p_TGAGCGCCTCGACGACAGAG4	0,120007023	5,154516456	0,76367331	0,943490612	T1D vs Control
hsa-miR-660-5p_TACCCATTGCATATCGGAGTTGT4	0,151534463	3,90808508	0,763710796	0,943490612	T1D vs Control
hsa-miR-183-5p_TATGGCACTGGTAGAATTCACT4	0,112938145	5,796604044	0,766871497	0,943490612	T1D vs Control
hsa-miR-361-5p_TTATCAGAATCTCCAGGGGTAC4	-0,072305082	6,634693775	0,767533249	0,943490612	T1D vs Control
hsa-miR-100-5p_AACCCGTAGATCCGAACTTGT4	-0,071105619	7,211971456	0,767665986	0,943490612	T1D vs Control
hsa-miR-30e-5p_GTAAACATCCTTGACTGGAAGCT4	0,031445506	8,781643893	0,768565218	0,943490612	T1D vs Control
hsa-miR-221-3p_AGCTACATTGTCTGCTGGGTT4	-0,090332723	6,683307876	0,769324281	0,943490612	T1D vs Control
hsa-miR-486-5p_GTACTGAGCTGCCCCGA4	0,113785897	4,609129379	0,77054471	0,943490612	T1D vs Control
hsa-miR-451a_ACCGTTACCATTACTGAGTT4	-0,156400634	2,004581894	0,770655443	0,943490612	T1D vs Control
hsa-miR-144-3p_TACAGTATAGATGATGTACT4	0,132955418	4,501383498	0,772151805	0,943872682	T1D vs Control
hsa-miR-320b_AAAAGCTGGGTTGAGAGGGCAA4	0,14345831	1,963551094	0,774591614	0,945405079	T1D vs Control
hsa-miR-345-5p_GCTGACTCCTAGTCCAGGGCT4	-0,115638535	5,057881275	0,777294694	0,946569331	T1D vs Control
hsa-miR-125b-5p_TCCCTGAGACCCTAACTTGTG4	0,117473326	5,039062696	0,777920843	0,946569331	T1D vs Control
hsa-miR-92a-3p_ATTGCACTTGTCCCGGCCT4	0,134397459	4,477303001	0,780250324	0,946682475	T1D vs Control
hsa-miR-15b-5p_TAGCAGCACATCATGGTTTA4	-0,091172461	5,794504936	0,780389443	0,946682475	T1D vs Control
hsa-miR-501-3p_ATGCACCCGGGCAAGGATTCT4	-0,151834193	2,081940648	0,786571576	0,952731833	T1D vs Control
hsa-miR-451a_AAACCGTTACCATTACTGAGTTTAGT4	0,114319572	4,187049919	0,788862803	0,95379583	T1D vs Control
hsa-miR-155-5p_TTAATGCTAATCGTGATAGGGGTT4	0,151752131	2,993899159	0,789843473	0,95379583	T1D vs Control
hsa-miR-92a-3p_TATTGCACTTGTCCCGGCCTGTG4	0,041839857	7,105169059	0,793496728	0,955397157	T1D vs Control
hsa-miR-122-5p_GGAGTGTGACAATGGTGTTTG4	-0,110724089	5,615777051	0,793567024	0,955397157	T1D vs Control
hsa-miR-10a-5p_ACCCTGTAGATCCGAATTTGT4	-0,05440227	7,643285241	0,795970396	0,956185866	T1D vs Control
hsa-miR-423-3p_GCTCGGTCTGAGGCCCCTCAG4	-0,142326446	1,954419998	0,797073035	0,956185866	T1D vs Control
hsa-miR-30c-5p_TGTAAACATCCTACACTCTCAGC4	-0,068871563	6,545485648	0,797821331	0,956185866	T1D vs Control
hsa-miR-191-5p_AACGGAATCCCAAAAGCAGCTG4	-0,080538829	5,747755673	0,799744617	0,957051742	T1D vs Control
hsa-miR-98-5p_TGAGGTAGTAAGTTGTATTGT4	0,078079893	6,55627531	0,802258456	0,958620674	T1D vs Control
hsa-miR-486-5p_TCCTGTACTGAGCTGCCCCGA4	-0,033643398	17,03890679	0,804651952	0,960041326	T1D vs Control
hsa-miR-150-5p_TCTCCCAACCCTTGTACCAG4	0,051850596	9,063580505	0,807556347	0,962066381	T1D vs Control
hsa-miR-505-3p_CGTCAACACTTGCTGGTTT4	-0,126798755	3,249239604	0,810759734	0,964441056	T1D vs Control
hsa-miR-30e-5p_TGTAAACATCCTTGACTGGAAG4	-0,040593234	7,619075745	0,812796816	0,965423341	T1D vs Control
hsa-let-7i-5p_TGAGGTAGTAGTTTGTGCT4	0,048298631	7,615350025	0,816077933	0,965802595	T1D vs Control
hsa-miR-140-3p_ACCACAGGGTAGAACCACG4	-0,124156825	2,665108795	0,817116577	0,965802595	T1D vs Control
hsa-miR-182-5p_TTTGGCAATGGTAGAACTCA4	-0,051965696	7,910992501	0,817689007	0,965802595	T1D vs Control
hsa-miR-140-3p_ACCACAGGGTAGAACCACGGACA4	0,108706887	4,192134912	0,817963302	0,965802595	T1D vs Control
hsa-miR-24-3p_GGCTCAGTTCAGCAGGAACAG4	0,050809587	6,812383366	0,82177868	0,966739127	T1D vs Control
hsa-miR-26a-5p_TTCAAGTAATCCAGGATAGG4	-0,046664685	6,813190078	0,8230225	0,966739127	T1D vs Control
hsa-miR-486-5p_CTGTACTGAGCTGCCCCGAG4	0,042424375	7,787642307	0,832289997	0,966739127	T1D vs Control
hsa-miR-30e-5p_TGTAAACATCCTTGACTGGA4	-0,115052204	2,511168014	0,833101785	0,966739127	T1D vs Control
hsa-miR-375-3p_TTTGTTCGTTCGGCTCGCGT4	-0,113051665	3,894636907	0,835863884	0,966739127	T1D vs Control
hsa-let-7i-5p_TGAGGTAGTAGTTTGTGCTGTTG4	-0,105256462	3,894577335	0,836143727	0,966739127	T1D vs Control
hsa-miR-181a-2-3p_ACCACTGACCGTTGACTGTACC4	-0,114372126	2,397891497	0,840035137	0,966739127	T1D vs Control
hsa-miR-342-3p_TCACACAGAAATCGCACCCGTC4	-0,109592716	2,309823181	0,840045942	0,966739127	T1D vs Control
hsa-miR-1306-5p_CCACCTCCCCTGCAAACGT4	0,120228838	2,734536196	0,840728498	0,966739127	T1D vs Control
hsa-miR-423-3p_AAGCTCGGTCTGAGGCCCCT4	0,112510531	2,46496229	0,841897162	0,966739127	T1D vs Control
hsa-miR-148a-3p_TCAGTGCACTACAGAACTTTG4	0,039467842	7,287277231	0,842659721	0,966739127	T1D vs Control
hsa-let-7d-3p_CTATACGACCTGCTGCCTTT4	-0,024949799	10,24742095	0,843425885	0,966739127	T1D vs Control
hsa-miR-16-2-3p_CCAATATTACTGTGCTGCTTT4	-0,087036714	4,35154905	0,844447697	0,966739127	T1D vs Control
hsa-miR-342-3p_TCACACAGAAATCGCACCCGT4	0,104705866	2,35866661	0,844928578	0,966739127	T1D vs Control
hsa-miR-192-5p_TGACCTATGAATTGACAGCC4	-0,054756095	6,449264096	0,84493623	0,966739127	T1D vs Control
hsa-miR-423-5p_GAGGGGCAGAGAGCGAGACTT4	0,076956676	5,367382166	0,845237877	0,966739127	T1D vs Control
hsa-miR-345-5p_GCTGACTCCTAGTCCAGGGCTC4	0,107927458	3,656556535	0,845657967	0,966739127	T1D vs Control
hsa-miR-451a_AAACCGTTACCATTACTGAGTTT4	0,030533827	8,99265157	0,846380565	0,966739127	T1D vs Control
hsa-miR-22-3p_AAGCTGCCAGTTGAAGAA4	-0,089788187	4,931866064	0,846743787	0,966739127	T1D vs Control
hsa-miR-335-3p_GTTTTTCATTATTGCTCCTGACC4	-0,108154666	2,935136252	0,850764843	0,966739127	T1D vs Control
hsa-let-7a-5p_TGAGGTAGTAGGTTGTATAGT4	-0,017993181	11,86713156	0,852896939	0,966739127	T1D vs Control
hsa-miR-223-3p_TGTCAGTTTGTCAAATACCC4	0,08358065	4,516322797	0,853338672	0,966739127	T1D vs Control
hsa-miR-139-5p_TCTACAGTGCACGTGTCTCCAGT4	-0,047229922	6,587018882	0,853465752	0,966739127	T1D vs Control
hsa-miR-15b-5p_TAGCAGCACATCATGGTTT4	0,030351001	8,514548835	0,854209329	0,966739127	T1D vs Control
hsa-let-7a-5p_GAGGTAGTAGGTTGTATAGTT4	-0,045647196	7,081547935	0,854656864	0,966739127	T1D vs Control
hsa-miR-10a-5p_TACCCTGTAGATCCGAATTTGT4	-0,0200995	10,44682905	0,855978433	0,966739127	T1D vs Control
hsa-let-7d-3p_CTATACGACCTGCTGCCTTTC4	0,018011736	10,32598693	0,85620254	0,966739127	T1D vs Control
hsa-miR-22-3p_AAGCTGCCAGTTGAAGAAC4	0,033200285	8,203664635	0,856297081	0,966739127	T1D vs Control

hsa-miR-16-5p_AGCAGCACGTAAATATTGG4	-0,093338494	3,167012316	0,857293859	0,966739127	T1D vs Control
hsa-miR-4446-3p_CAGGGCTGGCAGTGACATGGGT4	-0,107739758	3,598813459	0,858446187	0,966739127	T1D vs Control
hsa-miR-150-5p_GTCTCCCAACCCTTGTACCAGTG4	-0,099033818	2,975164068	0,859619908	0,966739127	T1D vs Control
hsa-miR-320a-3p_AAAAGCTGGGTTGAGAGGGCGAAA4	0,048463276	6,744969015	0,859829444	0,966739127	T1D vs Control
hsa-miR-146a-5p_TGAGAACTGAATTCCATGGGT4	-0,023240273	8,67354952	0,85988939	0,966739127	T1D vs Control
hsa-let-7i-5p_GAGGTAGTAGTTTGTGCTGT4	-0,080260588	4,789553904	0,860175529	0,966739127	T1D vs Control
hsa-miR-320a-3p_AAAGCTGGGTTGAGAGGGCGAA4	-0,07914744	4,271030608	0,862097987	0,966739127	T1D vs Control
hsa-let-7a-5p_AGGTAGTAGGTTGTATAGTT4	-0,096125364	1,879378714	0,862423487	0,966739127	T1D vs Control
hsa-miR-144-3p_CTACAGTATAGATGATGTAC4	-0,09111523	3,24422031	0,864452091	0,967214315	T1D vs Control
hsa-miR-10b-5p_TACCCTGTAGAACCGAATTTGTG4	0,033517507	7,78613959	0,865982292	0,967214315	T1D vs Control
hsa-miR-182-5p_TTTGGCAATGGTAGAACT4	-0,09359747	3,421424723	0,866488107	0,967214315	T1D vs Control
hsa-miR-99b-5p_CACCCGTAGAACCGACCTT4	-0,089568038	2,002435028	0,870401694	0,970223987	T1D vs Control
hsa-miR-99a-5p_AACCCGTAGATCCGATCTTG4	0,028449339	8,041845005	0,872143826	0,970808142	T1D vs Control
hsa-miR-23b-3p_ATCACATTGCCAGGGATTACCA4	-0,045421158	6,460852955	0,874105774	0,971351145	T1D vs Control
hsa-miR-342-5p_AGGGGTGCTATCTGTGATTGA4	-0,088556558	3,958584465	0,875944359	0,971351145	T1D vs Control
hsa-let-7c-5p_TGAGGTAGTAGGTTGTATGGTTT4	0,076398132	3,566946574	0,876287921	0,971351145	T1D vs Control
hsa-miR-140-3p_ACCACAGGGTAGAACCACGGA4	-0,042050866	6,646596672	0,878684226	0,971463946	T1D vs Control
hsa-let-7g-5p_TGAGGTAGTAGTTTGTACAG4	-0,019215506	9,407543438	0,879623768	0,971463946	T1D vs Control
hsa-miR-26a-5p_TTCAAGTAATCCAGGATAG4	0,07615348	3,646262645	0,881666046	0,971463946	T1D vs Control
hsa-miR-22-3p_AGCTGCCAGTTGAAGAACT4	0,083891394	2,67043052	0,882195853	0,971463946	T1D vs Control
hsa-miR-10a-5p_TACCCTGTAGATCCGAATT4	-0,075269439	3,933865051	0,884562774	0,971463946	T1D vs Control
hsa-miR-122-5p_GAGTGTGACAATGGTGTTT4	0,086234419	2,929528654	0,885801408	0,971463946	T1D vs Control
hsa-miR-451a_AACCGTTACCATTACTGAGT4	-0,021396531	10,87302044	0,888339196	0,971463946	T1D vs Control
hsa-miR-629-5p_TGGGTTTACGTTGGGAGAA4	-0,07950835	2,515869067	0,890761309	0,971463946	T1D vs Control
hsa-miR-19b-3p_TGTGCAAATCCATGCAAAACTGA4	0,063333557	4,916784309	0,890902297	0,971463946	T1D vs Control
hsa-miR-342-3p_TCTCACACAGAAATCGCACCCGT4	0,025486782	8,706959622	0,891312333	0,971463946	T1D vs Control
hsa-miR-363-3p_AATTGCACGGTATCCATCT4	-0,063594038	4,478964619	0,8936651	0,971463946	T1D vs Control
hsa-miR-10a-5p_TACCCTGTAGATCCGAAT4	-0,075919011	2,33323359	0,893752184	0,971463946	T1D vs Control
hsa-miR-192-5p_TGACCTATGAATTGACAGCCAGT4	0,068034083	3,322199511	0,895021567	0,971463946	T1D vs Control
hsa-miR-186-5p_AAAGAATTCTCCTTTTGGGCT4	-0,066379229	3,890936215	0,898676588	0,971463946	T1D vs Control
hsa-miR-361-3p_TCCCCCAGGTGTGATTCTGATTTG4	0,044089458	5,241633262	0,89935074	0,971463946	T1D vs Control
hsa-miR-140-5p_CAGTGGTTTTACCCTATGGTAG4	0,06111161	3,871751643	0,901281142	0,971463946	T1D vs Control
hsa-miR-148a-3p_CAGTGCACTACAGAACTTTGT4	0,069736226	2,126116505	0,90161846	0,971463946	T1D vs Control
hsa-miR-194-5p_TGTAACAGCAACTCCATGTGG4	-0,029738838	6,660853419	0,902476118	0,971463946	T1D vs Control
hsa-miR-192-5p_TGACCTATGAATTGACAGCCA4	0,057132532	4,726203979	0,902498447	0,971463946	T1D vs Control
hsa-miR-183-5p_TATGGCACTGGTAGAATTCA4	0,033048308	7,32183424	0,904232183	0,971463946	T1D vs Control
hsa-miR-423-3p_AGCTCGGTCTGAGGCCCCTCAGT4	-0,014305007	11,76682308	0,906447653	0,971463946	T1D vs Control
hsa-miR-181a-5p_AACATTCAACGCTGTCGGTGAGTT4	0,064818656	2,334562434	0,907064507	0,971463946	T1D vs Control
hsa-miR-222-3p_AGCTACATCTGGCTACTGGGTC4	-0,056452832	3,362212617	0,909786729	0,971463946	T1D vs Control
hsa-miR-29a-3p_CTAGCACCATCTGAAATCGGTT4	-0,053669407	3,457099062	0,911381051	0,971463946	T1D vs Control
hsa-miR-664a-3p_TATTCATTTATCCCCAGCCTACA4	-0,057561284	3,022366025	0,913419702	0,971463946	T1D vs Control
hsa-miR-26b-5p_TTCAAGTAATTCAGGATAGGT4	-0,020686716	7,343481575	0,914530011	0,971463946	T1D vs Control
hsa-miR-193b-5p_CGGGGTTTTGAGGGCGAGATGA4	0,062007268	2,282241357	0,915324576	0,971463946	T1D vs Control
hsa-miR-183-5p_ATGGCACTGGTAGAATTCACTG4	0,059190843	2,888906189	0,915468274	0,971463946	T1D vs Control
hsa-miR-221-3p_AGCTACATTGTCTGCTGGGTTT4	-0,018621181	7,916859704	0,915701307	0,971463946	T1D vs Control
hsa-miR-484_TCAGGCTCAGTCCCCTCCCGA4	0,016605992	10,80053241	0,916540313	0,971463946	T1D vs Control
hsa-miR-133a-3p_TTGGTCCCCTTCAACCAGCTGT4	-0,061268716	2,527387136	0,917816228	0,971463946	T1D vs Control
hsa-miR-30a-5p_TGTAAACATCCTCGACTGGA4	-0,053902503	3,19956619	0,918174437	0,971463946	T1D vs Control
hsa-miR-103a-3p_AGCAGCATTGTACAGGGCT4	0,084242836	5,118424827	0,918625405	0,971463946	T1D vs Control
hsa-miR-29a-3p_TAGCACCATCTGAAATCGGTT4	0,017448511	8,641484144	0,91885791	0,971463946	T1D vs Control
hsa-miR-92a-3p_TTGCACTTGTCCCGGCCTGT4	0,013766714	8,384006303	0,919417949	0,971463946	T1D vs Control
hsa-miR-16-5p_TAGCAGCACGTAAATATTG4	-0,033385263	5,566162995	0,920270112	0,971463946	T1D vs Control
hsa-miR-10a-5p_TACCCTGTAGATCCGAATTTGTG4	-0,020247737	7,257876357	0,92675055	0,97496365	T1D vs Control
hsa-miR-425-5p_AATGACACGATCACTCCCGT4	-0,02225073	6,738341736	0,927607911	0,97496365	T1D vs Control
hsa-miR-7-5p_TGGAAGACTAGTGATTTTGTTGT4	-0,033888433	6,176558156	0,928650771	0,97496365	T1D vs Control
hsa-miR-487b-3p_TCGTACAGGGTCATCCACTTT4	0,051476076	2,776617041	0,93056293	0,97496365	T1D vs Control
hsa-miR-423-5p_GAGGGGCAGAGAGCGAGACT4	0,02554986	6,874414686	0,930992019	0,97496365	T1D vs Control
hsa-miR-24-3p_GGCTCAGTTCAGCAGGAAC4	0,051182102	2,859699768	0,932065822	0,97496365	T1D vs Control
hsa-miR-181a-5p_AACATTCAACGCTGTCGGTGAG4	-0,015188284	7,366086853	0,932148434	0,97496365	T1D vs Control
hsa-miR-483-3p_TCACTCCTCTCCTCCCGTCT4	-0,046831885	2,217026305	0,936589517	0,97832483	T1D vs Control
hsa-miR-30e-5p_TGTAAACATCCTTGACTGGAA4	-0,014722152	6,536675988	0,944738743	0,983601332	T1D vs Control

hsa-miR-505-3p_GTCAACACTTGCTGGTTTCCTCT4	0,039723748	2,066469778	0,944889489	0,983601332	T1D vs Control
hsa-miR-30c-5p_TGTAAACATCCTACACTCTCA4	0,013927248	7,955436132	0,946057766	0,983601332	T1D vs Control
hsa-miR-181a-5p_AACATTCAACGCTGTCGG4	0,033490843	2,192700831	0,946681185	0,983601332	T1D vs Control
hsa-miR-25-3p_CATTGCACTTGTCTCGGT4	0,030108186	3,623128964	0,950489422	0,983601332	T1D vs Control
hsa-miR-423-3p_AGCTCGGTCTGAGGCCCCT4	0,01847505	6,038348398	0,951810772	0,983601332	T1D vs Control
hsa-miR-191-5p_AACGGAATCCCAAAAGCAGCT4	-0,014774272	6,816281646	0,95282559	0,983601332	T1D vs Control
hsa-let-7i-5p_TGAGGTAGTAGTTTGTGCTG4	0,008267952	10,07708045	0,954644917	0,983601332	T1D vs Control
hsa-miR-424-5p_CAGCAGCAATTCATGTTTTGA4	0,030264858	2,646439112	0,956273983	0,983601332	T1D vs Control
hsa-miR-21-5p_GTAGCTTATCAGACTGATGTTGA4	-0,030293488	2,217922154	0,956877958	0,983601332	T1D vs Control
hsa-miR-18a-3p_ACTGCCCTAAGTGCTCCTTCT4	0,029221368	3,273299722	0,957664695	0,983601332	T1D vs Control
hsa-let-7g-5p_TGAGGTAGTAGTTTGTACAGT4	-0,004351501	10,7052364	0,958313951	0,983601332	T1D vs Control
hsa-miR-30a-5p_TGTAAACATCCTCGACTGGAAG4	0,008569821	8,337829601	0,960659328	0,983601332	T1D vs Control
hsa-miR-320b_AAAAGCTGGGTTGAGAGGGC4	-0,038688003	5,061955788	0,962056977	0,983601332	T1D vs Control
hsa-miR-193a-5p_TGGGTCTTTGCGGGCGAGATG4	-0,017437613	5,884704134	0,963021384	0,983601332	T1D vs Control
hsa-miR-27b-3p_TTCACAGTGGCTAAGTTCTGCA4	-0,023871246	3,596858077	0,96343252	0,983601332	T1D vs Control
hsa-miR-221-3p_AGCTACATTGTCTGCTGGGT4	0,013695444	6,226471989	0,964186151	0,983601332	T1D vs Control
hsa-miR-29c-3p_TAGCACCATTTGAAATCGGTT4	0,020747412	3,850371057	0,964945077	0,983601332	T1D vs Control
hsa-miR-3173-5p_CCCTGCCTGTTTTCTCCTTTGT4	0,021701955	1,879782778	0,965868889	0,983601332	T1D vs Control
hsa-miR-374a-5p_TTATAATACAACCTGATAAGTG4	-0,018422804	4,272038394	0,966323516	0,983601332	T1D vs Control
hsa-miR-99b-5p_CACCCGTAGAACCGACCTTGC4	0,005623353	7,880557566	0,970345849	0,984814475	T1D vs Control
hsa-miR-92a-3p_TGCACTTGTCCCGGCCTGT4	-0,013304527	5,28105363	0,971110767	0,984814475	T1D vs Control
hsa-miR-363-3p_AATTGCACGGTATCCATCTG4	-0,005995379	8,264741807	0,971343595	0,984814475	T1D vs Control
hsa-miR-423-3p_AAGCTCGGTCTGAGGCCCCTCAGT4	-0,008155377	7,286433576	0,972457957	0,984814475	T1D vs Control
hsa-miR-222-3p_AGCTACATCTGGCTACTGGG4	0,012074107	2,549442498	0,982759248	0,992422669	T1D vs Control
hsa-let-7b-5p_TGAGGTAGTAGGTTGTGTGGTT4	-0,001858265	12,77995871	0,98361283	0,992422669	T1D vs Control
hsa-let-7a-5p_GTGAGGTAGTAGGTTGTATAGTT4	-0,011975707	2,866169762	0,983866762	0,992422669	T1D vs Control
hsa-miR-451a_AACCGTTACCATTACTGAG4	-0,00343033	8,074719646	0,984951482	0,992422669	T1D vs Control
hsa-miR-92a-3p_TATTGCACTTGTCCCGGC4	0,003828229	6,608463448	0,988058539	0,994296283	T1D vs Control
hsa-miR-93-3p_ACTGCTGAGCTAGCACTTCCCGA4	0,005858593	3,371774968	0,991320854	0,996070013	T1D vs Control
hsa-miR-99b-5p_ACCCGTAGAACCGACCTTGCG4	-0,004912008	2,082938158	0,992778267	0,996070013	T1D vs Control
hsa-miR-25-3p_CATTGCACTTGTCTCGGTC4	0,002747194	5,77988375	0,993570465	0,996070013	T1D vs Control
hsa-miR-182-5p_TTTGGCAATGGTAGAACTCACA4	-0,001142358	6,943076472	0,99659235	0,997572099	T1D vs Control
hsa-miR-486-5p_ATCCTGTACTGAGCTGCCCCGAG4	0,000585584	7,990918242	0,997572099	0,997572099	T1D vs Control
hsa-miR-22-3p_AAGCTGCCAGTTGAAGAACTGT5	0,412059013	12,01293	3,54384E-06	0,002824441	T2D vs Control
hsa-miR-10a-5p_ACCCTGTAGATCCGAATTTGTG5	1,181992882	6,001615458	0,000184254	0,073425347	T2D vs Control
hsa-miR-424-3p_CAAAACGTGAGGCGCTGCT5	1,996769929	2,803316018	0,00034468	0,090072103	T2D vs Control
hsa-miR-197-3p_TTCACCACCTTCTCCACCCAGC5	-0,56985226	10,1606215	0,000452056	0,090072103	T2D vs Control
hsa-miR-139-5p_TCTACAGTGCACGTGTCTCCAG5	1,570320601	4,676823231	0,000658552	0,101831882	T2D vs Control
hsa-miR-652-3p_AATGGCGCCACTAGGGTTGT5	0,553365631	7,904624885	0,000766614	0,101831882	T2D vs Control
hsa-miR-99a-5p_AACCCGTAGATCCGATCTTGTG5	1,184409948	5,156061862	0,001606836	0,151235206	T2D vs Control
hsa-miR-197-3p_TTCACCACCTTCTCCACCCAG5	-0,575241329	8,762482756	0,001658957	0,151235206	T2D vs Control
hsa-let-7d-3p_CTATACGACCTGCTGCCTTTC5	-0,32938942	10,32598693	0,0017078	0,151235206	T2D vs Control
hsa-let-7d-3p_TATACGACCTGCTGCCTTTCT5	-0,410685415	8,488338468	0,001973701	0,15730397	T2D vs Control
hsa-miR-183-5p_ATGGCACTGGTAGAATTCACTGT5	1,747059884	2,224937851	0,002613461	0,17788963	T2D vs Control
hsa-miR-363-3p_AATTGCACGGTATCCATCTGT5	0,458060572	8,977144446	0,002965293	0,17788963	T2D vs Control
hsa-miR-122-5p_TGGAGTGTGACAATGGTGTTT5	0,800571781	13,55430102	0,003094405	0,17788963	T2D vs Control
hsa-miR-181a-5p_ACATTCAACGCTGTCGGTGA5	1,581434325	3,569893472	0,003366243	0,17788963	T2D vs Control
hsa-miR-122-5p_TGGAGTGTGACAATGGTGTTTG5	0,818353262	11,63439763	0,003426387	0,17788963	T2D vs Control
hsa-miR-92a-3p_TATTGCACTTGTCCCGGCCTGTT5	-0,365849865	10,41769689	0,003778441	0,17788963	T2D vs Control
hsa-miR-7-5p_TGGAAGACTAGTGATTTTGT5	1,605816528	3,91952121	0,003899719	0,17788963	T2D vs Control
hsa-miR-132-3p_TAACAGTCTACAGCCATGGTCG5	1,634807804	2,233528136	0,004017583	0,17788963	T2D vs Control
hsa-miR-10b-5p_TACCCTGTAGAACCGAATTTG5	-0,366474911	9,833049317	0,004480076	0,187314395	T2D vs Control
hsa-miR-16-5p_TAGCAGCACGTAAATATTGGC5	0,357337505	9,94541903	0,004829332	0,187314395	T2D vs Control
hsa-miR-361-3p_TCCCCCAGGTGTGATTCTGATT5	1,376846118	3,650889743	0,005022646	0,187314395	T2D vs Control
hsa-miR-29a-3p_TAGCACCATCTGAAATCGG5	0,700452408	7,213630267	0,005170535	0,187314395	T2D vs Control
hsa-miR-22-3p_AAGCTGCCAGTTGAAGAACTG5	0,500922063	9,134304454	0,005500841	0,188636979	T2D vs Control
hsa-miR-1306-5p_CCACCTCCCCTGCAAACGTC5	-1,27539216	4,129732962	0,006117922	0,188636979	T2D vs Control
hsa-miR-130b-3p_CAGTGCAATGATGAAAGGGCA5	1,216769014	4,137452675	0,00623796	0,188636979	T2D vs Control
hsa-miR-30c-5p_TGTAAACATCCTACACTCTC5	1,583410596	2,069916362	0,006301777	0,188636979	T2D vs Control
hsa-miR-4433b-5p_TGTCCCACCCCCACTCCTGTTT5	-0,911197099	8,596146134	0,00677911	0,188636979	T2D vs Control
hsa-miR-6803-3p_TCCCTCGCCTTCTCACCCTCA5	-0,898315955	6,298415012	0,006879336	0,188636979	T2D vs Control

hsa-miR-25-3p_CATTGCACTTGTCTCGGT5	1,371311445	3,623128964	0,007064778	0,188636979	T2D vs Control
hsa-miR-361-5p_TTATCAGAATCTCCAGGGGT5	1,143919305	4,747764862	0,007194315	0,188636979	T2D vs Control
hsa-miR-425-5p_ATGACACGATCACTCCCGTTGAGT5	-1,272426634	4,72212581	0,007337197	0,188636979	T2D vs Control
hsa-let-7d-3p_CTATACGACCTGCTGCCTTTCT5	-0,304827652	11,76128234	0,007856133	0,195666822	T2D vs Control
hsa-miR-10b-5p_ACCCTGTAGAACCGAATTTGTG5	0,852724267	5,829978228	0,008437533	0,200564541	T2D vs Control
hsa-miR-23b-3p_ATCACATTGCCAGGGATTAC5	1,428208542	2,694770844	0,008556078	0,200564541	T2D vs Control
hsa-miR-140-3p_ACCACAGGGTAGAACCACGGACA5	1,295108362	4,192134912	0,009041706	0,202054892	T2D vs Control
hsa-miR-27b-3p_TTCACAGTGGCTAAGTTCTG5	0,476842441	8,279294906	0,009132994	0,202054892	T2D vs Control
hsa-miR-192-5p_TGACCTATGAATTGACAGCCAGT5	1,403557342	3,322199511	0,009380215	0,202054892	T2D vs Control
hsa-miR-27b-3p_TTCACAGTGGCTAAGTTCTGCA5	1,416679098	3,596858077	0,009841419	0,202661113	T2D vs Control
hsa-miR-181a-5p_AACATTCAACGCTGTCGGTGAGT5	0,516193829	8,031521361	0,010169431	0,202661113	T2D vs Control
hsa-miR-25-3p_ATTGCACTTGTCTCGGTCTG5	1,329107928	3,200649251	0,010171198	0,202661113	T2D vs Control
hsa-miR-199a-3p_ACAGTAGTCTGCACATTGGT5	-1,507982968	1,952185074	0,010725798	0,205850388	T2D vs Control
hsa-miR-421_ATCAACAGACATTAATTGGGCG5	1,332535028	2,09434672	0,011074403	0,205850388	T2D vs Control
hsa-miR-423-5p_CTGAGGGGCAGAGAGCGAGACT5	1,41284743	2,475285897	0,011106106	0,205850388	T2D vs Control
hsa-miR-27b-3p_TTCACAGTGGCTAAGTTCT5	0,467934247	8,299608545	0,012266371	0,222188585	T2D vs Control
hsa-miR-30a-5p_GTAAACATCCTCGACTGGAAGCT5	1,297647779	4,1060576	0,012925091	0,228917726	T2D vs Control
hsa-miR-6803-3p_TCCCTCGCCTTCTCACCCTC5	-1,406470542	2,004774634	0,013362603	0,231521628	T2D vs Control
hsa-let-7c-5p_TGAGGTAGTAGGTTGTATGG5	0,910449768	3,04066672	0,013835619	0,234616776	T2D vs Control
hsa-miR-320a-3p_GAAAAGCTGGGTTGAGAGGGCGA5	1,312133436	3,456353792	0,0162374	0,264371844	T2D vs Control
hsa-miR-320a-3p_AAAAGCTGGGTTGAGAGGGCG5	0,301717796	10,00052492	0,016253727	0,264371844	T2D vs Control
hsa-miR-24-3p_TGGCTCAGTTCAGCAGGAACA5	0,273072813	9,988806411	0,017151551	0,273395726	T2D vs Control
hsa-miR-363-3p_ATTGCACGGTATCCATCTGT5	1,178665283	4,343538892	0,017857267	0,276430795	T2D vs Control
hsa-miR-4433b-5p_ATGTCCCACCCCCACTCCTGTT5	-0,915294314	8,226543503	0,018035635	0,276430795	T2D vs Control
hsa-miR-1306-5p_CCACCTCCCCTGCAAACGT5	-1,478468476	2,734536196	0,018518427	0,27847521	T2D vs Control
hsa-miR-361-5p_TTATCAGAATCTCCAGGGG5	1,302426056	2,056970291	0,020313754	0,297733945	T2D vs Control
hsa-miR-451a_CGTTACCATTACTGAGT5	1,152251595	4,012413349	0,020546257	0,297733945	T2D vs Control
hsa-miR-93-5p_CAAAGTGCTGTTCGTGCAGGT5	0,866645568	4,918359962	0,022927929	0,326313563	T2D vs Control
hsa-miR-339-3p_TGAGCGCCTCGACGACAGAGC5	1,170828817	3,99309945	0,023558784	0,329409665	T2D vs Control
hsa-miR-10a-5p_TACCCTGTAGATCCGAATTTGTG5	0,510597873	7,257876357	0,027168196	0,37053302	T2D vs Control
hsa-let-7g-5p_TGAGGTAGTAGTTTGTACA5	0,978004113	5,425002874	0,027498974	0,37053302	T2D vs Control
hsa-miR-451a_CCGTTACCATTACTGAGT5	1,108397646	4,189894806	0,027894581	0,37053302	T2D vs Control
hsa-miR-3173-5p_TGCCCTGCCTGTTTTCTCCTTT5	-0,956972589	4,766192857	0,029370855	0,378106309	T2D vs Control
hsa-miR-139-5p_TCTACAGTGCACGTGTCTCCAGT5	0,584084937	6,587018882	0,029687534	0,378106309	T2D vs Control
hsa-miR-22-3p_AGCTGCCAGTTGAAGAACTGT5	0,845305918	5,523006419	0,0299099	0,378106309	T2D vs Control
hsa-miR-1306-5p_CCACCTCCCCTGCAAACGTCC5	-1,037230969	4,76371819	0,030362364	0,378106309	T2D vs Control
hsa-miR-335-5p_TCAAGAGCAATAACGAAAAAT5	-0,67250761	6,715048961	0,031097098	0,378739666	T2D vs Control
hsa-miR-486-5p_GTACTGAGCTGCCCCGAG5	1,017818972	4,883397866	0,031363636	0,378739666	T2D vs Control
hsa-miR-183-5p_ATGGCACTGGTAGAATTCACTG5	1,237295338	2,888906189	0,033045072	0,390797345	T2D vs Control
hsa-miR-483-5p_AAGACGGGAGGAAAGAAGGGA5	1,192534393	4,456894738	0,03334281	0,390797345	T2D vs Control
hsa-miR-28-3p_CACTAGATTGTGAGCTCCTGGAG5	1,169767561	2,845963244	0,034691444	0,393594103	T2D vs Control
hsa-miR-21-5p_TAGCTTATCAGACTGATG5	1,181289348	1,940313304	0,035488503	0,393594103	T2D vs Control
hsa-miR-30e-5p_TGTAAACATCCTTGACTGG5	0,960382194	5,076073317	0,036594585	0,393594103	T2D vs Control
hsa-let-7a-5p_TGAGGTAGTAGGTTGTATA5	0,739287722	5,663865648	0,036993562	0,393594103	T2D vs Control
hsa-miR-10b-5p_TACCCTGTAGAACCGAATTTGTGT5	1,19265924	2,268128931	0,037382974	0,393594103	T2D vs Control
hsa-miR-183-5p_TATGGCACTGGTAGAATTCACT5	0,833261825	5,796604044	0,037568634	0,393594103	T2D vs Control
hsa-miR-30a-5p_TGTAAACATCCTCGACTGGAA5	0,433535273	7,169227794	0,037906627	0,393594103	T2D vs Control
hsa-miR-29a-3p_CTAGCACCATCTGAAATCGG5	1,116438679	3,050349736	0,03809586	0,393594103	T2D vs Control
hsa-miR-361-3p_TCCCCCAGGTGTGATTCTGATTT5	1,036574786	3,977031543	0,039460708	0,393594103	T2D vs Control
hsa-miR-181a-2-3p_ACCACTGACCGTTGACTGT5	1,16627825	2,531276889	0,039586448	0,393594103	T2D vs Control
hsa-miR-140-3p_TACCACAGGGTAGAACCACGGACA5	1,043950829	3,637619462	0,040240472	0,393594103	T2D vs Control
hsa-miR-29c-3p_TAGCACCATTTGAAATCGGTT5	1,012118051	3,850371057	0,040456382	0,393594103	T2D vs Control
hsa-miR-4433b-5p_ATGTCCCACCCCCACTCCTGTTT5	-0,968929288	5,620502964	0,040773325	0,393594103	T2D vs Control
hsa-miR-142-3p_TGTAGTGTTTCCTACTTTATGGA5	1,168826313	3,252758122	0,041138314	0,393594103	T2D vs Control
hsa-miR-29a-3p_TAGCACCATCTGAAATCGGT5	0,388836645	8,663780214	0,041394957	0,393594103	T2D vs Control
hsa-miR-4433b-5p_TGTCCCACCCCCACTCCTGTT5	-0,934145129	6,729375179	0,041482942	0,393594103	T2D vs Control
hsa-let-7c-5p_TGAGGTAGTAGGTTGTATGGTT5	0,407126605	7,467576739	0,042024144	0,394038151	T2D vs Control
hsa-miR-191-5p_CAACGGAATCCCAAAAGCAGCT5	-0,258624449	11,11779618	0,043836062	0,396621309	T2D vs Control
hsa-miR-150-5p_TCTCCCAACCCTTGTACCAGTG5	0,3140938	10,93881631	0,043910632	0,396621309	T2D vs Control
hsa-miR-16-2-3p_ACCAATATTACTGTGCTGCT5	0,929472256	4,606374691	0,044243924	0,396621309	T2D vs Control
hsa-miR-30a-5p_TGTAAACATCCTCGACTGGAAGCT5	0,252153829	8,778765877	0,044290209	0,396621309	T2D vs Control

hsa-miR-139-5p_TCTACAGTGCACGTGTCTCCA5	0,978571662	4,69390328	0,045258628	0,400790293	T2D vs Control
hsa-miR-181a-5p_AACATTCAACGCTGTCGGTGAGTT5	1,159216157	2,334562434	0,046117307	0,40390652	T2D vs Control
hsa-miR-363-3p_AATTGCACGGTATCCATCTG5	0,348821304	8,264741807	0,046896701	0,406268158	T2D vs Control
hsa-miR-4433b-5p_ATGTCCCACCCCCACTCCTGT5	-0,736970309	7,752662063	0,048790293	0,417562341	T2D vs Control
hsa-miR-145-5p_GTCCAGTTTTCCCAGGAATCCC5	1,170909421	3,12605684	0,049994146	0,417562341	T2D vs Control
hsa-miR-130a-3p_CAGTGCAATGTTAAAAGGGCA5	0,916948665	4,214432691	0,050440375	0,417562341	T2D vs Control
hsa-miR-320a-3p_AAAGCTGGGTTGAGAGGGCGA5	0,968788327	4,98279454	0,050918986	0,417562341	T2D vs Control
hsa-miR-122-5p_GGAGTGTGACAATGGTGTTT5	0,694908656	7,666405464	0,051038054	0,417562341	T2D vs Control
hsa-miR-4732-5p_TGTAGAGCAGGGAGCAGGAAG5	-1,142687559	3,155376397	0,051343927	0,417562341	T2D vs Control
hsa-miR-4433b-5p_TGTCCCACCCCCACTCCTGT5	-0,975591591	4,771975689	0,053719249	0,430773959	T2D vs Control
hsa-miR-30d-5p_TGTAAACATCCCCGACTGGAAGCT5	-0,245183632	13,20515822	0,054198107	0,430773959	T2D vs Control
hsa-miR-4433b-5p_TATGTCCCACCCCCACTCCTG5	-1,260323317	3,068037559	0,054589924	0,430773959	T2D vs Control
hsa-miR-329-3p_ACACACCTGGTTAACCTCTTTT5	-1,159588146	2,079797897	0,056538444	0,435976253	T2D vs Control
hsa-miR-223-5p_CGTGTATTTGACAAGCTGAGTTGG5	-1,129085418	2,566442122	0,057392965	0,435976253	T2D vs Control
hsa-miR-155-5p_TTAATGCTAATCGTGATAGGGGTT5	1,125477173	2,993899159	0,058060086	0,435976253	T2D vs Control
hsa-miR-181a-2-3p_ACCACTGACCGTTGACTGTAC5	1,037721266	3,544826687	0,058774529	0,435976253	T2D vs Control
hsa-miR-23a-5p_GGGGTTCCTGGGGATGGGATT5	1,045245267	3,585240207	0,058830657	0,435976253	T2D vs Control
hsa-miR-128-3p_TCACAGTGAACCGGTCTCTTT5	-0,258438357	8,228392837	0,059004247	0,435976253	T2D vs Control
hsa-miR-148b-3p_TCAGTGCATCACAGAACTTTG5	0,845326143	4,882892663	0,059078338	0,435976253	T2D vs Control
hsa-let-7e-5p_TGAGGTAGGAGGTTGTATAGT5	0,898434852	4,645402812	0,061581052	0,444891704	T2D vs Control
hsa-miR-93-5p_CAAAGTGCTGTTCGTGCAGGTA5	0,747673709	5,442816943	0,062074354	0,444891704	T2D vs Control
hsa-miR-145-5p_GTCCAGTTTTCCCAGGAATCCCT5	-1,114663264	2,146327793	0,062086153	0,444891704	T2D vs Control
hsa-miR-122-5p_GGAGTGTGACAATGGTGTTTG5	0,824100472	5,615777051	0,062519286	0,444891704	T2D vs Control
hsa-miR-21-5p_TAGCTTATCAGACTGATGTTGAC5	-0,434719622	10,44971368	0,063148668	0,445393705	T2D vs Control
hsa-miR-22-3p_AAGCTGCCAGTTGAAGAACT5	0,228296541	10,21399499	0,064484477	0,450825687	T2D vs Control
hsa-miR-155-5p_TTAATGCTAATCGTGATAGGGGT5	0,942861663	3,972069578	0,066043804	0,453769003	T2D vs Control
hsa-miR-21-5p_AGCTTATCAGACTGATGTTG5	0,708480892	5,031654467	0,066044171	0,453769003	T2D vs Control
hsa-miR-30c-5p_TGTAAACATCCTACACTCTCAGC5	0,514935483	6,545485648	0,068449015	0,466272351	T2D vs Control
hsa-miR-423-3p_AGCTCGGTCTGAGGCCCCT5	0,581447377	6,038348398	0,071182215	0,480781569	T2D vs Control
hsa-miR-99a-5p_AACCCGTAGATCCGATCTTGT5	0,306783433	8,529135227	0,072014921	0,482318418	T2D vs Control
hsa-miR-505-3p_CGTCAACACTTGCTGGTTTCCT5	0,973296101	3,899410508	0,073271418	0,486644333	T2D vs Control
hsa-miR-505-3p_CGTCAACACTTGCTGGTTTCCTCT5	1,080390725	2,947139365	0,074527597	0,490617446	T2D vs Control
hsa-miR-451a_AAACCGTTACCATTACTGAGTTTAGT5	0,791843832	4,187049919	0,075100789	0,490617446	T2D vs Control
hsa-miR-423-5p_GAGGGGCAGAGAGCGAGA5	-1,022267807	1,916584014	0,075819171	0,491283568	T2D vs Control
hsa-miR-589-5p_TGAGAACCACGTCTGCTCTGA5	1,007464102	2,896824842	0,076896102	0,494243495	T2D vs Control
hsa-miR-451a_ACCGTTACCATTACTGAGT5	0,43962776	7,452815255	0,079481658	0,497913509	T2D vs Control
hsa-miR-30c-5p_TGTAAACATCCTACACTCTCA5	0,378252324	7,955436132	0,081229078	0,497913509	T2D vs Control
hsa-miR-181a-5p_AACATTCAACGCTGTCGGTGA5	0,187133313	9,980124889	0,081414455	0,497913509	T2D vs Control
hsa-miR-150-5p_CTCCCAACCCTTGTACCAGTG5	0,859538149	4,313974888	0,082579549	0,497913509	T2D vs Control
hsa-miR-483-5p_AAGACGGGAGGAAAGAAGGGAGT5	1,050525295	3,821071217	0,08286394	0,497913509	T2D vs Control
hsa-miR-423-5p_GAGGGGCAGAGAGCGAGACTT5	0,713861056	5,367382166	0,084581189	0,497913509	T2D vs Control
hsa-miR-182-5p_TTTGGCAATGGTAGAACTCACACTG5	1,00668831	3,598930548	0,087463175	0,497913509	T2D vs Control
hsa-miR-93-3p_ACTGCTGAGCTAGCACTTCCCGA5	0,958362139	3,371774968	0,087592418	0,497913509	T2D vs Control
hsa-miR-4732-5p_TGTAGAGCAGGGAGCAGGAAGCT5	0,55535148	6,276959061	0,088527629	0,497913509	T2D vs Control
hsa-miR-144-5p_GATATCATCATATACTGTAAGTT5	-0,998115354	2,459029553	0,089066928	0,497913509	T2D vs Control
hsa-miR-320a-3p_GAAAAGCTGGGTTGAGAGGGCG5	0,551718044	6,114738168	0,089121112	0,497913509	T2D vs Control
hsa-miR-223-3p_TGTCAGTTTGTCAAATACCC5	0,803002882	4,516322797	0,089320684	0,497913509	T2D vs Control
hsa-miR-125a-5p_TCCCTGAGACCCTTTAACCTGTGA5	-0,35458225	8,237628665	0,08988782	0,497913509	T2D vs Control
hsa-miR-142-5p_CCCATAAAGTAGAAAGCA5	0,82377488	2,550307907	0,090246426	0,497913509	T2D vs Control
hsa-miR-329-3p_AACACACCTGGTTAACCTCTTT5	-0,975490838	3,759288959	0,090616174	0,497913509	T2D vs Control
hsa-miR-30e-3p_CTTTCAGTCGGATGTTTACAG5	0,958968885	2,31966353	0,091113717	0,497913509	T2D vs Control
hsa-miR-100-5p_AACCCGTAGATCCGAACT5	-0,930217091	1,926738636	0,091469597	0,497913509	T2D vs Control
hsa-miR-379-5p_TGGTAGACTATGGAACGTAGG5	-1,0319535	3,839701767	0,091733333	0,497913509	T2D vs Control
hsa-miR-423-5p_AGGGGCAGAGAGCGAGACT5	0,982852224	3,04923014	0,091817246	0,497913509	T2D vs Control
hsa-miR-375-3p_TTTGTTCGTTCGGCTCGCGTGA5	0,408279521	10,08392589	0,091946752	0,497913509	T2D vs Control
hsa-let-7f-5p_TGAGGTAGTAGATTG5	0,911719267	4,164688749	0,092068496	0,497913509	T2D vs Control
hsa-miR-126-3p_TCGTACCGTGAGTAATAATGCG5	0,23565759	11,19647801	0,092408645	0,497913509	T2D vs Control
hsa-miR-181a-5p_AACATTCAACGCTGTCGG5	0,880944899	2,192700831	0,092449164	0,497913509	T2D vs Control
hsa-miR-150-5p_GTCTCCCAACCCTTGTACCAGT5	0,93951421	2,340151294	0,092587544	0,497913509	T2D vs Control
hsa-miR-328-3p_CTGGCCCTCTCTGCCCTTCCGT5	-0,37581907	7,228551867	0,093509308	0,497913509	T2D vs Control
hsa-miR-542-3p_TGTGACAGATTGATAACTGA5	0,956627853	1,954666629	0,093710196	0,497913509	T2D vs Control

hsa-miR-361-3p_TCCCCCAGGTGTGATTCTGA5	0,896171572	3,113214743	0,094935936	0,498583343	T2D vs Control
hsa-let-7a-5p_TGAGGTAGTAGGTTGTATAGTTT5	-0,365867164	9,423297293	0,095087413	0,498583343	T2D vs Control
hsa-miR-191-5p_AACGGAATCCCAAAAGCAGCTG5	0,55163411	5,747755673	0,099286108	0,515050739	T2D vs Control
hsa-miR-19a-3p_TGTGCAAATCTATGCAAAACTGA5	0,919396974	2,326203362	0,099520469	0,515050739	T2D vs Control
hsa-miR-140-5p_CAGTGGTTTTACCCTATGGTAG5	0,840330118	3,871751643	0,100754048	0,517947169	T2D vs Control
hsa-miR-328-3p_CTGGCCCTCTCTGCCCTTCCG5	0,809536103	3,770890003	0,101573354	0,517947169	T2D vs Control
hsa-miR-16-5p_AGCAGCACGTAAATATTGG5	0,886123052	3,167012316	0,102029743	0,517947169	T2D vs Control
hsa-miR-22-3p_AGCTGCCAGTTGAAGAACT5	0,962327186	2,67043052	0,102737647	0,518239904	T2D vs Control
hsa-miR-451a_ACCGTTACCATTACTGAGTT5	0,909987901	2,004581894	0,104351532	0,52036348	T2D vs Control
hsa-miR-320b_AAAAGCTGGGTTGAGAGGGCAA5	0,849425156	1,963551094	0,104464438	0,52036348	T2D vs Control
hsa-let-7d-3p_CTATACGACCTGCTGCCTTT5	-0,213555443	10,24742095	0,106644167	0,527424388	T2D vs Control
hsa-miR-122-5p_TGGAGTGTGACAATGGTGTTTGT5	0,510952001	8,198260953	0,10851138	0,527424388	T2D vs Control
hsa-miR-363-3p_AATTGCACGGTATCCATCTGTA5	0,828709963	3,912624597	0,109038397	0,527424388	T2D vs Control
hsa-miR-1301-3p_TTGCAGCTGCCTGGGAGTGACTTC5	-0,95981991	2,697320641	0,109306564	0,527424388	T2D vs Control
hsa-let-7b-5p_TGAGGTAGTAGGTTGTGTGGTTT5	-0,199649079	11,28589984	0,110906112	0,527424388	T2D vs Control
hsa-miR-363-3p_ATTGCACGGTATCCATCTG5	0,902970723	2,060471037	0,111930262	0,527424388	T2D vs Control
hsa-miR-361-5p_TTATCAGAATCTCCAGGGGTA5	0,881457793	3,298750595	0,112262595	0,527424388	T2D vs Control
hsa-let-7d-3p_CTATACGACCTGCTGCCTT5	-0,596666723	4,847124715	0,112419705	0,527424388	T2D vs Control
hsa-miR-486-5p_CCTGTACTGAGCTGCCCCGAG5	-0,245318761	11,29662351	0,113077652	0,527424388	T2D vs Control
hsa-miR-451a_AAACCGTTACCATTACTGAGTTT5	0,261914574	8,99265157	0,113810254	0,527424388	T2D vs Control
hsa-miR-140-3p_TACCACAGGGTAGAACCACGGA5	0,335709615	7,894124989	0,113843157	0,527424388	T2D vs Control
hsa-miR-30d-5p_GTAAACATCCCCGACTGGAAGC5	-0,703983707	4,874885211	0,114333524	0,527424388	T2D vs Control
hsa-miR-361-5p_TTATCAGAATCTCCAGGGGTAC5	0,404330783	6,634693775	0,115737807	0,527424388	T2D vs Control
hsa-miR-21-5p_TAGCTTATCAGACTGATGTTGACT5	-0,728640044	5,943834972	0,116296482	0,527424388	T2D vs Control
hsa-miR-369-3p_AATAATACATGGTTGATCTTT5	-0,899048729	4,326255371	0,116617829	0,527424388	T2D vs Control
hsa-miR-502-3p_AATGCACCTGGGCAAGGATTCA5	0,662994496	4,709212787	0,116758045	0,527424388	T2D vs Control
hsa-miR-222-3p_AGCTACATCTGGCTACTGGGT5	0,315893586	7,595221992	0,11713189	0,527424388	T2D vs Control
hsa-miR-1-3p_TGGAATGTAAAGAAGTATGTAT5	-0,817221197	5,995923161	0,119235168	0,5328674	T2D vs Control
hsa-miR-486-3p_CGGGGCAGCTCAGTACAGGA5	0,486925713	6,539637794	0,119677873	0,5328674	T2D vs Control
hsa-miR-140-3p_TACCACAGGGTAGAACCACGGAC5	0,85247557	3,947173881	0,123031146	0,544754576	T2D vs Control
hsa-miR-106b-3p_CCGCACTGTGGGTACTTGCTG5	0,709460402	4,162035136	0,126975361	0,559112503	T2D vs Control
hsa-let-7c-5p_TGAGGTAGTAGGTTGTATGGT5	0,566703496	4,585920454	0,129812731	0,568465639	T2D vs Control
hsa-miR-29c-3p_TAGCACCATTTGAAATCGGT5	0,806265814	4,339459305	0,130621034	0,568879587	T2D vs Control
hsa-miR-6803-3p_TCCCTCGCCTTCTCACCCTCAGT5	-0,921502709	2,14031641	0,133847692	0,575334085	T2D vs Control
hsa-miR-1294_TGTGAGGTTGGCATTGTTGT5	0,853694515	2,481086687	0,134095924	0,575334085	T2D vs Control
hsa-miR-128-3p_TCACAGTGAACCGGTCTCT5	0,278464446	7,683472613	0,13494588	0,575334085	T2D vs Control
hsa-miR-1306-5p_CCACCTCCCCTGCAAACGTCCA5	-0,897198977	3,283279778	0,134990557	0,575334085	T2D vs Control
hsa-miR-654-3p_TATGTCTGCTGACCATCACC5	-0,917508288	2,711988254	0,135826776	0,575818833	T2D vs Control
hsa-miR-505-3p_CGTCAACACTTGCTGGTTT5	0,819744606	3,249239604	0,138506554	0,57856814	T2D vs Control
hsa-miR-323a-3p_CACATTACACGGTCGACCTCT5	-0,869734536	3,585538292	0,138842779	0,57856814	T2D vs Control
hsa-miR-197-3p_TTCACCACCTTCTCCACCCA5	-0,760867646	3,958344822	0,139138283	0,57856814	T2D vs Control
hsa-miR-126-5p_ATTATTACTTTTGGTACGCGCT5	0,83002073	2,122638684	0,140024495	0,57856814	T2D vs Control
hsa-miR-425-5p_ATGACACGATCACTCCCGTTG5	0,846974338	2,398751928	0,140104957	0,57856814	T2D vs Control
hsa-miR-30d-5p_GTAAACATCCCCGACTGGAAG5	0,4901414	5,727486807	0,141558066	0,58155556	T2D vs Control
hsa-miR-25-3p_CATTGCACTTGTCTCGGTCTGA5	0,218764836	11,99390817	0,142308119	0,581638824	T2D vs Control
hsa-miR-140-3p_ACCACAGGGTAGAACCACGG5	0,775938979	3,491351538	0,143493385	0,583490958	T2D vs Control
hsa-miR-22-3p_AAGCTGCCAGTTGAAGAA5	0,709066254	4,931866064	0,146333759	0,58824157	T2D vs Control
hsa-miR-130b-5p_ACTCTTTCCCTGTTGCACTACT5	-0,528611709	5,742085569	0,146454247	0,58824157	T2D vs Control
hsa-miR-423-3p_AAGCTCGGTCTGAGGCCCCTCAG5	0,769701508	3,402313099	0,146875875	0,58824157	T2D vs Control
hsa-miR-2110_TTGGGGAAACGGCCGCTGAGTG5	0,880425784	2,932814217	0,148021801	0,589866879	T2D vs Control
hsa-let-7g-5p_TGAGGTAGTAGTTTGTACAGT5	0,126090291	10,7052364	0,14883455	0,590154908	T2D vs Control
hsa-miR-451a_AAACCGTTACCATTACTG5	0,753364222	2,491678877	0,153393519	0,600530166	T2D vs Control
hsa-miR-423-5p_AGGGGCAGAGAGCGAGACTTTT5	0,713125218	4,864713364	0,153449435	0,600530166	T2D vs Control
hsa-miR-320a-3p_AAAAGCTGGGTTGAGAGGGCGA5	0,155462646	10,74836293	0,154002097	0,600530166	T2D vs Control
hsa-miR-193a-5p_TGGGTCTTTGCGGGCGAGA5	-0,844780797	3,488597754	0,155971233	0,600530166	T2D vs Control
hsa-miR-16-5p_TAGCAGCACGTAAATATTGGCGT5	0,272791601	7,90073048	0,156086494	0,600530166	T2D vs Control
hsa-miR-223-3p_TCAGTTTGTCAAATACCCCAA5	0,787365597	2,060154898	0,156307999	0,600530166	T2D vs Control
hsa-miR-484_TCAGGCTCAGTCCCCTCCCGA5	0,234901159	10,80053241	0,157351484	0,600530166	T2D vs Control
hsa-miR-1180-3p_TTTCCGGCTCGCGTGGGTGTGT5	0,766273677	2,874816557	0,157479052	0,600530166	T2D vs Control
hsa-miR-382-5p_GAAGTTGTTCGTGGTGGATTCG5	-0,493126943	7,607601722	0,162035713	0,61496411	T2D vs Control
hsa-miR-25-3p_CATTGCACTTGTCTCGGTCT5	0,209699994	10,26006574	0,163210161	0,616485774	T2D vs Control

hsa-miR-194-5p_TGTAACAGCAACTCCATGTGG5	0,352172709	6,660853419	0,165682034	0,616731387	T2D vs Control
hsa-miR-28-3p_CACTAGATTGTGAGCTCCTGGA5	0,383006967	6,806864009	0,16570786	0,616731387	T2D vs Control
hsa-let-7i-5p_TGAGGTAGTAGTTTGTGCT5	0,301757519	7,615350025	0,166009558	0,616731387	T2D vs Control
hsa-miR-3615_TCTCTCGGCTCCTCGCGGCTC5	0,72500307	2,193732344	0,16637045	0,616731387	T2D vs Control
hsa-miR-425-5p_AATGACACGATCACTCCCGTTGAGT5	-0,329483645	8,386717544	0,168668994	0,620681224	T2D vs Control
hsa-miR-26b-5p_TTCAAGTAATTCAGGATAGGTT5	-0,247162563	9,842825606	0,169908259	0,620681224	T2D vs Control
hsa-miR-19b-3p_TGTGCAAATCCATGCAAAACTGA5	0,665504683	4,916784309	0,170098386	0,620681224	T2D vs Control
hsa-let-7i-5p_GAGGTAGTAGTTTGTGCTGT5	0,653759317	4,789553904	0,170735042	0,620681224	T2D vs Control
hsa-miR-30b-5p_TGTAAACATCCTACACTCAGCT5	0,362598876	6,722043115	0,172107375	0,620681224	T2D vs Control
hsa-miR-7-5p_TGGAAGACTAGTGATTTTGTTGT5	0,543442913	6,176558156	0,172259381	0,620681224	T2D vs Control
hsa-miR-32-5p_TATTGCACATTACTAAGTTG5	0,73150487	3,984130302	0,172887367	0,620681224	T2D vs Control
hsa-miR-16-5p_TAGCAGCACGTAAATATTGG5	0,186057977	10,97240393	0,174577963	0,623940074	T2D vs Control
hsa-miR-222-3p_AGCTACATCTGGCTACTGGGTCTC5	-0,547227883	6,101293098	0,176081014	0,626502537	T2D vs Control
hsa-miR-146b-5p_TGAGAACTGAATTCCATAGGCTG5	0,499899929	5,765289472	0,177664277	0,629195012	T2D vs Control
hsa-miR-484_CAGGCTCAGTCCCCTCCCGA5	0,773567854	3,768443499	0,179196429	0,629195012	T2D vs Control
hsa-miR-92a-3p_TATTGCACTTGTCCCGGCCTG5	-0,192146164	13,95782504	0,179445644	0,629195012	T2D vs Control
hsa-miR-423-5p_AGGGGCAGAGAGCGAGACTTT5	-0,481797007	5,332499304	0,179995562	0,629195012	T2D vs Control
hsa-miR-92b-3p_TATTGCACTCGTCCCGGCC5	0,724905596	3,837730088	0,181122963	0,629552034	T2D vs Control
hsa-miR-486-5p_ATCCTGTACTGAGCTGCCCCG5	-0,381800013	6,350028468	0,1816775	0,629552034	T2D vs Control
hsa-let-7d-5p_AGAGGTAGTAGGTTGCATAGTT5	-0,273111719	9,075101282	0,182639907	0,630147211	T2D vs Control
hsa-miR-126-3p_CGTACCGTGAGTAATAATGC5	0,588456147	4,46578231	0,184347261	0,63329641	T2D vs Control
hsa-miR-629-5p_TGGGTTTACGTTGGGAGAA5	0,800727745	2,515869067	0,186012204	0,636273505	T2D vs Control
hsa-miR-30d-5p_TGTAAACATCCCCGACTGG5	0,183067908	8,045778015	0,189314774	0,644802884	T2D vs Control
hsa-miR-101-3p_GTACAGTACTGTGATAACTGA5	-0,204162891	8,248925167	0,191640726	0,649524849	T2D vs Control
hsa-let-7f-5p_TGAGGTAGTAGATTGTAT5	0,799328159	3,174737293	0,192331072	0,649524849	T2D vs Control
hsa-miR-126-3p_TCGTACCGTGAGTAATAATG5	0,410819289	5,867396204	0,193413533	0,650424414	T2D vs Control
hsa-miR-24-3p_GCTCAGTTCAGCAGGAACAG5	0,705539287	2,25101941	0,194429267	0,65075262	T2D vs Control
hsa-miR-374a-5p_TTATAATACAACCTGATAAGT5	-0,745668826	2,487762486	0,195144136	0,65075262	T2D vs Control
hsa-miR-181a-5p_AACATTCAACGCTGTCGGTGAG5	0,240484676	7,366086853	0,197310008	0,655233651	T2D vs Control
hsa-miR-16-5p_TAGCAGCACGTAAATATTG5	0,450139295	5,566162995	0,200016832	0,658887019	T2D vs Control
hsa-miR-584-5p_TTATGGTTTGCCTGGGACT5	0,584370512	4,595669388	0,200500447	0,658887019	T2D vs Control
hsa-miR-199a-3p_ACAGTAGTCTGCACATTGGTTA5	1,139754194	4,827856003	0,200986216	0,658887019	T2D vs Control
hsa-miR-10a-5p_ACCCTGTAGATCCGAATTTG5	-0,409319052	5,718851669	0,201716979	0,658887019	T2D vs Control
hsa-miR-2110_TTGGGGAAACGGCCGCTGAGT5	0,42517075	5,974874685	0,203706827	0,659612968	T2D vs Control
hsa-miR-146a-5p_TGAGAACTGAATTCCATGGGT5	0,175261397	8,67354952	0,203880591	0,659612968	T2D vs Control
hsa-miR-148a-3p_CAGTGCACTACAGAACTTTGT5	0,743633626	2,126116505	0,206623635	0,659612968	T2D vs Control
hsa-miR-425-5p_ATGACACGATCACTCCCGTTGA5	0,584808997	4,729605379	0,206804183	0,659612968	T2D vs Control
hsa-miR-16-5p_AGCAGCACGTAAATATTGGC5	0,691200905	2,995434135	0,207689297	0,659612968	T2D vs Control
hsa-miR-361-3p_TCCCCCAGGTGTGATTCTGATTTG5	0,457189894	5,241633262	0,209382278	0,659612968	T2D vs Control
hsa-miR-24-3p_TGGCTCAGTTCAGCAGGAACAG5	0,138967849	12,76835022	0,209513558	0,659612968	T2D vs Control
hsa-miR-25-3p_CATTGCACTTGTCTCGGTC5	0,44590954	5,77988375	0,211024133	0,659612968	T2D vs Control
hsa-miR-148a-3p_TCAGTGCACTACAGAACTTTG5	0,26065501	7,287277231	0,211873724	0,659612968	T2D vs Control
hsa-miR-375-3p_TTTGTTCGTTCGGCTCGCGTG5	0,434911567	6,919662337	0,212501754	0,659612968	T2D vs Control
hsa-miR-101-3p_GTACAGTACTGTGATAACTGAA5	0,709396213	3,655178095	0,213832414	0,659612968	T2D vs Control
hsa-miR-10a-5p_TACCCTGTAGATCCGAATTTG5	-0,188654824	8,855203262	0,214200257	0,659612968	T2D vs Control
hsa-miR-486-5p_CCTGTACTGAGCTGCCCCGA5	-0,19301001	11,08007536	0,214847597	0,659612968	T2D vs Control
hsa-miR-99b-5p_CACCCGTAGAACCGACCTTGCG5	-0,200351815	9,378733966	0,214902425	0,659612968	T2D vs Control
hsa-miR-144-5p_GGATATCATCATATACTGTAA5	0,701114306	3,901874288	0,215041397	0,659612968	T2D vs Control
hsa-miR-423-5p_TGAGGGGCAGAGAGCGAGACT5	0,148956999	13,29479943	0,215181144	0,659612968	T2D vs Control
hsa-miR-30a-5p_TGTAAACATCCTCGACTGGAAGC5	0,21866164	7,798673355	0,217202669	0,663011551	T2D vs Control
hsa-miR-451a_AACCGTTACCATTACTGAGTT5	0,383374261	6,297926904	0,218195823	0,663011551	T2D vs Control
hsa-miR-92a-3p_ATTGCACTTGTCCCGGCCTGT5	-0,134880453	12,31240602	0,219789929	0,663011551	T2D vs Control
hsa-let-7f-5p_TGAGGTAGTAGATTGTATAGTTT5	-0,321828836	8,114676232	0,220417754	0,663011551	T2D vs Control
hsa-miR-32-5p_TATTGCACATTACTAAGTTGC5	0,701061013	2,00688677	0,220585271	0,663011551	T2D vs Control
hsa-let-7g-5p_GAGGTAGTAGTTTGTACAGT5	0,708226758	3,423010066	0,221917786	0,663011551	T2D vs Control
hsa-miR-501-3p_AATGCACCCGGGCAAGGATT5	0,344322186	6,44960483	0,222527369	0,663011551	T2D vs Control
hsa-miR-126-5p_CATTATTACTTTTGGTACGCG5	-0,297170161	6,678225687	0,223815218	0,663011551	T2D vs Control
hsa-miR-487b-3p_TCGTACAGGGTCATCCACTTT5	0,753278003	2,776617041	0,224539026	0,663011551	T2D vs Control
hsa-miR-98-5p_TGAGGTAGTAAGTTGTATTG5	0,590211692	4,611342852	0,22529702	0,663011551	T2D vs Control
hsa-miR-92a-3p_TATTGCACTTGTCCCGGCCTGT5	-0,117845181	17,23184948	0,226377848	0,663011551	T2D vs Control
hsa-let-7b-5p_GAGGTAGTAGGTTGTGTGGTT5	0,359989658	6,209954592	0,226909568	0,663011551	T2D vs Control

hsa-miR-194-5p_TGTAACAGCAACTCCATGTGGAA5	0,663545386	2,659899918	0,227104333	0,663011551	T2D vs Control
hsa-miR-133a-3p_TTGGTCCCCTTCAACCAGCTGT5	-0,746376324	2,527387136	0,228230299	0,663866963	T2D vs Control
hsa-miR-192-5p_TGACCTATGAATTGACAGCCAG5	0,67240765	3,652730706	0,23006521	0,664886462	T2D vs Control
hsa-let-7d-5p_AGAGGTAGTAGGTTGCATAGTTT5	-0,579152054	5,420694347	0,230662172	0,664886462	T2D vs Control
hsa-miR-451a_AAACCGTTACCATTACTGAGT5	0,17976361	16,51409079	0,2310835	0,664886462	T2D vs Control
hsa-miR-182-5p_TTTGGCAATGGTAGAACTCACACT5	-0,273440708	7,651119737	0,238969397	0,681545402	T2D vs Control
hsa-miR-93-5p_AAAGTGCTGTTCGTGCAGGTAG5	0,616464572	3,611226251	0,239535865	0,681545402	T2D vs Control
hsa-miR-16-2-3p_CCAATATTACTGTGCTGCTT5	0,48354648	4,446564737	0,240781612	0,681545402	T2D vs Control
hsa-miR-425-5p_AATGACACGATCACTCCCG5	0,563234679	4,547943713	0,242174669	0,681545402	T2D vs Control
hsa-miR-10a-5p_TACCCTGTAGATCCGAATTTGTGT5	0,661232484	2,404757373	0,242417188	0,681545402	T2D vs Control
hsa-miR-122-5p_GAGTGTGACAATGGTGTTT5	0,731240717	2,929528654	0,243693221	0,681545402	T2D vs Control
hsa-miR-30d-5p_TGTAAACATCCCCGACTGGAAGC5	-0,119395568	11,59455452	0,243754505	0,681545402	T2D vs Control
hsa-miR-182-5p_TTTGGCAATGGTAGAACT5	0,677261651	3,421424723	0,244033647	0,681545402	T2D vs Control
hsa-miR-222-3p_AGCTACATCTGGCTACTGGGTCTCT5	-0,299401461	7,967322162	0,246900102	0,681545402	T2D vs Control
hsa-miR-4685-3p_TCTCCCTTCCTGCCCTGGCT5	-0,533534231	4,862788515	0,246966549	0,681545402	T2D vs Control
hsa-miR-16-5p_TAGCAGCACGTAAATATTGGCG5	0,132107406	14,03055319	0,247686491	0,681545402	T2D vs Control
hsa-miR-423-5p_TGAGGGGCAGAGAGCGAGACTTTT5	-0,128193545	11,39034963	0,248562727	0,681545402	T2D vs Control
hsa-miR-103a-3p_AGCAGCATTGTACAGGGCTATGA5	-0,253751403	8,631709381	0,248676088	0,681545402	T2D vs Control
hsa-let-7i-5p_TGAGGTAGTAGTTTGTGCTGT5	0,133070965	11,39282357	0,24884531	0,681545402	T2D vs Control
hsa-miR-222-3p_AGCTACATCTGGCTACTGGGTC5	0,59658522	3,362212617	0,25176379	0,685210734	T2D vs Control
hsa-miR-423-5p_TGAGGGGCAGAGAGCGAGACTT5	0,109625541	12,19565674	0,251903068	0,685210734	T2D vs Control
hsa-miR-423-3p_AGCTCGGTCTGAGGCCCCTCAG5	0,143267267	8,372128456	0,254065621	0,6864908	T2D vs Control
hsa-miR-1228-3p_TCACACCTGCCTCGCCCCCCA5	-0,71695179	1,951009842	0,255387985	0,6864908	T2D vs Control
hsa-miR-423-3p_AGCTCGGTCTGAGGCCCC5	0,613767076	3,540860079	0,256137141	0,6864908	T2D vs Control
hsa-miR-192-5p_TGACCTATGAATTGACAGCCA5	0,553398977	4,726203979	0,256251918	0,6864908	T2D vs Control
hsa-miR-24-3p_GGCTCAGTTCAGCAGGAACAG5	0,267937068	6,812383366	0,256680374	0,6864908	T2D vs Control
hsa-miR-451a_ACCGTTACCATTACTGAG5	0,583384962	3,857793403	0,259512772	0,690073211	T2D vs Control
hsa-miR-342-3p_TCTCACACAGAAATCGCACCCGT5	0,219020819	8,706959622	0,261751154	0,690073211	T2D vs Control
hsa-miR-25-3p_ATTGCACTTGTCTCGGTCT5	0,558440858	3,620590845	0,263107689	0,690073211	T2D vs Control
hsa-miR-451a_AAACCGTTACCATTACTGA5	0,203800435	8,929457213	0,263429722	0,690073211	T2D vs Control
hsa-miR-192-5p_CTGACCTATGAATTGACAGC5	0,651503233	1,977431527	0,26372947	0,690073211	T2D vs Control
hsa-miR-223-5p_CGTGTATTTGACAAGCTGAGTTG5	-0,389140392	6,07891061	0,265114449	0,690073211	T2D vs Control
hsa-miR-23b-3p_ATCACATTGCCAGGGATTACCA5	0,335037962	6,460852955	0,26593005	0,690073211	T2D vs Control
hsa-miR-451a_AAACCGTTACCATTACTGAGTTTAG5	0,628153707	2,164703683	0,266162022	0,690073211	T2D vs Control
hsa-miR-451a_AAACCGTTACCATTACTGAGTT5	0,169739766	12,14012514	0,266474544	0,690073211	T2D vs Control
hsa-miR-7-5p_TGGAAGACTAGTGATTTTGTTG5	0,53819895	3,890826404	0,268051436	0,690073211	T2D vs Control
hsa-miR-92a-3p_CACTTGTCCCGGCCTGT5	0,579124323	4,072780053	0,26847377	0,690073211	T2D vs Control
hsa-miR-486-5p_TCCTGTACTGAGCTGCCCCG5	-0,180776928	14,12822837	0,269768058	0,690073211	T2D vs Control
hsa-let-7d-5p_AGAGGTAGTAGGTTGCATAGT5	-0,268129317	7,455921597	0,270680197	0,690073211	T2D vs Control
hsa-miR-425-5p_AATGACACGATCACTCCCGTT5	0,231497493	8,024407696	0,270807706	0,690073211	T2D vs Control
hsa-miR-486-5p_TCCTGTACTGAGCTGCCCCGAG5	-0,150169749	17,24661108	0,271007422	0,690073211	T2D vs Control
hsa-miR-125a-5p_TCCCTGAGACCCTTTAACCTG5	0,201221343	8,290201937	0,27382329	0,691851261	T2D vs Control
hsa-miR-340-5p_TTATAAAGCAATGAGACTGAT5	-0,625930785	1,968507467	0,274536047	0,691851261	T2D vs Control
hsa-miR-501-3p_AATGCACCCGGGCAAGGATTCT5	-0,162835132	7,911300847	0,274881744	0,691851261	T2D vs Control
hsa-miR-320b_AAAAGCTGGGTTGAGAGGGCA5	0,553830888	2,618155177	0,27517798	0,691851261	T2D vs Control
hsa-let-7f-5p_TGAGGTAGTAGATTGTATA5	0,592031888	3,61271535	0,277351209	0,693999796	T2D vs Control
hsa-let-7a-5p_TGAGGTAGTAGGTTGTAT5	0,753445987	3,216663859	0,277774071	0,693999796	T2D vs Control
hsa-miR-500a-3p_AATGCACCTGGGCAAGGATTCT5	0,661705256	2,780066987	0,279455413	0,696018639	T2D vs Control
hsa-miR-25-3p_ATTGCACTTGTCTCGGTCTGA5	0,363203375	5,837958082	0,280496187	0,696434457	T2D vs Control
hsa-miR-335-5p_TCAAGAGCAATAACGAAAAATG5	-0,198646502	8,412522999	0,28573202	0,706685958	T2D vs Control
hsa-miR-30e-3p_CTTTCAGTCGGATGTTTACAGC5	-0,60438267	3,314941715	0,28639845	0,706685958	T2D vs Control
hsa-miR-339-3p_TGAGCGCCTCGACGACAGAG5	0,443247808	5,154516456	0,28793243	0,708278231	T2D vs Control
hsa-miR-126-3p_CGTACCGTGAGTAATAATGCG5	0,156121108	9,683358758	0,291961622	0,710559826	T2D vs Control
hsa-miR-1908-5p_CGGCGGGGACGGCGATTGGTC5	0,621596769	2,612171571	0,292788639	0,710559826	T2D vs Control
hsa-miR-151a-3p_CTAGACTGAAGCTCCTTGAGGA5	-0,190225482	8,444952042	0,292938992	0,710559826	T2D vs Control
hsa-miR-340-3p_TCCGTCTCAGTTACTTTATAGC5	-0,566437799	1,820184636	0,293241078	0,710559826	T2D vs Control
hsa-miR-92a-3p_ATTGCACTTGTCCCGGCCT5	0,530165683	4,477303001	0,29331767	0,710559826	T2D vs Control
hsa-let-7d-3p_TATACGACCTGCTGCCTTTC5	-0,26820959	6,429871038	0,295097055	0,7127041	T2D vs Control
hsa-miR-146b-5p_TGAGAACTGAATTCCATAGGCT5	0,302542068	6,34592746	0,296559063	0,714071218	T2D vs Control
hsa-miR-30a-5p_TGTAAACATCCTCGACTGGAAG5	0,188256268	8,337829601	0,300154097	0,718429568	T2D vs Control
hsa-miR-191-5p_AACGGAATCCCAAAAGCAGC5	0,569153383	2,920397045	0,300171953	0,718429568	T2D vs Control

hsa-miR-493-5p_TTGTACATGGTAGGCTTTCATT5	-0,644835059	3,64649227	0,303368909	0,719553947	T2D vs Control
hsa-miR-486-5p_TCCTGTACTGAGCTGCCCCGA5	-0,146195747	17,03890679	0,303447345	0,719553947	T2D vs Control
hsa-miR-29a-3p_TAGCACCATCTGAAATCGGTT5	0,183650495	8,641484144	0,305489624	0,719553947	T2D vs Control
hsa-miR-23a-3p_TCACATTGCCAGGGATTTCC5	-0,497480218	4,540513631	0,306811204	0,719553947	T2D vs Control
hsa-miR-486-5p_TGTACTGAGCTGCCCCGAG5	0,437353708	5,036658236	0,30711762	0,719553947	T2D vs Control
hsa-miR-409-3p_CGAATGTTGCTCGGTGAACCCCTTT5	-0,650714175	2,598256943	0,308105076	0,719553947	T2D vs Control
hsa-miR-125b-5p_TCCCTGAGACCCTAACTTG5	-0,172593483	8,171400371	0,309172345	0,719553947	T2D vs Control
hsa-miR-16-2-3p_CCAATATTACTGTGCTGCTTT5	0,471078649	4,35154905	0,309290353	0,719553947	T2D vs Control
hsa-miR-485-3p_GTCATACACGGCTCTCCTCTCT5	-0,531964991	5,913980943	0,309459281	0,719553947	T2D vs Control
hsa-miR-221-3p_AGCTACATTGTCTGCTGGGT5	0,323836228	6,226471989	0,310377704	0,719553947	T2D vs Control
hsa-let-7i-5p_TGAGGTAGTAGTTTGTGCTGTTG5	0,537809999	3,894577335	0,311870326	0,719553947	T2D vs Control
hsa-miR-139-3p_TGGAGACGCGGCCCTGTTGGAGT5	0,595207457	3,435697412	0,312332828	0,719553947	T2D vs Control
hsa-miR-345-5p_GCTGACTCCTAGTCCAGGGCTC5	0,588478664	3,656556535	0,312378501	0,719553947	T2D vs Control
hsa-miR-484_TCAGGCTCAGTCCCCTCCCGAT5	0,137743371	9,18414004	0,313967999	0,720954728	T2D vs Control
hsa-let-7b-5p_GAGGTAGTAGGTTGTGTGG5	0,33258925	6,125260792	0,314795791	0,720954728	T2D vs Control
hsa-miR-30d-5p_TAAACATCCCCGACTGGAAGCT5	0,558982184	2,174183718	0,315923047	0,721463234	T2D vs Control
hsa-miR-433-3p_ATCATGATGGGCTCCTCGGTGT5	-0,595350811	4,303379714	0,318369021	0,7231005	T2D vs Control
hsa-miR-144-3p_TACAGTATAGATGATGTACT5	0,47724301	4,501383498	0,319609073	0,7231005	T2D vs Control
hsa-miR-145-3p_ATTCCTGGAAATACTGTTCT5	-0,558102918	3,209907444	0,319721065	0,7231005	T2D vs Control
hsa-miR-125a-5p_TCCCTGAGACCCTTTAACC5	0,544337326	2,158659604	0,321035693	0,7231005	T2D vs Control
hsa-miR-323a-3p_GCACATTACACGGTCGACCTCT5	-0,5529633	3,90036256	0,322588759	0,7231005	T2D vs Control
hsa-miR-16-5p_TTAGCAGCACGTAAATATTGGCG5	0,496696908	4,199452303	0,323063281	0,7231005	T2D vs Control
hsa-miR-21-5p_TAGCTTATCAGACTGATGTTGA5	-0,082932381	12,88530966	0,323655484	0,7231005	T2D vs Control
hsa-miR-199a-5p_CCCAGTGTTCAGACTACCTGTTC5	-0,489165677	4,567460238	0,325042807	0,7231005	T2D vs Control
hsa-miR-340-5p_TTATAAAGCAATGAGACTGATT5	-0,361684881	5,815012818	0,325800344	0,7231005	T2D vs Control
hsa-miR-140-3p_ACCACAGGGTAGAACCACGGAC5	0,339443773	5,852260421	0,326418939	0,7231005	T2D vs Control
hsa-miR-10b-5p_TACCCTGTAGAACCGAATTTGTG5	0,202524329	7,78613959	0,326861134	0,7231005	T2D vs Control
hsa-miR-3173-5p_CCCTGCCTGTTTTCTCCTTTGT5	0,516709168	1,879782778	0,327959789	0,7231005	T2D vs Control
hsa-miR-25-3p_CATTGCACTTGTCTCGGTCTG5	0,172022984	9,990945876	0,330111859	0,7231005	T2D vs Control
hsa-miR-484_CAGGCTCAGTCCCCTCCCGAT5	0,517674843	2,404164158	0,331325026	0,7231005	T2D vs Control
hsa-miR-451a_GAAACCGTTACCATTACTGAG5	0,51110668	3,176702933	0,331508776	0,7231005	T2D vs Control
hsa-miR-27a-3p_TCACAGTGGCTAAGTTCCG5	0,555186149	2,668037972	0,33234718	0,7231005	T2D vs Control
hsa-miR-221-3p_AGCTACATTGTCTGCTGGGTT5	0,31185964	6,683307876	0,332582734	0,7231005	T2D vs Control
hsa-miR-3615_TCTCTCGGCTCCTCGCGGCTCGC5	-0,494602816	3,835837244	0,332970996	0,7231005	T2D vs Control
hsa-miR-191-5p_CAACGGAATCCCAAAAGCAGCTG5	-0,176093679	9,658129048	0,335787726	0,727235918	T2D vs Control
hsa-let-7b-5p_TGAGGTAGTAGGTTGTGT5	0,367466698	5,292834315	0,33763388	0,72925258	T2D vs Control
hsa-miR-543_AAACATTCGCGGTGCACTTCTT5	-0,513991114	3,845833985	0,340026156	0,73243472	T2D vs Control
hsa-miR-3158-3p_AAGGGCTTCCTCTCTGCAGGA5	0,567939912	2,734692583	0,344728246	0,740561759	T2D vs Control
hsa-miR-10b-5p_TACCCTGTAGAACCGAATT5	-0,458537771	4,675674869	0,346012807	0,741055262	T2D vs Control
hsa-miR-145-5p_GTCCAGTTTTCCCAGGAATCC5	0,530968832	2,283533911	0,346817582	0,741055262	T2D vs Control
hsa-miR-425-5p_AATGACACGATCACTCCCGTTGAG5	-0,368093403	6,063293525	0,348104226	0,74147088	T2D vs Control
hsa-miR-30a-3p_CTTTCAGTCGGATGTTTGCAG5	0,524954801	1,943754996	0,349581646	0,74147088	T2D vs Control
hsa-miR-148a-3p_TCAGTGCACTACAGAACTT5	0,572179082	2,124123364	0,349803075	0,74147088	T2D vs Control
hsa-let-7a-5p_TGAGGTAGTAGGTTGTATAGTT5	-0,13190574	13,39549393	0,352129889	0,74294001	T2D vs Control
hsa-miR-30e-5p_TGTAAACATCCTTGACTGGAAGCT5	-0,06982476	11,72480372	0,353087284	0,74294001	T2D vs Control
hsa-miR-221-3p_AGCTACATTGTCTGCTGGGTTTC5	-0,290474978	6,752581432	0,35377852	0,74294001	T2D vs Control
hsa-miR-10b-5p_ACCCTGTAGAACCGAATTTG5	-0,413913161	4,835525593	0,354985662	0,74294001	T2D vs Control
hsa-miR-409-3p_CGAATGTTGCTCGGTGAACCCCT5	-0,481423603	5,738161577	0,355157019	0,74294001	T2D vs Control
hsa-let-7c-5p_TGAGGTAGTAGGTTGTATGGTTT5	0,473537415	3,566946574	0,356631935	0,744072389	T2D vs Control
hsa-miR-146a-5p_TGAGAACTGAATTCCATGGGTT5	0,109836655	12,1607009	0,358435112	0,745881943	T2D vs Control
hsa-miR-942-5p_TCTTCTCTGTTTTGGCCATGT5	0,52737044	2,592733465	0,360515153	0,748256711	T2D vs Control
hsa-miR-4433b-5p_TATGTCCCACCCCCACTCCTGT5	-0,452741112	5,397905397	0,361712718	0,748792302	T2D vs Control
hsa-miR-223-3p_TGTCAGTTTGTCAAATACCCC5	-0,264042313	7,314719746	0,363961572	0,751495785	T2D vs Control
hsa-miR-24-3p_GGCTCAGTTCAGCAGGAACA5	0,516804417	2,640585933	0,36522657	0,752159111	T2D vs Control
hsa-miR-125b-5p_CCCTGAGACCCTAACTTGT5	-0,509351662	2,290772166	0,371183422	0,762456668	T2D vs Control
hsa-let-7f-5p_TGAGGTAGTAGATTGTATAGTT5	-0,148203932	12,51171334	0,372595463	0,76250989	T2D vs Control
hsa-miR-140-3p_TACCACAGGGTAGAACCACGG5	0,289154634	5,563733367	0,373175925	0,76250989	T2D vs Control
hsa-miR-128-3p_TCACAGTGAACCGGTCTCTTTT5	-0,351082732	5,404238817	0,374079507	0,76250989	T2D vs Control
hsa-miR-15b-5p_TAGCAGCACATCATGGTT5	0,228217153	7,417344831	0,375699952	0,763859341	T2D vs Control
hsa-miR-10b-5p_TACCCTGTAGAACCGAATTTGT5	-0,105663304	11,12588043	0,381875541	0,772786974	T2D vs Control
hsa-miR-92a-3p_GTATTGCACTTGTCCCGGCCTG5	0,493508227	3,065032086	0,383118028	0,772786974	T2D vs Control

hsa-miR-221-3p_AGCTACATTGTCTGCTGGGTTTCA5	-0,505790446	2,390031097	0,383637907	0,772786974	T2D vs Control
hsa-miR-375-3p_TTGTTCGTTCGGCTCGCGTGA5	0,3680563	5,662970821	0,384687405	0,772786974	T2D vs Control
hsa-let-7g-5p_TGAGGTAGTAGTTTGTACAG5	0,115414419	9,407543438	0,385473917	0,772786974	T2D vs Control
hsa-miR-320a-3p_AAAGCTGGGTTGAGAGGGCG5	-0,379185877	4,890613401	0,386770675	0,772786974	T2D vs Control
hsa-miR-409-3p_GAATGTTGCTCGGTGAACCCCT5	-0,312683803	7,973406619	0,386878297	0,772786974	T2D vs Control
hsa-miR-21-3p_CAACACCAGTCGATGGGCTGT5	0,458402148	2,296740898	0,388563864	0,774213499	T2D vs Control
hsa-miR-99b-5p_CACCCGTAGAACCGACCTTGC5	-0,135084086	7,880557566	0,391992036	0,777361279	T2D vs Control
hsa-miR-423-3p_GCTCGGTCTGAGGCCCCTCAG5	0,490990027	1,954419998	0,394514204	0,777361279	T2D vs Control
hsa-miR-29a-3p_CTAGCACCATCTGAAATCGGTT5	0,425051899	3,457099062	0,395886779	0,777361279	T2D vs Control
hsa-miR-99b-5p_CACCCGTAGAACCGACCTT5	0,486137382	2,002435028	0,396601758	0,777361279	T2D vs Control
hsa-miR-409-3p_AATGTTGCTCGGTGAACCCCT5	-0,49408851	4,366315065	0,397695551	0,777361279	T2D vs Control
hsa-miR-652-3p_AATGGCGCCACTAGGGTTGTG5	0,238847087	6,468098031	0,398664826	0,777361279	T2D vs Control
hsa-miR-451a_AACCGTTACCATTACTGAGT5	0,134588155	10,87302044	0,398917919	0,777361279	T2D vs Control
hsa-miR-660-5p_TACCCATTGCATATCGGAGTTGT5	0,438911215	3,90808508	0,404236197	0,777361279	T2D vs Control
hsa-miR-183-5p_TATGGCACTGGTAGAATTCA5	0,239190802	7,32183424	0,405980842	0,777361279	T2D vs Control
hsa-miR-23a-3p_ATCACATTGCCAGGGATTTCC5	0,08860725	11,08225248	0,406222066	0,777361279	T2D vs Control
hsa-miR-181a-2-3p_ACCACTGACCGTTGACTGTACC5	0,491083804	2,397891497	0,406822486	0,777361279	T2D vs Control
hsa-miR-16-2-3p_ACCAATATTACTGTGCTGCTT5	0,137023545	8,312552057	0,407226169	0,777361279	T2D vs Control
hsa-miR-144-3p_CTACAGTATAGATGATGTAC5	0,461883352	3,24422031	0,40788726	0,777361279	T2D vs Control
hsa-miR-181b-5p_AACATTCATTGCTGTCGGTGG5	0,310085914	5,135854331	0,408719151	0,777361279	T2D vs Control
hsa-miR-21-5p_TAGCTTATCAGACTGATGTT5	0,168764985	7,170728426	0,408922219	0,777361279	T2D vs Control
hsa-miR-16-5p_GCAGCACGTAAATATTGGCG5	0,412948526	3,625120505	0,409039893	0,777361279	T2D vs Control
hsa-miR-140-3p_ACCACAGGGTAGAACCACGGA5	0,238785289	6,646596672	0,409144849	0,777361279	T2D vs Control
hsa-miR-26b-5p_TTCAAGTAATTCAGGATAGG5	-0,390145952	3,436379997	0,410070701	0,777361279	T2D vs Control
hsa-miR-532-3p_CCTCCCACACCCAAGGCTTG5	0,479734336	2,745637111	0,411439086	0,777361279	T2D vs Control
hsa-miR-30e-5p_TGTAAACATCCTTGACTGGAAGC5	-0,100140716	8,385302075	0,412182584	0,777361279	T2D vs Control
hsa-let-7i-5p_TGAGGTAGTAGTTTGTGCTG5	0,124921075	10,07708045	0,412307194	0,777361279	T2D vs Control
hsa-miR-150-3p_CTGGTACAGGCCTGGGGGACA5	0,445567266	4,060108896	0,412528126	0,777361279	T2D vs Control
hsa-miR-629-5p_TGGGTTTACGTTGGGAGAACTT5	0,419431096	4,034612462	0,41257694	0,777361279	T2D vs Control
hsa-miR-329-3p_AACACACCTGGTTAACCTCTT5	-0,478004362	3,637419766	0,418123781	0,785954372	T2D vs Control
hsa-miR-483-3p_TCACTCCTCTCCTCCCGTCT5	-0,495592383	2,217026305	0,419586579	0,786848243	T2D vs Control
hsa-miR-423-3p_AAGCTCGGTCTGAGGCCCCTCA5	0,444010433	2,923364981	0,421883209	0,787220046	T2D vs Control
hsa-let-7e-5p_TGAGGTAGGAGGTTGTATAGTT5	0,172257012	7,813417368	0,422295019	0,787220046	T2D vs Control
hsa-miR-486-5p_TCCTGTACTGAGCTGCCCC5	-0,176797374	6,793601467	0,424489581	0,787220046	T2D vs Control
hsa-miR-30d-5p_GTAAACATCCCCGACTGGAAGCT5	-0,206315944	6,998439264	0,424557994	0,787220046	T2D vs Control
hsa-let-7a-5p_GTGAGGTAGTAGGTTGTATAGTT5	0,493716005	2,866169762	0,424723488	0,787220046	T2D vs Control
hsa-miR-92a-3p_ATTGCACTTGTCCCGGCCTG5	-0,131373236	9,121941427	0,426126644	0,787471988	T2D vs Control
hsa-miR-342-3p_TCTCACACAGAAATCGCACCCG5	0,167301993	9,658047597	0,426835507	0,787471988	T2D vs Control
hsa-let-7f-5p_TGAGGTAGTAGATTGTATAGTTG5	-0,472196046	2,64125494	0,428804753	0,789278032	T2D vs Control
hsa-miR-92a-3p_TTGCACTTGTCCCGGCCTG5	0,38965176	4,313701526	0,430598532	0,790753524	T2D vs Control
hsa-miR-423-5p_TGAGGGGCAGAGAGCGAGAC5	0,080678087	9,751921415	0,434735006	0,795324091	T2D vs Control
hsa-miR-142-5p_CCCATAAAGTAGAAAGCACTAC5	0,191466662	7,021131164	0,436042982	0,795324091	T2D vs Control
hsa-miR-320a-3p_AAAGCTGGGTTGAGAGGGCGAA5	0,369983682	4,271030608	0,436081089	0,795324091	T2D vs Control
hsa-miR-23a-3p_TCACATTGCCAGGGATTTC5	0,456458133	2,403793771	0,43919559	0,798370395	T2D vs Control
hsa-miR-501-3p_ATGCACCCGGGCAAGGATTCT5	0,451608652	2,081940648	0,439754835	0,798370395	T2D vs Control
hsa-miR-182-5p_TTTGGCAATGGTAGAACTCA5	0,181372021	7,910992501	0,442531372	0,801585235	T2D vs Control
hsa-miR-199a-3p_ACAGTAGTCTGCACATTGGTT5	-0,677210114	4,64378834	0,443809421	0,801791877	T2D vs Control
hsa-miR-186-5p_AAAGAATTCTCCTTTTGGGCT5	0,4169912	3,890936215	0,44517655	0,801791877	T2D vs Control
hsa-miR-21-5p_AGCTTATCAGACTGATGTTGA5	0,169570905	6,721668578	0,446742892	0,801791877	T2D vs Control
hsa-miR-29b-3p_TAGCACCATTTGAAATCAGT5	0,375064091	4,363760053	0,446869837	0,801791877	T2D vs Control
hsa-miR-29a-3p_TAGCACCATCTGAAATCGGTTA5	0,356196378	4,716466347	0,447997951	0,801791877	T2D vs Control
hsa-miR-26a-5p_TTCAAGTAATCCAGGATAGGCT5	-0,129340043	11,92893731	0,448681527	0,801791877	T2D vs Control
hsa-miR-125b-5p_TCCCTGAGACCCTAACTTGT5	-0,10888027	9,802438562	0,455986051	0,813022108	T2D vs Control
hsa-miR-423-5p_GAGGGGCAGAGAGCGAGACTTT5	-0,095096919	8,388365906	0,463219952	0,822259879	T2D vs Control
hsa-miR-103a-3p_AGCAGCATTGTACAGGGCTAT5	-0,508750146	3,428123511	0,463879053	0,822259879	T2D vs Control
hsa-miR-4446-3p_CAGGGCTGGCAGTGACATGGGT5	0,463415913	3,598813459	0,464506751	0,822259879	T2D vs Control
hsa-miR-92a-3p_TATTGCACTTGTCCCGGC5	0,194921876	6,608463448	0,465365349	0,822259879	T2D vs Control
hsa-miR-101-3p_GTACAGTACTGTGATAACT5	-0,319726498	4,990449736	0,466325552	0,822259879	T2D vs Control
hsa-miR-26b-5p_TTCAAGTAATTCAGGATAGGT5	0,146351369	7,343481575	0,469380703	0,823964389	T2D vs Control
hsa-miR-342-5p_AGGGGTGCTATCTGTGATTGA5	0,42661501	3,958584465	0,470122012	0,823964389	T2D vs Control
hsa-miR-23a-3p_ATCACATTGCCAGGGATTTCCA5	-0,103156688	11,15500363	0,471662625	0,823964389	T2D vs Control

hsa-miR-3173-5p_TGCCCTGCCTGTTTTCTCCTT5	-0,414010258	2,97364428	0,471888923	0,823964389	T2D vs Control
hsa-miR-142-5p_CATAAAGTAGAAAGCACTACT5	0,37164044	3,650597591	0,473022876	0,823964389	T2D vs Control
hsa-miR-487b-3p_AATCGTACAGGGTCATCCACTT5	0,432580177	4,048228768	0,47349522	0,823964389	T2D vs Control
hsa-miR-92b-3p_TATTGCACTCGTCCCGGCCT5	-0,272937534	5,68469958	0,480193422	0,83213073	T2D vs Control
hsa-miR-375-3p_TTTGTTCGTTCGGCTCGCG5	0,281229298	5,830563474	0,480276206	0,83213073	T2D vs Control
hsa-miR-150-5p_GTCTCCCAACCCTTGTACCAGTG5	0,411953212	2,975164068	0,481520097	0,832476176	T2D vs Control
hsa-miR-93-5p_CAAAGTGCTGTTCGTGCAGGTAG5	0,083511992	9,991178453	0,487444502	0,838577971	T2D vs Control
hsa-miR-183-5p_TATGGCACTGGTAGAATTC5	0,382036204	3,376816673	0,48748645	0,838577971	T2D vs Control
hsa-miR-1180-3p_TTTCCGGCTCGCGTGGGTGT5	0,329818354	4,070138113	0,488205996	0,838577971	T2D vs Control
hsa-miR-21-5p_TAGCTTATCAGACTGATGTTG5	0,078857845	11,59436943	0,494743421	0,847249379	T2D vs Control
hsa-miR-150-3p_CTGGTACAGGCCTGGGGGAC5	0,399932053	2,308717969	0,496005039	0,847249379	T2D vs Control
hsa-miR-484_TCAGGCTCAGTCCCCTCCCG5	0,165972956	7,780454359	0,496443488	0,847249379	T2D vs Control
hsa-miR-100-5p_AACCCGTAGATCCGAACTTGTG5	0,40765023	3,590895666	0,497737184	0,84764217	T2D vs Control
hsa-miR-423-5p_GAGGGGCAGAGAGCGAGACTTTT5	-0,341828593	4,029696332	0,499342755	0,848563275	T2D vs Control
hsa-miR-28-3p_CACTAGATTGTGAGCTCCTGG5	0,217369397	5,876182682	0,50077018	0,849178369	T2D vs Control
hsa-miR-3605-3p_CCTCCGTGTTACCTGTCCTCT5	-0,295836692	5,168364179	0,501937482	0,849350687	T2D vs Control
hsa-miR-21-5p_GTAGCTTATCAGACTGATGTTGA5	0,39058565	2,217922154	0,503894097	0,850855075	T2D vs Control
hsa-miR-423-5p_GAGGGGCAGAGAGCGAGACT5	0,204154591	6,874414686	0,508327915	0,856081962	T2D vs Control
hsa-miR-451a_AAACCGTTACCATTACTGAG5	0,109035455	13,72832534	0,510192156	0,856081962	T2D vs Control
hsa-miR-193a-5p_TGGGTCTTTGCGGGCGAGAT5	0,299678472	4,669337288	0,511142137	0,856081962	T2D vs Control
hsa-miR-486-5p_ATCCTGTACTGAGCTGCCCCGAG5	0,132003247	7,990918242	0,511688187	0,856081962	T2D vs Control
hsa-miR-1249-3p_ACGCCCTTCCCCCCCTTCTTCA5	-0,415727984	2,195504284	0,512708752	0,856081962	T2D vs Control
hsa-miR-652-3p_AATGGCGCCACTAGGGTTGTGC5	-0,340003789	3,594273542	0,513434351	0,856081962	T2D vs Control
hsa-miR-146b-5p_TGAGAACTGAATTCCATAGGCTGT5	0,113077024	8,368012324	0,51599448	0,856497265	T2D vs Control
hsa-miR-30e-5p_TGTAAACATCCTTGACTGGA5	0,369610287	2,511168014	0,516214939	0,856497265	T2D vs Control
hsa-miR-4732-3p_GCCCTGACCTGTCCTGTTCTG5	-0,568978983	4,156168491	0,520157322	0,856497265	T2D vs Control
hsa-miR-486-5p_CCTGTACTGAGCTGCCCCG5	-0,137725065	8,015321722	0,520172124	0,856497265	T2D vs Control
hsa-miR-98-5p_TGAGGTAGTAAGTTGTATTGTT5	-0,169367433	7,877215034	0,52029899	0,856497265	T2D vs Control
hsa-miR-363-3p_AATTGCACGGTATCCATCT5	0,318186829	4,478964619	0,522090643	0,856497265	T2D vs Control
hsa-miR-744-5p_TGCGGGGCTAGGGCTAACAGC5	0,326080307	3,991128142	0,522733546	0,856497265	T2D vs Control
hsa-miR-652-3p_AATGGCGCCACTAGGGTTG5	0,347549169	4,499546053	0,522933675	0,856497265	T2D vs Control
hsa-let-7f-5p_TGAGGTAGTAGATTGTATAG5	-0,076917114	10,59314063	0,524458584	0,856497265	T2D vs Control
hsa-miR-150-5p_CTCCCAACCCTTGTACCAGT5	-0,364021131	3,181356832	0,525794357	0,856497265	T2D vs Control
hsa-let-7a-5p_GAGGTAGTAGGTTGTATAG5	0,257464015	5,403501302	0,525800935	0,856497265	T2D vs Control
hsa-miR-30e-5p_TAAACATCCTTGACTGGAAGCT5	-0,364299744	2,076536633	0,528103721	0,856497265	T2D vs Control
hsa-miR-26a-5p_TTCAAGTAATCCAGGATAGGC5	0,104760428	7,510198846	0,528669827	0,856497265	T2D vs Control
hsa-miR-100-5p_AACCCGTAGATCCGAACTTGT5	0,157852328	7,211971456	0,529719186	0,856497265	T2D vs Control
hsa-miR-125b-5p_TCCCTGAGACCCTAACTT5	-0,326368802	3,016083692	0,531188409	0,856497265	T2D vs Control
hsa-miR-485-5p_AGAGGCTGGCCGTGATGAATTC5	-0,389706415	2,300221452	0,532078093	0,856497265	T2D vs Control
hsa-miR-30e-5p_GTAAACATCCTTGACTGGAAGCT5	0,06991331	8,781643893	0,532382541	0,856497265	T2D vs Control
hsa-miR-152-3p_TCAGTGCATGACAGAACTTGG5	0,328322434	2,073986305	0,534090384	0,856497265	T2D vs Control
hsa-miR-150-5p_TCTCCCAACCCTTGTACCAG5	-0,138063815	9,063580505	0,534101808	0,856497265	T2D vs Control
hsa-miR-106b-5p_TAAAGTGCTGACAGTGCAGA5	-0,251494281	5,144709005	0,53554414	0,857085702	T2D vs Control
hsa-miR-183-5p_TATGGCACTGGTAGAATT5	0,364197468	2,805102899	0,537062447	0,857793126	T2D vs Control
hsa-miR-103a-3p_AGCAGCATTGTACAGGGC5	0,417997738	3,081760646	0,543257615	0,86443908	T2D vs Control
hsa-miR-101-3p_TACAGTACTGTGATAACTGAAG5	-0,141859609	7,012156412	0,543392696	0,86443908	T2D vs Control
hsa-let-7f-5p_TGAGGTAGTAGATTGTATAGT5	0,065142864	10,56301224	0,545823218	0,866575906	T2D vs Control
hsa-miR-186-5p_CAAAGAATTCTCCTTTTGGGCT5	0,077494143	9,032677455	0,548898414	0,868073561	T2D vs Control
hsa-miR-99b-5p_ACCCGTAGAACCGACCTTGCG5	0,33902364	2,082938158	0,548944887	0,868073561	T2D vs Control
hsa-miR-142-5p_CCCATAAAGTAGAAAGCACT5	0,059072701	11,13638127	0,552882657	0,872081956	T2D vs Control
hsa-miR-375-3p_TTTGTTCGTTCGGCTCGCGT5	0,338535167	3,894636907	0,553874181	0,872081956	T2D vs Control
hsa-miR-885-5p_TCCATTACACTACCCTGCCTCT5	0,665683909	4,714772841	0,556313679	0,872081956	T2D vs Control
hsa-miR-185-5p_TGGAGAGAAAGGCAGTTCCTGA5	-0,082944918	8,851967404	0,556345524	0,872081956	T2D vs Control
hsa-miR-26a-5p_TCAAGTAATCCAGGATAGGCT5	0,177327145	6,522605962	0,55695071	0,872081956	T2D vs Control
hsa-miR-423-5p_TGAGGGGCAGAGAGCGAGACTTT5	-0,042017493	14,42674435	0,559855209	0,874910983	T2D vs Control
hsa-miR-191-5p_CAACGGAATCCCAAAAGCAGC5	0,112079394	8,150484012	0,566596809	0,882553392	T2D vs Control
hsa-miR-15a-5p_TAGCAGCACATAATGGTTTGT5	0,179147709	6,478410353	0,566960272	0,882553392	T2D vs Control
hsa-miR-6803-3p_TCCCTCGCCTTCTCACCCTCAG5	-0,31348374	2,710817974	0,568556267	0,883312562	T2D vs Control
hsa-miR-486-5p_TCCTGTACTGAGCTGCCCCGAGG5	-0,104350988	7,467002764	0,570616854	0,88405473	T2D vs Control
hsa-miR-500a-3p_ATGCACCTGGGCAAGGATTCT5	0,286090319	4,513942296	0,5720144	0,88405473	T2D vs Control
hsa-miR-15b-5p_TAGCAGCACATCATGGTTTAC5	0,117958309	7,368236859	0,572361657	0,88405473	T2D vs Control

hsa-let-7i-5p_TGAGGTAGTAGTTTGTGCTGTT5	-0,08917046	10,48466024	0,577005219	0,886838902	T2D vs Control
hsa-miR-423-3p_AAGCTCGGTCTGAGGCCCCT5	0,327478712	2,46496229	0,577316262	0,886838902	T2D vs Control
hsa-miR-27a-3p_TTCACAGTGGCTAAGTTCCGC5	-0,158162042	6,28650114	0,578332716	0,886838902	T2D vs Control
hsa-miR-486-5p_GTACTGAGCTGCCCCGA5	0,225742156	4,609129379	0,578615093	0,886838902	T2D vs Control
hsa-miR-27a-3p_TTCACAGTGGCTAAGTTCCG5	-0,069058642	9,740987062	0,582879953	0,891660888	T2D vs Control
hsa-miR-10b-5p_TACCCTGTAGAACCGAAT5	-0,313403629	2,220184724	0,590036917	0,893710632	T2D vs Control
hsa-miR-128-3p_TCACAGTGAACCGGTCTCTT5	-0,119929756	6,808413156	0,590473128	0,893710632	T2D vs Control
hsa-miR-15b-5p_TAGCAGCACATCATGGTTTA5	0,183933117	5,794504936	0,590686692	0,893710632	T2D vs Control
hsa-miR-664a-3p_TATTCATTTATCCCCAGCCTACA5	-0,296324308	3,022366025	0,591313201	0,893710632	T2D vs Control
hsa-miR-191-5p_CAACGGAATCCCAAAAGCA5	0,156492596	6,203677568	0,592031963	0,893710632	T2D vs Control
hsa-miR-142-5p_CCCATAAAGTAGAAAGCACTA5	-0,19996733	5,405299379	0,592474257	0,893710632	T2D vs Control
hsa-miR-654-5p_TGGTGGGCCGCAGAACATGTGC5	0,348675371	3,027916369	0,593745906	0,893710632	T2D vs Control
hsa-miR-26a-5p_TTCAAGTAATCCAGGATAGG5	0,115964976	6,813190078	0,595191315	0,893710632	T2D vs Control
hsa-miR-18a-3p_ACTGCCCTAAGTGCTCCTTCT5	0,303666578	3,273299722	0,596193687	0,893710632	T2D vs Control
hsa-miR-192-5p_TGACCTATGAATTGACAGC5	0,31248409	3,665629227	0,596421699	0,893710632	T2D vs Control
hsa-miR-320a-3p_AAAAGCTGGGTTGAGAGGGCGAAA5	0,152436877	6,744969015	0,596835735	0,893710632	T2D vs Control
hsa-miR-15b-3p_CGAATCATTATTTGCTGCTCT5	0,241546585	4,350054835	0,597675993	0,893710632	T2D vs Control
hsa-miR-423-5p_CTGAGGGGCAGAGAGCGAGACTTT5	0,266909768	4,419437576	0,601186929	0,897277121	T2D vs Control
hsa-miR-451a_GAAACCGTTACCATTACTGAGT5	0,123798851	6,98332366	0,603902007	0,899644672	T2D vs Control
hsa-miR-342-3p_TCACACAGAAATCGCACCCGT5	0,286835356	2,35866661	0,607391813	0,901728379	T2D vs Control
hsa-miR-24-3p_GGCTCAGTTCAGCAGGAAC5	-0,321514596	2,859699768	0,607563538	0,901728379	T2D vs Control
hsa-miR-182-5p_TTTGGCAATGGTAGAACTCACA5	0,142689526	6,943076472	0,609794349	0,902289674	T2D vs Control
hsa-miR-3613-5p_TGTTGTACTTTTTTTTTTGT5	0,083901922	7,586394808	0,61020594	0,902289674	T2D vs Control
hsa-let-7a-5p_TGAGGTAGTAGGTTGTATAG5	0,055540871	12,05467063	0,612105351	0,902606445	T2D vs Control
hsa-miR-338-5p_AACAATATCCTGGTGCTGAGT5	-0,160802515	5,993094666	0,615690247	0,902606445	T2D vs Control
hsa-miR-125a-5p_TCCCTGAGACCCTTTAACCTGTG5	0,079450441	8,600810288	0,615846655	0,902606445	T2D vs Control
hsa-miR-192-5p_TGACCTATGAATTGACAGCC5	0,147387127	6,449264096	0,616016888	0,902606445	T2D vs Control
hsa-miR-181b-5p_AACATTCATTGCTGTCGGTGGGT5	0,249162645	3,594503523	0,616082693	0,902606445	T2D vs Control
hsa-miR-193a-5p_TGGGTCTTTGCGGGCGAGATG5	0,19238482	5,884704134	0,623798724	0,908912927	T2D vs Control
hsa-miR-485-5p_AGAGGCTGGCCGTGATGAATTCG5	-0,270528557	4,543642396	0,624348324	0,908912927	T2D vs Control
hsa-let-7g-5p_TGAGGTAGTAGTTTGTACAGTTT5	-0,131065466	6,811439564	0,624430729	0,908912927	T2D vs Control
hsa-let-7d-3p_ATACGACCTGCTGCCTTTCT5	-0,275402735	2,048536833	0,627094621	0,908912927	T2D vs Control
hsa-miR-374b-5p_ATATAATACAACCTGCTAAGT5	0,249080294	3,95574137	0,627132669	0,908912927	T2D vs Control
hsa-miR-101-3p_GTACAGTACTGTGATAACTG5	0,185388092	5,734652626	0,627229749	0,908912927	T2D vs Control
hsa-miR-191-5p_AACGGAATCCCAAAAGCAGCT5	0,125249082	6,816281646	0,630796589	0,910558017	T2D vs Control
hsa-miR-144-5p_GGATATCATCATATACTGTAAGT5	0,138412682	6,405140764	0,631404745	0,910558017	T2D vs Control
hsa-miR-125b-5p_TCCCTGAGACCCTAACTTGTGA5	0,179793589	5,6455681	0,632753737	0,910558017	T2D vs Control
hsa-miR-486-3p_CGGGGCAGCTCAGTACAGGAT5	0,116445135	7,803889597	0,633138508	0,910558017	T2D vs Control
hsa-miR-532-5p_CATGCCTTGAGTGTAGGACCG5	0,255899441	2,388684445	0,6367834	0,910558017	T2D vs Control
hsa-miR-342-3p_TCACACAGAAATCGCACCCGTCA5	0,257371949	3,930198822	0,637381837	0,910558017	T2D vs Control
hsa-let-7g-5p_GAGGTAGTAGTTTGTACAGTT5	-0,270211784	4,344091711	0,638825738	0,910558017	T2D vs Control
hsa-miR-222-3p_AGCTACATCTGGCTACTGGG5	0,272611594	2,549442498	0,639189667	0,910558017	T2D vs Control
hsa-miR-3615_TCTCTCGGCTCCTCGCGGC5	0,260801005	2,019798482	0,640456211	0,910558017	T2D vs Control
hsa-miR-424-3p_CAAAACGTGAGGCGCTGCTAT5	-0,158300475	5,519904886	0,642062715	0,910558017	T2D vs Control
hsa-miR-146a-5p_GAGAACTGAATTCCATGGGTT5	-0,151089111	6,481720435	0,643303458	0,910558017	T2D vs Control
hsa-let-7b-3p_CTATACAACCTACTGCCTTCC5	-0,131827067	6,023895535	0,644025305	0,910558017	T2D vs Control
hsa-miR-584-5p_TTATGGTTTGCCTGGGACTGA5	-0,094944225	8,070467342	0,644050773	0,910558017	T2D vs Control
hsa-miR-185-5p_TGGAGAGAAAGGCAGTTCCTG5	0,230793405	4,508281621	0,645956105	0,910558017	T2D vs Control
hsa-miR-150-5p_TCTCCCAACCCTTGTACCAGT5	0,083679437	10,25222562	0,646367793	0,910558017	T2D vs Control
hsa-miR-194-5p_TGTAACAGCAACTCCATGTGGA5	-0,258990002	2,048630169	0,64677548	0,910558017	T2D vs Control
hsa-miR-370-3p_GCCTGCTGGGGTGGAACCTGGT5	-0,282665193	2,498088509	0,648469756	0,910558017	T2D vs Control
hsa-miR-10b-5p_ACCCTGTAGAACCGAATTTGTGT5	0,242258763	3,356457419	0,648929678	0,910558017	T2D vs Control
hsa-miR-151a-3p_TACTAGACTGAAGCTCCTTGAG5	0,25036562	3,055291554	0,652588808	0,913452404	T2D vs Control
hsa-let-7b-5p_GAGGTAGTAGGTTGTGTGGT5	0,199493952	4,944887656	0,65530634	0,913452404	T2D vs Control
hsa-miR-183-5p_TATGGCACTGGTAGAATTCACTG5	0,26069224	3,516695948	0,656568734	0,913452404	T2D vs Control
hsa-let-7f-5p_GAGGTAGTAGATTGTATAGTT5	-0,158848824	6,051011881	0,658776301	0,913452404	T2D vs Control
hsa-miR-193b-5p_CGGGGTTTTGAGGGCGAGATGA5	0,267484875	2,282241357	0,65992073	0,913452404	T2D vs Control
hsa-miR-425-5p_AATGACACGATCACTCCCGTTGA5	-0,04678426	10,37803313	0,660703899	0,913452404	T2D vs Control
hsa-miR-125b-5p_TCCCTGAGACCCTAACTTGTG5	0,190668838	5,039062696	0,660910763	0,913452404	T2D vs Control
hsa-miR-142-5p_CATAAAGTAGAAAGCACTAC5	0,2489628	2,376484394	0,663360903	0,913452404	T2D vs Control
hsa-let-7f-5p_GAGGTAGTAGATTGTATAG5	-0,22846518	3,245137192	0,664626879	0,913452404	T2D vs Control

hsa-miR-3613-5p_TGTTGTACTTTTTTTTTTGTTC5	0,196269562	4,388236924	0,664630542	0,913452404	T2D vs Control
hsa-miR-3615_TCTCTCGGCTCCTCGCGGCT5	-0,119246002	6,584121427	0,66512219	0,913452404	T2D vs Control
hsa-miR-18a-3p_ACTGCCCTAAGTGCTCCTTCTG5	0,237274942	2,22678804	0,665250835	0,913452404	T2D vs Control
hsa-miR-93-5p_CAAAGTGCTGTTCGTGCAGGTAGT5	-0,231718701	4,129822813	0,668033076	0,913452404	T2D vs Control
hsa-miR-16-5p_AGCAGCACGTAAATATTGGCG5	0,07008347	8,17366975	0,668925207	0,913452404	T2D vs Control
hsa-let-7b-5p_TGAGGTAGTAGGTTGTGTGGT5	0,04590469	11,84645942	0,669747745	0,913452404	T2D vs Control
hsa-miR-26a-5p_TTCAAGTAATCCAGGATAG5	0,226943747	3,646262645	0,670458633	0,913452404	T2D vs Control
hsa-miR-30c-5p_GTAAACATCCTACACTCTCAGCT5	0,247337596	3,052199181	0,671945701	0,913452404	T2D vs Control
hsa-miR-342-3p_CTCACACAGAAATCGCACCCG5	0,249695721	2,495329338	0,673354121	0,913452404	T2D vs Control
hsa-miR-223-3p_TGTCAGTTTGTCAAATACCCCA5	-0,076619039	10,41019198	0,673918643	0,913452404	T2D vs Control
hsa-miR-92a-3p_TGCACTTGTCCCGGCCTGT5	0,160553332	5,28105363	0,674942714	0,913452404	T2D vs Control
hsa-miR-28-5p_AAGGAGCTCACAGTCTATTGA5	0,257713678	3,11393162	0,67615624	0,913452404	T2D vs Control
hsa-miR-143-3p_TGAGATGAAGCACTGTAGCTC5	0,065729057	9,842973082	0,676206924	0,913452404	T2D vs Control
hsa-miR-423-5p_TGAGGGGCAGAGAGCGAGA5	0,047855504	9,723922848	0,680691391	0,91795438	T2D vs Control
hsa-miR-28-3p_ACTAGATTGTGAGCTCCTGGAG5	0,191832655	4,615150746	0,683128891	0,918030715	T2D vs Control
hsa-miR-7-5p_TGGAAGACTAGTGATTTTGTTGTT5	0,227146083	4,415695214	0,683693254	0,918030715	T2D vs Control
hsa-let-7b-5p_TGAGGTAGTAGGTTGTGTG5	-0,088376487	6,962968331	0,684203569	0,918030715	T2D vs Control
hsa-miR-183-5p_ATGGCACTGGTAGAATTCACT5	0,194718178	4,652978697	0,687766829	0,921260778	T2D vs Control
hsa-miR-451a_AACCGTTACCATTACTGAG5	0,07523406	8,074719646	0,692351423	0,924721529	T2D vs Control
hsa-miR-501-3p_AATGCACCCGGGCAAGGATTC5	0,218830764	3,102124992	0,694686641	0,924721529	T2D vs Control
hsa-miR-30d-5p_TGTAAACATCCCCGACTGGAAG5	0,031931803	12,16128446	0,694751219	0,924721529	T2D vs Control
hsa-miR-423-3p_GCTCGGTCTGAGGCCCCTCAGT5	-0,124470723	6,432183419	0,694991463	0,924721529	T2D vs Control
hsa-miR-126-3p_GTACCGTGAGTAATAATGCG5	-0,217637392	2,067690175	0,698094292	0,927301918	T2D vs Control
hsa-miR-150-5p_TCTCCCAACCCTTGTACCA5	-0,13103772	6,588601483	0,701893919	0,930666712	T2D vs Control
hsa-miR-126-3p_CTCGTACCGTGAGTAATAATGCG5	-0,185597236	4,682348296	0,702962812	0,930666712	T2D vs Control
hsa-miR-342-3p_TCACACAGAAATCGCACCCGTC5	0,214558038	2,309823181	0,7048879	0,930712556	T2D vs Control
hsa-miR-23a-3p_ATCACATTGCCAGGGATTTC5	0,055325873	9,459376505	0,705332978	0,930712556	T2D vs Control
hsa-miR-98-5p_TGAGGTAGTAAGTTGTATTGT5	-0,120972754	6,55627531	0,710400845	0,93585037	T2D vs Control
hsa-miR-142-5p_CCATAAAGTAGAAAGCACT5	0,139733658	4,774985235	0,711940099	0,93633046	T2D vs Control
hsa-miR-30d-5p_TGTAAACATCCCCGACTGGAA5	0,036723783	11,66083446	0,713264083	0,936385881	T2D vs Control
hsa-miR-574-3p_CACGCTCATGCACACACCCAC5	0,178511449	4,117697394	0,714332014	0,936385881	T2D vs Control
hsa-miR-27a-3p_TTCACAGTGGCTAAGTTCC5	-0,128306198	5,369078847	0,717979485	0,939621756	T2D vs Control
hsa-miR-92a-3p_ATTGCACTTGTCCCGGCCTGTT5	-0,124631407	5,604292671	0,721381594	0,942526443	T2D vs Control
hsa-miR-24-3p_TGGCTCAGTTCAGCAGGAA5	0,142047918	5,648746767	0,725213697	0,945982515	T2D vs Control
hsa-miR-30d-5p_GTAAACATCCCCGACTGGAA5	0,143194568	4,975135303	0,72870623	0,948985073	T2D vs Control
hsa-miR-186-5p_CAAAGAATTCTCCTTTTGGGC5	0,099389114	6,535812742	0,73050875	0,949291705	T2D vs Control
hsa-miR-125a-5p_CCCTGAGACCCTTTAACCTGT5	0,141092868	5,351640533	0,733235996	0,949291705	T2D vs Control
hsa-miR-29c-3p_TAGCACCATTTGAAATCGG5	0,190814044	3,272314395	0,733682815	0,949291705	T2D vs Control
hsa-miR-182-5p_TTTGGCAATGGTAGAACTCACACTGG5	0,203709851	2,315372824	0,734158505	0,949291705	T2D vs Control
hsa-miR-101-3p_TACAGTACTGTGATAACTGAA5	-0,193875441	3,070531279	0,734897092	0,949291705	T2D vs Control
hsa-miR-27b-3p_TTCACAGTGGCTAAGTTCTGC5	0,062874991	7,903620851	0,739039311	0,951692178	T2D vs Control
hsa-miR-92a-3p_GTATTGCACTTGTCCCGGCCTGT5	-0,058450984	7,715362913	0,739404334	0,951692178	T2D vs Control
hsa-let-7b-5p_GAGGTAGTAGGTTGTGTGGTTT5	0,179491125	3,859782449	0,740414776	0,951692178	T2D vs Control
hsa-miR-126-5p_CATTATTACTTTTGGTACGC5	-0,174474954	3,620244945	0,741531797	0,951692178	T2D vs Control
hsa-miR-92a-3p_TATTGCACTTGTCCCGGCC5	-0,068107911	8,088512139	0,743111468	0,952186238	T2D vs Control
hsa-miR-222-3p_AGCTACATCTGGCTACTGGGTCT5	0,091830911	6,5045561	0,746423378	0,954894755	T2D vs Control
hsa-let-7d-3p_TATACGACCTGCTGCCTTT5	-0,085189648	6,275229414	0,749896035	0,955173253	T2D vs Control
hsa-miR-23a-3p_TCACATTGCCAGGGATTTCCAAC5	-0,166988709	2,126412114	0,754949775	0,955173253	T2D vs Control
hsa-miR-142-5p_CATAAAGTAGAAAGCACT5	0,08581624	5,539841362	0,759625264	0,955173253	T2D vs Control
hsa-miR-103a-3p_AGCAGCATTGTACAGGGCTATG5	-0,166598178	4,276844631	0,76071249	0,955173253	T2D vs Control
hsa-miR-99b-5p_CACCCGTAGAACCGACCTTG5	0,075101724	6,765454723	0,761417789	0,955173253	T2D vs Control
hsa-miR-125b-5p_TCCCTGAGACCCTAACT5	0,187280536	2,233733889	0,764961457	0,955173253	T2D vs Control
hsa-miR-425-5p_AATGACACGATCACTCCCGT5	0,076668546	6,738341736	0,765172385	0,955173253	T2D vs Control
hsa-miR-92b-3p_TATTGCACTCGTCCCGGCCTCC5	0,142854687	4,414239688	0,765296206	0,955173253	T2D vs Control
hsa-miR-345-5p_GCTGACTCCTAGTCCAGGGCT5	0,127452075	5,057881275	0,765332471	0,955173253	T2D vs Control
hsa-miR-10a-5p_TACCCTGTAGATCCGAAT5	-0,175800024	2,33323359	0,766706275	0,955173253	T2D vs Control
hsa-miR-151a-3p_TACTAGACTGAAGCTCCTTGA5	0,159817878	1,940948476	0,769686771	0,955173253	T2D vs Control
hsa-miR-126-3p_TCGTACCGTGAGTAATAATGC5	0,070687763	6,426310401	0,770215731	0,955173253	T2D vs Control
hsa-miR-30c-5p_TGTAAACATCCTACACTCTCAGCT5	-0,044261772	10,20131272	0,771336544	0,955173253	T2D vs Control
hsa-miR-382-5p_AAGTTGTTCGTGGTGGATTCG5	0,179636731	2,227150711	0,771729321	0,955173253	T2D vs Control
hsa-miR-30e-5p_GTAAACATCCTTGACTGGAAGC5	0,167828722	2,49990334	0,772540048	0,955173253	T2D vs Control

hsa-miR-340-3p_TCCGTCTCAGTTACTTTATAGCC5	0,168020501	3,094594983	0,772993683	0,955173253	T2D vs Control
hsa-miR-1307-3p_CTCGGCGTGGCGTCGGTCGTGG5	0,171325113	2,190246459	0,7734753	0,955173253	T2D vs Control
hsa-miR-23a-3p_TCACATTGCCAGGGATTTCCA5	-0,117064814	5,236791923	0,774586799	0,955173253	T2D vs Control
hsa-let-7g-5p_GAGGTAGTAGTTTGTACAG5	0,16658997	2,076526463	0,775142654	0,955173253	T2D vs Control
hsa-miR-486-5p_CTGTACTGAGCTGCCCCGAG5	-0,059778146	7,787642307	0,775275525	0,955173253	T2D vs Control
hsa-miR-99a-5p_AACCCGTAGATCCGATCTT5	-0,164667538	3,048788798	0,775470454	0,955173253	T2D vs Control
hsa-miR-221-3p_AGCTACATTGTCTGCTGGGTTT5	0,052119405	7,916859704	0,776791406	0,955173253	T2D vs Control
hsa-let-7a-5p_ATGAGGTAGTAGGTTGTATAGTT5	0,168703986	2,103462699	0,777465197	0,955173253	T2D vs Control
hsa-let-7b-5p_TGAGGTAGTAGGTTGTGTGG5	-0,030419509	12,43476497	0,780441575	0,955173253	T2D vs Control
hsa-miR-28-5p_AAGGAGCTCACAGTCTATTGAG5	0,163822931	3,384277878	0,780620285	0,955173253	T2D vs Control
hsa-miR-143-3p_TGAGATGAAGCACTGTAGCT5	0,048337654	9,047255511	0,780802811	0,955173253	T2D vs Control
hsa-miR-335-5p_TCAAGAGCAATAACGAAAAATGT5	0,097556804	6,149787911	0,780838178	0,955173253	T2D vs Control
hsa-miR-145-3p_ATTCCTGGAAATACTGTTCTT5	0,156602082	3,50539909	0,781678249	0,955173253	T2D vs Control
hsa-miR-494-3p_TGAAACATACACGGGAAACCTCT5	0,153682004	4,269868739	0,782906708	0,955173253	T2D vs Control
hsa-miR-629-5p_TGGGTTTACGTTGGGAGAAC5	0,154270743	3,68380122	0,783340262	0,955173253	T2D vs Control
hsa-miR-486-5p_TCCTGTACTGAGCTGCCC5	0,106013477	3,88539215	0,78379336	0,955173253	T2D vs Control
hsa-miR-215-5p_ATGACCTATGAATTGACAGA5	0,142822154	4,790158483	0,787661143	0,955315231	T2D vs Control
hsa-miR-2110_TTGGGGAAACGGCCGCTGAG5	0,153285986	2,165591375	0,787911015	0,955315231	T2D vs Control
hsa-miR-223-3p_TGTCAGTTTGTCAAATACCCCAA5	-0,051596744	10,91791178	0,789711941	0,955315231	T2D vs Control
hsa-miR-486-5p_CTGTACTGAGCTGCCCCGA5	-0,053472831	7,583597035	0,789920051	0,955315231	T2D vs Control
hsa-miR-125a-5p_TCCCTGAGACCCTTTAACCTGT5	-0,032122462	11,48945871	0,792407943	0,955315231	T2D vs Control
hsa-miR-342-3p_TCTCACACAGAAATCGCACCCGTCA5	-0,148893684	3,422364811	0,792888508	0,955315231	T2D vs Control
hsa-miR-146a-5p_TGAGAACTGAATTCCATGGG5	-0,141955179	2,424854764	0,794124784	0,955315231	T2D vs Control
hsa-miR-1908-5p_CGGCGGGGACGGCGATTGGT5	0,159103886	2,686402078	0,796349414	0,955315231	T2D vs Control
hsa-miR-99a-5p_AACCCGTAGATCCGATCTTG5	0,047270625	8,041845005	0,79747611	0,955315231	T2D vs Control
hsa-miR-1301-3p_TTGCAGCTGCCTGGGAGTGA5	-0,144317995	3,347749467	0,797531674	0,955315231	T2D vs Control
hsa-miR-224-5p_CAAGTCACTAGTGGTTCCGTTT5	0,144693874	2,885758309	0,800950611	0,955315231	T2D vs Control
hsa-let-7d-5p_AGAGGTAGTAGGTTGCATAG5	0,047737596	8,573362788	0,801267157	0,955315231	T2D vs Control
hsa-miR-191-5p_ACGGAATCCCAAAAGCAGCT5	0,143973776	1,904502114	0,801623853	0,955315231	T2D vs Control
hsa-miR-10b-5p_ACCCTGTAGAACCGAATTTGT5	-0,055558112	6,785991828	0,801980928	0,955315231	T2D vs Control
hsa-miR-146a-5p_TGAGAACTGAATTCCATGGGTTGT5	0,073390893	7,265118969	0,802643323	0,955315231	T2D vs Control
hsa-let-7g-5p_TGAGGTAGTAGTTTGTACAGTT5	-0,033024326	11,23391478	0,805468779	0,955315231	T2D vs Control
hsa-let-7i-5p_GAGGTAGTAGTTTGTGCTG5	-0,14278377	2,272109077	0,805766195	0,955315231	T2D vs Control
hsa-miR-423-3p_AAGCTCGGTCTGAGGCCCCTCAGT5	0,060384478	7,286433576	0,806790408	0,955315231	T2D vs Control
hsa-miR-495-3p_AAACAAACATGGTGCACTTCTT5	-0,152301171	2,485368847	0,807325991	0,955315231	T2D vs Control
hsa-miR-125a-5p_TCCCTGAGACCCTTTAACCT5	0,06132191	6,34415738	0,807882642	0,955315231	T2D vs Control
hsa-let-7a-5p_TGAGGTAGTAGGTTGTATAGT5	-0,024032246	11,86713156	0,812769747	0,959670353	T2D vs Control
hsa-miR-223-3p_GTCAGTTTGTCAAATACCCCA5	-0,072576269	6,454169749	0,816046553	0,962114057	T2D vs Control
hsa-miR-486-5p_TGTACTGAGCTGCCCCGA5	-0,115573349	4,617811312	0,819092185	0,964278392	T2D vs Control
hsa-let-7b-3p_CTATACAACCTACTGCCTTC5	-0,12278484	3,159200497	0,822169119	0,965427969	T2D vs Control
hsa-miR-181b-5p_AACATTCATTGCTGTCGGTG5	-0,127133116	2,058689202	0,823283092	0,965427969	T2D vs Control
hsa-miR-23b-3p_ATCACATTGCCAGGGATTACCAC5	0,104107225	5,113281158	0,824804102	0,965427969	T2D vs Control
hsa-miR-425-5p_AATGACACGATCACTCCCGTTG5	0,02796249	9,36928751	0,827054933	0,965427969	T2D vs Control
hsa-miR-181a-5p_AACATTCAACGCTGTCGGTG5	0,054616792	6,983532365	0,828421728	0,965427969	T2D vs Control
hsa-miR-361-5p_TTATCAGAATCTCCAGGGGTACT5	0,121136752	2,611707431	0,829147758	0,965427969	T2D vs Control
hsa-miR-191-5p_CAACGGAATCCCAAAAGCAG5	-0,024223985	10,17632421	0,829689205	0,965427969	T2D vs Control
hsa-miR-191-5p_CAACGGAATCCCAAAAGC5	-0,12043359	3,190312182	0,829759296	0,965427969	T2D vs Control
hsa-miR-106b-3p_CCGCACTGTGGGTACTTGCT5	0,068361039	6,396206525	0,834458229	0,967489802	T2D vs Control
hsa-miR-423-3p_AGCTCGGTCTGAGGCCCCTCAGT5	-0,026128429	11,76682308	0,837486716	0,967489802	T2D vs Control
hsa-miR-223-3p_GTCAGTTTGTCAAATACCCCAA5	-0,056235758	7,740128632	0,83765781	0,967489802	T2D vs Control
hsa-miR-22-3p_AAGCTGCCAGTTGAAGAACTGTT5	0,113636056	2,793276847	0,838809928	0,967489802	T2D vs Control
hsa-miR-92a-3p_ACTTGTCCCGGCCTGT5	0,116699687	2,134254049	0,840311371	0,967489802	T2D vs Control
hsa-miR-29a-3p_TAGCACCATCTGAAATCGGTTAT5	0,114908566	2,486065735	0,840897195	0,967489802	T2D vs Control
hsa-miR-140-3p_TACCACAGGGTAGAACCACG5	-0,077372958	5,026346909	0,841429674	0,967489802	T2D vs Control
hsa-miR-144-3p_TACAGTATAGATGATGTAC5	0,055516752	6,460339014	0,84268637	0,967489802	T2D vs Control
hsa-miR-182-5p_TTTGGCAATGGTAGAACTC5	-0,116341554	2,382812326	0,84365477	0,967489802	T2D vs Control
hsa-miR-451a_AACCGTTACCATTACTGAGTTT5	0,111428946	2,400911204	0,843681463	0,967489802	T2D vs Control
hsa-miR-224-5p_CAAGTCACTAGTGGTTCCGTTTAG5	-0,10661114	4,65780231	0,846938736	0,967489802	T2D vs Control
hsa-miR-30e-5p_TGTAAACATCCTTGACTGGAA5	0,042695488	6,536675988	0,847581597	0,967489802	T2D vs Control
hsa-miR-450b-5p_TTTTGCAATATGTTCCTGAAT5	-0,085276928	5,016886951	0,847826646	0,967489802	T2D vs Control
hsa-miR-142-5p_CATAAAGTAGAAAGCACTA5	0,100463451	2,153538022	0,850052394	0,967489802	T2D vs Control

hsa-miR-100-5p_AACCCGTAGATCCGAACTTG5	0,058852079	5,941970306	0,850427458	0,967489802	T2D vs Control
hsa-miR-22-3p_AAGCTGCCAGTTGAAGAAC5	0,035882739	8,203664635	0,851431425	0,967489802	T2D vs Control
hsa-miR-323b-3p_CCCAATACACGGTCGACCTCT5	-0,092724954	5,853793118	0,852167931	0,967489802	T2D vs Control
hsa-miR-15b-5p_TAGCAGCACATCATGGTTT5	0,031571183	8,514548835	0,85512956	0,969404473	T2D vs Control
hsa-let-7a-5p_TTGAGGTAGTAGGTTGTATAGTT5	0,101737605	1,956823269	0,857892811	0,969404473	T2D vs Control
hsa-miR-186-5p_CAAAGAATTCTCCTTTTGGGCTTT5	-0,094580655	4,003798957	0,859362845	0,969404473	T2D vs Control
hsa-miR-92a-3p_GCACTTGTCCCGGCCTGT5	-0,075185178	4,476222162	0,861967313	0,969404473	T2D vs Control
hsa-miR-140-3p_ACCACAGGGTAGAACCACG5	0,096718305	2,665108795	0,862914716	0,969404473	T2D vs Control
hsa-miR-26b-5p_TCAAGTAATTCAGGATAGGTT5	-0,095105699	3,747955651	0,864269473	0,969404473	T2D vs Control
hsa-miR-505-3p_GTCAACACTTGCTGGTTTCCTCT5	-0,102211578	2,066469778	0,864506051	0,969404473	T2D vs Control
hsa-miR-584-5p_TTATGGTTTGCCTGGGACTG5	0,092701711	2,558465624	0,864589445	0,969404473	T2D vs Control
hsa-miR-320a-3p_AAAAGCTGGGTTGAGAGGGCGAAAA5	0,095630738	3,186668725	0,866636521	0,969404473	T2D vs Control
hsa-miR-342-3p_TCTCACACAGAAATCGCACCC5	-0,095537264	2,049205595	0,86680219	0,969404473	T2D vs Control
hsa-miR-320b_AAAAGCTGGGTTGAGAGGGC5	0,142471786	5,061955788	0,867233863	0,969404473	T2D vs Control
hsa-miR-148b-3p_TCAGTGCATCACAGAACTTTGT5	-0,033521765	6,965383646	0,870009176	0,970994232	T2D vs Control
hsa-miR-181a-5p_AACATTCAACGCTGTCGGT5	0,060129215	5,505188114	0,871092693	0,970994232	T2D vs Control
hsa-miR-23a-3p_ATCACATTGCCAGGGATTT5	0,028130888	7,810237088	0,873202375	0,971986443	T2D vs Control
hsa-let-7a-5p_AGGTAGTAGGTTGTATAGTT5	0,091227652	1,879378714	0,874637603	0,972226178	T2D vs Control
hsa-miR-30c-5p_TGTAAACATCCTACACTCTCAG5	0,070405141	4,767658059	0,87586064	0,972229708	T2D vs Control
hsa-miR-532-5p_CATGCCTTGAGTGTAGGACCGT5	0,070614003	4,730944055	0,878283981	0,973563746	T2D vs Control
hsa-miR-30e-5p_TGTAAACATCCTTGACTGGAAG5	0,026967479	7,619075745	0,88040769	0,974562402	T2D vs Control
hsa-miR-30a-5p_TGTAAACATCCTCGACTGG5	0,071464447	4,494972654	0,882296393	0,974882138	T2D vs Control
hsa-miR-182-5p_TTTGGCAATGGTAGAACTCAC5	0,081073955	3,148572393	0,884646149	0,974882138	T2D vs Control
hsa-miR-483-5p_AAGACGGGAGGAAAGAAGGGAG5	0,051273088	6,618430366	0,885334006	0,974882138	T2D vs Control
hsa-miR-421_ATCAACAGACATTAATTGGGCGC5	-0,077157913	2,04052248	0,886743749	0,974882138	T2D vs Control
hsa-miR-148a-3p_TCAGTGCACTACAGAACTTT5	-0,0790152	4,280926656	0,886812484	0,974882138	T2D vs Control
hsa-miR-142-5p_CCCATAAAGTAGAAAGCAC5	-0,049933015	5,201334155	0,888367659	0,97524659	T2D vs Control
hsa-miR-423-3p_AGCTCGGTCTGAGGCCCCTCA5	-0,045719825	6,171912051	0,890557947	0,976306305	T2D vs Control
hsa-miR-3615_TCTCTCGGCTCCTCGCGGCTCG5	0,018181297	8,091694617	0,895590676	0,978722095	T2D vs Control
hsa-miR-92b-3p_TATTGCACTCGTCCCGGCCTC5	-0,064437929	3,886574178	0,896798511	0,978722095	T2D vs Control
hsa-miR-486-5p_TCCTGTACTGAGCTGCCCCGAGC5	-0,028862074	6,188232343	0,897673514	0,978722095	T2D vs Control
hsa-miR-101-3p_TACAGTACTGTGATAACTGA5	-0,064615651	4,333316093	0,90081556	0,978722095	T2D vs Control
hsa-miR-3613-5p_TGTTGTACTTTTTTTTTTGTT5	-0,049431341	5,269511804	0,901709818	0,978722095	T2D vs Control
hsa-miR-193a-5p_TGGGTCTTTGCGGGCGAGATGA5	0,020932531	8,630407946	0,903306413	0,978722095	T2D vs Control
hsa-miR-30a-5p_TGTAAACATCCTCGACTGGA5	0,066270843	3,19956619	0,903736773	0,978722095	T2D vs Control
hsa-miR-339-5p_TCCCTGTCCTCCAGGAGCTCACG5	-0,069996929	3,416847552	0,90373801	0,978722095	T2D vs Control
hsa-miR-574-3p_CACGCTCATGCACACACCCA5	0,06576322	3,365756558	0,905105769	0,978722095	T2D vs Control
hsa-miR-92a-3p_TATTGCACTTGTCCCGGCCT5	0,01812607	10,27945719	0,905169155	0,978722095	T2D vs Control
hsa-miR-10a-5p_TACCCTGTAGATCCGAATTTGT5	0,012573145	10,44682905	0,913426527	0,978722095	T2D vs Control
hsa-miR-17-5p_CAAAGTGCTTACAGTGCAGGTAG5	-0,061436059	3,276961308	0,913530779	0,978722095	T2D vs Control
hsa-miR-2110_TTGGGGAAACGGCCGCTGAGTGA5	0,034766107	6,066188802	0,913708849	0,978722095	T2D vs Control
hsa-miR-409-3p_GAATGTTGCTCGGTGAACCCCTTT5	0,065169857	4,139609499	0,914773587	0,978722095	T2D vs Control
hsa-miR-23a-5p_GGGGTTCCTGGGGATGGGATTT5	0,056214821	3,81817711	0,916659637	0,978722095	T2D vs Control
hsa-miR-10b-5p_TACCCTGTAGAACCGAATTT5	0,041200901	5,2763128	0,91698892	0,978722095	T2D vs Control
hsa-miR-20a-5p_TAAAGTGCTTATAGTGCAGGTAG5	-0,048450671	4,004629847	0,917389112	0,978722095	T2D vs Control
hsa-miR-92a-3p_TATTGCACTTGTCCCGG5	0,046050228	4,594134091	0,917681419	0,978722095	T2D vs Control
hsa-miR-24-3p_TGGCTCAGTTCAGCAGGAAC5	-0,012097169	10,07194833	0,918395841	0,978722095	T2D vs Control
hsa-let-7a-5p_GAGGTAGTAGGTTGTATAGTT5	-0,026486727	7,081547935	0,919262936	0,978722095	T2D vs Control
hsa-miR-16-5p_CTAGCAGCACGTAAATATTGGCG5	-0,041253145	4,959212286	0,924616641	0,978722095	T2D vs Control
hsa-miR-342-3p_TCTCACACAGAAATCGCACCCGTC5	0,018851473	7,583667414	0,925333989	0,978722095	T2D vs Control
hsa-miR-223-5p_CGTGTATTTGACAAGCTGAGTT5	0,045170041	3,93706767	0,926370783	0,978722095	T2D vs Control
hsa-miR-21-5p_AGCTTATCAGACTGATGTTGAC5	-0,05480559	3,189772648	0,928379511	0,978722095	T2D vs Control
hsa-let-7f-5p_GAGGTAGTAGATTGTATAGT5	-0,051745417	3,023408308	0,931138337	0,978722095	T2D vs Control
hsa-miR-103a-3p_AGCAGCATTGTACAGGGCT5	0,074035159	5,118424827	0,931196394	0,978722095	T2D vs Control
hsa-miR-409-3p_GAATGTTGCTCGGTGAACCCCTT5	-0,049268516	5,101558557	0,931413301	0,978722095	T2D vs Control
hsa-miR-320a-3p_AAAAGCTGGGTTGAGAGGGCGAA5	-0,010163562	9,770949231	0,932350733	0,978722095	T2D vs Control
hsa-miR-574-3p_CACGCTCATGCACACACCCACA5	0,024986041	6,300877473	0,932352795	0,978722095	T2D vs Control
hsa-miR-374a-5p_TTATAATACAACCTGATAAGTG5	-0,037620114	4,272038394	0,934013878	0,978722095	T2D vs Control
hsa-miR-4433b-5p_ATGTCCCACCCCCACTCCTG5	-0,046618137	2,006165102	0,93413729	0,978722095	T2D vs Control
hsa-miR-15b-5p_TAGCAGCACATCATGGTTTACA5	0,02540966	6,32576652	0,934744099	0,978722095	T2D vs Control
hsa-miR-10a-5p_ACCCTGTAGATCCGAATTTGT5	-0,017949553	7,643285241	0,934773715	0,978722095	T2D vs Control

hsa-miR-629-5p_TGGGTTTACGTTGGGAGAACT5	0,018656237	7,034835335	0,937762928	0,978722095	T2D vs Control
hsa-miR-10a-5p_TACCCTGTAGATCCGAATT5	-0,041392816	3,933865051	0,939217401	0,978722095	T2D vs Control
hsa-miR-4433b-5p_TGTCCCACCCCCACTCCTG5	-0,047631943	2,497163245	0,939356288	0,978722095	T2D vs Control
hsa-miR-144-5p_GGATATCATCATATACTGTAAG5	-0,043457516	4,224705339	0,940436252	0,978722095	T2D vs Control
hsa-miR-664a-5p_ACTGGCTAGGGAAAATGATTGG5	0,039815381	3,587998944	0,940466638	0,978722095	T2D vs Control
hsa-miR-335-3p_TTTTTCATTATTGCTCCTGACC5	0,041160584	4,076732498	0,94128496	0,978722095	T2D vs Control
hsa-miR-143-3p_TGAGATGAAGCACTGTAGCTCA5	0,021931516	6,738882834	0,941881865	0,978722095	T2D vs Control
hsa-miR-151a-3p_TACTAGACTGAAGCTCCTTGAGG5	0,034669413	4,127573648	0,947705316	0,98252787	T2D vs Control
hsa-miR-23a-3p_ATCACATTGCCAGGGATTTCCAA5	-0,021453239	6,530990344	0,948862606	0,98252787	T2D vs Control
hsa-miR-339-3p_TGAGCGCCTCGACGACAGAGCCG5	-0,035882166	4,16247252	0,949242735	0,98252787	T2D vs Control
hsa-let-7i-5p_GAGGTAGTAGTTTGTGCTGTT5	0,034860832	3,257044134	0,95144278	0,98352775	T2D vs Control
hsa-let-7b-5p_TGAGGTAGTAGGTTGTGTGGTT5	0,005176095	12,77995871	0,956381272	0,985619829	T2D vs Control
hsa-miR-148a-3p_TCAGTGCACTACAGAACTTTGT5	-0,007683681	9,289411705	0,956839485	0,985619829	T2D vs Control
hsa-miR-186-5p_CAAAGAATTCTCCTTTTGGGCTT5	-0,017592969	6,345307365	0,957176597	0,985619829	T2D vs Control
hsa-miR-409-3p_CGAATGTTGCTCGGTGAACCCCTT5	-0,032325833	3,646109852	0,958594176	0,985805882	T2D vs Control
hsa-miR-543_AAACATTCGCGGTGCACTTCTTT5	-0,030936752	3,173881718	0,961139466	0,987149684	T2D vs Control
hsa-miR-191-5p_CAACGGAATCCCAAAAGCAGCTGT5	-0,020503542	5,688164696	0,963736782	0,987303796	T2D vs Control
hsa-miR-486-5p_ATCCTGTACTGAGCTGCCCCGA5	0,007929617	8,988899635	0,964055982	0,987303796	T2D vs Control
hsa-miR-106b-5p_TAAAGTGCTGACAGTGCAGAT5	-0,023417183	2,655083548	0,965005843	0,987303796	T2D vs Control
hsa-let-7a-5p_GAGGTAGTAGGTTGTATAGT5	0,020088002	4,701370889	0,966701975	0,987771121	T2D vs Control
hsa-miR-501-3p_AATGCACCCGGGCAAGGAT5	-0,021301637	2,760223516	0,970096336	0,989970269	T2D vs Control
hsa-miR-10a-5p_TACCCTGTAGATCCGAATTT5	-0,017042662	4,308439737	0,973076521	0,99081673	T2D vs Control
hsa-miR-484_TCAGGCTCAGTCCCCTCCCGATA5	0,014846268	4,637634197	0,97341217	0,99081673	T2D vs Control
hsa-miR-92a-3p_TTGCACTTGTCCCGGCCTGT5	-0,00416783	8,384006303	0,976618971	0,992205696	T2D vs Control
hsa-miR-23b-3p_ATCACATTGCCAGGGATTACC5	0,00970288	5,483511586	0,977407638	0,992205696	T2D vs Control
hsa-miR-335-3p_GTTTTTCATTATTGCTCCTGACC5	-0,014999712	2,935136252	0,980036982	0,992205696	T2D vs Control
hsa-miR-223-3p_TGTCAGTTTGTCAAATACC5	-0,009693729	5,409428462	0,980706341	0,992205696	T2D vs Control
hsa-miR-92a-3p_TATTGCACTTGTCCCGGCCTGTG5	0,003975806	7,105169059	0,981001365	0,992205696	T2D vs Control
hsa-miR-374b-5p_ATATAATACAACCTGCTAAGTG5	0,007969563	2,723967557	0,988576819	0,997743241	T2D vs Control
hsa-miR-432-5p_TCTTGGAGTAGGTCATTGGGTGG5	-0,007196956	3,502755168	0,990952385	0,997743241	T2D vs Control
hsa-miR-30d-5p_TGTAAACATCCCCGACTGGA5	0,001542539	9,18587305	0,991800954	0,997743241	T2D vs Control
hsa-let-7a-5p_TGAGGTAGTAGGTTGTATAGTTTT5	-0,005778441	3,044731157	0,992291854	0,997743241	T2D vs Control
hsa-miR-424-5p_CAGCAGCAATTCATGTTTTGA5	0,005238741	2,646439112	0,992735747	0,997743241	T2D vs Control
hsa-miR-16-2-3p_ACCAATATTACTGTGCTGCTTT5	-0,000836012	7,879139867	0,995824709	0,998575167	T2D vs Control
hsa-miR-486-5p_CTGTACTGAGCTGCCCCG5	0,002730641	3,40982355	0,99614149	0,998575167	T2D vs Control
hsa-miR-146a-5p_TGAGAACTGAATTCCATGGGTTG5	0,000562825	7,878355546	0,997850234	0,998575167	T2D vs Control
hsa-miR-21-5p_TAGCTTATCAGACTGATGT5	0,000858934	4,699515355	0,998575167	0,998575167	T2D vs Control
hsa-miR-140-3p_TACCACAGGGTAGAACCACGG	-1,76085605	5,398078089	1,13793E-06	0,000900104	T1D vs T2D Time-adjusted
hsa-miR-23b-3p_ATCACATTGCCAGGGATTAC	-2,404551085	2,73633577	6,09394E-06	0,001888021	T1D vs T2D Time-adjusted
hsa-miR-223-3p_TGTCAGTTTGTCAAATACC	-1,904976664	5,277889688	7,16064E-06	0,001888021	T1D vs T2D Time-adjusted
hsa-miR-142-5p_CCCATAAAGTAGAAAGCA	-2,283260967	2,402819725	1,545E-05	0,003055247	T1D vs T2D Time-adjusted
hsa-miR-183-5p_TATGGCACTGGTAGAATTCAC	-2,377795903	2,19049992	3,64587E-05	0,00576776	T1D vs T2D Time-adjusted
hsa-miR-29a-3p_CTAGCACCATCTGAAATCGG	-2,175542945	3,1053029	8,92073E-05	0,010538057	T1D vs T2D Time-adjusted
hsa-miR-16-2-3p_ACCAATATTACTGTGCTGCT	-1,750528461	4,640125469	9,32571E-05	0,010538057	T1D vs T2D Time-adjusted
hsa-miR-25-3p_CATTGCACTTGTCTCGGT	-1,949421452	3,82970246	0,000122384	0,012100728	T1D vs T2D Time-adjusted
hsa-miR-25-3p_ATTGCACTTGTCTCGGTCT	-2,05505383	3,534900996	0,000182045	0,015999766	T1D vs T2D Time-adjusted
hsa-miR-421_ATCAACAGACATTAATTGGGCG	-2,064277817	2,15305225	0,000286784	0,019720025	T1D vs T2D Time-adjusted
hsa-miR-363-3p_ATTGCACGGTATCCATCTGT	-1,841520907	4,449987277	0,000287117	0,019720025	T1D vs T2D Time-adjusted
hsa-miR-130b-3p_CAGTGCAATGATGAAAGGGCA	-1,592210629	4,356329159	0,000301561	0,019720025	T1D vs T2D Time-adjusted
hsa-miR-181b-5p_AACATTCATTGCTGTCGGTGGGT	-2,034643372	3,495629927	0,000324096	0,019720025	T1D vs T2D Time-adjusted
hsa-miR-101-3p_GTACAGTACTGTGATAACTG	-1,486989634	5,567307108	0,000374365	0,01978664	T1D vs T2D Time-adjusted
hsa-miR-361-3p_TCCCCCAGGTGTGATTCTGATT	-1,703489442	3,75062111	0,000400746	0,01978664	T1D vs T2D Time-adjusted
hsa-miR-151a-3p_TACTAGACTGAAGCTCCTTGAGG	-1,827876206	3,949800351	0,000401178	0,01978664	T1D vs T2D Time-adjusted
hsa-miR-451a_GAAACCGTTACCATTACTGAG	-1,946464463	3,175112451	0,00042525	0,01978664	T1D vs T2D Time-adjusted
hsa-miR-139-5p_TCTACAGTGCACGTGTCTCCA	-1,615006902	4,89012497	0,00053381	0,022265069	T1D vs T2D Time-adjusted
hsa-miR-140-3p_TACCACAGGGTAGAACCACGGACA	-1,686662047	3,592791188	0,000534812	0,022265069	T1D vs T2D Time-adjusted
hsa-miR-423-5p_CTGAGGGGCAGAGAGCGAGACT	-1,999793371	2,738837203	0,000680895	0,026929386	T1D vs T2D Time-adjusted
hsa-miR-584-5p_TTATGGTTTGCCTGGGACT	-1,558959969	4,566540106	0,001006011	0,037588601	T1D vs T2D Time-adjusted
hsa-let-7f-5p_TGAGGTAGTAGATTGTATA	-1,840742306	3,718372609	0,001102451	0,037588601	T1D vs T2D Time-adjusted
hsa-miR-186-5p_CAAAGAATTCTCCTTTTGGGC	-1,156180409	6,378401215	0,001138421	0,037588601	T1D vs T2D Time-adjusted
hsa-miR-140-3p_ACCACAGGGTAGAACCACGGACA	-1,525129568	4,273604017	0,001172802	0,037588601	T1D vs T2D Time-adjusted

hsa-miR-25-3p_ATTGCACTTGTCTCGGTCTG	-1,746929143	3,333905317	0,001245952	0,037588601	T1D vs T2D Time-adjusted
hsa-miR-7-5p_TGGAAGACTAGTGATTTTGTTG	-1,662512335	3,867797174	0,001338638	0,037588601	T1D vs T2D Time-adjusted
hsa-miR-335-5p_TCAAGAGCAATAACGAAAAATGT	-1,162113372	6,087791959	0,001376349	0,037588601	T1D vs T2D Time-adjusted
hsa-miR-451a_AAACCGTTACCATTACTG	-1,774860628	2,543007428	0,00139571	0,037588601	T1D vs T2D Time-adjusted
hsa-miR-93-5p_CAAAGTGCTGTTCGTGCAGGT	-1,187254538	5,072528945	0,001403334	0,037588601	T1D vs T2D Time-adjusted
hsa-miR-106b-3p_CCGCACTGTGGGTACTTGCTG	-1,566382164	4,149873679	0,001425611	0,037588601	T1D vs T2D Time-adjusted
hsa-miR-192-5p_TGACCTATGAATTGACAGCCAGT	-1,674114725	3,442809963	0,001612628	0,041148019	T1D vs T2D Time-adjusted
hsa-miR-128-3p_TCACAGTGAACCGGTCTCTTTT	-1,431007573	5,206370676	0,001786956	0,043947823	T1D vs T2D Time-adjusted
hsa-let-7e-5p_TGAGGTAGGAGGTTGTATAGT	-1,478214152	4,76183341	0,001833474	0,043947823	T1D vs T2D Time-adjusted
hsa-miR-186-5p_CAAAGAATTCTCCTTTTGGGCT	-0,48751642	8,984782262	0,002094466	0,047841705	T1D vs T2D Time-adjusted
hsa-miR-192-5p_TGACCTATGAATTGACAGCCAG	-1,659894706	3,649860914	0,00211689	0,047841705	T1D vs T2D Time-adjusted
hsa-miR-27b-3p_TTCACAGTGGCTAAGTTCTGCA	-1,667833835	3,816952744	0,002213528	0,048636126	T1D vs T2D Time-adjusted
hsa-miR-92a-3p_TATTGCACTTGTCCCGG	-1,53278054	4,506722639	0,002463199	0,051305698	T1D vs T2D Time-adjusted
hsa-miR-93-5p_CAAAGTGCTGTTCGTGCAGGTA	-1,095781091	5,408235597	0,002532168	0,051305698	T1D vs T2D Time-adjusted
hsa-miR-185-5p_TGGAGAGAAAGGCAGTTCCTG	-1,560945694	4,445039784	0,002566097	0,051305698	T1D vs T2D Time-adjusted
hsa-miR-23a-3p_TCACATTGCCAGGGATTTCCAAC	-1,736948018	1,909179417	0,002594473	0,051305698	T1D vs T2D Time-adjusted
hsa-let-7a-5p_TGAGGTAGTAGGTTGTATA	-1,111346751	5,691720869	0,003013076	0,058130326	T1D vs T2D Time-adjusted
hsa-miR-181a-2-3p_ACCACTGACCGTTGACTGT	-1,78257078	2,626525168	0,003096093	0,058309748	T1D vs T2D Time-adjusted
hsa-miR-30d-5p_TGTAAACATCCCCGACTGGA	-0,536344376	9,147110661	0,003256568	0,058967571	T1D vs T2D Time-adjusted
hsa-miR-150-3p_CTGGTACAGGCCTGGGGGACA	-1,728892197	4,065180007	0,003280118	0,058967571	T1D vs T2D Time-adjusted
hsa-miR-30a-5p_TGTAAACATCCTCGACTGGAA	-0,672933806	7,206633348	0,00388576	0,066716231	T1D vs T2D Time-adjusted
hsa-miR-505-3p_CGTCAACACTTGCTGGTTTCCT	-1,612908602	3,930461517	0,003900959	0,066716231	T1D vs T2D Time-adjusted
hsa-miR-125a-5p_TCCCTGAGACCCTTTAACCT	-0,789267425	6,333504859	0,003964176	0,066716231	T1D vs T2D Time-adjusted
hsa-miR-139-5p_TCTACAGTGCACGTGTCTCCAGT	-0,789635547	6,693764684	0,004287026	0,070646615	T1D vs T2D Time-adjusted
hsa-miR-30d-5p_GTAAACATCCCCGACTGGAA	-1,284474908	4,880801026	0,004428764	0,070676683	T1D vs T2D Time-adjusted
hsa-miR-10a-5p_TACCCTGTAGATCCGAATTTGTGT	-1,679067484	2,457813121	0,004467553	0,070676683	T1D vs T2D Time-adjusted
hsa-miR-2110_TTGGGGAAACGGCCGCTGAGTG	-1,731696941	3,022786946	0,004747153	0,073627417	T1D vs T2D Time-adjusted
hsa-miR-29a-3p_TAGCACCATCTGAAATCGGTTAT	-1,671552969	2,458427946	0,005066902	0,077075377	T1D vs T2D Time-adjusted
hsa-miR-423-3p_AAGCTCGGTCTGAGGCCCCTCAG	-1,473215502	3,379819075	0,006033636	0,090049176	T1D vs T2D Time-adjusted
hsa-miR-30c-5p_TGTAAACATCCTACACTCTCAGC	-0,699791477	6,576373518	0,006245156	0,091479974	T1D vs T2D Time-adjusted
hsa-miR-25-3p_ATTGCACTTGTCTCGGTCTGA	-1,00269991	5,817358164	0,006484643	0,093260959	T1D vs T2D Time-adjusted
hsa-miR-542-3p_TGTGACAGATTGATAACTGA	-1,645177673	2,0314731	0,007219825	0,099993568	T1D vs T2D Time-adjusted
hsa-miR-10a-5p_TACCCTGTAGATCCGAATTTGTG	-0,681342877	7,331499904	0,007327875	0,099993568	T1D vs T2D Time-adjusted
hsa-miR-99a-5p_AACCCGTAGATCCGATCTTGTG	-0,976786117	5,465860433	0,007332019	0,099993568	T1D vs T2D Time-adjusted
hsa-miR-2110_TTGGGGAAACGGCCGCTGAGT	-0,92079672	6,041635722	0,007469369	0,100140188	T1D vs T2D Time-adjusted
hsa-let-7b-5p_GAGGTAGTAGGTTGTGTGGTTT	-1,475665946	3,820264571	0,007728457	0,100792148	T1D vs T2D Time-adjusted
hsa-miR-423-3p_AAGCTCGGTCTGAGGCCCCTCA	-1,523162654	2,829290184	0,007886016	0,100792148	T1D vs T2D Time-adjusted
hsa-miR-140-3p_TACCACAGGGTAGAACCACGGA	-0,613746212	7,862508769	0,007930132	0,100792148	T1D vs T2D Time-adjusted
hsa-miR-409-3p_GAATGTTGCTCGGTGAACCCCTTT	-1,651675065	3,983254098	0,008027693	0,100792148	T1D vs T2D Time-adjusted
hsa-miR-142-5p_CCCATAAAGTAGAAAGCACTA	-1,072580566	5,238891455	0,008440698	0,101549516	T1D vs T2D Time-adjusted
hsa-miR-221-3p_AGCTACATTGTCTGCTGGGTTTC	-0,973584094	6,662543722	0,008551912	0,101549516	T1D vs T2D Time-adjusted
hsa-miR-543_AAACATTCGCGGTGCACTTCTT	-1,584990575	3,525271253	0,008639968	0,101549516	T1D vs T2D Time-adjusted
hsa-miR-1294_TGTGAGGTTGGCATTGTTGT	-1,57243014	2,446198713	0,00869638	0,101549516	T1D vs T2D Time-adjusted
hsa-let-7b-5p_TGAGGTAGTAGGTTGTGT	-1,081965185	5,334260563	0,00872992	0,101549516	T1D vs T2D Time-adjusted
hsa-miR-1180-3p_TTTCCGGCTCGCGTGGGTGTGT	-1,465260902	2,841243966	0,009129726	0,10370685	T1D vs T2D Time-adjusted
hsa-miR-28-3p_CACTAGATTGTGAGCTCCTGGA	-0,737220995	6,790249103	0,009207831	0,10370685	T1D vs T2D Time-adjusted
hsa-miR-29c-3p_TAGCACCATTTGAAATCGG	-1,528343245	3,275982402	0,009348966	0,10370685	T1D vs T2D Time-adjusted
hsa-let-7i-5p_GAGGTAGTAGTTTGTGCTGTT	-1,583442538	3,203293849	0,009899306	0,10370685	T1D vs T2D Time-adjusted
hsa-miR-183-5p_ATGGCACTGGTAGAATTCACT	-1,266334896	4,551574409	0,00995411	0,10370685	T1D vs T2D Time-adjusted
hsa-miR-451a_AACCGTTACCATTACTGAGTT	-0,912643235	6,235439111	0,00997023	0,10370685	T1D vs T2D Time-adjusted
hsa-miR-126-5p_ATTATTACTTTTGGTACGCGCT	-1,55253668	2,229095104	0,0101063	0,10370685	T1D vs T2D Time-adjusted
hsa-miR-142-5p_CATAAAGTAGAAAGCACTA	-1,4860118	2,092424464	0,010157463	0,10370685	T1D vs T2D Time-adjusted
hsa-miR-484_CAGGCTCAGTCCCCTCCCGAT	-1,446436883	2,348599265	0,010278603	0,10370685	T1D vs T2D Time-adjusted
hsa-miR-1306-5p_CCACCTCCCCTGCAAACGT	1,78416683	2,609394089	0,010353458	0,10370685	T1D vs T2D Time-adjusted
hsa-miR-22-3p_AAGCTGCCAGTTGAAGAACTG	-0,51449096	9,199421356	0,010357574	0,10370685	T1D vs T2D Time-adjusted
hsa-miR-1180-3p_TTTCCGGCTCGCGTGGGTGT	-1,344976128	4,031270991	0,011112706	0,109876882	T1D vs T2D Time-adjusted
hsa-miR-142-5p_CATAAAGTAGAAAGCACT	-0,829129936	5,448401815	0,011494352	0,111892788	T1D vs T2D Time-adjusted
hsa-miR-10a-5p_ACCCTGTAGATCCGAATTTGTG	-0,674963179	6,333214322	0,011731069	0,111892788	T1D vs T2D Time-adjusted
hsa-let-7a-5p_GAGGTAGTAGGTTGTATAG	-1,097208924	5,313092713	0,011740963	0,111892788	T1D vs T2D Time-adjusted
hsa-miR-106b-3p_CCGCACTGTGGGTACTTGCT	-0,941063495	6,292504346	0,011904697	0,112102564	T1D vs T2D Time-adjusted
hsa-miR-93-5p_CAAAGTGCTGTTCGTGCAGGTAG	-0,331653038	9,966857204	0,012265899	0,113781659	T1D vs T2D Time-adjusted

hsa-miR-744-5p_TGCGGGGCTAGGGCTAACAGC	-1,266502175	3,989518529	0,012370699	0,113781659	T1D vs T2D Time-adjusted
hsa-miR-340-3p_TCCGTCTCAGTTACTTTATAGCC	-1,554974748	3,011838282	0,012692448	0,114247359	T1D vs T2D Time-adjusted
hsa-miR-361-5p_TTATCAGAATCTCCAGGGGTAC	-0,640673162	6,68645525	0,012710199	0,114247359	T1D vs T2D Time-adjusted
hsa-miR-451a_ACCGTTACCATTACTGAGT	-0,740580611	7,438165305	0,012963671	0,115216451	T1D vs T2D Time-adjusted
hsa-miR-125b-5p_TCCCTGAGACCCTAACTTGTGA	-1,000729262	5,621036711	0,013338785	0,117233101	T1D vs T2D Time-adjusted
hsa-miR-126-3p_TCGTACCGTGAGTAATAATGC	-0,654905927	6,39389942	0,014422646	0,125366079	T1D vs T2D Time-adjusted
hsa-miR-584-5p_TTATGGTTTGCCTGGGACTG	-1,432789117	2,451626756	0,015037039	0,129285849	T1D vs T2D Time-adjusted
hsa-miR-16-5p_TAGCAGCACGTAAATATTGGC	-0,343983514	9,966980138	0,01527559	0,129924643	T1D vs T2D Time-adjusted
hsa-miR-182-5p_TTTGGCAATGGTAGAACTCACACTG	-1,421916599	3,704867925	0,015573451	0,131048933	T1D vs T2D Time-adjusted
hsa-miR-30d-5p_GTAAACATCCCCGACTGGAAGCT	-0,719781304	6,871318415	0,016207164	0,133631083	T1D vs T2D Time-adjusted
hsa-miR-28-3p_CACTAGATTGTGAGCTCCTGG	-0,776022064	5,951344612	0,016218185	0,133631083	T1D vs T2D Time-adjusted
hsa-miR-22-3p_AAGCTGCCAGTTGAAGAA	-1,183380914	5,032500306	0,016400264	0,133738238	T1D vs T2D Time-adjusted
hsa-miR-425-5p_AATGACACGATCACTCCCGTT	-0,543190113	8,013037563	0,017104441	0,136936917	T1D vs T2D Time-adjusted
hsa-miR-140-3p_ACCACAGGGTAGAACCACGG	-1,318869789	3,499713471	0,017138754	0,136936917	T1D vs T2D Time-adjusted
hsa-miR-425-5p_AATGACACGATCACTCCCGTTG	-0,354782827	9,327235486	0,017798541	0,140786461	T1D vs T2D Time-adjusted
hsa-miR-484_CAGGCTCAGTCCCCTCCCGA	-1,488831262	3,800267749	0,018194228	0,142000965	T1D vs T2D Time-adjusted
hsa-miR-10b-5p_ACCCTGTAGAACCGAATTTGTGT	-1,317055977	3,358063133	0,018311123	0,142000965	T1D vs T2D Time-adjusted
hsa-miR-146a-5p_TGAGAACTGAATTCCATGGGTTGT	-0,664174172	7,268590607	0,018713466	0,142007519	T1D vs T2D Time-adjusted
hsa-miR-181a-2-3p_ACCACTGACCGTTGACTGTAC	-1,36065274	3,539126124	0,019057871	0,142007519	T1D vs T2D Time-adjusted
hsa-miR-21-5p_AGCTTATCAGACTGATGTTGA	-0,556036408	6,713348932	0,019219934	0,142007519	T1D vs T2D Time-adjusted
hsa-miR-502-3p_AATGCACCTGGGCAAGGATTCA	-0,985023475	4,779774283	0,019289934	0,142007519	T1D vs T2D Time-adjusted
hsa-miR-222-3p_AGCTACATCTGGCTACTGGGTCT	-0,747474157	6,477997223	0,019311564	0,142007519	T1D vs T2D Time-adjusted
hsa-miR-342-3p_TCACACAGAAATCGCACCCGTCA	-1,276586331	3,93860325	0,019442513	0,142007519	T1D vs T2D Time-adjusted
hsa-let-7b-5p_GAGGTAGTAGGTTGTGTGGT	-1,119418267	4,880278721	0,019884861	0,142007519	T1D vs T2D Time-adjusted
hsa-let-7d-3p_CTATACGACCTGCTGCCTTTC	0,275091317	10,31942736	0,01989145	0,142007519	T1D vs T2D Time-adjusted
hsa-miR-363-3p_AATTGCACGGTATCCATCTG	-0,465161284	8,30423721	0,01992773	0,142007519	T1D vs T2D Time-adjusted
hsa-miR-191-5p_AACGGAATCCCAAAAGCAGCTG	-0,746136088	5,768928318	0,020759693	0,146615335	T1D vs T2D Time-adjusted
hsa-miR-142-5p_CCCATAAAGTAGAAAGCACTAC	-0,635272924	6,93738446	0,020956087	0,146692607	T1D vs T2D Time-adjusted
hsa-miR-181a-5p_AACATTCAACGCTGTCGGTGAGT	-0,488417721	8,108797195	0,021676562	0,150404917	T1D vs T2D Time-adjusted
hsa-miR-181b-5p_AACATTCATTGCTGTCGGTGG	-0,912822692	5,17184581	0,021992257	0,150707971	T1D vs T2D Time-adjusted
hsa-miR-145-5p_GTCCAGTTTTCCCAGGAATCCC	-1,382469456	3,263854619	0,022121495	0,150707971	T1D vs T2D Time-adjusted
hsa-miR-25-3p_CATTGCACTTGTCTCGGTCTGA	-0,392362871	11,97256306	0,022291824	0,150707971	T1D vs T2D Time-adjusted
hsa-miR-451a_AAACCGTTACCATTACTGA	-0,499366256	8,906066815	0,022537424	0,15107714	T1D vs T2D Time-adjusted
hsa-miR-215-5p_ATGACCTATGAATTGACAGA	1,256330713	5,04356396	0,022931609	0,15208523	T1D vs T2D Time-adjusted
hsa-miR-30e-5p_TGTAAACATCCTTGACTGG	-0,948505912	5,259392082	0,023191271	0,15208523	T1D vs T2D Time-adjusted
hsa-miR-27b-3p_TTCACAGTGGCTAAGTTCTG	-0,452194339	8,329497842	0,023343661	0,15208523	T1D vs T2D Time-adjusted
hsa-miR-30d-5p_TGTAAACATCCCCGACTGGAAGC	-0,245894721	11,545304	0,023561063	0,15208523	T1D vs T2D Time-adjusted
hsa-miR-150-5p_GTCTCCCAACCCTTGTACCAGT	-1,314442532	2,385284805	0,023649157	0,15208523	T1D vs T2D Time-adjusted
hsa-miR-126-5p_CATTATTACTTTTGGTACGCG	-0,67209812	6,552150257	0,024336579	0,155243825	T1D vs T2D Time-adjusted
hsa-miR-500a-3p_AATGCACCTGGGCAAGGATTCT	-1,434434153	2,908982628	0,024911475	0,157639812	T1D vs T2D Time-adjusted
hsa-miR-30a-5p_TGTAAACATCCTCGACTGGAAGC	-0,442697475	7,811257993	0,025576892	0,160566046	T1D vs T2D Time-adjusted
hsa-miR-93-3p_ACTGCTGAGCTAGCACTTCCCGA	-1,212175776	3,420021767	0,025967828	0,161736629	T1D vs T2D Time-adjusted
hsa-miR-425-5p_AATGACACGATCACTCCCGTTGA	-0,255284054	10,33255905	0,026318896	0,161799076	T1D vs T2D Time-adjusted
hsa-miR-140-3p_ACCACAGGGTAGAACCACGGAC	-0,769231582	5,842587001	0,02644664	0,161799076	T1D vs T2D Time-adjusted
hsa-miR-139-5p_TCTACAGTGCACGTGTCTCCAG	-0,947533811	4,962736668	0,026591504	0,161799076	T1D vs T2D Time-adjusted
hsa-miR-92b-3p_TATTGCACTCGTCCCGGCC	-1,277661449	3,918389355	0,027049995	0,161881172	T1D vs T2D Time-adjusted
hsa-miR-182-5p_TTTGGCAATGGTAGAACTCAC	-1,334887198	3,088555901	0,02720303	0,161881172	T1D vs T2D Time-adjusted
hsa-miR-425-5p_ATGACACGATCACTCCCGTTG	-1,329916707	2,364893848	0,027218958	0,161881172	T1D vs T2D Time-adjusted
hsa-miR-500a-3p_ATGCACCTGGGCAAGGATTCT	-1,08899424	4,574890345	0,028502629	0,168250596	T1D vs T2D Time-adjusted
hsa-miR-30d-5p_TAAACATCCCCGACTGGAAGCT	-1,242467618	2,129907594	0,029166779	0,170895721	T1D vs T2D Time-adjusted
hsa-miR-10b-5p_TACCCTGTAGAACCGAATTT	-0,926466747	5,284240397	0,029635865	0,172367419	T1D vs T2D Time-adjusted
hsa-miR-23b-3p_ATCACATTGCCAGGGATTACC	-0,835634586	5,450967457	0,030020899	0,173332345	T1D vs T2D Time-adjusted
hsa-miR-451a_GAAACCGTTACCATTACTGAGT	-0,640640934	6,902919522	0,030568321	0,174408791	T1D vs T2D Time-adjusted
hsa-miR-146b-5p_TGAGAACTGAATTCCATAGGCTG	-0,846874298	5,796387655	0,030648321	0,174408791	T1D vs T2D Time-adjusted
hsa-miR-451a_AAACCGTTACCATTACTGAGTT	-0,376631807	12,10291266	0,031323167	0,176220819	T1D vs T2D Time-adjusted
hsa-miR-223-3p_TGTCAGTTTGTCAAATACCCCAA	-0,457205283	10,82178642	0,031412308	0,176220819	T1D vs T2D Time-adjusted
hsa-let-7g-5p_GAGGTAGTAGTTTGTACAGT	-1,289222932	3,503297517	0,031710161	0,176638995	T1D vs T2D Time-adjusted
hsa-miR-181a-5p_AACATTCAACGCTGTCGGTGAGTT	-1,254481047	2,473533262	0,032420247	0,178895626	T1D vs T2D Time-adjusted
hsa-miR-942-5p_TCTTCTCTGTTTTGGCCATGT	-1,311838201	2,575024753	0,032627871	0,178895626	T1D vs T2D Time-adjusted
hsa-miR-183-5p_TATGGCACTGGTAGAATTC	-1,247235017	3,259461606	0,033046951	0,178895626	T1D vs T2D Time-adjusted
hsa-miR-100-5p_AACCCGTAGATCCGAACTTGTG	-1,331150076	3,697610074	0,033300706	0,178895626	T1D vs T2D Time-adjusted

hsa-miR-361-3p_TCCCCCAGGTGTGATTCTGATTT	-1,025902554	4,004503719	0,033434238	0,178895626	T1D vs T2D Time-adjusted
hsa-miR-361-5p_TTATCAGAATCTCCAGGGGTA	-1,227999715	3,316391684	0,033472254	0,178895626	T1D vs T2D Time-adjusted
hsa-miR-151a-3p_TACTAGACTGAAGCTCCTTGAG	-1,236010414	2,948484967	0,034000836	0,179669487	T1D vs T2D Time-adjusted
hsa-miR-92b-3p_TATTGCACTCGTCCCGGCCTCC	-1,081835046	4,339207773	0,034071331	0,179669487	T1D vs T2D Time-adjusted
hsa-miR-181a-5p_ACATTCAACGCTGTCGGTGA	-1,158254151	3,835029228	0,034691391	0,181727752	T1D vs T2D Time-adjusted
hsa-miR-363-3p_AATTGCACGGTATCCATCTGT	-0,363709947	9,045234632	0,035022326	0,182254343	T1D vs T2D Time-adjusted
hsa-miR-151a-3p_CTAGACTGAAGCTCCTTGAGGA	-0,41558723	8,369867531	0,03529332	0,182464158	T1D vs T2D Time-adjusted
hsa-miR-361-5p_TTATCAGAATCTCCAGGGGT	-0,830638048	4,851644504	0,03598345	0,184421947	T1D vs T2D Time-adjusted
hsa-miR-145-5p_GTCCAGTTTTCCCAGGAATCC	-1,265224573	2,346106601	0,036138308	0,184421947	T1D vs T2D Time-adjusted
hsa-miR-320a-3p_AAAAGCTGGGTTGAGAGGGCGAA	0,272439503	9,820743233	0,036834073	0,18676764	T1D vs T2D Time-adjusted
hsa-miR-183-5p_TATGGCACTGGTAGAATTCACT	-0,822206643	5,890825812	0,037713608	0,189788818	T1D vs T2D Time-adjusted
hsa-miR-143-3p_TGAGATGAAGCACTGTAGCTCA	-0,715908783	6,633635761	0,038081023	0,189788818	T1D vs T2D Time-adjusted
hsa-let-7g-5p_TGAGGTAGTAGTTTGTACA	-0,85022367	5,551691527	0,038602268	0,189788818	T1D vs T2D Time-adjusted
hsa-miR-30a-5p_TGTAAACATCCTCGACTGGAAGCT	-0,280621706	8,865025747	0,03876024	0,189788818	T1D vs T2D Time-adjusted
hsa-miR-93-5p_AAAGTGCTGTTCGTGCAGGTAG	-1,111116667	3,678777222	0,038975056	0,189788818	T1D vs T2D Time-adjusted
hsa-miR-10b-5p_TACCCTGTAGAACCGAATTTGTGT	-1,226088384	2,423805448	0,039056536	0,189788818	T1D vs T2D Time-adjusted
hsa-miR-181a-5p_AACATTCAACGCTGTCGG	-1,111965313	2,244051199	0,039885301	0,189788818	T1D vs T2D Time-adjusted
hsa-miR-125a-5p_TCCCTGAGACCCTTTAACCTGTG	-0,352849149	8,651356777	0,039920445	0,189788818	T1D vs T2D Time-adjusted
hsa-miR-30c-5p_GTAAACATCCTACACTCTCAGCT	-1,32715662	3,000834251	0,039931528	0,189788818	T1D vs T2D Time-adjusted
hsa-miR-30c-5p_TGTAAACATCCTACACTCTCA	-0,511307574	7,960145287	0,040069241	0,189788818	T1D vs T2D Time-adjusted
hsa-miR-122-5p_GGAGTGTGACAATGGTGTTTG	-0,981072271	5,619888581	0,040447948	0,189788818	T1D vs T2D Time-adjusted
hsa-miR-24-3p_GCTCAGTTCAGCAGGAACAG	-1,195884599	2,351084248	0,040543648	0,189788818	T1D vs T2D Time-adjusted
hsa-miR-532-5p_CATGCCTTGAGTGTAGGACCGT	-0,99841863	4,686169319	0,040549065	0,189788818	T1D vs T2D Time-adjusted
hsa-miR-194-5p_TGTAACAGCAACTCCATGTGGAA	-1,174607038	2,71610017	0,04094009	0,19049183	T1D vs T2D Time-adjusted
hsa-miR-191-5p_CAACGGAATCCCAAAAGCA	-0,674214129	6,200760212	0,041440798	0,191693985	T1D vs T2D Time-adjusted
hsa-miR-501-3p_ATGCACCCGGGCAAGGATTCT	-1,197772204	2,108861011	0,042675641	0,196258324	T1D vs T2D Time-adjusted
hsa-let-7f-5p_GAGGTAGTAGATTGTATAG	-1,166606147	3,124116914	0,043402648	0,198241273	T1D vs T2D Time-adjusted
hsa-miR-30e-3p_CTTTCAGTCGGATGTTTACAGC	-1,256010975	3,112145052	0,04373829	0,198241273	T1D vs T2D Time-adjusted
hsa-miR-484_TCAGGCTCAGTCCCCTCCCGAT	-0,304418006	9,170955833	0,043922086	0,198241273	T1D vs T2D Time-adjusted
hsa-miR-194-5p_TGTAACAGCAACTCCATGTGG	-0,586475782	6,643496377	0,04410931	0,198241273	T1D vs T2D Time-adjusted
hsa-miR-3615_TCTCTCGGCTCCTCGCGGCTCG	0,317578393	8,148580046	0,044922848	0,199383705	T1D vs T2D Time-adjusted
hsa-let-7g-5p_TGAGGTAGTAGTTTGTACAGT	-0,206962741	10,71493101	0,044970209	0,199383705	T1D vs T2D Time-adjusted
hsa-miR-320b_AAAAGCTGGGTTGAGAGGGCA	-1,083461164	2,615161599	0,04536471	0,199383705	T1D vs T2D Time-adjusted
hsa-miR-99b-5p_CACCCGTAGAACCGACCTTG	-0,523923675	6,823079979	0,045371766	0,199383705	T1D vs T2D Time-adjusted
hsa-let-7f-5p_TGAGGTAGTAGATTGTAT	-1,234038352	3,218494088	0,046765761	0,201059844	T1D vs T2D Time-adjusted
hsa-miR-197-3p_TTCACCACCTTCTCCACCCAGC	0,339143401	10,0726909	0,046789095	0,201059844	T1D vs T2D Time-adjusted
hsa-miR-146a-5p_TGAGAACTGAATTCCATGGGT	-0,296223368	8,701643613	0,046927714	0,201059844	T1D vs T2D Time-adjusted
hsa-miR-486-5p_GTACTGAGCTGCCCCGAG	-0,883649962	5,002102428	0,0471296	0,201059844	T1D vs T2D Time-adjusted
hsa-miR-584-5p_TTATGGTTTGCCTGGGACTGA	-0,428496524	8,05116289	0,047228226	0,201059844	T1D vs T2D Time-adjusted
hsa-miR-122-5p_GAGTGTGACAATGGTGTTT	-1,265801542	3,048399419	0,047278295	0,201059844	T1D vs T2D Time-adjusted
hsa-miR-29c-3p_TAGCACCATTTGAAATCGGTT	-0,966371632	3,979110528	0,048596826	0,203943387	T1D vs T2D Time-adjusted
hsa-miR-363-3p_AATTGCACGGTATCCATCTGTA	-0,932297079	4,05384132	0,048851286	0,203943387	T1D vs T2D Time-adjusted
hsa-miR-92a-3p_GTATTGCACTTGTCCCGGCCTG	-1,18396218	3,050162886	0,04894311	0,203943387	T1D vs T2D Time-adjusted
hsa-let-7e-5p_TGAGGTAGGAGGTTGTATAGTT	-0,445232859	7,821946446	0,048987666	0,203943387	T1D vs T2D Time-adjusted
hsa-miR-181b-5p_AACATTCATTGCTGTCGGTG	-1,179695291	2,055578696	0,049408486	0,20461839	T1D vs T2D Time-adjusted
hsa-miR-183-5p_ATGGCACTGGTAGAATTCACTGT	-1,198267067	2,504851483	0,050604425	0,208479687	T1D vs T2D Time-adjusted
hsa-miR-4433b-5p_TGTCCCACCCCCACTCCTG	1,288487215	2,575504162	0,05145273	0,210876213	T1D vs T2D Time-adjusted
hsa-miR-532-5p_CATGCCTTGAGTGTAGGACCG	-1,098388691	2,328095035	0,052470579	0,21337756	T1D vs T2D Time-adjusted
hsa-miR-30b-5p_TGTAAACATCCTACACTCAGCT	-0,555756587	6,702165067	0,052602559	0,21337756	T1D vs T2D Time-adjusted
hsa-miR-92a-3p_ATTGCACTTGTCCCGGCCTGTT	-0,756842641	5,582760418	0,055610193	0,22442685	T1D vs T2D Time-adjusted
hsa-miR-30d-5p_TGTAAACATCCCCGACTGGAA	-0,229399633	11,65154018	0,055924642	0,224550214	T1D vs T2D Time-adjusted
hsa-miR-423-5p_AGGGGCAGAGAGCGAGACTTTT	-0,918155028	5,009061922	0,056418957	0,225390883	T1D vs T2D Time-adjusted
hsa-miR-451a_ACCGTTACCATTACTGAG	-1,014471949	3,859377434	0,057249405	0,227559194	T1D vs T2D Time-adjusted
hsa-miR-140-5p_CAGTGGTTTTACCCTATGGTAG	-0,918789903	3,907116242	0,057580934	0,227732595	T1D vs T2D Time-adjusted
hsa-miR-142-5p_CCCATAAAGTAGAAAGCAC	-0,737090929	5,073422111	0,05807501	0,228543946	T1D vs T2D Time-adjusted
hsa-miR-423-5p_TGAGGGGCAGAGAGCGAGACTTT	0,144923651	14,46310907	0,059371205	0,232205377	T1D vs T2D Time-adjusted
hsa-miR-197-3p_TTCACCACCTTCTCCACCCAG	0,389986176	8,701233583	0,05959253	0,232205377	T1D vs T2D Time-adjusted
hsa-miR-122-5p_TGGAGTGTGACAATGGTGTTTG	-0,545311561	11,7376523	0,060513172	0,234636858	T1D vs T2D Time-adjusted
hsa-miR-101-3p_TACAGTACTGTGATAACTGAA	-1,175370718	2,97176202	0,062082228	0,238281353	T1D vs T2D Time-adjusted
hsa-miR-25-3p_CATTGCACTTGTCTCGGTCTG	-0,398443034	9,96694873	0,062088304	0,238281353	T1D vs T2D Time-adjusted
hsa-let-7i-5p_TGAGGTAGTAGTTTGTGCT	-0,395011152	7,631066138	0,062356814	0,238281353	T1D vs T2D Time-adjusted

hsa-miR-532-3p_CCTCCCACACCCAAGGCTTG	-1,139843911	2,771296807	0,064759232	0,246271887	T1D vs T2D Time-adjusted
hsa-miR-142-5p_CATAAAGTAGAAAGCACTAC	-1,091329467	2,318773636	0,065093386	0,246358222	T1D vs T2D Time-adjusted
hsa-miR-143-3p_TGAGATGAAGCACTGTAGCTC	-0,31263971	9,81453422	0,065880058	0,2471294	T1D vs T2D Time-adjusted
hsa-miR-142-5p_CCATAAAGTAGAAAGCACT	-0,750264462	4,725847494	0,065922002	0,2471294	T1D vs T2D Time-adjusted
hsa-miR-186-5p_CAAAGAATTCTCCTTTTGGGCTTT	-1,03853226	3,921463053	0,066750488	0,249054886	T1D vs T2D Time-adjusted
hsa-miR-19a-3p_TGTGCAAATCTATGCAAAACTGA	-1,070360215	2,482431203	0,067980559	0,252453625	T1D vs T2D Time-adjusted
hsa-miR-3613-5p_TGTTGTACTTTTTTTTTTGTTC	-0,865797398	4,360142246	0,069384398	0,256147989	T1D vs T2D Time-adjusted
hsa-miR-451a_AAACCGTTACCATTACTGAGTTTAG	-1,089593644	2,222514405	0,069623031	0,256147989	T1D vs T2D Time-adjusted
hsa-miR-320a-3p_AAAAGCTGGGTTGAGAGGGCGAAAA	-1,072981857	3,105258929	0,074527974	0,272924201	T1D vs T2D Time-adjusted
hsa-miR-7-5p_TGGAAGACTAGTGATTTTGT	-0,974365311	4,179429072	0,075329562	0,274588404	T1D vs T2D Time-adjusted
hsa-miR-193a-5p_TGGGTCTTTGCGGGCGAGAT	-0,864862883	4,70145732	0,076508793	0,276546609	T1D vs T2D Time-adjusted
hsa-miR-361-3p_TCCCCCAGGTGTGATTCTGATTTG	-0,600833588	5,300626315	0,076947917	0,276546609	T1D vs T2D Time-adjusted
hsa-miR-146b-5p_TGAGAACTGAATTCCATAGGCT	-0,526371706	6,379112274	0,077018607	0,276546609	T1D vs T2D Time-adjusted
hsa-miR-146b-5p_TGAGAACTGAATTCCATAGGCTGT	-0,32376161	8,367181967	0,077265235	0,276546609	T1D vs T2D Time-adjusted
hsa-let-7f-5p_TGAGGTAGTAGATTG	-0,939130659	4,368619365	0,079496485	0,283250989	T1D vs T2D Time-adjusted
hsa-miR-574-3p_CACGCTCATGCACACACCCAC	-0,925287247	3,990306713	0,081837205	0,288911458	T1D vs T2D Time-adjusted
hsa-miR-423-5p_GAGGGGCAGAGAGCGAGACTTTT	-0,955319734	3,8847694	0,082093533	0,288911458	T1D vs T2D Time-adjusted
hsa-miR-16-2-3p_CCAATATTACTGTGCTGCTT	-0,729053557	4,45076213	0,082180882	0,288911458	T1D vs T2D Time-adjusted
hsa-miR-100-5p_AACCCGTAGATCCGAACTTG	-0,573242949	5,980148473	0,086665625	0,303329689	T1D vs T2D Time-adjusted
hsa-miR-122-5p_GGAGTGTGACAATGGTGTTT	-0,633422603	7,754720279	0,087584139	0,305188569	T1D vs T2D Time-adjusted
hsa-miR-223-3p_TGTCAGTTTGTCAAATACCC	-0,772426216	4,539198612	0,087968387	0,305188569	T1D vs T2D Time-adjusted
hsa-miR-7-5p_TGGAAGACTAGTGATTTTGTTGT	-0,689049165	6,235025385	0,088918926	0,306684528	T1D vs T2D Time-adjusted
hsa-miR-140-3p_TACCACAGGGTAGAACCACG	-0,714382272	4,858224511	0,089722266	0,306684528	T1D vs T2D Time-adjusted
hsa-miR-15b-5p_TAGCAGCACATCATGGTTTACA	-0,543713323	6,304858351	0,089876996	0,306684528	T1D vs T2D Time-adjusted
hsa-miR-484_TCAGGCTCAGTCCCCTCCCG	-0,483938341	7,776089511	0,089950456	0,306684528	T1D vs T2D Time-adjusted
hsa-miR-339-5p_TCCCTGTCCTCCAGGAGCTCACG	-1,056532466	3,320095685	0,090992854	0,30890707	T1D vs T2D Time-adjusted
hsa-let-7a-5p_GAGGTAGTAGGTTGTATAGT	-0,851850966	4,561721311	0,091592229	0,309613049	T1D vs T2D Time-adjusted
hsa-miR-374b-5p_ATATAATACAACCTGCTAAGT	-0,85467428	3,94114477	0,094191365	0,317044127	T1D vs T2D Time-adjusted
hsa-miR-223-3p_TCAGTTTGTCAAATACCCCAA	-0,93376035	2,097761681	0,09469364	0,317384192	T1D vs T2D Time-adjusted
hsa-miR-16-2-3p_CCAATATTACTGTGCTGCTTT	-0,798702863	4,408566959	0,096388181	0,317634714	T1D vs T2D Time-adjusted
hsa-miR-101-3p_GTACAGTACTGTGATAACTGAA	-0,982787285	3,734790611	0,096406425	0,317634714	T1D vs T2D Time-adjusted
hsa-miR-3173-5p_CCCTGCCTGTTTTCTCCTTTGT	-0,921972701	1,917401292	0,096599199	0,317634714	T1D vs T2D Time-adjusted
hsa-miR-374b-5p_ATATAATACAACCTGCTAAGTG	-0,9873993	2,591933676	0,096604801	0,317634714	T1D vs T2D Time-adjusted
hsa-miR-148a-3p_TCAGTGCACTACAGAACTTTGT	-0,259028194	9,218931731	0,097066714	0,317634714	T1D vs T2D Time-adjusted
hsa-miR-629-5p_TGGGTTTACGTTGGGAGAA	-1,063702824	2,664864204	0,097177751	0,317634714	T1D vs T2D Time-adjusted
hsa-let-7a-5p_TTGAGGTAGTAGGTTGTATAGTT	-0,986716892	1,879153168	0,098817764	0,321666055	T1D vs T2D Time-adjusted
hsa-miR-21-5p_TAGCTTATCAGACTGATG	-0,974611684	2,041272314	0,100012205	0,324219893	T1D vs T2D Time-adjusted
hsa-miR-30a-5p_TGTAAACATCCTCGACTGGAAG	-0,323135116	8,371847242	0,10320871	0,333216693	T1D vs T2D Time-adjusted
hsa-miR-423-3p_AGCTCGGTCTGAGGCCCCTCA	-0,578168648	6,070547639	0,104263529	0,334053135	T1D vs T2D Time-adjusted
hsa-miR-128-3p_TCACAGTGAACCGGTCTCTT	-0,421319707	6,752700404	0,10431242	0,334053135	T1D vs T2D Time-adjusted
hsa-miR-30a-5p_GTAAACATCCTCGACTGGAAGCT	-0,874271693	4,346285408	0,105855853	0,336945499	T1D vs T2D Time-adjusted
hsa-miR-182-5p_TTTGGCAATGGTAGAACT	-1,004114372	3,433367687	0,106067547	0,336945499	T1D vs T2D Time-adjusted
hsa-miR-99b-5p_CACCCGTAGAACCGACCTT	-0,987409252	2,15225541	0,107102803	0,337347355	T1D vs T2D Time-adjusted
hsa-miR-664a-5p_ACTGGCTAGGGAAAATGATTGG	-0,921829843	3,605476953	0,107506528	0,337347355	T1D vs T2D Time-adjusted
hsa-miR-221-3p_AGCTACATTGTCTGCTGGGTTTCA	-0,991763199	2,292055081	0,107811193	0,337347355	T1D vs T2D Time-adjusted
hsa-miR-505-3p_CGTCAACACTTGCTGGTTTCCTCT	-1,00106517	3,132189778	0,107899976	0,337347355	T1D vs T2D Time-adjusted
hsa-miR-22-3p_AGCTGCCAGTTGAAGAACTGT	-0,621215518	5,657855582	0,109490213	0,340971489	T1D vs T2D Time-adjusted
hsa-miR-21-5p_AGCTTATCAGACTGATGTTGAC	-1,024605	3,019001358	0,110473979	0,341867168	T1D vs T2D Time-adjusted
hsa-let-7c-5p_TGAGGTAGTAGGTTGTATGGTT	-0,313384329	7,624637986	0,111083788	0,341867168	T1D vs T2D Time-adjusted
hsa-miR-223-3p_TGTCAGTTTGTCAAATACCCCA	-0,30589124	10,34583114	0,111373897	0,341867168	T1D vs T2D Time-adjusted
hsa-miR-100-5p_AACCCGTAGATCCGAACTTGT	-0,440047932	7,209550932	0,111506611	0,341867168	T1D vs T2D Time-adjusted
hsa-miR-451a_ACCGTTACCATTACTGAGTT	-0,957348975	2,058885942	0,112504731	0,343595531	T1D vs T2D Time-adjusted
hsa-miR-152-3p_TCAGTGCATGACAGAACTTGG	-0,866241519	2,028197906	0,114831238	0,349351959	T1D vs T2D Time-adjusted
hsa-miR-342-3p_TCTCACACAGAAATCGCACCCGTC	-0,34082033	7,588021781	0,118196568	0,358212588	T1D vs T2D Time-adjusted
hsa-miR-23b-3p_ATCACATTGCCAGGGATTACCA	-0,46504306	6,519069588	0,118819306	0,358725462	T1D vs T2D Time-adjusted
hsa-miR-26b-5p_TTCAAGTAATTCAGGATAGG	-0,845677467	3,267725483	0,121034655	0,362685383	T1D vs T2D Time-adjusted
hsa-miR-625-3p_GACTATAGAACTTTCCCCCTCA	-1,284496664	3,448522418	0,12110569	0,362685383	T1D vs T2D Time-adjusted
hsa-miR-146a-5p_TGAGAACTGAATTCCATGGGTT	-0,183651826	12,18952455	0,121506481	0,362685383	T1D vs T2D Time-adjusted
hsa-miR-191-5p_AACGGAATCCCAAAAGCAGC	-0,905316398	2,971417206	0,122384374	0,363932481	T1D vs T2D Time-adjusted
hsa-miR-191-5p_CAACGGAATCCCAAAAGCAGC	-0,336470488	8,118131191	0,122934368	0,36419882	T1D vs T2D Time-adjusted
hsa-miR-23a-3p_TCACATTGCCAGGGATTTCCA	-0,67131219	5,160556193	0,123818166	0,365448392	T1D vs T2D Time-adjusted

hsa-miR-342-3p_TCTCACACAGAAATCGCACCCGT	-0,348649318	8,723994136	0,125720942	0,369684999	T1D vs T2D Time-adjusted
hsa-miR-222-3p_AGCTACATCTGGCTACTGGGTC	-0,836006504	3,53327113	0,128131058	0,374228228	T1D vs T2D Time-adjusted
hsa-let-7b-3p_CTATACAACCTACTGCCTTCC	-0,49670486	5,967940681	0,128212199	0,374228228	T1D vs T2D Time-adjusted
hsa-miR-425-5p_AATGACACGATCACTCCCG	-0,764726702	4,588646685	0,130401062	0,378729324	T1D vs T2D Time-adjusted
hsa-miR-425-5p_ATGACACGATCACTCCCGTTGA	-0,687783445	4,777521295	0,130999362	0,378729324	T1D vs T2D Time-adjusted
hsa-let-7i-5p_GAGGTAGTAGTTTGTGCTGT	-0,734725987	4,86484784	0,131862032	0,378729324	T1D vs T2D Time-adjusted
hsa-miR-16-5p_GCAGCACGTAAATATTGGCG	-0,764622408	3,590895157	0,131863796	0,378729324	T1D vs T2D Time-adjusted
hsa-miR-451a_AAACCGTTACCATTACTGAGTTTAGT	-0,669193161	4,347393724	0,132905828	0,378729324	T1D vs T2D Time-adjusted
hsa-miR-338-5p_AACAATATCCTGGTGCTGAGT	-0,567880307	5,921373435	0,132910753	0,378729324	T1D vs T2D Time-adjusted
hsa-miR-486-5p_CTGTACTGAGCTGCCCCG	-0,920135233	3,36154621	0,133933019	0,378729324	T1D vs T2D Time-adjusted
hsa-miR-28-3p_ACTAGATTGTGAGCTCCTGGAG	-0,761362938	4,665468153	0,134057276	0,378729324	T1D vs T2D Time-adjusted
hsa-miR-106b-5p_TAAAGTGCTGACAGTGCAGAT	-0,84397128	2,555392208	0,13486727	0,378729324	T1D vs T2D Time-adjusted
hsa-miR-493-5p_TTGTACATGGTAGGCTTTCATT	0,937527855	3,42834388	0,134931105	0,378729324	T1D vs T2D Time-adjusted
hsa-miR-181a-5p_AACATTCAACGCTGTCGGTGAG	-0,295436902	7,417004171	0,135021074	0,378729324	T1D vs T2D Time-adjusted
hsa-miR-222-3p_AGCTACATCTGGCTACTGGGTCTC	-0,717575821	5,925164986	0,135731924	0,379377921	T1D vs T2D Time-adjusted
hsa-miR-423-5p_CTGAGGGGCAGAGAGCGAGACTTT	-0,804241528	4,393029535	0,138156452	0,384794907	T1D vs T2D Time-adjusted
hsa-miR-654-5p_TGGTGGGCCGCAGAACATGTGC	1,013946648	3,180891958	0,139703983	0,386596819	T1D vs T2D Time-adjusted
hsa-miR-27b-3p_TTCACAGTGGCTAAGTTCT	-0,295297572	8,397674944	0,140155097	0,386596819	T1D vs T2D Time-adjusted
hsa-miR-146a-5p_TGAGAACTGAATTCCATGGGTTG	-0,334868384	7,862859516	0,140458209	0,386596819	T1D vs T2D Time-adjusted
hsa-miR-24-3p_TGGCTCAGTTCAGCAGGAA	-0,643431478	5,597444609	0,140758387	0,386596819	T1D vs T2D Time-adjusted
hsa-miR-4433b-5p_TGTCCCACCCCCACTCCTGTT	0,754417159	6,506356169	0,14260306	0,390308029	T1D vs T2D Time-adjusted
hsa-miR-186-5p_CAAAGAATTCTCCTTTTGGGCTT	-0,536632976	6,241384756	0,143565248	0,391586589	T1D vs T2D Time-adjusted
hsa-miR-494-3p_TGAAACATACACGGGAAACCTCT	-0,844134826	4,130022225	0,144969269	0,392948327	T1D vs T2D Time-adjusted
hsa-miR-652-3p_AATGGCGCCACTAGGGTTGTG	-0,449071321	6,436258449	0,145058042	0,392948327	T1D vs T2D Time-adjusted
hsa-miR-181a-5p_AACATTCAACGCTGTCGGTGA	-0,181294169	10,01113916	0,146068624	0,393175619	T1D vs T2D Time-adjusted
hsa-miR-26a-5p_TCAAGTAATCCAGGATAGGCT	-0,486949543	6,500759116	0,146512423	0,393175619	T1D vs T2D Time-adjusted
hsa-miR-155-5p_TTAATGCTAATCGTGATAGGGGTT	-0,865714098	3,129748949	0,146633132	0,393175619	T1D vs T2D Time-adjusted
hsa-let-7c-5p_TGAGGTAGTAGGTTGTATGG	-0,564323363	3,127922361	0,150127014	0,401184013	T1D vs T2D Time-adjusted
hsa-miR-486-5p_TCCTGTACTGAGCTGCCCCGAGG	-0,303029691	7,407933707	0,150781936	0,401577478	T1D vs T2D Time-adjusted
hsa-miR-25-3p_CATTGCACTTGTCTCGGTC	-0,54073523	5,824634034	0,151626572	0,402471874	T1D vs T2D Time-adjusted
hsa-miR-223-3p_GTCAGTTTGTCAAATACCCCA	-0,484235494	6,353871952	0,154303008	0,408206286	T1D vs T2D Time-adjusted
hsa-miR-192-5p_TGACCTATGAATTGACAGCCA	-0,704777513	4,775035894	0,155803772	0,410199689	T1D vs T2D Time-adjusted
hsa-miR-375-3p_TTTGTTCGTTCGGCTCGCGT	-0,860111146	4,026890223	0,156130462	0,410199689	T1D vs T2D Time-adjusted
hsa-miR-22-3p_AAGCTGCCAGTTGAAGAACTGT	-0,132593943	12,1121356	0,156612271	0,410199689	T1D vs T2D Time-adjusted
hsa-miR-18a-3p_ACTGCCCTAAGTGCTCCTTCTG	-0,814174209	2,147867886	0,157294021	0,410625647	T1D vs T2D Time-adjusted
hsa-miR-30d-5p_TGTAAACATCCCCGACTGGAAGCT	-0,190667787	13,12660906	0,158455361	0,412296678	T1D vs T2D Time-adjusted
hsa-miR-148a-3p_TCAGTGCACTACAGAACTTTG	-0,31941778	7,255293658	0,159389592	0,412321963	T1D vs T2D Time-adjusted
hsa-miR-423-5p_TGAGGGGCAGAGAGCGAGACTT	0,151942666	12,27072707	0,160353657	0,412321963	T1D vs T2D Time-adjusted
hsa-miR-99a-5p_AACCCGTAGATCCGATCTTGT	-0,26499006	8,596582119	0,160431872	0,412321963	T1D vs T2D Time-adjusted
hsa-miR-339-3p_TGAGCGCCTCGACGACAGAGCCG	-0,832483232	4,074916381	0,161060641	0,412321963	T1D vs T2D Time-adjusted
hsa-miR-10a-5p_TACCCTGTAGATCCGAATTT	-0,749772154	4,289428906	0,161071412	0,412321963	T1D vs T2D Time-adjusted
hsa-miR-6803-3p_TCCCTCGCCTTCTCACCCTCA	0,552636783	6,152212628	0,1640886	0,418690589	T1D vs T2D Time-adjusted
hsa-miR-339-3p_TGAGCGCCTCGACGACAGAG	-0,607697249	5,283296909	0,165174552	0,419116037	T1D vs T2D Time-adjusted
hsa-miR-451a_AAACCGTTACCATTACTGAGTTT	-0,264068402	9,000647073	0,165603271	0,419116037	T1D vs T2D Time-adjusted
hsa-miR-4732-5p_TGTAGAGCAGGGAGCAGGAAGCT	-0,474645359	6,317387172	0,165844905	0,419116037	T1D vs T2D Time-adjusted
hsa-let-7f-5p_GAGGTAGTAGATTGTATAGT	-0,871848101	3,007465312	0,166552104	0,419562784	T1D vs T2D Time-adjusted
hsa-miR-191-5p_CAACGGAATCCCAAAAGCAGCTGT	-0,622653619	5,75430435	0,167481968	0,4205493	T1D vs T2D Time-adjusted
hsa-miR-132-3p_TAACAGTCTACAGCCATGGTCG	-0,827531614	2,429322611	0,168900265	0,4205493	T1D vs T2D Time-adjusted
hsa-miR-423-3p_AGCTCGGTCTGAGGCCCCT	-0,447357325	6,073013782	0,169021583	0,4205493	T1D vs T2D Time-adjusted
hsa-miR-2110_TTGGGGAAACGGCCGCTGAG	-0,816762746	2,146127874	0,169316262	0,4205493	T1D vs T2D Time-adjusted
hsa-miR-30e-5p_GTAAACATCCTTGACTGGAAGC	-0,854845163	2,516119605	0,169602056	0,4205493	T1D vs T2D Time-adjusted
hsa-miR-125a-5p_CCCTGAGACCCTTTAACCTGT	-0,616810835	5,393774828	0,172928367	0,427045726	T1D vs T2D Time-adjusted
hsa-miR-3613-5p_TGTTGTACTTTTTTTTTTGTT	-0,5834001	5,198438116	0,173624187	0,427045726	T1D vs T2D Time-adjusted
hsa-miR-150-3p_CTGGTACAGGCCTGGGGGAC	-0,825861316	2,452241426	0,173841623	0,427045726	T1D vs T2D Time-adjusted
hsa-miR-484_TCAGGCTCAGTCCCCTCCCGATA	-0,669454242	4,542352365	0,175816036	0,430053015	T1D vs T2D Time-adjusted
hsa-miR-24-3p_TGGCTCAGTTCAGCAGGAACA	-0,163714605	10,02516895	0,176504156	0,430053015	T1D vs T2D Time-adjusted
hsa-miR-142-5p_CCCATAAAGTAGAAAGCACT	-0,170996856	11,11931665	0,176696877	0,430053015	T1D vs T2D Time-adjusted
hsa-miR-181a-5p_AACATTCAACGCTGTCGGTG	-0,378301041	6,965832799	0,177710668	0,430571624	T1D vs T2D Time-adjusted
hsa-miR-425-5p_AATGACACGATCACTCCCGTTGAGT	-0,347387465	8,285934125	0,17801223	0,430571624	T1D vs T2D Time-adjusted
hsa-miR-320a-3p_GAAAAGCTGGGTTGAGAGGGCGA	-0,744867264	3,726965694	0,178542974	0,430571624	T1D vs T2D Time-adjusted
hsa-miR-4433b-5p_TATGTCCCACCCCCACTCCTG	0,935333543	2,841400639	0,179894988	0,431940258	T1D vs T2D Time-adjusted

hsa-miR-487b-3p_AATCGTACAGGGTCATCCACTT	-0,793283493	3,93004941	0,181115119	0,431940258	T1D vs T2D Time-adjusted
hsa-miR-335-5p_TCAAGAGCAATAACGAAAAAT	0,496001079	6,614922062	0,181745899	0,431940258	T1D vs T2D Time-adjusted
hsa-miR-21-3p_CAACACCAGTCGATGGGCTGT	-0,751180083	2,288436309	0,181766454	0,431940258	T1D vs T2D Time-adjusted
hsa-miR-10b-5p_TACCCTGTAGAACCGAATTTGTG	-0,295289757	7,887217076	0,182240113	0,431940258	T1D vs T2D Time-adjusted
hsa-miR-1249-3p_ACGCCCTTCCCCCCCTTCTTCA	-0,881457514	2,093528347	0,18238691	0,431940258	T1D vs T2D Time-adjusted
hsa-miR-146a-5p_TGAGAACTGAATTCCATGGG	-0,756094316	2,326140979	0,183915559	0,43426032	T1D vs T2D Time-adjusted
hsa-let-7d-3p_TATACGACCTGCTGCCTTTC	-0,400335852	6,350176431	0,185003416	0,435498935	T1D vs T2D Time-adjusted
hsa-let-7b-3p_CTATACAACCTACTGCCTTC	-0,789276686	3,17192614	0,185541266	0,435498935	T1D vs T2D Time-adjusted
hsa-miR-126-3p_TCGTACCGTGAGTAATAATGCG	-0,183756999	11,26241255	0,186729559	0,436991365	T1D vs T2D Time-adjusted
hsa-miR-30d-5p_GTAAACATCCCCGACTGGAAGC	-0,695288637	4,700243003	0,187850736	0,438318385	T1D vs T2D Time-adjusted
hsa-miR-589-5p_TGAGAACCACGTCTGCTCTGA	-0,793149675	3,05621435	0,188724525	0,439062058	T1D vs T2D Time-adjusted
hsa-miR-183-5p_ATGGCACTGGTAGAATTCACTG	-0,77873251	2,958551449	0,190624298	0,44196509	T1D vs T2D Time-adjusted
hsa-let-7b-5p_TGAGGTAGTAGGTTGTGTGG	0,162133545	12,46184935	0,191089837	0,44196509	T1D vs T2D Time-adjusted
hsa-miR-10b-5p_ACCCTGTAGAACCGAATTTGTG	-0,402288249	6,035000543	0,191937216	0,442630722	T1D vs T2D Time-adjusted
hsa-miR-10a-5p_TACCCTGTAGATCCGAATTTGT	-0,164015834	10,48518	0,193381696	0,44466547	T1D vs T2D Time-adjusted
hsa-miR-27a-3p_TCACAGTGGCTAAGTTCCG	-0,759148565	2,793419478	0,195850827	0,449037694	T1D vs T2D Time-adjusted
hsa-miR-423-5p_GAGGGGCAGAGAGCGAGACTT	-0,515000577	5,546353182	0,196822071	0,449960285	T1D vs T2D Time-adjusted
hsa-let-7a-5p_TGAGGTAGTAGGTTGTATAG	-0,16860591	12,03070271	0,197728897	0,450730713	T1D vs T2D Time-adjusted
hsa-miR-1307-3p_CTCGGCGTGGCGTCGGTCGTGG	-0,814125329	2,234349501	0,200028983	0,45344017	T1D vs T2D Time-adjusted
hsa-miR-103a-3p_AGCAGCATTGTACAGGGCTATG	-0,751206075	4,200669238	0,200063994	0,45344017	T1D vs T2D Time-adjusted
hsa-miR-574-3p_CACGCTCATGCACACACCCACA	-0,405296143	6,242074994	0,202462011	0,456936538	T1D vs T2D Time-adjusted
hsa-miR-342-3p_TCACACAGAAATCGCACCCGTC	-0,780876086	2,415699371	0,202761978	0,456936538	T1D vs T2D Time-adjusted
hsa-miR-92a-3p_TTGCACTTGTCCCGGCCTG	-0,649252198	4,224665197	0,206106725	0,462310065	T1D vs T2D Time-adjusted
hsa-miR-451a_AAACCGTTACCATTACTGAG	-0,233058326	13,72351402	0,206315364	0,462310065	T1D vs T2D Time-adjusted
hsa-miR-495-3p_AAACAAACATGGTGCACTTCTT	-0,820088419	2,371725028	0,207671412	0,464034145	T1D vs T2D Time-adjusted
hsa-miR-221-3p_AGCTACATTGTCTGCTGGGTT	-0,457262812	6,712650504	0,210808626	0,468775208	T1D vs T2D Time-adjusted
hsa-let-7d-3p_CTATACGACCTGCTGCCTT	-0,584053342	4,663499808	0,210978475	0,468775208	T1D vs T2D Time-adjusted
hsa-miR-487b-3p_TCGTACAGGGTCATCCACTTT	-0,80272072	2,989944331	0,214939136	0,476237694	T1D vs T2D Time-adjusted
hsa-let-7b-5p_TGAGGTAGTAGGTTGTGTGGT	-0,142684764	11,86683629	0,217909104	0,477608292	T1D vs T2D Time-adjusted
hsa-miR-451a_CCGTTACCATTACTGAGT	-0,608615749	4,375477916	0,218136734	0,477608292	T1D vs T2D Time-adjusted
hsa-miR-484_TCAGGCTCAGTCCCCTCCCGA	-0,234659796	10,82608497	0,21873865	0,477608292	T1D vs T2D Time-adjusted
hsa-miR-30c-5p_TGTAAACATCCTACACTCTCAGCT	-0,192020917	10,19324434	0,219688169	0,477608292	T1D vs T2D Time-adjusted
hsa-miR-363-3p_ATTGCACGGTATCCATCTG	-0,751587626	2,190954814	0,219903056	0,477608292	T1D vs T2D Time-adjusted
hsa-miR-30e-5p_TGTAAACATCCTTGACTGGAA	-0,306919966	6,485643066	0,220021133	0,477608292	T1D vs T2D Time-adjusted
hsa-miR-29a-3p_TAGCACCATCTGAAATCGGTTA	-0,581155035	4,650458238	0,220299109	0,477608292	T1D vs T2D Time-adjusted
hsa-miR-30e-5p_TGTAAACATCCTTGACTGGA	-0,731138107	2,526545713	0,22038815	0,477608292	T1D vs T2D Time-adjusted
hsa-miR-320b_AAAAGCTGGGTTGAGAGGGCAA	-0,686047169	2,061631881	0,221877704	0,479522578	T1D vs T2D Time-adjusted
hsa-miR-99b-5p_CACCCGTAGAACCGACCTTGCG	-0,207138144	9,362081621	0,225284192	0,485558027	T1D vs T2D Time-adjusted
hsa-miR-122-5p_TGGAGTGTGACAATGGTGTTT	-0,336619579	13,69364351	0,227779268	0,488401655	T1D vs T2D Time-adjusted
hsa-miR-29a-3p_TAGCACCATCTGAAATCGGTT	-0,246823204	8,673627549	0,227838446	0,488401655	T1D vs T2D Time-adjusted
hsa-miR-10b-5p_ACCCTGTAGAACCGAATTTGT	-0,300380765	6,802181051	0,228890659	0,489331112	T1D vs T2D Time-adjusted
hsa-miR-423-5p_GAGGGGCAGAGAGCGAGACTTT	0,17138868	8,417582901	0,231471655	0,493515038	T1D vs T2D Time-adjusted
hsa-miR-30d-5p_TGTAAACATCCCCGACTGGAAG	-0,116830348	12,1725605	0,232525867	0,494430002	T1D vs T2D Time-adjusted
hsa-miR-16-5p_TTAGCAGCACGTAAATATTGGCG	-0,641577335	4,214912823	0,234424149	0,497130031	T1D vs T2D Time-adjusted
hsa-miR-126-3p_CTCGTACCGTGAGTAATAATGCG	-0,644943044	4,641889164	0,235371187	0,497803768	T1D vs T2D Time-adjusted
hsa-miR-16-5p_CTAGCAGCACGTAAATATTGGCG	-0,570849942	4,886130646	0,238695231	0,503487808	T1D vs T2D Time-adjusted
hsa-miR-486-5p_ATCCTGTACTGAGCTGCCCCGA	0,234830906	9,024331858	0,240213593	0,504209945	T1D vs T2D Time-adjusted
hsa-miR-191-5p_CAACGGAATCCCAAAAGCAG	-0,154164423	10,17364723	0,240312452	0,504209945	T1D vs T2D Time-adjusted
hsa-miR-125b-5p_TCCCTGAGACCCTAACTT	-0,674356905	2,928962579	0,241651322	0,505677767	T1D vs T2D Time-adjusted
hsa-miR-4446-3p_CAGGGCTGGCAGTGACATGGGT	-0,762291642	3,595995315	0,246087981	0,513603148	T1D vs T2D Time-adjusted
hsa-let-7d-3p_TATACGACCTGCTGCCTTTCT	0,168899477	8,434108679	0,248172034	0,516589682	T1D vs T2D Time-adjusted
hsa-miR-3605-3p_CCTCCGTGTTACCTGTCCTCT	-0,569175256	5,156629775	0,251008345	0,517921477	T1D vs T2D Time-adjusted
hsa-miR-26a-5p_TTCAAGTAATCCAGGATAGGC	-0,208851376	7,521939438	0,251172929	0,517921477	T1D vs T2D Time-adjusted
hsa-miR-125b-5p_TCCCTGAGACCCTAACT	-0,770290471	2,244613923	0,251654953	0,517921477	T1D vs T2D Time-adjusted
hsa-miR-432-5p_TCTTGGAGTAGGTCATTGGGTGG	-0,731388071	3,31432182	0,252078841	0,517921477	T1D vs T2D Time-adjusted
hsa-miR-361-3p_TCCCCCAGGTGTGATTCTGA	-0,638204684	3,266035349	0,252768944	0,517921477	T1D vs T2D Time-adjusted
hsa-miR-574-3p_CACGCTCATGCACACACCCA	-0,664918528	3,290108497	0,253605971	0,517921477	T1D vs T2D Time-adjusted
hsa-miR-424-3p_CAAAACGTGAGGCGCTGCT	-0,637226581	3,07909127	0,253714283	0,517921477	T1D vs T2D Time-adjusted
hsa-miR-30c-5p_TGTAAACATCCTACACTCTC	-0,720443566	2,337351039	0,254049978	0,517921477	T1D vs T2D Time-adjusted
hsa-miR-193a-5p_TGGGTCTTTGCGGGCGAGA	0,727586448	3,485272507	0,255768879	0,51933024	T1D vs T2D Time-adjusted
hsa-miR-10b-5p_TACCCTGTAGAACCGAAT	-0,7083613	2,102144035	0,256565877	0,51933024	T1D vs T2D Time-adjusted

hsa-miR-23a-3p_ATCACATTGCCAGGGATTTCCAA	-0,397070051	6,524370613	0,257842314	0,51933024	T1D vs T2D Time-adjusted
hsa-miR-191-5p_ACGGAATCCCAAAAGCAGCT	-0,687354596	1,917089883	0,257996563	0,51933024	T1D vs T2D Time-adjusted
hsa-miR-1306-5p_CCACCTCCCCTGCAAACGTC	0,627438974	3,895275761	0,258369225	0,51933024	T1D vs T2D Time-adjusted
hsa-miR-361-5p_TTATCAGAATCTCCAGGGG	-0,660715327	2,375013248	0,258680297	0,51933024	T1D vs T2D Time-adjusted
hsa-miR-191-5p_CAACGGAATCCCAAAAGC	-0,663170572	3,145120995	0,259897198	0,520452363	T1D vs T2D Time-adjusted
hsa-miR-23a-3p_TCACATTGCCAGGGATTTC	-0,685232638	2,507144033	0,261733029	0,522805116	T1D vs T2D Time-adjusted
hsa-miR-148b-3p_TCAGTGCATCACAGAACTTTGT	-0,243567382	6,884283732	0,262397792	0,522812729	T1D vs T2D Time-adjusted
hsa-miR-186-5p_AAAGAATTCTCCTTTTGGGCT	-0,644328934	3,93470427	0,267000335	0,530646394	T1D vs T2D Time-adjusted
hsa-miR-22-3p_AGCTGCCAGTTGAAGAACT	-0,687356907	2,719982364	0,269635773	0,534541094	T1D vs T2D Time-adjusted
hsa-miR-486-5p_TGTACTGAGCTGCCCCGAG	-0,482896181	5,107200977	0,272465661	0,538800845	T1D vs T2D Time-adjusted
hsa-miR-22-3p_AAGCTGCCAGTTGAAGAACTGTT	0,64271646	2,813129247	0,275178738	0,542808932	T1D vs T2D Time-adjusted
hsa-miR-501-3p_AATGCACCCGGGCAAGGATTC	-0,650745599	3,029908341	0,277953344	0,546383897	T1D vs T2D Time-adjusted
hsa-miR-99b-5p_CACCCGTAGAACCGACCTTGC	0,185571354	7,928619761	0,279068349	0,546383897	T1D vs T2D Time-adjusted
hsa-miR-155-5p_TTAATGCTAATCGTGATAGGGGT	-0,550842064	4,228033966	0,279598587	0,546383897	T1D vs T2D Time-adjusted
hsa-miR-3615_TCTCTCGGCTCCTCGCGGC	-0,645791579	2,023397327	0,279754081	0,546383897	T1D vs T2D Time-adjusted
hsa-miR-182-5p_TTTGGCAATGGTAGAACTCACA	-0,291688236	6,980287172	0,283046854	0,551453353	T1D vs T2D Time-adjusted
hsa-miR-409-3p_CGAATGTTGCTCGGTGAACCCCTT	-0,689949698	3,582607068	0,288755225	0,561192587	T1D vs T2D Time-adjusted
hsa-let-7d-3p_CTATACGACCTGCTGCCTTT	0,16717476	10,25191544	0,290240213	0,562696099	T1D vs T2D Time-adjusted
hsa-miR-223-5p_CGTGTATTTGACAAGCTGAGTT	-0,555619919	3,834084658	0,291651696	0,564050102	T1D vs T2D Time-adjusted
hsa-miR-4433b-5p_ATGTCCCACCCCCACTCCTGTT	0,473717175	8,088855349	0,293113891	0,56429382	T1D vs T2D Time-adjusted
hsa-miR-3158-3p_AAGGGCTTCCTCTCTGCAGGA	-0,66036151	2,786574243	0,293708258	0,56429382	T1D vs T2D Time-adjusted
hsa-miR-30a-3p_CTTTCAGTCGGATGTTTGCAG	-0,619602359	2,025868208	0,293917894	0,56429382	T1D vs T2D Time-adjusted
hsa-miR-150-5p_CTCCCAACCCTTGTACCAGTG	-0,499970479	4,444846731	0,298176397	0,571083609	T1D vs T2D Time-adjusted
hsa-miR-148a-3p_TCAGTGCACTACAGAACTTT	-0,646032077	4,13799763	0,299264109	0,571782393	T1D vs T2D Time-adjusted
hsa-miR-30e-5p_TGTAAACATCCTTGACTGGAAGCT	-0,084992073	11,68636005	0,304570092	0,578193421	T1D vs T2D Time-adjusted
hsa-miR-425-5p_AATGACACGATCACTCCCGTTGAG	-0,433026162	5,989511543	0,30524396	0,578193421	T1D vs T2D Time-adjusted
hsa-miR-629-5p_TGGGTTTACGTTGGGAGAACT	0,251146372	7,102407997	0,30551216	0,578193421	T1D vs T2D Time-adjusted
hsa-miR-144-5p_GGATATCATCATATACTGTAAG	-0,610923652	4,158150293	0,305543426	0,578193421	T1D vs T2D Time-adjusted
hsa-miR-423-5p_TGAGGGGCAGAGAGCGAGACT	0,139805972	13,36591214	0,306752398	0,579095817	T1D vs T2D Time-adjusted
hsa-miR-125a-5p_TCCCTGAGACCCTTTAACCTGT	-0,138844531	11,50918116	0,307496655	0,5791187	T1D vs T2D Time-adjusted
hsa-miR-2110_TTGGGGAAACGGCCGCTGAGTGA	-0,367283073	6,031153966	0,308340226	0,579328074	T1D vs T2D Time-adjusted
hsa-miR-103a-3p_AGCAGCATTGTACAGGGCTATGA	0,2245253	8,59094218	0,312025007	0,582310396	T1D vs T2D Time-adjusted
hsa-miR-221-3p_AGCTACATTGTCTGCTGGGT	-0,374349225	6,247180713	0,313083988	0,582310396	T1D vs T2D Time-adjusted
hsa-miR-103a-3p_AGCAGCATTGTACAGGGCTAT	-0,743680092	3,194453722	0,313879617	0,582310396	T1D vs T2D Time-adjusted
hsa-miR-23a-3p_ATCACATTGCCAGGGATTTCCA	-0,147369216	11,09453661	0,314247891	0,582310396	T1D vs T2D Time-adjusted
hsa-miR-21-5p_TAGCTTATCAGACTGATGT	-0,520963281	4,642902172	0,31486456	0,582310396	T1D vs T2D Time-adjusted
hsa-miR-3615_TCTCTCGGCTCCTCGCGGCTCGC	-0,566018723	3,765572795	0,315217937	0,582310396	T1D vs T2D Time-adjusted
hsa-miR-451a_CGTTACCATTACTGAGT	-0,504654167	4,231569487	0,31565355	0,582310396	T1D vs T2D Time-adjusted
hsa-miR-144-3p_TACAGTATAGATGATGTACT	-0,501567175	4,591697634	0,315972042	0,582310396	T1D vs T2D Time-adjusted
hsa-miR-223-3p_GTCAGTTTGTCAAATACCCCAA	-0,314420093	7,633945564	0,31655306	0,582310396	T1D vs T2D Time-adjusted
hsa-miR-10b-5p_TACCCTGTAGAACCGAATTTGT	-0,124453041	11,1550449	0,317507002	0,582710067	T1D vs T2D Time-adjusted
hsa-miR-345-5p_GCTGACTCCTAGTCCAGGGCTC	-0,596084824	3,791671294	0,320409892	0,583050517	T1D vs T2D Time-adjusted
hsa-miR-7-5p_TGGAAGACTAGTGATTTTGTTGTT	-0,598162706	4,490449806	0,32053777	0,583050517	T1D vs T2D Time-adjusted
hsa-miR-486-5p_GTACTGAGCTGCCCCGA	-0,439005646	4,600446211	0,321157168	0,583050517	T1D vs T2D Time-adjusted
hsa-miR-342-3p_CTCACACAGAAATCGCACCCG	-0,634044598	2,546589991	0,321799108	0,583050517	T1D vs T2D Time-adjusted
hsa-miR-16-5p_AGCAGCACGTAAATATTGG	-0,567374572	3,202629707	0,32194204	0,583050517	T1D vs T2D Time-adjusted
hsa-let-7g-5p_TGAGGTAGTAGTTTGTACAGTTT	-0,285373422	6,732316769	0,32211514	0,583050517	T1D vs T2D Time-adjusted
hsa-miR-30e-5p_TGTAAACATCCTTGACTGGAAGC	-0,126708188	8,361780675	0,324280092	0,585629116	T1D vs T2D Time-adjusted
hsa-miR-126-3p_CGTACCGTGAGTAATAATGC	-0,418921806	4,480402803	0,328942159	0,592695325	T1D vs T2D Time-adjusted
hsa-miR-23a-5p_GGGGTTCCTGGGGATGGGATTT	-0,546725683	3,892718076	0,330661497	0,593586143	T1D vs T2D Time-adjusted
hsa-miR-320a-3p_AAAAGCTGGGTTGAGAGGGCGA	0,11547297	10,81566962	0,330937407	0,593586143	T1D vs T2D Time-adjusted
hsa-miR-19b-3p_TGTGCAAATCCATGCAAAACTGA	-0,453258992	5,03945579	0,334027282	0,596771173	T1D vs T2D Time-adjusted
hsa-miR-16-2-3p_ACCAATATTACTGTGCTGCTTT	-0,181469446	7,823388697	0,335155291	0,596771173	T1D vs T2D Time-adjusted
hsa-miR-92a-3p_GCACTTGTCCCGGCCTGT	-0,469958329	4,472613341	0,335262531	0,596771173	T1D vs T2D Time-adjusted
hsa-miR-148a-3p_CAGTGCACTACAGAACTTTGT	-0,599424943	2,153358606	0,335730938	0,596771173	T1D vs T2D Time-adjusted
hsa-let-7f-5p_GAGGTAGTAGATTGTATAGTT	-0,372183768	5,929108602	0,336706107	0,597162624	T1D vs T2D Time-adjusted
hsa-miR-10a-5p_ACCCTGTAGATCCGAATTTG	-0,350542862	5,676118316	0,33967636	0,60039723	T1D vs T2D Time-adjusted
hsa-miR-342-3p_TCTCACACAGAAATCGCACCCGTCA	-0,560299115	3,434989001	0,340682636	0,60039723	T1D vs T2D Time-adjusted
hsa-let-7i-5p_TGAGGTAGTAGTTTGTGCTGTTG	-0,519645596	3,944192775	0,341674823	0,60039723	T1D vs T2D Time-adjusted
hsa-miR-24-3p_GGCTCAGTTCAGCAGGAACAG	-0,249392283	6,840568144	0,341717917	0,60039723	T1D vs T2D Time-adjusted
hsa-miR-486-5p_CCTGTACTGAGCTGCCCCGA	0,170655095	11,06703044	0,342325096	0,60039723	T1D vs T2D Time-adjusted

hsa-miR-183-5p_TATGGCACTGGTAGAATTCA	-0,299542541	7,316738774	0,343291519	0,600760158	T1D vs T2D Time-adjusted
hsa-miR-486-5p_TCCTGTACTGAGCTGCCCCGAGC	0,237508542	6,153079419	0,349935874	0,610719713	T1D vs T2D Time-adjusted
hsa-miR-125a-5p_TCCCTGAGACCCTTTAACC	-0,541597933	2,3001783	0,350526865	0,610719713	T1D vs T2D Time-adjusted
hsa-miR-32-5p_TATTGCACATTACTAAGTTGC	-0,567665493	2,139133623	0,353203981	0,613041269	T1D vs T2D Time-adjusted
hsa-miR-423-5p_TGAGGGGCAGAGAGCGAGA	0,118119637	9,771757288	0,353409379	0,613041269	T1D vs T2D Time-adjusted
hsa-miR-150-5p_GTCTCCCAACCCTTGTACCAGTG	-0,576424881	3,077989788	0,354914798	0,613519741	T1D vs T2D Time-adjusted
hsa-miR-92a-3p_TATTGCACTTGTCCCGGCCTG	0,151729916	13,9436113	0,355236462	0,613519741	T1D vs T2D Time-adjusted
hsa-miR-193a-5p_TGGGTCTTTGCGGGCGAGATGA	-0,170373632	8,658900701	0,356371889	0,614139791	T1D vs T2D Time-adjusted
hsa-miR-222-3p_AGCTACATCTGGCTACTGGG	-0,567873591	2,571182702	0,357371379	0,614523393	T1D vs T2D Time-adjusted
hsa-miR-143-3p_GAGATGAAGCACTGTAGCTC	-0,592691896	2,215337676	0,361671414	0,618884615	T1D vs T2D Time-adjusted
hsa-let-7d-5p_AGAGGTAGTAGGTTGCATAGT	-0,248105267	7,384387884	0,361836575	0,618884615	T1D vs T2D Time-adjusted
hsa-miR-223-5p_CGTGTATTTGACAAGCTGAGTTG	-0,371960388	5,926691134	0,362761815	0,618884615	T1D vs T2D Time-adjusted
hsa-let-7d-3p_ATACGACCTGCTGCCTTTCT	-0,55470996	2,005732528	0,363037246	0,618884615	T1D vs T2D Time-adjusted
hsa-miR-433-3p_ATCATGATGGGCTCCTCGGTGT	0,58354401	4,238564116	0,365036546	0,62095464	T1D vs T2D Time-adjusted
hsa-miR-4433b-5p_TGTCCCACCCCCACTCCTGTTT	0,357910495	8,44296889	0,366447512	0,622017128	T1D vs T2D Time-adjusted
hsa-let-7g-5p_TGAGGTAGTAGTTTGTACAG	-0,140850476	9,388462879	0,369865664	0,626474819	T1D vs T2D Time-adjusted
hsa-let-7b-5p_GAGGTAGTAGGTTGTGTGGTT	-0,280639533	6,256027764	0,371218717	0,627423088	T1D vs T2D Time-adjusted
hsa-miR-29a-3p_TAGCACCATCTGAAATCGG	-0,235198232	7,313106214	0,375115132	0,632506312	T1D vs T2D Time-adjusted
hsa-miR-3615_TCTCTCGGCTCCTCGCGGCT	0,283288639	6,628968416	0,375900472	0,632506312	T1D vs T2D Time-adjusted
hsa-miR-17-5p_CAAAGTGCTTACAGTGCAGGTAG	-0,516325529	3,22312992	0,376625124	0,632506312	T1D vs T2D Time-adjusted
hsa-let-7i-5p_GAGGTAGTAGTTTGTGCTG	-0,548081414	2,219476679	0,377971481	0,632536118	T1D vs T2D Time-adjusted
hsa-let-7f-5p_TGAGGTAGTAGATTGTATAG	-0,128273752	10,5471638	0,378300548	0,632536118	T1D vs T2D Time-adjusted
hsa-miR-143-3p_TGAGATGAAGCACTGTAGCT	-0,161183618	9,047567862	0,379421102	0,632536118	T1D vs T2D Time-adjusted
hsa-miR-191-5p_AACGGAATCCCAAAAGCAGCT	-0,235898858	6,832593963	0,379841537	0,632536118	T1D vs T2D Time-adjusted
hsa-miR-4433b-5p_ATGTCCCACCCCCACTCCTGT	0,368363256	7,641633929	0,381704337	0,634302795	T1D vs T2D Time-adjusted
hsa-miR-1301-3p_TTGCAGCTGCCTGGGAGTGACTTC	-0,590905143	2,475326197	0,38253405	0,634348917	T1D vs T2D Time-adjusted
hsa-miR-144-3p_TACAGTATAGATGATGTAC	-0,289771626	6,411669154	0,38454984	0,635553549	T1D vs T2D Time-adjusted
hsa-miR-10b-5p_TACCCTGTAGAACCGAATTTG	0,120074507	9,829860922	0,384908876	0,635553549	T1D vs T2D Time-adjusted
hsa-miR-328-3p_CTGGCCCTCTCTGCCCTTCCGT	0,21813132	7,133297312	0,386612553	0,635553549	T1D vs T2D Time-adjusted
hsa-miR-26b-5p_TTCAAGTAATTCAGGATAGGT	-0,195429211	7,33079836	0,386916996	0,635553549	T1D vs T2D Time-adjusted
hsa-miR-425-5p_AATGACACGATCACTCCCGT	-0,253955657	6,660694598	0,38727789	0,635553549	T1D vs T2D Time-adjusted
hsa-miR-181a-5p_AACATTCAACGCTGTCGGT	-0,359334054	5,49798078	0,390257052	0,638989544	T1D vs T2D Time-adjusted
hsa-let-7c-5p_TGAGGTAGTAGGTTGTATGGTTT	-0,449004286	3,632126079	0,390987281	0,638989544	T1D vs T2D Time-adjusted
hsa-miR-4732-3p_GCCCTGACCTGTCCTGTTCTG	0,806145378	4,166204088	0,39599946	0,645066213	T1D vs T2D Time-adjusted
hsa-miR-486-5p_TCCTGTACTGAGCTGCCCCG	0,15613012	14,12849188	0,396942872	0,645066213	T1D vs T2D Time-adjusted
hsa-miR-144-5p_GATATCATCATATACTGTAAGTT	-0,541734008	2,274281107	0,398175462	0,645066213	T1D vs T2D Time-adjusted
hsa-let-7a-5p_GTGAGGTAGTAGGTTGTATAGTT	-0,549901343	3,003012908	0,398315715	0,645066213	T1D vs T2D Time-adjusted
hsa-miR-16-5p_TAGCAGCACGTAAATATTG	-0,3005172	5,552847943	0,399842761	0,645066213	T1D vs T2D Time-adjusted
hsa-miR-30e-3p_CTTTCAGTCGGATGTTTACAG	-0,494800495	2,479912206	0,399877457	0,645066213	T1D vs T2D Time-adjusted
hsa-miR-485-5p_AGAGGCTGGCCGTGATGAATTCG	-0,492973254	4,445743314	0,401246762	0,645066213	T1D vs T2D Time-adjusted
hsa-miR-339-3p_TGAGCGCCTCGACGACAGAGC	-0,428496235	4,202757825	0,402435142	0,645066213	T1D vs T2D Time-adjusted
hsa-miR-451a_AACCGTTACCATTACTGAGTTT	-0,505675691	2,393187549	0,402671749	0,645066213	T1D vs T2D Time-adjusted
hsa-miR-451a_AACCGTTACCATTACTGAGT	-0,154567697	10,87118749	0,402860568	0,645066213	T1D vs T2D Time-adjusted
hsa-miR-223-3p_TGTCAGTTTGTCAAATACCCC	-0,270338656	7,234009162	0,405878647	0,646170516	T1D vs T2D Time-adjusted
hsa-miR-92a-3p_ACTTGTCCCGGCCTGT	-0,509785456	2,211098977	0,405885071	0,646170516	T1D vs T2D Time-adjusted
hsa-miR-192-5p_TGACCTATGAATTGACAGCC	-0,270480374	6,46151299	0,406000944	0,646170516	T1D vs T2D Time-adjusted
hsa-miR-16-5p_TAGCAGCACGTAAATATTGGCGT	-0,18192057	7,887777382	0,407347593	0,64701194	T1D vs T2D Time-adjusted
hsa-miR-27b-3p_TTCACAGTGGCTAAGTTCTGC	-0,172167978	7,857204391	0,411986902	0,652532444	T1D vs T2D Time-adjusted
hsa-miR-128-3p_TCACAGTGAACCGGTCTCT	-0,179917467	7,735806596	0,4124731	0,652532444	T1D vs T2D Time-adjusted
hsa-miR-421_ATCAACAGACATTAATTGGGCGC	0,469714168	2,015098416	0,413351449	0,65261676	T1D vs T2D Time-adjusted
hsa-miR-10b-5p_TACCCTGTAGAACCGAATT	0,422487908	4,497780085	0,418026527	0,658683232	T1D vs T2D Time-adjusted
hsa-miR-423-3p_AAGCTCGGTCTGAGGCCCCT	-0,511140051	2,52885255	0,421837776	0,663367159	T1D vs T2D Time-adjusted
hsa-miR-133a-3p_TTGGTCCCCTTCAACCAGCTGT	0,535668168	2,487795126	0,423649205	0,664893891	T1D vs T2D Time-adjusted
hsa-miR-93-5p_CAAAGTGCTGTTCGTGCAGGTAGT	-0,459675787	4,055351232	0,426576991	0,665366683	T1D vs T2D Time-adjusted
hsa-miR-1908-5p_CGGCGGGGACGGCGATTGGT	-0,529673713	2,728932328	0,427055192	0,665366683	T1D vs T2D Time-adjusted
hsa-miR-660-5p_TACCCATTGCATATCGGAGTTGT	-0,434340008	4,013928314	0,427141487	0,665366683	T1D vs T2D Time-adjusted
hsa-miR-126-3p_CGTACCGTGAGTAATAATGCG	-0,120015172	9,728392522	0,427651754	0,665366683	T1D vs T2D Time-adjusted
hsa-miR-92a-3p_ATTGCACTTGTCCCGGCCT	-0,41148206	4,481036729	0,428156311	0,665366683	T1D vs T2D Time-adjusted
hsa-miR-486-3p_CGGGGCAGCTCAGTACAGGAT	-0,222955685	7,767064538	0,430098392	0,667074173	T1D vs T2D Time-adjusted
hsa-miR-505-3p_CGTCAACACTTGCTGGTTT	-0,449270956	3,312021603	0,431430156	0,667830242	T1D vs T2D Time-adjusted
hsa-miR-486-5p_ATCCTGTACTGAGCTGCCCCGAG	-0,17915183	8,021810439	0,433645157	0,668662594	T1D vs T2D Time-adjusted

hsa-miR-375-3p_TTTGTTCGTTCGGCTCGCG	-0,339896516	5,940750727	0,434929485	0,668662594	T1D vs T2D Time-adjusted
hsa-miR-363-3p_AATTGCACGGTATCCATCT	-0,410719428	4,448241713	0,43511595	0,668662594	T1D vs T2D Time-adjusted
hsa-miR-140-3p_ACCACAGGGTAGAACCACGGA	-0,256536034	6,607249039	0,435349223	0,668662594	T1D vs T2D Time-adjusted
hsa-miR-92a-3p_TATTGCACTTGTCCCGGCC	-0,191800595	8,042410264	0,437823425	0,671159553	T1D vs T2D Time-adjusted
hsa-miR-182-5p_TTTGGCAATGGTAGAACTCACACTGG	-0,484304729	2,345773362	0,438894145	0,671499553	T1D vs T2D Time-adjusted
hsa-miR-221-3p_AGCTACATTGTCTGCTGGGTTT	-0,151501486	7,93162947	0,440007352	0,671903118	T1D vs T2D Time-adjusted
hsa-miR-323a-3p_CACATTACACGGTCGACCTCT	-0,492928467	3,386474425	0,44312987	0,675367489	T1D vs T2D Time-adjusted
hsa-miR-409-3p_CGAATGTTGCTCGGTGAACCCCTTT	-0,515378768	2,311149768	0,44415011	0,675620649	T1D vs T2D Time-adjusted
hsa-miR-183-5p_TATGGCACTGGTAGAATT	-0,476554874	2,845053887	0,446372479	0,677697949	T1D vs T2D Time-adjusted
hsa-miR-144-5p_GGATATCATCATATACTGTAAGT	0,222164836	6,455704528	0,448832997	0,677958025	T1D vs T2D Time-adjusted
hsa-miR-29a-3p_CTAGCACCATCTGAAATCGGTT	-0,410720226	3,443400968	0,448861556	0,677958025	T1D vs T2D Time-adjusted
hsa-miR-30e-5p_TGTAAACATCCTTGACTGGAAG	-0,156919697	7,594156561	0,449115051	0,677958025	T1D vs T2D Time-adjusted
hsa-miR-1306-5p_CCACCTCCCCTGCAAACGTCC	0,429377942	4,59875793	0,451200996	0,679161261	T1D vs T2D Time-adjusted
hsa-miR-27a-3p_TTCACAGTGGCTAAGTTCCGC	-0,251324985	6,161654231	0,451629359	0,679161261	T1D vs T2D Time-adjusted
hsa-miR-22-3p_AAGCTGCCAGTTGAAGAAC	-0,159470754	8,253643329	0,453180759	0,679484752	T1D vs T2D Time-adjusted
hsa-miR-125b-5p_TCCCTGAGACCCTAACTTGTG	-0,335326213	5,122028633	0,453562514	0,679484752	T1D vs T2D Time-adjusted
hsa-miR-126-5p_CATTATTACTTTTGGTACGC	0,424087453	3,708699797	0,454669158	0,679855017	T1D vs T2D Time-adjusted
hsa-miR-21-5p_TAGCTTATCAGACTGATGTTG	0,099909329	11,65018988	0,456667625	0,680004826	T1D vs T2D Time-adjusted
hsa-miR-199a-5p_CCCAGTGTTCAGACTACCTGTTC	-0,406229542	4,322462141	0,457670963	0,680004826	T1D vs T2D Time-adjusted
hsa-miR-3173-5p_TGCCCTGCCTGTTTTCTCCTT	0,455464572	2,961448405	0,458708495	0,680004826	T1D vs T2D Time-adjusted
hsa-miR-451a_AAACCGTTACCATTACTGAGT	-0,13281801	16,52329399	0,459694716	0,680004826	T1D vs T2D Time-adjusted
hsa-miR-652-3p_AATGGCGCCACTAGGGTTGT	-0,128916972	8,007167607	0,459881364	0,680004826	T1D vs T2D Time-adjusted
hsa-miR-10b-5p_ACCCTGTAGAACCGAATTTG	-0,377816786	4,807768063	0,459964684	0,680004826	T1D vs T2D Time-adjusted
hsa-miR-409-3p_GAATGTTGCTCGGTGAACCCCTT	-0,452074085	5,003955447	0,460787088	0,680004826	T1D vs T2D Time-adjusted
hsa-let-7b-5p_GAGGTAGTAGGTTGTGTGG	0,249928278	6,238396809	0,465805243	0,686130255	T1D vs T2D Time-adjusted
hsa-miR-335-3p_TTTTTCATTATTGCTCCTGACC	-0,421233235	3,947185728	0,46967522	0,688414098	T1D vs T2D Time-adjusted
hsa-let-7d-5p_AGAGGTAGTAGGTTGCATAG	-0,163729173	8,568558263	0,470456113	0,688414098	T1D vs T2D Time-adjusted
hsa-miR-92a-3p_ATTGCACTTGTCCCGGCCTG	0,139319937	9,128354405	0,470904113	0,688414098	T1D vs T2D Time-adjusted
hsa-miR-15b-5p_TAGCAGCACATCATGGTTTA	-0,248033932	5,81103732	0,471376354	0,688414098	T1D vs T2D Time-adjusted
hsa-miR-22-3p_AAGCTGCCAGTTGAAGAACT	-0,102974241	10,281857	0,471707258	0,688414098	T1D vs T2D Time-adjusted
hsa-miR-342-3p_TCTCACACAGAAATCGCACCC	-0,435265568	2,016844553	0,472971747	0,688988309	T1D vs T2D Time-adjusted
hsa-miR-18a-3p_ACTGCCCTAAGTGCTCCTTCT	-0,433098218	3,355078821	0,474912578	0,689285371	T1D vs T2D Time-adjusted
hsa-miR-92a-3p_TGCACTTGTCCCGGCCTGT	-0,289333986	5,30065349	0,474918492	0,689285371	T1D vs T2D Time-adjusted
hsa-miR-29a-3p_TAGCACCATCTGAAATCGGT	-0,15454347	8,735508595	0,479522916	0,692629882	T1D vs T2D Time-adjusted
hsa-miR-486-5p_TCCTGTACTGAGCTGCCCCGA	0,122525818	17,02608896	0,479647657	0,692629882	T1D vs T2D Time-adjusted
hsa-miR-25-3p_CATTGCACTTGTCTCGGTCT	-0,122864743	10,31281854	0,482530094	0,692629882	T1D vs T2D Time-adjusted
hsa-miR-222-3p_AGCTACATCTGGCTACTGGGT	-0,149034825	7,650900477	0,48368321	0,692629882	T1D vs T2D Time-adjusted
hsa-miR-486-5p_TGTACTGAGCTGCCCCGA	-0,404682513	4,53697007	0,483911521	0,692629882	T1D vs T2D Time-adjusted
hsa-miR-140-3p_TACCACAGGGTAGAACCACGGAC	-0,39134728	4,133710403	0,484475627	0,692629882	T1D vs T2D Time-adjusted
hsa-let-7f-5p_TGAGGTAGTAGATTGTATAGTTG	-0,450892916	2,479162802	0,485071321	0,692629882	T1D vs T2D Time-adjusted
hsa-let-7d-3p_TATACGACCTGCTGCCTTT	-0,209839755	6,258064372	0,485718244	0,692629882	T1D vs T2D Time-adjusted
hsa-miR-885-5p_TCCATTACACTACCCTGCCTCT	-0,788396409	4,712163409	0,486285736	0,692629882	T1D vs T2D Time-adjusted
hsa-miR-652-3p_AATGGCGCCACTAGGGTTG	-0,400391741	4,5440088	0,487927621	0,692629882	T1D vs T2D Time-adjusted
hsa-miR-15b-5p_TAGCAGCACATCATGGTTTAC	-0,156596634	7,359413397	0,487936425	0,692629882	T1D vs T2D Time-adjusted
hsa-miR-125b-5p_CCCTGAGACCCTAACTTGT	-0,424521948	2,224797164	0,487962603	0,692629882	T1D vs T2D Time-adjusted
hsa-miR-150-5p_CTCCCAACCCTTGTACCAGT	-0,438825267	3,109098502	0,489499237	0,692629882	T1D vs T2D Time-adjusted
hsa-miR-379-5p_TGGTAGACTATGGAACGTAGG	-0,477883431	3,519537069	0,490279505	0,692629882	T1D vs T2D Time-adjusted
hsa-miR-3173-5p_TGCCCTGCCTGTTTTCTCCTTT	-0,360165642	4,474811161	0,490357438	0,692629882	T1D vs T2D Time-adjusted
hsa-miR-101-3p_GTACAGTACTGTGATAACT	-0,354982509	4,834369948	0,492767028	0,694792725	T1D vs T2D Time-adjusted
hsa-miR-23a-5p_GGGGTTCCTGGGGATGGGATT	-0,390318559	3,763349774	0,494168363	0,695528781	T1D vs T2D Time-adjusted
hsa-miR-320a-3p_AAAGCTGGGTTGAGAGGGCGAA	-0,34088356	4,245030119	0,495345225	0,695946843	T1D vs T2D Time-adjusted
hsa-miR-24-3p_CTGGCTCAGTTCAGCAGGAACAG	-0,406755064	2,043456364	0,496814192	0,696773096	T1D vs T2D Time-adjusted
hsa-miR-342-5p_AGGGGTGCTATCTGTGATTGA	-0,421541361	4,01644439	0,498266448	0,697573028	T1D vs T2D Time-adjusted
hsa-miR-28-5p_AAGGAGCTCACAGTCTATTGAG	-0,413510456	3,305855814	0,501821587	0,700487709	T1D vs T2D Time-adjusted
hsa-miR-182-5p_TTTGGCAATGGTAGAACTCA	-0,180626451	7,904920515	0,503408388	0,700487709	T1D vs T2D Time-adjusted
hsa-miR-101-3p_TACAGTACTGTGATAACTGA	-0,384419234	4,251229709	0,503653769	0,700487709	T1D vs T2D Time-adjusted
hsa-miR-10a-5p_ACCCTGTAGATCCGAATTTGT	-0,141410343	7,710604015	0,503890653	0,700487709	T1D vs T2D Time-adjusted
hsa-miR-193b-5p_CGGGGTTTTGAGGGCGAGATGA	-0,432476249	2,359786934	0,505862413	0,701848843	T1D vs T2D Time-adjusted
hsa-miR-92a-3p_ATTGCACTTGTCCCGGCCTGT	0,085381871	12,30748627	0,506697576	0,701848843	T1D vs T2D Time-adjusted
hsa-miR-3615_TCTCTCGGCTCCTCGCGGCTC	-0,37312155	2,389605161	0,507531654	0,701848843	T1D vs T2D Time-adjusted
hsa-miR-92a-3p_TATTGCACTTGTCCCGGCCT	0,116994091	10,29739458	0,509779861	0,703727521	T1D vs T2D Time-adjusted

hsa-miR-4433b-5p_TGTCCCACCCCCACTCCTGT	0,381698876	4,523145905	0,513153377	0,706158504	T1D vs T2D Time-adjusted
hsa-miR-181a-2-3p_ACCACTGACCGTTGACTGTACC	-0,40391808	2,432112395	0,514341098	0,706158504	T1D vs T2D Time-adjusted
hsa-miR-30a-5p_TGTAAACATCCTCGACTGGA	-0,373098655	3,318329214	0,515019991	0,706158504	T1D vs T2D Time-adjusted
hsa-miR-125a-5p_TCCCTGAGACCCTTTAACCTG	-0,131413985	8,335881469	0,515111829	0,706158504	T1D vs T2D Time-adjusted
hsa-miR-26b-5p_TCAAGTAATTCAGGATAGGTT	-0,378864352	3,617629587	0,516768132	0,707203448	T1D vs T2D Time-adjusted
hsa-miR-328-3p_CTGGCCCTCTCTGCCCTTCCG	-0,322961405	3,896716502	0,519582988	0,709827536	T1D vs T2D Time-adjusted
hsa-miR-486-5p_ATCCTGTACTGAGCTGCCCCG	0,214588395	6,267380926	0,520834077	0,710309922	T1D vs T2D Time-adjusted
hsa-miR-486-3p_CGGGGCAGCTCAGTACAGGA	-0,217272398	6,565196714	0,522602289	0,711494682	T1D vs T2D Time-adjusted
hsa-miR-4685-3p_TCTCCCTTCCTGCCCTGGCT	-0,324945469	4,82214565	0,528723697	0,718591829	T1D vs T2D Time-adjusted
hsa-miR-342-3p_TCACACAGAAATCGCACCCGT	-0,375034537	2,497948576	0,530079471	0,718714277	T1D vs T2D Time-adjusted
hsa-miR-6803-3p_TCCCTCGCCTTCTCACCCTCAG	-0,366642725	2,545548118	0,530631022	0,718714277	T1D vs T2D Time-adjusted
hsa-miR-197-3p_TTCACCACCTTCTCCACCCA	0,357225408	3,766113409	0,534670575	0,722319059	T1D vs T2D Time-adjusted
hsa-miR-486-5p_CCTGTACTGAGCTGCCCCG	0,155028281	8,001774251	0,535118797	0,722319059	T1D vs T2D Time-adjusted
hsa-miR-329-3p_AACACACCTGGTTAACCTCTT	-0,377596717	3,530220069	0,536864681	0,723441162	T1D vs T2D Time-adjusted
hsa-miR-23a-3p_ATCACATTGCCAGGGATTTC	0,095794456	9,519525675	0,543102329	0,73035848	T1D vs T2D Time-adjusted
hsa-miR-224-5p_CAAGTCACTAGTGGTTCCGTTT	-0,375301561	2,890382654	0,543844684	0,73035848	T1D vs T2D Time-adjusted
hsa-miR-16-5p_TAGCAGCACGTAAATATTGG	-0,094402594	10,9873739	0,546870132	0,733176737	T1D vs T2D Time-adjusted
hsa-miR-199a-3p_ACAGTAGTCTGCACATTGGTT	-0,553175771	4,587596	0,54813102	0,733623752	T1D vs T2D Time-adjusted
hsa-miR-26a-5p_TTCAAGTAATCCAGGATAGG	-0,144919738	6,804916619	0,550683441	0,735794935	T1D vs T2D Time-adjusted
hsa-let-7a-5p_TGAGGTAGTAGGTTGTATAGT	-0,058866912	11,89008946	0,552324088	0,736742587	T1D vs T2D Time-adjusted
hsa-let-7d-5p_AGAGGTAGTAGGTTGCATAGTTT	-0,329572314	5,258609386	0,553734674	0,737380685	T1D vs T2D Time-adjusted
hsa-miR-223-5p_CGTGTATTTGACAAGCTGAGTTGG	-0,38395543	2,254955516	0,554860823	0,737638505	T1D vs T2D Time-adjusted
hsa-miR-15b-3p_CGAATCATTATTTGCTGCTCT	0,285783705	4,370495899	0,556059138	0,737991238	T1D vs T2D Time-adjusted
hsa-miR-148b-3p_TCAGTGCATCACAGAACTTTG	-0,256944575	4,984825627	0,557034977	0,738048018	T1D vs T2D Time-adjusted
hsa-miR-486-5p_CCTGTACTGAGCTGCCCCGAG	0,104742988	11,24764136	0,558379302	0,73859202	T1D vs T2D Time-adjusted
hsa-miR-128-3p_TCACAGTGAACCGGTCTCTTT	0,084776213	8,165938943	0,561637179	0,741661117	T1D vs T2D Time-adjusted
hsa-miR-320a-3p_AAAAGCTGGGTTGAGAGGGCGAAA	-0,177592078	6,765627538	0,565066037	0,74428114	T1D vs T2D Time-adjusted
hsa-miR-130b-5p_ACTCTTTCCCTGTTGCACTACT	-0,230826376	5,624339176	0,565876699	0,74428114	T1D vs T2D Time-adjusted
hsa-miR-450b-5p_TTTTGCAATATGTTCCTGAAT	-0,28370248	4,972105489	0,566444054	0,74428114	T1D vs T2D Time-adjusted
hsa-miR-92a-3p_TATTGCACTTGTCCCGGC	-0,163513046	6,622543799	0,570144178	0,747900571	T1D vs T2D Time-adjusted
hsa-miR-505-3p_GTCAACACTTGCTGGTTTCCTCT	0,358877791	2,102281537	0,573437934	0,750975837	T1D vs T2D Time-adjusted
hsa-miR-92a-3p_GTATTGCACTTGTCCCGGCCTGT	-0,112597696	7,697377602	0,581261876	0,759963874	T1D vs T2D Time-adjusted
hsa-miR-140-3p_ACCACAGGGTAGAACCACG	-0,332341151	2,745546867	0,582851325	0,760784486	T1D vs T2D Time-adjusted
hsa-miR-21-5p_GTAGCTTATCAGACTGATGTTGA	-0,336625203	2,233806863	0,586916692	0,764828835	T1D vs T2D Time-adjusted
hsa-miR-125a-5p_TCCCTGAGACCCTTTAACCTGTGA	-0,118335506	8,184259766	0,593194214	0,770750849	T1D vs T2D Time-adjusted
hsa-miR-23a-3p_ATCACATTGCCAGGGATTTCC	-0,057515617	11,11444486	0,593409946	0,770750849	T1D vs T2D Time-adjusted
hsa-miR-10a-5p_TACCCTGTAGATCCGAATT	-0,300326893	3,885245168	0,597385286	0,774417131	T1D vs T2D Time-adjusted
hsa-let-7i-5p_TGAGGTAGTAGTTTGTGCTG	-0,093368373	10,0893919	0,598190729	0,774417131	T1D vs T2D Time-adjusted
hsa-miR-6803-3p_TCCCTCGCCTTCTCACCCTCAGT	0,343721012	2,013232161	0,599685001	0,775083065	T1D vs T2D Time-adjusted
hsa-let-7f-5p_TGAGGTAGTAGATTGTATAGT	-0,054855276	10,58330635	0,601857597	0,775428448	T1D vs T2D Time-adjusted
hsa-miR-92a-3p_TATTGCACTTGTCCCGGCCTGT	0,062911751	17,21206299	0,602240713	0,775428448	T1D vs T2D Time-adjusted
hsa-miR-483-5p_AAGACGGGAGGAAAGAAGGGA	-0,291824149	4,912658385	0,603330906	0,775428448	T1D vs T2D Time-adjusted
hsa-miR-144-3p_CTACAGTATAGATGATGTAC	-0,296460108	3,365989189	0,60476841	0,775428448	T1D vs T2D Time-adjusted
hsa-miR-483-3p_TCACTCCTCTCCTCCCGTCT	0,33934657	2,17353874	0,604853796	0,775428448	T1D vs T2D Time-adjusted
hsa-miR-125b-5p_TCCCTGAGACCCTAACTTGT	0,084858659	9,835959153	0,60614034	0,775512561	T1D vs T2D Time-adjusted
hsa-miR-1-3p_TGGAATGTAAAGAAGTATGTAT	-0,291968775	5,884629396	0,607727576	0,775512561	T1D vs T2D Time-adjusted
hsa-miR-30d-5p_TGTAAACATCCCCGACTGG	-0,082086978	8,103513785	0,607860667	0,775512561	T1D vs T2D Time-adjusted
hsa-miR-145-3p_ATTCCTGGAAATACTGTTCTT	0,30842422	3,68129034	0,609333749	0,776140089	T1D vs T2D Time-adjusted
hsa-miR-24-3p_TGGCTCAGTTCAGCAGGAAC	0,059096097	10,09466061	0,614721046	0,779970339	T1D vs T2D Time-adjusted
hsa-miR-15a-5p_TAGCAGCACATAATGGTTTGT	0,180329616	6,55286919	0,615195483	0,779970339	T1D vs T2D Time-adjusted
hsa-let-7g-5p_GAGGTAGTAGTTTGTACAGTT	-0,309908354	4,325250462	0,615298978	0,779970339	T1D vs T2D Time-adjusted
hsa-miR-150-5p_TCTCCCAACCCTTGTACCA	-0,195516134	6,56516459	0,619758434	0,784366274	T1D vs T2D Time-adjusted
hsa-let-7b-5p_TGAGGTAGTAGGTTGTGTGGTTT	0,063490525	11,27639147	0,620792156	0,784419482	T1D vs T2D Time-adjusted
hsa-miR-99a-5p_AACCCGTAGATCCGATCTT	0,31223386	3,162993064	0,622315187	0,785089814	T1D vs T2D Time-adjusted
hsa-miR-335-3p_GTTTTTCATTATTGCTCCTGACC	0,307047992	2,856849935	0,627959296	0,787842576	T1D vs T2D Time-adjusted
hsa-miR-191-5p_CAACGGAATCCCAAAAGCAGCT	-0,063028396	11,08359666	0,62797152	0,787842576	T1D vs T2D Time-adjusted
hsa-miR-185-5p_TGGAGAGAAAGGCAGTTCCTGA	-0,072419225	8,779876669	0,62811147	0,787842576	T1D vs T2D Time-adjusted
hsa-let-7a-5p_TGAGGTAGTAGGTTGTATAGTTTT	-0,299260826	2,919534024	0,628481246	0,787842576	T1D vs T2D Time-adjusted
hsa-miR-320a-3p_AAAAGCTGGGTTGAGAGGGCG	0,064219173	10,10122965	0,6302663	0,787997408	T1D vs T2D Time-adjusted
hsa-miR-485-5p_AGAGGCTGGCCGTGATGAATTC	-0,319284251	2,188589843	0,630597167	0,787997408	T1D vs T2D Time-adjusted
hsa-miR-99a-5p_AACCCGTAGATCCGATCTTG	-0,102424829	8,068040532	0,636342871	0,793923046	T1D vs T2D Time-adjusted

hsa-miR-183-5p_TATGGCACTGGTAGAATTCACTG	-0,287981255	3,607840844	0,638824467	0,795764021	T1D vs T2D Time-adjusted
hsa-miR-98-5p_TGAGGTAGTAAGTTGTATTG	-0,209514711	4,697784917	0,644608848	0,801706916	T1D vs T2D Time-adjusted
hsa-miR-370-3p_GCCTGCTGGGGTGGAACCTGGT	-0,291184612	2,366107994	0,653037961	0,809003843	T1D vs T2D Time-adjusted
hsa-miR-23b-3p_ATCACATTGCCAGGGATTACCAC	-0,222316857	5,125539346	0,653645581	0,809003843	T1D vs T2D Time-adjusted
hsa-let-7i-5p_TGAGGTAGTAGTTTGTGCTGT	0,057556231	11,42837325	0,654185432	0,809003843	T1D vs T2D Time-adjusted
hsa-miR-122-5p_TGGAGTGTGACAATGGTGTTTGT	-0,15300551	8,317298507	0,654566953	0,809003843	T1D vs T2D Time-adjusted
hsa-let-7i-5p_TGAGGTAGTAGTTTGTGCTGTT	-0,074155002	10,46534644	0,657046379	0,810801382	T1D vs T2D Time-adjusted
hsa-miR-4433b-5p_TATGTCCCACCCCCACTCCTGT	-0,243748656	5,223541258	0,660684564	0,813652622	T1D vs T2D Time-adjusted
hsa-let-7a-5p_TGAGGTAGTAGGTTGTATAGTTT	-0,10100567	9,339383804	0,661434093	0,813652622	T1D vs T2D Time-adjusted
hsa-miR-192-5p_CTGACCTATGAATTGACAGC	-0,264819099	2,069053686	0,662442843	0,813652622	T1D vs T2D Time-adjusted
hsa-miR-486-5p_TCCTGTACTGAGCTGCCC	-0,179798451	3,876048404	0,666825785	0,816369834	T1D vs T2D Time-adjusted
hsa-miR-423-3p_AGCTCGGTCTGAGGCCCC	-0,234678949	3,601847184	0,667014428	0,816369834	T1D vs T2D Time-adjusted
hsa-miR-1301-3p_TTGCAGCTGCCTGGGAGTGA	-0,269519353	3,323484613	0,667751305	0,816369834	T1D vs T2D Time-adjusted
hsa-miR-1306-5p_CCACCTCCCCTGCAAACGTCCA	0,268022915	3,045463797	0,670430121	0,818001092	T1D vs T2D Time-adjusted
hsa-miR-224-5p_CAAGTCACTAGTGGTTCCGTTTAG	-0,254230252	4,656228151	0,671153867	0,818001092	T1D vs T2D Time-adjusted
hsa-miR-26b-5p_TTCAAGTAATTCAGGATAGGTT	-0,08174787	9,792607635	0,672882964	0,818846807	T1D vs T2D Time-adjusted
hsa-miR-182-5p_TTTGGCAATGGTAGAACTCACACT	0,110342124	7,646583987	0,675015579	0,82018022	T1D vs T2D Time-adjusted
hsa-miR-15b-5p_TAGCAGCACATCATGGTTT	0,085515662	8,509089064	0,67658793	0,820829835	T1D vs T2D Time-adjusted
hsa-miR-409-3p_GAATGTTGCTCGGTGAACCCCT	-0,172203534	7,83201482	0,680880107	0,824721668	T1D vs T2D Time-adjusted
hsa-miR-340-5p_TTATAAAGCAATGAGACTGATT	-0,160156284	5,720859509	0,681881126	0,824721668	T1D vs T2D Time-adjusted
hsa-miR-654-3p_TATGTCTGCTGACCATCACC	-0,269002732	2,446876993	0,684483669	0,826021442	T1D vs T2D Time-adjusted
hsa-miR-10a-5p_TACCCTGTAGATCCGAATTTG	-0,068245023	8,877087473	0,685044331	0,826021442	T1D vs T2D Time-adjusted
hsa-miR-106b-5p_TAAAGTGCTGACAGTGCAGA	-0,17688278	5,030812023	0,689545991	0,828731075	T1D vs T2D Time-adjusted
hsa-miR-374a-5p_TTATAATACAACCTGATAAGTG	-0,183810403	4,298678316	0,689707834	0,828731075	T1D vs T2D Time-adjusted
hsa-miR-1908-5p_CGGCGGGGACGGCGATTGGTC	-0,243452837	2,7409573	0,690802271	0,828731075	T1D vs T2D Time-adjusted
hsa-let-7f-5p_TGAGGTAGTAGATTGTATAGTTT	-0,11709486	8,014292524	0,691482313	0,828731075	T1D vs T2D Time-adjusted
hsa-let-7g-5p_TGAGGTAGTAGTTTGTACAGTT	-0,051787841	11,2336953	0,693508344	0,829901816	T1D vs T2D Time-adjusted
hsa-miR-329-3p_AACACACCTGGTTAACCTCTTT	-0,242229395	3,551980192	0,694767605	0,830152834	T1D vs T2D Time-adjusted
hsa-miR-486-5p_TCCTGTACTGAGCTGCCCC	0,100322643	6,726361248	0,696733975	0,831246719	T1D vs T2D Time-adjusted
hsa-miR-126-3p_TCGTACCGTGAGTAATAATG	-0,120296431	5,99055736	0,698100314	0,831622513	T1D vs T2D Time-adjusted
hsa-miR-382-5p_AAGTTGTTCGTGGTGGATTCG	-0,256147829	2,276351927	0,699913009	0,831894619	T1D vs T2D Time-adjusted
hsa-let-7c-5p_TGAGGTAGTAGGTTGTATGGT	-0,141370156	4,826004461	0,700432132	0,831894619	T1D vs T2D Time-adjusted
hsa-miR-28-5p_AAGGAGCTCACAGTCTATTGA	-0,244924929	3,153941235	0,701748199	0,831897788	T1D vs T2D Time-adjusted
hsa-miR-501-3p_AATGCACCCGGGCAAGGAT	-0,235008922	2,776935459	0,702538208	0,831897788	T1D vs T2D Time-adjusted
hsa-miR-423-5p_TGAGGGGCAGAGAGCGAGACTTTT	0,042557913	11,36605873	0,704060342	0,832454007	T1D vs T2D Time-adjusted
hsa-miR-423-3p_GCTCGGTCTGAGGCCCCTCAGT	-0,126630333	6,437706495	0,708610776	0,836583766	T1D vs T2D Time-adjusted
hsa-miR-320a-3p_GAAAAGCTGGGTTGAGAGGGCG	-0,118912285	6,257483702	0,714223662	0,841953676	T1D vs T2D Time-adjusted
hsa-miR-185-5p_TGGAGAGAAAGGCAGTTCCT	-0,225906745	2,073411525	0,718456823	0,845683552	T1D vs T2D Time-adjusted
hsa-miR-30a-5p_TGTAAACATCCTCGACTGG	-0,181827283	4,667345631	0,720901028	0,846375959	T1D vs T2D Time-adjusted
hsa-miR-340-5p_TTATAAAGCAATGAGACTGAT	0,21544454	1,903930861	0,722115983	0,846375959	T1D vs T2D Time-adjusted
hsa-miR-142-3p_TGTAGTGTTTCCTACTTTATGGA	0,204750359	3,574791071	0,722996876	0,846375959	T1D vs T2D Time-adjusted
hsa-miR-21-5p_AGCTTATCAGACTGATGTTG	-0,134228667	5,242282712	0,723325093	0,846375959	T1D vs T2D Time-adjusted
hsa-miR-451a_AACCGTTACCATTACTGAG	-0,073980074	8,086006518	0,729708983	0,852584647	T1D vs T2D Time-adjusted
hsa-miR-32-5p_TATTGCACATTACTAAGTTG	-0,192758061	4,122651019	0,731412317	0,852698107	T1D vs T2D Time-adjusted
hsa-miR-501-3p_AATGCACCCGGGCAAGGATT	-0,098358429	6,566869383	0,732486792	0,852698107	T1D vs T2D Time-adjusted
hsa-miR-15b-5p_TAGCAGCACATCATGGTT	0,102962919	7,409647991	0,734516611	0,852698107	T1D vs T2D Time-adjusted
hsa-miR-375-3p_TTTGTTCGTTCGGCTCGCGTGA	0,088549622	10,24220516	0,734896314	0,852698107	T1D vs T2D Time-adjusted
hsa-let-7d-5p_AGAGGTAGTAGGTTGCATAGTT	0,072967762	9,029809844	0,735247336	0,852698107	T1D vs T2D Time-adjusted
hsa-miR-486-5p_CTGTACTGAGCTGCCCCGAG	0,080640882	7,807179841	0,736274092	0,852698107	T1D vs T2D Time-adjusted
hsa-miR-30c-5p_TGTAAACATCCTACACTCTCAG	-0,156869885	4,704062458	0,744319894	0,8607559	T1D vs T2D Time-adjusted
hsa-miR-206_TGGAATGTAAGGAAGTGTGTGG	-0,211607379	2,186074312	0,749586724	0,865581166	T1D vs T2D Time-adjusted
hsa-miR-16-5p_AGCAGCACGTAAATATTGGC	-0,180673266	3,090604583	0,750941375	0,865881381	T1D vs T2D Time-adjusted
hsa-miR-652-3p_AATGGCGCCACTAGGGTTGTGC	0,174124133	3,398629288	0,756499519	0,871020552	T1D vs T2D Time-adjusted
hsa-miR-424-3p_CAAAACGTGAGGCGCTGCTAT	-0,10847711	5,546308926	0,758270387	0,871790518	T1D vs T2D Time-adjusted
hsa-miR-425-5p_ATGACACGATCACTCCCGTTGAGT	0,162411577	4,481314722	0,767965079	0,88022111	T1D vs T2D Time-adjusted
hsa-miR-335-5p_TCAAGAGCAATAACGAAAAATG	-0,063076921	8,353751053	0,768754936	0,88022111	T1D vs T2D Time-adjusted
hsa-miR-15a-5p_TAGCAGCACATAATGGTTTG	-0,224827652	2,765762945	0,77064109	0,88022111	T1D vs T2D Time-adjusted
hsa-miR-29b-3p_TAGCACCATTTGAAATCAGT	-0,149871809	4,397473372	0,770887936	0,88022111	T1D vs T2D Time-adjusted
hsa-miR-146a-5p_GAGAACTGAATTCCATGGGTT	-0,109838088	6,482797306	0,772785519	0,88022111	T1D vs T2D Time-adjusted
hsa-miR-409-3p_CGAATGTTGCTCGGTGAACCCCT	0,158764183	5,552259607	0,773272532	0,88022111	T1D vs T2D Time-adjusted
hsa-miR-629-5p_TGGGTTTACGTTGGGAGAAC	0,170737782	3,80742001	0,773392758	0,88022111	T1D vs T2D Time-adjusted

hsa-miR-361-3p_CCCCCAGGTGTGATTCTGATTTG	0,175401124	1,935808496	0,77553511	0,881391195	T1D vs T2D Time-adjusted
hsa-miR-92a-3p_TATTGCACTTGTCCCGGCCTGTG	-0,052066509	7,12439601	0,777747574	0,88263749	T1D vs T2D Time-adjusted
hsa-miR-23a-3p_ATCACATTGCCAGGGATTT	-0,051919513	7,89161665	0,779430909	0,883118743	T1D vs T2D Time-adjusted
hsa-miR-28-3p_CACTAGATTGTGAGCTCCTGGAG	-0,158507811	3,068939292	0,780404553	0,883118743	T1D vs T2D Time-adjusted
hsa-miR-423-5p_GAGGGGCAGAGAGCGAGACT	-0,093157289	6,892897172	0,784146093	0,885466069	T1D vs T2D Time-adjusted
hsa-miR-4732-5p_TGTAGAGCAGGGAGCAGGAAG	-0,179138466	2,884522745	0,785161451	0,885466069	T1D vs T2D Time-adjusted
hsa-miR-222-3p_AGCTACATCTGGCTACTGGGTCTCT	-0,077262017	7,874596057	0,785941366	0,885466069	T1D vs T2D Time-adjusted
hsa-miR-16-5p_TAGCAGCACGTAAATATTGGCG	-0,035407077	14,02532692	0,787947945	0,885466069	T1D vs T2D Time-adjusted
hsa-miR-342-3p_TCTCACACAGAAATCGCACCCG	0,064001183	9,698837248	0,788075995	0,885466069	T1D vs T2D Time-adjusted
hsa-miR-483-5p_AAGACGGGAGGAAAGAAGGGAG	-0,104098848	6,695020301	0,794436704	0,89104677	T1D vs T2D Time-adjusted
hsa-miR-4433b-5p_ATGTCCCACCCCCACTCCTG	0,1559465	1,885888634	0,795295853	0,89104677	T1D vs T2D Time-adjusted
hsa-miR-99b-5p_ACCCGTAGAACCGACCTTGCG	-0,157393626	2,144852077	0,798691257	0,893585267	T1D vs T2D Time-adjusted
hsa-miR-10a-5p_TACCCTGTAGATCCGAAT	0,157799605	2,390493785	0,802647802	0,895704362	T1D vs T2D Time-adjusted
hsa-miR-629-5p_TGGGTTTACGTTGGGAGAACTT	-0,130562841	4,102285673	0,802850054	0,895704362	T1D vs T2D Time-adjusted
hsa-miR-193a-5p_TGGGTCTTTGCGGGCGAGATG	-0,101420549	5,874910096	0,804143313	0,895883606	T1D vs T2D Time-adjusted
hsa-let-7b-5p_TGAGGTAGTAGGTTGTGTGGTT	-0,023966648	12,80806781	0,806243704	0,896960295	T1D vs T2D Time-adjusted
hsa-miR-486-5p_CTGTACTGAGCTGCCCCGA	-0,055960177	7,578479757	0,807854513	0,897490056	T1D vs T2D Time-adjusted
hsa-miR-92a-3p_CACTTGTCCCGGCCTGT	-0,12896795	4,184588919	0,809488448	0,897563192	T1D vs T2D Time-adjusted
hsa-miR-323b-3p_CCCAATACACGGTCGACCTCT	0,124498443	5,843242142	0,810189784	0,897563192	T1D vs T2D Time-adjusted
hsa-miR-382-5p_GAAGTTGTTCGTGGTGGATTCG	0,093303145	7,539547941	0,812868092	0,899165813	T1D vs T2D Time-adjusted
hsa-miR-21-5p_TAGCTTATCAGACTGATGTTGACT	0,122147836	5,746314182	0,814618672	0,899165813	T1D vs T2D Time-adjusted
hsa-miR-423-3p_AGCTCGGTCTGAGGCCCCTCAGT	0,031004714	11,77370697	0,81589779	0,899165813	T1D vs T2D Time-adjusted
hsa-miR-424-5p_CAGCAGCAATTCATGTTTTGA	0,141951916	2,664190598	0,81618338	0,899165813	T1D vs T2D Time-adjusted
hsa-miR-199a-3p_ACAGTAGTCTGCACATTGGTTA	-0,19991455	4,923172942	0,819219433	0,90103824	T1D vs T2D Time-adjusted
hsa-miR-16-5p_AGCAGCACGTAAATATTGGCG	-0,043530434	8,159714087	0,821099806	0,90103824	T1D vs T2D Time-adjusted
hsa-miR-24-3p_TGGCTCAGTTCAGCAGGAACAG	0,025122673	12,80068155	0,821300342	0,90103824	T1D vs T2D Time-adjusted
hsa-miR-144-5p_GGATATCATCATATACTGTAA	-0,13377675	4,029614813	0,822487018	0,901090348	T1D vs T2D Time-adjusted
hsa-miR-375-3p_TTGTTCGTTCGGCTCGCGTGA	0,101275104	5,794174671	0,82396114	0,901456794	T1D vs T2D Time-adjusted
hsa-miR-92b-3p_TATTGCACTCGTCCCGGCCT	-0,098184363	5,642753779	0,826476302	0,902959606	T1D vs T2D Time-adjusted
hsa-miR-145-3p_ATTCCTGGAAATACTGTTCT	0,130633689	3,093740679	0,832483235	0,908267916	T1D vs T2D Time-adjusted
hsa-miR-664a-3p_TATTCATTTATCCCCAGCCTACA	-0,119890187	2,990893166	0,836830173	0,910675078	T1D vs T2D Time-adjusted
hsa-miR-30d-5p_GTAAACATCCCCGACTGGAAG	0,066260101	5,943376472	0,836992138	0,910675078	T1D vs T2D Time-adjusted
hsa-miR-21-5p_TAGCTTATCAGACTGATGTTGAC	0,051254848	10,35786285	0,838354654	0,910904576	T1D vs T2D Time-adjusted
hsa-miR-148a-3p_TCAGTGCACTACAGAACTT	0,128818463	2,313074364	0,84156786	0,91098718	T1D vs T2D Time-adjusted
hsa-miR-26a-5p_TTCAAGTAATCCAGGATAGGCT	-0,033935699	11,89898908	0,843398155	0,91098718	T1D vs T2D Time-adjusted
hsa-miR-101-3p_GTACAGTACTGTGATAACTGA	-0,034865731	8,212735415	0,84410573	0,91098718	T1D vs T2D Time-adjusted
hsa-miR-139-3p_TGGAGACGCGGCCCTGTTGGAGT	0,118952087	3,616305041	0,844219524	0,91098718	T1D vs T2D Time-adjusted
hsa-miR-21-5p_TAGCTTATCAGACTGATGTT	-0,042124688	7,181930249	0,845139696	0,91098718	T1D vs T2D Time-adjusted
hsa-miR-150-5p_TCTCCCAACCCTTGTACCAG	0,049517066	9,064957556	0,845340822	0,91098718	T1D vs T2D Time-adjusted
hsa-miR-543_AAACATTCGCGGTGCACTTCTTT	-0,1265157	3,18614348	0,851766264	0,915832181	T1D vs T2D Time-adjusted
hsa-miR-192-5p_TGACCTATGAATTGACAGC	0,117188735	3,769796882	0,852478582	0,915832181	T1D vs T2D Time-adjusted
hsa-miR-485-3p_GTCATACACGGCTCTCCTCTCT	-0,112353335	5,721064879	0,853310135	0,915832181	T1D vs T2D Time-adjusted
hsa-miR-92a-3p_TATTGCACTTGTCCCGGCCTGTT	0,024997655	10,36371218	0,855451352	0,916886206	T1D vs T2D Time-adjusted
hsa-miR-375-3p_TTTGTTCGTTCGGCTCGCGTG	-0,062759273	7,069245953	0,860030714	0,920278938	T1D vs T2D Time-adjusted
hsa-let-7a-5p_TGAGGTAGTAGGTTGTAT	0,125659066	3,337435577	0,861917374	0,920278938	T1D vs T2D Time-adjusted
hsa-miR-101-3p_TACAGTACTGTGATAACTGAAG	-0,048763552	6,912528742	0,862107071	0,920278938	T1D vs T2D Time-adjusted
hsa-miR-98-5p_TGAGGTAGTAAGTTGTATTGT	-0,058521285	6,564741993	0,863908485	0,920959045	T1D vs T2D Time-adjusted
hsa-miR-103a-3p_AGCAGCATTGTACAGGGCT	0,149222927	5,276241931	0,867520494	0,923564886	T1D vs T2D Time-adjusted
hsa-miR-320a-3p_AAAGCTGGGTTGAGAGGGCG	0,082016584	4,800125051	0,871282906	0,926323627	T1D vs T2D Time-adjusted
hsa-miR-191-5p_CAACGGAATCCCAAAAGCAGCTG	-0,029610562	9,63370255	0,874112797	0,928084862	T1D vs T2D Time-adjusted
hsa-miR-486-5p_TCCTGTACTGAGCTGCCCCGAG	0,025652765	17,20272224	0,87727846	0,930197402	T1D vs T2D Time-adjusted
hsa-let-7d-3p_CTATACGACCTGCTGCCTTTCT	0,017756398	11,72675264	0,878667057	0,930422547	T1D vs T2D Time-adjusted
hsa-miR-23a-3p_TCACATTGCCAGGGATTTCC	-0,079969328	4,52192031	0,883462804	0,934250104	T1D vs T2D Time-adjusted
hsa-miR-320b_AAAAGCTGGGTTGAGAGGGC	-0,134877516	5,096748857	0,885840151	0,935513431	T1D vs T2D Time-adjusted
hsa-miR-27a-3p_TTCACAGTGGCTAAGTTCCG	-0,018233332	9,746526676	0,894498008	0,943397233	T1D vs T2D Time-adjusted
hsa-miR-92b-3p_TATTGCACTCGTCCCGGCCTC	0,067443852	3,950927699	0,899122865	0,947012232	T1D vs T2D Time-adjusted
hsa-miR-423-3p_AAGCTCGGTCTGAGGCCCCTCAGT	-0,027484513	7,381534421	0,903114935	0,949952013	T1D vs T2D Time-adjusted
hsa-miR-142-5p_CATAAAGTAGAAAGCACTACT	-0,063743205	3,738908441	0,90564731	0,951350627	T1D vs T2D Time-adjusted
hsa-miR-16-2-3p_ACCAATATTACTGTGCTGCTT	-0,021523715	8,335786686	0,909984494	0,954596597	T1D vs T2D Time-adjusted
hsa-miR-92a-3p_TTGCACTTGTCCCGGCCTGT	0,018972444	8,379067596	0,911150987	0,954596597	T1D vs T2D Time-adjusted
hsa-miR-29c-3p_TAGCACCATTTGAAATCGGT	-0,05903494	4,535898634	0,913236852	0,954902746	T1D vs T2D Time-adjusted

hsa-miR-423-3p_AGCTCGGTCTGAGGCCCCTCAG	0,015554808	8,394585646	0,914793639	0,954902746	T1D vs T2D Time-adjusted
hsa-let-7b-5p_TGAGGTAGTAGGTTGTGTG	-0,028378009	6,901751547	0,915064831	0,954902746	T1D vs T2D Time-adjusted
hsa-let-7a-5p_TGAGGTAGTAGGTTGTATAGTT	-0,013582253	13,3879175	0,920957609	0,959785861	T1D vs T2D Time-adjusted
hsa-miR-499a-5p_TTAAGACTTGCAGTGATGTTT	-0,060305571	2,030187923	0,922737636	0,960375618	T1D vs T2D Time-adjusted
hsa-miR-145-5p_GTCCAGTTTTCCCAGGAATCCCT	0,058544283	1,974319363	0,92538637	0,961866779	T1D vs T2D Time-adjusted
hsa-miR-323a-3p_GCACATTACACGGTCGACCTCT	-0,053823403	3,791926149	0,928274385	0,96298545	T1D vs T2D Time-adjusted
hsa-miR-26a-5p_TTCAAGTAATCCAGGATAG	-0,049268442	3,674214058	0,930387538	0,96298545	T1D vs T2D Time-adjusted
hsa-miR-21-5p_TAGCTTATCAGACTGATGTTGA	-0,007899432	12,8658918	0,930466734	0,96298545	T1D vs T2D Time-adjusted
hsa-miR-103a-3p_AGCAGCATTGTACAGGGC	-0,061071381	3,250498272	0,932073404	0,96298545	T1D vs T2D Time-adjusted
hsa-miR-345-5p_GCTGACTCCTAGTCCAGGGCT	-0,039179297	5,007179685	0,932549753	0,96298545	T1D vs T2D Time-adjusted
hsa-miR-30e-5p_GTAAACATCCTTGACTGGAAGCT	0,00834091	8,789096373	0,946319178	0,975082996	T1D vs T2D Time-adjusted
hsa-miR-20a-5p_TAAAGTGCTTATAGTGCAGGTAG	-0,033093988	3,856097276	0,946730393	0,975082996	T1D vs T2D Time-adjusted
hsa-miR-3613-5p_TGTTGTACTTTTTTTTTTGT	0,011629551	7,575367225	0,950806133	0,978007349	T1D vs T2D Time-adjusted
hsa-miR-125b-5p_TCCCTGAGACCCTAACTTG	0,011456724	8,160127514	0,953063377	0,978889207	T1D vs T2D Time-adjusted
hsa-miR-150-5p_TCTCCCAACCCTTGTACCAGTG	-0,009952914	11,04001159	0,954138532	0,978889207	T1D vs T2D Time-adjusted
hsa-miR-130a-3p_CAGTGCAATGTTAAAAGGGCA	-0,024662706	4,392768352	0,958374624	0,981961564	T1D vs T2D Time-adjusted
hsa-miR-423-5p_TGAGGGGCAGAGAGCGAGAC	-0,005088009	9,81018722	0,964204832	0,985014416	T1D vs T2D Time-adjusted
hsa-miR-320a-3p_AAAGCTGGGTTGAGAGGGCGA	0,020955079	5,194526481	0,964882604	0,985014416	T1D vs T2D Time-adjusted
hsa-miR-24-3p_GGCTCAGTTCAGCAGGAACA	0,023867348	2,68639799	0,967868536	0,985014416	T1D vs T2D Time-adjusted
hsa-let-7f-5p_TGAGGTAGTAGATTGTATAGTT	0,006569639	12,47888054	0,968263419	0,985014416	T1D vs T2D Time-adjusted
hsa-miR-150-5p_TCTCCCAACCCTTGTACCAGT	0,008195974	10,28667682	0,968305864	0,985014416	T1D vs T2D Time-adjusted
hsa-miR-24-3p_GGCTCAGTTCAGCAGGAAC	-0,025961068	2,918934836	0,96882581	0,985014416	T1D vs T2D Time-adjusted
hsa-miR-423-5p_AGGGGCAGAGAGCGAGACT	-0,021780731	3,23300129	0,971687667	0,986655898	T1D vs T2D Time-adjusted
hsa-miR-369-3p_AATAATACATGGTTGATCTTT	-0,020820746	4,128023185	0,9745982	0,988342534	T1D vs T2D Time-adjusted
hsa-miR-98-5p_TGAGGTAGTAAGTTGTATTGTT	-0,007935556	7,833511387	0,977729592	0,990248537	T1D vs T2D Time-adjusted
hsa-miR-374a-5p_TTATAATACAACCTGATAAGT	-0,016160256	2,272272471	0,979368083	0,990639583	T1D vs T2D Time-adjusted
hsa-miR-501-3p_AATGCACCCGGGCAAGGATTCT	0,002763434	7,8797274	0,987621701	0,994859783	T1D vs T2D Time-adjusted
hsa-let-7a-5p_ATGAGGTAGTAGGTTGTATAGTT	-0,008450872	2,145462754	0,98932963	0,994859783	T1D vs T2D Time-adjusted
hsa-miR-423-5p_AGGGGCAGAGAGCGAGACTTT	0,0055855	5,179351064	0,989661559	0,994859783	T1D vs T2D Time-adjusted
hsa-miR-409-3p_AATGTTGCTCGGTGAACCCCT	-0,008188887	4,168161381	0,98990407	0,994859783	T1D vs T2D Time-adjusted
hsa-miR-27a-3p_TTCACAGTGGCTAAGTTCC	-0,004899862	5,362754207	0,989983975	0,994859783	T1D vs T2D Time-adjusted
hsa-miR-483-5p_AAGACGGGAGGAAAGAAGGGAGT	-0,006533821	4,22603991	0,99155946	0,994859783	T1D vs T2D Time-adjusted
hsa-miR-361-5p_TTATCAGAATCTCCAGGGGTACT	0,005612636	2,680756048	0,992344335	0,994859783	T1D vs T2D Time-adjusted
hsa-miR-4433b-5p_ATGTCCCACCCCCACTCCTGTTT	0,0022755	5,3584586	0,996728889	0,997849634	T1D vs T2D Time-adjusted
hsa-let-7a-5p_GAGGTAGTAGGTTGTATAGTT	-0,000641066	7,12979206	0,997849634	0,997849634	T1D vs T2D Time-adjusted
hsa-miR-125a-5p_TCCCTGAGACCCTTTAACCT1	1,056747689	6,333504859	6,88668E-05	0,042587562	LADA vs T1D Time-adjusted
hsa-miR-130b-3p_CAGTGCAATGATGAAAGGGCA1	1,634693493	4,356329159	0,00010768	0,042587562	LADA vs T1D Time-adjusted
hsa-miR-10b-5p_TACCCTGTAGAACCGAATTTGT1	0,410917687	11,1550449	0,000656409	0,103882797	LADA vs T1D Time-adjusted
hsa-miR-423-5p_CTGAGGGGCAGAGAGCGAGACT1	1,91207212	2,738837203	0,00066246	0,103882797	LADA vs T1D Time-adjusted
hsa-miR-139-5p_TCTACAGTGCACGTGTCTCCA1	1,499264584	4,89012497	0,000717861	0,103882797	LADA vs T1D Time-adjusted
hsa-miR-23b-3p_ATCACATTGCCAGGGATTACC1	1,237550312	5,450967457	0,000787986	0,103882797	LADA vs T1D Time-adjusted
hsa-miR-23b-3p_ATCACATTGCCAGGGATTAC1	1,603658027	2,73633577	0,001493275	0,152343595	LADA vs T1D Time-adjusted
hsa-miR-27b-3p_TTCACAGTGGCTAAGTTCTGCA1	1,633426886	3,816952744	0,00169702	0,152343595	LADA vs T1D Time-adjusted
hsa-miR-30a-5p_TGTAAACATCCTCGACTGGAAGCT1	0,408432279	8,865025747	0,001733366	0,152343595	LADA vs T1D Time-adjusted
hsa-let-7f-5p_TGAGGTAGTAGATTGTATA1	1,662080063	3,718372609	0,001958328	0,15490378	LADA vs T1D Time-adjusted
hsa-miR-125a-5p_TCCCTGAGACCCTTTAACCTGTG1	0,505616584	8,651356777	0,002176706	0,156524934	LADA vs T1D Time-adjusted
hsa-miR-10a-5p_TACCCTGTAGATCCGAATTTGTG1	0,735043776	7,331499904	0,002509382	0,165410087	LADA vs T1D Time-adjusted
hsa-miR-451a_AAACCGTTACCATTACTG1	1,533340828	2,543007428	0,003891081	0,23675734	LADA vs T1D Time-adjusted
hsa-miR-223-3p_TGTCAGTTTGTCAAATACC1	1,101121484	5,277889688	0,005491482	0,304645168	LADA vs T1D Time-adjusted
hsa-miR-451a_GAAACCGTTACCATTACTGAG1	1,446895287	3,175112451	0,005785153	0,304645168	LADA vs T1D Time-adjusted
hsa-miR-542-3p_TGTGACAGATTGATAACTGA1	1,60072986	2,0314731	0,006162228	0,304645168	LADA vs T1D Time-adjusted
hsa-miR-30a-5p_TGTAAACATCCTCGACTGGAA1	0,596247519	7,206633348	0,007122012	0,32545822	LADA vs T1D Time-adjusted
hsa-miR-10b-5p_TACCCTGTAGAACCGAATTTGTG1	0,565776188	7,887217076	0,00765554	0,32545822	LADA vs T1D Time-adjusted
hsa-miR-99b-5p_CACCCGTAGAACCGACCTTGCG1	0,432148479	9,362081621	0,008417618	0,32545822	LADA vs T1D Time-adjusted
hsa-miR-10a-5p_ACCCTGTAGATCCGAATTTGTG1	0,672040565	6,333214322	0,008436125	0,32545822	LADA vs T1D Time-adjusted
hsa-miR-23a-3p_TCACATTGCCAGGGATTTCCAAC1	1,44036638	1,909179417	0,008640484	0,32545822	LADA vs T1D Time-adjusted
hsa-miR-151a-3p_TACTAGACTGAAGCTCCTTGAGG1	1,2880224	3,949800351	0,00920233	0,326993584	LADA vs T1D Time-adjusted
hsa-miR-128-3p_TCACAGTGAACCGGTCTCTTTT1	1,126073451	5,206370676	0,009814059	0,326993584	LADA vs T1D Time-adjusted
hsa-miR-342-3p_TCTCACACAGAAATCGCACCCGTC1	0,538546803	7,588021781	0,01024757	0,326993584	LADA vs T1D Time-adjusted
hsa-let-7e-5p_TGAGGTAGGAGGTTGTATAGT1	1,13790392	4,76183341	0,01073539	0,326993584	LADA vs T1D Time-adjusted
hsa-miR-126-5p_CATTATTACTTTTGGTACGCG1	0,727406498	6,552150257	0,010802757	0,326993584	LADA vs T1D Time-adjusted

hsa-miR-342-3p_TCTCACACAGAAATCGCACCCGTCA1	1,438468832	3,434989001	0,011161601	0,326993584	LADA vs T1D Time-adjusted
hsa-miR-30a-5p_TGTAAACATCCTCGACTGGAAGC1	0,476726577	7,811257993	0,01205249	0,32989029	LADA vs T1D Time-adjusted
hsa-miR-125a-5p_TCCCTGAGACCCTTTAACCTGT1	0,328069173	11,50918116	0,012094587	0,32989029	LADA vs T1D Time-adjusted
hsa-miR-20a-5p_TAAAGTGCTTATAGTGCAGGTAG1	-1,159699505	3,856097276	0,015528746	0,399896546	LADA vs T1D Time-adjusted
hsa-miR-500a-3p_AATGCACCTGGGCAAGGATTCT1	1,474406259	2,908982628	0,015926751	0,399896546	LADA vs T1D Time-adjusted
hsa-miR-361-3p_TCCCCCAGGTGTGATTCTGATT1	1,108429859	3,75062111	0,016177863	0,399896546	LADA vs T1D Time-adjusted
hsa-miR-140-3p_ACCACAGGGTAGAACCACGGA1	-0,748543614	6,607249039	0,017939604	0,422744591	LADA vs T1D Time-adjusted
hsa-miR-150-3p_CTGGTACAGGCCTGGGGGACA1	1,306403195	4,065180007	0,018914308	0,422744591	LADA vs T1D Time-adjusted
hsa-miR-335-3p_GTTTTTCATTATTGCTCCTGACC1	-1,419726813	2,856849935	0,019211779	0,422744591	LADA vs T1D Time-adjusted
hsa-miR-99b-5p_CACCCGTAGAACCGACCTTG1	0,584975799	6,823079979	0,019239956	0,422744591	LADA vs T1D Time-adjusted
hsa-let-7e-5p_TGAGGTAGGAGGTTGTATAGTT1	0,498242686	7,821946446	0,020913741	0,422952258	LADA vs T1D Time-adjusted
hsa-miR-139-5p_TCTACAGTGCACGTGTCTCCAGT1	0,602999166	6,693764684	0,020958681	0,422952258	LADA vs T1D Time-adjusted
hsa-miR-92a-3p_TATTGCACTTGTCCCGG1	1,110364041	4,506722639	0,021070572	0,422952258	LADA vs T1D Time-adjusted
hsa-miR-421_ATCAACAGACATTAATTGGGCG1	1,231864357	2,15305225	0,021567028	0,422952258	LADA vs T1D Time-adjusted
hsa-miR-29a-3p_TAGCACCATCTGAAATCGGTTAT1	1,303873405	2,458427946	0,021922936	0,422952258	LADA vs T1D Time-adjusted
hsa-miR-181b-5p_AACATTCATTGCTGTCGGTG1	1,304447329	2,055578696	0,022990783	0,431787777	LADA vs T1D Time-adjusted
hsa-miR-320a-3p_AAAGCTGGGTTGAGAGGGCGAA1	-1,082852465	4,245030119	0,023552012	0,431787777	LADA vs T1D Time-adjusted
hsa-miR-10a-5p_TACCCTGTAGATCCGAATTTGT1	0,27276332	10,48518	0,024018536	0,431787777	LADA vs T1D Time-adjusted
hsa-miR-10b-5p_ACCCTGTAGAACCGAATTTGT1	0,532359396	6,802181051	0,025749169	0,452613168	LADA vs T1D Time-adjusted
hsa-miR-23a-3p_TCACATTGCCAGGGATTTCCA1	0,930427196	5,160556193	0,026603918	0,457471727	LADA vs T1D Time-adjusted
hsa-miR-487b-3p_AATCGTACAGGGTCATCCACTT1	-1,258547264	3,93004941	0,027437285	0,461763666	LADA vs T1D Time-adjusted
hsa-miR-25-3p_CATTGCACTTGTCTCGGT1	1,047757788	3,82970246	0,028261485	0,463720801	LADA vs T1D Time-adjusted
hsa-miR-140-3p_TACCACAGGGTAGAACCACGG1	0,734710289	5,398078089	0,030004218	0,463720801	LADA vs T1D Time-adjusted
hsa-miR-24-3p_GGCTCAGTTCAGCAGGAACA1	-1,233646643	2,68639799	0,030351919	0,463720801	LADA vs T1D Time-adjusted
hsa-miR-423-5p_GAGGGGCAGAGAGCGAGACTT1	0,832162718	5,546353182	0,030728615	0,463720801	LADA vs T1D Time-adjusted
hsa-miR-30d-5p_TGTAAACATCCCCGACTGGA1	0,372829967	9,147110661	0,030849344	0,463720801	LADA vs T1D Time-adjusted
hsa-miR-183-5p_TATGGCACTGGTAGAATTCAC1	1,164568987	2,19049992	0,031880384	0,463720801	LADA vs T1D Time-adjusted
hsa-miR-335-5p_TCAAGAGCAATAACGAAAAATGT1	0,733636685	6,087791959	0,031916505	0,463720801	LADA vs T1D Time-adjusted
hsa-miR-421_ATCAACAGACATTAATTGGGCGC1	-1,168673691	2,015098416	0,032709676	0,463720801	LADA vs T1D Time-adjusted
hsa-miR-92b-3p_TATTGCACTCGTCCCGGCCTC1	-1,079147267	3,950927699	0,033157179	0,463720801	LADA vs T1D Time-adjusted
hsa-miR-139-5p_TCTACAGTGCACGTGTCTCCAG1	0,874269363	4,962736668	0,033416037	0,463720801	LADA vs T1D Time-adjusted
hsa-miR-27a-3p_TCACAGTGGCTAAGTTCCG1	1,177058875	2,793419478	0,035945842	0,477964223	LADA vs T1D Time-adjusted
hsa-miR-6803-3p_TCCCTCGCCTTCTCACCCTCAGT1	1,315976637	2,013232161	0,036053746	0,477964223	LADA vs T1D Time-adjusted
hsa-miR-142-5p_CCCATAAAGTAGAAAGCAC1	0,787036027	5,073422111	0,036296526	0,477964223	LADA vs T1D Time-adjusted
hsa-miR-423-3p_AGCTCGGTCTGAGGCCCCTCAG1	-0,290695532	8,394585646	0,036859441	0,477964223	LADA vs T1D Time-adjusted
hsa-miR-15b-3p_CGAATCATTATTTGCTGCTCT1	-0,977096266	4,370495899	0,038151263	0,479543974	LADA vs T1D Time-adjusted
hsa-miR-10b-5p_TACCCTGTAGAACCGAATTT1	0,834798061	5,284240397	0,038193768	0,479543974	LADA vs T1D Time-adjusted
hsa-miR-145-5p_GTCCAGTTTTCCCAGGAATCCC1	1,196227305	3,263854619	0,039230725	0,484867237	LADA vs T1D Time-adjusted
hsa-let-7b-5p_TGAGGTAGTAGGTTGTGTG1	-0,520615086	6,901751547	0,041125852	0,500469978	LADA vs T1D Time-adjusted
hsa-miR-10b-5p_TACCCTGTAGAACCGAATTTG1	0,26626335	9,829860922	0,044041518	0,522065685	LADA vs T1D Time-adjusted
hsa-miR-3615_TCTCTCGGCTCCTCGCGGCTCGC1	1,083217918	3,765572795	0,045087414	0,522065685	LADA vs T1D Time-adjusted
hsa-miR-126-5p_ATTATTACTTTTGGTACGCGCT1	1,147221825	2,229095104	0,045440321	0,522065685	LADA vs T1D Time-adjusted
hsa-miR-145-5p_GTCCAGTTTTCCCAGGAATCC1	1,148962462	2,346106601	0,045540496	0,522065685	LADA vs T1D Time-adjusted
hsa-miR-1249-3p_ACGCCCTTCCCCCCCTTCTTCA1	1,257222586	2,093528347	0,046651686	0,522736168	LADA vs T1D Time-adjusted
hsa-miR-142-5p_CATAAAGTAGAAAGCACT1	0,620625745	5,448401815	0,047392556	0,522736168	LADA vs T1D Time-adjusted
hsa-miR-191-5p_CAACGGAATCCCAAAAGCAG1	0,249035568	10,17364723	0,047581548	0,522736168	LADA vs T1D Time-adjusted
hsa-miR-21-5p_AGCTTATCAGACTGATGTTGA1	0,444292645	6,713348932	0,049558227	0,536993943	LADA vs T1D Time-adjusted
hsa-miR-10b-5p_TACCCTGTAGAACCGAATTTGTGT1	1,106283519	2,423805448	0,050616202	0,541046159	LADA vs T1D Time-adjusted
hsa-miR-221-3p_AGCTACATTGTCTGCTGGGTTTCA1	1,149090966	2,292055081	0,051442401	0,542545851	LADA vs T1D Time-adjusted
hsa-miR-185-5p_TGGAGAGAAAGGCAGTTCCTGA1	-0,276560556	8,779876669	0,053317353	0,554921401	LADA vs T1D Time-adjusted
hsa-miR-320a-3p_AAAAGCTGGGTTGAGAGGGCGAA1	-0,238244904	9,820743233	0,054453333	0,559384243	LADA vs T1D Time-adjusted
hsa-miR-101-3p_GTACAGTACTGTGATAACTG1	0,752089143	5,567307108	0,056817816	0,576190932	LADA vs T1D Time-adjusted
hsa-miR-30d-5p_GTAAACATCCCCGACTGGAA1	0,809553935	4,880801026	0,058767768	0,588421579	LADA vs T1D Time-adjusted
hsa-miR-106b-5p_TAAAGTGCTGACAGTGCAGAT1	1,020541649	2,555392208	0,059784527	0,591119512	LADA vs T1D Time-adjusted
hsa-miR-10b-5p_TACCCTGTAGAACCGAATT1	-0,926718673	4,497780085	0,061386008	0,599393975	LADA vs T1D Time-adjusted
hsa-miR-10b-5p_ACCCTGTAGAACCGAATTTGTG1	0,549060391	6,035000543	0,062490043	0,599393975	LADA vs T1D Time-adjusted
hsa-let-7b-5p_TGAGGTAGTAGGTTGTGT1	0,724047562	5,334260563	0,06289469	0,599393975	LADA vs T1D Time-adjusted
hsa-miR-16-5p_TAGCAGCACGTAAATATTG1	-0,623704819	5,552847943	0,067911813	0,6308918	LADA vs T1D Time-adjusted
hsa-miR-328-3p_CTGGCCCTCTCTGCCCTTCCGT1	-0,439067204	7,133297312	0,068730331	0,6308918	LADA vs T1D Time-adjusted
hsa-miR-25-3p_ATTGCACTTGTCTCGGTCT1	0,938894971	3,534900996	0,069121773	0,6308918	LADA vs T1D Time-adjusted
hsa-miR-15b-5p_TAGCAGCACATCATGGTTTACA1	0,557003049	6,304858351	0,070172946	0,6308918	LADA vs T1D Time-adjusted

hsa-miR-30a-5p_TGTAAACATCCTCGACTGGAAG1	0,34200085	8,371847242	0,070578929	0,6308918	LADA vs T1D Time-adjusted
hsa-miR-15b-5p_TAGCAGCACATCATGGTT1	-0,522721266	7,409647991	0,071399033	0,6308918	LADA vs T1D Time-adjusted
hsa-miR-375-3p_TTGTTCGTTCGGCTCGCGTGA1	-0,782320127	5,794174671	0,071782885	0,6308918	LADA vs T1D Time-adjusted
hsa-miR-363-3p_AATTGCACGGTATCCATCTGTA1	0,821982499	4,05384132	0,073219709	0,633921715	LADA vs T1D Time-adjusted
hsa-miR-145-5p_GTCCAGTTTTCCCAGGAATCCCT1	1,070307275	1,974319363	0,073730465	0,633921715	LADA vs T1D Time-adjusted
hsa-miR-29c-3p_TAGCACCATTTGAAATCGG1	0,989373771	3,275982402	0,075333346	0,63964283	LADA vs T1D Time-adjusted
hsa-miR-501-3p_ATGCACCCGGGCAAGGATTCT1	1,00221184	2,108861011	0,076176114	0,63964283	LADA vs T1D Time-adjusted
hsa-miR-345-5p_GCTGACTCCTAGTCCAGGGCT1	-0,783578389	5,007179685	0,076821832	0,63964283	LADA vs T1D Time-adjusted
hsa-miR-23a-5p_GGGGTTCCTGGGGATGGGATTT1	0,94835888	3,892718076	0,078313845	0,641175353	LADA vs T1D Time-adjusted
hsa-miR-584-5p_TTATGGTTTGCCTGGGACT1	0,790687215	4,566540106	0,079477938	0,641175353	LADA vs T1D Time-adjusted
hsa-let-7c-5p_TGAGGTAGTAGGTTGTATGGTT1	0,32800293	7,624637986	0,079508963	0,641175353	LADA vs T1D Time-adjusted
hsa-miR-142-5p_CATAAAGTAGAAAGCACTA1	0,957887946	2,092424464	0,081163563	0,641175353	LADA vs T1D Time-adjusted
hsa-miR-423-5p_CTGAGGGGCAGAGAGCGAGACTTT1	0,906348362	4,393029535	0,081437316	0,641175353	LADA vs T1D Time-adjusted
hsa-miR-182-5p_TTTGGCAATGGTAGAACTCAC1	1,000601096	3,088555901	0,082936129	0,641175353	LADA vs T1D Time-adjusted
hsa-miR-191-5p_CAACGGAATCCCAAAAGCAGCTGT1	0,746820701	5,75430435	0,083069968	0,641175353	LADA vs T1D Time-adjusted
hsa-miR-126-3p_TCGTACCGTGAGTAATAATGCG1	0,229703883	11,26241255	0,083490596	0,641175353	LADA vs T1D Time-adjusted
hsa-let-7i-5p_GAGGTAGTAGTTTGTGCTG1	1,02762861	2,219476679	0,084760268	0,641330561	LADA vs T1D Time-adjusted
hsa-miR-30c-5p_TGTAAACATCCTACACTCTCAGC1	0,42151233	6,576373518	0,085132375	0,641330561	LADA vs T1D Time-adjusted
hsa-miR-320b_AAAAGCTGGGTTGAGAGGGC1	-1,514755223	5,096748857	0,086537054	0,645492774	LADA vs T1D Time-adjusted
hsa-miR-185-5p_TGGAGAGAAAGGCAGTTCCT1	1,021279057	2,073411525	0,088596629	0,645492774	LADA vs T1D Time-adjusted
hsa-miR-93-5p_AAAGTGCTGTTCGTGCAGGTAG1	0,867249895	3,678777222	0,090241446	0,645492774	LADA vs T1D Time-adjusted
hsa-let-7f-5p_GAGGTAGTAGATTGTATAG1	0,931619652	3,124116914	0,090468455	0,645492774	LADA vs T1D Time-adjusted
hsa-miR-10a-5p_ACCCTGTAGATCCGAATTTGT1	0,341204971	7,710604015	0,091741741	0,645492774	LADA vs T1D Time-adjusted
hsa-miR-150-3p_CTGGTACAGGCCTGGGGGAC1	0,968803231	2,452241426	0,092944971	0,645492774	LADA vs T1D Time-adjusted
hsa-miR-28-3p_CACTAGATTGTGAGCTCCTGG1	0,514251397	5,951344612	0,093672115	0,645492774	LADA vs T1D Time-adjusted
hsa-miR-10a-5p_TACCCTGTAGATCCGAATTTG1	0,269341627	8,877087473	0,094277764	0,645492774	LADA vs T1D Time-adjusted
hsa-miR-100-5p_AACCCGTAGATCCGAACTTG1	0,530229845	5,980148473	0,094973783	0,645492774	LADA vs T1D Time-adjusted
hsa-miR-150-5p_GTCTCCCAACCCTTGTACCAGT1	0,92406027	2,385284805	0,095378679	0,645492774	LADA vs T1D Time-adjusted
hsa-miR-338-5p_AACAATATCCTGGTGCTGAGT1	0,603493819	5,921373435	0,095429934	0,645492774	LADA vs T1D Time-adjusted
hsa-miR-10a-5p_ACCCTGTAGATCCGAATTTG1	0,582328973	5,676118316	0,095645219	0,645492774	LADA vs T1D Time-adjusted
hsa-let-7c-5p_TGAGGTAGTAGGTTGTATGG1	-0,621166316	3,127922361	0,096293486	0,645492774	LADA vs T1D Time-adjusted
hsa-miR-24-3p_CTGGCTCAGTTCAGCAGGAACAG1	-0,942741622	2,043456364	0,099534833	0,654639911	LADA vs T1D Time-adjusted
hsa-miR-24-3p_TGGCTCAGTTCAGCAGGAAC1	0,184689826	10,09466061	0,099621838	0,654639911	LADA vs T1D Time-adjusted
hsa-miR-181a-5p_AACATTCAACGCTGTCGGTGAGT1	0,331888363	8,108797195	0,100140871	0,654639911	LADA vs T1D Time-adjusted
hsa-miR-92a-3p_ACTTGTCCCGGCCTGT1	0,957933091	2,211098977	0,101305767	0,656826734	LADA vs T1D Time-adjusted
hsa-miR-652-3p_AATGGCGCCACTAGGGTTGTGC1	-0,881155965	3,398629288	0,1023904	0,65846184	LADA vs T1D Time-adjusted
hsa-miR-505-3p_CGTCAACACTTGCTGGTTTCCTCT1	0,967473406	3,132189778	0,104647522	0,664569288	LADA vs T1D Time-adjusted
hsa-miR-342-3p_CTCACACAGAAATCGCACCCG1	0,989382518	2,546589991	0,10585453	0,664569288	LADA vs T1D Time-adjusted
hsa-miR-22-3p_AGCTGCCAGTTGAAGAACTGT1	-0,60039229	5,657855582	0,105860595	0,664569288	LADA vs T1D Time-adjusted
hsa-miR-29a-3p_CTAGCACCATCTGAAATCGG1	0,842757801	3,1053029	0,106730926	0,664757183	LADA vs T1D Time-adjusted
hsa-miR-4433b-5p_TGTCCCACCCCCACTCCTGTT1	-0,786381554	6,506356169	0,108722427	0,671870624	LADA vs T1D Time-adjusted
hsa-miR-125a-5p_CCCTGAGACCCTTTAACCTGT1	0,687861821	5,393774828	0,111150532	0,680006456	LADA vs T1D Time-adjusted
hsa-miR-361-5p_TTATCAGAATCTCCAGGGGTAC1	0,38884086	6,68645525	0,11175833	0,680006456	LADA vs T1D Time-adjusted
hsa-miR-486-5p_TCCTGTACTGAGCTGCCCCGAGC1	-0,383818302	6,153079419	0,113821913	0,687275827	LADA vs T1D Time-adjusted
hsa-miR-30e-3p_CTTTCAGTCGGATGTTTACAGC1	0,923821696	3,112145052	0,116730751	0,690932859	LADA vs T1D Time-adjusted
hsa-miR-27a-3p_TTCACAGTGGCTAAGTTCCG1	0,206025932	9,746526676	0,117052751	0,690932859	LADA vs T1D Time-adjusted
hsa-let-7i-5p_GAGGTAGTAGTTTGTGCTGTT1	0,909299752	3,203293849	0,118472798	0,690932859	LADA vs T1D Time-adjusted
hsa-let-7a-5p_GAGGTAGTAGGTTGTATAGTT1	0,354388464	7,12979206	0,118675577	0,690932859	LADA vs T1D Time-adjusted
hsa-miR-29a-3p_TAGCACCATCTGAAATCGGTT1	0,304435265	8,673627549	0,119482877	0,690932859	LADA vs T1D Time-adjusted
hsa-miR-126-3p_CTCGTACCGTGAGTAATAATGCG1	0,813542508	4,641889164	0,119668523	0,690932859	LADA vs T1D Time-adjusted
hsa-miR-2110_TTGGGGAAACGGCCGCTGAGT1	0,503581154	6,041635722	0,122662789	0,700359548	LADA vs T1D Time-adjusted
hsa-miR-142-5p_CCCATAAAGTAGAAAGCA1	0,759864852	2,402819725	0,123072032	0,700359548	LADA vs T1D Time-adjusted
hsa-let-7b-5p_GAGGTAGTAGGTTGTGTGGTT1	-0,455029413	6,256027764	0,129662097	0,724126241	LADA vs T1D Time-adjusted
hsa-miR-486-3p_CGGGGCAGCTCAGTACAGGA1	-0,492148474	6,565196714	0,132103421	0,724126241	LADA vs T1D Time-adjusted
hsa-miR-363-3p_AATTGCACGGTATCCATCT1	-0,757926273	4,448241713	0,132700149	0,724126241	LADA vs T1D Time-adjusted
hsa-miR-215-5p_ATGACCTATGAATTGACAGA1	-0,784711343	5,04356396	0,132883934	0,724126241	LADA vs T1D Time-adjusted
hsa-miR-16-5p_TAGCAGCACGTAAATATTGGC1	-0,202239345	9,966980138	0,132934233	0,724126241	LADA vs T1D Time-adjusted
hsa-miR-26a-5p_TTCAAGTAATCCAGGATAGGC1	0,259610629	7,521939438	0,134675144	0,724126241	LADA vs T1D Time-adjusted
hsa-miR-1306-5p_CCACCTCCCCTGCAAACGT1	-0,984330063	2,609394089	0,134937023	0,724126241	LADA vs T1D Time-adjusted
hsa-miR-423-5p_GAGGGGCAGAGAGCGAGACTTTT1	0,785450175	3,8847694	0,135754617	0,724126241	LADA vs T1D Time-adjusted
hsa-miR-409-3p_GAATGTTGCTCGGTGAACCCCT1	-0,59584523	7,83201482	0,135886897	0,724126241	LADA vs T1D Time-adjusted

hsa-miR-140-5p_CAGTGGTTTTACCCTATGGTAG1	0,693048263	3,907116242	0,13916256	0,724126241	LADA vs T1D Time-adjusted
hsa-miR-1306-5p_CCACCTCCCCTGCAAACGTC1	-0,778409255	3,895275761	0,141208564	0,724126241	LADA vs T1D Time-adjusted
hsa-miR-629-5p_TGGGTTTACGTTGGGAGAA1	0,896289081	2,664864204	0,142012324	0,724126241	LADA vs T1D Time-adjusted
hsa-let-7d-3p_CTATACGACCTGCTGCCTTTCT1	0,163148777	11,72675264	0,142727044	0,724126241	LADA vs T1D Time-adjusted
hsa-miR-425-5p_AATGACACGATCACTCCCGTTGAGT1	0,359880088	8,285934125	0,143520636	0,724126241	LADA vs T1D Time-adjusted
hsa-miR-181a-5p_AACATTCAACGCTGTCGGTGAG1	0,275075329	7,417004171	0,144547044	0,724126241	LADA vs T1D Time-adjusted
hsa-miR-374b-5p_ATATAATACAACCTGCTAAGTG1	0,824965585	2,591933676	0,144783872	0,724126241	LADA vs T1D Time-adjusted
hsa-miR-340-3p_TCCGTCTCAGTTACTTTATAGCC1	0,854844247	3,011838282	0,144965963	0,724126241	LADA vs T1D Time-adjusted
hsa-miR-500a-3p_ATGCACCTGGGCAAGGATTCT1	0,686791963	4,574890345	0,145002664	0,724126241	LADA vs T1D Time-adjusted
hsa-miR-30d-5p_TGTAAACATCCCCGACTGG1	0,222536748	8,103513785	0,145857193	0,724126241	LADA vs T1D Time-adjusted
hsa-miR-126-3p_TCGTACCGTGAGTAATAATG1	0,430841237	5,99055736	0,146166402	0,724126241	LADA vs T1D Time-adjusted
hsa-miR-652-3p_AATGGCGCCACTAGGGTTGTG1	-0,427006495	6,436258449	0,146634135	0,724126241	LADA vs T1D Time-adjusted
hsa-miR-148b-3p_TCAGTGCATCACAGAACTTTG1	-0,607993511	4,984825627	0,147388527	0,724126241	LADA vs T1D Time-adjusted
hsa-miR-191-5p_CAACGGAATCCCAAAAGCA1	0,447370269	6,200760212	0,153860349	0,74216148	LADA vs T1D Time-adjusted
hsa-miR-375-3p_TTTGTTCGTTCGGCTCGCGT1	0,825635464	4,026890223	0,153874177	0,74216148	LADA vs T1D Time-adjusted
hsa-miR-125a-5p_TCCCTGAGACCCTTTAACCTGTGA1	0,300601314	8,184259766	0,155303299	0,74216148	LADA vs T1D Time-adjusted
hsa-miR-1908-5p_CGGCGGGGACGGCGATTGGTC1	0,832765645	2,7409573	0,155546364	0,74216148	LADA vs T1D Time-adjusted
hsa-miR-150-5p_CTCCCAACCCTTGTACCAGTG1	0,658200294	4,444846731	0,155750703	0,74216148	LADA vs T1D Time-adjusted
hsa-let-7a-5p_TGAGGTAGTAGGTTGTATAGT1	0,134019561	11,89008946	0,156715029	0,742284956	LADA vs T1D Time-adjusted
hsa-miR-363-3p_ATTGCACGGTATCCATCTGT1	0,671887169	4,449987277	0,158032228	0,743749566	LADA vs T1D Time-adjusted
hsa-miR-146a-5p_TGAGAACTGAATTCCATGGGTT1	0,15870508	12,18952455	0,158904774	0,743749566	LADA vs T1D Time-adjusted
hsa-miR-320a-3p_AAAGCTGGGTTGAGAGGGCGA1	-0,631591726	5,194526481	0,161496588	0,749787505	LADA vs T1D Time-adjusted
hsa-miR-486-5p_TCCTGTACTGAGCTGCCCC1	-0,344499644	6,726361248	0,162090598	0,749787505	LADA vs T1D Time-adjusted
hsa-miR-144-5p_GATATCATCATATACTGTAAGTT1	0,85246598	2,274281107	0,163693799	0,752801132	LADA vs T1D Time-adjusted
hsa-let-7a-5p_GTGAGGTAGTAGGTTGTATAGTT1	0,863931172	3,003012908	0,164835294	0,753668887	LADA vs T1D Time-adjusted
hsa-miR-499a-5p_TTAAGACTTGCAGTGATGTTT1	0,821621058	2,030187923	0,166413847	0,756513524	LADA vs T1D Time-adjusted
hsa-let-7g-5p_TGAGGTAGTAGTTTGTACAG1	-0,206338884	9,388462879	0,168260907	0,757996059	LADA vs T1D Time-adjusted
hsa-miR-29c-3p_TAGCACCATTTGAAATCGGTT1	0,639711999	3,979110528	0,168656519	0,757996059	LADA vs T1D Time-adjusted
hsa-miR-423-5p_AGGGGCAGAGAGCGAGACTTTT1	0,636189817	5,009061922	0,169959516	0,759536592	LADA vs T1D Time-adjusted
hsa-miR-4685-3p_TCTCCCTTCCTGCCCTGGCT1	0,670969268	4,82214565	0,172535948	0,763823479	LADA vs T1D Time-adjusted
hsa-miR-342-3p_TCACACAGAAATCGCACCCGT1	0,777401078	2,497948576	0,172850067	0,763823479	LADA vs T1D Time-adjusted
hsa-miR-18a-3p_ACTGCCCTAAGTGCTCCTTCT1	0,787758194	3,355078821	0,175237918	0,763900471	LADA vs T1D Time-adjusted
hsa-miR-361-3p_TCCCCCAGGTGTGATTCTGATTT1	-0,631525577	4,004503719	0,175525374	0,763900471	LADA vs T1D Time-adjusted
hsa-miR-342-3p_TCACACAGAAATCGCACCCGTCA1	0,708128758	3,93860325	0,17576471	0,763900471	LADA vs T1D Time-adjusted
hsa-miR-126-3p_TCGTACCGTGAGTAATAATGC1	0,339890027	6,39389942	0,178152715	0,770048074	LADA vs T1D Time-adjusted
hsa-miR-221-3p_AGCTACATTGTCTGCTGGGTTTC1	0,464564017	6,662543722	0,182006883	0,782431762	LADA vs T1D Time-adjusted
hsa-miR-106b-3p_CCGCACTGTGGGTACTTGCT1	-0,467569807	6,292504346	0,187322428	0,789124137	LADA vs T1D Time-adjusted
hsa-miR-23b-3p_ATCACATTGCCAGGGATTACCA1	0,373226113	6,519069588	0,190258985	0,789124137	LADA vs T1D Time-adjusted
hsa-miR-22-3p_AAGCTGCCAGTTGAAGAACTGTT1	-0,737548318	2,813129247	0,190429034	0,789124137	LADA vs T1D Time-adjusted
hsa-miR-30e-5p_TGTAAACATCCTTGACTGG1	0,51418475	5,259392082	0,192773888	0,789124137	LADA vs T1D Time-adjusted
hsa-miR-23a-3p_ATCACATTGCCAGGGATTTCCAA1	0,435460473	6,524370613	0,194229594	0,789124137	LADA vs T1D Time-adjusted
hsa-let-7b-3p_CTATACAACCTACTGCCTTCC1	0,405826078	5,967940681	0,194324003	0,789124137	LADA vs T1D Time-adjusted
hsa-miR-126-3p_CGTACCGTGAGTAATAATGCG1	0,187190676	9,728392522	0,194484015	0,789124137	LADA vs T1D Time-adjusted
hsa-miR-142-5p_CCCATAAAGTAGAAAGCACTA1	0,503069018	5,238891455	0,196514057	0,789124137	LADA vs T1D Time-adjusted
hsa-miR-4433b-5p_TGTCCCACCCCCACTCCTG1	-0,810863467	2,575504162	0,197119931	0,789124137	LADA vs T1D Time-adjusted
hsa-miR-103a-3p_AGCAGCATTGTACAGGGCTATG1	0,723075416	4,200669238	0,197334034	0,789124137	LADA vs T1D Time-adjusted
hsa-miR-451a_AAACCGTTACCATTACTGAGTTTAGT1	0,546459433	4,347393724	0,197890716	0,789124137	LADA vs T1D Time-adjusted
hsa-miR-320a-3p_AAAAGCTGGGTTGAGAGGGCGA1	-0,145193429	10,81566962	0,198380798	0,789124137	LADA vs T1D Time-adjusted
hsa-miR-30b-5p_TGTAAACATCCTACACTCAGCT1	0,350486479	6,702165067	0,200102038	0,789124137	LADA vs T1D Time-adjusted
hsa-miR-100-5p_AACCCGTAGATCCGAACTTGTG1	0,750081095	3,697610074	0,202279585	0,789124137	LADA vs T1D Time-adjusted
hsa-miR-92a-3p_GTATTGCACTTGTCCCGGCCTG1	0,723668062	3,050162886	0,204675984	0,789124137	LADA vs T1D Time-adjusted
hsa-miR-191-5p_AACGGAATCCCAAAAGCAGC1	0,707974882	2,971417206	0,205270275	0,789124137	LADA vs T1D Time-adjusted
hsa-miR-505-3p_CGTCAACACTTGCTGGTTTCCT1	0,672003279	3,930461517	0,207118566	0,789124137	LADA vs T1D Time-adjusted
hsa-miR-99b-5p_CACCCGTAGAACCGACCTT1	0,733055975	2,15225541	0,208427108	0,789124137	LADA vs T1D Time-adjusted
hsa-miR-30e-5p_TGTAAACATCCTTGACTGGAAGCT1	-0,099113585	11,68636005	0,209267237	0,789124137	LADA vs T1D Time-adjusted
hsa-miR-495-3p_AAACAAACATGGTGCACTTCTT1	0,777869477	2,371725028	0,209331121	0,789124137	LADA vs T1D Time-adjusted
hsa-miR-323a-3p_CACATTACACGGTCGACCTCT1	0,769461729	3,386474425	0,210198729	0,789124137	LADA vs T1D Time-adjusted
hsa-miR-30a-5p_TGTAAACATCCTCGACTGGA1	0,682410215	3,318329214	0,210975431	0,789124137	LADA vs T1D Time-adjusted
hsa-miR-320a-3p_AAAAGCTGGGTTGAGAGGGCG1	-0,158668315	10,10122965	0,211395888	0,789124137	LADA vs T1D Time-adjusted
hsa-miR-27b-3p_TTCACAGTGGCTAAGTTCTG1	0,235957795	8,329497842	0,211767223	0,789124137	LADA vs T1D Time-adjusted
hsa-miR-451a_GAAACCGTTACCATTACTGAGT1	0,352173941	6,902919522	0,211853343	0,789124137	LADA vs T1D Time-adjusted

hsa-miR-16-5p_TAGCAGCACGTAAATATTGGCG1	-0,157512005	14,02532692	0,212053179	0,789124137	LADA vs T1D Time-adjusted
hsa-miR-16-5p_AGCAGCACGTAAATATTGGC1	-0,679525324	3,090604583	0,212090863	0,789124137	LADA vs T1D Time-adjusted
hsa-miR-101-3p_TACAGTACTGTGATAACTGAAG1	-0,333614294	6,912528742	0,212467501	0,789124137	LADA vs T1D Time-adjusted
hsa-miR-7-5p_TGGAAGACTAGTGATTTTGT1	0,655132223	4,179429072	0,212865468	0,789124137	LADA vs T1D Time-adjusted
hsa-miR-99a-5p_AACCCGTAGATCCGATCTTGTG1	0,425273543	5,465860433	0,213492497	0,789124137	LADA vs T1D Time-adjusted
hsa-miR-423-5p_GAGGGGCAGAGAGCGAGACTTT1	-0,168655582	8,417582901	0,215759754	0,793795189	LADA vs T1D Time-adjusted
hsa-let-7b-3p_CTATACAACCTACTGCCTTC1	0,699857679	3,17192614	0,217224659	0,795484745	LADA vs T1D Time-adjusted
hsa-miR-532-5p_CATGCCTTGAGTGTAGGACCG1	0,659210009	2,328095035	0,221644426	0,807929682	LADA vs T1D Time-adjusted
hsa-miR-101-3p_GTACAGTACTGTGATAACTGAA1	0,683831839	3,734790611	0,224135814	0,813263436	LADA vs T1D Time-adjusted
hsa-miR-664a-5p_ACTGGCTAGGGAAAATGATTGG1	0,653999785	3,605476953	0,226858707	0,817523419	LADA vs T1D Time-adjusted
hsa-miR-142-5p_CCATAAAGTAGAAAGCACT1	0,470660966	4,725847494	0,227376931	0,817523419	LADA vs T1D Time-adjusted
hsa-miR-182-5p_TTTGGCAATGGTAGAACT1	-0,701530463	3,433367687	0,235004902	0,819906036	LADA vs T1D Time-adjusted
hsa-miR-150-5p_CTCCCAACCCTTGTACCAGT1	0,717508923	3,109098502	0,236392886	0,819906036	LADA vs T1D Time-adjusted
hsa-miR-409-3p_GAATGTTGCTCGGTGAACCCCTTT1	0,701129911	3,983254098	0,237391362	0,819906036	LADA vs T1D Time-adjusted
hsa-miR-125a-5p_TCCCTGAGACCCTTTAACC1	0,65562198	2,3001783	0,237809818	0,819906036	LADA vs T1D Time-adjusted
hsa-miR-146a-5p_TGAGAACTGAATTCCATGGG1	0,640741681	2,326140979	0,238143283	0,819906036	LADA vs T1D Time-adjusted
hsa-miR-92a-3p_ATTGCACTTGTCCCGGCCTGTT1	0,438577646	5,582760418	0,240061282	0,819906036	LADA vs T1D Time-adjusted
hsa-miR-144-5p_GGATATCATCATATACTGTAA1	-0,6693842	4,029614813	0,240448193	0,819906036	LADA vs T1D Time-adjusted
hsa-let-7a-5p_TGAGGTAGTAGGTTGTATAGTT1	0,153473532	13,3879175	0,240500353	0,819906036	LADA vs T1D Time-adjusted
hsa-miR-320a-3p_GAAAAGCTGGGTTGAGAGGGCG1	-0,365217444	6,257483702	0,24066098	0,819906036	LADA vs T1D Time-adjusted
hsa-miR-629-5p_TGGGTTTACGTTGGGAGAACT1	-0,272184556	7,102407997	0,242138328	0,819906036	LADA vs T1D Time-adjusted
hsa-miR-23a-3p_ATCACATTGCCAGGGATTT1	0,206815828	7,89161665	0,242269135	0,819906036	LADA vs T1D Time-adjusted
hsa-let-7f-5p_TGAGGTAGTAGATTGTAT1	0,692186687	3,218494088	0,242416441	0,819906036	LADA vs T1D Time-adjusted
hsa-miR-128-3p_TCACAGTGAACCGGTCTCTT1	0,28858039	6,752700404	0,243653255	0,819906036	LADA vs T1D Time-adjusted
hsa-miR-30c-5p_GTAAACATCCTACACTCTCAGCT1	0,70853998	3,000834251	0,246614562	0,819906036	LADA vs T1D Time-adjusted
hsa-miR-28-5p_AAGGAGCTCACAGTCTATTGAG1	-0,681776032	3,305855814	0,247435459	0,819906036	LADA vs T1D Time-adjusted
hsa-miR-320a-3p_AAAGCTGGGTTGAGAGGGCG1	-0,553007135	4,800125051	0,248569147	0,819906036	LADA vs T1D Time-adjusted
hsa-let-7g-5p_TGAGGTAGTAGTTTGTACAGTT1	0,144635878	11,2336953	0,248584922	0,819906036	LADA vs T1D Time-adjusted
hsa-miR-182-5p_TTTGGCAATGGTAGAACTCACACTG1	0,643605581	3,704867925	0,248966681	0,819906036	LADA vs T1D Time-adjusted
hsa-miR-1-3p_TGGAATGTAAAGAAGTATGTAT1	0,624118334	5,884629396	0,249260563	0,819906036	LADA vs T1D Time-adjusted
hsa-miR-451a_CGTTACCATTACTGAGT1	-0,551829279	4,231569487	0,249878965	0,819906036	LADA vs T1D Time-adjusted
hsa-miR-451a_AAACCGTTACCATTACTGA1	0,238024182	8,906066815	0,250473305	0,819906036	LADA vs T1D Time-adjusted
hsa-let-7g-5p_TGAGGTAGTAGTTTGTACAGTTT1	-0,313825534	6,732316769	0,252390562	0,819906036	LADA vs T1D Time-adjusted
hsa-miR-532-3p_CCTCCCACACCCAAGGCTTG1	0,677001797	2,771296807	0,252697668	0,819906036	LADA vs T1D Time-adjusted
hsa-miR-26b-5p_TTCAAGTAATTCAGGATAGG1	-0,59456648	3,267725483	0,253325419	0,819906036	LADA vs T1D Time-adjusted
hsa-miR-192-5p_TGACCTATGAATTGACAGC1	-0,689604512	3,769796882	0,254890784	0,819906036	LADA vs T1D Time-adjusted
hsa-miR-409-3p_CGAATGTTGCTCGGTGAACCCCT1	-0,600412803	5,552259607	0,254989741	0,819906036	LADA vs T1D Time-adjusted
hsa-miR-3605-3p_CCTCCGTGTTACCTGTCCTCT1	0,533083916	5,156629775	0,258194479	0,82546373	LADA vs T1D Time-adjusted
hsa-miR-10a-5p_TACCCTGTAGATCCGAATTTGTGT1	0,625625315	2,457813121	0,259934461	0,82546373	LADA vs T1D Time-adjusted
hsa-miR-30e-5p_GTAAACATCCTTGACTGGAAGC1	0,662862401	2,516119605	0,262684144	0,82546373	LADA vs T1D Time-adjusted
hsa-miR-423-5p_TGAGGGGCAGAGAGCGAGACT1	-0,145037974	13,36591214	0,264695142	0,82546373	LADA vs T1D Time-adjusted
hsa-let-7b-5p_TGAGGTAGTAGGTTGTGTGGTT1	-0,103907429	12,80806781	0,264838539	0,82546373	LADA vs T1D Time-adjusted
hsa-miR-543_AAACATTCGCGGTGCACTTCTTT1	-0,717091958	3,18614348	0,265899265	0,82546373	LADA vs T1D Time-adjusted
hsa-miR-16-5p_AGCAGCACGTAAATATTGGCG1	-0,203660735	8,159714087	0,268138757	0,82546373	LADA vs T1D Time-adjusted
hsa-miR-155-5p_TTAATGCTAATCGTGATAGGGGT1	0,536625419	4,228033966	0,268154092	0,82546373	LADA vs T1D Time-adjusted
hsa-miR-192-5p_TGACCTATGAATTGACAGCCAGT1	0,560104666	3,442809963	0,27028664	0,82546373	LADA vs T1D Time-adjusted
hsa-miR-3615_TCTCTCGGCTCCTCGCGGCTC1	0,591077444	2,389605161	0,272189585	0,82546373	LADA vs T1D Time-adjusted
hsa-let-7i-5p_TGAGGTAGTAGTTTGTGCTGT1	-0,13397595	11,42837325	0,274042224	0,82546373	LADA vs T1D Time-adjusted
hsa-miR-654-5p_TGGTGGGCCGCAGAACATGTGC1	-0,71348562	3,180891958	0,274105876	0,82546373	LADA vs T1D Time-adjusted
hsa-let-7b-5p_TGAGGTAGTAGGTTGTGTGGT1	-0,120447201	11,86683629	0,274170604	0,82546373	LADA vs T1D Time-adjusted
hsa-miR-629-5p_TGGGTTTACGTTGGGAGAACTT1	-0,54300815	4,102285673	0,27549958	0,82546373	LADA vs T1D Time-adjusted
hsa-miR-19a-3p_TGTGCAAATCTATGCAAAACTGA1	0,609850431	2,482431203	0,275653091	0,82546373	LADA vs T1D Time-adjusted
hsa-miR-16-2-3p_CCAATATTACTGTGCTGCTTT1	0,497329234	4,408566959	0,277123476	0,82546373	LADA vs T1D Time-adjusted
hsa-miR-223-5p_CGTGTATTTGACAAGCTGAGTTGG1	0,669994289	2,254955516	0,280549459	0,82546373	LADA vs T1D Time-adjusted
hsa-miR-16-5p_CTAGCAGCACGTAAATATTGGCG1	0,497087105	4,886130646	0,281085524	0,82546373	LADA vs T1D Time-adjusted
hsa-miR-144-3p_CTACAGTATAGATGATGTAC1	0,58783956	3,365989189	0,281347644	0,82546373	LADA vs T1D Time-adjusted
hsa-miR-22-3p_AAGCTGCCAGTTGAAGAAC1	0,217253971	8,253643329	0,283313882	0,82546373	LADA vs T1D Time-adjusted
hsa-miR-329-3p_AACACACCTGGTTAACCTCTT1	0,623340178	3,530220069	0,284203114	0,82546373	LADA vs T1D Time-adjusted
hsa-miR-25-3p_ATTGCACTTGTCTCGGTCTGA1	0,373248712	5,817358164	0,284309114	0,82546373	LADA vs T1D Time-adjusted
hsa-miR-146a-5p_TGAGAACTGAATTCCATGGGT1	0,151162726	8,701643613	0,284516676	0,82546373	LADA vs T1D Time-adjusted
hsa-miR-191-5p_CAACGGAATCCCAAAAGCAGCT1	0,131622396	11,08359666	0,289504906	0,82546373	LADA vs T1D Time-adjusted

hsa-miR-3615_TCTCTCGGCTCCTCGCGGCTCG1	-0,158734639	8,148580046	0,290447007	0,82546373	LADA vs T1D Time-adjusted
hsa-miR-182-5p_TTTGGCAATGGTAGAACTCA1	-0,271148369	7,904920515	0,290509806	0,82546373	LADA vs T1D Time-adjusted
hsa-let-7d-3p_TATACGACCTGCTGCCTTTC1	0,302876161	6,350176431	0,291243169	0,82546373	LADA vs T1D Time-adjusted
hsa-miR-10a-5p_TACCCTGTAGATCCGAAT1	0,629642564	2,390493785	0,295478749	0,82546373	LADA vs T1D Time-adjusted
hsa-miR-18a-3p_ACTGCCCTAAGTGCTCCTTCTG1	-0,572440527	2,147867886	0,296405839	0,82546373	LADA vs T1D Time-adjusted
hsa-miR-128-3p_TCACAGTGAACCGGTCTCTTT1	-0,14480523	8,165938943	0,298472447	0,82546373	LADA vs T1D Time-adjusted
hsa-miR-3615_TCTCTCGGCTCCTCGCGGCT1	-0,315613272	6,628968416	0,298860504	0,82546373	LADA vs T1D Time-adjusted
hsa-let-7a-5p_TGAGGTAGTAGGTTGTATAGTTT1	0,228451495	9,339383804	0,299801136	0,82546373	LADA vs T1D Time-adjusted
hsa-miR-409-3p_GAATGTTGCTCGGTGAACCCCTT1	-0,604161155	5,003955447	0,300925436	0,82546373	LADA vs T1D Time-adjusted
hsa-miR-320a-3p_AAAAGCTGGGTTGAGAGGGCGAAAA1	0,590921354	3,105258929	0,302340326	0,82546373	LADA vs T1D Time-adjusted
hsa-miR-30a-3p_CTTTCAGTCGGATGTTTGCAG1	0,575561358	2,025868208	0,30541248	0,82546373	LADA vs T1D Time-adjusted
hsa-miR-193a-5p_TGGGTCTTTGCGGGCGAGA1	0,622764193	3,485272507	0,306129471	0,82546373	LADA vs T1D Time-adjusted
hsa-miR-150-5p_TCTCCCAACCCTTGTACCAGTG1	0,169341592	11,04001159	0,306670302	0,82546373	LADA vs T1D Time-adjusted
hsa-miR-122-5p_GGAGTGTGACAATGGTGTTT1	0,36017641	7,754720279	0,307067532	0,82546373	LADA vs T1D Time-adjusted
hsa-miR-363-3p_AATTGCACGGTATCCATCTGT1	0,167046891	9,045234632	0,30716155	0,82546373	LADA vs T1D Time-adjusted
hsa-miR-16-5p_TAGCAGCACGTAAATATTGGCGT1	-0,213559789	7,887777382	0,308011608	0,82546373	LADA vs T1D Time-adjusted
hsa-miR-182-5p_TTTGGCAATGGTAGAACTCACA1	-0,263589436	6,980287172	0,308528948	0,82546373	LADA vs T1D Time-adjusted
hsa-miR-30c-5p_TGTAAACATCCTACACTCTCAGCT1	0,150334219	10,19324434	0,312523407	0,82546373	LADA vs T1D Time-adjusted
hsa-miR-423-5p_TGAGGGGCAGAGAGCGAGACTT1	-0,103612472	12,27072707	0,312934195	0,82546373	LADA vs T1D Time-adjusted
hsa-miR-425-5p_AATGACACGATCACTCCCGTT1	0,217728119	8,013037563	0,313232243	0,82546373	LADA vs T1D Time-adjusted
hsa-miR-328-3p_CTGGCCCTCTCTGCCCTTCCG1	-0,487524858	3,896716502	0,313318074	0,82546373	LADA vs T1D Time-adjusted
hsa-miR-339-5p_TCCCTGTCCTCCAGGAGCTCACG1	0,60079104	3,320095685	0,315221896	0,82546373	LADA vs T1D Time-adjusted
hsa-miR-185-5p_TGGAGAGAAAGGCAGTTCCTG1	0,494337063	4,445039784	0,316334421	0,82546373	LADA vs T1D Time-adjusted
hsa-miR-101-3p_GTACAGTACTGTGATAACT1	-0,496385525	4,834369948	0,316431875	0,82546373	LADA vs T1D Time-adjusted
hsa-let-7a-5p_TGAGGTAGTAGGTTGTATAG1	-0,12443876	12,03070271	0,318843446	0,82546373	LADA vs T1D Time-adjusted
hsa-let-7i-5p_GAGGTAGTAGTTTGTGCTGT1	0,462754322	4,86484784	0,319138485	0,82546373	LADA vs T1D Time-adjusted
hsa-miR-181b-5p_AACATTCATTGCTGTCGGTGG1	0,375405414	5,17184581	0,319635313	0,82546373	LADA vs T1D Time-adjusted
hsa-miR-150-5p_TCTCCCAACCCTTGTACCAG1	0,241899297	9,064957556	0,319721137	0,82546373	LADA vs T1D Time-adjusted
hsa-miR-2110_TTGGGGAAACGGCCGCTGAGTG1	0,576355262	3,022786946	0,320032739	0,82546373	LADA vs T1D Time-adjusted
hsa-miR-423-3p_AGCTCGGTCTGAGGCCCCTCAGT1	-0,126214647	11,77370697	0,320532642	0,82546373	LADA vs T1D Time-adjusted
hsa-miR-15b-5p_TAGCAGCACATCATGGTTTA1	-0,328335299	5,81103732	0,320534724	0,82546373	LADA vs T1D Time-adjusted
hsa-miR-451a_AAACCGTTACCATTACTGAGTTTAG1	0,56594235	2,222514405	0,3207479	0,82546373	LADA vs T1D Time-adjusted
hsa-miR-484_TCAGGCTCAGTCCCCTCCCGATA1	-0,466558953	4,542352365	0,320796227	0,82546373	LADA vs T1D Time-adjusted
hsa-miR-103a-3p_AGCAGCATTGTACAGGGC1	0,682800777	3,250498272	0,320854188	0,82546373	LADA vs T1D Time-adjusted
hsa-miR-27b-3p_TTCACAGTGGCTAAGTTCT1	0,188397191	8,397674944	0,32142539	0,82546373	LADA vs T1D Time-adjusted
hsa-miR-122-5p_TGGAGTGTGACAATGGTGTTTG1	-0,273927018	11,7376523	0,322524153	0,82546373	LADA vs T1D Time-adjusted
hsa-miR-122-5p_GAGTGTGACAATGGTGTTT1	0,604399262	3,048399419	0,322908397	0,82546373	LADA vs T1D Time-adjusted
hsa-miR-26a-5p_TTCAAGTAATCCAGGATAG1	-0,528694511	3,674214058	0,323166208	0,82546373	LADA vs T1D Time-adjusted
hsa-miR-181a-5p_AACATTCAACGCTGTCGGTGAGTT1	0,550860421	2,473533262	0,323753636	0,82546373	LADA vs T1D Time-adjusted
hsa-miR-24-3p_TGGCTCAGTTCAGCAGGAACA1	-0,113773254	10,02516895	0,324161658	0,82546373	LADA vs T1D Time-adjusted
hsa-miR-126-3p_CGTACCGTGAGTAATAATGC1	-0,405671656	4,480402803	0,324674188	0,82546373	LADA vs T1D Time-adjusted
hsa-let-7a-5p_GAGGTAGTAGGTTGTATAG1	0,408796294	5,313092713	0,325593785	0,82546373	LADA vs T1D Time-adjusted
hsa-miR-191-5p_CAACGGAATCCCAAAAGC1	0,552849534	3,145120995	0,327594511	0,827882614	LADA vs T1D Time-adjusted
hsa-miR-15b-5p_TAGCAGCACATCATGGTTT1	-0,190303969	8,509089064	0,329551119	0,830174953	LADA vs T1D Time-adjusted
hsa-miR-375-3p_TTTGTTCGTTCGGCTCGCGTGA1	-0,240917332	10,24220516	0,33364904	0,83394642	LADA vs T1D Time-adjusted
hsa-miR-26b-5p_TTCAAGTAATTCAGGATAGGTT1	0,178115514	9,792607635	0,333936637	0,83394642	LADA vs T1D Time-adjusted
hsa-miR-486-5p_TGTACTGAGCTGCCCCGAG1	-0,410336059	5,107200977	0,33498599	0,83394642	LADA vs T1D Time-adjusted
hsa-miR-424-5p_CAGCAGCAATTCATGTTTTGA1	0,561070282	2,664190598	0,336280784	0,83394642	LADA vs T1D Time-adjusted
hsa-miR-423-5p_TGAGGGGCAGAGAGCGAGACTTTT1	-0,102440663	11,36605873	0,337249374	0,83394642	LADA vs T1D Time-adjusted
hsa-miR-24-3p_TGGCTCAGTTCAGCAGGAACAG1	-0,101648958	12,80068155	0,338354406	0,83394642	LADA vs T1D Time-adjusted
hsa-miR-10b-5p_ACCCTGTAGAACCGAATTTGTGT1	0,504946935	3,358063133	0,338635364	0,83394642	LADA vs T1D Time-adjusted
hsa-miR-142-5p_CCCATAAAGTAGAAAGCACTAC1	0,249380873	6,93738446	0,339798539	0,83394642	LADA vs T1D Time-adjusted
hsa-miR-323b-3p_CCCAATACACGGTCGACCTCT1	-0,471331968	5,843242142	0,342123801	0,83394642	LADA vs T1D Time-adjusted
hsa-miR-19b-3p_TGTGCAAATCCATGCAAAACTGA1	0,425743868	5,03945579	0,342859934	0,83394642	LADA vs T1D Time-adjusted
hsa-miR-99a-5p_AACCCGTAGATCCGATCTT1	-0,569264726	3,162993064	0,344375735	0,83394642	LADA vs T1D Time-adjusted
hsa-miR-24-3p_TGGCTCAGTTCAGCAGGAA1	0,394763646	5,597444609	0,345305359	0,83394642	LADA vs T1D Time-adjusted
hsa-miR-493-5p_TTGTACATGGTAGGCTTTCATT1	-0,562270108	3,42834388	0,345755699	0,83394642	LADA vs T1D Time-adjusted
hsa-miR-101-3p_TACAGTACTGTGATAACTGA1	-0,513113252	4,251229709	0,346830866	0,83394642	LADA vs T1D Time-adjusted
hsa-miR-361-5p_TTATCAGAATCTCCAGGGG1	0,523280601	2,375013248	0,34686267	0,83394642	LADA vs T1D Time-adjusted
hsa-miR-16-2-3p_ACCAATATTACTGTGCTGCTTT1	-0,167362779	7,823388697	0,351191227	0,841794729	LADA vs T1D Time-adjusted
hsa-miR-92a-3p_GTATTGCACTTGTCCCGGCCTGT1	0,180519712	7,697377602	0,353986967	0,843764644	LADA vs T1D Time-adjusted

hsa-miR-423-5p_TGAGGGGCAGAGAGCGAGA1	-0,111830681	9,771757288	0,354686747	0,843764644	LADA vs T1D Time-adjusted
hsa-miR-486-5p_GTACTGAGCTGCCCCGA1	-0,392656358	4,600446211	0,355591215	0,843764644	LADA vs T1D Time-adjusted
hsa-miR-543_AAACATTCGCGGTGCACTTCTT1	0,528337142	3,525271253	0,356915012	0,843764644	LADA vs T1D Time-adjusted
hsa-miR-191-5p_AACGGAATCCCAAAAGCAGCTG1	0,283039525	5,768928318	0,357346594	0,843764644	LADA vs T1D Time-adjusted
hsa-miR-7-5p_TGGAAGACTAGTGATTTTGTTGTT1	0,523975756	4,490449806	0,358572385	0,844139155	LADA vs T1D Time-adjusted
hsa-let-7a-5p_GAGGTAGTAGGTTGTATAGT1	0,441297671	4,561721311	0,363810248	0,853928505	LADA vs T1D Time-adjusted
hsa-let-7i-5p_TGAGGTAGTAGTTTGTGCT1	-0,181467549	7,631066138	0,366238251	0,857084192	LADA vs T1D Time-adjusted
hsa-miR-92a-3p_TTGCACTTGTCCCGGCCTGT1	-0,145918673	8,379067596	0,367806004	0,858214009	LADA vs T1D Time-adjusted
hsa-miR-150-5p_TCTCCCAACCCTTGTACCA1	0,33746834	6,56516459	0,370546101	0,862064605	LADA vs T1D Time-adjusted
hsa-miR-486-5p_CCTGTACTGAGCTGCCCCGAG1	-0,152421489	11,24764136	0,372550068	0,863879866	LADA vs T1D Time-adjusted
hsa-miR-7-5p_TGGAAGACTAGTGATTTTGTTG1	0,435384917	3,867797174	0,373510637	0,863879866	LADA vs T1D Time-adjusted
hsa-miR-942-5p_TCTTCTCTGTTTTGGCCATGT1	0,515700629	2,575024753	0,375678616	0,866360889	LADA vs T1D Time-adjusted
hsa-miR-22-3p_AAGCTGCCAGTTGAAGAA1	0,407489183	5,032500306	0,38101525	0,876113555	LADA vs T1D Time-adjusted
hsa-miR-486-5p_TGTACTGAGCTGCCCCGA1	0,480221381	4,53697007	0,384622191	0,881769274	LADA vs T1D Time-adjusted
hsa-miR-24-3p_GGCTCAGTTCAGCAGGAAC1	0,549617676	2,918934836	0,385896014	0,881769274	LADA vs T1D Time-adjusted
hsa-miR-145-3p_ATTCCTGGAAATACTGTTCT1	-0,507130155	3,093740679	0,386819138	0,881769274	LADA vs T1D Time-adjusted
hsa-miR-320b_AAAAGCTGGGTTGAGAGGGCA1	-0,439437862	2,615161599	0,392838016	0,892916295	LADA vs T1D Time-adjusted
hsa-miR-186-5p_CAAAGAATTCTCCTTTTGGGCT1	0,126682959	8,984782262	0,394848982	0,893249571	LADA vs T1D Time-adjusted
hsa-miR-4433b-5p_TGTCCCACCCCCACTCCTGT1	-0,469340918	4,523145905	0,395243173	0,893249571	LADA vs T1D Time-adjusted
hsa-miR-342-3p_TCTCACACAGAAATCGCACCCGT1	0,183028253	8,723994136	0,398598411	0,894702223	LADA vs T1D Time-adjusted
hsa-miR-93-5p_CAAAGTGCTGTTCGTGCAGGT1	0,29457682	5,072528945	0,399296467	0,894702223	LADA vs T1D Time-adjusted
hsa-miR-486-5p_ATCCTGTACTGAGCTGCCCCGA1	-0,159868045	9,024331858	0,400364486	0,894702223	LADA vs T1D Time-adjusted
hsa-miR-3615_TCTCTCGGCTCCTCGCGGC1	0,478674534	2,023397327	0,40041035	0,894702223	LADA vs T1D Time-adjusted
hsa-miR-21-3p_CAACACCAGTCGATGGGCTGT1	-0,443616352	2,288436309	0,403096725	0,89483061	LADA vs T1D Time-adjusted
hsa-miR-3173-5p_CCCTGCCTGTTTTCTCCTTTGT1	0,443333933	1,917401292	0,403108274	0,89483061	LADA vs T1D Time-adjusted
hsa-let-7i-5p_TGAGGTAGTAGTTTGTGCTGTTG1	-0,428671128	3,944192775	0,40472202	0,89483061	LADA vs T1D Time-adjusted
hsa-miR-222-3p_AGCTACATCTGGCTACTGGGT1	-0,168438084	7,650900477	0,407378087	0,89483061	LADA vs T1D Time-adjusted
hsa-miR-183-5p_ATGGCACTGGTAGAATTCACTGT1	0,478738508	2,504851483	0,410155691	0,89483061	LADA vs T1D Time-adjusted
hsa-miR-150-5p_GTCTCCCAACCCTTGTACCAGTG1	0,486950793	3,077989788	0,412806343	0,89483061	LADA vs T1D Time-adjusted
hsa-miR-16-5p_TAGCAGCACGTAAATATTGG1	-0,122241936	10,9873739	0,413015627	0,89483061	LADA vs T1D Time-adjusted
hsa-miR-93-5p_CAAAGTGCTGTTCGTGCAGGTAGT1	0,450104744	4,055351232	0,415064503	0,89483061	LADA vs T1D Time-adjusted
hsa-miR-342-3p_TCTCACACAGAAATCGCACCC1	0,469142979	2,016844553	0,415878538	0,89483061	LADA vs T1D Time-adjusted
hsa-miR-133a-3p_TTGGTCCCCTTCAACCAGCTGT1	-0,517823599	2,487795126	0,41602126	0,89483061	LADA vs T1D Time-adjusted
hsa-miR-27b-3p_TTCACAGTGGCTAAGTTCTGC1	-0,16214204	7,857204391	0,416473728	0,89483061	LADA vs T1D Time-adjusted
hsa-let-7f-5p_TGAGGTAGTAGATTGTATAG1	-0,112468769	10,5471638	0,417617665	0,89483061	LADA vs T1D Time-adjusted
hsa-miR-125b-5p_TCCCTGAGACCCTAACTTGTGA1	0,310285173	5,621036711	0,418676487	0,89483061	LADA vs T1D Time-adjusted
hsa-miR-140-3p_TACCACAGGGTAGAACCACGGA1	-0,175617351	7,862508769	0,420148407	0,89483061	LADA vs T1D Time-adjusted
hsa-miR-329-3p_AACACACCTGGTTAACCTCTTT1	0,475825386	3,551980192	0,420854359	0,89483061	LADA vs T1D Time-adjusted
hsa-miR-106b-5p_TAAAGTGCTGACAGTGCAGA1	-0,342675536	5,030812023	0,421158023	0,89483061	LADA vs T1D Time-adjusted
hsa-miR-142-5p_CATAAAGTAGAAAGCACTAC1	0,452080522	2,318773636	0,421876563	0,89483061	LADA vs T1D Time-adjusted
hsa-miR-625-3p_GACTATAGAACTTTCCCCCTCA1	0,646662703	3,448522418	0,423118163	0,89483061	LADA vs T1D Time-adjusted
hsa-miR-193a-5p_TGGGTCTTTGCGGGCGAGAT1	0,368114071	4,70145732	0,423954703	0,89483061	LADA vs T1D Time-adjusted
hsa-miR-143-3p_TGAGATGAAGCACTGTAGCTCA1	0,262456949	6,633635761	0,424387008	0,89483061	LADA vs T1D Time-adjusted
hsa-miR-144-5p_GGATATCATCATATACTGTAAGT1	-0,223243164	6,455704528	0,424744171	0,89483061	LADA vs T1D Time-adjusted
hsa-miR-92a-3p_TGCACTTGTCCCGGCCTGT1	0,311412101	5,30065349	0,425355638	0,89483061	LADA vs T1D Time-adjusted
hsa-let-7d-3p_TATACGACCTGCTGCCTTT1	0,226098569	6,258064372	0,433152504	0,907437686	LADA vs T1D Time-adjusted
hsa-miR-122-5p_GGAGTGTGACAATGGTGTTTG1	-0,356521773	5,619888581	0,433642788	0,907437686	LADA vs T1D Time-adjusted
hsa-miR-502-3p_AATGCACCTGGGCAAGGATTCA1	0,308718489	4,779774283	0,435947332	0,909853139	LADA vs T1D Time-adjusted
hsa-miR-584-5p_TTATGGTTTGCCTGGGACTGA1	0,157915779	8,05116289	0,439123895	0,914071055	LADA vs T1D Time-adjusted
hsa-miR-885-5p_TCCATTACACTACCCTGCCTCT1	-0,807791911	4,712163409	0,442013334	0,917670728	LADA vs T1D Time-adjusted
hsa-miR-423-3p_AAGCTCGGTCTGAGGCCCCTCA1	0,414421294	2,829290184	0,443299004	0,91793066	LADA vs T1D Time-adjusted
hsa-let-7f-5p_GAGGTAGTAGATTGTATAGT1	0,46100992	3,007465312	0,445058326	0,919167457	LADA vs T1D Time-adjusted
hsa-miR-199a-3p_ACAGTAGTCTGCACATTGGTTA1	-0,628964086	4,923172942	0,448516185	0,923503366	LADA vs T1D Time-adjusted
hsa-miR-93-5p_CAAAGTGCTGTTCGTGCAGGTAG1	-0,09470411	9,966857204	0,449492789	0,923503366	LADA vs T1D Time-adjusted
hsa-miR-92a-3p_CACTTGTCCCGGCCTGT1	-0,387780179	4,184588919	0,450987765	0,923975053	LADA vs T1D Time-adjusted
hsa-miR-140-3p_TACCACAGGGTAGAACCACG1	0,305596861	4,858224511	0,452058591	0,923975053	LADA vs T1D Time-adjusted
hsa-miR-142-3p_TGTAGTGTTTCCTACTTTATGGA1	-0,413601757	3,574791071	0,455756027	0,929131489	LADA vs T1D Time-adjusted
hsa-miR-15a-5p_TAGCAGCACATAATGGTTTG1	0,546884637	2,765762945	0,457485146	0,930258998	LADA vs T1D Time-adjusted
hsa-miR-486-5p_CTGTACTGAGCTGCCCCGAG1	-0,168965209	7,807179841	0,45891736	0,930444284	LADA vs T1D Time-adjusted
hsa-miR-744-5p_TGCGGGGCTAGGGCTAACAGC1	0,358242177	3,989518529	0,459928844	0,930444284	LADA vs T1D Time-adjusted
hsa-miR-199a-5p_CCCAGTGTTCAGACTACCTGTTC1	-0,381925468	4,322462141	0,465050014	0,936347966	LADA vs T1D Time-adjusted

hsa-miR-150-5p_TCTCCCAACCCTTGTACCAGT1	0,143995287	10,28667682	0,465214603	0,936347966	LADA vs T1D Time-adjusted
hsa-miR-17-5p_CAAAGTGCTTACAGTGCAGGTAG1	0,407475195	3,22312992	0,466616452	0,936360929	LADA vs T1D Time-adjusted
hsa-miR-222-3p_AGCTACATCTGGCTACTGGGTC1	0,374226497	3,53327113	0,470793284	0,936360929	LADA vs T1D Time-adjusted
hsa-miR-151a-3p_TACTAGACTGAAGCTCCTTGAG1	0,3989266	2,948484967	0,471555973	0,936360929	LADA vs T1D Time-adjusted
hsa-miR-92a-3p_TATTGCACTTGTCCCGGCCTG1	-0,112016577	13,9436113	0,473900786	0,936360929	LADA vs T1D Time-adjusted
hsa-miR-21-5p_TAGCTTATCAGACTGATGT1	0,352402299	4,642902172	0,474433636	0,936360929	LADA vs T1D Time-adjusted
hsa-miR-93-3p_ACTGCTGAGCTAGCACTTCCCGA1	0,375166516	3,420021767	0,47458694	0,936360929	LADA vs T1D Time-adjusted
hsa-miR-16-5p_AGCAGCACGTAAATATTGG1	-0,389526428	3,202629707	0,474634639	0,936360929	LADA vs T1D Time-adjusted
hsa-miR-125b-5p_TCCCTGAGACCCTAACTTG1	0,13218967	8,160127514	0,476257264	0,936360929	LADA vs T1D Time-adjusted
hsa-miR-375-3p_TTTGTTCGTTCGGCTCGCGTG1	-0,24077512	7,069245953	0,476469187	0,936360929	LADA vs T1D Time-adjusted
hsa-miR-451a_AAACCGTTACCATTACTGAGTT1	-0,118206069	12,10291266	0,477058729	0,936360929	LADA vs T1D Time-adjusted
hsa-miR-192-5p_TGACCTATGAATTGACAGCCAG1	0,365388245	3,649860914	0,478401525	0,936672292	LADA vs T1D Time-adjusted
hsa-miR-183-5p_TATGGCACTGGTAGAATT1	0,41843344	2,845053887	0,482181598	0,937703578	LADA vs T1D Time-adjusted
hsa-miR-144-5p_GGATATCATCATATACTGTAAG1	0,40270376	4,158150293	0,482531596	0,937703578	LADA vs T1D Time-adjusted
hsa-miR-486-3p_CGGGGCAGCTCAGTACAGGAT1	-0,18886238	7,767064538	0,48344262	0,937703578	LADA vs T1D Time-adjusted
hsa-miR-155-5p_TTAATGCTAATCGTGATAGGGGTT1	0,399446495	3,129748949	0,483670114	0,937703578	LADA vs T1D Time-adjusted
hsa-miR-92b-3p_TATTGCACTCGTCCCGGCC1	0,379828986	3,918389355	0,486191604	0,937849155	LADA vs T1D Time-adjusted
hsa-miR-32-5p_TATTGCACATTACTAAGTTG1	-0,370110221	4,122651019	0,487890139	0,937849155	LADA vs T1D Time-adjusted
hsa-miR-22-3p_AAGCTGCCAGTTGAAGAACTG1	0,131539465	9,199421356	0,488266532	0,937849155	LADA vs T1D Time-adjusted
hsa-miR-192-5p_CTGACCTATGAATTGACAGC1	-0,398930759	2,069053686	0,489435027	0,937849155	LADA vs T1D Time-adjusted
hsa-let-7d-3p_CTATACGACCTGCTGCCTTT1	0,103447251	10,25191544	0,491203837	0,937849155	LADA vs T1D Time-adjusted
hsa-miR-423-3p_AAGCTCGGTCTGAGGCCCCT1	0,416645036	2,52885255	0,492036637	0,937849155	LADA vs T1D Time-adjusted
hsa-miR-4732-5p_TGTAGAGCAGGGAGCAGGAAGCT1	-0,224020183	6,317387172	0,493426036	0,937849155	LADA vs T1D Time-adjusted
hsa-miR-4732-3p_GCCCTGACCTGTCCTGTTCTG1	-0,62425856	4,166204088	0,493669452	0,937849155	LADA vs T1D Time-adjusted
hsa-miR-182-5p_TTTGGCAATGGTAGAACTCACACTGG1	0,407905697	2,345773362	0,494641176	0,937849155	LADA vs T1D Time-adjusted
hsa-let-7d-5p_AGAGGTAGTAGGTTGCATAGT1	0,1765456	7,384387884	0,496184999	0,937849155	LADA vs T1D Time-adjusted
hsa-miR-181a-2-3p_ACCACTGACCGTTGACTGTACC1	-0,401146805	2,432112395	0,496787353	0,937849155	LADA vs T1D Time-adjusted
hsa-miR-223-5p_CGTGTATTTGACAAGCTGAGTTG1	0,264949008	5,926691134	0,499928486	0,941531981	LADA vs T1D Time-adjusted
hsa-miR-186-5p_CAAAGAATTCTCCTTTTGGGC1	0,222632829	6,378401215	0,504307977	0,945136698	LADA vs T1D Time-adjusted
hsa-miR-374b-5p_ATATAATACAACCTGCTAAGT1	0,324321427	3,94114477	0,506038699	0,945136698	LADA vs T1D Time-adjusted
hsa-miR-1301-3p_TTGCAGCTGCCTGGGAGTGA1	-0,396748278	3,323484613	0,506502377	0,945136698	LADA vs T1D Time-adjusted
hsa-miR-424-3p_CAAAACGTGAGGCGCTGCTAT1	0,221437617	5,546308926	0,507540104	0,945136698	LADA vs T1D Time-adjusted
hsa-let-7a-5p_ATGAGGTAGTAGGTTGTATAGTT1	-0,399022912	2,145462754	0,507977459	0,945136698	LADA vs T1D Time-adjusted
hsa-miR-339-3p_TGAGCGCCTCGACGACAGAGC1	0,323299159	4,202757825	0,50965832	0,945136698	LADA vs T1D Time-adjusted
hsa-miR-100-5p_AACCCGTAGATCCGAACTTGT1	0,173619671	7,209550932	0,510206536	0,945136698	LADA vs T1D Time-adjusted
hsa-miR-222-3p_AGCTACATCTGGCTACTGGGTCT1	0,197041171	6,477997223	0,51206963	0,94538242	LADA vs T1D Time-adjusted
hsa-miR-424-3p_CAAAACGTGAGGCGCTGCT1	-0,352184511	3,07909127	0,51272953	0,94538242	LADA vs T1D Time-adjusted
hsa-miR-140-3p_TACCACAGGGTAGAACCACGGACA1	-0,302406716	3,592791188	0,517018903	0,948739953	LADA vs T1D Time-adjusted
hsa-miR-140-3p_ACCACAGGGTAGAACCACGGAC1	-0,213633291	5,842587001	0,517947573	0,948739953	LADA vs T1D Time-adjusted
hsa-miR-151a-3p_CTAGACTGAAGCTCCTTGAGGA1	0,120552697	8,369867531	0,518609662	0,948739953	LADA vs T1D Time-adjusted
hsa-miR-146b-5p_TGAGAACTGAATTCCATAGGCTGT1	0,111848573	8,367181967	0,519348166	0,948739953	LADA vs T1D Time-adjusted
hsa-miR-146a-5p_TGAGAACTGAATTCCATGGGTTGT1	0,170514514	7,268590607	0,522702417	0,952414556	LADA vs T1D Time-adjusted
hsa-miR-450b-5p_TTTTGCAATATGTTCCTGAAT1	-0,298171156	4,972105489	0,523988895	0,952414556	LADA vs T1D Time-adjusted
hsa-miR-423-5p_AGGGGCAGAGAGCGAGACTTT1	0,260632684	5,179351064	0,526802105	0,952414556	LADA vs T1D Time-adjusted
hsa-miR-505-3p_CGTCAACACTTGCTGGTTT1	-0,339557036	3,312021603	0,529160248	0,952414556	LADA vs T1D Time-adjusted
hsa-let-7c-5p_TGAGGTAGTAGGTTGTATGGTTT1	-0,311540168	3,632126079	0,530206783	0,952414556	LADA vs T1D Time-adjusted
hsa-miR-146a-5p_TGAGAACTGAATTCCATGGGTTG1	0,134566774	7,862859516	0,533261809	0,952414556	LADA vs T1D Time-adjusted
hsa-miR-181a-2-3p_ACCACTGACCGTTGACTGT1	0,35209003	2,626525168	0,53428717	0,952414556	LADA vs T1D Time-adjusted
hsa-miR-451a_ACCGTTACCATTACTGAGT1	0,175209622	7,438165305	0,534580623	0,952414556	LADA vs T1D Time-adjusted
hsa-miR-26a-5p_TCAAGTAATCCAGGATAGGCT1	0,19738978	6,500759116	0,536066291	0,952414556	LADA vs T1D Time-adjusted
hsa-miR-30e-3p_CTTTCAGTCGGATGTTTACAG1	-0,346608505	2,479912206	0,536085785	0,952414556	LADA vs T1D Time-adjusted
hsa-miR-485-3p_GTCATACACGGCTCTCCTCTCT1	-0,356306333	5,721064879	0,538583572	0,952414556	LADA vs T1D Time-adjusted
hsa-miR-423-3p_AGCTCGGTCTGAGGCCCC1	-0,322062507	3,601847184	0,539780319	0,952414556	LADA vs T1D Time-adjusted
hsa-let-7b-5p_GAGGTAGTAGGTTGTGTGG1	-0,199018329	6,238396809	0,540647325	0,952414556	LADA vs T1D Time-adjusted
hsa-miR-224-5p_CAAGTCACTAGTGGTTCCGTTTAG1	-0,344448398	4,656228151	0,541232379	0,952414556	LADA vs T1D Time-adjusted
hsa-miR-126-5p_CATTATTACTTTTGGTACGC1	-0,329672812	3,708699797	0,541421181	0,952414556	LADA vs T1D Time-adjusted
hsa-let-7f-5p_TGAGGTAGTAGATTG1	0,310237266	4,368619365	0,54169366	0,952414556	LADA vs T1D Time-adjusted
hsa-miR-28-3p_CACTAGATTGTGAGCTCCTGGA1	0,163465837	6,790249103	0,542486895	0,952414556	LADA vs T1D Time-adjusted
hsa-miR-125b-5p_TCCCTGAGACCCTAACTT1	-0,330178893	2,928962579	0,543395295	0,952414556	LADA vs T1D Time-adjusted
hsa-miR-122-5p_TGGAGTGTGACAATGGTGTTTGT1	-0,196998068	8,317298507	0,545411038	0,952414556	LADA vs T1D Time-adjusted
hsa-miR-363-3p_ATTGCACGGTATCCATCTG1	0,352583423	2,190954814	0,546317109	0,952414556	LADA vs T1D Time-adjusted

hsa-miR-15b-5p_TAGCAGCACATCATGGTTTAC1	0,129591971	7,359413397	0,547460206	0,952414556	LADA vs T1D Time-adjusted
hsa-miR-486-5p_TCCTGTACTGAGCTGCCCCGAGG1	0,120584938	7,407933707	0,547966425	0,952414556	LADA vs T1D Time-adjusted
hsa-miR-142-5p_CCCATAAAGTAGAAAGCACT1	0,072187781	11,11931665	0,549053145	0,952414556	LADA vs T1D Time-adjusted
hsa-miR-629-5p_TGGGTTTACGTTGGGAGAAC1	-0,335861271	3,80742001	0,551651897	0,954828556	LADA vs T1D Time-adjusted
hsa-miR-486-5p_CCTGTACTGAGCTGCCCCGA1	-0,101183595	11,06703044	0,554015463	0,956102833	LADA vs T1D Time-adjusted
hsa-miR-106b-3p_CCGCACTGTGGGTACTTGCTG1	-0,269369745	4,149873679	0,556876982	0,956102833	LADA vs T1D Time-adjusted
hsa-miR-21-5p_GTAGCTTATCAGACTGATGTTGA1	-0,345812218	2,233806863	0,557949261	0,956102833	LADA vs T1D Time-adjusted
hsa-miR-486-5p_ATCCTGTACTGAGCTGCCCCG1	-0,185626157	6,267380926	0,560735074	0,956102833	LADA vs T1D Time-adjusted
hsa-miR-584-5p_TTATGGTTTGCCTGGGACTG1	0,323857088	2,451626756	0,562721997	0,956102833	LADA vs T1D Time-adjusted
hsa-miR-194-5p_TGTAACAGCAACTCCATGTGG1	0,160270447	6,643496377	0,564271999	0,956102833	LADA vs T1D Time-adjusted
hsa-miR-199a-3p_ACAGTAGTCTGCACATTGGTT1	0,508410439	4,587596	0,564703734	0,956102833	LADA vs T1D Time-adjusted
hsa-miR-99b-5p_CACCCGTAGAACCGACCTTGC1	0,093418849	7,928619761	0,565500253	0,956102833	LADA vs T1D Time-adjusted
hsa-miR-425-5p_ATGACACGATCACTCCCGTTG1	-0,328327642	2,364893848	0,566422846	0,956102833	LADA vs T1D Time-adjusted
hsa-miR-339-3p_TGAGCGCCTCGACGACAGAG1	-0,23686823	5,283296909	0,566698723	0,956102833	LADA vs T1D Time-adjusted
hsa-miR-21-5p_TAGCTTATCAGACTGATGTTG1	0,072846417	11,65018988	0,567455773	0,956102833	LADA vs T1D Time-adjusted
hsa-miR-484_CAGGCTCAGTCCCCTCCCGAT1	-0,305410947	2,348599265	0,567823188	0,956102833	LADA vs T1D Time-adjusted
hsa-miR-423-5p_GAGGGGCAGAGAGCGAGACT1	-0,183359087	6,892897172	0,570826834	0,956102833	LADA vs T1D Time-adjusted
hsa-miR-382-5p_GAAGTTGTTCGTGGTGGATTCG1	-0,210601653	7,539547941	0,573318693	0,956102833	LADA vs T1D Time-adjusted
hsa-miR-484_TCAGGCTCAGTCCCCTCCCG1	-0,150942824	7,776089511	0,5769883	0,956102833	LADA vs T1D Time-adjusted
hsa-miR-92a-3p_ATTGCACTTGTCCCGGCCTG1	-0,102381051	9,128354405	0,577962048	0,956102833	LADA vs T1D Time-adjusted
hsa-miR-197-3p_TTCACCACCTTCTCCACCCAG1	0,108673202	8,701233583	0,579953436	0,956102833	LADA vs T1D Time-adjusted
hsa-miR-24-3p_GCTCAGTTCAGCAGGAACAG1	0,305564648	2,351084248	0,581021497	0,956102833	LADA vs T1D Time-adjusted
hsa-let-7b-5p_TGAGGTAGTAGGTTGTGTGGTTT1	-0,067288613	11,27639147	0,581329788	0,956102833	LADA vs T1D Time-adjusted
hsa-miR-183-5p_TATGGCACTGGTAGAATTCA1	-0,165390717	7,316738774	0,583340482	0,956102833	LADA vs T1D Time-adjusted
hsa-miR-361-3p_TCCCCCAGGTGTGATTCTGATTTG1	0,177938986	5,300626315	0,583526731	0,956102833	LADA vs T1D Time-adjusted
hsa-miR-223-3p_GTCAGTTTGTCAAATACCCCAA1	-0,163886112	7,633945564	0,583999179	0,956102833	LADA vs T1D Time-adjusted
hsa-miR-92a-3p_TATTGCACTTGTCCCGGCCTGTT1	0,071412389	10,36371218	0,585319139	0,956102833	LADA vs T1D Time-adjusted
hsa-miR-27a-3p_TTCACAGTGGCTAAGTTCC1	-0,201074355	5,362754207	0,585641613	0,956102833	LADA vs T1D Time-adjusted
hsa-miR-25-3p_CATTGCACTTGTCTCGGTCTG1	-0,110622317	9,96694873	0,586399115	0,956102833	LADA vs T1D Time-adjusted
hsa-miR-30d-5p_TGTAAACATCCCCGACTGGAAG1	0,050434351	12,1725605	0,588273772	0,956102833	LADA vs T1D Time-adjusted
hsa-miR-26a-5p_TTCAAGTAATCCAGGATAGGCT1	0,088565757	11,89898908	0,588389752	0,956102833	LADA vs T1D Time-adjusted
hsa-miR-92a-3p_TATTGCACTTGTCCCGGCCT1	-0,091137869	10,29739458	0,590339481	0,956102833	LADA vs T1D Time-adjusted
hsa-miR-342-3p_TCACACAGAAATCGCACCCGTC1	0,312961562	2,415699371	0,590826706	0,956102833	LADA vs T1D Time-adjusted
hsa-miR-382-5p_AAGTTGTTCGTGGTGGATTCG1	0,339446786	2,276351927	0,592006989	0,956102833	LADA vs T1D Time-adjusted
hsa-miR-24-3p_GGCTCAGTTCAGCAGGAACAG1	-0,133867108	6,840568144	0,592079204	0,956102833	LADA vs T1D Time-adjusted
hsa-miR-4732-5p_TGTAGAGCAGGGAGCAGGAAG1	0,332623924	2,884522745	0,594542552	0,956102833	LADA vs T1D Time-adjusted
hsa-let-7b-5p_GAGGTAGTAGGTTGTGTGGTTT1	0,280424035	3,820264571	0,594659775	0,956102833	LADA vs T1D Time-adjusted
hsa-miR-652-3p_AATGGCGCCACTAGGGTTGT1	-0,088269626	8,007167607	0,594686745	0,956102833	LADA vs T1D Time-adjusted
hsa-miR-181b-5p_AACATTCATTGCTGTCGGTGGGT1	0,280068536	3,495629927	0,595036567	0,956102833	LADA vs T1D Time-adjusted
hsa-miR-222-3p_AGCTACATCTGGCTACTGGG1	0,312533094	2,571182702	0,595902271	0,956102833	LADA vs T1D Time-adjusted
hsa-miR-451a_AACCGTTACCATTACTGAGTTT1	0,303049068	2,393187549	0,597944011	0,957436665	LADA vs T1D Time-adjusted
hsa-miR-223-3p_GTCAGTTTGTCAAATACCCCA1	0,170075064	6,353871952	0,601884229	0,958401716	LADA vs T1D Time-adjusted
hsa-miR-23a-3p_TCACATTGCCAGGGATTTC1	0,304152245	2,507144033	0,601994598	0,958401716	LADA vs T1D Time-adjusted
hsa-miR-3613-5p_TGTTGTACTTTTTTTTTTGT1	-0,093684292	7,575367225	0,602181609	0,958401716	LADA vs T1D Time-adjusted
hsa-miR-148a-3p_TCAGTGCACTACAGAACTTT1	0,305037803	4,13799763	0,606627068	0,962567973	LADA vs T1D Time-adjusted
hsa-miR-29c-3p_TAGCACCATTTGAAATCGGT1	-0,264954259	4,535898634	0,60783033	0,962567973	LADA vs T1D Time-adjusted
hsa-miR-664a-3p_TATTCATTTATCCCCAGCCTACA1	0,285301245	2,990893166	0,609566455	0,962567973	LADA vs T1D Time-adjusted
hsa-miR-30e-5p_TGTAAACATCCTTGACTGGA1	0,290459125	2,526545713	0,609981967	0,962567973	LADA vs T1D Time-adjusted
hsa-miR-28-3p_CACTAGATTGTGAGCTCCTGGAG1	-0,277277438	3,068939292	0,611438536	0,962567973	LADA vs T1D Time-adjusted
hsa-miR-92b-3p_TATTGCACTCGTCCCGGCCT1	-0,216256502	5,642753779	0,612567196	0,962567973	LADA vs T1D Time-adjusted
hsa-miR-16-2-3p_ACCAATATTACTGTGCTGCTT1	-0,091671246	8,335786686	0,613317646	0,962567973	LADA vs T1D Time-adjusted
hsa-miR-25-3p_CATTGCACTTGTCTCGGTC1	-0,180018173	5,824634034	0,617062285	0,966527263	LADA vs T1D Time-adjusted
hsa-miR-433-3p_ATCATGATGGGCTCCTCGGTGT1	-0,303759748	4,238564116	0,62094284	0,970043971	LADA vs T1D Time-adjusted
hsa-miR-16-2-3p_ACCAATATTACTGTGCTGCT1	0,205723817	4,640125469	0,622467929	0,970043971	LADA vs T1D Time-adjusted
hsa-miR-26b-5p_TTCAAGTAATTCAGGATAGGT1	-0,104899471	7,33079836	0,625441567	0,970043971	LADA vs T1D Time-adjusted
hsa-miR-221-3p_AGCTACATTGTCTGCTGGGTT1	-0,168414512	6,712650504	0,627665572	0,970043971	LADA vs T1D Time-adjusted
hsa-miR-30d-5p_TAAACATCCCCGACTGGAAGCT1	0,261940938	2,129907594	0,630589588	0,970043971	LADA vs T1D Time-adjusted
hsa-miR-451a_ACCGTTACCATTACTGAGTT1	-0,275071568	2,058885942	0,630594869	0,970043971	LADA vs T1D Time-adjusted
hsa-miR-4446-3p_CAGGGCTGGCAGTGACATGGGT1	0,300776325	3,595995315	0,632147353	0,970043971	LADA vs T1D Time-adjusted
hsa-miR-342-3p_TCTCACACAGAAATCGCACCCG1	-0,10825734	9,698837248	0,634060893	0,970043971	LADA vs T1D Time-adjusted
hsa-miR-29b-3p_TAGCACCATTTGAAATCAGT1	-0,236610695	4,397473372	0,634723429	0,970043971	LADA vs T1D Time-adjusted

hsa-miR-23b-3p_ATCACATTGCCAGGGATTACCAC1	-0,223288553	5,125539346	0,635609054	0,970043971	LADA vs T1D Time-adjusted
hsa-miR-30e-5p_TGTAAACATCCTTGACTGGAAGC1	0,057127774	8,361780675	0,64020689	0,970043971	LADA vs T1D Time-adjusted
hsa-miR-423-5p_TGAGGGGCAGAGAGCGAGACTTT1	-0,033879198	14,46310907	0,641206092	0,970043971	LADA vs T1D Time-adjusted
hsa-miR-191-5p_ACGGAATCCCAAAAGCAGCT1	0,268549766	1,917089883	0,642165593	0,970043971	LADA vs T1D Time-adjusted
hsa-miR-140-3p_ACCACAGGGTAGAACCACGG1	0,242487477	3,499713471	0,643741123	0,970043971	LADA vs T1D Time-adjusted
hsa-miR-485-5p_AGAGGCTGGCCGTGATGAATTCG1	0,257963153	4,445743314	0,643983408	0,970043971	LADA vs T1D Time-adjusted
hsa-miR-486-5p_TCCTGTACTGAGCTGCCCCGAG1	-0,072531344	17,20272224	0,644067679	0,970043971	LADA vs T1D Time-adjusted
hsa-miR-361-5p_TTATCAGAATCTCCAGGGGTA1	-0,250284574	3,316391684	0,646786239	0,970043971	LADA vs T1D Time-adjusted
hsa-miR-6803-3p_TCCCTCGCCTTCTCACCCTCA1	-0,173821698	6,152212628	0,64686796	0,970043971	LADA vs T1D Time-adjusted
hsa-miR-4433b-5p_TGTCCCACCCCCACTCCTGTTT1	-0,171164311	8,44296889	0,649092035	0,970043971	LADA vs T1D Time-adjusted
hsa-miR-374a-5p_TTATAATACAACCTGATAAGT1	0,270799304	2,272272471	0,649894123	0,970043971	LADA vs T1D Time-adjusted
hsa-miR-92a-3p_TATTGCACTTGTCCCGGCCTGT1	-0,051291893	17,21206299	0,653316909	0,970043971	LADA vs T1D Time-adjusted
hsa-let-7a-5p_TGAGGTAGTAGGTTGTATA1	-0,155503684	5,691720869	0,657074751	0,970043971	LADA vs T1D Time-adjusted
hsa-miR-10b-5p_ACCCTGTAGAACCGAATTTG1	0,215448527	4,807768063	0,658259991	0,970043971	LADA vs T1D Time-adjusted
hsa-miR-451a_AACCGTTACCATTACTGAGTT1	-0,147544553	6,235439111	0,658550464	0,970043971	LADA vs T1D Time-adjusted
hsa-miR-425-5p_AATGACACGATCACTCCCGTTG1	0,062476055	9,327235486	0,659038479	0,970043971	LADA vs T1D Time-adjusted
hsa-miR-3613-5p_TGTTGTACTTTTTTTTTTGTTC1	0,198353607	4,360142246	0,660086963	0,970043971	LADA vs T1D Time-adjusted
hsa-miR-140-3p_ACCACAGGGTAGAACCACG1	-0,252391662	2,745546867	0,661314829	0,970043971	LADA vs T1D Time-adjusted
hsa-miR-101-3p_GTACAGTACTGTGATAACTGA1	-0,073816881	8,212735415	0,661402969	0,970043971	LADA vs T1D Time-adjusted
hsa-miR-28-3p_ACTAGATTGTGAGCTCCTGGAG1	0,210491116	4,665468153	0,661725631	0,970043971	LADA vs T1D Time-adjusted
hsa-miR-130a-3p_CAGTGCAATGTTAAAAGGGCA1	-0,198384201	4,392768352	0,661969712	0,970043971	LADA vs T1D Time-adjusted
hsa-let-7i-5p_TGAGGTAGTAGTTTGTGCTG1	-0,073322063	10,0893919	0,663148705	0,970043971	LADA vs T1D Time-adjusted
hsa-miR-320a-3p_GAAAAGCTGGGTTGAGAGGGCGA1	-0,22547434	3,726965694	0,665411327	0,970043971	LADA vs T1D Time-adjusted
hsa-miR-99b-5p_ACCCGTAGAACCGACCTTGCG1	0,252711368	2,144852077	0,667405788	0,970043971	LADA vs T1D Time-adjusted
hsa-miR-125b-5p_TCCCTGAGACCCTAACTTGTG1	-0,182973453	5,122028633	0,668835064	0,970043971	LADA vs T1D Time-adjusted
hsa-miR-125a-5p_TCCCTGAGACCCTTTAACCTG1	0,082414559	8,335881469	0,668940106	0,970043971	LADA vs T1D Time-adjusted
hsa-miR-1301-3p_TTGCAGCTGCCTGGGAGTGACTTC1	-0,274618802	2,475326197	0,669151709	0,970043971	LADA vs T1D Time-adjusted
hsa-miR-494-3p_TGAAACATACACGGGAAACCTCT1	-0,238041768	4,130022225	0,669710665	0,970043971	LADA vs T1D Time-adjusted
hsa-miR-29a-3p_TAGCACCATCTGAAATCGGTTA1	-0,193789945	4,650458238	0,670421628	0,970043971	LADA vs T1D Time-adjusted
hsa-miR-23a-3p_TCACATTGCCAGGGATTTCC1	-0,219166809	4,52192031	0,671482765	0,970043971	LADA vs T1D Time-adjusted
hsa-miR-451a_AAACCGTTACCATTACTGAG1	0,074308292	13,72351402	0,672390168	0,970043971	LADA vs T1D Time-adjusted
hsa-miR-320a-3p_AAAAGCTGGGTTGAGAGGGCGAAA1	-0,124138722	6,765627538	0,674216575	0,970043971	LADA vs T1D Time-adjusted
hsa-miR-361-3p_CCCCCAGGTGTGATTCTGATTTG1	0,244046628	1,935808496	0,67691372	0,970043971	LADA vs T1D Time-adjusted
hsa-miR-409-3p_AATGTTGCTCGGTGAACCCCT1	-0,255527648	4,168161381	0,676995887	0,970043971	LADA vs T1D Time-adjusted
hsa-miR-1306-5p_CCACCTCCCCTGCAAACGTCC1	0,225044812	4,59875793	0,677542396	0,970043971	LADA vs T1D Time-adjusted
hsa-miR-27a-3p_TTCACAGTGGCTAAGTTCCGC1	-0,131534987	6,161654231	0,679196958	0,970043971	LADA vs T1D Time-adjusted
hsa-miR-451a_AAACCGTTACCATTACTGAGT1	0,070295595	16,52329399	0,679934914	0,970043971	LADA vs T1D Time-adjusted
hsa-miR-148a-3p_TCAGTGCACTACAGAACTT1	-0,251200457	2,313074364	0,682191767	0,970043971	LADA vs T1D Time-adjusted
hsa-miR-99a-5p_AACCCGTAGATCCGATCTTGT1	0,073400489	8,596582119	0,682193712	0,970043971	LADA vs T1D Time-adjusted
hsa-let-7d-3p_CTATACGACCTGCTGCCTTTC1	0,045626572	10,31942736	0,682461454	0,970043971	LADA vs T1D Time-adjusted
hsa-miR-222-3p_AGCTACATCTGGCTACTGGGTCTC1	0,186910007	5,925164986	0,682750472	0,970043971	LADA vs T1D Time-adjusted
hsa-miR-574-3p_CACGCTCATGCACACACCCACA1	0,123096589	6,242074994	0,686485437	0,970043971	LADA vs T1D Time-adjusted
hsa-miR-98-5p_TGAGGTAGTAAGTTGTATTG1	0,176306839	4,697784917	0,689440113	0,970043971	LADA vs T1D Time-adjusted
hsa-miR-92a-3p_GCACTTGTCCCGGCCTGT1	-0,184573132	4,472613341	0,690562528	0,970043971	LADA vs T1D Time-adjusted
hsa-miR-140-3p_ACCACAGGGTAGAACCACGGACA1	-0,178431561	4,273604017	0,691433228	0,970043971	LADA vs T1D Time-adjusted
hsa-miR-323a-3p_GCACATTACACGGTCGACCTCT1	-0,226714823	3,791926149	0,692036707	0,970043971	LADA vs T1D Time-adjusted
hsa-miR-181a-5p_AACATTCAACGCTGTCGG1	0,201791649	2,244051199	0,695596947	0,970043971	LADA vs T1D Time-adjusted
hsa-miR-1294_TGTGAGGTTGGCATTGTTGT1	0,221080058	2,446198713	0,697006419	0,970043971	LADA vs T1D Time-adjusted
hsa-miR-23a-3p_ATCACATTGCCAGGGATTTC1	-0,058281553	9,519525675	0,697238638	0,970043971	LADA vs T1D Time-adjusted
hsa-miR-191-5p_CAACGGAATCCCAAAAGCAGCTG1	-0,069247715	9,63370255	0,697430851	0,970043971	LADA vs T1D Time-adjusted
hsa-miR-23a-5p_GGGGTTCCTGGGGATGGGATT1	0,210503559	3,763349774	0,697638955	0,970043971	LADA vs T1D Time-adjusted
hsa-miR-223-3p_TGTCAGTTTGTCAAATACCCC1	0,120228818	7,234009162	0,698101535	0,970043971	LADA vs T1D Time-adjusted
hsa-miR-181a-5p_AACATTCAACGCTGTCGGTGA1	-0,045934141	10,01113916	0,69817668	0,970043971	LADA vs T1D Time-adjusted
hsa-miR-7-5p_TGGAAGACTAGTGATTTTGTTGT1	0,148826609	6,235025385	0,69951406	0,970043971	LADA vs T1D Time-adjusted
hsa-miR-487b-3p_TCGTACAGGGTCATCCACTTT1	0,235623783	2,989944331	0,700609732	0,970043971	LADA vs T1D Time-adjusted
hsa-miR-6803-3p_TCCCTCGCCTTCTCACCCTCAG1	-0,212953498	2,545548118	0,703688176	0,970043971	LADA vs T1D Time-adjusted
hsa-miR-30e-5p_GTAAACATCCTTGACTGGAAGCT1	0,044478135	8,789096373	0,706240206	0,970043971	LADA vs T1D Time-adjusted
hsa-miR-92a-3p_TATTGCACTTGTCCCGGCC1	-0,088169296	8,042410264	0,708235216	0,970043971	LADA vs T1D Time-adjusted
hsa-miR-193a-5p_TGGGTCTTTGCGGGCGAGATG1	0,146383561	5,874910096	0,708540822	0,970043971	LADA vs T1D Time-adjusted
hsa-miR-99a-5p_AACCCGTAGATCCGATCTTG1	0,076928999	8,068040532	0,708971177	0,970043971	LADA vs T1D Time-adjusted
hsa-miR-21-5p_TAGCTTATCAGACTGATGTTGA1	0,032070013	12,8658918	0,7097248	0,970043971	LADA vs T1D Time-adjusted

hsa-miR-335-3p_TTTTTCATTATTGCTCCTGACC1	-0,208594664	3,947185728	0,710475905	0,970043971	LADA vs T1D Time-adjusted
hsa-miR-101-3p_TACAGTACTGTGATAACTGAA1	0,220293759	2,97176202	0,71080766	0,970043971	LADA vs T1D Time-adjusted
hsa-miR-194-5p_TGTAACAGCAACTCCATGTGGAA1	-0,200577349	2,71610017	0,711807275	0,970043971	LADA vs T1D Time-adjusted
hsa-miR-432-5p_TCTTGGAGTAGGTCATTGGGTGG1	0,225793732	3,31432182	0,714164308	0,970043971	LADA vs T1D Time-adjusted
hsa-miR-152-3p_TCAGTGCATGACAGAACTTGG1	0,191164986	2,028197906	0,714645566	0,970043971	LADA vs T1D Time-adjusted
hsa-miR-505-3p_GTCAACACTTGCTGGTTTCCTCT1	0,221853448	2,102281537	0,714836429	0,970043971	LADA vs T1D Time-adjusted
hsa-miR-425-5p_ATGACACGATCACTCCCGTTGAGT1	0,192588167	4,481314722	0,715494494	0,970043971	LADA vs T1D Time-adjusted
hsa-let-7a-5p_TTGAGGTAGTAGGTTGTATAGTT1	0,206640504	1,879153168	0,716098689	0,970043971	LADA vs T1D Time-adjusted
hsa-miR-30c-5p_TGTAAACATCCTACACTCTCA1	0,085709945	7,960145287	0,7165294	0,970043971	LADA vs T1D Time-adjusted
hsa-miR-146b-5p_TGAGAACTGAATTCCATAGGCTG1	-0,134441782	5,796387655	0,717415579	0,970043971	LADA vs T1D Time-adjusted
hsa-miR-29a-3p_TAGCACCATCTGAAATCGG1	-0,090833254	7,313106214	0,720090074	0,970959607	LADA vs T1D Time-adjusted
hsa-miR-425-5p_AATGACACGATCACTCCCG1	-0,171970847	4,588646685	0,720547774	0,970959607	LADA vs T1D Time-adjusted
hsa-miR-21-5p_TAGCTTATCAGACTGATGTTGACT1	0,176214874	5,746314182	0,724808621	0,975040169	LADA vs T1D Time-adjusted
hsa-miR-197-3p_TTCACCACCTTCTCCACCCA1	-0,191802484	3,766113409	0,728014349	0,977689898	LADA vs T1D Time-adjusted
hsa-miR-186-5p_CAAAGAATTCTCCTTTTGGGCTTT1	-0,18643781	3,921463053	0,729870151	0,978520829	LADA vs T1D Time-adjusted
hsa-miR-425-5p_AATGACACGATCACTCCCGTTGAG1	0,136763723	5,989511543	0,734095588	0,981019783	LADA vs T1D Time-adjusted
hsa-let-7a-5p_TGAGGTAGTAGGTTGTATAGTTTT1	0,200487195	2,919534024	0,734214553	0,981019783	LADA vs T1D Time-adjusted
hsa-miR-148a-3p_CAGTGCACTACAGAACTTTGT1	-0,199675126	2,153358606	0,736514487	0,982433321	LADA vs T1D Time-adjusted
hsa-miR-10a-5p_TACCCTGTAGATCCGAATTT1	0,168916754	4,289428906	0,738705307	0,982508111	LADA vs T1D Time-adjusted
hsa-let-7a-5p_TGAGGTAGTAGGTTGTAT1	-0,228701647	3,337435577	0,740249159	0,982508111	LADA vs T1D Time-adjusted
hsa-miR-92a-3p_ATTGCACTTGTCCCGGCCT1	-0,165086705	4,481036729	0,741506244	0,982508111	LADA vs T1D Time-adjusted
hsa-miR-589-5p_TGAGAACCACGTCTGCTCTGA1	0,189261163	3,05621435	0,741538991	0,982508111	LADA vs T1D Time-adjusted
hsa-miR-22-3p_AGCTGCCAGTTGAAGAACT1	0,193640702	2,719982364	0,744214995	0,984340326	LADA vs T1D Time-adjusted
hsa-miR-3158-3p_AAGGGCTTCCTCTCTGCAGGA1	0,195663882	2,786574243	0,745410689	0,984340326	LADA vs T1D Time-adjusted
hsa-miR-501-3p_AATGCACCCGGGCAAGGAT1	-0,187191376	2,776935459	0,748623145	0,986518947	LADA vs T1D Time-adjusted
hsa-miR-30d-5p_GTAAACATCCCCGACTGGAAG1	-0,098130161	5,943376472	0,750445098	0,986518947	LADA vs T1D Time-adjusted
hsa-miR-181a-5p_AACATTCAACGCTGTCGGT1	0,126134541	5,49798078	0,75080203	0,986518947	LADA vs T1D Time-adjusted
hsa-miR-122-5p_TGGAGTGTGACAATGGTGTTT1	-0,083317846	13,69364351	0,753589265	0,988539152	LADA vs T1D Time-adjusted
hsa-miR-140-3p_TACCACAGGGTAGAACCACGGAC1	-0,167197343	4,133710403	0,755504861	0,988610748	LADA vs T1D Time-adjusted
hsa-miR-425-5p_AATGACACGATCACTCCCGTTGA1	-0,033250603	10,33255905	0,760277169	0,988610748	LADA vs T1D Time-adjusted
hsa-let-7g-5p_GAGGTAGTAGTTTGTACAGT1	0,172720056	3,503297517	0,761441223	0,988610748	LADA vs T1D Time-adjusted
hsa-miR-574-3p_CACGCTCATGCACACACCCAC1	-0,153905607	3,990306713	0,762861854	0,988610748	LADA vs T1D Time-adjusted
hsa-miR-183-5p_ATGGCACTGGTAGAATTCACTG1	-0,172026178	2,958551449	0,762872271	0,988610748	LADA vs T1D Time-adjusted
hsa-miR-183-5p_TATGGCACTGGTAGAATTCACTG1	-0,175034768	3,607840844	0,766013353	0,988610748	LADA vs T1D Time-adjusted
hsa-miR-10a-5p_TACCCTGTAGATCCGAATT1	0,16267018	3,885245168	0,766090462	0,988610748	LADA vs T1D Time-adjusted
hsa-miR-1180-3p_TTTCCGGCTCGCGTGGGTGTGT1	-0,159010988	2,841243966	0,766432965	0,988610748	LADA vs T1D Time-adjusted
hsa-miR-197-3p_TTCACCACCTTCTCCACCCAGC1	-0,04803243	10,0726909	0,766445014	0,988610748	LADA vs T1D Time-adjusted
hsa-miR-423-3p_AGCTCGGTCTGAGGCCCCT1	0,092384753	6,073013782	0,767484992	0,988610748	LADA vs T1D Time-adjusted
hsa-miR-103a-3p_AGCAGCATTGTACAGGGCTATGA1	0,062368715	8,59094218	0,767719461	0,988610748	LADA vs T1D Time-adjusted
hsa-miR-486-5p_GTACTGAGCTGCCCCGAG1	-0,126094979	5,002102428	0,769373192	0,988610748	LADA vs T1D Time-adjusted
hsa-miR-148b-3p_TCAGTGCATCACAGAACTTTGT1	0,060273382	6,884283732	0,770608129	0,988610748	LADA vs T1D Time-adjusted
hsa-let-7c-5p_TGAGGTAGTAGGTTGTATGGT1	-0,101600608	4,826004461	0,77114138	0,988610748	LADA vs T1D Time-adjusted
hsa-miR-146a-5p_GAGAACTGAATTCCATGGGTT1	0,104425159	6,482797306	0,772945867	0,988711896	LADA vs T1D Time-adjusted
hsa-miR-451a_CCGTTACCATTACTGAGT1	-0,136224389	4,375477916	0,773735798	0,988711896	LADA vs T1D Time-adjusted
hsa-miR-144-3p_TACAGTATAGATGATGTAC1	-0,088955923	6,411669154	0,778791489	0,988711896	LADA vs T1D Time-adjusted
hsa-let-7f-5p_TGAGGTAGTAGATTGTATAGTTG1	-0,171381427	2,479162802	0,780085699	0,988711896	LADA vs T1D Time-adjusted
hsa-let-7i-5p_TGAGGTAGTAGTTTGTGCTGTT1	-0,044200337	10,46534644	0,780834964	0,988711896	LADA vs T1D Time-adjusted
hsa-let-7f-5p_GAGGTAGTAGATTGTATAGTT1	0,101075875	5,929108602	0,784516203	0,988711896	LADA vs T1D Time-adjusted
hsa-miR-103a-3p_AGCAGCATTGTACAGGGCTAT1	0,190459652	3,194453722	0,785308486	0,988711896	LADA vs T1D Time-adjusted
hsa-miR-423-3p_GCTCGGTCTGAGGCCCCTCAGT1	-0,087938058	6,437706495	0,785880413	0,988711896	LADA vs T1D Time-adjusted
hsa-miR-4433b-5p_ATGTCCCACCCCCACTCCTGT1	0,106939724	7,641633929	0,789048489	0,988711896	LADA vs T1D Time-adjusted
hsa-miR-22-3p_AAGCTGCCAGTTGAAGAACTGT1	-0,023575665	12,1121356	0,790572474	0,988711896	LADA vs T1D Time-adjusted
hsa-miR-423-5p_AGGGGCAGAGAGCGAGACT1	0,15331477	3,23300129	0,793688724	0,988711896	LADA vs T1D Time-adjusted
hsa-miR-25-3p_CATTGCACTTGTCTCGGTCTGA1	-0,041763587	11,97256306	0,79802761	0,988711896	LADA vs T1D Time-adjusted
hsa-miR-125b-5p_TCCCTGAGACCCTAACTTGT1	0,040067047	9,835959153	0,798042099	0,988711896	LADA vs T1D Time-adjusted
hsa-miR-132-3p_TAACAGTCTACAGCCATGGTCG1	0,146243874	2,429322611	0,798595988	0,988711896	LADA vs T1D Time-adjusted
hsa-miR-2110_TTGGGGAAACGGCCGCTGAG1	-0,142086844	2,146127874	0,799997627	0,988711896	LADA vs T1D Time-adjusted
hsa-miR-370-3p_GCCTGCTGGGGTGGAACCTGGT1	0,154330809	2,366107994	0,802481684	0,988711896	LADA vs T1D Time-adjusted
hsa-miR-23a-3p_ATCACATTGCCAGGGATTTCCA1	0,034716957	11,09453661	0,803720379	0,988711896	LADA vs T1D Time-adjusted
hsa-miR-148a-3p_TCAGTGCACTACAGAACTTTG1	-0,053158417	7,255293658	0,80540787	0,988711896	LADA vs T1D Time-adjusted
hsa-miR-483-5p_AAGACGGGAGGAAAGAAGGGA1	-0,12600384	4,912658385	0,812496452	0,988711896	LADA vs T1D Time-adjusted

hsa-miR-4433b-5p_TATGTCCCACCCCCACTCCTG1	0,156519859	2,841400639	0,813246839	0,988711896	LADA vs T1D Time-adjusted
hsa-miR-409-3p_CGAATGTTGCTCGGTGAACCCCTTT1	-0,150536019	2,311149768	0,814316782	0,988711896	LADA vs T1D Time-adjusted
hsa-miR-483-3p_TCACTCCTCTCCTCCCGTCT1	-0,146161343	2,17353874	0,815175473	0,988711896	LADA vs T1D Time-adjusted
hsa-miR-29a-3p_CTAGCACCATCTGAAATCGGTT1	-0,120675974	3,443400968	0,816352388	0,988711896	LADA vs T1D Time-adjusted
hsa-miR-501-3p_AATGCACCCGGGCAAGGATT1	-0,063267016	6,566869383	0,816864238	0,988711896	LADA vs T1D Time-adjusted
hsa-miR-92a-3p_ATTGCACTTGTCCCGGCCTGT1	-0,028242178	12,30748627	0,81769593	0,988711896	LADA vs T1D Time-adjusted
hsa-miR-103a-3p_AGCAGCATTGTACAGGGCT1	0,194263221	5,276241931	0,819093141	0,988711896	LADA vs T1D Time-adjusted
hsa-miR-148a-3p_TCAGTGCACTACAGAACTTTGT1	0,033618412	9,218931731	0,820625358	0,988711896	LADA vs T1D Time-adjusted
hsa-let-7f-5p_TGAGGTAGTAGATTGTATAGTTT1	0,063676012	8,014292524	0,820748963	0,988711896	LADA vs T1D Time-adjusted
hsa-let-7d-3p_TATACGACCTGCTGCCTTTCT1	-0,031424283	8,434108679	0,821114017	0,988711896	LADA vs T1D Time-adjusted
hsa-miR-1306-5p_CCACCTCCCCTGCAAACGTCCA1	0,135413571	3,045463797	0,821449427	0,988711896	LADA vs T1D Time-adjusted
hsa-miR-361-5p_TTATCAGAATCTCCAGGGGTACT1	0,12567856	2,680756048	0,821861451	0,988711896	LADA vs T1D Time-adjusted
hsa-miR-486-5p_TCCTGTACTGAGCTGCCCCG1	-0,039494429	14,12849188	0,821963206	0,988711896	LADA vs T1D Time-adjusted
hsa-miR-30d-5p_GTAAACATCCCCGACTGGAAGC1	-0,11261878	4,700243003	0,823528323	0,988711896	LADA vs T1D Time-adjusted
hsa-miR-451a_AACCGTTACCATTACTGAGT1	-0,039223387	10,87118749	0,823608098	0,988711896	LADA vs T1D Time-adjusted
hsa-miR-30d-5p_TGTAAACATCCCCGACTGGAAGCT1	-0,028652233	13,12660906	0,823737769	0,988711896	LADA vs T1D Time-adjusted
hsa-miR-335-5p_TCAAGAGCAATAACGAAAAATG1	-0,045343844	8,353751053	0,82399851	0,988711896	LADA vs T1D Time-adjusted
hsa-miR-16-2-3p_CCAATATTACTGTGCTGCTT1	0,088295414	4,45076213	0,825700102	0,988711896	LADA vs T1D Time-adjusted
hsa-miR-4433b-5p_ATGTCCCACCCCCACTCCTG1	-0,126036891	1,885888634	0,825748775	0,988711896	LADA vs T1D Time-adjusted
hsa-miR-26b-5p_TCAAGTAATTCAGGATAGGTT1	0,121968972	3,617629587	0,826779832	0,988711896	LADA vs T1D Time-adjusted
hsa-miR-92a-3p_TTGCACTTGTCCCGGCCTG1	0,106946168	4,224665197	0,827188642	0,988711896	LADA vs T1D Time-adjusted
hsa-miR-4433b-5p_TATGTCCCACCCCCACTCCTGT1	-0,113679347	5,223541258	0,830923224	0,988711896	LADA vs T1D Time-adjusted
hsa-miR-486-5p_CTGTACTGAGCTGCCCCG1	-0,122380956	3,36154621	0,831989079	0,988711896	LADA vs T1D Time-adjusted
hsa-miR-3173-5p_TGCCCTGCCTGTTTTCTCCTT1	0,124343186	2,961448405	0,832068957	0,988711896	LADA vs T1D Time-adjusted
hsa-let-7d-3p_ATACGACCTGCTGCCTTTCT1	0,120516446	2,005732528	0,835533001	0,988711896	LADA vs T1D Time-adjusted
hsa-miR-193a-5p_TGGGTCTTTGCGGGCGAGATGA1	0,036391868	8,658900701	0,835693539	0,988711896	LADA vs T1D Time-adjusted
hsa-miR-223-3p_TGTCAGTTTGTCAAATACCCCAA1	0,041652931	10,82178642	0,836603833	0,988711896	LADA vs T1D Time-adjusted
hsa-miR-223-3p_TGTCAGTTTGTCAAATACCC1	0,08934033	4,539198612	0,838634682	0,988711896	LADA vs T1D Time-adjusted
hsa-miR-193b-5p_CGGGGTTTTGAGGGCGAGATGA1	0,123668671	2,359786934	0,841637276	0,988711896	LADA vs T1D Time-adjusted
hsa-miR-192-5p_TGACCTATGAATTGACAGCCA1	-0,094223326	4,775035894	0,843732055	0,988711896	LADA vs T1D Time-adjusted
hsa-miR-423-3p_AAGCTCGGTCTGAGGCCCCTCAGT1	0,042204693	7,381534421	0,844093616	0,988711896	LADA vs T1D Time-adjusted
hsa-miR-191-5p_CAACGGAATCCCAAAAGCAGC1	0,040654007	8,118131191	0,844479405	0,988711896	LADA vs T1D Time-adjusted
hsa-let-7f-5p_TGAGGTAGTAGATTGTATAGT1	0,019432491	10,58330635	0,846175447	0,988711896	LADA vs T1D Time-adjusted
hsa-miR-224-5p_CAAGTCACTAGTGGTTCCGTTT1	0,113054575	2,890382654	0,84740676	0,988711896	LADA vs T1D Time-adjusted
hsa-miR-320b_AAAAGCTGGGTTGAGAGGGCAA1	0,102695454	2,061631881	0,847427431	0,988711896	LADA vs T1D Time-adjusted
hsa-miR-652-3p_AATGGCGCCACTAGGGTTG1	0,10505767	4,5440088	0,847790826	0,988711896	LADA vs T1D Time-adjusted
hsa-miR-183-5p_ATGGCACTGGTAGAATTCACT1	0,089752184	4,551574409	0,848471423	0,988711896	LADA vs T1D Time-adjusted
hsa-miR-425-5p_ATGACACGATCACTCCCGTTGA1	0,083155897	4,777521295	0,848688909	0,988711896	LADA vs T1D Time-adjusted
hsa-miR-361-3p_TCCCCCAGGTGTGATTCTGA1	-0,100833112	3,266035349	0,850530014	0,988711896	LADA vs T1D Time-adjusted
hsa-miR-92b-3p_TATTGCACTCGTCCCGGCCTCC1	0,091224837	4,339207773	0,850603967	0,988711896	LADA vs T1D Time-adjusted
hsa-miR-340-5p_TTATAAAGCAATGAGACTGAT1	-0,108037494	1,903930861	0,851428281	0,988711896	LADA vs T1D Time-adjusted
hsa-miR-21-5p_AGCTTATCAGACTGATGTTGAC1	0,113568596	3,019001358	0,851983145	0,988711896	LADA vs T1D Time-adjusted
hsa-miR-361-5p_TTATCAGAATCTCCAGGGGT1	-0,069591887	4,851644504	0,852815634	0,988711896	LADA vs T1D Time-adjusted
hsa-miR-484_TCAGGCTCAGTCCCCTCCCGAT1	-0,026152555	9,170955833	0,855075879	0,988711896	LADA vs T1D Time-adjusted
hsa-miR-486-5p_CTGTACTGAGCTGCCCCGA1	0,039269735	7,578479757	0,857396305	0,988711896	LADA vs T1D Time-adjusted
hsa-let-7d-3p_CTATACGACCTGCTGCCTT1	-0,078823597	4,663499808	0,85934459	0,988711896	LADA vs T1D Time-adjusted
hsa-miR-484_TCAGGCTCAGTCCCCTCCCGA1	-0,031132321	10,82608497	0,863911818	0,988711896	LADA vs T1D Time-adjusted
hsa-miR-485-5p_AGAGGCTGGCCGTGATGAATTC1	-0,106907559	2,188589843	0,865889398	0,988711896	LADA vs T1D Time-adjusted
hsa-miR-379-5p_TGGTAGACTATGGAACGTAGG1	-0,110051752	3,519537069	0,867482233	0,988711896	LADA vs T1D Time-adjusted
hsa-let-7d-5p_AGAGGTAGTAGGTTGCATAGTT1	0,034122255	9,029809844	0,867873994	0,988711896	LADA vs T1D Time-adjusted
hsa-miR-4433b-5p_ATGTCCCACCCCCACTCCTGTT1	0,070763323	8,088855349	0,868804469	0,988711896	LADA vs T1D Time-adjusted
hsa-miR-574-3p_CACGCTCATGCACACACCCA1	-0,091904602	3,290108497	0,868874282	0,988711896	LADA vs T1D Time-adjusted
hsa-miR-142-5p_CATAAAGTAGAAAGCACTACT1	0,084132922	3,738908441	0,869415544	0,988711896	LADA vs T1D Time-adjusted
hsa-miR-3613-5p_TGTTGTACTTTTTTTTTTGTT1	0,066026049	5,198438116	0,871767181	0,988711896	LADA vs T1D Time-adjusted
hsa-miR-192-5p_TGACCTATGAATTGACAGCC1	-0,049648433	6,46151299	0,87214334	0,988711896	LADA vs T1D Time-adjusted
hsa-miR-125b-5p_TCCCTGAGACCCTAACT1	-0,098455718	2,244613923	0,877400564	0,988711896	LADA vs T1D Time-adjusted
hsa-miR-21-5p_TAGCTTATCAGACTGATGTTGAC1	-0,036805696	10,35786285	0,87756127	0,988711896	LADA vs T1D Time-adjusted
hsa-miR-186-5p_CAAAGAATTCTCCTTTTGGGCTT1	0,053420096	6,241384756	0,877633664	0,988711896	LADA vs T1D Time-adjusted
hsa-miR-369-3p_AATAATACATGGTTGATCTTT1	-0,094928948	4,128023185	0,878450687	0,988711896	LADA vs T1D Time-adjusted
hsa-miR-423-3p_AGCTCGGTCTGAGGCCCCTCA1	0,051245725	6,070547639	0,879102945	0,988711896	LADA vs T1D Time-adjusted
hsa-let-7g-5p_TGAGGTAGTAGTTTGTACA1	-0,057463182	5,551691527	0,881668033	0,988711896	LADA vs T1D Time-adjusted

hsa-miR-92a-3p_TATTGCACTTGTCCCGGCCTGTG1	-0,026029302	7,12439601	0,882385391	0,988711896	LADA vs T1D Time-adjusted
hsa-miR-30d-5p_TGTAAACATCCCCGACTGGAA1	0,016815868	11,65154018	0,88265919	0,988711896	LADA vs T1D Time-adjusted
hsa-miR-143-3p_GAGATGAAGCACTGTAGCTC1	0,090981665	2,215337676	0,882928279	0,988711896	LADA vs T1D Time-adjusted
hsa-miR-25-3p_CATTGCACTTGTCTCGGTCT1	-0,02450558	10,31281854	0,883030912	0,988711896	LADA vs T1D Time-adjusted
hsa-miR-423-5p_TGAGGGGCAGAGAGCGAGAC1	-0,015829232	9,81018722	0,883178156	0,988711896	LADA vs T1D Time-adjusted
hsa-miR-15a-5p_TAGCAGCACATAATGGTTTGT1	0,050080242	6,55286919	0,883738442	0,988711896	LADA vs T1D Time-adjusted
hsa-miR-483-5p_AAGACGGGAGGAAAGAAGGGAG1	0,054988741	6,695020301	0,884472756	0,988711896	LADA vs T1D Time-adjusted
hsa-let-7g-5p_GAGGTAGTAGTTTGTACAGTT1	-0,085500997	4,325250462	0,884497855	0,988711896	LADA vs T1D Time-adjusted
hsa-miR-484_CAGGCTCAGTCCCCTCCCGA1	-0,085407899	3,800267749	0,884877441	0,988711896	LADA vs T1D Time-adjusted
hsa-miR-223-3p_TGTCAGTTTGTCAAATACCCCA1	0,026434007	10,34583114	0,885270288	0,988711896	LADA vs T1D Time-adjusted
hsa-miR-30c-5p_TGTAAACATCCTACACTCTC1	-0,084896378	2,337351039	0,887602692	0,988711896	LADA vs T1D Time-adjusted
hsa-miR-23a-3p_ATCACATTGCCAGGGATTTCC1	-0,014383353	11,11444486	0,888594146	0,988711896	LADA vs T1D Time-adjusted
hsa-miR-486-5p_CCTGTACTGAGCTGCCCCG1	-0,033187429	8,001774251	0,888961991	0,988711896	LADA vs T1D Time-adjusted
hsa-miR-30e-5p_TGTAAACATCCTTGACTGGAA1	-0,033012044	6,485643066	0,889976555	0,988711896	LADA vs T1D Time-adjusted
hsa-miR-345-5p_GCTGACTCCTAGTCCAGGGCTC1	0,07786138	3,791671294	0,891114067	0,988711896	LADA vs T1D Time-adjusted
hsa-let-7g-5p_TGAGGTAGTAGTTTGTACAGT1	0,013190095	10,71493101	0,892670397	0,988711896	LADA vs T1D Time-adjusted
hsa-miR-26a-5p_TTCAAGTAATCCAGGATAGG1	0,030843347	6,804916619	0,894325196	0,988711896	LADA vs T1D Time-adjusted
hsa-miR-4433b-5p_ATGTCCCACCCCCACTCCTGTTT1	-0,069075623	5,3584586	0,895939672	0,988711896	LADA vs T1D Time-adjusted
hsa-miR-221-3p_AGCTACATTGTCTGCTGGGTTT1	0,024294392	7,93162947	0,896526702	0,988711896	LADA vs T1D Time-adjusted
hsa-miR-340-5p_TTATAAAGCAATGAGACTGATT1	0,047566441	5,720859509	0,898281079	0,988711896	LADA vs T1D Time-adjusted
hsa-miR-221-3p_AGCTACATTGTCTGCTGGGT1	0,045292924	6,247180713	0,898328301	0,988711896	LADA vs T1D Time-adjusted
hsa-miR-16-5p_TTAGCAGCACGTAAATATTGGCG1	0,063882895	4,214912823	0,899955709	0,988711896	LADA vs T1D Time-adjusted
hsa-let-7d-5p_AGAGGTAGTAGGTTGCATAG1	-0,027106417	8,568558263	0,899965316	0,988711896	LADA vs T1D Time-adjusted
hsa-let-7b-5p_TGAGGTAGTAGGTTGTGTGG1	-0,013856459	12,46184935	0,906289949	0,992602451	LADA vs T1D Time-adjusted
hsa-miR-98-5p_TGAGGTAGTAAGTTGTATTGT1	-0,037399412	6,564741993	0,908563495	0,992602451	LADA vs T1D Time-adjusted
hsa-miR-451a_ACCGTTACCATTACTGAG1	0,058624856	3,859377434	0,909056058	0,992602451	LADA vs T1D Time-adjusted
hsa-miR-223-3p_TCAGTTTGTCAAATACCCCAA1	0,060225278	2,097761681	0,910555643	0,992602451	LADA vs T1D Time-adjusted
hsa-miR-143-3p_TGAGATGAAGCACTGTAGCT1	-0,019585037	9,047567862	0,910691786	0,992602451	LADA vs T1D Time-adjusted
hsa-miR-660-5p_TACCCATTGCATATCGGAGTTGT1	-0,057853767	4,013928314	0,911276051	0,992602451	LADA vs T1D Time-adjusted
hsa-miR-30d-5p_GTAAACATCCCCGACTGGAAGCT1	-0,031183878	6,871318415	0,912290749	0,992602451	LADA vs T1D Time-adjusted
hsa-miR-29a-3p_TAGCACCATCTGAAATCGGT1	-0,022184491	8,735508595	0,915045335	0,992984039	LADA vs T1D Time-adjusted
hsa-miR-335-5p_TCAAGAGCAATAACGAAAAAT1	-0,037190403	6,614922062	0,915944003	0,992984039	LADA vs T1D Time-adjusted
hsa-miR-145-3p_ATTCCTGGAAATACTGTTCTT1	-0,059916158	3,68129034	0,916757321	0,992984039	LADA vs T1D Time-adjusted
hsa-miR-30e-5p_TGTAAACATCCTTGACTGGAAG1	-0,020158541	7,594156561	0,918928182	0,992984039	LADA vs T1D Time-adjusted
hsa-miR-486-5p_ATCCTGTACTGAGCTGCCCCGAG1	-0,021678896	8,021810439	0,920771494	0,992984039	LADA vs T1D Time-adjusted
hsa-miR-183-5p_TATGGCACTGGTAGAATTC1	-0,054932258	3,259461606	0,921303962	0,992984039	LADA vs T1D Time-adjusted
hsa-miR-22-3p_AAGCTGCCAGTTGAAGAACT1	0,012916881	10,281857	0,924305747	0,992984039	LADA vs T1D Time-adjusted
hsa-miR-3173-5p_TGCCCTGCCTGTTTTCTCCTTT1	-0,047759715	4,474811161	0,924331808	0,992984039	LADA vs T1D Time-adjusted
hsa-miR-25-3p_ATTGCACTTGTCTCGGTCTG1	0,047708013	3,333905317	0,924958003	0,992984039	LADA vs T1D Time-adjusted
hsa-miR-532-5p_CATGCCTTGAGTGTAGGACCGT1	0,043857738	4,686169319	0,925194989	0,992984039	LADA vs T1D Time-adjusted
hsa-miR-206_TGGAATGTAAGGAAGTGTGTGG1	0,055755645	2,186074312	0,929595767	0,993015577	LADA vs T1D Time-adjusted
hsa-let-7b-5p_GAGGTAGTAGGTTGTGTGGT1	0,039843532	4,880278721	0,930677684	0,993015577	LADA vs T1D Time-adjusted
hsa-miR-130b-5p_ACTCTTTCCCTGTTGCACTACT1	-0,033036589	5,624339176	0,931283535	0,993015577	LADA vs T1D Time-adjusted
hsa-miR-223-5p_CGTGTATTTGACAAGCTGAGTT1	0,043058395	3,834084658	0,931830462	0,993015577	LADA vs T1D Time-adjusted
hsa-miR-30d-5p_TGTAAACATCCCCGACTGGAAGC1	-0,008739091	11,545304	0,932176395	0,993015577	LADA vs T1D Time-adjusted
hsa-miR-16-5p_GCAGCACGTAAATATTGGCG1	0,041303413	3,590895157	0,932756731	0,993015577	LADA vs T1D Time-adjusted
hsa-miR-32-5p_TATTGCACATTACTAAGTTGC1	-0,047475703	2,139133623	0,934949374	0,99360492	LADA vs T1D Time-adjusted
hsa-miR-146b-5p_TGAGAACTGAATTCCATAGGCT1	0,022394155	6,379112274	0,936801165	0,99360492	LADA vs T1D Time-adjusted
hsa-miR-654-3p_TATGTCTGCTGACCATCACC1	-0,04993764	2,446876993	0,937078723	0,99360492	LADA vs T1D Time-adjusted
hsa-miR-501-3p_AATGCACCCGGGCAAGGATTC1	0,04062842	3,029908341	0,943044842	0,998223531	LADA vs T1D Time-adjusted
hsa-miR-30c-5p_TGTAAACATCCTACACTCTCAG1	-0,032330414	4,704062458	0,944175478	0,998223531	LADA vs T1D Time-adjusted
hsa-miR-1307-3p_CTCGGCGTGGCGTCGGTCGTGG1	-0,039188406	2,234349501	0,948114318	0,998223531	LADA vs T1D Time-adjusted
hsa-miR-21-5p_AGCTTATCAGACTGATGTTG1	-0,023372175	5,242282712	0,948330078	0,998223531	LADA vs T1D Time-adjusted
hsa-miR-1908-5p_CGGCGGGGACGGCGATTGGT1	0,038205141	2,728932328	0,951918841	0,998223531	LADA vs T1D Time-adjusted
hsa-miR-21-5p_TAGCTTATCAGACTGATGTT1	0,01202863	7,181930249	0,953216712	0,998223531	LADA vs T1D Time-adjusted
hsa-miR-144-3p_TACAGTATAGATGATGTACT1	0,027583392	4,591697634	0,95373833	0,998223531	LADA vs T1D Time-adjusted
hsa-miR-375-3p_TTTGTTCGTTCGGCTCGCG1	-0,022802893	5,940750727	0,955961864	0,998223531	LADA vs T1D Time-adjusted
hsa-miR-451a_AACCGTTACCATTACTGAG1	0,011094848	8,086006518	0,956607089	0,998223531	LADA vs T1D Time-adjusted
hsa-miR-182-5p_TTTGGCAATGGTAGAACTCACACT1	0,01352754	7,646583987	0,956837682	0,998223531	LADA vs T1D Time-adjusted
hsa-miR-30a-5p_GTAAACATCCTCGACTGGAAGCT1	0,027461004	4,346285408	0,957167063	0,998223531	LADA vs T1D Time-adjusted
hsa-miR-486-5p_TCCTGTACTGAGCTGCCCCGA1	-0,008086255	17,02608896	0,960719749	0,998223531	LADA vs T1D Time-adjusted

hsa-miR-98-5p_TGAGGTAGTAAGTTGTATTGTT1	0,012284066	7,833511387	0,963881737	0,998223531	LADA vs T1D Time-adjusted
hsa-let-7f-5p_TGAGGTAGTAGATTGTATAGTT1	0,007013772	12,47888054	0,964435042	0,998223531	LADA vs T1D Time-adjusted
hsa-miR-363-3p_AATTGCACGGTATCCATCTG1	0,00828006	8,30423721	0,964963036	0,998223531	LADA vs T1D Time-adjusted
hsa-miR-93-5p_CAAAGTGCTGTTCGTGCAGGTA1	0,014651707	5,408235597	0,966080227	0,998223531	LADA vs T1D Time-adjusted
hsa-miR-191-5p_AACGGAATCCCAAAAGCAGCT1	0,008429985	6,832593963	0,973792112	0,998223531	LADA vs T1D Time-adjusted
hsa-miR-92a-3p_TATTGCACTTGTCCCGGC1	-0,00892832	6,622543799	0,973984731	0,998223531	LADA vs T1D Time-adjusted
hsa-miR-21-5p_TAGCTTATCAGACTGATG1	-0,017060062	2,041272314	0,975845575	0,998223531	LADA vs T1D Time-adjusted
hsa-miR-128-3p_TCACAGTGAACCGGTCTCT1	0,006153057	7,735806596	0,976560964	0,998223531	LADA vs T1D Time-adjusted
hsa-miR-2110_TTGGGGAAACGGCCGCTGAGTGA1	-0,00941006	6,031153966	0,978207669	0,998223531	LADA vs T1D Time-adjusted
hsa-miR-10b-5p_TACCCTGTAGAACCGAAT1	0,015989424	2,102144035	0,978492074	0,998223531	LADA vs T1D Time-adjusted
hsa-miR-339-3p_TGAGCGCCTCGACGACAGAGCCG1	-0,013452759	4,074916381	0,981094949	0,998223531	LADA vs T1D Time-adjusted
hsa-miR-143-3p_TGAGATGAAGCACTGTAGCTC1	-0,00350327	9,81453422	0,98264928	0,998223531	LADA vs T1D Time-adjusted
hsa-miR-501-3p_AATGCACCCGGGCAAGGATTCT1	-0,003547386	7,8797274	0,98333479	0,998223531	LADA vs T1D Time-adjusted
hsa-miR-425-5p_AATGACACGATCACTCCCGT1	-0,005800907	6,660694598	0,98360619	0,998223531	LADA vs T1D Time-adjusted
hsa-miR-486-5p_TCCTGTACTGAGCTGCCC1	0,007039381	3,876048404	0,985900425	0,998223531	LADA vs T1D Time-adjusted
hsa-miR-1180-3p_TTTCCGGCTCGCGTGGGTGT1	-0,008519508	4,031270991	0,9863685	0,998223531	LADA vs T1D Time-adjusted
hsa-miR-222-3p_AGCTACATCTGGCTACTGGGTCTCT1	0,004439933	7,874596057	0,986943958	0,998223531	LADA vs T1D Time-adjusted
hsa-miR-125b-5p_CCCTGAGACCCTAACTTGT1	-0,009313107	2,224797164	0,987189791	0,998223531	LADA vs T1D Time-adjusted
hsa-miR-181a-2-3p_ACCACTGACCGTTGACTGTAC1	0,007987232	3,539126124	0,988358693	0,998223531	LADA vs T1D Time-adjusted
hsa-miR-483-5p_AAGACGGGAGGAAAGAAGGGAGT1	-0,008448689	4,22603991	0,988454816	0,998223531	LADA vs T1D Time-adjusted
hsa-miR-30a-5p_TGTAAACATCCTCGACTGG1	0,00631986	4,667345631	0,989483671	0,998223531	LADA vs T1D Time-adjusted
hsa-miR-409-3p_CGAATGTTGCTCGGTGAACCCCTT1	-0,008154197	3,582607068	0,98948622	0,998223531	LADA vs T1D Time-adjusted
hsa-miR-181a-5p_ACATTCAACGCTGTCGGTGA1	-0,006612509	3,835029228	0,989829993	0,998223531	LADA vs T1D Time-adjusted
hsa-miR-423-3p_AAGCTCGGTCTGAGGCCCCTCAG1	-0,003709824	3,379819075	0,994161811	0,998223531	LADA vs T1D Time-adjusted
hsa-miR-374a-5p_TTATAATACAACCTGATAAGTG1	-0,003171931	4,298678316	0,994264666	0,998223531	LADA vs T1D Time-adjusted
hsa-miR-139-3p_TGGAGACGCGGCCCTGTTGGAGT1	-0,00370183	3,616305041	0,994886014	0,998223531	LADA vs T1D Time-adjusted
hsa-miR-183-5p_TATGGCACTGGTAGAATTCACT1	0,002208229	5,890825812	0,995303911	0,998223531	LADA vs T1D Time-adjusted
hsa-miR-181a-5p_AACATTCAACGCTGTCGGTG1	0,001481246	6,965832799	0,99557097	0,998223531	LADA vs T1D Time-adjusted
hsa-miR-28-5p_AAGGAGCTCACAGTCTATTGA1	0,003292614	3,153941235	0,995684378	0,998223531	LADA vs T1D Time-adjusted
hsa-miR-342-5p_AGGGGTGCTATCTGTGATTGA1	-0,002474916	4,01644439	0,996686643	0,998223531	LADA vs T1D Time-adjusted
hsa-miR-451a_AAACCGTTACCATTACTGAGTTT1	-0,000742461	9,000647073	0,996732313	0,998223531	LADA vs T1D Time-adjusted
hsa-miR-186-5p_AAAGAATTCTCCTTTTGGGCT1	-0,002099982	3,93470427	0,996961554	0,998223531	LADA vs T1D Time-adjusted
hsa-let-7d-5p_AGAGGTAGTAGGTTGCATAGTTT1	0,000167078	5,258609386	0,999747824	0,999747824	LADA vs T1D Time-adjusted
hsa-miR-140-3p_TACCACAGGGTAGAACCACGGACA2	-1,989068763	3,592791188	1,84454E-05	0,010536836	LADA vs T2D Time-adjusted
hsa-miR-16-5p_TAGCAGCACGTAAATATTGGC2	-0,546222859	9,966980138	3,74582E-05	0,010536836	LADA vs T2D Time-adjusted
hsa-miR-106b-3p_CCGCACTGTGGGTACTTGCT2	-1,408633302	6,292504346	5,27654E-05	0,010536836	LADA vs T2D Time-adjusted
hsa-miR-106b-3p_CCGCACTGTGGGTACTTGCTG2	-1,835751908	4,149873679	5,32836E-05	0,010536836	LADA vs T2D Time-adjusted
hsa-miR-140-3p_ACCACAGGGTAGAACCACGGACA2	-1,703561129	4,273604017	0,000131877	0,020862886	LADA vs T2D Time-adjusted
hsa-miR-16-2-3p_ACCAATATTACTGTGCTGCT2	-1,544804644	4,640125469	0,00018887	0,02219786	LADA vs T2D Time-adjusted
hsa-miR-140-3p_TACCACAGGGTAGAACCACGGA2	-0,789363563	7,862508769	0,000223907	0,02219786	LADA vs T2D Time-adjusted
hsa-miR-487b-3p_AATCGTACAGGGTCATCCACTT2	-2,051830757	3,93004941	0,000231769	0,02219786	LADA vs T2D Time-adjusted
hsa-let-7a-5p_TGAGGTAGTAGGTTGTATA2	-1,266850435	5,691720869	0,000256993	0,02219786	LADA vs T2D Time-adjusted
hsa-miR-361-3p_TCCCCCAGGTGTGATTCTGATTT2	-1,657428131	4,004503719	0,00028063	0,02219786	LADA vs T2D Time-adjusted
hsa-miR-93-5p_CAAAGTGCTGTTCGTGCAGGTAG2	-0,426357148	9,966857204	0,000512408	0,036846791	LADA vs T2D Time-adjusted
hsa-miR-25-3p_ATTGCACTTGTCTCGGTCTG2	-1,69922113	3,333905317	0,000657759	0,041660356	LADA vs T2D Time-adjusted
hsa-miR-22-3p_AGCTGCCAGTTGAAGAACTGT2	-1,221607808	5,657855582	0,000721397	0,041660356	LADA vs T2D Time-adjusted
hsa-miR-181b-5p_AACATTCATTGCTGTCGGTGGGT2	-1,754574837	3,495629927	0,000737351	0,041660356	LADA vs T2D Time-adjusted
hsa-miR-484_CAGGCTCAGTCCCCTCCCGAT2	-1,75184783	2,348599265	0,000886825	0,046765226	LADA vs T2D Time-adjusted
hsa-miR-140-3p_ACCACAGGGTAGAACCACGGA2	-1,005079648	6,607249039	0,001032585	0,051048423	LADA vs T2D Time-adjusted
hsa-miR-451a_AACCGTTACCATTACTGAGTT2	-1,060187788	6,235439111	0,001199489	0,053469965	LADA vs T2D Time-adjusted
hsa-let-7c-5p_TGAGGTAGTAGGTTGTATGG2	-1,185489679	3,127922361	0,001216763	0,053469965	LADA vs T2D Time-adjusted
hsa-miR-93-5p_CAAAGTGCTGTTCGTGCAGGTA2	-1,081129384	5,408235597	0,001287188	0,05358767	LADA vs T2D Time-adjusted
hsa-miR-142-5p_CCCATAAAGTAGAAAGCA2	-1,523396116	2,402819725	0,001647714	0,065167075	LADA vs T2D Time-adjusted
hsa-miR-140-3p_TACCACAGGGTAGAACCACGG2	-1,026145761	5,398078089	0,00178665	0,067297132	LADA vs T2D Time-adjusted
hsa-miR-1180-3p_TTTCCGGCTCGCGTGGGTGTGT2	-1,62427189	2,841243966	0,002052544	0,069665437	LADA vs T2D Time-adjusted
hsa-miR-140-3p_ACCACAGGGTAGAACCACGGAC2	-0,982864873	5,842587001	0,002136916	0,069665437	LADA vs T2D Time-adjusted
hsa-miR-652-3p_AATGGCGCCACTAGGGTTGTG2	-0,876077815	6,436258449	0,002146871	0,069665437	LADA vs T2D Time-adjusted
hsa-miR-451a_AAACCGTTACCATTACTGAGTT2	-0,494837876	12,10291266	0,002245441	0,069665437	LADA vs T2D Time-adjusted
hsa-miR-320a-3p_AAAGCTGGGTTGAGAGGGCGAA2	-1,423736025	4,245030119	0,002289888	0,069665437	LADA vs T2D Time-adjusted
hsa-miR-122-5p_TGGAGTGTGACAATGGTGTTTG2	-0,819238579	11,7376523	0,002448597	0,071734816	LADA vs T2D Time-adjusted
hsa-miR-320b_AAAAGCTGGGTTGAGAGGGCA2	-1,522899025	2,615161599	0,002603467	0,072302326	LADA vs T2D Time-adjusted

hsa-miR-122-5p_GGAGTGTGACAATGGTGTTTG2	-1,337594044	5,619888581	0,0026657	0,072302326	LADA vs T2D Time-adjusted
hsa-miR-10b-5p_TACCCTGTAGAACCGAATTTG2	0,386337857	9,829860922	0,002742187	0,072302326	LADA vs T2D Time-adjusted
hsa-miR-423-3p_AAGCTCGGTCTGAGGCCCCTCAG2	-1,476925325	3,379819075	0,002993877	0,074776609	LADA vs T2D Time-adjusted
hsa-let-7i-5p_TGAGGTAGTAGTTTGTGCT2	-0,576478701	7,631066138	0,003195803	0,074776609	LADA vs T2D Time-adjusted
hsa-miR-425-5p_ATGACACGATCACTCCCGTTG2	-1,658244348	2,364893848	0,00320908	0,074776609	LADA vs T2D Time-adjusted
hsa-miR-182-5p_TTTGGCAATGGTAGAACT2	-1,705644835	3,433367687	0,003214165	0,074776609	LADA vs T2D Time-adjusted
hsa-let-7d-3p_CTATACGACCTGCTGCCTTTC2	0,320717889	10,31942736	0,003341903	0,075527006	LADA vs T2D Time-adjusted
hsa-miR-186-5p_CAAAGAATTCTCCTTTTGGGC2	-0,933547579	6,378401215	0,003990224	0,087674099	LADA vs T2D Time-adjusted
hsa-miR-26b-5p_TTCAAGTAATTCAGGATAGG2	-1,440243947	3,267725483	0,004612826	0,098614733	LADA vs T2D Time-adjusted
hsa-miR-16-5p_TAGCAGCACGTAAATATTG2	-0,924222019	5,552847943	0,005186333	0,107957624	LADA vs T2D Time-adjusted
hsa-miR-1180-3p_TTTCCGGCTCGCGTGGGTGT2	-1,353495636	4,031270991	0,005788857	0,114730693	LADA vs T2D Time-adjusted
hsa-miR-361-5p_TTATCAGAATCTCCAGGGGTA2	-1,478284289	3,316391684	0,005801805	0,114730693	LADA vs T2D Time-adjusted
hsa-miR-25-3p_CATTGCACTTGTCTCGGTCTGA2	-0,434126458	11,97256306	0,006122406	0,116459997	LADA vs T2D Time-adjusted
hsa-miR-30d-5p_GTAAACATCCCCGACTGGAAGCT2	-0,750965182	6,871318415	0,006282826	0,116459997	LADA vs T2D Time-adjusted
hsa-miR-425-5p_AATGACACGATCACTCCCGTTGA2	-0,288534657	10,33255905	0,006454274	0,116459997	LADA vs T2D Time-adjusted
hsa-miR-484_CAGGCTCAGTCCCCTCCCGA2	-1,574239162	3,800267749	0,006478179	0,116459997	LADA vs T2D Time-adjusted
hsa-miR-6803-3p_TCCCTCGCCTTCTCACCCTCAGT2	1,659697649	2,013232161	0,006639293	0,116704017	LADA vs T2D Time-adjusted
hsa-miR-146b-5p_TGAGAACTGAATTCCATAGGCTG2	-0,98131608	5,796387655	0,006864405	0,118037927	LADA vs T2D Time-adjusted
hsa-miR-93-5p_CAAAGTGCTGTTCGTGCAGGT2	-0,892677718	5,072528945	0,008924135	0,143941455	LADA vs T2D Time-adjusted
hsa-miR-197-3p_TTCACCACCTTCTCCACCCAG2	0,498659378	8,701233583	0,009085216	0,143941455	LADA vs T2D Time-adjusted
hsa-miR-29a-3p_CTAGCACCATCTGAAATCGG2	-1,332785144	3,1053029	0,009109583	0,143941455	LADA vs T2D Time-adjusted
hsa-miR-20a-5p_TAAAGTGCTTATAGTGCAGGTAG2	-1,192793493	3,856097276	0,009615516	0,143941455	LADA vs T2D Time-adjusted
hsa-miR-18a-3p_ACTGCCCTAAGTGCTCCTTCTG2	-1,386614737	2,147867886	0,009729668	0,143941455	LADA vs T2D Time-adjusted
hsa-miR-194-5p_TGTAACAGCAACTCCATGTGGAA2	-1,375184387	2,71610017	0,009740605	0,143941455	LADA vs T2D Time-adjusted
hsa-miR-25-3p_CATTGCACTTGTCTCGGTCTG2	-0,509065351	9,96694873	0,009909399	0,143941455	LADA vs T2D Time-adjusted
hsa-miR-181a-2-3p_ACCACTGACCGTTGACTGT2	-1,43048075	2,626525168	0,009915165	0,143941455	LADA vs T2D Time-adjusted
hsa-miR-183-5p_ATGGCACTGGTAGAATTCACT2	-1,176582712	4,551574409	0,010008571	0,143941455	LADA vs T2D Time-adjusted
hsa-miR-7-5p_TGGAAGACTAGTGATTTTGTTG2	-1,227127417	3,867797174	0,010380478	0,145551965	LADA vs T2D Time-adjusted
hsa-miR-192-5p_TGACCTATGAATTGACAGCCAG2	-1,294506461	3,649860914	0,010488574	0,145551965	LADA vs T2D Time-adjusted
hsa-miR-30d-5p_TGTAAACATCCCCGACTGGAAGC2	-0,254633811	11,545304	0,010865068	0,148177046	LADA vs T2D Time-adjusted
hsa-let-7b-5p_GAGGTAGTAGGTTGTGTGGTT2	-0,735668946	6,256027764	0,011205847	0,150063025	LADA vs T2D Time-adjusted
hsa-miR-181a-2-3p_ACCACTGACCGTTGACTGTAC2	-1,352665508	3,539126124	0,011653036	0,150063025	LADA vs T2D Time-adjusted
hsa-miR-363-3p_ATTGCACGGTATCCATCTGT2	-1,169633738	4,449987277	0,01169431	0,150063025	LADA vs T2D Time-adjusted
hsa-miR-185-5p_TGGAGAGAAAGGCAGTTCCTGA2	-0,348979781	8,779876669	0,011762209	0,150063025	LADA vs T2D Time-adjusted
hsa-miR-363-3p_AATTGCACGGTATCCATCTG2	-0,456881224	8,30423721	0,012601515	0,156033606	LADA vs T2D Time-adjusted
hsa-miR-186-5p_CAAAGAATTCTCCTTTTGGGCT2	-0,360833461	8,984782262	0,012624717	0,156033606	LADA vs T2D Time-adjusted
hsa-miR-24-3p_TGGCTCAGTTCAGCAGGAACA2	-0,277487859	10,02516895	0,01321308	0,158706549	LADA vs T2D Time-adjusted
hsa-miR-10b-5p_TACCCTGTAGAACCGAATTTGT2	0,286464646	11,1550449	0,0132464	0,158706549	LADA vs T2D Time-adjusted
hsa-miR-484_TCAGGCTCAGTCCCCTCCCGATA2	-1,136013196	4,542352365	0,013442906	0,158706549	LADA vs T2D Time-adjusted
hsa-let-7b-5p_TGAGGTAGTAGGTTGTGTGGT2	-0,263131966	11,86683629	0,01386794	0,161316776	LADA vs T2D Time-adjusted
hsa-miR-361-5p_TTATCAGAATCTCCAGGGGT2	-0,900229935	4,851644504	0,014648817	0,166219525	LADA vs T2D Time-adjusted
hsa-miR-1294_TGTGAGGTTGGCATTGTTGT2	-1,351350083	2,446198713	0,015104136	0,166219525	LADA vs T2D Time-adjusted
hsa-let-7b-5p_GAGGTAGTAGGTTGTGTGGT2	-1,079574735	4,880278721	0,015324665	0,166219525	LADA vs T2D Time-adjusted
hsa-let-7a-5p_TGAGGTAGTAGGTTGTATAG2	-0,29304467	12,03070271	0,015356829	0,166219525	LADA vs T2D Time-adjusted
hsa-miR-24-3p_CTGGCTCAGTTCAGCAGGAACAG2	-1,349496686	2,043456364	0,01547372	0,166219525	LADA vs T2D Time-adjusted
hsa-miR-486-5p_GTACTGAGCTGCCCCGAG2	-1,009744942	5,002102428	0,015550246	0,166219525	LADA vs T2D Time-adjusted
hsa-miR-484_TCAGGCTCAGTCCCCTCCCG2	-0,634881165	7,776089511	0,015881708	0,166550757	LADA vs T2D Time-adjusted
hsa-miR-30e-5p_TGTAAACATCCTTGACTGGAAGCT2	-0,184105659	11,68636005	0,016237014	0,166550757	LADA vs T2D Time-adjusted
hsa-miR-183-5p_TATGGCACTGGTAGAATTC2	-1,302167275	3,259461606	0,016430941	0,166550757	LADA vs T2D Time-adjusted
hsa-let-7g-5p_TGAGGTAGTAGTTTGTACA2	-0,907686852	5,551691527	0,016504647	0,166550757	LADA vs T2D Time-adjusted
hsa-let-7g-5p_TGAGGTAGTAGTTTGTACAG2	-0,34718936	9,388462879	0,016752432	0,166550757	LADA vs T2D Time-adjusted
hsa-miR-363-3p_AATTGCACGGTATCCATCT2	-1,1686457	4,448241713	0,016844577	0,166550757	LADA vs T2D Time-adjusted
hsa-miR-484_TCAGGCTCAGTCCCCTCCCGAT2	-0,330570561	9,170955833	0,017469615	0,170598338	LADA vs T2D Time-adjusted
hsa-miR-186-5p_CAAAGAATTCTCCTTTTGGGCTTT2	-1,22497007	3,921463053	0,020211452	0,19301361	LADA vs T2D Time-adjusted
hsa-let-7b-5p_GAGGTAGTAGGTTGTGTGGTTT2	-1,19524191	3,820264571	0,020253008	0,19301361	LADA vs T2D Time-adjusted
hsa-miR-21-3p_CAACACCAGTCGATGGGCTGT2	-1,194796435	2,288436309	0,021108268	0,198769521	LADA vs T2D Time-adjusted
hsa-miR-183-5p_TATGGCACTGGTAGAATTCAC2	-1,213226916	2,19049992	0,021504294	0,199086088	LADA vs T2D Time-adjusted
hsa-miR-181a-5p_ACATTCAACGCTGTCGGTGA2	-1,16486666	3,835029228	0,021645264	0,199086088	LADA vs T2D Time-adjusted
hsa-miR-193a-5p_TGGGTCTTTGCGGGCGAGA2	1,350350641	3,485272507	0,023196524	0,210901733	LADA vs T2D Time-adjusted
hsa-miR-183-5p_TATGGCACTGGTAGAATTCACT2	-0,819998415	5,890825812	0,023825757	0,211719214	LADA vs T2D Time-adjusted
hsa-miR-486-3p_CGGGGCAGCTCAGTACAGGA2	-0,709420872	6,565196714	0,023962723	0,211719214	LADA vs T2D Time-adjusted

hsa-miR-451a_CGTTACCATTACTGAGT2	-1,056483446	4,231569487	0,024089418	0,211719214	LADA vs T2D Time-adjusted
hsa-let-7g-5p_TGAGGTAGTAGTTTGTACAGTTT2	-0,599198956	6,732316769	0,02446179	0,212071052	LADA vs T2D Time-adjusted
hsa-miR-192-5p_TGACCTATGAATTGACAGCCAGT2	-1,114010059	3,442809963	0,02466566	0,212071052	LADA vs T2D Time-adjusted
hsa-miR-24-3p_TGGCTCAGTTCAGCAGGAAC2	0,243785923	10,09466061	0,02499661	0,212605575	LADA vs T2D Time-adjusted
hsa-let-7b-5p_TGAGGTAGTAGGTTGTGTG2	-0,548993095	6,901751547	0,025442664	0,214097307	LADA vs T2D Time-adjusted
hsa-miR-185-5p_TGGAGAGAAAGGCAGTTCCTG2	-1,066608631	4,445039784	0,026239303	0,216308028	LADA vs T2D Time-adjusted
hsa-miR-25-3p_ATTGCACTTGTCTCGGTCT2	-1,116158859	3,534900996	0,026467283	0,216308028	LADA vs T2D Time-adjusted
hsa-miR-182-5p_TTTGGCAATGGTAGAACTCACA2	-0,555277672	6,980287172	0,026751181	0,216308028	LADA vs T2D Time-adjusted
hsa-miR-4732-5p_TGTAGAGCAGGGAGCAGGAAGCT2	-0,698665543	6,317387172	0,026985694	0,216308028	LADA vs T2D Time-adjusted
hsa-miR-28-3p_CACTAGATTGTGAGCTCCTGGA2	-0,573755157	6,790249103	0,027076971	0,216308028	LADA vs T2D Time-adjusted
hsa-miR-451a_ACCGTTACCATTACTGAGTT2	-1,232420543	2,058885942	0,027346148	0,216308028	LADA vs T2D Time-adjusted
hsa-miR-24-3p_GGCTCAGTTCAGCAGGAACA2	-1,209779295	2,68639799	0,028188319	0,220761983	LADA vs T2D Time-adjusted
hsa-miR-574-3p_CACGCTCATGCACACACCCAC2	-1,079192854	3,990306713	0,029185401	0,226329924	LADA vs T2D Time-adjusted
hsa-miR-486-5p_TGTACTGAGCTGCCCCGAG2	-0,89323224	5,107200977	0,029574538	0,22712097	LADA vs T2D Time-adjusted
hsa-miR-148b-3p_TCAGTGCATCACAGAACTTTG2	-0,864938086	4,984825627	0,032641591	0,247480666	LADA vs T2D Time-adjusted
hsa-miR-425-5p_AATGACACGATCACTCCCGTTG2	-0,292306772	9,327235486	0,032851416	0,247480666	LADA vs T2D Time-adjusted
hsa-miR-223-3p_TGTCAGTTTGTCAAATACCCCAA2	-0,415552351	10,82178642	0,033167096	0,247501634	LADA vs T2D Time-adjusted
hsa-miR-140-3p_ACCACAGGGTAGAACCACGG2	-1,076382312	3,499713471	0,034822998	0,253396491	LADA vs T2D Time-adjusted
hsa-miR-92b-3p_TATTGCACTCGTCCCGGCCTCC2	-0,990610209	4,339207773	0,03518561	0,253396491	LADA vs T2D Time-adjusted
hsa-miR-532-5p_CATGCCTTGAGTGTAGGACCGT2	-0,954560892	4,686169319	0,035214665	0,253396491	LADA vs T2D Time-adjusted
hsa-miR-423-3p_AAGCTCGGTCTGAGGCCCCTCA2	-1,10874136	2,829290184	0,03523845	0,253396491	LADA vs T2D Time-adjusted
hsa-miR-223-3p_TGTCAGTTTGTCAAATACC2	-0,803855179	5,277889688	0,035915677	0,255939647	LADA vs T2D Time-adjusted
hsa-miR-339-3p_TGAGCGCCTCGACGACAGAG2	-0,844565479	5,283296909	0,036865757	0,258616187	LADA vs T2D Time-adjusted
hsa-miR-22-3p_AAGCTGCCAGTTGAAGAACTG2	-0,382951495	9,199421356	0,03694517	0,258616187	LADA vs T2D Time-adjusted
hsa-miR-451a_ACCGTTACCATTACTGAGT2	-0,56537099	7,438165305	0,037791898	0,261786132	LADA vs T2D Time-adjusted
hsa-miR-126-3p_CGTACCGTGAGTAATAATGC2	-0,824593461	4,480402803	0,038059931	0,261786132	LADA vs T2D Time-adjusted
hsa-miR-25-3p_CATTGCACTTGTCTCGGTC2	-0,720753403	5,824634034	0,038404191	0,261876854	LADA vs T2D Time-adjusted
hsa-miR-423-3p_AGCTCGGTCTGAGGCCCCTCAG2	-0,275140724	8,394585646	0,040608206	0,270615308	LADA vs T2D Time-adjusted
hsa-miR-2110_TTGGGGAAACGGCCGCTGAGTG2	-1,15534168	3,022786946	0,040682933	0,270615308	LADA vs T2D Time-adjusted
hsa-let-7g-5p_TGAGGTAGTAGTTTGTACAGT2	-0,193772646	10,71493101	0,041003173	0,270615308	LADA vs T2D Time-adjusted
hsa-miR-92b-3p_TATTGCACTCGTCCCGGCCTC2	-1,011703416	3,950927699	0,041054155	0,270615308	LADA vs T2D Time-adjusted
hsa-miR-584-5p_TTATGGTTTGCCTGGGACTG2	-1,108932029	2,451626756	0,042168027	0,275660407	LADA vs T2D Time-adjusted
hsa-miR-143-3p_TGAGATGAAGCACTGTAGCTC2	-0,31614298	9,81453422	0,043230889	0,280292076	LADA vs T2D Time-adjusted
hsa-miR-486-5p_GTACTGAGCTGCCCCGA2	-0,831662004	4,600446211	0,04379519	0,281642241	LADA vs T2D Time-adjusted
hsa-miR-16-2-3p_ACCAATATTACTGTGCTGCTTT2	-0,348832225	7,823388697	0,044583429	0,282759181	LADA vs T2D Time-adjusted
hsa-let-7g-5p_GAGGTAGTAGTTTGTACAGT2	-1,116502876	3,503297517	0,044683815	0,282759181	LADA vs T2D Time-adjusted
hsa-miR-494-3p_TGAAACATACACGGGAAACCTCT2	-1,082176594	4,130022225	0,045083754	0,28302579	LADA vs T2D Time-adjusted
hsa-miR-425-5p_AATGACACGATCACTCCCG2	-0,93669755	4,588646685	0,045506894	0,283432705	LADA vs T2D Time-adjusted
hsa-miR-409-3p_GAATGTTGCTCGGTGAACCCCT2	-0,768048765	7,83201482	0,04639689	0,286718283	LADA vs T2D Time-adjusted
hsa-miR-181a-5p_AACATTCAACGCTGTCGGTGA2	-0,22722831	10,01113916	0,047907514	0,293758478	LADA vs T2D Time-adjusted
hsa-miR-16-5p_TAGCAGCACGTAAATATTGGCGT2	-0,395480359	7,887777382	0,050510587	0,307337495	LADA vs T2D Time-adjusted
hsa-miR-10a-5p_TACCCTGTAGATCCGAATTTGTGT2	-1,053442168	2,457813121	0,051270221	0,308306979	LADA vs T2D Time-adjusted
hsa-miR-25-3p_CATTGCACTTGTCTCGGT2	-0,901663664	3,82970246	0,051758141	0,308306979	LADA vs T2D Time-adjusted
hsa-miR-145-5p_GTCCAGTTTTCCCAGGAATCCCT2	1,128851557	1,974319363	0,051839227	0,308306979	LADA vs T2D Time-adjusted
hsa-miR-101-3p_GTACAGTACTGTGATAACTG2	-0,73490049	5,567307108	0,052710527	0,311149455	LADA vs T2D Time-adjusted
hsa-miR-30d-5p_TGTAAACATCCCCGACTGGAA2	-0,212583765	11,65154018	0,053946995	0,313836593	LADA vs T2D Time-adjusted
hsa-miR-744-5p_TGCGGGGCTAGGGCTAACAGC2	-0,908259997	3,989518529	0,054071312	0,313836593	LADA vs T2D Time-adjusted
hsa-miR-345-5p_GCTGACTCCTAGTCCAGGGCT2	-0,822757686	5,007179685	0,054356022	0,313836593	LADA vs T2D Time-adjusted
hsa-miR-451a_ACCGTTACCATTACTGAG2	-0,955847093	3,859377434	0,055419939	0,314661247	LADA vs T2D Time-adjusted
hsa-miR-146a-5p_TGAGAACTGAATTCCATGGGTTGT2	-0,493659658	7,268590607	0,055507358	0,314661247	LADA vs T2D Time-adjusted
hsa-miR-28-5p_AAGGAGCTCACAGTCTATTGAG2	-1,095286488	3,305855814	0,056005891	0,314661247	LADA vs T2D Time-adjusted
hsa-miR-320a-3p_GAAAAGCTGGGTTGAGAGGGCGA2	-0,970341604	3,726965694	0,056090058	0,314661247	LADA vs T2D Time-adjusted
hsa-miR-320b_AAAAGCTGGGTTGAGAGGGC2	-1,649632738	5,096748857	0,057537037	0,315726302	LADA vs T2D Time-adjusted
hsa-miR-424-3p_CAAAACGTGAGGCGCTGCT2	-0,989411091	3,07909127	0,057803845	0,315726302	LADA vs T2D Time-adjusted
hsa-miR-125b-5p_TCCCTGAGACCCTAACTT2	-1,004535798	2,928962579	0,057860974	0,315726302	LADA vs T2D Time-adjusted
hsa-miR-543_AAACATTCGCGGTGCACTTCTT2	-1,056653433	3,525271253	0,058234849	0,315726302	LADA vs T2D Time-adjusted
hsa-miR-335-3p_GTTTTTCATTATTGCTCCTGACC2	-1,11267882	2,856849935	0,058275651	0,315726302	LADA vs T2D Time-adjusted
hsa-let-7i-5p_TGAGGTAGTAGTTTGTGCTGTTG2	-0,948316724	3,944192775	0,059208143	0,318596197	LADA vs T2D Time-adjusted
hsa-miR-222-3p_AGCTACATCTGGCTACTGGGTCT2	-0,550432986	6,477997223	0,060472282	0,322705347	LADA vs T2D Time-adjusted
hsa-miR-409-3p_GAATGTTGCTCGGTGAACCCCTT2	-1,05623524	5,003955447	0,060871191	0,322705347	LADA vs T2D Time-adjusted
hsa-miR-30c-5p_TGTAAACATCCTACACTCTCA2	-0,425597629	7,960145287	0,061195704	0,322705347	LADA vs T2D Time-adjusted

hsa-miR-197-3p_TTCACCACCTTCTCCACCCAGC2	0,291110971	10,0726909	0,062910133	0,322735859	LADA vs T2D Time-adjusted
hsa-miR-125b-5p_TCCCTGAGACCCTAACTTGTGA2	-0,690444089	5,621036711	0,063114221	0,322735859	LADA vs T2D Time-adjusted
hsa-miR-25-3p_ATTGCACTTGTCTCGGTCTGA2	-0,629451198	5,817358164	0,063245516	0,322735859	LADA vs T2D Time-adjusted
hsa-miR-221-3p_AGCTACATTGTCTGCTGGGTT2	-0,625677324	6,712650504	0,063621847	0,322735859	LADA vs T2D Time-adjusted
hsa-let-7d-3p_CTATACGACCTGCTGCCTTT2	0,270622011	10,25191544	0,06363589	0,322735859	LADA vs T2D Time-adjusted
hsa-miR-486-5p_CTGTACTGAGCTGCCCCG2	-1,042516189	3,36154621	0,06364955	0,322735859	LADA vs T2D Time-adjusted
hsa-miR-30d-5p_TAAACATCCCCGACTGGAAGCT2	-0,98052668	2,129907594	0,064721655	0,326081716	LADA vs T2D Time-adjusted
hsa-miR-146b-5p_TGAGAACTGAATTCCATAGGCT2	-0,503977551	6,379112274	0,065733342	0,329082744	LADA vs T2D Time-adjusted
hsa-miR-505-3p_CGTCAACACTTGCTGGTTTCCT2	-0,940905323	3,930461517	0,069462795	0,340154526	LADA vs T2D Time-adjusted
hsa-miR-15b-5p_TAGCAGCACATCATGGTTTA2	-0,576369231	5,81103732	0,069500651	0,340154526	LADA vs T2D Time-adjusted
hsa-miR-182-5p_TTTGGCAATGGTAGAACTCA2	-0,451774821	7,904920515	0,069639932	0,340154526	LADA vs T2D Time-adjusted
hsa-miR-22-3p_AAGCTGCCAGTTGAAGAACTGT2	-0,156169608	12,1121356	0,070047019	0,340154526	LADA vs T2D Time-adjusted
hsa-miR-181a-5p_AACATTCAACGCTGTCGG2	-0,910173664	2,244051199	0,070211942	0,340154526	LADA vs T2D Time-adjusted
hsa-miR-16-5p_AGCAGCACGTAAATATTGG2	-0,956901001	3,202629707	0,0709165	0,340154526	LADA vs T2D Time-adjusted
hsa-miR-21-5p_TAGCTTATCAGACTGATG2	-0,991671746	2,041272314	0,070955116	0,340154526	LADA vs T2D Time-adjusted
hsa-let-7f-5p_TGAGGTAGTAGATTGTATAG2	-0,240742522	10,5471638	0,072556021	0,345733811	LADA vs T2D Time-adjusted
hsa-miR-148a-3p_TCAGTGCACTACAGAACTTTG2	-0,372576197	7,255293658	0,074312135	0,351696261	LADA vs T2D Time-adjusted
hsa-miR-101-3p_GTACAGTACTGTGATAACT2	-0,851368034	4,834369948	0,074696551	0,351696261	LADA vs T2D Time-adjusted
hsa-miR-29a-3p_TAGCACCATCTGAAATCGGTTA2	-0,77494498	4,650458238	0,077277949	0,359810194	LADA vs T2D Time-adjusted
hsa-miR-30d-5p_TGTAAACATCCCCGACTGGAAGCT2	-0,21932002	13,12660906	0,07771265	0,359810194	LADA vs T2D Time-adjusted
hsa-miR-99b-5p_CACCCGTAGAACCGACCTTGC2	0,278990203	7,928619761	0,078023337	0,359810194	LADA vs T2D Time-adjusted
hsa-miR-584-5p_TTATGGTTTGCCTGGGACT2	-0,768272755	4,566540106	0,078239385	0,359810194	LADA vs T2D Time-adjusted
hsa-miR-2110_TTGGGGAAACGGCCGCTGAG2	-0,95884959	2,146127874	0,079137375	0,361836208	LADA vs T2D Time-adjusted
hsa-miR-502-3p_AATGCACCTGGGCAAGGATTCA2	-0,676304986	4,779774283	0,079908626	0,363262779	LADA vs T2D Time-adjusted
hsa-miR-328-3p_CTGGCCCTCTCTGCCCTTCCG2	-0,810486264	3,896716502	0,082285444	0,371930208	LADA vs T2D Time-adjusted
hsa-miR-192-5p_TGACCTATGAATTGACAGCCA2	-0,799000839	4,775035894	0,082799098	0,372125491	LADA vs T2D Time-adjusted
hsa-miR-27b-3p_TTCACAGTGGCTAAGTTCTGC2	-0,334310018	7,857204391	0,083517298	0,37323267	LADA vs T2D Time-adjusted
hsa-miR-22-3p_AAGCTGCCAGTTGAAGAA2	-0,775891731	5,032500306	0,085284471	0,377133436	LADA vs T2D Time-adjusted
hsa-let-7a-5p_GAGGTAGTAGGTTGTATAG2	-0,68841263	5,313092713	0,085558277	0,377133436	LADA vs T2D Time-adjusted
hsa-miR-183-5p_ATGGCACTGGTAGAATTCACTG2	-0,950758688	2,958551449	0,085820504	0,377133436	LADA vs T2D Time-adjusted
hsa-miR-30a-5p_GTAAACATCCTCGACTGGAAGCT2	-0,846810688	4,346285408	0,08930546	0,390279662	LADA vs T2D Time-adjusted
hsa-miR-4433b-5p_TATGTCCCACCCCCACTCCTG2	1,091853401	2,841400639	0,090327612	0,392488515	LADA vs T2D Time-adjusted
hsa-miR-92b-3p_TATTGCACTCGTCCCGGCC2	-0,897832463	3,918389355	0,090921556	0,392488515	LADA vs T2D Time-adjusted
hsa-miR-101-3p_TACAGTACTGTGATAACTGA2	-0,897532487	4,251229709	0,091299478	0,392488515	LADA vs T2D Time-adjusted
hsa-let-7d-3p_CTATACGACCTGCTGCCTTTCT2	0,180905175	11,72675264	0,092245928	0,394413672	LADA vs T2D Time-adjusted
hsa-miR-223-3p_TCAGTTTGTCAAATACCCCAA2	-0,873535071	2,097761681	0,094174256	0,400493745	LADA vs T2D Time-adjusted
hsa-miR-223-3p_GTCAGTTTGTCAAATACCCCAA2	-0,478306204	7,633945564	0,09652435	0,404003876	LADA vs T2D Time-adjusted
hsa-miR-409-3p_GAATGTTGCTCGGTGAACCCCTTT2	-0,950545153	3,983254098	0,097055571	0,404003876	LADA vs T2D Time-adjusted
hsa-miR-30d-5p_GTAAACATCCCCGACTGGAAGC2	-0,807907418	4,700243003	0,097451364	0,404003876	LADA vs T2D Time-adjusted
hsa-miR-24-3p_GCTCAGTTCAGCAGGAACAG2	-0,890319952	2,351084248	0,098158452	0,404003876	LADA vs T2D Time-adjusted
hsa-miR-99a-5p_AACCCGTAGATCCGATCTTGTG2	-0,551512575	5,465860433	0,098528783	0,404003876	LADA vs T2D Time-adjusted
hsa-miR-101-3p_TACAGTACTGTGATAACTGAA2	-0,955076959	2,97176202	0,098715082	0,404003876	LADA vs T2D Time-adjusted
hsa-miR-93-3p_ACTGCTGAGCTAGCACTTCCCGA2	-0,837009261	3,420021767	0,099312113	0,404003876	LADA vs T2D Time-adjusted
hsa-miR-16-2-3p_CCAATATTACTGTGCTGCTT2	-0,640758143	4,45076213	0,099355142	0,404003876	LADA vs T2D Time-adjusted
hsa-miR-23b-3p_ATCACATTGCCAGGGATTAC2	-0,800893058	2,73633577	0,099596404	0,404003876	LADA vs T2D Time-adjusted
hsa-miR-151a-3p_CTAGACTGAAGCTCCTTGAGGA2	-0,295034533	8,369867531	0,102456071	0,412874259	LADA vs T2D Time-adjusted
hsa-miR-16-5p_AGCAGCACGTAAATATTGGC2	-0,860198591	3,090604583	0,102918367	0,412874259	LADA vs T2D Time-adjusted
hsa-miR-122-5p_TGGAGTGTGACAATGGTGTTT2	-0,419937425	13,69364351	0,103349056	0,412874259	LADA vs T2D Time-adjusted
hsa-miR-451a_CCGTTACCATTACTGAGT2	-0,744840138	4,375477916	0,104533406	0,413574193	LADA vs T2D Time-adjusted
hsa-miR-375-3p_TTGTTCGTTCGGCTCGCGTGA2	-0,681045023	5,794174671	0,104757894	0,413574193	LADA vs T2D Time-adjusted
hsa-miR-423-3p_AGCTCGGTCTGAGGCCCCTCA2	-0,526922923	6,070547639	0,105199102	0,413574193	LADA vs T2D Time-adjusted
hsa-miR-222-3p_AGCTACATCTGGCTACTGGGT2	-0,317472909	7,650900477	0,105841996	0,413574193	LADA vs T2D Time-adjusted
hsa-let-7a-5p_GAGGTAGTAGGTTGTATAGTT2	0,353747398	7,12979206	0,106141201	0,413574193	LADA vs T2D Time-adjusted
hsa-miR-223-3p_TGTCAGTTTGTCAAATACCC2	-0,683085887	4,539198612	0,107303173	0,413574193	LADA vs T2D Time-adjusted
hsa-miR-421_ATCAACAGACATTAATTGGGCG2	-0,83241346	2,15305225	0,107835982	0,413574193	LADA vs T2D Time-adjusted
hsa-miR-320a-3p_GAAAAGCTGGGTTGAGAGGGCG2	-0,48412973	6,257483702	0,107953324	0,413574193	LADA vs T2D Time-adjusted
hsa-miR-183-5p_TATGGCACTGGTAGAATTCA2	-0,464933258	7,316738774	0,108229909	0,413574193	LADA vs T2D Time-adjusted
hsa-miR-342-3p_TCTCACACAGAAATCGCACCCGTCA2	0,878169717	3,434989001	0,108854459	0,413960946	LADA vs T2D Time-adjusted
hsa-miR-194-5p_TGTAACAGCAACTCCATGTGG2	-0,426205335	6,643496377	0,112115476	0,421250217	LADA vs T2D Time-adjusted
hsa-miR-16-5p_TAGCAGCACGTAAATATTGGCG2	-0,192919083	14,02532692	0,11230469	0,421250217	LADA vs T2D Time-adjusted
hsa-miR-486-3p_CGGGGCAGCTCAGTACAGGAT2	-0,411818065	7,767064538	0,112785015	0,421250217	LADA vs T2D Time-adjusted

hsa-miR-24-3p_GGCTCAGTTCAGCAGGAACAG2	-0,383259391	6,840568144	0,112939927	0,421250217	LADA vs T2D Time-adjusted
hsa-miR-223-3p_TGTCAGTTTGTCAAATACCCCA2	-0,279457233	10,34583114	0,113487341	0,421250217	LADA vs T2D Time-adjusted
hsa-miR-10b-5p_ACCCTGTAGAACCGAATTTGTGT2	-0,812109042	3,358063133	0,113966557	0,421250217	LADA vs T2D Time-adjusted
hsa-let-7c-5p_TGAGGTAGTAGGTTGTATGGTTT2	-0,760544455	3,632126079	0,115365014	0,421664329	LADA vs T2D Time-adjusted
hsa-miR-423-5p_TGAGGGGCAGAGAGCGAGACTTT2	0,111044454	14,46310907	0,11545503	0,421664329	LADA vs T2D Time-adjusted
hsa-miR-148a-3p_TCAGTGCACTACAGAACTTTGT2	-0,225409782	9,218931731	0,115832114	0,421664329	LADA vs T2D Time-adjusted
hsa-miR-191-5p_AACGGAATCCCAAAAGCAGCTG2	-0,463096563	5,768928318	0,116246857	0,421664329	LADA vs T2D Time-adjusted
hsa-miR-425-5p_AATGACACGATCACTCCCGTT2	-0,325461994	8,013037563	0,11674398	0,421664329	LADA vs T2D Time-adjusted
hsa-miR-151a-3p_TACTAGACTGAAGCTCCTTGAG2	-0,837083814	2,948484967	0,11975599	0,429050541	LADA vs T2D Time-adjusted
hsa-miR-199a-5p_CCCAGTGTTCAGACTACCTGTTC2	-0,78815501	4,322462141	0,119873792	0,429050541	LADA vs T2D Time-adjusted
hsa-miR-30e-3p_CTTTCAGTCGGATGTTTACAG2	-0,841409001	2,479912206	0,122185702	0,433611906	LADA vs T2D Time-adjusted
hsa-miR-15b-3p_CGAATCATTATTTGCTGCTCT2	-0,691312562	4,370495899	0,122451881	0,433611906	LADA vs T2D Time-adjusted
hsa-miR-339-3p_TGAGCGCCTCGACGACAGAGCCG2	-0,845935991	4,074916381	0,123319725	0,433611906	LADA vs T2D Time-adjusted
hsa-miR-21-5p_AGCTTATCAGACTGATGTTGAC2	-0,911036405	3,019001358	0,124103643	0,433611906	LADA vs T2D Time-adjusted
hsa-miR-142-5p_CCCATAAAGTAGAAAGCACTAC2	-0,385892051	6,93738446	0,124201502	0,433611906	LADA vs T2D Time-adjusted
hsa-let-7d-3p_CTATACGACCTGCTGCCTT2	-0,662876939	4,663499808	0,124835088	0,433611906	LADA vs T2D Time-adjusted
hsa-miR-885-5p_TCCATTACACTACCCTGCCTCT2	-1,59618832	4,712163409	0,12498548	0,433611906	LADA vs T2D Time-adjusted
hsa-miR-16-5p_GCAGCACGTAAATATTGGCG2	-0,723318996	3,590895157	0,127190635	0,43766884	LADA vs T2D Time-adjusted
hsa-miR-3613-5p_TGTTGTACTTTTTTTTTTGTTC2	-0,667443791	4,360142246	0,127261483	0,43766884	LADA vs T2D Time-adjusted
hsa-miR-484_TCAGGCTCAGTCCCCTCCCGA2	-0,265792117	10,82608497	0,129578897	0,44137787	LADA vs T2D Time-adjusted
hsa-miR-142-5p_CCCATAAAGTAGAAAGCACTA2	-0,569511548	5,238891455	0,129978359	0,44137787	LADA vs T2D Time-adjusted
hsa-miR-451a_AAACCGTTACCATTACTGAGTTT2	-0,264810863	9,000647073	0,130013962	0,44137787	LADA vs T2D Time-adjusted
hsa-miR-221-3p_AGCTACATTGTCTGCTGGGTTTC2	-0,509020077	6,662543722	0,130842942	0,441393959	LADA vs T2D Time-adjusted
hsa-miR-125a-5p_TCCCTGAGACCCTTTAACCTGT2	0,189224643	11,50918116	0,131134741	0,441393959	LADA vs T2D Time-adjusted
hsa-miR-15b-5p_TAGCAGCACATCATGGTT2	-0,419758347	7,409647991	0,133018809	0,444338221	LADA vs T2D Time-adjusted
hsa-miR-16-5p_TAGCAGCACGTAAATATTGG2	-0,216644529	10,9873739	0,13325514	0,444338221	LADA vs T2D Time-adjusted
hsa-miR-505-3p_CGTCAACACTTGCTGGTTT2	-0,788827991	3,312021603	0,133694686	0,444338221	LADA vs T2D Time-adjusted
hsa-miR-101-3p_TACAGTACTGTGATAACTGAAG2	-0,382377846	6,912528742	0,138869619	0,456932219	LADA vs T2D Time-adjusted
hsa-miR-27a-3p_TTCACAGTGGCTAAGTTCCG2	0,1877926	9,746526676	0,138963217	0,456932219	LADA vs T2D Time-adjusted
hsa-miR-191-5p_CAACGGAATCCCAAAAGCAGC2	-0,295816481	8,118131191	0,139217022	0,456932219	LADA vs T2D Time-adjusted
hsa-miR-30e-5p_TGTAAACATCCTTGACTGGAA2	-0,33993201	6,485643066	0,14031378	0,458628925	LADA vs T2D Time-adjusted
hsa-miR-181b-5p_AACATTCATTGCTGTCGGTGG2	-0,537417278	5,17184581	0,141764367	0,461463433	LADA vs T2D Time-adjusted
hsa-miR-181a-5p_AACATTCAACGCTGTCGGTG2	-0,376819795	6,965832799	0,143494092	0,465179617	LADA vs T2D Time-adjusted
hsa-miR-144-5p_GGATATCATCATATACTGTAA2	-0,80316095	4,029614813	0,145149892	0,467562691	LADA vs T2D Time-adjusted
hsa-miR-1307-3p_CTCGGCGTGGCGTCGGTCGTGG2	-0,853313735	2,234349501	0,145411406	0,467562691	LADA vs T2D Time-adjusted
hsa-miR-92a-3p_GCACTTGTCCCGGCCTGT2	-0,654531461	4,472613341	0,146247789	0,467723327	LADA vs T2D Time-adjusted
hsa-miR-7-5p_TGGAAGACTAGTGATTTTGTTGT2	-0,540222556	6,235025385	0,146793071	0,467723327	LADA vs T2D Time-adjusted
hsa-miR-26b-5p_TTCAAGTAATTCAGGATAGGT2	-0,300328682	7,33079836	0,147235282	0,467723327	LADA vs T2D Time-adjusted
hsa-miR-425-5p_ATGACACGATCACTCCCGTTGA2	-0,604627548	4,777521295	0,150847225	0,472542054	LADA vs T2D Time-adjusted
hsa-miR-143-3p_TGAGATGAAGCACTGTAGCTCA2	-0,453451834	6,633635761	0,151055565	0,472542054	LADA vs T2D Time-adjusted
hsa-miR-186-5p_CAAAGAATTCTCCTTTTGGGCTT2	-0,483212879	6,241384756	0,151230126	0,472542054	LADA vs T2D Time-adjusted
hsa-miR-182-5p_TTTGGCAATGGTAGAACTCACACTG2	-0,778311018	3,704867925	0,151636644	0,472542054	LADA vs T2D Time-adjusted
hsa-miR-99b-5p_CACCCGTAGAACCGACCTTGCG2	0,225010335	9,362081621	0,151739168	0,472542054	LADA vs T2D Time-adjusted
hsa-miR-361-3p_TCCCCCAGGTGTGATTCTGA2	-0,739037796	3,266035349	0,155041896	0,479494794	LADA vs T2D Time-adjusted
hsa-let-7b-5p_TGAGGTAGTAGGTTGTGTGGTT2	-0,127874077	12,80806781	0,155184156	0,479494794	LADA vs T2D Time-adjusted
hsa-let-7a-5p_TTGAGGTAGTAGGTTGTATAGTT2	-0,780076388	1,879153168	0,157950164	0,486142335	LADA vs T2D Time-adjusted
hsa-miR-942-5p_TCTTCTCTGTTTTGGCCATGT2	-0,796137571	2,575024753	0,159150147	0,487394586	LADA vs T2D Time-adjusted
hsa-miR-181a-2-3p_ACCACTGACCGTTGACTGTACC2	-0,805064885	2,432112395	0,160091934	0,487394586	LADA vs T2D Time-adjusted
hsa-miR-103a-3p_AGCAGCATTGTACAGGGCTATGA2	0,286894015	8,59094218	0,160205553	0,487394586	LADA vs T2D Time-adjusted
hsa-miR-574-3p_CACGCTCATGCACACACCCA2	-0,756823129	3,290108497	0,161433735	0,488495963	LADA vs T2D Time-adjusted
hsa-miR-125b-5p_TCCCTGAGACCCTAACT2	-0,868746189	2,244613923	0,161802708	0,488495963	LADA vs T2D Time-adjusted
hsa-miR-21-5p_TAGCTTATCAGACTGATGTTG2	0,172755746	11,65018988	0,163019393	0,488576866	LADA vs T2D Time-adjusted
hsa-miR-16-5p_AGCAGCACGTAAATATTGGCG2	-0,24719117	8,159714087	0,163262617	0,488576866	LADA vs T2D Time-adjusted
hsa-miR-320a-3p_AAAGCTGGGTTGAGAGGGCGA2	-0,610636647	5,194526481	0,163682515	0,488576866	LADA vs T2D Time-adjusted
hsa-miR-629-5p_TGGGTTTACGTTGGGAGAACTT2	-0,673570991	4,102285673	0,16575127	0,48945453	LADA vs T2D Time-adjusted
hsa-miR-148a-3p_CAGTGCACTACAGAACTTTGT2	-0,799100069	2,153358606	0,165799726	0,48945453	LADA vs T2D Time-adjusted
hsa-miR-1301-3p_TTGCAGCTGCCTGGGAGTGACTTC2	-0,865523945	2,475326197	0,165832887	0,48945453	LADA vs T2D Time-adjusted
hsa-miR-30c-5p_TGTAAACATCCTACACTCTC2	-0,805339944	2,337351039	0,167573734	0,492753992	LADA vs T2D Time-adjusted
hsa-miR-185-5p_TGGAGAGAAAGGCAGTTCCT2	0,795372312	2,073411525	0,170627057	0,498600639	LADA vs T2D Time-adjusted
hsa-miR-584-5p_TTATGGTTTGCCTGGGACTGA2	-0,270580745	8,05116289	0,170822722	0,498600639	LADA vs T2D Time-adjusted
hsa-miR-652-3p_AATGGCGCCACTAGGGTTGTGC2	-0,707031831	3,398629288	0,173507401	0,504574832	LADA vs T2D Time-adjusted

hsa-miR-652-3p_AATGGCGCCACTAGGGTTGT2	-0,217186598	8,007167607	0,175591796	0,508765972	LADA vs T2D Time-adjusted
hsa-miR-361-3p_TCCCCCAGGTGTGATTCTGATTTG2	-0,422894602	5,300626315	0,17654586	0,50928366	LADA vs T2D Time-adjusted
hsa-miR-543_AAACATTCGCGGTGCACTTCTTT2	-0,843607658	3,18614348	0,177065731	0,50928366	LADA vs T2D Time-adjusted
hsa-miR-10a-5p_TACCCTGTAGATCCGAAT2	0,787442168	2,390493785	0,17770201	0,50928366	LADA vs T2D Time-adjusted
hsa-miR-335-5p_TCAAGAGCAATAACGAAAAAT2	0,458810676	6,614922062	0,18022295	0,513837268	LADA vs T2D Time-adjusted
hsa-miR-361-3p_TCCCCCAGGTGTGATTCTGATT2	-0,595059583	3,75062111	0,180590089	0,513837268	LADA vs T2D Time-adjusted
hsa-miR-29a-3p_TAGCACCATCTGAAATCGG2	-0,326031485	7,313106214	0,182378792	0,517066755	LADA vs T2D Time-adjusted
hsa-miR-152-3p_TCAGTGCATGACAGAACTTGG2	-0,675076534	2,028197906	0,183957959	0,519681233	LADA vs T2D Time-adjusted
hsa-miR-10b-5p_TACCCTGTAGAACCGAATTTGTG2	0,270486431	7,887217076	0,185781797	0,519764575	LADA vs T2D Time-adjusted
hsa-miR-2110_TTGGGGAAACGGCCGCTGAGT2	-0,417215566	6,041635722	0,185804209	0,519764575	LADA vs T2D Time-adjusted
hsa-miR-499a-5p_TTAAGACTTGCAGTGATGTTT2	0,761315487	2,030187923	0,185958754	0,519764575	LADA vs T2D Time-adjusted
hsa-miR-421_ATCAACAGACATTAATTGGGCGC2	-0,698959522	2,015098416	0,186793944	0,520260596	LADA vs T2D Time-adjusted
hsa-miR-3613-5p_TGTTGTACTTTTTTTTTTGTT2	-0,517374051	5,198438116	0,189024097	0,523730571	LADA vs T2D Time-adjusted
hsa-miR-4433b-5p_ATGTCCCACCCCCACTCCTGTT2	0,544480498	8,088855349	0,189364024	0,523730571	LADA vs T2D Time-adjusted
hsa-miR-451a_AAACCGTTACCATTACTGA2	-0,261342074	8,906066815	0,190156383	0,524089542	LADA vs T2D Time-adjusted
hsa-let-7b-5p_TGAGGTAGTAGGTTGTGTGG2	0,148277086	12,46184935	0,192872493	0,528297584	LADA vs T2D Time-adjusted
hsa-miR-335-5p_TCAAGAGCAATAACGAAAAATGT2	-0,428476687	6,087791959	0,193018966	0,528297584	LADA vs T2D Time-adjusted
hsa-miR-181a-5p_AACATTCAACGCTGTCGGTGAGTT2	-0,703620626	2,473533262	0,194242658	0,529813594	LADA vs T2D Time-adjusted
hsa-miR-10a-5p_TACCCTGTAGATCCGAATTTG2	0,201096604	8,877087473	0,19493402	0,529872198	LADA vs T2D Time-adjusted
hsa-miR-126-3p_TCGTACCGTGAGTAATAATGC2	-0,3150159	6,39389942	0,197927613	0,536166925	LADA vs T2D Time-adjusted
hsa-let-7f-5p_TGAGGTAGTAGATTG2	-0,628893393	4,368619365	0,200829466	0,541382516	LADA vs T2D Time-adjusted
hsa-miR-183-5p_ATGGCACTGGTAGAATTCACTGT2	-0,719528559	2,504851483	0,20208531	0,541382516	LADA vs T2D Time-adjusted
hsa-miR-450b-5p_TTTTGCAATATGTTCCTGAAT2	-0,581873636	4,972105489	0,202395875	0,541382516	LADA vs T2D Time-adjusted
hsa-miR-106b-5p_TAAAGTGCTGACAGTGCAGA2	-0,519558316	5,030812023	0,202590676	0,541382516	LADA vs T2D Time-adjusted
hsa-miR-146b-5p_TGAGAACTGAATTCCATAGGCTGT2	-0,211913037	8,367181967	0,206985933	0,551265566	LADA vs T2D Time-adjusted
hsa-miR-1306-5p_CCACCTCCCCTGCAAACGT2	0,799836767	2,609394089	0,209226846	0,555363876	LADA vs T2D Time-adjusted
hsa-miR-125b-5p_TCCCTGAGACCCTAACTTGTG2	-0,518299666	5,122028633	0,210019618	0,555603738	LADA vs T2D Time-adjusted
hsa-miR-363-3p_AATTGCACGGTATCCATCTGT2	-0,196663057	9,045234632	0,212132609	0,555605106	LADA vs T2D Time-adjusted
hsa-miR-27a-3p_TTCACAGTGGCTAAGTTCCGC2	-0,382859972	6,161654231	0,213192302	0,555605106	LADA vs T2D Time-adjusted
hsa-miR-150-5p_TCTCCCAACCCTTGTACCAG2	0,291416364	9,064957556	0,213351777	0,555605106	LADA vs T2D Time-adjusted
hsa-miR-424-5p_CAGCAGCAATTCATGTTTTGA2	0,703022198	2,664190598	0,21350329	0,555605106	LADA vs T2D Time-adjusted
hsa-miR-1306-5p_CCACCTCCCCTGCAAACGTCC2	0,654422755	4,59875793	0,213532177	0,555605106	LADA vs T2D Time-adjusted
hsa-miR-144-3p_TACAGTATAGATGATGTAC2	-0,37872755	6,411669154	0,216743189	0,559890527	LADA vs T2D Time-adjusted
hsa-miR-340-3p_TCCGTCTCAGTTACTTTATAGCC2	-0,700130501	3,011838282	0,21724813	0,559890527	LADA vs T2D Time-adjusted
hsa-miR-92a-3p_TATTGCACTTGTCCCGGCC2	-0,27996989	8,042410264	0,217302644	0,559890527	LADA vs T2D Time-adjusted
hsa-miR-4433b-5p_ATGTCCCACCCCCACTCCTGT2	0,475302979	7,641633929	0,22050354	0,565208001	LADA vs T2D Time-adjusted
hsa-miR-132-3p_TAACAGTCTACAGCCATGGTCG2	-0,681287741	2,429322611	0,22079554	0,565208001	LADA vs T2D Time-adjusted
hsa-miR-186-5p_AAAGAATTCTCCTTTTGGGCT2	-0,646428916	3,93470427	0,225702634	0,575905754	LADA vs T2D Time-adjusted
hsa-miR-92a-3p_ATTGCACTTGTCCCGGCCT2	-0,576568764	4,481036729	0,229163535	0,581219849	LADA vs T2D Time-adjusted
hsa-miR-10b-5p_TACCCTGTAGAACCGAAT2	-0,692371876	2,102144035	0,22973716	0,581219849	LADA vs T2D Time-adjusted
hsa-miR-222-3p_AGCTACATCTGGCTACTGGGTCTC2	-0,530665813	5,925164986	0,229989649	0,581219849	LADA vs T2D Time-adjusted
hsa-miR-30c-5p_TGTAAACATCCTACACTCTCAGC2	-0,278279147	6,576373518	0,231522247	0,582902876	LADA vs T2D Time-adjusted
hsa-let-7i-5p_GAGGTAGTAGTTTGTGCTGTT2	-0,674142786	3,203293849	0,232129464	0,582902876	LADA vs T2D Time-adjusted
hsa-miR-21-5p_GTAGCTTATCAGACTGATGTTGA2	-0,68243742	2,233806863	0,233716101	0,585029861	LADA vs T2D Time-adjusted
hsa-miR-27b-3p_TTCACAGTGGCTAAGTTCTG2	-0,216236543	8,329497842	0,235541168	0,586206677	LADA vs T2D Time-adjusted
hsa-miR-423-3p_AGCTCGGTCTGAGGCCCCT2	-0,354972572	6,073013782	0,235959822	0,586206677	LADA vs T2D Time-adjusted
hsa-miR-192-5p_CTGACCTATGAATTGACAGC2	-0,663749859	2,069053686	0,23640952	0,586206677	LADA vs T2D Time-adjusted
hsa-miR-10a-5p_TACCCTGTAGATCCGAATTT2	-0,5808554	4,289428906	0,23836608	0,589211154	LADA vs T2D Time-adjusted
hsa-miR-28-3p_ACTAGATTGTGAGCTCCTGGAG2	-0,550871821	4,665468153	0,239299933	0,589676781	LADA vs T2D Time-adjusted
hsa-miR-142-5p_CATAAAGTAGAAAGCACTAC2	-0,639248945	2,318773636	0,242222755	0,593957901	LADA vs T2D Time-adjusted
hsa-miR-335-3p_TTTTTCATTATTGCTCCTGACC2	-0,629827899	3,947185728	0,242759816	0,593957901	LADA vs T2D Time-adjusted
hsa-miR-16-5p_TTAGCAGCACGTAAATATTGGCG2	-0,57769444	4,214912823	0,243289962	0,593957901	LADA vs T2D Time-adjusted
hsa-miR-409-3p_CGAATGTTGCTCGGTGAACCCCTT2	-0,698103895	3,582607068	0,244821022	0,595856703	LADA vs T2D Time-adjusted
hsa-miR-1301-3p_TTGCAGCTGCCTGGGAGTGA2	-0,66626763	3,323484613	0,250183899	0,605959706	LADA vs T2D Time-adjusted
hsa-miR-30d-5p_GTAAACATCCCCGACTGGAA2	-0,474920973	4,880801026	0,250504202	0,605959706	LADA vs T2D Time-adjusted
hsa-miR-92a-3p_TTGCACTTGTCCCGGCCTG2	-0,54230603	4,224665197	0,253514505	0,608846404	LADA vs T2D Time-adjusted
hsa-miR-23b-3p_ATCACATTGCCAGGGATTACC2	0,401915726	5,450967457	0,253927992	0,608846404	LADA vs T2D Time-adjusted
hsa-miR-451a_AACCGTTACCATTACTGAGT2	-0,193791083	10,87118749	0,254006717	0,608846404	LADA vs T2D Time-adjusted
hsa-miR-2110_TTGGGGAAACGGCCGCTGAGTGA2	-0,376693133	6,031153966	0,254862135	0,609051205	LADA vs T2D Time-adjusted
hsa-miR-30e-5p_TGTAAACATCCTTGACTGG2	-0,434321162	5,259392082	0,256558646	0,609527863	LADA vs T2D Time-adjusted
hsa-miR-151a-3p_TACTAGACTGAAGCTCCTTGAGG2	-0,539853806	3,949800351	0,256602754	0,609527863	LADA vs T2D Time-adjusted

hsa-miR-342-3p_TCACACAGAAATCGCACCCGTCA2	-0,568457573	3,93860325	0,259520141	0,614612072	LADA vs T2D Time-adjusted
hsa-miR-320b_AAAAGCTGGGTTGAGAGGGCAA2	-0,583351716	2,061631881	0,260640992	0,615423954	LADA vs T2D Time-adjusted
hsa-miR-374b-5p_ATATAATACAACCTGCTAAGT2	-0,530352853	3,94114477	0,263394525	0,620045277	LADA vs T2D Time-adjusted
hsa-miR-122-5p_GAGTGTGACAATGGTGTTT2	-0,66140228	3,048399419	0,265058053	0,620045277	LADA vs T2D Time-adjusted
hsa-miR-26a-5p_TTCAAGTAATCCAGGATAG2	-0,577962953	3,674214058	0,265306016	0,620045277	LADA vs T2D Time-adjusted
hsa-let-7a-5p_TGAGGTAGTAGGTTGTATAGTT2	0,139891279	13,3879175	0,266967516	0,620045277	LADA vs T2D Time-adjusted
hsa-miR-122-5p_TGGAGTGTGACAATGGTGTTTGT2	-0,350003578	8,317298507	0,2671384	0,620045277	LADA vs T2D Time-adjusted
hsa-miR-193a-5p_TGGGTCTTTGCGGGCGAGAT2	-0,496748812	4,70145732	0,267301441	0,620045277	LADA vs T2D Time-adjusted
hsa-miR-99a-5p_AACCCGTAGATCCGATCTTGT2	-0,191589572	8,596582119	0,269205839	0,621433635	LADA vs T2D Time-adjusted
hsa-miR-501-3p_AATGCACCCGGGCAAGGATTC2	-0,610117179	3,029908341	0,269471222	0,621433635	LADA vs T2D Time-adjusted
hsa-miR-423-3p_AGCTCGGTCTGAGGCCCC2	-0,556741457	3,601847184	0,272317226	0,625879985	LADA vs T2D Time-adjusted
hsa-miR-3615_TCTCTCGGCTCCTCGCGGCTCG2	0,158843754	8,148580046	0,272981789	0,625879985	LADA vs T2D Time-adjusted
hsa-miR-32-5p_TATTGCACATTACTAAGTTGC2	-0,615141197	2,139133623	0,276248382	0,629633219	LADA vs T2D Time-adjusted
hsa-miR-126-3p_TCGTACCGTGAGTAATAATG2	0,310544806	5,99055736	0,277744516	0,629633219	LADA vs T2D Time-adjusted
hsa-miR-32-5p_TATTGCACATTACTAAGTTG2	-0,562868282	4,122651019	0,278000049	0,629633219	LADA vs T2D Time-adjusted
hsa-miR-224-5p_CAAGTCACTAGTGGTTCCGTTTAG2	-0,59867865	4,656228151	0,278366145	0,629633219	LADA vs T2D Time-adjusted
hsa-miR-589-5p_TGAGAACCACGTCTGCTCTGA2	-0,603888513	3,05621435	0,27859877	0,629633219	LADA vs T2D Time-adjusted
hsa-miR-140-3p_TACCACAGGGTAGAACCACGGAC2	-0,558544623	4,133710403	0,281086646	0,631580374	LADA vs T2D Time-adjusted
hsa-miR-143-3p_TGAGATGAAGCACTGTAGCT2	-0,180768654	9,047567862	0,283417857	0,631580374	LADA vs T2D Time-adjusted
hsa-miR-361-5p_TTATCAGAATCTCCAGGGGTAC2	-0,251832302	6,68645525	0,283647866	0,631580374	LADA vs T2D Time-adjusted
hsa-miR-125a-5p_TCCCTGAGACCCTTTAACCT2	0,267480264	6,333504859	0,283897497	0,631580374	LADA vs T2D Time-adjusted
hsa-miR-192-5p_TGACCTATGAATTGACAGCC2	-0,320128806	6,46151299	0,284196191	0,631580374	LADA vs T2D Time-adjusted
hsa-miR-409-3p_CGAATGTTGCTCGGTGAACCCCTTT2	-0,665914786	2,311149768	0,284519088	0,631580374	LADA vs T2D Time-adjusted
hsa-miR-6803-3p_TCCCTCGCCTTCTCACCCTCAG2	-0,579596223	2,545548118	0,285312255	0,631580374	LADA vs T2D Time-adjusted
hsa-miR-320a-3p_AAAAGCTGGGTTGAGAGGGCGAAA2	-0,3017308	6,765627538	0,28616828	0,631580374	LADA vs T2D Time-adjusted
hsa-miR-451a_GAAACCGTTACCATTACTGAGT2	-0,288466993	6,902919522	0,28704759	0,631580374	LADA vs T2D Time-adjusted
hsa-miR-146a-5p_TGAGAACTGAATTCCATGGGT2	-0,145060642	8,701643613	0,287444924	0,631580374	LADA vs T2D Time-adjusted
hsa-miR-29a-3p_CTAGCACCATCTGAAATCGGTT2	-0,5313962	3,443400968	0,290018497	0,635469893	LADA vs T2D Time-adjusted
hsa-miR-140-3p_TACCACAGGGTAGAACCACG2	-0,408785411	4,858224511	0,293621458	0,636511161	LADA vs T2D Time-adjusted
hsa-miR-100-5p_AACCCGTAGATCCGAACTTGT2	-0,266428261	7,209550932	0,293755861	0,636511161	LADA vs T2D Time-adjusted
hsa-miR-223-5p_CGTGTATTTGACAAGCTGAGTT2	-0,512561525	3,834084658	0,295157509	0,636511161	LADA vs T2D Time-adjusted
hsa-miR-140-3p_ACCACAGGGTAGAACCACG2	-0,584732813	2,745546867	0,295652867	0,636511161	LADA vs T2D Time-adjusted
hsa-let-7f-5p_TGAGGTAGTAGATTGTATAGTTG2	-0,622274343	2,479162802	0,296479538	0,636511161	LADA vs T2D Time-adjusted
hsa-miR-30c-5p_GTAAACATCCTACACTCTCAGCT2	-0,618616639	3,000834251	0,29662861	0,636511161	LADA vs T2D Time-adjusted
hsa-miR-92a-3p_CACTTGTCCCGGCCTGT2	-0,51674813	4,184588919	0,296843798	0,636511161	LADA vs T2D Time-adjusted
hsa-miR-10b-5p_TACCCTGTAGAACCGAATT2	-0,504230765	4,497780085	0,29693125	0,636511161	LADA vs T2D Time-adjusted
hsa-miR-1908-5p_CGGCGGGGACGGCGATTGGTC2	0,589312808	2,7409573	0,298835079	0,638089828	LADA vs T2D Time-adjusted
hsa-miR-6803-3p_TCCCTCGCCTTCTCACCCTCA2	0,378815085	6,152212628	0,29928107	0,638089828	LADA vs T2D Time-adjusted
hsa-miR-486-5p_TCCTGTACTGAGCTGCCCC2	-0,244177	6,726361248	0,302631404	0,64024334	LADA vs T2D Time-adjusted
hsa-miR-30a-5p_TGTAAACATCCTCGACTGGAAGCT2	0,127810573	8,865025747	0,303421086	0,64024334	LADA vs T2D Time-adjusted
hsa-miR-10a-5p_ACCCTGTAGATCCGAATTTGT2	0,199794627	7,710604015	0,304304821	0,64024334	LADA vs T2D Time-adjusted
hsa-miR-144-3p_TACAGTATAGATGATGTACT2	-0,473983783	4,591697634	0,304901585	0,64024334	LADA vs T2D Time-adjusted
hsa-let-7i-5p_TGAGGTAGTAGTTTGTGCTG2	-0,166690435	10,0893919	0,306093941	0,64024334	LADA vs T2D Time-adjusted
hsa-let-7d-3p_TATACGACCTGCTGCCTTTCT2	0,137475194	8,434108679	0,306405611	0,64024334	LADA vs T2D Time-adjusted
hsa-miR-100-5p_AACCCGTAGATCCGAACTTGTG2	-0,581068981	3,697610074	0,30660088	0,64024334	LADA vs T2D Time-adjusted
hsa-miR-3173-5p_TGCCCTGCCTGTTTTCTCCTT2	0,579807757	2,961448405	0,307138892	0,64024334	LADA vs T2D Time-adjusted
hsa-miR-199a-3p_ACAGTAGTCTGCACATTGGTTA2	-0,828878636	4,923172942	0,307575814	0,64024334	LADA vs T2D Time-adjusted
hsa-miR-320a-3p_AAAGCTGGGTTGAGAGGGCG2	-0,470990551	4,800125051	0,311798073	0,647328808	LADA vs T2D Time-adjusted
hsa-miR-223-3p_GTCAGTTTGTCAAATACCCCA2	-0,314160429	6,353871952	0,31297627	0,6480739	LADA vs T2D Time-adjusted
hsa-miR-10b-5p_ACCCTGTAGAACCGAATTTGT2	0,231978631	6,802181051	0,313988594	0,648472526	LADA vs T2D Time-adjusted
hsa-miR-29c-3p_TAGCACCATTTGAAATCGG2	-0,538969473	3,275982402	0,316475112	0,651905765	LADA vs T2D Time-adjusted
hsa-miR-150-5p_TCTCCCAACCCTTGTACCAGTG2	0,159388678	11,04001159	0,31825117	0,653861495	LADA vs T2D Time-adjusted
hsa-miR-142-5p_CATAAAGTAGAAAGCACTA2	-0,528123854	2,092424464	0,319791449	0,654615004	LADA vs T2D Time-adjusted
hsa-miR-3615_TCTCTCGGCTCCTCGCGGCTCGC2	0,517199195	3,765572795	0,321175658	0,654615004	LADA vs T2D Time-adjusted
hsa-miR-451a_GAAACCGTTACCATTACTGAG2	-0,499569176	3,175112451	0,321887926	0,654615004	LADA vs T2D Time-adjusted
hsa-miR-342-3p_TCTCACACAGAAATCGCACCCGTC2	0,197726473	7,588021781	0,322707975	0,654615004	LADA vs T2D Time-adjusted
hsa-miR-30d-5p_TGTAAACATCCCCGACTGGA2	-0,163514409	9,147110661	0,322755818	0,654615004	LADA vs T2D Time-adjusted
hsa-miR-505-3p_GTCAACACTTGCTGGTTTCCTCT2	0,580731239	2,102281537	0,324435098	0,656338012	LADA vs T2D Time-adjusted
hsa-miR-192-5p_TGACCTATGAATTGACAGC2	-0,572415778	3,769796882	0,326553974	0,65893927	LADA vs T2D Time-adjusted
hsa-miR-23b-3p_ATCACATTGCCAGGGATTACCAC2	-0,44560541	5,125539346	0,329615573	0,662343821	LADA vs T2D Time-adjusted
hsa-miR-125a-5p_TCCCTGAGACCCTTTAACCTGTG2	0,152767435	8,651356777	0,33064073	0,662343821	LADA vs T2D Time-adjusted

hsa-miR-660-5p_TACCCATTGCATATCGGAGTTGT2	-0,492193775	4,013928314	0,330753236	0,662343821	LADA vs T2D Time-adjusted
hsa-miR-574-3p_CACGCTCATGCACACACCCACA2	-0,282199554	6,242074994	0,333643721	0,666444907	LADA vs T2D Time-adjusted
hsa-miR-146a-5p_TGAGAACTGAATTCCATGGGTTG2	-0,20030161	7,862859516	0,335534041	0,666495498	LADA vs T2D Time-adjusted
hsa-miR-221-3p_AGCTACATTGTCTGCTGGGT2	-0,329056301	6,247180713	0,335859076	0,666495498	LADA vs T2D Time-adjusted
hsa-miR-425-5p_AATGACACGATCACTCCCGT2	-0,259756564	6,660694598	0,336196844	0,666495498	LADA vs T2D Time-adjusted
hsa-miR-328-3p_CTGGCCCTCTCTGCCCTTCCGT2	-0,220935885	7,133297312	0,338310033	0,667598685	LADA vs T2D Time-adjusted
hsa-miR-30d-5p_TGTAAACATCCCCGACTGG2	0,14044977	8,103513785	0,339515226	0,667598685	LADA vs T2D Time-adjusted
hsa-miR-486-5p_ATCCTGTACTGAGCTGCCCCGAG2	-0,200830726	8,021810439	0,339996992	0,667598685	LADA vs T2D Time-adjusted
hsa-miR-487b-3p_TCGTACAGGGTCATCCACTTT2	-0,567096938	2,989944331	0,340296152	0,667598685	LADA vs T2D Time-adjusted
hsa-let-7b-5p_TGAGGTAGTAGGTTGTGT2	-0,357917624	5,334260563	0,340973285	0,667598685	LADA vs T2D Time-adjusted
hsa-miR-451a_AAACCGTTACCATTACTGAGTTTAG2	-0,523651294	2,222514405	0,34319135	0,669113336	LADA vs T2D Time-adjusted
hsa-let-7f-5p_TGAGGTAGTAGATTGTAT2	-0,541851665	3,218494088	0,3439719	0,669113336	LADA vs T2D Time-adjusted
hsa-miR-26a-5p_TCAAGTAATCCAGGATAGGCT2	-0,289559762	6,500759116	0,344505554	0,669113336	LADA vs T2D Time-adjusted
hsa-miR-486-5p_TCCTGTACTGAGCTGCCCCGAGG2	-0,182444754	7,407933707	0,34513052	0,669113336	LADA vs T2D Time-adjusted
hsa-miR-451a_AAACCGTTACCATTACTGAG2	-0,158750034	13,72351402	0,347874699	0,670398591	LADA vs T2D Time-adjusted
hsa-miR-10a-5p_TACCCTGTAGATCCGAATTTGT2	0,108747486	10,48518	0,348260739	0,670398591	LADA vs T2D Time-adjusted
hsa-miR-345-5p_GCTGACTCCTAGTCCAGGGCTC2	-0,518223444	3,791671294	0,348336057	0,670398591	LADA vs T2D Time-adjusted
hsa-miR-215-5p_ATGACCTATGAATTGACAGA2	0,47161937	5,04356396	0,351060338	0,672366597	LADA vs T2D Time-adjusted
hsa-miR-3173-5p_CCCTGCCTGTTTTCTCCTTTGT2	-0,478638769	1,917401292	0,351781233	0,672366597	LADA vs T2D Time-adjusted
hsa-miR-103a-3p_AGCAGCATTGTACAGGGC2	0,621729396	3,250498272	0,352626228	0,672366597	LADA vs T2D Time-adjusted
hsa-miR-30e-5p_TGTAAACATCCTTGACTGGAAG2	-0,177078238	7,594156561	0,353046438	0,672366597	LADA vs T2D Time-adjusted
hsa-miR-375-3p_TTTGTTCGTTCGGCTCGCGTG2	-0,303534393	7,069245953	0,353608728	0,672366597	LADA vs T2D Time-adjusted
hsa-miR-191-5p_AACGGAATCCCAAAAGCAGCT2	-0,227468873	6,832593963	0,356632166	0,673795475	LADA vs T2D Time-adjusted
hsa-miR-379-5p_TGGTAGACTATGGAACGTAGG2	-0,587935183	3,519537069	0,356866641	0,673795475	LADA vs T2D Time-adjusted
hsa-miR-148b-3p_TCAGTGCATCACAGAACTTTGT2	-0,183294	6,884283732	0,358891318	0,673795475	LADA vs T2D Time-adjusted
hsa-let-7d-5p_AGAGGTAGTAGGTTGCATAG2	-0,190835591	8,568558263	0,359109172	0,673795475	LADA vs T2D Time-adjusted
hsa-miR-25-3p_CATTGCACTTGTCTCGGTCT2	-0,147370323	10,31281854	0,359419627	0,673795475	LADA vs T2D Time-adjusted
hsa-miR-222-3p_AGCTACATCTGGCTACTGGGTC2	-0,461780008	3,53327113	0,359471163	0,673795475	LADA vs T2D Time-adjusted
hsa-miR-92a-3p_TATTGCACTTGTCCCGG2	-0,422416498	4,506722639	0,363077515	0,67889174	LADA vs T2D Time-adjusted
hsa-miR-23a-3p_ATCACATTGCCAGGGATTT2	0,154896315	7,89161665	0,363906571	0,67889174	LADA vs T2D Time-adjusted
hsa-miR-375-3p_TTTGTTCGTTCGGCTCGCG2	-0,362699409	5,940750727	0,365732917	0,680693499	LADA vs T2D Time-adjusted
hsa-miR-125a-5p_TCCCTGAGACCCTTTAACCTGTGA2	0,182265808	8,184259766	0,370250113	0,687483192	LADA vs T2D Time-adjusted
hsa-miR-28-3p_CACTAGATTGTGAGCTCCTGG2	-0,261770668	5,951344612	0,374408111	0,69357568	LADA vs T2D Time-adjusted
hsa-miR-423-5p_GAGGGGCAGAGAGCGAGACT2	-0,276516377	6,892897172	0,376589635	0,693637926	LADA vs T2D Time-adjusted
hsa-let-7a-5p_GAGGTAGTAGGTTGTATAGT2	-0,410553295	4,561721311	0,378332804	0,693637926	LADA vs T2D Time-adjusted
hsa-miR-92a-3p_ATTGCACTTGTCCCGGCCTGT2	-0,318264995	5,582760418	0,378343262	0,693637926	LADA vs T2D Time-adjusted
hsa-miR-500a-3p_ATGCACCTGGGCAAGGATTCT2	-0,402202278	4,574890345	0,378881139	0,693637926	LADA vs T2D Time-adjusted
hsa-miR-29a-3p_TAGCACCATCTGAAATCGGT2	-0,176727961	8,735508595	0,379390462	0,693637926	LADA vs T2D Time-adjusted
hsa-miR-409-3p_CGAATGTTGCTCGGTGAACCCCT2	-0,44164862	5,552259607	0,379703188	0,693637926	LADA vs T2D Time-adjusted
hsa-miR-320a-3p_AAAAGCTGGGTTGAGAGGGCGAAAA2	-0,482060503	3,105258929	0,385991406	0,703500465	LADA vs T2D Time-adjusted
hsa-miR-128-3p_TCACAGTGAACCGGTCTCT2	-0,173764409	7,735806596	0,389407874	0,707355518	LADA vs T2D Time-adjusted
hsa-miR-423-5p_GAGGGGCAGAGAGCGAGACTT2	0,317162141	5,546353182	0,390272197	0,707355518	LADA vs T2D Time-adjusted
hsa-miR-22-3p_AGCTGCCAGTTGAAGAACT2	-0,493716205	2,719982364	0,390789332	0,707355518	LADA vs T2D Time-adjusted
hsa-miR-432-5p_TCTTGGAGTAGGTCATTGGGTGG2	-0,505594339	3,31432182	0,394077982	0,709645676	LADA vs T2D Time-adjusted
hsa-miR-24-3p_GGCTCAGTTCAGCAGGAAC2	0,523656607	2,918934836	0,394655577	0,709645676	LADA vs T2D Time-adjusted
hsa-miR-142-5p_CCCATAAAGTAGAAAGCACT2	-0,098809075	11,11931665	0,394746014	0,709645676	LADA vs T2D Time-adjusted
hsa-miR-19a-3p_TGTGCAAATCTATGCAAAACTGA2	-0,460509784	2,482431203	0,396070276	0,710411765	LADA vs T2D Time-adjusted
hsa-miR-155-5p_TTAATGCTAATCGTGATAGGGGTT2	-0,466267603	3,129748949	0,399101075	0,713481618	LADA vs T2D Time-adjusted
hsa-miR-532-5p_CATGCCTTGAGTGTAGGACCG2	-0,439178682	2,328095035	0,399959724	0,713481618	LADA vs T2D Time-adjusted
hsa-miR-3173-5p_TGCCCTGCCTGTTTTCTCCTTT2	-0,407925357	4,474811161	0,401126699	0,713481618	LADA vs T2D Time-adjusted
hsa-miR-23a-3p_ATCACATTGCCAGGGATTTCCA2	-0,112652259	11,09453661	0,401654912	0,713481618	LADA vs T2D Time-adjusted
hsa-miR-143-3p_GAGATGAAGCACTGTAGCTC2	-0,501710231	2,215337676	0,402710856	0,713481618	LADA vs T2D Time-adjusted
hsa-miR-485-3p_GTCATACACGGCTCTCCTCTCT2	-0,468659668	5,721064879	0,403193784	0,713481618	LADA vs T2D Time-adjusted
hsa-miR-92a-3p_GTATTGCACTTGTCCCGGCCTG2	-0,460294117	3,050162886	0,404309681	0,713859281	LADA vs T2D Time-adjusted
hsa-let-7i-5p_GAGGTAGTAGTTTGTGCTG2	0,479547196	2,219476679	0,405477074	0,714325982	LADA vs T2D Time-adjusted
hsa-miR-342-3p_TCACACAGAAATCGCACCCGTC2	-0,467914524	2,415699371	0,407505888	0,715695241	LADA vs T2D Time-adjusted
hsa-miR-28-3p_CACTAGATTGTGAGCTCCTGGAG2	-0,435785248	3,068939292	0,408825826	0,715695241	LADA vs T2D Time-adjusted
hsa-let-7a-5p_TGAGGTAGTAGGTTGTATAGT2	0,07515265	11,89008946	0,408968709	0,715695241	LADA vs T2D Time-adjusted
hsa-miR-125b-5p_TCCCTGAGACCCTAACTTGT2	0,124925706	9,835959153	0,410144919	0,716169164	LADA vs T2D Time-adjusted
hsa-miR-625-3p_GACTATAGAACTTTCCCCCTCA2	-0,637833961	3,448522418	0,412186158	0,71757503	LADA vs T2D Time-adjusted
hsa-miR-183-5p_TATGGCACTGGTAGAATTCACTG2	-0,463016023	3,607840844	0,415957306	0,71757503	LADA vs T2D Time-adjusted

hsa-miR-103a-3p_AGCAGCATTGTACAGGGCTAT2	-0,553220441	3,194453722	0,416540245	0,71757503	LADA vs T2D Time-adjusted
hsa-miR-92a-3p_TTGCACTTGTCCCGGCCTGT2	-0,126946229	8,379067596	0,417541181	0,71757503	LADA vs T2D Time-adjusted
hsa-miR-483-5p_AAGACGGGAGGAAAGAAGGGA2	-0,417827989	4,912658385	0,419368884	0,71757503	LADA vs T2D Time-adjusted
hsa-miR-181a-5p_AACATTCAACGCTGTCGGTGAGT2	-0,156529358	8,108797195	0,419381336	0,71757503	LADA vs T2D Time-adjusted
hsa-miR-29b-3p_TAGCACCATTTGAAATCAGT2	-0,386482504	4,397473372	0,420379844	0,71757503	LADA vs T2D Time-adjusted
hsa-miR-532-3p_CCTCCCACACCCAAGGCTTG2	-0,462842114	2,771296807	0,420910124	0,71757503	LADA vs T2D Time-adjusted
hsa-miR-122-5p_GGAGTGTGACAATGGTGTTT2	-0,273246193	7,754720279	0,420972398	0,71757503	LADA vs T2D Time-adjusted
hsa-miR-150-5p_TCTCCCAACCCTTGTACCAGT2	0,15219126	10,28667682	0,422912862	0,71757503	LADA vs T2D Time-adjusted
hsa-miR-125b-5p_TCCCTGAGACCCTAACTTG2	0,143646395	8,160127514	0,423408385	0,71757503	LADA vs T2D Time-adjusted
hsa-miR-1908-5p_CGGCGGGGACGGCGATTGGT2	-0,491468572	2,728932328	0,424196802	0,71757503	LADA vs T2D Time-adjusted
hsa-miR-30e-5p_TGTAAACATCCTTGACTGGA2	-0,440678982	2,526545713	0,424679216	0,71757503	LADA vs T2D Time-adjusted
hsa-miR-3158-3p_AAGGGCTTCCTCTCTGCAGGA2	-0,464697628	2,786574243	0,426169192	0,71757503	LADA vs T2D Time-adjusted
hsa-miR-342-3p_TCTCACACAGAAATCGCACCCGT2	-0,165621064	8,723994136	0,426553302	0,71757503	LADA vs T2D Time-adjusted
hsa-miR-92a-3p_ACTTGTCCCGGCCTGT2	0,448147635	2,211098977	0,426934167	0,71757503	LADA vs T2D Time-adjusted
hsa-let-7e-5p_TGAGGTAGGAGGTTGTATAGT2	-0,340310233	4,76183341	0,427218015	0,71757503	LADA vs T2D Time-adjusted
hsa-miR-150-3p_CTGGTACAGGCCTGGGGGACA2	-0,422489002	4,065180007	0,430420027	0,71757503	LADA vs T2D Time-adjusted
hsa-miR-193a-5p_TGGGTCTTTGCGGGCGAGATGA2	-0,133981764	8,658900701	0,430533409	0,71757503	LADA vs T2D Time-adjusted
hsa-miR-4433b-5p_TGTCCCACCCCCACTCCTG2	0,477623748	2,575504162	0,430586183	0,71757503	LADA vs T2D Time-adjusted
hsa-miR-339-5p_TCCCTGTCCTCCAGGAGCTCACG2	-0,455741426	3,320095685	0,430785854	0,71757503	LADA vs T2D Time-adjusted
hsa-miR-191-5p_CAACGGAATCCCAAAAGCAG2	0,094871145	10,17364723	0,430907888	0,71757503	LADA vs T2D Time-adjusted
hsa-miR-30b-5p_TGTAAACATCCTACACTCAGCT2	-0,205270108	6,702165067	0,432168536	0,718162421	LADA vs T2D Time-adjusted
hsa-miR-423-3p_AGCTCGGTCTGAGGCCCCTCAGT2	-0,095209934	11,77370697	0,43673526	0,722407178	LADA vs T2D Time-adjusted
hsa-miR-27a-3p_TCACAGTGGCTAAGTTCCG2	0,417910311	2,793419478	0,439520907	0,722407178	LADA vs T2D Time-adjusted
hsa-let-7d-3p_ATACGACCTGCTGCCTTTCT2	-0,434193513	2,005732528	0,440684356	0,722407178	LADA vs T2D Time-adjusted
hsa-let-7i-5p_TGAGGTAGTAGTTTGTGCTGTT2	-0,118355339	10,46534644	0,440763828	0,722407178	LADA vs T2D Time-adjusted
hsa-miR-125b-5p_CCCTGAGACCCTAACTTGT2	-0,433835055	2,224797164	0,441050442	0,722407178	LADA vs T2D Time-adjusted
hsa-miR-23a-5p_GGGGTTCCTGGGGATGGGATTT2	0,401633197	3,892718076	0,44119779	0,722407178	LADA vs T2D Time-adjusted
hsa-miR-320a-3p_AAAAGCTGGGTTGAGAGGGCG2	-0,094449142	10,10122965	0,441704479	0,722407178	LADA vs T2D Time-adjusted
hsa-let-7g-5p_TGAGGTAGTAGTTTGTACAGTT2	0,092848037	11,2336953	0,442029171	0,722407178	LADA vs T2D Time-adjusted
hsa-miR-425-5p_AATGACACGATCACTCCCGTTGAG2	-0,296262439	5,989511543	0,444859481	0,723634042	LADA vs T2D Time-adjusted
hsa-miR-92a-3p_TATTGCACTTGTCCCGGCCTGTT2	0,096410045	10,36371218	0,444971393	0,723634042	LADA vs T2D Time-adjusted
hsa-let-7f-5p_GAGGTAGTAGATTGTATAGTT2	-0,271107893	5,929108602	0,445524372	0,723634042	LADA vs T2D Time-adjusted
hsa-miR-4446-3p_CAGGGCTGGCAGTGACATGGGT2	-0,461515316	3,595995315	0,44717834	0,724605964	LADA vs T2D Time-adjusted
hsa-miR-92b-3p_TATTGCACTCGTCCCGGCCT2	-0,314440865	5,642753779	0,447954888	0,724605964	LADA vs T2D Time-adjusted
hsa-miR-191-5p_CAACGGAATCCCAAAAGCA2	-0,22684386	6,200760212	0,453021299	0,729669686	LADA vs T2D Time-adjusted
hsa-miR-24-3p_TGGCTCAGTTCAGCAGGAACAG2	-0,076526285	12,80068155	0,453842148	0,729669686	LADA vs T2D Time-adjusted
hsa-miR-191-5p_ACGGAATCCCAAAAGCAGCT2	-0,41880483	1,917089883	0,455077406	0,729669686	LADA vs T2D Time-adjusted
hsa-miR-142-5p_CCATAAAGTAGAAAGCACT2	-0,279603496	4,725847494	0,455586711	0,729669686	LADA vs T2D Time-adjusted
hsa-miR-139-5p_TCTACAGTGCACGTGTCTCCAGT2	-0,186636381	6,693764684	0,455968179	0,729669686	LADA vs T2D Time-adjusted
hsa-miR-501-3p_AATGCACCCGGGCAAGGAT2	-0,422200297	2,776935459	0,456620094	0,729669686	LADA vs T2D Time-adjusted
hsa-miR-30d-5p_TGTAAACATCCCCGACTGGAAG2	-0,066395997	12,1725605	0,459896928	0,73268697	LADA vs T2D Time-adjusted
hsa-miR-361-3p_CCCCCAGGTGTGATTCTGATTTG2	0,419447751	1,935808496	0,460517291	0,73268697	LADA vs T2D Time-adjusted
hsa-miR-342-5p_AGGGGTGCTATCTGTGATTGA2	-0,424016276	4,01644439	0,462112163	0,73268697	LADA vs T2D Time-adjusted
hsa-miR-126-5p_ATTATTACTTTTGGTACGCGCT2	-0,405314855	2,229095104	0,463709523	0,73268697	LADA vs T2D Time-adjusted
hsa-miR-323b-3p_CCCAATACACGGTCGACCTCT2	-0,346833525	5,843242142	0,464858203	0,73268697	LADA vs T2D Time-adjusted
hsa-miR-128-3p_TCACAGTGAACCGGTCTCTTTT2	-0,304934122	5,206370676	0,465268051	0,73268697	LADA vs T2D Time-adjusted
hsa-miR-342-3p_TCACACAGAAATCGCACCCGT2	0,40236654	2,497948576	0,465754443	0,73268697	LADA vs T2D Time-adjusted
hsa-miR-150-5p_GTCTCCCAACCCTTGTACCAGT2	-0,390382262	2,385284805	0,465974302	0,73268697	LADA vs T2D Time-adjusted
hsa-miR-4685-3p_TCTCCCTTCCTGCCCTGGCT2	0,346023799	4,82214565	0,466945189	0,73268697	LADA vs T2D Time-adjusted
hsa-miR-23a-3p_ATCACATTGCCAGGGATTTCC2	-0,07189897	11,11444486	0,468115626	0,73268697	LADA vs T2D Time-adjusted
hsa-miR-29c-3p_TAGCACCATTTGAAATCGGTT2	-0,326659633	3,979110528	0,468697354	0,73268697	LADA vs T2D Time-adjusted
hsa-miR-486-5p_TCCTGTACTGAGCTGCCCCGA2	0,114439563	17,02608896	0,471666167	0,735873644	LADA vs T2D Time-adjusted
hsa-let-7c-5p_TGAGGTAGTAGGTTGTATGGT2	-0,242970764	4,826004461	0,474056757	0,73814743	LADA vs T2D Time-adjusted
hsa-miR-130b-5p_ACTCTTTCCCTGTTGCACTACT2	-0,263862965	5,624339176	0,477474613	0,742008681	LADA vs T2D Time-adjusted
hsa-miR-221-3p_AGCTACATTGTCTGCTGGGTTT2	-0,127207094	7,93162947	0,479728808	0,742639122	LADA vs T2D Time-adjusted
hsa-miR-363-3p_ATTGCACGGTATCCATCTG2	-0,399004203	2,190954814	0,481236578	0,742639122	LADA vs T2D Time-adjusted
hsa-let-7f-5p_GAGGTAGTAGATTGTATAGT2	-0,410838181	3,007465312	0,482107857	0,742639122	LADA vs T2D Time-adjusted
hsa-let-7g-5p_GAGGTAGTAGTTTGTACAGTT2	-0,395409351	4,325250462	0,485361803	0,742639122	LADA vs T2D Time-adjusted
hsa-let-7a-5p_ATGAGGTAGTAGGTTGTATAGTT2	-0,407473784	2,145462754	0,485404873	0,742639122	LADA vs T2D Time-adjusted
hsa-miR-15a-5p_TAGCAGCACATAATGGTTTGT2	0,230409858	6,55286919	0,485745307	0,742639122	LADA vs T2D Time-adjusted
hsa-miR-4433b-5p_TATGTCCCACCCCCACTCCTGT2	-0,357428003	5,223541258	0,485766218	0,742639122	LADA vs T2D Time-adjusted

hsa-miR-425-5p_ATGACACGATCACTCCCGTTGAGT2	0,354999745	4,481314722	0,486663673	0,742639122	LADA vs T2D Time-adjusted
hsa-miR-142-5p_CATAAAGTAGAAAGCACT2	-0,208504191	5,448401815	0,487174825	0,742639122	LADA vs T2D Time-adjusted
hsa-miR-485-5p_AGAGGCTGGCCGTGATGAATTC2	-0,42619181	2,188589843	0,487268905	0,742639122	LADA vs T2D Time-adjusted
hsa-miR-1306-5p_CCACCTCCCCTGCAAACGTCCA2	0,403436486	3,045463797	0,488336877	0,742835518	LADA vs T2D Time-adjusted
hsa-miR-486-5p_TCCTGTACTGAGCTGCCCCG2	0,116635691	14,12849188	0,491005111	0,744527766	LADA vs T2D Time-adjusted
hsa-miR-423-3p_GCTCGGTCTGAGGCCCCTCAGT2	-0,214568391	6,437706495	0,491331851	0,744527766	LADA vs T2D Time-adjusted
hsa-miR-22-3p_AAGCTGCCAGTTGAAGAACT2	-0,090057359	10,281857	0,493338453	0,744862079	LADA vs T2D Time-adjusted
hsa-miR-10a-5p_ACCCTGTAGATCCGAATTTG2	0,23178611	5,676118316	0,493435815	0,744862079	LADA vs T2D Time-adjusted
hsa-miR-16-2-3p_CCAATATTACTGTGCTGCTTT2	-0,301373629	4,408566959	0,497035833	0,748867322	LADA vs T2D Time-adjusted
hsa-miR-23a-3p_TCACATTGCCAGGGATTTC2	-0,381080393	2,507144033	0,500457765	0,752589529	LADA vs T2D Time-adjusted
hsa-miR-29a-3p_TAGCACCATCTGAAATCGGTTAT2	-0,367679564	2,458427946	0,502905359	0,754044144	LADA vs T2D Time-adjusted
hsa-miR-423-5p_AGGGGCAGAGAGCGAGACTTT2	0,266218184	5,179351064	0,503331616	0,754044144	LADA vs T2D Time-adjusted
hsa-miR-101-3p_GTACAGTACTGTGATAACTGA2	-0,108682612	8,212735415	0,504645792	0,754583783	LADA vs T2D Time-adjusted
hsa-miR-145-3p_ATTCCTGGAAATACTGTTCT2	-0,376496466	3,093740679	0,508755583	0,75929371	LADA vs T2D Time-adjusted
hsa-miR-92a-3p_TATTGCACTTGTCCCGGC2	-0,172441367	6,622543799	0,514965516	0,765129631	LADA vs T2D Time-adjusted
hsa-miR-493-5p_TTGTACATGGTAGGCTTTCATT2	0,375257747	3,42834388	0,515303027	0,765129631	LADA vs T2D Time-adjusted
hsa-miR-16-2-3p_ACCAATATTACTGTGCTGCTT2	-0,113194962	8,335786686	0,517123907	0,765129631	LADA vs T2D Time-adjusted
hsa-let-7i-5p_TGAGGTAGTAGTTTGTGCTGT2	-0,076419719	11,42837325	0,517481825	0,765129631	LADA vs T2D Time-adjusted
hsa-miR-29c-3p_TAGCACCATTTGAAATCGGT2	-0,3239892	4,535898634	0,517502342	0,765129631	LADA vs T2D Time-adjusted
hsa-miR-23a-3p_TCACATTGCCAGGGATTTCCA2	0,259115005	5,160556193	0,519180814	0,766179149	LADA vs T2D Time-adjusted
hsa-let-7d-5p_AGAGGTAGTAGGTTGCATAGTTT2	-0,329405235	5,258609386	0,521231163	0,767772533	LADA vs T2D Time-adjusted
hsa-miR-1-3p_TGGAATGTAAAGAAGTATGTAT2	0,33214956	5,884629396	0,525420799	0,771003947	LADA vs T2D Time-adjusted
hsa-miR-375-3p_TTTGTTCGTTCGGCTCGCGTGA2	-0,15236771	10,24220516	0,526887745	0,771003947	LADA vs T2D Time-adjusted
hsa-miR-18a-3p_ACTGCCCTAAGTGCTCCTTCT2	0,354659976	3,355078821	0,528052356	0,771003947	LADA vs T2D Time-adjusted
hsa-miR-423-5p_AGGGGCAGAGAGCGAGACTTTT2	-0,281965212	5,009061922	0,528096467	0,771003947	LADA vs T2D Time-adjusted
hsa-miR-7-5p_TGGAAGACTAGTGATTTTGT2	-0,319233088	4,179429072	0,528298532	0,771003947	LADA vs T2D Time-adjusted
hsa-miR-486-5p_TCCTGTACTGAGCTGCCCCGAGC2	-0,14630976	6,153079419	0,530583738	0,772912959	LADA vs T2D Time-adjusted
hsa-miR-21-5p_TAGCTTATCAGACTGATGTTGACT2	0,29836271	5,746314182	0,535597213	0,778404483	LADA vs T2D Time-adjusted
hsa-miR-24-3p_TGGCTCAGTTCAGCAGGAA2	-0,248667832	5,597444609	0,536654693	0,778404483	LADA vs T2D Time-adjusted
hsa-miR-1249-3p_ACGCCCTTCCCCCCCTTCTTCA2	0,375765072	2,093528347	0,537305749	0,778404483	LADA vs T2D Time-adjusted
hsa-miR-501-3p_AATGCACCCGGGCAAGGATT2	-0,161625445	6,566869383	0,542278664	0,783677347	LADA vs T2D Time-adjusted
hsa-miR-181a-5p_AACATTCAACGCTGTCGGT2	-0,233199514	5,49798078	0,54329152	0,783677347	LADA vs T2D Time-adjusted
hsa-let-7i-5p_GAGGTAGTAGTTTGTGCTGT2	-0,271971665	4,86484784	0,543917653	0,783677347	LADA vs T2D Time-adjusted
hsa-let-7a-5p_TGAGGTAGTAGGTTGTATAGTTT2	0,127445825	9,339383804	0,547509028	0,786074391	LADA vs T2D Time-adjusted
hsa-miR-342-3p_CTCACACAGAAATCGCACCCG2	0,355337919	2,546589991	0,547910928	0,786074391	LADA vs T2D Time-adjusted
hsa-miR-182-5p_TTTGGCAATGGTAGAACTCAC2	-0,334286102	3,088555901	0,54856266	0,786074391	LADA vs T2D Time-adjusted
hsa-miR-148a-3p_TCAGTGCACTACAGAACTTT2	-0,340994274	4,13799763	0,55177493	0,78865085	LADA vs T2D Time-adjusted
hsa-miR-23a-3p_TCACATTGCCAGGGATTTCC2	-0,299136137	4,52192031	0,552354704	0,78865085	LADA vs T2D Time-adjusted
hsa-miR-30e-5p_TGTAAACATCCTTGACTGGAAGC2	-0,069580414	8,361780675	0,555141436	0,791201578	LADA vs T2D Time-adjusted
hsa-miR-30a-5p_TGTAAACATCCTCGACTGGA2	0,30931156	3,318329214	0,557516411	0,79315734	LADA vs T2D Time-adjusted
hsa-miR-30e-3p_CTTTCAGTCGGATGTTTACAGC2	-0,332189279	3,112145052	0,559350557	0,793546268	LADA vs T2D Time-adjusted
hsa-miR-423-5p_TGAGGGGCAGAGAGCGAGACTTTT2	-0,05988275	11,36605873	0,560580319	0,793546268	LADA vs T2D Time-adjusted
hsa-miR-27b-3p_TTCACAGTGGCTAAGTTCT2	-0,106900381	8,397674944	0,560799449	0,793546268	LADA vs T2D Time-adjusted
hsa-miR-191-5p_CAACGGAATCCCAAAAGCAGCTG2	-0,098858276	9,63370255	0,564808684	0,79695628	LADA vs T2D Time-adjusted
hsa-miR-27a-3p_TTCACAGTGGCTAAGTTCC2	-0,205974217	5,362754207	0,565797257	0,79695628	LADA vs T2D Time-adjusted
hsa-miR-191-5p_CAACGGAATCCCAAAAGCAGCT2	0,068594	11,08359666	0,566231896	0,79695628	LADA vs T2D Time-adjusted
hsa-miR-23a-3p_TCACATTGCCAGGGATTTCCAAC2	-0,296581638	1,909179417	0,573082689	0,805165909	LADA vs T2D Time-adjusted
hsa-miR-128-3p_TCACAGTGAACCGGTCTCTT2	-0,132739317	6,752700404	0,576410897	0,807469511	LADA vs T2D Time-adjusted
hsa-miR-15b-5p_TAGCAGCACATCATGGTTT2	-0,104788306	8,509089064	0,577734567	0,807469511	LADA vs T2D Time-adjusted
hsa-miR-652-3p_AATGGCGCCACTAGGGTTG2	-0,295334071	4,5440088	0,577784757	0,807469511	LADA vs T2D Time-adjusted
hsa-miR-144-3p_CTACAGTATAGATGATGTAC2	0,291379452	3,365989189	0,580864764	0,810342202	LADA vs T2D Time-adjusted
hsa-miR-335-5p_TCAAGAGCAATAACGAAAAATG2	-0,108420765	8,353751053	0,582205083	0,810473695	LADA vs T2D Time-adjusted
hsa-miR-101-3p_GTACAGTACTGTGATAACTGAA2	-0,298955445	3,734790611	0,583008258	0,810473695	LADA vs T2D Time-adjusted
hsa-miR-26b-5p_TTCAAGTAATTCAGGATAGGTT2	0,096367643	9,792607635	0,588218941	0,815903845	LADA vs T2D Time-adjusted
hsa-let-7d-5p_AGAGGTAGTAGGTTGCATAGTT2	0,107090017	9,029809844	0,588977365	0,815903845	LADA vs T2D Time-adjusted
hsa-miR-486-5p_CCTGTACTGAGCTGCCCCG2	0,121840852	8,001774251	0,595768834	0,823869139	LADA vs T2D Time-adjusted
hsa-miR-144-5p_GATATCATCATATACTGTAAGTT2	0,310731972	2,274281107	0,599345909	0,826692953	LADA vs T2D Time-adjusted
hsa-let-7a-5p_GTGAGGTAGTAGGTTGTATAGTT2	0,314029829	3,003012908	0,601304388	0,826692953	LADA vs T2D Time-adjusted
hsa-miR-654-3p_TATGTCTGCTGACCATCACC2	-0,318940372	2,446876993	0,60239362	0,826692953	LADA vs T2D Time-adjusted
hsa-miR-21-5p_AGCTTATCAGACTGATGTTGA2	-0,111743763	6,713348932	0,605821743	0,826692953	LADA vs T2D Time-adjusted
hsa-miR-10b-5p_ACCCTGTAGAACCGAATTTGTG2	0,146772142	6,035000543	0,605908223	0,826692953	LADA vs T2D Time-adjusted

hsa-miR-193b-5p_CGGGGTTTTGAGGGCGAGATGA2	-0,308807578	2,359786934	0,607172751	0,826692953	LADA vs T2D Time-adjusted
hsa-miR-26a-5p_TTCAAGTAATCCAGGATAGG2	-0,114076391	6,804916619	0,607437338	0,826692953	LADA vs T2D Time-adjusted
hsa-miR-4433b-5p_TGTCCCACCCCCACTCCTGTTT2	0,186746184	8,44296889	0,608068276	0,826692953	LADA vs T2D Time-adjusted
hsa-miR-182-5p_TTTGGCAATGGTAGAACTCACACT2	0,123869664	7,646583987	0,608348987	0,826692953	LADA vs T2D Time-adjusted
hsa-miR-664a-5p_ACTGGCTAGGGAAAATGATTGG2	-0,267830058	3,605476953	0,608756843	0,826692953	LADA vs T2D Time-adjusted
hsa-miR-130a-3p_CAGTGCAATGTTAAAAGGGCA2	-0,223046907	4,392768352	0,609307196	0,826692953	LADA vs T2D Time-adjusted
hsa-miR-323a-3p_GCACATTACACGGTCGACCTCT2	-0,280538225	3,791926149	0,612611088	0,829752347	LADA vs T2D Time-adjusted
hsa-miR-223-3p_TGTCAGTTTGTCAAATACCCC2	-0,150109838	7,234009162	0,615776947	0,832614641	LADA vs T2D Time-adjusted
hsa-miR-140-5p_CAGTGGTTTTACCCTATGGTAG2	-0,22574164	3,907116242	0,617187882	0,833098319	LADA vs T2D Time-adjusted
hsa-miR-93-5p_AAAGTGCTGTTCGTGCAGGTAG2	-0,243866772	3,678777222	0,621668583	0,83771695	LADA vs T2D Time-adjusted
hsa-miR-423-5p_TGAGGGGCAGAGAGCGAGACTT2	0,048330194	12,27072707	0,6262939	0,842514414	LADA vs T2D Time-adjusted
hsa-miR-126-3p_CGTACCGTGAGTAATAATGCG2	0,067175504	9,728392522	0,628471911	0,842986324	LADA vs T2D Time-adjusted
hsa-miR-92a-3p_ATTGCACTTGTCCCGGCCTGT2	0,057139693	12,30748627	0,628776146	0,842986324	LADA vs T2D Time-adjusted
hsa-miR-26b-5p_TCAAGTAATTCAGGATAGGTT2	-0,25689538	3,617629587	0,633545439	0,843294368	LADA vs T2D Time-adjusted
hsa-miR-223-5p_CGTGTATTTGACAAGCTGAGTTGG2	0,28603886	2,254955516	0,63393203	0,843294368	LADA vs T2D Time-adjusted
hsa-miR-150-5p_CTCCCAACCCTTGTACCAGT2	0,278683656	3,109098502	0,634081271	0,843294368	LADA vs T2D Time-adjusted
hsa-miR-654-5p_TGGTGGGCCGCAGAACATGTGC2	0,300461027	3,180891958	0,634144491	0,843294368	LADA vs T2D Time-adjusted
hsa-miR-3613-5p_TGTTGTACTTTTTTTTTTGT2	-0,082054741	7,575367225	0,635013751	0,843294368	LADA vs T2D Time-adjusted
hsa-miR-451a_AAACCGTTACCATTACTG2	-0,2415198	2,543007428	0,635402583	0,843294368	LADA vs T2D Time-adjusted
hsa-miR-433-3p_ATCATGATGGGCTCCTCGGTGT2	0,279784262	4,238564116	0,636650815	0,84353567	LADA vs T2D Time-adjusted
hsa-miR-323a-3p_CACATTACACGGTCGACCTCT2	0,276533262	3,386474425	0,641404914	0,848413524	LADA vs T2D Time-adjusted
hsa-miR-30e-5p_GTAAACATCCTTGACTGGAAGCT2	0,052819045	8,789096373	0,642487443	0,848426657	LADA vs T2D Time-adjusted
hsa-miR-92a-3p_TATTGCACTTGTCCCGGCCTGTG2	-0,078095811	7,12439601	0,644870186	0,849812049	LADA vs T2D Time-adjusted
hsa-miR-224-5p_CAAGTCACTAGTGGTTCCGTTT2	-0,262246986	2,890382654	0,645685261	0,849812049	LADA vs T2D Time-adjusted
hsa-miR-21-5p_AGCTTATCAGACTGATGTTG2	-0,157600842	5,242282712	0,650856373	0,851764002	LADA vs T2D Time-adjusted
hsa-miR-15a-5p_TAGCAGCACATAATGGTTTG2	0,322056984	2,765762945	0,650922042	0,851764002	LADA vs T2D Time-adjusted
hsa-miR-99b-5p_CACCCGTAGAACCGACCTT2	-0,254353277	2,15225541	0,651954435	0,851764002	LADA vs T2D Time-adjusted
hsa-miR-128-3p_TCACAGTGAACCGGTCTCTTT2	-0,060029017	8,165938943	0,65363261	0,851764002	LADA vs T2D Time-adjusted
hsa-miR-486-5p_TCCTGTACTGAGCTGCCC2	-0,17275907	3,876048404	0,654371866	0,851764002	LADA vs T2D Time-adjusted
hsa-miR-222-3p_AGCTACATCTGGCTACTGGG2	-0,255340497	2,571182702	0,655625045	0,851764002	LADA vs T2D Time-adjusted
hsa-miR-145-3p_ATTCCTGGAAATACTGTTCTT2	0,248508062	3,68129034	0,65637745	0,851764002	LADA vs T2D Time-adjusted
hsa-miR-409-3p_AATGTTGCTCGGTGAACCCCT2	-0,263716535	4,168161381	0,657688877	0,851764002	LADA vs T2D Time-adjusted
hsa-let-7f-5p_GAGGTAGTAGATTGTATAG2	-0,234986495	3,124116914	0,658342064	0,851764002	LADA vs T2D Time-adjusted
hsa-miR-374a-5p_TTATAATACAACCTGATAAGT2	0,254639048	2,272272471	0,659310281	0,851764002	LADA vs T2D Time-adjusted
hsa-miR-99a-5p_AACCCGTAGATCCGATCTT2	-0,257030866	3,162993064	0,660214907	0,851764002	LADA vs T2D Time-adjusted
hsa-miR-374a-5p_TTATAATACAACCTGATAAGTG2	-0,186982334	4,298678316	0,660768528	0,851764002	LADA vs T2D Time-adjusted
hsa-miR-485-5p_AGAGGCTGGCCGTGATGAATTCG2	-0,235010101	4,445743314	0,662057473	0,851764002	LADA vs T2D Time-adjusted
hsa-miR-329-3p_AACACACCTGGTTAACCTCTT2	0,245743461	3,530220069	0,66224382	0,851764002	LADA vs T2D Time-adjusted
hsa-miR-30c-5p_TGTAAACATCCTACACTCTCAG2	-0,189200299	4,704062458	0,669916684	0,860233924	LADA vs T2D Time-adjusted
hsa-miR-486-5p_CCTGTACTGAGCTGCCCCGA2	0,0694715	11,06703044	0,673657223	0,86363511	LADA vs T2D Time-adjusted
hsa-miR-3615_TCTCTCGGCTCCTCGCGGCTC2	0,217955894	2,389605161	0,675889524	0,865094844	LADA vs T2D Time-adjusted
hsa-miR-103a-3p_AGCAGCATTGTACAGGGCT2	0,343486148	5,276241931	0,678230723	0,866689018	LADA vs T2D Time-adjusted
hsa-miR-28-5p_AAGGAGCTCACAGTCTATTGA2	-0,241632316	3,153941235	0,681746609	0,868355625	LADA vs T2D Time-adjusted
hsa-miR-486-5p_ATCCTGTACTGAGCTGCCCCGA2	0,074962861	9,024331858	0,682771147	0,868355625	LADA vs T2D Time-adjusted
hsa-miR-329-3p_AACACACCTGGTTAACCTCTTT2	0,233595991	3,551980192	0,682828317	0,868355625	LADA vs T2D Time-adjusted
hsa-miR-486-5p_CTGTACTGAGCTGCCCCGAG2	-0,088324328	7,807179841	0,688130872	0,873694253	LADA vs T2D Time-adjusted
hsa-miR-150-5p_TCTCCCAACCCTTGTACCA2	0,141952207	6,56516459	0,695309446	0,88006275	LADA vs T2D Time-adjusted
hsa-miR-142-3p_TGTAGTGTTTCCTACTTTATGGA2	-0,208851398	3,574791071	0,695371958	0,88006275	LADA vs T2D Time-adjusted
hsa-miR-451a_AAACCGTTACCATTACTGAGT2	-0,062522416	16,52329399	0,703224503	0,888579204	LADA vs T2D Time-adjusted
hsa-miR-30a-5p_TGTAAACATCCTCGACTGG2	-0,175507424	4,667345631	0,706767021	0,891631122	LADA vs T2D Time-adjusted
hsa-miR-144-5p_GGATATCATCATATACTGTAAG2	-0,208219892	4,158150293	0,707966819	0,891722538	LADA vs T2D Time-adjusted
hsa-let-7f-5p_TGAGGTAGTAGATTGTATAGT2	-0,035422785	10,58330635	0,713456991	0,895869714	LADA vs T2D Time-adjusted
hsa-miR-191-5p_AACGGAATCCCAAAAGCAGC2	-0,197341516	2,971417206	0,715223542	0,895869714	LADA vs T2D Time-adjusted
hsa-miR-30a-5p_TGTAAACATCCTCGACTGGAA2	-0,076686287	7,206633348	0,715935786	0,895869714	LADA vs T2D Time-adjusted
hsa-miR-451a_AACCGTTACCATTACTGAGTTT2	-0,202626623	2,393187549	0,716069565	0,895869714	LADA vs T2D Time-adjusted
hsa-miR-92a-3p_GTATTGCACTTGTCCCGGCCTGT2	0,067922016	7,697377602	0,716922287	0,895869714	LADA vs T2D Time-adjusted
hsa-miR-126-3p_TCGTACCGTGAGTAATAATGCG2	0,045946884	11,26241255	0,718626576	0,896582999	LADA vs T2D Time-adjusted
hsa-miR-501-3p_ATGCACCCGGGCAAGGATTCT2	-0,195560364	2,108861011	0,719925064	0,896788544	LADA vs T2D Time-adjusted
hsa-miR-150-5p_CTCCCAACCCTTGTACCAGTG2	0,158229816	4,444846731	0,723794477	0,898678309	LADA vs T2D Time-adjusted
hsa-miR-21-5p_TAGCTTATCAGACTGATGT2	-0,168560982	4,642902172	0,724149652	0,898678309	LADA vs T2D Time-adjusted
hsa-let-7d-3p_TATACGACCTGCTGCCTTTC2	-0,09745969	6,350176431	0,72485052	0,898678309	LADA vs T2D Time-adjusted

hsa-miR-424-3p_CAAAACGTGAGGCGCTGCTAT2	0,112960507	5,546308926	0,727037775	0,899635876	LADA vs T2D Time-adjusted
hsa-let-7f-5p_TGAGGTAGTAGATTGTATA2	-0,178662243	3,718372609	0,727897548	0,899635876	LADA vs T2D Time-adjusted
hsa-miR-26a-5p_TTCAAGTAATCCAGGATAGGCT2	0,054630058	11,89898908	0,729174544	0,899808213	LADA vs T2D Time-adjusted
hsa-miR-10b-5p_ACCCTGTAGAACCGAATTTG2	-0,162368258	4,807768063	0,731490676	0,900456439	LADA vs T2D Time-adjusted
hsa-miR-23a-5p_GGGGTTCCTGGGGATGGGATT2	-0,179814999	3,763349774	0,731976599	0,900456439	LADA vs T2D Time-adjusted
hsa-miR-106b-5p_TAAAGTGCTGACAGTGCAGAT2	0,176570369	2,555392208	0,735413522	0,901133453	LADA vs T2D Time-adjusted
hsa-miR-23b-3p_ATCACATTGCCAGGGATTACCA2	-0,091816947	6,519069588	0,736830996	0,901133453	LADA vs T2D Time-adjusted
hsa-miR-126-3p_CTCGTACCGTGAGTAATAATGCG2	0,168599464	4,641889164	0,737136293	0,901133453	LADA vs T2D Time-adjusted
hsa-miR-30e-5p_GTAAACATCCTTGACTGGAAGC2	-0,191982762	2,516119605	0,737342852	0,901133453	LADA vs T2D Time-adjusted
hsa-miR-423-5p_GAGGGGCAGAGAGCGAGACTTTT2	-0,169869559	3,8847694	0,738646426	0,901133453	LADA vs T2D Time-adjusted
hsa-miR-145-5p_GTCCAGTTTTCCCAGGAATCCC2	-0,18624215	3,263854619	0,73936234	0,901133453	LADA vs T2D Time-adjusted
hsa-miR-382-5p_GAAGTTGTTCGTGGTGGATTCG2	-0,117298508	7,539547941	0,746079955	0,907921914	LADA vs T2D Time-adjusted
hsa-miR-451a_AACCGTTACCATTACTGAG2	-0,062885227	8,086006518	0,748813771	0,909446572	LADA vs T2D Time-adjusted
hsa-miR-483-3p_TCACTCCTCTCCTCCCGTCT2	0,193185227	2,17353874	0,74963232	0,909446572	LADA vs T2D Time-adjusted
hsa-miR-340-5p_TTATAAAGCAATGAGACTGATT2	-0,112589843	5,720859509	0,754918616	0,909999355	LADA vs T2D Time-adjusted
hsa-miR-197-3p_TTCACCACCTTCTCCACCCA2	0,165422924	3,766113409	0,755718513	0,909999355	LADA vs T2D Time-adjusted
hsa-miR-486-5p_TCCTGTACTGAGCTGCCCCGAG2	-0,046878579	17,20272224	0,757537842	0,909999355	LADA vs T2D Time-adjusted
hsa-miR-29a-3p_TAGCACCATCTGAAATCGGTT2	0,05761206	8,673627549	0,758983223	0,909999355	LADA vs T2D Time-adjusted
hsa-miR-98-5p_TGAGGTAGTAAGTTGTATTGT2	-0,095920697	6,564741993	0,758997652	0,909999355	LADA vs T2D Time-adjusted
hsa-miR-664a-3p_TATTCATTTATCCCCAGCCTACA2	0,165411058	2,990893166	0,759356023	0,909999355	LADA vs T2D Time-adjusted
hsa-miR-26a-5p_TTCAAGTAATCCAGGATAGGC2	0,050759253	7,521939438	0,760760298	0,909999355	LADA vs T2D Time-adjusted
hsa-miR-3615_TCTCTCGGCTCCTCGCGGC2	-0,167117045	2,023397327	0,761877284	0,909999355	LADA vs T2D Time-adjusted
hsa-let-7b-3p_CTATACAACCTACTGCCTTCC2	-0,090878781	5,967940681	0,762102178	0,909999355	LADA vs T2D Time-adjusted
hsa-miR-629-5p_TGGGTTTACGTTGGGAGAAC2	-0,165123489	3,80742001	0,762741135	0,909999355	LADA vs T2D Time-adjusted
hsa-miR-191-5p_CAACGGAATCCCAAAAGCAGCTGT2	0,124167081	5,75430435	0,764139729	0,909999355	LADA vs T2D Time-adjusted
hsa-miR-451a_AAACCGTTACCATTACTGAGTTTAGT2	-0,122733728	4,347393724	0,765822272	0,909999355	LADA vs T2D Time-adjusted
hsa-miR-374b-5p_ATATAATACAACCTGCTAAGTG2	-0,162433715	2,591933676	0,766317081	0,909999355	LADA vs T2D Time-adjusted
hsa-miR-22-3p_AAGCTGCCAGTTGAAGAAC2	0,057783217	8,253643329	0,767318597	0,909999355	LADA vs T2D Time-adjusted
hsa-miR-1306-5p_CCACCTCCCCTGCAAACGTC2	-0,150970281	3,895275761	0,767344589	0,909999355	LADA vs T2D Time-adjusted
hsa-miR-30c-5p_TGTAAACATCCTACACTCTCAGCT2	-0,041686699	10,19324434	0,771369231	0,91117003	LADA vs T2D Time-adjusted
hsa-miR-21-5p_TAGCTTATCAGACTGATGTTGA2	0,024170581	12,8658918	0,771566343	0,91117003	LADA vs T2D Time-adjusted
hsa-miR-486-5p_CCTGTACTGAGCTGCCCCGAG2	-0,047678501	11,24764136	0,771908429	0,91117003	LADA vs T2D Time-adjusted
hsa-let-7d-5p_AGAGGTAGTAGGTTGCATAGT2	-0,071559668	7,384387884	0,773776423	0,91117003	LADA vs T2D Time-adjusted
hsa-miR-320a-3p_AAAAGCTGGGTTGAGAGGGCGAA2	0,034194599	9,820743233	0,774091353	0,91117003	LADA vs T2D Time-adjusted
hsa-miR-223-5p_CGTGTATTTGACAAGCTGAGTTG2	-0,10701138	5,926691134	0,776381625	0,911282939	LADA vs T2D Time-adjusted
hsa-miR-629-5p_TGGGTTTACGTTGGGAGAA2	-0,167413743	2,664864204	0,776491405	0,911282939	LADA vs T2D Time-adjusted
hsa-miR-222-3p_AGCTACATCTGGCTACTGGGTCTCT2	-0,072822084	7,874596057	0,780415356	0,91453118	LADA vs T2D Time-adjusted
hsa-miR-221-3p_AGCTACATTGTCTGCTGGGTTTCA2	0,157327767	2,292055081	0,782064161	0,91510762	LADA vs T2D Time-adjusted
hsa-miR-139-5p_TCTACAGTGCACGTGTCTCCA2	-0,115742318	4,89012497	0,783269901	0,915164685	LADA vs T2D Time-adjusted
hsa-miR-320a-3p_AAAAGCTGGGTTGAGAGGGCGA2	-0,029720458	10,81566962	0,78527316	0,91615202	LADA vs T2D Time-adjusted
hsa-miR-125a-5p_TCCCTGAGACCCTTTAACCTG2	-0,048999426	8,335881469	0,791621239	0,91960392	LADA vs T2D Time-adjusted
hsa-miR-92a-3p_TATTGCACTTGTCCCGGCCTG2	0,03971334	13,9436113	0,791993745	0,91960392	LADA vs T2D Time-adjusted
hsa-miR-10a-5p_TACCCTGTAGATCCGAATT2	-0,137656712	3,885245168	0,793727272	0,91960392	LADA vs T2D Time-adjusted
hsa-miR-23a-3p_ATCACATTGCCAGGGATTTC2	0,037512903	9,519525675	0,795660942	0,91960392	LADA vs T2D Time-adjusted
hsa-let-7e-5p_TGAGGTAGGAGGTTGTATAGTT2	0,053009827	7,821946446	0,796946808	0,91960392	LADA vs T2D Time-adjusted
hsa-miR-150-3p_CTGGTACAGGCCTGGGGGAC2	0,142941915	2,452241426	0,797349195	0,91960392	LADA vs T2D Time-adjusted
hsa-miR-99b-5p_CACCCGTAGAACCGACCTTG2	0,061052124	6,823079979	0,798276454	0,91960392	LADA vs T2D Time-adjusted
hsa-miR-361-5p_TTATCAGAATCTCCAGGGG2	-0,137434726	2,375013248	0,79873853	0,91960392	LADA vs T2D Time-adjusted
hsa-miR-206_TGGAATGTAAGGAAGTGTGTGG2	-0,155851733	2,186074312	0,799047413	0,91960392	LADA vs T2D Time-adjusted
hsa-miR-4732-5p_TGTAGAGCAGGGAGCAGGAAG2	0,153485458	2,884522745	0,799857771	0,91960392	LADA vs T2D Time-adjusted
hsa-miR-363-3p_AATTGCACGGTATCCATCTGTA2	-0,11031458	4,05384132	0,801931761	0,920650251	LADA vs T2D Time-adjusted
hsa-miR-361-5p_TTATCAGAATCTCCAGGGGTACT2	0,131291196	2,680756048	0,808154443	0,926449513	LADA vs T2D Time-adjusted
hsa-miR-10b-5p_TACCCTGTAGAACCGAATTT2	-0,091668686	5,284240397	0,813488021	0,931214218	LADA vs T2D Time-adjusted
hsa-miR-10a-5p_TACCCTGTAGATCCGAATTTGTG2	0,053700899	7,331499904	0,816200167	0,931866165	LADA vs T2D Time-adjusted
hsa-miR-423-5p_AGGGGCAGAGAGCGAGACT2	0,131534039	3,23300129	0,816719256	0,931866165	LADA vs T2D Time-adjusted
hsa-miR-146a-5p_TGAGAACTGAATTCCATGGGTT2	-0,024946746	12,18952455	0,818335156	0,931866165	LADA vs T2D Time-adjusted
hsa-miR-370-3p_GCCTGCTGGGGTGGAACCTGGT2	-0,136853803	2,366107994	0,818769892	0,931866165	LADA vs T2D Time-adjusted
hsa-miR-181b-5p_AACATTCATTGCTGTCGGTG2	0,124752038	2,055578696	0,821215437	0,933306625	LADA vs T2D Time-adjusted
hsa-miR-339-3p_TGAGCGCCTCGACGACAGAGC2	-0,105197075	4,202757825	0,824494315	0,93519395	LADA vs T2D Time-adjusted
hsa-miR-10b-5p_TACCCTGTAGAACCGAATTTGTGT2	-0,119804865	2,423805448	0,826152446	0,93519395	LADA vs T2D Time-adjusted
hsa-miR-146a-5p_TGAGAACTGAATTCCATGGG2	-0,115352634	2,326140979	0,826422972	0,93519395	LADA vs T2D Time-adjusted

hsa-miR-125a-5p_TCCCTGAGACCCTTTAACC2	0,114024047	2,3001783	0,831772941	0,935641332	LADA vs T2D Time-adjusted
hsa-miR-145-5p_GTCCAGTTTTCCCAGGAATCC2	-0,11626211	2,346106601	0,833686689	0,935641332	LADA vs T2D Time-adjusted
hsa-miR-92a-3p_ATTGCACTTGTCCCGGCCTG2	0,036938886	9,128354405	0,835134201	0,935641332	LADA vs T2D Time-adjusted
hsa-miR-4732-3p_GCCCTGACCTGTCCTGTTCTG2	0,181886818	4,166204088	0,835353239	0,935641332	LADA vs T2D Time-adjusted
hsa-miR-139-3p_TGGAGACGCGGCCCTGTTGGAGT2	0,115250257	3,616305041	0,836082977	0,935641332	LADA vs T2D Time-adjusted
hsa-miR-148a-3p_TCAGTGCACTACAGAACTT2	-0,122381994	2,313074364	0,836901371	0,935641332	LADA vs T2D Time-adjusted
hsa-miR-423-5p_CTGAGGGGCAGAGAGCGAGACTTT2	0,102106833	4,393029535	0,838301756	0,935641332	LADA vs T2D Time-adjusted
hsa-miR-126-5p_CATTATTACTTTTGGTACGCG2	0,055308378	6,552150257	0,838674588	0,935641332	LADA vs T2D Time-adjusted
hsa-miR-342-3p_TCTCACACAGAAATCGCACCCG2	-0,044256157	9,698837248	0,83966423	0,935641332	LADA vs T2D Time-adjusted
hsa-miR-191-5p_CAACGGAATCCCAAAAGC2	-0,110321038	3,145120995	0,840039686	0,935641332	LADA vs T2D Time-adjusted
hsa-miR-17-5p_CAAAGTGCTTACAGTGCAGGTAG2	-0,108850334	3,22312992	0,840121136	0,935641332	LADA vs T2D Time-adjusted
hsa-miR-423-5p_TGAGGGGCAGAGAGCGAGAC2	-0,020917242	9,81018722	0,841012626	0,935641332	LADA vs T2D Time-adjusted
hsa-let-7f-5p_TGAGGTAGTAGATTGTATAGTTT2	-0,053418848	8,014292524	0,843464695	0,937051368	LADA vs T2D Time-adjusted
hsa-miR-369-3p_AATAATACATGGTTGATCTTT2	-0,115749694	4,128023185	0,847011948	0,939025661	LADA vs T2D Time-adjusted
hsa-miR-340-5p_TTATAAAGCAATGAGACTGAT2	0,107407047	1,903930861	0,847616083	0,939025661	LADA vs T2D Time-adjusted
hsa-miR-30a-5p_TGTAAACATCCTCGACTGGAAGC2	0,034029102	7,811257993	0,850969989	0,941422743	LADA vs T2D Time-adjusted
hsa-miR-139-5p_TCTACAGTGCACGTGTCTCCAG2	-0,073264448	4,962736668	0,852565505	0,941870551	LADA vs T2D Time-adjusted
hsa-miR-126-5p_CATTATTACTTTTGGTACGC2	0,094414641	3,708699797	0,856868962	0,945304531	LADA vs T2D Time-adjusted
hsa-miR-22-3p_AAGCTGCCAGTTGAAGAACTGTT2	-0,094831858	2,813129247	0,861350148	0,948924745	LADA vs T2D Time-adjusted
hsa-let-7a-5p_TGAGGTAGTAGGTTGTATAGTTTT2	-0,09877363	2,919534024	0,862693681	0,949026619	LADA vs T2D Time-adjusted
hsa-miR-125a-5p_CCCTGAGACCCTTTAACCTGT2	0,071050986	5,393774828	0,863842182	0,949026619	LADA vs T2D Time-adjusted
hsa-miR-99b-5p_ACCCGTAGAACCGACCTTGCG2	0,095317742	2,144852077	0,867111847	0,949096578	LADA vs T2D Time-adjusted
hsa-miR-16-5p_CTAGCAGCACGTAAATATTGGCG2	-0,073762837	4,886130646	0,868361976	0,949096578	LADA vs T2D Time-adjusted
hsa-miR-423-5p_CTGAGGGGCAGAGAGCGAGACT2	-0,087721251	2,738837203	0,86986886	0,949096578	LADA vs T2D Time-adjusted
hsa-miR-4433b-5p_TGTCCCACCCCCACTCCTGT2	-0,087642042	4,523145905	0,870382062	0,949096578	LADA vs T2D Time-adjusted
hsa-let-7b-3p_CTATACAACCTACTGCCTTC2	-0,089419007	3,17192614	0,870501404	0,949096578	LADA vs T2D Time-adjusted
hsa-let-7b-5p_GAGGTAGTAGGTTGTGTGG2	0,050909948	6,238396809	0,871405465	0,949096578	LADA vs T2D Time-adjusted
hsa-miR-423-3p_AAGCTCGGTCTGAGGCCCCT2	-0,094495015	2,52885255	0,872304946	0,949096578	LADA vs T2D Time-adjusted
hsa-miR-92a-3p_TATTGCACTTGTCCCGGCCT2	0,025856222	10,29739458	0,873898653	0,949524498	LADA vs T2D Time-adjusted
hsa-miR-150-5p_GTCTCCCAACCCTTGTACCAGTG2	-0,089474088	3,077989788	0,876473608	0,950477071	LADA vs T2D Time-adjusted
hsa-let-7a-5p_TGAGGTAGTAGGTTGTAT2	-0,103042581	3,337435577	0,877178586	0,950477071	LADA vs T2D Time-adjusted
hsa-miR-21-5p_TAGCTTATCAGACTGATGTT2	-0,030096058	7,181930249	0,879294219	0,951466111	LADA vs T2D Time-adjusted
hsa-miR-486-5p_TGTACTGAGCTGCCCCGA2	0,075538867	4,53697007	0,887657961	0,957766345	LADA vs T2D Time-adjusted
hsa-miR-100-5p_AACCCGTAGATCCGAACTTG2	-0,043013105	5,980148473	0,888213793	0,957766345	LADA vs T2D Time-adjusted
hsa-miR-142-5p_CCCATAAAGTAGAAAGCAC2	0,049945098	5,073422111	0,889436312	0,957766345	LADA vs T2D Time-adjusted
hsa-miR-382-5p_AAGTTGTTCGTGGTGGATTCG2	0,083298957	2,276351927	0,891969885	0,957766345	LADA vs T2D Time-adjusted
hsa-miR-7-5p_TGGAAGACTAGTGATTTTGTTGTT2	-0,07418695	4,490449806	0,893303105	0,957766345	LADA vs T2D Time-adjusted
hsa-miR-483-5p_AAGACGGGAGGAAAGAAGGGAG2	-0,049110106	6,695020301	0,894154734	0,957766345	LADA vs T2D Time-adjusted
hsa-miR-182-5p_TTTGGCAATGGTAGAACTCACACTGG2	-0,076399032	2,345773362	0,8948765	0,957766345	LADA vs T2D Time-adjusted
hsa-miR-4433b-5p_ATGTCCCACCCCCACTCCTGTTT2	-0,066800123	5,3584586	0,896007512	0,957766345	LADA vs T2D Time-adjusted
hsa-miR-15b-5p_TAGCAGCACATCATGGTTTAC2	-0,027004664	7,359413397	0,896014027	0,957766345	LADA vs T2D Time-adjusted
hsa-miR-99a-5p_AACCCGTAGATCCGATCTTG2	-0,02549583	8,068040532	0,89834755	0,958964793	LADA vs T2D Time-adjusted
hsa-miR-23a-3p_ATCACATTGCCAGGGATTTCCAA2	0,038390422	6,524370613	0,90489464	0,96350226	LADA vs T2D Time-adjusted
hsa-miR-193a-5p_TGGGTCTTTGCGGGCGAGATG2	0,044963012	5,874910096	0,90503436	0,96350226	LADA vs T2D Time-adjusted
hsa-miR-181a-5p_AACATTCAACGCTGTCGGTGAG2	-0,020361573	7,417004171	0,910535355	0,968055733	LADA vs T2D Time-adjusted
hsa-miR-3615_TCTCTCGGCTCCTCGCGGCT2	-0,032324632	6,628968416	0,912670508	0,968296522	LADA vs T2D Time-adjusted
hsa-miR-30d-5p_GTAAACATCCCCGACTGGAAG2	-0,031870059	5,943376472	0,914382578	0,968296522	LADA vs T2D Time-adjusted
hsa-miR-130b-3p_CAGTGCAATGATGAAAGGGCA2	0,042482864	4,356329159	0,915571675	0,968296522	LADA vs T2D Time-adjusted
hsa-miR-92a-3p_TATTGCACTTGTCCCGGCCTGT2	0,011619858	17,21206299	0,916201249	0,968296522	LADA vs T2D Time-adjusted
hsa-miR-30a-5p_TGTAAACATCCTCGACTGGAAG2	0,018865734	8,371847242	0,917273634	0,968296522	LADA vs T2D Time-adjusted
hsa-miR-338-5p_AACAATATCCTGGTGCTGAGT2	0,035613512	5,921373435	0,918106689	0,968296522	LADA vs T2D Time-adjusted
hsa-miR-183-5p_TATGGCACTGGTAGAATT2	-0,058121435	2,845053887	0,919646473	0,968628975	LADA vs T2D Time-adjusted
hsa-miR-486-5p_ATCCTGTACTGAGCTGCCCCG2	0,028962238	6,267380926	0,924776912	0,972517794	LADA vs T2D Time-adjusted
hsa-miR-629-5p_TGGGTTTACGTTGGGAGAACT2	-0,021038185	7,102407997	0,925797597	0,972517794	LADA vs T2D Time-adjusted
hsa-let-7f-5p_TGAGGTAGTAGATTGTATAGTT2	0,013583411	12,47888054	0,928655869	0,974226516	LADA vs T2D Time-adjusted
hsa-let-7c-5p_TGAGGTAGTAGGTTGTATGGTT2	0,014618601	7,624637986	0,935390941	0,975363817	LADA vs T2D Time-adjusted
hsa-miR-30a-3p_CTTTCAGTCGGATGTTTGCAG2	-0,044041001	2,025868208	0,935420985	0,975363817	LADA vs T2D Time-adjusted
hsa-miR-542-3p_TGTGACAGATTGATAACTGA2	-0,044447813	2,0314731	0,93675144	0,975363817	LADA vs T2D Time-adjusted
hsa-miR-3605-3p_CCTCCGTGTTACCTGTCCTCT2	-0,03609134	5,156629775	0,937035512	0,975363817	LADA vs T2D Time-adjusted
hsa-miR-486-5p_CTGTACTGAGCTGCCCCGA2	-0,016690441	7,578479757	0,937114653	0,975363817	LADA vs T2D Time-adjusted
hsa-miR-98-5p_TGAGGTAGTAAGTTGTATTG2	-0,033207872	4,697784917	0,937138434	0,975363817	LADA vs T2D Time-adjusted

hsa-miR-423-3p_AAGCTCGGTCTGAGGCCCCTCAGT2	0,01472018	7,381534421	0,943377185	0,975860231	LADA vs T2D Time-adjusted
hsa-miR-495-3p_AAACAAACATGGTGCACTTCTT2	-0,042218943	2,371725028	0,943787091	0,975860231	LADA vs T2D Time-adjusted
hsa-miR-27b-3p_TTCACAGTGGCTAAGTTCTGCA2	-0,034406949	3,816952744	0,944864474	0,975860231	LADA vs T2D Time-adjusted
hsa-miR-4433b-5p_TGTCCCACCCCCACTCCTGTT2	-0,031964396	6,506356169	0,945726491	0,975860231	LADA vs T2D Time-adjusted
hsa-miR-500a-3p_AATGCACCTGGGCAAGGATTCT2	0,039972107	2,908982628	0,945826755	0,975860231	LADA vs T2D Time-adjusted
hsa-miR-19b-3p_TGTGCAAATCCATGCAAAACTGA2	-0,027515124	5,03945579	0,949297732	0,975860231	LADA vs T2D Time-adjusted
hsa-miR-21-5p_TAGCTTATCAGACTGATGTTGAC2	0,014449151	10,35786285	0,950124946	0,975860231	LADA vs T2D Time-adjusted
hsa-miR-375-3p_TTTGTTCGTTCGGCTCGCGT2	-0,034475682	4,026890223	0,950994514	0,975860231	LADA vs T2D Time-adjusted
hsa-miR-342-3p_TCTCACACAGAAATCGCACCC2	0,033877411	2,016844553	0,951606755	0,975860231	LADA vs T2D Time-adjusted
hsa-let-7d-3p_TATACGACCTGCTGCCTTT2	0,016258814	6,258064372	0,953081728	0,975860231	LADA vs T2D Time-adjusted
hsa-miR-92a-3p_TGCACTTGTCCCGGCCTGT2	0,022078115	5,30065349	0,953248079	0,975860231	LADA vs T2D Time-adjusted
hsa-miR-505-3p_CGTCAACACTTGCTGGTTTCCTCT2	-0,033591763	3,132189778	0,953437924	0,975860231	LADA vs T2D Time-adjusted
hsa-miR-4433b-5p_ATGTCCCACCCCCACTCCTG2	0,029909609	1,885888634	0,957021889	0,975860231	LADA vs T2D Time-adjusted
hsa-miR-423-5p_TGAGGGGCAGAGAGCGAGA2	0,006288956	9,771757288	0,957055973	0,975860231	LADA vs T2D Time-adjusted
hsa-miR-425-5p_AATGACACGATCACTCCCGTTGAGT2	0,012492624	8,285934125	0,957779541	0,975860231	LADA vs T2D Time-adjusted
hsa-miR-199a-3p_ACAGTAGTCTGCACATTGGTT2	-0,044765332	4,587596	0,9577924	0,975860231	LADA vs T2D Time-adjusted
hsa-miR-103a-3p_AGCAGCATTGTACAGGGCTATG2	-0,028130659	4,200669238	0,958588368	0,975860231	LADA vs T2D Time-adjusted
hsa-miR-15b-5p_TAGCAGCACATCATGGTTTACA2	0,013289725	6,304858351	0,964006598	0,980114677	LADA vs T2D Time-adjusted
hsa-miR-423-5p_TGAGGGGCAGAGAGCGAGACT2	-0,005232002	13,36591214	0,966731573	0,980934219	LADA vs T2D Time-adjusted
hsa-miR-142-5p_CATAAAGTAGAAAGCACTACT2	0,020389717	3,738908441	0,967292909	0,980934219	LADA vs T2D Time-adjusted
hsa-let-7b-5p_TGAGGTAGTAGGTTGTGTGGTTT2	-0,003798088	11,27639147	0,974280691	0,986755476	LADA vs T2D Time-adjusted
hsa-miR-155-5p_TTAATGCTAATCGTGATAGGGGT2	-0,014216645	4,228033966	0,975849449	0,986888031	LADA vs T2D Time-adjusted
hsa-miR-133a-3p_TTGGTCCCCTTCAACCAGCTGT2	0,017844569	2,487795126	0,976906863	0,986888031	LADA vs T2D Time-adjusted
hsa-miR-483-5p_AAGACGGGAGGAAAGAAGGGAGT2	-0,01498251	4,22603991	0,978994848	0,987735873	LADA vs T2D Time-adjusted
hsa-miR-423-5p_GAGGGGCAGAGAGCGAGACTTT2	0,002733098	8,417582901	0,983395187	0,990911584	LADA vs T2D Time-adjusted
hsa-miR-93-5p_CAAAGTGCTGTTCGTGCAGGTAGT2	-0,009571043	4,055351232	0,985676876	0,991418985	LADA vs T2D Time-adjusted
hsa-miR-98-5p_TGAGGTAGTAAGTTGTATTGTT2	0,004348511	7,833511387	0,986724682	0,991418985	LADA vs T2D Time-adjusted
hsa-miR-146a-5p_GAGAACTGAATTCCATGGGTT2	-0,005412928	6,482797306	0,987658863	0,991418985	LADA vs T2D Time-adjusted
hsa-miR-10a-5p_ACCCTGTAGATCCGAATTTGTG2	-0,002922614	6,333214322	0,990471633	0,992982334	LADA vs T2D Time-adjusted
hsa-miR-501-3p_AATGCACCCGGGCAAGGATTCT2	-0,000783952	7,8797274	0,99618258	0,996806151	LADA vs T2D Time-adjusted
hsa-miR-144-5p_GGATATCATCATATACTGTAAGT2	-0,001078328	6,455704528	0,996806151	0,996806151	LADA vs T2D Time-adjusted

ESM Table 3

ESM Table 3: Results from differential expression analysis of ncRNAs from RNACentral. The comparions are indicated in the column "Comparison" such that an ncRNA with positive "logFC" value in T1D vs T2D means that the ncRNA is up-regulated in T1D vs T2D. "ncRNA" indicate the ncRNA from RNACentral; "logFC" is the log2 fold change from limma; "Average Expression" is the average expression of the ncRNA across all samples; "P-Value" is the P-value for the specific ncRNA for the specific comparions before correction for multiple testing; "Adjusted P-Value" is the benjamini-hochberg adjusted P-value.

ncRNA	logFC	Average Expression	P-Value	Adjusted P-Value	Comparison
URS0000278E1B-tRNA	-2,392314321	2,766067348	3,12093E-05	0,014619123	T1D vs T2D
URS000011BDAF-antisense	-2,161504407	2,89470373	4,18287E-05	0,014619123	T1D vs T2D
URS00000A00A2-antisense	-2,533033463	4,833316746	7,04613E-05	0,015465226	T1D vs T2D
URS00000FB60D-tRNA	-2,296902953	3,59514453	8,84991E-05	0,015465226	T1D vs T2D
URS00006E3DE1-snRNA	-2,517496786	3,143314554	0,000136002	0,018839051	T1D vs T2D
URS0000145C5E-tRNA	-2,412481563	2,892697082	0,000161709	0,018839051	T1D vs T2D
URS000024B38F-tRNA	-2,091487054	4,404533737	0,00020843	0,020813231	T1D vs T2D
URS00006529EE-Y_RNA	-2,140330149	3,147668241	0,000348534	0,028865627	T1D vs T2D
URS0000249329-lncRNA	-1,845635981	1,740243504	0,00037166	0,028865627	T1D vs T2D
URS000065DC2A-tRNA	-1,483043697	4,471494404	0,000476498	0,033307239	T1D vs T2D
URS0000415026-tRNA	-1,530031492	4,426745558	0,000908221	0,057713334	T1D vs T2D
URS00000D1D87-lncRNA	-1,732003859	1,722539329	0,001070994	0,062385403	T1D vs T2D
URS00006729E8-Y_RNA	-1,002880496	0,88430075	0,001214479	0,06530161	T1D vs T2D
URS00007125F9-rRNA	-1,896835323	2,409823294	0,001379683	0,068885589	T1D vs T2D
URS00004A68E9-antisense	-1,335024585	3,654195066	0,001719045	0,08010752	T1D vs T2D
URS000069ED7F-Y_RNA	-1,831897518	4,579144673	0,002239738	0,097848565	T1D vs T2D
URS0000644222-tRNA	-0,670441528	7,91366141	0,002952924	0,10469833	T1D vs T2D
URS00006C133C-tRNA	-1,690337695	3,680848373	0,003029508	0,10469833	T1D vs T2D
URS000061F57C-tRNA	-1,473933294	4,675906559	0,003117359	0,10469833	T1D vs T2D
URS00009290F1-rRNA	-1,631323513	1,980245991	0,003193575	0,10469833	T1D vs T2D
URS000069E2A5-tRNA	-1,86618072	3,421985706	0,003249865	0,10469833	T1D vs T2D
URS00002E367B-antisense	-1,654650759	1,915955023	0,003444733	0,10469833	T1D vs T2D
URS0000537899-antisense	-1,690863877	2,143848964	0,003559015	0,10469833	T1D vs T2D
URS00006D1735-snRNA	-1,389123799	5,596569208	0,003743742	0,10469833	T1D vs T2D
URS000071ED2F-tRNA	-1,873271644	2,991616318	0,003744575	0,10469833	T1D vs T2D
URS000096196A-SRP_RNA	-1,587297468	2,043342413	0,004338764	0,116646004	T1D vs T2D
URS000039557B-antisense	-1,505618581	1,694045712	0,005302128	0,137266202	T1D vs T2D
URS0000679FAF-tRNA	-1,289074677	5,101125315	0,006086648	0,15194881	T1D vs T2D
URS0000365006-lncRNA	-1,493060263	2,397164181	0,006511891	0,152467672	T1D vs T2D
URS000099D184-rRNA	-1,103214762	4,977808511	0,006543677	0,152467672	T1D vs T2D
URS00000540AC-antisense	-1,493493886	3,451834854	0,006940944	0,156507096	T1D vs T2D
URS00009C6070-lncRNA	-1,423176186	2,53934864	0,007382169	0,16125426	T1D vs T2D
URS0000624312-Y_RNA	-1,073971627	5,698207316	0,008170219	0,165568311	T1D vs T2D
URS00006C9A71-Y_RNA	-1,363230181	2,255341052	0,008507456	0,165568311	T1D vs T2D
URS000064506B-tRNA	-1,692587957	2,909810805	0,008538576	0,165568311	T1D vs T2D
URS000047AE74-lncRNA	-1,521606122	2,522838605	0,008671448	0,165568311	T1D vs T2D
URS000029CCC5-tRNA	-1,445444373	2,732517708	0,008763988	0,165568311	T1D vs T2D
URS0000A7AB58-Y_RNA	-1,400814047	4,159115672	0,009397636	0,172867042	T1D vs T2D
URS000099C20E-rRNA	-1,655338566	3,326722503	0,010813639	0,193813676	T1D vs T2D
URS000063FB43-tRNA	-0,491880928	12,66119095	0,011140401	0,1946785	T1D vs T2D
URS000063A7A5-Y_RNA	-0,758300843	8,2571435	0,012090819	0,195253127	T1D vs T2D
URS00001DBD56-lncRNA	-1,27879292	3,720321434	0,012297317	0,195253127	T1D vs T2D
URS00006C900C-tRNA	-1,372689164	2,991743424	0,012314653	0,195253127	T1D vs T2D
URS000006044C-lncRNA	-1,349261188	1,808588154	0,012433654	0,195253127	T1D vs T2D
URS000047C79B-tRNA	-0,415310568	8,413478971	0,012569944	0,195253127	T1D vs T2D
URS0000716B70-tRNA	-1,276937264	3,560279134	0,014030464	0,209263087	T1D vs T2D
URS0000222FD2-tRNA	-1,515880055	3,623751413	0,014353977	0,209263087	T1D vs T2D
URS00006F4E76-Y_RNA	-0,656021567	0,766333934	0,014369997	0,209263087	T1D vs T2D
URS000064E10F-tRNA	-1,442278639	3,013775094	0,014824312	0,211112413	T1D vs T2D
URS00004AE46A-lncRNA	-1,369058326	3,574152246	0,01514611	0,211112413	T1D vs T2D
URS00004AE57B-lncRNA	-1,411293943	2,181566953	0,015403052	0,211112413	T1D vs T2D
URS000042F13F-tRNA	-0,669083831	6,573577673	0,016487292	0,22162725	T1D vs T2D

URS00003B6188-lncRNA	-1,258318797	3,075648025	0,016878548	0,222605755	T1D vs T2D
URS0000495A30-lncRNA	-1,266296933	2,253557055	0,017301062	0,223952634	T1D vs T2D
URS00004BF687-tRNA	-1,476485489	13,4414794	0,017806637	0,226306172	T1D vs T2D
URS00008C3E41-lncRNA	0,978434279	5,384577661	0,020993789	0,262047472	T1D vs T2D
URS0000684921-rRNA	-1,350718354	2,725738393	0,021719293	0,263363194	T1D vs T2D
URS000038803E-tRNA	-0,733948313	5,940754642	0,02185274	0,263363194	T1D vs T2D
URS0000ABD8C6-rRNA	-2,801122242	7,712316992	0,022550551	0,265576195	T1D vs T2D
URS00002750C5-lncRNA	-1,224933251	1,848936086	0,023144946	0,265576195	T1D vs T2D
URS00006F4F8C-snoRNA	-0,499115189	8,039702247	0,023424183	0,265576195	T1D vs T2D
URS0000166229-lncRNA	-1,184366035	2,097439644	0,023562681	0,265576195	T1D vs T2D
URS0000446770-snoRNA	-1,385077703	2,104005039	0,023936052	0,265576195	T1D vs T2D
URS0000699390-Y_RNA	-1,070647302	13,62373063	0,025634376	0,279975452	T1D vs T2D
URS00006BBD5F-Y_RNA	-1,283156762	3,385968133	0,026177229	0,281505897	T1D vs T2D
URS00000E43DB-antisense	-1,19407108	3,522079692	0,026787693	0,283518834	T1D vs T2D
URS000056B96A-antisense	-1,124705163	2,621722386	0,02740501	0,283518834	T1D vs T2D
URS000062C4DE-tRNA	-1,273582853	3,676191563	0,027946052	0,283518834	T1D vs T2D
URS00000DE490-lncRNA	-1,109112114	3,392102095	0,028225536	0,283518834	T1D vs T2D
URS00001FBD75-misc_RNA]	1,897832904	3,307722647	0,028392444	0,283518834	T1D vs T2D
URS00000DA56A-lncRNA	-1,271282713	2,589490843	0,029477433	0,290207403	T1D vs T2D
URS0000233E9C-lncRNA	1,674956532	2,822247929	0,030412747	0,292950739	T1D vs T2D
URS00006D56C0-Y_RNA	-0,863686432	4,210228585	0,030922921	0,292950739	T1D vs T2D
URS0000422302-lncRNA	-1,134864856	2,914915679	0,031031068	0,292950739	T1D vs T2D
URS00006C06E6-Y_RNA	-1,238530489	1,912207487	0,031601168	0,292950739	T1D vs T2D
URS000034E03C-antisense	-1,248504359	2,078398724	0,031851583	0,292950739	T1D vs T2D
URS000003870B-lncRNA	-0,451564258	9,957227048	0,032473348	0,293012124	T1D vs T2D
URS0000A7F61B-lncRNA	-1,153438389	2,242573569	0,033059954	0,293012124	T1D vs T2D
URS00006FCBA3-Y_RNA	-1,197041583	2,601539284	0,033291954	0,293012124	T1D vs T2D
URS0000120E41-tRNA	-0,679953557	6,378757577	0,033535007	0,293012124	T1D vs T2D
URS00002EDD28-lncRNA	-0,800093845	5,436899358	0,035037027	0,302232782	T1D vs T2D
URS00006F0FC0-tRNA	-0,822882318	5,435006769	0,035889518	0,302232782	T1D vs T2D
URS000057C597-antisense	-1,230221075	2,337861425	0,036314831	0,302232782	T1D vs T2D
URS00003D2CC9-tRNA	-1,112324976	4,271817892	0,036433611	0,302232782	T1D vs T2D
URS0000478C87-lncRNA	-1,13581222	2,830369346	0,036752198	0,302232782	T1D vs T2D
URS0000672E5A-rRNA	-0,762698334	5,608838646	0,037247372	0,302743178	T1D vs T2D
URS000029A6A6-antisense	-0,655460615	5,872434994	0,037898833	0,304497521	T1D vs T2D
URS000063CFC9-Y_RNA	-1,239829263	2,480380401	0,038413132	0,305122489	T1D vs T2D
URS0000209048-tRNA	-1,247053546	3,508601642	0,039655026	0,308169462	T1D vs T2D
URS00003475A2-lncRNA	-0,679954484	6,073704566	0,039678472	0,308169462	T1D vs T2D
URS00001EC8D7-snRNA	-0,850111348	4,577467935	0,04037843	0,308886989	T1D vs T2D
URS000070E3CE-rRNA	-1,078680667	4,081177344	0,040654654	0,308886989	T1D vs T2D
URS00004E7DF9-lncRNA	-0,999798573	4,28189465	0,041539284	0,312214615	T1D vs T2D
URS00008BB15B-lncRNA	-1,359609091	2,482102831	0,04351588	0,323591492	T1D vs T2D
URS0000A774C0-lncRNA	-0,595130764	6,402978234	0,045727383	0,334700209	T1D vs T2D
URS00002598CF-lncRNA	-1,00128246	4,630900569	0,046427108	0,334700209	T1D vs T2D
URS000049B61C-antisense	-0,446714187	6,879627508	0,046446238	0,334700209	T1D vs T2D
URS0000639DBE-tRNA	-0,655748386	5,433019134	0,048749901	0,347716131	T1D vs T2D
URS00006BFB96-tRNA	-1,139168849	2,209793928	0,050915901	0,359497122	T1D vs T2D
URS00002A865C-lncRNA	-1,045300233	1,751381992	0,051600275	0,359844691	T1D vs T2D
URS0000192C05-lncRNA	-1,063795481	2,748839188	0,051994726	0,359844691	T1D vs T2D
URS0000006840-lncRNA	-0,992409543	3,802540443	0,052575426	0,3602963	T1D vs T2D
URS00006E1108-Y_RNA	-0,6561238	4,049124964	0,053224225	0,360509722	T1D vs T2D
URS000059900F-tRNA	-1,097373395	3,409718688	0,05363807	0,360509722	T1D vs T2D
URS0000907730-rRNA	-0,66423258	6,755248399	0,056727414	0,369335119	T1D vs T2D
URS0000996BBC-rRNA	-0,353661598	8,622403976	0,056766781	0,369335119	T1D vs T2D
URS0000617C6A-antisense	-0,907255268	3,572347898	0,056897574	0,369335119	T1D vs T2D
URS0000389FBF-lncRNA	-1,060320519	2,547635864	0,057064654	0,369335119	T1D vs T2D
URS000051EF4B-lncRNA	-1,033335976	1,97882489	0,060987027	0,391056692	T1D vs T2D
URS00006D74B2-tRNA	-0,87316551	4,411009625	0,061753884	0,391056692	T1D vs T2D
URS0000397210-lncRNA	-1,012142017	2,372225381	0,062099131	0,391056692	T1D vs T2D
URS000093FF31-rRNA	-0,996544803	2,038384139	0,063186323	0,394350354	T1D vs T2D
URS00005CEC24-lncRNA	-1,11773027	2,918669315	0,064361992	0,395983292	T1D vs T2D
URS000076B0D1-lncRNA	-1,041289959	3,094918128	0,065886982	0,395983292	T1D vs T2D
URS0000ABD87F-rRNA	-1,824959411	10,81861494	0,066475454	0,395983292	T1D vs T2D
URS00001E15E2-lncRNA	-0,945426453	2,285605947	0,066494461	0,395983292	T1D vs T2D
URS0000181B59-lncRNA	-1,253795394	2,274734234	0,067972554	0,395983292	T1D vs T2D
URS000007383C-antisense	-0,986092995	2,496432723	0,068041237	0,395983292	T1D vs T2D

URS0000188F7D-scRNA	-2,761466365	6,577829264	0,068418662	0,395983292	T1D vs T2D
URS00001424D6-lncRNA	-1,05126897	1,834670251	0,068437732	0,395983292	T1D vs T2D
URS0000527686-lncRNA	-0,71875162	1,162280834	0,068546464	0,395983292	T1D vs T2D
URS0000391360-lncRNA	-1,093354211	2,256356542	0,070437873	0,403574373	T1D vs T2D
URS00001A48CC-lncRNA	-0,981798573	2,650296854	0,073423744	0,412562233	T1D vs T2D
URS00005DB87D-tRNA	-0,359286338	7,975358739	0,074247818	0,412562233	T1D vs T2D
URS0000ABD7E9-lncRNA	-1,070417517	3,512762788	0,07425195	0,412562233	T1D vs T2D
URS000038F4B2-tRNA	-1,10179872	2,156583414	0,074726884	0,412562233	T1D vs T2D
URS0000371842-lncRNA	-1,022105279	2,020987369	0,075069768	0,412562233	T1D vs T2D
URS00004D9E92-tRNA	-0,483968711	7,483097251	0,076052268	0,412562233	T1D vs T2D
URS000034AAC2-tRNA	-0,961946009	4,895139042	0,07677536	0,412562233	T1D vs T2D
URS0000690F87-snRNA	-1,244612177	3,114315559	0,076953643	0,412562233	T1D vs T2D
URS0000677B31-tRNA	0,73353949	7,303130994	0,07731853	0,412562233	T1D vs T2D
URS00006C8EDF-tRNA	-1,079144438	3,590220694	0,078138231	0,413777452	T1D vs T2D
URS000031A1AE-antisense	-1,095125293	2,086098634	0,079292371	0,413848403	T1D vs T2D
URS00002A28BF-lncRNA	-1,148394028	2,823053274	0,079335745	0,413848403	T1D vs T2D
URS00006C2C4A-Y_RNA	-1,294797391	4,927831545	0,08212288	0,421542334	T1D vs T2D
URS00000347CF-lncRNA	-0,848718367	3,718528756	0,082727305	0,421542334	T1D vs T2D
URS0000633F75-ribozyme	-0,92093542	4,438697312	0,083093264	0,421542334	T1D vs T2D
URS00006AE6F1-Y_RNA	-0,769130187	2,11506442	0,08322295	0,421542334	T1D vs T2D
URS000075A564-lncRNA	-0,986718483	2,096352205	0,084180893	0,423326935	T1D vs T2D
URS00003C9A26-tRNA	-0,323007519	8,822917147	0,085646685	0,425267065	T1D vs T2D
URS000013B42D-tRNA	-0,590301947	16,60649921	0,085783485	0,425267065	T1D vs T2D
URS000036E063-antisense	0,202941696	10,32347827	0,090614762	0,443105198	T1D vs T2D
URS00003ADE1B-lncRNA	-0,987273836	2,400792438	0,091912634	0,443105198	T1D vs T2D
URS0000784C7B-lncRNA	0,959990688	2,086288772	0,092230294	0,443105198	T1D vs T2D
URS00006B0E5A-precursor_RNA	-0,682680615	6,734564531	0,092891509	0,443105198	T1D vs T2D
URS000050E9EC-lncRNA	-0,86578763	3,382080638	0,094869928	0,443105198	T1D vs T2D
URS00006D23E9-snRNA	-0,94780445	3,580811892	0,095041491	0,443105198	T1D vs T2D
URS00002D1F9F-lncRNA	-0,851029088	3,254441607	0,095324289	0,443105198	T1D vs T2D
URS0000AA014E-lncRNA	-0,893437043	2,880951371	0,09576867	0,443105198	T1D vs T2D
URS0000197DBF-antisense	-0,886935377	3,806884628	0,095895298	0,443105198	T1D vs T2D
URS000022006F-lncRNA	-0,916065482	3,198783691	0,096178574	0,443105198	T1D vs T2D
URS0000A85AEE-lncRNA	-1,075709836	3,200285639	0,096354778	0,443105198	T1D vs T2D
URS0000997FE9-rRNA	-0,882663698	2,876398415	0,100433037	0,450613854	T1D vs T2D
URS000006D0D7-lncRNA	-0,922702705	2,152173172	0,101627014	0,450613854	T1D vs T2D
URS00003AA49D-lncRNA	0,916185191	2,72551685	0,102007621	0,450613854	T1D vs T2D
URS0000265843-antisense	-0,861520613	2,862204159	0,102153875	0,450613854	T1D vs T2D
URS00000AD0C2-lncRNA	-0,906013437	1,982015887	0,102696571	0,450613854	T1D vs T2D
URS0000637E4A-tRNA	-0,890966626	1,552702938	0,103456647	0,450613854	T1D vs T2D
URS00000081EA-snRNA	-0,943222042	2,66468092	0,103630363	0,450613854	T1D vs T2D
URS0000687FC3-Y_RNA	-0,4613445	4,184219214	0,104569828	0,450613854	T1D vs T2D
URS00002D40C8-tRNA	-0,398009491	7,830242615	0,104622438	0,450613854	T1D vs T2D
URS00002D0015-lncRNA	-0,835336675	3,226559685	0,106576034	0,450613854	T1D vs T2D
URS00000F6ECB-lncRNA	-0,614877763	5,128846246	0,107102541	0,450613854	T1D vs T2D
URS00002E81F5-lncRNA	-0,803279524	4,084000104	0,107424665	0,450613854	T1D vs T2D
URS00002901EC-tRNA	-0,946800784	2,043121881	0,107542132	0,450613854	T1D vs T2D
URS000053EAB5-lncRNA	0,849490614	3,036408354	0,10756023	0,450613854	T1D vs T2D
URS00006C14B2-tRNA	-1,077827499	3,304706256	0,107657387	0,450613854	T1D vs T2D
URS00008116E3-lncRNA	-0,811159813	4,000279228	0,109895135	0,453191204	T1D vs T2D
URS00002034DC-tRNA	-0,904967073	5,180504267	0,110423866	0,453191204	T1D vs T2D
URS0000610FFE-tRNA	-0,902556834	2,353369293	0,111025011	0,453191204	T1D vs T2D
URS000075D48E-lncRNA	-0,789100701	1,602010302	0,111798686	0,453191204	T1D vs T2D
URS000064C567-Y_RNA	-0,377912025	5,523874961	0,112097558	0,453191204	T1D vs T2D
URS00001E6C0A-lncRNA	-0,84967394	3,103912065	0,112865215	0,453191204	T1D vs T2D
URS00007C72D0-lncRNA	-0,778771062	1,699191247	0,113546226	0,453191204	T1D vs T2D
URS0000920597-rRNA	-0,733203283	8,520273949	0,113857031	0,453191204	T1D vs T2D
URS0000590507-lncRNA	-0,856274093	1,90331514	0,114108229	0,453191204	T1D vs T2D
URS00000A7F2D-lncRNA	-0,837625494	2,135027243	0,115251166	0,455144437	T1D vs T2D
URS0000196BD3-lncRNA	-0,866038646	2,056335374	0,116442826	0,456152362	T1D vs T2D
URS000048EB2F-antisense	-0,89362198	2,157167592	0,116811549	0,456152362	T1D vs T2D
URS00006D1A54-Y_RNA	-0,810086439	3,828527713	0,118253717	0,456497351	T1D vs T2D
URS000075AD80-snRNA	-0,943773369	2,124304339	0,119081232	0,456497351	T1D vs T2D
URS00007D6D04-lncRNA	-0,78349639	3,22797681	0,119088201	0,456497351	T1D vs T2D
URS000076E14E-lncRNA	1,064570916	2,947769863	0,119512182	0,456497351	T1D vs T2D
URS000009DDCA-tRNA	-0,414056545	8,707527281	0,120291203	0,456975822	T1D vs T2D

URS00002C9C48-lncRNA	0,863285537	5,358879491	0,122480975	0,460439662	T1D vs T2D
URS0000539731-antisense	-0,798087427	2,084997113	0,122941368	0,460439662	T1D vs T2D
URS00006BF71F-rRNA	-0,888788247	2,492314255	0,123179137	0,460439662	T1D vs T2D
URS0000AAECF4-lncRNA	-0,718145487	3,702161679	0,123957661	0,460885131	T1D vs T2D
URS00006D1C46-snRNA	-0,728362291	6,026449126	0,125129182	0,462779356	T1D vs T2D
URS0000005EDF-lncRNA	0,793823799	4,116716413	0,128095337	0,470974222	T1D vs T2D
URS0000067204-antisense	-0,851582791	1,800108533	0,128692527	0,470974222	T1D vs T2D
URS00004D4BFC-lncRNA	-0,756884836	2,209794014	0,129510971	0,471500879	T1D vs T2D
URS00001B2779-lncRNA	-0,870104112	1,848580988	0,131505819	0,47449314	T1D vs T2D
URS0000717DFB-Y_RNA	-0,786085063	2,95584216	0,131690514	0,47449314	T1D vs T2D
URS000064B6FC-tRNA	-0,951146579	3,113360192	0,134260822	0,480259033	T1D vs T2D
URS00006BB04D-tRNA	0,844777884	3,054494403	0,134664908	0,480259033	T1D vs T2D
URS000011ABD1-lncRNA	0,948974275	2,282001703	0,137525892	0,487176891	T1D vs T2D
URS000068F7ED-Y_RNA	-0,748525437	6,623874775	0,137998604	0,487176891	T1D vs T2D
URS00000E0DE1-antisense	-0,804208877	3,104520142	0,139129376	0,488038524	T1D vs T2D
URS00004F2E54-lncRNA	-0,849418798	1,792153167	0,139639063	0,488038524	T1D vs T2D
URS0000100BA4-antisense	-0,848045784	1,836907151	0,144918648	0,503970822	T1D vs T2D
URS0000341866-lncRNA	-0,867370874	4,848112839	0,14656188	0,507162148	T1D vs T2D
URS0000121433-tRNA	-1,10061652	4,021698317	0,147711051	0,508024823	T1D vs T2D
URS00002200B0-lncRNA	-0,850488155	3,176288712	0,148264755	0,508024823	T1D vs T2D
URS00006772C0-tRNA	-0,887815967	2,278759881	0,149905964	0,511142773	T1D vs T2D
URS00005B6FC3-lncRNA	0,61786048	4,468244677	0,152352842	0,516964255	T1D vs T2D
URS000012730D-lncRNA	0,836342032	3,696451386	0,155042931	0,522784111	T1D vs T2D
URS00006D9244-tRNA	-0,897563721	3,430512932	0,155563799	0,522784111	T1D vs T2D
URS00004C6AFE-antisense	-0,598995917	5,444648432	0,156727989	0,524176383	T1D vs T2D
URS00006FC298-tRNA	-0,879978148	2,919601035	0,159308822	0,52933559	T1D vs T2D
URS000001AE93-lncRNA	0,326420099	6,898854125	0,160132889	0,52933559	T1D vs T2D
URS000076FC5A-lncRNA	-0,87573585	2,343917503	0,160806403	0,52933559	T1D vs T2D
URS00000D8A7A-lncRNA	-0,761905805	2,120449279	0,162270443	0,52933559	T1D vs T2D
URS00001662B7-lncRNA	-0,915134081	2,511216373	0,162734301	0,52933559	T1D vs T2D
URS00002B0998-lncRNA	-0,785106762	2,419217026	0,163650222	0,52933559	T1D vs T2D
URS0000189042-lncRNA	-0,838550021	1,901016294	0,164053133	0,52933559	T1D vs T2D
URS0000629ECF-Y_RNA	-0,513305981	4,37154222	0,164635535	0,52933559	T1D vs T2D
URS0000395C70-lncRNA	0,992703499	2,694736211	0,165335957	0,52933559	T1D vs T2D
URS00003F5471-lncRNA	-0,729966789	3,0793045	0,165843339	0,52933559	T1D vs T2D
URS0000682FB0-Y_RNA	-0,578509319	10,97882695	0,169168068	0,533758751	T1D vs T2D
URS00009C606C-lncRNA	0,489244921	5,246519845	0,170166722	0,533758751	T1D vs T2D
URS000041FE38-lncRNA	-0,750465623	2,353684167	0,170477923	0,533758751	T1D vs T2D
URS00002CDF5D-lncRNA	-0,705534446	2,151438672	0,17049684	0,533758751	T1D vs T2D
URS00000D6053-lncRNA	-1,145032943	5,001351593	0,171065321	0,533758751	T1D vs T2D
URS00000AD390-antisense	-0,754599502	1,744059197	0,171810757	0,533758751	T1D vs T2D
URS000096E1E3-rRNA	-0,808473168	2,495902245	0,172736279	0,534259552	T1D vs T2D
URS000062C73B-tRNA	-0,597934882	4,544224584	0,173962297	0,534395341	T1D vs T2D
URS00006C2A6C-Y_RNA	-0,677200388	10,4018413	0,17430921	0,534395341	T1D vs T2D
URS0000075AD1-lncRNA	-0,722408398	1,794828601	0,176162245	0,536439742	T1D vs T2D
URS00005B6D58-lncRNA	-0,658131884	5,324374408	0,176510931	0,536439742	T1D vs T2D
URS0000726FAB-rRNA	0,199766433	13,91320925	0,178348478	0,538073282	T1D vs T2D
URS00001E5F12-lncRNA	-0,785089469	2,516521413	0,181368588	0,538073282	T1D vs T2D
URS000058CCC6-antisense	-0,751251896	2,503982133	0,181829304	0,538073282	T1D vs T2D
URS000097B164-rRNA	-0,577985308	5,947136128	0,182434199	0,538073282	T1D vs T2D
URS000045FD15-lncRNA	-0,725371902	4,004446293	0,18244667	0,538073282	T1D vs T2D
URS0000591DFC-lncRNA	-0,700752538	1,976731906	0,182961461	0,538073282	T1D vs T2D
URS00000E0E97-antisense	-1,147092482	4,066934958	0,183869827	0,538073282	T1D vs T2D
URS0000928682-rRNA	-0,170751778	11,56053599	0,184890137	0,538073282	T1D vs T2D
URS000071E736-Y_RNA	-0,817383334	2,593633412	0,184898864	0,538073282	T1D vs T2D
URS00009C6074-antisense	-0,848999255	2,11618012	0,186002385	0,538073282	T1D vs T2D
URS00000DAC34-lncRNA	-0,740803485	2,636836099	0,186281139	0,538073282	T1D vs T2D
URS0000684E4B-tRNA	-0,326146784	6,262078663	0,186353144	0,538073282	T1D vs T2D
URS00007080E9-Y_RNA	-0,984806421	2,936384424	0,187709333	0,538073282	T1D vs T2D
URS000047AF1F-lncRNA	1,149562613	4,480968908	0,187825294	0,538073282	T1D vs T2D
URS0000759CF4-lncRNA	-0,740394388	1,845197414	0,189213255	0,538202028	T1D vs T2D
URS00006EB1B5-Y_RNA	-0,677354873	4,084821132	0,189410156	0,538202028	T1D vs T2D
URS0000016595-lncRNA	-0,781758662	2,839797195	0,190686584	0,539635314	T1D vs T2D
URS00006CE1FB-misc_RNA]	1,21275003	6,644778412	0,192245214	0,540719656	T1D vs T2D
URS000041043F-antisense	-0,735305813	1,994452875	0,193389568	0,540719656	T1D vs T2D
URS00004AAD0A-lncRNA	0,720380802	2,996600067	0,193390435	0,540719656	T1D vs T2D

URS0000942121-rRNA	-0,097263041	14,48998565	0,19482359	0,541354202	T1D vs T2D
URS0000990012-rRNA	-0,422081429	8,57303772	0,195572262	0,541354202	T1D vs T2D
URS0000A76F22-lncRNA	-0,718164114	2,591384678	0,196551188	0,541354202	T1D vs T2D
URS0000172E58-lncRNA	-0,693988115	3,538272714	0,196715261	0,541354202	T1D vs T2D
URS00001B59BD-lncRNA	-0,335878308	6,767957763	0,198889763	0,542692634	T1D vs T2D
URS000065D78F-Y_RNA	-0,223935639	5,875001903	0,199819531	0,542692634	T1D vs T2D
URS0000A765F3-lncRNA	-0,669544554	3,81544174	0,20024793	0,542692634	T1D vs T2D
URS00006174C2-tRNA	-0,289664922	8,218629708	0,200307152	0,542692634	T1D vs T2D
URS0000727FD6-tRNA	-0,769112659	3,320399515	0,20203262	0,543449208	T1D vs T2D
URS0000515429-lncRNA	0,474959741	7,164323775	0,202141336	0,543449208	T1D vs T2D
URS00000C18F2-tRNA	-0,556664865	6,19082938	0,205599917	0,545743417	T1D vs T2D
URS000017D264-lncRNA	-0,727682241	2,092743225	0,206218759	0,545743417	T1D vs T2D
URS0000511A5F-lncRNA	-0,7231309	1,837603255	0,20637561	0,545743417	T1D vs T2D
URS00006CDFA7-rRNA	-0,675006697	2,666787507	0,206768778	0,545743417	T1D vs T2D
URS00002172B5-lncRNA	-0,887939345	2,390444931	0,206898434	0,545743417	T1D vs T2D
URS00005BB09E-lncRNA	-0,497072795	6,047129544	0,208955335	0,548905658	T1D vs T2D
URS0000383A48-tRNA	0,728725539	3,229014707	0,210264449	0,548905658	T1D vs T2D
URS0000676525-Y_RNA	-0,638133595	4,329395557	0,210453099	0,548905658	T1D vs T2D
URS0000A88906-antisense	-0,724771052	1,925012855	0,21489234	0,553425042	T1D vs T2D
URS00002548DF-lncRNA	-0,654273729	1,501270697	0,216103526	0,553425042	T1D vs T2D
URS00007D436A-antisense	-0,679345377	2,239346559	0,216430493	0,553425042	T1D vs T2D
URS000053D4AB-snoRNA	-0,674016062	1,689585927	0,216813017	0,553425042	T1D vs T2D
URS00001EE979-lncRNA	-0,782878847	2,774714775	0,216987725	0,553425042	T1D vs T2D
URS0000635FFC-Y_RNA	-0,707851416	2,322009496	0,217981417	0,553425042	T1D vs T2D
URS000028E102-lncRNA	-0,692006178	2,546561157	0,218893233	0,553425042	T1D vs T2D
URS00006A9AE8-Y_RNA	-0,89730429	5,946221857	0,219267528	0,553425042	T1D vs T2D
URS000072C165-Y_RNA	-0,319173216	5,305209475	0,219311498	0,553425042	T1D vs T2D
URS000069B369-Y_RNA	0,845388452	9,690811268	0,223384293	0,561674896	T1D vs T2D
URS0000594305-lncRNA	-0,656296263	1,940137107	0,225321023	0,563465444	T1D vs T2D
URS00006CC125-rRNA	-0,687805439	2,366732734	0,225708619	0,563465444	T1D vs T2D
URS00006A3E7F-snRNA	-0,23130913	8,935478024	0,227100269	0,563705893	T1D vs T2D
URS00009612D1-rRNA	-0,520491165	5,646389432	0,227417828	0,563705893	T1D vs T2D
URS000029E713-antisense	0,596034078	4,061405427	0,23147907	0,571745124	T1D vs T2D
URS0000007D24-misc_RNA]	-1,753871973	6,091419652	0,233267768	0,574134401	T1D vs T2D
URS000022CCD7-lncRNA	-0,327230774	6,400235397	0,23453711	0,575233122	T1D vs T2D
URS00007062F7-Y_RNA	-0,460679808	4,160857713	0,23806135	0,580761025	T1D vs T2D
URS0000776086-lncRNA	-0,428784326	7,320712579	0,238452667	0,580761025	T1D vs T2D
URS0000ABD7E8-rRNA	1,082140852	10,98616314	0,241209944	0,585106754	T1D vs T2D
URS00004106BA-lncRNA	-0,568876506	1,798128499	0,242077274	0,585106754	T1D vs T2D
URS00003AD33F-lncRNA	0,728774777	3,336093709	0,242748153	0,585106754	T1D vs T2D
URS0000134A86-antisense	0,765051151	3,351781773	0,244661371	0,586411583	T1D vs T2D
URS0000676AED-precursor_RNA	-0,681577062	2,736959381	0,244967356	0,586411583	T1D vs T2D
URS000065A213-Y_RNA	-0,548538439	10,7844197	0,252433906	0,602222869	T1D vs T2D
URS0000605748-lncRNA	-0,65649727	3,689393404	0,258330268	0,614193391	T1D vs T2D
URS00000C653A-lncRNA	-0,620026946	2,462989968	0,26163601	0,619944309	T1D vs T2D
URS00009840C1-rRNA	0,447114778	6,418341272	0,264321723	0,624192176	T1D vs T2D
URS0000630B8A-tRNA	-0,525000742	5,357715317	0,266740587	0,625693066	T1D vs T2D
URS000075DF54-lncRNA	0,366073045	6,14755677	0,267426694	0,625693066	T1D vs T2D
URS0000974435-SRP_RNA	-0,575611914	2,886817066	0,268154735	0,625693066	T1D vs T2D
URS0000161EE9-lncRNA	-0,436648565	6,078069715	0,268537797	0,625693066	T1D vs T2D
URS00000FCDE9-misc_RNA]	-0,814420535	3,011608195	0,271959833	0,631561207	T1D vs T2D
URS0000782759-lncRNA	0,603466903	2,907193443	0,273224443	0,632396971	T1D vs T2D
URS000071DF37-tRNA	-0,712011092	2,691836943	0,276690829	0,636422139	T1D vs T2D
URS000040CE64-lncRNA	-0,720887601	2,883976268	0,276784449	0,636422139	T1D vs T2D
URS00006C48EB-Y_RNA]	-0,718226701	2,788995838	0,280605001	0,64309146	T1D vs T2D
URS0000A7BA37-lncRNA	-0,576541899	1,699099075	0,28400921	0,644915756	T1D vs T2D
URS000009AC8B-tRNA	-0,728331035	10,95540037	0,284134321	0,644915756	T1D vs T2D
URS0000320E71-lncRNA	-0,526982686	2,909981717	0,284392232	0,644915756	T1D vs T2D
URS0000ABD7D5-rRNA	0,100086322	13,17747017	0,285091514	0,644915756	T1D vs T2D
URS00004D1520-lncRNA	-0,610755227	3,203873127	0,286511316	0,646036807	T1D vs T2D
URS000063E4FD-tRNA	-0,653757197	3,061724682	0,288200319	0,647425534	T1D vs T2D
URS000063B690-snRNA	-0,616896166	3,324962274	0,28923565	0,647425534	T1D vs T2D
URS000024E9CC-lncRNA	0,635255828	2,021333988	0,289905854	0,647425534	T1D vs T2D
URS0000641C1B-Y_RNA	-0,330559818	4,028164364	0,292161695	0,649181273	T1D vs T2D
URS00006C6D0A-tRNA	0,614601971	3,831692982	0,293800855	0,649181273	T1D vs T2D
URS000070792C-rRNA	-0,586809215	1,756278837	0,294210209	0,649181273	T1D vs T2D

URS0000031963-antisense	-0,736752418	3,414756941	0,294869543	0,649181273	T1D vs T2D
URS00006642D4-Y_RNA	-0,201672732	4,428261145	0,295335686	0,649181273	T1D vs T2D
URS00005B2DE5-lncRNA	-0,554556897	4,758632634	0,29702243	0,649466077	T1D vs T2D
URS00001AD596-tRNA	-0,171422434	11,15468011	0,297323526	0,649466077	T1D vs T2D
URS000070B37B-tRNA	-0,192448135	9,295263071	0,299041178	0,649962363	T1D vs T2D
URS0000994031-rRNA	-0,155507417	11,10957664	0,299848509	0,649962363	T1D vs T2D
URS00001A4293-lncRNA	-0,51297756	4,616590583	0,301538592	0,649962363	T1D vs T2D
URS00002AD8DA-lncRNA	-0,544096918	2,376170134	0,302802936	0,649962363	T1D vs T2D
URS000064F96B-Y_RNA	-0,349067151	5,069805695	0,30285752	0,649962363	T1D vs T2D
URS0000A9CA30-lncRNA	-0,579678938	3,103219008	0,303842889	0,649962363	T1D vs T2D
URS00003DEE5B-lncRNA	0,563054925	3,256575112	0,304059646	0,649962363	T1D vs T2D
URS00009A5DA8-rRNA	-0,450759941	5,880019281	0,305169122	0,650345172	T1D vs T2D
URS00006D4008-tRNA	-0,708875244	2,742010752	0,307833395	0,653571025	T1D vs T2D
URS00005AF005-lncRNA	-0,561503639	3,522774737	0,308552844	0,653571025	T1D vs T2D
URS000017CF23-antisense	-0,556490263	2,666340903	0,311007009	0,654415903	T1D vs T2D
URS00000A3F50-lncRNA	-0,542709882	1,749216608	0,311017387	0,654415903	T1D vs T2D
URS0000561169-lncRNA	-0,57517899	3,951068111	0,311788404	0,654415903	T1D vs T2D
URS00004A7F9B-antisense	0,253720062	6,391504235	0,313020414	0,654415903	T1D vs T2D
URS0000397495-antisense	-0,344149714	5,990008067	0,314401409	0,654415903	T1D vs T2D
URS00001E9163-lncRNA	-0,567640046	2,764922404	0,314804691	0,654415903	T1D vs T2D
URS00006005A4-lncRNA	-0,697456883	2,906455771	0,315505235	0,654415903	T1D vs T2D
URS00006CB0C3-Y_RNA	-0,237804365	5,291889769	0,319881395	0,661529868	T1D vs T2D
URS0000700D52-tRNA	-0,58534395	2,144408558	0,322137112	0,664229619	T1D vs T2D
URS000076DAC1-lncRNA	-0,407671529	5,661423845	0,32533037	0,668840967	T1D vs T2D
URS00005B7465-tRNA	-0,55568387	1,847741502	0,327494444	0,671315591	T1D vs T2D
URS0000098211-lncRNA	-0,598440716	2,630527968	0,328867883	0,672159796	T1D vs T2D
URS0000AAB7F4-lncRNA	-0,54263778	3,962630959	0,330954005	0,674451456	T1D vs T2D
URS000034EAB6-lncRNA	-0,594514105	2,280705495	0,33295787	0,676562649	T1D vs T2D
URS00001B6230-lncRNA	-0,52706404	2,056088304	0,335889989	0,680542326	T1D vs T2D
URS00005580B2-lncRNA	-0,63650543	3,442360628	0,339382365	0,685630847	T1D vs T2D
URS00004F0321-tRNA	-0,290279889	14,08130095	0,340614989	0,685919089	T1D vs T2D
URS00006ABCCE-tRNA	0,707890742	11,64642261	0,342883942	0,685919089	T1D vs T2D
URS00001D081D-antisense	-0,529178398	1,824716399	0,343415256	0,685919089	T1D vs T2D
URS00003E3BDF-antisense	0,510675812	2,398707663	0,343450187	0,685919089	T1D vs T2D
URS00005C51E2-lncRNA	-0,500830138	1,604886726	0,347718808	0,69113797	T1D vs T2D
URS0000931B54-rRNA	-0,073123051	14,46696978	0,348894814	0,69113797	T1D vs T2D
URS00006C0413-Y_RNA	-0,56698184	2,155330761	0,349029618	0,69113797	T1D vs T2D
URS000021BDC3-snRNA	-0,501112119	2,084780028	0,350387976	0,691867784	T1D vs T2D
URS00005508F6-antisense	-0,522954993	3,129098015	0,352019614	0,693131578	T1D vs T2D
URS000038D8D3-tRNA	-0,287208821	7,544518208	0,354598522	0,696248222	T1D vs T2D
URS00006CBBB9-Y_RNA	-0,527987099	3,554703445	0,361184559	0,705697892	T1D vs T2D
URS0000811D9C-antisense	-0,336127781	0,958216717	0,362391714	0,705697892	T1D vs T2D
URS0000ABD879-rRNA	-1,088620151	7,136004534	0,363201117	0,705697892	T1D vs T2D
URS00003A72DC-antisense	0,496491224	3,084869652	0,363449558	0,705697892	T1D vs T2D
URS000098FA76-rRNA	-0,269644992	2,496348799	0,36687688	0,710379334	T1D vs T2D
URS00009A0DCD-rRNA	-0,394560534	5,942865971	0,368514785	0,710552049	T1D vs T2D
URS00009A050E-rRNA	-0,157168451	10,767947	0,369049213	0,710552049	T1D vs T2D
URS000005AEAB-tRNA	-0,416414758	4,48776437	0,370015659	0,710552049	T1D vs T2D
URS0000204428-lncRNA	-0,541565822	2,674128924	0,372665915	0,712292208	T1D vs T2D
URS00002AD6F3-antisense	-0,429569679	1,553235742	0,373991904	0,712292208	T1D vs T2D
URS000034E9D0-misc_RNA]	0,743372798	3,097582218	0,375444205	0,712292208	T1D vs T2D
URS0000649B00-rRNA	-0,128480073	10,09773081	0,378255925	0,712292208	T1D vs T2D
URS000096EEE8-rRNA	0,114330804	8,595030803	0,3800956	0,712292208	T1D vs T2D
URS00007D24CA-lncRNA	-0,658880158	2,948889293	0,380804508	0,712292208	T1D vs T2D
URS000063A6E6-rRNA	0,103218885	11,70425189	0,380812287	0,712292208	T1D vs T2D
URS0000097924-lncRNA	-0,297984516	5,60446115	0,383940773	0,712292208	T1D vs T2D
URS000067843B-Y_RNA	-0,344002103	5,925250166	0,384059527	0,712292208	T1D vs T2D
URS0000287398-tRNA	-0,555328917	2,440463804	0,385726521	0,712292208	T1D vs T2D
URS0000611F3E-lncRNA	-0,48087747	2,798037489	0,385863428	0,712292208	T1D vs T2D
URS00001AA18A-lncRNA	-0,468870923	1,870939952	0,386074522	0,712292208	T1D vs T2D
URS00000C7470-antisense	-0,485206937	3,497403787	0,386860005	0,712292208	T1D vs T2D
URS0000AAD5AA-lncRNA	-0,699544199	10,83600588	0,3879275	0,712292208	T1D vs T2D
URS000020220C-antisense	-0,480934548	2,749759905	0,388014497	0,712292208	T1D vs T2D
URS000000199E-lncRNA	0,348862396	6,442848869	0,389148791	0,712292208	T1D vs T2D
URS00002B7986-antisense	-0,4540914	1,782132427	0,389167062	0,712292208	T1D vs T2D
URS00006E19EA-Y_RNA	0,334451259	9,597384946	0,390736544	0,712292208	T1D vs T2D

URS00002C4609-lncRNA	-0,506002441	2,010894744	0,390863347	0,712292208	T1D vs T2D
URS0000701607-Y_RNA	-0,478744557	3,386959123	0,391302157	0,712292208	T1D vs T2D
URS0000664EAD-tRNA	-0,540112765	2,046606184	0,393145479	0,712841899	T1D vs T2D
URS00003D279B-lncRNA	-0,46599492	3,869764687	0,393990549	0,712841899	T1D vs T2D
URS000091563C-rRNA	-0,099942407	10,26950568	0,396915548	0,712841899	T1D vs T2D
URS00006361F3-Y_RNA	-0,634709259	4,856921385	0,397541137	0,712841899	T1D vs T2D
URS000075AF5F-antisense	-0,512887803	2,789120304	0,399908182	0,712841899	T1D vs T2D
URS00003008EB-lncRNA	-0,406458685	4,342465078	0,399935666	0,712841899	T1D vs T2D
URS000050471D-lncRNA	-0,525422708	2,378838415	0,400691447	0,712841899	T1D vs T2D
URS00001BF716-lncRNA	0,16118941	7,608759782	0,402509831	0,712841899	T1D vs T2D
URS00001D0305-lncRNA	-0,15131084	7,373693454	0,402549523	0,712841899	T1D vs T2D
URS00002D2D8F-misc_RNA]	0,526383742	1,939760416	0,402880941	0,712841899	T1D vs T2D
URS0000941729-rRNA	0,409773298	4,187919105	0,403393296	0,712841899	T1D vs T2D
URS0000920CBD-rRNA	-1,041530958	5,869762745	0,403841762	0,712841899	T1D vs T2D
URS00009C60C3-lncRNA	0,635696567	3,075209684	0,405221573	0,713475766	T1D vs T2D
URS00006C246E-snRNA	-0,534730725	4,016094545	0,406562259	0,714037736	T1D vs T2D
URS00002F2DED-lncRNA	0,43927934	4,602442219	0,411006992	0,71829318	T1D vs T2D
URS00002840A7-lncRNA	0,472497577	2,142345929	0,411040446	0,71829318	T1D vs T2D
URS000096D31D-rRNA	-0,155520463	10,50581429	0,412449523	0,718958146	T1D vs T2D
URS0000701637-rRNA	-0,496977889	2,670549892	0,414204201	0,720220737	T1D vs T2D
URS0000504A1A-lncRNA	-0,425066706	1,470603839	0,416625512	0,722633332	T1D vs T2D
URS0000378BB8-lncRNA	-0,404286526	4,18272789	0,418035927	0,723284932	T1D vs T2D
URS00001AE429-lncRNA	-0,499594704	3,26760494	0,420173522	0,725188376	T1D vs T2D
URS00000AED6F-tRNA	-0,375156602	6,83625311	0,422498032	0,727404247	T1D vs T2D
URS0000391A04-lncRNA	-0,338058456	5,482878094	0,426538956	0,731448097	T1D vs T2D
URS000098604A-rRNA	-0,448626721	1,872688165	0,426939662	0,731448097	T1D vs T2D
URS000047EBB5-tRNA	-0,503483544	2,438958701	0,434638135	0,740264805	T1D vs T2D
URS000090169F-rRNA	0,422072652	1,691620139	0,436284597	0,740264805	T1D vs T2D
URS000099BDEE-rRNA	0,448843885	5,090607985	0,4366854	0,740264805	T1D vs T2D
URS000060D3B6-lncRNA	0,595356201	4,588963786	0,436960703	0,740264805	T1D vs T2D
URS0000082453-lncRNA	0,421753467	2,456766093	0,437527092	0,740264805	T1D vs T2D
URS000061A10B-tRNA	0,537702337	2,36803002	0,438440099	0,740264805	T1D vs T2D
URS000035D229-lncRNA	-0,618404081	3,593153184	0,439974112	0,740523079	T1D vs T2D
URS00007E2F7C-lncRNA	-0,473882284	3,279760012	0,440711875	0,740523079	T1D vs T2D
URS000032B6B6-snRNA	-0,161991056	8,295722512	0,445658789	0,747039552	T1D vs T2D
URS0000986F6F-rRNA	-0,343037737	5,818355153	0,44718694	0,747807825	T1D vs T2D
URS000008089F-lncRNA	-0,489876436	3,494287219	0,451015719	0,750625432	T1D vs T2D
URS000051CFB0-lncRNA	-0,699917221	3,801776289	0,451019573	0,750625432	T1D vs T2D
URS000095D156-rRNA	0,30731006	6,045790564	0,458330487	0,75689679	T1D vs T2D
URS000048B807-lncRNA	-0,366917371	4,663045601	0,460767232	0,75689679	T1D vs T2D
URS00001142E8-lncRNA	0,271777093	5,750896426	0,461005429	0,75689679	T1D vs T2D
URS000096B970-rRNA	-0,099497094	10,27519744	0,4611364	0,75689679	T1D vs T2D
URS0000A9525F-antisense	-0,40165553	2,995531648	0,461426904	0,75689679	T1D vs T2D
URS000068089E-Y_RNA	-0,434715164	2,479289353	0,461564345	0,75689679	T1D vs T2D
URS000022DD4A-tRNA	-0,169481404	9,64520168	0,462858516	0,75689679	T1D vs T2D
URS0000759AE0-lncRNA	-0,386838717	1,586466937	0,463654919	0,75689679	T1D vs T2D
URS0000502C74-tRNA	0,129616032	14,17701257	0,466000978	0,75689679	T1D vs T2D
URS00008119F8-lncRNA	-0,464711671	3,00844528	0,46665023	0,75689679	T1D vs T2D
URS00000EB76F-lncRNA	-0,426811095	2,382363845	0,466698879	0,75689679	T1D vs T2D
URS00001C6042-antisense	-0,409474552	1,983328915	0,472876394	0,761416495	T1D vs T2D
URS0000399BDA-lncRNA	0,3933565	5,117165985	0,474694675	0,761416495	T1D vs T2D
URS000050FA69-lncRNA	-0,374013749	3,930388911	0,475159866	0,761416495	T1D vs T2D
URS000097FEDE-rRNA	-0,375584635	3,690975927	0,475168788	0,761416495	T1D vs T2D
URS00006F135B-Y_RNA	-0,266893334	3,60198893	0,475427227	0,761416495	T1D vs T2D
URS000075D353-lncRNA	-0,375725689	1,653940152	0,476021471	0,761416495	T1D vs T2D
URS000020AD62-lncRNA	0,379336467	1,816448524	0,478644401	0,76386401	T1D vs T2D
URS000067474D-tRNA	-0,438059707	5,826738993	0,481145743	0,764583993	T1D vs T2D
URS000064D54F-tRNA	-0,384951667	2,523144202	0,4812832	0,764583993	T1D vs T2D
URS00000CD33E-antisense	0,369095192	3,630533758	0,484561161	0,768045922	T1D vs T2D
URS0000667737-rRNA	-0,815930574	4,834926248	0,486455888	0,769304673	T1D vs T2D
URS00007116F9-Y_RNA	-0,376694691	3,042319437	0,488768539	0,769558176	T1D vs T2D
URS00002D33E9-lncRNA	0,369125468	2,231641646	0,488818069	0,769558176	T1D vs T2D
URS00001D0B96-lncRNA	-0,08979811	10,38627035	0,492107017	0,771882558	T1D vs T2D
URS000091CD45-rRNA	0,36635595	6,581914152	0,492655529	0,771882558	T1D vs T2D
URS00008120D6-lncRNA	-0,367815229	1,844896317	0,49393856	0,771882558	T1D vs T2D
URS000024383A-lncRNA	0,370277584	1,637698963	0,494711568	0,771882558	T1D vs T2D

URS000000898B-lncRNA	-0,421056228	2,509136511	0,497316678	0,773476246	T1D vs T2D
URS00000734D4-lncRNA	0,328321466	2,320562453	0,498457323	0,773476246	T1D vs T2D
URS000097CDD0-rRNA	0,15146942	6,617743447	0,49932515	0,773476246	T1D vs T2D
URS00005B5F85-lncRNA	-0,371520158	5,187781526	0,500159175	0,773476246	T1D vs T2D
URS00000DA554-lncRNA	-0,385233145	2,522581542	0,501584141	0,773967582	T1D vs T2D
URS000075D341-rRNA	0,617108841	3,481242239	0,50273323	0,774032	T1D vs T2D
URS0000515855-lncRNA	-0,349805405	2,502117237	0,504462088	0,774986813	T1D vs T2D
URS00009A7848-rRNA	0,082786862	11,54132926	0,51012662	0,78134491	T1D vs T2D
URS00004227BE-lncRNA	-0,382444776	1,875905236	0,511755162	0,78134491	T1D vs T2D
URS00003C2ECD-antisense	-0,435528209	2,559377792	0,512717632	0,78134491	T1D vs T2D
URS00003C98B0-lncRNA	-0,396636554	3,045496821	0,51307198	0,78134491	T1D vs T2D
URS000030C934-lncRNA	-0,321943969	4,417263546	0,520214693	0,788633696	T1D vs T2D
URS0000ABD82A-rRNA	-0,950899145	6,247493519	0,521495427	0,788633696	T1D vs T2D
URS000063455F-rRNA	-0,358049184	2,601127664	0,521552034	0,788633696	T1D vs T2D
URS00003CBFB8-lncRNA	-0,344862639	2,075423342	0,522371103	0,788633696	T1D vs T2D
URS000002FD7D-lncRNA	-0,339881676	1,677824648	0,524314194	0,789861254	T1D vs T2D
URS000097171C-SRP_RNA	-0,346212619	2,924722071	0,528363158	0,794249135	T1D vs T2D
URS00003F2CFE-lncRNA	-0,350410195	4,520438351	0,53225308	0,79837962	T1D vs T2D
URS00005FFC78-lncRNA	0,356308194	2,519542247	0,5351435	0,800996373	T1D vs T2D
URS00001A72CE-tRNA	-0,355373031	4,122188898	0,538750998	0,804544482	T1D vs T2D
URS00006EBF05-misc_RNA]	0,565560944	3,622681484	0,5399964	0,804544482	T1D vs T2D
URS00008B26D4-antisense	-0,324447024	4,369068967	0,541022378	0,804544482	T1D vs T2D
URS00004D7012-lncRNA	-0,434872689	2,906892949	0,543979002	0,804544482	T1D vs T2D
URS00008BA2B6-lncRNA	-0,319571183	2,826253026	0,545074507	0,804544482	T1D vs T2D
URS00005D1950-antisense	-0,317474539	2,067377939	0,545194904	0,804544482	T1D vs T2D
URS0000944F10-rRNA	-0,243106026	5,261748123	0,546594827	0,804544482	T1D vs T2D
URS00004199FB-antisense	0,393922824	3,00203969	0,54672193	0,804544482	T1D vs T2D
URS00007BE6D3-lncRNA	0,839059041	10,22836282	0,550887682	0,807756718	T1D vs T2D
URS0000635088-tRNA	-0,290285726	10,57667068	0,551215957	0,807756718	T1D vs T2D
URS000013899F-tRNA	0,290218467	4,501396588	0,553266855	0,809065966	T1D vs T2D
URS000018267A-antisense	-0,379263848	3,83534964	0,559232011	0,816081787	T1D vs T2D
URS0000AABA8B-lncRNA	0,145844499	9,325488704	0,565429955	0,823407372	T1D vs T2D
URS000069466F-Y_RNA	-0,163094118	7,597826288	0,567230491	0,823781234	T1D vs T2D
URS000009738A-lncRNA	0,340985532	2,916436163	0,568228256	0,823781234	T1D vs T2D
URS00005E51DB-lncRNA	0,272926977	4,114269415	0,569897247	0,823781234	T1D vs T2D
URS000062FB25-rRNA	0,074737801	9,742461609	0,57184761	0,823781234	T1D vs T2D
URS0000166FF8-lncRNA	-0,273098209	4,546451597	0,57295132	0,823781234	T1D vs T2D
URS00005AAAF0-antisense	-0,316205889	3,447551336	0,573121664	0,823781234	T1D vs T2D
URS00006271F5-Y_RNA	-0,253078852	3,66489103	0,573936282	0,823781234	T1D vs T2D
URS00003D60C0-lncRNA	0,302300473	1,735581371	0,578434818	0,827726178	T1D vs T2D
URS000075AC32-lncRNA	0,332011006	2,916319528	0,579053078	0,827726178	T1D vs T2D
URS00002064F6-tRNA	0,326452747	7,265714406	0,581092918	0,828946836	T1D vs T2D
URS00006481F8-Y_RNA	0,168252084	7,303708498	0,583817749	0,831137691	T1D vs T2D
URS00006B479B-tRNA	-0,247211502	1,462161631	0,589858879	0,835845032	T1D vs T2D
URS00009554B1-rRNA	-0,278081059	4,916492139	0,590495443	0,835845032	T1D vs T2D
URS0000A81496-Y_RNA	0,353631662	2,190786251	0,590711654	0,835845032	T1D vs T2D
URS00005B30A9-tRNA	-0,35244332	2,750819629	0,592164107	0,836207496	T1D vs T2D
URS000014D40F-tRNA	-0,381061715	3,081622422	0,595407317	0,837747713	T1D vs T2D
URS000033F395-lncRNA	-0,290882435	1,897833694	0,595651807	0,837747713	T1D vs T2D
URS0000381123-lncRNA	-0,296254802	2,471894131	0,599609401	0,840899241	T1D vs T2D
URS0000543B4D-lncRNA	-0,314455772	2,605909076	0,601825465	0,840899241	T1D vs T2D
URS000077A114-lncRNA	0,087803898	7,694058287	0,601959633	0,840899241	T1D vs T2D
URS00002C130C-tRNA	-0,463754069	3,549564243	0,602704606	0,840899241	T1D vs T2D
URS00004B48CD-lncRNA	-0,292450555	1,80838911	0,605438564	0,843030988	T1D vs T2D
URS0000022477-lncRNA	-0,286889085	1,898475606	0,608445637	0,843952412	T1D vs T2D
URS0000093DF5-lncRNA	-0,286266265	2,157430584	0,608515044	0,843952412	T1D vs T2D
URS00002811B6-lncRNA	0,265245456	3,62513136	0,611042542	0,845779676	T1D vs T2D
URS000092B92B-rRNA	-0,703253207	5,336196426	0,613721807	0,847809374	T1D vs T2D
URS00006FF680-Y_RNA	0,290002552	2,916835355	0,616080978	0,849389751	T1D vs T2D
URS00001B506A-tRNA	0,332281562	2,259860507	0,617413604	0,849551396	T1D vs T2D
URS000031A3C9-lncRNA	-0,249764659	4,013881647	0,618747667	0,84971438	T1D vs T2D
URS00002EA13A-lncRNA	-0,275394141	2,908290455	0,621046172	0,85008545	T1D vs T2D
URS0000228E94-lncRNA	0,288651139	2,335958891	0,622522295	0,85008545	T1D vs T2D
URS0000918AFB-rRNA	0,120971283	8,610055402	0,623009216	0,85008545	T1D vs T2D
URS000019B78E-tRNA	0,405261044	3,458896569	0,62440929	0,85008545	T1D vs T2D
URS00008FEDF1-rRNA	-0,296838749	2,23767686	0,625763016	0,85008545	T1D vs T2D

URS00001F3EF6-lncRNA	-0,332977125	2,384806636	0,626314745	0,85008545	T1D vs T2D
URS0000063647-antisense	-0,145861862	6,169603323	0,631275715	0,855158381	T1D vs T2D
URS0000811AFB-antisense	0,25646226	2,040750323	0,64108927	0,866772534	T1D vs T2D
URS0000780E15-lncRNA	-0,291613602	2,764657639	0,643328068	0,866920874	T1D vs T2D
URS000090AA7A-rRNA	-0,271848637	2,615865166	0,643731449	0,866920874	T1D vs T2D
URS00005D36AA-lncRNA	-0,273408744	2,523255562	0,644919678	0,866920874	T1D vs T2D
URS00006C0715-rRNA	0,066081	11,18891955	0,646576425	0,867479694	T1D vs T2D
URS00006428FD-Y_RNA	-0,197154221	6,936586919	0,651783969	0,87055608	T1D vs T2D
URS00006AD70A-tRNA	0,279539	4,843339833	0,656359661	0,87055608	T1D vs T2D
URS000045E276-antisense	-0,237318748	7,301870906	0,659637709	0,87055608	T1D vs T2D
URS00004E5B9A-lncRNA	0,23790989	1,8276158	0,659770093	0,87055608	T1D vs T2D
URS000062F68C-rRNA	-0,058604968	10,93938784	0,660775317	0,87055608	T1D vs T2D
URS000049BB82-lncRNA	-0,215110701	4,039331254	0,661358707	0,87055608	T1D vs T2D
URS00000B8842-lncRNA	0,202159847	4,912142401	0,662712913	0,87055608	T1D vs T2D
URS00001DFAE4-lncRNA	-0,303520708	3,289114858	0,66296119	0,87055608	T1D vs T2D
URS0000086FDD-antisense	0,295212151	2,945683418	0,663695193	0,87055608	T1D vs T2D
URS000056B231-lncRNA	0,2560211	4,1586305	0,66471414	0,87055608	T1D vs T2D
URS0000593A4A-lncRNA	-0,262100455	3,380617038	0,66517366	0,87055608	T1D vs T2D
URS0000493225-tRNA	-0,121834311	7,026724216	0,666289698	0,87055608	T1D vs T2D
URS00003F2105-lncRNA	0,245702251	2,406932528	0,666801746	0,87055608	T1D vs T2D
URS00007CA557-lncRNA	0,102935658	9,085280894	0,667373057	0,87055608	T1D vs T2D
URS000003B6E2-lncRNA	-0,328878818	2,652355198	0,66757928	0,87055608	T1D vs T2D
URS0000702B40-Y_RNA	-0,281548967	2,572703997	0,668796302	0,87055608	T1D vs T2D
URS0000A8428E-Y_RNA	0,335862791	5,101971983	0,672012986	0,873117244	T1D vs T2D
URS0000977FD2-SRP_RNA	-0,235427956	2,037495207	0,674701308	0,874308198	T1D vs T2D
URS0000640661-Y_RNA	-0,200831961	11,13979054	0,676365982	0,874308198	T1D vs T2D
URS00006F5B12-Y_RNA	-0,250787962	5,757189507	0,676682025	0,874308198	T1D vs T2D
URS000075B143-lncRNA	0,224763468	2,661955926	0,681873327	0,879390139	T1D vs T2D
URS00005BB5C9-antisense	0,214615823	1,956136305	0,68456454	0,881235015	T1D vs T2D
URS00006952C9-Y_RNA	-0,28696831	4,013121466	0,686503687	0,88210676	T1D vs T2D
URS000018BCE5-antisense	0,230819678	2,555076799	0,688724984	0,882586216	T1D vs T2D
URS000066C003-Y_RNA]	0,470870567	5,557417709	0,689402108	0,882586216	T1D vs T2D
URS000019B78E-misc_RNA]	-0,326089671	3,588733066	0,694655114	0,88768542	T1D vs T2D
URS0000A90D33-snRNA	0,242582753	3,877391031	0,698991062	0,890280266	T1D vs T2D
URS0000543A1A-lncRNA	0,19867148	5,169008445	0,700028468	0,890280266	T1D vs T2D
URS000066AF0D-Y_RNA	-0,214343137	12,73751355	0,700506647	0,890280266	T1D vs T2D
URS00006F7C66-rRNA	0,214683658	1,804743197	0,703643824	0,89122909	T1D vs T2D
URS00000AF0EF-lncRNA	0,213480412	3,804446661	0,70380323	0,89122909	T1D vs T2D
URS0000488EA7-lncRNA	0,224215072	2,312667922	0,709000559	0,896186964	T1D vs T2D
URS0000A8472C-Y_RNA	-0,235827259	3,823381815	0,713851518	0,900689911	T1D vs T2D
URS000023352F-lncRNA	0,188117187	2,158198536	0,716348786	0,901965938	T1D vs T2D
URS0000576D5D-lncRNA	-0,198099175	1,73318224	0,717443579	0,901965938	T1D vs T2D
URS00009C6042-lncRNA	0,104788653	6,706154397	0,720246665	0,902281049	T1D vs T2D
URS0000005270-rRNA	0,32176127	6,166410111	0,720275859	0,902281049	T1D vs T2D
URS0000907244-rRNA	-0,086729845	6,675718226	0,724591893	0,902391155	T1D vs T2D
URS000014D914-antisense	-0,174368226	4,160373223	0,724783892	0,902391155	T1D vs T2D
URS00003A00A4-lncRNA	0,20847923	3,039610494	0,724793216	0,902391155	T1D vs T2D
URS000033268A-lncRNA	-0,193299093	4,132479222	0,725527653	0,902391155	T1D vs T2D
URS000055C9F8-antisense	0,158856013	4,017918826	0,72812491	0,902809536	T1D vs T2D
URS0000812136-lncRNA	0,076831522	9,121672555	0,728447179	0,902809536	T1D vs T2D
URS0000955796-rRNA	0,039782499	10,7330721	0,730317518	0,902927065	T1D vs T2D
URS0000995AA3-rRNA	-0,112257653	8,311403687	0,731125492	0,902927065	T1D vs T2D
URS0000417E86-lncRNA	0,203633051	2,54225615	0,733641164	0,904435933	T1D vs T2D
URS0000696377-Y_RNA	-0,182931711	2,848796511	0,741370824	0,912355997	T1D vs T2D
URS0000653BD1-Y_RNA	0,163370143	4,226797259	0,750233901	0,921640591	T1D vs T2D
URS000015D954-antisense	-0,175160931	1,950160466	0,754770152	0,924493759	T1D vs T2D
URS000064FE59-rRNA	0,042852661	11,09750364	0,755201626	0,924493759	T1D vs T2D
URS00003CAB47-antisense	0,178299702	3,257126248	0,759745668	0,92794288	T1D vs T2D
URS0000767DAE-lncRNA	-0,107090896	5,637395825	0,760674206	0,92794288	T1D vs T2D
URS00002968BD-antisense	0,098298737	7,356778365	0,762457942	0,928498434	T1D vs T2D
URS000038EEDC-lncRNA	-0,210568018	3,360541304	0,766580463	0,931895206	T1D vs T2D
URS0000762146-antisense	0,109015805	5,166011847	0,769465267	0,933778162	T1D vs T2D
URS00009C6137-lncRNA	-0,146321242	3,379050945	0,77617133	0,93951702	T1D vs T2D
URS00001DE9CD-antisense	-0,166620378	3,351892383	0,778796611	0,93951702	T1D vs T2D
URS00007BC71B-lncRNA	-0,167297297	1,944342872	0,778803094	0,93951702	T1D vs T2D
URS0000605144-antisense	-0,159333228	2,730860041	0,779570631	0,93951702	T1D vs T2D

URS0000042FD3-lncRNA	-0,140525953	3,024717754	0,782590537	0,941533194	T1D vs T2D
URS0000689904-Y_RNA	-0,08660344	0,877747286	0,784596383	0,942324521	T1D vs T2D
URS0000038B1E-antisense	-0,141179143	3,244363175	0,788861871	0,94582238	T1D vs T2D
URS000012C80D-lncRNA	-0,144494228	2,726619231	0,794489505	0,950938637	T1D vs T2D
URS000064217E-Y_RNA	0,149009021	2,41296644	0,801906903	0,958175941	T1D vs T2D
URS000013BB40-lncRNA	-0,141562608	2,666260296	0,805845732	0,95841066	T1D vs T2D
URS000075D28F-lncRNA	-0,138316333	2,277607756	0,805932416	0,95841066	T1D vs T2D
URS0000282AB2-tRNA	-0,191611589	4,591558308	0,807771205	0,95841066	T1D vs T2D
URS00006A2BF7-Y_RNA	0,142817244	10,1864015	0,808678265	0,95841066	T1D vs T2D
URS0000417A0F-tRNA	-0,100287684	7,465949428	0,810122438	0,95841066	T1D vs T2D
URS00006F3305-rRNA	0,025125546	11,47052611	0,81230619	0,95841066	T1D vs T2D
URS000019F398-lncRNA	0,116846206	1,659002941	0,817316346	0,95841066	T1D vs T2D
URS000071C9A6-Y_RNA	-0,083570776	2,18048286	0,817792563	0,95841066	T1D vs T2D
URS00002C2371-lncRNA	-0,126134431	3,691720987	0,817941996	0,95841066	T1D vs T2D
URS00003CA240-antisense	-0,093589834	5,133383215	0,82070337	0,95841066	T1D vs T2D
URS0000626233-Y_RNA	0,08661098	4,209755669	0,824048027	0,95841066	T1D vs T2D
URS00009407E6-rRNA	-0,132244955	2,030446175	0,82642287	0,95841066	T1D vs T2D
URS00005A57D3-lncRNA	0,128951888	2,579526429	0,826851508	0,95841066	T1D vs T2D
URS000000513C-antisense	0,085620508	4,840741267	0,827517912	0,95841066	T1D vs T2D
URS00002CC2A4-antisense	0,124659118	2,224906264	0,827540512	0,95841066	T1D vs T2D
URS00001A86BB-tRNA	0,107823405	2,65628615	0,828190115	0,95841066	T1D vs T2D
URS00006AD81D-Y_RNA	0,130753541	1,867732365	0,829073371	0,95841066	T1D vs T2D
URS00006CFDFE-Y_RNA	0,090296966	2,249644641	0,831691781	0,95841066	T1D vs T2D
URS00006CE1FB-rRNA	0,209810305	5,102179845	0,832181286	0,95841066	T1D vs T2D
URS00007DFA49-antisense	0,146904049	2,453720457	0,836016562	0,95841066	T1D vs T2D
URS0000704D22-rRNA	-0,037277521	14,1067509	0,836769675	0,95841066	T1D vs T2D
URS00005F1728-lncRNA	0,10956152	2,661157063	0,837309439	0,95841066	T1D vs T2D
URS00005BC8EC-lncRNA	0,155152421	5,143820106	0,83860829	0,95841066	T1D vs T2D
URS00002FBC9E-lncRNA	-0,121886477	3,539604284	0,838608956	0,95841066	T1D vs T2D
URS00004C5EDB-lncRNA	0,126898211	2,83469135	0,838700267	0,95841066	T1D vs T2D
URS0000089048-antisense	-0,107603328	1,859342356	0,840003863	0,95841066	T1D vs T2D
URS00000A586F-lncRNA	0,063442771	0,916847513	0,841595753	0,95841066	T1D vs T2D
URS000037D0FB-tRNA	-0,135943851	2,792154767	0,841713846	0,95841066	T1D vs T2D
URS0000633321-snoRNA	-0,049917594	7,948494424	0,841962315	0,95841066	T1D vs T2D
URS00003D4983-antisense	-0,111096014	1,851867325	0,844596318	0,95841066	T1D vs T2D
URS0000A9F786-lncRNA	0,131687079	3,099717163	0,844607964	0,95841066	T1D vs T2D
URS000075BB81-lncRNA	-0,105912636	2,83062659	0,846192192	0,958652095	T1D vs T2D
URS00006144FC-lncRNA	0,077372067	7,117689109	0,851226666	0,962307749	T1D vs T2D
URS000055B99E-lncRNA	0,11715428	2,716513459	0,853265807	0,962307749	T1D vs T2D
URS00006B33E0-Y_RNA	-0,075452697	14,2625662	0,853549077	0,962307749	T1D vs T2D
URS00004BE455-lncRNA	-0,076142188	5,228003516	0,85620448	0,962504074	T1D vs T2D
URS000052A1C9-tRNA	-0,0939596	4,427113529	0,856477159	0,962504074	T1D vs T2D
URS00005C220A-antisense	0,112561534	2,894185806	0,863128528	0,968289978	T1D vs T2D
URS0000038397-lncRNA	-0,09227762	5,458381823	0,865230942	0,968289978	T1D vs T2D
URS00004DDD71-lncRNA	0,060780184	5,854746665	0,865781454	0,968289978	T1D vs T2D
URS000091B709-rRNA	-0,028148596	9,600448226	0,868241391	0,969489987	T1D vs T2D
URS000047A7F4-misc_RNA]	-0,147683598	4,238929678	0,871695177	0,96970014	T1D vs T2D
URS00007D61AD-antisense	-0,118799988	2,769938297	0,871851593	0,96970014	T1D vs T2D
URS00003EAC96-antisense	-0,09896754	2,99991317	0,872591399	0,96970014	T1D vs T2D
URS000023F3B4-lncRNA	0,074303935	3,955198744	0,875711641	0,971622916	T1D vs T2D
URS000075BA00-lncRNA	-0,194421124	7,953608312	0,878221977	0,972863965	T1D vs T2D
URS0000177135-lncRNA	0,121830627	3,040442935	0,882923457	0,976344095	T1D vs T2D
URS00001823EB-lncRNA	-0,086414448	3,483455808	0,88769712	0,976344095	T1D vs T2D
URS000011812A-antisense	-0,068623376	4,132007293	0,887889375	0,976344095	T1D vs T2D
URS0000271FCA-antisense	-0,063366822	5,084020409	0,888944325	0,976344095	T1D vs T2D
URS00004F6629-lncRNA	0,053691926	1,127896398	0,891740302	0,976344095	T1D vs T2D
URS0000669D0F-snRNA	0,080214635	2,759909955	0,894680764	0,976344095	T1D vs T2D
URS000068483A-rRNA	0,077041301	2,397770416	0,896158414	0,976344095	T1D vs T2D
URS0000A77003-lncRNA	-0,072351703	2,821180105	0,898967555	0,976344095	T1D vs T2D
URS000005F65D-antisense	-0,063758242	4,947813537	0,901668219	0,976344095	T1D vs T2D
URS000002E930-antisense	0,082756204	4,041839683	0,902196881	0,976344095	T1D vs T2D
URS000090DF6D-rRNA	-0,013717765	11,93047681	0,902966201	0,976344095	T1D vs T2D
URS000047A7F4-rRNA	0,115187674	4,287976289	0,903325972	0,976344095	T1D vs T2D
URS000015BB33-lncRNA	-0,060974014	3,745978664	0,903487293	0,976344095	T1D vs T2D
URS000013BA50-antisense	-0,074710712	1,971579125	0,905087254	0,976344095	T1D vs T2D
URS0000257C23-lncRNA	0,078613476	4,584581553	0,906437846	0,976344095	T1D vs T2D

URS00004F482C-lncRNA	-0,067935228	2,9888845	0,906829594	0,976344095	T1D vs T2D
URS00002AECD7-lncRNA	-0,073857266	2,148873116	0,907869961	0,976344095	T1D vs T2D
URS00001D4EE9-tRNA	0,077133218	2,839543714	0,908082004	0,976344095	T1D vs T2D
URS00000B6370-lncRNA	-0,051090073	3,6717613	0,909506214	0,976344095	T1D vs T2D
URS000094439F-rRNA	-0,013183759	11,62287065	0,910058664	0,976344095	T1D vs T2D
URS000030BAD5-tRNA	-0,029618251	9,963231125	0,91069578	0,976344095	T1D vs T2D
URS000052A6A6-lncRNA	-0,059189358	3,111397823	0,912456136	0,976733291	T1D vs T2D
URS00007CB156-lncRNA	-0,02189656	7,841069642	0,915163835	0,976768146	T1D vs T2D
URS00004CE099-lncRNA	0,087857148	3,298450018	0,915283456	0,976768146	T1D vs T2D
URS00006D4DB0-snRNA	-0,066729026	3,391553507	0,916945615	0,977050282	T1D vs T2D
URS00003F41E9-lncRNA	0,01980799	8,007323205	0,921656053	0,980574705	T1D vs T2D
URS0000A89523-lncRNA	0,056405357	2,553603804	0,923919375	0,981488819	T1D vs T2D
URS00004DA951-rRNA	0,027039417	10,73867185	0,93350378	0,98894477	T1D vs T2D
URS0000462D45-lncRNA	-0,042890183	3,263068544	0,933767594	0,98894477	T1D vs T2D
URS0000A827F4-antisense	0,051884039	8,224586053	0,935678545	0,989469445	T1D vs T2D
URS0000AA0C30-lncRNA	0,041523643	3,233082441	0,940042179	0,991105582	T1D vs T2D
URS00009AAC46-rRNA	-0,013381525	9,797442669	0,941431243	0,991105582	T1D vs T2D
URS00008120F8-lncRNA	-0,041994146	1,863766293	0,941630374	0,991105582	T1D vs T2D
URS000060A110-antisense	0,03310957	1,367875278	0,942897299	0,991105582	T1D vs T2D
URS00000E9A71-lncRNA	0,037439199	3,823249127	0,945284005	0,991672802	T1D vs T2D
URS00003870EC-lncRNA	-0,040904481	2,283625175	0,947637708	0,991672802	T1D vs T2D
URS0000028D07-lncRNA	0,032401089	5,403821621	0,948547432	0,991672802	T1D vs T2D
URS0000697465-tRNA	0,037598324	4,721333977	0,950209052	0,991672802	T1D vs T2D
URS00005BE013-lncRNA	-0,045516457	2,759573682	0,95053044	0,991672802	T1D vs T2D
URS00005AF1AA-antisense	-0,025820597	1,232925367	0,952048352	0,991776153	T1D vs T2D
URS00003875B8-lncRNA	-0,036348976	2,156965412	0,953554568	0,991867028	T1D vs T2D
URS00006E23A8-rRNA	-0,034637241	2,840982885	0,956063423	0,992279299	T1D vs T2D
URS0000432B92-antisense	-0,00927828	7,647433512	0,959639025	0,992279299	T1D vs T2D
URS00006F4537-tRNA	-0,030698553	4,037892922	0,959779794	0,992279299	T1D vs T2D
URS0000918BC5-rRNA	-0,043661089	3,816571784	0,960359628	0,992279299	T1D vs T2D
URS00003869EC-lncRNA	-0,032829012	2,98234075	0,961048763	0,992279299	T1D vs T2D
URS00000CCACD-antisense	-0,031271936	4,642488599	0,964933797	0,992440499	T1D vs T2D
URS000042199A-lncRNA	-0,027068546	2,107510848	0,965396542	0,992440499	T1D vs T2D
URS00004C82E1-antisense	0,026752925	4,084336348	0,96546429	0,992440499	T1D vs T2D
URS000094F5D5-rRNA	-0,016909116	7,000982595	0,970377194	0,99602593	T1D vs T2D
URS000038D781-lncRNA	0,020529867	2,120835096	0,9730976	0,997225801	T1D vs T2D
URS000075A823-rRNA	-0,042349961	6,786069906	0,974860118	0,997225801	T1D vs T2D
URS0000112A1A-antisense	0,015517055	4,257411498	0,976673718	0,997225801	T1D vs T2D
URS00002CFBEA-lncRNA	0,013695669	2,003500281	0,978403434	0,997225801	T1D vs T2D
URS00009843EB-rRNA	0,004676417	12,28502234	0,979623612	0,997225801	T1D vs T2D
URS0000418239-snRNA	-0,012581235	2,184896985	0,980106045	0,997225801	T1D vs T2D
URS000020BB55-lncRNA	-0,013675678	3,131770633	0,98299834	0,997895627	T1D vs T2D
URS00004AFADD-lncRNA	0,010033366	1,695028896	0,985980163	0,997895627	T1D vs T2D
URS0000766C83-lncRNA	-0,00924667	2,482009239	0,988265121	0,997895627	T1D vs T2D
URS000044BAE3-tRNA	-0,002688768	7,695683129	0,989828873	0,997895627	T1D vs T2D
URS00008116FE-lncRNA	-0,006692582	2,826119493	0,990420578	0,997895627	T1D vs T2D
URS00001AF592-antisense	0,007277404	3,230887811	0,9907277	0,997895627	T1D vs T2D
URS00006744D5-tRNA	-0,006152315	2,786516677	0,992364071	0,997895627	T1D vs T2D
URS00006D484A-rRNA	-0,000922762	11,67221899	0,992584403	0,997895627	T1D vs T2D
URS00005C7D80-antisense	-0,004703197	2,279201521	0,993612814	0,997895627	T1D vs T2D
URS00004EDF08-lncRNA	0,002526033	4,609717254	0,995262629	0,998118476	T1D vs T2D
URS000095C3C7-rRNA	-0,001218023	4,667191645	0,997876762	0,998643004	T1D vs T2D
URS00004AC036-lncRNA	0,000848032	4,515610764	0,998643004	0,998643004	T1D vs T2D
URS00000DA56A-lncRNA1	2,319304107	2,589490843	8,51406E-05	0,059513268	LADA vs T1D
URS000011BDAF-antisense1	1,96237033	2,89470373	0,000179298	0,062664608	LADA vs T1D
URS0000249329-lncRNA1	1,869172891	1,740243504	0,000305875	0,071268767	LADA vs T1D
URS000064F96B-Y_RNA1	-1,095195924	5,069805695	0,001382297	0,201272145	LADA vs T1D
URS0000495A30-lncRNA1	1,71197286	2,253557055	0,001439715	0,201272145	LADA vs T1D
URS00000D1D87-lncRNA1	1,648606142	1,722539329	0,001774896	0,206775357	LADA vs T1D
URS000069E2A5-tRNA1	1,941461144	3,421985706	0,002224965	0,217418259	LADA vs T1D
URS0000371842-lncRNA1	1,728637801	2,020987369	0,002753642	0,217418259	LADA vs T1D
URS00001EC8D7-snRNA1	1,245815754	4,577467935	0,002843025	0,217418259	LADA vs T1D
URS000093FF31-rRNA1	1,589050508	2,038384139	0,003110419	0,217418259	LADA vs T1D
URS0000278E1B-tRNA1	1,647518966	2,766067348	0,003670709	0,233256901	LADA vs T1D
URS0000478C87-lncRNA1	1,560511881	2,830369346	0,00423816	0,241662902	LADA vs T1D
URS0000196BD3-lncRNA1	1,577155287	2,056335374	0,004495187	0,241662902	LADA vs T1D

URS00005CEC24-lncRNA1	1,709166914	2,918669315	0,004840173	0,241662902	LADA vs T1D
URS000064C567-Y_RNA1	-0,654343718	5,523874961	0,006198566	0,278587243	LADA vs T1D
URS00002EDD28-lncRNA1	1,038408019	5,436899358	0,006376818	0,278587243	LADA vs T1D
URS00006C133C-tRNA1	1,483973751	3,680848373	0,009027061	0,371171511	LADA vs T1D
URS00009A5DA8-rRNA1	1,142487912	5,880019281	0,010038511	0,389828854	LADA vs T1D
URS000099C20E-rRNA1	1,612053461	3,326722503	0,012784885	0,411202497	LADA vs T1D
URS0000591DFC-lncRNA1	1,31052815	1,976731906	0,012918395	0,411202497	LADA vs T1D
URS00006E3DE1-snRNA1	1,62162115	3,143314554	0,01308817	0,411202497	LADA vs T1D
URS00009290F1-rRNA1	1,365377313	1,980245991	0,013226611	0,411202497	LADA vs T1D
URS0000527686-lncRNA1	0,97615037	1,162280834	0,013530268	0,411202497	LADA vs T1D
URS00004D4BFC-lncRNA1	1,19250939	2,209794014	0,017128007	0,49885319	LADA vs T1D
URS00003B6188-lncRNA1	1,2396099	3,075648025	0,018280943	0,511135158	LADA vs T1D
URS000039557B-antisense1	1,251120599	1,694045712	0,020043067	0,534119591	LADA vs T1D
URS000071C9A6-Y_RNA1	-0,839587178	2,18048286	0,021302678	0,534119591	LADA vs T1D
URS0000716B70-tRNA1	1,175448086	3,560279134	0,023254325	0,534119591	LADA vs T1D
URS0000684E4B-tRNA1	-0,559576357	6,262078663	0,024125782	0,534119591	LADA vs T1D
URS00002E367B-antisense1	1,263108904	1,915955023	0,024759113	0,534119591	LADA vs T1D
URS00006B33E0-Y_RNA1	-0,913065907	14,2625662	0,026041879	0,534119591	LADA vs T1D
URS0000A9CA30-lncRNA1	1,241400151	3,103219008	0,028539286	0,534119591	LADA vs T1D
URS00000DE490-lncRNA1	1,096744162	3,392102095	0,029671319	0,534119591	LADA vs T1D
URS00002E81F5-lncRNA1	1,079522309	4,084000104	0,030761809	0,534119591	LADA vs T1D
URS000049B61C-antisense1	0,480662065	6,879627508	0,032170228	0,534119591	LADA vs T1D
URS0000ABD7E8-rRNA1	-1,979684933	10,98616314	0,032327253	0,534119591	LADA vs T1D
URS000097B164-rRNA1	0,92877847	5,947136128	0,032632095	0,534119591	LADA vs T1D
URS000057C597-antisense1	1,251254496	2,337861425	0,032830387	0,534119591	LADA vs T1D
URS00009C6070-lncRNA1	1,129306991	2,53934864	0,033002879	0,534119591	LADA vs T1D
URS00006F135B-Y_RNA1	-0,797848773	3,60198893	0,033164825	0,534119591	LADA vs T1D
URS0000920CBD-rRNA1	2,66288077	5,869762745	0,033502819	0,534119591	LADA vs T1D
URS00002C9C48-lncRNA1	-1,190912478	5,358879491	0,033577648	0,534119591	LADA vs T1D
URS0000AA0C30-lncRNA1	-1,169720775	3,233082441	0,033968509	0,534119591	LADA vs T1D
URS00006D1735-snRNA1	1,013150634	5,596569208	0,034033752	0,534119591	LADA vs T1D
URS00001E6C0A-lncRNA1	1,133858296	3,103912065	0,034409452	0,534119591	LADA vs T1D
URS0000415026-tRNA1	0,963484862	4,426745558	0,035149501	0,534119591	LADA vs T1D
URS00000A00A2-antisense1	1,314981243	4,833316746	0,036531399	0,543307398	LADA vs T1D
URS000011ABD1-lncRNA1	-1,323810851	2,282001703	0,038384838	0,553868335	LADA vs T1D
URS00009C606C-lncRNA1	-0,73726809	5,246519845	0,03882625	0,553868335	LADA vs T1D
URS00004D1520-lncRNA1	1,183066645	3,203873127	0,039731465	0,555445883	LADA vs T1D
URS0000633F75-ribozyme1	1,087740697	4,438697312	0,040866428	0,560110455	LADA vs T1D
URS0000397210-lncRNA1	1,102621458	2,372225381	0,04190684	0,563324635	LADA vs T1D
URS000075BB81-lncRNA1	1,104412024	2,83062659	0,043448219	0,573024627	LADA vs T1D
URS00000540AC-antisense1	1,091900214	3,451834854	0,047032848	0,585540084	LADA vs T1D
URS0000537899-antisense1	1,142403521	2,143848964	0,04752841	0,585540084	LADA vs T1D
URS00003AA49D-lncRNA1	-1,107567058	2,72551685	0,047648237	0,585540084	LADA vs T1D
URS00002D0015-lncRNA1	1,02610748	3,226559685	0,047747904	0,585540084	LADA vs T1D
URS0000A90D33-snRNA1	1,231344438	3,877391031	0,050876829	0,612203474	LADA vs T1D
URS00001DBD56-lncRNA1	0,988429927	3,720321434	0,051922508	0,612203474	LADA vs T1D
URS00002AD8DA-lncRNA1	1,020494689	2,376170134	0,052936847	0,612203474	LADA vs T1D
URS0000672E5A-rRNA1	0,706387034	5,608838646	0,053425482	0,612203474	LADA vs T1D
URS0000617C6A-antisense1	0,915085564	3,572347898	0,055090663	0,621102796	LADA vs T1D
URS0000767DAE-lncRNA1	0,670987811	5,637395825	0,05794049	0,642863536	LADA vs T1D
URS00001A86BB-tRNA1	-0,935046037	2,65628615	0,060882908	0,647058625	LADA vs T1D
URS0000365006-lncRNA1	1,024529957	2,397164181	0,060925861	0,647058625	LADA vs T1D
URS000021BDC3-snRNA1	1,003362475	2,084780028	0,061863913	0,647058625	LADA vs T1D
URS0000A77003-lncRNA1	-1,066776725	2,821180105	0,06294075	0,647058625	LADA vs T1D
URS0000997FE9-rRNA1	0,995065933	2,876398415	0,063773754	0,647058625	LADA vs T1D
URS00006C14B2-tRNA1	1,240425167	3,304706256	0,064105376	0,647058625	LADA vs T1D
URS000075DF54-lncRNA1	-0,606558918	6,14755677	0,06665434	0,647058625	LADA vs T1D
URS0000644222-tRNA1	-0,410165422	7,91366141	0,067041168	0,647058625	LADA vs T1D
URS000024B38F-tRNA1	1,019028755	4,404533737	0,067095077	0,647058625	LADA vs T1D
URS000029A6A6-antisense1	0,571115689	5,872434994	0,070062191	0,647058625	LADA vs T1D
URS000056B96A-antisense1	0,92059656	2,621722386	0,07093273	0,647058625	LADA vs T1D
URS00003F5471-lncRNA1	0,950026105	3,0793045	0,071204972	0,647058625	LADA vs T1D
URS0000664EAD-tRNA1	1,136017402	2,046606184	0,073449786	0,647058625	LADA vs T1D
URS0000006840-lncRNA1	0,912465591	3,802540443	0,07371274	0,647058625	LADA vs T1D
URS00004C6AFE-antisense1	0,755041757	5,444648432	0,074057532	0,647058625	LADA vs T1D
URS0000974435-SRP_RNA1	-0,928502027	2,886817066	0,074441369	0,647058625	LADA vs T1D

URS00001A48CC-lncRNA1	0,973205234	2,650296854	0,075551991	0,647058625	LADA vs T1D
URS0000907730-rRNA1	0,616339045	6,755248399	0,076698339	0,647058625	LADA vs T1D
URS00004E7DF9-lncRNA1	0,865965166	4,28189465	0,076894126	0,647058625	LADA vs T1D
URS0000134A86-antisense1	-1,167940702	3,351781773	0,077723574	0,647058625	LADA vs T1D
URS000031A1AE-antisense1	1,098926617	2,086098634	0,077758118	0,647058625	LADA vs T1D
URS000040CE64-lncRNA1	1,163135057	2,883976268	0,079626216	0,651520744	LADA vs T1D
URS000035D229-lncRNA1	-1,385529567	3,593153184	0,081431875	0,651520744	LADA vs T1D
URS0000A76F22-lncRNA1	0,966612321	2,591384678	0,081821284	0,651520744	LADA vs T1D
URS000034EAB6-lncRNA1	1,06948363	2,280705495	0,082401184	0,651520744	LADA vs T1D
URS00005B5F85-lncRNA1	0,955732129	5,187781526	0,082958075	0,651520744	LADA vs T1D
URS0000776086-lncRNA1	0,629206657	7,320712579	0,084406165	0,651520744	LADA vs T1D
URS0000766C83-lncRNA1	1,087865333	2,482009239	0,084818866	0,651520744	LADA vs T1D
URS0000422302-lncRNA1	0,894792054	2,914915679	0,088125832	0,668329279	LADA vs T1D
URS000075AD80-snRNA1	1,028288859	2,124304339	0,088919346	0,668329279	LADA vs T1D
URS00003475A2-lncRNA1	0,55477566	6,073704566	0,092376784	0,685106795	LADA vs T1D
URS00002D40C8-tRNA1	-0,411815384	7,830242615	0,093111796	0,685106795	LADA vs T1D
URS00006D1A54-Y_RNA1	0,862497956	3,828527713	0,095998995	0,688001932	LADA vs T1D
URS00007125F9-rRNA1	0,972443034	2,409823294	0,097476145	0,688001932	LADA vs T1D
URS00000FB60D-tRNA1	0,953118161	3,59514453	0,097761054	0,688001932	LADA vs T1D
URS000031A3C9-lncRNA1	0,83178863	4,013881647	0,098056011	0,688001932	LADA vs T1D
URS000071ED2F-tRNA1	1,056065976	2,991616318	0,099526212	0,688001932	LADA vs T1D
URS00001424D6-lncRNA1	0,949569373	1,834670251	0,09987177	0,688001932	LADA vs T1D
URS000096E1E3-rRNA1	0,97340695	2,495902245	0,101200006	0,688001932	LADA vs T1D
URS0000697465-tRNA1	-0,990609586	4,721333977	0,102273982	0,688001932	LADA vs T1D
URS00009554B1-rRNA1	-0,841886066	4,916492139	0,103431814	0,688001932	LADA vs T1D
URS0000075AD1-lncRNA1	0,867800131	1,794828601	0,104998331	0,688001932	LADA vs T1D
URS0000515429-lncRNA1	-0,603527136	7,164323775	0,105195782	0,688001932	LADA vs T1D
URS0000539731-antisense1	0,838497289	2,084997113	0,106488642	0,688001932	LADA vs T1D
URS00000EB76F-lncRNA1	0,947469246	2,382363845	0,106896868	0,688001932	LADA vs T1D
URS000038D781-lncRNA1	0,980740855	2,120835096	0,107284994	0,688001932	LADA vs T1D
URS0000391360-lncRNA1	0,964288019	2,256356542	0,110600276	0,69358037	LADA vs T1D
URS00000E0E97-antisense1	1,376301673	4,066934958	0,111193866	0,69358037	LADA vs T1D
URS00006C48EB-Y_RNA]1	1,05867857	2,788995838	0,111715293	0,69358037	LADA vs T1D
URS00004A68E9-antisense1	0,669089904	3,654195066	0,112123865	0,69358037	LADA vs T1D
URS0000282AB2-tRNA1	1,23795486	4,591558308	0,116534192	0,70408373	LADA vs T1D
URS00000E0DE1-antisense1	0,849757334	3,104520142	0,117831234	0,70408373	LADA vs T1D
URS00006D1C46-snRNA1	0,741850121	6,026449126	0,118748288	0,70408373	LADA vs T1D
URS0000630B8A-tRNA1	-0,732749573	5,357715317	0,119913034	0,70408373	LADA vs T1D
URS0000007D24-misc_RNA]1	2,275381248	6,091419652	0,120294214	0,70408373	LADA vs T1D
URS000076DAC1-lncRNA1	-0,641983018	5,661423845	0,121349475	0,70408373	LADA vs T1D
URS000067843B-Y_RNA1	-0,611672279	5,925250166	0,122101495	0,70408373	LADA vs T1D
URS000060A110-antisense1	0,716233024	1,367875278	0,122150237	0,70408373	LADA vs T1D
URS0000676AED-precursor_RNA1	-0,900066275	2,736959381	0,123077743	0,70408373	LADA vs T1D
URS0000667737-rRNA1	1,817290975	4,834926248	0,123894562	0,70408373	LADA vs T1D
URS00007D24CA-lncRNA1	1,147452451	2,948889293	0,127017983	0,707981894	LADA vs T1D
URS000062C4DE-tRNA1	0,872217107	3,676191563	0,129968921	0,707981894	LADA vs T1D
URS00000A7F2D-lncRNA1	0,801100913	2,135027243	0,130513899	0,707981894	LADA vs T1D
URS000070792C-rRNA1	0,843851278	1,756278837	0,130707619	0,707981894	LADA vs T1D
URS000038D8D3-tRNA1	-0,469120774	7,544518208	0,13084071	0,707981894	LADA vs T1D
URS0000811D9C-antisense1	0,557515796	0,958216717	0,13087076	0,707981894	LADA vs T1D
URS0000942121-rRNA1	0,11241327	14,48998565	0,134325676	0,707981894	LADA vs T1D
URS000047AE74-lncRNA1	0,860477919	2,522838605	0,134668187	0,707981894	LADA vs T1D
URS0000A8472C-Y_RNA1	0,960640727	3,823381815	0,136128726	0,707981894	LADA vs T1D
URS00000B8842-lncRNA1	-0,690569218	4,912142401	0,136288495	0,707981894	LADA vs T1D
URS000038803E-tRNA1	0,474742554	5,940754642	0,136916514	0,707981894	LADA vs T1D
URS000030BAD5-tRNA1	-0,391198809	9,963231125	0,139186698	0,707981894	LADA vs T1D
URS00002D33E9-lncRNA1	-0,787786218	2,231641646	0,139537033	0,707981894	LADA vs T1D
URS0000320E71-lncRNA1	0,726925071	2,909981717	0,139565144	0,707981894	LADA vs T1D
URS000013B42D-tRNA1	-0,504131326	16,60649921	0,139773249	0,707981894	LADA vs T1D
URS0000028D07-lncRNA1	0,742481135	5,403821621	0,140967893	0,708896093	LADA vs T1D
URS0000067204-antisense1	0,82454617	1,800108533	0,142267322	0,710320417	LADA vs T1D
URS00006C0413-Y_RNA1	0,886669009	2,155330761	0,145243111	0,715974082	LADA vs T1D
URS0000811AFB-antisense1	0,803037241	2,040750323	0,14544824	0,715974082	LADA vs T1D
URS000075D353-lncRNA1	0,764215949	1,653940152	0,147196635	0,719513621	LADA vs T1D
URS00004A7F9B-antisense1	-0,362663701	6,391504235	0,149126577	0,723885259	LADA vs T1D
URS000099D184-rRNA1	0,579901288	4,977808511	0,150377413	0,724922837	LADA vs T1D

URS000029CCC5-tRNA1	0,775176028	2,732517708	0,15683616	0,731738256	LADA vs T1D
URS0000233E9C-lncRNA1	-1,084605861	2,822247929	0,15781108	0,731738256	LADA vs T1D
URS00008BB15B-lncRNA1	0,944552913	2,482102831	0,15915272	0,731738256	LADA vs T1D
URS00007D6D04-lncRNA1	0,705395802	3,22797681	0,159852996	0,731738256	LADA vs T1D
URS000053EAB5-lncRNA1	-0,738660916	3,036408354	0,16196627	0,731738256	LADA vs T1D
URS000066AF0D-Y_RNA1	-0,77835473	12,73751355	0,162682516	0,731738256	LADA vs T1D
URS00006FC298-tRNA1	-0,870639872	2,919601035	0,162694523	0,731738256	LADA vs T1D
URS000069ED7F-Y_RNA1	0,826163317	4,579144673	0,162774312	0,731738256	LADA vs T1D
URS0000995AA3-rRNA1	-0,456681409	8,311403687	0,162878108	0,731738256	LADA vs T1D
URS0000022477-lncRNA1	0,779407065	1,898475606	0,164453303	0,731738256	LADA vs T1D
URS00003C98B0-lncRNA1	0,843440857	3,045496821	0,165241626	0,731738256	LADA vs T1D
URS00006C06E6-Y_RNA1	0,792669323	1,912207487	0,16681008	0,731738256	LADA vs T1D
URS0000624312-Y_RNA1	0,554524487	5,698207316	0,168937776	0,731738256	LADA vs T1D
URS0000561169-lncRNA1	0,78042233	3,951068111	0,16919195	0,731738256	LADA vs T1D
URS00004C5EDB-lncRNA1	0,855882155	2,83469135	0,17018484	0,731738256	LADA vs T1D
URS00009C6074-antisense1	0,880491739	2,11618012	0,170603586	0,731738256	LADA vs T1D
URS00002200B0-lncRNA1	0,803382777	3,176288712	0,171395727	0,731738256	LADA vs T1D
URS000064506B-tRNA1	0,872613386	2,909810805	0,171662168	0,731738256	LADA vs T1D
URS000060D3B6-lncRNA1	-1,042582033	4,588963786	0,171681079	0,731738256	LADA vs T1D
URS00004199FB-antisense1	-0,886127524	3,00203969	0,175537286	0,743639777	LADA vs T1D
URS00006E1108-Y_RNA1	0,456624901	4,049124964	0,177445627	0,747195745	LADA vs T1D
URS000048EB2F-antisense1	0,764999904	2,157167592	0,179946771	0,753190376	LADA vs T1D
URS000012730D-lncRNA1	-0,773556496	3,696451386	0,187329009	0,779422485	LADA vs T1D
URS00006EB1B5-Y_RNA1	0,678164611	4,084821132	0,18911207	0,782185427	LADA vs T1D
URS00003C9A26-tRNA1	-0,244071649	8,822917147	0,193041908	0,793742903	LADA vs T1D
URS00000AD0C2-lncRNA1	0,718161043	1,982015887	0,194559632	0,795305164	LADA vs T1D
URS00003F2105-lncRNA1	-0,736748447	2,406932528	0,196553513	0,798784334	LADA vs T1D
URS00001BF716-lncRNA1	0,247326707	7,608759782	0,199453176	0,803320509	LADA vs T1D
URS000041043F-antisense1	0,722423401	1,994452875	0,199968195	0,803320509	LADA vs T1D
URS000011812A-antisense1	-0,61933967	4,132007293	0,202880112	0,810361132	LADA vs T1D
URS00006529EE-Y_RNA1	0,745317808	3,147668241	0,20601489	0,811617314	LADA vs T1D
URS00000F6ECB-lncRNA1	0,481478592	5,128846246	0,206145777	0,811617314	LADA vs T1D
URS0000A9525F-antisense1	0,685536908	2,995531648	0,208986971	0,811617314	LADA vs T1D
URS0000A9F786-lncRNA1	-0,846858654	3,099717163	0,210017743	0,811617314	LADA vs T1D
URS00006C6D0A-tRNA1	-0,733462856	3,831692982	0,210551634	0,811617314	LADA vs T1D
URS0000399BDA-lncRNA1	-0,688085706	5,117165985	0,21058464	0,811617314	LADA vs T1D
URS00005508F6-antisense1	0,703182271	3,129098015	0,211322391	0,811617314	LADA vs T1D
URS000005AEAB-tRNA1	-0,576300878	4,48776437	0,214606946	0,812618664	LADA vs T1D
URS000076B0D1-lncRNA1	0,695990204	3,094918128	0,217342425	0,812618664	LADA vs T1D
URS00002598CF-lncRNA1	0,615362865	4,630900569	0,217920993	0,812618664	LADA vs T1D
URS000090DF6D-rRNA1	0,138313971	11,93047681	0,21943562	0,812618664	LADA vs T1D
URS00006A2BF7-Y_RNA1	-0,723206918	10,1864015	0,221626942	0,812618664	LADA vs T1D
URS00001662B7-lncRNA1	0,798987974	2,511216373	0,22201709	0,812618664	LADA vs T1D
URS000077A114-lncRNA1	-0,205243698	7,694058287	0,222944272	0,812618664	LADA vs T1D
URS00004B48CD-lncRNA1	0,684323514	1,80838911	0,225813627	0,812618664	LADA vs T1D
URS00006C246E-snRNA1	0,777379818	4,016094545	0,228091015	0,812618664	LADA vs T1D
URS000042199A-lncRNA1	0,74965718	2,107510848	0,229333493	0,812618664	LADA vs T1D
URS000061A10B-tRNA1	-0,829624743	2,36803002	0,23044745	0,812618664	LADA vs T1D
URS000006044C-lncRNA1	0,642939671	1,808588154	0,231005775	0,812618664	LADA vs T1D
URS0000A89523-lncRNA1	0,70371428	2,553603804	0,232898298	0,812618664	LADA vs T1D
URS000075D48E-lncRNA1	0,589849015	1,602010302	0,233378062	0,812618664	LADA vs T1D
URS00006C2C4A-Y_RNA1	0,882355341	4,927831545	0,235375339	0,812618664	LADA vs T1D
URS000064B6FC-tRNA1	0,752804553	3,113360192	0,235504548	0,812618664	LADA vs T1D
URS0000AABA8B-lncRNA1	0,30086205	9,325488704	0,235552161	0,812618664	LADA vs T1D
URS0000515855-lncRNA1	0,623661476	2,502117237	0,237362613	0,812618664	LADA vs T1D
URS0000605748-lncRNA1	0,6831096	3,689393404	0,238538727	0,812618664	LADA vs T1D
URS0000166229-lncRNA1	0,609661152	2,097439644	0,240664294	0,812618664	LADA vs T1D
URS000063FB43-tRNA1	-0,225640591	12,66119095	0,241386351	0,812618664	LADA vs T1D
URS00000A3F50-lncRNA1	0,624105253	1,749216608	0,242956993	0,812618664	LADA vs T1D
URS000024E9CC-lncRNA1	-0,699527161	2,021333988	0,24360064	0,812618664	LADA vs T1D
URS0000701607-Y_RNA1	0,649717263	3,386959123	0,244601898	0,812618664	LADA vs T1D
URS000068483A-rRNA1	0,689904247	2,397770416	0,246229314	0,812618664	LADA vs T1D
URS000068089E-Y_RNA1	0,678543478	2,479289353	0,251194269	0,812618664	LADA vs T1D
URS0000782759-lncRNA1	-0,630287452	2,907193443	0,251413509	0,812618664	LADA vs T1D
URS00003870EC-lncRNA1	-0,713382032	2,283625175	0,251546553	0,812618664	LADA vs T1D
URS000018BCE5-antisense1	-0,658755034	2,555076799	0,251560017	0,812618664	LADA vs T1D

URS000096D31D-rRNA1	0,217108797	10,50581429	0,252347301	0,812618664	LADA vs T1D
URS0000759AE0-lncRNA1	0,601873469	1,586466937	0,253591661	0,812618664	LADA vs T1D
URS00003DEE5B-lncRNA1	-0,624390181	3,256575112	0,254564403	0,812618664	LADA vs T1D
URS00006F4E76-Y_RNA1	0,30258574	0,766333934	0,255831019	0,812618664	LADA vs T1D
URS000066C003-Y_RNA]1	-1,323241774	5,557417709	0,258226349	0,812618664	LADA vs T1D
URS0000A85AEE-lncRNA1	0,729167651	3,200285639	0,258437929	0,812618664	LADA vs T1D
URS000063CFC9-Y_RNA1	0,672815495	2,480380401	0,259248658	0,812618664	LADA vs T1D
URS00001E15E2-lncRNA1	0,578835758	2,285605947	0,259373224	0,812618664	LADA vs T1D
URS000033F395-lncRNA1	-0,618489902	1,897833694	0,259408819	0,812618664	LADA vs T1D
URS000075AF5F-antisense1	0,685742289	2,789120304	0,259634856	0,812618664	LADA vs T1D
URS00000DAC34-lncRNA1	0,628297991	2,636836099	0,261651847	0,812618664	LADA vs T1D
URS0000955796-rRNA1	0,129321698	10,7330721	0,26290076	0,812618664	LADA vs T1D
URS00009C6042-lncRNA1	-0,327227175	6,706154397	0,264470329	0,812618664	LADA vs T1D
URS000034E03C-antisense1	0,645343006	2,078398724	0,264884939	0,812618664	LADA vs T1D
URS0000395C70-lncRNA1	-0,794377456	2,694736211	0,265057074	0,812618664	LADA vs T1D
URS0000690F87-snRNA1	0,780882008	3,114315559	0,26564268	0,812618664	LADA vs T1D
URS00001DE9CD-antisense1	0,658675773	3,351892383	0,26720477	0,812618664	LADA vs T1D
URS0000417E86-lncRNA1	0,665211116	2,54225615	0,267404312	0,812618664	LADA vs T1D
URS0000986F6F-rRNA1	0,500470564	5,818355153	0,26743169	0,812618664	LADA vs T1D
URS000038EEDC-lncRNA1	0,784716194	3,360541304	0,268547799	0,812618664	LADA vs T1D
URS00002CDF5D-lncRNA1	0,56632536	2,151438672	0,270553007	0,81515755	LADA vs T1D
URS00002CC2A4-antisense1	-0,613020861	2,224906264	0,283935788	0,849438525	LADA vs T1D
URS00000D8A7A-lncRNA1	0,58167267	2,120449279	0,285295361	0,849438525	LADA vs T1D
URS00006C2A6C-Y_RNA1	0,530256873	10,4018413	0,288096491	0,849438525	LADA vs T1D
URS00005E51DB-lncRNA1	-0,508135274	4,114269415	0,290836087	0,849438525	LADA vs T1D
URS00002FBC9E-lncRNA1	-0,634369637	3,539604284	0,292441408	0,849438525	LADA vs T1D
URS00001B6230-lncRNA1	0,574690434	2,056088304	0,292598912	0,849438525	LADA vs T1D
URS000049BB82-lncRNA1	0,51693405	4,039331254	0,292629074	0,849438525	LADA vs T1D
URS0000418239-snRNA1	0,531193782	2,184896985	0,293110628	0,849438525	LADA vs T1D
URS00003D60C0-lncRNA1	0,573395863	1,735581371	0,293895378	0,849438525	LADA vs T1D
URS0000145C5E-tRNA1	0,657205239	2,892697082	0,295256796	0,849438525	LADA vs T1D
URS0000462D45-lncRNA1	0,540403032	3,263068544	0,295298371	0,849438525	LADA vs T1D
URS00006CE1FB-misc_RNA]1	0,963839967	6,644778412	0,299558108	0,855364334	LADA vs T1D
URS00006C9A71-Y_RNA1	0,528342888	2,255341052	0,303800097	0,855364334	LADA vs T1D
URS000061F57C-tRNA1	-0,505019309	4,675906559	0,30449531	0,855364334	LADA vs T1D
URS00000AED6F-tRNA1	-0,479765158	6,83625311	0,304946885	0,855364334	LADA vs T1D
URS0000417A0F-tRNA1	-0,424489007	7,465949428	0,309646813	0,855364334	LADA vs T1D
URS00003ADE1B-lncRNA1	0,59168782	2,400792438	0,310561347	0,855364334	LADA vs T1D
URS00002064F6-tRNA1	-0,598836217	7,265714406	0,311690659	0,855364334	LADA vs T1D
URS00007D61AD-antisense1	0,742781228	2,769938297	0,31298936	0,855364334	LADA vs T1D
URS00003EAC96-antisense1	0,622542726	2,99991317	0,313169784	0,855364334	LADA vs T1D
URS000051EF4B-lncRNA1	0,554036331	1,97882489	0,31331939	0,855364334	LADA vs T1D
URS0000990012-rRNA1	0,327182752	8,57303772	0,315022081	0,855364334	LADA vs T1D
URS0000005270-rRNA1	-0,904112426	6,166410111	0,315398612	0,855364334	LADA vs T1D
URS00001D0B96-lncRNA1	0,131175918	10,38627035	0,315693173	0,855364334	LADA vs T1D
URS00001AF592-antisense1	-0,625907031	3,230887811	0,316644736	0,855364334	LADA vs T1D
URS000091563C-rRNA1	0,117652884	10,26950568	0,318341112	0,855364334	LADA vs T1D
URS00001EE979-lncRNA1	0,62670924	2,774714775	0,322438179	0,855364334	LADA vs T1D
URS000052A1C9-tRNA1	-0,513538525	4,427113529	0,322500386	0,855364334	LADA vs T1D
URS00005BB5C9-antisense1	-0,521272127	1,956136305	0,323482653	0,855364334	LADA vs T1D
URS0000677B31-tRNA1	-0,408618724	7,303130994	0,324533805	0,855364334	LADA vs T1D
URS00002B0998-lncRNA1	0,552865481	2,419217026	0,325774063	0,855364334	LADA vs T1D
URS000070E3CE-rRNA1	0,515353363	4,081177344	0,326258983	0,855364334	LADA vs T1D
URS000020220C-antisense1	0,546275974	2,749759905	0,326272997	0,855364334	LADA vs T1D
URS0000197DBF-antisense1	0,517356991	3,806884628	0,32944038	0,855364334	LADA vs T1D
URS00000FCDE9-misc_RNA]1	0,721444744	3,011608195	0,329939027	0,855364334	LADA vs T1D
URS00006729E8-Y_RNA1	-0,297749841	0,88430075	0,330119051	0,855364334	LADA vs T1D
URS000058CCC6-antisense1	0,545777385	2,503982133	0,331024546	0,855364334	LADA vs T1D
URS00004AE46A-lncRNA1	0,542738393	3,574152246	0,331475017	0,855364334	LADA vs T1D
URS00009A0DCD-rRNA1	0,424081886	5,942865971	0,33210833	0,855364334	LADA vs T1D
URS00002AD6F3-antisense1	0,468117199	1,553235742	0,332845635	0,855364334	LADA vs T1D
URS00006E23A8-rRNA1	-0,607487793	2,840982885	0,335536113	0,858031611	LADA vs T1D
URS00003D4983-antisense1	-0,544515703	1,851867325	0,336378889	0,858031611	LADA vs T1D
URS000090169F-rRNA1	0,518383332	1,691620139	0,338363043	0,858031611	LADA vs T1D
URS000005F65D-antisense1	0,492164304	4,947813537	0,339919728	0,858031611	LADA vs T1D
URS0000172E58-lncRNA1	0,511245382	3,538272714	0,340021111	0,858031611	LADA vs T1D

URS00004AE57B-lncRNA1	0,548314914	2,181566953	0,342879323	0,862131823	LADA vs T1D
URS000019B78E-tRNA1	-0,776960792	3,458896569	0,348613512	0,870415483	LADA vs T1D
URS00008120D6-lncRNA1	0,50273609	1,844896317	0,348664285	0,870415483	LADA vs T1D
URS00005A57D3-lncRNA1	-0,549389758	2,579526429	0,351949123	0,87313444	LADA vs T1D
URS0000941729-rRNA1	0,454776591	4,187919105	0,353236162	0,87313444	LADA vs T1D
URS0000120E41-tRNA1	0,29553806	6,378757577	0,353500782	0,87313444	LADA vs T1D
URS00003C2ECD-antisense1	0,615177732	2,559377792	0,355374684	0,874672197	LADA vs T1D
URS0000726FAB-rRNA1	0,135439355	13,91320925	0,36084069	0,876891479	LADA vs T1D
URS00006D4DB0-snRNA1	-0,578403966	3,391553507	0,364949298	0,876891479	LADA vs T1D
URS00008119F8-lncRNA1	0,578141091	3,00844528	0,365744469	0,876891479	LADA vs T1D
URS000006D0D7-lncRNA1	0,508006195	2,152173172	0,366330348	0,876891479	LADA vs T1D
URS0000093DF5-lncRNA1	0,503655878	2,157430584	0,36656195	0,876891479	LADA vs T1D
URS0000977FD2-SRP_RNA1	-0,504737082	2,037495207	0,367898069	0,876891479	LADA vs T1D
URS00005B6FC3-lncRNA1	-0,387727745	4,468244677	0,368439107	0,876891479	LADA vs T1D
URS00006FF680-Y_RNA1	0,519034414	2,916835355	0,368530529	0,876891479	LADA vs T1D
URS00002D1F9F-lncRNA1	0,455467584	3,254441607	0,370377474	0,876891479	LADA vs T1D
URS000075BA00-lncRNA1	-1,137147267	7,953608312	0,371785618	0,876891479	LADA vs T1D
URS0000488EA7-lncRNA1	0,538138079	2,312667922	0,372357952	0,876891479	LADA vs T1D
URS000072C165-Y_RNA1	-0,231407703	5,305209475	0,373066921	0,876891479	LADA vs T1D
URS00001A72CE-tRNA1	-0,512289255	4,122188898	0,37426365	0,876891479	LADA vs T1D
URS0000378BB8-lncRNA1	0,442788826	4,18272789	0,374812436	0,876891479	LADA vs T1D
URS00002548DF-lncRNA1	0,466627848	1,501270697	0,3769185	0,876891479	LADA vs T1D
URS0000687FC3-Y_RNA1	0,250664182	4,184219214	0,376959819	0,876891479	LADA vs T1D
URS000094F5D5-rRNA1	-0,402005486	7,000982595	0,377602769	0,876891479	LADA vs T1D
URS00006D4008-tRNA1	0,60598414	2,742010752	0,382610988	0,885579738	LADA vs T1D
URS0000038B1E-antisense1	0,455605331	3,244363175	0,387635928	0,893629773	LADA vs T1D
URS000024383A-lncRNA1	-0,4667219	1,637698963	0,388645852	0,893629773	LADA vs T1D
URS00006ABCCE-tRNA1	-0,639090985	11,64642261	0,39171771	0,894607591	LADA vs T1D
URS0000944F10-rRNA1	0,342704658	5,261748123	0,39428462	0,894607591	LADA vs T1D
URS00004227BE-lncRNA1	-0,494465059	1,875905236	0,395719827	0,894607591	LADA vs T1D
URS00006BFB96-tRNA1	-0,490444149	2,209793928	0,396822608	0,894607591	LADA vs T1D
URS000029E713-antisense1	0,421087344	4,061405427	0,396862407	0,894607591	LADA vs T1D
URS0000098211-lncRNA1	0,518078712	2,630527968	0,398064219	0,894607591	LADA vs T1D
URS000064E10F-tRNA1	0,495321047	3,013775094	0,398515597	0,894607591	LADA vs T1D
URS000007383C-antisense1	0,451534834	2,496432723	0,401297071	0,894607591	LADA vs T1D
URS000075B143-lncRNA1	-0,458172385	2,661955926	0,402184472	0,894607591	LADA vs T1D
URS000030C934-lncRNA1	0,416802208	4,417263546	0,40418085	0,894607591	LADA vs T1D
URS00003F41E9-lncRNA1	0,167902971	8,007323205	0,404215474	0,894607591	LADA vs T1D
URS00005DB87D-tRNA1	0,167042412	7,975358739	0,404429183	0,894607591	LADA vs T1D
URS0000381123-lncRNA1	0,467926807	2,471894131	0,40680073	0,895484354	LADA vs T1D
URS0000181B59-lncRNA1	0,566378166	2,274734234	0,407387732	0,895484354	LADA vs T1D
URS000047AF1F-lncRNA1	-0,724119096	4,480968908	0,40983001	0,898028768	LADA vs T1D
URS0000641C1B-Y_RNA1	-0,256872956	4,028164364	0,412914287	0,899199517	LADA vs T1D
URS00008120F8-lncRNA1	0,470859995	1,863766293	0,412937117	0,899199517	LADA vs T1D
URS00007DFA49-antisense1	0,576659046	2,453720457	0,416580027	0,902040969	LADA vs T1D
URS000075D28F-lncRNA1	-0,454226036	2,277607756	0,418677438	0,902040969	LADA vs T1D
URS0000257C23-lncRNA1	-0,542429483	4,584581553	0,419770853	0,902040969	LADA vs T1D
URS000097CDD0-rRNA1	-0,180316035	6,617743447	0,421205056	0,902040969	LADA vs T1D
URS000094439F-rRNA1	0,09376983	11,62287065	0,42159211	0,902040969	LADA vs T1D
URS0000610FFE-tRNA1	0,442587525	2,353369293	0,433686543	0,902040969	LADA vs T1D
URS000045E276-antisense1	0,419442666	7,301870906	0,436064004	0,902040969	LADA vs T1D
URS00006A3E7F-snRNA1	-0,148924849	8,935478024	0,436084797	0,902040969	LADA vs T1D
URS00002901EC-tRNA1	0,456259901	2,043121881	0,436452941	0,902040969	LADA vs T1D
URS000069B369-Y_RNA1	0,536590989	9,690811268	0,439224583	0,902040969	LADA vs T1D
URS0000605144-antisense1	0,436732102	2,730860041	0,442443264	0,902040969	LADA vs T1D
URS00005B7465-tRNA1	0,435341498	1,847741502	0,442529257	0,902040969	LADA vs T1D
URS00000D6053-lncRNA1	-0,634286588	5,001351593	0,445726976	0,902040969	LADA vs T1D
URS0000639DBE-tRNA1	-0,25065807	5,433019134	0,448165586	0,902040969	LADA vs T1D
URS00002A28BF-lncRNA1	0,493907286	2,823053274	0,448524466	0,902040969	LADA vs T1D
URS00000734D4-lncRNA1	-0,366255362	2,320562453	0,448950385	0,902040969	LADA vs T1D
URS00006271F5-Y_RNA1	-0,33776144	3,66489103	0,451649349	0,902040969	LADA vs T1D
URS00009A050E-rRNA1	0,131485199	10,767947	0,452216087	0,902040969	LADA vs T1D
URS0000ABD7E9-lncRNA1	0,447366194	3,512762788	0,453449636	0,902040969	LADA vs T1D
URS000015D954-antisense1	0,421516804	1,950160466	0,453869646	0,902040969	LADA vs T1D
URS00007E2F7C-lncRNA1	0,459516348	3,279760012	0,454086558	0,902040969	LADA vs T1D
URS0000780E15-lncRNA1	0,470864334	2,764657639	0,454242897	0,902040969	LADA vs T1D

URS00005FFC78-lncRNA1	0,428004353	2,519542247	0,455335038	0,902040969	LADA vs T1D
URS00006BF71F-rRNA1	-0,428174281	2,492314255	0,455666896	0,902040969	LADA vs T1D
URS00001A4293-lncRNA1	-0,367148742	4,616590583	0,458371773	0,902040969	LADA vs T1D
URS0000341866-lncRNA1	0,440590581	4,848112839	0,458550268	0,902040969	LADA vs T1D
URS00004D7012-lncRNA1	0,53066176	2,906892949	0,458575601	0,902040969	LADA vs T1D
URS00006CE1FB-rRNA1	0,732684491	5,102179845	0,46064809	0,902040969	LADA vs T1D
URS00002172B5-lncRNA1	0,515893235	2,390444931	0,462101211	0,902040969	LADA vs T1D
URS0000ABD82A-rRNA1	1,098530395	6,247493519	0,462505468	0,902040969	LADA vs T1D
URS00006C8EDF-tRNA1	0,445219962	3,590220694	0,464625787	0,902040969	LADA vs T1D
URS0000A827F4-antisense1	0,470253387	8,224586053	0,464871683	0,902040969	LADA vs T1D
URS0000759CF4-lncRNA1	0,410189742	1,845197414	0,465319447	0,902040969	LADA vs T1D
URS00004DA951-rRNA1	-0,236172356	10,73867185	0,465725892	0,902040969	LADA vs T1D
URS00002C2371-lncRNA1	-0,397404511	3,691720987	0,466852223	0,902040969	LADA vs T1D
URS0000166FF8-lncRNA1	0,35029787	4,546451597	0,468373016	0,902040969	LADA vs T1D
URS00006BBD5F-Y_RNA1	0,415435373	3,385968133	0,468414583	0,902040969	LADA vs T1D
URS00002840A7-lncRNA1	-0,415450489	2,142345929	0,469484318	0,902040969	LADA vs T1D
URS00000B6370-lncRNA1	-0,323741319	3,6717613	0,471453947	0,902040969	LADA vs T1D
URS0000189042-lncRNA1	0,432222255	1,901016294	0,471715302	0,902040969	LADA vs T1D
URS00000CCACD-antisense1	0,512787393	4,642488599	0,471984542	0,902040969	LADA vs T1D
URS000034E9D0-misc_RNA]1	-0,598516154	3,097582218	0,475637429	0,902040969	LADA vs T1D
URS0000AAD5AA-lncRNA1	-0,575249098	10,83600588	0,475802146	0,902040969	LADA vs T1D
URS00003CBFB8-lncRNA1	0,383185112	2,075423342	0,475868261	0,902040969	LADA vs T1D
URS000047C79B-tRNA1	0,117664541	8,413478971	0,475983473	0,902040969	LADA vs T1D
URS0000A88906-antisense1	0,414230952	1,925012855	0,47786382	0,902040969	LADA vs T1D
URS000091B709-rRNA1	0,119693684	9,600448226	0,480062208	0,902040969	LADA vs T1D
URS00004AFADD-lncRNA1	0,401063249	1,695028896	0,481265812	0,902040969	LADA vs T1D
URS00009612D1-rRNA1	0,303098838	5,646389432	0,481590627	0,902040969	LADA vs T1D
URS000022CCD7-lncRNA1	0,193266141	6,400235397	0,481837377	0,902040969	LADA vs T1D
URS0000097924-lncRNA1	0,238748849	5,60446115	0,485576976	0,902040969	LADA vs T1D
URS00002F2DED-lncRNA1	-0,370222761	4,602442219	0,487981014	0,902040969	LADA vs T1D
URS0000161EE9-lncRNA1	0,272465667	6,078069715	0,488825523	0,902040969	LADA vs T1D
URS00008FEDF1-rRNA1	0,421572006	2,23767686	0,488969853	0,902040969	LADA vs T1D
URS00001E9163-lncRNA1	0,389127723	2,764922404	0,489995914	0,902040969	LADA vs T1D
URS0000689904-Y_RNA1	0,218482536	0,877747286	0,490483977	0,902040969	LADA vs T1D
URS0000699390-Y_RNA1	0,32673906	13,62373063	0,491373345	0,902040969	LADA vs T1D
URS0000996BBC-rRNA1	0,127016386	8,622403976	0,492356578	0,902040969	LADA vs T1D
URS00002C4609-lncRNA1	-0,402668429	2,010894744	0,493620411	0,902040969	LADA vs T1D
URS00006144FC-lncRNA1	-0,280914775	7,117689109	0,496074081	0,902040969	LADA vs T1D
URS0000005EDF-lncRNA1	-0,353826523	4,116716413	0,496814954	0,902040969	LADA vs T1D
URS00007BC71B-lncRNA1	-0,401807364	1,944342872	0,499268162	0,902040969	LADA vs T1D
URS0000918BC5-rRNA1	-0,597915169	3,816571784	0,499670804	0,902040969	LADA vs T1D
URS00006D56C0-Y_RNA1	-0,267563262	4,210228585	0,500820363	0,902040969	LADA vs T1D
URS0000928682-rRNA1	0,086310399	11,56053599	0,501625663	0,902040969	LADA vs T1D
URS0000907244-rRNA1	0,164786448	6,675718226	0,503308857	0,902040969	LADA vs T1D
URS00000C18F2-tRNA1	0,293214782	6,19082938	0,503404578	0,902040969	LADA vs T1D
URS00006FCBA3-Y_RNA1	-0,372640771	2,601539284	0,504022662	0,902040969	LADA vs T1D
URS0000A81496-Y_RNA1	0,438163336	2,190786251	0,504736018	0,902040969	LADA vs T1D
URS000003870B-lncRNA1	0,139503857	9,957227048	0,506477713	0,902040969	LADA vs T1D
URS00001DFAE4-lncRNA1	-0,463449938	3,289114858	0,506547749	0,902040969	LADA vs T1D
URS0000A8428E-Y_RNA1	-0,524881077	5,101971983	0,507156081	0,902040969	LADA vs T1D
URS000075AC32-lncRNA1	0,392735877	2,916319528	0,511880304	0,907354464	LADA vs T1D
URS000023F3B4-lncRNA1	0,310229979	3,955198744	0,512739647	0,907354464	LADA vs T1D
URS0000ABD8C6-rRNA1	0,789585991	7,712316992	0,517550846	0,912579758	LADA vs T1D
URS0000762146-antisense1	0,239851794	5,166011847	0,518838698	0,912579758	LADA vs T1D
URS000047EBB5-tRNA1	-0,413041059	2,438958701	0,520405816	0,912579758	LADA vs T1D
URS00005AAAF0-antisense1	0,360125353	3,447551336	0,520914626	0,912579758	LADA vs T1D
URS00006772C0-tRNA1	0,391931196	2,278759881	0,523851496	0,913796589	LADA vs T1D
URS00004C82E1-antisense1	-0,392393243	4,084336348	0,524223794	0,913796589	LADA vs T1D
URS000022006F-lncRNA1	-0,34457493	3,198783691	0,528750629	0,917244614	LADA vs T1D
URS00006952C9-Y_RNA1	-0,44561387	4,013121466	0,52980584	0,917244614	LADA vs T1D
URS00006F0FC0-tRNA1	0,244624562	5,435006769	0,530138518	0,917244614	LADA vs T1D
URS000075A564-lncRNA1	0,353892223	2,096352205	0,534736122	0,922914936	LADA vs T1D
URS000022DD4A-tRNA1	-0,141844781	9,64520168	0,538698294	0,927463319	LADA vs T1D
URS00002EA13A-lncRNA1	-0,339066444	2,908290455	0,541452303	0,929914398	LADA vs T1D
URS00006F5B12-Y_RNA1	-0,362896593	5,757189507	0,545353368	0,934318638	LADA vs T1D
URS00003008EB-lncRNA1	0,288428018	4,342465078	0,549267262	0,938723268	LADA vs T1D

URS000065D78F-Y_RNA1	-0,103560408	5,875001903	0,552510011	0,939156918	LADA vs T1D
URS00001FBD75-misc_RNA]1	-0,508919698	3,307722647	0,55410685	0,939156918	LADA vs T1D
URS00007CA557-lncRNA1	0,141190005	9,085280894	0,554720691	0,939156918	LADA vs T1D
URS00009A7848-rRNA1	-0,074151527	11,54132926	0,554895289	0,939156918	LADA vs T1D
URS00009AAC46-rRNA1	-0,106167486	9,797442669	0,559954021	0,945197778	LADA vs T1D
URS00000AD390-antisense1	0,319699938	1,744059197	0,561168924	0,945197778	LADA vs T1D
URS00006A9AE8-Y_RNA1	0,420737594	5,946221857	0,563036809	0,946064254	LADA vs T1D
URS00006CC125-rRNA1	-0,324433334	2,366732734	0,56675183	0,946777661	LADA vs T1D
URS0000576D5D-lncRNA1	-0,313098703	1,73318224	0,567066499	0,946777661	LADA vs T1D
URS000062C73B-tRNA1	-0,249312394	4,544224584	0,568824946	0,946777661	LADA vs T1D
URS00002811B6-lncRNA1	-0,294769089	3,62513136	0,570877603	0,946777661	LADA vs T1D
URS0000A7BA37-lncRNA1	0,301371466	1,699099075	0,57483038	0,946777661	LADA vs T1D
URS000098FA76-rRNA1	-0,166880435	2,496348799	0,575825449	0,946777661	LADA vs T1D
URS00005BE013-lncRNA1	-0,410602854	2,759573682	0,57638252	0,946777661	LADA vs T1D
URS0000502C74-tRNA1	-0,098040706	14,17701257	0,580686517	0,946777661	LADA vs T1D
URS0000287398-tRNA1	0,349942921	2,440463804	0,585110526	0,946777661	LADA vs T1D
URS00004D9E92-tRNA1	0,147875095	7,483097251	0,585937374	0,946777661	LADA vs T1D
URS00006B0E5A-precursor_RNA1	-0,216622694	6,734564531	0,592439107	0,946777661	LADA vs T1D
URS00006CFDFE-Y_RNA1	0,225131627	2,249644641	0,595238148	0,946777661	LADA vs T1D
URS0000493225-tRNA1	0,149848453	7,026724216	0,595848115	0,946777661	LADA vs T1D
URS00002968BD-antisense1	-0,172360647	7,356778365	0,59623611	0,946777661	LADA vs T1D
URS000012C80D-lncRNA1	-0,290843377	2,726619231	0,599465637	0,946777661	LADA vs T1D
URS0000391A04-lncRNA1	0,222189236	5,482878094	0,600473483	0,946777661	LADA vs T1D
URS00000E9A71-lncRNA1	0,282876496	3,823249127	0,603810657	0,946777661	LADA vs T1D
URS0000812136-lncRNA1	0,114504985	9,121672555	0,604015318	0,946777661	LADA vs T1D
URS000009AC8B-tRNA1	0,352327592	10,95540037	0,604166651	0,946777661	LADA vs T1D
URS0000A774C0-lncRNA1	-0,153479668	6,402978234	0,604504933	0,946777661	LADA vs T1D
URS00000A586F-lncRNA1	0,164434388	0,916847513	0,604566163	0,946777661	LADA vs T1D
URS000000199E-lncRNA1	0,20922721	6,442848869	0,605964358	0,946777661	LADA vs T1D
URS00004AAD0A-lncRNA1	-0,28422571	2,996600067	0,606323061	0,946777661	LADA vs T1D
URS000065DC2A-tRNA1	-0,215112951	4,471494404	0,606971109	0,946777661	LADA vs T1D
URS0000543A1A-lncRNA1	-0,26500995	5,169008445	0,607285502	0,946777661	LADA vs T1D
URS00001C6042-antisense1	0,292203941	1,983328915	0,608242785	0,946777661	LADA vs T1D
URS00001B2779-lncRNA1	0,294916906	1,848580988	0,608384768	0,946777661	LADA vs T1D
URS00006F7C66-rRNA1	-0,288577524	1,804743197	0,608781983	0,946777661	LADA vs T1D
URS00005C51E2-lncRNA1	0,267985478	1,604886726	0,614066604	0,946777661	LADA vs T1D
URS00008BA2B6-lncRNA1	0,265629697	2,826253026	0,614409342	0,946777661	LADA vs T1D
URS0000594305-lncRNA1	0,271193625	1,940137107	0,615763491	0,946777661	LADA vs T1D
URS000099BDEE-rRNA1	0,289635814	5,090607985	0,615860324	0,946777661	LADA vs T1D
URS00006C0715-rRNA1	-0,072222063	11,18891955	0,616160529	0,946777661	LADA vs T1D
URS000069466F-Y_RNA1	0,142741064	7,597826288	0,616499415	0,946777661	LADA vs T1D
URS0000112A1A-antisense1	0,265128724	4,257411498	0,617498898	0,946777661	LADA vs T1D
URS000051CFB0-lncRNA1	-0,458300684	3,801776289	0,618880584	0,946777661	LADA vs T1D
URS00002034DC-tRNA1	-0,278574006	5,180504267	0,620762326	0,946777661	LADA vs T1D
URS000064FE59-rRNA1	-0,067808367	11,09750364	0,62142454	0,946777661	LADA vs T1D
URS000017CF23-antisense1	0,270289598	2,666340903	0,621984156	0,946777661	LADA vs T1D
URS000009738A-lncRNA1	0,294090796	2,916436163	0,622187513	0,946777661	LADA vs T1D
URS00001AA18A-lncRNA1	0,265107196	1,870939952	0,623187217	0,946777661	LADA vs T1D
URS000095D156-rRNA1	0,202567919	6,045790564	0,624509446	0,946777661	LADA vs T1D
URS000068F7ED-Y_RNA1	0,245551516	6,623874775	0,626059142	0,946777661	LADA vs T1D
URS0000031963-antisense1	-0,340092823	3,414756941	0,626930908	0,946777661	LADA vs T1D
URS0000635088-tRNA1	0,2354873	10,57667068	0,627991387	0,946777661	LADA vs T1D
URS0000383A48-tRNA1	0,280892441	3,229014707	0,628254367	0,946777661	LADA vs T1D
URS0000637E4A-tRNA1	0,263387207	1,552702938	0,629024415	0,946777661	LADA vs T1D
URS000018267A-antisense1	0,313693549	3,83534964	0,629466075	0,946777661	LADA vs T1D
URS00005BB09E-lncRNA1	0,189756011	6,047129544	0,629830633	0,946777661	LADA vs T1D
URS000041FE38-lncRNA1	0,26082125	2,353684167	0,632561481	0,947335362	LADA vs T1D
URS0000063647-antisense1	0,145061619	6,169603323	0,63291218	0,947335362	LADA vs T1D
URS000063A7A5-Y_RNA1	0,141395517	8,2571435	0,637000235	0,951417017	LADA vs T1D
URS0000626233-Y_RNA1	-0,18148674	4,209755669	0,641437897	0,956002325	LADA vs T1D
URS000002FD7D-lncRNA1	0,246599148	1,677824648	0,643074057	0,956335777	LADA vs T1D
URS00001D0305-lncRNA1	0,082109768	7,373693454	0,649222332	0,956335777	LADA vs T1D
URS00000AF0EF-lncRNA1	-0,25457045	3,804446661	0,649652543	0,956335777	LADA vs T1D
URS000071DF37-tRNA1	-0,296485863	2,691836943	0,65011687	0,956335777	LADA vs T1D
URS00009C6137-lncRNA1	0,2328337	3,379050945	0,650433705	0,956335777	LADA vs T1D
URS000032B6B6-snRNA1	-0,09598779	8,295722512	0,650769034	0,956335777	LADA vs T1D

URS00004BE455-lncRNA1	-0,190028572	5,228003516	0,651254018	0,956335777	LADA vs T1D
URS0000727FD6-tRNA1	-0,270715861	3,320399515	0,653539326	0,956335777	LADA vs T1D
URS00006D74B2-tRNA1	-0,208192626	4,411009625	0,653974966	0,956335777	LADA vs T1D
URS00002D2D8F-misc_RNA]1	-0,279555803	1,939760416	0,656142137	0,957501782	LADA vs T1D
URS0000A7AB58-Y_RNA1	0,234539821	4,159115672	0,661195917	0,961387712	LADA vs T1D
URS000096EEE8-rRNA1	-0,056470157	8,595030803	0,664268332	0,961387712	LADA vs T1D
URS00006005A4-lncRNA1	-0,297415503	2,906455771	0,667526579	0,961387712	LADA vs T1D
URS00006B479B-tRNA1	-0,196489364	1,462161631	0,667736143	0,961387712	LADA vs T1D
URS000044BAE3-tRNA1	-0,09048198	7,695683129	0,667751301	0,961387712	LADA vs T1D
URS0000701637-rRNA1	-0,259312412	2,670549892	0,66940678	0,961387712	LADA vs T1D
URS00003875B8-lncRNA1	-0,263601719	2,156965412	0,67256133	0,961387712	LADA vs T1D
URS0000653BD1-Y_RNA1	0,215491207	4,226797259	0,675296601	0,961387712	LADA vs T1D
URS00005B30A9-tRNA1	-0,273783928	2,750819629	0,676914293	0,961387712	LADA vs T1D
URS00001AD596-tRNA1	-0,068103531	11,15468011	0,678374493	0,961387712	LADA vs T1D
URS00004E5B9A-lncRNA1	-0,22261149	1,8276158	0,679872845	0,961387712	LADA vs T1D
URS00001F3EF6-lncRNA1	0,278970333	2,384806636	0,682726617	0,961387712	LADA vs T1D
URS000020AD62-lncRNA1	-0,217932881	1,816448524	0,683095602	0,961387712	LADA vs T1D
URS000098604A-rRNA1	0,230162436	1,872688165	0,683217017	0,961387712	LADA vs T1D
URS00008B26D4-antisense1	0,216001558	4,369068967	0,68338075	0,961387712	LADA vs T1D
URS000091CD45-rRNA1	-0,217216336	6,581914152	0,683822037	0,961387712	LADA vs T1D
URS00006642D4-Y_RNA1	-0,078244424	4,428261145	0,684105509	0,961387712	LADA vs T1D
URS00004CE099-lncRNA1	0,334677285	3,298450018	0,684961398	0,961387712	LADA vs T1D
URS000052A6A6-lncRNA1	-0,215663403	3,111397823	0,687768932	0,961387712	LADA vs T1D
URS00009843EB-rRNA1	-0,073029229	12,28502234	0,689999579	0,961387712	LADA vs T1D
URS000063E4FD-tRNA1	0,242960688	3,061724682	0,692681773	0,961387712	LADA vs T1D
URS00005C7D80-antisense1	-0,230872699	2,279201521	0,694274651	0,961387712	LADA vs T1D
URS000015BB33-lncRNA1	-0,194400577	3,745978664	0,698212302	0,961387712	LADA vs T1D
URS00000347CF-lncRNA1	0,188278908	3,718528756	0,699454134	0,961387712	LADA vs T1D
URS0000192C05-lncRNA1	-0,208974077	2,748839188	0,699909312	0,961387712	LADA vs T1D
URS00004DDD71-lncRNA1	-0,138373823	5,854746665	0,700034043	0,961387712	LADA vs T1D
URS00003CAB47-antisense1	-0,222190844	3,257126248	0,702311675	0,961387712	LADA vs T1D
URS0000016595-lncRNA1	0,225371525	2,839797195	0,705062629	0,961387712	LADA vs T1D
URS00003E3BDF-antisense1	-0,201633021	2,398707663	0,707862294	0,961387712	LADA vs T1D
URS000064D54F-tRNA1	-0,203319133	2,523144202	0,709048097	0,961387712	LADA vs T1D
URS0000511A5F-lncRNA1	0,212615534	1,837603255	0,709303101	0,961387712	LADA vs T1D
URS000092B92B-rRNA1	0,515638688	5,336196426	0,710044549	0,961387712	LADA vs T1D
URS00007080E9-Y_RNA1	0,276229833	2,936384424	0,7106533	0,961387712	LADA vs T1D
URS0000446770-snoRNA1	-0,22232387	2,104005039	0,714938624	0,961387712	LADA vs T1D
URS0000204428-lncRNA1	-0,220999691	2,674128924	0,715049797	0,961387712	LADA vs T1D
URS00003A00A4-lncRNA1	-0,215040567	3,039610494	0,717052058	0,961387712	LADA vs T1D
URS00005F1728-lncRNA1	-0,193031157	2,661157063	0,71743336	0,961387712	LADA vs T1D
URS00007062F7-Y_RNA1	-0,139923022	4,160857713	0,719120053	0,961387712	LADA vs T1D
URS00006F4537-tRNA1	0,218537775	4,037892922	0,719396057	0,961387712	LADA vs T1D
URS00000C7470-antisense1	-0,201077278	3,497403787	0,720114055	0,961387712	LADA vs T1D
URS000076E14E-lncRNA1	-0,242881577	2,947769863	0,721380577	0,961387712	LADA vs T1D
URS0000AA014E-lncRNA1	-0,190142404	2,880951371	0,721535425	0,961387712	LADA vs T1D
URS00004BF687-tRNA1	0,220105482	13,4414794	0,721639797	0,961387712	LADA vs T1D
URS00001142E8-lncRNA1	-0,13054568	5,750896426	0,72216481	0,961387712	LADA vs T1D
URS0000682FB0-Y_RNA1	0,146987032	10,97882695	0,72587828	0,961387712	LADA vs T1D
URS0000222FD2-tRNA1	0,214720361	3,623751413	0,726184719	0,961387712	LADA vs T1D
URS0000086FDD-antisense1	-0,236554542	2,945683418	0,727328938	0,961387712	LADA vs T1D
URS0000265843-antisense1	0,182254946	2,862204159	0,728277253	0,961387712	LADA vs T1D
URS00004F6629-lncRNA1	-0,135281611	1,127896398	0,731251318	0,961387712	LADA vs T1D
URS0000ABD879-rRNA1	-0,410088194	7,136004534	0,731669361	0,961387712	LADA vs T1D
URS000055C9F8-antisense1	0,15506009	4,017918826	0,73400086	0,961387712	LADA vs T1D
URS00003D279B-lncRNA1	0,18264227	3,869764687	0,73833408	0,961387712	LADA vs T1D
URS00008116FE-lncRNA1	0,18503315	2,826119493	0,739602428	0,961387712	LADA vs T1D
URS000033268A-lncRNA1	0,182125067	4,132479222	0,740139532	0,961387712	LADA vs T1D
URS0000676525-Y_RNA1	0,167068379	4,329395557	0,74247608	0,961387712	LADA vs T1D
URS0000089048-antisense1	-0,175012281	1,859342356	0,742636156	0,961387712	LADA vs T1D
URS000071E736-Y_RNA1	-0,201688997	2,593633412	0,742898028	0,961387712	LADA vs T1D
URS00006D23E9-snRNA1	-0,184742325	3,580811892	0,742931599	0,961387712	LADA vs T1D
URS0000649B00-rRNA1	0,047738715	10,09773081	0,742950676	0,961387712	LADA vs T1D
URS0000700D52-tRNA1	-0,192917	2,144408558	0,743459296	0,961387712	LADA vs T1D
URS000075A823-rRNA1	-0,43904648	6,786069906	0,743566616	0,961387712	LADA vs T1D
URS00003AD33F-lncRNA1	-0,202837489	3,336093709	0,744372965	0,961387712	LADA vs T1D

URS00006D484A-rRNA1	0,032039204	11,67221899	0,746688192	0,961387712	LADA vs T1D
URS000037D0FB-tRNA1	-0,218594162	2,792154767	0,74752105	0,961387712	LADA vs T1D
URS00000E43DB-antisense1	-0,171168436	3,522079692	0,748204457	0,961387712	LADA vs T1D
URS0000920597-rRNA1	-0,147422187	8,520273949	0,750037042	0,961580255	LADA vs T1D
URS0000082453-lncRNA1	0,171921186	2,456766093	0,751105607	0,961580255	LADA vs T1D
URS000003B6E2-lncRNA1	0,238737252	2,652355198	0,754919026	0,964126536	LADA vs T1D
URS00006174C2-tRNA1	-0,069584684	8,218629708	0,757636682	0,964126536	LADA vs T1D
URS00000C653A-lncRNA1	0,169805951	2,462989968	0,758191611	0,964126536	LADA vs T1D
URS00005D36AA-lncRNA1	0,181736392	2,523255562	0,758893079	0,964126536	LADA vs T1D
URS0000684921-rRNA1	0,178322371	2,725738393	0,759991017	0,964126536	LADA vs T1D
URS0000AAECF4-lncRNA1	0,140136167	3,702161679	0,762480714	0,964403291	LADA vs T1D
URS0000188F7D-scRNA1	0,453228016	6,577829264	0,763531596	0,964403291	LADA vs T1D
URS000014D40F-tRNA1	0,214818729	3,081622422	0,764348245	0,964403291	LADA vs T1D
URS000063B690-snRNA1	0,170241551	3,324962274	0,769043223	0,964890508	LADA vs T1D
URS0000504A1A-lncRNA1	-0,151233524	1,470603839	0,772230269	0,964890508	LADA vs T1D
URS00005BC8EC-lncRNA1	0,220315699	5,143820106	0,77252863	0,964890508	LADA vs T1D
URS0000209048-tRNA1	0,17372273	3,508601642	0,773495357	0,964890508	LADA vs T1D
URS00006AD70A-tRNA1	-0,180920465	4,843339833	0,773663873	0,964890508	LADA vs T1D
URS00001D081D-antisense1	-0,159008003	1,824716399	0,775450786	0,964890508	LADA vs T1D
URS000050471D-lncRNA1	0,178030016	2,378838415	0,775563104	0,964890508	LADA vs T1D
URS00007C72D0-lncRNA1	-0,139547449	1,699191247	0,77577749	0,964890508	LADA vs T1D
URS000020BB55-lncRNA1	0,180053427	3,131770633	0,779032441	0,96614401	LADA vs T1D
URS00004F0321-tRNA1	-0,08455807	14,08130095	0,781230131	0,96614401	LADA vs T1D
URS00009407E6-rRNA1	-0,16583846	2,030446175	0,78301979	0,96614401	LADA vs T1D
URS00009C60C3-lncRNA1	-0,208499876	3,075209684	0,784371549	0,96614401	LADA vs T1D
URS00006F3305-rRNA1	-0,028441472	11,47052611	0,78783357	0,96614401	LADA vs T1D
URS00007D436A-antisense1	0,146543888	2,239346559	0,789220265	0,96614401	LADA vs T1D
URS0000931B54-rRNA1	0,020797089	14,46696978	0,789559287	0,96614401	LADA vs T1D
URS000097FEDE-rRNA1	0,139976312	3,690975927	0,789903462	0,96614401	LADA vs T1D
URS000034AAC2-tRNA1	0,142711519	4,895139042	0,79185815	0,96614401	LADA vs T1D
URS000014D914-antisense1	0,130043059	4,160373223	0,792753362	0,96614401	LADA vs T1D
URS00000DA554-lncRNA1	0,150339502	2,522581542	0,792967723	0,96614401	LADA vs T1D
URS000096196A-SRP_RNA1	0,142771774	2,043342413	0,795258006	0,96614401	LADA vs T1D
URS00006AE6F1-Y_RNA1	-0,114446165	2,11506442	0,7956417	0,96614401	LADA vs T1D
URS00009840C1-rRNA1	-0,103029029	6,418341272	0,797002261	0,96614401	LADA vs T1D
URS00006AD81D-Y_RNA1	-0,155105288	1,867732365	0,797518017	0,96614401	LADA vs T1D
URS00001E5F12-lncRNA1	0,147694604	2,516521413	0,80074059	0,968369675	LADA vs T1D
URS000013BA50-antisense1	0,15537257	1,971579125	0,803821871	0,968452955	LADA vs T1D
URS00005B6D58-lncRNA1	0,119844067	5,324374408	0,805391682	0,968452955	LADA vs T1D
URS000070B37B-tRNA1	-0,045459662	9,295263071	0,805967027	0,968452955	LADA vs T1D
URS000042F13F-tRNA1	0,067857128	6,573577673	0,806351388	0,968452955	LADA vs T1D
URS000036E063-antisense1	-0,028518423	10,32347827	0,811241671	0,971316043	LADA vs T1D
URS0000397495-antisense1	-0,081425305	5,990008067	0,811607825	0,971316043	LADA vs T1D
URS000062F68C-rRNA1	-0,031283289	10,93938784	0,814565127	0,971316043	LADA vs T1D
URS000048B807-lncRNA1	-0,115906069	4,663045601	0,816255087	0,971316043	LADA vs T1D
URS00007CB156-lncRNA1	0,04762485	7,841069642	0,816390538	0,971316043	LADA vs T1D
URS00006EBF05-misc_RNA]1	-0,210237756	3,622681484	0,819678379	0,971316043	LADA vs T1D
URS000096B970-rRNA1	0,030454653	10,27519744	0,821257466	0,971316043	LADA vs T1D
URS000063455F-rRNA1	-0,125077015	2,601127664	0,822325726	0,971316043	LADA vs T1D
URS0000679FAF-tRNA1	0,101603459	5,101125315	0,826770891	0,971316043	LADA vs T1D
URS00004EDF08-lncRNA1	-0,092358835	4,609717254	0,827855176	0,971316043	LADA vs T1D
URS000064217E-Y_RNA1	0,12890319	2,41296644	0,827888252	0,971316043	LADA vs T1D
URS0000A7F61B-lncRNA1	-0,116414992	2,242573569	0,828382134	0,971316043	LADA vs T1D
URS0000121433-tRNA1	0,163744475	4,021698317	0,828755374	0,971316043	LADA vs T1D
URS00005B2DE5-lncRNA1	0,11265164	4,758632634	0,831258887	0,971316043	LADA vs T1D
URS0000702B40-Y_RNA1	0,13815663	2,572703997	0,83347353	0,971316043	LADA vs T1D
URS0000100BA4-antisense1	0,121036444	1,836907151	0,8348759	0,971316043	LADA vs T1D
URS00006481F8-Y_RNA1	0,063472216	7,303708498	0,836139047	0,971316043	LADA vs T1D
URS00002750C5-lncRNA1	0,109705286	1,848936086	0,837591688	0,971316043	LADA vs T1D
URS00003CA240-antisense1	0,08411206	5,133383215	0,838346395	0,971316043	LADA vs T1D
URS00003D2CC9-tRNA1	0,107656289	4,271817892	0,83841207	0,971316043	LADA vs T1D
URS00004F2E54-lncRNA1	0,116559281	1,792153167	0,838692051	0,971316043	LADA vs T1D
URS000038F4B2-tRNA1	0,121713261	2,156583414	0,842994766	0,971316043	LADA vs T1D
URS0000918AFB-rRNA1	0,048700724	8,610055402	0,843189815	0,971316043	LADA vs T1D
URS00008C3E41-lncRNA1	0,082029776	5,384577661	0,845550282	0,971316043	LADA vs T1D
URS0000432B92-antisense1	-0,035481799	7,647433512	0,846392896	0,971316043	LADA vs T1D

URS00001B506A-tRNA1	-0,127138625	2,259860507	0,847877749	0,971316043	LADA vs T1D
URS000019B78E-misc_RNA]1	-0,157984063	3,588733066	0,848673424	0,971316043	LADA vs T1D
URS00007BE6D3-lncRNA1	0,265594819	10,22836282	0,849234237	0,971316043	LADA vs T1D
URS0000ABD87F-rRNA1	0,186247849	10,81861494	0,84994658	0,971316043	LADA vs T1D
URS00001AE429-lncRNA1	0,115714018	3,26760494	0,851427398	0,971316043	LADA vs T1D
URS000055B99E-lncRNA1	0,117868503	2,716513459	0,852208777	0,971316043	LADA vs T1D
URS000053D4AB-snoRNA1	0,100149617	1,689585927	0,853900523	0,971316043	LADA vs T1D
URS00004AC036-lncRNA1	-0,090849537	4,515610764	0,855456683	0,971316043	LADA vs T1D
URS000065A213-Y_RNA1	-0,084918431	10,7844197	0,858853501	0,971316043	LADA vs T1D
URS0000629ECF-Y_RNA1	-0,065369028	4,37154222	0,859114389	0,971316043	LADA vs T1D
URS000056B231-lncRNA1	-0,10476292	4,1586305	0,85988446	0,971316043	LADA vs T1D
URS000023352F-lncRNA1	0,090926433	2,158198536	0,860573485	0,971316043	LADA vs T1D
URS0000994031-rRNA1	0,025944966	11,10957664	0,862298875	0,971316043	LADA vs T1D
URS00002C130C-tRNA1	0,153478261	3,549564243	0,862928845	0,971316043	LADA vs T1D
URS000017D264-lncRNA1	-0,09427934	2,092743225	0,869404546	0,977031797	LADA vs T1D
URS000045FD15-lncRNA1	-0,081476475	4,004446293	0,880387186	0,986797512	LADA vs T1D
URS000050FA69-lncRNA1	0,078375855	3,930388911	0,881020586	0,986797512	LADA vs T1D
URS00002CFBEA-lncRNA1	0,07151125	2,003500281	0,887396045	0,986797512	LADA vs T1D
URS00003F2CFE-lncRNA1	0,077286077	4,520438351	0,889981517	0,986797512	LADA vs T1D
URS00000CD33E-antisense1	0,071691019	3,630533758	0,891896788	0,986797512	LADA vs T1D
URS00008116E3-lncRNA1	-0,067277109	4,000279228	0,893939214	0,986797512	LADA vs T1D
URS000002E930-antisense1	-0,089650268	4,041839683	0,893943116	0,986797512	LADA vs T1D
URS000000898B-lncRNA1	0,081960451	2,509136511	0,894696085	0,986797512	LADA vs T1D
URS0000228E94-lncRNA1	0,07618379	2,335958891	0,896327939	0,986797512	LADA vs T1D
URS00004106BA-lncRNA1	-0,062100992	1,798128499	0,898016304	0,986797512	LADA vs T1D
URS0000635FFC-Y_RNA1	0,073325602	2,322009496	0,898046452	0,986797512	LADA vs T1D
URS0000717DFB-Y_RNA1	0,066340886	2,95584216	0,898520663	0,986797512	LADA vs T1D
URS0000543B4D-lncRNA1	-0,076388704	2,605909076	0,899018301	0,986797512	LADA vs T1D
URS00006F4F8C-snoRNA1	0,027718732	8,039702247	0,899147271	0,986797512	LADA vs T1D
URS000013899F-tRNA1	-0,060958621	4,501396588	0,900657828	0,986797512	LADA vs T1D
URS00006CBBB9-Y_RNA1	0,071383587	3,554703445	0,90158508	0,986797512	LADA vs T1D
URS00006E19EA-Y_RNA1	-0,047292189	9,597384946	0,903336324	0,986797512	LADA vs T1D
URS00002A865C-lncRNA1	-0,060955027	1,751381992	0,909094007	0,986797512	LADA vs T1D
URS0000633321-snoRNA1	0,028481302	7,948494424	0,90934457	0,986797512	LADA vs T1D
URS00005AF005-lncRNA1	-0,06248909	3,522774737	0,909355919	0,986797512	LADA vs T1D
URS0000669D0F-snRNA1	-0,06793773	2,759909955	0,910622819	0,986797512	LADA vs T1D
URS00006428FD-Y_RNA1	-0,04674489	6,936586919	0,914778786	0,986797512	LADA vs T1D
URS0000038397-lncRNA1	-0,058068019	5,458381823	0,914865019	0,986797512	LADA vs T1D
URS000019F398-lncRNA1	0,053565611	1,659002941	0,915448543	0,986797512	LADA vs T1D
URS000067474D-tRNA1	-0,06595064	5,826738993	0,915550291	0,986797512	LADA vs T1D
URS000076FC5A-lncRNA1	-0,065614402	2,343917503	0,915927692	0,986797512	LADA vs T1D
URS00001D4EE9-tRNA1	0,070115289	2,839543714	0,916211138	0,986797512	LADA vs T1D
URS00001823EB-lncRNA1	-0,060378618	3,483455808	0,921319133	0,990772422	LADA vs T1D
URS000001AE93-lncRNA1	0,021846423	6,898854125	0,924952557	0,992131259	LADA vs T1D
URS00005AF1AA-antisense1	0,03883517	1,232925367	0,927801488	0,992131259	LADA vs T1D
URS00006CB0C3-Y_RNA1	0,020445976	5,291889769	0,931733183	0,992131259	LADA vs T1D
URS00007116F9-Y_RNA1	0,045245365	3,042319437	0,93348894	0,992131259	LADA vs T1D
URS0000593A4A-lncRNA1	-0,049975703	3,380617038	0,934186516	0,992131259	LADA vs T1D
URS000095C3C7-rRNA1	-0,037407808	4,667191645	0,934859797	0,992131259	LADA vs T1D
URS00006D9244-tRNA1	0,050557254	3,430512932	0,935886578	0,992131259	LADA vs T1D
URS00001B59BD-lncRNA1	-0,020278407	6,767957763	0,938044575	0,992131259	LADA vs T1D
URS0000271FCA-antisense1	0,034789731	5,084020409	0,93898576	0,992131259	LADA vs T1D
URS00003869EC-lncRNA1	-0,05148261	2,98234075	0,9390056	0,992131259	LADA vs T1D
URS000047A7F4-rRNA1	-0,071648988	4,287976289	0,940060847	0,992131259	LADA vs T1D
URS000009DDCA-tRNA1	-0,019928334	8,707527281	0,940220446	0,992131259	LADA vs T1D
URS000059900F-tRNA1	-0,041588202	3,409718688	0,941203609	0,992131259	LADA vs T1D
URS000075D341-rRNA1	0,064241487	3,481242239	0,94438273	0,992131259	LADA vs T1D
URS00006361F3-Y_RNA1	0,052212894	4,856921385	0,944672562	0,992131259	LADA vs T1D
URS0000784C7B-lncRNA1	-0,03779376	2,086288772	0,946832518	0,992131259	LADA vs T1D
URS00006BB04D-tRNA1	0,037347651	3,054494403	0,9470173	0,992131259	LADA vs T1D
URS0000A765F3-lncRNA1	0,033040903	3,81544174	0,949302049	0,992131259	LADA vs T1D
URS0000177135-lncRNA1	0,050649201	3,040442935	0,951085429	0,992131259	LADA vs T1D
URS000013BB40-lncRNA1	0,034725448	2,666260296	0,95190046	0,992131259	LADA vs T1D
URS00000081EA-snRNA1	0,033723266	2,66468092	0,953401208	0,992131259	LADA vs T1D
URS0000042FD3-lncRNA1	0,028750969	3,024717754	0,954884654	0,992131259	LADA vs T1D
URS000062FB25-rRNA1	-0,007410037	9,742461609	0,95522795	0,992131259	LADA vs T1D

URS000008089F-lncRNA1	-0,034248501	3,494287219	0,957943955	0,993476001	LADA vs T1D
URS0000AAB7F4-lncRNA1	0,024212396	3,962630959	0,965282541	0,995025787	LADA vs T1D
URS0000640661-Y_RNA1	-0,020528582	11,13979054	0,965914096	0,995025787	LADA vs T1D
URS00002B7986-antisense1	-0,021800803	1,782132427	0,966968141	0,995025787	LADA vs T1D
URS00006744D5-tRNA1	-0,024816754	2,786516677	0,969161584	0,995025787	LADA vs T1D
URS000063A6E6-rRNA1	-0,004464275	11,70425189	0,969709201	0,995025787	LADA vs T1D
URS000050E9EC-lncRNA1	0,018517004	3,382080638	0,971344918	0,995025787	LADA vs T1D
URS0000ABD7D5-rRNA1	0,003311513	13,17747017	0,97172631	0,995025787	LADA vs T1D
URS0000696377-Y_RNA1	-0,01859509	2,848796511	0,973189747	0,995025787	LADA vs T1D
URS0000389FBF-lncRNA1	0,012452445	2,547635864	0,982052748	0,995025787	LADA vs T1D
URS0000611F3E-lncRNA1	0,011339926	2,798037489	0,983631417	0,995025787	LADA vs T1D
URS00002AECD7-lncRNA1	-0,012679072	2,148873116	0,984124481	0,995025787	LADA vs T1D
URS0000704D22-rRNA1	-0,003539656	14,1067509	0,984396509	0,995025787	LADA vs T1D
URS00004F482C-lncRNA1	-0,010220178	2,9888845	0,985938473	0,995025787	LADA vs T1D
URS00003A72DC-antisense1	-0,009041388	3,084869652	0,986781353	0,995025787	LADA vs T1D
URS00006CDFA7-rRNA1	-0,008808286	2,666787507	0,986802237	0,995025787	LADA vs T1D
URS000047A7F4-misc_RNA]1	-0,015030499	4,238929678	0,98683592	0,995025787	LADA vs T1D
URS0000590507-lncRNA1	-0,008582703	1,90331514	0,987313083	0,995025787	LADA vs T1D
URS000097171C-SRP_RNA1	0,008518602	2,924722071	0,987642192	0,995025787	LADA vs T1D
URS000028E102-lncRNA1	0,008415811	2,546561157	0,988030896	0,995025787	LADA vs T1D
URS00006C900C-tRNA1	-0,007280291	2,991743424	0,989287279	0,995025787	LADA vs T1D
URS000000513C-antisense1	-0,005258063	4,840741267	0,989331791	0,995025787	LADA vs T1D
URS00005C220A-antisense1	0,002943318	2,894185806	0,996392745	0,999624541	LADA vs T1D
URS000090AA7A-rRNA1	-0,000891715	2,615865166	0,998788614	0,999624541	LADA vs T1D
URS00005580B2-lncRNA1	-0,000545581	3,442360628	0,999344231	0,999624541	LADA vs T1D
URS00005D1950-antisense1	-0,000246355	2,067377939	0,999624541	0,999624541	LADA vs T1D
URS0000644222-tRNA2	-1,08060695	7,91366141	2,19061E-06	0,001531237	LADA vs T2D
URS000064C567-Y_RNA2	-1,032255743	5,523874961	1,92564E-05	0,005093667	LADA vs T2D
URS000064F96B-Y_RNA2	-1,444263075	5,069805695	2,77125E-05	0,005093667	LADA vs T2D
URS00006729E8-Y_RNA2	-1,300630337	0,88430075	2,91483E-05	0,005093667	LADA vs T2D
URS000065DC2A-tRNA2	-1,698156648	4,471494404	6,47039E-05	0,00864061	LADA vs T2D
URS000061F57C-tRNA2	-1,978952603	4,675906559	7,41683E-05	0,00864061	LADA vs T2D
URS000063FB43-tRNA2	-0,717521519	12,66119095	0,000228541	0,022821461	LADA vs T2D
URS0000684E4B-tRNA2	-0,885723141	6,262078663	0,000385399	0,033674196	LADA vs T2D
URS00002D40C8-tRNA2	-0,809824875	7,830242615	0,001025097	0,079615898	LADA vs T2D
URS000013B42D-tRNA2	-1,094433274	16,60649921	0,001415353	0,098933181	LADA vs T2D
URS00003C9A26-tRNA2	-0,567079168	8,822917147	0,002609447	0,165818488	LADA vs T2D
URS0000974435-SRP_RNA2	-1,504113941	2,886817066	0,004061163	0,21190276	LADA vs T2D
URS00006F135B-Y_RNA2	-1,064742107	3,60198893	0,004648945	0,21190276	LADA vs T2D
URS00006D56C0-Y_RNA2	-1,131249694	4,210228585	0,004677927	0,21190276	LADA vs T2D
URS00006BFB96-tRNA2	-1,629612998	2,209793928	0,004942271	0,21190276	LADA vs T2D
URS00006FCBA3-Y_RNA2	-1,569682354	2,601539284	0,005038362	0,21190276	LADA vs T2D
URS00006FC298-tRNA2	-1,75061802	2,919601035	0,005296446	0,21190276	LADA vs T2D
URS0000145C5E-tRNA2	-1,755276323	2,892697082	0,005456723	0,21190276	LADA vs T2D
URS0000639DBE-tRNA2	-0,906406456	5,433019134	0,006357294	0,233881502	LADA vs T2D
URS0000676AED-precursor_RNA2	-1,581643337	2,736959381	0,006926215	0,24207121	LADA vs T2D
URS0000630B8A-tRNA2	-1,257750315	5,357715317	0,007786701	0,25918592	LADA vs T2D
URS0000446770-snoRNA2	-1,607401573	2,104005039	0,008783776	0,277691565	LADA vs T2D
URS000096196A-SRP_RNA2	-1,444525693	2,043342413	0,009137205	0,277691565	LADA vs T2D
URS00000E43DB-antisense2	-1,365239516	3,522079692	0,010880749	0,281558284	LADA vs T2D
URS0000679FAF-tRNA2	-1,187471218	5,101125315	0,010946367	0,281558284	LADA vs T2D
URS000076DAC1-lncRNA2	-1,049654547	5,661423845	0,011356913	0,281558284	LADA vs T2D
URS00006C900C-tRNA2	-1,379969455	2,991743424	0,011369335	0,281558284	LADA vs T2D
URS000071C9A6-Y_RNA2	-0,923157955	2,18048286	0,011480665	0,281558284	LADA vs T2D
URS0000A774C0-lncRNA2	-0,748610432	6,402978234	0,011904274	0,281558284	LADA vs T2D
URS000035D229-lncRNA2	-2,003933648	3,593153184	0,012198998	0,281558284	LADA vs T2D
URS00008C3E41-lncRNA2	1,060464054	5,384577661	0,012486848	0,281558284	LADA vs T2D
URS000038D8D3-tRNA2	-0,756329596	7,544518208	0,014973197	0,327070771	LADA vs T2D
URS00006B33E0-Y_RNA2	-0,988518604	14,2625662	0,015739122	0,327425816	LADA vs T2D
URS000067843B-Y_RNA2	-0,955674382	5,925250166	0,015926291	0,327425816	LADA vs T2D
URS0000A7F61B-lncRNA2	-1,269853381	2,242573569	0,018341779	0,350375395	LADA vs T2D
URS00006529EE-Y_RNA2	-1,39501234	3,147668241	0,018838122	0,350375395	LADA vs T2D
URS0000192C05-lncRNA2	-1,272769558	2,748839188	0,019045268	0,350375395	LADA vs T2D
URS00006CE1FB-misc_RNA]2	2,176589998	6,644778412	0,019207402	0,350375395	LADA vs T2D
URS0000A90D33-snRNA2	1,473927191	3,877391031	0,019551401	0,350375395	LADA vs T2D
URS00000FB60D-tRNA2	-1,343784793	3,59514453	0,020050094	0,350375395	LADA vs T2D

URS00006D74B2-tRNA2	-1,081358136	4,411009625	0,020720067	0,353251875	LADA vs T2D
URS000022006F-lncRNA2	-1,260640412	3,198783691	0,021432463	0,356697415	LADA vs T2D
URS00006BF71F-rRNA2	-1,316962528	2,492314255	0,022151759	0,360094875	LADA vs T2D
URS0000726FAB-rRNA2	0,335205789	13,91320925	0,023953531	0,380534498	LADA vs T2D
URS00006B0E5A-precursor_RNA2	-0,899303309	6,734564531	0,026522028	0,411975502	LADA vs T2D
URS000042F13F-tRNA2	-0,601226703	6,573577673	0,030163836	0,441849317	LADA vs T2D
URS0000A7AB58-Y_RNA2	-1,166274225	4,159115672	0,030209799	0,441849317	LADA vs T2D
URS00009554B1-rRNA2	-1,119967124	4,916492139	0,030341584	0,441849317	LADA vs T2D
URS00006F4F8C-snoRNA2	-0,471396458	8,039702247	0,031588288	0,445931146	LADA vs T2D
URS000005AEAB-tRNA2	-0,992715636	4,48776437	0,032719268	0,445931146	LADA vs T2D
URS00000D6053-lncRNA2	-1,779319531	5,001351593	0,032731185	0,445931146	LADA vs T2D
URS00001BF716-lncRNA2	0,408516117	7,608759782	0,033985173	0,445931146	LADA vs T2D
URS000072C165-Y_RNA2	-0,550580919	5,305209475	0,034288644	0,445931146	LADA vs T2D
URS0000222FD2-tRNA2	-1,301159694	3,623751413	0,034449616	0,445931146	LADA vs T2D
URS00002034DC-tRNA2	-1,183541079	5,180504267	0,03598028	0,457276646	LADA vs T2D
URS00002750C5-lncRNA2	-1,115227966	1,848936086	0,037533383	0,468497055	LADA vs T2D
URS00002A865C-lncRNA2	-1,10625526	1,751381992	0,038595581	0,473303704	LADA vs T2D
URS000063A7A5-Y_RNA2	-0,616905326	8,2571435	0,039680418	0,474409979	LADA vs T2D
URS0000AA0C30-lncRNA2	-1,128197132	3,233082441	0,041028866	0,474409979	LADA vs T2D
URS000029E713-antisense2	1,017121422	4,061405427	0,041190454	0,474409979	LADA vs T2D
URS00004BF687-tRNA2	-1,256380007	13,4414794	0,04180771	0,474409979	LADA vs T2D
URS0000AA014E-lncRNA2	-1,083579447	2,880951371	0,043037295	0,474409979	LADA vs T2D
URS000059900F-tRNA2	-1,138961597	3,409718688	0,044011962	0,474409979	LADA vs T2D
URS0000684921-rRNA2	-1,172395982	2,725738393	0,045313145	0,474409979	LADA vs T2D
URS00006D23E9-snRNA2	-1,132546775	3,580811892	0,045313961	0,474409979	LADA vs T2D
URS00006AE6F1-Y_RNA2	-0,883576352	2,11506442	0,04654609	0,474409979	LADA vs T2D
URS0000A77003-lncRNA2	-1,139128427	2,821180105	0,046785983	0,474409979	LADA vs T2D
URS00006A3E7F-snRNA2	-0,380233979	8,935478024	0,04682264	0,474409979	LADA vs T2D
URS000069B369-Y_RNA2	1,381979441	9,690811268	0,04683017	0,474409979	LADA vs T2D
URS00000A00A2-antisense2	-1,21805222	4,833316746	0,053049198	0,521921688	LADA vs T2D
URS000062C73B-tRNA2	-0,847247276	4,544224584	0,053451967	0,521921688	LADA vs T2D
URS000024B38F-tRNA2	-1,072458299	4,404533737	0,054231894	0,521921688	LADA vs T2D
URS0000811AFB-antisense2	1,0594995	2,040750323	0,054506843	0,521921688	LADA vs T2D
URS0000920597-rRNA2	-0,88062547	8,520273949	0,056627598	0,529444793	LADA vs T2D
URS0000389FBF-lncRNA2	-1,047868073	2,547635864	0,057327493	0,529444793	LADA vs T2D
URS00003D2CC9-tRNA2	-1,004668687	4,271817892	0,057564813	0,529444793	LADA vs T2D
URS000065D78F-Y_RNA2	-0,327496047	5,875001903	0,060474575	0,54898348	LADA vs T2D
URS0000641C1B-Y_RNA2	-0,587432774	4,028164364	0,061601818	0,550422143	LADA vs T2D
URS00007C72D0-lncRNA2	-0,918318512	1,699191247	0,062207939	0,550422143	LADA vs T2D
URS00000AED6F-tRNA2	-0,85492176	6,83625311	0,067690759	0,586129968	LADA vs T2D
URS000075BB81-lncRNA2	0,998499388	2,83062659	0,06792064	0,586129968	LADA vs T2D
URS000047C79B-tRNA2	-0,297646027	8,413478971	0,071390571	0,601902089	LADA vs T2D
URS00000DA56A-lncRNA2	1,048021393	2,589490843	0,071470491	0,601902089	LADA vs T2D
URS000066AF0D-Y_RNA2	-0,992697866	12,73751355	0,074469002	0,614474005	LADA vs T2D
URS00006CC125-rRNA2	-1,012238774	2,366732734	0,075522139	0,614474005	LADA vs T2D
URS00001A4293-lncRNA2	-0,880126303	4,616590583	0,075956681	0,614474005	LADA vs T2D
URS0000209048-tRNA2	-1,073330816	3,508601642	0,076479597	0,614474005	LADA vs T2D
URS0000AABA8B-lncRNA2	0,446706549	9,325488704	0,077859107	0,616774865	LADA vs T2D
URS0000941729-rRNA2	0,864549889	4,187919105	0,078530705	0,616774865	LADA vs T2D
URS0000995AA3-rRNA2	-0,568939062	8,311403687	0,08104919	0,621218634	LADA vs T2D
URS00008116E3-lncRNA2	-0,878436922	4,000279228	0,08202886	0,621218634	LADA vs T2D
URS000090169F-rRNA2	0,940455984	1,691620139	0,082140246	0,621218634	LADA vs T2D
URS0000383A48-tRNA2	1,009617979	3,229014707	0,082651406	0,621218634	LADA vs T2D
URS0000727FD6-tRNA2	-1,03982852	3,320399515	0,085090034	0,632743975	LADA vs T2D
URS0000766C83-lncRNA2	1,078618663	2,482009239	0,086651749	0,637574451	LADA vs T2D
URS000069ED7F-Y_RNA2	-1,005734201	4,579144673	0,088599148	0,64511255	LADA vs T2D
URS0000ABD87F-rRNA2	-1,638711562	10,81861494	0,095031203	0,65928592	LADA vs T2D
URS000033F395-lncRNA2	-0,909372337	1,897833694	0,096447118	0,65928592	LADA vs T2D
URS00001A86BB-tRNA2	-0,827222631	2,65628615	0,096545397	0,65928592	LADA vs T2D
URS000071E736-Y_RNA2	-1,01907233	2,593633412	0,099491203	0,65928592	LADA vs T2D
URS000038D781-lncRNA2	1,001270722	2,120835096	0,099701975	0,65928592	LADA vs T2D
URS0000ABD8C6-rRNA2	-2,011536251	7,712316992	0,100518646	0,65928592	LADA vs T2D
URS000050E9EC-lncRNA2	-0,847270626	3,382080638	0,100990525	0,65928592	LADA vs T2D
URS000009DDCA-tRNA2	-0,433984879	8,707527281	0,102283297	0,65928592	LADA vs T2D
URS000060A110-antisense2	0,749342593	1,367875278	0,104110076	0,65928592	LADA vs T2D
URS0000784C7B-lncRNA2	0,922196929	2,086288772	0,104233937	0,65928592	LADA vs T2D

URS00006C9A71-Y_RNA2	-0,834887293	2,255341052	0,105195108	0,65928592	LADA vs T2D
URS00001FBD75-misc_RNA]2	1,388913206	3,307722647	0,105328107	0,65928592	LADA vs T2D
URS000064E10F-tRNA2	-0,946957592	3,013775094	0,106350474	0,65928592	LADA vs T2D
URS00003D60C0-lncRNA2	0,875696336	1,735581371	0,108205011	0,65928592	LADA vs T2D
URS0000590507-lncRNA2	-0,864856796	1,90331514	0,109370812	0,65928592	LADA vs T2D
URS0000767DAE-lncRNA2	0,563896916	5,637395825	0,109603037	0,65928592	LADA vs T2D
URS000038F4B2-tRNA2	-0,980085459	2,156583414	0,109772814	0,65928592	LADA vs T2D
URS000030BAD5-tRNA2	-0,42081706	9,963231125	0,110356613	0,65928592	LADA vs T2D
URS00006174C2-tRNA2	-0,359249606	8,218629708	0,111100812	0,65928592	LADA vs T2D
URS00004A68E9-antisense2	-0,665934681	3,654195066	0,11314728	0,65928592	LADA vs T2D
URS0000AAD5AA-lncRNA2	-1,274793297	10,83600588	0,113244541	0,65928592	LADA vs T2D
URS0000697465-tRNA2	-0,953011262	4,721333977	0,114020057	0,65928592	LADA vs T2D
URS00007125F9-rRNA2	-0,924392289	2,409823294	0,114420929	0,65928592	LADA vs T2D
URS00004C5EDB-lncRNA2	0,982780366	2,83469135	0,115140294	0,65928592	LADA vs T2D
URS00009A5DA8-rRNA2	0,69172797	5,880019281	0,11559587	0,65928592	LADA vs T2D
URS0000629ECF-Y_RNA2	-0,578675009	4,37154222	0,116577534	0,65928592	LADA vs T2D
URS0000699390-Y_RNA2	-0,743908242	13,62373063	0,11691878	0,65928592	LADA vs T2D
URS00000081EA-snRNA2	-0,909498776	2,66468092	0,117184891	0,65928592	LADA vs T2D
URS00006BB04D-tRNA2	0,882125535	3,054494403	0,117898054	0,65928592	LADA vs T2D
URS00002C4609-lncRNA2	-0,90867087	2,010894744	0,121698629	0,665079725	LADA vs T2D
URS0000028D07-lncRNA2	0,774882224	5,403821621	0,122713131	0,665079725	LADA vs T2D
URS00007062F7-Y_RNA2	-0,60060283	4,160857713	0,123077444	0,665079725	LADA vs T2D
URS0000188F7D-scRNA2	-2,308238348	6,577829264	0,123941206	0,665079725	LADA vs T2D
URS0000031963-antisense2	-1,076845242	3,414756941	0,124350577	0,665079725	LADA vs T2D
URS000071DF37-tRNA2	-1,008496954	2,691836943	0,124642981	0,665079725	LADA vs T2D
URS000034AAC2-tRNA2	-0,81923449	4,895139042	0,129598535	0,678438314	LADA vs T2D
URS00006BBD5F-Y_RNA2	-0,867721388	3,385968133	0,129733187	0,678438314	LADA vs T2D
URS000076FC5A-lncRNA2	-0,941350251	2,343917503	0,130058275	0,678438314	LADA vs T2D
URS00004227BE-lncRNA2	-0,876909835	1,875905236	0,132089737	0,679106278	LADA vs T2D
URS00001A72CE-tRNA2	-0,867662286	4,122188898	0,13247887	0,679106278	LADA vs T2D
URS000001AE93-lncRNA2	0,348266522	6,898854125	0,133100944	0,679106278	LADA vs T2D
URS00004AE57B-lncRNA2	-0,862979029	2,181566953	0,134852683	0,680129503	LADA vs T2D
URS000003870B-lncRNA2	-0,312060401	9,957227048	0,136333884	0,680129503	LADA vs T2D
URS000045FD15-lncRNA2	-0,806848377	4,004446293	0,136995985	0,680129503	LADA vs T2D
URS00006F0FC0-tRNA2	-0,578257757	5,435006769	0,137193505	0,680129503	LADA vs T2D
URS00004AE46A-lncRNA2	-0,826319933	3,574152246	0,138907433	0,683776729	LADA vs T2D
URS0000955796-rRNA2	0,169104197	10,7330721	0,141942682	0,689882335	LADA vs T2D
URS000098FA76-rRNA2	-0,436525427	2,496348799	0,14394582	0,689882335	LADA vs T2D
URS000036E063-antisense2	0,174423272	10,32347827	0,143961212	0,689882335	LADA vs T2D
URS00001AD596-tRNA2	-0,239525965	11,15468011	0,144095595	0,689882335	LADA vs T2D
URS00006642D4-Y_RNA2	-0,279917157	4,428261145	0,146039551	0,692930048	LADA vs T2D
URS0000417E86-lncRNA2	0,868844168	2,54225615	0,146714803	0,692930048	LADA vs T2D
URS000017D264-lncRNA2	-0,821961582	2,092743225	0,150189706	0,702141468	LADA vs T2D
URS00006005A4-lncRNA2	-0,994872385	2,906455771	0,150674135	0,702141468	LADA vs T2D
URS000047EBB5-tRNA2	-0,916524602	2,438958701	0,154877984	0,716951726	LADA vs T2D
URS000011812A-antisense2	-0,687963046	4,132007293	0,157250186	0,723143946	LADA vs T2D
URS00006FF680-Y_RNA2	0,809036966	2,916835355	0,161439443	0,73755667	LADA vs T2D
URS00006E3DE1-snRNA2	-0,895875636	3,143314554	0,163099476	0,740302169	LADA vs T2D
URS000000199E-lncRNA2	0,558089606	6,442848869	0,167543143	0,752518057	LADA vs T2D
URS0000717DFB-Y_RNA2	-0,719744177	2,95584216	0,167943944	0,752518057	LADA vs T2D
URS00001B59BD-lncRNA2	-0,356156715	6,767957763	0,170990839	0,756276546	LADA vs T2D
URS00005FFC78-lncRNA2	0,784312547	2,519542247	0,171645641	0,756276546	LADA vs T2D
URS000022DD4A-tRNA2	-0,311326185	9,64520168	0,176286828	0,756276546	LADA vs T2D
URS00000347CF-lncRNA2	-0,66043946	3,718528756	0,176351678	0,756276546	LADA vs T2D
URS00006D9244-tRNA2	-0,847006467	3,430512932	0,176507442	0,756276546	LADA vs T2D
URS00006F4E76-Y_RNA2	-0,353435827	0,766333934	0,182517163	0,756276546	LADA vs T2D
URS0000282AB2-tRNA2	1,046343271	4,591558308	0,183260012	0,756276546	LADA vs T2D
URS0000278E1B-tRNA2	-0,744795355	2,766067348	0,183838777	0,756276546	LADA vs T2D
URS000065A213-Y_RNA2	-0,63345687	10,7844197	0,184448686	0,756276546	LADA vs T2D
URS000006044C-lncRNA2	-0,706321517	1,808588154	0,186381124	0,756276546	LADA vs T2D
URS0000977FD2-SRP_RNA2	-0,740165038	2,037495207	0,186890874	0,756276546	LADA vs T2D
URS0000700D52-tRNA2	-0,77826095	2,144408558	0,187815191	0,756276546	LADA vs T2D
URS00006271F5-Y_RNA2	-0,590840292	3,66489103	0,188695943	0,756276546	LADA vs T2D
URS00004106BA-lncRNA2	-0,630977498	1,798128499	0,192580784	0,756276546	LADA vs T2D
URS000099D184-rRNA2	-0,523313474	4,977808511	0,193062096	0,756276546	LADA vs T2D
URS0000920CBD-rRNA2	1,621349812	5,869762745	0,194132536	0,756276546	LADA vs T2D

URS0000196BD3-lncRNA2	0,711116641	2,056335374	0,194147775	0,756276546	LADA vs T2D
URS000068483A-rRNA2	0,766945548	2,397770416	0,194230461	0,756276546	LADA vs T2D
URS0000624312-Y_RNA2	-0,51944714	5,698207316	0,195611989	0,756276546	LADA vs T2D
URS0000265843-antisense2	-0,679265666	2,862204159	0,196167954	0,756276546	LADA vs T2D
URS000071ED2F-tRNA2	-0,817205668	2,991616318	0,197407569	0,756276546	LADA vs T2D
URS000070B37B-tRNA2	-0,237907797	9,295263071	0,197459424	0,756276546	LADA vs T2D
URS0000A89523-lncRNA2	0,760119636	2,553603804	0,197618046	0,756276546	LADA vs T2D
URS00006CDFA7-rRNA2	-0,683814982	2,666787507	0,199187067	0,756276546	LADA vs T2D
URS00004F2E54-lncRNA2	-0,732859517	1,792153167	0,200396467	0,756276546	LADA vs T2D
URS000099BDEE-rRNA2	0,738479698	5,090607985	0,200811558	0,756276546	LADA vs T2D
URS000064506B-tRNA2	-0,81997457	2,909810805	0,202159024	0,756276546	LADA vs T2D
URS0000488EA7-lncRNA2	0,762353151	2,312667922	0,204312211	0,756276546	LADA vs T2D
URS0000204428-lncRNA2	-0,762565514	2,674128924	0,204417715	0,756276546	LADA vs T2D
URS0000417A0F-tRNA2	-0,524776691	7,465949428	0,20781757	0,756276546	LADA vs T2D
URS00002FBC9E-lncRNA2	-0,756256114	3,539604284	0,208130129	0,756276546	LADA vs T2D
URS0000100BA4-antisense2	-0,72700934	1,836907151	0,208138599	0,756276546	LADA vs T2D
URS000051CFB0-lncRNA2	-1,158217904	3,801776289	0,208264708	0,756276546	LADA vs T2D
URS0000ABD879-rRNA2	-1,498708346	7,136004534	0,211210701	0,756276546	LADA vs T2D
URS0000AAECF4-lncRNA2	-0,57800932	3,702161679	0,211605399	0,756276546	LADA vs T2D
URS0000397495-antisense2	-0,425575019	5,990008067	0,212484274	0,756276546	LADA vs T2D
URS0000415026-tRNA2	-0,566546631	4,426745558	0,213174294	0,756276546	LADA vs T2D
URS0000701637-rRNA2	-0,756290301	2,670549892	0,213678806	0,756276546	LADA vs T2D
URS00004D9E92-tRNA2	-0,336093616	7,483097251	0,215372421	0,756276546	LADA vs T2D
URS0000371842-lncRNA2	0,706532523	2,020987369	0,215670046	0,756276546	LADA vs T2D
URS0000121433-tRNA2	-0,936872046	4,021698317	0,216855304	0,756276546	LADA vs T2D
URS00001D081D-antisense2	-0,688186401	1,824716399	0,217309122	0,756276546	LADA vs T2D
URS00004F0321-tRNA2	-0,374837959	14,08130095	0,217622613	0,756276546	LADA vs T2D
URS000095D156-rRNA2	0,509877979	6,045790564	0,218100842	0,756276546	LADA vs T2D
URS000029CCC5-tRNA2	-0,670268345	2,732517708	0,218832599	0,756276546	LADA vs T2D
URS0000996BBC-rRNA2	-0,226645212	8,622403976	0,219253436	0,756276546	LADA vs T2D
URS0000A765F3-lncRNA2	-0,63650365	3,81544174	0,22035534	0,756276546	LADA vs T2D
URS00000C7470-antisense2	-0,686284215	3,497403787	0,22133156	0,756276546	LADA vs T2D
URS000028E102-lncRNA2	-0,683590368	2,546561157	0,222755898	0,756276546	LADA vs T2D
URS000032B6B6-snRNA2	-0,257978846	8,295722512	0,222879783	0,756276546	LADA vs T2D
URS00003870EC-lncRNA2	-0,754286513	2,283625175	0,224677022	0,757175988	LADA vs T2D
URS0000120E41-tRNA2	-0,384415497	6,378757577	0,225768846	0,757175988	LADA vs T2D
URS000075AC32-lncRNA2	0,724746883	2,916319528	0,226394537	0,757175988	LADA vs T2D
URS0000A81496-Y_RNA2	0,791794998	2,190786251	0,228054014	0,758902416	LADA vs T2D
URS000076E14E-lncRNA2	0,821689339	2,947769863	0,229082131	0,758902416	LADA vs T2D
URS0000A9CA30-lncRNA2	0,661721212	3,103219008	0,239385446	0,789294466	LADA vs T2D
URS000052A1C9-tRNA2	-0,607498125	4,427113529	0,241919498	0,792756086	LADA vs T2D
URS0000591DFC-lncRNA2	0,609775613	1,976731906	0,244583053	0,792756086	LADA vs T2D
URS000031A3C9-lncRNA2	0,582023972	4,013881647	0,245229587	0,792756086	LADA vs T2D
URS000042199A-lncRNA2	0,722588634	2,107510848	0,246007762	0,792756086	LADA vs T2D
URS00003D4983-antisense2	-0,655611717	1,851867325	0,246105967	0,792756086	LADA vs T2D
URS0000637E4A-tRNA2	-0,627579419	1,552702938	0,248446227	0,796623454	LADA vs T2D
URS000047AE74-lncRNA2	-0,661128204	2,522838605	0,252510019	0,805956636	LADA vs T2D
URS00005AF005-lncRNA2	-0,623992729	3,522774737	0,255571447	0,812020187	LADA vs T2D
URS0000A8472C-Y_RNA2	0,724813468	3,823381815	0,260656018	0,822036079	LADA vs T2D
URS000075A564-lncRNA2	-0,63282626	2,096352205	0,264285253	0,822036079	LADA vs T2D
URS0000635FFC-Y_RNA2	-0,634525814	2,322009496	0,26580305	0,822036079	LADA vs T2D
URS000090DF6D-rRNA2	0,124596206	11,93047681	0,266487614	0,822036079	LADA vs T2D
URS000093FF31-rRNA2	0,592505705	2,038384139	0,26658059	0,822036079	LADA vs T2D
URS00005B6D58-lncRNA2	-0,538287817	5,324374408	0,267065229	0,822036079	LADA vs T2D
URS0000ABD7D5-rRNA2	0,103397834	13,17747017	0,267311211	0,822036079	LADA vs T2D
URS00002EA13A-lncRNA2	-0,614460585	2,908290455	0,268963529	0,822036079	LADA vs T2D
URS0000504A1A-lncRNA2	-0,576300229	1,470603839	0,269378471	0,822036079	LADA vs T2D
URS0000166229-lncRNA2	-0,574704883	2,097439644	0,270483974	0,822036079	LADA vs T2D
URS00001DFAE4-lncRNA2	-0,766970645	3,289114858	0,272642944	0,82367546	LADA vs T2D
URS0000082453-lncRNA2	0,593674654	2,456766093	0,273380124	0,82367546	LADA vs T2D
URS00001E5F12-lncRNA2	-0,637394865	2,516521413	0,276896773	0,830690319	LADA vs T2D
URS000064D54F-tRNA2	-0,5882708	2,523144202	0,279371503	0,834532822	LADA vs T2D
URS000070E3CE-rRNA2	-0,563327304	4,081177344	0,282933547	0,841576807	LADA vs T2D
URS00005B5F85-lncRNA2	0,584211972	5,187781526	0,28695946	0,844871111	LADA vs T2D
URS0000A9F786-lncRNA2	-0,715171574	3,099717163	0,287617789	0,844871111	LADA vs T2D
URS000009738A-lncRNA2	0,635076328	2,916436163	0,287667131	0,844871111	LADA vs T2D

URS00000B8842-lncRNA2	-0,488409372	4,912142401	0,289898549	0,845090986	LADA vs T2D
URS000053D4AB-snoRNA2	-0,573866444	1,689585927	0,291022578	0,845090986	LADA vs T2D
URS000075D28F-lncRNA2	-0,592542369	2,277607756	0,291368995	0,845090986	LADA vs T2D
URS000075BA00-lncRNA2	-1,331568391	7,953608312	0,295512436	0,849529418	LADA vs T2D
URS0000ABD7E9-lncRNA2	-0,623051323	3,512762788	0,296134473	0,849529418	LADA vs T2D
URS00006C8EDF-tRNA2	-0,633924476	3,590220694	0,296545319	0,849529418	LADA vs T2D
URS00006952C9-Y_RNA2	-0,73258218	4,013121466	0,301212118	0,852680383	LADA vs T2D
URS000034E03C-antisense2	-0,603161353	2,078398724	0,301852938	0,852680383	LADA vs T2D
URS0000682FB0-Y_RNA2	-0,431522287	10,97882695	0,302951327	0,852680383	LADA vs T2D
URS0000418239-snRNA2	0,518612547	2,184896985	0,303124742	0,852680383	LADA vs T2D
URS00007CA557-lncRNA2	0,244125662	9,085280894	0,306312362	0,852680383	LADA vs T2D
URS00006F5B12-Y_RNA2	-0,613684555	5,757189507	0,3068106	0,852680383	LADA vs T2D
URS00006E23A8-rRNA2	-0,642125033	2,840982885	0,30739205	0,852680383	LADA vs T2D
URS00007DFA49-antisense2	0,723563095	2,453720457	0,307404087	0,852680383	LADA vs T2D
URS00006D4DB0-snRNA2	-0,645132992	3,391553507	0,312605361	0,859155203	LADA vs T2D
URS00002A28BF-lncRNA2	-0,654486742	2,823053274	0,312922019	0,859155203	LADA vs T2D
URS00001B2779-lncRNA2	-0,575187206	1,848580988	0,3155991	0,859155203	LADA vs T2D
URS0000181B59-lncRNA2	-0,687417228	2,274734234	0,315838883	0,859155203	LADA vs T2D
URS000068F7ED-Y_RNA2	-0,502973921	6,623874775	0,316343852	0,859155203	LADA vs T2D
URS00004D1520-lncRNA2	0,572311418	3,203873127	0,317113079	0,859155203	LADA vs T2D
URS000007383C-antisense2	-0,534558161	2,496432723	0,321909877	0,860658797	LADA vs T2D
URS00001AF592-antisense2	-0,618629628	3,230887811	0,321945881	0,860658797	LADA vs T2D
URS00006A2BF7-Y_RNA2	-0,580389674	10,1864015	0,3241417	0,860658797	LADA vs T2D
URS00005CEC24-lncRNA2	0,591436643	2,918669315	0,326386739	0,860658797	LADA vs T2D
URS0000ABD7E8-rRNA2	-0,89754408	10,98616314	0,328394442	0,860658797	LADA vs T2D
URS00007D436A-antisense2	-0,532801489	2,239346559	0,330068072	0,860658797	LADA vs T2D
URS00006B479B-tRNA2	-0,443700866	1,462161631	0,331833318	0,860658797	LADA vs T2D
URS000048B807-lncRNA2	-0,48282344	4,663045601	0,333083488	0,860658797	LADA vs T2D
URS0000462D45-lncRNA2	0,497512849	3,263068544	0,335127186	0,860658797	LADA vs T2D
URS00005DB87D-tRNA2	-0,192243926	7,975358739	0,335472235	0,860658797	LADA vs T2D
URS00005580B2-lncRNA2	-0,637051011	3,442360628	0,337227537	0,860658797	LADA vs T2D
URS0000537899-antisense2	-0,548460356	2,143848964	0,337555426	0,860658797	LADA vs T2D
URS00002C2371-lncRNA2	-0,523538942	3,691720987	0,337746112	0,860658797	LADA vs T2D
URS000063CFC9-Y_RNA2	-0,567013768	2,480380401	0,338352281	0,860658797	LADA vs T2D
URS00001EC8D7-snRNA2	0,395704406	4,577467935	0,338423982	0,860658797	LADA vs T2D
URS00007BC71B-lncRNA2	-0,56910466	1,944342872	0,338703806	0,860658797	LADA vs T2D
URS00007080E9-Y_RNA2	-0,708576589	2,936384424	0,339773872	0,860658797	LADA vs T2D
URS00005B30A9-tRNA2	-0,626227248	2,750819629	0,340821886	0,860658797	LADA vs T2D
URS00006CE1FB-rRNA2	0,942494796	5,102179845	0,341062213	0,860658797	LADA vs T2D
URS0000664EAD-tRNA2	0,595904637	2,046606184	0,344499768	0,863714603	LADA vs T2D
URS0000762146-antisense2	0,348867599	5,166011847	0,347721395	0,863714603	LADA vs T2D
URS000021BDC3-snRNA2	0,502250356	2,084780028	0,348416592	0,863714603	LADA vs T2D
URS0000016595-lncRNA2	-0,556387137	2,839797195	0,34861343	0,863714603	LADA vs T2D
URS0000576D5D-lncRNA2	-0,511197878	1,73318224	0,348628395	0,863714603	LADA vs T2D
URS00003F41E9-lncRNA2	0,187710961	8,007323205	0,349687028	0,863714603	LADA vs T2D
URS0000AAB7F4-lncRNA2	-0,518425384	3,962630959	0,350995205	0,863893128	LADA vs T2D
URS0000676525-Y_RNA2	-0,471065216	4,329395557	0,354242431	0,868826174	LADA vs T2D
URS000094F5D5-rRNA2	-0,418914602	7,000982595	0,355853218	0,869725172	LADA vs T2D
URS00006CB0C3-Y_RNA2	-0,217358389	5,291889769	0,361743029	0,881039642	LADA vs T2D
URS00002B7986-antisense2	-0,475892203	1,782132427	0,364979819	0,885836435	LADA vs T2D
URS00002AD8DA-lncRNA2	0,476397771	2,376170134	0,366264902	0,88587947	LADA vs T2D
URS000041FE38-lncRNA2	-0,489644373	2,353684167	0,368887416	0,887772533	LADA vs T2D
URS0000511A5F-lncRNA2	-0,510515366	1,837603255	0,369587707	0,887772533	LADA vs T2D
URS00003A72DC-antisense2	0,487449836	3,084869652	0,37145873	0,889211139	LADA vs T2D
URS00000EB76F-lncRNA2	0,520658151	2,382363845	0,372837879	0,889466476	LADA vs T2D
URS0000022477-lncRNA2	0,49251798	1,898475606	0,377105136	0,894935975	LADA vs T2D
URS000051EF4B-lncRNA2	-0,479299644	1,97882489	0,37970103	0,894935975	LADA vs T2D
URS00004D4BFC-lncRNA2	0,435624554	2,209794014	0,38127777	0,894935975	LADA vs T2D
URS0000365006-lncRNA2	-0,468530306	2,397164181	0,382221829	0,894935975	LADA vs T2D
URS0000994031-rRNA2	-0,129562451	11,10957664	0,385007095	0,894935975	LADA vs T2D
URS0000812136-lncRNA2	0,191336508	9,121672555	0,385151155	0,894935975	LADA vs T2D
URS000063455F-rRNA2	-0,483126199	2,601127664	0,386287416	0,894935975	LADA vs T2D
URS00009840C1-rRNA2	0,344085748	6,418341272	0,388618693	0,894935975	LADA vs T2D
URS00003F2105-lncRNA2	-0,491046196	2,406932528	0,388644486	0,894935975	LADA vs T2D
URS00002CC2A4-antisense2	-0,488361743	2,224906264	0,392359199	0,894935975	LADA vs T2D
URS0000611F3E-lncRNA2	-0,469537544	2,798037489	0,394226837	0,894935975	LADA vs T2D

URS0000667737-rRNA2	1,0013604	4,834926248	0,394624216	0,894935975	LADA vs T2D
URS00003EAC96-antisense2	0,523575186	2,99991317	0,395319385	0,894935975	LADA vs T2D
URS0000495A30-lncRNA2	0,445675927	2,253557055	0,395773828	0,894935975	LADA vs T2D
URS00007D61AD-antisense2	0,62398124	2,769938297	0,396891953	0,894935975	LADA vs T2D
URS0000005EDF-lncRNA2	0,439997276	4,116716413	0,397490139	0,894935975	LADA vs T2D
URS00003AD33F-lncRNA2	0,525937289	3,336093709	0,399233078	0,894935975	LADA vs T2D
URS000063A6E6-rRNA2	0,098754611	11,70425189	0,399381456	0,894935975	LADA vs T2D
URS00002901EC-tRNA2	-0,490540883	2,043121881	0,400648032	0,894935975	LADA vs T2D
URS00005B2DE5-lncRNA2	-0,441905258	4,758632634	0,401978944	0,894935975	LADA vs T2D
URS00000CD33E-antisense2	0,44078621	3,630533758	0,403999015	0,894935975	LADA vs T2D
URS00000B6370-lncRNA2	-0,374831393	3,6717613	0,40452572	0,894935975	LADA vs T2D
URS000005F65D-antisense2	0,428406062	4,947813537	0,404672781	0,894935975	LADA vs T2D
URS00001DE9CD-antisense2	0,492055395	3,351892383	0,406585184	0,894935975	LADA vs T2D
URS0000610FFE-tRNA2	-0,459969308	2,353369293	0,410884832	0,894935975	LADA vs T2D
URS000038803E-tRNA2	-0,259205759	5,940754642	0,414683515	0,894935975	LADA vs T2D
URS000067474D-tRNA2	-0,504010347	5,826738993	0,415530776	0,894935975	LADA vs T2D
URS00000C653A-lncRNA2	-0,450220994	2,462989968	0,41562396	0,894935975	LADA vs T2D
URS000097B164-rRNA2	0,350793161	5,947136128	0,416385671	0,894935975	LADA vs T2D
URS000023F3B4-lncRNA2	0,384533914	3,955198744	0,416533222	0,894935975	LADA vs T2D
URS0000A827F4-antisense2	0,522137426	8,224586053	0,416720991	0,894935975	LADA vs T2D
URS000038EEDC-lncRNA2	0,574148176	3,360541304	0,417718893	0,894935975	LADA vs T2D
URS00006772C0-tRNA2	-0,495884771	2,278759881	0,418368792	0,894935975	LADA vs T2D
URS000008089F-lncRNA2	-0,524124937	3,494287219	0,419641261	0,894935975	LADA vs T2D
URS00006D1735-snRNA2	-0,375973165	5,596569208	0,428214494	0,894935975	LADA vs T2D
URS00007BE6D3-lncRNA2	1,10465386	10,22836282	0,429148707	0,894935975	LADA vs T2D
URS00006CBBB9-Y_RNA2	-0,456603512	3,554703445	0,42955891	0,894935975	LADA vs T2D
URS00000AD390-antisense2	-0,434899564	1,744059197	0,430137798	0,894935975	LADA vs T2D
URS00004AAD0A-lncRNA2	0,436155091	2,996600067	0,430372915	0,894935975	LADA vs T2D
URS0000677B31-tRNA2	0,324920766	7,303130994	0,430750802	0,894935975	LADA vs T2D
URS00002D33E9-lncRNA2	-0,41866075	2,231641646	0,431705219	0,894935975	LADA vs T2D
URS000012C80D-lncRNA2	-0,435337605	2,726619231	0,431780738	0,894935975	LADA vs T2D
URS0000478C87-lncRNA2	0,424699661	2,830369346	0,431954637	0,894935975	LADA vs T2D
URS00005BB09E-lncRNA2	-0,307316784	6,047129544	0,433860308	0,894935975	LADA vs T2D
URS00006C06E6-Y_RNA2	-0,445861166	1,912207487	0,436023845	0,894935975	LADA vs T2D
URS00002D1F9F-lncRNA2	-0,395561503	3,254441607	0,436079302	0,894935975	LADA vs T2D
URS000034EAB6-lncRNA2	0,474969525	2,280705495	0,437331279	0,894935975	LADA vs T2D
URS00002598CF-lncRNA2	-0,385919595	4,630900569	0,437851291	0,894935975	LADA vs T2D
URS00006361F3-Y_RNA2	-0,582496365	4,856921385	0,43786567	0,894935975	LADA vs T2D
URS000063B690-snRNA2	-0,446654616	3,324962274	0,441634799	0,900007942	LADA vs T2D
URS0000233E9C-lncRNA2	0,590350672	2,822247929	0,445031041	0,90182019	LADA vs T2D
URS00009C6042-lncRNA2	-0,222438522	6,706154397	0,445729967	0,90182019	LADA vs T2D
URS00006481F8-Y_RNA2	0,2317243	7,303708498	0,449200566	0,90182019	LADA vs T2D
URS00004199FB-antisense2	-0,4922047	3,00203969	0,450920124	0,90182019	LADA vs T2D
URS00008120F8-lncRNA2	0,428865849	1,863766293	0,452718875	0,90182019	LADA vs T2D
URS00006CFDFE-Y_RNA2	0,315428592	2,249644641	0,456353755	0,90182019	LADA vs T2D
URS000018BCE5-antisense2	-0,427935356	2,555076799	0,456503831	0,90182019	LADA vs T2D
URS0000687FC3-Y_RNA2	-0,210680319	4,184219214	0,456716367	0,90182019	LADA vs T2D
URS000006D0D7-lncRNA2	-0,41469651	2,152173172	0,457550308	0,90182019	LADA vs T2D
URS000075D341-rRNA2	0,681350328	3,481242239	0,458834793	0,90182019	LADA vs T2D
URS00003C98B0-lncRNA2	0,446804304	3,045496821	0,459108577	0,90182019	LADA vs T2D
URS00006E19EA-Y_RNA2	0,28715907	9,597384946	0,459226909	0,90182019	LADA vs T2D
URS000075D353-lncRNA2	0,38849026	1,653940152	0,45929612	0,90182019	LADA vs T2D
URS0000653BD1-Y_RNA2	0,37886135	4,226797259	0,461013355	0,9026564	LADA vs T2D
URS000075DF54-lncRNA2	-0,240485873	6,14755677	0,464020577	0,903294042	LADA vs T2D
URS00000540AC-antisense2	-0,401593672	3,451834854	0,464952004	0,903294042	LADA vs T2D
URS0000918BC5-rRNA2	-0,641576258	3,816571784	0,465215816	0,903294042	LADA vs T2D
URS000066C003-Y_RNA]2	-0,852371207	5,557417709	0,46773861	0,904873737	LADA vs T2D
URS00004AFADD-lncRNA2	0,411096615	1,695028896	0,469048967	0,904873737	LADA vs T2D
URS00000A586F-lncRNA2	0,227877159	0,916847513	0,471149981	0,904873737	LADA vs T2D
URS0000341866-lncRNA2	-0,426780294	4,848112839	0,471423422	0,904873737	LADA vs T2D
URS0000594305-lncRNA2	-0,385102638	1,940137107	0,473907578	0,904873737	LADA vs T2D
URS00001E15E2-lncRNA2	-0,366590695	2,285605947	0,473961345	0,904873737	LADA vs T2D
URS00005A57D3-lncRNA2	-0,42043787	2,579526429	0,475091075	0,904873737	LADA vs T2D
URS00002E367B-antisense2	-0,391541855	1,915955023	0,483397282	0,908767095	LADA vs T2D
URS000077A114-lncRNA2	-0,1174398	7,694058287	0,483923551	0,908767095	LADA vs T2D
URS000062C4DE-tRNA2	-0,401365746	3,676191563	0,484268498	0,908767095	LADA vs T2D

URS00009C606C-lncRNA2	-0,248023169	5,246519845	0,48451239	0,908767095	LADA vs T2D
URS0000197DBF-antisense2	-0,369578387	3,806884628	0,485114394	0,908767095	LADA vs T2D
URS00004B48CD-lncRNA2	0,391872959	1,80838911	0,486653579	0,908767095	LADA vs T2D
URS0000257C23-lncRNA2	-0,463816007	4,584581553	0,487977089	0,908767095	LADA vs T2D
URS000094439F-rRNA2	0,080586072	11,62287065	0,488194269	0,908767095	LADA vs T2D
URS0000918AFB-rRNA2	0,169672008	8,610055402	0,488836091	0,908767095	LADA vs T2D
URS000055C9F8-antisense2	0,313916103	4,017918826	0,491573019	0,911431141	LADA vs T2D
URS00000CCACD-antisense2	0,481515458	4,642488599	0,497563442	0,915280956	LADA vs T2D
URS0000189042-lncRNA2	-0,406327766	1,901016294	0,498123233	0,915280956	LADA vs T2D
URS00003ADE1B-lncRNA2	-0,395586016	2,400792438	0,498580872	0,915280956	LADA vs T2D
URS000062F68C-rRNA2	-0,089888257	10,93938784	0,498887045	0,915280956	LADA vs T2D
URS0000931B54-rRNA2	-0,052325962	14,46696978	0,500370205	0,915598883	LADA vs T2D
URS000040CE64-lncRNA2	0,442247456	2,883976268	0,503786515	0,917918772	LADA vs T2D
URS000063E4FD-tRNA2	-0,410796509	3,061724682	0,504581768	0,917918772	LADA vs T2D
URS0000690F87-snRNA2	-0,463730169	3,114315559	0,507743065	0,917918772	LADA vs T2D
URS0000928682-rRNA2	-0,084441379	11,56053599	0,509122739	0,917918772	LADA vs T2D
URS00009AAC46-rRNA2	-0,11954901	9,797442669	0,509621881	0,917918772	LADA vs T2D
URS0000527686-lncRNA2	0,25739875	1,162280834	0,510465162	0,917918772	LADA vs T2D
URS00006A9AE8-Y_RNA2	-0,476566696	5,946221857	0,510830333	0,917918772	LADA vs T2D
URS00007D24CA-lncRNA2	0,488572294	2,948889293	0,515116927	0,920473407	LADA vs T2D
URS0000543B4D-lncRNA2	-0,390844476	2,605909076	0,515680869	0,920473407	LADA vs T2D
URS00004DA951-rRNA2	-0,209132939	10,73867185	0,51620254	0,920473407	LADA vs T2D
URS0000005270-rRNA2	-0,582351157	6,166410111	0,517543535	0,920516364	LADA vs T2D
URS00004BE455-lncRNA2	-0,266170761	5,228003516	0,526030681	0,932472306	LADA vs T2D
URS00002EDD28-lncRNA2	0,238314175	5,436899358	0,526933563	0,932472306	LADA vs T2D
URS0000228E94-lncRNA2	0,364834929	2,335958891	0,532460617	0,935068963	LADA vs T2D
URS00001AE429-lncRNA2	-0,383880686	3,26760494	0,533004162	0,935068963	LADA vs T2D
URS00005BE013-lncRNA2	-0,456119311	2,759573682	0,533870451	0,935068963	LADA vs T2D
URS000049BB82-lncRNA2	0,301823349	4,039331254	0,538025588	0,935068963	LADA vs T2D
URS000076B0D1-lncRNA2	-0,345299755	3,094918128	0,538676069	0,935068963	LADA vs T2D
URS0000134A86-antisense2	-0,402889551	3,351781773	0,538869152	0,935068963	LADA vs T2D
URS00008BB15B-lncRNA2	-0,415056178	2,482102831	0,539038289	0,935068963	LADA vs T2D
URS000097171C-SRP_RNA2	-0,337694017	2,924722071	0,539102707	0,935068963	LADA vs T2D
URS00007116F9-Y_RNA2	-0,331449326	3,042319437	0,541835583	0,937482853	LADA vs T2D
URS00005D1950-antisense2	-0,317720894	2,067377939	0,544299348	0,938911435	LADA vs T2D
URS0000811D9C-antisense2	0,221388015	0,958216717	0,545429455	0,938911435	LADA vs T2D
URS00000C18F2-tRNA2	-0,263450083	6,19082938	0,54683075	0,938911435	LADA vs T2D
URS0000038B1E-antisense2	0,314426187	3,244363175	0,5506709	0,938911435	LADA vs T2D
URS00004C82E1-antisense2	-0,365640317	4,084336348	0,551876559	0,938911435	LADA vs T2D
URS00006E1108-Y_RNA2	-0,199498899	4,049124964	0,554290302	0,938911435	LADA vs T2D
URS00000E9A71-lncRNA2	0,320315695	3,823249127	0,555857118	0,938911435	LADA vs T2D
URS000011ABD1-lncRNA2	-0,374836576	2,282001703	0,555874293	0,938911435	LADA vs T2D
URS0000759CF4-lncRNA2	-0,330204647	1,845197414	0,556202711	0,938911435	LADA vs T2D
URS00002C9C48-lncRNA2	-0,32762694	5,358879491	0,557251077	0,938911435	LADA vs T2D
URS000060D3B6-lncRNA2	-0,447225832	4,588963786	0,557580008	0,938911435	LADA vs T2D
URS000019B78E-misc_RNA]2	-0,484073735	3,588733066	0,55877991	0,938911435	LADA vs T2D
URS00005BB5C9-antisense2	-0,306656303	1,956136305	0,560598282	0,939509248	LADA vs T2D
URS00001DBD56-lncRNA2	-0,290362993	3,720321434	0,563299246	0,939509248	LADA vs T2D
URS00003E3BDF-antisense2	0,309042791	2,398707663	0,565745573	0,939509248	LADA vs T2D
URS000050FA69-lncRNA2	-0,295637894	3,930388911	0,572019122	0,939509248	LADA vs T2D
URS00009C6070-lncRNA2	-0,293869196	2,53934864	0,573972846	0,939509248	LADA vs T2D
URS00009C60C3-lncRNA2	0,427196691	3,075209684	0,575756783	0,939509248	LADA vs T2D
URS00006428FD-Y_RNA2	-0,243899111	6,936586919	0,575873085	0,939509248	LADA vs T2D
URS000050471D-lncRNA2	-0,347392692	2,378838415	0,577340863	0,939509248	LADA vs T2D
URS0000649B00-rRNA2	-0,080741358	10,09773081	0,577464444	0,939509248	LADA vs T2D
URS00002E81F5-lncRNA2	0,276242785	4,084000104	0,577482095	0,939509248	LADA vs T2D
URS00006C2C4A-Y_RNA2	-0,412442051	4,927831545	0,577887185	0,939509248	LADA vs T2D
URS000009AC8B-tRNA2	-0,376003443	10,95540037	0,578460465	0,939509248	LADA vs T2D
URS0000776086-lncRNA2	0,200422331	7,320712579	0,579610159	0,939509248	LADA vs T2D
URS000000898B-lncRNA2	-0,339095778	2,509136511	0,584159573	0,939509248	LADA vs T2D
URS000091B709-rRNA2	0,091545088	9,600448226	0,587893522	0,939509248	LADA vs T2D
URS000023352F-lncRNA2	0,27904362	2,158198536	0,588781462	0,939509248	LADA vs T2D
URS0000399BDA-lncRNA2	-0,294729206	5,117165985	0,591166364	0,939509248	LADA vs T2D
URS0000A88906-antisense2	-0,3105401	1,925012855	0,592784583	0,939509248	LADA vs T2D
URS0000A85AEE-lncRNA2	-0,346542185	3,200285639	0,59295275	0,939509248	LADA vs T2D
URS00005B6FC3-lncRNA2	0,230132735	4,468244677	0,593047649	0,939509248	LADA vs T2D

URS00001E6C0A-lncRNA2	0,284184355	3,103912065	0,594623944	0,939509248	LADA vs T2D
URS0000089048-antisense2	-0,28261561	1,859342356	0,595010928	0,939509248	LADA vs T2D
URS00006C0413-Y_RNA2	0,319687169	2,155330761	0,595495583	0,939509248	LADA vs T2D
URS00002172B5-lncRNA2	-0,37204611	2,390444931	0,596391482	0,939509248	LADA vs T2D
URS0000112A1A-antisense2	0,280645779	4,257411498	0,596622734	0,939509248	LADA vs T2D
URS0000515855-lncRNA2	0,273856071	2,502117237	0,599559005	0,939509248	LADA vs T2D
URS000017CF23-antisense2	-0,286200665	2,666340903	0,600610709	0,939509248	LADA vs T2D
URS000037D0FB-tRNA2	-0,354538013	2,792154767	0,600742938	0,939509248	LADA vs T2D
URS0000A9525F-antisense2	0,283881378	2,995531648	0,601916088	0,939509248	LADA vs T2D
URS00003D279B-lncRNA2	-0,28335265	3,869764687	0,603870026	0,939509248	LADA vs T2D
URS0000593A4A-lncRNA2	-0,312076159	3,380617038	0,605697052	0,939509248	LADA vs T2D
URS000096B970-rRNA2	-0,06904244	10,27519744	0,60722903	0,939509248	LADA vs T2D
URS0000A7BA37-lncRNA2	-0,275170433	1,699099075	0,608092722	0,939509248	LADA vs T2D
URS000052A6A6-lncRNA2	-0,274852761	3,111397823	0,608667953	0,939509248	LADA vs T2D
URS000062FB25-rRNA2	0,067327764	9,742461609	0,608754714	0,939509248	LADA vs T2D
URS00004CE099-lncRNA2	0,422534433	3,298450018	0,608903668	0,939509248	LADA vs T2D
URS00006C48EB-Y_RNA]2	0,340451869	2,788995838	0,609890364	0,939509248	LADA vs T2D
URS000015BB33-lncRNA2	-0,255374591	3,745978664	0,610210585	0,939509248	LADA vs T2D
URS00009612D1-rRNA2	-0,217392327	5,646389432	0,613367343	0,942294006	LADA vs T2D
URS00009407E6-rRNA2	-0,298083415	2,030446175	0,61951289	0,943144525	LADA vs T2D
URS00006144FC-lncRNA2	-0,203542708	7,117689109	0,620751067	0,943144525	LADA vs T2D
URS00005BC8EC-lncRNA2	0,37546812	5,143820106	0,622417126	0,943144525	LADA vs T2D
URS00005E51DB-lncRNA2	-0,235208297	4,114269415	0,623482941	0,943144525	LADA vs T2D
URS00003F2CFE-lncRNA2	-0,273124118	4,520438351	0,624607687	0,943144525	LADA vs T2D
URS000022CCD7-lncRNA2	-0,133964633	6,400235397	0,624664111	0,943144525	LADA vs T2D
URS000047AF1F-lncRNA2	0,425443518	4,480968908	0,625196203	0,943144525	LADA vs T2D
URS0000605144-antisense2	0,277398874	2,730860041	0,625724057	0,943144525	LADA vs T2D
URS00009290F1-rRNA2	-0,265946201	1,980245991	0,626064463	0,943144525	LADA vs T2D
URS00003875B8-lncRNA2	-0,299950695	2,156965412	0,630350631	0,947559336	LADA vs T2D
URS000039557B-antisense2	-0,254497982	1,694045712	0,632494378	0,948741567	LADA vs T2D
URS000013899F-tRNA2	0,229259846	4,501396588	0,63849994	0,952388265	LADA vs T2D
URS000064217E-Y_RNA2	0,277912211	2,41296644	0,63894898	0,952388265	LADA vs T2D
URS000090AA7A-rRNA2	-0,272740353	2,615865166	0,642719086	0,952388265	LADA vs T2D
URS00002064F6-tRNA2	-0,27238347	7,265714406	0,643892634	0,952388265	LADA vs T2D
URS0000640661-Y_RNA2	-0,221360543	11,13979054	0,643944096	0,952388265	LADA vs T2D
URS0000422302-lncRNA2	-0,240072802	2,914915679	0,644320887	0,952388265	LADA vs T2D
URS000070792C-rRNA2	0,257042064	1,756278837	0,644463018	0,952388265	LADA vs T2D
URS000019B78E-tRNA2	-0,371699748	3,458896569	0,652832576	0,959952971	LADA vs T2D
URS000097FEDE-rRNA2	-0,235608323	3,690975927	0,653414941	0,959952971	LADA vs T2D
URS0000A76F22-lncRNA2	0,248448207	2,591384678	0,654365339	0,959952971	LADA vs T2D
URS000096EEE8-rRNA2	0,057860647	8,595030803	0,655075203	0,959952971	LADA vs T2D
URS000044BAE3-tRNA2	-0,093170748	7,695683129	0,657353453	0,961276283	LADA vs T2D
URS000015D954-antisense2	0,246355872	1,950160466	0,659372649	0,962216037	LADA vs T2D
URS00005C51E2-lncRNA2	-0,23284466	1,604886726	0,661028518	0,962622779	LADA vs T2D
URS00004A7F9B-antisense2	-0,108943639	6,391504235	0,662960508	0,963429095	LADA vs T2D
URS000075B143-lncRNA2	-0,233408917	2,661955926	0,669697108	0,971199748	LADA vs T2D
URS000061A10B-tRNA2	-0,291922406	2,36803002	0,673076752	0,971658262	LADA vs T2D
URS0000689904-Y_RNA2	0,131879097	0,877747286	0,675607391	0,971658262	LADA vs T2D
URS0000161EE9-lncRNA2	-0,164182898	6,078069715	0,67567721	0,971658262	LADA vs T2D
URS00003F5471-lncRNA2	0,220059316	3,0793045	0,675698911	0,971658262	LADA vs T2D
URS00002B0998-lncRNA2	-0,232241281	2,419217026	0,678219293	0,971658262	LADA vs T2D
URS000068089E-Y_RNA2	0,243828313	2,479289353	0,678353694	0,971658262	LADA vs T2D
URS00000DA554-lncRNA2	-0,234893643	2,522581542	0,680406299	0,972605323	LADA vs T2D
URS0000759AE0-lncRNA2	0,215034752	1,586466937	0,682435061	0,972684547	LADA vs T2D
URS0000320E71-lncRNA2	0,199942385	2,909981717	0,684468429	0,972684547	LADA vs T2D
URS000056B96A-antisense2	-0,204108603	2,621722386	0,68465462	0,972684547	LADA vs T2D
URS000075D48E-lncRNA2	-0,199251686	1,602010302	0,686027871	0,972684547	LADA vs T2D
URS00005C7D80-antisense2	-0,235575896	2,279201521	0,687419455	0,972684613	LADA vs T2D
URS00002D2D8F-misc_RNA]2	0,246827939	1,939760416	0,693500385	0,977580492	LADA vs T2D
URS0000093DF5-lncRNA2	0,217389613	2,157430584	0,696042376	0,977580492	LADA vs T2D
URS000098604A-rRNA2	-0,218464285	1,872688165	0,697448254	0,977580492	LADA vs T2D
URS00006EBF05-misc_RNA]2	0,355323188	3,622681484	0,699113929	0,977580492	LADA vs T2D
URS00001142E8-lncRNA2	0,141231413	5,750896426	0,699630257	0,977580492	LADA vs T2D
URS00001D0305-lncRNA2	-0,069201072	7,373693454	0,700641748	0,977580492	LADA vs T2D
URS000011BDAF-antisense2	-0,199134077	2,89470373	0,700695032	0,977580492	LADA vs T2D
URS00003475A2-lncRNA2	-0,125178824	6,073704566	0,702067822	0,977580492	LADA vs T2D

URS00001AA18A-lncRNA2	-0,203763726	1,870939952	0,70539143	0,978043207	LADA vs T2D
URS00006C246E-snRNA2	0,242649092	4,016094545	0,706254501	0,978043207	LADA vs T2D
URS00009843EB-rRNA2	-0,068352812	12,28502234	0,707612588	0,978043207	LADA vs T2D
URS000055B99E-lncRNA2	0,235022782	2,716513459	0,710108768	0,978043207	LADA vs T2D
URS00004C6AFE-antisense2	0,15604584	5,444648432	0,710332064	0,978043207	LADA vs T2D
URS00002D0015-lncRNA2	0,190770805	3,226559685	0,711280378	0,978043207	LADA vs T2D
URS00006C133C-tRNA2	-0,206363943	3,680848373	0,713173995	0,978043207	LADA vs T2D
URS000058CCC6-antisense2	-0,205474512	2,503982133	0,713635778	0,978043207	LADA vs T2D
URS0000696377-Y_RNA2	-0,201526801	2,848796511	0,716017047	0,978043207	LADA vs T2D
URS0000561169-lncRNA2	0,20524334	3,951068111	0,716392163	0,978043207	LADA vs T2D
URS000075A823-rRNA2	-0,481396441	6,786069906	0,718275306	0,978476968	LADA vs T2D
URS0000007D24-misc_RNA]2	0,521509275	6,091419652	0,71950953	0,978476968	LADA vs T2D
URS00002548DF-lncRNA2	-0,187645881	1,501270697	0,721319996	0,979034325	LADA vs T2D
URS00000F6ECB-lncRNA2	-0,133399171	5,128846246	0,725432496	0,980328461	LADA vs T2D
URS00002C130C-tRNA2	-0,310275808	3,549564243	0,726373481	0,980328461	LADA vs T2D
URS0000986F6F-rRNA2	0,157432827	5,818355153	0,726556671	0,980328461	LADA vs T2D
URS0000515429-lncRNA2	-0,128567395	7,164323775	0,727883364	0,980328461	LADA vs T2D
URS0000172E58-lncRNA2	-0,182742733	3,538272714	0,732128109	0,98040058	LADA vs T2D
URS00003AA49D-lncRNA2	-0,191381868	2,72551685	0,732181981	0,98040058	LADA vs T2D
URS000045E276-antisense2	0,182123918	7,301870906	0,733779237	0,98040058	LADA vs T2D
URS00000AD0C2-lncRNA2	-0,187852394	1,982015887	0,733990574	0,98040058	LADA vs T2D
URS000019F398-lncRNA2	0,170411818	1,659002941	0,734949791	0,98040058	LADA vs T2D
URS00000D8A7A-lncRNA2	-0,180233135	2,120449279	0,739175916	0,984159934	LADA vs T2D
URS000096D31D-rRNA2	0,061588335	10,50581429	0,744029226	0,985465812	LADA vs T2D
URS0000287398-tRNA2	-0,205385996	2,440463804	0,74564497	0,985465812	LADA vs T2D
URS00005508F6-antisense2	0,180227278	3,129098015	0,74702322	0,985465812	LADA vs T2D
URS00008116FE-lncRNA2	0,178340567	2,826119493	0,748718687	0,985465812	LADA vs T2D
URS00001E9163-lncRNA2	-0,178512323	2,764922404	0,750279539	0,985465812	LADA vs T2D
URS00001D0B96-lncRNA2	0,041377808	10,38627035	0,750297484	0,985465812	LADA vs T2D
URS0000907244-rRNA2	0,078056603	6,675718226	0,750810463	0,985465812	LADA vs T2D
URS0000633F75-ribozyme2	0,166805277	4,438697312	0,752051875	0,985465812	LADA vs T2D
URS00006D484A-rRNA2	0,031116442	11,67221899	0,752845127	0,985465812	LADA vs T2D
URS000064B6FC-tRNA2	-0,198342027	3,113360192	0,754726741	0,985773639	LADA vs T2D
URS00001B506A-tRNA2	0,205142937	2,259860507	0,756490644	0,985773639	LADA vs T2D
URS00006F4537-tRNA2	0,187839222	4,037892922	0,757311079	0,985773639	LADA vs T2D
URS0000701607-Y_RNA2	0,170972706	3,386959123	0,759256401	0,986029019	LADA vs T2D
URS0000381123-lncRNA2	0,171672006	2,471894131	0,760328528	0,986029019	LADA vs T2D
URS000020AD62-lncRNA2	0,161403586	1,816448524	0,761912408	0,986253283	LADA vs T2D
URS00006C2A6C-Y_RNA2	-0,146943516	10,4018413	0,767126068	0,98891787	LADA vs T2D
URS0000990012-rRNA2	-0,094898677	8,57303772	0,7696883	0,98891787	LADA vs T2D
URS0000780E15-lncRNA2	0,179250732	2,764657639	0,775696707	0,98891787	LADA vs T2D
URS000075AF5F-antisense2	0,172854486	2,789120304	0,776630705	0,98891787	LADA vs T2D
URS000091CD45-rRNA2	0,149139614	6,581914152	0,778977711	0,98891787	LADA vs T2D
URS000096E1E3-rRNA2	0,164933782	2,495902245	0,779197154	0,98891787	LADA vs T2D
URS0000038397-lncRNA2	-0,150345639	5,458381823	0,781198236	0,98891787	LADA vs T2D
URS0000395C70-lncRNA2	0,198326043	2,694736211	0,781338361	0,98891787	LADA vs T2D
URS00004E7DF9-lncRNA2	-0,133833407	4,28189465	0,783488084	0,98891787	LADA vs T2D
URS0000075AD1-lncRNA2	0,145391733	1,794828601	0,784171351	0,98891787	LADA vs T2D
URS0000391A04-lncRNA2	-0,11586922	5,482878094	0,784199363	0,98891787	LADA vs T2D
URS00002CDF5D-lncRNA2	-0,139209086	2,151438672	0,785453042	0,98891787	LADA vs T2D
URS00003C2ECD-antisense2	0,179649522	2,559377792	0,786232029	0,98891787	LADA vs T2D
URS000029A6A6-antisense2	-0,084344926	5,872434994	0,78760681	0,98891787	LADA vs T2D
URS00000E0E97-antisense2	0,229209192	4,066934958	0,790086451	0,98891787	LADA vs T2D
URS000020BB55-lncRNA2	0,166377749	3,131770633	0,795219535	0,98891787	LADA vs T2D
URS000056B231-lncRNA2	0,15125818	4,1586305	0,797920376	0,98891787	LADA vs T2D
URS00008120D6-lncRNA2	0,134920861	1,844896317	0,80121558	0,98891787	LADA vs T2D
URS0000944F10-rRNA2	0,099598631	5,261748123	0,80375253	0,98891787	LADA vs T2D
URS00001EE979-lncRNA2	-0,156169608	2,774714775	0,804010142	0,98891787	LADA vs T2D
URS00003008EB-lncRNA2	-0,118030667	4,342465078	0,806172032	0,98891787	LADA vs T2D
URS0000432B92-antisense2	-0,044760078	7,647433512	0,806293472	0,98891787	LADA vs T2D
URS00006C14B2-tRNA2	0,162597668	3,304706256	0,807283358	0,98891787	LADA vs T2D
URS0000626233-Y_RNA2	-0,09487576	4,209755669	0,807440962	0,98891787	LADA vs T2D
URS00001823EB-lncRNA2	-0,146793066	3,483455808	0,810081027	0,98891787	LADA vs T2D
URS0000A8428E-Y_RNA2	-0,189018286	5,101971983	0,810455268	0,98891787	LADA vs T2D
URS000014D40F-tRNA2	-0,166242986	3,081622422	0,816230257	0,98891787	LADA vs T2D
URS00002968BD-antisense2	-0,07406191	7,356778365	0,819074648	0,98891787	LADA vs T2D

URS000048EB2F-antisense2	-0,128622076	2,157167592	0,819716157	0,98891787	LADA vs T2D
URS0000704D22-rRNA2	-0,040817177	14,1067509	0,820920733	0,98891787	LADA vs T2D
URS00001D4EE9-tRNA2	0,147248507	2,839543714	0,825298443	0,98891787	LADA vs T2D
URS0000042FD3-lncRNA2	-0,111774984	3,024717754	0,825915137	0,98891787	LADA vs T2D
URS0000702B40-Y_RNA2	-0,143392336	2,572703997	0,826783364	0,98891787	LADA vs T2D
URS0000391360-lncRNA2	-0,129066192	2,256356542	0,828647321	0,98891787	LADA vs T2D
URS00004DDD71-lncRNA2	-0,077593639	5,854746665	0,828787495	0,98891787	LADA vs T2D
URS00005B7465-tRNA2	-0,120342371	1,847741502	0,831005773	0,98891787	LADA vs T2D
URS00004EDF08-lncRNA2	-0,089832803	4,609717254	0,832266088	0,98891787	LADA vs T2D
URS000053EAB5-lncRNA2	0,110829698	3,036408354	0,832729407	0,98891787	LADA vs T2D
URS0000997FE9-rRNA2	0,112402235	2,876398415	0,833667222	0,98891787	LADA vs T2D
URS0000177135-lncRNA2	0,172479829	3,040442935	0,834224062	0,98891787	LADA vs T2D
URS00004F6629-lncRNA2	-0,081589684	1,127896398	0,835518034	0,98891787	LADA vs T2D
URS00001C6042-antisense2	-0,117270612	1,983328915	0,836376649	0,98891787	LADA vs T2D
URS00008FEDF1-rRNA2	0,124733256	2,23767686	0,836971284	0,98891787	LADA vs T2D
URS00008B26D4-antisense2	-0,108445465	4,369068967	0,837479383	0,98891787	LADA vs T2D
URS000000513C-antisense2	0,080362445	4,840741267	0,837547751	0,98891787	LADA vs T2D
URS00006C6D0A-tRNA2	-0,118860885	3,831692982	0,838729433	0,98891787	LADA vs T2D
URS0000942121-rRNA2	0,015150229	14,48998565	0,83905085	0,98891787	LADA vs T2D
URS00000DAC34-lncRNA2	-0,112505494	2,636836099	0,839917594	0,98891787	LADA vs T2D
URS0000716B70-tRNA2	-0,101489178	3,560279134	0,843487075	0,98891787	LADA vs T2D
URS000030C934-lncRNA2	0,094858239	4,417263546	0,849042001	0,98891787	LADA vs T2D
URS000013BB40-lncRNA2	-0,106837159	2,666260296	0,852237934	0,98891787	LADA vs T2D
URS000064FE59-rRNA2	-0,024955706	11,09750364	0,855122691	0,98891787	LADA vs T2D
URS00004AC036-lncRNA2	-0,090001505	4,515610764	0,856615429	0,98891787	LADA vs T2D
URS000047A7F4-misc_RNA]2	-0,162714097	4,238929678	0,858213751	0,98891787	LADA vs T2D
URS00008119F8-lncRNA2	0,11342942	3,00844528	0,858229687	0,98891787	LADA vs T2D
URS000024383A-lncRNA2	-0,096444316	1,637698963	0,858305733	0,98891787	LADA vs T2D
URS0000502C74-tRNA2	0,031575325	14,17701257	0,85836998	0,98891787	LADA vs T2D
URS00001424D6-lncRNA2	-0,101699597	1,834670251	0,858933454	0,98891787	LADA vs T2D
URS00005C220A-antisense2	0,115504852	2,894185806	0,859195957	0,98891787	LADA vs T2D
URS00001662B7-lncRNA2	-0,116146107	2,511216373	0,859572945	0,98891787	LADA vs T2D
URS000002FD7D-lncRNA2	-0,093282528	1,677824648	0,86074479	0,98891787	LADA vs T2D
URS0000097924-lncRNA2	-0,059235667	5,60446115	0,862083403	0,98891787	LADA vs T2D
URS000034E9D0-misc_RNA]2	0,144856644	3,097582218	0,862277438	0,98891787	LADA vs T2D
URS00002CFBEA-lncRNA2	0,085206919	2,003500281	0,865749048	0,98891787	LADA vs T2D
URS00009C6137-lncRNA2	0,086512458	3,379050945	0,866135178	0,98891787	LADA vs T2D
URS0000397210-lncRNA2	0,090479441	2,372225381	0,866955097	0,98891787	LADA vs T2D
URS0000166FF8-lncRNA2	0,077199661	4,546451597	0,872692938	0,98891787	LADA vs T2D
URS00000D1D87-lncRNA2	-0,083397717	1,722539329	0,873031179	0,98891787	LADA vs T2D
URS0000006840-lncRNA2	-0,079943952	3,802540443	0,874563668	0,98891787	LADA vs T2D
URS00006AD70A-tRNA2	0,098618535	4,843339833	0,874979321	0,98891787	LADA vs T2D
URS00005F1728-lncRNA2	-0,083469638	2,661157063	0,875427005	0,98891787	LADA vs T2D
URS00007D6D04-lncRNA2	-0,078100588	3,22797681	0,876119442	0,98891787	LADA vs T2D
URS00005D36AA-lncRNA2	-0,091672353	2,523255562	0,876572368	0,98891787	LADA vs T2D
URS0000672E5A-rRNA2	-0,0563113	5,608838646	0,876689247	0,98891787	LADA vs T2D
URS000049B61C-antisense2	0,033947878	6,879627508	0,878585009	0,98891787	LADA vs T2D
URS00000A3F50-lncRNA2	0,081395371	1,749216608	0,878762579	0,98891787	LADA vs T2D
URS000091563C-rRNA2	0,017710478	10,26950568	0,880017747	0,98891787	LADA vs T2D
URS00006D4008-tRNA2	-0,102891104	2,742010752	0,881764723	0,98891787	LADA vs T2D
URS00009A050E-rRNA2	-0,025683253	10,767947	0,882655349	0,98891787	LADA vs T2D
URS000075AD80-snRNA2	0,08451549	2,124304339	0,888492332	0,98891787	LADA vs T2D
URS0000907730-rRNA2	-0,047893535	6,755248399	0,889763617	0,98891787	LADA vs T2D
URS00002AECD7-lncRNA2	-0,086536338	2,148873116	0,891912444	0,98891787	LADA vs T2D
URS000092B92B-rRNA2	-0,187614519	5,336196426	0,891917803	0,98891787	LADA vs T2D
URS00004F482C-lncRNA2	-0,078155406	2,9888845	0,892781016	0,98891787	LADA vs T2D
URS00004D7012-lncRNA2	0,095789071	2,906892949	0,893319164	0,98891787	LADA vs T2D
URS0000098211-lncRNA2	-0,080362004	2,630527968	0,895059502	0,98891787	LADA vs T2D
URS00006F7C66-rRNA2	-0,073893866	1,804743197	0,895336728	0,98891787	LADA vs T2D
URS00002F2DED-lncRNA2	0,06905658	4,602442219	0,896837966	0,98891787	LADA vs T2D
URS000097CDD0-rRNA2	-0,028846615	6,617743447	0,897248832	0,98891787	LADA vs T2D
URS000013BA50-antisense2	0,080661859	1,971579125	0,897421464	0,98891787	LADA vs T2D
URS0000543A1A-lncRNA2	-0,06633847	5,169008445	0,897448351	0,98891787	LADA vs T2D
URS00000FCDE9-misc_RNA]2	-0,092975791	3,011608195	0,899353121	0,98891787	LADA vs T2D
URS00007CB156-lncRNA2	0,02572829	7,841069642	0,899833387	0,98891787	LADA vs T2D
URS00003869EC-lncRNA2	-0,084311622	2,98234075	0,90001871	0,98891787	LADA vs T2D

URS000069E2A5-tRNA2	0,075280424	3,421985706	0,902580602	0,98891787	LADA vs T2D
URS000003B6E2-lncRNA2	-0,090141566	2,652355198	0,906019063	0,98891787	LADA vs T2D
URS000020220C-antisense2	0,065341426	2,749759905	0,906572091	0,98891787	LADA vs T2D
URS0000635088-tRNA2	-0,054798427	10,57667068	0,909703193	0,98891787	LADA vs T2D
URS00003DEE5B-lncRNA2	-0,061335256	3,256575112	0,910654445	0,98891787	LADA vs T2D
URS000024E9CC-lncRNA2	-0,064271333	2,021333988	0,914519481	0,98891787	LADA vs T2D
URS000012730D-lncRNA2	0,062785536	3,696451386	0,914678991	0,98891787	LADA vs T2D
URS00008BA2B6-lncRNA2	-0,053941486	2,826253026	0,918377305	0,98891787	LADA vs T2D
URS00006D1A54-Y_RNA2	0,052411517	3,828527713	0,919028029	0,98891787	LADA vs T2D
URS000018267A-antisense2	-0,065570299	3,83534964	0,91938953	0,98891787	LADA vs T2D
URS00002840A7-lncRNA2	0,057047088	2,142345929	0,92060248	0,98891787	LADA vs T2D
URS0000493225-tRNA2	0,028014142	7,026724216	0,920823357	0,98891787	LADA vs T2D
URS0000ABD82A-rRNA2	0,14763125	6,247493519	0,920993664	0,98891787	LADA vs T2D
URS00006ABCCE-tRNA2	0,068799756	11,64642261	0,926095244	0,98891787	LADA vs T2D
URS000014D914-antisense2	-0,044325167	4,160373223	0,928678221	0,98891787	LADA vs T2D
URS00001B6230-lncRNA2	0,047626394	2,056088304	0,930337818	0,98891787	LADA vs T2D
URS0000086FDD-antisense2	0,05865761	2,945683418	0,930911359	0,98891787	LADA vs T2D
URS0000633321-snoRNA2	-0,021436291	7,948494424	0,93150587	0,98891787	LADA vs T2D
URS000095C3C7-rRNA2	-0,038625831	4,667191645	0,932496844	0,98891787	LADA vs T2D
URS00000E0DE1-antisense2	0,045548457	3,104520142	0,932859833	0,98891787	LADA vs T2D
URS00002200B0-lncRNA2	-0,047105378	3,176288712	0,935946652	0,98891787	LADA vs T2D
URS00002AD6F3-antisense2	0,03854752	1,553235742	0,93603654	0,98891787	LADA vs T2D
URS00001F3EF6-lncRNA2	-0,054006792	2,384806636	0,936946258	0,98891787	LADA vs T2D
URS0000539731-antisense2	0,040409862	2,084997113	0,937061869	0,98891787	LADA vs T2D
URS00000734D4-lncRNA2	-0,037933895	2,320562453	0,937405921	0,98891787	LADA vs T2D
URS00005AAAF0-antisense2	0,043919464	3,447551336	0,937527841	0,98891787	LADA vs T2D
URS0000378BB8-lncRNA2	0,038502299	4,18272789	0,938415238	0,98891787	LADA vs T2D
URS00003CAB47-antisense2	-0,043891142	3,257126248	0,93981863	0,98891787	LADA vs T2D
URS00000AF0EF-lncRNA2	-0,041090039	3,804446661	0,941496858	0,98891787	LADA vs T2D
URS000069466F-Y_RNA2	-0,020353053	7,597826288	0,942814164	0,98891787	LADA vs T2D
URS00003CBFB8-lncRNA2	0,038322473	2,075423342	0,943075438	0,98891787	LADA vs T2D
URS00009A7848-rRNA2	0,008635336	11,54132926	0,944937757	0,98891787	LADA vs T2D
URS00000A7F2D-lncRNA2	-0,036524581	2,135027243	0,945087404	0,98891787	LADA vs T2D
URS00009A0DCD-rRNA2	0,029521352	5,942865971	0,945947765	0,98891787	LADA vs T2D
URS000099C20E-rRNA2	-0,043285105	3,326722503	0,946475043	0,98891787	LADA vs T2D
URS0000271FCA-antisense2	-0,028577091	5,084020409	0,949774773	0,990884427	LADA vs T2D
URS00002811B6-lncRNA2	-0,029523633	3,62513136	0,954754712	0,993246761	LADA vs T2D
URS00009C6074-antisense2	0,031492484	2,11618012	0,960613235	0,993246761	LADA vs T2D
URS0000782759-lncRNA2	-0,02682055	2,907193443	0,961029048	0,993246761	LADA vs T2D
URS0000067204-antisense2	-0,027036621	1,800108533	0,961228516	0,993246761	LADA vs T2D
URS00006744D5-tRNA2	-0,030969069	2,786516677	0,961458363	0,993246761	LADA vs T2D
URS0000249329-lncRNA2	0,023536909	1,740243504	0,963213246	0,993246761	LADA vs T2D
URS0000605748-lncRNA2	0,02661233	3,689393404	0,963254747	0,993246761	LADA vs T2D
URS000047A7F4-rRNA2	0,043538685	4,287976289	0,963406729	0,993246761	LADA vs T2D
URS00006C0715-rRNA2	-0,006141063	11,18891955	0,965846652	0,993771013	LADA vs T2D
URS00006AD81D-Y_RNA2	-0,024351747	1,867732365	0,967814203	0,993771013	LADA vs T2D
URS000057C597-antisense2	0,021033421	2,337861425	0,971292875	0,993771013	LADA vs T2D
URS00003B6188-lncRNA2	-0,018708897	3,075648025	0,971493419	0,993771013	LADA vs T2D
URS00006F3305-rRNA2	-0,003315926	11,47052611	0,974869415	0,993771013	LADA vs T2D
URS00005AF1AA-antisense2	0,013014573	1,232925367	0,975680054	0,993771013	LADA vs T2D
URS00006D1C46-snRNA2	0,013487831	6,026449126	0,977240672	0,993771013	LADA vs T2D
URS00004E5B9A-lncRNA2	0,0152984	1,8276158	0,977326426	0,993771013	LADA vs T2D
URS00000DE490-lncRNA2	-0,012367951	3,392102095	0,980380688	0,993771013	LADA vs T2D
URS00007E2F7C-lncRNA2	-0,014365936	3,279760012	0,981277175	0,993771013	LADA vs T2D
URS00003CA240-antisense2	-0,009477774	5,133383215	0,981638018	0,993771013	LADA vs T2D
URS000041043F-antisense2	-0,012882412	1,994452875	0,981752571	0,993771013	LADA vs T2D
URS000033268A-lncRNA2	-0,011174026	4,132479222	0,983750571	0,993771013	LADA vs T2D
URS0000669D0F-snRNA2	0,012276905	2,759909955	0,983819086	0,993771013	LADA vs T2D
URS0000617C6A-antisense2	0,007830296	3,572347898	0,986872989	0,994549066	LADA vs T2D
URS00001A48CC-lncRNA2	-0,008593339	2,650296854	0,987434981	0,994549066	LADA vs T2D
URS00003A00A4-lncRNA2	-0,006561337	3,039610494	0,991151317	0,996101661	LADA vs T2D
URS000002E930-antisense2	-0,006894064	4,041839683	0,991826546	0,996101661	LADA vs T2D
URS000031A1AE-antisense2	0,003801324	2,086098634	0,995118773	0,997974207	LADA vs T2D
URS0000063647-antisense2	-0,000800243	6,169603323	0,997889994	0,998745169	LADA vs T2D
URS00006EB1B5-Y_RNA2	0,000809738	4,084821132	0,998745169	0,998745169	LADA vs T2D
URS0000684E4B-tRNA3	-1,678341666	6,262078663	1,43068E-09	1,00005E-06	LADA vs Control

URS00006CE1FB-misc_RNA]3	3,125292863	6,644778412	0,001696724	0,528851437	LADA vs Control
URS00006C48EB-Y_RNA]3	2,109976114	2,788995838	0,002553968	0,528851437	LADA vs Control
URS000098FA76-rRNA3	0,952091977	2,496348799	0,003026332	0,528851437	LADA vs Control
URS00006FCBA3-Y_RNA3	-1,680231379	2,601539284	0,004566431	0,605644033	LADA vs Control
URS0000ABD8C6-rRNA3	-3,544619389	7,712316992	0,006012043	0,605644033	LADA vs Control
URS00002C9C48-lncRNA3	-1,520454713	5,358879491	0,010137819	0,605644033	LADA vs Control
URS0000A774C0-lncRNA3	-0,809519204	6,402978234	0,010885264	0,605644033	LADA vs Control
URS00006642D4-Y_RNA3	-0,51591375	4,428261145	0,012335178	0,605644033	LADA vs Control
URS0000ABD879-rRNA3	-3,195469931	7,136004534	0,013005062	0,605644033	LADA vs Control
URS00006F135B-Y_RNA3	-0,988064828	3,60198893	0,013007234	0,605644033	LADA vs Control
URS0000341866-lncRNA3	-1,552875292	4,848112839	0,01401995	0,605644033	LADA vs Control
URS00001662B7-lncRNA3	1,674790276	2,511216373	0,0145159	0,605644033	LADA vs Control
URS00006FC298-tRNA3	-1,609769317	2,919601035	0,015369195	0,605644033	LADA vs Control
URS0000A7AB58-Y_RNA3	-1,381611563	4,159115672	0,016830836	0,605644033	LADA vs Control
URS00004D4BFC-lncRNA3	1,263530035	2,209794014	0,017262628	0,605644033	LADA vs Control
URS00006B0E5A-precursor_RNA3	-1,029834059	6,734564531	0,018047479	0,605644033	LADA vs Control
URS000077A114-lncRNA3	-0,425199147	7,694058287	0,018361418	0,605644033	LADA vs Control
URS0000676AED-precursor_RNA3	-1,455657454	2,736959381	0,018661242	0,605644033	LADA vs Control
URS0000726FAB-rRNA3	0,374065714	13,91320925	0,019017842	0,605644033	LADA vs Control
URS000021BDC3-snRNA3	1,34310498	2,084780028	0,019429767	0,605644033	LADA vs Control
URS00000B8842-lncRNA3	-1,151398984	4,912142401	0,020266488	0,605644033	LADA vs Control
URS000063A7A5-Y_RNA3	-0,743609264	8,2571435	0,020377724	0,605644033	LADA vs Control
URS000003870B-lncRNA3	-0,52188387	9,957227048	0,021045802	0,605644033	LADA vs Control
URS0000249329-lncRNA3	1,232319153	1,740243504	0,021792976	0,605644033	LADA vs Control
URS000065A213-Y_RNA3	-1,148709084	10,7844197	0,023669829	0,605644033	LADA vs Control
URS000038D8D3-tRNA3	-0,746887623	7,544518208	0,025017085	0,605644033	LADA vs Control
URS000064C567-Y_RNA3	-0,569599963	5,523874961	0,02505484	0,605644033	LADA vs Control
URS000064F96B-Y_RNA3	-0,809681052	5,069805695	0,025227272	0,605644033	LADA vs Control
URS0000591DFC-lncRNA3	1,250185898	1,976731906	0,025993306	0,605644033	LADA vs Control
URS00006D4DB0-snRNA3	-1,4766663	3,391553507	0,030290306	0,682997555	LADA vs Control
URS000064B6FC-tRNA3	1,438989012	3,113360192	0,031624345	0,690794281	LADA vs Control
URS000031A1AE-antisense3	1,413323379	2,086098634	0,033826098	0,698376799	LADA vs Control
URS0000389FBF-lncRNA3	-1,238403473	2,547635864	0,034229462	0,698376799	LADA vs Control
URS00008C3E41-lncRNA3	0,940205702	5,384577661	0,034968795	0,698376799	LADA vs Control
URS0000996BBC-rRNA3	-0,41718867	8,622403976	0,036271378	0,70426925	LADA vs Control
URS000066C003-Y_RNA]3	-2,506215779	5,557417709	0,038561274	0,728495414	LADA vs Control
URS0000811AFB-antisense3	1,181035456	2,040750323	0,045112711	0,751233253	LADA vs Control
URS000096EEE8-rRNA3	0,279611107	8,595030803	0,045898896	0,751233253	LADA vs Control
URS000014D914-antisense3	-1,049972805	4,160373223	0,046497439	0,751233253	LADA vs Control
URS000072C165-Y_RNA3	-0,555135487	5,305209475	0,047301926	0,751233253	LADA vs Control
URS0000028D07-lncRNA3	1,086172401	5,403821621	0,047772619	0,751233253	LADA vs Control
URS000022DD4A-tRNA3	-0,482163543	9,64520168	0,051691747	0,751233253	LADA vs Control
URS0000676525-Y_RNA3	-1,057964072	4,329395557	0,052561276	0,751233253	LADA vs Control
URS000013B42D-tRNA3	-0,700623927	16,60649921	0,05397467	0,751233253	LADA vs Control
URS00000D1D87-lncRNA3	1,053482451	1,722539329	0,054717105	0,751233253	LADA vs Control
URS00006CBBB9-Y_RNA3	-1,190091235	3,554703445	0,055474	0,751233253	LADA vs Control
URS00009C606C-lncRNA3	-0,726766578	5,246519845	0,055503623	0,751233253	LADA vs Control
URS000061F57C-tRNA3	-0,99336453	4,675906559	0,057948613	0,751233253	LADA vs Control
URS00000DA56A-lncRNA3	1,174428035	2,589490843	0,058428975	0,751233253	LADA vs Control
URS00007CA557-lncRNA3	-0,480275378	9,085280894	0,059175592	0,751233253	LADA vs Control
URS000009DDCA-tRNA3	-0,537588964	8,707527281	0,059415483	0,751233253	LADA vs Control
URS0000100BA4-antisense3	-1,161661801	1,836907151	0,059455024	0,751233253	LADA vs Control
URS000070E3CE-rRNA3	1,051961479	4,081177344	0,060088057	0,751233253	LADA vs Control
URS00006B33E0-Y_RNA3	-0,824641181	14,2625662	0,060897406	0,751233253	LADA vs Control
URS00000A3F50-lncRNA3	1,065400128	1,749216608	0,063125512	0,751233253	LADA vs Control
URS0000995AA3-rRNA3	-0,653502524	8,311403687	0,063668534	0,751233253	LADA vs Control
URS0000617C6A-antisense3	0,948612065	3,572347898	0,064802015	0,751233253	LADA vs Control
URS0000716B70-tRNA3	1,013119112	3,560279134	0,065104266	0,751233253	LADA vs Control
URS000060D3B6-lncRNA3	-1,45802606	4,588963786	0,065656912	0,751233253	LADA vs Control
URS00006F5B12-Y_RNA3	-1,148263001	5,757189507	0,066628018	0,751233253	LADA vs Control
URS0000005270-rRNA3	-1,735903307	6,166410111	0,069368087	0,751233253	LADA vs Control
URS00009A5DA8-rRNA3	0,863036618	5,880019281	0,070373342	0,751233253	LADA vs Control
URS0000766C83-lncRNA3	1,216954843	2,482009239	0,071193659	0,751233253	LADA vs Control
URS000034E03C-antisense3	1,101234271	2,078398724	0,071790905	0,751233253	LADA vs Control
URS00001E6C0A-lncRNA3	1,026212661	3,103912065	0,071865488	0,751233253	LADA vs Control
URS0000A7BA37-lncRNA3	1,025918501	1,699099075	0,072006621	0,751233253	LADA vs Control

URS00006A2BF7-Y_RNA3	-1,127705577	10,1864015	0,076615467	0,767564828	LADA vs Control
URS00007BE6D3-lncRNA3	2,605079688	10,22836282	0,077181489	0,767564828	LADA vs Control
URS00007D61AD-antisense3	1,381741007	2,769938297	0,077859252	0,767564828	LADA vs Control
URS00000B6370-lncRNA3	-0,839575455	3,6717613	0,080199214	0,767564828	LADA vs Control
URS00003F2CFE-lncRNA3	-1,022426379	4,520438351	0,082045097	0,767564828	LADA vs Control
URS0000204428-lncRNA3	-1,098578264	2,674128924	0,083217451	0,767564828	LADA vs Control
URS0000907244-rRNA3	-0,452445412	6,675718226	0,083643493	0,767564828	LADA vs Control
URS0000A90D33-snRNA3	1,16627461	3,877391031	0,08481065	0,767564828	LADA vs Control
URS000097171C-SRP_RNA3	1,001062675	2,924722071	0,084986787	0,767564828	LADA vs Control
URS00006C0413-Y_RNA3	-1,08067813	2,155330761	0,088438031	0,767564828	LADA vs Control
URS000066AF0D-Y_RNA3	-1,00995798	12,73751355	0,089009044	0,767564828	LADA vs Control
URS000018BCE5-antisense3	-1,030444248	2,555076799	0,09007533	0,767564828	LADA vs Control
URS000063FB43-tRNA3	-0,348425913	12,66119095	0,092039057	0,767564828	LADA vs Control
URS000002E930-antisense3	-1,190194365	4,041839683	0,094492909	0,767564828	LADA vs Control
URS00004BE455-lncRNA3	-0,749932492	5,228003516	0,095186724	0,767564828	LADA vs Control
URS00006F4E76-Y_RNA3	0,47359455	0,766333934	0,095818432	0,767564828	LADA vs Control
URS0000664EAD-tRNA3	1,132903226	2,046606184	0,09595428	0,767564828	LADA vs Control
URS0000478C87-lncRNA3	0,963120729	2,830369346	0,096244236	0,767564828	LADA vs Control
URS000005F65D-antisense3	0,909383084	4,947813537	0,09780243	0,767564828	LADA vs Control
URS000011812A-antisense3	-0,851947332	4,132007293	0,097838374	0,767564828	LADA vs Control
URS00003AA49D-lncRNA3	-0,978717783	2,72551685	0,098423034	0,767564828	LADA vs Control
URS0000644222-tRNA3	-0,395902252	7,91366141	0,099572884	0,767564828	LADA vs Control
URS00000CCACD-antisense3	1,24887218	4,642488599	0,101552945	0,767564828	LADA vs Control
URS0000446770-snoRNA3	-1,062651644	2,104005039	0,102895342	0,767564828	LADA vs Control
URS00000E9A71-lncRNA3	0,949490984	3,823249127	0,103440359	0,767564828	LADA vs Control
URS0000399BDA-lncRNA3	-0,941471383	5,117165985	0,10440562	0,767564828	LADA vs Control
URS00004227BE-lncRNA3	-1,007537605	1,875905236	0,105481403	0,767564828	LADA vs Control
URS00006A3E7F-snRNA3	-0,330292394	8,935478024	0,105915812	0,767564828	LADA vs Control
URS00003008EB-lncRNA3	-0,821635839	4,342465078	0,106693523	0,767564828	LADA vs Control
URS000006044C-lncRNA3	-0,920107946	1,808588154	0,106948745	0,767564828	LADA vs Control
URS00003B6188-lncRNA3	0,900702268	3,075648025	0,107954521	0,767564828	LADA vs Control
URS000063E4FD-tRNA3	1,049479956	3,061724682	0,109670748	0,767564828	LADA vs Control
URS0000AA0C30-lncRNA3	-0,931737335	3,233082441	0,109808988	0,767564828	LADA vs Control
URS0000527686-lncRNA3	0,662059018	1,162280834	0,112043075	0,774499736	LADA vs Control
URS0000629ECF-Y_RNA3	-0,624256623	4,37154222	0,113017129	0,774499736	LADA vs Control
URS00006D74B2-tRNA3	-0,772649409	4,411009625	0,115113718	0,778629768	LADA vs Control
URS00001A48CC-lncRNA3	0,918326924	2,650296854	0,115847634	0,778629768	LADA vs Control
URS00001424D6-lncRNA3	0,960844496	1,834670251	0,118870884	0,778676149	LADA vs Control
URS000075B143-lncRNA3	-0,905454313	2,661955926	0,118999371	0,778676149	LADA vs Control
URS0000727FD6-tRNA3	0,990771705	3,320399515	0,119926562	0,778676149	LADA vs Control
URS00005B6FC3-lncRNA3	0,708220517	4,468244677	0,121375955	0,778676149	LADA vs Control
URS0000682FB0-Y_RNA3	-0,690906109	10,97882695	0,121424464	0,778676149	LADA vs Control
URS000062C4DE-tRNA3	0,936634904	3,676191563	0,125766253	0,789063565	LADA vs Control
URS00003A00A4-lncRNA3	0,956444159	3,039610494	0,128039519	0,789063565	LADA vs Control
URS0000397210-lncRNA3	0,877153375	2,372225381	0,12817392	0,789063565	LADA vs Control
URS000071DF37-tRNA3	-1,069113039	2,691836943	0,129308668	0,789063565	LADA vs Control
URS0000371842-lncRNA3	0,921786583	2,020987369	0,129334465	0,789063565	LADA vs Control
URS000050E9EC-lncRNA3	-0,831622708	3,382080638	0,129817325	0,789063565	LADA vs Control
URS00001A4293-lncRNA3	-0,793430513	4,616590583	0,130972684	0,789152611	LADA vs Control
URS00005CEC24-lncRNA3	0,974896572	2,918669315	0,132089922	0,789152611	LADA vs Control
URS00005FFC78-lncRNA3	0,915268242	2,519542247	0,133934386	0,793390979	LADA vs Control
URS0000812136-lncRNA3	-0,350533773	9,121672555	0,135548044	0,796202376	LADA vs Control
URS00000F6ECB-lncRNA3	-0,597787347	5,128846246	0,138424456	0,801923803	LADA vs Control
URS000044BAE3-tRNA3	0,333350737	7,695683129	0,138816567	0,801923803	LADA vs Control
URS000011BDAF-antisense3	0,811532493	2,89470373	0,144149092	0,825903407	LADA vs Control
URS00009554B1-rRNA3	-0,797943308	4,916492139	0,146911463	0,834887094	LADA vs Control
URS000075BB81-lncRNA3	0,843012	2,83062659	0,148569563	0,837501004	LADA vs Control
URS00005D36AA-lncRNA3	-0,899223176	2,523255562	0,149788938	0,837619742	LADA vs Control
URS00004C6AFE-antisense3	0,638219841	5,444648432	0,151950886	0,838516678	LADA vs Control
URS000022006F-lncRNA3	-0,834795476	3,198783691	0,152910371	0,838516678	LADA vs Control
URS00002AD8DA-lncRNA3	0,798327951	2,376170134	0,153548118	0,838516678	LADA vs Control
URS0000696377-Y_RNA3	0,840625718	2,848796511	0,157880006	0,84754287	LADA vs Control
URS000097B164-rRNA3	0,647164731	5,947136128	0,158601743	0,84754287	LADA vs Control
URS00000AD390-antisense3	0,819905842	1,744059197	0,158838506	0,84754287	LADA vs Control
URS0000931B54-rRNA3	0,117427636	14,46696978	0,160288016	0,848797901	LADA vs Control
URS0000776086-lncRNA3	0,544107864	7,320712579	0,163409039	0,858818934	LADA vs Control

URS00002E81F5-lncRNA3	0,737587329	4,084000104	0,167790968	0,861389594	LADA vs Control
URS0000067204-antisense3	0,831936269	1,800108533	0,168804572	0,861389594	LADA vs Control
URS000065D78F-Y_RNA3	-0,256054876	5,875001903	0,170292543	0,861389594	LADA vs Control
URS00004DDD71-lncRNA3	-0,51962955	5,854746665	0,170752869	0,861389594	LADA vs Control
URS00000E0E97-antisense3	1,287544435	4,066934958	0,171183254	0,861389594	LADA vs Control
URS000052A1C9-tRNA3	-0,750884858	4,427113529	0,171292065	0,861389594	LADA vs Control
URS0000944F10-rRNA3	-0,582779868	5,261748123	0,17278883	0,862558694	LADA vs Control
URS0000422302-lncRNA3	0,760355145	2,914915679	0,17425682	0,862558694	LADA vs Control
URS000075AD80-snRNA3	0,868517552	2,124304339	0,175226516	0,862558694	LADA vs Control
URS0000A76F22-lncRNA3	0,798093597	2,591384678	0,177147652	0,865872047	LADA vs Control
URS0000699390-Y_RNA3	-0,668398801	13,62373063	0,178872008	0,865872047	LADA vs Control
URS0000A81496-Y_RNA3	0,941405656	2,190786251	0,182259143	0,865872047	LADA vs Control
URS00002D40C8-tRNA3	-0,34884848	7,830242615	0,182692695	0,865872047	LADA vs Control
URS000047AF1F-lncRNA3	1,260109566	4,480968908	0,183358294	0,865872047	LADA vs Control
URS000075D28F-lncRNA3	-0,794269541	2,277607756	0,184629122	0,865872047	LADA vs Control
URS00004D1520-lncRNA3	0,811490153	3,203873127	0,185351678	0,865872047	LADA vs Control
URS0000610FFE-tRNA3	-0,787911868	2,353369293	0,185809452	0,865872047	LADA vs Control
URS0000282AB2-tRNA3	1,101440461	4,591558308	0,188862504	0,867100105	LADA vs Control
URS00004A7F9B-antisense3	-0,349761247	6,391504235	0,192046249	0,867100105	LADA vs Control
URS00002EA13A-lncRNA3	-0,765889569	2,908290455	0,192855839	0,867100105	LADA vs Control
URS00006D23E9-snRNA3	-0,769951081	3,580811892	0,194361496	0,867100105	LADA vs Control
URS00005B6D58-lncRNA3	0,674787383	5,324374408	0,196820966	0,867100105	LADA vs Control
URS0000641C1B-Y_RNA3	-0,433973508	4,028164364	0,197066284	0,867100105	LADA vs Control
URS0000667737-rRNA3	1,662363702	4,834926248	0,198982116	0,867100105	LADA vs Control
URS00009A050E-rRNA3	0,240680288	10,767947	0,199240509	0,867100105	LADA vs Control
URS00006BFB96-tRNA3	-0,795792324	2,209793928	0,20003137	0,867100105	LADA vs Control
URS00006A9AE8-Y_RNA3	-0,98873497	5,946221857	0,200746818	0,867100105	LADA vs Control
URS000063455F-rRNA3	-0,755017587	2,601127664	0,202477339	0,867100105	LADA vs Control
URS00006D56C0-Y_RNA3	-0,536043261	4,210228585	0,207236234	0,867100105	LADA vs Control
URS00009290F1-rRNA3	0,733071922	1,980245991	0,207887985	0,867100105	LADA vs Control
URS00003F5471-lncRNA3	0,703338097	3,0793045	0,209924815	0,867100105	LADA vs Control
URS00000540AC-antisense3	-0,747730622	3,451834854	0,210245886	0,867100105	LADA vs Control
URS00004199FB-antisense3	-0,876640797	3,00203969	0,210376419	0,867100105	LADA vs Control
URS0000188F7D-scRNA3	2,079278498	6,577829264	0,210953331	0,867100105	LADA vs Control
URS000053D4AB-snoRNA3	-0,726604659	1,689585927	0,211786377	0,867100105	LADA vs Control
URS00006CE1FB-rRNA3	1,329870371	5,102179845	0,211853247	0,867100105	LADA vs Control
URS000068089E-Y_RNA3	0,785190226	2,479289353	0,213206926	0,867100105	LADA vs Control
URS0000515855-lncRNA3	-0,685344396	2,502117237	0,21416962	0,867100105	LADA vs Control
URS0000677B31-tRNA3	0,555625404	7,303130994	0,214190871	0,867100105	LADA vs Control
URS000018267A-antisense3	0,864197128	3,83534964	0,21548153	0,867100105	LADA vs Control
URS00006C6D0A-tRNA3	-0,771294575	3,831692982	0,215844661	0,867100105	LADA vs Control
URS0000031963-antisense3	-0,914095369	3,414756941	0,218074039	0,871050018	LADA vs Control
URS0000633F75-ribozyme3	0,694318261	4,438697312	0,221581797	0,877645528	LADA vs Control
URS000094F5D5-rRNA3	-0,601568869	7,000982595	0,223436628	0,877645528	LADA vs Control
URS000075D353-lncRNA3	0,680026952	1,653940152	0,223491994	0,877645528	LADA vs Control
URS00004C5EDB-lncRNA3	0,80914316	2,83469135	0,224748457	0,877649003	LADA vs Control
URS00001AF592-antisense3	-0,792149667	3,230887811	0,231138668	0,892044593	LADA vs Control
URS00000D6053-lncRNA3	-1,048329392	5,001351593	0,234711412	0,892044593	LADA vs Control
URS00001D0305-lncRNA3	-0,228777428	7,373693454	0,234882058	0,892044593	LADA vs Control
URS00003CAB47-antisense3	-0,726172383	3,257126248	0,235075877	0,892044593	LADA vs Control
URS00006729E8-Y_RNA3	-0,383917992	0,88430075	0,235994621	0,892044593	LADA vs Control
URS000034AAC2-tRNA3	0,682508997	4,895139042	0,236484381	0,892044593	LADA vs Control
URS000056B231-lncRNA3	0,756998951	4,1586305	0,237368089	0,892044593	LADA vs Control
URS00006C9A71-Y_RNA3	0,638331601	2,255341052	0,239775487	0,89293076	LADA vs Control
URS000075BA00-lncRNA3	-1,58822745	7,953608312	0,240158774	0,89293076	LADA vs Control
URS000051CFB0-lncRNA3	-1,120066105	3,801776289	0,243322582	0,896333641	LADA vs Control
URS000075A564-lncRNA3	-0,705601252	2,096352205	0,243638615	0,896333641	LADA vs Control
URS000065DC2A-tRNA3	-0,521491185	4,471494404	0,245278662	0,896779292	LADA vs Control
URS000099C20E-rRNA3	0,804749511	3,326722503	0,247679299	0,896779292	LADA vs Control
URS00003870EC-lncRNA3	-0,768677062	2,283625175	0,24824068	0,896779292	LADA vs Control
URS00001A72CE-tRNA3	-0,704874775	4,122188898	0,249286162	0,896779292	LADA vs Control
URS00007D436A-antisense3	-0,671402153	2,239346559	0,250775942	0,896779292	LADA vs Control
URS00001DBD56-lncRNA3	-0,609089144	3,720321434	0,25146788	0,896779292	LADA vs Control
URS000075AC32-lncRNA3	-0,729619726	2,916319528	0,253477756	0,896779292	LADA vs Control
URS0000561169-lncRNA3	0,684780091	3,951068111	0,254023319	0,896779292	LADA vs Control
URS000042199A-lncRNA3	0,757010898	2,107510848	0,255647139	0,897952749	LADA vs Control

URS000038D781-lncRNA3	0,732471144	2,120835096	0,257442881	0,897952749	LADA vs Control
URS00000FCDE9-misc_RNA]3	-0,862957152	3,011608195	0,260859637	0,897952749	LADA vs Control
URS00004F6629-lncRNA3	0,472934039	1,127896398	0,261414519	0,897952749	LADA vs Control
URS00003A72DC-antisense3	0,647951043	3,084869652	0,265035467	0,897952749	LADA vs Control
URS000062C73B-tRNA3	-0,512472337	4,544224584	0,268846166	0,897952749	LADA vs Control
URS00006F7C66-rRNA3	-0,66809755	1,804743197	0,268892681	0,897952749	LADA vs Control
URS000093FF31-rRNA3	0,62627794	2,038384139	0,269148444	0,897952749	LADA vs Control
URS00002D2D8F-misc_RNA]3	-0,73997967	1,939760416	0,269393606	0,897952749	LADA vs Control
URS0000462D45-lncRNA3	0,601299642	3,263068544	0,274754893	0,897952749	LADA vs Control
URS0000955796-rRNA3	0,134686087	10,7330721	0,275550269	0,897952749	LADA vs Control
URS00006CB0C3-Y_RNA3	-0,277116478	5,291889769	0,275660202	0,897952749	LADA vs Control
URS00004F0321-tRNA3	-0,354221237	14,08130095	0,277913708	0,897952749	LADA vs Control
URS0000415026-tRNA3	0,526743026	4,426745558	0,279124276	0,897952749	LADA vs Control
URS0000397495-antisense3	-0,395257389	5,990008067	0,279198601	0,897952749	LADA vs Control
URS000047AE74-lncRNA3	0,65965828	2,522838605	0,279435605	0,897952749	LADA vs Control
URS00002598CF-lncRNA3	-0,567045552	4,630900569	0,280141175	0,897952749	LADA vs Control
URS00002CC2A4-antisense3	-0,656094092	2,224906264	0,282345626	0,897952749	LADA vs Control
URS00005BE013-lncRNA3	0,827960787	2,759573682	0,283795046	0,897952749	LADA vs Control
URS000033F395-lncRNA3	-0,628742263	1,897833694	0,284627589	0,897952749	LADA vs Control
URS00000D8A7A-lncRNA3	0,627456801	2,120449279	0,284900985	0,897952749	LADA vs Control
URS0000640661-Y_RNA3	-0,54743282	11,13979054	0,284945042	0,897952749	LADA vs Control
URS0000417A0F-tRNA3	-0,474725107	7,465949428	0,285968118	0,897952749	LADA vs Control
URS000019B78E-tRNA3	-0,950257655	3,458896569	0,288757812	0,897952749	LADA vs Control
URS00008119F8-lncRNA3	-0,712145534	3,00844528	0,289053757	0,897952749	LADA vs Control
URS0000418239-snRNA3	0,574114132	2,184896985	0,289788261	0,897952749	LADA vs Control
URS0000192C05-lncRNA3	-0,608504505	2,748839188	0,289965303	0,897952749	LADA vs Control
URS000067843B-Y_RNA3	-0,444814675	5,925250166	0,291727592	0,897952749	LADA vs Control
URS00002A28BF-lncRNA3	-0,728825031	2,823053274	0,292827334	0,897952749	LADA vs Control
URS000013BA50-antisense3	0,694449702	1,971579125	0,29381459	0,897952749	LADA vs Control
URS000038F4B2-tRNA3	-0,685501198	2,156583414	0,294179084	0,897952749	LADA vs Control
URS00005BC8EC-lncRNA3	-0,842544734	5,143820106	0,299039433	0,905391333	LADA vs Control
URS00002D33E9-lncRNA3	-0,59386937	2,231641646	0,299206578	0,905391333	LADA vs Control
URS00006BBD5F-Y_RNA3	-0,630975243	3,385968133	0,301925039	0,907702445	LADA vs Control
URS00001823EB-lncRNA3	-0,67583461	3,483455808	0,302567482	0,907702445	LADA vs Control
URS00001E15E2-lncRNA3	0,558469414	2,285605947	0,304695205	0,908478163	LADA vs Control
URS0000543A1A-lncRNA3	-0,560320903	5,169008445	0,305705139	0,908478163	LADA vs Control
URS0000ABD7E8-rRNA3	-1,002330384	10,98616314	0,307929596	0,908478163	LADA vs Control
URS000002FD7D-lncRNA3	0,573614575	1,677824648	0,308024785	0,908478163	LADA vs Control
URS000052A6A6-lncRNA3	-0,576365642	3,111397823	0,309366292	0,908601001	LADA vs Control
URS000069ED7F-Y_RNA3	-0,625762815	4,579144673	0,31117573	0,910091361	LADA vs Control
URS000097CDD0-rRNA3	0,24120919	6,617743447	0,3125993	0,91044546	LADA vs Control
URS00006C246E-snRNA3	0,687317486	4,016094545	0,317286714	0,917714916	LADA vs Control
URS0000704D22-rRNA3	0,194200731	14,1067509	0,317721044	0,917714916	LADA vs Control
URS00000734D4-lncRNA3	-0,514379809	2,320562453	0,31919038	0,918164919	LADA vs Control
URS0000042FD3-lncRNA3	-0,533745397	3,024717754	0,321574942	0,921233133	LADA vs Control
URS0000ABD7D5-rRNA3	0,098172568	13,17747017	0,326035854	0,926781409	LADA vs Control
URS00000A586F-lncRNA3	-0,334321436	0,916847513	0,326705068	0,926781409	LADA vs Control
URS000012C80D-lncRNA3	-0,576757865	2,726619231	0,328301465	0,926781409	LADA vs Control
URS00006F3305-rRNA3	-0,109808489	11,47052611	0,331063717	0,926781409	LADA vs Control
URS000047C79B-tRNA3	0,169625625	8,413478971	0,333460077	0,926781409	LADA vs Control
URS000064506B-tRNA3	0,657942242	2,909810805	0,335704907	0,926781409	LADA vs Control
URS00002FBC9E-lncRNA3	-0,630710461	3,539604284	0,336394583	0,926781409	LADA vs Control
URS0000AAB7F4-lncRNA3	-0,569576097	3,962630959	0,336455603	0,926781409	LADA vs Control
URS00009407E6-rRNA3	-0,617043391	2,030446175	0,338835654	0,926781409	LADA vs Control
URS000048B807-lncRNA3	-0,511448661	4,663045601	0,339979723	0,926781409	LADA vs Control
URS0000271FCA-antisense3	-0,460954903	5,084020409	0,343217903	0,926781409	LADA vs Control
URS00006C2A6C-Y_RNA3	-0,510261701	10,4018413	0,344723599	0,926781409	LADA vs Control
URS0000038B1E-antisense3	0,532636975	3,244363175	0,345172707	0,926781409	LADA vs Control
URS000040CE64-lncRNA3	0,666280353	2,883976268	0,345201936	0,926781409	LADA vs Control
URS00007D6D04-lncRNA3	-0,505354788	3,22797681	0,346172306	0,926781409	LADA vs Control
URS00003D60C0-lncRNA3	0,543369092	1,735581371	0,347656302	0,926781409	LADA vs Control
URS0000233E9C-lncRNA3	-0,76177416	2,822247929	0,349005648	0,926781409	LADA vs Control
URS00006C8EDF-tRNA3	0,607878999	3,590220694	0,349169181	0,926781409	LADA vs Control
URS0000432B92-antisense3	-0,18283001	7,647433512	0,349508129	0,926781409	LADA vs Control
URS000053EAB5-lncRNA3	-0,526721826	3,036408354	0,350305855	0,926781409	LADA vs Control
URS000070792C-rRNA3	0,551162254	1,756278837	0,351396163	0,926781409	LADA vs Control

URS000022CCD7-lncRNA3	0,27322445	6,400235397	0,352680765	0,926781409	LADA vs Control
URS00007E2F7C-lncRNA3	-0,591683519	3,279760012	0,36019004	0,942969431	LADA vs Control
URS000045FD15-lncRNA3	-0,518571459	4,004446293	0,365062927	0,950360334	LADA vs Control
URS0000022477-lncRNA3	-0,537933119	1,898475606	0,366332721	0,950360334	LADA vs Control
URS00002064F6-tRNA3	-0,566648742	7,265714406	0,367704106	0,950360334	LADA vs Control
URS000058CCC6-antisense3	-0,537696779	2,503982133	0,369488406	0,950360334	LADA vs Control
URS0000006840-lncRNA3	0,483848323	3,802540443	0,371865153	0,950360334	LADA vs Control
URS00001DE9CD-antisense3	0,559693165	3,351892383	0,376615188	0,950360334	LADA vs Control
URS000051EF4B-lncRNA3	0,520235444	1,97882489	0,378027333	0,950360334	LADA vs Control
URS000068483A-rRNA3	0,560836025	2,397770416	0,378484825	0,950360334	LADA vs Control
URS00001B6230-lncRNA3	-0,514069152	2,056088304	0,378863666	0,950360334	LADA vs Control
URS000064FE59-rRNA3	0,129237491	11,09750364	0,379054095	0,950360334	LADA vs Control
URS0000AAD5AA-lncRNA3	0,752458218	10,83600588	0,379967143	0,950360334	LADA vs Control
URS00004B48CD-lncRNA3	0,525872639	1,80838911	0,381213509	0,950360334	LADA vs Control
URS000029CCC5-tRNA3	0,509417446	2,732517708	0,382521463	0,950360334	LADA vs Control
URS0000AAECF4-lncRNA3	-0,423991131	3,702161679	0,382968942	0,950360334	LADA vs Control
URS000017CF23-antisense3	-0,505304664	2,666340903	0,38736192	0,950360334	LADA vs Control
URS0000A7F61B-lncRNA3	-0,493923745	2,242573569	0,387815795	0,950360334	LADA vs Control
URS00002C2371-lncRNA3	-0,496923659	3,691720987	0,388962151	0,950360334	LADA vs Control
URS00009612D1-rRNA3	-0,392761603	5,646389432	0,390796787	0,950360334	LADA vs Control
URS00006D1735-snRNA3	-0,439524496	5,596569208	0,392435298	0,950360334	LADA vs Control
URS00001DFAE4-lncRNA3	0,634097224	3,289114858	0,392622362	0,950360334	LADA vs Control
URS00006952C9-Y_RNA3	-0,641204491	4,013121466	0,394105129	0,950360334	LADA vs Control
URS0000537899-antisense3	-0,520863746	2,143848964	0,394319171	0,950360334	LADA vs Control
URS00002E367B-antisense3	0,504150318	1,915955023	0,396311992	0,950360334	LADA vs Control
URS0000086FDD-antisense3	0,610440708	2,945683418	0,397920559	0,950360334	LADA vs Control
URS000033268A-lncRNA3	0,491673102	4,132479222	0,398164803	0,950360334	LADA vs Control
URS000096196A-SRP_RNA3	0,494854461	2,043342413	0,401527729	0,950360334	LADA vs Control
URS0000784C7B-lncRNA3	0,507387964	2,086288772	0,402501586	0,950360334	LADA vs Control
URS0000543B4D-lncRNA3	0,53490668	2,605909076	0,403034417	0,950360334	LADA vs Control
URS00007080E9-Y_RNA3	-0,65623778	2,936384424	0,403467388	0,950360334	LADA vs Control
URS00003869EC-lncRNA3	0,597272391	2,98234075	0,403801172	0,950360334	LADA vs Control
URS0000669D0F-snRNA3	0,53683415	2,759909955	0,405874887	0,950871838	LADA vs Control
URS0000515429-lncRNA3	0,330133424	7,164323775	0,40673917	0,950871838	LADA vs Control
URS00005B5F85-lncRNA3	0,476087595	5,187781526	0,410662962	0,951732368	LADA vs Control
URS00006AE6F1-Y_RNA3	-0,387501357	2,11506442	0,410897389	0,951732368	LADA vs Control
URS0000717DFB-Y_RNA3	-0,457384739	2,95584216	0,41211252	0,951732368	LADA vs Control
URS00005F1728-lncRNA3	0,461420257	2,661157063	0,415816314	0,951732368	LADA vs Control
URS000047EBB5-tRNA3	-0,557745869	2,438958701	0,416411038	0,951732368	LADA vs Control
URS0000672E5A-rRNA3	0,310940017	5,608838646	0,421489461	0,951732368	LADA vs Control
URS000003B6E2-lncRNA3	0,65466119	2,652355198	0,422666195	0,951732368	LADA vs Control
URS0000AABA8B-lncRNA3	-0,216520932	9,325488704	0,422692688	0,951732368	LADA vs Control
URS00002D0015-lncRNA3	-0,440122574	3,226559685	0,422866444	0,951732368	LADA vs Control
URS0000120E41-tRNA3	0,272808335	6,378757577	0,423036662	0,951732368	LADA vs Control
URS000063A6E6-rRNA3	0,100846802	11,70425189	0,423064779	0,951732368	LADA vs Control
URS00009C6137-lncRNA3	0,437908478	3,379050945	0,423446018	0,951732368	LADA vs Control
URS000076DAC1-lncRNA3	-0,349682951	5,661423845	0,431276384	0,958117149	LADA vs Control
URS00006BB04D-tRNA3	0,47051163	3,054494403	0,432133826	0,958117149	LADA vs Control
URS00006E23A8-rRNA3	-0,530380865	2,840982885	0,434097045	0,958117149	LADA vs Control
URS00000AD0C2-lncRNA3	0,457554191	1,982015887	0,434477302	0,958117149	LADA vs Control
URS0000702B40-Y_RNA3	0,553675758	2,572703997	0,435492975	0,958117149	LADA vs Control
URS000024E9CC-lncRNA3	-0,498785958	2,021333988	0,436559397	0,958117149	LADA vs Control
URS00003EAC96-antisense3	0,510977514	2,99991317	0,436705589	0,958117149	LADA vs Control
URS0000A827F4-antisense3	0,530872201	8,224586053	0,437720061	0,958117149	LADA vs Control
URS000032B6B6-snRNA3	0,174483172	8,295722512	0,438623015	0,958117149	LADA vs Control
URS000030BAD5-tRNA3	-0,217477194	9,963231125	0,440295583	0,958774494	LADA vs Control
URS00009C6070-lncRNA3	-0,425462996	2,53934864	0,443791462	0,961009331	LADA vs Control
URS000075DF54-lncRNA3	-0,269955455	6,14755677	0,444071551	0,961009331	LADA vs Control
URS00004AE46A-lncRNA3	-0,449212823	3,574152246	0,446567951	0,963429005	LADA vs Control
URS00000081EA-snRNA3	-0,467625952	2,66468092	0,44824668	0,964075167	LADA vs Control
URS0000974435-SRP_RNA3	0,415271544	2,886817066	0,453229221	0,967250135	LADA vs Control
URS000042F13F-tRNA3	0,221758435	6,573577673	0,453692517	0,967250135	LADA vs Control
URS0000994031-rRNA3	-0,119102618	11,10957664	0,45441249	0,967250135	LADA vs Control
URS0000196BD3-lncRNA3	0,438146678	2,056335374	0,455324865	0,967250135	LADA vs Control
URS000039557B-antisense3	0,424789373	1,694045712	0,458159495	0,967250135	LADA vs Control
URS00006271F5-Y_RNA3	-0,350995939	3,66489103	0,460185532	0,967250135	LADA vs Control

URS00002EDD28-lncRNA3	-0,292721552	5,436899358	0,463646522	0,967250135	LADA vs Control
URS000071E736-Y_RNA3	-0,481096962	2,593633412	0,464380327	0,967250135	LADA vs Control
URS0000637E4A-tRNA3	-0,42519152	1,552702938	0,466028599	0,967250135	LADA vs Control
URS0000016595-lncRNA3	-0,452319772	2,839797195	0,47498538	0,967250135	LADA vs Control
URS0000ABD7E9-lncRNA3	0,453799535	3,512762788	0,475483131	0,967250135	LADA vs Control
URS0000493225-tRNA3	-0,213618529	7,026724216	0,475705841	0,967250135	LADA vs Control
URS0000626233-Y_RNA3	-0,29565736	4,209755669	0,476003704	0,967250135	LADA vs Control
URS000037D0FB-tRNA3	-0,512219435	2,792154767	0,477956104	0,967250135	LADA vs Control
URS00009C6042-lncRNA3	-0,221436697	6,706154397	0,479834113	0,967250135	LADA vs Control
URS00006C0715-rRNA3	0,109127716	11,18891955	0,479836557	0,967250135	LADA vs Control
URS0000633321-snoRNA3	0,187020219	7,948494424	0,480814001	0,967250135	LADA vs Control
URS000017D264-lncRNA3	-0,433605037	2,092743225	0,482129485	0,967250135	LADA vs Control
URS0000395C70-lncRNA3	-0,53112096	2,694736211	0,483533185	0,967250135	LADA vs Control
URS0000488EA7-lncRNA3	-0,441110985	2,312667922	0,487923664	0,967250135	LADA vs Control
URS0000166FF8-lncRNA3	-0,347725618	4,546451597	0,49416978	0,967250135	LADA vs Control
URS000049B61C-antisense3	0,161594229	6,879627508	0,496068948	0,967250135	LADA vs Control
URS00002B7986-antisense3	-0,380305183	1,782132427	0,496586471	0,967250135	LADA vs Control
URS00007062F7-Y_RNA3	-0,280634863	4,160857713	0,496772461	0,967250135	LADA vs Control
URS00005580B2-lncRNA3	-0,475435193	3,442360628	0,499024186	0,967250135	LADA vs Control
URS0000635088-tRNA3	0,352321158	10,57667068	0,499317168	0,967250135	LADA vs Control
URS000030C934-lncRNA3	0,356534881	4,417263546	0,499444033	0,967250135	LADA vs Control
URS00006361F3-Y_RNA3	0,544943773	4,856921385	0,500112564	0,967250135	LADA vs Control
URS00009C6074-antisense3	0,45911841	2,11618012	0,501545195	0,967250135	LADA vs Control
URS0000759AE0-lncRNA3	0,373816191	1,586466937	0,502152636	0,967250135	LADA vs Control
URS00004D7012-lncRNA3	-0,505444328	2,906892949	0,50382958	0,967250135	LADA vs Control
URS0000700D52-tRNA3	-0,417664739	2,144408558	0,506716881	0,967250135	LADA vs Control
URS0000097924-lncRNA3	0,243640563	5,60446115	0,509324193	0,967250135	LADA vs Control
URS0000A765F3-lncRNA3	-0,361478432	3,81544174	0,510046343	0,967250135	LADA vs Control
URS00009A0DCD-rRNA3	-0,305848624	5,942865971	0,51032012	0,967250135	LADA vs Control
URS0000ABD87F-rRNA3	-0,694171248	10,81861494	0,512078246	0,967250135	LADA vs Control
URS0000A8472C-Y_RNA3	0,446960816	3,823381815	0,513069007	0,967250135	LADA vs Control
URS000000898B-lncRNA3	0,429019838	2,509136511	0,516686066	0,967250135	LADA vs Control
URS00001EC8D7-snRNA3	0,28716758	4,577467935	0,517936423	0,967250135	LADA vs Control
URS000091B709-rRNA3	0,116440722	9,600448226	0,518023151	0,967250135	LADA vs Control
URS0000639DBE-tRNA3	-0,226848464	5,433019134	0,518024513	0,967250135	LADA vs Control
URS000075AF5F-antisense3	0,41860093	2,789120304	0,519319297	0,967250135	LADA vs Control
URS00005B2DE5-lncRNA3	-0,359972966	4,758632634	0,519397643	0,967250135	LADA vs Control
URS00008120D6-lncRNA3	0,365256383	1,844896317	0,519725617	0,967250135	LADA vs Control
URS00006C2C4A-Y_RNA3	-0,510971423	4,927831545	0,524108658	0,967250135	LADA vs Control
URS00000CD33E-antisense3	-0,356972361	3,630533758	0,524320433	0,967250135	LADA vs Control
URS0000197DBF-antisense3	0,357529169	3,806884628	0,526132913	0,967250135	LADA vs Control
URS0000391A04-lncRNA3	-0,285984462	5,482878094	0,526192675	0,967250135	LADA vs Control
URS000067474D-tRNA3	0,422114158	5,826738993	0,527121645	0,967250135	LADA vs Control
URS000094439F-rRNA3	0,078454626	11,62287065	0,528287041	0,967250135	LADA vs Control
URS00005C7D80-antisense3	-0,395259018	2,279201521	0,528413702	0,967250135	LADA vs Control
URS000009738A-lncRNA3	0,399379435	2,916436163	0,531211571	0,967250135	LADA vs Control
URS00006E19EA-Y_RNA3	0,260005254	9,597384946	0,532405077	0,967250135	LADA vs Control
URS00003D4983-antisense3	-0,380771173	1,851867325	0,532793401	0,967250135	LADA vs Control
URS0000689904-Y_RNA3	0,209843895	0,877747286	0,534745771	0,967250135	LADA vs Control
URS000024B38F-tRNA3	-0,366224466	4,404533737	0,538109036	0,967250135	LADA vs Control
URS000049BB82-lncRNA3	-0,32117284	4,039331254	0,540133274	0,967250135	LADA vs Control
URS00002750C5-lncRNA3	0,349328968	1,848936086	0,541967063	0,967250135	LADA vs Control
URS00003AD33F-lncRNA3	-0,397289997	3,336093709	0,547088614	0,967250135	LADA vs Control
URS0000611F3E-lncRNA3	-0,350545285	2,798037489	0,550320531	0,967250135	LADA vs Control
URS000012730D-lncRNA3	-0,367005266	3,696451386	0,551536099	0,967250135	LADA vs Control
URS00005A57D3-lncRNA3	-0,374392276	2,579526429	0,551551968	0,967250135	LADA vs Control
URS0000A88906-antisense3	0,36983677	1,925012855	0,552843716	0,967250135	LADA vs Control
URS0000089048-antisense3	0,336475153	1,859342356	0,554229955	0,967250135	LADA vs Control
URS0000942121-rRNA3	0,047454551	14,48998565	0,554391026	0,967250135	LADA vs Control
URS0000918AFB-rRNA3	0,156008065	8,610055402	0,554805845	0,967250135	LADA vs Control
URS00001AA18A-lncRNA3	0,335385216	1,870939952	0,558229134	0,967250135	LADA vs Control
URS00003C9A26-tRNA3	-0,116672509	8,822917147	0,559460541	0,967250135	LADA vs Control
URS000075D341-rRNA3	0,572595225	3,481242239	0,561472827	0,967250135	LADA vs Control
URS0000098211-lncRNA3	-0,373390074	2,630527968	0,566180272	0,967250135	LADA vs Control
URS00000C7470-antisense3	-0,345854241	3,497403787	0,566238148	0,967250135	LADA vs Control
URS00006C133C-tRNA3	-0,345910852	3,680848373	0,566326261	0,967250135	LADA vs Control

URS00000C18F2-tRNA3	-0,261773972	6,19082938	0,571717266	0,967250135	LADA vs Control
URS0000A9CA30-lncRNA3	0,335711136	3,103219008	0,573083452	0,967250135	LADA vs Control
URS0000189042-lncRNA3	-0,359635165	1,901016294	0,574139894	0,967250135	LADA vs Control
URS0000811D9C-antisense3	0,219835581	0,958216717	0,574921895	0,967250135	LADA vs Control
URS00006428FD-Y_RNA3	-0,259113757	6,936586919	0,576684041	0,967250135	LADA vs Control
URS0000AA014E-lncRNA3	0,317518279	2,880951371	0,577103468	0,967250135	LADA vs Control
URS000029A6A6-antisense3	-0,187153368	5,872434994	0,577368067	0,967250135	LADA vs Control
URS000076E14E-lncRNA3	0,405074799	2,947769863	0,577805069	0,967250135	LADA vs Control
URS000006D0D7-lncRNA3	0,333653158	2,152173172	0,57785213	0,967250135	LADA vs Control
URS000050471D-lncRNA3	-0,370842721	2,378838415	0,577946662	0,967250135	LADA vs Control
URS00005C51E2-lncRNA3	0,312574538	1,604886726	0,579786611	0,967250135	LADA vs Control
URS0000782759-lncRNA3	0,325115436	2,907193443	0,579975717	0,967250135	LADA vs Control
URS00008116E3-lncRNA3	-0,295003127	4,000279228	0,583816255	0,967250135	LADA vs Control
URS0000177135-lncRNA3	0,486371469	3,040442935	0,584488956	0,967250135	LADA vs Control
URS00003ADE1B-lncRNA3	0,339297221	2,400792438	0,584751457	0,967250135	LADA vs Control
URS0000005EDF-lncRNA3	-0,298199248	4,116716413	0,591615025	0,967250135	LADA vs Control
URS0000687FC3-Y_RNA3	-0,163496533	4,184219214	0,592282278	0,967250135	LADA vs Control
URS00006481F8-Y_RNA3	-0,175091392	7,303708498	0,592505757	0,967250135	LADA vs Control
URS000050FA69-lncRNA3	-0,299951085	3,930388911	0,593976486	0,967250135	LADA vs Control
URS0000690F87-snRNA3	0,397910604	3,114315559	0,594190845	0,967250135	LADA vs Control
URS0000780E15-lncRNA3	0,357919321	2,764657639	0,595618761	0,967250135	LADA vs Control
URS0000228E94-lncRNA3	0,330282412	2,335958891	0,596099678	0,967250135	LADA vs Control
URS00006F0FC0-tRNA3	-0,220420968	5,435006769	0,596719995	0,967250135	LADA vs Control
URS00000FB60D-tRNA3	0,324103572	3,59514453	0,597602022	0,967250135	LADA vs Control
URS000071C9A6-Y_RNA3	0,203882179	2,18048286	0,597610505	0,967250135	LADA vs Control
URS0000624312-Y_RNA3	0,226444971	5,698207316	0,597766644	0,967250135	LADA vs Control
URS0000222FD2-tRNA3	-0,341864583	3,623751413	0,599880942	0,967250135	LADA vs Control
URS00006E3DE1-snRNA3	-0,35590114	3,143314554	0,600601479	0,967250135	LADA vs Control
URS000076FC5A-lncRNA3	-0,345355137	2,343917503	0,602052872	0,967250135	LADA vs Control
URS00001D0B96-lncRNA3	-0,07263583	10,38627035	0,603412744	0,967250135	LADA vs Control
URS00003D2CC9-tRNA3	-0,288360112	4,271817892	0,608728863	0,967250135	LADA vs Control
URS0000907730-rRNA3	0,189359714	6,755248399	0,60970515	0,967250135	LADA vs Control
URS0000ABD82A-rRNA3	0,82052787	6,247493519	0,610488409	0,967250135	LADA vs Control
URS000091CD45-rRNA3	-0,28755102	6,581914152	0,612410478	0,967250135	LADA vs Control
URS00008FEDF1-rRNA3	0,33138449	2,23767686	0,612870703	0,967250135	LADA vs Control
URS0000145C5E-tRNA3	-0,337295301	2,892697082	0,613518758	0,967250135	LADA vs Control
URS000031A3C9-lncRNA3	-0,264684571	4,013881647	0,614954866	0,967250135	LADA vs Control
URS0000928682-rRNA3	-0,068790333	11,56053599	0,616387525	0,967250135	LADA vs Control
URS000000513C-antisense3	0,209689602	4,840741267	0,619291262	0,967250135	LADA vs Control
URS00006D1C46-snRNA3	0,250503104	6,026449126	0,62035898	0,967250135	LADA vs Control
URS00007BC71B-lncRNA3	0,316940481	1,944342872	0,620602155	0,967250135	LADA vs Control
URS00004D9E92-tRNA3	0,141081995	7,483097251	0,621243448	0,967250135	LADA vs Control
URS000014D40F-tRNA3	0,378329572	3,081622422	0,621660044	0,967250135	LADA vs Control
URS00003D279B-lncRNA3	0,2878024	3,869764687	0,623122338	0,967250135	LADA vs Control
URS00008BA2B6-lncRNA3	-0,274893581	2,826253026	0,623174499	0,967250135	LADA vs Control
URS000070B37B-tRNA3	-0,097207411	9,295263071	0,623430931	0,967250135	LADA vs Control
URS00004F482C-lncRNA3	-0,304008635	2,9888845	0,62393101	0,967250135	LADA vs Control
URS0000172E58-lncRNA3	0,278861418	3,538272714	0,624260766	0,967250135	LADA vs Control
URS000063CFC9-Y_RNA3	-0,308837568	2,480380401	0,625093169	0,967250135	LADA vs Control
URS00002F2DED-lncRNA3	-0,276635216	4,602442219	0,625734446	0,967250135	LADA vs Control
URS000029E713-antisense3	-0,257061719	4,061405427	0,626236588	0,967250135	LADA vs Control
URS000055C9F8-antisense3	-0,233326589	4,017918826	0,629600922	0,967250135	LADA vs Control
URS000097FEDE-rRNA3	-0,271906692	3,690975927	0,629722094	0,967250135	LADA vs Control
URS00000E43DB-antisense3	-0,272344595	3,522079692	0,62977959	0,967250135	LADA vs Control
URS00006FF680-Y_RNA3	-0,29760738	2,916835355	0,631566134	0,967250135	LADA vs Control
URS000000199E-lncRNA3	0,207277435	6,442848869	0,634066734	0,967250135	LADA vs Control
URS00006144FC-lncRNA3	-0,210090613	7,117689109	0,634870829	0,967250135	LADA vs Control
URS00002034DC-tRNA3	-0,281897717	5,180504267	0,636429447	0,967250135	LADA vs Control
URS00004EDF08-lncRNA3	-0,209221341	4,609717254	0,642549335	0,967250135	LADA vs Control
URS00001B2779-lncRNA3	0,284536112	1,848580988	0,643518641	0,967250135	LADA vs Control
URS00002CFBEA-lncRNA3	0,251124397	2,003500281	0,644278431	0,967250135	LADA vs Control
URS00004F2E54-lncRNA3	-0,281462741	1,792153167	0,644824211	0,967250135	LADA vs Control
URS00000C653A-lncRNA3	0,269475054	2,462989968	0,64584892	0,967250135	LADA vs Control
URS0000320E71-lncRNA3	0,239643858	2,909981717	0,647428979	0,967250135	LADA vs Control
URS00004AFADD-lncRNA3	0,274624442	1,695028896	0,649902132	0,967250135	LADA vs Control
URS0000511A5F-lncRNA3	-0,27599587	1,837603255	0,651364285	0,967250135	LADA vs Control

URS000009AC8B-tRNA3	-0,32688484	10,95540037	0,65392399	0,967250135	LADA vs Control
URS00002200B0-lncRNA3	0,280753294	3,176288712	0,654899772	0,967250135	LADA vs Control
URS00000AED6F-tRNA3	-0,221606329	6,83625311	0,655207169	0,967250135	LADA vs Control
URS00006D4008-tRNA3	0,328858206	2,742010752	0,65631191	0,967250135	LADA vs Control
URS00009840C1-rRNA3	0,192984722	6,418341272	0,656488244	0,967250135	LADA vs Control
URS000090DF6D-rRNA3	0,053377539	11,93047681	0,656813068	0,967250135	LADA vs Control
URS00007CB156-lncRNA3	-0,096724506	7,841069642	0,657999229	0,967250135	LADA vs Control
URS000036E063-antisense3	0,056021959	10,32347827	0,659749969	0,967250135	LADA vs Control
URS00006B479B-tRNA3	-0,215490796	1,462161631	0,660530852	0,967250135	LADA vs Control
URS00001C6042-antisense3	0,267351329	1,983328915	0,660807635	0,967250135	LADA vs Control
URS0000767DAE-lncRNA3	0,166096798	5,637395825	0,661043764	0,967250135	LADA vs Control
URS000096B970-rRNA3	-0,063043058	10,27519744	0,661101426	0,967250135	LADA vs Control
URS000038EEDC-lncRNA3	0,331772583	3,360541304	0,661176328	0,967250135	LADA vs Control
URS0000539731-antisense3	0,238164705	2,084997113	0,669321891	0,967250135	LADA vs Control
URS000038803E-tRNA3	-0,143825946	5,940754642	0,67275023	0,967250135	LADA vs Control
URS00003875B8-lncRNA3	0,279839779	2,156965412	0,672755192	0,967250135	LADA vs Control
URS00009AAC46-rRNA3	0,082222849	9,797442669	0,673440572	0,967250135	LADA vs Control
URS000061A10B-tRNA3	-0,311702018	2,36803002	0,673886874	0,967250135	LADA vs Control
URS00004AE57B-lncRNA3	0,256913547	2,181566953	0,675902569	0,967250135	LADA vs Control
URS00008BB15B-lncRNA3	0,302413672	2,482102831	0,676016489	0,967250135	LADA vs Control
URS00006D9244-tRNA3	-0,278337532	3,430512932	0,677200772	0,967250135	LADA vs Control
URS0000495A30-lncRNA3	0,232078429	2,253557055	0,680864877	0,967250135	LADA vs Control
URS00008120F8-lncRNA3	0,253481427	1,863766293	0,681917172	0,967250135	LADA vs Control
URS000099D184-rRNA3	-0,174758659	4,977808511	0,68534087	0,967250135	LADA vs Control
URS00004E7DF9-lncRNA3	-0,211551673	4,28189465	0,685885147	0,967250135	LADA vs Control
URS0000257C23-lncRNA3	0,292661921	4,584581553	0,685906052	0,967250135	LADA vs Control
URS000023352F-lncRNA3	-0,22271854	2,158198536	0,687670031	0,967250135	LADA vs Control
URS00006AD81D-Y_RNA3	-0,257655259	1,867732365	0,688714867	0,967250135	LADA vs Control
URS00006174C2-tRNA3	-0,095811121	8,218629708	0,688861807	0,967250135	LADA vs Control
URS00007C72D0-lncRNA3	-0,205757814	1,699191247	0,691389084	0,967250135	LADA vs Control
URS00000A7F2D-lncRNA3	0,223493481	2,135027243	0,692546468	0,967250135	LADA vs Control
URS000063B690-snRNA3	0,242537413	3,324962274	0,693366534	0,967250135	LADA vs Control
URS000041FE38-lncRNA3	-0,228686266	2,353684167	0,693857209	0,967250135	LADA vs Control
URS000028E102-lncRNA3	-0,234621885	2,546561157	0,694702459	0,967250135	LADA vs Control
URS000076B0D1-lncRNA3	0,235189749	3,094918128	0,695315001	0,967250135	LADA vs Control
URS000064217E-Y_RNA3	-0,246607347	2,41296644	0,695993128	0,967250135	LADA vs Control
URS0000007D24-misc_RNA]3	-0,598516135	6,091419652	0,696193804	0,967250135	LADA vs Control
URS00002840A7-lncRNA3	0,236760501	2,142345929	0,696754926	0,967250135	LADA vs Control
URS00001AD596-tRNA3	-0,068030506	11,15468011	0,69838734	0,967250135	LADA vs Control
URS00002A865C-lncRNA3	-0,221130827	1,751381992	0,699669016	0,967250135	LADA vs Control
URS00001EE979-lncRNA3	0,25852361	2,774714775	0,701089543	0,967250135	LADA vs Control
URS00000A00A2-antisense3	-0,259531165	4,833316746	0,701722013	0,967250135	LADA vs Control
URS00000DA554-lncRNA3	0,233857293	2,522581542	0,702137736	0,967250135	LADA vs Control
URS000099BDEE-rRNA3	0,235111006	5,090607985	0,704468801	0,967250135	LADA vs Control
URS00004DA951-rRNA3	0,131964023	10,73867185	0,704661493	0,967250135	LADA vs Control
URS0000278E1B-tRNA3	0,226433807	2,766067348	0,705212462	0,967250135	LADA vs Control
URS000090AA7A-rRNA3	0,2355047	2,615865166	0,706298949	0,967250135	LADA vs Control
URS0000684921-rRNA3	0,233787346	2,725738393	0,707102745	0,967250135	LADA vs Control
URS0000381123-lncRNA3	0,223596065	2,471894131	0,709704945	0,968913588	LADA vs Control
URS00006F4537-tRNA3	0,236480014	4,037892922	0,71436992	0,972014228	LADA vs Control
URS0000920597-rRNA3	-0,182431079	8,520273949	0,714757244	0,972014228	LADA vs Control
URS00000347CF-lncRNA3	-0,189482323	3,718528756	0,71777111	0,974059241	LADA vs Control
URS00006CFDFE-Y_RNA3	-0,161896578	2,249644641	0,720593738	0,974059241	LADA vs Control
URS0000697465-tRNA3	0,231257444	4,721333977	0,720772754	0,974059241	LADA vs Control
URS0000941729-rRNA3	-0,182659453	4,187919105	0,723214759	0,974059241	LADA vs Control
URS00003CA240-antisense3	-0,153441498	5,133383215	0,724620981	0,974059241	LADA vs Control
URS000059900F-tRNA3	0,211180425	3,409718688	0,725051859	0,974059241	LADA vs Control
URS0000075AD1-lncRNA3	-0,197734692	1,794828601	0,727159132	0,974059241	LADA vs Control
URS0000918BC5-rRNA3	0,332672066	3,816571784	0,727451259	0,974059241	LADA vs Control
URS0000977FD2-SRP_RNA3	0,207076035	2,037495207	0,728802551	0,974059241	LADA vs Control
URS00006529EE-Y_RNA3	-0,214121474	3,147668241	0,734437228	0,974990024	LADA vs Control
URS00002811B6-lncRNA3	-0,185764475	3,62513136	0,735454282	0,974990024	LADA vs Control
URS0000383A48-tRNA3	0,208543532	3,229014707	0,735697416	0,974990024	LADA vs Control
URS00004AAD0A-lncRNA3	-0,197343043	2,996600067	0,736296255	0,974990024	LADA vs Control
URS00008B26D4-antisense3	0,189231629	4,369068967	0,736473152	0,974990024	LADA vs Control
URS0000605144-antisense3	-0,202711467	2,730860041	0,738984315	0,975148337	LADA vs Control

URS00004E5B9A-lncRNA3	-0,190863096	1,8276158	0,739382859	0,975148337	LADA vs Control
URS000047A7F4-rRNA3	-0,343679104	4,287976289	0,741090597	0,97543827	LADA vs Control
URS00001BF716-lncRNA3	0,067584991	7,608759782	0,742484878	0,97543827	LADA vs Control
URS00003E3BDF-antisense3	-0,187619861	2,398707663	0,743789125	0,97543827	LADA vs Control
URS00002C130C-tRNA3	0,307829607	3,549564243	0,746463304	0,977112078	LADA vs Control
URS0000166229-lncRNA3	0,174401446	2,097439644	0,751312197	0,981620982	LADA vs Control
URS0000986F6F-rRNA3	-0,149497606	5,818355153	0,753532281	0,982684822	LADA vs Control
URS00005AF1AA-antisense3	0,140893093	1,232925367	0,757337358	0,983823285	LADA vs Control
URS00000DE490-lncRNA3	0,166041745	3,392102095	0,757527489	0,983823285	LADA vs Control
URS00006ABCCE-tRNA3	0,247775373	11,64642261	0,75886165	0,983823285	LADA vs Control
URS000056B96A-antisense3	0,164160523	2,621722386	0,760035156	0,983823285	LADA vs Control
URS0000701637-rRNA3	-0,193622888	2,670549892	0,765084635	0,985114039	LADA vs Control
URS0000121433-tRNA3	0,238442829	4,021698317	0,765713363	0,985114039	LADA vs Control
URS000091563C-rRNA3	0,037333031	10,26950568	0,765974367	0,985114039	LADA vs Control
URS00006744D5-tRNA3	0,200618098	2,786516677	0,770623585	0,985114039	LADA vs Control
URS00002AD6F3-antisense3	-0,14975512	1,553235742	0,772070658	0,985114039	LADA vs Control
URS000041043F-antisense3	0,172659014	1,994452875	0,773154464	0,985114039	LADA vs Control
URS00001E9163-lncRNA3	-0,171574272	2,764922404	0,773685557	0,985114039	LADA vs Control
URS00006F4F8C-snoRNA3	0,066705219	8,039702247	0,774118358	0,985114039	LADA vs Control
URS000068F7ED-Y_RNA3	0,153562205	6,623874775	0,77683684	0,985114039	LADA vs Control
URS00001D4EE9-tRNA3	-0,199707504	2,839543714	0,777490155	0,985114039	LADA vs Control
URS000034E9D0-misc_RNA]3	0,249626899	3,097582218	0,780302742	0,985114039	LADA vs Control
URS0000082453-lncRNA3	0,160768568	2,456766093	0,780882077	0,985114039	LADA vs Control
URS00003475A2-lncRNA3	0,096392907	6,073704566	0,78522881	0,985114039	LADA vs Control
URS000015BB33-lncRNA3	0,14441232	3,745978664	0,785332345	0,985114039	LADA vs Control
URS00000AF0EF-lncRNA3	0,159562569	3,804446661	0,789157338	0,985114039	LADA vs Control
URS00005C220A-antisense3	-0,184701233	2,894185806	0,789652488	0,985114039	LADA vs Control
URS00002D1F9F-lncRNA3	-0,144785319	3,254441607	0,790202413	0,985114039	LADA vs Control
URS00005DB87D-tRNA3	0,056608614	7,975358739	0,790896396	0,985114039	LADA vs Control
URS00005AF005-lncRNA3	-0,153892055	3,522774737	0,791308573	0,985114039	LADA vs Control
URS0000287398-tRNA3	-0,177065147	2,440463804	0,794482362	0,985114039	LADA vs Control
URS00005AAAF0-antisense3	0,15545349	3,447551336	0,795294098	0,985114039	LADA vs Control
URS0000593A4A-lncRNA3	0,166975987	3,380617038	0,7963809	0,985114039	LADA vs Control
URS00006772C0-tRNA3	0,16800502	2,278759881	0,79666249	0,985114039	LADA vs Control
URS0000038397-lncRNA3	0,150453275	5,458381823	0,797375245	0,985114039	LADA vs Control
URS00007DFA49-antisense3	0,193520391	2,453720457	0,797843366	0,985114039	LADA vs Control
URS00003C2ECD-antisense3	-0,176364721	2,559377792	0,802410763	0,985114039	LADA vs Control
URS00004C82E1-antisense3	-0,163823568	4,084336348	0,802973089	0,985114039	LADA vs Control
URS00003C98B0-lncRNA3	0,159926224	3,045496821	0,803023093	0,985114039	LADA vs Control
URS000095D156-rRNA3	0,107897392	6,045790564	0,805650762	0,985114039	LADA vs Control
URS00006CDFA7-rRNA3	-0,139532574	2,666787507	0,805862157	0,985114039	LADA vs Control
URS0000605748-lncRNA3	0,149842873	3,689393404	0,806146053	0,985114039	LADA vs Control
URS00003CBFB8-lncRNA3	-0,139695493	2,075423342	0,807377955	0,985114039	LADA vs Control
URS000069E2A5-tRNA3	0,157240388	3,421985706	0,809321924	0,985114039	LADA vs Control
URS0000265843-antisense3	-0,134241934	2,862204159	0,810263288	0,985114039	LADA vs Control
URS0000161EE9-lncRNA3	-0,099847372	6,078069715	0,811337785	0,985114039	LADA vs Control
URS00001F3EF6-lncRNA3	-0,174432279	2,384806636	0,811767791	0,985114039	LADA vs Control
URS000020220C-antisense3	-0,139916422	2,749759905	0,814742215	0,985569806	LADA vs Control
URS000064E10F-tRNA3	-0,145503811	3,013775094	0,815056767	0,985569806	LADA vs Control
URS00006D484A-rRNA3	0,024489528	11,67221899	0,817056307	0,985569806	LADA vs Control
URS00002968BD-antisense3	0,079015888	7,356778365	0,820898304	0,985569806	LADA vs Control
URS0000630B8A-tRNA3	-0,111668205	5,357715317	0,821700074	0,985569806	LADA vs Control
URS0000590507-lncRNA3	-0,127608922	1,90331514	0,824021096	0,985569806	LADA vs Control
URS0000A85AEE-lncRNA3	-0,154282033	3,200285639	0,8251719	0,985569806	LADA vs Control
URS00002901EC-tRNA3	-0,137508718	2,043121881	0,825583305	0,985569806	LADA vs Control
URS00005BB09E-lncRNA3	-0,091165108	6,047129544	0,827158945	0,985569806	LADA vs Control
URS000013BB40-lncRNA3	0,133885181	2,666260296	0,828328417	0,985569806	LADA vs Control
URS000005AEAB-tRNA3	-0,105872165	4,48776437	0,829942205	0,985569806	LADA vs Control
URS00001AE429-lncRNA3	0,141054579	3,26760494	0,830214332	0,985569806	LADA vs Control
URS00007D24CA-lncRNA3	-0,172183133	2,948889293	0,830472984	0,985569806	LADA vs Control
URS00006C14B2-tRNA3	0,150915799	3,304706256	0,832524069	0,986329363	LADA vs Control
URS0000112A1A-antisense3	0,116581201	4,257411498	0,837141505	0,989927488	LADA vs Control
URS0000391360-lncRNA3	-0,127483285	2,256356542	0,841682369	0,989927488	LADA vs Control
URS000090169F-rRNA3	0,112631336	1,691620139	0,845363528	0,989927488	LADA vs Control
URS000024383A-lncRNA3	0,11078587	1,637698963	0,846995326	0,989927488	LADA vs Control
URS000013899F-tRNA3	0,099211486	4,501396588	0,847646853	0,989927488	LADA vs Control

URS00007125F9-rRNA3	-0,11748631	2,409823294	0,85043003	0,989927488	LADA vs Control
URS00006CC125-rRNA3	0,113202025	2,366732734	0,851406764	0,989927488	LADA vs Control
URS0000A9F786-lncRNA3	0,134148802	3,099717163	0,85178672	0,989927488	LADA vs Control
URS0000134A86-antisense3	-0,13180247	3,351781773	0,851789348	0,989927488	LADA vs Control
URS00001142E8-lncRNA3	-0,0715546	5,750896426	0,853730094	0,989927488	LADA vs Control
URS00002AECD7-lncRNA3	0,123675143	2,148873116	0,854706378	0,989927488	LADA vs Control
URS0000093DF5-lncRNA3	0,107574571	2,157430584	0,856269653	0,989927488	LADA vs Control
URS0000759CF4-lncRNA3	0,106936293	1,845197414	0,857880539	0,989927488	LADA vs Control
URS000095C3C7-rRNA3	0,086886965	4,667191645	0,859162885	0,989927488	LADA vs Control
URS00001A86BB-tRNA3	0,093760398	2,65628615	0,859533727	0,989927488	LADA vs Control
URS00000EB76F-lncRNA3	0,110108022	2,382363845	0,859806162	0,989927488	LADA vs Control
URS000019B78E-misc_RNA]3	0,152999885	3,588733066	0,861767832	0,989927488	LADA vs Control
URS00002CDF5D-lncRNA3	0,093999462	2,151438672	0,863484615	0,989927488	LADA vs Control
URS00004A68E9-antisense3	0,076788354	3,654195066	0,863484707	0,989927488	LADA vs Control
URS0000063647-antisense3	0,053251754	6,169603323	0,868909005	0,989927488	LADA vs Control
URS00001B59BD-lncRNA3	0,045864917	6,767957763	0,870369148	0,989927488	LADA vs Control
URS000047A7F4-misc_RNA]3	0,153507232	4,238929678	0,872692657	0,989927488	LADA vs Control
URS00001FBD75-misc_RNA]3	0,147225135	3,307722647	0,872881552	0,989927488	LADA vs Control
URS000060A110-antisense3	-0,078131049	1,367875278	0,874057685	0,989927488	LADA vs Control
URS000064D54F-tRNA3	-0,090044493	2,523144202	0,877401334	0,989927488	LADA vs Control
URS00002172B5-lncRNA3	-0,113093912	2,390444931	0,880141416	0,989927488	LADA vs Control
URS000071ED2F-tRNA3	-0,10186713	2,991616318	0,880615614	0,989927488	LADA vs Control
URS0000504A1A-lncRNA3	-0,079113134	1,470603839	0,887535243	0,989927488	LADA vs Control
URS00003F2105-lncRNA3	0,085994802	2,406932528	0,887811109	0,989927488	LADA vs Control
URS000062F68C-rRNA3	0,019927973	10,93938784	0,888734212	0,989927488	LADA vs Control
URS000075D48E-lncRNA3	0,072453711	1,602010302	0,890125487	0,989927488	LADA vs Control
URS00009C60C3-lncRNA3	0,110601989	3,075209684	0,891642031	0,989927488	LADA vs Control
URS0000594305-lncRNA3	0,076444583	1,940137107	0,894513051	0,989927488	LADA vs Control
URS000069B369-Y_RNA3	-0,095709359	9,690811268	0,896161476	0,989927488	LADA vs Control
URS000045E276-antisense3	0,075416836	7,301870906	0,896283779	0,989927488	LADA vs Control
URS0000990012-rRNA3	0,044963909	8,57303772	0,897030435	0,989927488	LADA vs Control
URS00006E1108-Y_RNA3	0,04649027	4,049124964	0,898202033	0,989927488	LADA vs Control
URS00007116F9-Y_RNA3	0,07319672	3,042319437	0,899100178	0,989927488	LADA vs Control
URS00002C4609-lncRNA3	0,077125971	2,010894744	0,903036424	0,989927488	LADA vs Control
URS000007383C-antisense3	-0,069473326	2,496432723	0,904032031	0,989927488	LADA vs Control
URS000019F398-lncRNA3	0,064527149	1,659002941	0,905167998	0,989927488	LADA vs Control
URS00008116FE-lncRNA3	0,070371287	2,826119493	0,905693688	0,989927488	LADA vs Control
URS0000502C74-tRNA3	-0,022230628	14,17701257	0,906266217	0,989927488	LADA vs Control
URS00004BF687-tRNA3	-0,077726893	13,4414794	0,906466816	0,989927488	LADA vs Control
URS000035D229-lncRNA3	-0,100835491	3,593153184	0,906815789	0,989927488	LADA vs Control
URS00005B30A9-tRNA3	0,081295948	2,750819629	0,907590529	0,989927488	LADA vs Control
URS0000576D5D-lncRNA3	-0,067848079	1,73318224	0,907887806	0,989927488	LADA vs Control
URS000092B92B-rRNA3	0,16571283	5,336196426	0,909797948	0,989927488	LADA vs Control
URS0000997FE9-rRNA3	0,063712277	2,876398415	0,910829194	0,989927488	LADA vs Control
URS0000635FFC-Y_RNA3	-0,06798813	2,322009496	0,911376942	0,989927488	LADA vs Control
URS0000A89523-lncRNA3	-0,069977386	2,553603804	0,911736239	0,989927488	LADA vs Control
URS00006BF71F-rRNA3	0,067676646	2,492314255	0,912295906	0,989927488	LADA vs Control
URS00000DAC34-lncRNA3	-0,064745165	2,636836099	0,913296555	0,989927488	LADA vs Control
URS000001AE93-lncRNA3	0,02671225	6,898854125	0,914027062	0,989927488	LADA vs Control
URS00009A7848-rRNA3	-0,014462578	11,54132926	0,91411652	0,989927488	LADA vs Control
URS00006C900C-tRNA3	0,060659415	2,991743424	0,916577706	0,989927488	LADA vs Control
URS000023F3B4-lncRNA3	-0,051492693	3,955198744	0,917882795	0,989927488	LADA vs Control
URS000096E1E3-rRNA3	-0,063941564	2,495902245	0,918738852	0,989927488	LADA vs Control
URS00009843EB-rRNA3	0,019953243	12,28502234	0,919117224	0,989927488	LADA vs Control
URS00006EB1B5-Y_RNA3	-0,055444077	4,084821132	0,920712222	0,990119759	LADA vs Control
URS0000649B00-rRNA3	0,014579485	10,09773081	0,925459219	0,993167104	LADA vs Control
URS00005508F6-antisense3	-0,054478416	3,129098015	0,926387628	0,993167104	LADA vs Control
URS000034EAB6-lncRNA3	0,058729431	2,280705495	0,928266963	0,993176045	LADA vs Control
URS00006005A4-lncRNA3	-0,063595263	2,906455771	0,93140279	0,993176045	LADA vs Control
URS00006D1A54-Y_RNA3	-0,045768983	3,828527713	0,933081922	0,993176045	LADA vs Control
URS0000A77003-lncRNA3	0,046787446	2,821180105	0,938486899	0,993176045	LADA vs Control
URS000096D31D-rRNA3	0,014972674	10,50581429	0,940867321	0,993176045	LADA vs Control
URS00006AD70A-tRNA3	-0,04945337	4,843339833	0,94151898	0,993176045	LADA vs Control
URS00006EBF05-misc_RNA]3	0,071861182	3,622681484	0,941651874	0,993176045	LADA vs Control
URS000008089F-lncRNA3	-0,044991083	3,494287219	0,948571427	0,993176045	LADA vs Control
URS00003DEE5B-lncRNA3	0,037567197	3,256575112	0,948743283	0,993176045	LADA vs Control

URS00002B0998-lncRNA3	0,038101435	2,419217026	0,949209956	0,993176045	LADA vs Control
URS000057C597-antisense3	0,039224228	2,337861425	0,949956394	0,993176045	LADA vs Control
URS00005B7465-tRNA3	0,035706762	1,847741502	0,953036465	0,993176045	LADA vs Control
URS00001D081D-antisense3	0,033760312	1,824716399	0,954515834	0,993176045	LADA vs Control
URS0000378BB8-lncRNA3	-0,029640406	4,18272789	0,955247208	0,993176045	LADA vs Control
URS000020BB55-lncRNA3	0,03571653	3,131770633	0,958690974	0,993176045	LADA vs Control
URS000055B99E-lncRNA3	-0,034822536	2,716513459	0,958965345	0,993176045	LADA vs Control
URS000098604A-rRNA3	-0,030683247	1,872688165	0,959540855	0,993176045	LADA vs Control
URS0000A9525F-antisense3	0,029147662	2,995531648	0,959855489	0,993176045	LADA vs Control
URS00005E51DB-lncRNA3	0,025643716	4,114269415	0,960294198	0,993176045	LADA vs Control
URS00001B506A-tRNA3	0,034504378	2,259860507	0,961029138	0,993176045	LADA vs Control
URS000075A823-rRNA3	0,068869849	6,786069906	0,962447249	0,993176045	LADA vs Control
URS00005BB5C9-antisense3	-0,026066973	1,956136305	0,963628525	0,993176045	LADA vs Control
URS0000762146-antisense3	0,017490772	5,166011847	0,964379443	0,993176045	LADA vs Control
URS000011ABD1-lncRNA3	-0,030191942	2,282001703	0,964729262	0,993176045	LADA vs Control
URS0000181B59-lncRNA3	0,031957279	2,274734234	0,964938104	0,993176045	LADA vs Control
URS000020AD62-lncRNA3	-0,025023336	1,816448524	0,965061338	0,993176045	LADA vs Control
URS0000209048-tRNA3	0,028045873	3,508601642	0,965380381	0,993176045	LADA vs Control
URS00006C06E6-Y_RNA3	-0,025807195	1,912207487	0,966179844	0,993176045	LADA vs Control
URS0000701607-Y_RNA3	-0,022734398	3,386959123	0,969544339	0,994473857	LADA vs Control
URS000015D954-antisense3	-0,021501017	1,950160466	0,97142054	0,994473857	LADA vs Control
URS0000417E86-lncRNA3	-0,022150426	2,54225615	0,972190329	0,994473857	LADA vs Control
URS00002548DF-lncRNA3	-0,018587672	1,501270697	0,97344756	0,994473857	LADA vs Control
URS0000365006-lncRNA3	0,018439941	2,397164181	0,974555926	0,994473857	LADA vs Control
URS0000920CBD-rRNA3	0,038525482	5,869762745	0,976244669	0,994744932	LADA vs Control
URS00004106BA-lncRNA3	-0,013805811	1,798128499	0,978865695	0,995963786	LADA vs Control
URS00005D1950-antisense3	-0,011891259	2,067377939	0,982977364	0,998176141	LADA vs Control
URS00004CE099-lncRNA3	-0,016513521	3,298450018	0,984929896	0,998176141	LADA vs Control
URS0000679FAF-tRNA3	-0,009017306	5,101125315	0,985324087	0,998176141	LADA vs Control
URS000069466F-Y_RNA3	0,004759671	7,597826288	0,987578576	0,99881972	LADA vs Control
URS000062FB25-rRNA3	0,001902413	9,742461609	0,989192133	0,99881972	LADA vs Control
URS00003F41E9-lncRNA3	-0,002574266	8,007323205	0,990415783	0,99881972	LADA vs Control
URS0000653BD1-Y_RNA3	-0,004854259	4,226797259	0,992993049	0,99881972	LADA vs Control
URS00001E5F12-lncRNA3	-0,00477988	2,516521413	0,99389694	0,99881972	LADA vs Control
URS00004AC036-lncRNA3	-0,003634382	4,515610764	0,994532941	0,99881972	LADA vs Control
URS00000E0DE1-antisense3	-0,001690217	3,104520142	0,997661345	0,999997358	LADA vs Control
URS0000A8428E-Y_RNA3	-0,000624152	5,101971983	0,99940488	0,999997358	LADA vs Control
URS000048EB2F-antisense3	-2,00533E-06	2,157167592	0,999997358	0,999997358	LADA vs Control
URS0000684E4B-tRNA4	-1,118765309	6,262078663	8,83264E-06	0,006174014	T1D vs Control
URS000098FA76-rRNA4	1,118972412	2,496348799	0,000213754	0,074706917	T1D vs Control
URS0000ABD8C6-rRNA4	-4,33420538	7,712316992	0,00040174	0,080100492	T1D vs Control
URS00002EDD28-lncRNA4	-1,331129571	5,436899358	0,000458372	0,080100492	T1D vs Control
URS0000341866-lncRNA4	-1,993465872	4,848112839	0,00084903	0,106251401	T1D vs Control
URS00006C0413-Y_RNA4	-1,967347138	2,155330761	0,001051583	0,106251401	T1D vs Control
URS00000540AC-antisense4	-1,839630836	3,451834854	0,001064034	0,106251401	T1D vs Control
URS00006C133C-tRNA4	-1,829884603	3,680848373	0,001286999	0,112451514	T1D vs Control
URS00001DBD56-lncRNA4	-1,597519071	3,720321434	0,001517929	0,117892488	T1D vs Control
URS000003870B-lncRNA4	-0,661387727	9,957227048	0,001762749	0,123216176	T1D vs Control
URS00006E3DE1-snRNA4	-1,97752229	3,143314554	0,00206053	0,130937299	T1D vs Control
URS00006D1735-snRNA4	-1,45267513	5,596569208	0,002498581	0,141792311	T1D vs Control
URS0000A7AB58-Y_RNA4	-1,616151385	4,159115672	0,002788578	0,141792311	T1D vs Control
URS00009C6070-lncRNA4	-1,554769986	2,53934864	0,003009527	0,141792311	T1D vs Control
URS000063A7A5-Y_RNA4	-0,885004782	8,2571435	0,003230659	0,141792311	T1D vs Control
URS0000996BBC-rRNA4	-0,544205056	8,622403976	0,003442171	0,141792311	T1D vs Control
URS000006044C-lncRNA4	-1,563047618	1,808588154	0,003561506	0,141792311	T1D vs Control
URS000069E2A5-tRNA4	-1,784220756	3,421985706	0,003658818	0,141792311	T1D vs Control
URS0000537899-antisense4	-1,663267267	2,143848964	0,003854154	0,141792311	T1D vs Control
URS000071C9A6-Y_RNA4	1,043469357	2,18048286	0,004262824	0,148120438	T1D vs Control
URS00000F6ECB-lncRNA4	-1,079265939	5,128846246	0,004622245	0,148120438	T1D vs Control
URS00002D0015-lncRNA4	-1,466230054	3,226559685	0,004661874	0,148120438	T1D vs Control
URS0000495A30-lncRNA4	-1,479894432	2,253557055	0,004930191	0,149834942	T1D vs Control
URS00007CA557-lncRNA4	-0,621465383	9,085280894	0,00944786	0,271005229	T1D vs Control
URS0000974435-SRP_RNA4	1,343773571	2,886817066	0,009961322	0,271005229	T1D vs Control
URS000096EEE8-rRNA4	0,336081264	8,595030803	0,010080309	0,271005229	T1D vs Control
URS00005B6FC3-lncRNA4	1,095948262	4,468244677	0,010929464	0,275309825	T1D vs Control
URS0000515855-lncRNA4	-1,309005872	2,502117237	0,011432181	0,275309825	T1D vs Control

URS0000278E1B-tRNA4	-1,421085158	2,766067348	0,01159487	0,275309825	T1D vs Control
URS0000907244-rRNA4	-0,61723186	6,675718226	0,012109882	0,275309825	T1D vs Control
URS0000515429-lncRNA4	0,933660561	7,164323775	0,012209735	0,275309825	T1D vs Control
URS00000A00A2-antisense4	-1,574512408	4,833316746	0,012958404	0,275443871	T1D vs Control
URS000024B38F-tRNA4	-1,385253222	4,404533737	0,013146199	0,275443871	T1D vs Control
URS000069ED7F-Y_RNA4	-1,451926132	4,579144673	0,013397842	0,275443871	T1D vs Control
URS000029A6A6-antisense4	-0,758269056	5,872434994	0,016048746	0,310309673	T1D vs Control
URS00007D6D04-lncRNA4	-1,21075059	3,22797681	0,016346349	0,310309673	T1D vs Control
URS0000676525-Y_RNA4	-1,225032451	4,329395557	0,016425548	0,310309673	T1D vs Control
URS000014D914-antisense4	-1,180015863	4,160373223	0,017258132	0,311736208	T1D vs Control
URS00002598CF-lncRNA4	-1,182408417	4,630900569	0,017393007	0,311736208	T1D vs Control
URS00006FCBA3-Y_RNA4	-1,307590608	2,601539284	0,018359936	0,315142485	T1D vs Control
URS0000022477-lncRNA4	-1,317340184	1,898475606	0,018484752	0,315142485	T1D vs Control
URS00006CE1FB-misc_RNA]4	2,161452896	6,644778412	0,019396134	0,318290862	T1D vs Control
URS0000677B31-tRNA4	0,964244129	7,303130994	0,020352066	0,318290862	T1D vs Control
URS0000ABD879-rRNA4	-2,785381737	7,136004534	0,020369064	0,318290862	T1D vs Control
URS00003008EB-lncRNA4	-1,110063857	4,342465078	0,020941298	0,318290862	T1D vs Control
URS00001EC8D7-snRNA4	-0,958648174	4,577467935	0,02110394	0,318290862	T1D vs Control
URS0000944F10-rRNA4	-0,925484526	5,261748123	0,021401531	0,318290862	T1D vs Control
URS0000389FBF-lncRNA4	-1,250855918	2,547635864	0,022502024	0,322237419	T1D vs Control
URS000047AF1F-lncRNA4	1,984228662	4,480968908	0,022994126	0,322237419	T1D vs Control
URS00006642D4-Y_RNA4	-0,437669326	4,428261145	0,023049887	0,322237419	T1D vs Control
URS000065A213-Y_RNA4	-1,063790653	10,7844197	0,025559118	0,342958015	T1D vs Control
URS0000100BA4-antisense4	-1,282698245	1,836907151	0,025923974	0,342958015	T1D vs Control
URS0000A774C0-lncRNA4	-0,656039536	6,402978234	0,026747298	0,342958015	T1D vs Control
URS0000610FFE-tRNA4	-1,230499394	2,353369293	0,027289658	0,342958015	T1D vs Control
URS000031A3C9-lncRNA4	-1,096473201	4,013881647	0,027736843	0,342958015	T1D vs Control
URS000011BDAF-antisense4	-1,150837837	2,89470373	0,027844453	0,342958015	T1D vs Control
URS00004E7DF9-lncRNA4	-1,077516839	4,28189465	0,027966533	0,342958015	T1D vs Control
URS00000FCDE9-misc_RNA]4	-1,584401896	3,011608195	0,02964783	0,353412468	T1D vs Control
URS00006CBBB9-Y_RNA4	-1,261474822	3,554703445	0,029830237	0,353412468	T1D vs Control
URS0000920CBD-rRNA4	-2,624355287	5,869762745	0,033370971	0,388771813	T1D vs Control
URS0000699390-Y_RNA4	-0,995137862	13,62373063	0,034808621	0,38877812	T1D vs Control
URS0000727FD6-tRNA4	1,261487566	3,320399515	0,034933546	0,38877812	T1D vs Control
URS0000812136-lncRNA4	-0,465038758	9,121672555	0,035040088	0,38877812	T1D vs Control
URS00006C2A6C-Y_RNA4	-1,040518574	10,4018413	0,037646622	0,407952843	T1D vs Control
URS0000196BD3-lncRNA4	-1,139008609	2,056335374	0,037935529	0,407952843	T1D vs Control
URS00001A86BB-tRNA4	1,028806434	2,65628615	0,038546303	0,40824039	T1D vs Control
URS000057C597-antisense4	-1,212030269	2,337861425	0,039390148	0,410231744	T1D vs Control
URS00008C3E41-lncRNA4	0,858175927	5,384577661	0,040576659	0,410231744	T1D vs Control
URS00008119F8-lncRNA4	-1,290286624	3,00844528	0,04106655	0,410231744	T1D vs Control
URS0000AABA8B-lncRNA4	-0,517382982	9,325488704	0,041081863	0,410231744	T1D vs Control
URS0000697465-tRNA4	1,22186703	4,721333977	0,042396896	0,415182846	T1D vs Control
URS000011ABD1-lncRNA4	1,293618908	2,282001703	0,043540039	0,415182846	T1D vs Control
URS000044BAE3-tRNA4	0,423832716	7,695683129	0,04412606	0,415182846	T1D vs Control
URS00006B0E5A-precursor_RNA4	-0,813211365	6,734564531	0,044484511	0,415182846	T1D vs Control
URS0000075AD1-lncRNA4	-1,065534822	1,794828601	0,044547516	0,415182846	T1D vs Control
URS0000682FB0-Y_RNA4	-0,837893142	10,97882695	0,045320363	0,416828079	T1D vs Control
URS00003A00A4-lncRNA4	1,171484726	3,039610494	0,0466223	0,418622776	T1D vs Control
URS00001B6230-lncRNA4	-1,088759585	2,056088304	0,047141376	0,418622776	T1D vs Control
URS0000007D24-misc_RNA]4	-2,873897383	6,091419652	0,047555296	0,418622776	T1D vs Control
URS00003F2CFE-lncRNA4	-1,099712456	4,520438351	0,047911047	0,418622776	T1D vs Control
URS00000DA56A-lncRNA4	-1,144876072	2,589490843	0,049012973	0,422963808	T1D vs Control
URS0000A77003-lncRNA4	1,11356417	2,821180105	0,050058034	0,426714214	T1D vs Control
URS000009DDCA-tRNA4	-0,517660629	8,707527281	0,051224705	0,427666401	T1D vs Control
URS000038803E-tRNA4	-0,6185685	5,940754642	0,051943745	0,427666401	T1D vs Control
URS00006A9AE8-Y_RNA4	-1,409472564	5,946221857	0,052005213	0,427666401	T1D vs Control
URS000058CCC6-antisense4	-1,083474164	2,503982133	0,053654254	0,436096782	T1D vs Control
URS00002A28BF-lncRNA4	-1,222732317	2,823053274	0,059354934	0,463166429	T1D vs Control
URS000097CDD0-rRNA4	0,421525224	6,617743447	0,059452859	0,463166429	T1D vs Control
URS0000365006-lncRNA4	-1,006090017	2,397164181	0,060472348	0,463166429	T1D vs Control
URS000075AC32-lncRNA4	-1,122355604	2,916319528	0,060623692	0,463166429	T1D vs Control
URS000075A564-lncRNA4	-1,059493475	2,096352205	0,060831216	0,463166429	T1D vs Control
URS000099D184-rRNA4	-0,754659947	4,977808511	0,060960388	0,463166429	T1D vs Control
URS00006C2C4A-Y_RNA4	-1,393326764	4,927831545	0,062067246	0,463443568	T1D vs Control
URS00007125F9-rRNA4	-1,089929345	2,409823294	0,062322883	0,463443568	T1D vs Control

URS00000DE490-lncRNA4	-0,930702418	3,392102095	0,065599387	0,477221683	T1D vs Control
URS00005D36AA-lncRNA4	-1,080959568	2,523255562	0,065954879	0,477221683	T1D vs Control
URS0000391360-lncRNA4	-1,091771304	2,256356542	0,066697286	0,477221683	T1D vs Control
URS000071ED2F-tRNA4	-1,157933107	2,991616318	0,067378984	0,477221683	T1D vs Control
URS00006BBD5F-Y_RNA4	-1,046410617	3,385968133	0,067589337	0,477221683	T1D vs Control
URS000097171C-SRP_RNA4	0,992544073	2,924722071	0,068671045	0,480010602	T1D vs Control
URS000093FF31-rRNA4	-0,962772568	2,038384139	0,071670592	0,496017268	T1D vs Control
URS00004AE46A-lncRNA4	-0,991951216	3,574152246	0,074348907	0,50950869	T1D vs Control
URS00006D1A54-Y_RNA4	-0,908266939	3,828527713	0,077091068	0,518204586	T1D vs Control
URS000096E1E3-rRNA4	-1,037348514	2,495902245	0,077100539	0,518204586	T1D vs Control
URS00007D24CA-lncRNA4	-1,319635585	2,948889293	0,080093222	0,533192018	T1D vs Control
URS0000997FE9-rRNA4	-0,931353655	2,876398415	0,082021458	0,537192601	T1D vs Control
URS0000782759-lncRNA4	0,955402888	2,907193443	0,083133372	0,537192601	T1D vs Control
URS00001D0305-lncRNA4	-0,310887196	7,373693454	0,084354706	0,537192601	T1D vs Control
URS00007E2F7C-lncRNA4	-1,051199867	3,279760012	0,084494829	0,537192601	T1D vs Control
URS000060A110-antisense4	-0,794364073	1,367875278	0,084536747	0,537192601	T1D vs Control
URS000049BB82-lncRNA4	-0,83810689	4,039331254	0,087805256	0,55101661	T1D vs Control
URS00005BE013-lncRNA4	1,238563641	2,759573682	0,088288784	0,55101661	T1D vs Control
URS00007BE6D3-lncRNA4	2,339484869	10,22836282	0,093243457	0,576789173	T1D vs Control
URS00009A0DCD-rRNA4	-0,72993051	5,942865971	0,09441641	0,578921669	T1D vs Control
URS000063CFC9-Y_RNA4	-0,981653062	2,480380401	0,096682256	0,584357848	T1D vs Control
URS000034EAB6-lncRNA4	-1,010754199	2,280705495	0,097823704	0,584357848	T1D vs Control
URS000050E9EC-lncRNA4	-0,850139712	3,382080638	0,098655686	0,584357848	T1D vs Control
URS0000AAD5AA-lncRNA4	1,327707316	10,83600588	0,0986999	0,584357848	T1D vs Control
URS0000488EA7-lncRNA4	-0,979249064	2,312667922	0,099775123	0,584357848	T1D vs Control
URS000002E930-antisense4	-1,100544098	4,041839683	0,100318944	0,584357848	T1D vs Control
URS00006C14B2-tRNA4	-1,089509368	3,304706256	0,103031232	0,592155489	T1D vs Control
URS00009612D1-rRNA4	-0,695860441	5,646389432	0,104907887	0,592155489	T1D vs Control
URS0000A9CA30-lncRNA4	-0,905689015	3,103219008	0,105013236	0,592155489	T1D vs Control
URS00006529EE-Y_RNA4	-0,959439282	3,147668241	0,105046181	0,592155489	T1D vs Control
URS0000726FAB-rRNA4	0,238626358	13,91320925	0,106597955	0,596095765	T1D vs Control
URS00006C48EB-Y_RNA]4	1,051297544	2,788995838	0,110508854	0,613061025	T1D vs Control
URS0000145C5E-tRNA4	-0,99450054	2,892697082	0,112256161	0,613398584	T1D vs Control
URS000035D229-lncRNA4	1,284694076	3,593153184	0,112916386	0,613398584	T1D vs Control
URS0000134A86-antisense4	1,036138232	3,351781773	0,113232301	0,613398584	T1D vs Control
URS00001DFAE4-lncRNA4	1,097547162	3,289114858	0,114248412	0,613398584	T1D vs Control
URS00000E0DE1-antisense4	-0,851447552	3,104520142	0,115299456	0,613398584	T1D vs Control
URS00000A586F-lncRNA4	-0,498755823	0,916847513	0,115834926	0,613398584	T1D vs Control
URS00001D0B96-lncRNA4	-0,203811748	10,38627035	0,118022605	0,62028422	T1D vs Control
URS000039557B-antisense4	-0,826331227	1,694045712	0,121622099	0,629418791	T1D vs Control
URS0000696377-Y_RNA4	0,859220807	2,848796511	0,122422	0,629418791	T1D vs Control
URS00004F6629-lncRNA4	0,60821565	1,127896398	0,122462025	0,629418791	T1D vs Control
URS000053D4AB-snoRNA4	-0,826754276	1,689585927	0,128471177	0,651017484	T1D vs Control
URS0000629ECF-Y_RNA4	-0,558887595	4,37154222	0,128527057	0,651017484	T1D vs Control
URS000056B96A-antisense4	-0,756436037	2,621722386	0,130743556	0,657480185	T1D vs Control
URS00007D436A-antisense4	-0,817946041	2,239346559	0,1343309	0,670694996	T1D vs Control
URS000022DD4A-tRNA4	-0,340318762	9,64520168	0,139219582	0,686245311	T1D vs Control
URS0000204428-lncRNA4	-0,877578573	2,674128924	0,140453073	0,686245311	T1D vs Control
URS0000A9F786-lncRNA4	0,981007455	3,099717163	0,142407967	0,686245311	T1D vs Control
URS0000098211-lncRNA4	-0,891468786	2,630527968	0,142498251	0,686245311	T1D vs Control
URS0000687FC3-Y_RNA4	-0,414160714	4,184219214	0,144923936	0,686245311	T1D vs Control
URS000056B231-lncRNA4	0,861761871	4,1586305	0,145017889	0,686245311	T1D vs Control
URS00004D7012-lncRNA4	-1,036106089	2,906892949	0,145117881	0,686245311	T1D vs Control
URS0000166FF8-lncRNA4	-0,698023488	4,546451597	0,145678129	0,686245311	T1D vs Control
URS0000986F6F-rRNA4	-0,649968171	5,818355153	0,146798063	0,686245311	T1D vs Control
URS00003F2105-lncRNA4	0,822743249	2,406932528	0,149675842	0,686245311	T1D vs Control
URS000064FE59-rRNA4	0,197045858	11,09750364	0,150381552	0,686245311	T1D vs Control
URS000049B61C-antisense4	-0,319067835	6,879627508	0,150658144	0,686245311	T1D vs Control
URS00000EB76F-lncRNA4	-0,837361224	2,382363845	0,151038121	0,686245311	T1D vs Control
URS0000767DAE-lncRNA4	-0,504891013	5,637395825	0,151459564	0,686245311	T1D vs Control
URS00006C06E6-Y_RNA4	-0,818476519	1,912207487	0,152171707	0,686245311	T1D vs Control
URS000017CF23-antisense4	-0,775594262	2,666340903	0,155894418	0,691860304	T1D vs Control
URS0000371842-lncRNA4	-0,806851219	2,020987369	0,156044542	0,691860304	T1D vs Control
URS00006EB1B5-Y_RNA4	-0,733608688	4,084821132	0,156746318	0,691860304	T1D vs Control
URS00004A68E9-antisense4	-0,59230155	3,654195066	0,157464429	0,691860304	T1D vs Control
URS00006D4DB0-snRNA4	-0,898262334	3,391553507	0,15922325	0,691860304	T1D vs Control

URS00006FF680-Y_RNA4	-0,816641794	2,916835355	0,159355521	0,691860304	T1D vs Control
URS00005BC8EC-lncRNA4	-1,062860433	5,143820106	0,161748627	0,697915373	T1D vs Control
URS00003475A2-lncRNA4	-0,458382753	6,073704566	0,163732848	0,702142704	T1D vs Control
URS0000446770-snoRNA4	-0,840327774	2,104005039	0,1673161	0,713133865	T1D vs Control
URS000029E713-antisense4	-0,678149063	4,061405427	0,170609722	0,719626145	T1D vs Control
URS00005508F6-antisense4	-0,757660687	3,129098015	0,171598926	0,719626145	T1D vs Control
URS00002E367B-antisense4	-0,758958586	1,915955023	0,173153994	0,719626145	T1D vs Control
URS000048EB2F-antisense4	-0,765001909	2,157167592	0,174162835	0,719626145	T1D vs Control
URS0000A7BA37-lncRNA4	0,724547035	1,699099075	0,174707439	0,719626145	T1D vs Control
URS0000A85AEE-lncRNA4	-0,883449684	3,200285639	0,175725925	0,719626145	T1D vs Control
URS00001662B7-lncRNA4	0,875802301	2,511216373	0,176045881	0,719626145	T1D vs Control
URS00004BE455-lncRNA4	-0,55990392	5,228003516	0,181320685	0,736878832	T1D vs Control
URS0000630B8A-tRNA4	0,621081368	5,357715317	0,183901304	0,741008925	T1D vs Control
URS00006F5B12-Y_RNA4	-0,785366409	5,757189507	0,185164237	0,741008925	T1D vs Control
URS000038F4B2-tRNA4	-0,80721446	2,156583414	0,186152509	0,741008925	T1D vs Control
URS0000189042-lncRNA4	-0,79185742	1,901016294	0,186577354	0,741008925	T1D vs Control
URS000063E4FD-tRNA4	0,806519269	3,061724682	0,188558386	0,741093579	T1D vs Control
URS000077A114-lncRNA4	-0,219955448	7,694058287	0,189397417	0,741093579	T1D vs Control
URS0000941729-rRNA4	-0,637436044	4,187919105	0,189925163	0,741093579	T1D vs Control
URS0000A89523-lncRNA4	-0,773691666	2,553603804	0,190839548	0,741093579	T1D vs Control
URS0000493225-tRNA4	-0,363466982	7,026724216	0,195296664	0,754211979	T1D vs Control
URS00002AD6F3-antisense4	-0,617872319	1,553235742	0,199006465	0,759022757	T1D vs Control
URS000032B6B6-snRNA4	0,270470962	8,295722512	0,199621805	0,759022757	T1D vs Control
URS00000C18F2-tRNA4	-0,554988754	6,19082938	0,202377702	0,759022757	T1D vs Control
URS0000977FD2-SRP_RNA4	0,711813117	2,037495207	0,203361898	0,759022757	T1D vs Control
URS0000637E4A-tRNA4	-0,688578728	1,552702938	0,205285185	0,759022757	T1D vs Control
URS00007080E9-Y_RNA4	-0,932467612	2,936384424	0,206317168	0,759022757	T1D vs Control
URS00006C0715-rRNA4	0,181349779	11,18891955	0,20715324	0,759022757	T1D vs Control
URS0000249329-lncRNA4	-0,636853738	1,740243504	0,207750949	0,759022757	T1D vs Control
URS0000086FDD-antisense4	0,84699525	2,945683418	0,209793267	0,759022757	T1D vs Control
URS00006CB0C3-Y_RNA4	-0,297562454	5,291889769	0,210391571	0,759022757	T1D vs Control
URS000072C165-Y_RNA4	-0,323727784	5,305209475	0,211567114	0,759022757	T1D vs Control
URS0000257C23-lncRNA4	0,835091404	4,584581553	0,211822561	0,759022757	T1D vs Control
URS00000DAC34-lncRNA4	-0,693043157	2,636836099	0,212752523	0,759022757	T1D vs Control
URS0000931B54-rRNA4	0,096630547	14,46696978	0,214018683	0,759022757	T1D vs Control
URS000099C20E-rRNA4	-0,80730395	3,326722503	0,214439648	0,759022757	T1D vs Control
URS0000907730-rRNA4	-0,426979331	6,755248399	0,217086605	0,759022757	T1D vs Control
URS00005F1728-lncRNA4	0,654451414	2,661157063	0,217666854	0,759022757	T1D vs Control
URS0000AAECF4-lncRNA4	-0,564127298	3,702161679	0,21884099	0,759022757	T1D vs Control
URS000020220C-antisense4	-0,686192396	2,749759905	0,219307508	0,759022757	T1D vs Control
URS00000E9A71-lncRNA4	0,666614489	3,823249127	0,220042716	0,759022757	T1D vs Control
URS00006D74B2-tRNA4	-0,564456783	4,411009625	0,221250285	0,759022757	T1D vs Control
URS00005CEC24-lncRNA4	-0,734270342	2,918669315	0,224306322	0,759022757	T1D vs Control
URS00003DEE5B-lncRNA4	0,661957378	3,256575112	0,225507495	0,759022757	T1D vs Control
URS00006E1108-Y_RNA4	-0,410134631	4,049124964	0,225538521	0,759022757	T1D vs Control
URS0000928682-rRNA4	-0,155100732	11,56053599	0,225725541	0,759022757	T1D vs Control
URS0000A9525F-antisense4	-0,656389246	2,995531648	0,226502136	0,759022757	T1D vs Control
URS00003A72DC-antisense4	0,656992431	3,084869652	0,22660742	0,759022757	T1D vs Control
URS0000701607-Y_RNA4	-0,672451661	3,386959123	0,22791387	0,759022757	T1D vs Control
URS00007BC71B-lncRNA4	0,718747845	1,944342872	0,229138403	0,759022757	T1D vs Control
URS0000391A04-lncRNA4	-0,508173698	5,482878094	0,229162568	0,759022757	T1D vs Control
URS00003C2ECD-antisense4	-0,791542453	2,559377792	0,230204327	0,759022757	T1D vs Control
URS00006F0FC0-tRNA4	-0,465045529	5,435006769	0,231536513	0,75983109	T1D vs Control
URS00006FC298-tRNA4	-0,739129445	2,919601035	0,233335029	0,762155072	T1D vs Control
URS00006ABCCE-tRNA4	0,886866358	11,64642261	0,235408991	0,765352952	T1D vs Control
URS00002D1F9F-lncRNA4	-0,600252903	3,254441607	0,237758914	0,769414262	T1D vs Control
URS000071DF37-tRNA4	-0,772627176	2,691836943	0,239124689	0,770268008	T1D vs Control
URS0000539731-antisense4	-0,600332584	2,084997113	0,241832892	0,77541831	T1D vs Control
URS00009290F1-rRNA4	-0,632305391	1,980245991	0,245234338	0,782734256	T1D vs Control
URS0000417E86-lncRNA4	-0,687361543	2,54225615	0,247417356	0,783946981	T1D vs Control
URS00000B6370-lncRNA4	-0,515834136	3,6717613	0,249599644	0,783946981	T1D vs Control
URS00000D1D87-lncRNA4	-0,595123691	1,722539329	0,249813745	0,783946981	T1D vs Control
URS00002840A7-lncRNA4	0,65221099	2,142345929	0,252185764	0,783946981	T1D vs Control
URS00005B6D58-lncRNA4	0,554943316	5,324374408	0,252342006	0,783946981	T1D vs Control
URS0000016595-lncRNA4	-0,677691298	2,839797195	0,252343449	0,783946981	T1D vs Control
URS00003C98B0-lncRNA4	-0,683514633	3,045496821	0,254469154	0,786518507	T1D vs Control

URS00004DA951-rRNA4	0,368136379	10,73867185	0,255421604	0,786518507	T1D vs Control
URS000063455F-rRNA4	-0,629940572	2,601127664	0,257140712	0,788339288	T1D vs Control
URS0000605144-antisense4	-0,639443569	2,730860041	0,261595253	0,798493806	T1D vs Control
URS00005E51DB-lncRNA4	0,533778991	4,114269415	0,265629737	0,802985703	T1D vs Control
URS0000042FD3-lncRNA4	-0,562496366	3,024717754	0,266314186	0,802985703	T1D vs Control
URS0000478C87-lncRNA4	-0,597391152	2,830369346	0,26881007	0,802985703	T1D vs Control
URS0000640661-Y_RNA4	-0,526904239	11,13979054	0,270566614	0,802985703	T1D vs Control
URS000064E10F-tRNA4	-0,640824857	3,013775094	0,272029847	0,802985703	T1D vs Control
URS0000271FCA-antisense4	-0,495744634	5,084020409	0,273511598	0,802985703	T1D vs Control
URS0000704D22-rRNA4	0,197740387	14,1067509	0,273533893	0,802985703	T1D vs Control
URS00000FB60D-tRNA4	-0,629014589	3,59514453	0,273820116	0,802985703	T1D vs Control
URS0000672E5A-rRNA4	-0,395447017	5,608838646	0,274258151	0,802985703	T1D vs Control
URS000064B6FC-tRNA4	0,686184459	3,113360192	0,274554482	0,802985703	T1D vs Control
URS00000A7F2D-lncRNA4	-0,577607432	2,135027243	0,277149492	0,807197895	T1D vs Control
URS000096D31D-rRNA4	-0,202136124	10,50581429	0,283641707	0,815569806	T1D vs Control
URS000024383A-lncRNA4	0,57750777	1,637698963	0,283848863	0,815569806	T1D vs Control
URS0000AAB7F4-lncRNA4	-0,593788493	3,962630959	0,284488958	0,815569806	T1D vs Control
URS00004DDD71-lncRNA4	-0,381255727	5,854746665	0,284691034	0,815569806	T1D vs Control
URS0000188F7D-scRNA4	1,626050482	6,577829264	0,286471448	0,81592471	T1D vs Control
URS0000ABD7E8-rRNA4	0,977354549	10,98616314	0,287219857	0,81592471	T1D vs Control
URS0000918BC5-rRNA4	0,930587234	3,816571784	0,288316743	0,81592471	T1D vs Control
URS000075D48E-lncRNA4	-0,517395304	1,602010302	0,292341315	0,823978141	T1D vs Control
URS00006D23E9-snRNA4	-0,585208756	3,580811892	0,295979517	0,827480121	T1D vs Control
URS00006D1C46-snRNA4	-0,491347017	6,026449126	0,297368739	0,827480121	T1D vs Control
URS00000CCACD-antisense4	0,736084787	4,642488599	0,29826192	0,827480121	T1D vs Control
URS00009AAC46-rRNA4	0,188390335	9,797442669	0,299307779	0,827480121	T1D vs Control
URS00008BA2B6-lncRNA4	-0,540523278	2,826253026	0,302893121	0,827480121	T1D vs Control
URS000070E3CE-rRNA4	0,536608116	4,081177344	0,303547506	0,827480121	T1D vs Control
URS000075DF54-lncRNA4	0,336603463	6,14755677	0,305639022	0,827480121	T1D vs Control
URS000066C003-Y_RNA]4	-1,182974005	5,557417709	0,307202568	0,827480121	T1D vs Control
URS0000543B4D-lncRNA4	0,611295383	2,605909076	0,307413024	0,827480121	T1D vs Control
URS000005AEAB-tRNA4	0,470428713	4,48776437	0,307835214	0,827480121	T1D vs Control
URS00002901EC-tRNA4	-0,593768619	2,043121881	0,308044065	0,827480121	T1D vs Control
URS0000ABD7D5-rRNA4	0,094861056	13,17747017	0,308246131	0,827480121	T1D vs Control
URS000034E9D0-misc_RNA]4	0,848143053	3,097582218	0,308973264	0,827480121	T1D vs Control
URS0000717DFB-Y_RNA4	-0,523725625	2,95584216	0,314166276	0,827658558	T1D vs Control
URS00001823EB-lncRNA4	-0,615455992	3,483455808	0,314206269	0,827658558	T1D vs Control
URS00001E9163-lncRNA4	-0,560701995	2,764922404	0,315510422	0,827658558	T1D vs Control
URS000034AAC2-tRNA4	0,539797478	4,895139042	0,31587829	0,827658558	T1D vs Control
URS000009AC8B-tRNA4	-0,679212432	10,95540037	0,31604251	0,827658558	T1D vs Control
URS0000669D0F-snRNA4	0,60477188	2,759909955	0,317093367	0,827658558	T1D vs Control
URS00000B8842-lncRNA4	-0,460829765	4,912142401	0,317460265	0,827658558	T1D vs Control
URS000061F57C-tRNA4	-0,488345222	4,675906559	0,318512378	0,827658558	T1D vs Control
URS0000320E71-lncRNA4	-0,487281213	2,909981717	0,321546612	0,832448452	T1D vs Control
URS000041043F-antisense4	-0,549764387	1,994452875	0,328701371	0,841106221	T1D vs Control
URS0000994031-rRNA4	-0,145047584	11,10957664	0,329567407	0,841106221	T1D vs Control
URS00003CBFB8-lncRNA4	-0,522880605	2,075423342	0,330429908	0,841106221	T1D vs Control
URS00003869EC-lncRNA4	0,648755001	2,98234075	0,331421552	0,841106221	T1D vs Control
URS000007383C-antisense4	-0,52100816	2,496432723	0,33410946	0,841106221	T1D vs Control
URS0000089048-antisense4	0,511487435	1,859342356	0,335175758	0,841106221	T1D vs Control
URS0000415026-tRNA4	-0,436741836	4,426745558	0,335813973	0,841106221	T1D vs Control
URS0000784C7B-lncRNA4	0,545181724	2,086288772	0,335905689	0,841106221	T1D vs Control
URS0000676AED-precursor_RNA4	-0,555591179	2,736959381	0,337423171	0,841106221	T1D vs Control
URS00006A3E7F-snRNA4	-0,181367545	8,935478024	0,33989391	0,841106221	T1D vs Control
URS0000378BB8-lncRNA4	-0,472429231	4,18272789	0,340654633	0,841106221	T1D vs Control
URS0000AA014E-lncRNA4	0,507660683	2,880951371	0,340767138	0,841106221	T1D vs Control
URS000076E14E-lncRNA4	0,647956376	2,947769863	0,34163495	0,841106221	T1D vs Control
URS0000161EE9-lncRNA4	-0,372313039	6,078069715	0,341737005	0,841106221	T1D vs Control
URS00008BB15B-lncRNA4	-0,642139241	2,482102831	0,343055782	0,841389445	T1D vs Control
URS00001BF716-lncRNA4	-0,179741716	7,608759782	0,348131128	0,845726349	T1D vs Control
URS0000005270-rRNA4	-0,83179088	6,166410111	0,351319837	0,845726349	T1D vs Control
URS00005BB5C9-antisense4	0,495205154	1,956136305	0,351397166	0,845726349	T1D vs Control
URS0000605748-lncRNA4	-0,533266727	3,689393404	0,35328093	0,845726349	T1D vs Control
URS00002548DF-lncRNA4	-0,48521552	1,501270697	0,353823571	0,845726349	T1D vs Control
URS00002CDF5D-lncRNA4	-0,472325898	2,151438672	0,354826149	0,845726349	T1D vs Control
URS0000811D9C-antisense4	-0,337680216	0,958216717	0,355899961	0,845726349	T1D vs Control

URS0000397495-antisense4	-0,313832084	5,990008067	0,356438517	0,845726349	T1D vs Control
URS00002B0998-lncRNA4	-0,514764046	2,419217026	0,356701116	0,845726349	T1D vs Control
URS000069B369-Y_RNA4	-0,632300348	9,690811268	0,357601224	0,845726349	T1D vs Control
URS00000AD390-antisense4	0,500205904	1,744059197	0,360611317	0,845726349	T1D vs Control
URS00006CFDFE-Y_RNA4	-0,387028204	2,249644641	0,361146672	0,845726349	T1D vs Control
URS0000222FD2-tRNA4	-0,556584944	3,623751413	0,36220397	0,845726349	T1D vs Control
URS000041FE38-lncRNA4	-0,489507516	2,353684167	0,367937674	0,845726349	T1D vs Control
URS00005B2DE5-lncRNA4	-0,472624606	4,758632634	0,368319992	0,845726349	T1D vs Control
URS000038D8D3-tRNA4	-0,277766848	7,544518208	0,36837226	0,845726349	T1D vs Control
URS000022006F-lncRNA4	-0,490220546	3,198783691	0,368399088	0,845726349	T1D vs Control
URS000063A6E6-rRNA4	0,105311076	11,70425189	0,368829273	0,845726349	T1D vs Control
URS00002172B5-lncRNA4	-0,628987147	2,390444931	0,370517814	0,845726349	T1D vs Control
URS0000ABD87F-rRNA4	-0,880419097	10,81861494	0,371789398	0,845726349	T1D vs Control
URS00002200B0-lncRNA4	-0,522629483	3,176288712	0,373190972	0,845726349	T1D vs Control
URS00004F0321-tRNA4	-0,269663168	14,08130095	0,374093513	0,845726349	T1D vs Control
URS00004227BE-lncRNA4	-0,513072546	1,875905236	0,376593108	0,845726349	T1D vs Control
URS000050471D-lncRNA4	-0,548872737	2,378838415	0,377765001	0,845726349	T1D vs Control
URS00005B5F85-lncRNA4	-0,479644534	5,187781526	0,378403343	0,845726349	T1D vs Control
URS000065D78F-Y_RNA4	-0,152494468	5,875001903	0,379793386	0,845726349	T1D vs Control
URS00003875B8-lncRNA4	0,543441498	2,156965412	0,381333093	0,845726349	T1D vs Control
URS00003CAB47-antisense4	-0,503981539	3,257126248	0,382306683	0,845726349	T1D vs Control
URS00007D61AD-antisense4	0,638959779	2,769938297	0,383472165	0,845726349	T1D vs Control
URS0000990012-rRNA4	-0,282218843	8,57303772	0,383723697	0,845726349	T1D vs Control
URS0000942121-rRNA4	-0,064958719	14,48998565	0,384208304	0,845726349	T1D vs Control
URS00000081EA-snRNA4	-0,501349219	2,66468092	0,385371711	0,845726349	T1D vs Control
URS000013BA50-antisense4	0,539077132	1,971579125	0,385997182	0,845726349	T1D vs Control
URS00001A4293-lncRNA4	-0,426281771	4,616590583	0,386275386	0,845726349	T1D vs Control
URS00006BF71F-rRNA4	0,495850926	2,492314255	0,387170861	0,845726349	T1D vs Control
URS0000511A5F-lncRNA4	-0,488611404	1,837603255	0,390873247	0,848410261	T1D vs Control
URS000055C9F8-antisense4	-0,388386679	4,017918826	0,392404837	0,848410261	T1D vs Control
URS00005FFC78-lncRNA4	0,487263888	2,519542247	0,393722748	0,848410261	T1D vs Control
URS00003F41E9-lncRNA4	-0,170477237	8,007323205	0,394672411	0,848410261	T1D vs Control
URS000018267A-antisense4	0,550503579	3,83534964	0,395196714	0,848410261	T1D vs Control
URS000064F96B-Y_RNA4	0,285514872	5,069805695	0,395682039	0,848410261	T1D vs Control
URS0000006840-lncRNA4	-0,428617268	3,802540443	0,397312856	0,849301794	T1D vs Control
URS0000166229-lncRNA4	-0,435259706	2,097439644	0,400927208	0,854414994	T1D vs Control
URS0000287398-tRNA4	-0,527008068	2,440463804	0,402992978	0,856206966	T1D vs Control
URS00000A3F50-lncRNA4	0,441294875	1,749216608	0,409009292	0,861921747	T1D vs Control
URS0000031963-antisense4	-0,574002545	3,414756941	0,410237113	0,861921747	T1D vs Control
URS000076B0D1-lncRNA4	-0,460800456	3,094918128	0,411254538	0,861921747	T1D vs Control
URS000075B143-lncRNA4	-0,447281928	2,661955926	0,412040207	0,861921747	T1D vs Control
URS0000624312-Y_RNA4	-0,328079517	5,698207316	0,412352952	0,861921747	T1D vs Control
URS00000CD33E-antisense4	-0,42866338	3,630533758	0,414473516	0,861921747	T1D vs Control
URS00002C4609-lncRNA4	0,4797944	2,010894744	0,415041385	0,861921747	T1D vs Control
URS000005F65D-antisense4	0,41721878	4,947813537	0,415547394	0,861921747	T1D vs Control
URS000045FD15-lncRNA4	-0,437094984	4,004446293	0,41681302	0,861989056	T1D vs Control
URS0000432B92-antisense4	-0,147348211	7,647433512	0,418910953	0,86301919	T1D vs Control
URS0000527686-lncRNA4	-0,314091352	1,162280834	0,419780436	0,86301919	T1D vs Control
URS0000A8472C-Y_RNA4	-0,513679911	3,823381815	0,422401087	0,865860293	T1D vs Control
URS000048B807-lncRNA4	-0,395542592	4,663045601	0,426071863	0,865868219	T1D vs Control
URS000015D954-antisense4	-0,443017821	1,950160466	0,426144746	0,865868219	T1D vs Control
URS00006E19EA-Y_RNA4	0,307297443	9,597384946	0,427689925	0,865868219	T1D vs Control
URS000034E03C-antisense4	0,455891265	2,078398724	0,428549691	0,865868219	T1D vs Control
URS000067474D-tRNA4	0,488064798	5,826738993	0,430451846	0,865868219	T1D vs Control
URS000097FEDE-rRNA4	-0,411883005	3,690975927	0,433103783	0,865868219	T1D vs Control
URS0000181B59-lncRNA4	-0,534420887	2,274734234	0,433537202	0,865868219	T1D vs Control
URS00006481F8-Y_RNA4	-0,238563609	7,303708498	0,434678306	0,865868219	T1D vs Control
URS00002968BD-antisense4	0,251376535	7,356778365	0,438208036	0,865868219	T1D vs Control
URS00000347CF-lncRNA4	-0,37776123	3,718528756	0,438720406	0,865868219	T1D vs Control
URS00006F3305-rRNA4	-0,081367017	11,47052611	0,439495301	0,865868219	T1D vs Control
URS00006CC125-rRNA4	0,43763536	2,366732734	0,439508869	0,865868219	T1D vs Control
URS00002EA13A-lncRNA4	-0,426823125	2,908290455	0,439998187	0,865868219	T1D vs Control
URS00006BB04D-tRNA4	0,433163979	3,054494403	0,440344227	0,865868219	T1D vs Control
URS000023F3B4-lncRNA4	-0,361722672	3,955198744	0,441742552	0,865868219	T1D vs Control
URS00001FBD75-misc_RNA]4	0,656144833	3,307722647	0,443220451	0,865868219	T1D vs Control
URS0000A81496-Y_RNA4	0,50324232	2,190786251	0,443655196	0,865868219	T1D vs Control

URS0000A765F3-lncRNA4	-0,394519335	3,81544174	0,44470199	0,865868219	T1D vs Control
URS000090DF6D-rRNA4	-0,084936433	11,93047681	0,447990339	0,869274778	T1D vs Control
URS000040CE64-lncRNA4	-0,496854704	2,883976268	0,451618465	0,869274778	T1D vs Control
URS000090169F-rRNA4	-0,405751997	1,691620139	0,451622796	0,869274778	T1D vs Control
URS00003D2CC9-tRNA4	-0,396016401	4,271817892	0,451856094	0,869274778	T1D vs Control
URS00009407E6-rRNA4	-0,451204931	2,030446175	0,452669555	0,869274778	T1D vs Control
URS000061A10B-tRNA4	0,517922726	2,36803002	0,455888312	0,871207461	T1D vs Control
URS0000633F75-ribozyme4	-0,393422436	4,438697312	0,456439853	0,871207461	T1D vs Control
URS0000192C05-lncRNA4	-0,399530428	2,748839188	0,458161875	0,871207461	T1D vs Control
URS00000AF0EF-lncRNA4	0,41413302	3,804446661	0,45881193	0,871207461	T1D vs Control
URS00009840C1-rRNA4	0,296013752	6,418341272	0,460283955	0,871207461	T1D vs Control
URS00002D2D8F-misc_RNA]4	-0,460423867	1,939760416	0,461647266	0,871207461	T1D vs Control
URS000065DC2A-tRNA4	-0,306378234	4,471494404	0,462400527	0,871207461	T1D vs Control
URS000051CFB0-lncRNA4	-0,661765422	3,801776289	0,467546192	0,878534377	T1D vs Control
URS000050FA69-lncRNA4	-0,37832694	3,930388911	0,469831291	0,880044481	T1D vs Control
URS00005580B2-lncRNA4	-0,474889612	3,442360628	0,472063637	0,880044481	T1D vs Control
URS00005BB09E-lncRNA4	-0,280921119	6,047129544	0,473057195	0,880044481	T1D vs Control
URS0000093DF5-lncRNA4	-0,396081307	2,157430584	0,47636834	0,880044481	T1D vs Control
URS000036E063-antisense4	0,084540382	10,32347827	0,476765044	0,880044481	T1D vs Control
URS00005B7465-tRNA4	-0,399634736	1,847741502	0,478730443	0,880044481	T1D vs Control
URS000076DAC1-lncRNA4	0,292300067	5,661423845	0,479062264	0,880044481	T1D vs Control
URS0000A7F61B-lncRNA4	-0,377508752	2,242573569	0,479566551	0,880044481	T1D vs Control
URS00007CB156-lncRNA4	-0,144349356	7,841069642	0,479680897	0,880044481	T1D vs Control
URS000012730D-lncRNA4	0,40655123	3,696451386	0,48428317	0,884107597	T1D vs Control
URS00004F2E54-lncRNA4	-0,398022022	1,792153167	0,485466475	0,884107597	T1D vs Control
URS000096B970-rRNA4	-0,093497711	10,27519744	0,485727883	0,884107597	T1D vs Control
URS0000811AFB-antisense4	0,377998215	2,040750323	0,489527201	0,884107597	T1D vs Control
URS00002E81F5-lncRNA4	-0,34193498	4,084000104	0,491329423	0,884107597	T1D vs Control
URS00006A2BF7-Y_RNA4	-0,404498659	10,1864015	0,491714595	0,884107597	T1D vs Control
URS000091563C-rRNA4	-0,080319853	10,26950568	0,492757742	0,884107597	T1D vs Control
URS00002B7986-antisense4	-0,358504381	1,782132427	0,492871848	0,884107597	T1D vs Control
URS00003C9A26-tRNA4	0,12739914	8,822917147	0,493673413	0,884107597	T1D vs Control
URS0000028D07-lncRNA4	0,343691266	5,403821621	0,495085674	0,884107597	T1D vs Control
URS000015BB33-lncRNA4	0,338812897	3,745978664	0,496873708	0,884107597	T1D vs Control
URS00006D56C0-Y_RNA4	-0,268479999	4,210228585	0,49776153	0,884107597	T1D vs Control
URS000052A6A6-lncRNA4	-0,360702239	3,111397823	0,499254388	0,884107597	T1D vs Control
URS00006F7C66-rRNA4	-0,379520026	1,804743197	0,499603006	0,884107597	T1D vs Control
URS0000A8428E-Y_RNA4	0,524256925	5,101971983	0,504239153	0,886601466	T1D vs Control
URS00009C6074-antisense4	-0,42137333	2,11618012	0,507389699	0,886601466	T1D vs Control
URS00001F3EF6-lncRNA4	-0,453402613	2,384806636	0,50759395	0,886601466	T1D vs Control
URS000030BAD5-tRNA4	0,173721615	9,963231125	0,50818204	0,886601466	T1D vs Control
URS0000611F3E-lncRNA4	-0,36188521	2,798037489	0,509949045	0,886601466	T1D vs Control
URS00006361F3-Y_RNA4	0,492730878	4,856921385	0,510257088	0,886601466	T1D vs Control
URS000097B164-rRNA4	-0,281613738	5,947136128	0,51153538	0,886601466	T1D vs Control
URS00004D1520-lncRNA4	-0,371576492	3,203873127	0,514740603	0,886601466	T1D vs Control
URS000018BCE5-antisense4	-0,371689214	2,555076799	0,515680714	0,886601466	T1D vs Control
URS00006F4E76-Y_RNA4	0,17100881	0,766333934	0,517261066	0,886601466	T1D vs Control
URS00003B6188-lncRNA4	-0,338907632	3,075648025	0,517642691	0,886601466	T1D vs Control
URS000063FB43-tRNA4	-0,122785322	12,66119095	0,521407111	0,886601466	T1D vs Control
URS000045E276-antisense4	-0,34402583	7,301870906	0,52196148	0,886601466	T1D vs Control
URS000096196A-SRP_RNA4	0,352082687	2,043342413	0,522033828	0,886601466	T1D vs Control
URS000038EEDC-lncRNA4	-0,452943611	3,360541304	0,522152223	0,886601466	T1D vs Control
URS0000633321-snoRNA4	0,158538916	7,948494424	0,523001524	0,886601466	T1D vs Control
URS00009A5DA8-rRNA4	-0,279451294	5,880019281	0,524552397	0,886601466	T1D vs Control
URS000021BDC3-snRNA4	0,339742505	2,084780028	0,525027093	0,886601466	T1D vs Control
URS000064217E-Y_RNA4	-0,375510537	2,41296644	0,525111598	0,886601466	T1D vs Control
URS0000702B40-Y_RNA4	0,415519128	2,572703997	0,528406759	0,890015239	T1D vs Control
URS00009A050E-rRNA4	0,109195089	10,767947	0,530282916	0,891028265	T1D vs Control
URS00006AE6F1-Y_RNA4	-0,273055193	2,11506442	0,535478505	0,897030368	T1D vs Control
URS000002FD7D-lncRNA4	0,327015427	1,677824648	0,536421594	0,897030368	T1D vs Control
URS000023352F-lncRNA4	-0,313644972	2,158198536	0,543055379	0,897745595	T1D vs Control
URS000075D28F-lncRNA4	-0,340043506	2,277607756	0,54392517	0,897745595	T1D vs Control
URS00006CE1FB-rRNA4	0,59718588	5,102179845	0,545011815	0,897745595	T1D vs Control
URS000062C73B-tRNA4	-0,263159943	4,544224584	0,545164149	0,897745595	T1D vs Control
URS0000265843-antisense4	-0,31649688	2,862204159	0,545429065	0,897745595	T1D vs Control
URS0000995AA3-rRNA4	-0,196821114	8,311403687	0,545692678	0,897745595	T1D vs Control

URS0000762146-antisense4	-0,222361021	5,166011847	0,545839597	0,897745595	T1D vs Control
URS00002C9C48-lncRNA4	-0,329542236	5,358879491	0,550623663	0,903488123	T1D vs Control
URS000017D264-lncRNA4	-0,339325696	2,092743225	0,553190663	0,905574411	T1D vs Control
URS00001EE979-lncRNA4	-0,36818563	2,774714775	0,557479885	0,910463643	T1D vs Control
URS00003CA240-antisense4	-0,237553558	5,133383215	0,561529064	0,913407348	T1D vs Control
URS000013B42D-tRNA4	-0,196492601	16,60649921	0,561895794	0,913407348	T1D vs Control
URS0000543A1A-lncRNA4	-0,295310953	5,169008445	0,563830015	0,914425012	T1D vs Control
URS000033268A-lncRNA4	0,309548035	4,132479222	0,570991392	0,921966086	T1D vs Control
URS0000641C1B-Y_RNA4	-0,177100552	4,028164364	0,571117761	0,921966086	T1D vs Control
URS000000898B-lncRNA4	0,347059388	2,509136511	0,574759588	0,923465854	T1D vs Control
URS000042F13F-tRNA4	0,153901307	6,573577673	0,576516442	0,923465854	T1D vs Control
URS00000AED6F-tRNA4	0,258158829	6,83625311	0,578009446	0,923465854	T1D vs Control
URS00005DB87D-tRNA4	-0,110433798	7,975358739	0,579277688	0,923465854	T1D vs Control
URS000075D341-rRNA4	0,508353738	3,481242239	0,57969251	0,923465854	T1D vs Control
URS000060D3B6-lncRNA4	-0,415444026	4,588963786	0,581029542	0,923465854	T1D vs Control
URS0000690F87-snRNA4	-0,382971404	3,114315559	0,583230139	0,923465854	T1D vs Control
URS000000513C-antisense4	0,214947665	4,840741267	0,583432347	0,923465854	T1D vs Control
URS000003B6E2-lncRNA4	0,415923938	2,652355198	0,585365047	0,923465854	T1D vs Control
URS00007DFA49-antisense4	-0,383138655	2,453720457	0,587310216	0,923465854	T1D vs Control
URS00005B30A9-tRNA4	0,355079876	2,750819629	0,587877984	0,923465854	T1D vs Control
URS0000759CF4-lncRNA4	-0,303253448	1,845197414	0,587900294	0,923465854	T1D vs Control
URS00006BFB96-tRNA4	-0,305348175	2,209793928	0,596305837	0,932002783	T1D vs Control
URS000070792C-rRNA4	-0,292689024	1,756278837	0,598207971	0,932002783	T1D vs Control
URS00006D9244-tRNA4	-0,328894786	3,430512932	0,598543497	0,932002783	T1D vs Control
URS0000177135-lncRNA4	0,435722268	3,040442935	0,598668454	0,932002783	T1D vs Control
URS000012C80D-lncRNA4	-0,285914488	2,726619231	0,60453183	0,939039443	T1D vs Control
URS00006F135B-Y_RNA4	-0,190216055	3,60198893	0,608401415	0,940665862	T1D vs Control
URS00009843EB-rRNA4	0,092982472	12,28502234	0,61037243	0,940665862	T1D vs Control
URS00004AE57B-lncRNA4	-0,291401367	2,181566953	0,611785851	0,940665862	T1D vs Control
URS00004F482C-lncRNA4	-0,293788457	2,9888845	0,612012139	0,940665862	T1D vs Control
URS000031A1AE-antisense4	0,314396762	2,086098634	0,612307535	0,940665862	T1D vs Control
URS00000D6053-lncRNA4	-0,414042804	5,001351593	0,616432652	0,944926368	T1D vs Control
URS00006428FD-Y_RNA4	-0,212368867	6,936586919	0,624335984	0,953638673	T1D vs Control
URS000029CCC5-tRNA4	-0,265758583	2,732517708	0,624844796	0,953638673	T1D vs Control
URS000069466F-Y_RNA4	-0,137981393	7,597826288	0,627113657	0,9546312	T1D vs Control
URS00004BF687-tRNA4	-0,297832375	13,4414794	0,628226541	0,9546312	T1D vs Control
URS000011812A-antisense4	-0,232607663	4,132007293	0,629814247	0,954967807	T1D vs Control
URS000075BB81-lncRNA4	-0,261400023	2,83062659	0,631841282	0,955378036	T1D vs Control
URS00009A7848-rRNA4	0,059688948	11,54132926	0,632818356	0,955378036	T1D vs Control
URS00000AD0C2-lncRNA4	-0,260606852	1,982015887	0,635356985	0,956077827	T1D vs Control
URS00003F5471-lncRNA4	-0,246688009	3,0793045	0,638532955	0,956077827	T1D vs Control
URS0000399BDA-lncRNA4	-0,253385677	5,117165985	0,64167858	0,956077827	T1D vs Control
URS000098604A-rRNA4	-0,260845683	1,872688165	0,642878136	0,956077827	T1D vs Control
URS000052A1C9-tRNA4	-0,237346332	4,427113529	0,644869849	0,956077827	T1D vs Control
URS000071E736-Y_RNA4	-0,279407965	2,593633412	0,649577737	0,956077827	T1D vs Control
URS00008116E3-lncRNA4	-0,227726019	4,000279228	0,650774293	0,956077827	T1D vs Control
URS000076FC5A-lncRNA4	-0,279740736	2,343917503	0,651459331	0,956077827	T1D vs Control
URS0000576D5D-lncRNA4	0,245250624	1,73318224	0,653087135	0,956077827	T1D vs Control
URS000059900F-tRNA4	0,252768628	3,409718688	0,653229333	0,956077827	T1D vs Control
URS00002750C5-lncRNA4	0,239623683	1,848936086	0,653346596	0,956077827	T1D vs Control
URS000075AF5F-antisense4	-0,267141358	2,789120304	0,660825441	0,956077827	T1D vs Control
URS0000918AFB-rRNA4	0,107307341	8,610055402	0,661440068	0,956077827	T1D vs Control
URS000094F5D5-rRNA4	-0,199563383	7,000982595	0,661906887	0,956077827	T1D vs Control
URS0000759AE0-lncRNA4	-0,228057279	1,586466937	0,662650075	0,956077827	T1D vs Control
URS0000172E58-lncRNA4	-0,232383964	3,538272714	0,662652822	0,956077827	T1D vs Control
URS0000228E94-lncRNA4	0,254098623	2,335958891	0,663225817	0,956077827	T1D vs Control
URS0000381123-lncRNA4	-0,244330743	2,471894131	0,663355157	0,956077827	T1D vs Control
URS000028E102-lncRNA4	-0,243037696	2,546561157	0,663467785	0,956077827	T1D vs Control
URS000037D0FB-tRNA4	-0,293625273	2,792154767	0,663865918	0,956077827	T1D vs Control
URS0000AA0C30-lncRNA4	0,237983439	3,233082441	0,664419797	0,956077827	T1D vs Control
URS00003ADE1B-lncRNA4	-0,2523906	2,400792438	0,664722772	0,956077827	T1D vs Control
URS0000653BD1-Y_RNA4	-0,220345466	4,226797259	0,667522732	0,956077827	T1D vs Control
URS0000502C74-tRNA4	0,075810078	14,17701257	0,667540116	0,956077827	T1D vs Control
URS00004CE099-lncRNA4	-0,351190806	3,298450018	0,668812742	0,956077827	T1D vs Control
URS000067843B-Y_RNA4	0,166857604	5,925250166	0,67125456	0,956077827	T1D vs Control
URS00002AD8DA-lncRNA4	-0,222166738	2,376170134	0,672867107	0,956077827	T1D vs Control

URS0000233E9C-lncRNA4	0,3228317	2,822247929	0,674494891	0,956077827	T1D vs Control
URS00009C60C3-lncRNA4	0,319101865	3,075209684	0,674982094	0,956077827	T1D vs Control
URS000066AF0D-Y_RNA4	-0,23160325	12,73751355	0,675416178	0,956077827	T1D vs Control
URS0000397210-lncRNA4	-0,225468083	2,372225381	0,675683042	0,956077827	T1D vs Control
URS000038D781-lncRNA4	-0,24826971	2,120835096	0,681649707	0,962572011	T1D vs Control
URS00001D4EE9-tRNA4	-0,269822793	2,839543714	0,684541327	0,962801087	T1D vs Control
URS000053EAB5-lncRNA4	0,211939089	3,036408354	0,685580673	0,962801087	T1D vs Control
URS000090AA7A-rRNA4	0,236396415	2,615865166	0,686210887	0,962801087	T1D vs Control
URS00006D4008-tRNA4	-0,277125934	2,742010752	0,688069426	0,962801087	T1D vs Control
URS00009C6137-lncRNA4	0,205074778	3,379050945	0,688698917	0,962801087	T1D vs Control
URS000062F68C-rRNA4	0,051211262	10,93938784	0,699658655	0,972160106	T1D vs Control
URS0000038397-lncRNA4	0,208521294	5,458381823	0,701385661	0,972160106	T1D vs Control
URS0000700D52-tRNA4	-0,224747739	2,144408558	0,702645289	0,972160106	T1D vs Control
URS00008120F8-lncRNA4	-0,217378568	1,863766293	0,703629309	0,972160106	T1D vs Control
URS000019B78E-misc_RNA]4	0,310983948	3,588733066	0,706298539	0,972160106	T1D vs Control
URS000075A823-rRNA4	0,507916329	6,786069906	0,706679107	0,972160106	T1D vs Control
URS00004C82E1-antisense4	0,228569674	4,084336348	0,709865553	0,972160106	T1D vs Control
URS0000395C70-lncRNA4	0,263256495	2,694736211	0,711840474	0,972160106	T1D vs Control
URS00006772C0-tRNA4	-0,223926176	2,278759881	0,713299851	0,972160106	T1D vs Control
URS00005AAAF0-antisense4	-0,204671862	3,447551336	0,714430666	0,972160106	T1D vs Control
URS00007062F7-Y_RNA4	-0,140711841	4,160857713	0,716053082	0,972160106	T1D vs Control
URS00009C6042-lncRNA4	0,105790478	6,706154397	0,716250584	0,972160106	T1D vs Control
URS00002D33E9-lncRNA4	0,193916848	2,231641646	0,716310284	0,972160106	T1D vs Control
URS0000594305-lncRNA4	-0,194749043	1,940137107	0,716589774	0,972160106	T1D vs Control
URS000020AD62-lncRNA4	0,192909544	1,816448524	0,717653289	0,972160106	T1D vs Control
URS000064C567-Y_RNA4	0,084743756	5,523874961	0,71922336	0,972160106	T1D vs Control
URS0000593A4A-lncRNA4	0,21695169	3,380617038	0,719271349	0,972160106	T1D vs Control
URS000075BA00-lncRNA4	-0,451080183	7,953608312	0,720427661	0,972160106	T1D vs Control
URS00002CFBEA-lncRNA4	0,179613147	2,003500281	0,722668983	0,973305624	T1D vs Control
URS00006744D5-tRNA4	0,225434852	2,786516677	0,725317488	0,974270057	T1D vs Control
URS000047AE74-lncRNA4	-0,200819639	2,522838605	0,726172675	0,974270057	T1D vs Control
URS00001D081D-antisense4	0,192768315	1,824716399	0,72799234	0,974840318	T1D vs Control
URS00006005A4-lncRNA4	0,23382024	2,906455771	0,734870525	0,980068867	T1D vs Control
URS00001A72CE-tRNA4	-0,19258552	4,122188898	0,736960113	0,980068867	T1D vs Control
URS000024E9CC-lncRNA4	0,200741203	2,021333988	0,737188156	0,980068867	T1D vs Control
URS000064506B-tRNA4	-0,214671145	2,909810805	0,737505328	0,980068867	T1D vs Control
URS000013899F-tRNA4	0,160170106	4,501396588	0,741240052	0,983162801	T1D vs Control
URS000047C79B-tRNA4	0,051961083	8,413478971	0,750983607	0,990332027	T1D vs Control
URS0000716B70-tRNA4	-0,162328974	3,560279134	0,751495693	0,990332027	T1D vs Control
URS00003AD33F-lncRNA4	-0,194452508	3,336093709	0,753648693	0,990332027	T1D vs Control
URS000006D0D7-lncRNA4	-0,174353037	2,152173172	0,754184437	0,990332027	T1D vs Control
URS00006EBF05-misc_RNA]4	0,282098938	3,622681484	0,75812281	0,990332027	T1D vs Control
URS00000734D4-lncRNA4	-0,148124447	2,320562453	0,759057146	0,990332027	T1D vs Control
URS0000063647-antisense4	-0,091809865	6,169603323	0,760679616	0,990332027	T1D vs Control
URS0000A76F22-lncRNA4	-0,168518724	2,591384678	0,760997388	0,990332027	T1D vs Control
URS0000197DBF-antisense4	-0,159827822	3,806884628	0,761977118	0,990332027	T1D vs Control
URS00002A865C-lncRNA4	-0,160175799	1,751381992	0,763832976	0,990332027	T1D vs Control
URS00005A57D3-lncRNA4	0,174997482	2,579526429	0,765345535	0,990332027	T1D vs Control
URS0000626233-Y_RNA4	-0,11417062	4,209755669	0,768207119	0,990332027	T1D vs Control
URS000022CCD7-lncRNA4	0,079958309	6,400235397	0,770069421	0,990332027	T1D vs Control
URS00005C220A-antisense4	-0,187644551	2,894185806	0,772708181	0,990332027	T1D vs Control
URS00003D4983-antisense4	0,16374453	1,851867325	0,772819629	0,990332027	T1D vs Control
URS000047A7F4-rRNA4	-0,272030116	4,287976289	0,775633882	0,990332027	T1D vs Control
URS00006729E8-Y_RNA4	-0,086168151	0,88430075	0,776083199	0,990332027	T1D vs Control
URS00004B48CD-lncRNA4	-0,158450875	1,80838911	0,778135924	0,990332027	T1D vs Control
URS00005C7D80-antisense4	-0,164386319	2,279201521	0,778416134	0,990332027	T1D vs Control
URS000070B37B-tRNA4	-0,051747749	9,295263071	0,778588279	0,990332027	T1D vs Control
URS0000112A1A-antisense4	-0,148547522	4,257411498	0,778816917	0,990332027	T1D vs Control
URS00004C6AFE-antisense4	-0,116821916	5,444648432	0,779570961	0,990332027	T1D vs Control
URS00006952C9-Y_RNA4	-0,195590621	4,013121466	0,78151977	0,990332027	T1D vs Control
URS00004EDF08-lncRNA4	-0,116862506	4,609717254	0,782018112	0,990332027	T1D vs Control
URS000095C3C7-rRNA4	0,124294773	4,667191645	0,785003731	0,990332027	T1D vs Control
URS00006C8EDF-tRNA4	0,162659037	3,590220694	0,788478991	0,990332027	T1D vs Control
URS00001AF592-antisense4	-0,166242636	3,230887811	0,788905089	0,990332027	T1D vs Control
URS000075AD80-snRNA4	-0,159771307	2,124304339	0,790125908	0,990332027	T1D vs Control
URS00001E5F12-lncRNA4	-0,152474485	2,516521413	0,794240626	0,990332027	T1D vs Control

URS00002D40C8-tRNA4	0,062966904	7,830242615	0,795599073	0,990332027	T1D vs Control
URS0000422302-lncRNA4	-0,134436909	2,914915679	0,795774983	0,990332027	T1D vs Control
URS00000C7470-antisense4	-0,144776963	3,497403787	0,795874949	0,990332027	T1D vs Control
URS00008120D6-lncRNA4	-0,137479707	1,844896317	0,796556124	0,990332027	T1D vs Control
URS000092B92B-rRNA4	-0,349925857	5,336196426	0,799052217	0,990332027	T1D vs Control
URS00001B59BD-lncRNA4	0,066143324	6,767957763	0,799476501	0,990332027	T1D vs Control
URS0000635FFC-Y_RNA4	-0,141313733	2,322009496	0,803971271	0,990332027	T1D vs Control
URS00006CDFA7-rRNA4	-0,130724288	2,666787507	0,805492353	0,990332027	T1D vs Control
URS00001B506A-tRNA4	0,161643003	2,259860507	0,806887206	0,990332027	T1D vs Control
URS0000209048-tRNA4	-0,145676857	3,508601642	0,808787746	0,990332027	T1D vs Control
URS000055B99E-lncRNA4	-0,152691039	2,716513459	0,809054996	0,990332027	T1D vs Control
URS0000635088-tRNA4	0,116833858	10,57667068	0,809450754	0,990332027	T1D vs Control
URS0000679FAF-tRNA4	-0,110620766	5,101125315	0,810122537	0,990332027	T1D vs Control
URS00005AF1AA-antisense4	0,102057923	1,232925367	0,810642794	0,990332027	T1D vs Control
URS0000776086-lncRNA4	-0,085098793	7,320712579	0,813967466	0,990332027	T1D vs Control
URS00003AA49D-lncRNA4	0,128849275	2,72551685	0,816945935	0,990332027	T1D vs Control
URS000095D156-rRNA4	-0,094670527	6,045790564	0,817780555	0,990332027	T1D vs Control
URS0000649B00-rRNA4	-0,033159231	10,09773081	0,819040566	0,990332027	T1D vs Control
URS000014D40F-tRNA4	0,163510843	3,081622422	0,819124615	0,990332027	T1D vs Control
URS000047EBB5-tRNA4	-0,14470481	2,438958701	0,82176539	0,990332027	T1D vs Control
URS000020BB55-lncRNA4	-0,144336897	3,131770633	0,822033435	0,990332027	T1D vs Control
URS00004AFADD-lncRNA4	-0,126438807	1,695028896	0,823380062	0,990332027	T1D vs Control
URS0000590507-lncRNA4	-0,119026219	1,90331514	0,824413692	0,990332027	T1D vs Control
URS000068483A-rRNA4	-0,129068222	2,397770416	0,825799881	0,990332027	T1D vs Control
URS00006B33E0-Y_RNA4	0,088424726	14,2625662	0,828364693	0,990332027	T1D vs Control
URS00006C9A71-Y_RNA4	0,109988713	2,255341052	0,829105953	0,990332027	T1D vs Control
URS00002AECD7-lncRNA4	0,136354215	2,148873116	0,829594507	0,990332027	T1D vs Control
URS00002811B6-lncRNA4	0,109004615	3,62513136	0,833163757	0,990332027	T1D vs Control
URS00006AD70A-tRNA4	0,131467095	4,843339833	0,833617916	0,990332027	T1D vs Control
URS000019B78E-tRNA4	-0,173296863	3,458896569	0,834418021	0,990332027	T1D vs Control
URS000064D54F-tRNA4	0,11327464	2,523144202	0,835134314	0,990332027	T1D vs Control
URS00008116FE-lncRNA4	-0,114661862	2,826119493	0,836518633	0,990332027	T1D vs Control
URS0000766C83-lncRNA4	0,129089511	2,482009239	0,836559167	0,990332027	T1D vs Control
URS00001E6C0A-lncRNA4	-0,107645635	3,103912065	0,839826008	0,990332027	T1D vs Control
URS00003D279B-lncRNA4	0,10516013	3,869764687	0,84697935	0,990332027	T1D vs Control
URS00000E43DB-antisense4	-0,10117616	3,522079692	0,848825704	0,990332027	T1D vs Control
URS0000ABD82A-rRNA4	-0,278002525	6,247493519	0,851125658	0,990332027	T1D vs Control
URS000047A7F4-misc_RNA]4	0,168537732	4,238929678	0,852135165	0,990332027	T1D vs Control
URS00002C2371-lncRNA4	-0,099519148	3,691720987	0,854518238	0,990332027	T1D vs Control
URS000068F7ED-Y_RNA4	-0,091989311	6,623874775	0,854575193	0,990332027	T1D vs Control
URS000068089E-Y_RNA4	0,106646749	2,479289353	0,855587114	0,990332027	T1D vs Control
URS00003EAC96-antisense4	-0,111565213	2,99991317	0,855733191	0,990332027	T1D vs Control
URS00000C653A-lncRNA4	0,099669103	2,462989968	0,855989733	0,990332027	T1D vs Control
URS00006F4F8C-snoRNA4	0,038986487	8,039702247	0,857489363	0,990332027	T1D vs Control
URS0000780E15-lncRNA4	-0,112945013	2,764657639	0,857599317	0,990332027	T1D vs Control
URS000009738A-lncRNA4	0,105288639	2,916436163	0,859687454	0,990332027	T1D vs Control
URS00002F2DED-lncRNA4	0,093587545	4,602442219	0,859918333	0,990332027	T1D vs Control
URS00004AC036-lncRNA4	0,087215155	4,515610764	0,860325213	0,990332027	T1D vs Control
URS0000282AB2-tRNA4	-0,136514399	4,591558308	0,861452832	0,990332027	T1D vs Control
URS00002C130C-tRNA4	0,154351347	3,549564243	0,86194547	0,990332027	T1D vs Control
URS000013BB40-lncRNA4	0,099159733	2,666260296	0,862842137	0,990332027	T1D vs Control
URS00006144FC-lncRNA4	0,070824162	7,117689109	0,863303739	0,990332027	T1D vs Control
URS00006AD81D-Y_RNA4	-0,102549971	1,867732365	0,864704583	0,990332027	T1D vs Control
URS0000561169-lncRNA4	-0,095642238	3,951068111	0,864970235	0,990332027	T1D vs Control
URS00001DE9CD-antisense4	-0,098982608	3,351892383	0,866962088	0,990332027	T1D vs Control
URS00005AF005-lncRNA4	-0,091402965	3,522774737	0,867071818	0,990332027	T1D vs Control
URS00001142E8-lncRNA4	0,058991079	5,750896426	0,871421333	0,992685596	T1D vs Control
URS000075D353-lncRNA4	-0,084188997	1,653940152	0,871972755	0,992685596	T1D vs Control
URS00004AAD0A-lncRNA4	0,086882667	2,996600067	0,874620683	0,994081069	T1D vs Control
URS00008FEDF1-rRNA4	-0,090187516	2,23767686	0,881876803	0,997568837	T1D vs Control
URS00000DA554-lncRNA4	0,083517791	2,522581542	0,883335786	0,997568837	T1D vs Control
URS0000038B1E-antisense4	0,077031645	3,244363175	0,883560317	0,997568837	T1D vs Control
URS00004D4BFC-lncRNA4	0,071020646	2,209794014	0,885894113	0,997568837	T1D vs Control
URS00006C246E-snRNA4	-0,090062332	4,016094545	0,888300467	0,997568837	T1D vs Control
URS0000504A1A-lncRNA4	0,07212039	1,470603839	0,889913785	0,997568837	T1D vs Control
URS00007C72D0-lncRNA4	-0,066210365	1,699191247	0,891855528	0,997568837	T1D vs Control

URS000091CD45-rRNA4	-0,070334685	6,581914152	0,894353067	0,997568837	T1D vs Control
URS000094439F-rRNA4	-0,015315204	11,62287065	0,894949727	0,997568837	T1D vs Control
URS00001AA18A-lncRNA4	0,07027802	1,870939952	0,895833389	0,997568837	T1D vs Control
URS0000667737-rRNA4	-0,154927273	4,834926248	0,895909135	0,997568837	T1D vs Control
URS00006C900C-tRNA4	0,067939706	2,991743424	0,900147796	0,997568837	T1D vs Control
URS0000383A48-tRNA4	-0,072348908	3,229014707	0,900340655	0,997568837	T1D vs Control
URS000063B690-snRNA4	0,072295862	3,324962274	0,900424488	0,997568837	T1D vs Control
URS00006E23A8-rRNA4	0,077106928	2,840982885	0,902267347	0,997568837	T1D vs Control
URS000030C934-lncRNA4	-0,060267327	4,417263546	0,903202266	0,997568837	T1D vs Control
URS0000417A0F-tRNA4	-0,050236099	7,465949428	0,903509575	0,997568837	T1D vs Control
URS0000462D45-lncRNA4	0,06089661	3,263068544	0,905703724	0,997568837	T1D vs Control
URS00006174C2-tRNA4	-0,026226437	8,218629708	0,906698506	0,997568837	T1D vs Control
URS0000591DFC-lncRNA4	-0,060342253	1,976731906	0,90820488	0,997568837	T1D vs Control
URS000062C4DE-tRNA4	0,064417797	3,676191563	0,910310371	0,997568837	T1D vs Control
URS0000701637-rRNA4	0,065689524	2,670549892	0,913679888	0,997568837	T1D vs Control
URS0000005EDF-lncRNA4	0,055627275	4,116716413	0,914478269	0,997568837	T1D vs Control
URS0000A90D33-snRNA4	-0,065069827	3,877391031	0,917336648	0,997568837	T1D vs Control
URS00000E0E97-antisense4	-0,088757239	4,066934958	0,918626921	0,997568837	T1D vs Control
URS00001A48CC-lncRNA4	-0,05487831	2,650296854	0,919741957	0,997568837	T1D vs Control
URS00004106BA-lncRNA4	0,048295181	1,798128499	0,920683598	0,997568837	T1D vs Control
URS0000121433-tRNA4	0,074698354	4,021698317	0,920883886	0,997568837	T1D vs Control
URS0000684921-rRNA4	0,055464975	2,725738393	0,924031079	0,997568837	T1D vs Control
URS0000A827F4-antisense4	0,060618814	8,224586053	0,924424801	0,997568837	T1D vs Control
URS000099BDEE-rRNA4	-0,054524808	5,090607985	0,924584278	0,997568837	T1D vs Control
URS00003870EC-lncRNA4	-0,05529503	2,283625175	0,929037402	0,997568837	T1D vs Control
URS00009554B1-rRNA4	0,043942758	4,916492139	0,931787209	0,997568837	T1D vs Control
URS0000418239-snRNA4	0,042920351	2,184896985	0,932039878	0,997568837	T1D vs Control
URS00000D8A7A-lncRNA4	0,045784131	2,120449279	0,932838823	0,997568837	T1D vs Control
URS00005C51E2-lncRNA4	0,04458906	1,604886726	0,932877929	0,997568837	T1D vs Control
URS0000A88906-antisense4	-0,044394182	1,925012855	0,938928243	0,997568837	T1D vs Control
URS00006D484A-rRNA4	-0,007549677	11,67221899	0,939016602	0,997568837	T1D vs Control
URS0000920597-rRNA4	-0,035008893	8,520273949	0,939513745	0,997568837	T1D vs Control
URS00002CC2A4-antisense4	-0,043073231	2,224906264	0,939715162	0,997568837	T1D vs Control
URS00004C5EDB-lncRNA4	-0,046738994	2,83469135	0,940036455	0,997568837	T1D vs Control
URS0000639DBE-tRNA4	0,023809606	5,433019134	0,942179678	0,997568837	T1D vs Control
URS0000120E41-tRNA4	-0,022729725	6,378757577	0,942715897	0,997568837	T1D vs Control
URS000062FB25-rRNA4	0,00931245	9,742461609	0,943385593	0,997568837	T1D vs Control
URS0000617C6A-antisense4	0,033526501	3,572347898	0,943828217	0,997568837	T1D vs Control
URS00006C6D0A-tRNA4	-0,037831718	3,831692982	0,948105271	0,997568837	T1D vs Control
URS0000644222-tRNA4	0,014263169	7,91366141	0,948823997	0,997568837	T1D vs Control
URS000051EF4B-lncRNA4	-0,033800887	1,97882489	0,950668467	0,997568837	T1D vs Control
URS00004E5B9A-lncRNA4	0,031748394	1,8276158	0,952829857	0,997568837	T1D vs Control
URS00003D60C0-lncRNA4	-0,030026771	1,735581371	0,955533943	0,997568837	T1D vs Control
URS00002064F6-tRNA4	0,032187475	7,265714406	0,956247668	0,997568837	T1D vs Control
URS00004A7F9B-antisense4	0,012902454	6,391504235	0,958785518	0,997568837	T1D vs Control
URS00007116F9-Y_RNA4	0,027951355	3,042319437	0,958879048	0,997568837	T1D vs Control
URS00008B26D4-antisense4	-0,02676993	4,369068967	0,959467624	0,997568837	T1D vs Control
URS0000955796-rRNA4	0,005364389	10,7330721	0,962720414	0,997568837	T1D vs Control
URS00001C6042-antisense4	-0,024852611	1,983328915	0,965046886	0,997568837	T1D vs Control
URS00006B479B-tRNA4	-0,019001432	1,462161631	0,966860208	0,997568837	T1D vs Control
URS00001AE429-lncRNA4	0,025340561	3,26760494	0,967111389	0,997568837	T1D vs Control
URS00001E15E2-lncRNA4	-0,020366344	2,285605947	0,968088054	0,997568837	T1D vs Control
URS00009C606C-lncRNA4	0,010501512	5,246519845	0,976326241	0,997568837	T1D vs Control
URS00006F4537-tRNA4	0,017942239	4,037892922	0,976338262	0,997568837	T1D vs Control
URS00006271F5-Y_RNA4	-0,0132345	3,66489103	0,97636409	0,997568837	T1D vs Control
URS0000689904-Y_RNA4	-0,008638641	0,877747286	0,978087978	0,997568837	T1D vs Control
URS00003E3BDF-antisense4	0,01401316	2,398707663	0,979162782	0,997568837	T1D vs Control
URS00004D9E92-tRNA4	-0,006793101	7,483097251	0,979807061	0,997568837	T1D vs Control
URS00005D1950-antisense4	-0,011644905	2,067377939	0,982212741	0,997568837	T1D vs Control
URS000019F398-lncRNA4	0,010961538	1,659002941	0,982677183	0,997568837	T1D vs Control
URS000001AE93-lncRNA4	0,004865827	6,898854125	0,983158343	0,997568837	T1D vs Control
URS0000082453-lncRNA4	-0,011152618	2,456766093	0,983530145	0,997568837	T1D vs Control
URS00001424D6-lncRNA4	0,011275123	1,834670251	0,984238282	0,997568837	T1D vs Control
URS000091B709-rRNA4	-0,003252962	9,600448226	0,984585044	0,997568837	T1D vs Control
URS000033F395-lncRNA4	-0,010252361	1,897833694	0,985007183	0,997568837	T1D vs Control
URS00001B2779-lncRNA4	-0,010380794	1,848580988	0,985507054	0,997568837	T1D vs Control

URS000008089F-lncRNA4	-0,010742581	3,494287219	0,986798699	0,997568837	T1D vs Control
URS00004199FB-antisense4	0,009486727	3,00203969	0,988392594	0,997568837	T1D vs Control
URS0000097924-lncRNA4	0,004891714	5,60446115	0,988585612	0,997568837	T1D vs Control
URS0000067204-antisense4	0,007390099	1,800108533	0,989404268	0,997568837	T1D vs Control
URS000042199A-lncRNA4	0,007353718	2,107510848	0,990562316	0,997568837	T1D vs Control
URS0000ABD7E9-lncRNA4	0,006433342	3,512762788	0,991363161	0,997568837	T1D vs Control
URS00002FBC9E-lncRNA4	0,003659176	3,539604284	0,995147038	0,997568837	T1D vs Control
URS00002034DC-tRNA4	-0,003323711	5,180504267	0,995261263	0,997568837	T1D vs Control
URS0000664EAD-tRNA4	-0,003114177	2,046606184	0,99605517	0,997568837	T1D vs Control
URS000000199E-lncRNA4	-0,001949775	6,442848869	0,9961417	0,997568837	T1D vs Control
URS00001AD596-tRNA4	7,30252E-05	11,15468011	0,999643375	0,999643375	T1D vs Control
URS000098FA76-rRNA5	1,388617404	2,496348799	1,11614E-05	0,007801824	T2D vs Control
URS0000974435-SRP_RNA5	1,919385485	2,886817066	0,000433662	0,151564889	T2D vs Control
URS000096196A-SRP_RNA5	1,939380154	2,043342413	0,000827205	0,192738793	T2D vs Control
URS0000727FD6-tRNA5	2,030600225	3,320399515	0,001134412	0,198238472	T2D vs Control
URS0000684E4B-tRNA5	-0,792618524	6,262078663	0,002223312	0,240638339	T2D vs Control
URS000071C9A6-Y_RNA5	1,127040133	2,18048286	0,002941843	0,240638339	T2D vs Control
URS00006F4E76-Y_RNA5	0,827030377	0,766333934	0,003000406	0,240638339	T2D vs Control
URS000070E3CE-rRNA5	1,615288783	4,081177344	0,003089296	0,240638339	T2D vs Control
URS0000644222-tRNA5	0,684704698	7,91366141	0,003574353	0,240638339	T2D vs Control
URS00007CA557-lncRNA5	-0,72440104	9,085280894	0,003729363	0,240638339	T2D vs Control
URS00006729E8-Y_RNA5	0,916712344	0,88430075	0,004020395	0,240638339	T2D vs Control
URS000034E03C-antisense5	1,704395624	2,078398724	0,00413113	0,240638339	T2D vs Control
URS000042F13F-tRNA5	0,822985138	6,573577673	0,004598241	0,247243901	T2D vs Control
URS00000FB60D-tRNA5	1,667888364	3,59514453	0,005552565	0,261196905	T2D vs Control
URS00006C9A71-Y_RNA5	1,473218894	2,255341052	0,005605084	0,261196905	T2D vs Control
URS000047C79B-tRNA5	0,467271652	8,413478971	0,00661067	0,271172042	T2D vs Control
URS0000188F7D-scRNA5	4,387516847	6,577829264	0,006945693	0,271172042	T2D vs Control
URS000065DC2A-tRNA5	1,176665463	4,471494404	0,007271047	0,271172042	T2D vs Control
URS00001662B7-lncRNA5	1,790936383	2,511216373	0,007370914	0,271172042	T2D vs Control
URS000034AAC2-tRNA5	1,501743487	4,895139042	0,007878384	0,27534952	T2D vs Control
URS00002750C5-lncRNA5	1,464556934	1,848936086	0,009107603	0,292297847	T2D vs Control
URS00006C48EB-Y_RNA]5	1,769524245	2,788995838	0,009199646	0,292297847	T2D vs Control
URS00006C900C-tRNA5	1,44062887	2,991743424	0,011474768	0,329923778	T2D vs Control
URS000064B6FC-tRNA5	1,637331038	3,113360192	0,011933632	0,329923778	T2D vs Control
URS0000AA014E-lncRNA5	1,401097726	2,880951371	0,012050103	0,329923778	T2D vs Control
URS0000AABA8B-lncRNA5	-0,663227481	9,325488704	0,012271843	0,329923778	T2D vs Control
URS000029E713-antisense5	-1,274183142	4,061405427	0,013767437	0,356423643	T2D vs Control
URS0000679FAF-tRNA5	1,178453911	5,101125315	0,014634156	0,361277255	T2D vs Control
URS0000AAD5AA-lncRNA5	2,027251515	10,83600588	0,016413617	0,361277255	T2D vs Control
URS00005B6D58-lncRNA5	1,2130752	5,324374408	0,017079338	0,361277255	T2D vs Control
URS000097171C-SRP_RNA5	1,338756692	2,924722071	0,017785778	0,361277255	T2D vs Control
URS00006F4F8C-snoRNA5	0,538101677	8,039702247	0,018019683	0,361277255	T2D vs Control
URS0000812136-lncRNA5	-0,541870281	9,121672555	0,018534223	0,361277255	T2D vs Control
URS0000630B8A-tRNA5	1,146082109	5,357715317	0,01920554	0,361277255	T2D vs Control
URS0000A7BA37-lncRNA5	1,301088933	1,699099075	0,01927445	0,361277255	T2D vs Control
URS000075AC32-lncRNA5	-1,45436661	2,916319528	0,019362655	0,361277255	T2D vs Control
URS0000684921-rRNA5	1,406183329	2,725738393	0,020949537	0,361277255	T2D vs Control
URS0000249329-lncRNA5	1,208782243	1,740243504	0,021023722	0,361277255	T2D vs Control
URS0000415026-tRNA5	1,093289657	4,426745558	0,021099133	0,361277255	T2D vs Control
URS00003C9A26-tRNA5	0,450406659	8,822917147	0,021275127	0,361277255	T2D vs Control
URS00006BF71F-rRNA5	1,384639174	2,492314255	0,021464221	0,361277255	T2D vs Control
URS000063E4FD-tRNA5	1,460276466	3,061724682	0,021958425	0,361277255	T2D vs Control
URS000059900F-tRNA5	1,350142023	3,409718688	0,022224495	0,361277255	T2D vs Control
URS00006C0413-Y_RNA5	-1,400365299	2,155330761	0,023677834	0,376154675	T2D vs Control
URS000062C4DE-tRNA5	1,33800065	3,676191563	0,025406093	0,379486127	T2D vs Control
URS000064506B-tRNA5	1,477916812	2,909810805	0,025610052	0,379486127	T2D vs Control
URS000035D229-lncRNA5	1,903098157	3,593153184	0,02575596	0,379486127	T2D vs Control
URS000047AE74-lncRNA5	1,320786483	2,522838605	0,026059133	0,379486127	T2D vs Control
URS00000AD390-antisense5	1,254805406	1,744059197	0,027246537	0,388680195	T2D vs Control
URS000031A1AE-antisense5	1,409522055	2,086098634	0,029227478	0,408600139	T2D vs Control
URS0000145C5E-tRNA5	1,417981023	2,892697082	0,030097517	0,412513025	T2D vs Control
URS00000D1D87-lncRNA5	1,136880168	1,722539329	0,033917979	0,455935914	T2D vs Control
URS0000907244-rRNA5	-0,530502014	6,675718226	0,037091154	0,46267998	T2D vs Control
URS00002C9C48-lncRNA5	-1,192827773	5,358879491	0,037241962	0,46267998	T2D vs Control
URS0000931B54-rRNA5	0,169753598	14,46696978	0,037252856	0,46267998	T2D vs Control

URS0000716B70-tRNA5	1,11460829	3,560279134	0,037502325	0,46267998	T2D vs Control
URS0000941729-rRNA5	-1,047209342	4,187919105	0,037964589	0,46267998	T2D vs Control
URS000029CCC5-tRNA5	1,17968579	2,732517708	0,038466618	0,46267998	T2D vs Control
URS000069B369-Y_RNA5	-1,4776888	9,690811268	0,039053103	0,46267998	T2D vs Control
URS0000A77003-lncRNA5	1,185915873	2,821180105	0,044103575	0,513806654	T2D vs Control
URS0000120E41-tRNA5	0,657223832	6,378757577	0,047560705	0,525827918	T2D vs Control
URS0000639DBE-tRNA5	0,679557993	5,433019134	0,048313953	0,525827918	T2D vs Control
URS000014D914-antisense5	-1,005647638	4,160373223	0,049362691	0,525827918	T2D vs Control
URS00000E43DB-antisense5	1,092894921	3,522079692	0,049605808	0,525827918	T2D vs Control
URS000032B6B6-snRNA5	0,432462018	8,295722512	0,04961117	0,525827918	T2D vs Control
URS00006C8EDF-tRNA5	1,241803475	3,590220694	0,050886497	0,525827918	T2D vs Control
URS00001DFAE4-lncRNA5	1,40106787	3,289114858	0,051358579	0,525827918	T2D vs Control
URS0000488EA7-lncRNA5	-1,203464136	2,312667922	0,051817911	0,525827918	T2D vs Control
URS000044BAE3-tRNA5	0,426521485	7,695683129	0,05190576	0,525827918	T2D vs Control
URS00006529EE-Y_RNA5	1,180890867	3,147668241	0,05408072	0,538016446	T2D vs Control
URS000061F57C-tRNA5	0,985588072	4,675906559	0,054648309	0,538016446	T2D vs Control
URS00006CC125-rRNA5	1,125440799	2,366732734	0,055544697	0,539246432	T2D vs Control
URS0000617C6A-antisense5	0,940781769	3,572347898	0,057005464	0,545846836	T2D vs Control
URS0000697465-tRNA5	1,184268706	4,721333977	0,058871143	0,556093636	T2D vs Control
URS000011BDAF-antisense5	1,01066657	2,89470373	0,060521749	0,559398799	T2D vs Control
URS000064C567-Y_RNA5	0,46265578	5,523874961	0,060821615	0,559398799	T2D vs Control
URS00004AE57B-lncRNA5	1,119892576	2,181566953	0,062310998	0,565654384	T2D vs Control
URS000005AEAB-tRNA5	0,886843472	4,48776437	0,065580917	0,576417451	T2D vs Control
URS000063FB43-tRNA5	0,369095606	12,66119095	0,065935301	0,576417451	T2D vs Control
URS0000422302-lncRNA5	1,000427946	2,914915679	0,066680964	0,576417451	T2D vs Control
URS00006FF680-Y_RNA5	-1,106644346	2,916835355	0,067577078	0,576417451	T2D vs Control
URS0000341866-lncRNA5	-1,126094998	4,848112839	0,067619787	0,576417451	T2D vs Control
URS00004BF687-tRNA5	1,178653114	13,4414794	0,068589708	0,577641037	T2D vs Control
URS00002D40C8-tRNA5	0,460976395	7,830242615	0,07037188	0,578993665	T2D vs Control
URS000064F96B-Y_RNA5	0,634582023	5,069805695	0,070686416	0,578993665	T2D vs Control
URS0000696377-Y_RNA5	1,042152519	2,848796511	0,071244415	0,578993665	T2D vs Control
URS0000515855-lncRNA5	-0,959200467	2,502117237	0,073636738	0,578993665	T2D vs Control
URS00001A86BB-tRNA5	0,920983029	2,65628615	0,074135022	0,578993665	T2D vs Control
URS0000624312-Y_RNA5	0,745892111	5,698207316	0,074981247	0,578993665	T2D vs Control
URS00001424D6-lncRNA5	1,062544093	1,834670251	0,075586616	0,578993665	T2D vs Control
URS0000022477-lncRNA5	-1,030451099	1,898475606	0,076271499	0,578993665	T2D vs Control
URS00000A3F50-lncRNA5	0,984004757	1,749216608	0,077451997	0,578993665	T2D vs Control
URS00009290F1-rRNA5	0,999018123	1,980245991	0,078056155	0,578993665	T2D vs Control
URS000077A114-lncRNA5	-0,307759347	7,694058287	0,078266455	0,578993665	T2D vs Control
URS0000209048-tRNA5	1,101376689	3,508601642	0,078690126	0,578993665	T2D vs Control
URS00001E15E2-lncRNA5	0,925060109	2,285605947	0,081296172	0,588633358	T2D vs Control
URS000051EF4B-lncRNA5	0,999535089	1,97882489	0,082377203	0,588633358	T2D vs Control
URS0000ABD7E9-lncRNA5	1,076850858	3,512762788	0,082526565	0,588633358	T2D vs Control
URS000060A110-antisense5	-0,827473642	1,367875278	0,085539988	0,590869466	T2D vs Control
URS0000233E9C-lncRNA5	-1,352124832	2,822247929	0,087544406	0,590869466	T2D vs Control
URS000002E930-antisense5	-1,183300301	4,041839683	0,087598862	0,590869466	T2D vs Control
URS00004D9E92-tRNA5	0,477175611	7,483097251	0,087746581	0,590869466	T2D vs Control
URS00005BE013-lncRNA5	1,284080098	2,759573682	0,087819362	0,590869466	T2D vs Control
URS00004A68E9-antisense5	0,742723035	3,654195066	0,088780619	0,590869466	T2D vs Control
URS00001BF716-lncRNA5	-0,340931126	7,608759782	0,088826821	0,590869466	T2D vs Control
URS00000A586F-lncRNA5	-0,562198594	0,916847513	0,089885295	0,590869466	T2D vs Control
URS00003B6188-lncRNA5	0,919411165	3,075648025	0,09044783	0,590869466	T2D vs Control
URS0000278E1B-tRNA5	0,971229162	2,766067348	0,096713413	0,625950702	T2D vs Control
URS000031A3C9-lncRNA5	-0,846708542	4,013881647	0,10022792	0,639646091	T2D vs Control
URS00001A48CC-lncRNA5	0,926920264	2,650296854	0,102009864	0,639646091	T2D vs Control
URS0000944F10-rRNA5	-0,682378499	5,261748123	0,102440931	0,639646091	T2D vs Control
URS000096EEE8-rRNA5	0,22175046	8,595030803	0,102489789	0,639646091	T2D vs Control
URS0000977FD2-SRP_RNA5	0,947241073	2,037495207	0,103653812	0,641185973	T2D vs Control
URS000076DAC1-lncRNA5	0,699971595	5,661423845	0,106441142	0,652652264	T2D vs Control
URS00004D4BFC-lncRNA5	0,827905482	2,209794014	0,107525997	0,653571058	T2D vs Control
URS00002C4609-lncRNA5	0,985796841	2,010894744	0,110768927	0,667478276	T2D vs Control
URS00002A865C-lncRNA5	0,885124433	1,751381992	0,113049371	0,674983084	T2D vs Control
URS00003A00A4-lncRNA5	0,963005496	3,039610494	0,113945642	0,674983084	T2D vs Control
URS00005BC8EC-lncRNA5	-1,218012854	5,143820106	0,121575782	0,709875649	T2D vs Control
URS00002E367B-antisense5	0,895692173	1,915955023	0,121867064	0,709875649	T2D vs Control
URS00002034DC-tRNA5	0,901643362	5,180504267	0,123487758	0,713371428	T2D vs Control

URS000021BDC3-snRNA5	0,840854624	2,084780028	0,130557921	0,740062855	T2D vs Control
URS00002D2D8F-misc_RNA]5	-0,986807609	1,939760416	0,130641268	0,740062855	T2D vs Control
URS0000222FD2-tRNA5	0,959295112	3,623751413	0,132024139	0,740062855	T2D vs Control
URS0000121433-tRNA5	1,175314874	4,021698317	0,132343143	0,740062855	T2D vs Control
URS0000543B4D-lncRNA5	0,925751155	2,605909076	0,137259774	0,76007449	T2D vs Control
URS00001B59BD-lncRNA5	0,402021632	6,767957763	0,141292875	0,76007449	T2D vs Control
URS0000067204-antisense5	0,85897289	1,800108533	0,141744804	0,76007449	T2D vs Control
URS000090169F-rRNA5	-0,827824649	1,691620139	0,141961089	0,76007449	T2D vs Control
URS00000A00A2-antisense5	0,958521055	4,833316746	0,141993681	0,76007449	T2D vs Control
URS00000CD33E-antisense5	-0,797758572	3,630533758	0,143139999	0,76007449	T2D vs Control
URS00009A050E-rRNA5	0,26636354	10,767947	0,14353338	0,76007449	T2D vs Control
URS00006361F3-Y_RNA5	1,127440137	4,856921385	0,147903188	0,763910317	T2D vs Control
URS0000417E86-lncRNA5	-0,890994594	2,54225615	0,149319111	0,763910317	T2D vs Control
URS0000920597-rRNA5	0,69819439	8,520273949	0,149660212	0,763910317	T2D vs Control
URS00003AD33F-lncRNA5	-0,923227286	3,336093709	0,150237201	0,763910317	T2D vs Control
URS00006D56C0-Y_RNA5	0,595206433	4,210228585	0,150415008	0,763910317	T2D vs Control
URS00001B2779-lncRNA5	0,859723318	1,848580988	0,150814912	0,763910317	T2D vs Control
URS000067474D-tRNA5	0,926124505	5,826738993	0,153180807	0,764455903	T2D vs Control
URS000022CCD7-lncRNA5	0,407189083	6,400235397	0,154368343	0,764455903	T2D vs Control
URS00007BC71B-lncRNA5	0,886045142	1,944342872	0,154650184	0,764455903	T2D vs Control
URS00000D8A7A-lncRNA5	0,807689936	2,120449279	0,156298956	0,764455903	T2D vs Control
URS00003008EB-lncRNA5	-0,703605172	4,342465078	0,156390836	0,764455903	T2D vs Control
URS00007C72D0-lncRNA5	0,712560698	1,699191247	0,158319804	0,768510714	T2D vs Control
URS0000397210-lncRNA5	0,786673935	2,372225381	0,159562133	0,769199523	T2D vs Control
URS0000166229-lncRNA5	0,749106329	2,097439644	0,161588774	0,77155384	T2D vs Control
URS0000A7F61B-lncRNA5	0,775929636	2,242573569	0,164907226	0,77155384	T2D vs Control
URS000066C003-Y_RNA]5	-1,653844572	5,557417709	0,165990775	0,77155384	T2D vs Control
URS00001FBD75-misc_RNA]5	-1,24168807	3,307722647	0,166589376	0,77155384	T2D vs Control
URS000018267A-antisense5	0,929767427	3,83534964	0,168486899	0,77155384	T2D vs Control
URS00000B8842-lncRNA5	-0,662989612	4,912142401	0,168498004	0,77155384	T2D vs Control
URS00006BFB96-tRNA5	0,833820674	2,209793928	0,170693685	0,77155384	T2D vs Control
URS0000ABD879-rRNA5	-1,696761586	7,136004534	0,170763571	0,77155384	T2D vs Control
URS00003AA49D-lncRNA5	-0,787335916	2,72551685	0,17105281	0,77155384	T2D vs Control
URS0000005EDF-lncRNA5	-0,738196524	4,116716413	0,171088477	0,77155384	T2D vs Control
URS00002EDD28-lncRNA5	-0,531035727	5,436899358	0,172446888	0,772694712	T2D vs Control
URS00004F6629-lncRNA5	0,554523723	1,127896398	0,176125871	0,784152765	T2D vs Control
URS0000A89523-lncRNA5	-0,830097023	2,553603804	0,177477844	0,785170968	T2D vs Control
URS00001E6C0A-lncRNA5	0,742028305	3,103912065	0,179565883	0,78941228	T2D vs Control
URS000001AE93-lncRNA5	-0,321554272	6,898854125	0,181078113	0,789649044	T2D vs Control
URS0000383A48-tRNA5	-0,801074447	3,229014707	0,182585461	0,789649044	T2D vs Control
URS00007125F9-rRNA5	0,806905979	2,409823294	0,183546075	0,789649044	T2D vs Control
URS00005D36AA-lncRNA5	-0,807550823	2,523255562	0,18529465	0,789649044	T2D vs Control
URS0000197DBF-antisense5	0,727107556	3,806884628	0,185993993	0,789649044	T2D vs Control
URS0000590507-lncRNA5	0,737247874	1,90331514	0,187351632	0,789649044	T2D vs Control
URS000064E10F-tRNA5	0,801453781	3,013775094	0,187527527	0,789649044	T2D vs Control
URS00000AED6F-tRNA5	0,633315431	6,83625311	0,190034585	0,793861857	T2D vs Control
URS00003D2CC9-tRNA5	0,716308575	4,271817892	0,192026518	0,793861857	T2D vs Control
URS00003F2CFE-lncRNA5	-0,749302261	4,520438351	0,192598324	0,793861857	T2D vs Control
URS000060D3B6-lncRNA5	-1,010800228	4,588963786	0,193873746	0,793861857	T2D vs Control
URS00009C606C-lncRNA5	-0,47874341	5,246519845	0,194206549	0,793861857	T2D vs Control
URS00006005A4-lncRNA5	0,931277123	2,906455771	0,196769992	0,799664094	T2D vs Control
URS000006D0D7-lncRNA5	0,748349668	2,152173172	0,200171948	0,803817353	T2D vs Control
URS00006481F8-Y_RNA5	-0,406815693	7,303708498	0,201231376	0,803817353	T2D vs Control
URS00000C653A-lncRNA5	0,719696048	2,462989968	0,205414228	0,803817353	T2D vs Control
URS00008119F8-lncRNA5	-0,825574953	3,00844528	0,207235213	0,803817353	T2D vs Control
URS000099C20E-rRNA5	0,848034616	3,326722503	0,208005655	0,803817353	T2D vs Control
URS000075AD80-snRNA5	0,784002062	2,124304339	0,208395512	0,803817353	T2D vs Control
URS00006D4DB0-snRNA5	-0,831533308	3,391553507	0,209025455	0,803817353	T2D vs Control
URS00001D081D-antisense5	0,721946713	1,824716399	0,210342964	0,803817353	T2D vs Control
URS0000920CBD-rRNA5	-1,582824329	5,869762745	0,211268924	0,803817353	T2D vs Control
URS000068F7ED-Y_RNA5	0,656536126	6,623874775	0,211448875	0,803817353	T2D vs Control
URS0000005270-rRNA5	-1,15355215	6,166410111	0,211670859	0,803817353	T2D vs Control
URS0000704D22-rRNA5	0,235017908	14,1067509	0,212423629	0,803817353	T2D vs Control
URS000067843B-Y_RNA5	0,510859707	5,925250166	0,21274136	0,803817353	T2D vs Control
URS0000ABD8C6-rRNA5	-1,533083138	7,712316992	0,218594275	0,810447625	T2D vs Control
URS000024B38F-tRNA5	0,706233833	4,404533737	0,220039538	0,810447625	T2D vs Control

URS0000A9F786-lncRNA5	0,849320376	3,099717163	0,221195904	0,810447625	T2D vs Control
URS000049BB82-lncRNA5	-0,622996189	4,039331254	0,221392834	0,810447625	T2D vs Control
URS00006CBBB9-Y_RNA5	-0,733487723	3,554703445	0,221407238	0,810447625	T2D vs Control
URS000039557B-antisense5	0,679287355	1,694045712	0,222736928	0,810447625	T2D vs Control
URS00003ADE1B-lncRNA5	0,734883237	2,400792438	0,222918229	0,810447625	T2D vs Control
URS00004106BA-lncRNA5	0,617171687	1,798128499	0,223932153	0,810447625	T2D vs Control
URS000002FD7D-lncRNA5	0,666897103	1,677824648	0,2249311	0,810447625	T2D vs Control
URS00004DDD71-lncRNA5	-0,442035911	5,854746665	0,231427484	0,815176854	T2D vs Control
URS000000898B-lncRNA5	0,768115616	2,509136511	0,232033066	0,815176854	T2D vs Control
URS00005DB87D-tRNA5	0,248852541	7,975358739	0,232060537	0,815176854	T2D vs Control
URS000075B143-lncRNA5	-0,672045395	2,661955926	0,23465647	0,815176854	T2D vs Control
URS0000690F87-snRNA5	0,861640774	3,114315559	0,235204957	0,815176854	T2D vs Control
URS00006642D4-Y_RNA5	-0,235996593	4,428261145	0,235768505	0,815176854	T2D vs Control
URS00000F6ECB-lncRNA5	-0,464388176	5,128846246	0,235851169	0,815176854	T2D vs Control
URS0000515429-lncRNA5	0,458700819	7,164323775	0,236258482	0,815176854	T2D vs Control
URS00002D0015-lncRNA5	-0,630893379	3,226559685	0,236739487	0,815176854	T2D vs Control
URS0000192C05-lncRNA5	0,664265054	2,748839188	0,239821938	0,818461088	T2D vs Control
URS0000591DFC-lncRNA5	0,640410285	1,976731906	0,240035083	0,818461088	T2D vs Control
URS00000E0E97-antisense5	1,058335243	4,066934958	0,242835247	0,820892889	T2D vs Control
URS000053EAB5-lncRNA5	-0,637551524	3,036408354	0,243204918	0,820892889	T2D vs Control
URS000097CDD0-rRNA5	0,270055804	6,617743447	0,244619836	0,820892889	T2D vs Control
URS000055C9F8-antisense5	-0,547242691	4,017918826	0,245445799	0,820892889	T2D vs Control
URS000063B690-snRNA5	0,689192029	3,324962274	0,250673602	0,834384988	T2D vs Control
URS0000399BDA-lncRNA5	-0,646742177	5,117165985	0,252170466	0,835389363	T2D vs Control
URS00003CAB47-antisense5	-0,682281241	3,257126248	0,25352319	0,835909009	T2D vs Control
URS00000AD0C2-lncRNA5	0,645406585	1,982015887	0,257387871	0,840574564	T2D vs Control
URS00006174C2-tRNA5	0,263438485	8,218629708	0,258791068	0,840574564	T2D vs Control
URS0000A88906-antisense5	0,680376869	1,925012855	0,261489229	0,840574564	T2D vs Control
URS0000089048-antisense5	0,619090763	1,859342356	0,262580753	0,840574564	T2D vs Control
URS0000676525-Y_RNA5	-0,586898857	4,329395557	0,265631896	0,840574564	T2D vs Control
URS00004BE455-lncRNA5	-0,483761731	5,228003516	0,265828742	0,840574564	T2D vs Control
URS00000E9A71-lncRNA5	0,629175289	3,823249127	0,266075715	0,840574564	T2D vs Control
URS00004AAD0A-lncRNA5	-0,633498135	2,996600067	0,266621131	0,840574564	T2D vs Control
URS00008116E3-lncRNA5	0,583433794	4,000279228	0,26671813	0,840574564	T2D vs Control
URS00004C6AFE-antisense5	0,482174001	5,444648432	0,266963595	0,840574564	T2D vs Control
URS000013B42D-tRNA5	0,393809346	16,60649921	0,268713273	0,842289587	T2D vs Control
URS0000767DAE-lncRNA5	-0,397800117	5,637395825	0,276926123	0,849886567	T2D vs Control
URS0000257C23-lncRNA5	0,756477928	4,584581553	0,277776642	0,849886567	T2D vs Control
URS00006AE6F1-Y_RNA5	0,496074995	2,11506442	0,278428309	0,849886567	T2D vs Control
URS00006CFDFE-Y_RNA5	-0,47732517	2,249644641	0,279551882	0,849886567	T2D vs Control
URS00006C6D0A-tRNA5	-0,652433689	3,831692982	0,280037834	0,849886567	T2D vs Control
URS00005B6FC3-lncRNA5	0,478087782	4,468244677	0,280389983	0,849886567	T2D vs Control
URS000071ED2F-tRNA5	0,715338537	2,991616318	0,280778617	0,849886567	T2D vs Control
URS000064FE59-rRNA5	0,154193197	11,09750364	0,280863801	0,849886567	T2D vs Control
URS0000006840-lncRNA5	0,563792275	3,802540443	0,286544362	0,858996703	T2D vs Control
URS0000918BC5-rRNA5	0,974248323	3,816571784	0,28736488	0,858996703	T2D vs Control
URS00009AAC46-rRNA5	0,20177186	9,797442669	0,287561128	0,858996703	T2D vs Control
URS000065A213-Y_RNA5	-0,515252214	10,7844197	0,296023682	0,866226653	T2D vs Control
URS00006772C0-tRNA5	0,663889791	2,278759881	0,296116702	0,866226653	T2D vs Control
URS00001E5F12-lncRNA5	0,632614985	2,516521413	0,297620126	0,866226653	T2D vs Control
URS00000CCACD-antisense5	0,767356722	4,642488599	0,297814334	0,866226653	T2D vs Control
URS00005B30A9-tRNA5	0,707523196	2,750819629	0,298646918	0,866226653	T2D vs Control
URS00007BE6D3-lncRNA5	1,500425828	10,22836282	0,299618234	0,866226653	T2D vs Control
URS00008BB15B-lncRNA5	0,71746985	2,482102831	0,301639086	0,866226653	T2D vs Control
URS00000FCDE9-misc_RNA]5	-0,769981361	3,011608195	0,306027102	0,866226653	T2D vs Control
URS0000181B59-lncRNA5	0,719374507	2,274734234	0,308283278	0,866226653	T2D vs Control
URS000018BCE5-antisense5	-0,602508892	2,555076799	0,309577493	0,866226653	T2D vs Control
URS00006F7C66-rRNA5	-0,594203684	1,804743197	0,31150298	0,866226653	T2D vs Control
URS0000702B40-Y_RNA5	0,697068094	2,572703997	0,311836695	0,866226653	T2D vs Control
URS00003D279B-lncRNA5	0,57115505	3,869764687	0,313516202	0,866226653	T2D vs Control
URS00004DA951-rRNA5	0,341096962	10,73867185	0,314383441	0,866226653	T2D vs Control
URS00001AD596-tRNA5	0,171495459	11,15468011	0,315038059	0,866226653	T2D vs Control
URS0000265843-antisense5	0,545023733	2,862204159	0,316178822	0,866226653	T2D vs Control
URS00000B6370-lncRNA5	-0,464744063	3,6717613	0,31667791	0,866226653	T2D vs Control
URS00007D61AD-antisense5	0,757759767	2,769938297	0,31840525	0,866226653	T2D vs Control
URS0000527686-lncRNA5	0,404660268	1,162280834	0,318483723	0,866226653	T2D vs Control

URS000076B0D1-lncRNA5	0,580489504	3,094918128	0,320789849	0,866226653	T2D vs Control
URS0000996BBC-rRNA5	-0,190543457	8,622403976	0,321673702	0,866226653	T2D vs Control
URS00005C51E2-lncRNA5	0,545419198	1,604886726	0,322060919	0,866226653	T2D vs Control
URS0000395C70-lncRNA5	-0,729447003	2,694736211	0,322115017	0,866226653	T2D vs Control
URS00006CE1FB-misc_RNA]5	0,948702865	6,644778412	0,322279783	0,866226653	T2D vs Control
URS00005F1728-lncRNA5	0,544889894	2,661157063	0,322644184	0,866226653	T2D vs Control
URS00001B6230-lncRNA5	-0,561695545	2,056088304	0,323765922	0,866226653	T2D vs Control
URS000056B231-lncRNA5	0,605740771	4,1586305	0,32471448	0,866226653	T2D vs Control
URS00006CDFA7-rRNA5	0,544282409	2,666787507	0,325242126	0,866226653	T2D vs Control
URS00003869EC-lncRNA5	0,681584013	2,98234075	0,325919327	0,866226653	T2D vs Control
URS0000672E5A-rRNA5	0,367251317	5,608838646	0,328941355	0,870871211	T2D vs Control
URS00003F2105-lncRNA5	0,577040999	2,406932528	0,330517369	0,870871211	T2D vs Control
URS00006F3305-rRNA5	-0,106492563	11,47052611	0,332632451	0,870871211	T2D vs Control
URS00001AA18A-lncRNA5	0,539148942	1,870939952	0,334013747	0,870871211	T2D vs Control
URS000003870B-lncRNA5	-0,209823469	9,957227048	0,336881105	0,870871211	T2D vs Control
URS0000478C87-lncRNA5	0,538421068	2,830369346	0,336939567	0,870871211	T2D vs Control
URS0000633F75-ribozyme5	0,527512984	4,438697312	0,337924009	0,870871211	T2D vs Control
URS0000A76F22-lncRNA5	0,54964539	2,591384678	0,338197085	0,870871211	T2D vs Control
URS000036E063-antisense5	-0,118401314	10,32347827	0,338879785	0,870871211	T2D vs Control
URS000013BA50-antisense5	0,613787844	1,971579125	0,340474641	0,87176474	T2D vs Control
URS0000635FFC-Y_RNA5	0,566537683	2,322009496	0,342233904	0,87280542	T2D vs Control
URS00000734D4-lncRNA5	-0,476445913	2,320562453	0,343378384	0,87280542	T2D vs Control
URS000095D156-rRNA5	-0,401980587	6,045790564	0,346290535	0,87700835	T2D vs Control
URS000003B6E2-lncRNA5	0,744802756	2,652355198	0,347866101	0,87700835	T2D vs Control
URS000023352F-lncRNA5	-0,501762159	2,158198536	0,350785104	0,87700835	T2D vs Control
URS0000543A1A-lncRNA5	-0,493982433	5,169008445	0,352359064	0,87700835	T2D vs Control
URS00000347CF-lncRNA5	0,470957137	3,718528756	0,352693586	0,87700835	T2D vs Control
URS00004A7F9B-antisense5	-0,240817608	6,391504235	0,35568229	0,87700835	T2D vs Control
URS000076FC5A-lncRNA5	0,595995114	2,343917503	0,35603222	0,87700835	T2D vs Control
URS000047AF1F-lncRNA5	0,834666048	4,480968908	0,357149558	0,87700835	T2D vs Control
URS0000271FCA-antisense5	-0,432377812	5,084020409	0,357479945	0,87700835	T2D vs Control
URS00007E2F7C-lncRNA5	-0,577317583	3,279760012	0,359829045	0,87700835	T2D vs Control
URS0000504A1A-lncRNA5	0,497187095	1,470603839	0,360457483	0,87700835	T2D vs Control
URS00003F41E9-lncRNA5	-0,190285227	8,007323205	0,36205439	0,87700835	T2D vs Control
URS0000776086-lncRNA5	0,343685533	7,320712579	0,36312624	0,87700835	T2D vs Control
URS0000ABD87F-rRNA5	0,944540314	10,81861494	0,363875431	0,87700835	T2D vs Control
URS000005F65D-antisense5	0,480977022	4,947813537	0,36756969	0,87700835	T2D vs Control
URS00003875B8-lncRNA5	0,579790474	2,156965412	0,367960781	0,87700835	T2D vs Control
URS0000701637-rRNA5	0,562667413	2,670549892	0,371704774	0,87700835	T2D vs Control
URS000023F3B4-lncRNA5	-0,436026607	3,955198744	0,372118386	0,87700835	T2D vs Control
URS00003E3BDF-antisense5	-0,496662652	2,398707663	0,372769059	0,87700835	T2D vs Control
URS00006A2BF7-Y_RNA5	-0,547315903	10,1864015	0,372951215	0,87700835	T2D vs Control
URS00002E81F5-lncRNA5	0,461344544	4,084000104	0,373279848	0,87700835	T2D vs Control
URS00003F5471-lncRNA5	0,483278781	3,0793045	0,37382728	0,87700835	T2D vs Control
URS000033268A-lncRNA5	0,502847128	4,132479222	0,375640291	0,87700835	T2D vs Control
URS000068089E-Y_RNA5	0,541361913	2,479289353	0,375926257	0,87700835	T2D vs Control
URS00006F0FC0-tRNA5	0,357836789	5,435006769	0,377631924	0,87700835	T2D vs Control
URS000064D54F-tRNA5	0,498226307	2,523144202	0,381570013	0,87700835	T2D vs Control
URS00006F5B12-Y_RNA5	-0,534578447	5,757189507	0,381702477	0,87700835	T2D vs Control
URS00006D9244-tRNA5	0,568668935	3,430512932	0,384736886	0,87700835	T2D vs Control
URS0000762146-antisense5	-0,331376827	5,166011847	0,385323979	0,87700835	T2D vs Control
URS0000446770-snoRNA5	0,544749929	2,104005039	0,388179673	0,87700835	T2D vs Control
URS0000365006-lncRNA5	0,486970247	2,397164181	0,388509244	0,87700835	T2D vs Control
URS0000166FF8-lncRNA5	-0,424925279	4,546451597	0,393207565	0,87700835	T2D vs Control
URS000064217E-Y_RNA5	-0,524519558	2,41296644	0,393234199	0,87700835	T2D vs Control
URS00001D0305-lncRNA5	-0,159576356	7,373693454	0,393463824	0,87700835	T2D vs Control
URS0000097924-lncRNA5	0,30287623	5,60446115	0,396480696	0,87700835	T2D vs Control
URS00000D6053-lncRNA5	0,730990139	5,001351593	0,397046276	0,87700835	T2D vs Control
URS00007D24CA-lncRNA5	-0,660755427	2,948889293	0,397239019	0,87700835	T2D vs Control
URS000071E736-Y_RNA5	0,537975368	2,593633412	0,39743058	0,87700835	T2D vs Control
URS000099BDEE-rRNA5	-0,503368692	5,090607985	0,401110976	0,87700835	T2D vs Control
URS00001D0B96-lncRNA5	-0,114013638	10,38627035	0,401482938	0,87700835	T2D vs Control
URS000090AA7A-rRNA5	0,508245052	2,615865166	0,401789843	0,87700835	T2D vs Control
URS0000669D0F-snRNA5	0,524557245	2,759909955	0,402572604	0,87700835	T2D vs Control
URS000099D184-rRNA5	0,348554815	4,977808511	0,404049356	0,87700835	T2D vs Control
URS000007383C-antisense5	0,465084835	2,496432723	0,404311658	0,87700835	T2D vs Control

URS000000199E-lncRNA5	-0,350812171	6,442848869	0,405080028	0,87700835	T2D vs Control
URS00001823EB-lncRNA5	-0,529041544	3,483455808	0,40509193	0,87700835	T2D vs Control
URS0000172E58-lncRNA5	0,461604151	3,538272714	0,405873669	0,87700835	T2D vs Control
URS0000493225-tRNA5	-0,241632671	7,026724216	0,406355611	0,87700835	T2D vs Control
URS00005AF005-lncRNA5	0,470100674	3,522774737	0,408415284	0,87700835	T2D vs Control
URS0000594305-lncRNA5	0,46154722	1,940137107	0,410478248	0,87700835	T2D vs Control
URS00007D6D04-lncRNA5	-0,4272542	3,22797681	0,410919935	0,87700835	T2D vs Control
URS0000561169-lncRNA5	0,479536752	3,951068111	0,413159797	0,87700835	T2D vs Control
URS00001AE429-lncRNA5	0,524935265	3,26760494	0,413694843	0,87700835	T2D vs Control
URS0000664EAD-tRNA5	0,536998589	2,046606184	0,414290845	0,87700835	T2D vs Control
URS00004D7012-lncRNA5	-0,601233399	2,906892949	0,414362865	0,87700835	T2D vs Control
URS0000605144-antisense5	-0,48011034	2,730860041	0,416348641	0,87700835	T2D vs Control
URS00006E3DE1-snRNA5	0,539974496	3,143314554	0,4165476	0,87700835	T2D vs Control
URS0000633321-snoRNA5	0,20845651	7,948494424	0,419537359	0,880561819	T2D vs Control
URS0000042FD3-lncRNA5	-0,421970413	3,024717754	0,420754861	0,880561819	T2D vs Control
URS0000635088-tRNA5	0,407119584	10,57667068	0,423299244	0,883242304	T2D vs Control
URS00007062F7-Y_RNA5	0,319967967	4,160857713	0,426534734	0,887344581	T2D vs Control
URS000062F68C-rRNA5	0,10981623	10,93938784	0,428299047	0,887433719	T2D vs Control
URS000091CD45-rRNA5	-0,436690634	6,581914152	0,429116734	0,887433719	T2D vs Control
URS00000DA554-lncRNA5	0,468750936	2,522581542	0,430481573	0,887513045	T2D vs Control
URS0000086FDD-antisense5	0,551783099	2,945683418	0,431694471	0,887513045	T2D vs Control
URS0000576D5D-lncRNA5	0,443349799	1,73318224	0,436719279	0,895210487	T2D vs Control
URS000015BB33-lncRNA5	0,399786912	3,745978664	0,440305448	0,895692763	T2D vs Control
URS000028E102-lncRNA5	0,448968482	2,546561157	0,440321627	0,895692763	T2D vs Control
URS0000082453-lncRNA5	-0,432906086	2,456766093	0,441332699	0,895692763	T2D vs Control
URS00006C0715-rRNA5	0,115268779	11,18891955	0,442080119	0,895692763	T2D vs Control
URS0000593A4A-lncRNA5	0,479052145	3,380617038	0,445450925	0,899913863	T2D vs Control
URS00004F2E54-lncRNA5	0,451396776	1,792153167	0,447565563	0,901580198	T2D vs Control
URS0000759CF4-lncRNA5	0,43714094	1,845197414	0,452510178	0,906350138	T2D vs Control
URS000022006F-lncRNA5	0,425844936	3,198783691	0,455082435	0,906350138	T2D vs Control
URS000019B78E-misc_RNA]5	0,63707362	3,588733066	0,457602724	0,906350138	T2D vs Control
URS000030BAD5-tRNA5	0,203339866	9,963231125	0,458154887	0,906350138	T2D vs Control
URS0000007D24-misc_RNA]5	-1,12002541	6,091419652	0,458589655	0,906350138	T2D vs Control
URS00000081EA-snRNA5	0,441872824	2,66468092	0,458876784	0,906350138	T2D vs Control
URS000062C73B-tRNA5	0,334774939	4,544224584	0,459329502	0,906350138	T2D vs Control
URS00009A0DCD-rRNA5	-0,335369977	5,942865971	0,460306579	0,906350138	T2D vs Control
URS000070B37B-tRNA5	0,140700386	9,295263071	0,464334883	0,90964873	T2D vs Control
URS000014D40F-tRNA5	0,544572559	3,081622422	0,464584545	0,90964873	T2D vs Control
URS0000100BA4-antisense5	-0,434652461	1,836907151	0,467218383	0,910101205	T2D vs Control
URS0000432B92-antisense5	-0,138069932	7,647433512	0,467419646	0,910101205	T2D vs Control
URS00007DFA49-antisense5	-0,530042704	2,453720457	0,470193668	0,910569807	T2D vs Control
URS0000653BD1-Y_RNA5	-0,383715609	4,226797259	0,471474501	0,910569807	T2D vs Control
URS00007116F9-Y_RNA5	0,404646046	3,042319437	0,471568341	0,910569807	T2D vs Control
URS000012730D-lncRNA5	-0,429790802	3,696451386	0,474465339	0,91168466	T2D vs Control
URS000022DD4A-tRNA5	-0,170837358	9,64520168	0,475945507	0,91168466	T2D vs Control
URS00006C06E6-Y_RNA5	0,420053971	1,912207487	0,477845099	0,91168466	T2D vs Control
URS000008089F-lncRNA5	0,479133854	3,494287219	0,477921332	0,91168466	T2D vs Control
URS00006BB04D-tRNA5	-0,411613904	3,054494403	0,479362978	0,91168466	T2D vs Control
URS000056B96A-antisense5	0,368269126	2,621722386	0,481592146	0,91168466	T2D vs Control
URS0000784C7B-lncRNA5	-0,414808964	2,086288772	0,481962991	0,91168466	T2D vs Control
URS000024E9CC-lncRNA5	-0,434514625	2,021333988	0,484340629	0,91168466	T2D vs Control
URS00006E1108-Y_RNA5	0,245989169	4,049124964	0,484510702	0,91168466	T2D vs Control
URS00006C2A6C-Y_RNA5	-0,363318185	10,4018413	0,485188403	0,91168466	T2D vs Control
URS00006A9AE8-Y_RNA5	-0,512168274	5,946221857	0,496771901	0,930947879	T2D vs Control
URS00000EB76F-lncRNA5	-0,410550129	2,382363845	0,498301221	0,931316987	T2D vs Control
URS000019B78E-tRNA5	-0,578557907	3,458896569	0,503322477	0,932633181	T2D vs Control
URS00006C246E-snRNA5	0,444668394	4,016094545	0,503716267	0,932633181	T2D vs Control
URS00002C130C-tRNA5	0,618105416	3,549564243	0,504761948	0,932633181	T2D vs Control
URS000097B164-rRNA5	0,29637157	5,947136128	0,506021531	0,932633181	T2D vs Control
URS0000986F6F-rRNA5	-0,306930434	5,818355153	0,507741587	0,932633181	T2D vs Control
URS00009C6137-lncRNA5	0,35139602	3,379050945	0,508931687	0,932633181	T2D vs Control
URS0000907730-rRNA5	0,237253249	6,755248399	0,510331271	0,932633181	T2D vs Control
URS000034EAB6-lncRNA5	-0,416240094	2,280705495	0,511486597	0,932633181	T2D vs Control
URS0000640661-Y_RNA5	-0,326072278	11,13979054	0,512523212	0,932633181	T2D vs Control
URS00004AE46A-lncRNA5	0,37710711	3,574152246	0,512715048	0,932633181	T2D vs Control
URS00001C6042-antisense5	0,384621941	1,983328915	0,515731108	0,932633181	T2D vs Control

URS00006D74B2-tRNA5	0,308708727	4,411009625	0,517578918	0,932633181	T2D vs Control
URS000017D264-lncRNA5	0,388356545	2,092743225	0,518222054	0,932633181	T2D vs Control
URS00003475A2-lncRNA5	0,221571731	6,073704566	0,518964604	0,932633181	T2D vs Control
URS00009C6074-antisense5	0,427625926	2,11618012	0,519019038	0,932633181	T2D vs Control
URS00001EE979-lncRNA5	0,414693217	2,774714775	0,527029688	0,942863793	T2D vs Control
URS0000649B00-rRNA5	0,095320843	10,09773081	0,529412862	0,942863793	T2D vs Control
URS000069ED7F-Y_RNA5	0,379971386	4,579144673	0,530342159	0,942863793	T2D vs Control
URS00002F2DED-lncRNA5	-0,345691796	4,602442219	0,530507676	0,942863793	T2D vs Control
URS00006D23E9-snRNA5	0,362595694	3,580811892	0,532270603	0,942863793	T2D vs Control
URS0000075AD1-lncRNA5	-0,343126424	1,794828601	0,53280572	0,942863793	T2D vs Control
URS0000782759-lncRNA5	0,351935985	2,907193443	0,537497648	0,947832924	T2D vs Control
URS00001DBD56-lncRNA5	-0,318726151	3,720321434	0,53951651	0,947832924	T2D vs Control
URS00005CEC24-lncRNA5	0,383459929	2,918669315	0,539681693	0,947832924	T2D vs Control
URS000090DF6D-rRNA5	-0,071218667	11,93047681	0,542135578	0,949756312	T2D vs Control
URS00000540AC-antisense5	-0,34613695	3,451834854	0,546819251	0,950920188	T2D vs Control
URS00009554B1-rRNA5	0,322023816	4,916492139	0,547278487	0,950920188	T2D vs Control
URS00006D4008-tRNA5	0,43174931	2,742010752	0,547992731	0,950920188	T2D vs Control
URS0000682FB0-Y_RNA5	-0,259383822	10,97882695	0,549275387	0,950920188	T2D vs Control
URS00003D60C0-lncRNA5	-0,332327244	1,735581371	0,552343903	0,950920188	T2D vs Control
URS00002AD8DA-lncRNA5	0,32193018	2,376170134	0,55421696	0,950920188	T2D vs Control
URS000076E14E-lncRNA5	-0,41661454	2,947769863	0,55462646	0,950920188	T2D vs Control
URS0000028D07-lncRNA5	0,311290177	5,403821621	0,554770319	0,950920188	T2D vs Control
URS0000700D52-tRNA5	0,360596211	2,144408558	0,555043543	0,950920188	T2D vs Control
URS00000C7470-antisense5	0,340429974	3,497403787	0,558509741	0,954519092	T2D vs Control
URS00002901EC-tRNA5	0,353032165	2,043121881	0,561232582	0,956833109	T2D vs Control
URS00007CB156-lncRNA5	-0,122452796	7,841069642	0,565438939	0,961658925	T2D vs Control
URS000058CCC6-antisense5	-0,332222268	2,503982133	0,567877697	0,963171108	T2D vs Control
URS00004199FB-antisense5	-0,384436097	3,00203969	0,570444915	0,963171108	T2D vs Control
URS0000610FFE-tRNA5	-0,32794256	2,353369293	0,572114435	0,963171108	T2D vs Control
URS00005D1950-antisense5	0,305829635	2,067377939	0,572473024	0,963171108	T2D vs Control
URS0000A9CA30-lncRNA5	-0,326010077	3,103219008	0,573217713	0,963171108	T2D vs Control
URS00001142E8-lncRNA5	-0,212786013	5,750896426	0,575493105	0,964675493	T2D vs Control
URS000049B61C-antisense5	0,127646351	6,879627508	0,580320668	0,970281156	T2D vs Control
URS000052A6A6-lncRNA5	-0,301512881	3,111397823	0,585795418	0,970281156	T2D vs Control
URS00008B26D4-antisense5	0,297677094	4,369068967	0,586826798	0,970281156	T2D vs Control
URS0000204428-lncRNA5	-0,336012751	2,674128924	0,588496718	0,970281156	T2D vs Control
URS00002200B0-lncRNA5	0,327858672	3,176288712	0,590294073	0,970281156	T2D vs Control
URS000047EBB5-tRNA5	0,358778733	2,438958701	0,59063454	0,970281156	T2D vs Control
URS000075D353-lncRNA5	0,291536692	1,653940152	0,591770699	0,970281156	T2D vs Control
URS0000667737-rRNA5	0,661003301	4,834926248	0,591881125	0,970281156	T2D vs Control
URS0000677B31-tRNA5	0,230704639	7,303130994	0,593213791	0,970281156	T2D vs Control
URS000075D48E-lncRNA5	0,271705397	1,602010302	0,594668668	0,970281156	T2D vs Control
URS00005BB09E-lncRNA5	0,216151676	6,047129544	0,596393056	0,970281156	T2D vs Control
URS0000038397-lncRNA5	0,300798914	5,458381823	0,596832924	0,970281156	T2D vs Control
URS00005E51DB-lncRNA5	0,260852013	4,114269415	0,601077095	0,970281156	T2D vs Control
URS000011ABD1-lncRNA5	0,344644633	2,282001703	0,603073274	0,970281156	T2D vs Control
URS00003C2ECD-antisense5	-0,356014244	2,559377792	0,603566049	0,970281156	T2D vs Control
URS00006271F5-Y_RNA5	0,239844352	3,66489103	0,604910567	0,970281156	T2D vs Control
URS00004CE099-lncRNA5	-0,439047954	3,298450018	0,605337836	0,970281156	T2D vs Control
URS000045FD15-lncRNA5	0,288276918	4,004446293	0,605566917	0,970281156	T2D vs Control
URS0000A765F3-lncRNA5	0,275025218	3,81544174	0,608692326	0,970281156	T2D vs Control
URS000070792C-rRNA5	0,294120191	1,756278837	0,609956972	0,970281156	T2D vs Control
URS00009407E6-rRNA5	-0,318959976	2,030446175	0,610884053	0,970281156	T2D vs Control
URS000030C934-lncRNA5	0,261676642	4,417263546	0,611422513	0,970281156	T2D vs Control
URS00005BB5C9-antisense5	0,28058933	1,956136305	0,612472456	0,970281156	T2D vs Control
URS00001D4EE9-tRNA5	-0,346956011	2,839543714	0,614079167	0,970281156	T2D vs Control
URS0000626233-Y_RNA5	-0,2007816	4,209755669	0,617347825	0,970281156	T2D vs Control
URS000033F395-lncRNA5	0,280630074	1,897833694	0,622663599	0,970281156	T2D vs Control
URS0000717DFB-Y_RNA5	0,262359438	2,95584216	0,625940844	0,970281156	T2D vs Control
URS00006D1C46-snRNA5	0,237015273	6,026449126	0,628344871	0,970281156	T2D vs Control
URS00002064F6-tRNA5	-0,294265272	7,265714406	0,630140248	0,970281156	T2D vs Control
URS00006B479B-tRNA5	0,22821007	1,462161631	0,632275122	0,970281156	T2D vs Control
URS000062FB25-rRNA5	-0,06542535	9,742461609	0,632381795	0,970281156	T2D vs Control
URS0000196BD3-lncRNA5	-0,272969963	2,056335374	0,632558572	0,970281156	T2D vs Control
URS00002D1F9F-lncRNA5	0,250776185	3,254441607	0,634695288	0,970281156	T2D vs Control
URS00000A7F2D-lncRNA5	0,260018062	2,135027243	0,635762796	0,970281156	T2D vs Control

URS000063455F-rRNA5	-0,271891388	2,601127664	0,636768187	0,970281156	T2D vs Control
URS0000A90D33-snRNA5	-0,30765258	3,877391031	0,636812487	0,970281156	T2D vs Control
URS0000641C1B-Y_RNA5	0,153459266	4,028164364	0,636889317	0,970281156	T2D vs Control
URS00002B0998-lncRNA5	0,270342716	2,419217026	0,642233377	0,970281156	T2D vs Control
URS00003D4983-antisense5	0,274840544	1,851867325	0,64280545	0,970281156	T2D vs Control
URS0000098211-lncRNA5	-0,29302807	2,630527968	0,643123251	0,970281156	T2D vs Control
URS00009843EB-rRNA5	0,088306055	12,28502234	0,643384119	0,970281156	T2D vs Control
URS000038F4B2-tRNA5	0,294584261	2,156583414	0,644062697	0,970281156	T2D vs Control
URS000041FE38-lncRNA5	0,260958107	2,353684167	0,64428732	0,970281156	T2D vs Control
URS000015D954-antisense5	-0,26785689	1,950160466	0,644563302	0,970281156	T2D vs Control
URS00003C98B0-lncRNA5	-0,28687808	3,045496821	0,645692027	0,970281156	T2D vs Control
URS0000A9525F-antisense5	-0,254733715	2,995531648	0,650631199	0,970281156	T2D vs Control
URS00002968BD-antisense5	0,153077798	7,356778365	0,651174903	0,970281156	T2D vs Control
URS00005C220A-antisense5	-0,300206085	2,894185806	0,6563117	0,970281156	T2D vs Control
URS00002CDF5D-lncRNA5	0,233208549	2,151438672	0,660899649	0,970281156	T2D vs Control
URS0000ABD82A-rRNA5	0,67289662	6,247493519	0,661837627	0,970281156	T2D vs Control
URS000038D781-lncRNA5	-0,268799578	2,120835096	0,669559142	0,970281156	T2D vs Control
URS0000A8472C-Y_RNA5	-0,277852652	3,823381815	0,674746928	0,970281156	T2D vs Control
URS000063CFC9-Y_RNA5	0,2581762	2,480380401	0,674958895	0,970281156	T2D vs Control
URS00008120D6-lncRNA5	0,230335522	1,844896317	0,677133858	0,970281156	T2D vs Control
URS0000942121-rRNA5	0,032304322	14,48998565	0,678072633	0,970281156	T2D vs Control
URS0000990012-rRNA5	0,139862586	8,57303772	0,678663272	0,970281156	T2D vs Control
URS000055B99E-lncRNA5	-0,269845319	2,716513459	0,681194631	0,970281156	T2D vs Control
URS000063A7A5-Y_RNA5	-0,126703938	8,2571435	0,682763487	0,970281156	T2D vs Control
URS00005508F6-antisense5	-0,234705694	3,129098015	0,683051758	0,970281156	T2D vs Control
URS00008BA2B6-lncRNA5	-0,220952095	2,826253026	0,684784252	0,970281156	T2D vs Control
URS00004D1520-lncRNA5	0,239178735	3,203873127	0,686451272	0,970281156	T2D vs Control
URS00009C60C3-lncRNA5	-0,316594702	3,075209684	0,687767735	0,970281156	T2D vs Control
URS000013BB40-lncRNA5	0,24072234	2,666260296	0,687880575	0,970281156	T2D vs Control
URS0000038B1E-antisense5	0,218210788	3,244363175	0,689492015	0,970281156	T2D vs Control
URS0000134A86-antisense5	0,271087082	3,351781773	0,690203521	0,970281156	T2D vs Control
URS00006BBD5F-Y_RNA5	0,236746145	3,385968133	0,690286935	0,970281156	T2D vs Control
URS00009612D1-rRNA5	-0,175369276	5,646389432	0,692549484	0,970281156	T2D vs Control
URS000065D78F-Y_RNA5	0,071441171	5,875001903	0,69280344	0,970281156	T2D vs Control
URS0000511A5F-lncRNA5	0,234519495	1,837603255	0,692970268	0,970281156	T2D vs Control
URS0000495A30-lncRNA5	-0,213597498	2,253557055	0,696000542	0,970281156	T2D vs Control
URS000075AF5F-antisense5	0,245746445	2,789120304	0,696297281	0,970281156	T2D vs Control
URS000047A7F4-rRNA5	-0,38721779	4,287976289	0,697463009	0,970281156	T2D vs Control
URS0000A7AB58-Y_RNA5	-0,215337338	4,159115672	0,697823853	0,970281156	T2D vs Control
URS000075A823-rRNA5	0,55026629	6,786069906	0,69844447	0,970281156	T2D vs Control
URS0000391A04-lncRNA5	-0,170115242	5,482878094	0,698512312	0,970281156	T2D vs Control
URS000006044C-lncRNA5	-0,213786429	1,808588154	0,698989718	0,970281156	T2D vs Control
URS000017CF23-antisense5	-0,219103999	2,666340903	0,69963192	0,970281156	T2D vs Control
URS00006B33E0-Y_RNA5	0,163877423	14,2625662	0,700870118	0,970281156	T2D vs Control
URS000094F5D5-rRNA5	-0,182654267	7,000982595	0,702278387	0,970281156	T2D vs Control
URS000009738A-lncRNA5	-0,235696893	2,916436163	0,703147458	0,970281156	T2D vs Control
URS00006CE1FB-rRNA5	0,387375576	5,102179845	0,705958798	0,970281156	T2D vs Control
URS000009DDCA-tRNA5	-0,103604084	8,707527281	0,706932337	0,970281156	T2D vs Control
URS00004F482C-lncRNA5	-0,225853229	2,9888845	0,707116182	0,970281156	T2D vs Control
URS000096E1E3-rRNA5	-0,228875346	2,495902245	0,707473158	0,970281156	T2D vs Control
URS00002AD6F3-antisense5	-0,18830264	1,553235742	0,707473294	0,970281156	T2D vs Control
URS00009A5DA8-rRNA5	0,171308648	5,880019281	0,708415304	0,970281156	T2D vs Control
URS00006AD81D-Y_RNA5	-0,233303512	1,867732365	0,709384372	0,970281156	T2D vs Control
URS000024383A-lncRNA5	0,207230186	1,637698963	0,710948474	0,970281156	T2D vs Control
URS00004E5B9A-lncRNA5	-0,206161496	1,8276158	0,712023197	0,970281156	T2D vs Control
URS0000539731-antisense5	0,197754843	2,084997113	0,713849065	0,970281156	T2D vs Control
URS0000371842-lncRNA5	0,21525406	2,020987369	0,715449104	0,970281156	T2D vs Control
URS0000177135-lncRNA5	0,313891641	3,040442935	0,716264942	0,970281156	T2D vs Control
URS00009840C1-rRNA5	-0,151101026	6,418341272	0,718313779	0,970281156	T2D vs Control
URS0000637E4A-tRNA5	0,202387898	1,552702938	0,720833707	0,970281156	T2D vs Control
URS00002172B5-lncRNA5	0,258952198	2,390444931	0,721641354	0,970281156	T2D vs Control
URS000020220C-antisense5	-0,205257848	2,749759905	0,722518983	0,970281156	T2D vs Control
URS00002598CF-lncRNA5	-0,181125957	4,630900569	0,724409463	0,970281156	T2D vs Control
URS000038803E-tRNA5	0,115379813	5,940754642	0,726200353	0,970281156	T2D vs Control
URS00006744D5-tRNA5	0,231587167	2,786516677	0,728889558	0,970281156	T2D vs Control
URS000075D28F-lncRNA5	-0,201727172	2,277607756	0,72911237	0,970281156	T2D vs Control

URS00000AF0EF-lncRNA5	0,200652608	3,804446661	0,729599133	0,970281156	T2D vs Control
URS0000AA0C30-lncRNA5	0,196459796	3,233082441	0,730241033	0,970281156	T2D vs Control
URS00000DE490-lncRNA5	0,178409696	3,392102095	0,731934985	0,970281156	T2D vs Control
URS00003CA240-antisense5	-0,143963724	5,133383215	0,734386102	0,970281156	T2D vs Control
URS000047A7F4-misc_RNA]5	0,316221329	4,238929678	0,735887366	0,970281156	T2D vs Control
URS000068483A-rRNA5	-0,206109523	2,397770416	0,736216271	0,970281156	T2D vs Control
URS0000701607-Y_RNA5	-0,193707105	3,386959123	0,737123718	0,970281156	T2D vs Control
URS000020AD62-lncRNA5	-0,186426922	1,816448524	0,73751554	0,970281156	T2D vs Control
URS0000389FBF-lncRNA5	-0,1905354	2,547635864	0,738244999	0,970281156	T2D vs Control
URS000038EEDC-lncRNA5	-0,242375593	3,360541304	0,741535344	0,970281156	T2D vs Control
URS000011812A-antisense5	-0,163984286	4,132007293	0,743215922	0,970281156	T2D vs Control
URS000040CE64-lncRNA5	0,224032897	2,883976268	0,743375941	0,970281156	T2D vs Control
URS00008FEDF1-rRNA5	0,206651233	2,23767686	0,744508639	0,970281156	T2D vs Control
URS0000AAECF4-lncRNA5	0,154018189	3,702161679	0,746631266	0,970281156	T2D vs Control
URS00002AECD7-lncRNA5	0,210211481	2,148873116	0,749082694	0,970281156	T2D vs Control
URS000098604A-rRNA5	0,187781039	1,872688165	0,749140749	0,970281156	T2D vs Control
URS00003CBFB8-lncRNA5	-0,178017966	2,075423342	0,749919821	0,970281156	T2D vs Control
URS000041043F-antisense5	0,185541426	1,994452875	0,750234453	0,970281156	T2D vs Control
URS000000513C-antisense5	0,129327157	4,840741267	0,751502323	0,970281156	T2D vs Control
URS00002D33E9-lncRNA5	-0,17520862	2,231641646	0,751999335	0,970281156	T2D vs Control
URS000029A6A6-antisense5	-0,102808442	5,872434994	0,752310123	0,970281156	T2D vs Control
URS00004C82E1-antisense5	0,201816749	4,084336348	0,752875683	0,970281156	T2D vs Control
URS00002CFBEA-lncRNA5	0,165917479	2,003500281	0,753367785	0,970281156	T2D vs Control
URS00002548DF-lncRNA5	0,169058209	1,501270697	0,755793746	0,970281156	T2D vs Control
URS00006B0E5A-precursor_RNA5	-0,130530751	6,734564531	0,756189198	0,970281156	T2D vs Control
URS00002840A7-lncRNA5	0,179713413	2,142345929	0,761101793	0,970281156	T2D vs Control
URS0000112A1A-antisense5	-0,164064577	4,257411498	0,765218357	0,970281156	T2D vs Control
URS00006EBF05-misc_RNA]5	-0,283462006	3,622681484	0,766341764	0,970281156	T2D vs Control
URS00008120F8-lncRNA5	-0,175384422	1,863766293	0,769424205	0,970281156	T2D vs Control
URS0000502C74-tRNA5	-0,053805954	14,17701257	0,769694762	0,970281156	T2D vs Control
URS0000759AE0-lncRNA5	0,158781439	1,586466937	0,769985727	0,970281156	T2D vs Control
URS00002811B6-lncRNA5	-0,156240841	3,62513136	0,770649002	0,970281156	T2D vs Control
URS00005AF1AA-antisense5	0,12787852	1,232925367	0,773580994	0,970281156	T2D vs Control
URS0000955796-rRNA5	-0,03441811	10,7330721	0,773977924	0,970281156	T2D vs Control
URS0000A85AEE-lncRNA5	0,192260152	3,200285639	0,774428434	0,970281156	T2D vs Control
URS00003A72DC-antisense5	0,160501206	3,084869652	0,775648102	0,970281156	T2D vs Control
URS00002CC2A4-antisense5	-0,167732348	2,224906264	0,777095834	0,970281156	T2D vs Control
URS00008C3E41-lncRNA5	-0,120258352	5,384577661	0,780123948	0,970281156	T2D vs Control
URS000075BB81-lncRNA5	-0,155487388	2,83062659	0,783706073	0,970281156	T2D vs Control
URS0000780E15-lncRNA5	0,178668589	2,764657639	0,784522036	0,970281156	T2D vs Control
URS00001A72CE-tRNA5	0,162787511	4,122188898	0,784921044	0,970281156	T2D vs Control
URS00004EDF08-lncRNA5	-0,119388539	4,609717254	0,785547844	0,970281156	T2D vs Control
URS000053D4AB-snoRNA5	-0,152738214	1,689585927	0,786806135	0,970281156	T2D vs Control
URS00001AF592-antisense5	-0,173520039	3,230887811	0,787837218	0,970281156	T2D vs Control
URS00004C5EDB-lncRNA5	-0,173637205	2,83469135	0,78812078	0,970281156	T2D vs Control
URS000052A1C9-tRNA5	-0,143386732	4,427113529	0,788176934	0,970281156	T2D vs Control
URS00005B7465-tRNA5	0,156049133	1,847741502	0,791131081	0,970281156	T2D vs Control
URS00002EA13A-lncRNA5	-0,151428984	2,908290455	0,79160935	0,970281156	T2D vs Control
URS000095C3C7-rRNA5	0,125512796	4,667191645	0,791653616	0,970281156	T2D vs Control
URS00005C7D80-antisense5	-0,159683122	2,279201521	0,793047936	0,970281156	T2D vs Control
URS000013899F-tRNA5	-0,13004836	4,501396588	0,796094146	0,970281156	T2D vs Control
URS00001EC8D7-snRNA5	-0,108536826	4,577467935	0,799996618	0,970281156	T2D vs Control
URS0000726FAB-rRNA5	0,038859925	13,91320925	0,800464878	0,970281156	T2D vs Control
URS00006A3E7F-snRNA5	0,049941585	8,935478024	0,800773027	0,970281156	T2D vs Control
URS0000995AA3-rRNA5	-0,084563462	8,311403687	0,803979933	0,970281156	T2D vs Control
URS00001B506A-tRNA5	-0,170638559	2,259860507	0,804479372	0,970281156	T2D vs Control
URS000012C80D-lncRNA5	-0,14142026	2,726619231	0,805110411	0,970281156	T2D vs Control
URS000092B92B-rRNA5	0,353327349	5,336196426	0,805565152	0,970281156	T2D vs Control
URS00007D436A-antisense5	-0,138600664	2,239346559	0,80704005	0,970281156	T2D vs Control
URS00009C6070-lncRNA5	-0,1315938	2,53934864	0,807951399	0,970281156	T2D vs Control
URS00006CB0C3-Y_RNA5	-0,059758089	5,291889769	0,808531177	0,970281156	T2D vs Control
URS00006C133C-tRNA5	-0,139546908	3,680848373	0,811721301	0,970281156	T2D vs Control
URS0000689904-Y_RNA5	0,077964798	0,877747286	0,812432178	0,970281156	T2D vs Control
URS000096D31D-rRNA5	-0,046615661	10,50581429	0,812463866	0,970281156	T2D vs Control
URS00005580B2-lncRNA5	0,161615819	3,442360628	0,81362016	0,970281156	T2D vs Control
URS00004AFADD-lncRNA5	-0,136472173	1,695028896	0,817381025	0,970281156	T2D vs Control

URS0000A8428E-Y_RNA5	0,188394133	5,101971983	0,817838977	0,970281156	T2D vs Control
URS00006ABCCE-tRNA5	0,178975616	11,64642261	0,818951895	0,970281156	T2D vs Control
URS00004B48CD-lncRNA5	0,13399968	1,80838911	0,81908262	0,970281156	T2D vs Control
URS00006AD70A-tRNA5	-0,148071905	4,843339833	0,820327542	0,970281156	T2D vs Control
URS0000031963-antisense5	0,162749873	3,414756941	0,822299085	0,970281156	T2D vs Control
URS000037D0FB-tRNA5	-0,157681422	2,792154767	0,822715601	0,970281156	T2D vs Control
URS00005FFC78-lncRNA5	0,130955694	2,519542247	0,82525159	0,970281156	T2D vs Control
URS00006FC298-tRNA5	0,140848703	2,919601035	0,826549532	0,970281156	T2D vs Control
URS0000A81496-Y_RNA5	0,149610658	2,190786251	0,826669651	0,970281156	T2D vs Control
URS000048EB2F-antisense5	0,12862007	2,157167592	0,826823205	0,970281156	T2D vs Control
URS00004227BE-lncRNA5	-0,13062777	1,875905236	0,828560307	0,970281156	T2D vs Control
URS0000811AFB-antisense5	0,121535955	2,040750323	0,830841434	0,970281156	T2D vs Control
URS0000766C83-lncRNA5	0,13833618	2,482009239	0,831742089	0,970281156	T2D vs Control
URS00000DA56A-lncRNA5	0,126406642	2,589490843	0,833451805	0,970281156	T2D vs Control
URS0000676AED-precursor_RNA5	0,125985883	2,736959381	0,834708284	0,970281156	T2D vs Control
URS0000611F3E-lncRNA5	0,11899226	2,798037489	0,835400783	0,970281156	T2D vs Control
URS0000605748-lncRNA5	0,123230543	3,689393404	0,836155701	0,970281156	T2D vs Control
URS000019F398-lncRNA5	-0,105884669	1,659002941	0,84081451	0,970281156	T2D vs Control
URS00002FBC9E-lncRNA5	0,125545653	3,539604284	0,841215803	0,970281156	T2D vs Control
URS00006F135B-Y_RNA5	0,076677279	3,60198893	0,842273091	0,970281156	T2D vs Control
URS0000A774C0-lncRNA5	-0,060908772	6,402978234	0,842467711	0,970281156	T2D vs Control
URS000075BA00-lncRNA5	-0,256659059	7,953608312	0,843935021	0,970281156	T2D vs Control
URS000020BB55-lncRNA5	-0,130661219	3,131770633	0,84474131	0,970281156	T2D vs Control
URS0000462D45-lncRNA5	0,103786793	3,263068544	0,84568706	0,970281156	T2D vs Control
URS00006FCBA3-Y_RNA5	-0,110549025	2,601539284	0,847557553	0,970281156	T2D vs Control
URS00005AAAF0-antisense5	0,111534027	3,447551336	0,847677443	0,970281156	T2D vs Control
URS00005B5F85-lncRNA5	-0,108124376	5,187781526	0,848336499	0,970281156	T2D vs Control
URS0000093DF5-lncRNA5	-0,109815042	2,157430584	0,849504692	0,970281156	T2D vs Control
URS000045E276-antisense5	-0,106707082	7,301870906	0,849516549	0,970281156	T2D vs Control
URS00008116FE-lncRNA5	-0,10796928	2,826119493	0,85146383	0,970918788	T2D vs Control
URS00006D1A54-Y_RNA5	-0,098180499	3,828527713	0,853290108	0,971416588	T2D vs Control
URS00009A7848-rRNA5	-0,023097914	11,54132926	0,859353164	0,972350231	T2D vs Control
URS00002B7986-antisense5	0,09558702	1,782132427	0,860481298	0,972350231	T2D vs Control
URS00003DEE5B-lncRNA5	0,098902453	3,256575112	0,861298287	0,972350231	T2D vs Control
URS0000063647-antisense5	0,054051997	6,169603323	0,863253354	0,972350231	T2D vs Control
URS00006E23A8-rRNA5	0,111744168	2,840982885	0,864230599	0,972350231	T2D vs Control
URS00001A4293-lncRNA5	0,08669579	4,616590583	0,865219653	0,972350231	T2D vs Control
URS00001F3EF6-lncRNA5	-0,120425487	2,384806636	0,865387293	0,972350231	T2D vs Control
URS0000016595-lncRNA5	0,104067365	2,839797195	0,86593652	0,972350231	T2D vs Control
URS00004AC036-lncRNA5	0,086367122	4,515610764	0,866629748	0,972350231	T2D vs Control
URS000091563C-rRNA5	0,019622554	10,26950568	0,872229328	0,9762962	T2D vs Control
URS0000687FC3-Y_RNA5	0,047183786	4,184219214	0,872940093	0,9762962	T2D vs Control
URS0000161EE9-lncRNA5	0,064335526	6,078069715	0,874492772	0,976470364	T2D vs Control
URS0000699390-Y_RNA5	0,075509441	13,62373063	0,87696012	0,977443249	T2D vs Control
URS00004E7DF9-lncRNA5	-0,077718266	4,28189465	0,878160745	0,977443249	T2D vs Control
URS00005B2DE5-lncRNA5	0,081932291	4,758632634	0,88109725	0,979152588	T2D vs Control
URS000091B709-rRNA5	0,024895634	9,600448226	0,887130251	0,984292136	T2D vs Control
URS0000378BB8-lncRNA5	-0,068142705	4,18272789	0,8943402	0,990686615	T2D vs Control
URS00006D1735-snRNA5	-0,063551331	5,596569208	0,897705918	0,990686615	T2D vs Control
URS000069E2A5-tRNA5	0,081959964	3,421985706	0,898130353	0,990686615	T2D vs Control
URS00006C2C4A-Y_RNA5	-0,098529373	4,927831545	0,898772866	0,990686615	T2D vs Control
URS00006952C9-Y_RNA5	0,09137769	4,013121466	0,900868773	0,990686615	T2D vs Control
URS000075A564-lncRNA5	-0,072774991	2,096352205	0,901397264	0,990686615	T2D vs Control
URS000034E9D0-misc_RNA]5	0,104770254	3,097582218	0,903903606	0,990706712	T2D vs Control
URS0000629ECF-Y_RNA5	-0,045581614	4,37154222	0,904814371	0,990706712	T2D vs Control
URS0000928682-rRNA5	0,015651046	11,56053599	0,906661994	0,990706712	T2D vs Control
URS0000417A0F-tRNA5	0,050051584	7,465949428	0,907682869	0,990706712	T2D vs Control
URS000075D341-rRNA5	-0,108755103	3,481242239	0,909220211	0,990706712	T2D vs Control
URS00001DE9CD-antisense5	0,06763777	3,351892383	0,912187905	0,990706712	T2D vs Control
URS00002A28BF-lncRNA5	-0,074338289	2,823053274	0,91223146	0,990706712	T2D vs Control
URS0000ABD7E8-rRNA5	-0,104786303	10,98616314	0,91275411	0,990706712	T2D vs Control
URS0000418239-snRNA5	0,055501586	2,184896985	0,915776875	0,991845993	T2D vs Control
URS00006EB1B5-Y_RNA5	-0,056253815	4,084821132	0,916641647	0,991845993	T2D vs Control
URS000071DF37-tRNA5	-0,060616085	2,691836943	0,928778947	0,994193838	T2D vs Control
URS0000381123-lncRNA5	0,051924059	2,471894131	0,929074706	0,994193838	T2D vs Control
URS0000AAB7F4-lncRNA5	-0,051150713	3,962630959	0,929306149	0,994193838	T2D vs Control

URS0000997FE9-rRNA5	-0,048689957	2,876398415	0,929775517	0,994193838	T2D vs Control
URS000075DF54-lncRNA5	-0,029469582	6,14755677	0,93140072	0,994193838	T2D vs Control
URS0000397495-antisense5	0,03031763	5,990008067	0,931725745	0,994193838	T2D vs Control
URS000069466F-Y_RNA5	0,025112725	7,597826288	0,932514335	0,994193838	T2D vs Control
URS00000E0DE1-antisense5	-0,047238675	3,104520142	0,932831874	0,994193838	T2D vs Control
URS00000DAC34-lncRNA5	0,047760328	2,636836099	0,934181943	0,994193838	T2D vs Control
URS0000320E71-lncRNA5	0,039701473	2,909981717	0,937685037	0,994193838	T2D vs Control
URS00006F4537-tRNA5	0,048640792	4,037892922	0,938214092	0,994193838	T2D vs Control
URS00005A57D3-lncRNA5	0,046045594	2,579526429	0,939771771	0,994193838	T2D vs Control
URS0000189042-lncRNA5	0,046692601	1,901016294	0,940152324	0,994193838	T2D vs Control
URS000009AC8B-tRNA5	0,049118604	10,95540037	0,944561332	0,994193838	T2D vs Control
URS00007080E9-Y_RNA5	0,052338809	2,936384424	0,945582201	0,994193838	T2D vs Control
URS0000282AB2-tRNA5	0,05509719	4,591558308	0,946033303	0,994193838	T2D vs Control
URS0000994031-rRNA5	0,010459833	11,10957664	0,946188278	0,994193838	T2D vs Control
URS00006E19EA-Y_RNA5	-0,027153816	9,597384946	0,946400996	0,994193838	T2D vs Control
URS000097FEDE-rRNA5	-0,03629837	3,690975927	0,947024363	0,994193838	T2D vs Control
URS00004F0321-tRNA5	0,020616722	14,08130095	0,947940234	0,994193838	T2D vs Control
URS00006D484A-rRNA5	-0,006626915	11,67221899	0,948679957	0,994193838	T2D vs Control
URS000093FF31-rRNA5	0,033772235	2,038384139	0,951247666	0,994557963	T2D vs Control
URS0000228E94-lncRNA5	-0,034552517	2,335958891	0,954580868	0,994557963	T2D vs Control
URS000048B807-lncRNA5	-0,028625221	4,663045601	0,955661424	0,994557963	T2D vs Control
URS0000ABD7D5-rRNA5	-0,005225266	13,17747017	0,95704985	0,994557963	T2D vs Control
URS0000918AFB-rRNA5	-0,013663942	8,610055402	0,957415613	0,994557963	T2D vs Control
URS000042199A-lncRNA5	0,034422265	2,107510848	0,95756439	0,994557963	T2D vs Control
URS00002C2371-lncRNA5	0,026615283	3,691720987	0,96233319	0,997183437	T2D vs Control
URS0000537899-antisense5	0,02759661	2,143848964	0,962945379	0,997183437	T2D vs Control
URS000096B970-rRNA5	0,005999383	10,27519744	0,965776942	0,997244681	T2D vs Control
URS0000287398-tRNA5	0,028320848	2,440463804	0,965857867	0,997244681	T2D vs Control
URS000051CFB0-lncRNA5	0,038151799	3,801776289	0,967877708	0,997618176	T2D vs Control
URS000050471D-lncRNA5	-0,023450029	2,378838415	0,971067321	0,997618176	T2D vs Control
URS00006428FD-Y_RNA5	-0,015214646	6,936586919	0,97305056	0,997618176	T2D vs Control
URS000057C597-antisense5	0,018190807	2,337861425	0,976055343	0,997618176	T2D vs Control
URS000066AF0D-Y_RNA5	-0,017260114	12,73751355	0,976116205	0,997618176	T2D vs Control
URS000050E9EC-lncRNA5	0,015647918	3,382080638	0,976583667	0,997618176	T2D vs Control
URS000038D8D3-tRNA5	0,009441973	7,544518208	0,976594814	0,997618176	T2D vs Control
URS000061A10B-tRNA5	-0,019779611	2,36803002	0,978113513	0,997618176	T2D vs Control
URS00003870EC-lncRNA5	-0,014390549	2,283625175	0,982260892	0,997618176	T2D vs Control
URS00003EAC96-antisense5	-0,012597673	2,99991317	0,98424465	0,997618176	T2D vs Control
URS000094439F-rRNA5	-0,002131446	11,62287065	0,985937305	0,997618176	T2D vs Control
URS000063A6E6-rRNA5	0,002092191	11,70425189	0,986345949	0,997618176	T2D vs Control
URS000072C165-Y_RNA5	-0,004554568	5,305209475	0,986520102	0,997618176	T2D vs Control
URS00006C14B2-tRNA5	-0,01168187	3,304706256	0,98653658	0,997618176	T2D vs Control
URS00006144FC-lncRNA5	-0,006547904	7,117689109	0,987815021	0,997618176	T2D vs Control
URS0000A827F4-antisense5	0,008734775	8,224586053	0,989483466	0,997618176	T2D vs Control
URS00001E9163-lncRNA5	0,006938051	2,764922404	0,990482137	0,997618176	T2D vs Control
URS000050FA69-lncRNA5	-0,004313191	3,930388911	0,993671662	0,997968853	T2D vs Control
URS0000811D9C-antisense5	-0,001552434	0,958216717	0,996759214	0,997968853	T2D vs Control
URS00000C18F2-tRNA5	0,00167611	6,19082938	0,997037979	0,997968853	T2D vs Control
URS00009C6042-lncRNA5	0,001001825	6,706154397	0,997366835	0,997968853	T2D vs Control
URS0000391360-lncRNA5	0,001582907	2,256356542	0,997968853	0,997968853	T2D vs Control
